# Benchmark data for identifying DNA methylation sites via pseudo trinucleotide composition

**DOI:** 10.1016/j.dib.2015.04.021

**Published:** 2015-05-07

**Authors:** Zi Liu, Xuan Xiao, Wang-Ren Qiu, Kuo-Chen Chou

**Affiliations:** a Computer Department, Jing-De-Zhen Ceramic Institute, Jing-De-Zhen 333403 China; b Information School, ZheJiang Textile & Fashion College, NingBo, 315211 China; c Gordon Life Science Institute, Boston, MA 02478, United States; d Center of Excellence in Genomic Medicine Research (CEGMR), King Abdulaziz University, Jeddah 21589, Saudi Arabia

## Abstract

This data article contains three benchmark datasets for training and testing iDNA-Methyl, a web-server predictor for identifying DNA methylation sites [Liu et al. *Anal. Biochem*. 474 (2015) 69–79].

**Specifications table**

**Table:** 

Subject area	*Biology*
More specific subject area	*Bioinformatics and Biomedicine*
Type of data	*Text file*
How data was acquired	*Using flexible sliding window approach*[2–5]
Data format	*Analyzed*
Experimental factors	*n/a*
Experimental features	*DNA sample was formulated by combining its trinucleotide composition (TNC)* [6–8] *and the pseudo amino acid components (PseAAC)* [9–11] *of the sequence translated from the DNA sample according to its genetic codons. Meanwhile, some novel techniques in statistical analysis were introduced to train and test the predictor, such as “Neighborhood Cleaning Rule”, “Synthetic Minority Over-Sampling Technique”, and “Target-Jackknife Test”* [12].
Data source location	*Jingdezhen 333403, China*
Data accessibility	With this paper and at: *http://www.jci-bioinfo.cn/DNAmethy/IDM_data.html*

## 1 Value of the data

• DNA methylation plays an important role in regulating a variety of biological processes and is very important for basic research and drug development as well.
• The datasets presented here are good for testing DNA methylation site identifying algorithms because of their realistic, highly unbalanced nature.
• For the first dataset (Supplementary material, File 1), users can use the original sequences to construct their own benchmark dataset, for the the 2nd dataset (Supplementary material, File 2) and the 3rd dataset (Supplementary material, File 1) users can use them to design their own predictor for identifying methylation sites.

## 2 Data, experimental design, materials and methods

The data presented here are three benchmark datasets for training and testing iDNA-Methyl [1] http://www.jci-bioinfo.cn/iDNA-Methyl, a web-server predictor for identifying DNA methylation sites. The DNA sample was formulated by combining its trinucleotide composition (TNC) and the pseudo amino acid components (PseAAC) of the sequence translated from the DNA sample according to its genetic codons. Sliding a window of nucleotides along each of the DNA sequences taken from MethDB (http://www.methdb.de/), and DNA sample was formulated by combining its trinucleotide composition (TNC) and the pseudo amino acid components (PseAAC) of the sequence translated from the DNA sample according to its genetic codons. In real world, the data very unbalanced. Target-jackknife was used to optimize the unbalanced benchmark dataset and minimize the consequence of this kind of mis-prediction.

I. The first dataset (Supplementary material, File 1) contains 2426 nucleotide segment samples, of which 787 are true methylation ones and 1639 are false methylation ones.
II. The 2nd dataset (Supplementary material, File 2) is the optimized benchmark dataset obtained after the NCR (Neighborhood Cleaning Rule) [13] treatments on the original benchmark dataset of the DNA methylation system. It contains 522 non-methylation samples that were removed from the negative subset, each of which corresponds to a vector with 72 components. For distinction, the real Non-methylation starts with a line of “>Non-Methylation code”.
III. The 3rd dataset (Supplementary material, File 1) is the optimized benchmark dataset obtained after both the NCR (Neighborhood Cleaning Rule) [13] and SMOTE (Synthetic Minority Over-Sampling Technique) [14] treatments on the 1st benchmark dataset. It contains 1117 DNA methylation (including 330 hypothetical methylation created by SMOTE) and 1117 non-methylation, each of which corresponds to a vector with 72 components. For distinction, the real DNA methylation starts with a line of “>Methylation code” while the hypothetical DNA methylation starts with a line of “Hypothetical” [6–8].
