# Supplementary material for: Benchmark data for identifying DNA methylation sites via pseudo trinucleotide composition
Source: Data Brief. 2015 May 7;4:87–9. doi: 10.1016/j.dib.2015.04.021 (PMC4510404; doi:10.1016/j.dib.2015.04.021)
Supplement: Supplementary file 1 — Supplementary data [file mmc1.zip › Supp1.doc]

**Supporting Information S1. The original benchmark dataset**. It contains 2,426 DNA segments, of which 787 are methylation segments or positive samples, and 1,639 non-methylation segments or negative samples, where the DNA ID is taken from the code in MethDB (<http://www.methdb.de/>), and the site of a segment refers to its sequence position in a DNA. Each segment sample contains 41 nucleotide residues. None of the samples include here is identical to any other.

**I. Positive subset contains 787 methylation segment samples**

| Segment# | ID | Site | Sequence |
| --- | --- | --- | --- |
| 1  2  3  4  5  6  7  8  9  10  11  12  13  14  15  16  17  18  19  20  21  22  23  24  25  26  27  28  29  30  31  32  33  34  35  36  37  38  39  40  41  42  43  44  45  46  47  48  49  50  51  52  53  54  55  56  57  58  59  60  61  62  63  64  65  66  67  68  69  70  71  72  73  74  75  76  77  78  79  80  81  82  83  84  85  86  87  88  89  90  91  92  93  94  95  96  97  98  99  100  101  102  103  104  105  106  107  108  109  110  111  112  113  114  115  116  117  118  119  120  121  122  123  124  125  126  127  128  129  130  131  132  133  134  135  136  137  138  139  140  141  142  143  144  145  146  147  148  149  150  151  152  153  154  155  156  157  158  159  160  161  162  163  164  165  166  167  168  169  170  171  172  173  174  175  176  177  178  179  180  181  182  183  184  185  186  187  188  189  190  191  192  193  194  195  196  197  198  199  200  201  202  203  204  205  206  207  208  209  210  211  212  213  214  215  216  217  218  219  220  221  222  223  224  225  226  227  228  229  230  231  232  233  234  235  236  237  238  239  240  241  242  243  244  245  246  247  248  249  250  251  252  253  254  255  256  257  258  259  260  261  262  263  264  265  266  267  268  269  270  271  272  273  274  275  276  277  278  279  280  281  282  283  284  285  286  287  288  289  290  291  292  293  294  295  296  297  298  299  300  301  302  303  304  305  306  307  308  309  310  311  312  313  314  315  316  317  318  319  320  321  322  323  324  325  326  327  328  329  330  331  332  333  334  335  336  337  338  339  340  341  342  343  344  345  346  347  348  349  350  351  352  353  354  355  356  357  358  359  360  361  362  363  364  365  366  367  368  369  370  371  372  373  374  375  376  377  378  379  380  381  382  383  384  385  386  387  388  389  390  391  392  393  394  395  396  397  398  399  400  401  402  403  404  405  406  407  408  409  410  411  412  413  414  415  416  417  418  419  420  421  422  423  424  425  426  427  428  429  430  431  432  433  434  435  436  437  438  439  440  441  442  443  444  445  446  447  448  449  450  451  452  453  454  455  456  457  458  459  460  461  462  463  464  465  466  467  468  469  470  471  472  473  474  475  476  477  478  479  480  481  482  483  484  485  486  487  488  489  490  491  492  493  494  495  496  497  498  499  500  501  502  503  504  505  506  507  508  509  510  511  512  513  514  515  516  517  518  519  520  521  522  523  524  525  526  527  528  529  530  531  532  533  534  535  536  537  538  539  540  541  542  543  544  545  546  547  548  549  550  551  552  553  554  555  556  557  558  559  560  561  562  563  564  565  566  567  568  569  570  571  572  573  574  575  576  577  578  579  580  581  582  583  584  585  586  587  588  589  590  591  592  593  594  595  596  597  598  599  600  601  602  603  604  605  606  607  608  609  610  611  612  613  614  615  616  617  618  619  620  621  622  623  624  625  626  627  628  629  630  631  632  633  634  635  636  637  638  639  640  641  642  643  644  645  646  647  648  649  650  651  652  653  654  655  656  657  658  659  660  661  662  663  664  665  666  667  668  669  670  671  672  673  674  675  676  677  678  679  680  681  682  683  684  685  686  687  688  689  690  691  692  693  694  695  696  697  698  699  700  701  702  703  704  705  706  707  708  709  710  711  712  713  714  715  716  717  718  719  720  721  722  723  724  725  726  727  728  729  730  731  732  733  734  735  736  737  738  739  740  741  742  743  744  745  746  747  748  749  750  751  752  753  754  755  756  757  758  759  760  761  762  763  764  765  766  767  768  769  770  771  772  773  774  775  776  777  778  779  780  781  782  783  784  785  786  787 | 381  381  381  381  381  381  381  776  776  776  776  776  776  776  776  776  776  776  776  776  861  861  862  862  862  862  862  862  862  862  862  862  863  863  863  863  875  875  897  897  897  897  897  897  897  897  897  897  897  897  897  897  897  897  897  906  906  906  906  906  906  906  906  906  906  906  906  906  906  906  906  906  906  957  957  957  957  957  957  957  957  957  957  957  957  957  957  957  957  957  957  957  957  957  957  957  1000  1000  1017  1017  1017  1017  1017  1017  1017  1017  1017  1017  1017  1017  1017  1017  1017  1017  1017  1017  1017  1017  1017  1017  1017  1017  1017  1017  1017  1017  1017  1017  1017  1017  1017  1017  1017  1017  1017  1017  1017  1017  1017  1017  1017  1017  1017  1017  1017  1017  1017  1019  1019  1019  1019  1019  1019  1019  1019  1019  1019  1019  1019  1019  1019  1019  1019  1019  1019  1019  1019  1019  1019  1019  1019  1019  1019  1019  1019  1019  1019  1019  1019  1019  1019  1027  1027  1027  1027  1027  1027  1027  1027  1027  1027  1027  1027  1027  1027  1027  1027  1027  1027  1027  1027  1066  1066  1066  1066  1066  1066  1066  1066  1066  1066  1066  1116  1116  1116  1116  1116  1116  1116  1116  1116  1116  1116  1116  1116  1116  1116  1116  1128  1128  1128  1128  1128  1128  1128  1128  1128  1128  1128  1128  1128  1128  1128  1128  1128  1128  1128  1128  1128  1128  1128  1128  1128  1128  1128  1128  1128  1128  1128  1128  1128  1128  1128  1128  1128  1128  1128  1128  1128  1128  1128  1128  1128  1128  1128  1128  1128  1261  1261  1329  1329  1329  1329  1329  1329  1329  1329  1329  1329  1329  1329  1329  1329  1329  1329  1345  1345  1345  1345  1345  1345  1345  1345  1345  1376  1376  1376  1376  1376  1376  1376  1376  1376  1376  1376  1376  1376  1376  1376  1437  1437  1437  1437  1437  1437  1437  1440  1440  1440  1440  1440  1440  1440  1440  1440  1440  1440  1440  1440  1440  1440  1440  1440  1440  1440  1440  1440  1440  1440  1440  1440  1440  1443  1443  1443  1443  1443  1443  1443  1443  1443  1443  1443  1443  1443  1443  1443  1443  1443  1443  1443  1443  1443  1443  1443  1443  1443  1443  1443  1443  1443  1443  1443  1443  1443  1443  1443  1443  1443  1443  1443  1445  1445  1445  1445  1445  1445  1445  1445  1445  1445  1445  1445  1445  1445  1445  1445  1445  1445  1445  1445  1445  1445  1445  1445  1445  1445  1445  1445  1445  1447  1447  1447  1447  1447  1447  1447  1447  1447  1447  1447  1447  1447  1447  1447  1447  1447  1447  1447  1447  1447  1447  1447  1447  1447  1447  1447  1447  1447  1447  1447  1447  1447  1447  1447  1447  1447  1470  1470  1470  1470  1470  1470  1470  1470  1470  1470  1470  1470  1470  1470  1470  1470  1470  1470  3162  3162  3162  3162  3162  3162  3162  3162  3162  3164  3167  3168  3168  3169  3169  3169  3169  3169  3169  3169  3170  3170  3170  3171  3171  3171  3171  3171  3171  3171  3171  3171  3173  3173  3173  3173  3173  3175  3175  3175  3175  3175  3175  3175  21046  21127  21127  21127  21127  21127  21127  21127  21127  21127  21135  21135  21135  21135  21135  21135  21139  21139  21139  21139  21139  21139  21139  21139  21139  21139  21139  21139  21139  21139  21139  21139  21139  21148  21148  21148  21148  21148  21148  21148  21148  21148  21148  21152  21152  21152  21152  21166  21166  21166  21166  21166  21166  21206  21206  21206  21206  21206  21206  21206  21206  21206  21206  21206  21206  21206  21206  21210  21210  21210  21210  21210  21210  21210  21210  21210  21210  21210  21210  21210  21210  21226  21226  21226  21226  21226  21226  21226  21226  21226  21226  21226  21226  21251  21251  21251  21251  21251  21251  21251  21251  21259  21259  21259  21259  21259  21259  21259  21259  21259  21259  21259  21259  21259  21259  21259  21259  21259  21285  21285  21285  21285  21285  21285  21285  21285  21285  21285  21285  21285  21285  21285  21285  21285  21285  21285  21285  21285  21285  21285  21285  21285  21285  21285  21285  21285  21285  21285  21285  21285  21285  21285  21285  21285  21285  21285  21285  21285  21352  21354  21354  21354  21357  21357  21357  21357  21357  21357  21357  21357  21357  21357  21357  21357  21357  21357  21357  21357  21357  21357  21357  21357  21357  21357  21357  21357  21357  21357  21357  21362  21362  21362  21362  21362  21362  21362  21362  21362  21362  21362  21362  21362  21362  21362  21362  21362  21362  21362  21362  21362  21362  21362  21362  21362  21362  21362  21362  21362  21362  21362  21362  21362  21362  21368  21368  21368  21368  21368  21368  21368  21368  21368  21368  21368  21368  21368  21368  21368  21368  21368  21368  21368  21368  21368  21368  21368  21368  21368  21368  21368  21368  21368  21368  21368  21368  21368  21368  21368  21368  21368  21368  21368  21368  21368  21368  21523  21523  21523 | 21  34  49  56  111  122  221  7  17  75  79  102  124  164  169  187  221  229  233  255  70  126  27  33  51  60  79  143  204  286  306  362  134  147  184  387  20  389  25  33  76  93  102  110  160  181  184  200  208  215  241  244  282  288  347  74  78  86  94  101  107  124  128  143  146  152  155  178  191  203  235  318  330  27  32  67  140  149  167  211  221  228  231  248  254  265  269  273  277  281  285  305  314  316  360  370  107  219  30  33  44  51  54  62  65  70  77  82  86  93  100  104  109  117  129  135  138  156  166  176  178  189  193  202  216  229  235  247  253  280  282  286  292  295  302  313  316  322  324  340  343  355  360  363  365  367  369  34  37  52  59  88  104  147  151  164  165  171  174  176  191  198  215  223  229  243  253  265  269  284  313  320  330  332  357  367  375  378  399  415  442  35  40  52  72  75  101  146  157  222  229  236  239  262  273  277  285  305  314  316  360  1  10  30  69  91  120  127  144  153  155  193  215  290  375  378  383  400  405  438  482  754  797  799  804  831  870  891  7  11  15  17  37  43  46  49  54  60  69  73  82  101  103  108  116  122  124  128  130  140  144  146  150  152  154  159  162  171  178  184  190  193  201  203  205  210  215  226  232  234  239  258  265  268  271  277  279  179  262  2  31  33  43  45  62  68  208  216  238  286  290  293  297  304  317  22  132  136  140  230  282  302  307  327  1  8  22  30  39  52  74  95  98  107  111  176  213  240  250  17  79  93  140  244  289  450  24  45  61  65  75  86  93  128  144  180  203  272  277  304  313  390  414  416  423  426  438  440  449  452  458  464  2  4  17  23  60  70  75  162  168  171  184  195  198  204  208  210  216  223  226  229  231  240  243  252  260  265  317  339  343  371  380  385  390  404  407  418  423  428  453  4  8  14  20  23  32  34  43  46  49  56  58  92  117  131  144  165  168  186  195  200  237  292  328  344  379  386  407  411  40  45  50  64  72  78  83  86  97  100  129  151  203  216  225  228  237  239  242  245  252  258  260  270  273  284  297  300  306  315  318  320  328  445  451  464  466  30  63  74  142  165  183  194  216  223  255  277  298  318  332  375  405  409  431  1  48  50  79  125  132  143  201  219  103  3  2  117  45  87  100  102  117  135  165  215  239  258  13  31  53  89  100  107  153  170  184  18  55  59  76  102  61  79  94  96  109  151  195  74  26  32  37  55  64  89  126  129  155  107  111  113  129  164  312  51  75  77  79  88  106  120  136  145  216  325  336  347  374  420  473  492  28  33  38  215  229  320  333  338  357  362  469  483  507  522  74  186  242  254  260  279  12  39  49  72  74  94  115  118  130  223  247  253  270  272  2  19  27  47  49  51  64  69  82  85  91  104  112  128  18  25  35  49  54  58  60  73  75  81  86  93  81  103  122  139  142  180  201  208  347  369  373  385  387  389  407  411  423  425  427  435  445  491  509  527  537  1  3  6  14  17  24  45  61  66  73  75  98  104  109  123  126  150  155  180  198  213  225  230  232  236  239  257  259  273  279  281  293  295  303  318  320  327  329  331  333  27  154  333  430  1  6  13  26  34  50  72  78  93  97  101  106  108  134  136  140  153  159  161  166  269  280  286  290  319  333  336  1  10  12  14  48  51  54  62  73  100  104  110  139  148  173  179  196  216  232  235  243  250  253  258  269  271  293  304  346  366  388  390  407  409  13  112  138  150  154  156  170  188  198  200  202  206  213  215  219  222  229  238  240  244  251  253  255  260  264  266  268  272  279  281  283  288  292  301  326  331  338  345  357  376  383  426  150  200  236 | TTTAATGGAGGCTGGAGAGG**C**TACAAGGTTCACCAGTAAAG  GGAGAGGCTACAAGGTTCAC**C**AGTAAAGATGCTTGCAAGAT  TTCACCAGTAAAGATGCTTG**C**AAGATCCTGAGGAATGATGA  GTAAAGATGCTTGCAAGATC**C**TGAGGAATGATGAAATCATC  GATTGGTTTGATTAACGAAT**C**AGAGCAAGATCTGGATCTAG  TTAACGAATCAGAGCAAGAT**C**TGGATCTAGAACTCCGTTTG  CTAGAAGATATGATGCTTCT**C**TAAGGTTAATTAGTTTCATC  CTTCCTACCCTCGGCATATA**C**GGCCTGGTCCGGTCCTAGCT  TCGGCATATACGGCCTGGTC**C**GGTCCTAGCTACCTGGGCCA  TTTGGTCAGTGCAGGAGACC**C**GGGCGGGACCCAGGCTGAGA  GTCAGTGCAGGAGACCCGGG**C**GGGACCCAGGCTGAGAACCA  GACCCAGGCTGAGAACCAGC**C**GAAGGAAGGGACTCTAGTGC  AAGGAAGGGACTCTAGTGCC**C**GACACCCAAATATGGCTTGG  GGAAGGGCAGCAACATTCTT**C**GGGGCGGTGTGGGGAGAGCT  GGCAGCAACATTCTTCGGGG**C**GGTGTGGGGAGAGCTCCCGG  GGCGGTGTGGGGAGAGCTCC**C**GGGACTATATAAAAACCTGT  AACCTGTGCAAGGGGACAGG**C**GGTCACACGGACGTAAGCCT  CAAGGGGACAGGCGGTCACA**C**GGACGTAAGCCTCACTTCCT  GGGACAGGCGGTCACACGGA**C**GTAAGCCTCACTTCCTACCC  TAAGCCTCACTTCCTACCCT**C**GGCATATACGGCCTGGTCCG  AAGCACCTCAAGGCCCCATA**C**GGAGAAAGTTCTAGACGCAG  GGGGTCCTTACAGTAGCCCT**C**GCGGGCCCCAGCGCCCACCC  AGTCACCAAATCAGCAACAA**C**GTGACCCAGTGGTGCAAGTC  CAAATCAGCAACAACGTGAC**C**CAGTGGTGCAAGTCTTCCGA  ACCCAGTGGTGCAAGTCTTC**C**GAATCCCACGCTACCATCCC  TGCAAGTCTTCCGAATCCCA**C**GCTACCATCCCAGCACCCCG  ACGCTACCATCCCAGCACCC**C**GTGGACCAAGAGTGGGTGGG  ACATAGATCTTCCTGCCTCT**C**GGGGGCATTCTGAAGACCAG  AGGAACTGAGCAAGTCATGA**C**GAAGCAGAACCCTGGGAAGG  ACAGGTTCTACCTGTTCCAT**C**GGGTCCCAATTCCAGGGTCC  CGGGTCCCAATTCCAGGGTC**C**ACCAGCTGCACACACCAGTC  AGGCACCCTCCTGCCTAACT**C**GCCTGCTTGCTCCATAGGTC  CTGGCAGGGCACAAAAGGTA**C**GGGACTTGTAAGCGCTGGGC  AAAGGTACGGGACTTGTAAG**C**GCTGGGCAGCCAAGAACATC  CATCCAGTTCAGTTACCCTG**C**GAGGCTGGGGCCGCCACTCT  AGCGGCCTCTCCCGACGATC**C**CTGCCCCAGGAAGCCCGAGA  GCGTTCACAGGCCCCACAAA**C**TTCCGTTCGCTGGTCAATTA  GAGGATCCAGCGGCCTTCCG**C**CCGGGGCCGCTTCGCCTCCG  GACTTGGCCTTGGGGACATG**C**GGCCTCACGGCCACTGCAGG  CTTGGGGACATGCGGCCTCA**C**GGCCACTGCAGGGACCCAGG  AGTCCTGGGCCCACATGGGC**C**AGATGGTCAATGGGGCCGAG  GGCCAGATGGTCAATGGGGC**C**GAGGTGTTCGGGGGCCCAGG  GTCAATGGGGCCGAGGTGTT**C**GGGGGCCCAGGGGAGTGAGA  GGCCGAGGTGTTCGGGGGCC**C**AGGGGAGTGAGAACCCCCTC  CTACATCTCCCCTGGGCAGG**C**GGCTACCACCCTGTGAAGAT  GGCTACCACCCTGTGAAGAT**C**GGCGACGTGTTCAATGGGCG  TACCACCCTGTGAAGATCGG**C**GACGTGTTCAATGGGCGGTA  TCGGCGACGTGTTCAATGGG**C**GGTACCACGTGGTGCGCAAA  GTGTTCAATGGGCGGTACCA**C**GTGGTGCGCAAACTGGGCTG  ATGGGCGGTACCACGTGGTG**C**GCAAACTGGGCTGGGGCCAC  CTGGGCTGGGGCCACTTCTC**C**ACCGTCTGGCTCTGCTGGGA  GGCTGGGGCCACTTCTCCAC**C**GTCTGGCTCTGCTGGGACAT  CATCCAGTGAGTGCCTCCTT**C**GCCTCCGGGGCGGAGCACTG  GTGAGTGCCTCCTTCGCCTC**C**GGGGCGGAGCACTGGCTGGG  CTTGGGGCCACCCTGATCCC**C**GCCGTGGGTCCTGCCAGTGA  CCGGGCCCCACAGGAGCAGC**C**GCCCGGGGCACCGGAGCTGC  GCCCCACAGGAGCAGCCGCC**C**GGGGCACCGGAGCTGCGGGC  GGAGCAGCCGCCCGGGGCAC**C**GGAGCTGCGGGCTGCGTGGC  CGCCCGGGGCACCGGAGCTG**C**GGGCTGCGTGGCCGGGATGA  GGCACCGGAGCTGCGGGCTG**C**GTGGCCGGGATGAGCGCCAG  GGAGCTGCGGGCTGCGTGGC**C**GGGATGAGCGCCAGCACGGG  GGCCGGGATGAGCGCCAGCA**C**GGGCGGTGGTGGGGACAGCG  GGGATGAGCGCCAGCACGGG**C**GGTGGTGGGGACAGCGGCGG  ACGGGCGGTGGTGGGGACAG**C**GGCGGCAGCGGCGGCAGTAG  GGCGGTGGTGGGGACAGCGG**C**GGCAGCGGCGGCAGTAGCAG  GGTGGGGACAGCGGCGGCAG**C**GGCGGCAGTAGCAGCAGGTA  GGGGACAGCGGCGGCAGCGG**C**GGCAGTAGCAGCAGGTAGGG  CAGTAGCAGCAGGTAGGGCT**C**GGCTGGGGCACCCGGAGCCC  TAGGGCTCGGCTGGGGCACC**C**GGAGCCCCTGGCGTCTCTCA  GGGGCACCCGGAGCCCCTGG**C**GTCTCTCATGCCCACTGCCA  CCACTGCCACTCACCCCACC**C**GCAGCTCACAGGCCTCCTGC  GTGCCTCAGAAGCTGCAGGG**C**CTTCTGGGCTCCGACGTCGA  CTGCAGGGCCTTCTGGGCTC**C**GACGTCGAGGAACAGGAAGA  AGGAGCCCTGGCTGCCCCCA**C**GGGTCGGGCACACAAACGGG  CCCTGGCTGCCCCCACGGGT**C**GGGCACACAAACGGGACATT  GACATTGTGTGGGCCCCCCA**C**GTGTGCACACACACGAACAC  CCTGCCCTCCCCTCCCCTCG**C**GGCCCTCCCGCCCCTCCCCT  CCCTCCCCTCGCGGCCCTCC**C**GCCCCTCCCCTCTGGCCCGG  CCCGCCCCTCCCCTCTGGCC**C**GGGCCTGGAACGCTGGGTGC  AGCCAGGCTTGGGAAGCCTG**C**GGCCTGGCCCGCCTGGCGCC  GGGAAGCCTGCGGCCTGGCC**C**GCCTGGCGCCGCCACTGGAC  CTGCGGCCTGGCCCGCCTGG**C**GCCGCCACTGGACACACTGC  CGGCCTGGCCCGCCTGGCGC**C**GCCACTGGACACACTGCATG  CGCCGCCACTGGACACACTG**C**ATGCACGTCCCATGCCCGCC  CACTGGACACACTGCATGCA**C**GTCCCATGCCCGCCCGCCCG  CTGCATGCACGTCCCATGCC**C**GCCCGCCCGCCCGCCCGCCC  ATGCACGTCCCATGCCCGCC**C**GCCCGCCCGCCCGCCCGGGC  ACGTCCCATGCCCGCCCGCC**C**GCCCGCCCGCCCGGGCCCAG  CCCATGCCCGCCCGCCCGCC**C**GCCCGCCCGGGCCCAGCTTA  TGCCCGCCCGCCCGCCCGCC**C**GCCCGGGCCCAGCTTAGCAA  CGCCCGCCCGCCCGCCCGCC**C**GGGCCCAGCTTAGCAACAGC  CGGGCCCAGCTTAGCAACAG**C**GATGGGCACGCGTGTGTCCT  CTTAGCAACAGCGATGGGCA**C**GCGTGTGTCCTGTGACTACA  TAGCAACAGCGATGGGCACG**C**GTGTGTCCTGTGACTACAAA  GCACTGGGGTTGCTGGAAGC**C**GAAGTGACCCGGTGATGGGT  TGCTGGAAGCCGAAGTGACC**C**GGTGATGGGTGGGAAACAGA  CTCGGGGCCCTCCCCGGTGC**C**GCCGGTGCCCCCCGCCTGAC  GCCGAGAACGGCATCTATAG**C**GTGTCCGGCGACGAGAAGAA  TGGTGGACTGCTTCTGACTG**C**GCCGCCTGTGACCCGCACCC  TGGACTGCTTCTGACTGCGC**C**GCCTGTGACCCGCACCCCGC  TGACTGCGCCGCCTGTGACC**C**GCACCCCGCCGCTCTCCCGC  GCCGCCTGTGACCCGCACCC**C**GCCGCTCTCCCGCCGCCCCG  GCCTGTGACCCGCACCCCGC**C**GCTCTCCCGCCGCCCCGTCC  CCCGCACCCCGCCGCTCTCC**C**GCCGCCCCGTCCCCCGGCCC  GCACCCCGCCGCTCTCCCGC**C**GCCCCGTCCCCCGGCCCGGC  CCGCCGCTCTCCCGCCGCCC**C**GTCCCCCGGCCCGGCCGCCC  TCTCCCGCCGCCCCGTCCCC**C**GGCCCGGCCGCCCCCCGGCC  CGCCGCCCCGTCCCCCGGCC**C**GGCCGCCCCCCGGCCCCCGG  GCCCCGTCCCCCGGCCCGGC**C**GCCCCCCGGCCCCCGGCCGG  CCCCCGGCCCGGCCGCCCCC**C**GGCCCCCGGCCGGTCCGCGC  CCCGGCCGCCCCCCGGCCCC**C**GGCCGGTCCGCGCCCTCGGG  GCCGCCCCCCGGCCCCCGGC**C**GGTCCGCGCCCTCGGGGCCC  CCCCCGGCCCCCGGCCGGTC**C**GCGCCCTCGGGGCCCTCCCC  CCCCGGCCGGTCCGCGCCCT**C**GGGGCCCTCCCCGGTGCCGC  CGCGCCCTCGGGGCCCTCCC**C**GGTGCCGCCGGTGCCCCGCG  CTCGGGGCCCTCCCCGGTGC**C**GCCGGTGCCCCGTGCCTGAC  GGGGCCCTCCCCGGTGCCGC**C**GGTGCCCCGCGCCTGACCGC  GCCGGTGCCCCGAGCCTGAC**C**GCAGCCCCCCGCAGGGTGCC  CGTGCCTGACCGCAGCCCCC**C**GCAGGGTGCCGCGACCCCAG  CGCAGCCCCCCGCAGGGTGC**C**GCGACCCCAGCCCGGCCGTG  CAGCCCCCCGCAGGGTGCCG**C**GACCCCAGCCCGGCCGTGAG  AGGGTGCCGCGACCCCAGCC**C**GGCCGTGAGGCCCGCAGGGG  TGCCGCGACCCCAGCCCGGC**C**GTGAGGCCCGCAGGGGCCAT  CCCAGCCCGGCCGTGAGGCC**C**GCAGGGGCCATGGCGAAGAA  GAGGCCCGCAGGGGCCATGG**C**GAAGAAGAGCGCCGAGAACG  GCCATGGCGAAGAAGAGCGC**C**GAGAACGGCATCTGTAGCGT  GCGAAGAAGAGCGCCGAGAT**C**GGCATTTGTAGCGTGTCCGG  GCCGAGAACGGCATGTGTAG**C**GTGTCCGGCGACCAGAAGAA  ATCGGCATTTGTAGCGTGTC**C**GGCGACCAGAAGAAGGGTCC  CAGAAGAAGGGTCCCCTCAT**C**GCGCCCGGGCCCGACGGGGC  GAAGAAGGGTCCCCTCATCG**C**GCCCGGGCCCGACGGGGCCC  AAGGGTCCCCTCATCGCGCC**C**GGGCCCGACGGGGCCCGGGC  CCCCTCATCGCGCCCGGGCC**C**GACGGGGCCCGGGCCAAGGG  CTCATCGCGCCCGGGCCCGA**C**GGGGCCCGGGCCAAGGGCGA  CGCCCGGGCCCGACGGGGCC**C**GGGCCAAGGGCGACGGCCCC  GACGGGGCCCGGGCCAAGGG**C**GACGGCCCCGCGGGCCTGGG  GGGGCCCGGGCCAAGGGCGA**C**GGCCCCGCGGGCCTGGGGAC  CGGGCCAAGGGCGACGGCCC**C**GCGGGCCTGGGGACACCCGG  GGCCAAGGGCGACGGCCCCG**C**GGGCCTGGGGACACCCGGCG  CCCGCGGGCCTGGGGACACC**C**GGCGGCGGCCTGGCCGTGCC  GCGGGCCTGGGGACACCCGG**C**GGCGGCCTGGCCGTGCCGCC  ACACCCGGCGGCGGCCTGGC**C**GTGCCGCCGCGCGCGATAGA  CGGCGGCGGCCTGGCCGTGC**C**GCCGCGCGCGATAGACCTGT  CGGCGGCCTGGCCGTGCCGC**C**GCGCGCGATAGACCTGTCAA  GCGGCCTGGCCGTGCCGCCG**C**GCGCGATAGACCTGTCAAGT  GGCCTGGCCGTGCCGCCGCG**C**GCGATAGACCTGTCAAGTGC  CCTGGCCGTGCCGCCGCGCG**C**GATAGACCTGTCAAGTGCTT  ATCATGTCGTGTGTGGGCTT**C**GCCGTGGGCCTGGGCAACGT  ATGTCGTGTGTGGGCTTCGC**C**GTGGGCCTGGGCAACGTGTG  TTCGCCGTGGGCCTGGGCAA**C**GTGTGGCGCTTCCCCTACCT  TGGGCCTGGGCAACGTGTGG**C**GCTTCCCCTACCTGTGCTAC  TACCTGTGCTACAAGAATGG**C**GGAGGTGAGCTCCCCCGCCC  ATGGCGGAGGTGAGCTCCCC**C**GCCCCCCGCGGCCCCCTCCC  AGCAGGCCGCCGGCCCCTGA**C**GCCCGACCCCCAACCCCCGG  GGCCGCCGGCCCCTGACGCC**C**GACCCCCAACCCCCGGAGCC  TGACGCCCGACCCCCAACCC**C**CGGAGCCGCCGCGGAGGGGT  GACGCCCGACCCCCAACCCC**C**GGAGCCGCCGCGGAGGGGTG  CGACCCCCAACCCCCGGAGC**C**GCCGCGGAGGGGTGAAGTCC  CCCCCAACCCCCGGAGCCGC**C**GCGGAGGGGTGAAGTCCGGG  CCCAACCCCCGGAGCCGCCG**C**GGAGGGGTGAAGTCCGGGCA  CGCCGCGGAGGGGTGAAGTC**C**GGGCAGCGGTTGGCCCCTGG  GAGGGGTGAAGTCCGGGCAG**C**GGTTGGCCCCTGGGCACGCG  CAGCGGTTGGCCCCTGGGCA**C**GCGGGGTCGGGGCCGCCCCT  GGCCCCTGGGCACGCGGGGT**C**GGGGCCGCCCCTGGTCCACC  TGGGCACGCGGGGTCGGGGC**C**GCCCCTGGTCCACCGCTGCT  CGGGGCCGCCCCTGGTCCAC**C**GCTGCTGCTCGGTGGCTGGG  CCTGGTCCACCGCTGCTGCT**C**GGTGGCTGGGCCGTCCGCCT  CTGCTGCTCGGTGGCTGGGC**C**GTCCGCCTCCACCCCTCTCG  TGCTCGGTGGCTGGGCCGTC**C**GCCTCCACCCCTCTCGCAGT  CCGTCCGCCTCCACCCCTCT**C**GCAGTCATGTGCCTGGCAGG  GTGCCTGGCAGGGTGAGGGG**C**GGGGGCCGGCGATGCCCGCG  GCAGGGTGAGGGGCGGGGGC**C**GGCGATGCCCGCGAGGCTGC  GGGCGGGGGCCGGCGATGCC**C**GCGAGGCTGCCCCCCAGACT  GCGGGGGCCGGCGATGCCCG**C**GAGGCTGCCCCCCAGACTCC  CTGCCCCCCAGACTCCTGGG**C**TGGAAGGAGCGATTGGCCGC  GACTCCTGGGCTGGAAGGAG**C**GATTGGCCGCCGAGGTGGGA  GGCTGGAAGGAGCGATTGGC**C**GCCGAGGTGGGAAAGCAGGC  TGGAAGGAGCGATTGGCCGC**C**GAGGTGGGAAAGCAGGCCTG  GAGGTGGGAAAGCAGGCCTG**C**GCCTTGGGGTCTCCTCGAGG  CCTGCGCCTTGGGGTCTCCT**C**GAGGTAAAAAGCCCTGGCTG  AAAAGCCCTGGCTGCCCCTG**C**GGGTCGGGCACACAAGGGGC  AGGAGCCCTGGCTGCCCCTG**C**GGGTCGGGCACACAAGCGGC  CCCTGGCTGCCCCTGCGGGT**C**GGGCACACAAGCGGCACATT  CTGCGGGTCGGGCACACAAG**C**GGCACATTGTGTGGGCCCCC  CGGCACATTGTGTGGGCCCC**C**CACGTGTGCACACACACGAA  CACATTGTGTGGGCCCCCCA**C**GTGTGCACACACACGAACAC  CACACACACGAACACACACA**C**ATATAATGGGCCACTCTGTC  TCCCTGCCCTCCCCTCCCCT**C**GCAGCCCTCCCGCTCCTCCC  CCCTCCCCTCGCAGCCCTCC**C**GCTCCTCCCCTCTGGCCCGG  CAGGCTTGGGAAGCCTGTGG**C**CTGGCCCGCCTGGCGCCGCC  GGGAAGCCTGTGGCCTGGCC**C**GCCTGGCGCCGCCACTGGAA  CTGTGGCCTGGCCCGCCTGG**C**GCCGCCACTGGAAACACTGC  TGGCCTGGCCCGCCTGGCGC**C**GCCACTGGAAACACTGCATG  CACTGGAAACACTGCATGCA**C**GTCCCATGCCCGCCCGCCTG  CTGCATGCACGTCCCATGCC**C**GCCCGCCTGCCCGGGCCCCG  ATGCACGTCCCATGCCCGCC**C**GCCTGCCCGGGCCCCGCTTA  CCCATGCCCGCCCGCCTGCC**C**GGGCCCCGCTTAGCAAGAGC  CGGGCCCCGCTTAGCAAGAG**C**GATGGGCACGCGTGTGTCCT  CTTAGCAAGAGCGATGGGCA**C**GCGTGTGTCCTGTGACTACA  TAGCAAGAGCGATGGGCACG**C**GTGTGTCCTGTGACTACAAA  GCACTGGGGTTTCTGGAAGC**C**GAAGTGACCCAGTGATGGGT  CACTTCGCTGCGGTCCTCTT**C**GACCAGCCCGGCCAACATGG  GCGGTCCTCTTCGACCAGCC**C**GGCCAACATGGTGAAACCCC  CGGCCAACATGGTGAAACCC**C**GTCTCTACTAAAAATACAAA  AAAATCAGCCAGATGTGGCA**C**GCACCTATAATTCCACCTAC  CACCTATAATTCCACCTACT**C**GGGAGGCTGAAGCAGAATTG  GAAGCAGAATTGCTTGAACC**C**GAGAGGCGGAGGTTGCAGTG  AATTGCTTGAACCCGAGAGG**C**GGAGGTTGCAGTGAGCCGCC  AGGCGGAGGTTGCAGTGAGC**C**GCCGAGATCGCGCCACTGCA  TTGCAGTGAGCCGCCGAGAT**C**GCGCCACTGCACTCCAGCCT  GCAGTGAGCCGCCGAGATCG**C**GCCACTGCACTCCAGCCTGG  TGGGCCACAGCGTGAGACTA**C**GTCATAAAATAAAATAAAAT  CCCCAATGATTCAGCTGATG**C**GCGTTTCTCTACTTGCCCTT  CCTGGAAATTCAACCTGTTT**C**GCAGTTTCTCGAGGAATCAG  GATTGGCTGGGCAGGAACAG**C**GCCGGGGCGTGGGCTGAGCA  TGGCTGGGCAGGAACAGCGC**C**GGGGCGTGGGCTGAGCACAG  GGGCAGGAACAGCGCCGGGG**C**GTGGGCTGAGCACAGCCGCT  GGGCGTGGGCTGAGCACAGC**C**GCTTCGCTCTCTTTGCCACA  TGGGCTGAGCACAGCCGCTT**C**GCTCTCTTTGCCACAGGAAG  ACAGGAAGCCTGAGCTCATT**C**GAGTAGCGGCTCTTCCAAGC  AAGAAGCAGAGGCCGCTGTT**C**GTTTCCTTTAGGTCTTTCCA  TCCTGGAGCAGCGCCCAAAC**C**GTAGTGGCACTGGACCATGT  CCCGGAGCGCGCACAGCCCG**C**GCGGTGCGGGGACCTGCTCT  CGGAGCGCGCACAGCCCGCG**C**GGTGCGGGGACCTGCTCTCT  CGCGCACAGCCCGCGCGGTG**C**GGGGACCTGCTCTCTGAGCC  CTGCTCTCTGAGCCCGCGGG**C**GGTGGGTGGGAGGAAGCATC  TCGTCCGCGGCGACTGGAAC**C**GGGAGGGAGAATCGCACTGG  GGGAGGGAGAATCGCACTGG**C**GGCGGGCAAAGTCCAGAACG  TGTTGGAGATCTGGTGGGTG**C**GGACGGGCGCGGCCAGGAGG  GGAGATCTGGTGGGTGCGGA**C**GGGCGCGGCCAGGAGGGGGT  ATCTGGTGGGTGCGGACGGG**C**GCGGCCAGGAGGGGGTTAAG  CTGGTGGGTGCGGACGGGCG**C**GGCCAGGAGGGGGTTAAGGC  CGGCCAGGAGGGGGTTAAGG**C**GCAGGCGGCGGCGGGGCGGG  GGAGGGGGTTAAGGCGCAGG**C**GGCGGCGGGGCGGGGGCGGG  GGGGGTTAAGGCGCAGGCGG**C**GGCGGGGCGGGGGCGGGCCT  GGTTAAGGCGCAGGCGGCGG**C**GGGGCGGGGGCGGGCCTGGC  AGGCGCAGGCGGCGGCGGGG**C**GGGGGCGGGCCTGGCGGGCG  AGGCGGCGGCGGGGCGGGGG**C**GGGCCTGGCGGGCGCCCTCT  CGGGGCGGGGGCGGGCCTGG**C**GGGCGCCCTCTCCGGGCCCT  GCGGGGGCGGGCCTGGCGGG**C**GCCCTCTCCGGGCCCTTTGT  GGCCTGGCGGGCGCCCTCTC**C**GGGCCCTTTGTTAACAGGCG  CCGGGCCCTTTGTTAACAGG**C**GCGTCCCGGCCAGGCGGAGA  GGGCCCTTTGTTAACAGGCG**C**GTCCCGGCCAGGCGGAGACG  CTTTGTTAACAGGCGCGTCC**C**GGCCAGGCGGAGACGCGGCC  ACAGGCGCGTCCCGGCCAGG**C**GGAGACGCGGCCGCGGCCAT  GCGTCCCGGCCAGGCGGAGA**C**GCGGCCGCGGCCATGGGCGG  GTCCCGGCCAGGCGGAGACG**C**GGCCGCGGCCATGGGCGGGC  CGGCCAGGCGGAGACGCGGC**C**GCGGCCATGGGCGGGCGCGG  GCCAGGCGGAGACGCGGCCG**C**GGCCATGGGCGGGCGCGGGC  GACGCGGCCGCGGCCATGGG**C**GGGCGCGGGCGCGCGGGGCG  CGGCCGCGGCCATGGGCGGG**C**GCGGGCGCGCGGGGCGGCGG  GCCGCGGCCATGGGCGGGCG**C**GGGCGCGCGGGGCGGCGGTG  CGGCCATGGGCGGGCGCGGG**C**GCGCGGGGCGGCGGTGAGGG  GCCATGGGCGGGCGCGGGCG**C**GCGGGGCGGCGGTGAGGGCG  CATGGGCGGGCGCGGGCGCG**C**GGGGCGGCGGTGAGGGCGGC  GCGGGCGCGGGCGCGCGGGG**C**GGCGGTGAGGGCGGCTGGCG  GGCGCGGGCGCGCGGGGCGG**C**GGTGAGGGCGGCTGGCGGGG  GCGCGGGGCGGCGGTGAGGG**C**GGCTGGCGGGGCCGGGGGCG  GCGGCGGTGAGGGCGGCTGG**C**GGGGCCGGGGGCGCCGGGGG  GTGAGGGCGGCTGGCGGGGC**C**GGGGGCGCCGGGGGGGCGCG  GCGGCTGGCGGGGCCGGGGG**C**GCCGGGGGGGCGCGCGGGCC  GCTGGCGGGGCCGGGGGCGC**C**GGGGGGGCGCGCGGGCCGAG  GGCCGGGGGCGCCGGGGGGG**C**GCGCGGGCCGAGCCGGGCCT  CCGGGGGCGCCGGGGGGGCG**C**GCGGGCCGAGCCGGGCCTGA  GGGGGCGCCGGGGGGGCGCG**C**GGGCCGAGCCGGGCCTGAGC  CGCCGGGGGGGCGCGCGGGC**C**GAGCCGGGCCTGAGCCGGGC  GGGGGGCGCGCGGGCCGAGC**C**GGGCCTGAGCCGGGCCCGCG  GGGCCGAGCCGGGCCTGAGC**C**GGGCCCGCGGACCGAGCTGG  AGCCGGGCCTGAGCCGGGCC**C**GCGGACCGAGCTGGGAGAGG  CCGGGCCTGAGCCGGGCCCG**C**GGACCGAGCTGGGAGAGGGG  CCTGAGCCGGGCCCGCGGAC**C**GAGCTGGGAGAGGGGTTCCG  CCGAGCTGGGAGAGGGGTTC**C**GGCCCCCGACGTCGCTGGCG  GGGAGAGGGGTTCCGGCCCC**C**GACGTCGCTGGCGCGGGAAA  AGAGGGGTTCCGGCCCCCGA**C**GTCGCTGGCGCGGGAAAATG  GGGGTTCCGGCCCCCGACGT**C**GCTGGCGCGGGAAAATGTTG  CCGGCCCCCGACGTCGCTGG**C**GCGGGAAAATGTTGGAGATC  GGCCCCCGACGTCGCTGGCG**C**GGGAAAATGTTGGAGATCTG  CGCCTGGCCCACCCCTGGAC**C**GCCCCCGCCCCGCCCCGCCC  CCCGCGACCGAAGCAGGGCG**C**GCAGCAGCGCTGAGTGCCCC  TATCACCATCTATGATCCCA**C**GTCTGCTGAATCAGTTGTGG  AATCAGTTGTGGGGTTTATA**C**GCGGGAGTTGTCGCGTGGTG  TCAGTTGTGGGGTTTATACG**C**GGGAGTTGTCGCGTGGTGGC  GGTTTATACGCGGGAGTTGT**C**GCGTGGTGGCAGCAAAATCG  TTTATACGCGGGAGTTGTCG**C**GTGGTGGCAGCAAAATCGAT  TCGCGTGGTGGCAGCAAAAT**C**GATTGCGCCAAACCTAAAGA  GGTGGCAGCAAAATCGATTG**C**GCCAAACCTAAAGAGCCCCC  CATTTTCTAGGCTGGTACCT**C**GTGGACTCGGACTCCCAAAT  AGGCTGGTACCTCGTGGACT**C**GGACTCCCAAATCAACAAGA  GACTCCCAAATCAACAAGAT**C**GGCTTACTCTCTGCAAAGAA  TGTGTAAAGACCAGGGTTGT**C**GCACGGCGCTCGTACATCGC  TAAAGACCAGGGTTGTCGCA**C**GGCGCTCGTACATCGCAGTC  AGACCAGGGTTGTCGCACGG**C**GCTCGTACATCGCAGTCCTG  CAGGGTTGTCGCACGGCGCT**C**GTACATCGCAGTCCTGAAAC  GTCGCACGGCGCTCGTACAT**C**GCAGTCCTGAAACGGATTGT  CGTACATCGCAGTCCTGAAA**C**GGATTGCGCAGTGAAGTAAC  CCTCCTGCCATCTGGAGCTC**C**GGTCACTTCCATCTAGAGCT  AAGCTAAGAGCAATGGTATA**C**GCACGCACGTGGCTGGAGGG  TAAGAGCAATGGTATACGCA**C**GCACGTGGCTGGAGGGACAG  AGCAATGGTATACGCACGCA**C**GTGGCTGGAGGGACAGGGCA  GGAGACCATACTGTGCCCTC**C**GGTTGACTGGGGTGACCAAG  AGATAACTAATAAAACTCAT**C**GCAAGAGAGTCCTGACAAAC  CGCAAGAGAGTCCTGACAAA**C**GTGACGACTCACCCAGAAAA  GAGAGTCCTGACAAACGTGA**C**GACTCACCCAGAAAAGGGAC  CGACTCACCCAGAAAAGGGA**C**GTCCTCCTGCCATCTGGAGC  GCTGCCCCCAGACCCCTGCT**C**GGTTCCCGCCAGGAAACATC  CCAGACCCCTGCTCGGTTCC**C**GCCAGGAAACATCCGGGTGC  GGTTCCCGCCAGGAAACATC**C**GGGTGCCCGGATGTGACGCC  CCAGGAAACATCCGGGTGCC**C**GGATGTGACGCCACTGACTT  ATCCGGGTGCCCGGATGTGA**C**GCCACTGACTTGCGCATTGT  GATGTGACGCCACTGACTTG**C**GCATTGTGGGGCAGAGAGAA  CATTGTGGGGCAGAGAGAAG**C**GAGGTTTCCATTCTGAGGGA  GAGGTTTCCATTCTGAGGGA**C**GGCGTAGAGTTCGGCCGAAG  GTTTCCATTCTGAGGGACGG**C**GTAGAGTTCGGCCGAAGGAA  CTGAGGGACGGCGTAGAGTT**C**GGCCGAAGGAACCTGACCCA  GGGACGGCGTAGAGTTCGGC**C**GAAGGAACCTGACCCAGGCT  TGAGGGAGGACTGAGGACCC**C**GCCACTCCAAATAGAGAGCC  AGCCCCAAATATTCCAGCGC**C**GCCCTTGCTGCCAGCCCTGG  GCTGCCAGCCCTGGCCCACC**C**GCGGGAAGACGTCTCAGCCT  CTGGCCCACCCGCGGGAAGA**C**GTCTCAGCCTGGGCTGCCCC  GTGTGGGGACCTAGACAGGG**C**GAGGCATCTCCATATTATCT  CACCCCACCCATTTTCCACC**C**GGAGCATGAAGCACGTGGGG  TCCACCCGGAGCATGAAGCA**C**GTGGGGAATGTTCCCCTTTC  CAGTCTGCATCAATCACCCC**C**GTGTCCCATTCCAAGAACAT  CCTGGCTTTTCTGGCCTCTC**C**GAGTTTGTGCCTATAGATTT  AAGCTTCCAGCTAGGAAGAC**C**GGTATGGTGATGTTCCTCAT  AAAAAAAAAATGGCAAGGGC**C**GGCAGAGGGGTTCTCTACCT  ACCAGCAACACTCCTTTCAA**C**GCAAATTCCAGCCTGCTGGA  GCAAATTCCAGCCTGCTGGA**C**GTTATGCATAATCCACGTAC  TGGACGTTATGCATAATCCA**C**GTACGTCCCACCCCGCCATG  CGTTATGCATAATCCACGTA**C**GTCCCACCCCGCCATGATGG  AATCCACGTACGTCCCACCC**C**GCCATGATGGCGCCATGCGG  GTCCCACCCCGCCATGATGG**C**GCCATGCGGAATTTTTTTGG  CCCGCCATGATGGCGCCATG**C**GGAATTTTTTTGGAAAAACG  AAAACGGGGCCCTACATGTA**C**GGGCTCTTTACACTTCGAAC  TGTACGGGCTCTTTACACTT**C**GAACAGTCGGCTGCAGGACT  GGACTACTTAATGCAAGCTG**C**GGGGCAGAGAAATTATAACA  GGCAGAGAAATTATAACAAG**C**GACCACTACATCTACTGTAT  TTTGTGGGCACCTGCAAGGG**C**GCCTCGTGATCTCCGGACTT  GGGCACCTGCAAGGGCGCCT**C**GTGATCTCCGGACTTTATCT  TCCGGACTTTATCTAGATCT**C**GACGGTATCGATAAGCTTGA  TATCTAGATCTCGACGGTAT**C**GATAAGCTTGATATCGAATT  CAATCAGCACCGGCTCAGGA**C**GAAACCATCAAGATGGGTGC  ACCATCAAGATGGGTGCCCT**C**GCGGTGTTCGCCGTCGCTTG  CATCAAGATGGGTGCCCTCG**C**GGTGTTCGCCGTCGCTTGCC  ATGGGTGCCCTCGCGGTGTT**C**GCCGTCGCTTGCCTCGCGGC  GGTGCCCTCGCGGTGTTCGC**C**GTCGCTTGCCTCGCGGCAGT  GTGTTCGCCGTCGCTTGCCT**C**GCGGCAGTGGCGTCGGTTGC  GTTCGCCGTCGCTTGCCTCG**C**GGCAGTGGCGTCGGTTGCGC  CGCTTGCCTCGCGGCAGTGG**C**GTCGGTTGCGCATGCGGCTG  TTGCCTCGCGGCAGTGGCGT**C**GGTTGCGCATGCGGCCGACA  CGCGGCAGTGGCGTCGGTTG**C**GCATGCGGCAGACAATTACC  AGTGGCGTCGGTTGCGCATG**C**GGCCGACAATTACCAGCAAC  TGTCGCTTGCACCCCAACCC**C**GCGTGTGTGGGGTGCGGGCA  TCGCTTGCACCCCAACCCCG**C**GTGTGTGGGGTGCGGGCACG  AACCCCGCGTGTGTGGGGTG**C**GGGCACGCGGCCCAGGCCTG  GCGTGTGTGGGGTGCGGGCA**C**GCGGCCCAGGCCTGCTGTTG  GTTGACCTTCTCACATGCAA**C**GCTCACCCCCGCCCCGTACT  TCACATGCAACGCTCACCCC**C**GCCCCGTACTAATTTCCATG  TGCAACGCTCACCCCCGCCC**C**GTACTAATTTCCATGCCTAA  CAGATCCGCGGCGCCAACCC**C**GCAGCCGGCGTGGGCCACCA  CGCGGCGCCAACCCCGCAGC**C**GGCGTGGGCCACCACCGCCT  GGCGCCAACCCCGCAGCCGG**C**GTGGGCCACCACCGCCTGCT  CAGCCGGCGTGGGCCACCAC**C**GCCTGCTCACCGCCGCCAGC  GGCCACCACCGCCTGCTCAC**C**GCCGCCAGCGAGCGCGCCAT  CACCACCGCCTGCTCACCGC**C**GCCAGCGAGCGCGCCATCGC  CGCCTGCTCACCGCCGCCAG**C**GAGCGCGCCATCGCCACCCG  TGCTCACCGCCGCCAGCGAG**C**GCGCCATCGCCACCCGCCGC  CTCACCGCCGCCAGCGAGCG**C**GCCATCGCCACCCGCCGCCG  GCCGCCAGCGAGCGCGCCAT**C**GCCACCCGCCGCCGCGCCCA  GCGAGCGCGCCATCGCCACC**C**GCCGCCGCGCCCAGGCCGCC  AGCGCGCCATCGCCACCCGC**C**GCCGCGCCCAGGCCGCCGTC  GCGCCATCGCCACCCGCCGC**C**GCGCCCAGGCCGCCGTCAGT  GCCATCGCCACCCGCCGCCG**C**GCCCAGGCCGCCGTCAGTGC  ACCCGCCGCCGCGCCCAGGC**C**GCCGTCAGTGCCGAGCTGGC  CGCCGCCGCGCCCAGGCCGC**C**GTCAGTGCCGAGCTGGCGGA  GCCCAGGCCGCCGTCAGTGC**C**GAGCTGGCGGAGCGGCCGGC  CGCCGTCAGTGCCGAGCTGG**C**GGAGCGGCCGGCTGTGTTCC  TCAGTGCCGAGCTGGCGGAG**C**GGCCGGCTGTGTTCCAGGCA  GAGAGAAAGAGTAGAGCAAG**C**GGGGGTGGGCCTTGTGGGGA  GGGGTGGGCCTTGTGGGGAG**C**GGGCGTCAGTGGAAGAAAGT  TGGGCCTTGTGGGGAGCGGG**C**GTCAGTGGAAGAAAGTGGTC  GAAGAAAGTGGTCTGGGCGG**C**GTTGGGGGCGCGCCGGGGCG  GGTCTGGGCGGCGTTGGGGG**C**GCGCCGGGGCGGGGACGTGG  GGGCGGCGTTGGGGGCGCGC**C**GGGGCGGGGACGTGGATTCG  GCGTTGGGGGCGCGCCGGGG**C**GGGGACGTGGATTCGACGGG  CCGGGGCGGGGACGTGGATT**C**GACGGGATAGGTTCGGCTCG  GGGCGGGGACGTGGATTCGA**C**GGGATAGGTTCGGCTCGTGT  TGGATTCGACGGGATAGGTT**C**GGCTCGTGTCGTTTGTCCTT  TCGACGGGATAGGTTCGGCT**C**GTGTCGTTTGTCCTTTTGGC  GGGATAGGTTCGGCTCGTGT**C**GTTTGTCCTTTTGGCTCACT  GTCCTTTTGGCTCACTGTGT**C**GCTTGCACCCCAACCCCGCG  GGAGTGTTGCTGGTAATTGT**C**GGCCGCATGCGCAACCGACG  TGTTGCTGGTAATTGTCGGC**C**GCATGCGCAACCGACGCCAC  TGGTAATTGTCGGCCGCATG**C**GCAACCGACGCCACTGCCGC  TTGTCGGCCGCATGCGCAAC**C**GACGCCACTGCCGCGAGGCA  TCGGCCGCATGCGCAACCGA**C**GCCACTGCCGCGAGGCAAGC  TGCGCAACCGACGCCACTGC**C**GCGAGGCAAGCGACGGCGAA  CGCAACCGACGCCACTGCCG**C**GAGGCAAGCGACGGCGAACA  CGCCACTGCCGCGAGGCAAG**C**GACGGCGAACACCGCGAGGG  CACTGCCGCGAGGCAAGCGA**C**GGCGAACACCGCGAGGGCAC  TGCCGCGAGGCAAGCGACGG**C**GAACACCGCGAGGGCACCCA  AGGCAAGCGACGGCGAACAC**C**GCGAGGGCACCCATCTTGAT  GCAAGCGACGGCGAACACCG**C**GAGGGCACCCATCTTGATGG  TTGATGGTTTCGTCCTGAGC**C**GGTGCTGATTGCCCCGATCC  CTGATTGCCCCGATCCCAAA**C**GATCCCTTGACAGCGTTGGC  CCCAAACGATCCCTTGACAG**C**GTTGGCCTAATTCGATATCA  TTGACAGCGTTGGCCTAATT**C**GATATCAAGCTTATCGATAC  GATATCAAGCTTATCGATAC**C**GTCGAGATCTAGATAAAGTC  ATCAAGCTTATCGATACCGT**C**GAGATCTAGATAAAGTCCGG  GTCGAGATCTAGATAAAGTC**C**GGAGATCACGAGGCGCCCTT  TAGATAAAGTCCGGAGATCA**C**GAGGCGCCCTTGCAGGTGCC  AAAGTCCGGAGATCACGAGG**C**GCCCTTGCAGGTGCCCACAA  ACAAAATAGCTTAGCAGAGG**C**GCAAGGTCTTATATACAGTA  TTGTTATAATTTCTCTGCCC**C**GCAGCTTGCATTAAGTAGTC  TAGTCCTGCAGCCGACTGTT**C**GAAGTGTAAAGAGCCCGTAC  TGTTCGAAGTGTAAAGAGCC**C**GTACATGTAGGGCCCCGTTT  CCGTTTTTCCAAAAAAATTC**C**GCATGGCGCCATCATGGCGG  TCCAAAAAAATTCCGCATGG**C**GCCATCATGGCGGGGTGGGA  GCCATCATGGCGGGGTGGGA**C**GTACGTGGATTATGCATAAC  TCATGGCGGGGTGGGACGTA**C**GTGGATTATGCATAACGTCC  CAGTGAGCCAAAAGGACAAA**C**GACACGAGCCGAACCTATCC  AGCCAAAAGGACAAACGACA**C**GAGCCGAACCTATCCCGTCG  AAAGGACAAACGACACGAGC**C**GAACCTATCCCGTCGAATCC  ACGAGCCGAACCTATCCCGT**C**GAATCCACGTCCCCGCCCCG  AACCTATCCCGTCGAATCCA**C**GTCCCCGCCCCGGCGCGCCC  TCCCGTCGAATCCACGTCCC**C**GCCCCGGCGCGCCCCCAACG  TCGAATCCACGTCCCCGCCC**C**GGCGCGCCCCCAACGCCGCC  AATCCACGTCCCCGCCCCGG**C**GCGCCCCCAACGCCGCCCAG  CCGCCCCGGCGCGCCCCCAA**C**GCCGCCCAGACCACTTTCTT  CCCCGGCGCGCCCCCAACGC**C**GCCCAGACCACTTTCTTCCA  CACTTTCTTCCACTGACGCC**C**GCTCCCCACAAGGCCCACCC  CTCCCCACAAGGCCCACCCC**C**GCTTGCTCTACTCTTTCTCT  TTGCCTGGAACACAGCCGGC**C**GCTCCGCCAGCTCGGCACTG  AGCCGGCCGCTCCGCCAGCT**C**GGCACTGACGGCGGCCTGGG  CTCCGCCAGCTCGGCACTGA**C**GGCGGCCTGGGCGCGGCGGC  CGCCAGCTCGGCACTGACGG**C**GGCCTGGGCGCGGCGGCGGG  GGCACTGACGGCGGCCTGGG**C**GCGGCGGCGGGTGGCGATGG  CACTGACGGCGGCCTGGGCG**C**GGCGGCGGGTGGCGATGGCG  TGACGGCGGCCTGGGCGCGG**C**GGCGGGTGGCGATGGCGCGC  CGGCGGCCTGGGCGCGGCGG**C**GGGTGGCGATGGCGCGCTCG  CTGGGCGCGGCGGCGGGTGG**C**GATGGCGCGCTCGCTGGCGG  GCGGCGGCGGGTGGCGATGG**C**GCGCTCGCTGGCGGCGGTGA  GGCGGCGGGTGGCGATGGCG**C**GCTCGCTGGCGGCGGTGAGC  GGCGATGGCGCGCTCGCTGG**C**GGCGGTGAGCAGGCGGTGGT  GATGGCGCGCTCGCTGGCGG**C**GGTGAGCAGGCGGTGGTGGC  CGCTGGCGGCGGTGAGCAGG**C**GGTGGTGGCCCACGCCGGCT  GAGCAGGCGGTGGTGGCCCA**C**GCCGGCTGCGGGGTTGGCGC  CAGGCGGTGGTGGCCCACGC**C**GGCTGCGGGGTTGGCGCCGC  GTGGTGGCCCACGCCGGCTG**C**GGGGTTGGCGCCGCGGATCT  CACGCCGGCTGCGGGGTTGG**C**GCCGCGGATCTGCGTCTTAA  GCCGGCTGCGGGGTTGGCGC**C**GCGGATCTGCGTCTTAAACT  CGGCTGCGGGGTTGGCGCCG**C**GGATCTGCGTCTTAAACTGA  GGGTTGGCGCCGCGGATCTG**C**GTCTTAAACTGACTGTCCTC  TCAACAGCAGGCCTGGGCCG**C**GTGCCCGCACCCCACACACG  GCAGGCCTGGGCCGCGTGCC**C**GCACCCCACACACGCGGGTT  GCGTGCCCGCACCCCACACA**C**GCGGGTTGGGGTGCAAGCGA  GTGCCCGCACCCCACACACG**C**GGGTTGGGGTGCAAGCGACA  TCACCCCTGCACTGCCTCCT**C**GCTCCACCCCAGCACCTTTC  CACCTTTCCATCGACACCTC**C**GCCCTCCCTCCGCAGTTCTC  CGACACCTCCGCCCTCCCTC**C**GCAGTTCTCTCCCCGCCTCC  GTCCGGGCACCCTCTGAGGG**C**GCGCGGGCTCGAGACGGAGG  GCGGGCTCGAGACGGAGGGA**C**GCGGGTCAGTGCAGGGTCGC  GACGCGGGTCAGTGCAGGGT**C**GCCAACTGCCCGCTCCCAGA  GTGCAGGGTCGCCAACTGCC**C**GCTCCCAGAGGAGGCTGGGA  CTCCCAGAGGAGGCTGGGAC**C**GGACGCCGCTGACTTCCGCA  AGGAGGCTGGGACCGGACGC**C**GCTGACTTCCGCATGAAGCG  CATGAAGCGCCCCGTCTACG**C**GTGGCCTCTTGGGAGTGGGA  TGGCCTCTTGGGAGTGGGAG**C**GCTCCTCCAATGGCAGCAGG  GCTCCTCCAATGGCAGCAGG**C**GACACGGCTAGGCCTGCTGC  CGACACGGCTAGGCCTGCTG**C**GTTCCCAGCCCCCCGCACTT  CTGCTGCGTTCCCAGCCCCC**C**GCACTTCCCAGGAGCCGTAC  CATTCTGGGAGTAGTAGTTC**C**GGTTGGAGTATCACTGAGTG  ATCACTGAGTGTTTCACGTT**C**GTTCGACAGCTCTTGTGGTA  CTGAGTGTTTCACGTTCGTT**C**GACAGCTCTTGTGGTAAACA  ACAGCTCTTGTGGTAAACAG**C**GTCAAATGCCGTATAGGAAG  TTTCTTCCGTTGCCCCAGTA**C**GGATACATATTTGAATGTAT  AAATAAACAAATAGGGGTTC**C**GCGCACATTTCCCCGAAAAG  ATAAACAAATAGGGGTTCCG**C**GCACATTTCCCCGAAAAGTG  CCCCGAAAAGTGCCACCTGA**C**GTCTAAGAAACCATTATTAT  CATTAACCTATAAAAATAGG**C**GTATCACGAGGCCCTTTCGT  CTATAAAAATAGGCGTATCA**C**GAGGCCCTTTCGTCTTCAAG  GGCGTATCACGAGGCCCTTT**C**GTCTTCAAGAATTCTCATGT  ATAAGCTGATCCTCACAGGC**C**GCACCCAGCTTTTCTTCCGT  GCCGCACCCAGCTTTTCTTC**C**GTTGCCCCAGTACGGATACA  AAGTGCTCATCATTGGAAAA**C**GTTCTTCGGGGCGAAAACTC  CGGAGACCCCAAAAACGTTC**C**GGCGGCAGTGGACCTCAAAG  CGGCCCACAACCTGGCCCGA**C**GTAGAAAGGACTACCGACGA  CCCTTGCTATTCCACAATGT**C**GTCTTACACCATTGAGTCGT  ATCTGTCTTGTCACCATTTT**C**GTCCTCCCAACATGGGGCAA  TGGGCATACCCATGTTGTCA**C**GTCACTCAGCTCCGCGCTCA  GTTGTCACGTCACTCAGCTC**C**GCGCTCAACACCTTCTCGCG  TGTCACGTCACTCAGCTCCG**C**GCTCAACACCTTCTCGCGTT  CTCCGCGCTCAACACCTTCT**C**GCGTTGGAAAACATTAGCGA  CTCGCGTTGGAAAACATTAG**C**GACATTTACCTGGTGAGCAA  CTGGTGAGCAATCAGACATG**C**GACGGCTTTAGCCTGGCCTC  TTTAAAGTTCTGCTATGTGG**C**GCGGTATTATCCCGTGTTGA  GTATTATCCCGTGTTGACGC**C**GGGCAAGAGCAACTCGGTCG  CCGGGCAAGAGCAACTCGGT**C**GCCGATTTCCGTGTCGCCCT  AATAACCCCTACTGGGGCAA**C**GGAAGAAAAGCTGGGTGCGG  AACGGAAGAAAAGCTGGGTG**C**GGCCTGTGAGGATCAGCTTA  GCCTGTGAGGATCAGCTTAT**C**GATGATAAGCTGTCAAACAT  AACATGAGAATTCTTGAAGA**C**GAAAGGGCCTCGTGATACGC  TCTTGAAGACGAAAGGGCCT**C**GTGATACGCCTATTTTTATA  GACGAAAGGGCCTCGTGATA**C**GCCTATTTTTATAGGTTAAT  GATAATAATGGTTTCTTAGA**C**GTCAGGTGGCACTTTTCGGG  AGACGTCAGGTGGCACTTTT**C**GGGGAAATGTGCGCGGAACC  ACTTTTCGGGGAAATGTGCG**C**GGAACCCCTATTTGTTTATT  CCGGACGTTTTTGGGGTCTC**C**GGACACCATCTCTATGTCTT  TCTTGGCCCTGATCCTGAGC**C**GCCCGGGGCTCCTGGTCTTC  GGCCCTGATCCTGAGCCGCC**C**GGGGCTCCTGGTCTTCCGCC  GCCCGGGGCTCCTGGTCTTC**C**GCCTCCTCGTCCTCGTCCTC  CTCGTCCTCGTCCTCTTCCC**C**GTCCTCGTCCATGGTTATCA  ATTGCTCACCAGGTAAATGT**C**GCTAATGTTTTCCAACGCGA  GTCGCTAATGTTTTCCAACG**C**GAGAAGGTGTTGAGCGCGGA  CAACGCGAGAAGGTGTTGAG**C**GCGGAGCTGAGTGACGTGAC  ACGCGAGAAGGTGTTGAGCG**C**GGAGCTGAGTGACGTGACAA  TTGAGCGCGGAGCTGAGTGA**C**GTGACAACATGGGTATGCCC  ATTGCCCCATGTTGGGAGGA**C**GAAAATGGTGACAAGACAGA  CCAGAAATACACCAACAGCA**C**GCATGTGAATTTAAGGAGGC  GGCTTTTGGGCTCTGCCCCT**C**GCTGCTCCCGGCGTTTGGCG  CCTGGAGGAGAAGACAGAAA**C**GGACTCGCCACGCTTGCCCT  GGAGAAGACAGAAACGGACT**C**GCCACGCTTGCCCTCCCCGC  AGACAGAAACGGACTCGCCA**C**GCTTGCCCTCCCCGCCACGC  CACGCTTGCCCTCCCCGCCA**C**GCCCCGCTCGGTTCGTTACT  CCTCCCCGCCACGCCCCGCT**C**GGTTCGTTACTCTGTAGAGG  CGTTACTCTGTAGAGGCTGG**C**GACCAGCAAAAGAGAAAGTT  AGTTCCCGAGCTTTACAACT**C**TCCGGGAAGCGTAGGGAACG  TCCCGAGCTTTACAACTCTC**C**GGGAAGCGTAGGGAACGTTT  GCGTAGGGAACGTTTCCTCT**C**GGAAAGCTCTTCGCCGCGAT  TTGCAACCCTGCGGGCCGCG**C**GGTCGCGCTTTCTCTGCCCT  AACCCTGCGGGCCGCGCGGT**C**GCGCTTTCTCTGCCCTCCGC  CCCTGCGGGCCGCGCGGTCG**C**GCTTTCTCTGCCCTCCGCCG  GTCGCGCTTTCTCTGCCCTC**C**GCCGGGTGGACCTGGAGCGC  GAGCGCTTGAGCGGTCGGCG**C**GCCTGGAGCAGCCAGGCGGG  GGGACGGCAAGAGAGGAGGG**C**GGGATGTGCCACACATCTTT  TCCTCCAACAAGAATTTAGA**C**GCTAGGTCCAATTATCACTC  AGGTCCAATTATCACTCCAC**C**GCGCGCACTTTCCGCAGGAG  GTCCAATTATCACTCCACCG**C**GCGCACTTTCCGCAGGAGCG  CCAATTATCACTCCACCGCG**C**GCACTTTCCGCAGGAGCGAT  ACTCCACCGCGCGCACTTTC**C**GCAGGAGCGATGTGATCCGT  TCCGCAGGAGCGATGTGATC**C**GTTATCATAACTGCGGACCT  GTGATCCGTTATCATAACTG**C**GGACCTGGGGTTCCACGTGG  ACTGCGGACCTGGGGTTCCA**C**GTGGAAGACGATTGGGATTT  CTGGGGTTCCACGTGGAAGA**C**GATTGGGATTTCACTGGCCG  TGGGGTTAGTGGACTCGAGA**C**GAAAGGCAGGACATGACAGA  AAAAGTGAGTCAGGGTTACC**C**GGAGGAACCACGGGGAAAGT  AGGGTTACCCGGAGGAACCA**C**GGGGAAAGTGCGCTTCTGAG  GAGGAACCACGGGGAAAGTG**C**GCTTCTGAGACTCTTGACAG  GAGACTCTTGACAGCCATTT**C**GTTCCCTTCCAAGCCAGATG  CCAAGAGTGTTGAAAGGCCA**C**GACTTCCCTCAGTTTCTCCA  GGATGGTATAGGGAGTGGCC**C**GTAGTATTTTTCCAGTGACG  CCGTAGTATTTTTCCAGTGA**C**GATGTCTCTCCATTGTTTTC  TTGCAGGCTTACAGGCTTTC**C**GCCGCTCCCCGTTGGCAGCC  GGCTTACAGGCTTTCCGCCG**C**TCCCCGTTGGCAGCCTTGGT  ACAGGCTTTCCGCCGCTCCC**C**GTTGGCAGCCTTTGTCGAAT  GGGCAGACGACCCCAGGCAT**C**GCGCACGTCCAGCCGCGCCC  AGGCATCGCGCACGTCCAGC**C**GCGCCCCGGCCCGGTGCAGC  GGTGAGAGTGGCAGGGTCTG**C**GCAGTTGGGCTCCGCGCCGT  GGGTCTGCGCAGTTGGGCTC**C**GCGCCGTGGAGCAGCAGCAG  TGCGCAGTTGGGCTCCGCGC**C**GTGGAGCAGCAGCAGCTCCG  CCGTGGAGCAGCAGCAGCTC**C**GCCACGCGGGCGCTGCCCAT  GAGCAGCAGCAGCTCCGCCA**C**GCGGGCGCTGCCCATCATCA  TGCAACTGCCGGTCAACACG**C**GCAGCCTACTCAGCGCTGCT  AACACGCGCAGCCTACTCAG**C**GCTGCTCCGGAGGCGCGGGA  GCTCCGGAGGCGCGGGACGC**C**GCCTCCACGTGAGCCGCGCT  GACGCCGCCTCCACGTGAGC**C**GCGCTCCTCCCAGTGCCCGG  GCCCCCAGCGGGCGAGAGAG**C**GGACTGGTCCAAGAGCTGCG  GGGGTTTCGCAGCTGGCGCG**C**GGCGGCAGGGCCTCGAAGCT  GTAGACGTGCAGGATCCGTG**C**GGACCCATCCCCGTACACGC  GATCCGTGCGGACCCATCCC**C**GTACACGCGCCCCTTTTCTT  TGCGGACCCATCCCCGTACA**C**GCGCCCCTTTTCTTCCTCCG  ACGCGCCCCTTTTCTTCCTC**C**GCCCAGGGTCCGCGCGGGCC  CCGCGGCCGGAGAAGGGCAG**C**GGGAAGTGCGCTGTGCAGGA  TGCGCTGTGCAGGAGCCAGA**C**GTCTCAGCCCGTGTGGCCAG  AGGAGCCAGACGTCTCAGCC**C**GTGTGGCCAGAGGTGGCAGG  GTGGCCAGAGGTGGCAGGGG**C**GCGGCCTGAGCGGGGCTGGG  GGCCAGAGGTGGCAGGGGCG**C**GGCCTGAGCGGGGCTGGGGC  CGGCCTGAGCGGGGCTGGGG**C**GCGGGCAGGATTTGGGGCTG  GCGGGCAGGATTTGGGGCTG**C**GCCGAGGGGCGTCCCGACCT  GGCAGGATTTGGGGCTGCGC**C**GAGGGGCGTCCCGACCTGGC  GGCTGCGCCGAGGGGCGTCC**C**GACCTGGCCCTTTGCCACGG  CGCCTTCACCCCCCTGGGTG**C**GTTTCAGCGGTCCCGGAGCC  TCAGCGGTCCCGGAGCCTGG**C**GGGAGCGGGGCCAGGAGCGC  GTCCCGGAGCCTGGCGGGAG**C**GGGGCCAGGAGCGCTCCGCG  GAGCGGGGCCAGGAGCGCTC**C**GCGGCCGGAGAAGGGCAGCG  GCGGGGCCAGGAGCGCTCCG**C**GGCCGGAGAAGGGCAGCGGG  TCGCATGTTCCTTTTGGCGA**C**GTAGCTTCCAGGAAAACGAA  CGACGTAGCTTCCAGGAAAA**C**GAAAGCACGGAAGCCTGGGG  CTTCCAGGAAAACGAAAGCA**C**GGAAGCCTGGGGTCCCCACC  CGGAAGCCTGGGGTCCCCAC**C**GCGCGTTCATCAGGTTCGGG  GAAGCCTGGGGTCCCCACCG**C**GCGTTCATCAGGTTCGGGGC  AGCCTGGGGTCCCCACCGCG**C**GTTCATCAGGTTCGGGGCGC  CACCGCGCGTTCATCAGGTT**C**GGGGCGCTGGGAATGCCCGG  CGCGTTCATCAGGTTCGGGG**C**GCTGGGAATGCCCGGCGCAT  TTCGGGGCGCTGGGAATGCC**C**GGCGCATGCGCAGAGGTAAC  GGGGCGCTGGGAATGCCCGG**C**GCATGCGCAGAGGTAACACG  CTGGGAATGCCCGGCGCATG**C**GCAGAGGTAACACGTCACAT  GCGCATGCGCAGAGGTAACA**C**GTCACATCGCATGTTCCTTT  GCAGAGGTAACACGTCACAT**C**GCATGTTCCTTTTGGCGACG  ACATCGCATGTTCCTTTTGG**C**GACGTAGCTTCCAGGAAAAC  GAGAATTTTTCTAAGCCCTG**C**GCAGACCGCAGCGCCCCGGT  TTTCTAAGCCCTGCGCAGAC**C**GCAGCGCCCCGGTGGGTCCC  CTGCGCAGACCGCAGCGCCC**C**GGTGGGTCCCGGGCGGGCCG  GCGCCCCGGTGGGTCCCGGG**C**GGGCCGGACGCGCCTCCCAA  CCGGTGGGTCCCGGGCGGGC**C**GGACGCGCCTCCCAAGGGCG  TGGGTCCCGGGCGGGCCGGA**C**GCGCCTCCCAAGGGCGCGGG  GGTCCCGGGCGGGCCGGACG**C**GCCTCCCAAGGGCGCGGGTC  CCGGACGCGCCTCCCAAGGG**C**GCGGGTCCGAGGCGCAAGGC  GGACGCGCCTCCCAAGGGCG**C**GGGTCCGAGGCGCAAGGCGA  GCCTCCCAAGGGCGCGGGTC**C**GAGGCGCAAGGCGAGCTGGA  CCAAGGGCGCGGGTCCGAGG**C**GCAAGGCGAGCTGGAGACCC  CGCGGGTCCGAGGCGCAAGG**C**GAGCTGGAGACCCCGAAAAC  CAGTCCCCTTTCACCAAGCC**C**GTCCTCCCCTTCCCGCTGCC  TCCTCCCCTTCCCGCTGCCA**C**GACACTGGTGCCTGCAGCCG  ACGACACTGGTGCCTGCAGC**C**GTGCCAGCTAGGAGGGCGAC  AGCCGTGCCAGCTAGGAGGG**C**GACGTGGACAGGATTTCTCT  CGTGCCAGCTAGGAGGGCGA**C**GTGGACAGGATTTCTCTCTA  CTAGAAAGGATCCTGGGGTC**C**GATTGAGAAGCTGGCTTGAA  GATTGAGAAGCTGGCTTGAA**C**GTTAGACGCGGCGGGCGTGG  AAGCTGGCTTGAACGTTAGA**C**GCGGCGGGCGTGGGTCTGAT  TGTACGCGTGGGTGTGTGCA**C**GTGGGTGTGTACATGTGGGT  TGGGTGTGTACATGTGGGTG**C**GCACGTGGGTGTGTGCGCGC  TGTGTACATGTGGGTGCGCA**C**GTGGGTGTGTGCGCGCGTGT  GGTGCGCACGTGGGTGTGTG**C**GCGCGTGTGTGTGCATGGGT  TGCGCACGTGGGTGTGTGCG**C**GCGTGTGTGTGCATGGGTGC  CGCACGTGGGTGTGTGCGCG**C**GTGTGTGTGCATGGGTGCGT  CGCGTGTGTGTGCATGGGTG**C**GTGCGCATGGGTGCACGCGC  TGTGTGTGCATGGGTGCGTG**C**GCATGGGTGCACGCGCGTGT  GGTGCGTGCGCATGGGTGCA**C**GCGCGTGTGTGCGTGCATGG  TGCGTGCGCATGGGTGCACG**C**GCGTGTGTGCGTGCATGGGC  CGTGCGCATGGGTGCACGCG**C**GTGTGTGCGTGCATGGGCGT  TGGGTGCACGCGCGTGTGTG**C**GTGCATGGGCGTGCAAGTGG  CGCGTGTGTGCGTGCATGGG**C**GTGCAAGTGGGTGCGTGTGC  GGTGTGCACGTGGGTGTGTG**C**GTGTGGGTGTGTGCATGCGT  TGCGTGTGGGTGTGTGCATG**C**GTGTACACACAGCTGCGCGG  TGCGTGTACACACAGCTGCG**C**GGAAGAACCCGGGACGCAGC  CACAGCTGCGCGGAAGAACC**C**GGGACGCAGCCACCCTTCCT  GGCCGCGTCCCCCGCGCGCG**C**GCGACGTGCACCCGGCGTTC  CCGCGTCCCCCGCGCGCGCG**C**GACGTGCACCCGGCGTTCAG  CGTCCCCCGCGCGCGCGCGA**C**GTGCACCCGGCGTTCAGCGA  GCGCGCGCGCGACGTGCACC**C**GGCGTTCAGCGAGTTCCTCA  CGCGCGCGACGTGCACCCGG**C**GTTCAGCGAGTTCCTCATCA  GACGTGCACCCGGCGTTCAG**C**GAGTTCCTCATCAACACCTA  GAGTTCCTCATCAACACCTACGGAATCCTGAAGCAGCGGCC  CCTACGGAATCCTGAAGCAG**C**GGCCCGACCTGCGCGCCAAC  GGAATCCTGAAGCAGCGGCC**C**GACCTGCGCGCCAACCCCCT  TGAAGCAGCGGCCCGACCTG**C**GCGCCAACCCCCTGCACAGC  AAGCAGCGGCCCGACCTGCG**C**GCCAACCCCCTGCACAGCAG  CAACCCCCTGCACAGCAGCC**C**GGCCGCGCTGCGCAAGCTGG  CCTGCACAGCAGCCCGGCCG**C**GCTGCGCAAGCTGGTCATCG  ACAGCAGCCCGGCCGCGCTG**C**GCAAGCTGGTCATCGACGTG  GCGCTGCGCAAGCTGGTCAT**C**GACGTGGTGCCCCCCAAGTT  CTGCGCAAGCTGGTCATCGA**C**GTGGTGCCCCCCAAGTTCCT  GTGCCCCCCAAGTTCCTGGG**C**GACTCGCTGCTGCTGCTCAA  CCCCAAGTTCCTGGGCGACT**C**GCTGCTGCTGCTCAACTGCC  CTGCTGCTCAACTGCCTGTG**C**GAGCTCTCCAAGGAGGACGG  TGCGAGCTCTCCAAGGAGGA**C**GGCAAGCCCCTCTTCGCCTG  GAGGACGGCAAGCCCCTCTT**C**GCCTGGTGAGCCGCCCCGCG  CCCCTCTTCGCCTGGTGAGC**C**GCCCCGCGCCCGCCGCCTTG  CTTCGCCTGGTGAGCCGCCC**C**GCGCCCGCCGCCTTGCCTGC  TCGCCTGGTGAGCCGCCCCG**C**GCCCGCCGCCTTGCCTGCAG  CTGGTGAGCCGCCCCGCGCC**C**GCCGCCTTGCCTGCAGTAAA  GTGAGCCGCCCCGCGCCCGC**C**GCCTTGCCTGCAGTAAACGC  GCCGCCTTGCCTGCAGTAAA**C**GCGTTTGTTCCAACCCGGGG  CGCCTTGCCTGCAGTAAACG**C**GTTTGTTCCAACCCGGGGCC  TAAACGCGTTTGTTCCAACC**C**GGGGCCGCGGTGCCTCCTGC  CGTTTGTTCCAACCCGGGGC**C**GCGGTGCCTCCTGCGCGTCC  TTTGTTCCAACCCGGGGCCG**C**GGTGCCTCCTGCGCGTCCCC  CGGGGCCGCGGTGCCTCCTG**C**GCGTCCCCCCGGAGGGGAAA  GGGCCGCGGTGCCTCCTGCG**C**GTCCCCCCGGAGGGGAAAGG  GTGCCTCCTGCGCGTCCCCC**C**GGAGGGGAAAGGGCCGCGTC  CCCCCCGGAGGGGAAAGGGC**C**GCGTCCCCCGCGCGCGCGCG  CCCCGGAGGGGAAAGGGCCG**C**GTCCCCCGCGCGCGCGCGAC  GGGGAAAGGGCCGCGTCCCC**C**GCGCGCGCGCGACGTGCACC  GGAAAGGGCCGCGTCCCCCG**C**GCGCGCGCGACGTGCACCCG  AAAGGGCCGCGTCCCCCGCG**C**GCGCGCGACGTGCACCCGGC  AGGGCCGCGTCCCCCGCGCG**C**GCGCGACGTGCACCCGGCGT  TGGGGACCGGGGAAAGCGCT**C**TTGGGACCCAGGCCGAATGC  CAAGTAGGATCGCGGGACCA**C**AGGTTTGATTATAAACGCCA  TTTGGTCAGAGCGATAGCTT**C**TCCGATTGCCTCAGAGCAAG  CACGTCTGGTACAGTCATCA**C**AAGCCTGTTCGGCGACGACC  GGCGTACACACTCAATCCTC**C**GGGGCGGAGTGCGTGTCCTC  ACACACTCAATCCTCCGGGG**C**GGAGTGCGTGTCCTCCCACC  CAATCCTCCGGGGCGGAGTG**C**GTGTCCTCCCACCGTGCTGG  CGGAGTGCGTGTCCTCCCAC**C**GTGCTGGCGCTGAACTGACT  GTGTCCTCCCACCGTGCTGG**C**GCTGAACTGACTGTCCGCTG  CTGGCGCTGAACTGACTGTC**C**GCTGCCAAGGGAAGTGACAG  CTGCCAAGGGAAGTGACAGC**C**GCAGCCGGGCTCTCAGCCAG  AGGGAAGTGACAGCCGCAGC**C**GGGCTCTCAGCCAGCGGCCG  GCAGCCGGGCTCTCAGCCAG**C**GGCCGGGCGCCCCGCGGACC  CCGGGCTCTCAGCCAGCGGC**C**GGGCGCCCCGCGGACCATGC  GCTCTCAGCCAGCGGCCGGG**C**GCCCCGCGGACCATGCTCTC  CAGCCAGCGGCCGGGCGCCC**C**GCGGACCATGCTCTCCAGTA  GCCAGCGGCCGGGCGCCCCG**C**GGACCATGCTCTCCAGTACG  ATGCTCTCCAGTACGCAGAA**C**GCGGGCGGCTCCTATCAGCG  GCTCTCCAGTACGCAGAACG**C**GGGCGGCTCCTATCAGCGGG  TCCAGTACGCAGAACGCGGG**C**GGCTCCTATCAGCGGGTCCG  ACGCGGGCGGCTCCTATCAG**C**GGGTCCGCGGGGCGCTTGAT  GCGGCTCCTATCAGCGGGTC**C**GCGGGGCGCTTGATACACAG  GGCTCCTATCAGCGGGTCCG**C**GGGGCGCTTGATACACAGGT  CTATCAGCGGGTCCGCGGGG**C**GCTTGATACACAGGTAAAGT  AGTGGGGAGAGGTGAGTCCTCGAACCCTGAACGTGGACGGA  GTGAGTCCTCGAACCCTGAA**C**GTGGACGGACGAGCCCTTGT  CCTCGAACCCTGAACGTGGA**C**GGACGAGCCCTTGTTTCAGT  GAACCCTGAACGTGGACGGA**C**GAGCCCTTGTTTCAGTAGCC  GTTTCAGTAGCCAACACACA**C**GGGACAGACAGACCGGCGTA  CACACACGGGACAGACAGAC**C**GGCGTACACACTCAATCCTC  ACACGGGACAGACAGACCGG**C**GTACACACTCAATCCTCCGG  TTCCTTCAACCCCCGTTTCT**C**GCCCCCAGCGCGCGCTCCCG  CCCCCGTTTCTCGCCCCCAG**C**GCGCGCTCCCGGCGCCGGCC  CCCGTTTCTCGCCCCCAGCG**C**GCGCTCCCGGCGCCGGCCCC  CGTTTCTCGCCCCCAGCGCG**C**GCTCCCGGCGCCGGCCCCGC  GCCCCGCCCCCTCACCCAGG**C**GGCGCCGTAAAGCCGGAGAA  CCGCCCCCTCACCCAGGCGG**C**GCCGTAAAGCCGGAGAAGGG  CCCCCTCACCCAGGCGGCGC**C**GTAAAGCCGGAGAAGGGGCG  CCCAGGCGGCGCCGTAAAGC**C**GGAGAAGGGGCGGGGTCTCA  CCGTAAAGCCGGAGAAGGGG**C**GGGGTCTCAGCTCCTACTTC  TCAGCTCCTACTTCATTCTA**C**GGCCGAGACCGGAGGATGTT  CTCCTACTTCATTCTACGGC**C**GAGACCGGAGGATGTTCCCT  CTTCATTCTACGGCCGAGAC**C**GGAGGATGTTCCCTGCTCAG  TTCCCTGCTCAGGAGGAGGC**C**GACAGGACCGTGTTTGTTGG  CAGGAGGAGGCCGACAGGAC**C**GTGTTTGTTGGGAATTTAGA  TTGTTGGGAATTTAGAGGCC**C**GAGTTCGGGAAGAGATTCTG  GGAATTTAGAGGCCCGAGTT**C**GGGAAGAGATTCTGTACGAG  GTTCGGGAAGAGATTCTGTA**C**GAGCTGTTCCTTCAGGTACC  CGAGCTGTTCCTTCAGGTAC**C**GTCTCTGGGAAGGGGCGGCG  GTACCGTCTCTGGGAAGGGG**C**GGCGGAGGGGCGGAGCACGT  CCGTCTCTGGGAAGGGGCGG**C**GGAGGGGCGGAGCACGTCGG  GGGAAGGGGCGGCGGAGGGG**C**GGAGCACGTCGGGCCGGAAA  GGCGGCGGAGGGGCGGAGCA**C**GTCGGGCCGGAAACATAGCG  GGCGGAGGGGCGGAGCACGT**C**GGGCCGGAAACATAGCGCGC  AGGGGCGGAGCACGTCGGGC**C**GGAAACATAGCGCGCACCTG  ACGTCGGGCCGGAAACATAG**C**GCGCACCTGGACCACCCGGA  GTCGGGCCGGAAACATAGCG**C**GCACCTGGACCACCCGGAGC  CACCTGGACCACCCGGAGCC**C**GGCCCCCTTCCGTCCCATCC  CCCGGAGCCCGGCCCCCTTC**C**GTCCCATCCTGAGCAGGGGT  TTAATTTCGTTTCCTCTTGG**C**GCACCTGTCTCCAGGTGAGC  CGCACCTGTCTCCAGGTGAG**C**GGCTGGGGGGTGAAATAAGG  GCTGGGGGGTGAAATAAGGG**C**GCGTTCCCCATCCCCATCCG  TGGGGGGTGAAATAAGGGCG**C**GTTCCCCATCCCCATCCGCG  GCGCGTTCCCCATCCCCATC**C**GCGCTGCAGAGTCCTGGGGT  GCGTTCCCCATCCCCATCCG**C**GCTGCAGAGTCCTGGGGTTG  CTCCCTCGTCGAGTTCCAGG**C**GCTCTAGCCCCAGTCCTGCT  CCCATGAACTTTGCCAGTAA**C**GCAAGGCTGCCACTGACATT  GCTGCCACTGACATTTGTGG**C**GCCAAGACTTGCGCACGCGG  ATTTGTGGCGCCAAGACTTG**C**GCACGCGGATTGCACACATC  GTGGCGCCAAGACTTGCGCA**C**GCGGATTGCACACATCGGCC  GGCGCCAAGACTTGCGCACG**C**GGATTGCACACATCGGCCAC  CGCACGCGGATTGCACACAT**C**GGCCACTTCCTGCACCACGT  ATCGGCCACTTCCTGCACCA**C**GTGCAATGACGCGCGCACGC  TCCTGCACCACGTGCAATGA**C**GCGCGCACGCCTCACGCGCA  CTGCACCACGTGCAATGACG**C**GCGCACGCCTCACGCGCACG  GCACCACGTGCAATGACGCG**C**GCACGCCTCACGCGCACGCC  CACGTGCAATGACGCGCGCA**C**GCCTCACGCGCACGCCGCAT  AATGACGCGCGCACGCCTCA**C**GCGCACGCCGCATAACGTCT  TGACGCGCGCACGCCTCACG**C**GCACGCCGCATAACGTCTGA  GCGCGCACGCCTCACGCGCA**C**GCCGCATAACGTCTGAGGCG  CGCACGCCTCACGCGCACGC**C**GCATAACGTCTGAGGCGCGC  CTCACGCGCACGCCGCATAA**C**GTCTGAGGCGCGCACGCCTC  ACGCCGCATAACGTCTGAGG**C**GCGCACGCCTCACGCGCGCC  GCCGCATAACGTCTGAGGCG**C**GCACGCCTCACGCGCGCCCC  CATAACGTCTGAGGCGCGCA**C**GCCTCACGCGCGCCCCGCAC  TCTGAGGCGCGCACGCCTCA**C**GCGCGCCCCGCACGCGCGCC  TGAGGCGCGCACGCCTCACG**C**GCGCCCCGCACGCGCGCCCG  AGGCGCGCACGCCTCACGCG**C**GCCCCGCACGCGCGCCCGCC  CGCACGCCTCACGCGCGCCC**C**GCACGCGCGCCCGCCTCACG  CGCCTCACGCGCGCCCCGCA**C**GCGCGCCCGCCTCACGCGCG  CCTCACGCGCGCCCCGCACG**C**GCGCCCGCCTCACGCGCGCC  TCACGCGCGCCCCGCACGCG**C**GCCCGCCTCACGCGCGCCCC  GCGCGCCCCGCACGCGCGCC**C**GCCTCACGCGCGCCCCGCAC  CCGCACGCGCGCCCGCCTCA**C**GCGCGCCCCGCACGCACATG  GCACGCGCGCCCGCCTCACG**C**GCGCCCCGCACGCACATGAC  ACGCGCGCCCGCCTCACGCG**C**GCCCCGCACGCACATGACGG  CGCCCGCCTCACGCGCGCCC**C**GCACGCACATGACGGCTTGG  CGCCTCACGCGCGCCCCGCA**C**GCACATGACGGCTTGGCTTA  CGCGCCCCGCACGCACATGA**C**GGCTTGGCTTACCTGTAATG  TGGCTTACCTGTAATGGGTG**C**GCTTCGCTTTGCGTTCCTCG  TACCTGTAATGGGTGCGCTT**C**GCTTTGCGTTCCTCGCTTGG  AATGGGTGCGCTTCGCTTTG**C**GTTCCTCGCTTGGCCTTGCG  GCGCTTCGCTTTGCGTTCCT**C**GCTTGGCCTTGCGTTCCTAG  GCGTTCCTCGCTTGGCCTTG**C**GTTCCTAGCTTGGCTTGGCG  GCGTTCCTAGCTTGGCTTGG**C**GTTAGTCGCTTTGCCTGGTG  TAGCTTGGCTTGGCGTTAGT**C**GCTTTGCCTGGTGTTTCTTG  TGGATTGGCGTTTTCTCCCT**C**GTCGAGTTCCAGGCGCTCTA  CAGTGTCGCTGTGGGTGCCA**C**GCAGGTAAGGAGAGAAGAGC  GGAAAAGGCGCTTGGGGGTG**C**GGGCATGACTCGTGCTCTGT  TCTGTGGATGTGGCAGAAAG**C**GCCAGAAGGCGGAGAGGCAA |

**II. Negative subset contains 1,639 non-methylation segment** samples

| Segment# | ID | Site | Sequence |
| --- | --- | --- | --- |
| 1  2  3  4  5  6  7  8  9  10  11  12  13  14  15  16  17  18  19  20  21  22  23  24  25  26  27  28  29  30  31  32  33  34  35  36  37  38  39  40  41  42  43  44  45  46  47  48  49  50  51  52  53  54  55  56  57  58  59  60  61  62  63  64  65  66  67  68  69  70  71  72  73  74  75  76  77  78  79  80  81  82  83  84  85  86  87  88  89  90  91  92  93  94  95  96  97  98  99  100  101  102  103  104  105  106  107  108  109  110  111  112  113  114  115  116  117  118  119  120  121  122  123  124  125  126  127  128  129  130  131  132  133  134  135  136  137  138  139  140  141  142  143  144  145  146  147  148  149  150  151  152  153  154  155  156  157  158  159  160  161  162  163  164  165  166  167  168  169  170  171  172  173  174  175  176  177  178  179  180  181  182  183  184  185  186  187  188  189  190  191  192  193  194  195  196  197  198  199  200  201  202  203  204  205  206  207  208  209  210  211  212  213  214  215  216  217  218  219  220  221  222  223  224  225  226  227  228  229  230  231  232  233  234  235  236  237  238  239  240  241  242  243  244  245  246  247  248  249  250  251  252  253  254  255  256  257  258  259  260  261  262  263  264  265  266  267  268  269  270  271  272  273  274  275  276  277  278  279  280  281  282  283  284  285  286  287  288  289  290  291  292  293  294  295  296  297  298  299  300  301  302  303  304  305  306  307  308  309  310  311  312  313  314  315  316  317  318  319  320  321  322  323  324  325  326  327  328  329  330  331  332  333  334  335  336  337  338  339  340  341  342  343  344  345  346  347  348  349  350  351  352  353  354  355  356  357  358  359  360  361  362  363  364  365  366  367  368  369  370  371  372  373  374  375  376  377  378  379  380  381  382  383  384  385  386  387  388  389  390  391  392  393  394  395  396  397  398  399  400  401  402  403  404  405  406  407  408  409  410  411  412  413  414  415  416  417  418  419  420  421  422  423  424  425  426  427  428  429  430  431  432  433  434  435  436  437  438  439  440  441  442  443  444  445  446  447  448  449  450  451  452  453  454  455  456  457  458  459  460  461  462  463  464  465  466  467  468  469  470  471  472  473  474  475  476  477  478  479  480  481  482  483  484  485  486  487  488  489  490  491  492  493  494  495  496  497  498  499  500  501  502  503  504  505  506  507  508  509  510  511  512  513  514  515  516  517  518  519  520  521  522  523  524  525  526  527  528  529  530  531  532  533  534  535  536  537  538  539  540  541  542  543  544  545  546  547  548  549  550  551  552  553  554  555  556  557  558  559  560  561  562  563  564  565  566  567  568  569  570  571  572  573  574  575  576  577  578  579  580  581  582  583  584  585  586  587  588  589  590  591  592  593  594  595  596  597  598  599  600  601  602  603  604  605  606  607  608  609  610  611  612  613  614  615  616  617  618  619  620  621  622  623  624  625  626  627  628  629  630  631  632  633  634  635  636  637  638  639  640  641  642  643  644  645  646  647  648  649  650  651  652  653  654  655  656  657  658  659  660  661  662  663  664  665  666  667  668  669  670  671  672  673  674  675  676  677  678  679  680  681  682  683  684  685  686  687  688  689  690  691  692  693  694  695  696  697  698  699  700  701  702  703  704  705  706  707  708  709  710  711  712  713  714  715  716  717  718  719  720  721  722  723  724  725  726  727  728  729  730  731  732  733  734  735  736  737  738  739  740  741  742  743  744  745  746  747  748  749  750  751  752  753  754  755  756  757  758  759  760  761  762  763  764  765  766  767  768  769  770  771  772  773  774  775  776  777  778  779  780  781  782  783  784  785  786  787  788  789  790  791  792  793  794  795  796  797  798  799  800  801  802  803  804  805  806  807  808  809  810  811  812  813  814  815  816  817  818  819  820  821  822  823  824  825  826  827  828  829  830  831  832  833  834  835  836  837  838  839  840  841  842  843  844  845  846  847  848  849  850  851  852  853  854  855  856  857  858  859  860  861  862  863  864  865  866  867  868  869  870  871  872  873  874  875  876  877  878  879  880  881  882  883  884  885  886  887  888  889  890  891  892  893  894  895  896  897  898  899  900  901  902  903  904  905  906  907  908  909  910  911  912  913  914  915  916  917  918  919  920  921  922  923  924  925  926  927  928  929  930  931  932  933  934  935  936  937  938  939  940  941  942  943  944  945  946  947  948  949  950  951  952  953  954  955  956  957  958  959  960  961  962  963  964  965  966  967  968  969  970  971  972  973  974  975  976  977  978  979  980  981  982  983  984  985  986  987  988  989  990  991  992  993  994  995  996  997  998  999  1000  1001  1002  1003  1004  1005  1006  1007  1008  1009  1010  1011  1012  1013  1014  1015  1016  1017  1018  1019  1020  1021  1022  1023  1024  1025  1026  1027  1028  1029  1030  1031  1032  1033  1034  1035  1036  1037  1038  1039  1040  1041  1042  1043  1044  1045  1046  1047  1048  1049  1050  1051  1052  1053  1054  1055  1056  1057  1058  1059  1060  1061  1062  1063  1064  1065  1066  1067  1068  1069  1070  1071  1072  1073  1074  1075  1076  1077  1078  1079  1080  1081  1082  1083  1084  1085  1086  1087  1088  1089  1090  1091  1092  1093  1094  1095  1096  1097  1098  1099  1100  1101  1102  1103  1104  1105  1106  1107  1108  1109  1110  1111  1112  1113  1114  1115  1116  1117  1118  1119  1120  1121  1122  1123  1124  1125  1126  1127  1128  1129  1130  1131  1132  1133  1134  1135  1136  1137  1138  1139  1140  1141  1142  1143  1144  1145  1146  1147  1148  1149  1150  1151  1152  1153  1154  1155  1156  1157  1158  1159  1160  1161  1162  1163  1164  1165  1166  1167  1168  1169  1170  1171  1172  1173  1174  1175  1176  1177  1178  1179  1180  1181  1182  1183  1184  1185  1186  1187  1188  1189  1190  1191  1192  1193  1194  1195  1196  1197  1198  1199  1200  1201  1202  1203  1204  1205  1206  1207  1208  1209  1210  1211  1212  1213  1214  1215  1216  1217  1218  1219  1220  1221  1222  1223  1224  1225  1226  1227  1228  1229  1230  1231  1232  1233  1234  1235  1236  1237  1238  1239  1240  1241  1242  1243  1244  1245  1246  1247  1248  1249  1250  1251  1252  1253  1254  1255  1256  1257  1258  1259  1260  1261  1262  1263  1264  1265  1266  1267  1268  1269  1270  1271  1272  1273  1274  1275  1276  1277  1278  1279  1280  1281  1282  1283  1284  1285  1286  1287  1288  1289  1290  1291  1292  1293  1294  1295  1296  1297  1298  1299  1300  1301  1302  1303  1304  1305  1306  1307  1308  1309  1310  1311  1312  1313  1314  1315  1316  1317  1318  1319  1320  1321  1322  1323  1324  1325  1326  1327  1328  1329  1330  1331  1332  1333  1334  1335  1336  1337  1338  1339  1340  1341  1342  1343  1344  1345  1346  1347  1348  1349  1350  1351  1352  1353  1354  1355  1356  1357  1358  1359  1360  1361  1362  1363  1364  1365  1366  1367  1368  1369  1370  1371  1372  1373  1374  1375  1376  1377  1378  1379  1380  1381  1382  1383  1384  1385  1386  1387  1388  1389  1390  1391  1392  1393  1394  1395  1396  1397  1398  1399  1400  1401  1402  1403  1404  1405  1406  1407  1408  1409  1410  1411  1412  1413  1414  1415  1416  1417  1418  1419  1420  1421  1422  1423  1424  1425  1426  1427  1428  1429  1430  1431  1432  1433  1434  1435  1436  1437  1438  1439  1440  1441  1442  1443  1444  1445  1446  1447  1448  1449  1450  1451  1452  1453  1454  1455  1456  1457  1458  1459  1460  1461  1462  1463  1464  1465  1466  1467  1468  1469  1470  1471  1472  1473  1474  1475  1476  1477  1478  1479  1480  1481  1482  1483  1484  1485  1486  1487  1488  1489  1490  1491  1492  1493  1494  1495  1496  1497  1498  1499  1500  1501  1502  1503  1504  1505  1506  1507  1508  1509  1510  1511  1512  1513  1514  1515  1516  1517  1518  1519  1520  1521  1522  1523  1524  1525  1526  1527  1528  1529  1530  1531  1532  1533  1534  1535  1536  1537  1538  1539  1540  1541  1542  1543  1544  1545  1546  1547  1548  1549  1550  1551  1552  1553  1554  1555  1556  1557  1558  1559  1560  1561  1562  1563  1564  1565  1566  1567  1568  1569  1570  1571  1572  1573  1574  1575  1576  1577  1578  1579  1580  1581  1582  1583  1584  1585  1586  1587  1588  1589  1590  1591  1592  1593  1594  1595  1596  1597  1598  1599  1600  1601  1602  1603  1604  1605  1606  1607  1608  1609  1610  1611  1612  1613  1614  1615  1616  1617  1618  1619  1620  1621  1622  1623  1624  1625  1626  1627  1628  1629  1630  1631  1632  1633  1634  1635  1636  1637  1638  1639 | 381  381  381  381  381  381  381  381  381  381  381  381  776  776  776  776  776  776  776  776  776  776  776  776  776  776  776  776  776  776  776  776  861  861  861  861  861  861  861  861  861  861  861  861  861  861  861  861  861  861  861  861  861  861  861  861  861  861  861  861  861  861  861  861  861  861  861  861  861  861  861  861  861  861  861  861  861  861  861  861  861  861  861  861  861  861  861  861  861  861  861  861  861  861  861  861  861  861  861  862  862  862  862  862  862  862  862  862  862  862  862  862  862  862  862  862  862  862  862  862  862  862  862  862  862  862  862  862  862  862  862  862  862  862  862  862  862  862  862  863  863  863  863  863  863  863  863  863  863  863  863  863  863  863  863  863  863  863  863  863  863  863  863  863  863  863  863  875  875  875  875  875  875  875  875  875  875  875  875  875  875  875  875  875  875  875  875  875  875  875  875  875  875  875  875  875  875  875  875  875  875  875  875  875  875  875  875  875  875  875  875  875  875  875  875  875  875  875  875  875  875  897  897  897  897  897  897  897  897  897  897  897  897  897  897  897  897  897  897  897  897  897  897  897  897  897  897  897  897  897  897  897  897  897  897  897  897  897  897  897  906  906  906  906  906  906  906  906  906  906  906  906  906  906  906  906  906  906  906  906  906  906  906  906  906  906  906  906  906  906  906  906  906  906  906  906  906  906  906  906  906  957  957  957  957  957  957  957  957  957  957  957  957  957  957  957  957  957  957  957  957  957  957  957  957  957  957  957  957  957  957  957  957  957  957  957  957  957  957  1000  1000  1000  1000  1000  1000  1000  1000  1000  1000  1000  1000  1000  1000  1000  1000  1000  1000  1000  1000  1000  1000  1000  1000  1000  1000  1000  1000  1000  1000  1000  1000  1000  1000  1000  1000  1000  1000  1000  1000  1000  1000  1000  1000  1000  1000  1000  1000  1017  1017  1017  1017  1017  1017  1017  1017  1017  1017  1017  1017  1017  1017  1017  1017  1017  1017  1017  1017  1017  1017  1017  1019  1019  1019  1019  1019  1019  1019  1019  1019  1019  1019  1019  1019  1019  1019  1019  1019  1019  1019  1019  1019  1019  1019  1019  1019  1019  1019  1019  1019  1019  1019  1019  1019  1019  1019  1027  1027  1027  1027  1027  1027  1027  1027  1027  1027  1027  1027  1027  1027  1027  1027  1027  1027  1027  1027  1027  1027  1027  1027  1027  1027  1027  1027  1027  1027  1027  1027  1027  1027  1027  1027  1027  1027  1027  1027  1027  1027  1027  1027  1027  1027  1027  1066  1066  1066  1066  1066  1066  1066  1066  1066  1066  1066  1066  1066  1066  1066  1066  1066  1066  1066  1066  1066  1066  1066  1066  1066  1066  1066  1066  1066  1066  1066  1066  1116  1116  1116  1116  1116  1116  1116  1116  1116  1116  1116  1116  1116  1116  1116  1116  1116  1116  1116  1116  1116  1116  1116  1116  1116  1116  1116  1116  1116  1116  1116  1116  1116  1116  1116  1116  1116  1116  1116  1116  1116  1116  1116  1116  1116  1116  1116  1116  1116  1116  1116  1116  1116  1116  1116  1116  1116  1116  1116  1116  1116  1116  1116  1116  1116  1116  1116  1116  1116  1116  1116  1116  1116  1116  1116  1116  1128  1128  1128  1128  1128  1128  1261  1261  1261  1261  1261  1261  1261  1261  1261  1261  1261  1261  1261  1261  1261  1261  1261  1261  1261  1261  1261  1261  1261  1261  1261  1261  1261  1261  1261  1261  1261  1261  1261  1261  1261  1261  1261  1261  1261  1261  1261  1261  1261  1261  1261  1261  1261  1261  1329  1329  1329  1329  1329  1329  1329  1329  1329  1329  1329  1329  1329  1329  1329  1329  1329  1329  1329  1329  1329  1329  1329  1329  1329  1329  1329  1329  1329  1329  1345  1345  1345  1345  1345  1345  1345  1345  1345  1345  1345  1345  1345  1345  1345  1345  1345  1345  1345  1345  1345  1345  1345  1345  1345  1345  1376  1376  1376  1376  1376  1376  1376  1376  1376  1376  1376  1376  1376  1376  1376  1376  1376  1376  1376  1376  1437  1437  1437  1437  1437  1437  1437  1437  1437  1437  1437  1437  1437  1437  1437  1437  1437  1437  1437  1437  1437  1437  1437  1437  1437  1437  1437  1437  1437  1437  1437  1437  1437  1437  1437  1437  1437  1437  1437  1437  1437  1437  1437  1437  1437  1437  1437  1437  1437  1437  1437  1437  1437  1437  1437  1437  1437  1437  1437  1437  1437  1437  1437  1437  1437  1437  1440  1440  1440  1440  1440  1440  1440  1440  1440  1440  1440  1440  1440  1440  1440  1440  1440  1440  1440  1440  1440  1440  1440  1440  1440  1440  1440  1440  1440  1440  1440  1443  1443  1443  1443  1443  1443  1443  1443  1443  1443  1443  1443  1443  1443  1443  1443  1443  1443  1443  1443  1443  1443  1443  1443  1443  1443  1443  1443  1443  1445  1445  1445  1445  1445  1445  1445  1445  1445  1445  1445  1445  1445  1445  1445  1445  1445  1445  1445  1445  1445  1445  1445  1445  1445  1445  1447  1447  1447  1447  1447  1447  1447  1447  1447  1447  1447  1447  1447  1447  1447  1447  1447  1447  1447  1447  1447  1447  1447  1447  1447  1447  1447  1447  1447  1447  1447  1447  1447  1447  1447  1447  1447  1447  1447  1447  1447  1447  1470  1470  1470  1470  1470  1470  1470  1470  1470  1470  1470  1470  1470  1470  1470  1470  1470  1470  1470  1470  1470  1470  1470  1470  1470  1470  1470  1470  1470  1470  1470  1470  1470  1470  1470  1470  1470  3162  3162  3162  3162  3162  3162  3162  3162  3164  3164  3164  3164  3164  3164  3164  3164  3164  3164  3164  3164  3164  3164  3164  3164  3167  3167  3167  3167  3167  3167  3167  3167  3167  3167  3167  3168  3168  3168  3168  3168  3168  3168  3168  3168  3168  3168  3168  3168  3168  3168  3168  3168  3169  3169  3169  3169  3169  3169  3169  3169  3169  3169  3169  3169  3169  3169  3169  3169  3169  3170  3170  3170  3170  3170  3170  3170  3170  3170  3170  3170  3170  3170  3170  3170  3171  3171  3171  3171  3171  3171  3171  3171  3171  3171  3171  3171  3171  3171  3173  3173  3173  3173  3173  3173  3173  3173  3173  3173  3173  3173  3173  3173  3173  3175  3175  3175  3175  3175  3175  3175  3175  21046  21046  21046  21046  21046  21046  21046  21046  21046  21046  21046  21046  21046  21046  21046  21046  21046  21046  21046  21046  21046  21046  21046  21046  21127  21127  21127  21127  21127  21127  21127  21127  21127  21127  21127  21127  21127  21127  21127  21135  21135  21135  21135  21135  21135  21135  21135  21135  21135  21135  21135  21135  21135  21135  21135  21135  21135  21135  21135  21135  21135  21135  21135  21135  21135  21135  21135  21135  21135  21135  21135  21135  21135  21135  21135  21135  21135  21135  21135  21135  21135  21135  21135  21135  21135  21135  21135  21135  21139  21139  21139  21139  21139  21139  21139  21139  21139  21139  21139  21139  21139  21139  21139  21139  21139  21139  21139  21139  21139  21139  21139  21139  21139  21139  21139  21139  21139  21139  21139  21139  21139  21139  21139  21139  21148  21148  21148  21148  21148  21148  21148  21148  21148  21148  21148  21148  21148  21148  21148  21148  21148  21148  21148  21148  21148  21148  21148  21148  21148  21148  21148  21148  21148  21148  21148  21148  21148  21148  21152  21152  21152  21152  21152  21152  21152  21152  21152  21152  21152  21152  21152  21152  21152  21152  21152  21152  21152  21152  21152  21152  21152  21152  21152  21152  21152  21152  21152  21152  21152  21152  21152  21152  21152  21152  21152  21152  21152  21166  21166  21166  21166  21166  21166  21166  21166  21166  21166  21166  21166  21166  21166  21166  21166  21166  21166  21166  21166  21166  21166  21166  21166  21166  21166  21166  21166  21166  21166  21166  21166  21206  21206  21206  21206  21206  21206  21206  21206  21206  21206  21206  21206  21206  21206  21206  21206  21206  21206  21206  21206  21206  21206  21206  21210  21210  21226  21226  21226  21226  21226  21226  21226  21226  21226  21226  21226  21226  21226  21226  21226  21226  21226  21226  21226  21226  21226  21251  21251  21251  21251  21251  21251  21251  21251  21251  21251  21251  21259  21259  21259  21259  21259  21259  21259  21259  21259  21259  21259  21259  21259  21259  21259  21259  21259  21259  21259  21259  21259  21259  21259  21259  21259  21259  21259  21259  21259  21259  21259  21259  21259  21259  21259  21259  21259  21285  21285  21285  21285  21285  21285  21285  21285  21285  21285  21285  21285  21285  21285  21285  21285  21285  21285  21285  21285  21285  21285  21285  21285  21285  21285  21285  21352  21352  21352  21352  21352  21352  21352  21352  21352  21352  21352  21352  21352  21352  21352  21352  21352  21352  21352  21352  21352  21352  21352  21352  21352  21352  21352  21352  21352  21352  21352  21352  21352  21352  21352  21352  21352  21352  21352  21352  21352  21352  21352  21352  21352  21352  21352  21352  21352  21352  21352  21352  21352  21354  21354  21354  21354  21354  21354  21354  21354  21354  21354  21354  21354  21354  21354  21354  21354  21354  21354  21354  21354  21354  21354  21354  21354  21354  21354  21354  21354  21354  21354  21354  21354  21354  21354  21357  21357  21357  21357  21357  21357  21357  21357  21357  21357  21357  21357  21357  21357  21357  21357  21357  21357  21357  21357  21357  21357  21362  21362  21362  21362  21362  21362  21362  21362  21362  21362  21362  21362  21362  21362  21362  21362  21362  21362  21362  21362  21362  21362  21362  21362  21362  21362  21362  21362  21362  21362  21362  21362  21362  21362  21362  21362  21362  21368  21368  21368  21368  21368  21368  21368  21368  21368  21368  21368  21368  21368  21368  21368  21368  21368  21368  21368  21368  21368  21368  21368  21368  21368  21368  21368  21368  21368  21368  21368  21368  21368  21523  21523  21523  21523  21523  21523  21523  21523  21523  21523  21523  21523  21523  21523  21523  21523  21523  21523  21523  21523  21523  21523  21523  21523  21523  21523  21523  21523 | 347  116  201  76  355  148  81  79  253  255  86  106  217  248  1  46  10  203  247  204  154  61  73  36  97  240  11  253  209  85  84  29  349  32  419  480  333  450  111  142  400  103  413  183  274  63  316  30  366  81  95  238  412  345  140  466  248  375  414  199  382  281  185  288  299  135  283  202  290  237  336  396  198  4  20  207  268  236  321  338  322  64  144  408  294  234  49  161  230  457  48  486  332  241  215  5  442  301  228  135  161  199  18  214  465  305  189  328  66  32  273  309  13  386  242  85  382  256  355  460  74  71  424  299  131  339  194  124  76  21  258  236  245  298  293  255  177  341  276  30  332  251  264  215  223  209  90  113  2  13  464  189  456  106  214  216  77  173  231  180  461  319  24  118  28  196  289  7  345  192  52  227  315  127  257  128  336  112  396  341  268  266  432  390  78  170  265  189  64  199  48  97  356  365  69  245  16  459  312  5  458  383  339  324  12  235  415  203  252  362  476  103  320  218  368  426  72  88  303  124  214  28  205  339  243  163  140  346  311  150  39  251  357  187  21  169  256  317  316  166  193  358  29  60  362  149  42  278  148  12  217  221  49  151  266  247  31  279  50  298  119  49  116  222  197  77  310  176  322  221  273  260  285  242  229  76  44  42  259  231  164  302  181  190  329  228  275  33  64  304  248  24  233  187  366  118  295  59  73  276  216  119  147  97  130  40  368  268  18  156  154  159  118  44  136  219  120  263  75  117  215  245  109  91  294  79  312  283  194  114  359  193  369  113  77  63  241  236  24  177  70  47  165  48  160  366  264  42  273  405  69  402  326  8  34  231  127  354  95  274  138  106  355  64  133  115  63  387  254  37  301  292  120  288  147  7  62  117  71  224  61  339  33  26  94  319  25  376  73  346  48  76  92  256  126  155  150  75  210  339  349  181  74  49  306  354  321  300  252  359  453  43  242  190  119  161  64  213  280  162  141  16  268  113  207  251  337  248  282  124  109  134  217  117  111  67  156  103  279  108  391  447  133  149  21  407  153  32  238  186  368  173  167  195  341  99  141  275  133  333  216  227  115  279  156  232  400  272  142  112  370  6  136  121  127  299  271  175  83  95  267  155  46  359  223  166  294  138  29  323  93  256  165  390  147  422  9  315  157  401  361  310  82  143  38  4  98  8  408  443  411  163  428  171  426  58  332  406  373  324  57  440  183  118  962  328  233  955  372  972  645  53  988  103  720  198  747  409  917  727  640  696  13  1069  851  683  270  342  581  824  1155  298  396  487  786  323  222  152  1033  1025  572  24  1121  855  458  11  643  258  811  116  448  268  559  749  311  1002  588  1104  68  705  922  450  1179  629  1014  180  938  736  535  924  394  848  40  524  445  1074  958  197  115  733  263  75  219  112  174  262  197  255  49  93  287  318  183  309  100  198  36  162  87  203  291  102  26  123  192  86  189  181  124  184  156  23  35  270  238  40  298  187  185  91  21  96  299  128  10  78  34  147  282  281  90  328  146  125  109  372  260  14  84  355  93  353  354  166  277  220  147  224  310  199  232  188  306  111  362  146  222  87  86  118  311  94  75  85  257  279  106  293  173  166  159  215  312  268  253  26  3  35  225  13  284  5  31  115  97  314  145  154  162  298  258  225  197  11  277  228  242  6  196  207  132  215  173  230  195  198  253  42  7  87  732  198  187  590  632  574  728  559  779  652  59  671  234  624  130  275  307  175  492  202  583  78  486  165  41  139  737  421  212  405  578  61  404  63  136  377  613  524  238  580  525  28  809  370  56  493  22  538  311  468  625  137  279  224  443  243  74  704  406  419  620  109  204  551  788  546  173  17  163  148  338  377  14  263  396  215  431  379  380  399  212  40  21  374  341  177  70  12  218  237  365  243  58  69  467  260  355  130  269  38  178  284  346  213  153  368  467  87  219  197  96  21  222  74  156  147  51  86  91  464  435  463  30  92  167  462  138  193  112  389  319  208  202  203  107  271  31  137  422  121  104  67  431  66  301  226  320  392  245  359  28  128  193  134  18  456  299  429  443  266  175  346  172  208  118  104  180  77  115  207  174  15  413  11  128  170  148  295  157  145  262  92  210  354  457  325  127  211  94  49  125  138  449  164  311  328  331  340  347  312  401  193  108  106  66  112  6  359  11  43  263  135  120  200  374  303  83  50  46  146  232  97  209  322  152  28  4  192  281  86  76  215  206  186  100  82  200  225  149  132  36  269  67  123  59  248  90  176  72  286  30  266  121  110  22  95  102  110  39  52  92  151  6  136  145  142  70  157  124  174  47  169  171  94  103  87  72  163  80  97  28  142  180  115  168  71  56  178  129  77  119  94  106  112  1  36  153  197  5  185  193  13  167  217  238  140  88  61  55  180  254  166  42  108  96  250  147  163  219  208  71  63  215  192  235  242  97  35  189  147  81  96  88  132  144  125  150  87  84  75  101  106  67  24  33  77  49  74  168  129  27  136  128  8  66  127  81  184  117  93  69  95  116  37  103  155  118  122  121  33  25  79  162  22  131  195  76  116  136  153  180  172  112  164  169  57  145  62  151  53  59  125  377  484  491  480  383  324  533  448  173  147  478  61  36  232  406  159  434  40  140  322  131  336  345  286  473  381  196  449  33  472  103  176  469  530  83  270  181  192  452  49  269  238  424  23  77  124  470  458  360  83  279  112  254  378  13  471  502  33  278  228  67  253  262  90  134  117  334  315  426  35  211  349  32  486  124  379  71  365  185  400  20  87  245  418  368  263  273  345  240  391  158  446  131  99  387  173  231  337  466  124  161  7  272  465  330  235  246  251  252  233  277  48  119  19  145  11  451  370  510  441  485  271  264  512  471  214  179  515  490  183  347  241  20  339  389  619  367  480  533  325  478  225  196  14  506  224  430  240  1  457  303  198  202  18  566  335  589  25  281  251  56  124  237  22  197  36  90  49  34  131  1  272  149  198  153  205  253  231  137  252  178  292  30  19  112  58  99  200  59  28  267  187  112  129  192  216  206  23  172  96  265  276  161  157  78  215  148  212  128  34  139  158  46  109  43  154  173  113  97  44  8  13  88  32  182  165  106  141  119  20  104  175  134  65  178  66  74  88  84  97  95  68  34  179  75  115  188  268  108  75  305  86  59  566  317  89  333  50  138  43  505  459  237  95  225  592  178  16  552  161  96  155  293  207  157  253  331  465  267  27  477  47  228  184  325  287  205  171  224  93  246  298  102  96  101  13  12  208  51  228  52  168  134  166  111  82  201  227  136  88  304  64  100  369  135  316  80  265  158  178  35  36  208  127  175  150  2  173  177  362  302  200  98  152  59  191  228  6  82  286  339  49  99  89  212  185  176  148  171  279  128  189  329  51  25  256  167  240  236  129  169  123  101  85  251  354  194  79  375  383  288  434  412  110  292  40  193  257  36  224  215  345  425  408  54  197  378  84  308  294  199  86  70  210  387  336  421  168  55  19  25  340  104  296  150  84  190  96  310  317  56  23  68  266  49  18  146  228  230  126  312  81  93  172  35  215  61  219  313  333  406  420  264  43  281  397  38  16  396  414  26  421  411  445  297  355  138  438  128  453  292  90  402  369  53  36  173  211  29  64  52  277  114  163  287  249  123  247  67  87  290  32  59  296  342  275  105  366  129  2  209  391  232  286  309  165  257  185  180  58  71  268  67  287  72  83  286  326  136  217  73  52  68  334  280  31  335  49  318  5  54  18  323  43  70  152  269 | TGTTATATAATATGTATGCG**C**TTGTTACCGTTTTATTTTAA  GTTTGATTAACGAATCAGAG**C**AAGATCTGGATCTAGAACTC  AGGATTCGAAGTTCACAATT**C**TAGAAGATATGATGCTTCTC  CTGAGGAATGATGAAATCAT**C**AGCTCGGAGCTTGAGATTGG  AATATGTATGCGCTTGTTAC**C**GTTTTATTTTAATGGAGGCT  CTAGAACTCCGTTTGGGTTT**C**GCTTAATTAGATGGTAATAA  GAATGATGAAATCATCAGCT**C**GGAGCTTGAGATTGGTTTGA  AGGAATGATGAAATCATCAG**C**TCGGAGCTTGAGATTGGTTT  AGTTTCATCCATATGAAATT**C**TCTAAGCTTGCTATTTAGTA  TTTCATCCATATGAAATTCT**C**TAAGCTTGCTATTTAGTAGA  ATGAAATCATCAGCTCGGAG**C**TTGAGATTGGTTTGATTAAC  CTTGAGATTGGTTTGATTAA**C**GAATCAGAGCAAGATCTGGA  TAAAAACCTGTGCAAGGGGA**C**AGGCGGTCACACGGACGTAA  ACGGACGTAAGCCTCACTTC**C**TACCCTCGGCATATACGGCC  GCCTCACTTCCTACCCTCGG**C**ATATACGGCCTGGTCCGGTC  CTACCTGGGCCAGGGCAGTC**C**TCTCCTTCTTTGGTCAGTGC  CCTACCCTCGGCATATACGG**C**CTGGTCCGGTCCTAGCTACC  CTCCCGGGACTATATAAAAA**C**CTGTGCAAGGGGACAGGCGG  CACGGACGTAAGCCTCACTT**C**CTACCCTCGGCATATACGGC  TCCCGGGACTATATAAAAAC**C**TGTGCAAGGGGACAGGCGGT  ATATGGCTTGGGAAGGGCAG**C**AACATTCTTCGGGGCGGTGT  CAGTCCTCTCCTTCTTTGGT**C**AGTGCAGGAGACCCGGGCGG  TCTTTGGTCAGTGCAGGAGA**C**CCGGGCGGGACCCAGGCTGA  CCGGTCCTAGCTACCTGGGC**C**AGGGCAGTCCTCTCCTTCTT  GGCGGGACCCAGGCTGAGAA**C**CAGCCGAAGGAAGGGACTCT  GCGGTCACACGGACGTAAGC**C**TCACTTCCTACCCTCGGCAT  CTACCCTCGGCATATACGGC**C**TGGTCCGGTCCTAGCTACCT  CGTAAGCCTCACTTCCTACC**C**TCGGCATATACGGCCTGGTC  GGACTATATAAAAACCTGTG**C**AAGGGGACAGGCGGTCACAC  GCAGGAGACCCGGGCGGGAC**C**CAGGCTGAGAACCAGCCGAA  TGCAGGAGACCCGGGCGGGA**C**CCAGGCTGAGAACCAGCCGA  GCCTGGTCCGGTCCTAGCTA**C**CTGGGCCAGGGCAGTCCTCT  TCCCCGGCCCCCGGGCCTCC**C**CGCGCCGCTCGCCTCTCTCC  GCAGGATTCGGGACCAAGCG**C**AAAGGCAGGTCTCGACCAAG  TCCCGGAGCCCAGCCCGGCG**C**GCGGAGCCCGCTCACCGAGT  GCCCCGCCGCAGCAACAGAA**C**TCTCCCACAGCAGCACTCCT  CTGCGGGCCCCGAGATTCCC**C**GGCCCCCGGGCCTCCCCGCG  CTCACCGAGTTTCCCACAGT**C**AACGTGCAGGCCCCGCCGCA  TATCCTCAGAAGCCAGGGGT**C**CTTACAGTAGCCCTCGCGGG  CCCTCGCGGGCCCCAGCGCC**C**ACCCAGAGCGAGGGGCCTCC  GGCCGGGGAGCCTCCGCGGT**C**CCGGAGCCCAGCCCGGCGCG  AGACGCAGTATCCTCAGAAG**C**CAGGGGTCCTTACAGTAGCC  CCGCGGTCCCGGAGCCCAGC**C**CGGCGCGCGGAGCCCGCTCA  GACTTGGCCCCGGCCTGGCA**C**ACCGTCCCGGAGGCCCATCC  CCAGCTTCTAGAGGCGCCGC**C**CGGTTTCCCCTCGCCCCTGC  CTCGACCAAGCACCTCAAGG**C**CCCATACGGAGAAAGTTCTA  TCTCACACGCAGGTAGGCTG**C**GGGCCCCGAGATTCCCCGGC  GGGCAGGATTCGGGACCAAG**C**GCAAAGGCAGGTCTCGACCA  TCCCCGCGCCGCTCGCCTCT**C**TCCCTCGTCGATGGGCCGGG  GGCCCCATACGGAGAAAGTT**C**TAGACGCAGTATCCTCAGAA  AAAGTTCTAGACGCAGTATC**C**TCAGAAGCCAGGGGTCCTTA  GGTGGGGCTTCACCGCCCCC**C**GCCCCGCCCCCGAGACCAGC  TCCGCGGTCCCGGAGCCCAG**C**CCGGCGCGCGGAGCCCGCTC  AGATTCCCCGGCCCCCGGGC**C**TCCCCGCGCCGCTCGCCTCT  AGCCCTCGCGGGCCCCAGCG**C**CCACCCAGAGCGAGGGGCCT  CAGTCAACGTGCAGGCCCCG**C**CGCAGCAACAGAACTCTCCC  CACCGCCCCCCGCCCCGCCC**C**CGAGACCAGCTTCTAGAGGC  CGCTCGCCTCTCTCCCTCGT**C**GATGGGCCGGGGAGCCTCCG  CGCGGTCCCGGAGCCCAGCC**C**GGCGCGCGGAGCCCGCTCAC  GGCACACCGTCCCGGAGGCC**C**ATCCCGGCCGCTCCTCCAGG  CTCTCTCCCTCGTCGATGGG**C**CGGGGAGCCTCCGCGGTCCC  CTAGAGGCGCCGCCCGGTTT**C**CCCTCGCCCCTGCCTCTCAC  CTTGGCCCCGGCCTGGCACA**C**CGTCCCGGAGGCCCATCCCG  CGCCGCCCGGTTTCCCCTCG**C**CCCTGCCTCTCACACGCAGG  TTCCCCTCGCCCCTGCCTCT**C**ACACGCAGGTAGGCTGCGGG  ACAGTAGCCCTCGCGGGCCC**C**AGCGCCCACCCAGAGCGAGG  AGAGGCGCCGCCCGGTTTCC**C**CTCGCCCCTGCCTCTCACAC  ACACCGTCCCGGAGGCCCAT**C**CCGGCCGCTCCTCCAGGTGG  CCGCCCGGTTTCCCCTCGCC**C**CTGCCTCTCACACGCAGGTA  AGGTGGGGCTTCACCGCCCC**C**CGCCCCGCCCCCGAGACCAG  CGGGCCCCGAGATTCCCCGG**C**CCCCGGGCCTCCCCGCGCCG  GATGGGCCGGGGAGCCTCCG**C**GGTCCCGGAGCCCAGCCCGG  TGGCACACCGTCCCGGAGGC**C**CATCCCGGCCGCTCCTCCAG  AACTCTCCCACAGCAGCACT**C**CTAGAGGGCAGGATTCGGGA  CACTCCTAGAGGGCAGGATT**C**GGGACCAAGCGCAAAGGCAG  GTCCCGGAGGCCCATCCCGG**C**CGCTCCTCCAGGTGGGGCTT  CCGAGACCAGCTTCTAGAGG**C**GCCGCCCGGTTTCCCCTCGC  CAGGTGGGGCTTCACCGCCC**C**CCGCCCCGCCCCCGAGACCA  CACGCAGGTAGGCTGCGGGC**C**CCGAGATTCCCCGGCCCCCG  GGCCCCGAGATTCCCCGGCC**C**CCGGGCCTCCCCGCGCCGCT  ACGCAGGTAGGCTGCGGGCC**C**CGAGATTCCCCGGCCCCCGG  TCGACCAAGCACCTCAAGGC**C**CCATACGGAGAAAGTTCTAG  CTCGCGGGCCCCAGCGCCCA**C**CCAGAGCGAGGGGCCTCCGA  AGCCTCCGCGGTCCCGGAGC**C**CAGCCCGGCGCGCGGAGCCC  CCGGTTTCCCCTCGCCCCTG**C**CTCTCACACGCAGGTAGGCT  TCCAGGTGGGGCTTCACCGC**C**CCCCGCCCCGCCCCCGAGAC  GCGCAAAGGCAGGTCTCGAC**C**AAGCACCTCAAGGCCCCATA  CCACCCAGAGCGAGGGGCCT**C**CGACTTGGCCCCGGCCTGGC  CTCCTCCAGGTGGGGCTTCA**C**CGCCCCCCGCCCCGCCCCCG  AGTTTCCCACAGTCAACGTG**C**AGGCCCCGCCGCAGCAACAG  AGCGCAAAGGCAGGTCTCGA**C**CAAGCACCTCAAGGCCCCAT  CCGCAGCAACAGAACTCTCC**C**ACAGCAGCACTCCTAGAGGG  GCTGCGGGCCCCGAGATTCC**C**CGGCCCCCGGGCCTCCCCGC  GGGGCTTCACCGCCCCCCGC**C**CCGCCCCCGAGACCAGCTTC  GGCCCATCCCGGCCGCTCCT**C**CAGGTGGGGCTTCACCGCCC  ACTCTCCCACAGCAGCACTC**C**TAGAGGGCAGGATTCGGGAC  GGAGCCCGCTCACCGAGTTT**C**CCACAGTCAACGTGCAGGCC  CCCCTCGCCCCTGCCTCTCA**C**ACGCAGGTAGGCTGCGGGCC  CGCTCCTCCAGGTGGGGCTT**C**ACCGCCCCCCGCCCCGCCCC  GAACTGTAACATAGATCTTC**C**TGCCTCTCGGGGGCATTCTG  CTCGGGGGCATTCTGAAGAC**C**AGAAGGCAGGGGACACAGGA  GGAAAAGGAACTGAGCAAGT**C**ATGACGAAGCAGAACCCTGG  ACTGACAAGAGTCACCAAAT**C**AGCAACAACGTGACCCAGTG  CAAGTCATGACGAAGCAGAA**C**CCTGGGAAGGGTTGGCAATA  GGAAGGTGGCAACTGCAGTC**C**ACTGCAGACTGACAAGAGTC  TCGGGTCCCAATTCCAGGGT**C**CACCAGCTGCACACACCAGT  AGGGGACACAGGAAAAGGAA**C**TGAGCAAGTCATGACGAAGC  CCAGCTGCACACACCAGTCC**C**AGGGCTAGGGCACAGGCACC  TCTTCCGAATCCCACGCTAC**C**ATCCCAGCACCCCGTGGACC  CCAAATCAGCAACAACGTGA**C**CCAGTGGTGCAAGTCTTCCG  GCCCCCACCCCAGACAGGTT**C**TACCTGTTCCATCGGGTCCC  GTCCCAATTCCAGGGTCCAC**C**AGCTGCACACACCAGTCCCA  TGCAGACTGACAAGAGTCAC**C**AAATCAGCAACAACGTGACC  TGCTTGCTCCATAGGTCATA**C**CTTTTAGGAGAAATGAAGGG  AGGGTTGGCAATAACAAACA**C**AACCCCTGCCGCCCCCACCC  CCATCCCAGCACCCCGTGGA**C**CAAGAGTGGGTGGGAGGGCA  CGCCTGCTTGCTCCATAGGT**C**ATACCTTTTAGGAGAAATGA  CAAACACAACCCCTGCCGCC**C**CCACCCCAGACAGGTTCTAC  AGGGCACAGGCACCCTCCTG**C**CTAACTCGCCTGCTTGCTCC  ATAGAGGAAGGTGGCAACTG**C**AGTCCACTGCAGACTGACAA  ATCCCACGCTACCATCCCAG**C**ACCCCGTGGACCAAGAGTGG  CGAATCCCACGCTACCATCC**C**AGCACCCCGTGGACCAAGAG  GGGAATGCAGAGAAGCAACA**C**AACAAAGACCTCCGCATAGA  GTTCCATCGGGTCCCAATTC**C**AGGGTCCACCAGCTGCACAC  TCCAGAACTGTAACATAGAT**C**TTCCTGCCTCTCGGGGGCAT  CACCAGTCCCAGGGCTAGGG**C**ACAGGCACCCTCCTGCCTAA  ACACAGGAAAAGGAACTGAG**C**AAGTCATGACGAAGCAGAAC  CATCATTTCCAGAACTGTAA**C**ATAGATCTTCCTGCCTCTCG  CCCACGCTACCATCCCAGCA**C**CCCGTGGACCAAGAGTGGGT  GACAAGAGTCACCAAATCAG**C**AACAACGTGACCCAGTGGTG  AACACAACCCCTGCCGCCCC**C**ACCCCAGACAGGTTCTACCT  CTGGGAAGGGTTGGCAATAA**C**AAACACAACCCCTGCCGCCC  GTTGGCAATAACAAACACAA**C**CCCTGCCGCCCCCACCCCAG  TGTTCCATCGGGTCCCAATT**C**CAGGGTCCACCAGCTGCACA  CTACCTGTTCCATCGGGTCC**C**AATTCCAGGGTCCACCAGCT  ACAAACACAACCCCTGCCGC**C**CCCACCCCAGACAGGTTCTA  AGACCAGAAGGCAGGGGACA**C**AGGAAAAGGAACTGAGCAAG  CCAGTCCCAGGGCTAGGGCA**C**AGGCACCCTCCTGCCTAACT  CCCACCCCAGACAGGTTCTA**C**CTGTTCCATCGGGTCCCAAT  CAACACAACAAAGACCTCCG**C**ATAGAGGAAGGTGGCAACTG  TTCCGTTCGCTGATCAATTA**C**CTGCCCCCTCCAGCACGTGT  CAAAGTCTCTGGTGCGCTGG**C**GGAAGCAGGGCTCCACTCGG  GCGCTGGCGGAAGCAGGGCT**C**CACTCGGCAGGGCCTCTTGG  CCGCCACTCTCCACCCTGAC**C**CCCTGCACTTTGCTTCAAAG  CTCCACCCTGACCCCCTGCA**C**TTTGCTTCAAAGTCTCTGGT  CTGGGGCCGCCACTCTCCAC**C**CTGACCCCCTGCACTTTGCT  CCTGTTGCTAGAAGGTTTCC**C**ACAGGAAGATGTGAGCTTGT  AGGAAGATGTGAGCTTGTTT**C**CTGGCAGGGCACAAAAGGTA  GTGCCAGGGCACCAAGAGGG**C**AGAGAAGCAACACAACAAAG  CCAAGAGGGCAGAGAAGCAA**C**ACAACAAAGACCTCCGCATA  GAAGAGCAGTGCCAGGGCAC**C**AAGAGGGCAGAGAAGCAACA  AGTTCAGTTACCCTGCGAGG**C**TGGGGCCGCCACTCTCCACC  TTCTTCGGGAAGAGCAGTGC**C**AGGGCACCAAGAGGGCAGAG  TTCCCACAGGAAGATGTGAG**C**TTGTTTCCTGGCAGGGCACA  GCCGCCACTCTCCACCCTGA**C**CCCCTGCACTTTGCTTCAAA  CGCCACTCTCCACCCTGACC**C**CCTGCACTTTGCTTCAAAGT  ACTGCAGACTCATCCTGTTG**C**TAGAAGGTTTCCCACAGGAA  GCAGCCAAGAACATCCAGTT**C**AGTTACCCTGCGAGGCTGGG  TGACCCCCTGCACTTTGCTT**C**AAAGTCTCTGGTGCGCTGGC  AGAACATCCAGTTCAGTTAC**C**CTGCGAGGCTGGGGCCGCCA  CGGGAAGAGCAGTGCCAGGG**C**ACCAAGAGGGCAGAGAAGCA  AGGCCCCACAAACTTCCGTT**C**GCTGATCAATTACCTGCCCC  GAGAAGCAACACAACAAAGA**C**CTCCGCATAGAGGAAGGTGG  GATGTGAGCTTGTTTCCTGG**C**AGGGCACAAAAGGTACGGGA  AGCAACACAACAAAGACCTC**C**GCATAGAGGAAGGTGGCAAC  TTACCCTGCGAGGCTGGGGC**C**GCCACTCTCCACCCTGACCC  CGGCAGGGCCTCTTGGCCAG**C**AGCGTTCACAGGCCCCACAA  AAAGGCAGGTCTCGCGTTCA**C**AGGCCCCACAAACTTCCGTT  CACCGTCCCCACGGCGCCCG**C**GGAGAGAAACCGGCACCTCC  CCAAGAGGAGGACGCCTCGG**C**ACCCATCGGGCGCTCCCTTC  GGTCAATTACCTGCCCCCTC**C**AGCACGTGTGTCAACACGTC  CCCTTCCCCGCCAGGCAGAA**C**TCAGCCGCAGAGGCGGGCGC  AGTCCAAGCGCTGCCGACGC**C**CCCGCCTCCCACCGTCCCCA  AAGCCCGAGATTTCCGACGC**C**CGTTTAACTGAAAGGCGTTC  GAGGCGGGCGCTCTGCGGGC**C**CAAATCCCCGCTACCAGGCA  AGCCCGAGATTTCCGACGCC**C**GTTTAACTGAAAGGCGTTCT  CCCGCCTCCCACCGTCCCCA**C**GGCGCCCGCGGAGAGAAACC  GATCCCTGCCCCAGGAAGCC**C**GAGATTTCCGACGCCCGTTT  CAGCGGCCTTCCGCCCGGGG**C**CGCTTCGCCTCCGGTGGGCT  CTCCCACCGTCCCCACGGCG**C**CCGCGGAGAGAAACCGGCAC  TCTGCGGGCCCAAATCCCCG**C**TACCAGGCAGGCCCAAGGCC  GCTCTGCGGGCCCAAATCCC**C**GCTACCAGGCAGGCCCAAGG  GGGCTAGAGGCCCGCAAGAG**C**GACTCCTAGAGGGCAGGATT  AGGATCCAGCGGCCTTCCGC**C**CGGGGCCGCTTCGCCTCCGG  GTGTGTCAACACGTCCAGAG**C**GGCCTCTCCCGACGATCCCT  CGGGAAGAGCAGTGCCAGGG**C**ACCAAGAGGAGGACGCCTCG  CGCTCTGCGGGCCCAAATCC**C**CGCTACCAGGCAGGCCCAAG  GCACCAAGAGGAGGACGCCT**C**GGCACCCATCGGGCGCTCCC  GCCCCCTCCAGCACGTGTGT**C**AACACGTCCAGAGCGGCCTC  GAGGACGCCTCGGCACCCAT**C**GGGCGCTCCCTTCCCCGCCA  CGCTGGTCAATTACCTGCCC**C**CTCCAGCACGTGTGTCAACA  GCGGCCTCTCCCGACGATCC**C**TGCCCCAGGAAGCCCGAGAT  CGGCGCCCGCGGAGAGAAAC**C**GGCACCTCCCTCGAGGATCC  CGGAGAGAAACCGGCACCTC**C**CTCGAGGATCCAGCGGCCTT  CTCCAGCACGTGTGTCAACA**C**GTCCAGAGCGGCCTCTCCCG  AACTCAGCCGCAGAGGCGGG**C**GCTCTGCGGGCCCAAATCCC  TCTCGCGTTCACAGGCCCCA**C**AAACTTCCGTTCGCTGGTCA  TAGAGGGCAGGATTCGGGAC**C**AAGCGCAAAGGCAGGTCTCG  CCTAGTCCAAGCGCTGCCGA**C**GCCCCCGCCTCCCACCGTCC  GCAAAGGCAGGTCTCGCGTT**C**ACAGGCCCCACAAACTTCCG  CTAGAGGGCAGGATTCGGGA**C**CAAGCGCAAAGGCAGGTCTC  TCCCTCGAGGATCCAGCGGC**C**TTCCGCCCGGGGCCGCTTCG  GCCTCCCACCGTCCCCACGG**C**GCCCGCGGAGAGAAACCGGC  GCTGCCGACGCCCCCGCCTC**C**CACCGTCCCCACGGCGCCCG  CAGGTCTCGCGTTCACAGGC**C**CCACAAACTTCCGTTCGCTG  CGCCAGGCAGAACTCAGCCG**C**AGAGGCGGGCGCTCTGCGGG  GCCGCTTCGCCTCCGGTGGG**C**TAGAGGCCCGCAAGAGCGAC  ACGCCTCGGCACCCATCGGG**C**GCTCCCTTCCCCGCCAGGCA  CCGCAGAGGCGGGCGCTCTG**C**GGGCCCAAATCCCCGCTACC  CCGCGGAGAGAAACCGGCAC**C**TCCCTCGAGGATCCAGCGGC  GACCAAGCGCAAAGGCAGGT**C**TCGCGTTCACAGGCCCCACA  TCTCCCGACGATCCCTGCCC**C**AGGAAGCCCGAGATTTCCGA  AAGCGCTGCCGACGCCCCCG**C**CTCCCACCGTCCCCACGGCG  TCGGGCGCTCCCTTCCCCGC**C**AGGCAGAACTCAGCCGCAGA  AGAGAAACCGGCACCTCCCT**C**GAGGATCCAGCGGCCTTCCG  TCCGGTGGGCTAGAGGCCCG**C**AAGAGCGACTCCTAGAGGGC  CAGCACGTGTGTCAACACGT**C**CAGAGCGGCCTCTCCCGACG  ACGTCCAGAGCGGCCTCTCC**C**GACGATCCCTGCCCCAGGAA  AAGGCCGCACCTAGTCCAAG**C**GCTGCCGACGCCCCCGCCTC  AGGAAGCCCGAGATTTCCGA**C**GCCCGTTTAACTGAAAGGCG  CCCATCGGGCGCTCCCTTCC**C**CGCCAGGCAGAACTCAGCCG  TTGGCCTTGGGGACATGCGG**C**CTCACGGCCACTGCAGGGAC  GACGTGTTCAATGGGCGGTA**C**CACGTGGTGCGCAAACTGGG  TGGGCTTGCTTGGGGCCACC**C**TGATCCCCGCCGTGGGTCCT  GGGCTGGGGCCACTTCTCCA**C**CGTCTGGCTCTGCTGGGACA  CATCTCCCCTGGGCAGGCGG**C**TACCACCCTGTGAAGATCGG  AGAACCCCCTCCACCCCAAT**C**TACATCTCCCCTGGGCAGGC  GCTTGGGGCCACCCTGATCC**C**CGCCGTGGGTCCTGCCAGTG  GGCGGAGCACTGGCTGGGAT**C**CTGTCCCTGGGCTTGCTTGG  CCACCCCAATCTACATCTCC**C**CTGGGCAGGCGGCTACCACC  GACATGCGGCCTCACGGCCA**C**TGCAGGGACCCAGGGCAGTC  GCCACTTCTCCACCGTCTGG**C**TCTGCTGGGACATCCAGTGA  CCCTGATCCCCGCCGTGGGT**C**CTGCCAGTGAGACTTGGCCT  CACCCTGTGAAGATCGGCGA**C**GTGTTCAATGGGCGGTACCA  GTGAGACTTGGCCTTGGGGA**C**ATGCGGCCTCACGGCCACTG  CCCTGGGCAGGCGGCTACCA**C**CCTGTGAAGATCGGCGACGT  TTCTCCACCGTCTGGCTCTG**C**TGGGACATCCAGTGAGTGCC  GCACTGGCTGGGATCCTGTC**C**CTGGGCTTGCTTGGGGCCAC  AGCACTGGCTGGGATCCTGT**C**CCTGGGCTTGCTTGGGGCCA  CTCCCCTGGGCAGGCGGCTA**C**CACCCTGTGAAGATCGGCGA  GTGAAGATCGGCGACGTGTT**C**AATGGGCGGTACCACGTGGT  CCTGATCCCCGCCGTGGGTC**C**TGCCAGTGAGACTTGGCCTT  TGGCCTTGGGGACATGCGGC**C**TCACGGCCACTGCAGGGACC  TGCAGGGACCCAGGGCAGTC**C**TGGGCCCACATGGGCCAGAT  ATCCCCGCCGTGGGTCCTGC**C**AGTGAGACTTGGCCTTGGGG  TCCACCCCAATCTACATCTC**C**CCTGGGCAGGCGGCTACCAC  ATGCGGCCTCACGGCCACTG**C**AGGGACCCAGGGCAGTCCTG  GGGACATCCAGTGAGTGCCT**C**CTTCGCCTCCGGGGCGGAGC  CTCCACCCCAATCTACATCT**C**CCCTGGGCAGGCGGCTACCA  GTCCTGCCAGTGAGACTTGG**C**CTTGGGGACATGCGGCCTCA  GGGCGGTACCACGTGGTGCG**C**AAACTGGGCTGGGGCCACTT  GGTACCACGTGGTGCGCAAA**C**TGGGCTGGGGCCACTTCTCC  CTCACGGCCACTGCAGGGAC**C**CAGGGCAGTCCTGGGCCCAC  CACCCCAATCTACATCTCCC**C**TGGGCAGGCGGCTACCACCC  TCTGGCTCTGCTGGGACATC**C**AGTGAGTGCCTCCTTCGCCT  TGGGGCCACTTCTCCACCGT**C**TGGCTCTGCTGGGACATCCA  GCCTTGGGGACATGCGGCCT**C**ACGGCCACTGCAGGGACCCA  GGACATCCAGTGAGTGCCTC**C**TTCGCCTCCGGGGCGGAGCA  TCACGGCCACTGCAGGGACC**C**AGGGCAGTCCTGGGCCCACA  CCTTCGCCTCCGGGGCGGAG**C**ACTGGCTGGGATCCTGTCCC  TGCGTGGCCGGGATGAGCGC**C**AGCACGGGCGGTGGTGGGGA  AAGGCTATAAATTCGCAGGC**C**GCGGCCGGGCCCCACAGGAG  GGCTGCGTGGCCGGGATGAG**C**GCCAGCACGGGCGGTGGTGG  GCGTCTCTCATGCCCACTGC**C**ACTCACCCCACCCGCAGCTC  TCGGCTGGGGCACCCGGAGC**C**CCTGGCGTCTCTCATGCCCA  GGCCCCACAGGAGCAGCCGC**C**CGGGGCACCGGAGCTGCGGG  CCACACCGGTGCCTCAGAAG**C**TGCAGGGCCTTCTGGGCTCC  GGCAGTAGCAGCAGGTAGGG**C**TCGGCTGGGGCACCCGGAGC  CTCAGAAGCTGCAGGGCCTT**C**TGGGCTCCGACGTCGAGGAA  GGCGTCTCTCATGCCCACTG**C**CACTCACCCCACCCGCAGCT  TGCGGGCCCGAGTCCTCGGG**C**TCCGAACTAGCCCTGGCCAC  CTCACAGGCCTCCTGCGGGC**C**CGAGTCCTCGGGCTCCGAAC  TCCTCGGGCTCCGAACTAGC**C**CTGGCCACACCGGTGCCTCA  CACTCACCCCACCCGCAGCT**C**ACAGGCCTCCTGCGGGCCCG  TCATGCCCACTGCCACTCAC**C**CCACCCGCAGCTCACAGGCC  GGGCCCCACAGGAGCAGCCG**C**CCGGGGCACCGGAGCTGCGG  CGGCCAAGGCTATAAATTCG**C**AGGCCGCGGCCGGGCCCCAC  GCCGGCCAAGGCTATAAATT**C**GCAGGCCGCGGCCGGGCCCC  GCTCACAGGCCTCCTGCGGG**C**CCGAGTCCTCGGGCTCCGAA  ATGCCCACTGCCACTCACCC**C**ACCCGCAGCTCACAGGCCTC  GGCGGCAGCGGCGGCAGTAG**C**AGCAGGTAGGGCTCGGCTGG  AGCCCTGGCCACACCGGTGC**C**TCAGAAGCTGCAGGGCCTTC  TAGCAGCAGGTAGGGCTCGG**C**TGGGGCACCCGGAGCCCCTG  GTAGGGCTCGGCTGGGGCAC**C**CGGAGCCCCTGGCGTCTCTC  GCTGCAGGGCCTTCTGGGCT**C**CGACGTCGAGGAACAGGAAG  CTCATGCCCACTGCCACTCA**C**CCCACCCGCAGCTCACAGGC  CGGGCCCGAGTCCTCGGGCT**C**CGAACTAGCCCTGGCCACAC  AGATCAGCGGCCGGCCAAGG**C**TATAAATTCGCAGGCCGCGG  CAGGCCGCGGCCGGGCCCCA**C**AGGAGCAGCCGCCCGGGGCA  CCCTGGCCACACCGGTGCCT**C**AGAAGCTGCAGGGCCTTCTG  CCCCACCCGCAGCTCACAGG**C**CTCCTGCGGGCCCGAGTCCT  AGGCGGGTGAGATCAGCGGC**C**GGCCAAGGCTATAAATTCGC  GCCCACTGCCACTCACCCCA**C**CCGCAGCTCACAGGCCTCCT  CAGGTAGGGCTCGGCTGGGG**C**ACCCGGAGCCCCTGGCGTCT  GAAGACCCCAAAGACTACTG**C**AAGGGTGAGACTTTAAAGGC  CTGCGTGGCCGGGATGAGCG**C**CAGCACGGGCGGTGGTGGGG  CCGAACTAGCCCTGGCCACA**C**CGGTGCCTCAGAAGCTGCAG  ATTCGCAGGCCGCGGCCGGG**C**CCCACAGGAGCAGCCGCCCG  GCCGGGCCCCACAGGAGCAG**C**CGCCCGGGGCACCGGAGCTG  GGGCCCGAGTCCTCGGGCTC**C**GAACTAGCCCTGGCCACACC  CCCCTGGCGTCTCTCATGCC**C**ACTGCCACTCACCCCACCCG  ATGGGCCACTCTGTCCCTCC**C**CCTGCCCTCCCCTCCCCTCG  TCCCCTCCCCTCGCGGCCCT**C**CCGCCCCTCCCCTCTGGCCC  CACACGAACACACACACACA**C**AATGGGCCACTCTGTCCCTC  TGTCCCTCCCCCTGCCCTCC**C**CTCCCCTCGCGGCCCTCCCG  GCCCCCACGGGTCGGGCACA**C**AAACGGGACATTGTGTGGGC  GTTGCTGGAAGCCGAAGTGA**C**CCGGTGATGGGTGGGAAACA  CATGCACGTCCCATGCCCGC**C**CGCCCGCCCGCCCGCCCGGG  CAGGAGGTAAGGAGCCCTGG**C**TGCCCCCACGGGTCGGGCAC  CTCGCGGCCCTCCCGCCCCT**C**CCCTCTGGCCCGGGCCTGGA  CCCTCGCGGCCCTCCCGCCC**C**TCCCCTCTGGCCCGGGCCTG  GCGGCCCTCCCGCCCCTCCC**C**TCTGGCCCGGGCCTGGAACG  AATGGGCCACTCTGTCCCTC**C**CCCTGCCCTCCCCTCCCCTC  CCACGGGTCGGGCACACAAA**C**GGGACATTGTGTGGGCCCCC  TCCCCCTGCCCTCCCCTCCC**C**TCGCGGCCCTCCCGCCCCTC  TTGGGAAGCCTGCGGCCTGG**C**CCGCCTGGCGCCGCCACTGG  TGGGCCACTCTGTCCCTCCC**C**CTGCCCTCCCCTCCCCTCGC  CACTGCATGCACGTCCCATG**C**CCGCCCGCCCGCCCGCCCGC  GTGGGCCCCCCACGTGTGCA**C**ACACACGAACACACACACAC  CAATGGGCCACTCTGTCCCT**C**CCCCTGCCCTCCCCTCCCCT  AGGCTTGGGAAGCCTGCGGC**C**TGGCCCGCCTGGCGCCGCCA  TGGCGCCGCCACTGGACACA**C**TGCATGCACGTCCCATGCCC  CACACACACAATGGGCCACT**C**TGTCCCTCCCCCTGCCCTCC  TGCACACACACGAACACACA**C**ACACACAATGGGCCACTCTG  GCCCGCCCGCCCGGGCCCAG**C**TTAGCAACAGCGATGGGCAC  GCCCCCCACGTGTGCACACA**C**ACGAACACACACACACACAA  AGCTTAGCAACAGCGATGGG**C**ACGCGTGTGTCCTGTGACTA  CCCGCCCGCCCGCCCGCCCG**C**CCGGGCCCAGCTTAGCAACA  GGAACGCTGGGTGCCCGAGC**C**AGGCTTGGGAAGCCTGCGGC  ACACAATGGGCCACTCTGTC**C**CTCCCCCTGCCCTCCCCTCC  AGCACTGGGGTTGCTGGAAG**C**CGAAGTGACCCGGTGATGGG  TGGAACGCTGGGTGCCCGAG**C**CAGGCTTGGGAAGCCTGCGG  TTGCTGGAAGCCGAAGTGAC**C**CGGTGATGGGTGGGAAACAG  CACACAATGGGCCACTCTGT**C**CCTCCCCCTGCCCTCCCCTC  GGGCCCCCCACGTGTGCACA**C**ACACGAACACACACACACAC  ACGGGACATTGTGTGGGCCC**C**CCACGTGTGCACACACACGA  CGCCTGGCGCCGCCACTGGA**C**ACACTGCATGCACGTCCCAT  TGGCCCGCCTGGCGCCGCCA**C**TGGACACACTGCATGCACGT  GTAAGGAGCCCTGGCTGCCC**C**CACGGGTCGGGCACACAAAC  GGCCCGGCCGTGCGGCCCGC**C**GGGGCCATGGCGAAGAAGAG  GGCCCGGCCGCCCCCCGGCC**C**CCGGCCGGCCCGCGCCCTCG  GCCCGCCCGCCGCCCCGTCC**C**CCGGCCCGGCCGCCCCCCGG  CGCCGCGACCCCGGCCCGGC**C**GTGCGGCCCGCCGGGGCCAT  CCCGCCCGCCGCCCCGTCCC**C**CGGCCCGGCCGCCCCCCGGC  TGAGGCGCCGCGACCCCGGC**C**CGGCCGTGCGGCCCGCCGGG  TGGACGCGCCAGGTGGACTT**C**ATCATGTCGTGCGTGGGCTT  CCCCTCATCGCGCCCGGGCC**C**GACGGGGCCCCGGCCAAGGG  CCGCCGCCCGCCCGCCGCCC**C**GTCCCCCGGCCCGGCCGCCC  GCGCCCGGGCCCGACGGGGC**C**CCGGCCAAGGGCGACGGCCC  TTCGCCGTGGGCTTGGGCAA**C**GTGTGGCGCGCCGCCCGCGC  CGGCCCGGCCGCCCCCCGGC**C**CCCGGCCGGCCCGCGCCCTC  GGCTTCGCCGTGGGCTTGGG**C**AACGTGTGGCGCGCCGCCCG  GACACCCGGCGGCCGCCTGG**C**CGTGCCGCCGCGCGAGACCT  GGCAACGTGTGGCGCGCCGC**C**CGCGCCCCGCACCCCGCCGC  CCCGCACCCCGCCGCCCGCC**C**GCCGCCCCGTCCCCCGGCCC  ATCTATAGCGTGTCCGGCGA**C**GAGAAGAAGGGCCCCCTCAT  CGCCGGTGCCCCCCGCCTGA**C**CGCCGCCCCCCGTGAGGCGC  CCGCGCGAGACCTGGACGCG**C**CAGGTGGACTTCATCATGTC  CCGGCCCGCGCCCTCGGGGC**C**CTCCCCGGTGCCGCCGGTGC  CGCCCGGGCCCGACGGGGCC**C**CGGCCAAGGGCGACGGCCCC  CCCGCCTGACCGCCGCCCCC**C**GTGAGGCGCCGCGACCCCGG  CCTCGGGGCCCTCCCCGGTG**C**CGCCGGTGCCCCCCGCCTGA  CGCGCGAGACCTGGACGCGC**C**AGGTGGACTTCATCATGTCG  TCCCCCGGCCCGGCCGCCCC**C**CGGCCCCCGGCCGGCCCGCG  TGCCCCCCGCCTGACCGCCG**C**CCCCCGTGAGGCGCCGCGAC  CCTCCCCGGTGCCGCCGGTG**C**CCCCCGCCTGACCGCCGCCC  GTCCCCCGGCCCGGCCGCCC**C**CCGGCCCCCGGCCGGCCCGC  ATCATGTCGTGCGTGGGCTT**C**GCCGTGGGCTTGGGCAACGT  GAAGAAGGGCCCCCTCATCG**C**GCCCGGGCCCGACGGGGCCC  GCACCCCGCCGCCCGCCCGC**C**GCCCCGTCCCCCGGCCCGGC  AGGGCGACGGCCCCGTGGGC**C**TGGGGACACCCGGCGGCCGC  CCCCGGCCAAGGGCGACGGC**C**CCGTGGGCCTGGGGACACCC  CCGGTGCCGCCGGTGCCCCC**C**GCCTGACCGCCGCCCCCCGT  GGGGCCCCGGCCAAGGGCGA**C**GGCCCCGTGGGCCTGGGGAC  CCGCCGCCCCCCGTGAGGCG**C**CGCGACCCCGGCCCGGCCGT  GGGCAACGTGTGGCGCGCCG**C**CCGCGCCCCGCACCCCGCCG  CGTCCCCCGGCCCGGCCGCC**C**CCCGGCCCCCGGCCGGCCCG  TCCCCGGTGCCGCCGGTGCC**C**CCCGCCTGACCGCCGCCCCC  GCCCGGCCGCCCCCCGGCCC**C**CGGCCGGCCCGCGCCCTCGG  GAACGGCATCTATAGCGTGT**C**CGGCGACGAGAAGAAGGGCC  CCGTCCCCCGGCCCGGCCGC**C**CCCCGGCCCCCGGCCGGCCC  CGCCTGGCCGTGCCGCCGCG**C**GAGACCTGGACGCGCCAGGT  CCCCGCACCCCGCCGCCCGC**C**CGCCGCCCCGTCCCCCGGCC  GCCCGCGCCCCGCACCCCGC**C**GCCCGCCCGCCGCCCCGTCC  GCCGGCCCGCGCCCTCGGGG**C**CCTCCCCGGTGCCGCCGGTG  GCCTGGGGACACCCGGCGGC**C**GCCTGGCCGTGCCGCCGCGC  CGCCCGCGCCCCGCACCCCG**C**CGCCCGCCCGCCGCCCCGTC  GTGCCGCCGCGCGCGATAGA**C**CTGTCAAGTGCTTGGTGGAC  CCGCTCTCCCGCCGCCCCGT**C**CCCCGGCCCGGCCGCCCCCC  GGCCTGGGGACACCCGGCGG**C**GGCCTGGCCGTGCCGCCGCG  TGCGCCGCCTGTGACCCGCA**C**CCCGCCGCTCTCCCGCCGCC  CTCTCCCGCCGCCCCGTCCC**C**CGGCCCGGCCGCCCCCCGGC  TCCCCCGGCCCGGCCGCCCC**C**CGGCCCCCGGCCGGTCCGCG  GGCATATGTAGCGTGTCCGG**C**GACCAGAAGAAGGGTCCCCT  GTCCGCGCCCTCGGGGCCCT**C**CCCGGTGCCGCCGGTGCCCC  CGCCGGTGCCCCGGGCCTGA**C**CGCAGCCCCCCGCAGGGTGC  GGTGCCGCCGGTGCCCCGGG**C**CTGACCGCAGCCCCCCGCAG  GCTCTCCCGCCGCCCCGTCC**C**CCGGCCCGGCCGCCCCCCGG  GGCCGTGAGGCCCGCAGGGG**C**CATGGCGAAGAAGAGCGCCG  CCCCGCGGGCCTGGGGACAC**C**CGGCGGCGGCCTGGCCGTGC  CTGGGGACACCCGGCGGCGG**C**CTGGCCGTGCCGCCGCGCGC  CCCCCCGCAGGGTGCCGCGA**C**CCCAGCCCGGCCGTGAGGCC  CGCTCTCCCGCCGCCCCGTC**C**CCCGGCCCGGCCGCCCCCCG  GCGCCGCCTGTGACCCGCAC**C**CCGCCGCTCTCCCGCCGCCC  CGGGCCCGACGGGGCCCGGG**C**CAAGGGCGACGGCCCCGCGG  GACACCCGGCGGCGGCCTGG**C**CGTGCCGCCGCGCGCGATAG  CCGGGCCAAGGGCGACGGCC**C**CGCGGGCCTGGGGACACCCG  CGCGCCCGGGCCCGACGGGG**C**CCGGGCCAAGGGCGACGGCC  GATCGGCATATGTAGCGTGT**C**CGGCGACCAGAAGAAGGGTC  CCGGCGGCGGCCTGGCCGTG**C**CGCCGCGCGCGATAGACCTG  CTGCCCCTGCGGGTCGGGCA**C**ACAAGGGGCACATTGTGTCC  TGTGTGGGCTTCGCCGTGGG**C**CTGGGCAACGTGTGGCGCTT  TCGGGGCCGCCCCTGGTCCA**C**CGCTGCTGCTCGGTGGCTGG  CCGCCGCGGAGGGGTGAAGT**C**CGGGCAGCGGTTGGCCCCTG  TCCCCCGCCCCCCGCGGCCC**C**CTCCCCCAGCAGGCCGCCGG  CCCTGACGCCCGACCCCCAA**C**CCCCGGAGCCGCCGCGGAGG  CTGGGCAACGTGTGGCGCTT**C**CCCTACCTGTGCTACAAGAA  GGCAGCGGTTGGCCCCTGGG**C**ACGCGGGGTCGGGGCCGCCC  TGGGCCGTCCGCCTCCACCC**C**TCTCGCAGTCATGTGCCTGG  CCTGACGCCCGACCCCCAAC**C**CCCGGAGCCGCCGCGGAGGG  TCCCCCAGCAGGCCGCCGGC**C**CCTGACGCCCGACCCCCAAC  TGTGTCCAGATGGACTTCAT**C**ATGTCGTGTGTGGGCTTCGC  CTGCTCGGTGGCTGGGCCGT**C**CGCCTCCACCCCTCTCGCAG  GTGAGCTCCCCCGCCCCCCG**C**GGCCCCCTCCCCCAGCAGGC  AGTCCGGGCAGCGGTTGGCC**C**CTGGGCACGCGGGGTCGGGG  CCCCTGGTCCACCGCTGCTG**C**TCGGTGGCTGGGCCGTCCGC  GGCCGGCGATGCCCGCGAGG**C**TGCCCCCCAGACTCCTGGGC  CCGCCCCTGGTCCACCGCTG**C**TGCTCGGTGGCTGGGCCGTC  GGCCGTCCGCCTCCACCCCT**C**TCGCAGTCATGTGCCTGGCA  CGCCCCCCGCGGCCCCCTCC**C**CCAGCAGGCCGCCGGCCCCT  GGAGGTGAGCTCCCCCGCCC**C**CCGCGGCCCCCTCCCCCAGC  GGCCCCCTCCCCCAGCAGGC**C**GCCGGCCCCTGACGCCCGAC  GCGGTTGGCCCCTGGGCACG**C**GGGGTCGGGGCCGCCCCTGG  GCTCCCCCGCCCCCCGCGGC**C**CCCTCCCCCAGCAGGCCGCC  AGGTGAGCTCCCCCGCCCCC**C**GCGGCCCCCTCCCCCAGCAG  GGCAACGTGTGGCGCTTCCC**C**TACCTGTGCTACAAGAATGG  CCGGCCCCTGACGCCCGACC**C**CCAACCCCCGGAGCCGCCGC  AATGGCGGAGGTGAGCTCCC**C**CGCCCCCCGCGGCCCCCTCC  CTGGGCCGTCCGCCTCCACC**C**CTCTCGCAGTCATGTGCCTG  CGGAGGTGAGCTCCCCCGCC**C**CCCGCGGCCCCCTCCCCCAG  TGGCCGCCGAGGTGGGAAAG**C**AGGCCTGCGCCTTGGGGTCT  CCCTGGCTGCCCCTGCGGGT**C**GGGCACACAAGGGGCACATT  CGGCCCCCTCCCCCAGCAGG**C**CGCCGGCCCCTGACGCCCGA  CAGGCCGCCGGCCCCTGACG**C**CCGACCCCCAACCCCCGGAG  CCAGATGGACTTCATCATGT**C**GTGTGTGGGCTTCGCCGTGG  CAGAGGTCCAGAGCAAAGGC**C**TGTCTCCGCGAGGTAAGGAG  CCTCCCCTCCCCTCGCAGCC**C**TCCCGCTCCTCCCCTCTGGC  GTAAGGAGCCCTGGCTGCCC**C**TGCGGGTCGGGCACACAAGC  GTGGCCTGGCCCGCCTGGCG**C**CGCCACTGGAAACACTGCAT  CCTCTGGCCCGGGGCTGGAA**C**ACTGGGTACCTGAGCCAGGC  GTTTCTGGAAGCCGAAGTGA**C**CCAGTGATGGGTGGGAAACA  CTCCCGCTCCTCCCCTCTGG**C**CCGGGGCTGGAACACTGGGT  GCAGCCCTCCCGCTCCTCCC**C**TCTGGCCCGGGGCTGGAACA  CGGGGCTGGAACACTGGGTA**C**CTGAGCCAGGCTTGGGAAGC  GTCCTGTGACTACAAAACAG**C**ACTGGGGTTTCTGGAAGCCG  TGCACACACACGAACACACA**C**ACATATAATGGGCCACTCTG  CCCTCTCCCTGCCCTCCCCT**C**CCCTCGCAGCCCTCCCGCTC  GCATGCACGTCCCATGCCCG**C**CCGCCTGCCCGGGCCCCGCT  CACTCTGTCCCTCTCCCTGC**C**CTCCCCTCCCCTCGCAGCCC  ACGCGTGTGTCCTGTGACTA**C**AAAACAGCACTGGGGTTTCT  CTGAGCCAGGCTTGGGAAGC**C**TGTGGCCTGGCCCGCCTGGC  TTGGGAAGCCTGTGGCCTGG**C**CCGCCTGGCGCCGCCACTGG  CACACACATATAATGGGCCA**C**TCTGTCCCTCTCCCTGCCCT  GCACGTCCCATGCCCGCCCG**C**CTGCCCGGGCCCCGCTTAGC  CCCCTCCCCTCGCAGCCCTC**C**CGCTCCTCCCCTCTGGCCCG  AAGCCTGTGGCCTGGCCCGC**C**TGGCGCCGCCACTGGAAACA  TGGGAAACAGAGGTCCAGAG**C**AAAGGCCTGTCTCCGCGAGG  ACTGCATGCACGTCCCATGC**C**CGCCCGCCTGCCCGGGCCCC  CCTCTCCCTGCCCTCCCCTC**C**CCTCGCAGCCCTCCCGCTCC  ACACACACACATATAATGGG**C**CACTCTGTCCCTCTCCCTGC  TTCTGGAAGCCGAAGTGACC**C**AGTGATGGGTGGGAAACAGA  CCAGAGCAAAGGCCTGTCTC**C**GCGAGGTAAGGAGCCCTGGC  TCTGTCCCTCTCCCTGCCCT**C**CCCTCCCCTCGCAGCCCTCC  CATATAATGGGCCACTCTGT**C**CCTCTCCCTGCCCTCCCCTC  ATGGGCCACTCTGTCCCTCT**C**CCTGCCCTCCCCTCCCCTCG  CCTGCCCGGGCCCCGCTTAG**C**AAGAGCGATGGGCACGCGTG  CACTGCATGCACGTCCCATG**C**CCGCCCGCCTGCCCGGGCCC  CCCGCTCCTCCCCTCTGGCC**C**GGGGCTGGAACACTGGGTAC  GTGGGCCCCCCACGTGTGCA**C**ACACACGAACACACACACAT  CGTGTGCACACACACGAACA**C**ACACACATATAATGGGCCAC  GAAACACTGCATGCACGTCC**C**ATGCCCGCCCGCCTGCCCGG  TCCCCTCCCCTCGCAGCCCT**C**CCGCTCCTCCCCTCTGGCCC  CTGCCCCTGCGGGTCGGGCA**C**ACAAGCGGCACATTGTGTGG  AGCACTGGGGTTTCTGGAAG**C**CGAAGTGACCCAGTGATGGG  AGGCTTGGGAAGCCTGTGGC**C**TGGCCCGCCTGGCGCCGCCA  CGCAGCCCTCCCGCTCCTCC**C**CTCTGGCCCGGGGCTGGAAC  GCCCGCCTGCCCGGGCCCCG**C**TTAGCAAGAGCGATGGGCAC  TGTCCCTCTCCCTGCCCTCC**C**CTCCCCTCGCAGCCCTCCCG  GAGGTAAGGAGCCCTGGCTG**C**CCCTGCGGGTCGGGCACACA  AGCGATGGGCACGCGTGTGT**C**CTGTGACTACAAAACAGCAC  CACGTGTGCACACACACGAA**C**ACACACACATATAATGGGCC  CGCCGCCACTGGAAACACTG**C**ATGCACGTCCCATGCCCGCC  GCCGAGATCGCGCCACTGCA**C**TCCAGCCTGGGCCACAGCGT  CAGCGGTCTTAGGGAATTCC**C**CCCGCGATGTCCCGGCGCGC  CGGAGGTTGCAGTGAGCCGC**C**GAGATCGCGCCACTGCACTC  CCGGCGCGCCAGTTCGCTTG**C**GCACACTTCGCTGCGGTCCT  TGCGGTCCTCTTCGACCAGC**C**CGGCCAACATGGTGAAACCC  GCAATTTCCTTTCCTCTAAG**C**GGCCTCCACCCCTCTCCCCT  AGTGAGCCGCCGAGATCGCG**C**CACTGCACTCCAGCCTGGGC  GGGAATTCCCCCCGCGATGT**C**CCGGCGCGCCAGTTCGCTTG  GTGAAGCGGGTGTGCAAGCT**C**CGGGATCGCAGCGGTCTTAG  ATAAAGCAATTTCCTTTCCT**C**TAAGCGGCCTCCACCCCTCT  TGTGGCACGCACCTATAATT**C**CACCTACTCGGGAGGCTGAA  GAGGCGGAGGTTGCAGTGAG**C**CGCCGAGATCGCGCCACTGC  ATGGTGAAACCCCGTCTCTA**C**TAAAAATACAAAAATCAGCC  TTCGCTGCGGTCCTCTTCGA**C**CAGCCCGGCCAACATGGTGA  AATTCCACCTACTCGGGAGG**C**TGAAGCAGAATTGCTTGAAC  CTGCGGTCCTCTTCGACCAG**C**CCGGCCAACATGGTGAAACC  CCCCCCGCGATGTCCCGGCG**C**GCCAGTTCGCTTGCGCACAC  GCACACTTCGCTGCGGTCCT**C**TTCGACCAGCCCGGCCAACA  CCCGCGATGTCCCGGCGCGC**C**AGTTCGCTTGCGCACACTTC  CCGCCGAGATCGCGCCACTG**C**ACTCCAGCCTGGGCCACAGC  CGCCAGTTCGCTTGCGCACA**C**TTCGCTGCGGTCCTCTTCGA  ATCGCGCCACTGCACTCCAG**C**CTGGGCCACAGCGTGAGACT  CGCGCCAGTTCGCTTGCGCA**C**ACTTCGCTGCGGTCCTCTTC  CTAAAAATACAAAAATCAGC**C**AGATGTGGCACGCACCTATA  AAGCGGCCTCCACCCCTCTC**C**CCTGCCCTGTGAAGCGGGTG  TTCCCCCCGCGATGTCCCGG**C**GCGCCAGTTCGCTTGCGCAC  TGCAAGCTCCGGGATCGCAG**C**GGTCTTAGGGAATTCCCCCC  TTTCCTCTAAGCGGCCTCCA**C**CCCTCTCCCCTGCCCTGTGA  ACTAAAAATACAAAAATCAG**C**CAGATGTGGCACGCACCTAT  TGCGCACACTTCGCTGCGGT**C**CTCTTCGACCAGCCCGGCCA  CACTCCAGCCTGGGCCACAG**C**GTGAGACTACGTCATAAAAT  CTGAAGCAGAATTGCTTGAA**C**CCGAGAGGCGGAGGTTGCAG  GAGATGCTGGAGACCCCGCG**C**ACAGGAAAGCCCCTGCAGTG  CAGCATTCAGTCAATCCGGG**C**CGGGAGCAGTCATCTGTGGT  TGCGCGTTTCTCTACTTGCC**C**TTTCTAGAGAGGTGCAACGG  CTTCGTGGAGATGCTGGAGA**C**CCCGCGCACAGGAAAGCCCC  GCTGATTGGCTGGGCAGGAA**C**AGCGCCGGGGCGTGGGCTGA  AGACCCCGCGCACAGGAAAG**C**CCCTGCAGTGCCCATCGCGG  TTTCCTGAACTTGGTCTTCA**C**GGGAGAAGGGCTTCTTGAGG  TAGGAAGCAGAAAGGTGATA**C**AGAATTGGAGAGGTCGGAGT  AAAGCCCCTGCAGTGCCCAT**C**GCGGCCAGAGCAGCTGGGGC  AACTGTATTAAATGCGAATC**C**CGAGAAAATTTCCCTTAACT  GGGACCAAGTGGGGTTAGAT**C**TAGACTCAGGAGCTCCTGGA  TACATAAGTTGAAATGTCCC**C**AATGATTCAGCTGATGCGCG  CAGGAGCTCCTGGAGCAGCG**C**CCAAACCGTAGTGGCACTGG  CTGAGCACAGCCGCTTCGCT**C**TCTTTGCCACAGGAAGCCTG  GCAAAGTCCAGAACGCGCTG**C**CAGACCCCCAACTCTGCCTT  AGTGGGGTTAGATCTAGACT**C**AGGAGCTCCTGGAGCAGCGC  TCAGATTTCCTGAACTTGGT**C**TTCACGGGAGAAGGGCTTCT  GTGAAGTCCTCTGGCAAGTC**C**ATGGGGACCAAGTGGGGTTA  TTTTTTAAAGAAAAATTTCT**C**CTAGCCTTTTCAAAGGTGTT  ACGGGGGACTAGAGGTTAGT**C**TCACCTCCAGCGCGCCTGAG  CGGTGGGTGGGAGGAAGCAT**C**GTCCGCGGCGACTGGAACCG  AGGCGTGGATAGTGTGAAGT**C**CTCTGGCAAGTCCATGGGGA  ACGGAAGCCAGAACATTCCT**C**CTGGAAATTCAACCTGTTTC  TCCGGGCCGGGAGCAGTCAT**C**TGTGGTGAGGCTGATTGGCT  CGAGGTAGGGGCACGCAAAG**C**TGGGAGCTACTATGGGACAG  CGGGGACCTGCTCTCTGAGC**C**CGCGGGCGGTGGGTGGGAGG  ATGGATCTTGAAGGGGACCG**C**AATGGAGGAGCAAAGAAGAA  TTCAACCTGTTTCGCAGTTT**C**TCGAGGAATCAGCATTCAGT  GCCGGGGCGTGGGCTGAGCA**C**AGCCGCTTCGCTCTCTTTGC  GCAGAGGCCGCTGTTCGTTT**C**CTTTAGGTCTTTCCACTAAA  GGACCATGTTGCCCGGAGCG**C**GCACAGCCCGCGCGGTGCGG  GGAATCAGCATTCAGTCAAT**C**CGGGCCGGGAGCAGTCATCT  GATTCAGCTGATGCGCGTTT**C**TCTACTTGCCCTTTCTAGAG  TAGTTATATGGATATGAAGA**C**TTATGTGAACTTTGAAAGAC  ACGGCGGGCGCTCCCTCTTA**C**TGCTCTCTGGCTTCGACGGG  GGGCATCAACGGCGGGCGCT**C**CCTCTTACTGCTCTCTGGCT  CAAGGAGCGCGAGGTAGGGG**C**ACGCAAAGCTGGGAGCTACT  AAAATTTCTCCTAGCCTTTT**C**AAAGGTGTTAGGAAGCAGAA  TGGCTAATGAGCTGCGGTTT**C**TCTTCAGGTCGGGATGGATC  GGGTGGGAGGAAGCATCGTC**C**GCGGCGACTGGAACCGGGAG  CGAGTAGCGGCTCTTCCAAG**C**TCAAAGAAGCAGAGGCCGCT  ACTTTTTTAAAGAAAAATTT**C**TCCTAGCCTTTTCAAAGGTG  GATTTCCTGAACTTGGTCTT**C**ACGGGAGAAGGGCTTCTTGA  TAGAGAGGTGCAACGGAAGC**C**AGAACATTCCTCCTGGAAAT  AGCCCGCGCGGTGCGGGGAC**C**TGCTCTCTGAGCCCGCGGGC  GCGAATCCCGAGAAAATTTC**C**CTTAACTACGTCCTGTAGTT  TGAGCTCATTCGAGTAGCGG**C**TCTTCCAAGCTCAAAGAAGC  CAACGGAAGCCAGAACATTC**C**TCCTGGAAATTCAACCTGTT  TTGGTGGCCGTTCCAAGGAG**C**GCGAGGTAGGGGCACGCAAA  GGAGCTCCTGGAGCAGCGCC**C**AAACCGTAGTGGCACTGGAC  GCAGTTTCTCGAGGAATCAG**C**ATTCAGTCAATCCGGGCCGG  GCCCATCGCGGCCAGAGCAG**C**TGGGGCATCAACGGCGGGCG  GGGGCACGCAAAGCTGGGAG**C**TACTATGGGACAGTTCCCAA  CCTGAGGCTCATGCATTTGG**C**TAATGAGCTGCGGTTTCTCT  TGATACAGAATTGGAGAGGT**C**GGAGTTTTTGTATTAACTGT  TCTGGCAAGTCCATGGGGAC**C**AAGTGGGGTTAGATCTAGAC  GTCCAGAACGCGCTGCCAGA**C**CCCCAACTCTGCCTTCGTGG  AGCTCATTCGAGTAGCGGCT**C**TTCCAAGCTCAAAGAAGCAG  GGAGGAGCAAAGAAGAAGAA**C**TTTTTTAAAGAAAAATTTCT  GTGTCAGGCTTTCAGATTTC**C**TGAACTTGGTCTTCACGGGA  CAGAGCAGCTGGGGCATCAA**C**GGCGGGCGCTCCCTCTTACT  AACTTTGAAAGACGTGTCTA**C**ATAAGTTGAAATGTCCCCAA  CAGACCCCCAACTCTGCCTT**C**GTGGAGATGCTGGAGACCCC  AGATCTAGACTCAGGAGCTC**C**TGGAGCAGCGCCCAAACCGT  TATCTTCTTCCAAAATTTCA**C**GTCTTGGTGGCCGTTCCAAG  CCAGAACGCGCTGCCAGACC**C**CCAACTCTGCCTTCGTGGAG  GCGCCGGGGCGTGGGCTGAG**C**ACAGCCGCTTCGCTCTCTTT  GGGCGGTGGGTGGGAGGAAG**C**ATCGTCCGCGGCGACTGGAA  TTTTCAAAGGTGTTAGGAAG**C**AGAAAGGTGATACAGAATTG  TAAAGTCGGAGTATCTTCTT**C**CAAAATTTCACGTCTTGGTG  GCCTGAGCTCATTCGAGTAG**C**GGCTCTTCCAAGCTCAAAGA  GGACTAGAGGTTAGTCTCAC**C**TCCAGCGCGCCTGAGGCTCA  CGTGGAGATGCTGGAGACCC**C**GCGCACAGGAAAGCCCCTGC  CTACATAAGTTGAAATGTCC**C**CAATGATTCAGCTGATGCGC  TGCGAATCCCGAGAAAATTT**C**CCTTAACTACGTCCTGTAGT  GTTAGATCTAGACTCAGGAG**C**TCCTGGAGCAGCGCCCAAAC  CTGGGAGAGGGGTTCCGGCC**C**CCGACGTCGCTGGCGCGGGA  GGGGGCGGGCCTGGCGGGCG**C**CCTCTCCGGGCCCTTTGTTA  GGCGCGCGGGCCGAGCCGGG**C**CTGAGCCGGGCCCGCGGACC  GTTAACAGGCGCGTCCCGGC**C**AGGCGGAGACGCGGCCGCGG  CGGGGCGGCGGTGAGGGCGG**C**TGGCGGGGCCGGGGGCGCCG  GCTGGGAGAGGGGTTCCGGC**C**CCCGACGTCGCTGGCGCGGG  ACCGCCCCCGCCCCGCCCCG**C**CCCTACCCGCTCCTCGGCGC  CTTCAGTCCCGCGACCGAAG**C**AGGGCGCGCAGCAGCGCTGA  CAAGCCCCACCCGGCCCAAG**C**CGCGCGTCCCGCACTGAGCT  ACACGCGCCGGCCCCGGCCG**C**ACCCCGCGCACGCAGAGCAA  CAGCGCTGAGTGCCCCGGAA**C**GTGCGTCGCGCCCCCAGTGT  CCCCCAGTGTCCGTCGCGTC**C**GCCGCGCCCCGGGCGGGGAT  TGGCCCACCCCTGGACCGCC**C**CCGCCCCGCCCCGCCCCTAC  TGCGTCGCGCCCCCAGTGTC**C**GTCGCGTCCGCCGCGCCCCG  CCGGCCCCGGCCGCACCCCG**C**GCACGCAGAGCAAGCACTGG  CCGCCCCCGCCCCGCCCCGC**C**CCTACCCGCTCCTCGGCGCA  CCCCGCTCCTCCCCAAGCCC**C**ACCCGGCCCAAGCCGCGCGT  CCACCCGCCTCCGGCCCCGC**C**TGGCCCACCCCTGGACCGCC  GCTCCTACACGCGCCGGCCC**C**GGCCGCACCCCGCGCACGCA  CCCGCCCCGCCCCGCCCCTA**C**CCGCTCCTCGGCGCAGCCGG  GCTGAGTGCCCCGGAACGTG**C**GTCGCGCCCCCAGTGTCCGT  GGCCCCGGCCGCACCCCGCG**C**ACGCAGAGCAAGCACTGGAG  CGCTCCCGCACCCCGCTCCT**C**CCCAAGCCCCACCCGGCCCA  ACGCAGAGCAAGCACTGGAG**C**CCCGCCCCTTCCCGCACCCC  CCTGGACCGCCCCCGCCCCG**C**CCCGCCCCTACCCGCTCCTC  AGCTCCTACACGCGCCGGCC**C**CGGCCGCACCCCGCGCACGC  ACCCCTGGACCGCCCCCGCC**C**CGCCCCGCCCCTACCCGCTC  CCTGGCCCACCCCTGGACCG**C**CCCCGCCCCGCCCCGCCCCT  CGCAGAGCAAGCACTGGAGC**C**CCGCCCCTTCCCGCACCCCA  GGCCCACCCCTGGACCGCCC**C**CGCCCCGCCCCGCCCCTACC  CGCACCCCACCCGCCTCCGG**C**CCCGCCTGGCCCACCCCTGG  CCCCGCTCCCGCACCCCGCT**C**CTCCCCAAGCCCCACCCGGC  ACCCCGCTCCTCCCCAAGCC**C**CACCCGGCCCAAGCCGCGCG  CGAAGCAGGGCGCGCAGCAG**C**GCTGAGTGCCCCGGAACGTG  AGCCGGCGCTTGCCTAGCTT**C**AGTCCCGCGACCGAAGCAGG  GCTCCTCCCCAAGCCCCACC**C**GGCCCAAGCCGCGCGTCCCG  GCCCCGGAACGTGCGTCGCG**C**CCCCAGTGTCCGTCGCGTCC  CCACCCCTGGACCGCCCCCG**C**CCCGCCCCGCCCCTACCCGC  GCCCACCCCTGGACCGCCCC**C**GCCCCGCCCCGCCCCTACCC  CTACACGCGCCGGCCCCGGC**C**GCACCCCGCGCACGCAGAGC  CACCCCGCTCCCGCACCCCG**C**TCCTCCCCAAGCCCCACCCG  CGCGCCGGCCCCGGCCGCAC**C**CCGCGCACGCAGAGCAAGCA  CCCCGGAACGTGCGTCGCGC**C**CCCAGTGTCCGTCGCGTCCG  GAGCAAGCACTGGAGCCCCG**C**CCCTTCCCGCACCCCACCCG  GGGCGGGGATGCACCCCGCT**C**CCGCACCCCGCTCCTCCCCA  CCGCACTGAGCTCCTACACG**C**GCCGGCCCCGGCCGCACCCC  CACCCCGCTCCTCCCCAAGC**C**CCACCCGGCCCAAGCCGCGC  GCCCCTTCCCGCACCCCACC**C**GCCTCCGGCCCCGCCTGGCC  CGCAGCAGCGCTGAGTGCCC**C**GGAACGTGCGTCGCGCCCCC  GCGCAGCAGCGCTGAGTGCC**C**CGGAACGTGCGTCGCGCCCC  CCTACACGCGCCGGCCCCGG**C**CGCACCCCGCGCACGCAGAG  CCGTCGCGTCCGCCGCGCCC**C**GGGCGGGGATGCACCCCGCT  CGCCCCTTCCCGCACCCCAC**C**CGCCTCCGGCCCCGCCTGGC  GCAGAGCAAGCACTGGAGCC**C**CGCCCCTTCCCGCACCCCAC  CCACCCCAGGTATTGGAATT**C**ACAAATGGCAATGCTGTGGG  CCCCTATCACCATCTATGAT**C**CCACGTCTGCTGAATCAGTT  GCTTACTCTCTGCAAAGAAT**C**CTTTGTGTGTAAAGACCAGG  TGATCCCACGTCTGCTGAAT**C**AGTTGTGGGGTTTATACGCG  ATTGCGCCAAACCTAAAGAG**C**CCCCCCACCCCAGGTATTGG  AACTGATTGAGTTTTCTCCC**C**TATCACCATCTATGATCCCA  AACCTAAAGAGCCCCCCCAC**C**CCCGGTATTGGAATTCACAA  GTAACTGATTGAGTTTTCTC**C**CCTATCACCATCTATGATCC  TAACTGATTGAGTTTTCTCC**C**CTATCACCATCTATGATCCC  CCTCAGGGGGTCACAAATGC**C**ACTAGGGGGGCAGGACACAT  AATCCTTTGTGTGTAAAGAC**C**AGGGTTGTCGCACGGCGCTC  TGGTACCTCGTGGACTCGGA**C**TCCCAAATCAACAAGATCGG  GGGTCACCCAAGTTCAGTAC**C**TCAGGGGGTCACAAATGCCA  ACCTCGTGGACTCGGACTCC**C**AAATCAACAAGATCGGCTTA  CGGCGCTCGTACATCGCAGT**C**CTGAAACGGATTGTGCAGTG  GGACACATGCATTTTCTAGG**C**TGGTACCTCGTGGACTCGGA  GACTCGGACTCCCAAATCAA**C**AAGATCGGCTTACTCTCTGC  CTAGGGGGGCAGGACACATG**C**ATTTTCTAGGCTGGTACCTC  CGCACGGCGCTCGTACATCG**C**AGTCCTGAAACGGATTGGGC  ACCCCTGGTATTGGAATTCA**C**AAATGGCAATGCTGTGGGTC  TGAGTTTTCTCCCCTATCAC**C**ATCTATGATCCCACGTCTGC  TGGGTCACCCAAGTTCAGTA**C**CTCAGGGGGTCACAAATGCC  GTACCTCGTGGACTCGGACT**C**CCAAATCAACAAGATCGGCT  GCGCCAAACCTAAAGAGCCC**C**CCCACCCCTGGTATTGGAAT  TGCGCCAAACCTAAAGAGCC**C**CCCCACCCCTGGTATTGGAA  GTATTGGAATTCACAAATGG**C**AATGCTGTGGGTCACCCAAG  GGCGCTCGTACATCGCAGTC**C**TGAAACGGATTGAGCAGTGA  ACCTAAAGAGCCCCCCCACC**C**CTGGTATTGGAATTCACAAA  GCAAAATCGATTGCGCCAAA**C**CTAAAGAGCCCCCCCACCCC  TTGCGCCAAACCTAAAGAGC**C**CCCCCACCCCCGGTATTGGA  CTGGGGTGACCAAGACCAGA**C**TGTAAGATAACTAATAAAAC  GTAAGATAACTAATAAAACT**C**ATCGCAAGAGAGTCCTGACA  GATTTCAGCTGCATAACTTT**C**AACAGAAGCTAAGAGCAATG  AAAACTCATCGCAAGAGAGT**C**CTGACAAACGTGACGACTCA  ACAGGGCATCTCTCCTGTCC**C**CATTCTATCACTGATAGTAG  TGGAGGGACAGGGCATCTCT**C**CTGTCCCCATTCTATCACTG  ACGTGGCTGGAGGGACAGGG**C**ATCTCTCCTGTCCCCATTCT  ACTTCAGTAGGATAGGGAGA**C**CATACTGTGCCCTCCGGTTG  TCCTGACAAACGTGACGACT**C**ACCCAGAAAAGGGACGTCCT  AAGACCAGACTGTAAGATAA**C**TAATAAAACTCATCGCAAGA  TTGACTGGGGTGACCAAGAC**C**AGACTGTAAGATAACTAATA  CTGCCATCTGGAGCTCCGGT**C**ACTTCCATCTAGAGCTGCTC  TCACCCAGAAAAGGGACGTC**C**TCCTGCCATCTGGAGCTCCG  GGAGCTCCGGTCACTTCCAT**C**TAGAGCTGCTCTACTCTCTG  GATAGGGAGACCATACTGTG**C**CCTCCGGTTGACTGGGGTGA  AAGGGACGTCCTCCTGCCAT**C**TGGAGCTCCGGTCACTTCCA  ATAACTAATAAAACTCATCG**C**AAGAGAGTCCTGACAAACGT  ACCCAGAAAAGGGACGTCCT**C**CTGCCATCTGGAGCTCCGGT  ATCTGGAGCTCCGGTCACTT**C**CATCTAGAGCTGCTCTACTC  TGCATAACTTTCAACAGAAG**C**TAAGAGCAATGGTATACGCA  GACTCTGTGGATTTCAGCTG**C**ATAACTTTCAACAGAAGCTA  CTGACAAACGTGACGACTCA**C**CCAGAAAAGGGACGTCCTCC  TGGTATACGCACGCACGTGG**C**TGGAGGGACAGGGCATCTCT  CACGCACGTGGCTGGAGGGA**C**AGGGCATCTCTCCTGTCCCC  TGGCTGGAGGGACAGGGCAT**C**TCTCCTGTCCCCATTCTATC  TCATCGCAAGAGAGTCCTGA**C**AAACGTGACGACTCACCCAG  CCCGCGGGAAGACGTCTCAG**C**CTGGGCTGCCCCCAGACCCC  TCCAGCGCCGCCCTTGCTGC**C**AGCCCTGGCCCACCCGCGGG  GCCACTCCAAATAGAGAGCC**C**CAAATATTCCAGCGCCGCCC  GACCCCTGCTCGGTTCCCGC**C**AGGAAACATCCGGGTGCCCG  GCCTGGGCTGCCCCCAGACC**C**CTGCTCGGTTCCCGCCAGGA  AGCGCCGCCCTTGCTGCCAG**C**CCTGGCCCACCCGCGGGAAG  TGCCAGCCCTGGCCCACCCG**C**GGGAAGACGTCTCAGCCTGG  CCCCAGACCCCTGCTCGGTT**C**CCGCCAGGAAACATCCGGGT  CGCCACTCCAAATAGAGAGC**C**CCAAATATTCCAGCGCCGCC  ATAGAGAGCCCCAAATATTC**C**AGCGCCGCCCTTGCTGCCAG  GAAGGAACCTGACCCAGGCT**C**TGTGAGGAGGCAAGGTGAGA  CCCCAAATATTCCAGCGCCG**C**CCTTGCTGCCAGCCCTGGCC  GGCTGAGGGAGGACTGAGGA**C**CCCGCCACTCCAAATAGAGA  CGCCGCCCTTGCTGCCAGCC**C**TGGCCCACCCGCGGGAAGAC  CCGCCACTCCAAATAGAGAG**C**CCCAAATATTCCAGCGCCGC  CCACTCCAAATAGAGAGCCC**C**AAATATTCCAGCGCCGCCCT  GCCCACCCGCGGGAAGACGT**C**TCAGCCTGGGCTGCCCCCAG  CGGGTGCCCGGATGTGACGC**C**ACTGACTTGCGCATTGTGGG  CCCAGACCCCTGCTCGGTTC**C**CGCCAGGAAACATCCGGGTG  AGAGAAGCGAGGTTTCCATT**C**TGAGGGACGGCGTAGAGTTC  GGTGTCCATTGAAGAGCCAC**C**AGGGCATGCAGGCGGGATAG  ACCCACACCCATGCTGCCCC**C**CTCCCCCCATCCACCAGCCT  TGTCTCCCCCCACCCACACC**C**ATGCTGCCCCCCTCCCCCCA  GCAGCAATCACAACCTGTGG**C**CTGTTTCAGAGCCTAATCTC  GCTTTCTGCCCTCCAGAGGT**C**TCTGGAGAAGCCGCTGACTC  AAGGGCATCTGAGAGGGCAG**C**AATCACAACCTGTGGCCTGT  TTAAGGTGTCCATTGAAGAG**C**CACCAGGGCATGCAGGCGGG  TGCCTTCCTTTTCCTAAGGG**C**ATCTGAGAGGGCAGCAATCA  GGCCAGGGTATGCATGTGTT**C**TTCTTCCTCTATGTGGCTGC  CTCTGGAGAAGCCGCTGACT**C**ACACCCCGGAGATGAACTCC  GGCAGGACTGAGAGAGCCCC**C**ACCCCACCCATTTTCCACCC  TCACACCCCGGAGATGAACT**C**CTGGACTTTGCCAGGTAGGA  AGCCTCTATCCCTGGCTTTT**C**TGGCCTCTCCGAGTTTGTGC  TAATCTCTGCTTTCTGCCCT**C**CAGAGGTCTCTGGAGAAGCC  TTTCAAATTCCAGTCTGCAT**C**AATCACCCCCGTGTCCCATT  CTATAGATTTGAGGAAGCTT**C**CAGCTAGGAAGACCGGTATG  ACCGGTATGGTGATGTTCCT**C**ATTCCAGGGAGACTTGTAAA  GAACATCTGGCCTGTCTCCC**C**CCACCCACACCCATGCTGCC  TCCCCAAGAAGGGGCACAGG**C**CAGAGATGAGCAAGGTTCTG  ACACCCATGCTGCCCCCCTC**C**CCCCATCCACCAGCCTCTAT  TGAGAGGGCAGCAATCACAA**C**CTGTGGCCTGTTTCAGAGCC  CCACCCCACCCATTTTCCAC**C**CGGAGCATGAAGCACGTGGG  TACCTTTCCCCAAGAAGGGG**C**ACAGGCCAGAGATGAGCAAG  CCCATTCCAAGAACATCTGG**C**CTGTCTCCCCCCACCCACAC  GCATCTCCATATTATCTTGG**C**AGGACTGAGAGAGCCCCCAC  CCAGTCTGCATCAATCACCC**C**CGTGTCCCATTCCAAGAACA  CCATTGAAGAGCCACCAGGG**C**ATGCAGGCGGGATAGGGTGG  AAGCCCTCTGCTAAGGGTCT**C**CTTTAGAGAAAAAAAAAATG  TGCCCCCCTCCCCCCATCCA**C**CAGCCTCTATCCCTGGCTTT  GGCCAGATTAGGCTGCAAGC**C**CTCTGCTAAGGGTCTCCTTT  GCATCTGAGAGGGCAGCAAT**C**ACAACCTGTGGCCTGTTTCA  CAGGACTGAGAGAGCCCCCA**C**CCCACCCATTTTCCACCCGG  AGGCCAGATTAGGCTGCAAG**C**CCTCTGCTAAGGGTCTCCTT  GGACTGAGAGAGCCCCCACC**C**CACCCATTTTCCACCCGGAG  ATTCCAGTCTGCATCAATCA**C**CCCCGTGTCCCATTCCAAGA  TGTTTGAGAACACCTCTAGT**C**CTTGTGAGGCCAGATTAGGC  GTTTCAGAGCCTAATCTCTG**C**TTTCTGCCCTCCAGAGGTCT  AAGGTTCTGGGAACAGAGTC**C**CGATTCTGGAGTCCTGCCTT  TCTATCCCTGGCTTTTCTGG**C**CTCTCCGAGTTTGTGCCTAT  ATCTGAGAGGGCAGCAATCA**C**AACCTGTGGCCTGTTTCAGA  AGGTTCTGGGAACAGAGTCC**C**GATTCTGGAGTCCTGCCTTC  TAGACAGGGCGAGGCATCTC**C**ATATTATCTTGGCAGGACTG  TATGTGGCTGCCCACAGAGC**C**TTCACCTGACCTTATTTAGT  AAAAAGGTGTTTGAGAACAC**C**TCTAGTCCTTGTGAGGCCAG  CTTGGCAGGACTGAGAGAGC**C**CCCACCCCACCCATTTTCCA  CCCCAAGAAGGGGCACAGGC**C**AGAGATGAGCAAGGTTCTGG  GGGACCTAGACAGGGCGAGG**C**ATCTCCATATTATCTTGGCA  AGAGTCCCGATTCTGGAGTC**C**TGCCTTCCTTTTCCTAAGGG  GTATGGTGATGTTCCTCATT**C**CAGGGAGACTTGTAAATTTG  GCCGGCAGAGGGGTTCTCTA**C**CTTTCCCCAAGAAGGGGCAC  AATCTCTGCTTTCTGCCCTC**C**AGAGGTCTCTGGAGAAGCCG  TTCCAGTCTGCATCAATCAC**C**CCCGTGTCCCATTCCAAGAA  AGATTTGAGGAAGCTTCCAG**C**TAGGAAGACCGGTATGGTGA  CCCATCCACCAGCCTCTATC**C**CTGGCTTTTCTGGCCTCTCC  TTTAGAGAAAAAAAAAATGG**C**AAGGGCCGGCAGAGGGGTTC  CCCTGGCTTTTCTGGCCTCT**C**CGAGTTTGTGCCTATAGATT  GCCCCCACCCCACCCATTTT**C**CACCCGGAGCATGAAGCACG  AGGTAGGAAAACGCTGAGGT**C**ACCTTAAGGTGTCCATTGAA  GCCAGATTAGGCTGCAAGCC**C**TCTGCTAAGGGTCTCCTTTA  GCAAGCCCTCTGCTAAGGGT**C**TCCTTTAGAGAAAAAAAAAA  AGCCTAATCTCTGCTTTCTG**C**CCTCCAGAGGTCTCTGGAGA  AGCACGTGGGGAATGTTCCC**C**TTTCAAATTCCAGTCTGCAT  ACCCATGCTGCCCCCCTCCC**C**CCATCCACCAGCCTCTATCC  TGGAGTCCTGCCTTCCTTTT**C**CTAAGGGCATCTGAGAGGGC  ATGCATGTGTTCTTCTTCCT**C**TATGTGGCTGCCCACAGAGC  GATTCTGGAGTCCTGCCTTC**C**TTTTCCTAAGGGCATCTGAG  GGCTGCAGGACTACTTAATG**C**AAGCTGCGGGGCAGAGAAAT  GACAATTACCAGCAACACTC**C**TTTCAACGCAAATTCCAGCC  TCGAACAGTCGGCTGCAGGA**C**TACTTAATGCAAGCTGCGGG  CGGGCTCTTTACACTTCGAA**C**AGTCGGCTGCAGGACTACTT  AGCTTGATATCGAATTAGGC**C**AACGCTGTCAAGGGATCGTT  TTTGGGATCGGGGCAATCAG**C**ACCGGCTCAGGACGAAACCA  GCGGACAATTACCAGCAACA**C**TCCTTTCAACGCAAATTCCA  CTAAGCTATTTTGTGGGCAC**C**TGCAAGGGCGCCTCGTGATC  GCACCGGCTCAGGACGAAAC**C**ATCAAGATGGGTGCCCTCGC  ATAACAAGCGACCACTACAT**C**TACTGTATATAAGACCTTGC  CCTCGCGGTGTTCGCCGTCG**C**TTGCCTCGCGGCAGTGGCGT  TGGGATCGGGGCAATCAGCA**C**CGGCTCAGGACGAAACCATC  GGGATCGGGGCAATCAGCAC**C**GGCTCAGGACGAAACCATCA  CCGGCTCAGGACGAAACCAT**C**AAGATGGGTGCCCTCGCGGT  ATTATAACAAGCGACCACTA**C**ATCTACTGTATATAAGACCT  TCAACGCAAATTCCAGCCTG**C**TGGACGTTATGCATAATCCA  ATTACCAGCAACACTCCTTT**C**AACGCAAATTCCAGCCTGCT  TCGTTTGGGATCGGGGCAAT**C**AGCACCGGCTCAGGACGAAA  TTGATATCGAATTAGGCCAA**C**GCTGTCAAGGGATCGTTTGG  GCAGGACTACTTAATGCAAG**C**TGCGGGGCAGAGAAATTATA  TGCATAATCCACGTACGTCC**C**ACCCCGCCATGATGGCGCCA  CGGCTGACAATTACCAGCAA**C**ACTCCTTTCAACGCAAATTC  ACAAGCGACCACTACATCTA**C**TGTATATAAGACCTTGCGCC  ACTGTATATAAGACCTTGCG**C**CTCTGCTAAGCTATTTTGTG  GTCAAGGGATCGTTTGGGAT**C**GGGGCAATCAGCACCGGCTC  TATAAGACCTTGCGCCTCTG**C**TAAGCTATTTTGTGGGCACC  TGCTGGACGTTATGCATAAT**C**CACGTACGTCCCACCCCGCC  ATGCATAATCCACGTACGTC**C**CACCCCGCCATGATGGCGCC  GGCGTCGGTTGCGCATGCGG**C**TGACAATTACCAGCAACACT  CTGCTAAGCTATTTTGTGGG**C**ACCTGCAAGGGCGCCTCGTG  GGCCAACGCTGTCAAGGGAT**C**GTTTGGGATCGGGGCAATCA  ATTGGGACAACGAGGACAGT**C**AGTTTAAGACGCAGATCCGC  TGCCGAGCTGGCGGAGCGGC**C**GGCTGTGTTCCAGGCAAAGG  GGGCACGCGGCCCAGGCCTG**C**TGTTGACCTTCTCACATGCA  ACCCCGCAGCCGGCGTGGGC**C**ACCACCGCCTGCTCACCGCC  GCGGCCGGCTGTGTTCCAGG**C**AAAGGTCGAGGTGAGAGAAA  GCCTTGTGGGGAGCGGGCGT**C**AGTGGAAGAAAGTGGTCTGG  ACCGCCGCCAGCGAGCGCGC**C**ATCGCCACCCGCCGCCGCGC  TTTAAGACGCAGATCCGCGG**C**GCCAACCCCGCAGCCGGCGT  GTGGAAGAAAGTGGTCTGGG**C**GGCGTTGGGGGCGCGCCGGG  CTGTGTCGCTTGCACCCCAA**C**CCCGCGTGTGTGGGGTGCGG  CCCCGCCCCGTACTAATTTC**C**ATGCCTAACACACACCCACA  GCCAGCGAGCGCGCCATCGC**C**ACCCGCCGCCGCGCCCAGGC  CCACCACCGCCTGCTCACCG**C**CGCCAGCGAGCGCGCCATCG  GTACTAATTTCCATGCCTAA**C**ACACACCCACAGGATTGGGA  CCGCGTGTGTGGGGTGCGGG**C**ACGCGGCCCAGGCCTGCTGT  AGCGAGCGCGCCATCGCCAC**C**CGCCGCCGCGCCCAGGCCGC  ATGCAACGCTCACCCCCGCC**C**CGTACTAATTTCCATGCCTA  AAGACGCAGATCCGCGGCGC**C**AACCCCGCAGCCGGCGTGGG  AGTCAGTTTAAGACGCAGAT**C**CGCGGCGCCAACCCCGCAGC  AGGCCTGCTGTTGACCTTCT**C**ACATGCAACGCTCACCCCCG  CCCCCGCCCCGTACTAATTT**C**CATGCCTAACACACACCCAC  GCCCCGTACTAATTTCCATG**C**CTAACACACACCCACAGGAT  TCACTGTGTCGCTTGCACCC**C**AACCCCGCGTGTGTGGGGTG  GTTCGGCTCGTGTCGTTTGT**C**CTTTTGGCTCACTGTGTCGC  CTCACTGTGTCGCTTGCACC**C**CAACCCCGCGTGTGTGGGGT  TGGGGTGCGGGCACGCGGCC**C**AGGCCTGCTGTTGACCTTCT  CCCCGTACTAATTTCCATGC**C**TAACACACACCCACAGGATT  CCGCGGCGCCAACCCCGCAG**C**CGGCGTGGGCCACCACCGCC  GCTCACTGTGTCGCTTGCAC**C**CCAACCCCGCGTGTGTGGGG  GATCCCTTGACAGCGTTGGC**C**TAATTCGATATCAAGCTTAT  TCTAGATAAAGTCCGGAGAT**C**ACGAGGCGCCCTTGCAGGTG  CGGTGCTGATTGCCCCGATC**C**CAAACGATCCCTTGACAGCG  AAAAAAATTCCGCATGGCGC**C**ATCATGGCGGGGTGGGACGT  TGCATTAAGTAGTCCTGCAG**C**CGACTGTTCGAAGTGTAAAG  GAGATCACGAGGCGCCCTTG**C**AGGTGCCCACAAAATAGCTT  AGTCCGGAGATCACGAGGCG**C**CCTTGCAGGTGCCCACAAAA  GTCCGGAGATCACGAGGCGC**C**CTTGCAGGTGCCCACAAAAT  TGAGCCGGTGCTGATTGCCC**C**GATCCCAAACGATCCCTTGA  TACAGTAGATGTAGTGGTCG**C**TTGTTATAATTTCTCTGCCC  ATGCGCAACCGACGCCACTG**C**CGCGAGGCAAGCGACGGCGA  CGATCCCTTGACAGCGTTGG**C**CTAATTCGATATCAAGCTTA  TGGGACGTACGTGGATTATG**C**ATAACGTCCAGCAGGCTGGA  TTGCCCCGATCCCAAACGAT**C**CCTTGACAGCGTTGGCCTAA  TCCTGAGCCGGTGCTGATTG**C**CCCGATCCCAAACGATCCCT  GGCGAACACCGCGAGGGCAC**C**CATCTTGATGGTTTCGTCCT  CGTGGATTATGCATAACGTC**C**AGCAGGCTGGAATTTGCGTT  CGGCGAACACCGCGAGGGCA**C**CCATCTTGATGGTTTCGTCC  TTTCTCTGCCCCGCAGCTTG**C**ATTAAGTAGTCCTGCAGCCG  TGCAGGTGCCCACAAAATAG**C**TTAGCAGAGGCGCAAGGTCT  GCATTAAGTAGTCCTGCAGC**C**GACTGTTCGAAGTGTAAAGA  AAAATTCCGCATGGCGCCAT**C**ATGGCGGGGTGGGACGTACG  GCTTAGCAGAGGCGCAAGGT**C**TTATATACAGTAGATGTAGT  GAGCCCGTACATGTAGGGCC**C**CGTTTTTCCAAAAAAATTCC  CGCATGCGCAACCGACGCCA**C**TGCCGCGAGGCAAGCGACGG  GATCCCAAACGATCCCTTGA**C**AGCGTTGGCCTAATTCGATA  ACCTCGACCTTTGCCTGGAA**C**ACAGCCGGCCGCTCCGCCAG  TCTTCCACTGACGCCCGCTC**C**CCACAAGGCCCACCCCCGCT  GCGGGTTGGGGTGCAAGCGA**C**ACAGTGAGCCAAAAGGACAA  CCTGGGCCGCGTGCCCGCAC**C**CCACACACGCGGGTTGGGGT  GCAGGCGGTGGTGGCCCACG**C**CGGCTGCGGGGTTGGCGCCG  GTTGCATGTGAGAAGGTCAA**C**AGCAGGCCTGGGCCGCGTGC  GGTCAACAGCAGGCCTGGGC**C**GCGTGCCCGCACCCCACACA  GGGTGGCGATGGCGCGCTCG**C**TGGCGGCGGTGAGCAGGCGG  TGCTCTACTCTTTCTCTCAC**C**TCGACCTTTGCCTGGAACAC  TGCGTCTTAAACTGACTGTC**C**TCGTTGTCCCAATCCTGTGG  GCTTGCTCTACTCTTTCTCT**C**ACCTCGACCTTTGCCTGGAA  TGGAACACAGCCGGCCGCTC**C**GCCAGCTCGGCACTGACGGC  GCCGCCCAGACCACTTTCTT**C**CACTGACGCCCGCTCCCCAC  GGCGCGCCCCCAACGCCGCC**C**AGACCACTTTCTTCCACTGA  TACTCTTTCTCTCACCTCGA**C**CTTTGCCTGGAACACAGCCG  ATCCCGTCGAATCCACGTCC**C**CGCCCCGGCGCGCCCCCAAC  AACGCCGCCCAGACCACTTT**C**TTCCACTGACGCCCGCTCCC  CTGGAACACAGCCGGCCGCT**C**CGCCAGCTCGGCACTGACGG  TTGCTCTACTCTTTCTCTCA**C**CTCGACCTTTGCCTGGAACA  CACGCGGGTTGGGGTGCAAG**C**GACACAGTGAGCCAAAAGGA  CGGGGCGGGGGTGAGCGTTG**C**ATGTGAGAAGGTCAACAGCA  CACACACGCGGGTTGGGGTG**C**AAGCGACACAGTGAGCCAAA  CCACTTTCTTCCACTGACGC**C**CGCTCCCCACAAGGCCCACC  CCGCTTGCTCTACTCTTTCT**C**TCACCTCGACCTTTGCCTGG  CCGCTCCCCACAAGGCCCAC**C**CCCGCTTGCTCTACTCTTTC  GTGAGCAGGCGGTGGTGGCC**C**ACGCCGGCTGCGGGGTTGGC  ACAAGGCCCACCCCCGCTTG**C**TCTACTCTTTCTCTCACCTC  CGCCCGCTCCCCACAAGGCC**C**ACCCCCGCTTGCTCTACTCT  CGGCGGGTGGCGATGGCGCG**C**TCGCTGGCGGCGGTGAGCAG  CGTCCCCGCCCCGGCGCGCC**C**CCAACGCCGCCCAGACCACT  GAACACAGCCGGCCGCTCCG**C**CAGCTCGGCACTGACGGCGG  AAACTGACTGTCCTCGTTGT**C**CCAATCCTGTGGGTGTGTGT  CTGGGCCGCGTGCCCGCACC**C**CACACACGCGGGTTGGGGTG  GCGGGGTTGGCGCCGCGGAT**C**TGCGTCTTAAACTGACTGTC  ACCACTTTCTTCCACTGACG**C**CCGCTCCCCACAAGGCCCAC  AACACAGCCGGCCGCTCCGC**C**AGCTCGGCACTGACGGCGGC  TCCCCGCCCCGGCGCGCCCC**C**AACGCCGCCCAGACCACTTT  AAAAGGACAAACGACACGAG**C**CGAACCTATCCCGTCGAATC  AGACCACTTTCTTCCACTGA**C**GCCCGCTCCCCACAAGGCCC  CCACTGACGCCCGCTCCCCA**C**AAGGCCCACCCCCGCTTGCT  CAGCAGGCCTGGGCCGCGTG**C**CCGCACCCCACACACGCGGG  CCACCCCCGCTTGCTCTACT**C**TTTCTCTCACCTCGACCTTT  CAGCAGGCGACACGGCTAGG**C**CTGCTGCGTTCCCAGCCCCC  AGGCCTGCTGCGTTCCCAGC**C**CCCCGCACTTCCCAGGAGCC  CCTGCTGCGTTCCCAGCCCC**C**CGCACTTCCCAGGAGCCGTA  TTCCCAGCCCCCCGCACTTC**C**CAGGAGCCGTACTGCATTCT  CCCCCCGCACTTCCCAGGAG**C**CGTACTGCATTCTGGGAGTA  AGCAGGCGACACGGCTAGGC**C**TGCTGCGTTCCCAGCCCCCC  GAGTATCACTGAGTGTTTCA**C**GTTCGTTCGACAGCTCTTGT  AGTGCAGGGTCGCCAACTGC**C**CGCTCCCAGAGGAGGCTGGG  CGCCTCCCGCAGGCGCACCC**C**ACTCCATTTACCTGTCCGGG  CCCGCCTCCCGCAGGCGCAC**C**CCACTCCATTTACCTGTCCG  CTTTCCATCGACACCTCCGC**C**CTCCCTCCGCAGTTCTCTCC  TCCCGCAGGCGCACCCCACT**C**CATTTACCTGTCCGGGCACC  CAAATGCCGTATAGGAAGCC**C**AAGTCACCCCTGCACTGCCT  CCCAGGAGCCGTACTGCATT**C**TGGGAGTAGTAGTTCCGGTT  GCCGTATAGGAAGCCCAAGT**C**ACCCCTGCACTGCCTCCTCG  GCCTCCTCGCTCCACCCCAG**C**ACCTTTCCATCGACACCTCC  GCCCCGTCTACGCGTGGCCT**C**TTGGGAGTGGGAGCGCTCCT  TTTACCTGTCCGGGCACCCT**C**TGAGGGCGCGCGGGCTCGAG  GCGCACCCCACTCCATTTAC**C**TGTCCGGGCACCCTCTGAGG  GGTCGCCAACTGCCCGCTCC**C**AGAGGAGGCTGGGACCGGAC  GCATTCTGGGAGTAGTAGTT**C**CGGTTGGAGTATCACTGAGT  TCCAATGGCAGCAGGCGACA**C**GGCTAGGCCTGCTGCGTTCC  CGCCCTCCCTCCGCAGTTCT**C**TCCCCGCCTCCCGCAGGCGC  CGCTCCACCCCAGCACCTTT**C**CATCGACACCTCCGCCCTCC  TCCTCGCTCCACCCCAGCAC**C**TTTCCATCGACACCTCCGCC  GGGCACCCTCTGAGGGCGCG**C**GGGCTCGAGACGGAGGGACG  GGACCGGACGCCGCTGACTT**C**CGCATGAAGCGCCCCGTCTA  AGTTCTCTCCCCGCCTCCCG**C**AGGCGCACCCCACTCCATTT  CTGCCCGCTCCCAGAGGAGG**C**TGGGACCGGACGCCGCTGAC  ACGGCTAGGCCTGCTGCGTT**C**CCAGCCCCCCGCACTTCCCA  CCTCTGAGGGCGCGCGGGCT**C**GAGACGGAGGGACGCGGGTC  AGTCACCCCTGCACTGCCTC**C**TCGCTCCACCCCAGCACCTT  GTCAAATGCCGTATAGGAAG**C**CCAAGTCACCCCTGCACTGC  CAGTGCAGGGTCGCCAACTG**C**CCGCTCCCAGAGGAGGCTGG  CTCTTGGGAGTGGGAGCGCT**C**CTCCAATGGCAGCAGGCGAC  CCTCCCTCCGCAGTTCTCTC**C**CCGCCTCCCGCAGGCGCACC  ACACCTCCGCCCTCCCTCCG**C**AGTTCTCTCCCCGCCTCCCG  ACAGGCCGCACCCAGCTTTT**C**TTCCGTTGCCCCAGTACGGA  CTGATCCTCACAGGCCGCAC**C**CAGCTTTTCTTCCGTTGCCC  GACAGCTTATCATCGATAAG**C**TGATCCTCACAGGCCGCACC  GTCTAAGAAACCATTATTAT**C**ATGACATTAACCTATAAAAA  CGAAAAGTGCCACCTGACGT**C**TAAGAAACCATTATTATCAT  GATAAGCTGATCCTCACAGG**C**CGCACCCAGCTTTTCTTCCG  CCCAGCTTTTCTTCCGTTGC**C**CCAGTACGGATACATATTTG  TCACGAGGCCCTTTCGTCTT**C**AAGAATTCTCATGTTTGACA  GGGCGAAAACTCTCAAGGAT**C**TTACCGCTGTTGAGATCCAG  CGGCGACCGAGTTGCTCTTG**C**CCGGCGTCAACACGGGATAA  AAAAAAGGGAATAAGGGCGA**C**ACGGAAATGTTGAATACTTG  CACGGGATAATACCGCGCCA**C**ATAGCAGAACTTTAAAAGTG  CGTTCTTCGGGGCGAAAACT**C**TCAAGGATCTTACCGCTGTT  GGCGTCAACACGGGATAATA**C**CGCGCCACATAGCAGAACTT  AACAGGAAGGCAAAATGCCG**C**AAAAAAGGGAATAAGGGCGA  AGCAGAACTTTAAAAGTGCT**C**ATCATTGGAAAACGTTCTTC  GATGTAACCCACTCGTGCAC**C**CAACTGATCTTCAGCATCTT  GATAATACCGCGCCACATAG**C**AGAACTTTAAAAGTGCTCAT  CGACACGGAAATGTTGAATA**C**TTGAGAATAGTGTATGCGGC  TGTATGCGGCGACCGAGTTG**C**TCTTGCCCGGCGTCAACACG  CGCAAAAAAGGGAATAAGGG**C**GACACGGAAATGTTGAATAC  AACGTTCTTCGGGGCGAAAA**C**TCTCAAGGATCTTACCGCTG  CATCATTGGAAAACGTTCTT**C**GGGGCGAAAACTCTCAAGGA  GAGAATAGTGTATGCGGCGA**C**CGAGTTGCTCTTGCCCGGCG  AGGCGGAAGACCAGGAGCCC**C**GGGCGGCTCAGGATCAGGGC  AGACCAGGAGCCCCGGGCGG**C**TCAGGATCAGGGCCAAGACA  AGCCCCGGGCGGCTCAGGAT**C**AGGGCCAAGACATAGAGATG  CAAAGAAGAGGGGGTGATAA**C**CATGGACGAGGACGGGGAAG  GTGATAACCATGGACGAGGA**C**GGGGAAGAGGACGAGGACGA  AGGAGGCGGAAGACCAGGAG**C**CCCGGGCGGCTCAGGATCAG  GTGTCCGGAGACCCCAAAAA**C**GTTCCGGCGGCAGTGGACCT  AGACCCCAAAAACGTTCCGG**C**GGCAGTGGACCTCAAAGAAG  CAAGACATAGAGATGGTGTC**C**GGAGACCCCAAAAACGTTCC  GAGATGGTGTCCGGAGACCC**C**AAAAACGTTCCGGCGGCAGT  ATAGAGATGGTGTCCGGAGA**C**CCCAAAAACGTTCCGGCGGC  ATATGGAGGTAGTAAGACCT**C**CCTTTACAACCTAAGGCGAG  TCTCCCCTTTGGAATGGCCC**C**TGGACCCGGCCCACAACCTG  TATTCCACAATGTCGTCTTA**C**ACCATTGAGTCGTCTCCCCT  CCCCTGGACCCGGCCCACAA**C**CTGGCCCGACGTAGAAAGGA  ACTTGGGTCGCCGGTGTGTT**C**GTATATGGAGGTAGTAAGAC  AATGGCCCCTGGACCCGGCC**C**ACAACCTGGCCCGACGTAGA  TGGCCCCTGGACCCGGCCCA**C**AACCTGGCCCGACGTAGAAA  TTACAACCTAAGGCGAGGAA**C**TGCCCTTGCTATTCCACAAT  AAGGCGAGGAACTGCCCTTG**C**TATTCCACAATGTCGTCTTA  CCTCCCTTTACAACCTAAGG**C**GAGGAACTGCCCTTGCTATT  ATGGAGGTAGTAAGACCTCC**C**TTTACAACCTAAGGCGAGGA  CTTTGGAATGGCCCCTGGAC**C**CGGCCCACAACCTGGCCCGA  AGTAAGACCTCCCTTTACAA**C**CTAAGGCGAGGAACTGCCCT  CAACCTAAGGCGAGGAACTG**C**CCTTGCTATTCCACAATGTC  AAGGACTACCGACGAAGGAA**C**TTGGGTCGCCGGTGTGTTCG  TACACCATTGAGTCGTCTCC**C**CTTTGGAATGGCCCCTGGAC  GACCCGGCCCACAACCTGGC**C**CGACGTAGAAAGGACTACCG  AGCTCCGCGCTCAACACCTT**C**TCGCGTTGGAAAACATTAGC  GTGAGCAATCAGACATGCGA**C**GGCTTTAGCCTGGCCTCCTT  CCCAACATGGGGCAATTGGG**C**ATACCCATGTTGTCACGTCA  CACCATTTTCGTCCTCCCAA**C**ATGGGGCAATTGGGCATACC  AGACATGCGACGGCTTTAGC**C**TGGCCTCCTTAAATTCACCT  CACCTTCTCGCGTTGGAAAA**C**ATTAGCGACATTTACCTGGT  ATGGGGCAATTGGGCATACC**C**ATGTTGTCACGTCACTCAGC  CCGCGCTCAACACCTTCTCG**C**GTTGGAAAACATTAGCGACA  ACCCATGTTGTCACGTCACT**C**AGCTCCGCGCTCAACACCTT  ACGTCACTCAGCTCCGCGCT**C**AACACCTTCTCGCGTTGGAA  CTCAGCTCCGCGCTCAACAC**C**TTCTCGCGTTGGAAAACATT  GCCTCCTTAAATTCACCTAA**C**GTGCTGTTGGTGTATTTCTG  TTTCTGGCCATCTGTCTTGT**C**ACCATTTTCGTCCTCCCAAC  AGCGACATTTACCTGGTGAG**C**AATCAGACATGCGACGGCTT  CCTGGCCTCCTTAAATTCAC**C**TAACGTGCTGTTGGTGTATT  CCTTAAATTCACCTAACGTG**C**TGTTGGTGTATTTCTGGCCA  CGACGGCTTTAGCCTGGCCT**C**CTTAAATTCACCTAACGTGC  ACGTTTTCCAATGATGAGCA**C**TTTTAAAGTTCTGCTATGTG  GGTCGCCGATTTCCGTGTCG**C**CCTTATTCCCTTTTTTGCGG  ATCCTTGAGAGTTTTCGCCC**C**GAAGAACGTTTTCCAATGAT  TAAAGTTCTGCTATGTGGCG**C**GGTATTATCCCGTGTTGACG  GGTATTATCCCGTGTTGACG**C**CGGGCAAGAGCAACTCGGTC  ATCGAACTGGATCTCAACAG**C**GGTAAGATCCTTGAGAGTTT  GCTGGTGAAAGTAAAAGATG**C**TGAAGATCAGTTGGGTGCAC  CCTTCCTGTTTTTGCTCACC**C**AGAAACGCTGGTGAAAGTAA  ATTTTGCCTTCCTGTTTTTG**C**TCACCCAGAAACGCTGGTGA  TTCGCCCCGAAGAACGTTTT**C**CAATGATGAGCACTTTTAAA  GACGCCGGGCAAGAGCAACT**C**GGTCGCCGATTTCCGTGTCG  GATCCTTGAGAGTTTTCGCC**C**CGAAGAACGTTTTCCAATGA  CCTTTTTTGCGGCATTTTGC**C**TTCCTGTTTTTGCTCACCCA  CTGAAGATCAGTTGGGTGCA**C**GAGTGGGTTACATCGAACTG  AAGTAAAAGATGCTGAAGAT**C**AGTTGGGTGCACGAGTGGGT  CCGCTCATGAGACAATAACC**C**CTACTGGGGCAACGGAAGAA  TGTCATGATAATAATGGTTT**C**TTAGACGTCAGGTGGCACTT  GTTTCTTAGACGTCAGGTGG**C**ACTTTTCGGGGAAATGTGCG  TTTATTTTTCTAAATACATT**C**AAATATGTATCCGCTCATGA  ACCCCTATTTGTTTATTTTT**C**TAAATACATTCAAATATGTA  ATCGATGATAAGCTGTCAAA**C**ATGAGAATTCTTGAAGACGA  ATCAGCTTATCGATGATAAG**C**TGTCAAACATGAGAATTCTT  TTTGTTTATTTTTCTAAATA**C**ATTCAAATATGTATCCGCTC  GGGAAATGTGCGCGGAACCC**C**TATTTGTTTATTTTTCTAAA  CATTCAAATATGTATCCGCT**C**ATGAGACAATAACCCCTACT  ATATGTATCCGCTCATGAGA**C**AATAACCCCTACTGGGGCAA  AATTCTTGAAGACGAAAGGG**C**CTCGTGATACGCCTATTTTT  GAAGAAAAGCTGGGTGCGGC**C**TGTGAGGATCAGCTTATCGA  TCGGGGAAATGTGCGCGGAA**C**CCCTATTTGTTTATTTTTCT  CTCTTCTTTGAGGTCCACTG**C**CGCCGGACGTTTTTGGGGTC  GGGCTCCTGGTCTTCCGCCT**C**CTCGTCCTCGTCCTCTTCCC  CGCCTCCTCGTCCTCGTCCT**C**TTCCCCGTCCTCGTCCATGG  TGGTCTTCCGCCTCCTCGTC**C**TCGTCCTCTTCCCCGTCCTC  CATGGTTATCACCCCCTCTT**C**TTTGAGGTCCACTGCCGCCG  CCCCTCTTCTTTGAGGTCCA**C**TGCCGCCGGACGTTTTTGGG  CCTCGTCCATGGTTATCACC**C**CCTCTTCTTTGAGGTCCACT  TTCTTTGAGGTCCACTGCCG**C**CGGACGTTTTTGGGGTCTCC  CTGGTCTTCCGCCTCCTCGT**C**CTCGTCCTCTTCCCCGTCCT  CTCCTGGTCTTCCGCCTCCT**C**GTCCTCGTCCTCTTCCCCGT  CGCCCGGGGCTCCTGGTCTT**C**CGCCTCCTCGTCCTCGTCCT  CCTCGTCCTCGTCCTCTTCC**C**CGTCCTCGTCCATGGTTATC  TCCTCGTCCTCTTCCCCGTC**C**TCGTCCATGGTTATCACCCC  TCCTGAGCCGCCCGGGGCTC**C**TGGTCTTCCGCCTCCTCGTC  GTTTTTGGGGTCTCCGGACA**C**CATCTCTATGTCTTGGCCCT  AGGCCAGGCTAAAGCCGTCG**C**ATGTCTGATTGCTCACCAGG  ATGTCGCTAATGTTTTCCAA**C**GCGAGAAGGTGTTGAGCGCG  GTCGCATGTCTGATTGCTCA**C**CAGGTAAATGTCGCTAATGT  TAAATGTCGCTAATGTTTTC**C**AACGCGAGAAGGTGTTGAGC  GGACGAAAATGGTGACAAGA**C**AGATGGCCAGAAATACACCA  CGTGACAACATGGGTATGCC**C**AATTGCCCCATGTTGGGAGG  TTAAGGAGGCCAGGCTAAAG**C**GTCGCATGTCTGATTGCTC  ACATGGGTATGCCCAATTGC**C**CCCATGTTGGGAGGACGAAAA  CCCCTGCGCCCGCCCCCGCC**C**CCCTCCCGCTCCCATTCTCT  GGGGAGTTTTGTTAACCGGA**C**TACAGGGGCAACTCCGCCGC  TCAGGGATGGCTTTTGGGCT**C**TGCCCCTCGCTGCTCCCGGC  CCCCCTGCGCCCGCCCCCGC**C**CCCCTCCCGCTCCCATTCTC  GGGCTCTGCCCCTCGCTGCT**C**CCGGCGTTTGGCGCCCGCGC  TTGCTTAACAACAGTAACGT**C**ACACGGACTACAGGGGAGTT  CGCGCCCCCTCCCCCTGCGC**C**CGCCCCCGCCCCCCTCCCGC  TCGCTGCTCCCGGCGTTTGG**C**GCCCGCGCCCCCTCCCCCTG  GGGATGGCTTTTGGGCTCTG**C**CCCTCGCTGCTCCCGGCGTT  GCTGCTCCCGGCGTTTGGCG**C**CCGCGCCCCCTCCCCCTGCG  CCGCGCCCCCTCCCCCTGCG**C**CCGCCCCCGCCCCCCTCCCG  CAACTCCGCCGCAGGGCAGG**C**GCGGCGCCTCAGGGATGGCT  CGGCGTTTGGCGCCCGCGCC**C**CCTCCCCCTGCGCCCGCCCC  CGCTCCCATTCTCTGCCGGG**C**TTTGATCTTTGCTTAACAAC  GCGCCCCCTCCCCCTGCGCC**C**GCCCCCGCCCCCCTCCCGCT  CCCCTCCCCCTGCGCCCGCC**C**CCGCCCCCCTCCCGCTCCCA  CCCCCTCCCCCTGCGCCCGC**C**CCCGCCCCCCTCCCGCTCCC  GGGGCAACTCCGCCGCAGGG**C**AGGCGCGGCGCCTCAGGGAT  GGACTACAGGGGCAACTCCG**C**CGCAGGGCAGGCGCGGCGCC  TTGGGCTCTGCCCCTCGCTG**C**TCCCGGCGTTTGGCGCCCGC  ATTCTCTGCCGGGCTTTGAT**C**TTTGCTTAACAACAGTAACG  ACCGGACTACAGGGGCAACT**C**CGCCGCAGGGCAGGCGCGGC  CTGCGCCCGCCCCCGCCCCC**C**TCCCGCTCCCATTCTCTGCC  CAGTAACGTCACACGGACTA**C**AGGGGAGTTTTGTTAACCGG  GCCCCGCTCGGTTCGTTACT**C**TGTAGAGGCTGGCGACCAGC  CAAAAGAGAAAGTTCCCGAG**C**TTTACAACTCTCCGGGAAGC  CTTTACAACTCTCCGGGAAG**C**GTAGGGAACGTTTCCTCTCG  AAGCGTAGGGAACGTTTCCT**C**TCGGAAAGCTCTTCGCCGCG  AGCTCTTCGCCGCGATTTTC**C**CAGGGCTGCACTTGAATGAA  TCTCGGAAAGCTCTTCGCCG**C**GATTTTCCCAGGGCTGCACT  CCAGCAAAAGAGAAAGTTCC**C**GAGCTTTACAACTCTCCGGG  ACGTTTCCTCTCGGAAAGCT**C**TTCGCCGCGATTTTCCCAGG  TCCTCTCGGAAAGCTCTTCG**C**CGCGATTTTCCCAGGGCTGC  CGCTTGCCCTCCCCGCCACG**C**CCCGCTCGGTTCGTTACTCT  TCTCCGGGAAGCGTAGGGAA**C**GTTTCCTCTCGGAAAGCTCT  GCCCTCCCCGCCACGCCCCG**C**TCGGTTCGTTACTCTGTAGA  GGAAGCGTAGGGAACGTTTC**C**TCTCGGAAAGCTCTTCGCCG  GCCACGCTTGCCCTCCCCGC**C**ACGCCCCGCTCGGTTCGTTA  CTTGCCCTCCCCGCCACGCC**C**CGCTCGGTTCGTTACTCTGT  CGCGGTCGCGCTTTCTCTGC**C**CTCCGCCGGGTGGACCTGGA  TTCTTTCTGCCCTCTGCAGA**C**ATCCCCGATTGAAAGAACCA  TGCCCCCGCCACAACCCACC**C**CGCTTTCGTAGTTTTCATTT  GCCACAACCCACCCCGCTTT**C**GTAGTTTTCATTTAGAAAAT  CAACTGCCCCCGCCACAACC**C**ACCCCGCTTTCGTAGTTTTC  CTGCCCTCTGCAGACATCCC**C**GATTGAAAGAACCAGAGAGG  GAGGAGGGCGGGATGTGCCA**C**ACATCTTTGACCTCAGGTTT  GAGCTTTTAAAAATGTCCTG**C**GGGAAGGGAAAGGCCACATC  GATCATCAGTCACCGAAGGT**C**CTACAGGGCCACAACTGCCC  AGCGGTCGGCGCGCCTGGAG**C**AGCCAGGCGGGCAGTGGACT  TCCGCCGGGTGGACCTGGAG**C**GCTTGAGCGGTCGGCGCGCC  CACAACTGCCCCCGCCACAA**C**CCACCCCGCTTTCGTAGTTT  ATTGGGAGCAACCACTGAGA**C**TCATTATATAACACTCGTTT  ACATCTTCACGCCTTCGCGC**C**TGGCATTGGGAGCAACCACT  GTGTGGGAGAGCGGTGGCGG**C**GGGTACATGCACGTGAAGCC  TTGAAAGAACCAGAGAGGCT**C**TGAGAAACCTCTGGAAACTT  ACCTGGAGCGCTTGAGCGGT**C**GGCGCGCCTGGAGCAGCCAG  CCTCTGGAAACTTAGATCAT**C**AGTCACCGAAGGTCCTACAG  CTTCACGCCTTCGCGCCTGG**C**ATTGGGAGCAACCACTGAGA  TCTGCCCTCCGCCGGGTGGA**C**CTGGAGCGCTTGAGCGGTCG  GAGAGGAGGGCGGGATGTGC**C**ACACATCTTTGACCTCAGGT  CGCGCTTTCTCTGCCCTCCG**C**CGGGTGGACCTGGAGCGCTT  ATGTGCCACACATCTTTGAC**C**TCAGGTTTCTAACGCCTGTT  ACATCTTTGACCTCAGGTTT**C**TAACGCCTGTTTTCTTTCTG  TGTCCATAAGTATTTCAATG**C**CGGTAGGGACGGCAAGAGAG  AGGGCCACAACTGCCCCCGC**C**ACAACCCACCCCGCTTTCGT  TTCTGCCCTCTGCAGACATC**C**CCGATTGAAAGAACCAGAGA  CCAGGCGGGCAGTGGACTAG**C**TGCTGGACCAGGGAGGTGTG  ATCATCAGTCACCGAAGGTC**C**TACAGGGCCACAACTGCCCC  GCCACATCTTCACGCCTTCG**C**GCCTGGCATTGGGAGCAACC  CAGGGCCACAACTGCCCCCG**C**CACAACCCACCCCGCTTTCG  CTTCTTGCAACCCTGCGGGC**C**GCGCGGTCGCGCTTTCTCTG  GGTCGGCGCGCCTGGAGCAG**C**CAGGCGGGCAGTGGACTAGC  CTACAGGGCCACAACTGCCC**C**CGCCACAACCCACCCCGCTT  ATAGAGCTTTTAAAAATGTC**C**TGCGGGAAGGGAAAGGCCAC  CATTATATAACACTCGTTTT**C**TTCTTGCAACCCTGCGGGCC  GCCATTGCGAGAACTTTGTC**C**ATAAGTATTTCAATGCCGGT  GCGCGCCTGGAGCAGCCAGG**C**GGGCAGTGGACTAGCTGCTG  GCAGCCAGGCGGGCAGTGGA**C**TAGCTGCTGGACCAGGGAGG  ATCAGTCACCGAAGGTCCTA**C**AGGGCCACAACTGCCCCCGC  TTCGCGCCTGGCATTGGGAG**C**AACCACTGAGACTCATTATA  AGCCATTGCGAGAACTTTGT**C**CATAAGTATTTCAATGCCGG  GAGAGCGGTGGCGGCGGGTA**C**ATGCACGTGAAGCCATTGCG  CTCTGAGAAACCTCTGGAAA**C**TTAGATCATCAGTCACCGAA  GAAGGGAAAGGCCACATCTT**C**ACGCCTTCGCGCCTGGCATT  GAGACTCATTATATAACACT**C**GTTTTCTTCTTGCAACCCTG  GCGCGGTCGCGCTTTCTCTG**C**CCTCCGCCGGGTGGACCTGG  TACAGGGCCACAACTGCCCC**C**GCCACAACCCACCCCGCTTT  CACCGAAGGTCCTACAGGGC**C**ACAACTGCCCCCGCCACAAC  GAAAGTGCGCTTCTGAGACT**C**TTGACAGCCATTTCGTTCCC  TTATCACTCCACCGCGCGCA**C**TTTCCGCAGGAGCGATGTGA  CAGCTTGGAGCTGGCTAGGC**C**TTGTTTTGGAGGGGATGGGT  GGAGCGATGTGATCCGTTAT**C**ATAACTGCGGACCTGGGGTT  AGAAGGCGACTCTGGGTCAC**C**TCTCCAGCTTGGAGCTGGCT  CTCTTGACAGCCATTTCGTT**C**CCTTCCAAGCCAGATGGAGA  GTTTTCTTCCAGAGGCTTAA**C**TGGCAGCTGGAACGAGTTCC  CAGGATGGTATAGGGAGTGG**C**CCGTAGTATTTTTCCAGTGA  TTTCCAGTGACGATGTCTCT**C**CATTGTTTTCTTCCAGAGGC  CTGGCAGCTGGAACGAGTTC**C**TCCAACAAGAATTTAGACGC  CCAGCTTGGAGCTGGCTAGG**C**CTTGTTTTGGAGGGGATGGG  ACTCGAGACGAAAGGCAGGA**C**ATGACAGAAGGCGACTCTGG  TAGACGCTAGGTCCAATTAT**C**ACTCCACCGCGCGCACTTTC  CAGAAGGCGACTCTGGGTCA**C**CTCTCCAGCTTGGAGCTGGC  ACTCTGGGTCACCTCTCCAG**C**TTGGAGCTGGCTAGGCCTTG  TCCACCGCGCGCACTTTCCG**C**AGGAGCGATGTGATCCGTTA  TAACTGCGGACCTGGGGTTC**C**ACGTGGAAGACGATTGGGAT  GATGTGATCCGTTATCATAA**C**TGCGGACCTGGGGTTCCACG  TCAGGGTTACCCGGAGGAAC**C**ACGGGGAAAGTGCGCTTCTG  TGGGTAGATGAAAAGTGAGT**C**AGGGTTACCCGGAGGAACCA  GTGTTGAAAGGCCACGACTT**CC**CTCAGTTTCTCCATCTGGG  GGCAGCTGGAACGAGTTCCT**C**CAACAAGAATTTAGACGCTA  CTGAGTGGGGTTAGTGGACT**C**GAGACGAAAGGCAGGACATG  GGAACCACGGGGAAAGTGCG**C**TTCTGAGACTCTTGACAGCC  ACTGGCAGCTGGAACGAGTT**C**CTCCAACAAGAATTTAGACG  AGTGGCCCGTAGTATTTTTC**C**AGTGACGATGTCTCTCCATT  TCCGTTATCATAACTGCGGA**C**CTGGGGTTCCACGTGGAAGA  TCTTGACAGCCATTTCGTTC**C**CTTCCAAGCCAGATGGAGAC  CGCTAGGTCCAATTATCACT**C**CACCGCGCGCACTTTCCGCA  TGCGCTTCTGAGACTCTTGA**C**AGCCATTTCGTTCCCTTCCA  GCGGTGGGGGTGGGAGCAGA**C**AGAGTCTGAGTGGGGTTAGT  CTTCCAAGCCAGATGGAGAC**C**CAAGAGTGTTGAAAGGCCAC  TCCAGAGGCTTAACTGGCAG**C**TGGAACGAGTTCCTCCAACA  CACTCCACCGCGCGCACTTT**C**CGCAGGAGCGATGTGATCCG  GGACATGACAGAAGGCGACT**C**TGGGTCACCTCTCCAGCTTG  ACCCAAGAGTGTTGAAAGGC**C**ACGACTTCCCTCAGTTTCTC  CAGCAGCTCCGCCACGCGGG**C**GCTGCCCATCATCATGACCT  GTGCAGCACCACCAGCGTGT**C**CAGGAAGCCCTCCCGGGCAG  ACCAGCGTGTCCAGGAAGCC**C**TCCCGGGCAGCATCATGCAC  TTGGGCTCCGCGCCGTGGAG**C**AGCAGCAGCTCCGCCACGCG  ACGTCCAGCCGCGCCCCGGC**C**CGGTGCAGCACCACCAGCGT  TGCCCATCATCATGACCTGC**C**AGAGAGAGCAAAGTGGTCAG  GGCTGTGCGCAGGTACCCTG**C**AACGTCGCGGTGGCCCCGCT  CAGGTATGGGAGATGCCGGC**C**GGGGCAAGGCAGGTGGAGCC  CTGGGGAACCTGGCGTCAGT**C**CCCCGTGGCTGTGCGCAGGT  ATTGGGTAAGAAAATAAAGT**C**GTTGTGGGCGGCTGGGGAAC  GCGCTGCCCATCATCATGAC**C**TGCCAGAGAGAGCAAAGTGG  CCCTGCAACGTCGCGGTGGC**C**CCGCTCCTCGGCCAAGTCCA  GCATCGCGCACGTCCAGCCG**C**GCCCCGGCCCGGTGCAGCAC  CTGCGCAGTTGGGCTCCGCG**C**CGTGGAGCAGCAGCAGCTCC  CGGGGCAAGGCAGGTGGAGC**C**ATTTAAAGGACAGGCTTGCA  TGGGCGGCTGGGGAACCTGG**C**GTCAGTCCCCCGTGGCTGTG  TGTGCGCAGGTACCCTGCAA**C**GTCGCGGTGGCCCCGCTCCT  GGAGCCATTTAAAGGACAGG**C**TTGCAGGCTTACAGGCTTTC  CACCAGCGTGTCCAGGAAGC**C**CTCCCGGGCAGCATCATGCA  CCGGGGCAAGGCAGGTGGAG**C**CATTTAAAGGACAGGCTTGC  GCAGGGTCTGCGCAGTTGGG**C**TCCGCGCCGTGGAGCAGCAG  CGCGCACGTCCAGCCGCGCC**C**CGGCCCGGTGCAGCACCACC  AGCCGCGCCCCGGCCCGGTG**C**AGCACCACCAGCGTGTCCAG  CGCCCCGGCCCGGTGCAGCA**C**CACCAGCGTGTCCAGGAAGC  GCCCCGGCCCGGTGCAGCAC**C**ACCAGCGTGTCCAGGAAGCC  ATCGCGCACGTCCAGCCGCG**C**CCCGGCCCGGTGCAGCACCA  GCGTGTCCAGGAAGCCCTCC**C**GGGCAGCATCATGCACCGGT  CGCCGCTCCCCGTTGGCAGC**C**TTGGTCGAATTAGGTGGGTG  CGTTGTGGGCGGCTGGGGAA**C**CTGGCGTCAGTCCCCCGTGG  AGGACAGGCTTGCAGGCTTA**C**AGGCTTTCCGCCGCTCCCCG  GTCAGTCCCCCGTGGCTGTG**C**GCAGGTACCCTGCAACGTCG  CCATTTAAAGGACAGGCTTGCAGGCTTACAGGCTTTCCGCC  ATGGGAGATGCCGGCCGGGG**C**AAGGCAGGTGGAGCCATTTA  GCAGCTCCGCCACGCGGGCG**C**TGCCCATCATCATGACCTGC  CCGGAGGCGCGGGACGCCGC**C**TCCACGTGAGCCGCGCTCCT  CGAGTGGGACGTTAACTGGC**C**AATAGTTTGCAACTGCCGGT  CACGCGCAGCCTACTCAGCG**C**TGCTCCGGAGGCGCGGGACG  CATTTTCTGCAGTCAGCGTT**C**AAAAAAAGTACGAAAACCAT  CCTATGCCATTTTCTGCAGT**C**AGCGTTCAAAAAAAGTACGA  GGAGGCGCGGGACGCCGCCT**C**CACGTGAGCCGCGCTCCTCC  CAACTGCCGGTCAACACGCG**C**AGCCTACTCAGCGCTGCTCC  TGCACACACACACACACACA**C**ACACACTTTCCTCCCTCTTC  TCACGGCCGTGATCGTTTCT**C**CGAAACAGTTAGATCGCACA  GGCGCGGGACGCCGCCTCCA**C**GTGAGCCGCGCTCCTCCCAG  GCAGCCTACTCAGCGCTGCT**C**CGGAGGCGCGGGACGCCGCC  GAGTCCTAACCGGGAACCTT**C**TGGTTGACCGAGCACACACA  AATTTATCCAAACACTTAAG**C**AAGGAGAGGATCCTAAAATC  TTTCCTCCCTCTTCCCCCAC**C**TCCCTATGCCATTTTCTGCA  CTCAGAGCTACGACTAGTGA**C**TATCCGCTCACATGGGGCAT  GGACGGCGAATTTATCCAAA**C**ACTTAAGCAAGGAGAGGATC  AAAAACGAAGGCGGAAGGGG**C**GAGAAGAACGGGGTGGAGTT  GGCCCTGGCTGGGGTTGAGA**C**TGCAGGAGTAACTTCGACTG  CAAGGAGAGGATCCTAAAAT**C**GAAAAACGAAGGCGGAAGGG  GTCAACACGCGCAGCCTACT**C**AGCGCTGCTCCGGAGGCGCG  CACGTGAGCCGCGCTCCTCC**C**AGTGCCCGGGACGGTGTCGG  ACTGAACCGATTACGGACGG**C**GAATTTATCCAAACACTTAA  CGGTCAACACGCGCAGCCTA**C**TCAGCGCTGCTCCGGAGGCG  ACACACACACACACACTTTC**C**TCCCTCTTCCCCCACCTCCC  GGGCTCTAGTCGTGTGTCAG**C**ACACACACACACACACACAC  TAACTTCTCGGACTAACCTA**C**GTGTTGTTCTTCTCAGGGAC  TGCTCCGGAGGCGCGGGACG**C**CGCCTCCACGTGAGCCGCGC  CACACACACACACACACTTT**C**CTCCCTCTTCCCCCACCTCC  GGGTGGCGGAGCGAGTGGGA**C**GTTAACTGGCCAATAGTTTG  CTTTCCTCCCTCTTCCCCCA**C**CTCCCTATGCCATTTTCTGC  GAGACTGCAGGAGTAACTTC**C**GGGCTCGATAACTACGGCGA  TGGCCAATAGTTTGCAACTG**C**CGGTCAACACGCGCAGCCTA  GAAAACCATCGGGTAATTTC**C**AACTGAACCGATTACGGACG  TCGGCTTGTGTATAAGAGCA**C**ACACACACACACACACACAC  CTGCCAATCTGAAGCACACA**C**ACACACACACACACACACTT  TTCTCGGTAAAAGCTCTGTC**C**GGCTTCGTCAAGGCTACGCC  GGTGTCGGGGGCGGGCTGCA**C**GGCGAGAAGGGCAAAATAAA  TTACGGACGGCGAATTTATC**C**AAACACTTAAGCAAGGAGAG  CGAGAAGGGCAAAATAAAAC**C**AGCCCGAGAGGCCCTGGCTG  CCCGGGAGATGGAGCGGCGC**C**GCCGCCGCCACCACGTCTGC  GCGCCCCTTTTCTTCCTCCG**C**CCAGGGTCCGCGCGGGCCCG  CAGGATCCGTGCGGACCCAT**C**CCCGTACACGCGCCCCTTTT  CCACGTCTGCGGTCCTGAGC**C**CCCAGCGGGCGAGAGAGCGG  CTGCGCGTCCGCTCGGGTTG**C**TGTGCGCCGGAGCATGCTCG  CGGGTGTAGACGTGCAGGAT**C**CGTGCGGACCCATCCCCGTA  GGGCCCGGGAGATGGAGCGG**C**GCCGCCGCCGCCACCACGTC  GCTGGCGCGCGGCGGCAGGG**C**CTCGAAGCTGAAGCCCTCGC  GAGCGGCGCCGCCGCCGCCA**C**CACGTCTGCGGTCCTGAGCC  AGAGCGGACTGGTCCAAGAG**C**TGCGAGGCCAGTGCTGCGCG  GCCGCCACCACGTCTGCGGT**C**CTGAGCCCCCAGCGGGCGAG  TGGAGCGGCGCCGCCGCCGC**C**ACCACGTCTGCGGTCCTGAG  TCCGCTCGGGTTGCTGTGCG**C**CGGAGCATGCTCGTGGGCCT  TCCTCCGCCCAGGGTCCGCG**C**GGGCCCGGGAGATGGAGCGG  CCCGTACACGCGCCCCTTTT**C**TTCCTCCGCCCAGGGTCCGC  CGCCGGAGCATGCTCGTGGG**C**CTCCGGTGTGGACGCGGGGG  CTGGCGCGCGGCGGCAGGGC**C**TCGAAGCTGAAGCCCTCGCG  GGAGCATGCTCGTGGGCCTC**C**GGTGTGGACGCGGGGGTTTC  GCGGCGGCAGGGCCTCGAAG**C**TGAAGCCCTCGCGGGTGTAG  GGATCCGTGCGGACCCATCC**C**CGTACACGCGCCCCTTTTCT  CCCTCGCGGGTGTAGACGTG**C**AGGATCCGTGCGGACCCATC  CGGGTTGCTGTGCGCCGGAG**C**ATGCTCGTGGGCCTCCGGTG  AGGATCCGTGCGGACCCATC**C**CCGTACACGCGCCCCTTTTC  TGGACGCGGGGGTTTCGCAG**C**TGGCGCGCGGCGGCAGGGCC  CTTCCTCCGCCCAGGGTCCG**C**GCGGGCCCGGGAGATGGAGC  GAGATGGAGCGGCGCCGCCG**C**CGCCACCACGTCTGCGGTCC  CGCGGGCCCGGGAGATGGAG**C**GGCGCCGCCGCCGCCACCAC  GCGAGGCCAGTGCTGCGCGT**C**CGCTCGGGTTGCTGTGCGCC  ACGTCTGCGGTCCTGAGCCC**C**CAGCGGGCGAGAGAGCGGAC  TGGTCCAAGAGCTGCGAGGC**C**AGTGCTGCGCGTCCGCTCGG  GGCGCGCGGCGGCAGGGCCT**C**GAAGCTGAAGCCCTCGCGGG  CGTCTGCGGTCCTGAGCCCC**C**AGCGGGCGAGAGAGCGGACT  GCAGCGGGAAGTGCGCTGTG**C**AGGAGCCAGACGTCTCAGCC  CGGGAGCGGGGCCAGGAGCG**C**TCCGCGGCCGGAGAAGGGCA  TGCCTCCGCTGCCATCTGCG**C**TGTCCTTGGCGACTACGCCT  GGCGCGGGCAGGATTTGGGG**C**TGCGCCGAGGGGCGTCCCGA  GGGCTGCGCCGAGGGGCGTC**C**CGACCTGGCCCTTTGCCACG  CCGCTGCCATCTGCGCTGTC**C**TTGGCGACTACGCCTTCACC  GCGACTACGCCTTCACCCCC**C**TGGGTGCGTTTCAGCGGTCC  GCTGTCCTTGGCGACTACGC**C**TTCACCCCCCTGGGTGCGTT  GAAGGGCAGCGGGAAGTGCG**C**TGTGCAGGAGCCAGACGTCT  AGGTTCCGGCTCCCATGCCT**C**CGCTGCCATCTGCGCTGTCC  GCCTGAGCGGGGCTGGGGCG**C**GGGCAGGATTTGGGGCTGCG  GGCGGGAGCGGGGCCAGGAG**C**GCTCCGCGGCCGGAGAAGGG  GGCCAGGAGCGCTCCGCGGC**C**GGAGAAGGGCAGCGGGAAGT  TTTGCCACGGTAGGTTCCGG**C**TCCCATGCCTCCGCTGCCAT  GCCCTTTGCCACGGTAGGTT**C**CGGCTCCCATGCCTCCGCTG  AGAGGTGGCAGGGGCGCGGC**C**TGAGCGGGGCTGGGGCGCGG  GGCGACTACGCCTTCACCCCCCTGGGTGCGTTTCAGCGGTC  CCCGACCTGGCCCTTTGCCACGGTAGGTTCCGGCTCCCATG  CTTGGCGACTACGCCTTCAC**C**CCCCTGGGTGCGTTTCAGCG  GGGGCTGCGCCGAGGGGCGT**C**CCGACCTGGCCCTTTGCCAC  GGAAGTGCGCTGTGCAGGAG**C**CAGACGTCTCAGCCCGTGTG  GAGGGGCGTCCCGACCTGGC**C**CTTTGCCACGGTAGGTTCCG  CCCTTTGCCACGGTAGGTTC**C**GGCTCCCATGCCTCCGCTGC  ACGGAAGCCTGGGGTCCCCA**C**CGCGCGTTCATCAGGTTCGG  TGCGCAGAGGTAACACGTCA**C**ATCGCATGTTCCTTTTGGCG  ACCGCAGCGCCCCGGTGGGT**C**CCGGGCGGGCCGGACGCGCC  CAGGAAGCACGACTGGGCGC**C**TTAGGACGTCCGGGCAGACG  CCTTAGGACGTCCGGGCAGA**C**GCGGCCCCCGAGAATTTTTC  CGAGCTGGAGACCCCGAAAA**C**CAGGGCCACTCGGGGAGTGT  GGTCCGAGGCGCAAGGCGAG**C**TGGAGACCCCGAAAACCAGG  CCGCAGCGCCCCGGTGGGTC**C**CGGGCGGGCCGGACGCGCCT  CGCGGCCCCCGAGAATTTTT**C**TAAGCCCTGCGCAGACCGCA  CCCCCGAGAATTTTTCTAAG**C**CCTGCGCAGACCGCAGCGCC  AAGGGCGCGGGTCCGAGGCG**C**AAGGCGAGCTGGAGACCCCG  GCCCTGCGCAGACCGCAGCG**C**CCCGGTGGGTCCCGGGCGGG  GTCCGGGCAGACGCGGCCCC**C**GAGAATTTTTCTAAGCCCTG  ACTGGGCGCCTTAGGACGTC**C**GGGCAGACGCGGCCCCCGAG  CGCAAGGCGAGCTGGAGACC**C**CGAAAACCAGGGCCACTCGG  ACTCGGGGAGTGTCAGGAAG**C**ACGACTGGGCGCCTTAGGAC  GGAGACCCCGAAAACCAGGG**C**CACTCGGGGAGTGTCAGGAA  GAATTTTTCTAAGCCCTGCG**C**AGACCGCAGCGCCCCGGTGG  GGCGCAAGGCGAGCTGGAGA**C**CCCGAAAACCAGGGCCACTC  TTAGGACGTCCGGGCAGACG**C**GGCCCCCGAGAATTTTTCTA  CAGGGCCACTCGGGGAGTGT**C**AGGAAGCACGACTGGGCGCC  CGGGCGGGCCGGACGCGCCT**C**CCAAGGGCGCGGGTCCGAGG  GGACGTCCGGGCAGACGCGG**C**CCCCGAGAATTTTTCTAAGC  GGAGTGAGGAGGCCCCAGTC**C**CCTTTCACCAAGCCCGTCCT  GAGGCCCCAGTCCCCTTTCA**C**CAAGCCCGTCCTCCCCTTCC  CTTTCACCAAGCCCGTCCTC**C**CCTTCCCGCTGCCACGACAC  TCCCCTTTCACCAAGCCCGT**C**CTCCCCTTCCCGCTGCCACG  AGCCCGTCCTCCCCTTCCCG**C**TGCCACGACACTGGTGCCTG  CAAGCCCGTCCTCCCCTTCC**C**GCTGCCACGACACTGGTGCC  AGTGAGGAGGCCCCAGTCCC**C**TTTCACCAAGCCCGTCCTCC  CTGATTTCAGGGTGAGGGAG**C**TGGCAGCCCCAGGAGTGAGG  TCTAGAAAGGATCCTGGGGT**C**CGATTGAGAAGCTGGCTTGA  AGGCCCCAGTCCCCTTTCAC**C**AAGCCCGTCCTCCCCTTCCC  CGCTGCCACGACACTGGTGC**C**TGCAGCCGTGCCAGCTAGGA  ACCTCTGCCTTGGCCGCCCC**C**TCTGAGAAGTGGGTGAAGCG  TGCTGCGTGTGGGTCGGAGC**C**GGGGACTCAGGGAGGATCGT  CCCTGCCCCAGTCCCAGCAT**C**TGGGAAAGGAAAAAGGTGGG  GTGACCTAGTAGGGTTCAGC**C**TGGGGAGCCCCTCCCTGCCC  TCGTTCTGCACGGCTGGGGA**C**AGCGGTGTGTGCGCGTGGGT  GGGTTCAGCCTGGGGAGCCC**C**TCCCTGCCCCCGTCCCAGCA  TTGCCTCGCTTCTAGGGTGA**C**CTAGTAGGGTTCAGCCTGGG  GCCACCCTTCCTTGGTTTTG**C**CGGTGCATGTTTTCTGAAGT  GCTGGGGACAGCGGTGTGTG**C**GCGTGGGTGTGTACGCGTGG  TTCAGCCTGGGGAGCCCCTC**C**CTGCCCCAGTCCCAGCATCT  TGTGCGCGTGGGTGTGTACG**C**GTGGGTGTGTGCACGTGGGT  AAGAGAAGCTCGCCTCGCTT**C**TAGGGTGACCTAGTCGGGTT  AAAATGGTGGAGGGGGGACC**C**CTGAGGCCCGGAGCTGCTTG  AAAACAGAAGAGAAGCTTGC**C**TTGCTTCTAGGGTGACCTAG  TGTGTGCGTGTGGGTGTGTG**C**ATGCGTGTACACACAGCTGC  CATGGGCGTGCAAGTGGGTG**C**GTGTGCATGGGGGTGTGCAC  CAAAACGACTGCGTGGGAAG**C**GTTTAGCGAGTGCTGCGTGT  CTGGGGAGCCCCTCCCTGCC**C**CCGTCCCAGCATCTGGGAAA  AGCGCAACCCAGCAAAACGA**C**TGCGTGGGAAGCGTTTAGCG  CATGTTTTCTGAAGTTTCTA**C**CAGGGGCTCAGGGGAGCGGA  GATCCCAGACACCTCTGCCT**C**GGCCGCCCCCTCTGAGAAGT  CTACCAGGGGCTCAGGGGCG**C**CGAAGAAAAACAGAAGAGAA  GAACCCGGGACGCAGCCACC**C**TTCCTTGGTTTTGCCGGTGC  GAGGCCCGGAGCTGCTAGAT**C**CCCGACACCTCTGCCTAGGC  TGGGGAGCCCCTCCCTGCCC**C**GGTCCCAGCATCTGGGAAAG  ACCCCTGAGGCCGGGAGCTG**C**TTGATCCCTGACACCTCTGC  ACTCAGGGAGGATCGTTCTG**C**ACGGCTGGGGACAGCGGTGT  CCTCTGAGAAGTGGGTGAAG**C**GCAACCCAGCAAAACGACTG  CCCTGAGGCCCGGAGCTGCT**C**GATCCCCGACACCTCTGCCT  GAAGCGTTTAGCGAGTGCTG**C**GTGTGGGTCGGAGCCGGGGA  TGTGTGCGCGTGGGTGTGTA**C**GCGTGGGTGTGTGCACGTGG  CGTGCAAGTGGGTGCGTGTG**C**ATGGGGGTGTGCACGTGGGT  GTGCTGCGTGTGGGTCGGAG**C**CGGGGACTCAGGGAGGATCG  TCAGGGGTGCGGAAGAAAAA**C**AGAAGAGAAGCTCGCCTAGC  TGCGTGTGCATGGGGGTGTG**C**ACGTGGGTGTGTGCGTGTGG  CAGAAGAGAAGCTCGCCTCG**C**TTCTAGGGTGACCTAGTCGG  GCAACCCAGCAAAACGACTG**C**GTGGGAAGCGTTTAGCGAGT  TGCTCAACTGCCTGTGCGAG**C**TCTCCAAGGAGGACGGCAAG  GAGGGGAAAGGGCCGCGTCC**C**CCGCGCGCGCGCGACGTGCA  CCAACCCGGGGCCGCGGTGC**C**TCCTGCGCGTCCCCCCGGAG  TCTCCAAGGAGGACGGCAAG**C**CCCTCTTCGCCTGGTGAGCC  GACTCGCTGCTGCTGCTCAA**C**TGCCTGTGCGAGCTCTCCAA  GCCCCTCTTCGCCTGGTGAG**C**CGCCCCGCGCCCGCCGCCTT  CGCGCCAACCCCCTGCACAG**C**AGCCCGGCCGCGCTGCGCAA  GCCCCGCGCCCGCCGCCTTG**C**CTGCAGTAAACGCGTTTGTT  CCGCGGTGCCTCCTGCGCGT**C**CCCCCGGAGGGGAAAGGGCC  CCCCTGCACAGCAGCCCGGC**C**GCGCTGCGCAAGCTGGTCAT  GCCAACCCCCTGCACAGCAG**C**CCGGCCGCGCTGCGCAAGCT  CCCCCTGCACAGCAGCCCGG**C**CGCGCTGCGCAAGCTGGTCA  CGCGCGCGCGCGACGTGCAC**C**CGGCGTTCAGCGAGTTCCTC  CCGCGCGCGCGCGACGTGCA**C**CCGGCGTTCAGCGAGTTCCT  CCAAGGAGGACGGCAAGCCC**C**TCTTCGCCTGGTGAGCCGCC  CTCATCAACACCTACGGAAT**C**CTGAAGCAGCGGCCCGACCT  CTCTTCGCCTGGTGAGCCGC**C**CCGCGCCCGCCGCCTTGCCT  TCATCAACACCTACGGAATC**C**TGAAGCAGCGGCCCGACCTG  GGCGACTCGCTGCTGCTGCT**C**AACTGCCTGTGCGAGCTCTC  GCTGGTCATCGACGTGGTGC**C**CCCCAAGTTCCTGGGCGACT  TGGGCGACTCGCTGCTGCTG**C**TCAACTGCCTGTGCGAGCTC  AGCAGCCCGGCCGCGCTGCG**C**AAGCTGGTCATCGACGTGGT  GGCCCGACCTGCGCGCCAAC**C**CCCTGCACAGCAGCCCGGCC  GAGCTCTCCAAGGAGGACGG**C**AAGCCCCTCTTCGCCTGGTG  CCTCTTCGCCTGGTGAGCCG**C**CCCGCGCCCGCCGCCTTGCC  TGGTCATCGACGTGGTGCCC**C**CCAAGTTCCTGGGCGACTCG  ACCTGCGCGCCAACCCCCTG**C**ACAGCAGCCCGGCCGCGCTG  AGCACTCGTGTGGAGCGACT**C**TGGGAGGAAAACTAGGGAGG  ATGCTCCCGTTTGGGCACAG**C**CTGGGGGCCGGGGCGCCCCA  CCCCAGACGCTCCAGGGTCC**C**CGCCTGCCTTAAGCGTCTAC  CTCCCTGGACAGACGTAGGA**C**CCAGGAGCAGGGTTAATTCC  GTCTACCTCCTCCCCTTCAC**C**CCACAACACGCGCTCTCTGG  GAGCGACTCTGGGAGGAAAA**C**TAGGGAGGAGGCCTGAGGAG  ACAGCCTGGGGGCCGGGGCG**C**CCCAGACGCTCCAGGGTCCC  GGTCACTTTGACCCCACGCC**C**AGCACGTAGTAGGCGCCCAG  ACAACACGCGCTCTCTGGGC**C**CCCTTTCACCCACCCACCCC  CCCCTTTCACCCACCCACCC**C**CAATTTCTATCTCCTTGTAA  GGGGAAAGCGCTCTTGGGAC**C**CAGGCCGAATGCTCCCGTTT  GGGAAAGCGCTCTTGGGACC**C**AGGCCGAATGCTCCCGTTTG  TCTCCTTGTAATCTGTGTGT**C**CTGCTTTAATGTTGGGGGGC  CCTTAAGCGTCTACCTCCTC**C**CCTTCACCCCACAACACGCG  GGCCCCCTTTCACCCACCCA**C**CCCCAATTTCTATCTCCTTG  TTCACCCCACAACACGCGCT**C**TCTGGGCCCCCTTTCACCCA  ACCCAGGAGCAGGGTTAATT**C**CTCCTGGGGACCGGGGAAAG  TGGGCCCCCTTTCACCCACC**C**ACCCCCAATTTCTATCTCCT  CCCCCTTTCACCCACCCACC**C**CCAATTTCTATCTCCTTGTA  CGCTCAGCTCCCTGGACAGA**C**GTAGGACCCAGGAGCAGGGT  CCAGCACTCGTGTGGAGCGA**C**TCTGGGAGGAAAACTAGGGA  AATTTCTATCTCCTTGTAAT**C**TGTGTGTCCTGCTTTAATGT  CGCCCCAGACGCTCCAGGGT**C**CCCGCCTGCCTTAAGCGTCT  CACCCCACAACACGCGCTCT**C**TGGGCCCCCTTTCACCCACC  GCCGAATGCTCCCGTTTGGG**C**ACAGCCTGGGGGCCGGGGCG  CCCACCCCCAATTTCTATCT**C**CTTGTAATCTGTGTGTCCTG  CCTGCTTTAATGTTGGGGGG**C**ACGGGCGCGAACTCTGGGTC  AGGAGCAGGGTTAATTCCTC**C**TGGGGACCGGGGAAAGCGCT  AGCCTGGGGGCCGGGGCGCC**C**CAGACGCTCCAGGGTCCCCG  AGCACGTAGTAGGCGCCCAG**C**ACTCGTGTGGAGCGACTCTG  GGGAGGAGGCCTGAGGAGCG**C**CCCGCTCAGCTCCCTGGACA  TGGGACCCAGGCCGAATGCT**C**CCGTTTGGGCACAGCCTGGG  GCCCCAGACGCTCCAGGGTC**C**CCGCCTGCCTTAAGCGTCTA  GGGCCGGGGCGCCCCAGACG**C**TCCAGGGTCCCCGCCTGCCT  CTTGTAATCTGTGTGTCCTG**C**TTTAATGTTGGGGGGCACGG  CACCCACCCACCCCCAATTT**C**TATCTCCTTGTAATCTGTGT  GCCCCCTTTCACCCACCCAC**C**CCCAATTTCTATCTCCTTGT  CCTTCACCCCACAACACGCG**C**TCTCTGGGCCCCCTTTCACC  TCTGGGCCCCCTTTCACCCA**C**CCACCCCCAATTTCTATCTC  CACGCCCAGCACGTAGTAGG**C**GCCCAGCACTCGTGTGGAGC  CTTAAGCGTCTACCTCCTCC**C**CTTCACCCCACAACACGCGC  CACCCACCCCCAATTTCTAT**C**TCCTTGTAATCTGTGTGTCC  AGGAAAACTAGGGAGGAGGC**C**TGAGGAGCGCCCCGCTCAGC  GGACCCAGGCCGAATGCTCC**C**GTTTGGGCACAGCCTGGGGG  CCTGGGGACCGGGGAAAGCG**C**TCTTGGGACCCAGGCCGAAT  CGAACTCTGGGTCACTTTGA**C**CCCACGCCCAGCACGTAGTA  GCTCTCTGGGCCCCCTTTCA**C**CCACCCACCCCCAATTTCTA  TTGGGGGGCACGGGCGCGAA**C**TCTGGGTCACTTTGACCCCA  AATGTTGGGGGGCACGGGCG**C**GAACTCTGGGTCACTTTGAC  TTAAGCGTCTACCTCCTCCC**C**TTCACCCCACAACACGCGCT  TCTCTGGGCCCCCTTTCACC**C**ACCCACCCCCAATTTCTATC  CCTGCCTTAAGCGTCTACCT**C**CTCCCCTTCACCCCACAACA  CCCAGACGCTCCAGGGTCCC**C**GCCTGCCTTAAGCGTCTACC  AAAGGCTGGAACTCCGCTTC**C**CAGAATGCAAAGCGCGGGGC  TGGAAGGGGGACTCAGTCGT**C**ACTGGCAGCGGTTGAATGGA  TCCGATTGCCTCAGAGCAAG**C**GGACTACATTTCCCAGGGGG  AGTCTCTCGCCACGGCGCTC**C**TCCCCCTCAGTCTCACTGGT  AAAGAGAAAGGCTGGAACTC**C**GCTTCCCAGAATGCAAAGCG  GGACTACATTTCCCAGGGGG**C**TGCGGGACTGCCGGGAGGAG  TTTCCCAGGGGGCTGCGGGA**C**TGCCGGGAGGAGTGGGGGCG  TGGAGGCTCTTTGCCCCGCG**C**CCTCACGGTAGATGAAGTCC  TCTGGTACAGTCATCACAAG**C**CTGTTCGGCGACGACCAAAG  GGAGTGGGGGCGGTGCCTCA**C**GTCTGGTACAGTCATCACAA  ATGCAAAGCGCGGGGCGGGG**C**TAAAGAGATAGCCTGTTTTC  GGCTCTTTGCCCCGCGCCCT**C**ACGGTAGATGAAGTCCTTGC  ACTAACGGTTCTCACCCTCC**C**TTTCATTCCCTCCCGCGGAA  CAGTCTCTCGCCACGGCGCT**C**CTCCCCCTCAGTCTCACTGG  GGGGACTCAGTCGTCACTGG**C**AGCGGTTGAATGGAGGCTCT  AATAACTAACGGTTCTCACC**C**TCCCTTTCATTCCCTCCCGC  GTCTCACTGGTCCTGCCGGC**C**GTTGCGTGGAAGGGGGACTC  TCCCCCTCAGTCTCACTGGT**C**CTGCCGGCCGTTGCGTGGAA  GATAGCTTCTCCGATTGCCT**C**AGAGCAAGCGGACTACATTT  TGCCTCACGTCTGGTACAGT**C**ATCACAAGCCTGTTCGGCGA  GGGAGGAGTGGGGGCGGTGC**C**TCACGTCTGGTACAGTCATC  CCCTCCCTTTCATTCCCTCC**C**GCGGAAAGAGAAAGGCTGGA  CTCTCGCCACGGCGCTCCTC**C**CCCTCAGTCTCACTGGTCCT  CTACATTTCCCAGGGGGCTG**C**GGGACTGCCGGGAGGAGTGG  GAAAGGCTGGAACTCCGCTT**C**CCAGAATGCAAAGCGCGGGG  CCCTCACGGTAGATGAAGTC**C**TTGCTTTGGTCAGAGCGATA  CTCTTTGCCCCGCGCCCTCA**C**GGTAGATGAAGTCCTTGCTT  CTCGCCACGGCGCTCCTCCC**C**CTCAGTCTCACTGGTCCTGC  AAGGCTGGAACTCCGCTTCC**C**AGAATGCAAAGCGCGGGGCG  CTCCCGCGGAAAGAGAAAGG**C**TGGAACTCCGCTTCCCAGAA  GCTCCTCCCCCTCAGTCTCA**C**TGGTCCTGCCGGCCGTTGCG  CCAGGGGGCTGCGGGACTGC**C**GGGAGGAGTGGGGGCGGTGC  GGTCAGAGCGATAGCTTCTC**C**GATTGCCTCAGAGCAAGCGG  GCGGTGCCTCACGTCTGGTA**C**AGTCATCACAAGCCTGTTCG  ATCAGCGGGTCCGCGGGGCG**C**TTGATACACAGGTAAAGTTC  GCTGAACTGACTGTCCGCTG**C**CAAGGGAAGTGACAGCCGCA  TCCGGGGCGGAGTGCGTGTC**C**TCCCACCGTGCTGGCGCTGA  GCGGAGTGCGTGTCCTCCCA**C**CGTGCTGGCGCTGAACTGAC  GGGACAGACAGACCGGCGTA**C**ACACTCAATCCTCCGGGGCG  CTCAGCCAGCGGCCGGGCGC**C**CCGCGGACCATGCTCTCCAG  TGAACGTGGACGGACGAGCC**C**TTGTTTCAGTAGCCAACACA  AGAACGCGGGCGGCTCCTAT**C**AGCGGGTCCGCGGGGCGCTT  GTGACAGCCGCAGCCGGGCT**C**TCAGCCAGCGGCCGGGCGCC  TGATACACAGGTAAAGTTCT**C**TGGTCCTTAGGGGAGAGGAG  GCCGGGCTCTCAGCCAGCGG**C**CGGGCGCCCCGCGGACCATG  CGAGCCCTTGTTTCAGTAGC**C**AACACACACGGGACAGACAG  TTGTTTCAGTAGCCAACACA**C**ACGGGACAGACAGACCGGCG  CTGAACTGACTGTCCGCTGC**C**AAGGGAAGTGACAGCCGCAG  GGGCGGAGTGCGTGTCCTCC**C**ACCGTGCTGGCGCTGAACTG  TCCGCTGCCAAGGGAAGTGA**C**AGCCGCAGCCGGGCTCTCAG  GGAAGTGGGGAGAGGTGAGT**C**CTCGAACCCTGAACGTGGAC  GCTGGCGCTGAACTGACTGT**C**CGCTGCCAAGGGAAGTGACA  CTCCGGGGCGGAGTGCGTGT**C**CTCCCACCGTGCTGGCGCTG  ACGCAGAACGCGGGCGGCTC**C**TATCAGCGGGTCCGCGGGGC  GAGAGGCGGGTCGTGGGGAC**C**ACTGTGGGTGCAGGAGTGGA  GAGGCGGGTCGTGGGGACCA**C**TGTGGGTGCAGGAGTGGAAG  AGACCGGAGGATGTTCCCTG**C**TCAGGAGGAGGCCGACAGGA  CCGGCCCCCTTCCGTCCCAT**C**CTGAGCAGGGGTTTTAATTT  CCGGAGAAGGGGCGGGGTCT**C**AGCTCCTACTTCATTCTACG  CGGGGTCTCAGCTCCTACTT**C**ATTCTACGGCCGAGACCGGA  TTTGTTGGGAATTTAGAGGC**C**CGAGTTCGGGAAGAGATTCT  GCTCCCGGCGCCGGCCCCGC**C**CCCTCACCCAGGCGGCGCCG  ACGAGCTGTTCCTTCAGGTA**C**CGTCTCTGGGAAGGGGCGGC  ACCCAGGCGGCGCCGTAAAG**C**CGGAGAAGGGGCGGGGTCTC  GCTGTTCCTTCAGGTACCGT**C**TCTGGGAAGGGGCGGCGGAG  CGGCCCCCTTCCGTCCCATC**C**TGAGCAGGGGTTTTAATTTC  CTGAGCAGGGGTTTTAATTT**C**GTTTCCTCTTGGCGCACCTG  GGCGCGTTCCCCATCCCCAT**C**CGCGCTGCAGAGTCCTGGGG  CCCCATCCGCGCTGCAGAGT**C**CTGGGGTTGGGCAGGGACGT  GGAGCACGTCGGGCCGGAAA**C**ATAGCGCGCACCTGGACCAC  CGCCGGCCCCGCCCCCTCAC**C**CAGGCGGCGCCGTAAAGCCG  AAACATAGCGCGCACCTGGA**C**CACCCGGAGCCCGGCCCCCT  TGAAATAAGGGCGCGTTCCC**C**ATCCCCATCCGCGCTGCAGA  CCCGGCGCCGGCCCCGCCCC**C**TCACCCAGGCGGCGCCGTAA  TTTCTCGCCCCCAGCGCGCG**C**TCCCGGCGCCGGCCCCGCCC  GTGAAATAAGGGCGCGTTCC**C**CATCCCCATCCGCGCTGCAG  CCCCATCCCCATCCGCGCTG**C**AGAGTCCTGGGGTTGGGCAG  CCAGCGCGCGCTCCCGGCGC**C**GGCCCCGCCCCCTCACCCAG  CCCATCCGCGCTGCAGAGTC**C**TGGGGTTGGGCAGGGACGTG  GTTCCCCATCCCCATCCGCG**C**TGCAGAGTCCTGGGGTTGGG  GGTTGGGCAGGGACGTGGTT**C**CTTCAACCCCCGTTTCTCGC  TGGACCACCCGGAGCCCGGC**C**CCCTTCCGTCCCATCCTGAG  TTTCCTCTTGGCGCACCTGT**C**TCCAGGTGAGCGGCTGGGGG  GTTCCCTGCTCAGGAGGAGG**C**CGACAGGACCGTGTTTGTTG  GTCCTGGGGTTGGGCAGGGA**C**GTGGTTCCTTCAACCCCCGT  ACCGGAGGATGTTCCCTGCT**C**AGGAGGAGGCCGACAGGACC  AGGGACGTGGTTCCTTCAAC**C**CCCGTTTCTCGCCCCCAGCG  GCACCTGGACCACCCGGAGC**C**CGGCCCCCTTCCGTCCCATC  GGGCGGGGTCTCAGCTCCTA**C**TTCATTCTACGGCCGAGACC  TAAGGGCGCGTTCCCCATCC**C**CATCCGCGCTGCAGAGTCCT  ACCTGTCTCCAGGTGAGCGG**C**TGGGGGGTGAAATAAGGGCG  GCCCCCTCACCCAGGCGGCG**C**CGTAAAGCCGGAGAAGGGGC  CTCCCGGCGCCGGCCCCGCC**C**CCTCACCCAGGCGGCGCCGT  ACGCGGATTGCACACATCGG**C**CACTTCCTGCACCACGTGCA  GCAATGACGCGCGCACGCCT**C**ACGCGCACGCCGCATAACGT  CAGGCGCTCTAGCCCCAGTC**C**TGCTTCAAGAGGCTTTTTCC  TTTTCCCACCACAGGCTTCT**C**CTCAGTGGCCAGAAAGCTGG  CTTCAAGAGGCTTTTTCCCA**C**CACAGGCTTCTCCTCAGTGG  CCCCGCACGCGCGCCCGCCT**C**ACGCGCGCCCCGCACGCACA  CATGAACTTTGCCAGTAACG**C**AAGGCTGCCACTGACATTTG  AGACTTGCGCACGCGGATTG**C**ACACATCGGCCACTTCCTGC  GCGCCCGCCTCACGCGCGCC**C**CGCACGCACATGACGGCTTG  CGTCTGAGGCGCGCACGCCT**C**ACGCGCGCCCCGCACGCGCG  TGCCAGTAACGCAAGGCTGC**C**ACTGACATTTGTGGCGCCAA  AACGTCTGAGGCGCGCACGC**C**TCACGCGCGCCCCGCACGCG  TCCCACCACAGGCTTCTCCT**C**AGTGGCCAGAAAGCTGGGTC  CAGTGGCCAGAAAGCTGGGT**C**AACTCCCATGAACTTTGCCA  CCCGCCTCACGCGCGCCCCG**C**ACGCACATGACGGCTTGGCT  GCGCTCTAGCCCCAGTCCTG**C**TTCAAGAGGCTTTTTCCCAC  AGGCTTTTTCCCACCACAGG**C**TTCTCCTCAGTGGCCAGAAA  TCACGCGCGCCCCGCACGCA**C**ATGACGGCTTGGCTTACCTG  GGTGCGCTTCGCTTTGCGTT**C**CTCGCTTGGCCTTGCGTTCC  CGCCCCGCACGCGCGCCCGC**C**TCACGCGCGCCCCGCACGCA  GTCAACTCCCATGAACTTTG**C**CAGTAACGCAAGGCTGCCAC  GCTTGGCCTTGCGTTCCTAG**C**TTGGCTTGGCGTTAGTCGCT  TAACGCAAGGCTGCCACTGA**C**ATTTGTGGCGCCAAGACTTG  ATTGGCGTTTTCTCCCTCGT**C**GAGTTCCAGGCGCTCTAGCC  GTGCAATGACGCGCGCACGC**C**TCACGCGCACGCCGCATAAC  CTTGGCGTTAGTCGCTTTGC**C**TGGTGTTTCTTGCTTGGATT  ACGCGCACGCCGCATAACGT**C**TGAGGCGCGCACGCCTCACG  CGCGCCCGCCTCACGCGCGC**C**CCGCACGCACATGACGGCTT  GCACGCACATGACGGCTTGG**C**TTACCTGTAATGGGTGCGCT  ACTTGCGCACGCGGATTGCA**C**ACATCGGCCACTTCCTGCAC  GCGCGCACGCCTCACGCGCG**C**CCCGCACGCGCGCCCGCCTC  CACATCGGCCACTTCCTGCA**C**CACGTGCAATGACGCGCGCA  TTGCACACATCGGCCACTTC**C**TGCACCACGTGCAATGACGC  GAAAGCTTAGGCTCCCCTCA**C**CACTCCCCCCTCCCCGACCT  CCCCTCACCACTCCCCCCTC**C**CCGACCTGCAGCAGCGCGGG  GAGAGGCAAGGAAGCTGGCA**C**CCGTGCCTCTTCTGCCGCCT  GGCTCCCCTCACCACTCCCC**C**CTCCCCGACCTGCAGCAGCG  ACCCGTGCCTCTTCTGCCGC**C**TGCCTGCTCCTTGAGCCGGG  CCCTCACCACTCCCCCCTCC**C**CGACCTGCAGCAGCGCGGGA  CCCCCCTCCCCGACCTGCAG**C**AGCGCGGGAAATTCTGTGAA  CACCCGTGCCTCTTCTGCCG**C**CTGCCTGCTCCTTGAGCCGG  GGGAAGTTGGGACTGGGCGG**C**AGTGACGCCTGGAGAACGTG  GGGACCACCGGAGGCAGTGT**C**GCTGTGGGTGCCACGCAGGT  GTGCGGGCATGACTCGTGCT**C**TGTGGATGTGGCAGAAAGCG  CCTCACCACTCCCCCCTCCC**C**GACCTGCAGCAGCGCGGGAA  TGAATGGAAAGCTTAGGCTC**C**CCTCACCACTCCCCCCTCCC  GCTCCCCTCACCACTCCCCC**C**TCCCCGACCTGCAGCAGCGC  GGGACTGGGCGGCAGTGACG**C**CTGGAGAACGTGGCGGGTAG  AGCTGGCACCCGTGCCTCTT**C**TGCCGCCTGCCTGCTCCTTG  GAGAGAGCGCCGGGAAGGGA**C**TGAATGGAAAGCTTAGGCTC  GGACTGGGCGGCAGTGACGC**C**CGGAGAACGTGGCGGGTAGA  GACTGAATGGAAAGCTTAGG**C**TCCCCTCACCACTCCCCCCT  TTGAGCCGGGGAAGTTGGGA**C**TGGGCGGCAGTGACGCCAGG  GTGACGCCGGGAGAACGTGG**C**GGGTAGAGAGAGCGCCGGGA  AATGGAAAGCTTAGGCTCCC**C**TCACCACTCCCCCCTCCCCG  AACGTGGCGGGTAGAGAGAG**C**GCCGGGAAGGGACTGAATGG  CCGGGGAAGTTGGGACTGGG**C**GGCAGTGACGCCGGGAGAAC  GGAAGGGACTGAATGGAAAG**C**TTAGGCTCCCCTCACCACTC  TCCCCTCACCACTCCCCCCT**C**CCCGACCTGCAGCAGCGCGG  GTGTCGCTGTGGGTGCCACG**C**AGGTAAGGAGAGAAGAGCGG  AGAGGCAAGGAAGCTGGCAC**C**CGTGCCTCTTCTGCCGCCTG |
