# Supplementary material for: Benchmark data for identifying DNA methylation sites via pseudo trinucleotide composition
Source: Data Brief. 2015 May 7;4:87–9. doi: 10.1016/j.dib.2015.04.021 (PMC4510404; doi:10.1016/j.dib.2015.04.021)
Supplement: Supplementary file 1 — Supplementary data [file mmc1.zip › Supp2.doc]

**Supporting Information S2**. **List of the 522 negative samples removed from the original negative subset via the NCR treatment.** Each of these samples is formulated by a vector consisting of 72 components (see **Eq.10** in ref [1]).

>Non-Methylation code 1

0.000 0.010 0.000 0.000 0.010 0.000 0.000 0.010 0.000 0.010

0.005 0.000 0.005 0.000 0.005 0.000 0.000 0.000 0.000 0.010

0.067 0.065 0.077 0.060 0.005 0.010 0.000 0.000 0.005 0.000

0.000 0.010 0.000 0.005 0.000 0.000 0.000 0.000 0.005 0.000

0.000 0.010 0.000 0.010 0.060 0.060 0.085 0.070 0.011 0.000

0.000 0.000 0.011 0.000 0.000 0.000 0.000 0.032 0.011 0.011

0.000 0.000 0.000 0.000 0.011 0.021 0.000 0.021 0.052 0.053

0.043 0.058

>Non-Methylation code 2

0.000 0.000 0.000 0.000 0.000 0.000 0.000 0.016 0.008 0.008

0.000 0.016 0.000 0.008 0.000 0.008 0.008 0.008 0.008 0.000

0.056 0.063 0.072 0.057 0.005 0.000 0.005 0.000 0.005 0.005

0.000 0.005 0.000 0.000 0.000 0.000 0.000 0.000 0.019 0.009

0.005 0.000 0.000 0.000 0.098 0.053 0.077 0.050 0.000 0.000

0.015 0.023 0.000 0.000 0.000 0.008 0.000 0.023 0.000 0.008

0.000 0.008 0.000 0.008 0.000 0.000 0.000 0.000 0.084 0.035

0.083 0.041

>Non-Methylation code 3

0.000 0.000 0.006 0.017 0.000 0.000 0.000 0.017 0.000 0.011

0.000 0.006 0.000 0.000 0.006 0.011 0.000 0.000 0.000 0.000

0.062 0.059 0.073 0.067 0.006 0.000 0.000 0.000 0.000 0.006

0.000 0.000 0.006 0.013 0.013 0.000 0.000 0.000 0.013 0.019

0.000 0.000 0.000 0.000 0.082 0.067 0.048 0.060 0.008 0.000

0.008 0.017 0.000 0.008 0.008 0.000 0.000 0.008 0.000 0.008

0.000 0.008 0.000 0.000 0.000 0.000 0.000 0.000 0.045 0.070

0.077 0.066

>Non-Methylation code 4

0.008 0.000 0.000 0.000 0.008 0.000 0.000 0.000 0.000 0.016

0.008 0.008 0.000 0.000 0.008 0.000 0.008 0.016 0.008 0.008

0.050 0.059 0.064 0.066 0.000 0.005 0.000 0.000 0.010 0.010

0.000 0.005 0.000 0.010 0.005 0.005 0.005 0.000 0.005 0.000

0.000 0.000 0.000 0.005 0.065 0.064 0.077 0.064 0.005 0.009

0.000 0.005 0.005 0.000 0.000 0.005 0.000 0.005 0.005 0.000

0.000 0.000 0.005 0.000 0.000 0.005 0.000 0.009 0.058 0.059

0.081 0.080

>Non-Methylation code 5

0.000 0.000 0.005 0.000 0.000 0.010 0.005 0.000 0.005 0.000

0.000 0.000 0.005 0.005 0.015 0.005 0.000 0.000 0.000 0.000

0.070 0.063 0.080 0.065 0.000 0.006 0.006 0.000 0.000 0.017

0.006 0.000 0.011 0.006 0.000 0.006 0.000 0.000 0.000 0.000

0.011 0.006 0.000 0.000 0.068 0.071 0.066 0.057 0.011 0.005

0.000 0.000 0.000 0.005 0.000 0.000 0.005 0.000 0.000 0.000

0.000 0.005 0.011 0.000 0.016 0.005 0.000 0.000 0.076 0.069

0.076 0.049

>Non-Methylation code 6

0.000 0.000 0.006 0.000 0.000 0.000 0.006 0.006 0.000 0.006

0.000 0.000 0.006 0.000 0.012 0.012 0.012 0.006 0.000 0.006

0.079 0.066 0.045 0.067 0.008 0.000 0.000 0.000 0.000 0.016

0.000 0.008 0.000 0.016 0.000 0.000 0.008 0.000 0.008 0.008

0.016 0.000 0.000 0.008 0.062 0.064 0.043 0.069 0.006 0.000

0.000 0.000 0.006 0.006 0.006 0.000 0.006 0.006 0.000 0.000

0.011 0.000 0.006 0.006 0.006 0.000 0.000 0.006 0.065 0.073

0.060 0.069

>Non-Methylation code 7

0.000 0.000 0.000 0.000 0.000 0.018 0.000 0.000 0.000 0.018

0.000 0.000 0.026 0.009 0.000 0.035 0.000 0.009 0.000 0.000

0.059 0.042 0.058 0.061 0.000 0.000 0.000 0.000 0.000 0.017

0.000 0.000 0.000 0.035 0.000 0.000 0.009 0.009 0.000 0.017

0.000 0.000 0.009 0.009 0.055 0.049 0.050 0.075 0.013 0.000

0.000 0.000 0.013 0.006 0.000 0.000 0.000 0.006 0.000 0.000

0.000 0.006 0.006 0.006 0.006 0.006 0.006 0.000 0.074 0.070

0.065 0.047

>Non-Methylation code 8

0.009 0.000 0.009 0.000 0.000 0.018 0.000 0.000 0.000 0.009

0.000 0.000 0.018 0.009 0.000 0.035 0.000 0.009 0.000 0.000

0.055 0.063 0.041 0.059 0.007 0.000 0.000 0.007 0.000 0.000

0.000 0.000 0.000 0.021 0.000 0.000 0.007 0.007 0.007 0.014

0.007 0.000 0.007 0.000 0.066 0.048 0.059 0.078 0.000 0.005

0.000 0.000 0.009 0.009 0.000 0.000 0.000 0.005 0.000 0.000

0.005 0.005 0.009 0.005 0.000 0.005 0.000 0.000 0.056 0.078

0.065 0.077

>Non-Methylation code 9

0.017 0.000 0.009 0.000 0.000 0.026 0.000 0.000 0.000 0.009

0.000 0.000 0.009 0.009 0.000 0.017 0.009 0.009 0.000 0.000

0.048 0.066 0.050 0.056 0.006 0.000 0.000 0.006 0.000 0.006

0.000 0.000 0.000 0.011 0.000 0.000 0.006 0.006 0.011 0.006

0.006 0.000 0.006 0.000 0.075 0.067 0.072 0.053 0.000 0.004

0.004 0.000 0.004 0.012 0.000 0.000 0.000 0.000 0.000 0.000

0.008 0.004 0.012 0.000 0.000 0.000 0.000 0.000 0.069 0.090

0.068 0.057

>Non-Methylation code 10

0.007 0.000 0.000 0.014 0.000 0.020 0.000 0.000 0.007 0.000

0.000 0.007 0.007 0.014 0.000 0.000 0.014 0.000 0.000 0.000

0.068 0.065 0.051 0.061 0.004 0.000 0.000 0.000 0.000 0.009

0.000 0.000 0.004 0.009 0.000 0.000 0.004 0.000 0.017 0.004

0.004 0.000 0.000 0.000 0.061 0.067 0.081 0.068 0.006 0.000

0.011 0.011 0.000 0.011 0.000 0.000 0.000 0.000 0.000 0.000

0.011 0.000 0.011 0.000 0.000 0.000 0.000 0.000 0.076 0.070

0.047 0.077

>Non-Methylation code 11

0.015 0.000 0.000 0.000 0.000 0.007 0.000 0.000 0.000 0.015

0.000 0.000 0.007 0.000 0.007 0.007 0.022 0.007 0.000 0.000

0.056 0.070 0.063 0.054 0.005 0.000 0.000 0.000 0.005 0.005

0.010 0.000 0.005 0.005 0.000 0.000 0.010 0.000 0.010 0.010

0.000 0.000 0.000 0.000 0.067 0.071 0.072 0.062 0.000 0.000

0.007 0.000 0.000 0.007 0.007 0.000 0.000 0.007 0.000 0.000

0.007 0.000 0.007 0.014 0.014 0.007 0.000 0.007 0.073 0.050

0.066 0.057

>Non-Methylation code 12

0.011 0.000 0.000 0.000 0.006 0.000 0.006 0.000 0.006 0.006

0.000 0.000 0.011 0.000 0.006 0.006 0.006 0.000 0.006 0.006

0.061 0.076 0.055 0.069 0.000 0.000 0.000 0.000 0.000 0.005

0.005 0.005 0.000 0.005 0.000 0.000 0.010 0.000 0.010 0.010

0.005 0.005 0.000 0.005 0.075 0.071 0.040 0.079 0.011 0.000

0.000 0.000 0.000 0.022 0.000 0.011 0.000 0.033 0.000 0.000

0.011 0.000 0.000 0.011 0.011 0.000 0.000 0.011 0.066 0.061

0.032 0.052

>Non-Methylation code 13

0.000 0.005 0.000 0.000 0.000 0.021 0.005 0.000 0.010 0.010

0.000 0.005 0.000 0.000 0.000 0.000 0.005 0.000 0.000 0.005

0.061 0.080 0.063 0.062 0.010 0.005 0.005 0.000 0.000 0.005

0.000 0.005 0.005 0.000 0.000 0.000 0.000 0.005 0.005 0.000

0.010 0.005 0.000 0.005 0.074 0.069 0.071 0.054 0.000 0.000

0.006 0.000 0.000 0.006 0.000 0.006 0.006 0.000 0.000 0.000

0.006 0.006 0.013 0.006 0.006 0.000 0.000 0.000 0.071 0.072

0.071 0.056

>Non-Methylation code 14

0.005 0.005 0.005 0.005 0.000 0.010 0.000 0.000 0.000 0.000

0.000 0.000 0.015 0.000 0.015 0.000 0.000 0.000 0.000 0.000

0.078 0.104 0.045 0.047 0.015 0.000 0.007 0.007 0.000 0.022

0.000 0.000 0.000 0.000 0.000 0.007 0.015 0.015 0.000 0.000

0.007 0.000 0.000 0.000 0.064 0.074 0.058 0.041 0.005 0.000

0.000 0.005 0.000 0.005 0.000 0.000 0.000 0.005 0.000 0.000

0.005 0.005 0.015 0.005 0.010 0.000 0.000 0.000 0.079 0.061

0.078 0.056

>Non-Methylation code 15

0.005 0.000 0.000 0.000 0.005 0.015 0.000 0.000 0.010 0.000

0.000 0.000 0.005 0.000 0.015 0.005 0.005 0.000 0.000 0.000

0.071 0.049 0.082 0.065 0.013 0.000 0.013 0.000 0.000 0.013

0.000 0.000 0.007 0.007 0.000 0.000 0.007 0.013 0.000 0.013

0.000 0.000 0.000 0.000 0.064 0.057 0.064 0.061 0.006 0.000

0.006 0.000 0.000 0.000 0.000 0.006 0.000 0.000 0.000 0.000

0.000 0.017 0.017 0.006 0.006 0.006 0.000 0.000 0.076 0.063

0.074 0.052

>Non-Methylation code 16

0.024 0.000 0.000 0.000 0.000 0.000 0.000 0.000 0.000 0.008

0.000 0.008 0.024 0.024 0.000 0.008 0.008 0.000 0.000 0.000

0.042 0.053 0.062 0.073 0.000 0.000 0.000 0.000 0.000 0.000

0.006 0.000 0.000 0.006 0.000 0.006 0.012 0.012 0.017 0.012

0.006 0.000 0.000 0.000 0.062 0.082 0.051 0.064 0.030 0.000

0.000 0.010 0.000 0.000 0.010 0.000 0.000 0.010 0.000 0.000

0.010 0.000 0.000 0.030 0.020 0.000 0.000 0.000 0.047 0.055

0.056 0.056

>Non-Methylation code 17

0.000 0.000 0.000 0.000 0.000 0.006 0.000 0.006 0.000 0.012

0.000 0.000 0.000 0.012 0.018 0.018 0.000 0.000 0.000 0.000

0.053 0.069 0.090 0.049 0.012 0.000 0.006 0.006 0.000 0.006

0.000 0.000 0.000 0.000 0.000 0.000 0.006 0.000 0.006 0.023

0.000 0.006 0.000 0.006 0.068 0.066 0.062 0.062 0.000 0.000

0.000 0.000 0.000 0.008 0.000 0.000 0.008 0.008 0.000 0.000

0.015 0.015 0.000 0.000 0.015 0.015 0.000 0.008 0.055 0.064

0.056 0.067

>Non-Methylation code 18

0.012 0.000 0.000 0.004 0.000 0.000 0.000 0.000 0.000 0.000

0.000 0.000 0.012 0.000 0.016 0.008 0.000 0.000 0.000 0.000

0.069 0.066 0.079 0.066 0.011 0.000 0.000 0.000 0.000 0.017

0.000 0.000 0.000 0.006 0.000 0.000 0.017 0.006 0.011 0.006

0.000 0.000 0.000 0.000 0.071 0.070 0.074 0.047 0.018 0.000

0.000 0.006 0.000 0.006 0.000 0.000 0.000 0.000 0.000 0.000

0.018 0.000 0.012 0.006 0.000 0.006 0.000 0.000 0.078 0.069

0.048 0.066

>Non-Methylation code 19

0.017 0.000 0.006 0.006 0.000 0.000 0.011 0.000 0.000 0.006

0.000 0.000 0.011 0.000 0.000 0.000 0.006 0.006 0.006 0.000

0.061 0.072 0.058 0.069 0.000 0.000 0.000 0.000 0.000 0.004

0.000 0.004 0.000 0.000 0.000 0.000 0.017 0.000 0.012 0.004

0.008 0.000 0.004 0.000 0.065 0.078 0.071 0.065 0.006 0.000

0.000 0.000 0.000 0.023 0.006 0.000 0.000 0.011 0.000 0.000

0.017 0.000 0.006 0.000 0.000 0.000 0.000 0.000 0.055 0.069

0.071 0.070

>Non-Methylation code 20

0.019 0.000 0.000 0.000 0.005 0.005 0.000 0.000 0.000 0.005

0.000 0.000 0.015 0.000 0.010 0.005 0.000 0.000 0.000 0.000

0.069 0.075 0.077 0.049 0.007 0.000 0.000 0.007 0.007 0.007

0.000 0.000 0.000 0.020 0.000 0.000 0.027 0.007 0.000 0.007

0.000 0.000 0.000 0.000 0.066 0.062 0.060 0.057 0.000 0.000

0.000 0.000 0.004 0.000 0.000 0.000 0.000 0.000 0.000 0.000

0.013 0.000 0.017 0.009 0.000 0.004 0.000 0.000 0.057 0.063

0.082 0.084

>Non-Methylation code 21

0.005 0.000 0.005 0.000 0.005 0.005 0.000 0.005 0.005 0.005

0.000 0.000 0.010 0.010 0.010 0.000 0.000 0.000 0.000 0.000

0.059 0.078 0.070 0.062 0.006 0.000 0.000 0.006 0.000 0.006

0.006 0.000 0.012 0.006 0.000 0.000 0.006 0.000 0.000 0.018

0.006 0.000 0.000 0.006 0.068 0.065 0.050 0.071 0.000 0.000

0.000 0.005 0.000 0.000 0.005 0.000 0.005 0.000 0.000 0.000

0.011 0.000 0.011 0.011 0.011 0.005 0.000 0.000 0.069 0.062

0.063 0.076

>Non-Methylation code 22

0.014 0.000 0.014 0.000 0.000 0.020 0.000 0.000 0.007 0.007

0.000 0.000 0.007 0.007 0.000 0.014 0.000 0.000 0.000 0.000

0.066 0.064 0.060 0.055 0.005 0.000 0.005 0.000 0.000 0.005

0.000 0.005 0.000 0.000 0.000 0.000 0.000 0.015 0.015 0.005

0.005 0.005 0.000 0.000 0.079 0.057 0.079 0.053 0.006 0.000

0.000 0.000 0.006 0.017 0.000 0.000 0.011 0.000 0.000 0.000

0.000 0.000 0.017 0.006 0.006 0.000 0.000 0.000 0.067 0.051

0.077 0.070

>Non-Methylation code 23

0.000 0.000 0.007 0.000 0.000 0.013 0.000 0.000 0.000 0.020

0.000 0.000 0.013 0.000 0.007 0.020 0.000 0.007 0.000 0.000

0.063 0.061 0.061 0.061 0.012 0.000 0.000 0.000 0.000 0.012

0.000 0.000 0.000 0.006 0.006 0.000 0.006 0.000 0.012 0.024

0.000 0.000 0.000 0.000 0.083 0.059 0.062 0.052 0.009 0.000

0.000 0.004 0.000 0.004 0.000 0.000 0.000 0.009 0.000 0.000

0.009 0.000 0.009 0.004 0.000 0.000 0.004 0.000 0.048 0.077

0.078 0.077

>Non-Methylation code 24

0.009 0.000 0.000 0.005 0.005 0.005 0.000 0.000 0.000 0.000

0.000 0.000 0.014 0.000 0.009 0.000 0.009 0.000 0.005 0.000

0.072 0.063 0.071 0.065 0.017 0.000 0.000 0.000 0.000 0.017

0.000 0.000 0.000 0.000 0.000 0.000 0.058 0.000 0.008 0.008

0.000 0.000 0.000 0.000 0.061 0.048 0.060 0.056 0.000 0.000

0.005 0.000 0.000 0.005 0.005 0.000 0.000 0.005 0.000 0.000

0.020 0.000 0.015 0.000 0.000 0.000 0.000 0.000 0.076 0.064

0.091 0.046

>Non-Methylation code 25

0.005 0.005 0.005 0.000 0.000 0.010 0.000 0.000 0.000 0.000

0.000 0.000 0.015 0.005 0.005 0.005 0.005 0.005 0.000 0.000

0.062 0.051 0.083 0.071 0.010 0.000 0.000 0.005 0.000 0.005

0.000 0.005 0.000 0.000 0.000 0.000 0.020 0.000 0.015 0.000

0.000 0.000 0.000 0.000 0.057 0.077 0.060 0.079 0.008 0.000

0.000 0.000 0.004 0.008 0.000 0.000 0.000 0.004 0.000 0.000

0.012 0.000 0.012 0.000 0.000 0.000 0.000 0.000 0.081 0.063

0.059 0.082

>Non-Methylation code 26

0.017 0.000 0.000 0.006 0.000 0.012 0.006 0.000 0.000 0.006

0.000 0.000 0.012 0.000 0.006 0.012 0.000 0.000 0.000 0.000

0.063 0.069 0.064 0.062 0.013 0.000 0.000 0.006 0.000 0.006

0.000 0.000 0.000 0.000 0.000 0.000 0.025 0.006 0.006 0.013

0.006 0.000 0.000 0.000 0.062 0.047 0.075 0.066 0.004 0.000

0.000 0.000 0.000 0.009 0.000 0.000 0.000 0.004 0.000 0.000

0.013 0.004 0.018 0.000 0.000 0.000 0.000 0.000 0.084 0.074

0.058 0.064

>Non-Methylation code 27

0.005 0.005 0.000 0.000 0.000 0.005 0.005 0.000 0.000 0.005

0.000 0.000 0.014 0.000 0.009 0.009 0.000 0.005 0.000 0.000

0.065 0.075 0.059 0.074 0.012 0.000 0.000 0.000 0.006 0.006

0.006 0.000 0.000 0.006 0.000 0.000 0.012 0.006 0.006 0.006

0.006 0.006 0.000 0.000 0.058 0.054 0.055 0.086 0.000 0.000

0.000 0.000 0.006 0.006 0.000 0.000 0.000 0.013 0.000 0.000

0.019 0.000 0.013 0.006 0.006 0.000 0.000 0.000 0.060 0.053

0.081 0.069

>Non-Methylation code 28

0.012 0.000 0.000 0.000 0.000 0.000 0.006 0.000 0.012 0.006

0.000 0.000 0.006 0.012 0.006 0.006 0.006 0.006 0.000 0.000

0.063 0.075 0.058 0.062 0.000 0.000 0.000 0.000 0.000 0.005

0.005 0.000 0.005 0.000 0.000 0.000 0.010 0.000 0.019 0.015

0.005 0.000 0.000 0.000 0.081 0.055 0.069 0.065 0.022 0.000

0.007 0.000 0.000 0.015 0.000 0.000 0.007 0.007 0.000 0.000

0.015 0.015 0.000 0.000 0.000 0.000 0.000 0.000 0.073 0.071

0.047 0.053

>Non-Methylation code 29

0.021 0.000 0.007 0.000 0.000 0.014 0.000 0.000 0.007 0.007

0.000 0.000 0.014 0.014 0.000 0.007 0.000 0.000 0.000 0.000

0.072 0.073 0.046 0.051 0.012 0.000 0.000 0.000 0.000 0.000

0.006 0.000 0.012 0.006 0.000 0.000 0.006 0.012 0.006 0.006

0.006 0.006 0.000 0.000 0.063 0.075 0.058 0.062 0.000 0.000

0.000 0.000 0.000 0.005 0.000 0.000 0.005 0.000 0.000 0.000

0.010 0.000 0.021 0.015 0.005 0.000 0.000 0.000 0.080 0.052

0.071 0.068

>Non-Methylation code 30

0.000 0.000 0.000 0.007 0.000 0.014 0.000 0.000 0.014 0.000

0.007 0.007 0.007 0.014 0.000 0.000 0.014 0.007 0.000 0.000

0.065 0.051 0.070 0.054 0.000 0.000 0.000 0.012 0.000 0.000

0.000 0.000 0.012 0.012 0.000 0.000 0.000 0.000 0.012 0.019

0.006 0.000 0.000 0.000 0.080 0.046 0.076 0.057 0.013 0.000

0.007 0.013 0.000 0.000 0.007 0.000 0.007 0.000 0.000 0.007

0.007 0.000 0.007 0.007 0.000 0.000 0.000 0.000 0.049 0.068

0.073 0.072

>Non-Methylation code 31

0.004 0.000 0.004 0.000 0.009 0.000 0.004 0.000 0.000 0.004

0.000 0.000 0.009 0.000 0.009 0.000 0.004 0.004 0.000 0.004

0.064 0.065 0.066 0.082 0.000 0.008 0.000 0.000 0.000 0.016

0.000 0.008 0.000 0.000 0.000 0.000 0.023 0.008 0.000 0.023

0.016 0.000 0.000 0.000 0.046 0.057 0.060 0.070 0.000 0.000

0.000 0.000 0.000 0.006 0.006 0.000 0.000 0.006 0.000 0.000

0.026 0.006 0.006 0.006 0.000 0.013 0.000 0.000 0.064 0.052

0.071 0.070

>Non-Methylation code 32

0.006 0.006 0.000 0.000 0.000 0.000 0.000 0.000 0.006 0.006

0.000 0.000 0.000 0.000 0.012 0.006 0.023 0.006 0.000 0.000

0.081 0.075 0.024 0.083 0.007 0.000 0.015 0.007 0.000 0.000

0.000 0.000 0.000 0.000 0.000 0.007 0.007 0.030 0.007 0.007

0.000 0.000 0.000 0.000 0.069 0.051 0.074 0.050 0.000 0.000

0.009 0.000 0.000 0.000 0.009 0.009 0.009 0.000 0.000 0.018

0.000 0.018 0.000 0.018 0.009 0.000 0.000 0.000 0.056 0.066

0.059 0.057

>Non-Methylation code 33

0.000 0.005 0.000 0.005 0.000 0.005 0.005 0.000 0.005 0.020

0.005 0.000 0.000 0.000 0.005 0.005 0.000 0.000 0.000 0.005

0.052 0.066 0.074 0.076 0.006 0.006 0.000 0.006 0.006 0.006

0.006 0.006 0.006 0.000 0.000 0.000 0.000 0.000 0.006 0.006

0.006 0.000 0.000 0.000 0.064 0.071 0.075 0.052 0.006 0.000

0.000 0.006 0.006 0.000 0.000 0.006 0.000 0.006 0.000 0.006

0.011 0.000 0.011 0.000 0.000 0.006 0.000 0.000 0.052 0.065

0.066 0.089

>Non-Methylation code 34

0.000 0.005 0.000 0.000 0.000 0.005 0.010 0.000 0.005 0.000

0.000 0.014 0.010 0.000 0.010 0.000 0.000 0.005 0.000 0.000

0.070 0.071 0.053 0.077 0.026 0.000 0.000 0.000 0.000 0.009

0.000 0.009 0.000 0.009 0.000 0.009 0.017 0.000 0.000 0.000

0.035 0.000 0.000 0.000 0.047 0.048 0.052 0.073 0.000 0.000

0.000 0.000 0.000 0.010 0.000 0.000 0.000 0.010 0.000 0.000

0.038 0.029 0.000 0.000 0.010 0.000 0.010 0.000 0.056 0.045

0.061 0.067

>Non-Methylation code 35

0.000 0.000 0.007 0.007 0.000 0.014 0.007 0.000 0.000 0.000

0.000 0.000 0.014 0.014 0.000 0.007 0.000 0.000 0.007 0.000

0.058 0.068 0.073 0.060 0.000 0.000 0.000 0.005 0.000 0.011

0.005 0.000 0.005 0.000 0.000 0.000 0.011 0.000 0.000 0.011

0.011 0.000 0.011 0.000 0.068 0.074 0.055 0.067 0.005 0.000

0.000 0.000 0.000 0.015 0.000 0.005 0.000 0.000 0.000 0.000

0.015 0.000 0.015 0.000 0.000 0.000 0.000 0.000 0.080 0.085

0.026 0.087

>Non-Methylation code 36

0.000 0.000 0.000 0.000 0.000 0.006 0.012 0.000 0.000 0.006

0.000 0.006 0.012 0.006 0.006 0.012 0.000 0.012 0.000 0.000

0.074 0.056 0.048 0.078 0.000 0.006 0.000 0.000 0.006 0.006

0.006 0.006 0.000 0.000 0.000 0.000 0.011 0.000 0.006 0.011

0.011 0.006 0.000 0.000 0.066 0.060 0.062 0.073 0.017 0.000

0.000 0.000 0.008 0.017 0.000 0.008 0.000 0.000 0.000 0.000

0.017 0.017 0.000 0.017 0.000 0.000 0.000 0.000 0.046 0.061

0.065 0.061

>Non-Methylation code 37

0.000 0.000 0.000 0.014 0.000 0.000 0.000 0.000 0.014 0.007

0.000 0.000 0.000 0.007 0.014 0.021 0.007 0.000 0.000 0.000

0.079 0.041 0.079 0.051 0.012 0.000 0.006 0.012 0.000 0.000

0.012 0.000 0.006 0.000 0.000 0.006 0.000 0.000 0.012 0.006

0.000 0.000 0.000 0.000 0.047 0.062 0.071 0.082 0.000 0.000

0.000 0.009 0.000 0.017 0.000 0.000 0.017 0.000 0.009 0.000

0.000 0.017 0.000 0.000 0.026 0.009 0.000 0.000 0.064 0.057

0.063 0.047

>Non-Methylation code 38

0.005 0.005 0.005 0.000 0.005 0.000 0.010 0.005 0.000 0.005

0.000 0.010 0.005 0.005 0.000 0.005 0.000 0.000 0.000 0.000

0.073 0.059 0.064 0.069 0.000 0.000 0.000 0.000 0.007 0.000

0.000 0.022 0.000 0.015 0.000 0.000 0.007 0.000 0.007 0.007

0.015 0.007 0.000 0.000 0.054 0.071 0.069 0.051 0.000 0.005

0.000 0.005 0.005 0.000 0.005 0.000 0.000 0.011 0.000 0.000

0.005 0.000 0.005 0.016 0.000 0.000 0.000 0.000 0.066 0.070

0.062 0.076

>Non-Methylation code 39

0.000 0.000 0.007 0.007 0.000 0.000 0.007 0.000 0.000 0.007

0.000 0.000 0.021 0.014 0.000 0.007 0.007 0.000 0.007 0.000

0.061 0.063 0.063 0.060 0.000 0.000 0.000 0.000 0.000 0.005

0.005 0.000 0.005 0.000 0.000 0.000 0.016 0.000 0.005 0.010

0.010 0.000 0.005 0.005 0.069 0.070 0.064 0.063 0.012 0.000

0.000 0.000 0.000 0.006 0.006 0.006 0.000 0.000 0.000 0.000

0.018 0.000 0.012 0.000 0.006 0.000 0.000 0.000 0.077 0.071

0.042 0.077

>Non-Methylation code 40

0.014 0.007 0.000 0.000 0.007 0.014 0.000 0.007 0.000 0.000

0.000 0.000 0.014 0.014 0.000 0.014 0.000 0.000 0.000 0.000

0.049 0.061 0.067 0.064 0.000 0.000 0.000 0.000 0.000 0.006

0.012 0.000 0.000 0.006 0.000 0.006 0.012 0.006 0.006 0.012

0.000 0.012 0.000 0.000 0.074 0.056 0.048 0.078 0.000 0.006

0.000 0.000 0.006 0.006 0.006 0.006 0.000 0.000 0.000 0.000

0.012 0.000 0.006 0.012 0.006 0.006 0.000 0.000 0.060 0.062

0.065 0.077

>Non-Methylation code 41

0.004 0.004 0.000 0.004 0.004 0.004 0.000 0.000 0.000 0.004

0.000 0.000 0.007 0.000 0.011 0.000 0.004 0.004 0.000 0.000

0.066 0.077 0.083 0.059 0.000 0.000 0.007 0.014 0.007 0.007

0.000 0.000 0.000 0.027 0.000 0.000 0.007 0.007 0.000 0.007

0.000 0.000 0.000 0.000 0.056 0.066 0.063 0.066 0.006 0.012

0.000 0.000 0.000 0.000 0.006 0.000 0.012 0.000 0.006 0.000

0.000 0.000 0.006 0.006 0.000 0.006 0.000 0.000 0.053 0.082

0.080 0.060

>Non-Methylation code 42

0.006 0.000 0.000 0.013 0.000 0.013 0.006 0.000 0.000 0.000

0.000 0.000 0.006 0.019 0.006 0.000 0.006 0.000 0.000 0.000

0.066 0.079 0.061 0.050 0.005 0.005 0.000 0.005 0.000 0.005

0.005 0.000 0.016 0.000 0.000 0.005 0.000 0.011 0.005 0.000

0.005 0.000 0.000 0.000 0.059 0.076 0.061 0.066 0.010 0.000

0.000 0.000 0.000 0.005 0.000 0.000 0.000 0.000 0.000 0.005

0.005 0.005 0.016 0.005 0.010 0.000 0.000 0.000 0.084 0.065

0.027 0.095

>Non-Methylation code 43

0.017 0.000 0.000 0.000 0.000 0.000 0.000 0.000 0.017 0.000

0.000 0.008 0.000 0.000 0.008 0.017 0.025 0.000 0.000 0.000

0.068 0.084 0.049 0.041 0.004 0.000 0.000 0.000 0.000 0.004

0.004 0.000 0.000 0.000 0.000 0.000 0.009 0.013 0.013 0.000

0.004 0.000 0.004 0.000 0.070 0.067 0.074 0.064 0.000 0.000

0.005 0.011 0.000 0.011 0.005 0.005 0.005 0.005 0.000 0.005

0.000 0.005 0.005 0.000 0.000 0.000 0.000 0.000 0.066 0.074

0.061 0.066

>Non-Methylation code 44

0.007 0.007 0.000 0.000 0.000 0.007 0.000 0.000 0.000 0.007

0.000 0.007 0.015 0.007 0.000 0.015 0.015 0.007 0.000 0.000

0.063 0.060 0.074 0.040 0.006 0.000 0.000 0.000 0.000 0.000

0.000 0.000 0.000 0.006 0.000 0.006 0.017 0.000 0.011 0.006

0.006 0.006 0.000 0.006 0.058 0.068 0.056 0.083 0.000 0.015

0.000 0.000 0.000 0.000 0.015 0.008 0.000 0.008 0.000 0.000

0.008 0.015 0.000 0.008 0.008 0.000 0.000 0.000 0.062 0.080

0.067 0.042

>Non-Methylation code 45

0.025 0.000 0.006 0.000 0.000 0.000 0.006 0.000 0.000 0.000

0.000 0.000 0.019 0.000 0.006 0.019 0.000 0.000 0.000 0.000

0.067 0.058 0.065 0.062 0.009 0.000 0.000 0.000 0.000 0.000

0.000 0.000 0.000 0.009 0.000 0.000 0.057 0.009 0.000 0.000

0.019 0.019 0.000 0.000 0.056 0.049 0.051 0.055 0.000 0.004

0.000 0.000 0.000 0.000 0.000 0.000 0.004 0.004 0.000 0.000

0.013 0.000 0.017 0.009 0.000 0.000 0.000 0.000 0.076 0.076

0.040 0.089

>Non-Methylation code 46

0.009 0.000 0.000 0.000 0.000 0.009 0.000 0.000 0.000 0.000

0.000 0.005 0.014 0.005 0.009 0.005 0.000 0.000 0.000 0.005

0.057 0.052 0.084 0.081 0.022 0.000 0.000 0.000 0.000 0.000

0.000 0.005 0.000 0.005 0.000 0.000 0.005 0.016 0.011 0.000

0.005 0.000 0.000 0.000 0.072 0.071 0.066 0.054 0.000 0.005

0.000 0.000 0.000 0.011 0.000 0.000 0.005 0.011 0.000 0.000

0.021 0.000 0.005 0.005 0.000 0.000 0.000 0.000 0.079 0.053

0.064 0.075

>Non-Methylation code 47

0.000 0.000 0.006 0.011 0.000 0.006 0.000 0.000 0.000 0.006

0.000 0.000 0.006 0.000 0.023 0.006 0.011 0.000 0.000 0.000

0.057 0.076 0.053 0.074 0.010 0.000 0.000 0.005 0.000 0.010

0.000 0.005 0.005 0.000 0.000 0.000 0.014 0.000 0.010 0.005

0.000 0.000 0.000 0.000 0.076 0.068 0.049 0.079 0.009 0.000

0.000 0.004 0.000 0.004 0.004 0.000 0.000 0.004 0.000 0.004

0.009 0.000 0.013 0.000 0.000 0.000 0.000 0.000 0.061 0.064

0.071 0.083

>Non-Methylation code 48

0.000 0.008 0.000 0.000 0.000 0.008 0.016 0.000 0.000 0.016

0.000 0.000 0.000 0.024 0.000 0.016 0.000 0.008 0.000 0.000

0.062 0.068 0.042 0.065 0.012 0.006 0.000 0.000 0.000 0.000

0.000 0.000 0.000 0.000 0.000 0.006 0.006 0.000 0.006 0.024

0.012 0.006 0.000 0.000 0.058 0.069 0.056 0.073 0.005 0.000

0.000 0.005 0.000 0.000 0.000 0.000 0.000 0.005 0.000 0.000

0.015 0.000 0.015 0.005 0.005 0.000 0.000 0.000 0.079 0.086

0.026 0.086

>Non-Methylation code 49

0.032 0.000 0.000 0.006 0.000 0.006 0.000 0.006 0.000 0.006

0.000 0.006 0.000 0.006 0.006 0.006 0.000 0.000 0.000 0.000

0.065 0.058 0.070 0.057 0.004 0.004 0.000 0.000 0.000 0.004

0.000 0.000 0.004 0.004 0.000 0.000 0.009 0.009 0.009 0.004

0.004 0.000 0.000 0.000 0.055 0.066 0.076 0.080 0.005 0.000

0.000 0.000 0.000 0.016 0.000 0.000 0.000 0.005 0.000 0.005

0.005 0.000 0.016 0.011 0.000 0.000 0.000 0.000 0.062 0.099

0.067 0.041

>Non-Methylation code 50

0.020 0.000 0.000 0.000 0.014 0.014 0.000 0.000 0.007 0.007

0.000 0.007 0.007 0.000 0.000 0.000 0.014 0.000 0.000 0.000

0.067 0.052 0.065 0.061 0.007 0.000 0.000 0.000 0.000 0.000

0.000 0.000 0.000 0.000 0.000 0.000 0.007 0.027 0.013 0.020

0.007 0.007 0.000 0.000 0.063 0.072 0.071 0.041 0.000 0.000

0.000 0.000 0.000 0.005 0.010 0.000 0.010 0.005 0.000 0.000

0.005 0.000 0.015 0.005 0.000 0.005 0.000 0.000 0.094 0.057

0.051 0.072

>Non-Methylation code 51

0.005 0.000 0.005 0.005 0.000 0.005 0.000 0.005 0.000 0.005

0.000 0.000 0.000 0.014 0.014 0.000 0.000 0.005 0.000 0.000

0.081 0.061 0.083 0.048 0.005 0.000 0.000 0.000 0.005 0.015

0.000 0.000 0.010 0.000 0.000 0.000 0.000 0.000 0.015 0.005

0.005 0.000 0.000 0.000 0.064 0.060 0.070 0.079 0.014 0.000

0.007 0.000 0.000 0.021 0.000 0.000 0.007 0.000 0.000 0.000

0.007 0.007 0.007 0.014 0.000 0.000 0.000 0.000 0.073 0.062

0.054 0.059

>Non-Methylation code 52

0.025 0.000 0.000 0.000 0.000 0.006 0.000 0.000 0.000 0.000

0.000 0.000 0.019 0.000 0.012 0.006 0.000 0.000 0.000 0.000

0.081 0.076 0.035 0.073 0.004 0.000 0.000 0.004 0.000 0.004

0.000 0.000 0.000 0.007 0.000 0.000 0.007 0.004 0.015 0.000

0.000 0.000 0.004 0.000 0.081 0.056 0.060 0.088 0.011 0.000

0.000 0.006 0.006 0.011 0.000 0.000 0.006 0.006 0.000 0.000

0.000 0.000 0.011 0.011 0.000 0.000 0.000 0.000 0.061 0.068

0.060 0.076

>Non-Methylation code 53

0.010 0.000 0.000 0.000 0.005 0.005 0.005 0.000 0.000 0.000

0.000 0.000 0.020 0.000 0.010 0.005 0.005 0.000 0.000 0.000

0.072 0.068 0.061 0.067 0.005 0.000 0.000 0.000 0.000 0.010

0.005 0.000 0.000 0.010 0.000 0.000 0.016 0.000 0.010 0.005

0.005 0.000 0.000 0.000 0.059 0.073 0.069 0.065 0.012 0.000

0.000 0.000 0.000 0.006 0.000 0.006 0.000 0.006 0.000 0.000

0.012 0.006 0.012 0.012 0.000 0.000 0.000 0.000 0.070 0.080

0.070 0.042

>Non-Methylation code 54

0.004 0.004 0.000 0.000 0.000 0.004 0.000 0.000 0.004 0.009

0.000 0.000 0.013 0.004 0.009 0.004 0.000 0.000 0.000 0.000

0.056 0.072 0.077 0.072 0.005 0.000 0.000 0.000 0.000 0.014

0.000 0.000 0.000 0.000 0.000 0.005 0.009 0.000 0.019 0.005

0.000 0.000 0.000 0.005 0.072 0.075 0.066 0.059 0.033 0.000

0.000 0.007 0.000 0.007 0.000 0.007 0.000 0.007 0.000 0.000

0.007 0.007 0.007 0.000 0.000 0.000 0.000 0.000 0.069 0.051

0.070 0.065

>Non-Methylation code 55

0.047 0.000 0.008 0.000 0.000 0.000 0.000 0.000 0.008 0.000

0.000 0.000 0.023 0.000 0.000 0.016 0.000 0.000 0.000 0.000

0.070 0.049 0.067 0.046 0.007 0.000 0.000 0.000 0.000 0.000

0.007 0.000 0.000 0.014 0.000 0.000 0.035 0.007 0.007 0.000

0.007 0.007 0.000 0.000 0.065 0.049 0.059 0.069 0.000 0.005

0.000 0.000 0.000 0.005 0.000 0.000 0.005 0.000 0.000 0.000

0.005 0.000 0.025 0.005 0.005 0.000 0.000 0.000 0.091 0.079

0.027 0.081

>Non-Methylation code 56

0.005 0.000 0.005 0.009 0.000 0.000 0.000 0.005 0.005 0.005

0.000 0.000 0.005 0.000 0.014 0.005 0.005 0.000 0.000 0.000

0.068 0.069 0.072 0.065 0.000 0.000 0.000 0.000 0.005 0.011

0.000 0.000 0.005 0.005 0.000 0.000 0.016 0.000 0.005 0.005

0.005 0.005 0.000 0.000 0.075 0.065 0.068 0.062 0.005 0.000

0.005 0.005 0.009 0.000 0.000 0.000 0.005 0.000 0.000 0.005

0.005 0.000 0.014 0.000 0.000 0.000 0.000 0.000 0.080 0.102

0.060 0.041

>Non-Methylation code 57

0.005 0.000 0.000 0.005 0.000 0.005 0.000 0.005 0.000 0.010

0.000 0.000 0.010 0.005 0.010 0.010 0.000 0.000 0.000 0.000

0.071 0.083 0.072 0.040 0.017 0.000 0.000 0.000 0.006 0.006

0.000 0.000 0.000 0.000 0.000 0.006 0.017 0.006 0.006 0.011

0.000 0.000 0.000 0.000 0.063 0.054 0.074 0.071 0.006 0.000

0.000 0.000 0.000 0.006 0.006 0.000 0.000 0.006 0.000 0.000

0.017 0.006 0.011 0.006 0.006 0.000 0.000 0.000 0.068 0.067

0.064 0.068

>Non-Methylation code 58

0.027 0.009 0.000 0.000 0.000 0.027 0.000 0.000 0.000 0.027

0.000 0.000 0.000 0.000 0.000 0.000 0.027 0.000 0.000 0.000

0.057 0.047 0.053 0.057 0.010 0.000 0.000 0.000 0.000 0.010

0.005 0.000 0.000 0.005 0.000 0.000 0.010 0.005 0.005 0.005

0.000 0.000 0.010 0.000 0.078 0.064 0.067 0.059 0.000 0.005

0.005 0.000 0.000 0.014 0.010 0.000 0.000 0.005 0.005 0.000

0.005 0.000 0.010 0.000 0.000 0.000 0.000 0.000 0.084 0.054

0.081 0.056

>Non-Methylation code 59

0.000 0.000 0.006 0.000 0.006 0.006 0.006 0.000 0.006 0.006

0.000 0.006 0.000 0.000 0.011 0.000 0.000 0.000 0.000 0.006

0.078 0.075 0.070 0.055 0.006 0.011 0.000 0.000 0.000 0.011

0.000 0.000 0.000 0.000 0.006 0.006 0.000 0.000 0.000 0.006

0.017 0.000 0.011 0.000 0.068 0.070 0.071 0.051 0.011 0.000

0.000 0.000 0.000 0.005 0.000 0.000 0.000 0.000 0.000 0.000

0.005 0.011 0.011 0.000 0.005 0.011 0.005 0.000 0.067 0.067

0.067 0.068

>Non-Methylation code 60

0.006 0.000 0.000 0.000 0.000 0.018 0.006 0.000 0.000 0.036

0.000 0.000 0.006 0.000 0.006 0.000 0.000 0.000 0.000 0.000

0.050 0.067 0.066 0.071 0.000 0.005 0.005 0.000 0.005 0.011

0.005 0.000 0.000 0.005 0.000 0.000 0.000 0.000 0.000 0.005

0.005 0.005 0.016 0.000 0.087 0.058 0.049 0.069 0.033 0.000

0.000 0.000 0.000 0.033 0.000 0.000 0.000 0.000 0.000 0.000

0.022 0.000 0.000 0.033 0.011 0.000 0.000 0.000 0.035 0.054

0.055 0.055

>Non-Methylation code 61

0.004 0.004 0.004 0.000 0.000 0.008 0.004 0.000 0.000 0.000

0.000 0.000 0.008 0.004 0.008 0.004 0.000 0.000 0.004 0.000

0.065 0.059 0.082 0.076 0.034 0.000 0.000 0.000 0.000 0.023

0.000 0.011 0.000 0.023 0.000 0.000 0.023 0.000 0.000 0.011

0.011 0.000 0.000 0.000 0.048 0.058 0.039 0.053 0.000 0.000

0.000 0.000 0.000 0.009 0.000 0.000 0.000 0.017 0.000 0.000

0.043 0.000 0.000 0.009 0.000 0.009 0.009 0.000 0.057 0.067

0.062 0.053

>Non-Methylation code 62

0.000 0.000 0.000 0.000 0.000 0.005 0.000 0.000 0.000 0.011

0.000 0.000 0.021 0.005 0.011 0.011 0.005 0.000 0.000 0.000

0.050 0.083 0.072 0.060 0.006 0.000 0.000 0.000 0.000 0.011

0.017 0.000 0.000 0.011 0.000 0.006 0.011 0.000 0.000 0.000

0.000 0.000 0.006 0.006 0.070 0.057 0.077 0.055 0.017 0.000

0.000 0.000 0.000 0.009 0.000 0.017 0.000 0.000 0.000 0.000

0.017 0.009 0.000 0.009 0.017 0.000 0.000 0.009 0.060 0.056

0.049 0.065

>Non-Methylation code 63

0.022 0.000 0.000 0.000 0.000 0.022 0.000 0.000 0.000 0.000

0.000 0.000 0.022 0.000 0.000 0.055 0.022 0.000 0.000 0.000

0.038 0.056 0.052 0.046 0.006 0.000 0.000 0.000 0.000 0.006

0.011 0.000 0.000 0.028 0.000 0.000 0.011 0.000 0.006 0.000

0.000 0.000 0.000 0.000 0.054 0.069 0.069 0.072 0.000 0.006

0.006 0.000 0.006 0.000 0.006 0.006 0.000 0.006 0.000 0.000

0.000 0.006 0.000 0.006 0.006 0.006 0.012 0.000 0.080 0.057

0.057 0.068

>Non-Methylation code 64

0.000 0.000 0.005 0.000 0.005 0.010 0.005 0.005 0.005 0.000

0.000 0.005 0.005 0.000 0.005 0.000 0.000 0.000 0.000 0.005

0.086 0.063 0.066 0.061 0.007 0.007 0.000 0.000 0.000 0.014

0.000 0.000 0.000 0.007 0.007 0.000 0.000 0.000 0.007 0.014

0.021 0.000 0.000 0.000 0.066 0.069 0.051 0.065 0.004 0.004

0.004 0.004 0.000 0.000 0.000 0.000 0.000 0.000 0.000 0.000

0.004 0.004 0.013 0.000 0.000 0.009 0.004 0.000 0.044 0.059

0.084 0.095

>Non-Methylation code 65

0.000 0.000 0.005 0.000 0.000 0.014 0.010 0.000 0.000 0.010

0.005 0.000 0.005 0.000 0.010 0.000 0.000 0.000 0.000 0.000

0.074 0.053 0.075 0.074 0.016 0.008 0.008 0.000 0.000 0.016

0.000 0.000 0.000 0.025 0.000 0.000 0.000 0.000 0.000 0.000

0.025 0.000 0.000 0.000 0.061 0.052 0.060 0.061 0.011 0.000

0.000 0.005 0.000 0.005 0.005 0.000 0.000 0.000 0.000 0.000

0.011 0.000 0.005 0.005 0.005 0.000 0.011 0.000 0.081 0.061

0.071 0.057

>Non-Methylation code 66

0.000 0.007 0.007 0.000 0.007 0.000 0.000 0.007 0.000 0.007

0.000 0.000 0.000 0.007 0.000 0.007 0.007 0.007 0.013 0.000

0.077 0.058 0.061 0.067 0.009 0.009 0.000 0.009 0.000 0.018

0.000 0.000 0.000 0.000 0.000 0.000 0.018 0.000 0.000 0.045

0.009 0.000 0.000 0.000 0.047 0.056 0.052 0.061 0.007 0.000

0.000 0.000 0.000 0.007 0.013 0.000 0.000 0.027 0.000 0.000

0.007 0.000 0.007 0.007 0.000 0.000 0.000 0.000 0.065 0.073

0.054 0.069

>Non-Methylation code 67

0.016 0.000 0.000 0.000 0.000 0.008 0.000 0.016 0.000 0.000

0.000 0.000 0.016 0.008 0.000 0.016 0.016 0.000 0.000 0.008

0.055 0.059 0.049 0.066 0.000 0.000 0.000 0.000 0.000 0.005

0.000 0.000 0.000 0.011 0.000 0.000 0.021 0.005 0.011 0.011

0.005 0.000 0.000 0.000 0.050 0.083 0.072 0.060 0.006 0.000

0.000 0.000 0.000 0.013 0.013 0.000 0.000 0.013 0.000 0.006

0.013 0.000 0.000 0.000 0.000 0.000 0.006 0.006 0.075 0.055

0.077 0.051

>Non-Methylation code 68

0.000 0.000 0.005 0.000 0.000 0.010 0.000 0.000 0.000 0.015

0.005 0.000 0.010 0.000 0.010 0.000 0.000 0.005 0.000 0.000

0.072 0.066 0.071 0.065 0.007 0.014 0.007 0.000 0.000 0.014

0.000 0.000 0.000 0.021 0.000 0.000 0.000 0.007 0.000 0.007

0.007 0.000 0.000 0.000 0.071 0.056 0.063 0.059 0.017 0.000

0.000 0.006 0.000 0.006 0.006 0.000 0.000 0.000 0.000 0.000

0.012 0.000 0.000 0.006 0.006 0.000 0.012 0.000 0.070 0.054

0.069 0.072

>Non-Methylation code 69

0.000 0.000 0.000 0.000 0.000 0.015 0.010 0.000 0.005 0.005

0.000 0.000 0.000 0.000 0.010 0.000 0.000 0.000 0.005 0.005

0.071 0.065 0.069 0.074 0.017 0.006 0.000 0.000 0.000 0.017

0.000 0.000 0.000 0.000 0.000 0.006 0.000 0.000 0.000 0.000

0.017 0.000 0.011 0.000 0.055 0.073 0.074 0.059 0.014 0.000

0.000 0.000 0.000 0.021 0.000 0.000 0.000 0.007 0.000 0.000

0.014 0.007 0.007 0.000 0.007 0.007 0.000 0.000 0.063 0.077

0.059 0.047

>Non-Methylation code 70

0.000 0.006 0.006 0.000 0.006 0.006 0.006 0.006 0.000 0.006

0.000 0.000 0.000 0.000 0.000 0.006 0.006 0.006 0.017 0.000

0.088 0.063 0.049 0.062 0.021 0.000 0.000 0.000 0.000 0.032

0.000 0.000 0.000 0.000 0.000 0.000 0.021 0.000 0.000 0.042

0.021 0.000 0.000 0.000 0.037 0.058 0.050 0.051 0.006 0.000

0.000 0.000 0.000 0.011 0.011 0.000 0.000 0.029 0.000 0.000

0.006 0.000 0.006 0.000 0.000 0.000 0.000 0.000 0.052 0.067

0.067 0.079

>Non-Methylation code 71

0.000 0.000 0.005 0.000 0.000 0.010 0.000 0.000 0.000 0.000

0.000 0.000 0.021 0.000 0.005 0.010 0.005 0.005 0.005 0.000

0.069 0.069 0.066 0.061 0.013 0.000 0.000 0.000 0.000 0.020

0.000 0.007 0.000 0.027 0.000 0.000 0.000 0.000 0.013 0.007

0.000 0.000 0.000 0.000 0.079 0.037 0.074 0.057 0.012 0.006

0.000 0.006 0.006 0.012 0.006 0.000 0.000 0.006 0.000 0.000

0.000 0.000 0.000 0.012 0.000 0.000 0.006 0.000 0.047 0.069

0.074 0.073

>Non-Methylation code 72

0.025 0.000 0.000 0.000 0.000 0.006 0.006 0.006 0.006 0.000

0.000 0.006 0.006 0.006 0.006 0.006 0.000 0.000 0.000 0.000

0.051 0.067 0.062 0.073 0.000 0.000 0.000 0.000 0.000 0.009

0.000 0.005 0.000 0.005 0.000 0.000 0.014 0.000 0.019 0.000

0.005 0.000 0.000 0.000 0.074 0.075 0.057 0.073 0.009 0.000

0.000 0.000 0.005 0.014 0.000 0.000 0.005 0.000 0.000 0.000

0.005 0.005 0.009 0.000 0.000 0.000 0.000 0.005 0.084 0.056

0.080 0.057

>Non-Methylation code 73

0.014 0.000 0.000 0.000 0.000 0.000 0.007 0.000 0.000 0.007

0.007 0.000 0.014 0.000 0.007 0.014 0.022 0.000 0.000 0.000

0.069 0.059 0.055 0.057 0.013 0.007 0.000 0.000 0.000 0.000

0.000 0.000 0.000 0.020 0.000 0.000 0.026 0.000 0.007 0.007

0.007 0.000 0.000 0.000 0.059 0.065 0.063 0.062 0.007 0.007

0.000 0.000 0.000 0.000 0.027 0.000 0.000 0.000 0.000 0.000

0.013 0.007 0.000 0.013 0.000 0.007 0.000 0.000 0.082 0.050

0.055 0.067

>Non-Methylation code 74

0.039 0.000 0.000 0.000 0.000 0.029 0.010 0.000 0.000 0.000

0.000 0.000 0.020 0.000 0.000 0.020 0.000 0.010 0.000 0.000

0.059 0.051 0.042 0.055 0.006 0.000 0.000 0.006 0.000 0.006

0.006 0.000 0.000 0.018 0.006 0.000 0.018 0.000 0.006 0.006

0.000 0.000 0.000 0.000 0.065 0.062 0.062 0.066 0.000 0.004

0.000 0.000 0.000 0.009 0.000 0.000 0.000 0.004 0.000 0.000

0.004 0.000 0.009 0.009 0.004 0.000 0.009 0.000 0.077 0.068

0.078 0.058

>Non-Methylation code 75

0.036 0.000 0.000 0.000 0.000 0.029 0.000 0.000 0.000 0.000

0.000 0.000 0.007 0.000 0.007 0.000 0.014 0.000 0.000 0.000

0.059 0.051 0.063 0.066 0.012 0.000 0.000 0.012 0.000 0.006

0.000 0.000 0.000 0.006 0.000 0.000 0.025 0.012 0.006 0.000

0.000 0.000 0.000 0.000 0.058 0.064 0.068 0.063 0.000 0.005

0.000 0.000 0.000 0.005 0.010 0.000 0.000 0.000 0.000 0.000

0.010 0.000 0.014 0.014 0.000 0.000 0.000 0.000 0.075 0.098

0.049 0.054

>Non-Methylation code 76

0.007 0.000 0.000 0.000 0.000 0.007 0.013 0.000 0.000 0.020

0.000 0.000 0.013 0.007 0.007 0.007 0.007 0.000 0.000 0.000

0.080 0.053 0.063 0.052 0.006 0.006 0.000 0.000 0.000 0.006

0.006 0.000 0.000 0.000 0.000 0.000 0.013 0.006 0.006 0.019

0.013 0.000 0.000 0.000 0.071 0.056 0.047 0.076 0.017 0.000

0.000 0.000 0.000 0.006 0.006 0.000 0.000 0.006 0.000 0.000

0.022 0.000 0.006 0.000 0.006 0.000 0.000 0.000 0.043 0.074

0.075 0.075

>Non-Methylation code 77

0.014 0.000 0.000 0.000 0.005 0.019 0.000 0.000 0.005 0.000

0.000 0.000 0.000 0.005 0.010 0.000 0.000 0.000 0.000 0.005

0.086 0.045 0.081 0.058 0.038 0.000 0.000 0.000 0.000 0.008

0.000 0.008 0.008 0.000 0.000 0.008 0.015 0.008 0.000 0.008

0.000 0.000 0.000 0.000 0.059 0.064 0.046 0.066 0.000 0.000

0.000 0.000 0.000 0.004 0.000 0.004 0.000 0.004 0.000 0.000

0.013 0.000 0.021 0.000 0.000 0.000 0.000 0.000 0.080 0.067

0.053 0.086

>Non-Methylation code 78

0.025 0.000 0.000 0.008 0.000 0.025 0.008 0.000 0.000 0.000

0.000 0.000 0.017 0.000 0.000 0.017 0.008 0.000 0.000 0.000

0.058 0.065 0.062 0.040 0.015 0.000 0.000 0.000 0.000 0.008

0.000 0.000 0.008 0.023 0.000 0.000 0.015 0.015 0.000 0.000

0.008 0.000 0.000 0.000 0.052 0.057 0.057 0.077 0.004 0.008

0.000 0.000 0.000 0.000 0.004 0.000 0.000 0.004 0.000 0.000

0.008 0.000 0.012 0.004 0.000 0.000 0.004 0.000 0.091 0.079

0.042 0.075

>Non-Methylation code 79

0.000 0.000 0.000 0.000 0.000 0.019 0.000 0.000 0.000 0.006

0.000 0.000 0.006 0.013 0.006 0.006 0.006 0.006 0.006 0.000

0.066 0.059 0.064 0.066 0.027 0.000 0.000 0.000 0.000 0.027

0.000 0.000 0.000 0.000 0.000 0.000 0.018 0.000 0.009 0.027

0.000 0.000 0.000 0.000 0.063 0.062 0.042 0.057 0.015 0.000

0.000 0.005 0.000 0.010 0.005 0.000 0.000 0.005 0.000 0.000

0.010 0.000 0.010 0.000 0.000 0.000 0.000 0.000 0.077 0.048

0.073 0.078

>Non-Methylation code 80

0.005 0.000 0.000 0.000 0.000 0.009 0.005 0.000 0.000 0.009

0.000 0.000 0.005 0.000 0.014 0.005 0.005 0.005 0.000 0.000

0.076 0.051 0.070 0.077 0.018 0.000 0.000 0.018 0.000 0.018

0.000 0.000 0.000 0.018 0.000 0.000 0.009 0.000 0.000 0.027

0.009 0.000 0.000 0.000 0.051 0.056 0.057 0.054 0.006 0.000

0.000 0.000 0.000 0.006 0.000 0.000 0.000 0.000 0.000 0.006

0.028 0.000 0.006 0.006 0.000 0.000 0.006 0.000 0.085 0.050

0.058 0.078

>Non-Methylation code 81

0.011 0.000 0.000 0.000 0.006 0.011 0.000 0.000 0.006 0.000

0.000 0.000 0.000 0.006 0.017 0.006 0.000 0.000 0.000 0.006

0.048 0.049 0.077 0.092 0.011 0.000 0.005 0.000 0.000 0.011

0.000 0.005 0.005 0.000 0.000 0.005 0.005 0.005 0.005 0.005

0.000 0.000 0.000 0.000 0.063 0.080 0.045 0.080 0.006 0.000

0.000 0.006 0.000 0.011 0.000 0.011 0.000 0.006 0.000 0.000

0.006 0.000 0.011 0.006 0.000 0.000 0.000 0.000 0.076 0.055

0.079 0.063

>Non-Methylation code 82

0.000 0.005 0.010 0.010 0.000 0.005 0.000 0.000 0.014 0.005

0.000 0.000 0.005 0.000 0.000 0.000 0.005 0.000 0.000 0.005

0.077 0.065 0.071 0.058 0.006 0.000 0.000 0.000 0.000 0.000

0.000 0.000 0.012 0.006 0.000 0.000 0.006 0.000 0.018 0.000

0.018 0.000 0.000 0.000 0.079 0.082 0.048 0.057 0.000 0.000

0.006 0.000 0.006 0.006 0.000 0.000 0.006 0.011 0.000 0.000

0.006 0.011 0.011 0.000 0.000 0.000 0.000 0.000 0.083 0.069

0.050 0.069

>Non-Methylation code 83

0.016 0.000 0.000 0.000 0.000 0.011 0.000 0.005 0.000 0.000

0.000 0.000 0.016 0.000 0.016 0.000 0.005 0.000 0.000 0.000

0.071 0.056 0.054 0.082 0.010 0.000 0.000 0.005 0.005 0.010

0.000 0.000 0.000 0.000 0.000 0.000 0.014 0.010 0.010 0.000

0.000 0.000 0.000 0.000 0.071 0.052 0.081 0.068 0.019 0.000

0.000 0.000 0.000 0.006 0.006 0.000 0.000 0.000 0.000 0.000

0.006 0.006 0.013 0.019 0.000 0.000 0.000 0.000 0.051 0.071

0.060 0.074

>Non-Methylation code 84

0.008 0.000 0.000 0.000 0.000 0.008 0.008 0.000 0.000 0.008

0.008 0.000 0.041 0.000 0.000 0.016 0.000 0.008 0.000 0.000

0.059 0.053 0.057 0.059 0.008 0.000 0.000 0.000 0.000 0.000

0.000 0.000 0.000 0.034 0.000 0.000 0.034 0.000 0.000 0.017

0.008 0.000 0.008 0.000 0.068 0.040 0.055 0.061 0.000 0.018

0.000 0.000 0.000 0.009 0.000 0.000 0.000 0.018 0.000 0.000

0.054 0.000 0.000 0.009 0.000 0.000 0.000 0.000 0.064 0.055

0.053 0.054

>Non-Methylation code 85

0.006 0.000 0.006 0.000 0.000 0.017 0.000 0.000 0.006 0.000

0.006 0.000 0.006 0.000 0.006 0.011 0.000 0.006 0.000 0.000

0.049 0.064 0.070 0.083 0.006 0.000 0.000 0.013 0.000 0.006

0.000 0.000 0.000 0.013 0.000 0.006 0.000 0.000 0.006 0.000

0.006 0.000 0.006 0.000 0.074 0.067 0.063 0.065 0.000 0.005

0.005 0.005 0.000 0.010 0.000 0.000 0.010 0.000 0.000 0.000

0.010 0.000 0.000 0.000 0.000 0.000 0.010 0.000 0.084 0.058

0.057 0.082

>Non-Methylation code 86

0.014 0.000 0.000 0.000 0.000 0.000 0.005 0.000 0.000 0.000

0.005 0.000 0.023 0.000 0.014 0.000 0.000 0.000 0.000 0.000

0.092 0.068 0.069 0.045 0.011 0.005 0.000 0.000 0.000 0.000

0.005 0.000 0.000 0.000 0.005 0.000 0.026 0.000 0.011 0.000

0.000 0.005 0.000 0.000 0.063 0.069 0.080 0.053 0.011 0.005

0.000 0.000 0.000 0.000 0.005 0.000 0.000 0.000 0.000 0.000

0.022 0.000 0.011 0.005 0.005 0.000 0.000 0.000 0.079 0.077

0.074 0.038

>Non-Methylation code 87

0.000 0.006 0.000 0.000 0.000 0.017 0.006 0.000 0.000 0.006

0.000 0.006 0.012 0.000 0.006 0.000 0.012 0.006 0.000 0.000

0.079 0.056 0.061 0.062 0.000 0.008 0.000 0.000 0.000 0.033

0.016 0.000 0.000 0.000 0.000 0.000 0.008 0.008 0.000 0.008

0.016 0.000 0.000 0.000 0.069 0.056 0.060 0.052 0.010 0.000

0.005 0.000 0.000 0.005 0.005 0.005 0.005 0.000 0.000 0.000

0.000 0.000 0.010 0.000 0.005 0.000 0.005 0.000 0.069 0.071

0.073 0.067

>Non-Methylation code 88

0.000 0.006 0.000 0.000 0.000 0.000 0.000 0.000 0.000 0.006

0.000 0.000 0.033 0.000 0.011 0.017 0.000 0.000 0.000 0.000

0.072 0.069 0.038 0.082 0.012 0.000 0.000 0.000 0.000 0.006

0.000 0.000 0.000 0.006 0.000 0.000 0.044 0.000 0.006 0.006

0.000 0.000 0.000 0.000 0.064 0.059 0.054 0.075 0.019 0.000

0.000 0.000 0.000 0.000 0.000 0.000 0.000 0.029 0.000 0.000

0.058 0.000 0.000 0.010 0.000 0.000 0.000 0.000 0.054 0.049

0.052 0.063

>Non-Methylation code 89

0.008 0.000 0.000 0.000 0.000 0.000 0.000 0.000 0.000 0.034

0.000 0.000 0.034 0.000 0.000 0.017 0.008 0.000 0.008 0.000

0.068 0.040 0.055 0.061 0.000 0.016 0.000 0.000 0.000 0.008

0.000 0.000 0.000 0.016 0.000 0.000 0.048 0.000 0.000 0.016

0.000 0.000 0.000 0.000 0.064 0.053 0.055 0.056 0.009 0.000

0.000 0.000 0.000 0.009 0.009 0.000 0.000 0.009 0.000 0.000

0.043 0.000 0.000 0.017 0.000 0.009 0.000 0.000 0.058 0.050

0.058 0.063

>Non-Methylation code 90

0.013 0.000 0.000 0.000 0.000 0.004 0.000 0.000 0.004 0.004

0.000 0.000 0.009 0.000 0.013 0.000 0.000 0.000 0.009 0.000

0.066 0.087 0.054 0.069 0.013 0.000 0.000 0.000 0.000 0.026

0.000 0.000 0.000 0.013 0.000 0.000 0.019 0.000 0.006 0.006

0.000 0.000 0.000 0.000 0.058 0.057 0.060 0.075 0.021 0.005

0.000 0.005 0.000 0.005 0.000 0.000 0.000 0.010 0.000 0.000

0.005 0.000 0.005 0.000 0.000 0.000 0.005 0.000 0.072 0.069

0.065 0.065

>Non-Methylation code 91

0.022 0.000 0.000 0.000 0.000 0.015 0.000 0.000 0.000 0.007

0.007 0.000 0.022 0.000 0.007 0.007 0.007 0.000 0.000 0.000

0.077 0.047 0.056 0.056 0.015 0.000 0.000 0.000 0.000 0.005

0.000 0.000 0.000 0.000 0.000 0.000 0.015 0.010 0.010 0.000

0.000 0.000 0.005 0.000 0.071 0.069 0.058 0.074 0.013 0.000

0.006 0.000 0.000 0.006 0.000 0.000 0.000 0.006 0.000 0.006

0.013 0.006 0.006 0.013 0.000 0.000 0.000 0.000 0.071 0.054

0.075 0.058

>Non-Methylation code 92

0.014 0.007 0.007 0.000 0.000 0.007 0.000 0.000 0.000 0.007

0.007 0.000 0.000 0.000 0.007 0.014 0.014 0.000 0.000 0.000

0.065 0.063 0.063 0.056 0.010 0.005 0.000 0.000 0.000 0.000

0.000 0.000 0.000 0.000 0.000 0.000 0.005 0.010 0.010 0.000

0.005 0.000 0.005 0.000 0.070 0.079 0.061 0.073 0.007 0.007

0.007 0.000 0.000 0.007 0.007 0.000 0.000 0.015 0.000 0.007

0.000 0.000 0.000 0.015 0.000 0.007 0.000 0.000 0.069 0.059

0.068 0.057

>Non-Methylation code 93

0.017 0.000 0.000 0.000 0.000 0.006 0.000 0.000 0.000 0.000

0.000 0.000 0.028 0.006 0.011 0.000 0.000 0.000 0.000 0.000

0.081 0.080 0.066 0.039 0.016 0.000 0.000 0.000 0.000 0.000

0.000 0.000 0.000 0.005 0.000 0.005 0.022 0.005 0.011 0.005

0.000 0.000 0.000 0.000 0.076 0.069 0.084 0.033 0.012 0.000

0.000 0.000 0.000 0.006 0.000 0.000 0.000 0.006 0.000 0.000

0.031 0.000 0.012 0.006 0.000 0.000 0.000 0.000 0.071 0.074

0.077 0.037

>Non-Methylation code 94

0.006 0.000 0.000 0.013 0.000 0.006 0.000 0.000 0.000 0.013

0.000 0.006 0.000 0.000 0.006 0.000 0.006 0.000 0.006 0.000

0.074 0.067 0.063 0.065 0.000 0.005 0.005 0.005 0.000 0.009

0.000 0.000 0.009 0.000 0.000 0.000 0.009 0.000 0.000 0.000

0.005 0.000 0.009 0.000 0.083 0.061 0.055 0.080 0.006 0.000

0.006 0.000 0.000 0.018 0.000 0.000 0.006 0.000 0.006 0.000

0.006 0.000 0.006 0.012 0.000 0.000 0.000 0.000 0.053 0.068

0.075 0.070

>Non-Methylation code 95

0.011 0.000 0.000 0.000 0.000 0.000 0.000 0.000 0.000 0.000

0.000 0.000 0.042 0.000 0.016 0.000 0.000 0.000 0.000 0.000

0.084 0.082 0.087 0.012 0.004 0.000 0.000 0.000 0.000 0.004

0.000 0.000 0.000 0.000 0.000 0.000 0.025 0.000 0.017 0.000

0.000 0.004 0.000 0.000 0.074 0.097 0.064 0.044 0.044 0.000

0.000 0.000 0.000 0.011 0.000 0.000 0.000 0.000 0.000 0.000

0.066 0.000 0.000 0.011 0.000 0.000 0.000 0.000 0.061 0.041

0.050 0.048

>Non-Methylation code 96

0.022 0.000 0.007 0.000 0.000 0.014 0.000 0.007 0.007 0.007

0.000 0.000 0.029 0.000 0.000 0.000 0.000 0.000 0.000 0.000

0.060 0.062 0.057 0.060 0.000 0.000 0.000 0.000 0.000 0.010

0.000 0.000 0.000 0.000 0.000 0.000 0.024 0.000 0.015 0.010

0.005 0.000 0.000 0.000 0.078 0.056 0.069 0.067 0.008 0.000

0.000 0.000 0.000 0.008 0.004 0.000 0.000 0.000 0.000 0.000

0.008 0.004 0.017 0.000 0.000 0.000 0.000 0.000 0.077 0.060

0.068 0.079

>Non-Methylation code 97

0.009 0.000 0.000 0.000 0.000 0.009 0.000 0.000 0.000 0.000

0.000 0.000 0.028 0.000 0.014 0.000 0.000 0.000 0.000 0.000

0.078 0.077 0.053 0.064 0.016 0.000 0.000 0.000 0.000 0.010

0.000 0.000 0.000 0.000 0.000 0.000 0.031 0.000 0.010 0.000

0.000 0.000 0.000 0.000 0.069 0.061 0.063 0.073 0.015 0.000

0.000 0.000 0.000 0.005 0.000 0.000 0.000 0.000 0.000 0.000

0.025 0.000 0.015 0.000 0.000 0.000 0.000 0.000 0.087 0.063

0.050 0.074

>Non-Methylation code 98

0.019 0.000 0.000 0.000 0.006 0.019 0.000 0.000 0.000 0.006

0.000 0.006 0.000 0.000 0.006 0.000 0.000 0.000 0.006 0.000

0.069 0.064 0.057 0.074 0.024 0.006 0.000 0.000 0.000 0.006

0.000 0.000 0.000 0.000 0.000 0.000 0.018 0.000 0.000 0.006

0.006 0.000 0.012 0.000 0.071 0.052 0.071 0.060 0.005 0.000

0.000 0.000 0.000 0.010 0.000 0.000 0.000 0.010 0.000 0.000

0.000 0.005 0.024 0.000 0.000 0.000 0.000 0.000 0.065 0.079

0.062 0.073

>Non-Methylation code 99

0.013 0.000 0.008 0.000 0.000 0.004 0.000 0.000 0.000 0.000

0.000 0.000 0.008 0.000 0.017 0.000 0.004 0.000 0.000 0.000

0.066 0.086 0.089 0.039 0.005 0.000 0.000 0.005 0.000 0.010

0.000 0.000 0.000 0.005 0.000 0.000 0.015 0.000 0.010 0.000

0.010 0.000 0.000 0.000 0.062 0.073 0.068 0.071 0.017 0.004

0.000 0.000 0.000 0.000 0.004 0.000 0.000 0.000 0.000 0.000

0.008 0.000 0.013 0.000 0.000 0.000 0.004 0.000 0.061 0.072

0.062 0.087

>Non-Methylation code 100

0.039 0.000 0.000 0.000 0.000 0.023 0.000 0.000 0.000 0.015

0.000 0.000 0.015 0.000 0.008 0.000 0.000 0.000 0.000 0.000

0.066 0.062 0.047 0.058 0.008 0.000 0.000 0.000 0.000 0.008

0.000 0.000 0.000 0.000 0.000 0.000 0.051 0.000 0.008 0.017

0.000 0.000 0.000 0.000 0.062 0.055 0.055 0.068 0.000 0.004

0.000 0.000 0.000 0.008 0.000 0.000 0.000 0.000 0.000 0.000

0.016 0.000 0.020 0.000 0.000 0.000 0.000 0.000 0.087 0.058

0.069 0.070

>Non-Methylation code 101

0.008 0.004 0.000 0.000 0.000 0.004 0.000 0.000 0.000 0.000

0.000 0.000 0.004 0.000 0.015 0.008 0.004 0.000 0.004 0.000

0.082 0.049 0.060 0.093 0.011 0.000 0.011 0.000 0.000 0.011

0.011 0.000 0.000 0.011 0.000 0.000 0.005 0.000 0.005 0.000

0.000 0.005 0.000 0.000 0.071 0.060 0.063 0.071 0.000 0.000

0.005 0.005 0.005 0.000 0.000 0.005 0.000 0.000 0.005 0.000

0.000 0.005 0.010 0.000 0.010 0.000 0.005 0.000 0.058 0.066

0.077 0.075

>Non-Methylation code 102

0.010 0.005 0.005 0.000 0.000 0.005 0.000 0.000 0.000 0.000

0.000 0.000 0.025 0.000 0.010 0.000 0.000 0.000 0.000 0.000

0.033 0.067 0.083 0.090 0.019 0.000 0.000 0.005 0.000 0.000

0.000 0.000 0.000 0.005 0.000 0.000 0.014 0.000 0.014 0.000

0.005 0.000 0.000 0.000 0.071 0.083 0.075 0.045 0.008 0.000

0.000 0.000 0.000 0.000 0.000 0.000 0.000 0.000 0.000 0.000

0.046 0.000 0.023 0.000 0.000 0.000 0.000 0.000 0.028 0.054

0.081 0.094

>Non-Methylation code 103

0.008 0.008 0.000 0.000 0.008 0.024 0.000 0.008 0.000 0.008

0.008 0.008 0.000 0.000 0.000 0.008 0.000 0.000 0.000 0.000

0.061 0.068 0.056 0.061 0.019 0.005 0.000 0.000 0.000 0.000

0.000 0.000 0.000 0.000 0.000 0.000 0.005 0.000 0.005 0.009

0.005 0.000 0.014 0.000 0.090 0.056 0.076 0.051 0.000 0.000

0.000 0.000 0.000 0.015 0.005 0.000 0.000 0.010 0.000 0.000

0.000 0.005 0.015 0.000 0.000 0.005 0.000 0.000 0.070 0.086

0.067 0.055

>Non-Methylation code 104

0.021 0.000 0.000 0.000 0.000 0.000 0.000 0.000 0.000 0.000

0.000 0.000 0.193 0.000 0.000 0.000 0.000 0.000 0.000 0.000

0.024 0.027 0.031 0.036 0.000 0.007 0.004 0.000 0.000 0.000

0.000 0.000 0.000 0.000 0.000 0.000 0.018 0.000 0.018 0.000

0.000 0.000 0.000 0.000 0.061 0.082 0.065 0.078 0.035 0.000

0.000 0.000 0.000 0.014 0.000 0.000 0.000 0.007 0.000 0.000

0.014 0.000 0.007 0.000 0.007 0.000 0.000 0.000 0.049 0.056

0.068 0.077

>Non-Methylation code 105

0.009 0.000 0.000 0.000 0.000 0.004 0.000 0.000 0.000 0.000

0.000 0.000 0.013 0.004 0.022 0.000 0.000 0.000 0.000 0.000

0.080 0.066 0.052 0.084 0.019 0.000 0.000 0.000 0.000 0.005

0.005 0.000 0.000 0.000 0.000 0.005 0.009 0.000 0.009 0.000

0.000 0.000 0.005 0.000 0.080 0.077 0.047 0.072 0.021 0.007

0.000 0.000 0.000 0.007 0.000 0.000 0.000 0.000 0.000 0.000

0.028 0.000 0.007 0.000 0.014 0.000 0.000 0.000 0.072 0.084

0.038 0.054

>Non-Methylation code 106

0.017 0.000 0.000 0.000 0.000 0.000 0.000 0.000 0.000 0.000

0.000 0.000 0.140 0.000 0.000 0.017 0.000 0.000 0.000 0.000

0.035 0.040 0.045 0.038 0.000 0.007 0.004 0.000 0.000 0.000

0.000 0.000 0.000 0.000 0.000 0.000 0.018 0.000 0.018 0.000

0.000 0.000 0.000 0.000 0.068 0.071 0.063 0.084 0.035 0.000

0.000 0.000 0.000 0.014 0.000 0.000 0.000 0.007 0.000 0.000

0.014 0.000 0.007 0.000 0.007 0.000 0.000 0.000 0.051 0.056

0.066 0.077

>Non-Methylation code 107

0.000 0.000 0.000 0.013 0.000 0.004 0.004 0.000 0.000 0.004

0.000 0.000 0.000 0.000 0.022 0.000 0.000 0.004 0.000 0.000

0.065 0.060 0.073 0.083 0.000 0.000 0.007 0.007 0.000 0.020

0.000 0.007 0.013 0.000 0.000 0.007 0.000 0.000 0.000 0.013

0.000 0.000 0.000 0.007 0.048 0.072 0.069 0.065 0.017 0.006

0.000 0.000 0.000 0.000 0.000 0.006 0.000 0.000 0.000 0.000

0.006 0.000 0.017 0.006 0.012 0.000 0.000 0.000 0.033 0.057

0.078 0.095

>Non-Methylation code 108

0.004 0.000 0.000 0.004 0.000 0.000 0.000 0.000 0.000 0.004

0.000 0.000 0.009 0.004 0.013 0.000 0.009 0.000 0.004 0.000

0.060 0.068 0.076 0.077 0.010 0.003 0.000 0.000 0.000 0.003

0.000 0.000 0.000 0.000 0.000 0.000 0.007 0.000 0.016 0.000

0.000 0.000 0.003 0.000 0.078 0.062 0.053 0.098 0.020 0.000

0.010 0.000 0.000 0.005 0.000 0.000 0.000 0.005 0.000 0.000

0.010 0.000 0.010 0.000 0.000 0.000 0.000 0.000 0.079 0.072

0.072 0.050

>Non-Methylation code 109

0.012 0.000 0.006 0.000 0.000 0.000 0.000 0.006 0.000 0.006

0.000 0.000 0.012 0.000 0.006 0.012 0.000 0.000 0.012 0.000

0.064 0.075 0.057 0.068 0.005 0.009 0.000 0.000 0.000 0.009

0.000 0.000 0.000 0.005 0.000 0.000 0.000 0.005 0.014 0.000

0.005 0.000 0.000 0.000 0.069 0.069 0.048 0.096 0.014 0.005

0.005 0.000 0.000 0.000 0.000 0.000 0.005 0.005 0.000 0.000

0.005 0.000 0.009 0.000 0.000 0.005 0.000 0.000 0.073 0.063

0.063 0.085

>Non-Methylation code 110

0.011 0.000 0.000 0.000 0.000 0.053 0.000 0.000 0.000 0.021

0.000 0.000 0.032 0.000 0.000 0.000 0.011 0.000 0.000 0.000

0.043 0.048 0.057 0.058 0.017 0.004 0.000 0.000 0.000 0.004

0.004 0.000 0.000 0.000 0.000 0.000 0.009 0.000 0.009 0.000

0.000 0.000 0.009 0.000 0.060 0.070 0.073 0.073 0.009 0.000

0.004 0.000 0.000 0.009 0.000 0.000 0.000 0.000 0.000 0.000

0.009 0.000 0.017 0.000 0.004 0.000 0.000 0.000 0.057 0.074

0.069 0.082

>Non-Methylation code 111

0.026 0.013 0.007 0.000 0.000 0.000 0.007 0.000 0.000 0.007

0.000 0.000 0.026 0.000 0.000 0.000 0.000 0.000 0.000 0.000

0.057 0.063 0.066 0.062 0.005 0.000 0.000 0.000 0.000 0.000

0.000 0.000 0.000 0.005 0.000 0.000 0.020 0.000 0.015 0.005

0.010 0.000 0.000 0.000 0.042 0.071 0.080 0.081 0.008 0.000

0.000 0.000 0.000 0.000 0.000 0.000 0.000 0.004 0.000 0.000

0.013 0.000 0.017 0.004 0.000 0.000 0.000 0.000 0.043 0.071

0.092 0.081

>Non-Methylation code 112

0.000 0.000 0.000 0.000 0.000 0.004 0.000 0.000 0.000 0.004

0.000 0.000 0.021 0.000 0.017 0.004 0.000 0.004 0.000 0.000

0.070 0.093 0.066 0.050 0.030 0.000 0.000 0.000 0.000 0.008

0.000 0.000 0.000 0.000 0.000 0.000 0.038 0.000 0.008 0.015

0.000 0.000 0.000 0.000 0.067 0.048 0.055 0.065 0.006 0.000

0.000 0.000 0.000 0.000 0.000 0.000 0.000 0.006 0.000 0.000

0.048 0.000 0.012 0.000 0.000 0.000 0.000 0.000 0.069 0.079

0.084 0.030

>Non-Methylation code 113

0.000 0.006 0.006 0.000 0.000 0.019 0.000 0.006 0.013 0.000

0.000 0.000 0.006 0.006 0.000 0.013 0.000 0.000 0.000 0.000

0.053 0.075 0.069 0.060 0.013 0.004 0.000 0.000 0.000 0.000

0.000 0.000 0.000 0.000 0.000 0.000 0.009 0.000 0.013 0.000

0.004 0.009 0.000 0.004 0.040 0.069 0.082 0.085 0.000 0.000

0.000 0.010 0.000 0.005 0.005 0.000 0.000 0.000 0.005 0.000

0.005 0.000 0.015 0.005 0.000 0.005 0.000 0.000 0.067 0.064

0.056 0.090

>Non-Methylation code 114

0.043 0.000 0.000 0.000 0.000 0.026 0.000 0.000 0.000 0.017

0.000 0.000 0.009 0.000 0.009 0.000 0.000 0.009 0.000 0.000

0.070 0.048 0.045 0.059 0.012 0.000 0.000 0.000 0.000 0.012

0.000 0.000 0.000 0.000 0.000 0.000 0.071 0.000 0.000 0.035

0.000 0.000 0.000 0.000 0.054 0.053 0.025 0.072 0.000 0.008

0.000 0.000 0.000 0.004 0.000 0.000 0.000 0.000 0.000 0.000

0.015 0.000 0.019 0.000 0.000 0.000 0.000 0.000 0.094 0.060

0.062 0.072

>Non-Methylation code 115

0.044 0.000 0.000 0.000 0.000 0.026 0.000 0.000 0.000 0.009

0.000 0.000 0.018 0.000 0.009 0.000 0.009 0.000 0.000 0.000

0.050 0.057 0.057 0.054 0.016 0.000 0.000 0.000 0.000 0.000

0.000 0.000 0.000 0.000 0.000 0.000 0.047 0.008 0.008 0.000

0.000 0.000 0.000 0.000 0.066 0.071 0.056 0.062 0.000 0.009

0.004 0.000 0.000 0.004 0.000 0.000 0.000 0.000 0.000 0.000

0.017 0.000 0.013 0.004 0.000 0.000 0.000 0.000 0.062 0.090

0.069 0.060

>Non-Methylation code 116

0.011 0.000 0.000 0.000 0.000 0.000 0.000 0.000 0.000 0.006

0.000 0.000 0.046 0.000 0.011 0.000 0.000 0.000 0.000 0.000

0.067 0.078 0.083 0.032 0.000 0.000 0.000 0.000 0.000 0.004

0.000 0.000 0.000 0.004 0.000 0.000 0.021 0.000 0.017 0.004

0.000 0.004 0.000 0.000 0.070 0.093 0.066 0.050 0.042 0.000

0.000 0.000 0.000 0.011 0.000 0.000 0.000 0.000 0.000 0.000

0.053 0.000 0.000 0.021 0.000 0.000 0.000 0.000 0.068 0.037

0.045 0.058

>Non-Methylation code 117

0.000 0.000 0.004 0.000 0.000 0.012 0.000 0.000 0.000 0.000

0.000 0.000 0.012 0.000 0.020 0.000 0.004 0.000 0.000 0.000

0.064 0.069 0.077 0.072 0.022 0.000 0.000 0.000 0.000 0.056

0.000 0.000 0.000 0.022 0.000 0.000 0.022 0.000 0.000 0.000

0.011 0.000 0.000 0.000 0.046 0.047 0.050 0.056 0.021 0.000

0.000 0.000 0.000 0.005 0.005 0.000 0.000 0.000 0.000 0.000

0.016 0.000 0.005 0.000 0.000 0.000 0.010 0.000 0.080 0.056

0.077 0.059

>Non-Methylation code 118

0.008 0.000 0.000 0.000 0.000 0.032 0.000 0.000 0.000 0.016

0.000 0.000 0.024 0.000 0.008 0.000 0.008 0.000 0.000 0.000

0.049 0.059 0.066 0.064 0.016 0.004 0.000 0.000 0.000 0.004

0.004 0.000 0.000 0.000 0.000 0.000 0.008 0.000 0.008 0.000

0.000 0.000 0.008 0.000 0.062 0.079 0.069 0.070 0.013 0.000

0.004 0.000 0.000 0.009 0.000 0.000 0.000 0.000 0.000 0.000

0.004 0.000 0.018 0.000 0.004 0.000 0.000 0.000 0.056 0.072

0.081 0.071

>Non-Methylation code 119

0.010 0.005 0.005 0.000 0.000 0.005 0.000 0.000 0.000 0.000

0.000 0.000 0.019 0.000 0.010 0.005 0.000 0.000 0.000 0.000

0.066 0.066 0.060 0.085 0.021 0.000 0.000 0.000 0.000 0.014

0.000 0.000 0.000 0.000 0.000 0.000 0.028 0.007 0.007 0.000

0.007 0.000 0.000 0.000 0.068 0.076 0.042 0.062 0.000 0.000

0.000 0.005 0.000 0.005 0.000 0.000 0.000 0.000 0.000 0.000

0.019 0.005 0.019 0.000 0.000 0.000 0.000 0.000 0.085 0.058

0.055 0.082

>Non-Methylation code 120

0.012 0.000 0.008 0.000 0.000 0.004 0.000 0.000 0.000 0.000

0.000 0.000 0.004 0.000 0.020 0.000 0.004 0.000 0.000 0.000

0.064 0.081 0.079 0.055 0.014 0.000 0.000 0.000 0.000 0.021

0.000 0.007 0.000 0.007 0.000 0.000 0.021 0.000 0.007 0.000

0.007 0.000 0.000 0.000 0.059 0.069 0.062 0.059 0.017 0.004

0.000 0.000 0.000 0.000 0.004 0.000 0.000 0.000 0.000 0.000

0.008 0.000 0.013 0.000 0.000 0.000 0.004 0.000 0.061 0.072

0.062 0.087

>Non-Methylation code 121

0.000 0.000 0.004 0.000 0.000 0.017 0.000 0.000 0.000 0.000

0.000 0.000 0.012 0.004 0.012 0.000 0.004 0.000 0.000 0.000

0.068 0.054 0.063 0.095 0.013 0.000 0.006 0.000 0.000 0.026

0.000 0.000 0.006 0.006 0.000 0.000 0.013 0.000 0.006 0.000

0.006 0.000 0.000 0.000 0.076 0.033 0.075 0.066 0.014 0.000

0.000 0.000 0.000 0.009 0.005 0.000 0.000 0.000 0.000 0.000

0.009 0.000 0.009 0.000 0.005 0.000 0.005 0.000 0.080 0.067

0.067 0.064

>Non-Methylation code 122

0.000 0.000 0.004 0.009 0.000 0.004 0.004 0.000 0.000 0.000

0.004 0.000 0.004 0.000 0.017 0.000 0.000 0.004 0.000 0.000

0.070 0.065 0.050 0.096 0.000 0.007 0.007 0.000 0.000 0.020

0.000 0.014 0.014 0.000 0.000 0.000 0.000 0.007 0.000 0.014

0.000 0.000 0.000 0.000 0.055 0.067 0.069 0.060 0.017 0.006

0.000 0.000 0.000 0.000 0.000 0.000 0.000 0.000 0.000 0.000

0.006 0.000 0.017 0.006 0.006 0.006 0.000 0.006 0.036 0.062

0.075 0.094

>Non-Methylation code 123

0.000 0.005 0.000 0.000 0.000 0.009 0.005 0.000 0.000 0.014

0.000 0.000 0.005 0.000 0.009 0.000 0.009 0.005 0.000 0.000

0.072 0.060 0.074 0.067 0.007 0.021 0.000 0.000 0.000 0.021

0.007 0.000 0.000 0.000 0.000 0.000 0.007 0.007 0.000 0.007

0.007 0.007 0.000 0.000 0.055 0.062 0.059 0.066 0.017 0.000

0.000 0.000 0.000 0.011 0.011 0.006 0.006 0.000 0.000 0.000

0.006 0.000 0.006 0.000 0.000 0.000 0.000 0.000 0.075 0.058

0.044 0.095

>Non-Methylation code 124

0.017 0.000 0.000 0.000 0.000 0.006 0.000 0.000 0.000 0.006

0.000 0.000 0.023 0.011 0.006 0.006 0.000 0.000 0.000 0.000

0.064 0.061 0.066 0.068 0.015 0.000 0.000 0.000 0.000 0.000

0.000 0.000 0.000 0.000 0.000 0.000 0.060 0.000 0.008 0.015

0.000 0.000 0.000 0.000 0.060 0.064 0.049 0.062 0.005 0.000

0.000 0.000 0.000 0.005 0.000 0.000 0.000 0.000 0.000 0.000

0.038 0.000 0.016 0.000 0.000 0.000 0.000 0.000 0.072 0.069

0.042 0.085

>Non-Methylation code 125

0.025 0.000 0.006 0.006 0.000 0.006 0.000 0.000 0.000 0.000

0.000 0.000 0.025 0.000 0.006 0.000 0.006 0.000 0.000 0.000

0.069 0.058 0.064 0.060 0.000 0.000 0.006 0.006 0.000 0.000

0.000 0.000 0.000 0.006 0.000 0.000 0.036 0.006 0.012 0.000

0.006 0.000 0.000 0.000 0.058 0.067 0.066 0.066 0.000 0.000

0.000 0.000 0.000 0.005 0.000 0.000 0.000 0.000 0.000 0.005

0.016 0.000 0.021 0.005 0.005 0.000 0.000 0.000 0.084 0.049

0.072 0.069

>Non-Methylation code 126

0.000 0.012 0.006 0.000 0.012 0.006 0.000 0.006 0.000 0.000

0.012 0.000 0.000 0.006 0.000 0.006 0.000 0.006 0.000 0.000

0.067 0.066 0.070 0.056 0.005 0.005 0.000 0.000 0.000 0.000

0.000 0.000 0.000 0.000 0.000 0.000 0.000 0.000 0.010 0.019

0.005 0.000 0.010 0.000 0.074 0.078 0.065 0.064 0.000 0.012

0.006 0.000 0.000 0.012 0.012 0.000 0.000 0.012 0.000 0.000

0.006 0.000 0.000 0.000 0.000 0.006 0.000 0.000 0.053 0.075

0.063 0.074

>Non-Methylation code 127

0.005 0.000 0.000 0.000 0.000 0.010 0.005 0.000 0.000 0.015

0.000 0.000 0.005 0.000 0.010 0.005 0.000 0.005 0.005 0.000

0.072 0.064 0.059 0.072 0.029 0.010 0.000 0.000 0.000 0.010

0.000 0.000 0.000 0.010 0.000 0.000 0.020 0.000 0.000 0.029

0.010 0.000 0.000 0.000 0.061 0.055 0.062 0.038 0.005 0.000

0.000 0.000 0.000 0.005 0.000 0.000 0.000 0.010 0.000 0.000

0.021 0.000 0.010 0.005 0.000 0.000 0.005 0.000 0.066 0.077

0.072 0.055

>Non-Methylation code 128

0.006 0.000 0.000 0.000 0.000 0.006 0.000 0.000 0.000 0.006

0.000 0.000 0.024 0.012 0.006 0.012 0.000 0.000 0.000 0.000

0.063 0.060 0.064 0.074 0.026 0.000 0.000 0.000 0.000 0.000

0.000 0.000 0.000 0.000 0.000 0.000 0.053 0.000 0.009 0.018

0.000 0.000 0.000 0.000 0.053 0.049 0.055 0.072 0.006 0.000

0.000 0.006 0.000 0.000 0.000 0.000 0.000 0.006 0.000 0.000

0.040 0.000 0.011 0.000 0.000 0.000 0.000 0.000 0.072 0.074

0.040 0.078

>Non-Methylation code 129

0.005 0.010 0.000 0.000 0.000 0.005 0.005 0.000 0.000 0.005

0.000 0.000 0.010 0.000 0.005 0.010 0.000 0.005 0.000 0.000

0.053 0.074 0.075 0.071 0.014 0.000 0.000 0.000 0.000 0.005

0.000 0.000 0.000 0.005 0.000 0.000 0.009 0.000 0.009 0.005

0.005 0.000 0.009 0.000 0.071 0.075 0.077 0.049 0.009 0.000

0.000 0.000 0.000 0.028 0.000 0.000 0.000 0.028 0.000 0.000

0.028 0.000 0.000 0.000 0.000 0.009 0.009 0.000 0.060 0.062

0.043 0.056

>Non-Methylation code 130

0.023 0.000 0.000 0.000 0.000 0.017 0.000 0.000 0.000 0.000

0.000 0.000 0.012 0.000 0.006 0.000 0.000 0.012 0.006 0.000

0.063 0.058 0.066 0.071 0.013 0.000 0.000 0.000 0.000 0.019

0.006 0.000 0.000 0.013 0.000 0.000 0.019 0.000 0.006 0.006

0.000 0.000 0.000 0.000 0.059 0.063 0.060 0.069 0.000 0.000

0.000 0.000 0.000 0.019 0.000 0.000 0.000 0.005 0.000 0.000

0.010 0.000 0.015 0.000 0.005 0.000 0.005 0.000 0.098 0.034

0.091 0.052

>Non-Methylation code 131

0.025 0.000 0.000 0.000 0.000 0.000 0.000 0.000 0.000 0.000

0.000 0.000 0.050 0.000 0.008 0.017 0.000 0.000 0.000 0.000

0.058 0.046 0.061 0.068 0.005 0.000 0.000 0.005 0.000 0.005

0.000 0.000 0.000 0.005 0.000 0.000 0.036 0.000 0.010 0.000

0.000 0.000 0.000 0.000 0.071 0.068 0.040 0.087 0.006 0.000

0.000 0.000 0.000 0.006 0.000 0.000 0.000 0.006 0.000 0.000

0.025 0.006 0.006 0.013 0.000 0.000 0.000 0.000 0.067 0.058

0.065 0.073

>Non-Methylation code 132

0.000 0.000 0.000 0.005 0.000 0.015 0.000 0.000 0.000 0.005

0.000 0.005 0.025 0.000 0.010 0.000 0.000 0.000 0.000 0.000

0.072 0.076 0.036 0.083 0.013 0.000 0.000 0.006 0.000 0.006

0.000 0.000 0.000 0.006 0.006 0.000 0.019 0.000 0.006 0.013

0.000 0.000 0.000 0.000 0.070 0.066 0.062 0.059 0.019 0.000

0.000 0.000 0.000 0.000 0.000 0.000 0.000 0.000 0.000 0.000

0.032 0.000 0.013 0.000 0.000 0.000 0.006 0.000 0.045 0.072

0.066 0.079

>Non-Methylation code 133

0.004 0.000 0.000 0.004 0.000 0.004 0.000 0.000 0.000 0.000

0.000 0.000 0.016 0.000 0.008 0.004 0.000 0.000 0.012 0.000

0.076 0.076 0.078 0.050 0.005 0.000 0.000 0.000 0.000 0.026

0.000 0.000 0.005 0.010 0.000 0.000 0.000 0.000 0.016 0.000

0.000 0.005 0.000 0.000 0.077 0.094 0.067 0.026 0.027 0.007

0.000 0.007 0.000 0.014 0.000 0.000 0.007 0.007 0.000 0.000

0.000 0.007 0.000 0.000 0.000 0.000 0.000 0.000 0.057 0.061

0.067 0.073

>Non-Methylation code 134

0.004 0.000 0.000 0.000 0.000 0.004 0.000 0.000 0.000 0.004

0.000 0.000 0.026 0.000 0.013 0.000 0.004 0.000 0.000 0.000

0.045 0.076 0.074 0.082 0.013 0.000 0.000 0.000 0.000 0.013

0.000 0.000 0.000 0.007 0.000 0.000 0.026 0.013 0.007 0.000

0.000 0.000 0.000 0.000 0.067 0.050 0.069 0.069 0.019 0.000

0.006 0.000 0.000 0.000 0.000 0.000 0.000 0.000 0.000 0.000

0.031 0.000 0.006 0.013 0.000 0.000 0.000 0.000 0.069 0.060

0.059 0.070

>Non-Methylation code 135

0.011 0.000 0.000 0.000 0.000 0.006 0.000 0.000 0.000 0.000

0.000 0.006 0.023 0.006 0.011 0.000 0.006 0.000 0.000 0.000

0.078 0.044 0.089 0.054 0.010 0.000 0.005 0.000 0.000 0.005

0.000 0.000 0.000 0.000 0.000 0.000 0.030 0.000 0.010 0.000

0.005 0.000 0.000 0.000 0.068 0.062 0.061 0.076 0.000 0.000

0.006 0.000 0.000 0.006 0.000 0.000 0.000 0.006 0.000 0.000

0.029 0.006 0.011 0.000 0.006 0.000 0.000 0.000 0.064 0.074

0.067 0.060

>Non-Methylation code 136

0.000 0.005 0.005 0.000 0.000 0.009 0.009 0.000 0.000 0.009

0.000 0.000 0.005 0.000 0.009 0.000 0.000 0.005 0.000 0.000

0.050 0.074 0.072 0.082 0.007 0.007 0.007 0.000 0.014 0.007

0.000 0.007 0.000 0.000 0.014 0.000 0.000 0.007 0.000 0.007

0.000 0.000 0.000 0.000 0.072 0.063 0.067 0.054 0.005 0.005

0.000 0.000 0.000 0.000 0.000 0.000 0.000 0.000 0.000 0.000

0.005 0.000 0.009 0.014 0.005 0.000 0.009 0.000 0.066 0.069

0.072 0.076

>Non-Methylation code 137

0.000 0.000 0.000 0.000 0.000 0.000 0.000 0.000 0.000 0.013

0.000 0.000 0.031 0.006 0.006 0.025 0.000 0.000 0.000 0.000

0.061 0.061 0.048 0.082 0.006 0.000 0.000 0.000 0.000 0.000

0.000 0.000 0.000 0.012 0.000 0.000 0.035 0.000 0.006 0.012

0.000 0.000 0.006 0.000 0.059 0.064 0.057 0.078 0.020 0.000

0.000 0.000 0.000 0.000 0.000 0.000 0.000 0.040 0.000 0.000

0.040 0.000 0.000 0.020 0.000 0.000 0.000 0.000 0.055 0.059

0.034 0.065

>Non-Methylation code 138

0.006 0.000 0.000 0.000 0.000 0.011 0.000 0.000 0.000 0.011

0.000 0.006 0.006 0.006 0.006 0.006 0.006 0.000 0.006 0.006

0.076 0.071 0.064 0.048 0.015 0.000 0.000 0.000 0.000 0.023

0.000 0.000 0.000 0.015 0.000 0.000 0.008 0.000 0.008 0.000

0.015 0.000 0.008 0.000 0.060 0.055 0.058 0.071 0.000 0.000

0.000 0.012 0.000 0.012 0.006 0.000 0.000 0.006 0.000 0.000

0.018 0.000 0.000 0.006 0.000 0.006 0.006 0.000 0.058 0.066

0.063 0.074

>Non-Methylation code 139

0.016 0.005 0.000 0.005 0.000 0.005 0.000 0.000 0.000 0.016

0.000 0.000 0.000 0.005 0.005 0.005 0.000 0.000 0.005 0.000

0.075 0.059 0.061 0.071 0.017 0.000 0.000 0.000 0.000 0.006

0.000 0.000 0.006 0.006 0.000 0.000 0.011 0.000 0.006 0.000

0.000 0.000 0.011 0.000 0.085 0.080 0.024 0.083 0.000 0.000

0.000 0.007 0.000 0.022 0.000 0.000 0.000 0.015 0.000 0.000

0.029 0.000 0.000 0.007 0.000 0.000 0.007 0.000 0.053 0.062

0.064 0.067

>Non-Methylation code 140

0.008 0.000 0.000 0.000 0.000 0.008 0.024 0.008 0.000 0.008

0.008 0.000 0.016 0.000 0.000 0.008 0.008 0.008 0.000 0.000

0.067 0.042 0.072 0.050 0.009 0.000 0.000 0.000 0.000 0.000

0.009 0.009 0.000 0.027 0.000 0.000 0.009 0.000 0.000 0.018

0.018 0.000 0.009 0.000 0.067 0.045 0.068 0.045 0.000 0.014

0.000 0.000 0.000 0.007 0.007 0.000 0.000 0.007 0.000 0.007

0.021 0.000 0.000 0.007 0.007 0.000 0.000 0.007 0.059 0.066

0.051 0.074

>Non-Methylation code 141

0.009 0.000 0.000 0.000 0.000 0.004 0.000 0.000 0.000 0.009

0.004 0.000 0.017 0.000 0.013 0.000 0.000 0.000 0.000 0.000

0.087 0.057 0.068 0.067 0.018 0.018 0.000 0.000 0.000 0.009

0.009 0.000 0.000 0.000 0.000 0.000 0.045 0.000 0.000 0.000

0.000 0.009 0.000 0.000 0.058 0.056 0.049 0.062 0.016 0.000

0.000 0.000 0.000 0.000 0.005 0.000 0.000 0.005 0.000 0.000

0.016 0.000 0.011 0.005 0.005 0.000 0.000 0.000 0.081 0.066

0.086 0.035

>Non-Methylation code 142

0.009 0.000 0.000 0.000 0.000 0.000 0.004 0.000 0.009 0.000

0.000 0.000 0.009 0.000 0.009 0.000 0.000 0.000 0.013 0.000

0.068 0.085 0.063 0.066 0.015 0.000 0.000 0.000 0.000 0.023

0.000 0.000 0.000 0.008 0.000 0.008 0.023 0.000 0.000 0.008

0.008 0.000 0.008 0.000 0.055 0.062 0.053 0.064 0.018 0.006

0.000 0.006 0.000 0.006 0.000 0.000 0.000 0.018 0.000 0.000

0.012 0.000 0.006 0.000 0.000 0.000 0.000 0.000 0.067 0.066

0.063 0.067

>Non-Methylation code 143

0.000 0.000 0.000 0.000 0.000 0.008 0.023 0.008 0.000 0.008

0.008 0.000 0.016 0.000 0.000 0.008 0.016 0.008 0.000 0.000

0.067 0.040 0.071 0.054 0.009 0.000 0.000 0.000 0.000 0.000

0.018 0.009 0.000 0.027 0.000 0.000 0.000 0.000 0.000 0.018

0.018 0.000 0.009 0.000 0.064 0.036 0.073 0.052 0.000 0.007

0.000 0.000 0.000 0.007 0.007 0.000 0.000 0.007 0.000 0.007

0.021 0.000 0.000 0.007 0.014 0.000 0.000 0.007 0.061 0.055

0.053 0.080

>Non-Methylation code 144

0.000 0.000 0.005 0.005 0.005 0.005 0.000 0.000 0.010 0.000

0.000 0.000 0.010 0.000 0.000 0.005 0.005 0.000 0.010 0.000

0.077 0.058 0.067 0.072 0.000 0.000 0.006 0.000 0.000 0.018

0.000 0.000 0.006 0.000 0.006 0.000 0.006 0.006 0.006 0.018

0.000 0.000 0.000 0.000 0.056 0.069 0.071 0.066 0.009 0.000

0.000 0.018 0.000 0.009 0.000 0.000 0.000 0.009 0.000 0.009

0.000 0.009 0.000 0.000 0.009 0.000 0.009 0.000 0.075 0.050

0.066 0.061

>Non-Methylation code 145

0.016 0.000 0.005 0.000 0.000 0.005 0.005 0.000 0.005 0.005

0.000 0.000 0.011 0.000 0.005 0.011 0.000 0.000 0.000 0.000

0.078 0.062 0.069 0.054 0.009 0.000 0.000 0.000 0.000 0.009

0.000 0.000 0.000 0.009 0.005 0.000 0.009 0.000 0.014 0.000

0.005 0.000 0.000 0.000 0.073 0.068 0.055 0.079 0.011 0.005

0.000 0.005 0.000 0.005 0.000 0.000 0.000 0.000 0.000 0.000

0.011 0.005 0.011 0.000 0.000 0.000 0.005 0.000 0.068 0.074

0.051 0.080

>Non-Methylation code 146

0.000 0.000 0.000 0.007 0.000 0.007 0.027 0.000 0.000 0.000

0.000 0.000 0.014 0.000 0.007 0.000 0.014 0.000 0.000 0.000

0.064 0.058 0.083 0.053 0.007 0.007 0.000 0.000 0.000 0.000

0.021 0.000 0.000 0.000 0.000 0.007 0.007 0.000 0.000 0.000

0.028 0.000 0.007 0.000 0.067 0.053 0.062 0.067 0.007 0.007

0.000 0.000 0.000 0.007 0.013 0.000 0.000 0.000 0.000 0.000

0.013 0.000 0.007 0.000 0.026 0.000 0.000 0.000 0.071 0.049

0.068 0.066

>Non-Methylation code 147

0.007 0.000 0.000 0.007 0.000 0.000 0.030 0.007 0.000 0.000

0.000 0.000 0.000 0.000 0.007 0.000 0.015 0.000 0.007 0.000

0.071 0.044 0.084 0.053 0.000 0.007 0.000 0.000 0.000 0.007

0.015 0.000 0.000 0.000 0.000 0.015 0.007 0.000 0.000 0.000

0.030 0.000 0.000 0.007 0.061 0.056 0.061 0.066 0.006 0.006

0.000 0.000 0.000 0.006 0.019 0.006 0.000 0.000 0.006 0.000

0.000 0.000 0.006 0.000 0.019 0.000 0.000 0.000 0.068 0.044

0.082 0.061

>Non-Methylation code 148

0.009 0.000 0.000 0.005 0.000 0.000 0.005 0.000 0.000 0.009

0.005 0.000 0.014 0.000 0.009 0.000 0.005 0.000 0.000 0.000

0.081 0.059 0.070 0.063 0.007 0.020 0.000 0.000 0.000 0.000

0.014 0.000 0.007 0.000 0.007 0.000 0.027 0.000 0.000 0.000

0.000 0.007 0.000 0.000 0.061 0.069 0.057 0.057 0.017 0.006

0.000 0.000 0.000 0.000 0.006 0.000 0.000 0.000 0.000 0.006

0.011 0.000 0.006 0.006 0.011 0.000 0.000 0.000 0.067 0.064

0.075 0.058

>Non-Methylation code 149

0.018 0.000 0.000 0.000 0.000 0.009 0.000 0.000 0.000 0.037

0.000 0.000 0.037 0.000 0.000 0.018 0.000 0.000 0.000 0.000

0.053 0.062 0.035 0.063 0.006 0.000 0.000 0.000 0.000 0.000

0.000 0.000 0.000 0.013 0.000 0.000 0.025 0.006 0.006 0.025

0.000 0.000 0.000 0.000 0.063 0.059 0.051 0.078 0.006 0.000

0.000 0.000 0.000 0.000 0.000 0.000 0.000 0.006 0.000 0.000

0.038 0.000 0.006 0.013 0.000 0.000 0.006 0.000 0.060 0.069

0.059 0.068

>Non-Methylation code 150

0.000 0.006 0.000 0.000 0.000 0.012 0.006 0.000 0.000 0.018

0.000 0.000 0.006 0.000 0.006 0.006 0.012 0.006 0.000 0.000

0.071 0.064 0.065 0.053 0.015 0.022 0.000 0.000 0.000 0.015

0.007 0.000 0.000 0.000 0.000 0.000 0.007 0.007 0.000 0.007

0.007 0.000 0.000 0.000 0.051 0.059 0.062 0.072 0.017 0.000

0.000 0.000 0.000 0.006 0.011 0.006 0.006 0.000 0.000 0.000

0.006 0.000 0.011 0.000 0.000 0.000 0.000 0.000 0.065 0.071

0.062 0.073

>Non-Methylation code 151

0.000 0.017 0.000 0.000 0.000 0.000 0.000 0.000 0.000 0.008

0.000 0.000 0.050 0.008 0.000 0.025 0.000 0.000 0.000 0.000

0.059 0.061 0.043 0.061 0.007 0.000 0.000 0.000 0.000 0.000

0.000 0.000 0.000 0.013 0.000 0.000 0.039 0.000 0.007 0.013

0.000 0.007 0.000 0.000 0.069 0.057 0.057 0.065 0.020 0.000

0.000 0.000 0.000 0.000 0.000 0.000 0.000 0.040 0.000 0.000

0.030 0.000 0.000 0.030 0.000 0.000 0.000 0.000 0.051 0.044

0.044 0.075

>Non-Methylation code 152

0.007 0.007 0.007 0.000 0.000 0.007 0.007 0.000 0.000 0.014

0.000 0.007 0.000 0.007 0.000 0.014 0.000 0.007 0.000 0.000

0.067 0.048 0.074 0.061 0.010 0.005 0.005 0.000 0.000 0.005

0.000 0.000 0.005 0.000 0.005 0.000 0.000 0.000 0.005 0.005

0.010 0.000 0.000 0.005 0.059 0.069 0.071 0.075 0.005 0.005

0.000 0.000 0.000 0.000 0.000 0.000 0.005 0.000 0.000 0.000

0.005 0.005 0.010 0.000 0.010 0.000 0.005 0.000 0.067 0.072

0.054 0.089

>Non-Methylation code 153

0.000 0.008 0.000 0.000 0.000 0.008 0.024 0.000 0.000 0.000

0.000 0.016 0.000 0.000 0.000 0.000 0.031 0.000 0.000 0.008

0.071 0.051 0.051 0.067 0.006 0.006 0.000 0.000 0.000 0.006

0.019 0.006 0.000 0.000 0.006 0.000 0.000 0.000 0.006 0.000

0.025 0.000 0.000 0.000 0.073 0.044 0.080 0.056 0.000 0.000

0.000 0.008 0.000 0.000 0.033 0.008 0.000 0.000 0.000 0.000

0.000 0.000 0.008 0.000 0.017 0.000 0.008 0.000 0.067 0.047

0.080 0.057

>Non-Methylation code 154

0.018 0.000 0.000 0.006 0.000 0.000 0.006 0.006 0.000 0.012

0.000 0.000 0.006 0.006 0.012 0.000 0.000 0.000 0.000 0.000

0.056 0.063 0.055 0.086 0.000 0.009 0.000 0.005 0.000 0.009

0.005 0.000 0.000 0.000 0.000 0.000 0.009 0.000 0.009 0.009

0.005 0.000 0.000 0.000 0.068 0.067 0.067 0.072 0.014 0.000

0.005 0.005 0.000 0.000 0.000 0.000 0.000 0.000 0.000 0.000

0.000 0.000 0.014 0.005 0.005 0.005 0.000 0.000 0.052 0.059

0.077 0.092

>Non-Methylation code 155

0.000 0.000 0.000 0.000 0.000 0.000 0.000 0.000 0.018 0.006

0.006 0.006 0.006 0.006 0.006 0.000 0.000 0.000 0.000 0.012

0.083 0.049 0.060 0.075 0.000 0.000 0.000 0.000 0.000 0.000

0.000 0.007 0.013 0.000 0.000 0.013 0.007 0.000 0.000 0.013

0.013 0.007 0.007 0.000 0.063 0.061 0.050 0.079 0.000 0.000

0.000 0.007 0.000 0.007 0.000 0.007 0.015 0.015 0.000 0.000

0.007 0.007 0.000 0.015 0.007 0.000 0.000 0.000 0.071 0.057

0.042 0.074

>Non-Methylation code 156

0.008 0.000 0.000 0.000 0.000 0.000 0.000 0.000 0.008 0.008

0.008 0.008 0.008 0.000 0.008 0.030 0.008 0.000 0.000 0.000

0.068 0.047 0.063 0.065 0.005 0.005 0.000 0.000 0.000 0.005

0.000 0.000 0.000 0.005 0.000 0.005 0.014 0.000 0.009 0.000

0.005 0.000 0.005 0.000 0.080 0.070 0.049 0.081 0.007 0.000

0.007 0.007 0.007 0.013 0.007 0.000 0.000 0.007 0.000 0.000

0.007 0.013 0.000 0.000 0.000 0.007 0.000 0.000 0.060 0.070

0.063 0.062

>Non-Methylation code 157

0.016 0.000 0.000 0.000 0.000 0.000 0.005 0.000 0.000 0.005

0.005 0.000 0.010 0.000 0.010 0.016 0.000 0.000 0.000 0.000

0.043 0.070 0.073 0.080 0.005 0.005 0.000 0.000 0.000 0.005

0.000 0.000 0.000 0.005 0.000 0.000 0.019 0.000 0.014 0.000

0.005 0.005 0.000 0.000 0.084 0.057 0.069 0.062 0.013 0.004

0.004 0.000 0.004 0.000 0.000 0.000 0.000 0.000 0.000 0.000

0.009 0.004 0.009 0.000 0.000 0.004 0.000 0.000 0.067 0.069

0.076 0.069

>Non-Methylation code 158

0.020 0.005 0.000 0.000 0.015 0.000 0.005 0.000 0.000 0.005

0.000 0.000 0.000 0.005 0.005 0.000 0.000 0.005 0.000 0.000

0.051 0.067 0.077 0.072 0.016 0.000 0.000 0.000 0.000 0.000

0.008 0.000 0.000 0.016 0.000 0.000 0.000 0.000 0.008 0.048

0.008 0.000 0.000 0.000 0.062 0.066 0.067 0.034 0.000 0.004

0.000 0.000 0.000 0.004 0.000 0.000 0.000 0.013 0.000 0.000

0.008 0.000 0.013 0.000 0.004 0.004 0.000 0.000 0.083 0.055

0.056 0.088

>Non-Methylation code 159

0.004 0.004 0.000 0.000 0.004 0.004 0.000 0.000 0.000 0.004

0.000 0.000 0.017 0.000 0.013 0.000 0.000 0.004 0.000 0.000

0.077 0.060 0.069 0.071 0.012 0.004 0.004 0.000 0.004 0.000

0.000 0.000 0.000 0.000 0.000 0.000 0.008 0.004 0.008 0.004

0.000 0.004 0.000 0.000 0.067 0.073 0.075 0.066 0.017 0.000

0.000 0.000 0.000 0.000 0.000 0.000 0.000 0.006 0.006 0.000

0.011 0.000 0.011 0.017 0.000 0.000 0.000 0.000 0.038 0.069

0.075 0.084

>Non-Methylation code 160

0.000 0.007 0.000 0.000 0.007 0.007 0.007 0.000 0.000 0.020

0.000 0.000 0.027 0.000 0.000 0.014 0.000 0.000 0.000 0.000

0.067 0.052 0.056 0.070 0.021 0.010 0.000 0.000 0.010 0.000

0.000 0.000 0.000 0.021 0.000 0.000 0.031 0.000 0.000 0.010

0.010 0.000 0.000 0.000 0.040 0.053 0.061 0.066 0.006 0.000

0.000 0.000 0.000 0.000 0.000 0.000 0.006 0.013 0.000 0.000

0.026 0.000 0.006 0.019 0.000 0.000 0.000 0.000 0.042 0.075

0.073 0.067

>Non-Methylation code 161

0.007 0.000 0.000 0.000 0.000 0.000 0.007 0.007 0.013 0.000

0.007 0.007 0.007 0.007 0.000 0.007 0.013 0.000 0.007 0.000

0.064 0.063 0.050 0.071 0.005 0.005 0.000 0.000 0.000 0.005

0.005 0.005 0.010 0.010 0.000 0.000 0.000 0.005 0.005 0.005

0.005 0.000 0.000 0.000 0.081 0.073 0.045 0.069 0.007 0.000

0.007 0.000 0.000 0.000 0.000 0.000 0.013 0.000 0.000 0.007

0.007 0.007 0.007 0.000 0.007 0.000 0.000 0.007 0.078 0.051

0.073 0.065

>Non-Methylation code 162

0.000 0.000 0.000 0.000 0.000 0.006 0.018 0.000 0.006 0.012

0.000 0.000 0.000 0.000 0.012 0.018 0.000 0.000 0.000 0.000

0.078 0.040 0.082 0.062 0.019 0.000 0.006 0.000 0.000 0.000

0.000 0.006 0.006 0.000 0.000 0.000 0.006 0.000 0.000 0.000

0.013 0.006 0.006 0.006 0.068 0.063 0.067 0.060 0.000 0.000

0.000 0.007 0.000 0.007 0.000 0.000 0.000 0.007 0.000 0.000

0.014 0.014 0.007 0.007 0.014 0.000 0.000 0.000 0.054 0.060

0.074 0.070

>Non-Methylation code 163

0.000 0.009 0.000 0.000 0.000 0.013 0.000 0.000 0.004 0.009

0.000 0.009 0.004 0.004 0.004 0.000 0.000 0.000 0.000 0.000

0.068 0.058 0.069 0.083 0.018 0.000 0.000 0.012 0.000 0.000

0.000 0.006 0.000 0.000 0.000 0.000 0.000 0.000 0.012 0.006

0.006 0.006 0.000 0.000 0.074 0.073 0.071 0.049 0.005 0.000

0.000 0.020 0.000 0.000 0.000 0.000 0.000 0.015 0.000 0.000

0.005 0.000 0.015 0.000 0.000 0.000 0.000 0.000 0.055 0.057

0.075 0.086

>Non-Methylation code 164

0.020 0.000 0.005 0.000 0.000 0.010 0.000 0.000 0.000 0.005

0.000 0.005 0.000 0.000 0.005 0.005 0.000 0.000 0.010 0.000

0.062 0.075 0.068 0.063 0.013 0.000 0.000 0.000 0.000 0.026

0.000 0.007 0.000 0.013 0.000 0.000 0.007 0.000 0.007 0.000

0.007 0.000 0.007 0.000 0.077 0.067 0.054 0.050 0.007 0.000

0.000 0.007 0.000 0.020 0.000 0.000 0.000 0.007 0.000 0.000

0.000 0.013 0.013 0.000 0.000 0.000 0.000 0.000 0.038 0.079

0.075 0.076

>Non-Methylation code 165

0.000 0.000 0.000 0.011 0.000 0.005 0.000 0.000 0.000 0.011

0.000 0.005 0.005 0.011 0.011 0.005 0.005 0.000 0.000 0.000

0.059 0.074 0.058 0.072 0.017 0.000 0.000 0.000 0.000 0.000

0.000 0.000 0.000 0.009 0.000 0.000 0.000 0.009 0.009 0.035

0.009 0.009 0.009 0.000 0.057 0.063 0.061 0.048 0.023 0.000

0.000 0.000 0.000 0.015 0.008 0.000 0.008 0.000 0.000 0.000

0.023 0.000 0.000 0.000 0.000 0.000 0.008 0.000 0.071 0.062

0.061 0.055

>Non-Methylation code 166

0.005 0.000 0.005 0.000 0.000 0.005 0.000 0.000 0.000 0.011

0.000 0.000 0.011 0.000 0.011 0.005 0.005 0.005 0.000 0.000

0.072 0.076 0.068 0.054 0.006 0.000 0.000 0.000 0.000 0.012

0.006 0.000 0.000 0.006 0.000 0.000 0.000 0.006 0.017 0.006

0.006 0.000 0.000 0.000 0.081 0.079 0.026 0.083 0.011 0.000

0.000 0.011 0.000 0.022 0.000 0.000 0.000 0.022 0.000 0.000

0.011 0.000 0.000 0.032 0.011 0.011 0.000 0.000 0.051 0.047

0.066 0.039

>Non-Methylation code 167

0.000 0.005 0.000 0.005 0.000 0.010 0.005 0.000 0.000 0.005

0.000 0.000 0.000 0.005 0.010 0.000 0.000 0.010 0.005 0.000

0.059 0.069 0.072 0.074 0.000 0.000 0.009 0.000 0.000 0.028

0.000 0.000 0.019 0.000 0.009 0.000 0.000 0.000 0.000 0.038

0.000 0.000 0.000 0.000 0.071 0.056 0.082 0.021 0.019 0.000

0.000 0.000 0.000 0.000 0.000 0.009 0.000 0.009 0.000 0.000

0.019 0.000 0.000 0.019 0.000 0.000 0.019 0.000 0.075 0.063

0.072 0.029

>Non-Methylation code 168

0.009 0.000 0.000 0.000 0.000 0.014 0.000 0.000 0.000 0.005

0.000 0.000 0.005 0.000 0.014 0.000 0.000 0.000 0.005 0.000

0.079 0.054 0.070 0.079 0.007 0.000 0.007 0.013 0.000 0.026

0.000 0.000 0.000 0.013 0.000 0.000 0.007 0.000 0.000 0.007

0.000 0.000 0.007 0.000 0.061 0.067 0.066 0.055 0.007 0.007

0.000 0.000 0.000 0.021 0.000 0.000 0.000 0.007 0.000 0.000

0.007 0.000 0.007 0.014 0.007 0.000 0.000 0.000 0.072 0.068

0.081 0.037

>Non-Methylation code 169

0.005 0.000 0.009 0.005 0.000 0.014 0.000 0.000 0.009 0.005

0.005 0.005 0.000 0.000 0.005 0.000 0.000 0.000 0.000 0.000

0.056 0.064 0.084 0.067 0.006 0.000 0.000 0.012 0.000 0.006

0.000 0.006 0.006 0.006 0.006 0.000 0.000 0.006 0.012 0.000

0.006 0.000 0.006 0.000 0.044 0.059 0.076 0.079 0.000 0.000

0.000 0.005 0.000 0.010 0.000 0.000 0.005 0.000 0.000 0.000

0.005 0.005 0.010 0.010 0.000 0.000 0.005 0.000 0.072 0.066

0.069 0.071

>Non-Methylation code 170

0.000 0.000 0.000 0.000 0.016 0.000 0.005 0.000 0.000 0.005

0.000 0.011 0.000 0.005 0.011 0.016 0.000 0.000 0.000 0.000

0.071 0.079 0.052 0.063 0.006 0.006 0.000 0.000 0.006 0.006

0.000 0.011 0.000 0.000 0.000 0.000 0.000 0.006 0.006 0.017

0.006 0.006 0.000 0.000 0.068 0.064 0.066 0.064 0.007 0.000

0.000 0.015 0.015 0.000 0.000 0.000 0.000 0.007 0.000 0.000

0.007 0.015 0.000 0.015 0.000 0.007 0.000 0.000 0.047 0.074

0.066 0.059

>Non-Methylation code 171

0.000 0.000 0.000 0.000 0.006 0.006 0.000 0.006 0.017 0.006

0.000 0.000 0.006 0.006 0.006 0.011 0.000 0.006 0.000 0.000

0.067 0.080 0.059 0.056 0.007 0.000 0.000 0.000 0.013 0.007

0.000 0.000 0.013 0.020 0.000 0.000 0.000 0.000 0.007 0.013

0.000 0.000 0.000 0.000 0.062 0.072 0.072 0.049 0.005 0.005

0.000 0.005 0.011 0.000 0.000 0.000 0.000 0.000 0.000 0.005

0.005 0.005 0.005 0.005 0.000 0.000 0.000 0.000 0.075 0.076

0.071 0.057

>Non-Methylation code 172

0.000 0.000 0.000 0.000 0.016 0.000 0.000 0.000 0.010 0.005

0.000 0.005 0.005 0.005 0.005 0.005 0.005 0.000 0.000 0.000

0.054 0.079 0.070 0.068 0.000 0.000 0.000 0.000 0.011 0.005

0.000 0.005 0.011 0.011 0.000 0.000 0.005 0.000 0.005 0.011

0.000 0.000 0.000 0.000 0.065 0.082 0.065 0.056 0.006 0.000

0.000 0.006 0.022 0.000 0.000 0.000 0.017 0.011 0.000 0.000

0.000 0.000 0.000 0.006 0.000 0.000 0.000 0.000 0.055 0.063

0.081 0.067

>Non-Methylation code 173

0.005 0.005 0.000 0.000 0.000 0.011 0.000 0.000 0.000 0.005

0.000 0.000 0.011 0.000 0.011 0.016 0.005 0.000 0.000 0.000

0.075 0.066 0.070 0.052 0.021 0.000 0.000 0.000 0.000 0.011

0.000 0.000 0.000 0.005 0.000 0.000 0.005 0.000 0.016 0.000

0.000 0.000 0.000 0.000 0.095 0.036 0.084 0.059 0.011 0.006

0.006 0.006 0.000 0.006 0.000 0.000 0.000 0.011 0.000 0.000

0.011 0.000 0.006 0.006 0.000 0.000 0.000 0.000 0.078 0.080

0.049 0.059

>Non-Methylation code 174

0.000 0.000 0.000 0.005 0.005 0.000 0.005 0.000 0.010 0.005

0.000 0.005 0.005 0.005 0.005 0.010 0.000 0.000 0.005 0.000

0.076 0.057 0.074 0.058 0.000 0.006 0.000 0.000 0.000 0.012

0.000 0.006 0.000 0.000 0.000 0.018 0.012 0.006 0.000 0.012

0.006 0.000 0.000 0.000 0.049 0.065 0.075 0.065 0.006 0.000

0.000 0.012 0.006 0.000 0.000 0.006 0.000 0.012 0.000 0.000

0.006 0.006 0.006 0.000 0.012 0.000 0.000 0.000 0.070 0.058

0.072 0.059

>Non-Methylation code 175

0.017 0.000 0.000 0.000 0.000 0.006 0.006 0.000 0.000 0.006

0.000 0.000 0.006 0.000 0.011 0.006 0.000 0.006 0.000 0.000

0.078 0.062 0.062 0.071 0.005 0.000 0.000 0.000 0.005 0.014

0.000 0.000 0.000 0.000 0.000 0.000 0.005 0.010 0.010 0.000

0.005 0.000 0.010 0.000 0.079 0.053 0.062 0.078 0.005 0.000

0.000 0.010 0.000 0.014 0.000 0.000 0.005 0.000 0.000 0.000

0.000 0.000 0.014 0.005 0.000 0.005 0.000 0.000 0.068 0.056

0.082 0.070

>Non-Methylation code 176

0.034 0.000 0.000 0.009 0.009 0.009 0.009 0.000 0.000 0.000

0.000 0.000 0.009 0.000 0.000 0.009 0.000 0.009 0.000 0.000

0.067 0.049 0.068 0.056 0.005 0.000 0.000 0.005 0.005 0.000

0.005 0.000 0.000 0.025 0.005 0.005 0.000 0.000 0.010 0.000

0.000 0.000 0.000 0.000 0.085 0.069 0.038 0.076 0.000 0.013

0.000 0.000 0.006 0.013 0.000 0.006 0.000 0.006 0.006 0.000

0.000 0.000 0.000 0.013 0.000 0.000 0.006 0.000 0.058 0.071

0.065 0.070

>Non-Methylation code 177

0.000 0.000 0.000 0.000 0.000 0.023 0.000 0.008 0.016 0.000

0.008 0.000 0.000 0.000 0.000 0.016 0.008 0.000 0.008 0.000

0.074 0.066 0.075 0.033 0.005 0.000 0.000 0.000 0.000 0.011

0.000 0.000 0.000 0.005 0.000 0.000 0.011 0.000 0.011 0.016

0.000 0.005 0.005 0.000 0.074 0.057 0.061 0.071 0.000 0.000

0.014 0.000 0.000 0.014 0.007 0.000 0.000 0.014 0.000 0.000

0.000 0.014 0.000 0.000 0.000 0.007 0.007 0.000 0.078 0.054

0.061 0.060

>Non-Methylation code 178

0.005 0.005 0.005 0.000 0.000 0.000 0.000 0.000 0.000 0.016

0.000 0.005 0.011 0.011 0.005 0.000 0.000 0.005 0.000 0.000

0.068 0.069 0.068 0.058 0.005 0.005 0.000 0.000 0.005 0.000

0.000 0.000 0.000 0.000 0.000 0.005 0.005 0.005 0.011 0.011

0.011 0.000 0.000 0.000 0.079 0.051 0.066 0.074 0.011 0.000

0.000 0.005 0.000 0.000 0.000 0.000 0.000 0.005 0.000 0.000

0.022 0.000 0.011 0.005 0.005 0.000 0.000 0.000 0.074 0.089

0.035 0.070

>Non-Methylation code 179

0.010 0.000 0.000 0.005 0.000 0.000 0.000 0.000 0.000 0.005

0.000 0.000 0.020 0.000 0.010 0.005 0.005 0.000 0.005 0.000

0.074 0.091 0.035 0.069 0.005 0.005 0.005 0.000 0.000 0.005

0.000 0.000 0.000 0.015 0.000 0.005 0.010 0.010 0.005 0.000

0.000 0.000 0.000 0.000 0.074 0.062 0.073 0.057 0.006 0.006

0.000 0.000 0.006 0.000 0.000 0.000 0.000 0.000 0.000 0.006

0.006 0.006 0.012 0.006 0.012 0.000 0.000 0.000 0.078 0.057

0.062 0.072

>Non-Methylation code 180

0.023 0.000 0.000 0.000 0.000 0.015 0.000 0.000 0.000 0.015

0.000 0.000 0.008 0.000 0.008 0.023 0.008 0.000 0.000 0.000

0.070 0.059 0.066 0.039 0.006 0.000 0.000 0.000 0.000 0.011

0.000 0.000 0.000 0.011 0.000 0.000 0.006 0.006 0.017 0.006

0.000 0.000 0.000 0.000 0.057 0.080 0.064 0.070 0.021 0.000

0.000 0.005 0.005 0.005 0.005 0.000 0.000 0.005 0.000 0.000

0.000 0.000 0.005 0.005 0.000 0.000 0.005 0.000 0.063 0.064

0.069 0.074

>Non-Methylation code 181

0.000 0.010 0.000 0.000 0.005 0.000 0.005 0.000 0.010 0.010

0.000 0.000 0.005 0.005 0.000 0.005 0.000 0.000 0.000 0.000

0.067 0.072 0.067 0.070 0.013 0.000 0.013 0.000 0.000 0.000

0.000 0.007 0.000 0.000 0.000 0.013 0.007 0.000 0.000 0.013

0.000 0.007 0.000 0.007 0.070 0.063 0.047 0.073 0.000 0.000

0.000 0.011 0.000 0.000 0.000 0.011 0.000 0.011 0.033 0.000

0.011 0.011 0.000 0.011 0.011 0.011 0.000 0.000 0.069 0.051

0.048 0.043

>Non-Methylation code 182

0.006 0.000 0.000 0.000 0.000 0.000 0.000 0.000 0.000 0.006

0.000 0.000 0.000 0.006 0.017 0.028 0.000 0.006 0.006 0.000

0.093 0.038 0.090 0.040 0.022 0.000 0.015 0.000 0.000 0.015

0.000 0.000 0.007 0.015 0.000 0.000 0.015 0.000 0.000 0.007

0.000 0.000 0.000 0.000 0.071 0.047 0.071 0.048 0.000 0.000

0.000 0.016 0.000 0.000 0.000 0.008 0.000 0.016 0.000 0.000

0.008 0.016 0.008 0.000 0.008 0.000 0.000 0.000 0.060 0.071

0.066 0.057

>Non-Methylation code 183

0.005 0.000 0.000 0.005 0.005 0.010 0.000 0.000 0.000 0.010

0.000 0.000 0.010 0.000 0.010 0.005 0.005 0.000 0.000 0.000

0.063 0.077 0.066 0.066 0.000 0.000 0.000 0.004 0.000 0.011

0.000 0.000 0.000 0.000 0.000 0.000 0.004 0.000 0.018 0.004

0.000 0.000 0.007 0.000 0.065 0.064 0.078 0.079 0.020 0.000

0.007 0.000 0.000 0.020 0.000 0.000 0.000 0.000 0.000 0.000

0.013 0.000 0.007 0.000 0.000 0.013 0.000 0.000 0.067 0.054

0.054 0.078

>Non-Methylation code 184

0.000 0.000 0.000 0.004 0.000 0.030 0.000 0.000 0.000 0.000

0.000 0.000 0.000 0.000 0.017 0.000 0.000 0.000 0.004 0.000

0.085 0.052 0.083 0.057 0.058 0.000 0.000 0.000 0.000 0.049

0.000 0.000 0.000 0.000 0.000 0.000 0.000 0.000 0.010 0.000

0.000 0.000 0.000 0.000 0.054 0.061 0.063 0.038 0.012 0.000

0.000 0.000 0.000 0.023 0.000 0.000 0.000 0.006 0.000 0.000

0.006 0.000 0.017 0.000 0.000 0.000 0.000 0.000 0.072 0.064

0.045 0.090

>Non-Methylation code 185

0.025 0.000 0.006 0.000 0.000 0.019 0.000 0.000 0.000 0.000

0.000 0.000 0.013 0.000 0.006 0.000 0.000 0.013 0.000 0.000

0.062 0.068 0.050 0.072 0.005 0.000 0.000 0.005 0.005 0.010

0.000 0.000 0.000 0.010 0.000 0.000 0.010 0.000 0.010 0.005

0.005 0.000 0.000 0.000 0.063 0.077 0.066 0.066 0.000 0.000

0.000 0.004 0.000 0.008 0.000 0.000 0.000 0.000 0.000 0.000

0.004 0.000 0.020 0.004 0.000 0.000 0.008 0.000 0.063 0.065

0.078 0.078

>Non-Methylation code 186

0.011 0.000 0.000 0.006 0.000 0.006 0.000 0.000 0.000 0.000

0.000 0.000 0.017 0.006 0.011 0.011 0.006 0.000 0.000 0.000

0.071 0.071 0.042 0.076 0.021 0.000 0.000 0.000 0.000 0.000

0.005 0.000 0.005 0.005 0.000 0.000 0.016 0.005 0.005 0.005

0.000 0.000 0.000 0.000 0.069 0.065 0.055 0.074 0.000 0.000

0.000 0.000 0.000 0.000 0.005 0.000 0.005 0.000 0.000 0.000

0.022 0.000 0.011 0.005 0.005 0.005 0.000 0.000 0.092 0.050

0.073 0.058

>Non-Methylation code 187

0.005 0.000 0.005 0.000 0.000 0.000 0.000 0.000 0.000 0.010

0.000 0.000 0.034 0.000 0.010 0.000 0.000 0.000 0.000 0.000

0.071 0.049 0.070 0.081 0.011 0.000 0.000 0.000 0.000 0.000

0.005 0.000 0.000 0.000 0.000 0.000 0.032 0.000 0.005 0.000

0.005 0.000 0.005 0.005 0.076 0.057 0.067 0.064 0.007 0.000

0.000 0.000 0.000 0.007 0.000 0.000 0.000 0.000 0.000 0.000

0.049 0.000 0.007 0.000 0.014 0.000 0.000 0.000 0.066 0.052

0.069 0.062

>Non-Methylation code 188

0.028 0.000 0.000 0.000 0.000 0.000 0.006 0.000 0.000 0.006

0.000 0.000 0.017 0.000 0.017 0.000 0.000 0.000 0.000 0.000

0.074 0.052 0.058 0.077 0.009 0.000 0.000 0.005 0.000 0.000

0.009 0.000 0.000 0.000 0.000 0.000 0.014 0.000 0.014 0.000

0.005 0.000 0.000 0.005 0.076 0.057 0.061 0.080 0.005 0.000

0.000 0.000 0.000 0.010 0.000 0.000 0.000 0.000 0.000 0.000

0.010 0.005 0.015 0.000 0.015 0.000 0.000 0.000 0.078 0.070

0.050 0.076

>Non-Methylation code 189

0.012 0.000 0.000 0.000 0.000 0.000 0.006 0.000 0.000 0.000

0.000 0.000 0.030 0.000 0.006 0.018 0.006 0.000 0.000 0.000

0.067 0.050 0.074 0.064 0.016 0.000 0.000 0.000 0.000 0.000

0.008 0.000 0.000 0.008 0.000 0.000 0.057 0.008 0.000 0.000

0.008 0.000 0.000 0.000 0.068 0.059 0.068 0.033 0.000 0.000

0.000 0.000 0.000 0.000 0.005 0.000 0.005 0.010 0.000 0.000

0.020 0.000 0.010 0.005 0.005 0.000 0.000 0.000 0.077 0.044

0.073 0.078

>Non-Methylation code 190

0.006 0.006 0.000 0.000 0.000 0.000 0.000 0.006 0.000 0.011

0.000 0.000 0.006 0.006 0.011 0.006 0.000 0.000 0.000 0.006

0.082 0.070 0.073 0.046 0.000 0.009 0.005 0.005 0.005 0.000

0.000 0.000 0.009 0.009 0.000 0.000 0.000 0.005 0.000 0.005

0.005 0.000 0.000 0.000 0.071 0.062 0.075 0.071 0.006 0.006

0.000 0.000 0.000 0.000 0.000 0.000 0.011 0.017 0.000 0.006

0.006 0.000 0.000 0.006 0.006 0.006 0.000 0.000 0.057 0.056

0.074 0.078

>Non-Methylation code 191

0.006 0.000 0.000 0.000 0.000 0.006 0.000 0.013 0.006 0.006

0.000 0.006 0.026 0.000 0.000 0.006 0.006 0.000 0.000 0.000

0.062 0.057 0.062 0.069 0.006 0.000 0.000 0.000 0.000 0.000

0.000 0.000 0.000 0.011 0.000 0.000 0.017 0.011 0.011 0.000

0.006 0.006 0.000 0.000 0.060 0.058 0.053 0.093 0.005 0.005

0.000 0.005 0.000 0.000 0.005 0.000 0.010 0.000 0.000 0.000

0.020 0.000 0.005 0.000 0.000 0.000 0.000 0.005 0.078 0.061

0.056 0.079

>Non-Methylation code 192

0.004 0.004 0.000 0.000 0.000 0.000 0.000 0.000 0.004 0.004

0.000 0.004 0.004 0.000 0.013 0.000 0.004 0.013 0.000 0.000

0.061 0.060 0.093 0.065 0.006 0.006 0.000 0.000 0.000 0.012

0.006 0.006 0.000 0.012 0.000 0.000 0.006 0.000 0.006 0.006

0.000 0.000 0.000 0.000 0.068 0.071 0.081 0.049 0.005 0.010

0.005 0.000 0.005 0.005 0.000 0.000 0.005 0.000 0.000 0.000

0.000 0.005 0.005 0.005 0.005 0.000 0.000 0.000 0.080 0.080

0.063 0.058

>Non-Methylation code 193

0.005 0.000 0.000 0.000 0.000 0.000 0.000 0.000 0.005 0.000

0.000 0.000 0.005 0.000 0.011 0.016 0.022 0.000 0.005 0.000

0.073 0.080 0.036 0.073 0.000 0.000 0.005 0.000 0.000 0.011

0.000 0.000 0.000 0.016 0.000 0.005 0.011 0.011 0.011 0.000

0.000 0.000 0.000 0.000 0.076 0.058 0.069 0.062 0.000 0.000

0.015 0.000 0.000 0.008 0.000 0.015 0.008 0.008 0.000 0.008

0.000 0.008 0.000 0.015 0.000 0.000 0.000 0.000 0.067 0.059

0.063 0.060

>Non-Methylation code 194

0.005 0.000 0.000 0.000 0.000 0.000 0.000 0.010 0.005 0.005

0.000 0.000 0.000 0.005 0.016 0.005 0.000 0.005 0.000 0.005

0.046 0.074 0.068 0.082 0.007 0.007 0.000 0.000 0.000 0.014

0.000 0.000 0.000 0.007 0.000 0.007 0.000 0.007 0.007 0.021

0.007 0.000 0.000 0.000 0.069 0.067 0.053 0.061 0.011 0.006

0.006 0.006 0.000 0.000 0.006 0.000 0.000 0.006 0.000 0.000

0.006 0.000 0.006 0.006 0.006 0.006 0.000 0.000 0.057 0.072

0.071 0.067

>Non-Methylation code 195

0.000 0.000 0.006 0.000 0.006 0.013 0.013 0.000 0.000 0.013

0.006 0.000 0.000 0.000 0.000 0.006 0.000 0.000 0.006 0.006

0.066 0.053 0.072 0.066 0.006 0.006 0.006 0.000 0.006 0.006

0.000 0.006 0.000 0.000 0.000 0.000 0.000 0.000 0.006 0.006

0.018 0.000 0.006 0.000 0.055 0.066 0.064 0.077 0.005 0.000

0.000 0.000 0.005 0.005 0.005 0.000 0.000 0.011 0.000 0.000

0.005 0.000 0.011 0.005 0.005 0.005 0.000 0.000 0.078 0.091

0.073 0.025

>Non-Methylation code 196

0.006 0.006 0.006 0.000 0.006 0.019 0.000 0.006 0.000 0.006

0.000 0.000 0.000 0.006 0.000 0.000 0.013 0.000 0.000 0.000

0.044 0.062 0.070 0.081 0.005 0.000 0.000 0.000 0.005 0.010

0.005 0.000 0.000 0.005 0.000 0.000 0.005 0.000 0.010 0.005

0.005 0.005 0.000 0.000 0.068 0.077 0.075 0.052 0.006 0.000

0.000 0.000 0.006 0.018 0.012 0.000 0.000 0.006 0.006 0.000

0.000 0.000 0.006 0.000 0.000 0.000 0.006 0.006 0.061 0.061

0.075 0.067

>Non-Methylation code 197

0.000 0.000 0.000 0.000 0.000 0.006 0.000 0.000 0.000 0.006

0.006 0.000 0.000 0.019 0.006 0.006 0.006 0.013 0.006 0.006

0.061 0.060 0.048 0.081 0.000 0.006 0.000 0.000 0.006 0.018

0.006 0.000 0.012 0.000 0.000 0.000 0.006 0.000 0.000 0.018

0.006 0.000 0.000 0.000 0.068 0.066 0.068 0.055 0.000 0.000

0.000 0.000 0.000 0.018 0.009 0.000 0.000 0.000 0.000 0.009

0.018 0.009 0.000 0.018 0.009 0.018 0.000 0.000 0.068 0.070

0.051 0.040

>Non-Methylation code 198

0.005 0.005 0.000 0.005 0.000 0.000 0.005 0.000 0.010 0.000

0.000 0.000 0.019 0.000 0.000 0.000 0.000 0.000 0.010 0.005

0.066 0.061 0.062 0.081 0.007 0.000 0.000 0.000 0.000 0.013

0.000 0.007 0.007 0.007 0.000 0.007 0.026 0.000 0.000 0.007

0.007 0.000 0.000 0.000 0.064 0.052 0.059 0.073 0.007 0.000

0.000 0.000 0.000 0.000 0.000 0.000 0.000 0.014 0.000 0.000

0.021 0.007 0.014 0.000 0.007 0.007 0.000 0.000 0.050 0.060

0.061 0.087

>Non-Methylation code 199

0.008 0.000 0.000 0.000 0.000 0.017 0.000 0.000 0.000 0.008

0.000 0.008 0.000 0.008 0.008 0.033 0.008 0.000 0.000 0.000

0.062 0.068 0.047 0.056 0.012 0.000 0.006 0.006 0.000 0.000

0.006 0.000 0.000 0.006 0.000 0.000 0.006 0.000 0.006 0.006

0.006 0.006 0.000 0.000 0.057 0.064 0.074 0.071 0.005 0.000

0.000 0.005 0.000 0.000 0.000 0.011 0.005 0.005 0.000 0.000

0.000 0.005 0.011 0.005 0.000 0.005 0.000 0.005 0.053 0.075

0.069 0.072

>Non-Methylation code 200

0.006 0.000 0.000 0.000 0.000 0.000 0.000 0.000 0.000 0.012

0.000 0.000 0.024 0.006 0.012 0.000 0.006 0.006 0.000 0.000

0.058 0.056 0.048 0.098 0.004 0.004 0.000 0.004 0.000 0.000

0.004 0.000 0.009 0.000 0.000 0.000 0.018 0.000 0.004 0.000

0.000 0.000 0.004 0.004 0.071 0.068 0.060 0.077 0.007 0.000

0.000 0.000 0.000 0.007 0.000 0.007 0.007 0.007 0.000 0.007

0.027 0.000 0.000 0.007 0.007 0.000 0.000 0.000 0.064 0.055

0.057 0.076

>Non-Methylation code 201

0.000 0.005 0.005 0.000 0.000 0.005 0.000 0.005 0.010 0.005

0.000 0.005 0.010 0.000 0.005 0.000 0.005 0.000 0.000 0.000

0.073 0.063 0.092 0.045 0.000 0.000 0.012 0.000 0.000 0.006

0.000 0.006 0.006 0.006 0.000 0.000 0.000 0.012 0.006 0.000

0.006 0.006 0.006 0.000 0.074 0.070 0.044 0.076 0.000 0.000

0.008 0.000 0.000 0.008 0.000 0.000 0.008 0.008 0.000 0.008

0.000 0.000 0.008 0.000 0.016 0.000 0.000 0.000 0.083 0.051

0.061 0.074

>Non-Methylation code 202

0.000 0.005 0.005 0.005 0.010 0.000 0.000 0.005 0.000 0.000

0.000 0.005 0.000 0.005 0.005 0.010 0.005 0.000 0.000 0.000

0.063 0.067 0.068 0.076 0.028 0.000 0.000 0.009 0.000 0.000

0.000 0.009 0.009 0.009 0.000 0.009 0.000 0.000 0.000 0.028

0.009 0.000 0.000 0.000 0.060 0.045 0.063 0.055 0.006 0.000

0.000 0.000 0.013 0.000 0.006 0.000 0.006 0.013 0.000 0.006

0.000 0.019 0.006 0.000 0.000 0.000 0.000 0.000 0.085 0.047

0.071 0.053

>Non-Methylation code 203

0.000 0.000 0.000 0.000 0.000 0.000 0.006 0.000 0.013 0.006

0.000 0.006 0.000 0.000 0.013 0.000 0.025 0.006 0.000 0.000

0.048 0.068 0.076 0.067 0.006 0.000 0.000 0.006 0.000 0.000

0.000 0.006 0.006 0.012 0.000 0.000 0.000 0.006 0.012 0.006

0.006 0.000 0.000 0.000 0.071 0.065 0.075 0.057 0.000 0.000

0.020 0.007 0.000 0.000 0.000 0.000 0.007 0.000 0.000 0.007

0.007 0.007 0.007 0.020 0.000 0.000 0.000 0.000 0.050 0.080

0.059 0.065

>Non-Methylation code 204

0.000 0.000 0.007 0.000 0.000 0.000 0.000 0.000 0.007 0.007

0.000 0.000 0.000 0.013 0.013 0.007 0.013 0.007 0.000 0.000

0.063 0.074 0.077 0.047 0.000 0.000 0.014 0.000 0.000 0.005

0.000 0.000 0.009 0.000 0.000 0.000 0.009 0.000 0.009 0.009

0.005 0.000 0.000 0.000 0.051 0.084 0.072 0.066 0.000 0.000

0.000 0.007 0.000 0.007 0.007 0.000 0.007 0.007 0.000 0.007

0.007 0.000 0.007 0.000 0.021 0.000 0.000 0.000 0.068 0.059

0.052 0.079

>Non-Methylation code 205

0.009 0.009 0.000 0.000 0.000 0.009 0.000 0.009 0.000 0.009

0.000 0.000 0.009 0.000 0.000 0.027 0.018 0.009 0.000 0.000

0.056 0.058 0.059 0.051 0.010 0.000 0.000 0.005 0.000 0.000

0.000 0.000 0.000 0.021 0.000 0.000 0.005 0.000 0.010 0.016

0.000 0.000 0.000 0.000 0.066 0.072 0.061 0.066 0.000 0.000

0.000 0.007 0.007 0.007 0.014 0.000 0.000 0.021 0.000 0.000

0.000 0.000 0.000 0.021 0.000 0.000 0.000 0.007 0.070 0.044

0.077 0.058

>Non-Methylation code 206

0.000 0.005 0.010 0.005 0.000 0.005 0.000 0.005 0.000 0.005

0.000 0.000 0.010 0.000 0.005 0.000 0.005 0.005 0.005 0.000

0.066 0.053 0.072 0.076 0.005 0.005 0.000 0.000 0.000 0.009

0.000 0.005 0.000 0.009 0.000 0.000 0.005 0.000 0.009 0.000

0.005 0.000 0.000 0.005 0.075 0.067 0.075 0.062 0.000 0.000

0.010 0.000 0.000 0.031 0.010 0.000 0.000 0.010 0.000 0.000

0.010 0.000 0.000 0.010 0.010 0.000 0.000 0.000 0.060 0.058

0.050 0.072

>Non-Methylation code 207

0.000 0.000 0.000 0.000 0.000 0.000 0.006 0.006 0.011 0.006

0.000 0.011 0.000 0.000 0.011 0.000 0.017 0.006 0.000 0.000

0.046 0.072 0.080 0.064 0.007 0.000 0.000 0.007 0.000 0.000

0.000 0.014 0.007 0.014 0.000 0.000 0.000 0.007 0.007 0.007

0.007 0.000 0.000 0.000 0.065 0.078 0.066 0.047 0.000 0.000

0.010 0.010 0.000 0.000 0.000 0.000 0.010 0.000 0.000 0.019

0.010 0.010 0.000 0.029 0.000 0.000 0.000 0.000 0.050 0.066

0.050 0.072

>Non-Methylation code 208

0.006 0.000 0.006 0.000 0.000 0.006 0.000 0.000 0.006 0.012

0.000 0.000 0.012 0.006 0.006 0.006 0.006 0.006 0.000 0.000

0.042 0.071 0.080 0.061 0.000 0.005 0.000 0.005 0.000 0.005

0.005 0.000 0.005 0.010 0.000 0.000 0.005 0.000 0.010 0.010

0.005 0.000 0.000 0.000 0.069 0.068 0.069 0.062 0.005 0.000

0.000 0.005 0.000 0.005 0.000 0.005 0.005 0.000 0.000 0.000

0.015 0.000 0.005 0.010 0.000 0.000 0.005 0.000 0.060 0.063

0.071 0.079

>Non-Methylation code 209

0.000 0.000 0.000 0.006 0.006 0.006 0.012 0.006 0.000 0.018

0.000 0.000 0.000 0.000 0.000 0.012 0.000 0.000 0.006 0.006

0.068 0.048 0.079 0.058 0.009 0.009 0.000 0.000 0.000 0.009

0.000 0.009 0.000 0.000 0.000 0.000 0.009 0.000 0.000 0.038

0.019 0.009 0.000 0.000 0.057 0.058 0.053 0.052 0.011 0.000

0.000 0.005 0.000 0.000 0.000 0.000 0.000 0.021 0.000 0.000

0.005 0.000 0.011 0.011 0.000 0.000 0.000 0.000 0.071 0.070

0.077 0.052

>Non-Methylation code 210

0.000 0.000 0.000 0.006 0.000 0.006 0.006 0.000 0.006 0.006

0.000 0.006 0.012 0.000 0.006 0.000 0.018 0.000 0.000 0.000

0.074 0.056 0.057 0.074 0.000 0.000 0.006 0.000 0.000 0.000

0.000 0.000 0.006 0.012 0.000 0.000 0.000 0.012 0.012 0.000

0.012 0.006 0.000 0.000 0.057 0.078 0.077 0.054 0.000 0.000

0.016 0.000 0.000 0.005 0.000 0.000 0.010 0.000 0.000 0.000

0.005 0.000 0.010 0.010 0.005 0.000 0.000 0.000 0.052 0.084

0.066 0.069

>Non-Methylation code 211

0.000 0.000 0.018 0.006 0.000 0.000 0.000 0.000 0.006 0.000

0.000 0.000 0.012 0.006 0.006 0.023 0.000 0.000 0.000 0.000

0.049 0.075 0.065 0.068 0.000 0.000 0.000 0.000 0.000 0.000

0.013 0.000 0.006 0.006 0.000 0.006 0.006 0.000 0.013 0.000

0.019 0.006 0.000 0.000 0.050 0.060 0.079 0.069 0.007 0.000

0.000 0.007 0.000 0.000 0.000 0.007 0.000 0.007 0.000 0.000

0.000 0.007 0.014 0.007 0.014 0.000 0.000 0.000 0.075 0.076

0.053 0.059

>Non-Methylation code 212

0.005 0.005 0.000 0.000 0.000 0.009 0.000 0.000 0.000 0.009

0.000 0.000 0.009 0.005 0.009 0.005 0.005 0.000 0.000 0.000

0.046 0.076 0.081 0.070 0.015 0.000 0.005 0.005 0.000 0.000

0.000 0.000 0.000 0.000 0.000 0.000 0.015 0.000 0.010 0.005

0.000 0.005 0.005 0.000 0.075 0.060 0.070 0.066 0.000 0.000

0.007 0.000 0.000 0.014 0.000 0.000 0.007 0.007 0.000 0.000

0.021 0.007 0.007 0.007 0.007 0.000 0.000 0.000 0.066 0.052

0.057 0.074

>Non-Methylation code 213

0.009 0.005 0.005 0.000 0.000 0.005 0.005 0.000 0.005 0.000

0.000 0.000 0.014 0.000 0.005 0.005 0.000 0.005 0.000 0.000

0.048 0.070 0.077 0.077 0.005 0.000 0.000 0.000 0.005 0.005

0.000 0.005 0.000 0.000 0.000 0.005 0.018 0.000 0.009 0.000

0.005 0.000 0.000 0.000 0.079 0.062 0.078 0.060 0.000 0.006

0.000 0.006 0.000 0.012 0.000 0.000 0.000 0.012 0.000 0.000

0.006 0.006 0.006 0.012 0.006 0.000 0.000 0.000 0.045 0.061

0.073 0.079

>Non-Methylation code 214

0.055 0.000 0.000 0.000 0.000 0.022 0.000 0.000 0.000 0.011

0.000 0.000 0.033 0.000 0.000 0.022 0.000 0.000 0.000 0.000

0.046 0.049 0.042 0.052 0.020 0.000 0.000 0.007 0.000 0.000

0.007 0.000 0.000 0.020 0.000 0.000 0.026 0.000 0.007 0.000

0.000 0.000 0.000 0.000 0.068 0.058 0.065 0.057 0.000 0.009

0.000 0.000 0.000 0.004 0.000 0.000 0.000 0.000 0.000 0.000

0.013 0.004 0.013 0.000 0.004 0.000 0.004 0.000 0.062 0.063

0.086 0.071

>Non-Methylation code 215

0.012 0.000 0.000 0.000 0.000 0.000 0.006 0.006 0.006 0.000

0.000 0.006 0.018 0.000 0.012 0.006 0.000 0.000 0.000 0.000

0.065 0.080 0.048 0.067 0.004 0.000 0.000 0.004 0.004 0.000

0.000 0.004 0.000 0.000 0.000 0.004 0.009 0.009 0.013 0.000

0.004 0.000 0.000 0.000 0.046 0.071 0.080 0.079 0.007 0.000

0.000 0.007 0.000 0.000 0.000 0.007 0.007 0.007 0.000 0.000

0.013 0.007 0.000 0.020 0.000 0.000 0.000 0.007 0.063 0.053

0.062 0.074

>Non-Methylation code 216

0.000 0.008 0.000 0.000 0.004 0.000 0.000 0.004 0.000 0.000

0.000 0.000 0.012 0.012 0.008 0.000 0.000 0.000 0.004 0.000

0.045 0.066 0.091 0.081 0.035 0.000 0.000 0.000 0.000 0.009

0.000 0.000 0.009 0.009 0.000 0.000 0.017 0.000 0.000 0.026

0.000 0.000 0.000 0.009 0.059 0.049 0.048 0.065 0.019 0.000

0.000 0.000 0.000 0.000 0.000 0.009 0.000 0.028 0.000 0.009

0.046 0.000 0.000 0.000 0.000 0.000 0.000 0.000 0.064 0.066

0.036 0.055

>Non-Methylation code 217

0.006 0.000 0.000 0.006 0.000 0.000 0.000 0.006 0.006 0.006

0.000 0.000 0.019 0.006 0.000 0.019 0.000 0.000 0.000 0.006

0.058 0.055 0.065 0.073 0.012 0.000 0.000 0.000 0.000 0.000

0.006 0.006 0.006 0.000 0.000 0.006 0.018 0.000 0.012 0.006

0.000 0.000 0.000 0.000 0.065 0.080 0.048 0.067 0.005 0.000

0.000 0.005 0.005 0.000 0.000 0.005 0.000 0.000 0.000 0.005

0.010 0.010 0.010 0.000 0.005 0.000 0.000 0.000 0.051 0.075

0.075 0.072

>Non-Methylation code 218

0.021 0.000 0.005 0.005 0.000 0.000 0.005 0.000 0.000 0.000

0.000 0.000 0.010 0.000 0.005 0.005 0.000 0.005 0.005 0.000

0.068 0.059 0.079 0.061 0.000 0.000 0.000 0.000 0.000 0.013

0.000 0.000 0.006 0.006 0.000 0.000 0.026 0.006 0.006 0.006

0.013 0.000 0.000 0.000 0.070 0.060 0.051 0.069 0.005 0.005

0.000 0.000 0.000 0.010 0.000 0.000 0.000 0.010 0.000 0.000

0.014 0.000 0.010 0.005 0.000 0.000 0.000 0.000 0.042 0.070

0.078 0.086

>Non-Methylation code 219

0.005 0.005 0.011 0.000 0.000 0.005 0.000 0.005 0.000 0.005

0.000 0.000 0.005 0.000 0.011 0.000 0.005 0.000 0.000 0.000

0.083 0.060 0.064 0.068 0.007 0.014 0.007 0.000 0.000 0.007

0.000 0.000 0.000 0.014 0.000 0.000 0.014 0.000 0.000 0.000

0.014 0.000 0.007 0.000 0.062 0.075 0.064 0.050 0.011 0.005

0.000 0.000 0.000 0.005 0.011 0.000 0.000 0.005 0.005 0.000

0.000 0.000 0.011 0.000 0.000 0.000 0.000 0.000 0.069 0.065

0.063 0.083

>Non-Methylation code 220

0.008 0.000 0.000 0.008 0.000 0.000 0.000 0.000 0.008 0.030

0.000 0.000 0.000 0.008 0.008 0.023 0.000 0.000 0.000 0.000

0.058 0.083 0.063 0.038 0.000 0.004 0.000 0.000 0.004 0.004

0.000 0.000 0.000 0.013 0.000 0.004 0.004 0.000 0.009 0.009

0.000 0.000 0.004 0.000 0.074 0.084 0.065 0.054 0.007 0.007

0.000 0.013 0.007 0.007 0.000 0.007 0.000 0.000 0.000 0.000

0.013 0.000 0.000 0.013 0.000 0.007 0.000 0.000 0.056 0.056

0.077 0.065

>Non-Methylation code 221

0.008 0.000 0.000 0.000 0.000 0.000 0.008 0.000 0.000 0.008

0.008 0.000 0.046 0.000 0.000 0.015 0.008 0.000 0.000 0.000

0.061 0.060 0.058 0.055 0.000 0.016 0.000 0.000 0.000 0.000

0.016 0.008 0.000 0.008 0.000 0.000 0.049 0.008 0.000 0.000

0.000 0.000 0.000 0.000 0.049 0.060 0.067 0.051 0.017 0.000

0.000 0.000 0.000 0.000 0.009 0.000 0.000 0.017 0.000 0.000

0.026 0.000 0.000 0.017 0.017 0.000 0.000 0.000 0.049 0.054

0.054 0.072

>Non-Methylation code 222

0.000 0.000 0.000 0.012 0.000 0.006 0.006 0.000 0.000 0.006

0.000 0.012 0.006 0.000 0.000 0.012 0.000 0.006 0.006 0.000

0.051 0.060 0.076 0.075 0.011 0.006 0.000 0.006 0.006 0.006

0.000 0.006 0.000 0.006 0.000 0.000 0.000 0.006 0.006 0.006

0.011 0.000 0.000 0.000 0.057 0.067 0.073 0.062 0.006 0.006

0.000 0.000 0.006 0.006 0.000 0.000 0.000 0.000 0.000 0.000

0.006 0.017 0.012 0.006 0.000 0.000 0.000 0.000 0.072 0.055

0.065 0.078

>Non-Methylation code 223

0.000 0.000 0.000 0.000 0.006 0.000 0.012 0.000 0.006 0.000

0.006 0.000 0.018 0.000 0.006 0.012 0.006 0.000 0.000 0.000

0.067 0.066 0.054 0.076 0.006 0.000 0.000 0.000 0.006 0.006

0.013 0.000 0.000 0.000 0.000 0.000 0.019 0.000 0.000 0.006

0.013 0.000 0.006 0.000 0.054 0.064 0.065 0.075 0.005 0.000

0.000 0.011 0.005 0.000 0.011 0.005 0.000 0.000 0.000 0.000

0.016 0.000 0.005 0.000 0.005 0.000 0.000 0.000 0.067 0.074

0.062 0.065

>Non-Methylation code 224

0.017 0.006 0.000 0.006 0.006 0.006 0.000 0.006 0.000 0.006

0.000 0.000 0.000 0.000 0.006 0.006 0.011 0.000 0.000 0.000

0.055 0.073 0.083 0.049 0.006 0.006 0.000 0.000 0.006 0.006

0.006 0.000 0.000 0.006 0.000 0.000 0.006 0.019 0.006 0.006

0.000 0.000 0.000 0.000 0.082 0.046 0.063 0.067 0.000 0.000

0.000 0.007 0.000 0.007 0.007 0.007 0.000 0.007 0.000 0.013

0.007 0.000 0.000 0.007 0.000 0.007 0.007 0.000 0.058 0.056

0.070 0.077

>Non-Methylation code 225

0.000 0.005 0.000 0.005 0.005 0.005 0.000 0.000 0.000 0.010

0.000 0.005 0.010 0.000 0.005 0.010 0.000 0.005 0.000 0.000

0.073 0.072 0.051 0.075 0.007 0.007 0.000 0.007 0.014 0.000

0.000 0.007 0.000 0.007 0.000 0.000 0.014 0.007 0.000 0.014

0.000 0.007 0.000 0.000 0.057 0.065 0.063 0.060 0.014 0.000

0.000 0.000 0.007 0.000 0.000 0.000 0.000 0.022 0.000 0.000

0.000 0.007 0.014 0.014 0.000 0.000 0.000 0.000 0.066 0.063

0.063 0.061

>Non-Methylation code 226

0.005 0.000 0.000 0.005 0.005 0.005 0.000 0.000 0.005 0.005

0.000 0.000 0.005 0.000 0.005 0.010 0.005 0.005 0.005 0.000

0.056 0.064 0.077 0.072 0.000 0.000 0.006 0.006 0.006 0.006

0.000 0.000 0.000 0.006 0.000 0.000 0.012 0.006 0.006 0.012

0.000 0.006 0.006 0.000 0.056 0.074 0.065 0.061 0.000 0.005

0.000 0.005 0.000 0.011 0.000 0.005 0.000 0.005 0.000 0.005

0.005 0.000 0.005 0.011 0.000 0.005 0.000 0.000 0.052 0.066

0.069 0.081

>Non-Methylation code 227

0.000 0.000 0.000 0.008 0.015 0.015 0.000 0.008 0.000 0.008

0.000 0.000 0.015 0.000 0.000 0.023 0.000 0.000 0.000 0.000

0.071 0.070 0.037 0.065 0.017 0.008 0.000 0.000 0.008 0.000

0.000 0.000 0.000 0.042 0.000 0.000 0.008 0.000 0.000 0.025

0.000 0.000 0.000 0.000 0.066 0.046 0.044 0.068 0.005 0.005

0.000 0.000 0.005 0.000 0.000 0.000 0.000 0.011 0.000 0.000

0.011 0.000 0.005 0.000 0.000 0.005 0.011 0.005 0.078 0.061

0.070 0.061

>Non-Methylation code 228

0.000 0.000 0.000 0.006 0.000 0.006 0.006 0.006 0.000 0.006

0.000 0.012 0.006 0.000 0.000 0.012 0.000 0.006 0.006 0.000

0.056 0.057 0.074 0.072 0.011 0.006 0.000 0.006 0.006 0.006

0.000 0.006 0.000 0.006 0.000 0.000 0.000 0.006 0.006 0.006

0.011 0.000 0.000 0.000 0.058 0.070 0.071 0.061 0.007 0.007

0.000 0.000 0.007 0.007 0.000 0.000 0.000 0.007 0.000 0.000

0.007 0.021 0.007 0.007 0.000 0.000 0.000 0.000 0.083 0.041

0.059 0.074

>Non-Methylation code 229

0.000 0.005 0.000 0.005 0.005 0.000 0.000 0.005 0.005 0.010

0.000 0.000 0.015 0.000 0.005 0.005 0.000 0.005 0.000 0.000

0.075 0.051 0.079 0.064 0.005 0.000 0.000 0.005 0.014 0.000

0.000 0.000 0.000 0.009 0.000 0.000 0.005 0.000 0.009 0.009

0.000 0.000 0.005 0.000 0.059 0.069 0.077 0.068 0.000 0.000

0.009 0.000 0.009 0.009 0.000 0.000 0.000 0.009 0.000 0.000

0.018 0.000 0.000 0.035 0.000 0.009 0.000 0.000 0.060 0.065

0.055 0.057

>Non-Methylation code 230

0.005 0.000 0.000 0.000 0.000 0.015 0.000 0.000 0.000 0.010

0.000 0.000 0.005 0.000 0.015 0.005 0.000 0.005 0.000 0.005

0.064 0.080 0.066 0.059 0.013 0.000 0.000 0.013 0.013 0.013

0.000 0.000 0.000 0.000 0.000 0.000 0.013 0.006 0.000 0.006

0.006 0.000 0.000 0.000 0.064 0.065 0.052 0.070 0.000 0.000

0.000 0.000 0.005 0.010 0.000 0.000 0.010 0.005 0.000 0.000

0.010 0.005 0.010 0.005 0.000 0.000 0.000 0.000 0.067 0.052

0.077 0.079

>Non-Methylation code 231

0.009 0.000 0.000 0.000 0.009 0.000 0.009 0.009 0.009 0.000

0.000 0.000 0.009 0.009 0.000 0.034 0.009 0.000 0.000 0.000

0.059 0.047 0.060 0.065 0.000 0.006 0.000 0.000 0.000 0.000

0.006 0.006 0.000 0.006 0.000 0.000 0.025 0.006 0.006 0.019

0.000 0.000 0.000 0.000 0.048 0.061 0.066 0.077 0.000 0.005

0.000 0.000 0.005 0.000 0.005 0.005 0.000 0.000 0.000 0.005

0.015 0.005 0.005 0.000 0.000 0.010 0.000 0.000 0.075 0.063

0.058 0.077

>Non-Methylation code 232

0.012 0.000 0.000 0.012 0.006 0.000 0.012 0.006 0.000 0.000

0.000 0.000 0.023 0.000 0.000 0.000 0.006 0.000 0.000 0.000

0.064 0.070 0.060 0.064 0.000 0.000 0.000 0.000 0.006 0.000

0.012 0.000 0.006 0.000 0.006 0.000 0.023 0.000 0.006 0.012

0.006 0.000 0.000 0.000 0.065 0.063 0.060 0.071 0.007 0.000

0.000 0.000 0.007 0.007 0.014 0.000 0.000 0.000 0.000 0.000

0.028 0.000 0.000 0.007 0.007 0.000 0.000 0.000 0.061 0.057

0.065 0.073

>Non-Methylation code 233

0.014 0.000 0.000 0.000 0.021 0.000 0.014 0.000 0.000 0.007

0.007 0.000 0.000 0.000 0.007 0.000 0.000 0.000 0.000 0.007

0.052 0.067 0.065 0.071 0.000 0.013 0.000 0.007 0.000 0.000

0.000 0.000 0.000 0.007 0.013 0.000 0.007 0.000 0.000 0.026

0.007 0.000 0.007 0.000 0.055 0.050 0.070 0.074 0.008 0.023

0.000 0.000 0.000 0.008 0.000 0.000 0.000 0.023 0.000 0.000

0.015 0.008 0.000 0.000 0.000 0.008 0.000 0.000 0.076 0.046

0.066 0.053

>Non-Methylation code 234

0.006 0.000 0.006 0.000 0.012 0.000 0.006 0.000 0.006 0.000

0.000 0.006 0.000 0.018 0.006 0.006 0.000 0.000 0.000 0.006

0.065 0.080 0.066 0.046 0.006 0.000 0.000 0.000 0.006 0.000

0.000 0.006 0.000 0.000 0.000 0.018 0.006 0.000 0.006 0.012

0.018 0.000 0.000 0.000 0.068 0.060 0.064 0.061 0.011 0.000

0.000 0.000 0.000 0.011 0.000 0.011 0.000 0.033 0.000 0.000

0.011 0.022 0.000 0.011 0.022 0.000 0.000 0.000 0.054 0.056

0.061 0.030

>Non-Methylation code 235

0.007 0.007 0.000 0.000 0.007 0.013 0.000 0.000 0.007 0.007

0.000 0.000 0.000 0.000 0.000 0.013 0.007 0.000 0.000 0.007

0.063 0.071 0.065 0.061 0.022 0.000 0.005 0.000 0.000 0.000

0.000 0.005 0.000 0.005 0.000 0.000 0.011 0.000 0.011 0.000

0.000 0.000 0.005 0.000 0.075 0.069 0.062 0.062 0.000 0.006

0.000 0.000 0.006 0.011 0.006 0.000 0.006 0.017 0.000 0.000

0.000 0.006 0.006 0.000 0.000 0.000 0.000 0.000 0.087 0.055

0.084 0.047

>Non-Methylation code 236

0.000 0.006 0.012 0.000 0.000 0.000 0.006 0.018 0.000 0.006

0.000 0.000 0.000 0.000 0.000 0.006 0.006 0.000 0.000 0.012

0.064 0.053 0.075 0.067 0.011 0.000 0.000 0.000 0.000 0.000

0.000 0.000 0.011 0.011 0.000 0.000 0.000 0.011 0.000 0.011

0.056 0.011 0.000 0.011 0.055 0.065 0.046 0.033 0.000 0.000

0.000 0.000 0.000 0.000 0.005 0.005 0.005 0.016 0.000 0.005

0.011 0.000 0.011 0.000 0.000 0.000 0.000 0.005 0.067 0.063

0.064 0.076

>Non-Methylation code 237

0.000 0.000 0.000 0.000 0.000 0.012 0.000 0.000 0.000 0.004

0.000 0.000 0.008 0.000 0.020 0.004 0.000 0.004 0.000 0.000

0.068 0.082 0.071 0.061 0.062 0.010 0.000 0.000 0.010 0.000

0.000 0.000 0.000 0.021 0.000 0.000 0.000 0.000 0.000 0.000

0.000 0.010 0.000 0.000 0.056 0.043 0.054 0.066 0.005 0.005

0.000 0.000 0.000 0.000 0.000 0.000 0.000 0.005 0.000 0.000

0.005 0.005 0.011 0.022 0.000 0.000 0.005 0.000 0.073 0.063

0.065 0.068

>Non-Methylation code 238

0.008 0.000 0.000 0.000 0.000 0.032 0.008 0.016 0.000 0.000

0.000 0.008 0.000 0.000 0.008 0.016 0.008 0.000 0.000 0.000

0.053 0.052 0.062 0.062 0.016 0.000 0.008 0.008 0.000 0.016

0.000 0.008 0.000 0.000 0.000 0.000 0.008 0.016 0.000 0.016

0.008 0.000 0.000 0.000 0.044 0.060 0.064 0.062 0.000 0.000

0.005 0.000 0.000 0.005 0.005 0.000 0.005 0.005 0.000 0.005

0.005 0.005 0.015 0.000 0.005 0.000 0.000 0.000 0.091 0.040

0.081 0.059

>Non-Methylation code 239

0.021 0.000 0.007 0.007 0.000 0.007 0.000 0.007 0.007 0.007

0.007 0.000 0.007 0.007 0.000 0.000 0.007 0.000 0.000 0.000

0.052 0.063 0.062 0.067 0.000 0.000 0.000 0.000 0.000 0.000

0.000 0.000 0.005 0.005 0.000 0.000 0.009 0.000 0.019 0.009

0.005 0.000 0.005 0.000 0.091 0.065 0.077 0.043 0.000 0.005

0.005 0.000 0.000 0.015 0.005 0.000 0.000 0.000 0.000 0.005

0.005 0.005 0.010 0.005 0.000 0.000 0.000 0.000 0.054 0.065

0.087 0.069

>Non-Methylation code 240

0.015 0.000 0.000 0.000 0.007 0.007 0.000 0.007 0.007 0.007

0.000 0.015 0.000 0.000 0.000 0.015 0.000 0.007 0.000 0.000

0.068 0.057 0.055 0.066 0.000 0.000 0.004 0.000 0.000 0.000

0.000 0.004 0.000 0.009 0.000 0.000 0.004 0.000 0.017 0.004

0.004 0.000 0.000 0.004 0.088 0.042 0.085 0.067 0.000 0.005

0.005 0.005 0.000 0.010 0.000 0.010 0.000 0.005 0.000 0.000

0.000 0.010 0.005 0.000 0.000 0.005 0.000 0.000 0.066 0.056

0.071 0.078

>Non-Methylation code 241

0.006 0.000 0.000 0.000 0.006 0.000 0.000 0.006 0.000 0.006

0.000 0.019 0.006 0.000 0.006 0.006 0.019 0.000 0.000 0.000

0.072 0.052 0.071 0.058 0.011 0.000 0.000 0.000 0.000 0.011

0.000 0.011 0.000 0.033 0.000 0.000 0.011 0.022 0.000 0.011

0.022 0.000 0.000 0.000 0.054 0.056 0.061 0.030 0.007 0.000

0.007 0.000 0.007 0.000 0.007 0.000 0.007 0.000 0.000 0.007

0.000 0.020 0.007 0.007 0.000 0.000 0.000 0.007 0.061 0.071

0.068 0.054

>Non-Methylation code 242

0.006 0.006 0.000 0.000 0.000 0.000 0.000 0.006 0.006 0.017

0.000 0.000 0.000 0.000 0.006 0.000 0.012 0.006 0.000 0.006

0.069 0.073 0.056 0.066 0.009 0.009 0.000 0.000 0.004 0.000

0.000 0.004 0.004 0.009 0.000 0.000 0.004 0.000 0.004 0.004

0.000 0.000 0.000 0.004 0.065 0.063 0.085 0.063 0.008 0.008

0.008 0.000 0.008 0.000 0.000 0.008 0.000 0.025 0.000 0.000

0.008 0.000 0.000 0.008 0.000 0.000 0.000 0.008 0.066 0.060

0.048 0.068

>Non-Methylation code 243

0.011 0.005 0.000 0.000 0.000 0.000 0.000 0.005 0.005 0.016

0.000 0.000 0.000 0.000 0.005 0.000 0.005 0.000 0.005 0.005

0.071 0.061 0.056 0.080 0.009 0.009 0.000 0.000 0.004 0.004

0.004 0.004 0.004 0.004 0.000 0.000 0.004 0.000 0.004 0.004

0.000 0.000 0.000 0.000 0.074 0.065 0.075 0.063 0.010 0.000

0.010 0.000 0.010 0.010 0.000 0.000 0.000 0.030 0.000 0.000

0.010 0.000 0.000 0.010 0.000 0.000 0.000 0.010 0.060 0.074

0.040 0.061

>Non-Methylation code 244

0.015 0.015 0.000 0.000 0.000 0.023 0.000 0.000 0.000 0.008

0.000 0.000 0.008 0.008 0.000 0.000 0.008 0.008 0.000 0.000

0.073 0.046 0.085 0.037 0.028 0.000 0.007 0.000 0.000 0.000

0.014 0.000 0.000 0.000 0.000 0.007 0.000 0.014 0.000 0.007

0.000 0.007 0.000 0.007 0.062 0.065 0.050 0.066 0.000 0.000

0.000 0.000 0.000 0.000 0.000 0.006 0.000 0.012 0.006 0.006

0.000 0.000 0.024 0.006 0.012 0.000 0.000 0.000 0.082 0.038

0.086 0.056

>Non-Methylation code 245

0.000 0.004 0.004 0.000 0.000 0.012 0.000 0.004 0.000 0.000

0.000 0.004 0.000 0.008 0.008 0.000 0.000 0.004 0.004 0.000

0.070 0.065 0.078 0.068 0.023 0.000 0.000 0.000 0.008 0.023

0.000 0.015 0.008 0.000 0.000 0.008 0.000 0.000 0.000 0.008

0.000 0.008 0.000 0.000 0.069 0.052 0.042 0.072 0.000 0.000

0.005 0.000 0.000 0.010 0.000 0.000 0.000 0.010 0.000 0.000

0.005 0.005 0.010 0.010 0.005 0.000 0.000 0.000 0.058 0.050

0.078 0.088

>Non-Methylation code 246

0.017 0.012 0.000 0.000 0.000 0.023 0.000 0.000 0.006 0.000

0.000 0.000 0.006 0.000 0.006 0.000 0.000 0.006 0.000 0.000

0.069 0.050 0.067 0.072 0.021 0.000 0.000 0.011 0.005 0.000

0.000 0.000 0.005 0.005 0.000 0.000 0.005 0.005 0.005 0.000

0.000 0.000 0.000 0.000 0.067 0.075 0.065 0.063 0.000 0.004

0.000 0.000 0.000 0.004 0.000 0.000 0.000 0.004 0.000 0.000

0.004 0.004 0.013 0.013 0.000 0.000 0.004 0.000 0.079 0.084

0.066 0.053

>Non-Methylation code 247

0.019 0.013 0.000 0.000 0.000 0.006 0.013 0.000 0.000 0.000

0.006 0.000 0.006 0.006 0.000 0.006 0.000 0.000 0.000 0.000

0.054 0.060 0.062 0.081 0.005 0.005 0.005 0.000 0.000 0.005

0.000 0.000 0.000 0.010 0.000 0.000 0.010 0.000 0.010 0.000

0.010 0.005 0.000 0.000 0.072 0.060 0.067 0.072 0.007 0.000

0.000 0.000 0.007 0.015 0.007 0.000 0.000 0.022 0.000 0.000

0.007 0.000 0.007 0.007 0.007 0.000 0.000 0.000 0.075 0.050

0.058 0.062

>Non-Methylation code 248

0.020 0.007 0.000 0.000 0.000 0.000 0.000 0.000 0.000 0.000

0.000 0.000 0.020 0.007 0.000 0.007 0.020 0.000 0.007 0.000

0.074 0.049 0.052 0.072 0.004 0.000 0.000 0.000 0.000 0.004

0.004 0.000 0.000 0.000 0.000 0.000 0.022 0.000 0.017 0.004

0.000 0.000 0.000 0.000 0.055 0.061 0.067 0.093 0.012 0.000

0.000 0.000 0.000 0.012 0.012 0.000 0.000 0.012 0.000 0.000

0.006 0.000 0.006 0.000 0.006 0.000 0.000 0.000 0.040 0.064

0.084 0.077

>Non-Methylation code 249

0.010 0.005 0.000 0.005 0.000 0.021 0.000 0.000 0.005 0.000

0.000 0.000 0.005 0.000 0.010 0.000 0.000 0.005 0.000 0.000

0.079 0.048 0.071 0.069 0.011 0.000 0.000 0.011 0.006 0.000

0.000 0.000 0.006 0.000 0.000 0.000 0.006 0.006 0.011 0.000

0.000 0.006 0.000 0.000 0.077 0.067 0.063 0.065 0.000 0.005

0.000 0.005 0.000 0.005 0.000 0.000 0.000 0.005 0.000 0.000

0.000 0.005 0.021 0.011 0.000 0.000 0.000 0.000 0.093 0.054

0.092 0.037

>Non-Methylation code 250

0.018 0.000 0.000 0.005 0.000 0.000 0.000 0.005 0.000 0.000

0.000 0.000 0.000 0.000 0.018 0.005 0.009 0.000 0.000 0.000

0.039 0.061 0.085 0.090 0.050 0.000 0.000 0.000 0.000 0.000

0.000 0.000 0.000 0.000 0.000 0.000 0.060 0.000 0.000 0.020

0.000 0.000 0.000 0.000 0.040 0.050 0.054 0.061 0.005 0.000

0.000 0.000 0.000 0.000 0.010 0.000 0.000 0.000 0.000 0.000

0.015 0.005 0.026 0.000 0.000 0.000 0.000 0.000 0.065 0.041

0.088 0.077

>Non-Methylation code 251

0.012 0.000 0.000 0.023 0.000 0.058 0.000 0.000 0.000 0.012

0.000 0.000 0.012 0.000 0.000 0.012 0.000 0.012 0.000 0.000

0.033 0.050 0.049 0.061 0.016 0.000 0.000 0.000 0.000 0.005

0.000 0.000 0.010 0.005 0.000 0.000 0.000 0.000 0.010 0.000

0.000 0.005 0.010 0.000 0.050 0.078 0.068 0.075 0.004 0.000

0.000 0.000 0.000 0.012 0.000 0.000 0.004 0.000 0.000 0.000

0.000 0.000 0.016 0.004 0.000 0.000 0.008 0.000 0.075 0.084

0.075 0.053

>Non-Methylation code 252

0.012 0.000 0.000 0.000 0.006 0.006 0.000 0.006 0.006 0.000

0.000 0.006 0.006 0.012 0.006 0.006 0.006 0.000 0.000 0.000

0.082 0.069 0.058 0.045 0.009 0.000 0.000 0.000 0.000 0.000

0.000 0.000 0.000 0.005 0.000 0.000 0.009 0.005 0.014 0.009

0.005 0.005 0.000 0.000 0.040 0.051 0.094 0.087 0.006 0.000

0.011 0.000 0.000 0.000 0.000 0.000 0.000 0.000 0.000 0.000

0.011 0.006 0.017 0.006 0.000 0.006 0.000 0.000 0.072 0.068

0.078 0.054

>Non-Methylation code 253

0.006 0.006 0.000 0.000 0.006 0.000 0.012 0.000 0.000 0.006

0.000 0.000 0.012 0.000 0.006 0.000 0.012 0.000 0.000 0.006

0.056 0.083 0.057 0.064 0.006 0.000 0.000 0.000 0.006 0.006

0.012 0.000 0.000 0.000 0.000 0.006 0.012 0.000 0.006 0.000

0.018 0.000 0.000 0.000 0.082 0.042 0.070 0.068 0.000 0.000

0.000 0.000 0.000 0.000 0.009 0.009 0.000 0.009 0.009 0.009

0.026 0.009 0.000 0.009 0.009 0.009 0.000 0.000 0.063 0.056

0.059 0.053

>Non-Methylation code 254

0.005 0.011 0.000 0.000 0.000 0.005 0.005 0.000 0.000 0.011

0.000 0.005 0.011 0.000 0.005 0.005 0.000 0.000 0.000 0.000

0.090 0.057 0.077 0.045 0.005 0.011 0.000 0.000 0.000 0.005

0.005 0.000 0.000 0.000 0.000 0.000 0.011 0.000 0.005 0.000

0.011 0.005 0.005 0.000 0.083 0.063 0.061 0.061 0.007 0.007

0.000 0.000 0.000 0.007 0.000 0.000 0.000 0.007 0.000 0.000

0.014 0.007 0.007 0.007 0.007 0.000 0.000 0.000 0.080 0.051

0.069 0.061

>Non-Methylation code 255

0.007 0.007 0.000 0.000 0.000 0.007 0.000 0.000 0.000 0.014

0.000 0.000 0.014 0.007 0.007 0.007 0.007 0.000 0.000 0.000

0.080 0.053 0.065 0.059 0.005 0.011 0.000 0.000 0.000 0.005

0.005 0.000 0.000 0.011 0.000 0.005 0.011 0.000 0.005 0.005

0.000 0.000 0.000 0.000 0.090 0.057 0.077 0.045 0.006 0.012

0.000 0.000 0.000 0.000 0.006 0.000 0.000 0.000 0.000 0.000

0.012 0.000 0.006 0.000 0.012 0.006 0.006 0.000 0.081 0.054

0.063 0.068

>Non-Methylation code 256

0.012 0.012 0.000 0.000 0.000 0.000 0.006 0.000 0.000 0.000

0.000 0.000 0.012 0.000 0.006 0.000 0.012 0.006 0.006 0.000

0.085 0.058 0.059 0.062 0.007 0.007 0.000 0.000 0.000 0.007

0.000 0.000 0.000 0.014 0.000 0.000 0.014 0.007 0.007 0.007

0.007 0.000 0.000 0.000 0.080 0.053 0.065 0.059 0.006 0.011

0.000 0.000 0.000 0.000 0.006 0.000 0.000 0.011 0.000 0.006

0.011 0.000 0.006 0.006 0.000 0.000 0.000 0.000 0.088 0.062

0.079 0.042

>Non-Methylation code 257

0.000 0.006 0.000 0.000 0.000 0.000 0.000 0.006 0.000 0.006

0.000 0.000 0.011 0.011 0.011 0.006 0.006 0.000 0.000 0.000

0.073 0.063 0.079 0.055 0.005 0.005 0.014 0.000 0.000 0.005

0.000 0.000 0.005 0.005 0.000 0.000 0.010 0.000 0.005 0.010

0.000 0.000 0.000 0.000 0.055 0.069 0.062 0.085 0.008 0.000

0.008 0.000 0.000 0.000 0.000 0.016 0.000 0.016 0.000 0.008

0.024 0.000 0.000 0.000 0.008 0.000 0.000 0.000 0.061 0.065

0.063 0.058

>Non-Methylation code 258

0.000 0.008 0.000 0.004 0.000 0.000 0.000 0.004 0.000 0.000

0.000 0.000 0.012 0.004 0.012 0.004 0.004 0.000 0.000 0.000

0.068 0.087 0.060 0.064 0.015 0.000 0.000 0.000 0.000 0.010

0.005 0.000 0.005 0.005 0.000 0.000 0.000 0.000 0.015 0.005

0.000 0.005 0.000 0.000 0.059 0.061 0.056 0.092 0.008 0.000

0.008 0.008 0.000 0.008 0.008 0.000 0.000 0.008 0.000 0.000

0.015 0.008 0.000 0.008 0.008 0.000 0.000 0.000 0.068 0.063

0.066 0.052

>Non-Methylation code 259

0.015 0.000 0.000 0.000 0.000 0.010 0.005 0.000 0.005 0.005

0.000 0.000 0.000 0.000 0.015 0.005 0.000 0.005 0.000 0.000

0.059 0.061 0.056 0.092 0.007 0.000 0.007 0.007 0.000 0.007

0.007 0.000 0.007 0.007 0.000 0.000 0.013 0.007 0.000 0.007

0.007 0.000 0.000 0.000 0.066 0.068 0.060 0.059 0.000 0.009

0.000 0.004 0.000 0.000 0.000 0.004 0.000 0.000 0.000 0.000

0.013 0.004 0.013 0.000 0.004 0.000 0.000 0.000 0.067 0.088

0.060 0.068

>Non-Methylation code 260

0.000 0.009 0.000 0.000 0.005 0.005 0.000 0.000 0.000 0.014

0.000 0.000 0.000 0.000 0.009 0.009 0.000 0.000 0.000 0.005

0.071 0.076 0.077 0.052 0.013 0.006 0.006 0.000 0.006 0.000

0.000 0.000 0.000 0.000 0.000 0.006 0.000 0.000 0.000 0.006

0.006 0.019 0.000 0.006 0.065 0.062 0.071 0.060 0.000 0.000

0.000 0.000 0.000 0.000 0.000 0.028 0.000 0.028 0.014 0.000

0.000 0.014 0.000 0.028 0.000 0.014 0.014 0.000 0.052 0.047

0.040 0.054

>Non-Methylation code 261

0.004 0.004 0.004 0.004 0.000 0.004 0.004 0.000 0.000 0.000

0.004 0.004 0.000 0.000 0.018 0.004 0.000 0.000 0.000 0.000

0.067 0.069 0.058 0.081 0.036 0.006 0.006 0.000 0.000 0.000

0.000 0.000 0.000 0.000 0.000 0.000 0.000 0.006 0.006 0.000

0.018 0.000 0.000 0.000 0.059 0.069 0.054 0.074 0.006 0.000

0.000 0.000 0.000 0.006 0.000 0.000 0.006 0.006 0.000 0.000

0.018 0.006 0.018 0.000 0.006 0.000 0.000 0.000 0.072 0.049

0.077 0.064

>Non-Methylation code 262

0.000 0.000 0.000 0.000 0.000 0.000 0.000 0.005 0.000 0.005

0.000 0.000 0.016 0.011 0.011 0.005 0.005 0.000 0.005 0.000

0.071 0.076 0.069 0.055 0.000 0.005 0.010 0.000 0.000 0.005

0.000 0.000 0.005 0.010 0.000 0.000 0.010 0.000 0.005 0.010

0.000 0.005 0.000 0.000 0.051 0.064 0.065 0.086 0.022 0.000

0.007 0.000 0.000 0.000 0.000 0.007 0.000 0.015 0.000 0.007

0.022 0.000 0.000 0.000 0.007 0.000 0.000 0.000 0.061 0.056

0.058 0.069

>Non-Methylation code 263

0.000 0.000 0.005 0.010 0.005 0.015 0.010 0.000 0.000 0.005

0.000 0.000 0.000 0.000 0.005 0.000 0.005 0.005 0.000 0.000

0.066 0.076 0.069 0.057 0.019 0.000 0.000 0.000 0.000 0.000

0.000 0.006 0.000 0.006 0.006 0.006 0.006 0.000 0.006 0.013

0.006 0.006 0.000 0.000 0.062 0.055 0.050 0.082 0.000 0.000

0.000 0.000 0.005 0.005 0.005 0.000 0.000 0.000 0.000 0.000

0.010 0.000 0.014 0.005 0.005 0.000 0.005 0.000 0.077 0.072

0.056 0.076

>Non-Methylation code 264

0.005 0.005 0.000 0.005 0.005 0.005 0.000 0.005 0.000 0.009

0.000 0.000 0.000 0.000 0.009 0.009 0.005 0.000 0.000 0.000

0.067 0.058 0.069 0.078 0.010 0.000 0.005 0.000 0.000 0.005

0.000 0.000 0.000 0.005 0.000 0.005 0.005 0.000 0.010 0.000

0.000 0.000 0.005 0.005 0.077 0.062 0.076 0.065 0.007 0.007

0.000 0.000 0.000 0.007 0.007 0.015 0.000 0.000 0.007 0.007

0.000 0.015 0.000 0.000 0.000 0.007 0.007 0.000 0.049 0.069

0.063 0.064

>Non-Methylation code 265

0.018 0.012 0.006 0.000 0.000 0.000 0.000 0.000 0.012 0.006

0.000 0.000 0.006 0.000 0.000 0.018 0.000 0.000 0.000 0.000

0.077 0.067 0.047 0.065 0.000 0.000 0.000 0.000 0.000 0.000

0.007 0.000 0.007 0.007 0.000 0.000 0.007 0.007 0.007 0.007

0.007 0.028 0.000 0.000 0.074 0.070 0.053 0.052 0.000 0.006

0.000 0.006 0.006 0.000 0.000 0.006 0.006 0.006 0.000 0.000

0.000 0.000 0.006 0.012 0.000 0.000 0.000 0.000 0.077 0.084

0.069 0.047

>Non-Methylation code 266

0.000 0.000 0.000 0.005 0.005 0.005 0.005 0.005 0.010 0.000

0.000 0.000 0.010 0.000 0.005 0.005 0.000 0.010 0.000 0.000

0.071 0.085 0.066 0.047 0.000 0.000 0.000 0.000 0.012 0.006

0.000 0.000 0.012 0.000 0.006 0.000 0.018 0.000 0.000 0.006

0.000 0.006 0.000 0.006 0.064 0.084 0.069 0.045 0.008 0.004

0.000 0.000 0.008 0.000 0.000 0.000 0.004 0.000 0.000 0.004

0.004 0.004 0.008 0.000 0.004 0.000 0.000 0.000 0.068 0.073

0.052 0.091

>Non-Methylation code 267

0.005 0.005 0.005 0.005 0.000 0.000 0.005 0.000 0.000 0.000

0.005 0.005 0.000 0.000 0.023 0.005 0.000 0.000 0.000 0.000

0.070 0.068 0.061 0.075 0.037 0.006 0.006 0.000 0.000 0.000

0.000 0.000 0.000 0.000 0.000 0.000 0.000 0.006 0.006 0.000

0.018 0.000 0.000 0.000 0.066 0.070 0.053 0.065 0.006 0.000

0.000 0.000 0.000 0.006 0.006 0.000 0.006 0.006 0.000 0.000

0.018 0.006 0.012 0.000 0.006 0.000 0.000 0.000 0.080 0.050

0.066 0.068

>Non-Methylation code 268

0.016 0.000 0.016 0.000 0.000 0.016 0.000 0.000 0.008 0.000

0.000 0.000 0.000 0.008 0.000 0.016 0.008 0.000 0.008 0.000

0.062 0.062 0.054 0.062 0.014 0.000 0.000 0.000 0.000 0.007

0.000 0.000 0.007 0.000 0.000 0.000 0.000 0.014 0.014 0.000

0.014 0.007 0.000 0.000 0.067 0.070 0.036 0.082 0.000 0.005

0.000 0.005 0.000 0.014 0.005 0.000 0.009 0.005 0.000 0.000

0.005 0.000 0.005 0.005 0.000 0.000 0.000 0.000 0.059 0.068

0.075 0.076

>Non-Methylation code 269

0.005 0.005 0.000 0.005 0.000 0.005 0.000 0.000 0.005 0.000

0.000 0.005 0.009 0.000 0.009 0.005 0.000 0.009 0.000 0.000

0.073 0.068 0.086 0.046 0.006 0.000 0.000 0.000 0.000 0.011

0.006 0.000 0.000 0.011 0.000 0.000 0.000 0.000 0.017 0.006

0.006 0.000 0.000 0.000 0.046 0.068 0.078 0.079 0.024 0.008

0.000 0.008 0.000 0.008 0.000 0.000 0.000 0.000 0.008 0.000

0.000 0.024 0.000 0.000 0.000 0.000 0.008 0.000 0.068 0.061

0.058 0.058

>Non-Methylation code 270

0.007 0.000 0.000 0.000 0.000 0.007 0.000 0.000 0.000 0.000

0.000 0.000 0.000 0.000 0.018 0.000 0.000 0.000 0.011 0.000

0.087 0.056 0.088 0.058 0.028 0.000 0.009 0.009 0.000 0.055

0.000 0.000 0.000 0.009 0.000 0.000 0.000 0.009 0.000 0.000

0.000 0.000 0.000 0.000 0.052 0.065 0.045 0.051 0.027 0.000

0.000 0.000 0.000 0.000 0.000 0.000 0.000 0.007 0.007 0.000

0.000 0.000 0.014 0.014 0.000 0.000 0.000 0.000 0.057 0.066

0.083 0.059

>Non-Methylation code 271

0.017 0.009 0.000 0.000 0.009 0.000 0.000 0.000 0.000 0.017

0.000 0.000 0.000 0.000 0.000 0.026 0.026 0.000 0.009 0.000

0.068 0.041 0.054 0.056 0.006 0.000 0.000 0.000 0.006 0.006

0.000 0.000 0.000 0.031 0.000 0.000 0.018 0.000 0.006 0.006

0.000 0.000 0.000 0.000 0.060 0.047 0.079 0.069 0.000 0.007

0.007 0.000 0.007 0.000 0.007 0.000 0.000 0.035 0.000 0.000

0.000 0.000 0.000 0.014 0.000 0.000 0.000 0.007 0.079 0.044

0.065 0.060

>Non-Methylation code 272

0.021 0.000 0.000 0.000 0.000 0.007 0.000 0.000 0.000 0.007

0.000 0.007 0.000 0.000 0.007 0.021 0.014 0.000 0.007 0.000

0.061 0.074 0.058 0.048 0.019 0.000 0.000 0.000 0.000 0.006

0.006 0.000 0.000 0.000 0.000 0.000 0.019 0.006 0.013 0.000

0.006 0.000 0.000 0.000 0.054 0.061 0.070 0.070 0.000 0.000

0.005 0.005 0.000 0.005 0.005 0.000 0.000 0.011 0.000 0.000

0.005 0.005 0.011 0.005 0.005 0.000 0.000 0.000 0.069 0.080

0.058 0.063

>Non-Methylation code 273

0.005 0.000 0.000 0.000 0.000 0.005 0.000 0.005 0.000 0.000

0.000 0.005 0.030 0.000 0.010 0.005 0.000 0.000 0.000 0.000

0.075 0.043 0.081 0.069 0.010 0.000 0.000 0.000 0.000 0.000

0.005 0.005 0.000 0.000 0.000 0.000 0.014 0.005 0.014 0.005

0.000 0.005 0.000 0.000 0.073 0.059 0.065 0.074 0.006 0.000

0.000 0.006 0.000 0.000 0.000 0.000 0.000 0.000 0.000 0.000

0.022 0.000 0.011 0.011 0.006 0.006 0.000 0.000 0.050 0.086

0.074 0.055

>Non-Methylation code 274

0.000 0.005 0.000 0.009 0.000 0.019 0.000 0.000 0.000 0.000

0.005 0.000 0.000 0.009 0.009 0.000 0.000 0.000 0.000 0.000

0.063 0.069 0.074 0.072 0.014 0.007 0.000 0.007 0.000 0.014

0.000 0.000 0.007 0.000 0.000 0.007 0.000 0.000 0.000 0.014

0.000 0.014 0.000 0.000 0.056 0.059 0.069 0.068 0.005 0.000

0.000 0.000 0.000 0.011 0.005 0.000 0.000 0.005 0.000 0.000

0.000 0.000 0.016 0.005 0.005 0.000 0.000 0.000 0.054 0.074

0.085 0.066

>Non-Methylation code 275

0.000 0.004 0.000 0.004 0.000 0.008 0.008 0.000 0.004 0.004

0.000 0.000 0.004 0.000 0.008 0.004 0.004 0.000 0.000 0.000

0.056 0.067 0.075 0.084 0.015 0.000 0.008 0.000 0.000 0.015

0.008 0.000 0.000 0.000 0.000 0.000 0.000 0.008 0.000 0.015

0.015 0.000 0.008 0.000 0.061 0.064 0.053 0.065 0.017 0.000

0.000 0.000 0.000 0.009 0.009 0.000 0.000 0.000 0.000 0.000

0.000 0.017 0.009 0.000 0.017 0.009 0.000 0.000 0.070 0.061

0.035 0.081

>Non-Methylation code 276

0.006 0.000 0.000 0.000 0.006 0.000 0.000 0.000 0.000 0.032

0.000 0.000 0.025 0.000 0.006 0.006 0.000 0.000 0.000 0.000

0.062 0.050 0.082 0.057 0.000 0.006 0.006 0.000 0.006 0.000

0.006 0.000 0.000 0.029 0.000 0.000 0.000 0.000 0.006 0.012

0.000 0.000 0.000 0.006 0.080 0.052 0.065 0.061 0.021 0.010

0.000 0.000 0.010 0.000 0.000 0.000 0.000 0.021 0.000 0.000

0.000 0.000 0.000 0.031 0.031 0.000 0.000 0.000 0.065 0.046

0.051 0.048

>Non-Methylation code 277

0.006 0.006 0.000 0.000 0.000 0.000 0.000 0.000 0.000 0.011

0.000 0.000 0.023 0.000 0.006 0.006 0.017 0.000 0.000 0.000

0.069 0.051 0.072 0.068 0.007 0.000 0.000 0.000 0.007 0.007

0.000 0.000 0.000 0.022 0.000 0.000 0.030 0.007 0.007 0.007

0.000 0.000 0.000 0.000 0.050 0.058 0.056 0.073 0.011 0.000

0.000 0.000 0.000 0.000 0.011 0.000 0.005 0.011 0.000 0.000

0.011 0.000 0.005 0.005 0.000 0.000 0.000 0.005 0.072 0.070

0.073 0.054

>Non-Methylation code 278

0.011 0.000 0.000 0.000 0.000 0.005 0.000 0.000 0.000 0.005

0.000 0.000 0.011 0.000 0.011 0.005 0.005 0.000 0.011 0.000

0.048 0.067 0.076 0.079 0.005 0.000 0.000 0.000 0.000 0.024

0.000 0.000 0.000 0.005 0.000 0.000 0.005 0.000 0.014 0.000

0.000 0.000 0.005 0.000 0.062 0.061 0.059 0.094 0.026 0.009

0.000 0.009 0.000 0.017 0.009 0.000 0.000 0.000 0.000 0.000

0.000 0.009 0.000 0.000 0.000 0.009 0.000 0.000 0.068 0.073

0.038 0.067

>Non-Methylation code 279

0.005 0.000 0.000 0.000 0.000 0.005 0.000 0.000 0.000 0.000

0.000 0.000 0.038 0.000 0.016 0.000 0.005 0.000 0.000 0.000

0.084 0.051 0.057 0.071 0.010 0.000 0.005 0.000 0.000 0.000

0.005 0.000 0.000 0.000 0.000 0.000 0.020 0.005 0.015 0.000

0.000 0.005 0.000 0.000 0.069 0.074 0.068 0.058 0.017 0.000

0.000 0.000 0.000 0.000 0.000 0.000 0.000 0.000 0.000 0.006

0.017 0.006 0.011 0.006 0.006 0.000 0.000 0.000 0.056 0.092

0.064 0.055

>Non-Methylation code 280

0.000 0.009 0.000 0.000 0.000 0.000 0.000 0.009 0.009 0.027

0.000 0.000 0.009 0.000 0.000 0.000 0.009 0.027 0.009 0.000

0.061 0.049 0.068 0.047 0.000 0.018 0.000 0.000 0.000 0.009

0.000 0.000 0.000 0.018 0.000 0.009 0.000 0.009 0.000 0.036

0.000 0.000 0.000 0.000 0.068 0.041 0.059 0.066 0.000 0.012

0.006 0.000 0.000 0.006 0.000 0.000 0.000 0.000 0.000 0.006

0.018 0.000 0.006 0.000 0.006 0.000 0.000 0.000 0.074 0.084

0.036 0.079

>Non-Methylation code 281

0.005 0.000 0.000 0.000 0.000 0.015 0.005 0.000 0.000 0.010

0.000 0.000 0.010 0.000 0.010 0.000 0.005 0.000 0.000 0.000

0.069 0.071 0.075 0.057 0.016 0.005 0.000 0.000 0.000 0.011

0.011 0.000 0.000 0.000 0.000 0.000 0.011 0.000 0.000 0.000

0.005 0.000 0.011 0.000 0.067 0.060 0.052 0.084 0.005 0.000

0.000 0.000 0.000 0.009 0.005 0.000 0.000 0.000 0.000 0.000

0.009 0.000 0.014 0.000 0.009 0.005 0.000 0.000 0.081 0.059

0.057 0.082

>Non-Methylation code 282

0.011 0.006 0.000 0.000 0.000 0.006 0.000 0.006 0.000 0.017

0.000 0.006 0.006 0.000 0.006 0.000 0.000 0.006 0.000 0.000

0.062 0.065 0.061 0.079 0.005 0.005 0.005 0.000 0.000 0.010

0.000 0.000 0.000 0.000 0.000 0.000 0.000 0.000 0.015 0.010

0.005 0.000 0.005 0.000 0.063 0.082 0.073 0.056 0.012 0.000

0.006 0.000 0.000 0.012 0.000 0.000 0.006 0.019 0.000 0.000

0.000 0.000 0.006 0.000 0.006 0.006 0.000 0.000 0.077 0.034

0.081 0.067

>Non-Methylation code 283

0.032 0.000 0.000 0.000 0.000 0.008 0.000 0.000 0.000 0.008

0.000 0.008 0.000 0.000 0.008 0.024 0.016 0.000 0.000 0.000

0.060 0.077 0.052 0.039 0.018 0.000 0.000 0.000 0.000 0.000

0.006 0.000 0.000 0.000 0.000 0.000 0.018 0.006 0.018 0.000

0.006 0.000 0.000 0.000 0.049 0.062 0.061 0.088 0.000 0.000

0.006 0.000 0.000 0.011 0.006 0.000 0.000 0.011 0.000 0.000

0.006 0.006 0.011 0.006 0.006 0.000 0.000 0.000 0.073 0.082

0.049 0.062

>Non-Methylation code 284

0.016 0.000 0.000 0.000 0.000 0.000 0.000 0.000 0.000 0.000

0.000 0.005 0.016 0.005 0.011 0.005 0.011 0.000 0.000 0.000

0.054 0.089 0.064 0.055 0.006 0.000 0.000 0.000 0.000 0.006

0.000 0.000 0.000 0.006 0.000 0.000 0.040 0.000 0.011 0.000

0.006 0.000 0.000 0.000 0.079 0.070 0.049 0.062 0.010 0.000

0.005 0.000 0.000 0.000 0.005 0.000 0.000 0.000 0.000 0.000

0.020 0.005 0.015 0.000 0.000 0.000 0.000 0.000 0.068 0.076

0.067 0.061

>Non-Methylation code 285

0.000 0.000 0.006 0.006 0.000 0.000 0.000 0.000 0.006 0.000

0.000 0.006 0.006 0.000 0.011 0.006 0.017 0.006 0.000 0.006

0.068 0.060 0.065 0.068 0.006 0.000 0.000 0.006 0.000 0.006

0.000 0.006 0.006 0.000 0.000 0.006 0.011 0.006 0.006 0.006

0.011 0.000 0.000 0.000 0.065 0.053 0.061 0.082 0.000 0.000

0.005 0.005 0.000 0.000 0.005 0.000 0.011 0.005 0.000 0.005

0.005 0.000 0.016 0.005 0.000 0.000 0.000 0.000 0.059 0.078

0.070 0.063

>Non-Methylation code 286

0.005 0.000 0.005 0.000 0.000 0.005 0.000 0.000 0.000 0.005

0.000 0.000 0.011 0.005 0.011 0.016 0.005 0.000 0.000 0.000

0.070 0.064 0.054 0.076 0.012 0.000 0.006 0.000 0.006 0.000

0.012 0.000 0.006 0.012 0.000 0.000 0.018 0.000 0.000 0.000

0.006 0.000 0.000 0.000 0.063 0.080 0.056 0.059 0.000 0.000

0.000 0.000 0.010 0.000 0.005 0.000 0.000 0.005 0.000 0.000

0.010 0.000 0.010 0.000 0.015 0.000 0.000 0.000 0.053 0.072

0.083 0.071

>Non-Methylation code 287

0.011 0.000 0.000 0.000 0.000 0.000 0.011 0.000 0.006 0.011

0.000 0.000 0.022 0.000 0.006 0.000 0.006 0.000 0.000 0.000

0.081 0.063 0.068 0.050 0.005 0.005 0.000 0.000 0.000 0.000

0.005 0.000 0.000 0.005 0.000 0.000 0.020 0.000 0.010 0.000

0.010 0.000 0.000 0.000 0.071 0.055 0.071 0.076 0.007 0.000

0.007 0.000 0.000 0.007 0.000 0.000 0.000 0.007 0.000 0.000

0.026 0.007 0.007 0.007 0.007 0.000 0.000 0.000 0.071 0.065

0.057 0.061

>Non-Methylation code 288

0.007 0.000 0.000 0.000 0.007 0.000 0.000 0.000 0.000 0.028

0.000 0.000 0.035 0.000 0.007 0.007 0.000 0.000 0.000 0.000

0.057 0.041 0.074 0.071 0.000 0.000 0.006 0.000 0.006 0.000

0.011 0.000 0.000 0.022 0.000 0.000 0.006 0.000 0.006 0.011

0.000 0.000 0.000 0.006 0.082 0.056 0.056 0.067 0.010 0.010

0.000 0.000 0.000 0.000 0.000 0.000 0.000 0.020 0.000 0.000

0.010 0.000 0.000 0.030 0.040 0.000 0.000 0.000 0.060 0.037

0.065 0.050

>Non-Methylation code 289

0.005 0.005 0.000 0.005 0.005 0.000 0.000 0.005 0.000 0.000

0.000 0.000 0.015 0.005 0.005 0.005 0.005 0.000 0.000 0.005

0.069 0.055 0.067 0.077 0.010 0.000 0.000 0.000 0.005 0.000

0.000 0.000 0.000 0.005 0.000 0.000 0.015 0.005 0.010 0.010

0.005 0.000 0.000 0.000 0.059 0.059 0.078 0.072 0.007 0.000

0.000 0.000 0.007 0.007 0.013 0.000 0.000 0.007 0.000 0.000

0.027 0.000 0.000 0.007 0.000 0.007 0.000 0.000 0.059 0.064

0.062 0.068

>Non-Methylation code 290

0.009 0.000 0.000 0.005 0.005 0.000 0.000 0.000 0.000 0.005

0.000 0.000 0.014 0.000 0.009 0.005 0.005 0.000 0.005 0.000

0.058 0.060 0.074 0.081 0.007 0.000 0.000 0.000 0.007 0.013

0.013 0.000 0.000 0.007 0.000 0.000 0.020 0.000 0.000 0.013

0.000 0.007 0.000 0.000 0.055 0.070 0.067 0.055 0.000 0.006

0.000 0.006 0.000 0.006 0.000 0.006 0.000 0.006 0.000 0.000

0.012 0.006 0.006 0.006 0.006 0.000 0.000 0.006 0.067 0.055

0.061 0.079

>Non-Methylation code 291

0.005 0.000 0.000 0.000 0.000 0.000 0.005 0.000 0.000 0.009

0.000 0.000 0.014 0.000 0.014 0.005 0.005 0.005 0.000 0.000

0.045 0.062 0.094 0.073 0.016 0.000 0.000 0.000 0.000 0.008

0.008 0.008 0.000 0.008 0.000 0.000 0.032 0.000 0.000 0.016

0.000 0.000 0.000 0.008 0.041 0.063 0.063 0.063 0.006 0.006

0.000 0.000 0.006 0.000 0.006 0.000 0.000 0.006 0.000 0.000

0.017 0.006 0.006 0.000 0.012 0.000 0.000 0.000 0.080 0.062

0.053 0.068

>Non-Methylation code 292

0.007 0.000 0.007 0.000 0.007 0.000 0.000 0.007 0.000 0.014

0.000 0.000 0.014 0.007 0.000 0.021 0.007 0.000 0.000 0.000

0.055 0.056 0.074 0.055 0.000 0.000 0.000 0.000 0.006 0.000

0.000 0.000 0.000 0.012 0.000 0.000 0.025 0.000 0.006 0.025

0.006 0.000 0.000 0.000 0.059 0.064 0.072 0.057 0.005 0.000

0.000 0.000 0.005 0.000 0.011 0.000 0.000 0.011 0.000 0.000

0.011 0.000 0.011 0.005 0.000 0.005 0.000 0.000 0.066 0.073

0.055 0.074

>Non-Methylation code 293

0.029 0.000 0.000 0.010 0.000 0.000 0.000 0.000 0.000 0.010

0.000 0.000 0.029 0.010 0.000 0.000 0.010 0.019 0.000 0.000

0.063 0.055 0.050 0.049 0.000 0.005 0.000 0.000 0.000 0.000

0.005 0.000 0.010 0.015 0.000 0.000 0.015 0.000 0.005 0.005

0.000 0.000 0.000 0.005 0.072 0.083 0.050 0.062 0.006 0.006

0.000 0.000 0.000 0.006 0.006 0.006 0.000 0.000 0.000 0.000

0.013 0.000 0.006 0.019 0.006 0.000 0.000 0.000 0.061 0.068

0.059 0.071

>Non-Methylation code 294

0.006 0.006 0.000 0.000 0.006 0.006 0.006 0.000 0.000 0.006

0.000 0.000 0.019 0.000 0.000 0.000 0.019 0.000 0.000 0.000

0.066 0.064 0.064 0.063 0.005 0.000 0.000 0.005 0.000 0.000

0.005 0.000 0.000 0.011 0.000 0.000 0.016 0.000 0.011 0.005

0.005 0.005 0.000 0.000 0.077 0.069 0.079 0.038 0.007 0.000

0.000 0.000 0.000 0.007 0.015 0.007 0.000 0.015 0.000 0.000

0.015 0.000 0.000 0.015 0.000 0.000 0.000 0.007 0.063 0.068

0.059 0.054

>Non-Methylation code 295

0.005 0.010 0.000 0.000 0.000 0.010 0.005 0.000 0.000 0.000

0.000 0.005 0.000 0.000 0.010 0.010 0.000 0.005 0.000 0.000

0.044 0.078 0.074 0.080 0.028 0.000 0.006 0.000 0.006 0.006

0.000 0.000 0.000 0.000 0.006 0.000 0.011 0.000 0.006 0.000

0.006 0.000 0.000 0.000 0.059 0.064 0.069 0.068 0.006 0.000

0.000 0.000 0.000 0.006 0.000 0.000 0.000 0.017 0.000 0.000

0.000 0.017 0.012 0.000 0.006 0.000 0.006 0.000 0.063 0.067

0.061 0.073

>Non-Methylation code 296

0.000 0.000 0.000 0.000 0.007 0.000 0.007 0.000 0.000 0.007

0.000 0.000 0.029 0.000 0.000 0.029 0.015 0.000 0.000 0.000

0.060 0.071 0.061 0.047 0.005 0.000 0.000 0.000 0.005 0.000

0.009 0.000 0.000 0.005 0.000 0.000 0.014 0.000 0.014 0.005

0.005 0.000 0.000 0.000 0.073 0.063 0.072 0.067 0.008 0.000

0.008 0.000 0.000 0.000 0.008 0.008 0.000 0.015 0.000 0.000

0.015 0.008 0.000 0.015 0.008 0.000 0.000 0.000 0.053 0.059

0.068 0.062

>Non-Methylation code 297

0.005 0.000 0.000 0.009 0.000 0.014 0.005 0.000 0.000 0.009

0.000 0.000 0.005 0.000 0.009 0.000 0.005 0.000 0.000 0.000

0.081 0.045 0.065 0.083 0.005 0.000 0.005 0.000 0.000 0.015

0.000 0.000 0.000 0.005 0.000 0.000 0.000 0.000 0.020 0.005

0.005 0.000 0.000 0.000 0.053 0.071 0.079 0.068 0.015 0.000

0.005 0.005 0.000 0.015 0.000 0.000 0.000 0.000 0.000 0.000

0.005 0.000 0.010 0.005 0.000 0.000 0.000 0.000 0.074 0.059

0.070 0.070

>Non-Methylation code 298

0.009 0.000 0.009 0.000 0.004 0.004 0.000 0.000 0.004 0.000

0.004 0.000 0.009 0.000 0.009 0.000 0.000 0.004 0.000 0.000

0.072 0.070 0.054 0.080 0.016 0.000 0.008 0.000 0.000 0.000

0.000 0.000 0.000 0.008 0.000 0.000 0.016 0.000 0.008 0.024

0.016 0.000 0.000 0.000 0.065 0.056 0.054 0.065 0.005 0.000

0.000 0.005 0.000 0.005 0.005 0.000 0.000 0.005 0.000 0.000

0.010 0.000 0.015 0.000 0.005 0.000 0.000 0.000 0.068 0.097

0.069 0.043

>Non-Methylation code 299

0.020 0.000 0.000 0.000 0.005 0.000 0.000 0.000 0.000 0.000

0.000 0.000 0.015 0.000 0.010 0.005 0.010 0.000 0.000 0.000

0.072 0.072 0.070 0.053 0.005 0.000 0.000 0.000 0.000 0.005

0.000 0.000 0.000 0.021 0.000 0.000 0.016 0.005 0.011 0.005

0.000 0.000 0.000 0.000 0.056 0.064 0.072 0.073 0.007 0.015

0.000 0.000 0.007 0.007 0.007 0.000 0.000 0.000 0.000 0.000

0.022 0.000 0.000 0.007 0.000 0.007 0.000 0.000 0.059 0.072

0.063 0.058

>Non-Methylation code 300

0.007 0.007 0.000 0.000 0.000 0.000 0.014 0.000 0.000 0.007

0.000 0.000 0.014 0.000 0.000 0.028 0.014 0.000 0.000 0.000

0.056 0.063 0.058 0.065 0.012 0.000 0.000 0.000 0.000 0.000

0.000 0.000 0.000 0.006 0.000 0.000 0.031 0.000 0.006 0.006

0.012 0.006 0.000 0.000 0.055 0.064 0.066 0.068 0.007 0.007

0.000 0.000 0.000 0.000 0.014 0.000 0.000 0.020 0.000 0.000

0.020 0.007 0.000 0.007 0.000 0.000 0.000 0.000 0.055 0.072

0.059 0.066

>Non-Methylation code 301

0.010 0.010 0.000 0.010 0.000 0.000 0.000 0.000 0.000 0.005

0.000 0.005 0.010 0.005 0.005 0.000 0.000 0.005 0.000 0.000

0.075 0.071 0.048 0.076 0.013 0.000 0.000 0.000 0.000 0.006

0.000 0.000 0.000 0.006 0.000 0.000 0.006 0.006 0.019 0.019

0.006 0.000 0.000 0.000 0.069 0.072 0.052 0.060 0.006 0.000

0.000 0.000 0.000 0.017 0.000 0.000 0.000 0.006 0.000 0.000

0.011 0.011 0.011 0.000 0.000 0.000 0.000 0.000 0.070 0.075

0.056 0.069

>Non-Methylation code 302

0.000 0.005 0.000 0.000 0.010 0.005 0.000 0.005 0.000 0.015

0.000 0.000 0.000 0.005 0.010 0.000 0.010 0.000 0.000 0.000

0.061 0.060 0.081 0.068 0.028 0.000 0.000 0.000 0.009 0.000

0.000 0.000 0.000 0.000 0.000 0.000 0.009 0.009 0.000 0.047

0.000 0.009 0.000 0.000 0.046 0.059 0.053 0.062 0.006 0.000

0.006 0.000 0.006 0.000 0.011 0.000 0.000 0.011 0.000 0.000

0.022 0.000 0.006 0.000 0.000 0.000 0.000 0.000 0.066 0.071

0.058 0.072

>Non-Methylation code 303

0.000 0.000 0.000 0.010 0.000 0.000 0.000 0.031 0.010 0.010

0.010 0.000 0.000 0.000 0.000 0.000 0.031 0.010 0.000 0.010

0.054 0.047 0.060 0.049 0.000 0.000 0.000 0.000 0.000 0.000

0.007 0.007 0.020 0.013 0.000 0.000 0.013 0.000 0.000 0.013

0.000 0.000 0.000 0.000 0.057 0.080 0.070 0.055 0.000 0.000

0.006 0.000 0.000 0.000 0.013 0.006 0.000 0.013 0.000 0.013

0.000 0.000 0.006 0.000 0.000 0.000 0.000 0.013 0.065 0.067

0.068 0.063

>Non-Methylation code 304

0.000 0.000 0.007 0.007 0.000 0.000 0.000 0.020 0.007 0.000

0.000 0.000 0.014 0.000 0.007 0.000 0.007 0.007 0.000 0.000

0.046 0.062 0.068 0.083 0.000 0.005 0.000 0.005 0.000 0.000

0.005 0.000 0.015 0.015 0.000 0.000 0.005 0.000 0.000 0.010

0.005 0.000 0.000 0.000 0.057 0.063 0.078 0.068 0.006 0.000

0.000 0.000 0.000 0.000 0.006 0.000 0.006 0.006 0.000 0.006

0.000 0.000 0.012 0.006 0.006 0.000 0.000 0.012 0.064 0.060

0.073 0.068

>Non-Methylation code 305

0.000 0.000 0.006 0.000 0.011 0.006 0.000 0.006 0.006 0.017

0.000 0.000 0.000 0.006 0.006 0.000 0.011 0.000 0.000 0.000

0.051 0.055 0.079 0.076 0.019 0.000 0.000 0.000 0.010 0.000

0.000 0.010 0.000 0.000 0.000 0.000 0.010 0.010 0.000 0.057

0.000 0.000 0.000 0.000 0.047 0.059 0.053 0.061 0.011 0.000

0.005 0.000 0.005 0.000 0.011 0.000 0.000 0.005 0.000 0.000

0.016 0.000 0.005 0.000 0.000 0.000 0.000 0.000 0.062 0.061

0.061 0.090

>Non-Methylation code 306

0.006 0.000 0.000 0.000 0.006 0.000 0.006 0.012 0.000 0.000

0.000 0.000 0.012 0.000 0.006 0.018 0.000 0.012 0.000 0.000

0.074 0.052 0.070 0.060 0.007 0.000 0.000 0.000 0.014 0.007

0.000 0.007 0.000 0.014 0.000 0.000 0.021 0.007 0.000 0.000

0.000 0.000 0.000 0.014 0.063 0.058 0.056 0.066 0.000 0.005

0.005 0.000 0.010 0.000 0.000 0.000 0.000 0.005 0.000 0.000

0.005 0.005 0.005 0.005 0.010 0.000 0.000 0.005 0.052 0.076

0.077 0.068

>Non-Methylation code 307

0.000 0.000 0.000 0.000 0.010 0.005 0.005 0.000 0.005 0.000

0.000 0.005 0.005 0.000 0.005 0.010 0.000 0.010 0.000 0.000

0.065 0.053 0.073 0.083 0.006 0.000 0.006 0.006 0.006 0.000

0.006 0.006 0.000 0.011 0.006 0.000 0.000 0.000 0.006 0.017

0.000 0.000 0.000 0.000 0.059 0.062 0.063 0.076 0.000 0.005

0.000 0.005 0.009 0.000 0.000 0.000 0.000 0.009 0.000 0.000

0.005 0.005 0.009 0.005 0.005 0.000 0.000 0.000 0.086 0.069

0.038 0.084

>Non-Methylation code 308

0.005 0.000 0.000 0.005 0.000 0.005 0.010 0.015 0.005 0.005

0.000 0.000 0.005 0.000 0.005 0.000 0.000 0.000 0.000 0.000

0.058 0.072 0.074 0.071 0.007 0.000 0.000 0.000 0.007 0.007

0.000 0.000 0.007 0.000 0.000 0.007 0.000 0.007 0.007 0.007

0.014 0.000 0.000 0.007 0.084 0.076 0.058 0.039 0.005 0.000

0.005 0.000 0.000 0.000 0.005 0.000 0.005 0.005 0.000 0.005

0.005 0.000 0.011 0.005 0.011 0.000 0.000 0.000 0.071 0.063

0.061 0.074

>Non-Methylation code 309

0.006 0.000 0.006 0.000 0.000 0.000 0.006 0.000 0.006 0.011

0.000 0.006 0.006 0.000 0.006 0.006 0.011 0.000 0.000 0.000

0.072 0.062 0.054 0.077 0.005 0.005 0.000 0.005 0.000 0.000

0.005 0.014 0.005 0.005 0.000 0.000 0.005 0.000 0.005 0.005

0.000 0.000 0.000 0.000 0.070 0.075 0.073 0.057 0.015 0.000

0.000 0.000 0.008 0.000 0.000 0.000 0.008 0.000 0.000 0.008

0.000 0.008 0.008 0.008 0.008 0.000 0.000 0.008 0.081 0.071

0.057 0.048

>Non-Methylation code 310

0.005 0.000 0.000 0.005 0.000 0.005 0.005 0.005 0.000 0.005

0.000 0.005 0.000 0.000 0.010 0.000 0.000 0.005 0.000 0.010

0.076 0.063 0.059 0.074 0.000 0.006 0.006 0.012 0.000 0.000

0.000 0.000 0.000 0.006 0.012 0.006 0.000 0.000 0.006 0.000

0.012 0.000 0.000 0.000 0.061 0.070 0.068 0.068 0.000 0.010

0.000 0.005 0.000 0.000 0.000 0.005 0.005 0.005 0.000 0.005

0.000 0.000 0.005 0.005 0.005 0.005 0.000 0.000 0.057 0.076

0.080 0.064

>Non-Methylation code 311

0.007 0.000 0.000 0.000 0.000 0.033 0.000 0.000 0.000 0.000

0.000 0.000 0.020 0.000 0.013 0.013 0.000 0.000 0.000 0.000

0.059 0.062 0.052 0.072 0.012 0.000 0.017 0.006 0.000 0.006

0.000 0.000 0.000 0.000 0.000 0.000 0.006 0.023 0.006 0.000

0.000 0.000 0.000 0.000 0.066 0.070 0.062 0.060 0.005 0.000

0.000 0.000 0.000 0.005 0.000 0.005 0.005 0.005 0.000 0.000

0.005 0.000 0.019 0.005 0.005 0.000 0.000 0.000 0.108 0.032

0.108 0.028

>Non-Methylation code 312

0.013 0.000 0.013 0.000 0.000 0.006 0.000 0.000 0.006 0.000

0.000 0.006 0.013 0.000 0.000 0.006 0.000 0.006 0.000 0.000

0.054 0.068 0.072 0.070 0.000 0.005 0.000 0.005 0.005 0.000

0.000 0.000 0.005 0.000 0.000 0.000 0.010 0.005 0.010 0.000

0.015 0.000 0.005 0.000 0.061 0.084 0.069 0.055 0.000 0.000

0.000 0.000 0.000 0.018 0.000 0.000 0.006 0.000 0.000 0.000

0.006 0.006 0.018 0.012 0.000 0.006 0.000 0.000 0.057 0.088

0.065 0.050

>Non-Methylation code 313

0.012 0.000 0.006 0.006 0.000 0.006 0.000 0.000 0.006 0.000

0.006 0.006 0.012 0.000 0.000 0.006 0.000 0.006 0.000 0.000

0.051 0.065 0.079 0.064 0.000 0.004 0.000 0.004 0.004 0.000

0.000 0.000 0.004 0.000 0.000 0.000 0.009 0.004 0.013 0.000

0.009 0.000 0.004 0.000 0.069 0.089 0.062 0.056 0.000 0.000

0.005 0.000 0.000 0.015 0.000 0.000 0.005 0.000 0.000 0.000

0.005 0.005 0.015 0.005 0.000 0.005 0.000 0.000 0.052 0.093

0.082 0.047

>Non-Methylation code 314

0.006 0.000 0.000 0.000 0.006 0.000 0.000 0.000 0.006 0.012

0.000 0.006 0.000 0.000 0.006 0.017 0.012 0.000 0.006 0.000

0.070 0.070 0.053 0.065 0.005 0.000 0.000 0.000 0.000 0.005

0.005 0.000 0.005 0.000 0.000 0.000 0.011 0.000 0.011 0.011

0.005 0.005 0.000 0.000 0.074 0.090 0.051 0.053 0.013 0.000

0.000 0.006 0.000 0.000 0.006 0.006 0.000 0.025 0.000 0.006

0.000 0.006 0.006 0.000 0.000 0.000 0.000 0.000 0.077 0.057

0.051 0.073

>Non-Methylation code 315

0.019 0.000 0.000 0.000 0.000 0.000 0.013 0.006 0.000 0.006

0.000 0.000 0.013 0.000 0.006 0.000 0.006 0.000 0.006 0.000

0.061 0.059 0.068 0.070 0.000 0.000 0.005 0.000 0.005 0.005

0.000 0.000 0.005 0.015 0.005 0.000 0.005 0.000 0.010 0.000

0.005 0.000 0.000 0.000 0.061 0.074 0.062 0.076 0.007 0.007

0.007 0.000 0.007 0.007 0.000 0.000 0.000 0.014 0.000 0.007

0.000 0.000 0.000 0.021 0.007 0.000 0.000 0.000 0.066 0.059

0.055 0.067

>Non-Methylation code 316

0.006 0.000 0.006 0.006 0.000 0.000 0.006 0.013 0.000 0.026

0.000 0.006 0.000 0.000 0.000 0.006 0.000 0.000 0.000 0.006

0.062 0.065 0.066 0.056 0.005 0.000 0.000 0.000 0.011 0.000

0.000 0.000 0.005 0.005 0.000 0.000 0.000 0.000 0.005 0.005

0.022 0.000 0.011 0.000 0.072 0.055 0.056 0.079 0.000 0.000

0.000 0.000 0.000 0.005 0.010 0.000 0.005 0.005 0.000 0.000

0.010 0.000 0.010 0.005 0.000 0.005 0.000 0.000 0.065 0.074

0.061 0.075

>Non-Methylation code 317

0.000 0.000 0.000 0.000 0.000 0.014 0.007 0.007 0.000 0.014

0.014 0.000 0.007 0.007 0.007 0.014 0.000 0.000 0.000 0.000

0.062 0.065 0.053 0.063 0.005 0.010 0.000 0.000 0.000 0.005

0.005 0.000 0.000 0.000 0.000 0.005 0.005 0.005 0.000 0.000

0.005 0.005 0.010 0.005 0.061 0.062 0.063 0.082 0.009 0.000

0.000 0.000 0.000 0.018 0.018 0.009 0.000 0.009 0.000 0.000

0.000 0.000 0.000 0.009 0.018 0.018 0.000 0.000 0.048 0.046

0.065 0.067

>Non-Methylation code 318

0.000 0.000 0.000 0.000 0.000 0.005 0.005 0.000 0.005 0.000

0.000 0.000 0.010 0.000 0.015 0.010 0.005 0.005 0.000 0.000

0.073 0.070 0.062 0.066 0.012 0.000 0.006 0.006 0.000 0.000

0.006 0.006 0.000 0.018 0.000 0.006 0.000 0.006 0.006 0.006

0.000 0.000 0.000 0.000 0.065 0.067 0.060 0.063 0.011 0.000

0.000 0.000 0.006 0.000 0.000 0.000 0.006 0.011 0.000 0.006

0.000 0.000 0.006 0.006 0.011 0.000 0.006 0.000 0.068 0.067

0.057 0.073

>Non-Methylation code 319

0.011 0.000 0.000 0.000 0.000 0.000 0.011 0.000 0.000 0.022

0.000 0.011 0.000 0.000 0.000 0.033 0.033 0.022 0.000 0.000

0.057 0.062 0.040 0.033 0.005 0.000 0.000 0.000 0.000 0.000

0.005 0.000 0.000 0.005 0.005 0.000 0.015 0.000 0.010 0.015

0.005 0.000 0.000 0.000 0.056 0.079 0.079 0.054 0.006 0.012

0.000 0.000 0.000 0.000 0.012 0.000 0.000 0.006 0.000 0.000

0.006 0.012 0.006 0.000 0.006 0.006 0.000 0.000 0.063 0.056

0.065 0.076

>Non-Methylation code 320

0.008 0.008 0.000 0.000 0.016 0.000 0.000 0.016 0.000 0.025

0.000 0.000 0.008 0.000 0.000 0.000 0.008 0.016 0.000 0.000

0.050 0.049 0.062 0.065 0.000 0.000 0.000 0.000 0.015 0.000

0.000 0.000 0.000 0.008 0.000 0.000 0.015 0.008 0.000 0.038

0.000 0.008 0.008 0.000 0.059 0.070 0.050 0.056 0.000 0.005

0.000 0.000 0.005 0.005 0.015 0.000 0.000 0.005 0.000 0.000

0.010 0.000 0.005 0.010 0.000 0.000 0.000 0.000 0.067 0.071

0.068 0.067

>Non-Methylation code 321

0.006 0.006 0.000 0.000 0.000 0.006 0.000 0.012 0.000 0.000

0.006 0.000 0.006 0.000 0.006 0.012 0.000 0.000 0.006 0.000

0.048 0.075 0.078 0.069 0.006 0.006 0.006 0.000 0.000 0.006

0.000 0.000 0.006 0.017 0.000 0.000 0.000 0.000 0.006 0.006

0.000 0.017 0.000 0.000 0.063 0.066 0.060 0.071 0.005 0.005

0.000 0.000 0.005 0.000 0.000 0.000 0.005 0.005 0.000 0.000

0.005 0.000 0.005 0.000 0.000 0.000 0.000 0.015 0.076 0.067

0.067 0.074

>Non-Methylation code 322

0.008 0.004 0.000 0.000 0.000 0.004 0.000 0.008 0.000 0.000

0.000 0.000 0.004 0.000 0.016 0.000 0.004 0.000 0.000 0.000

0.060 0.068 0.057 0.101 0.006 0.000 0.006 0.006 0.000 0.012

0.000 0.000 0.000 0.012 0.000 0.000 0.000 0.012 0.006 0.006

0.000 0.012 0.000 0.000 0.060 0.067 0.073 0.057 0.000 0.000

0.000 0.000 0.000 0.008 0.000 0.000 0.008 0.008 0.000 0.008

0.016 0.000 0.000 0.016 0.008 0.000 0.000 0.016 0.065 0.058

0.051 0.070

>Non-Methylation code 323

0.000 0.000 0.005 0.005 0.009 0.000 0.000 0.000 0.000 0.000

0.000 0.005 0.009 0.000 0.009 0.009 0.005 0.000 0.000 0.000

0.056 0.076 0.085 0.061 0.000 0.000 0.000 0.013 0.009 0.000

0.000 0.004 0.000 0.004 0.004 0.000 0.009 0.000 0.009 0.004

0.000 0.000 0.000 0.000 0.072 0.075 0.073 0.057 0.005 0.000

0.000 0.000 0.010 0.000 0.000 0.000 0.005 0.005 0.000 0.005

0.005 0.005 0.005 0.005 0.000 0.010 0.000 0.000 0.064 0.077

0.065 0.067

>Non-Methylation code 324

0.006 0.000 0.000 0.000 0.024 0.000 0.006 0.000 0.000 0.024

0.000 0.000 0.006 0.000 0.006 0.000 0.006 0.000 0.000 0.000

0.063 0.065 0.079 0.050 0.000 0.018 0.000 0.000 0.012 0.006

0.000 0.006 0.000 0.012 0.000 0.000 0.012 0.000 0.000 0.012

0.000 0.000 0.000 0.000 0.097 0.052 0.085 0.022 0.019 0.006

0.000 0.000 0.026 0.000 0.006 0.000 0.000 0.006 0.000 0.000

0.006 0.000 0.000 0.000 0.000 0.006 0.000 0.000 0.067 0.059

0.058 0.073

>Non-Methylation code 325

0.000 0.000 0.000 0.007 0.000 0.000 0.007 0.007 0.020 0.007

0.007 0.000 0.000 0.000 0.000 0.007 0.000 0.000 0.013 0.000

0.076 0.071 0.053 0.060 0.000 0.010 0.000 0.000 0.000 0.010

0.000 0.000 0.005 0.000 0.000 0.000 0.000 0.000 0.010 0.015

0.005 0.005 0.000 0.000 0.068 0.068 0.066 0.073 0.013 0.000

0.013 0.006 0.000 0.006 0.000 0.000 0.006 0.006 0.000 0.000

0.000 0.006 0.006 0.000 0.000 0.006 0.000 0.000 0.064 0.074

0.063 0.063

>Non-Methylation code 326

0.000 0.004 0.008 0.000 0.004 0.004 0.008 0.000 0.000 0.004

0.000 0.008 0.000 0.000 0.004 0.000 0.000 0.004 0.004 0.000

0.069 0.060 0.074 0.077 0.000 0.000 0.000 0.000 0.000 0.031

0.000 0.031 0.000 0.000 0.031 0.000 0.000 0.000 0.000 0.031

0.031 0.031 0.000 0.000 0.029 0.036 0.035 0.048 0.006 0.000

0.000 0.000 0.006 0.000 0.000 0.000 0.000 0.006 0.000 0.000

0.000 0.006 0.013 0.006 0.000 0.000 0.006 0.000 0.043 0.085

0.088 0.067

>Non-Methylation code 327

0.000 0.006 0.000 0.000 0.013 0.000 0.006 0.013 0.000 0.006

0.006 0.006 0.006 0.000 0.000 0.006 0.006 0.000 0.000 0.000

0.055 0.052 0.079 0.069 0.000 0.006 0.000 0.000 0.013 0.000

0.000 0.013 0.006 0.006 0.000 0.000 0.006 0.006 0.000 0.006

0.000 0.006 0.000 0.013 0.059 0.071 0.047 0.074 0.000 0.000

0.000 0.000 0.023 0.000 0.000 0.008 0.008 0.015 0.000 0.008

0.008 0.000 0.000 0.000 0.008 0.000 0.000 0.015 0.071 0.050

0.057 0.062

>Non-Methylation code 328

0.000 0.005 0.005 0.000 0.000 0.005 0.000 0.010 0.000 0.000

0.000 0.010 0.005 0.000 0.005 0.005 0.000 0.000 0.005 0.005

0.071 0.077 0.070 0.058 0.016 0.000 0.000 0.008 0.000 0.008

0.008 0.008 0.000 0.000 0.000 0.000 0.008 0.000 0.000 0.008

0.024 0.008 0.000 0.008 0.061 0.054 0.070 0.046 0.000 0.000

0.000 0.000 0.000 0.007 0.000 0.000 0.000 0.020 0.013 0.000

0.013 0.007 0.007 0.000 0.000 0.000 0.000 0.007 0.052 0.071

0.069 0.069

>Non-Methylation code 329

0.000 0.000 0.000 0.000 0.006 0.006 0.000 0.006 0.006 0.011

0.000 0.006 0.000 0.000 0.017 0.006 0.000 0.000 0.000 0.000

0.057 0.080 0.081 0.055 0.011 0.000 0.005 0.011 0.005 0.000

0.000 0.005 0.000 0.005 0.000 0.000 0.000 0.000 0.005 0.005

0.000 0.000 0.000 0.011 0.054 0.061 0.065 0.087 0.000 0.000

0.005 0.000 0.005 0.005 0.000 0.005 0.010 0.005 0.000 0.000

0.010 0.000 0.005 0.000 0.010 0.000 0.000 0.000 0.065 0.063

0.071 0.074

>Non-Methylation code 330

0.000 0.000 0.000 0.000 0.015 0.007 0.000 0.000 0.000 0.045

0.000 0.000 0.000 0.000 0.007 0.000 0.000 0.022 0.000 0.000

0.051 0.055 0.057 0.073 0.012 0.000 0.000 0.000 0.000 0.012

0.000 0.000 0.000 0.000 0.000 0.000 0.000 0.000 0.000 0.125

0.000 0.000 0.012 0.000 0.047 0.054 0.032 0.039 0.006 0.000

0.000 0.000 0.000 0.006 0.000 0.000 0.000 0.011 0.000 0.000

0.034 0.000 0.011 0.000 0.000 0.000 0.000 0.000 0.076 0.047

0.071 0.070

>Non-Methylation code 331

0.000 0.000 0.000 0.000 0.000 0.006 0.000 0.000 0.000 0.018

0.000 0.000 0.031 0.000 0.006 0.006 0.000 0.000 0.006 0.000

0.066 0.064 0.082 0.048 0.000 0.000 0.000 0.006 0.011 0.006

0.017 0.000 0.000 0.011 0.000 0.000 0.006 0.000 0.000 0.000

0.000 0.011 0.000 0.006 0.062 0.073 0.063 0.062 0.000 0.000

0.000 0.000 0.000 0.000 0.000 0.009 0.000 0.009 0.009 0.000

0.009 0.000 0.009 0.043 0.009 0.009 0.000 0.000 0.064 0.051

0.051 0.064

>Non-Methylation code 332

0.000 0.004 0.000 0.004 0.012 0.004 0.004 0.000 0.000 0.004

0.000 0.000 0.000 0.000 0.008 0.004 0.004 0.004 0.000 0.000

0.049 0.068 0.075 0.091 0.010 0.000 0.000 0.000 0.005 0.010

0.000 0.000 0.000 0.010 0.000 0.000 0.000 0.000 0.010 0.010

0.005 0.000 0.005 0.000 0.065 0.069 0.071 0.065 0.000 0.000

0.008 0.000 0.008 0.015 0.000 0.000 0.000 0.015 0.000 0.000

0.023 0.000 0.000 0.000 0.000 0.015 0.000 0.000 0.055 0.059

0.060 0.074

>Non-Methylation code 333

0.021 0.000 0.000 0.000 0.000 0.007 0.000 0.000 0.000 0.000

0.000 0.000 0.000 0.000 0.007 0.042 0.000 0.000 0.007 0.000

0.066 0.068 0.057 0.057 0.007 0.000 0.000 0.007 0.000 0.014

0.000 0.000 0.000 0.007 0.000 0.000 0.051 0.000 0.007 0.000

0.000 0.000 0.000 0.000 0.057 0.064 0.052 0.067 0.000 0.000

0.000 0.000 0.006 0.006 0.000 0.000 0.000 0.028 0.000 0.000

0.006 0.000 0.011 0.006 0.000 0.006 0.000 0.000 0.067 0.082

0.055 0.061

>Non-Methylation code 334

0.000 0.005 0.005 0.000 0.000 0.005 0.000 0.000 0.000 0.000

0.005 0.000 0.009 0.000 0.014 0.014 0.000 0.000 0.000 0.000

0.056 0.079 0.078 0.064 0.027 0.007 0.000 0.000 0.000 0.007

0.007 0.007 0.007 0.007 0.000 0.000 0.000 0.013 0.000 0.000

0.000 0.007 0.000 0.000 0.068 0.054 0.071 0.054 0.007 0.000

0.000 0.000 0.000 0.000 0.007 0.000 0.007 0.020 0.000 0.000

0.007 0.000 0.007 0.007 0.007 0.007 0.000 0.000 0.082 0.041

0.068 0.068

>Non-Methylation code 335

0.011 0.005 0.000 0.000 0.005 0.000 0.000 0.000 0.000 0.005

0.011 0.005 0.000 0.000 0.011 0.011 0.000 0.000 0.000 0.000

0.063 0.075 0.064 0.066 0.000 0.008 0.000 0.008 0.004 0.004

0.000 0.000 0.000 0.000 0.000 0.000 0.004 0.000 0.012 0.000

0.004 0.004 0.000 0.000 0.080 0.063 0.078 0.065 0.006 0.000

0.000 0.006 0.006 0.000 0.000 0.000 0.006 0.006 0.000 0.006

0.000 0.006 0.006 0.006 0.000 0.012 0.000 0.000 0.062 0.062

0.075 0.066

>Non-Methylation code 336

0.022 0.007 0.000 0.000 0.000 0.000 0.007 0.007 0.000 0.007

0.000 0.014 0.000 0.007 0.000 0.000 0.000 0.022 0.000 0.000

0.066 0.070 0.044 0.060 0.000 0.000 0.000 0.000 0.000 0.000

0.008 0.000 0.000 0.023 0.015 0.000 0.000 0.000 0.008 0.015

0.008 0.008 0.000 0.000 0.053 0.058 0.069 0.071 0.000 0.010

0.005 0.000 0.000 0.005 0.000 0.000 0.005 0.000 0.005 0.000

0.005 0.000 0.010 0.010 0.000 0.000 0.000 0.000 0.076 0.051

0.061 0.092

>Non-Methylation code 337

0.000 0.005 0.005 0.000 0.000 0.010 0.000 0.000 0.000 0.005

0.015 0.010 0.010 0.000 0.005 0.000 0.000 0.000 0.000 0.000

0.064 0.052 0.068 0.084 0.006 0.013 0.000 0.006 0.000 0.000

0.000 0.006 0.000 0.000 0.000 0.000 0.013 0.000 0.000 0.000

0.013 0.006 0.013 0.000 0.060 0.083 0.068 0.046 0.008 0.000

0.000 0.000 0.000 0.016 0.016 0.000 0.000 0.008 0.000 0.000

0.008 0.016 0.000 0.000 0.000 0.008 0.000 0.008 0.075 0.057

0.050 0.065

>Non-Methylation code 338

0.000 0.004 0.004 0.000 0.000 0.004 0.000 0.000 0.000 0.004

0.004 0.000 0.004 0.000 0.017 0.008 0.000 0.000 0.000 0.000

0.050 0.089 0.082 0.062 0.031 0.008 0.000 0.000 0.000 0.015

0.000 0.008 0.008 0.008 0.000 0.000 0.000 0.008 0.000 0.000

0.000 0.008 0.000 0.000 0.063 0.052 0.067 0.058 0.006 0.000

0.000 0.006 0.000 0.000 0.006 0.000 0.012 0.012 0.000 0.000

0.006 0.000 0.006 0.006 0.000 0.006 0.000 0.000 0.082 0.055

0.058 0.074

>Non-Methylation code 339

0.030 0.000 0.000 0.000 0.010 0.000 0.000 0.000 0.000 0.020

0.000 0.000 0.071 0.000 0.000 0.000 0.000 0.000 0.000 0.000

0.086 0.012 0.086 0.018 0.000 0.000 0.000 0.000 0.000 0.000

0.006 0.000 0.000 0.006 0.000 0.000 0.033 0.000 0.011 0.017

0.000 0.000 0.000 0.000 0.065 0.064 0.067 0.065 0.011 0.005

0.000 0.000 0.000 0.000 0.000 0.005 0.000 0.000 0.000 0.000

0.032 0.000 0.011 0.000 0.000 0.000 0.000 0.000 0.072 0.047

0.076 0.074

>Non-Methylation code 340

0.018 0.000 0.000 0.000 0.006 0.006 0.000 0.000 0.000 0.012

0.000 0.000 0.018 0.000 0.006 0.012 0.000 0.000 0.000 0.000

0.068 0.063 0.056 0.069 0.010 0.005 0.000 0.000 0.000 0.000

0.000 0.000 0.000 0.005 0.000 0.000 0.026 0.000 0.016 0.005

0.000 0.000 0.000 0.000 0.059 0.078 0.066 0.062 0.009 0.000

0.000 0.000 0.000 0.005 0.000 0.000 0.000 0.005 0.000 0.000

0.019 0.000 0.009 0.000 0.000 0.005 0.005 0.000 0.067 0.053

0.079 0.078

>Non-Methylation code 341

0.010 0.000 0.000 0.000 0.000 0.021 0.000 0.000 0.000 0.000

0.000 0.000 0.010 0.005 0.010 0.005 0.005 0.000 0.000 0.000

0.046 0.073 0.063 0.084 0.014 0.000 0.000 0.000 0.000 0.014

0.000 0.000 0.000 0.005 0.000 0.000 0.005 0.009 0.014 0.000

0.000 0.000 0.000 0.000 0.042 0.059 0.089 0.083 0.018 0.000

0.000 0.000 0.000 0.012 0.000 0.000 0.000 0.006 0.000 0.006

0.000 0.006 0.018 0.006 0.000 0.000 0.000 0.000 0.070 0.060

0.046 0.083

>Non-Methylation code 342

0.005 0.000 0.000 0.010 0.000 0.000 0.000 0.000 0.010 0.005

0.000 0.005 0.016 0.000 0.000 0.005 0.000 0.005 0.000 0.005

0.063 0.069 0.072 0.063 0.000 0.000 0.000 0.000 0.010 0.000

0.000 0.000 0.010 0.005 0.000 0.000 0.005 0.010 0.010 0.005

0.010 0.000 0.000 0.000 0.054 0.068 0.061 0.083 0.007 0.000

0.000 0.007 0.000 0.000 0.000 0.000 0.007 0.013 0.000 0.000

0.000 0.007 0.013 0.026 0.000 0.000 0.000 0.000 0.065 0.079

0.061 0.049

>Non-Methylation code 343

0.013 0.000 0.000 0.000 0.020 0.007 0.000 0.007 0.007 0.013

0.000 0.000 0.013 0.000 0.000 0.000 0.007 0.000 0.000 0.000

0.070 0.064 0.064 0.050 0.000 0.000 0.000 0.005 0.011 0.000

0.000 0.000 0.000 0.005 0.000 0.000 0.005 0.005 0.011 0.027

0.000 0.000 0.000 0.000 0.066 0.074 0.065 0.057 0.004 0.000

0.004 0.000 0.004 0.000 0.000 0.000 0.004 0.004 0.000 0.000

0.004 0.000 0.013 0.009 0.000 0.004 0.000 0.000 0.059 0.090

0.089 0.042

>Non-Methylation code 344

0.006 0.000 0.000 0.000 0.000 0.013 0.000 0.000 0.006 0.006

0.000 0.006 0.000 0.000 0.013 0.019 0.000 0.013 0.000 0.000

0.062 0.085 0.062 0.043 0.013 0.000 0.000 0.007 0.013 0.013

0.000 0.000 0.007 0.013 0.000 0.000 0.007 0.000 0.000 0.000

0.007 0.000 0.000 0.000 0.065 0.065 0.069 0.056 0.000 0.000

0.000 0.012 0.006 0.000 0.000 0.000 0.006 0.006 0.000 0.000

0.000 0.000 0.018 0.024 0.000 0.000 0.000 0.000 0.052 0.060

0.071 0.079

>Non-Methylation code 345

0.006 0.000 0.012 0.000 0.000 0.000 0.000 0.006 0.006 0.006

0.000 0.006 0.012 0.006 0.006 0.006 0.000 0.000 0.000 0.000

0.062 0.064 0.048 0.086 0.000 0.005 0.000 0.011 0.000 0.000

0.000 0.005 0.000 0.005 0.000 0.000 0.011 0.005 0.011 0.005

0.011 0.000 0.000 0.000 0.063 0.056 0.074 0.071 0.004 0.004

0.000 0.009 0.000 0.000 0.004 0.000 0.004 0.009 0.000 0.000

0.009 0.000 0.009 0.000 0.000 0.000 0.000 0.000 0.077 0.063

0.058 0.083

>Non-Methylation code 346

0.000 0.007 0.000 0.014 0.000 0.021 0.007 0.000 0.000 0.007

0.007 0.000 0.000 0.000 0.000 0.014 0.014 0.000 0.000 0.000

0.045 0.064 0.070 0.064 0.010 0.005 0.000 0.000 0.000 0.005

0.005 0.000 0.000 0.005 0.000 0.000 0.005 0.005 0.010 0.000

0.005 0.005 0.000 0.000 0.053 0.071 0.062 0.083 0.000 0.000

0.009 0.000 0.005 0.009 0.005 0.005 0.000 0.005 0.000 0.000

0.005 0.000 0.009 0.000 0.000 0.000 0.000 0.000 0.069 0.071

0.062 0.082

>Non-Methylation code 347

0.000 0.006 0.006 0.000 0.000 0.006 0.012 0.000 0.006 0.006

0.000 0.006 0.006 0.012 0.000 0.006 0.000 0.006 0.000 0.000

0.070 0.060 0.055 0.072 0.010 0.000 0.000 0.000 0.000 0.000

0.005 0.010 0.000 0.000 0.000 0.000 0.000 0.000 0.015 0.010

0.010 0.000 0.000 0.005 0.051 0.074 0.065 0.079 0.000 0.000

0.000 0.009 0.000 0.018 0.000 0.000 0.000 0.009 0.000 0.000

0.018 0.009 0.000 0.018 0.018 0.009 0.000 0.000 0.063 0.066

0.034 0.060

>Non-Methylation code 348

0.034 0.000 0.000 0.000 0.000 0.017 0.000 0.000 0.000 0.000

0.000 0.000 0.000 0.000 0.000 0.017 0.008 0.008 0.017 0.000

0.057 0.041 0.078 0.057 0.012 0.000 0.000 0.006 0.000 0.018

0.000 0.000 0.000 0.006 0.000 0.000 0.018 0.000 0.012 0.006

0.000 0.000 0.000 0.000 0.063 0.080 0.051 0.059 0.000 0.000

0.005 0.005 0.000 0.005 0.000 0.000 0.000 0.010 0.000 0.000

0.000 0.000 0.024 0.005 0.000 0.000 0.000 0.000 0.061 0.082

0.085 0.052

>Non-Methylation code 349

0.000 0.000 0.006 0.000 0.000 0.006 0.006 0.006 0.000 0.006

0.000 0.012 0.000 0.000 0.006 0.012 0.006 0.000 0.000 0.006

0.050 0.063 0.074 0.073 0.009 0.000 0.000 0.009 0.000 0.009

0.009 0.009 0.000 0.018 0.000 0.000 0.000 0.000 0.000 0.000

0.036 0.009 0.000 0.009 0.042 0.054 0.052 0.070 0.000 0.000

0.000 0.006 0.000 0.000 0.006 0.012 0.000 0.012 0.000 0.000

0.006 0.006 0.006 0.006 0.000 0.000 0.006 0.000 0.071 0.069

0.075 0.049

>Non-Methylation code 350

0.026 0.000 0.000 0.000 0.006 0.000 0.006 0.000 0.000 0.000

0.000 0.000 0.013 0.000 0.006 0.000 0.026 0.000 0.000 0.000

0.053 0.066 0.068 0.061 0.000 0.000 0.000 0.000 0.000 0.008

0.000 0.000 0.000 0.016 0.000 0.000 0.049 0.016 0.000 0.008

0.008 0.000 0.000 0.000 0.067 0.051 0.058 0.051 0.000 0.004

0.000 0.000 0.004 0.004 0.009 0.000 0.000 0.000 0.000 0.009

0.013 0.000 0.009 0.000 0.000 0.000 0.000 0.000 0.070 0.080

0.068 0.063

>Non-Methylation code 351

0.010 0.000 0.000 0.000 0.000 0.000 0.005 0.010 0.000 0.000

0.000 0.000 0.000 0.000 0.015 0.010 0.010 0.000 0.000 0.005

0.051 0.074 0.065 0.079 0.000 0.000 0.000 0.008 0.000 0.017

0.000 0.000 0.000 0.008 0.000 0.000 0.025 0.008 0.000 0.017

0.017 0.008 0.000 0.000 0.064 0.069 0.030 0.061 0.000 0.006

0.000 0.000 0.000 0.006 0.012 0.000 0.006 0.006 0.000 0.006

0.006 0.012 0.000 0.006 0.000 0.006 0.000 0.000 0.069 0.062

0.055 0.072

>Non-Methylation code 352

0.014 0.000 0.000 0.000 0.005 0.000 0.000 0.000 0.000 0.019

0.000 0.000 0.000 0.000 0.014 0.005 0.005 0.000 0.000 0.000

0.072 0.061 0.056 0.083 0.004 0.004 0.000 0.000 0.008 0.008

0.000 0.000 0.000 0.004 0.000 0.000 0.008 0.004 0.008 0.004

0.000 0.000 0.000 0.000 0.070 0.050 0.078 0.080 0.019 0.006

0.000 0.000 0.000 0.006 0.000 0.000 0.000 0.006 0.000 0.006

0.006 0.000 0.006 0.012 0.000 0.006 0.000 0.000 0.076 0.059

0.071 0.053

>Non-Methylation code 353

0.015 0.000 0.000 0.000 0.000 0.000 0.000 0.022 0.000 0.007

0.007 0.000 0.007 0.000 0.007 0.022 0.007 0.000 0.000 0.000

0.067 0.055 0.071 0.046 0.000 0.009 0.000 0.004 0.004 0.000

0.000 0.000 0.000 0.009 0.000 0.000 0.009 0.004 0.009 0.000

0.000 0.004 0.000 0.000 0.067 0.078 0.072 0.064 0.005 0.005

0.000 0.005 0.010 0.000 0.010 0.000 0.005 0.000 0.000 0.010

0.000 0.000 0.000 0.000 0.000 0.005 0.000 0.005 0.067 0.082

0.069 0.054

>Non-Methylation code 354

0.007 0.000 0.007 0.000 0.007 0.014 0.007 0.007 0.000 0.007

0.000 0.007 0.007 0.000 0.000 0.014 0.000 0.000 0.000 0.000

0.049 0.072 0.068 0.059 0.015 0.000 0.000 0.007 0.000 0.007

0.007 0.000 0.000 0.015 0.000 0.000 0.000 0.000 0.007 0.007

0.022 0.000 0.000 0.007 0.060 0.053 0.060 0.065 0.005 0.000

0.000 0.005 0.000 0.000 0.005 0.005 0.000 0.010 0.000 0.000

0.005 0.005 0.010 0.000 0.000 0.000 0.010 0.000 0.079 0.072

0.060 0.065

>Non-Methylation code 355

0.005 0.005 0.000 0.005 0.010 0.000 0.010 0.000 0.005 0.000

0.000 0.010 0.000 0.000 0.000 0.005 0.000 0.005 0.000 0.005

0.067 0.084 0.067 0.054 0.015 0.000 0.000 0.000 0.000 0.000

0.000 0.022 0.000 0.007 0.007 0.000 0.007 0.000 0.007 0.022

0.007 0.000 0.000 0.000 0.067 0.055 0.071 0.046 0.000 0.010

0.000 0.005 0.005 0.000 0.000 0.000 0.000 0.010 0.000 0.000

0.010 0.005 0.005 0.000 0.000 0.005 0.000 0.000 0.061 0.087

0.069 0.060

>Non-Methylation code 356

0.018 0.000 0.000 0.000 0.000 0.000 0.006 0.000 0.000 0.000

0.000 0.000 0.006 0.000 0.018 0.006 0.018 0.000 0.000 0.006

0.065 0.080 0.050 0.060 0.000 0.000 0.000 0.008 0.000 0.016

0.000 0.000 0.000 0.008 0.000 0.000 0.039 0.016 0.000 0.000

0.016 0.000 0.000 0.000 0.062 0.066 0.046 0.058 0.000 0.005

0.000 0.000 0.000 0.005 0.011 0.000 0.005 0.005 0.000 0.005

0.011 0.005 0.005 0.000 0.000 0.005 0.000 0.000 0.073 0.074

0.055 0.067

>Non-Methylation code 357

0.013 0.000 0.000 0.007 0.007 0.000 0.000 0.007 0.000 0.027

0.000 0.000 0.000 0.000 0.007 0.013 0.007 0.000 0.000 0.000

0.060 0.063 0.058 0.066 0.000 0.000 0.006 0.000 0.011 0.000

0.000 0.000 0.006 0.000 0.000 0.000 0.006 0.006 0.011 0.011

0.000 0.000 0.000 0.000 0.079 0.070 0.058 0.070 0.006 0.006

0.006 0.006 0.006 0.000 0.006 0.000 0.006 0.006 0.000 0.000

0.000 0.000 0.000 0.013 0.006 0.006 0.000 0.000 0.073 0.069

0.059 0.056

>Non-Methylation code 358

0.013 0.000 0.000 0.000 0.006 0.019 0.000 0.000 0.000 0.013

0.000 0.000 0.000 0.006 0.006 0.000 0.000 0.000 0.013 0.000

0.058 0.073 0.061 0.066 0.005 0.005 0.005 0.005 0.005 0.014

0.000 0.000 0.000 0.009 0.000 0.000 0.005 0.000 0.005 0.005

0.000 0.000 0.000 0.000 0.067 0.070 0.066 0.072 0.008 0.000

0.000 0.008 0.000 0.017 0.000 0.000 0.000 0.025 0.008 0.000

0.000 0.000 0.000 0.008 0.000 0.008 0.017 0.000 0.074 0.049

0.051 0.060

>Non-Methylation code 359

0.005 0.000 0.000 0.000 0.000 0.000 0.000 0.000 0.000 0.005

0.000 0.000 0.005 0.000 0.015 0.010 0.005 0.005 0.015 0.000

0.050 0.054 0.085 0.081 0.008 0.000 0.008 0.008 0.000 0.034

0.000 0.000 0.000 0.017 0.000 0.000 0.008 0.008 0.000 0.017

0.000 0.000 0.000 0.000 0.060 0.046 0.053 0.064 0.008 0.000

0.000 0.008 0.000 0.008 0.008 0.000 0.008 0.032 0.000 0.000

0.000 0.000 0.000 0.016 0.008 0.000 0.000 0.000 0.070 0.048

0.042 0.076

>Non-Methylation code 360

0.000 0.000 0.018 0.012 0.000 0.006 0.000 0.000 0.000 0.000

0.006 0.000 0.000 0.006 0.012 0.006 0.012 0.000 0.000 0.000

0.078 0.065 0.043 0.069 0.006 0.000 0.000 0.006 0.000 0.006

0.000 0.000 0.013 0.013 0.000 0.000 0.000 0.006 0.006 0.000

0.019 0.000 0.000 0.000 0.076 0.046 0.068 0.067 0.004 0.000

0.004 0.000 0.000 0.004 0.004 0.000 0.004 0.004 0.000 0.000

0.000 0.000 0.021 0.004 0.000 0.000 0.000 0.000 0.046 0.069

0.087 0.080

>Non-Methylation code 361

0.007 0.000 0.000 0.007 0.000 0.007 0.007 0.000 0.000 0.026

0.000 0.000 0.007 0.000 0.007 0.013 0.007 0.000 0.000 0.000

0.079 0.060 0.043 0.067 0.007 0.000 0.000 0.000 0.000 0.007

0.000 0.000 0.000 0.020 0.000 0.000 0.007 0.000 0.000 0.013

0.007 0.007 0.020 0.000 0.060 0.052 0.064 0.071 0.023 0.000

0.000 0.000 0.000 0.034 0.000 0.000 0.000 0.023 0.000 0.000

0.011 0.011 0.000 0.023 0.000 0.000 0.000 0.000 0.046 0.042

0.057 0.062

>Non-Methylation code 362

0.000 0.000 0.000 0.000 0.000 0.012 0.000 0.006 0.006 0.006

0.000 0.000 0.000 0.000 0.006 0.006 0.018 0.000 0.012 0.000

0.075 0.071 0.067 0.049 0.000 0.004 0.000 0.000 0.000 0.017

0.000 0.000 0.000 0.004 0.000 0.004 0.009 0.000 0.013 0.000

0.000 0.004 0.000 0.000 0.074 0.066 0.061 0.077 0.006 0.000

0.017 0.006 0.006 0.006 0.006 0.000 0.000 0.006 0.000 0.000

0.000 0.000 0.000 0.000 0.006 0.000 0.006 0.000 0.062 0.061

0.076 0.072

>Non-Methylation code 363

0.000 0.005 0.005 0.000 0.000 0.010 0.005 0.005 0.000 0.000

0.000 0.005 0.010 0.000 0.005 0.000 0.000 0.005 0.000 0.005

0.073 0.052 0.064 0.082 0.008 0.000 0.008 0.000 0.008 0.008

0.008 0.016 0.000 0.008 0.008 0.000 0.000 0.000 0.000 0.008

0.008 0.008 0.000 0.000 0.064 0.051 0.071 0.049 0.000 0.006

0.006 0.000 0.000 0.006 0.000 0.000 0.000 0.011 0.000 0.000

0.006 0.000 0.006 0.011 0.006 0.000 0.006 0.000 0.066 0.060

0.065 0.079

>Non-Methylation code 364

0.000 0.000 0.000 0.000 0.007 0.030 0.000 0.000 0.007 0.007

0.000 0.000 0.007 0.000 0.007 0.007 0.015 0.000 0.000 0.000

0.057 0.074 0.057 0.057 0.005 0.005 0.000 0.009 0.000 0.009

0.000 0.000 0.005 0.000 0.000 0.000 0.009 0.005 0.005 0.005

0.000 0.000 0.000 0.005 0.077 0.060 0.069 0.067 0.006 0.000

0.000 0.006 0.000 0.006 0.006 0.000 0.000 0.006 0.000 0.006

0.000 0.000 0.017 0.006 0.006 0.006 0.000 0.000 0.054 0.074

0.073 0.066

>Non-Methylation code 365

0.000 0.000 0.005 0.005 0.000 0.000 0.000 0.000 0.005 0.000

0.000 0.005 0.000 0.000 0.015 0.000 0.005 0.015 0.005 0.000

0.066 0.070 0.072 0.067 0.000 0.000 0.000 0.008 0.000 0.034

0.000 0.000 0.008 0.008 0.008 0.000 0.008 0.000 0.000 0.017

0.008 0.000 0.000 0.000 0.055 0.064 0.056 0.057 0.000 0.000

0.000 0.014 0.000 0.014 0.000 0.000 0.007 0.000 0.000 0.000

0.007 0.007 0.007 0.007 0.000 0.000 0.000 0.007 0.061 0.060

0.064 0.078

>Non-Methylation code 366

0.000 0.000 0.006 0.000 0.011 0.006 0.011 0.000 0.006 0.017

0.000 0.000 0.006 0.006 0.000 0.000 0.000 0.000 0.000 0.000

0.082 0.064 0.048 0.073 0.008 0.008 0.000 0.000 0.008 0.000

0.000 0.008 0.008 0.008 0.000 0.000 0.000 0.000 0.000 0.025

0.017 0.000 0.008 0.000 0.076 0.047 0.054 0.056 0.000 0.000

0.000 0.005 0.000 0.000 0.000 0.000 0.000 0.005 0.000 0.000

0.016 0.000 0.011 0.016 0.000 0.011 0.000 0.000 0.080 0.052

0.059 0.077

>Non-Methylation code 367

0.000 0.000 0.006 0.000 0.006 0.013 0.000 0.000 0.006 0.000

0.000 0.000 0.000 0.006 0.006 0.006 0.013 0.000 0.000 0.000

0.058 0.073 0.066 0.073 0.005 0.005 0.005 0.010 0.000 0.005

0.000 0.000 0.005 0.005 0.000 0.000 0.005 0.000 0.005 0.010

0.005 0.000 0.000 0.000 0.083 0.062 0.063 0.058 0.006 0.000

0.000 0.006 0.000 0.006 0.006 0.000 0.000 0.026 0.000 0.006

0.000 0.000 0.006 0.006 0.006 0.000 0.000 0.000 0.065 0.070

0.067 0.054

>Non-Methylation code 368

0.000 0.000 0.000 0.011 0.011 0.006 0.000 0.000 0.000 0.006

0.000 0.006 0.000 0.011 0.006 0.006 0.006 0.000 0.006 0.000

0.073 0.072 0.059 0.056 0.017 0.000 0.009 0.000 0.000 0.009

0.000 0.000 0.009 0.017 0.000 0.017 0.000 0.000 0.000 0.026

0.009 0.000 0.000 0.000 0.040 0.056 0.062 0.065 0.000 0.000

0.000 0.005 0.000 0.000 0.000 0.005 0.000 0.005 0.000 0.000

0.010 0.005 0.010 0.000 0.005 0.005 0.005 0.000 0.069 0.067

0.065 0.077

>Non-Methylation code 369

0.000 0.000 0.013 0.000 0.006 0.006 0.000 0.000 0.000 0.000

0.000 0.000 0.013 0.000 0.000 0.026 0.000 0.006 0.000 0.006

0.047 0.072 0.070 0.066 0.007 0.000 0.000 0.000 0.007 0.000

0.007 0.007 0.000 0.007 0.007 0.000 0.007 0.000 0.007 0.014

0.007 0.000 0.000 0.000 0.073 0.061 0.060 0.062 0.000 0.005

0.000 0.000 0.011 0.000 0.000 0.000 0.000 0.005 0.000 0.000

0.005 0.005 0.005 0.005 0.000 0.011 0.005 0.000 0.057 0.072

0.080 0.067

>Non-Methylation code 370

0.007 0.000 0.007 0.007 0.007 0.007 0.007 0.015 0.000 0.007

0.000 0.000 0.000 0.000 0.000 0.007 0.007 0.007 0.000 0.000

0.065 0.057 0.071 0.053 0.000 0.000 0.000 0.000 0.000 0.006

0.000 0.000 0.006 0.011 0.000 0.000 0.006 0.000 0.006 0.011

0.011 0.000 0.011 0.000 0.070 0.063 0.072 0.061 0.000 0.005

0.000 0.000 0.000 0.015 0.005 0.000 0.000 0.000 0.000 0.005

0.010 0.000 0.010 0.000 0.000 0.005 0.000 0.005 0.074 0.055

0.063 0.081

>Non-Methylation code 371

0.000 0.000 0.000 0.006 0.006 0.000 0.000 0.000 0.000 0.006

0.006 0.000 0.012 0.012 0.006 0.018 0.000 0.000 0.000 0.000

0.067 0.072 0.059 0.063 0.005 0.000 0.005 0.000 0.010 0.000

0.005 0.000 0.000 0.005 0.000 0.000 0.005 0.005 0.010 0.005

0.000 0.005 0.005 0.000 0.084 0.055 0.074 0.059 0.007 0.000

0.007 0.000 0.007 0.007 0.000 0.007 0.007 0.007 0.000 0.000

0.014 0.000 0.000 0.014 0.007 0.000 0.000 0.000 0.073 0.063

0.052 0.061

>Non-Methylation code 372

0.005 0.000 0.000 0.000 0.005 0.000 0.010 0.000 0.000 0.010

0.000 0.000 0.005 0.005 0.010 0.005 0.000 0.005 0.000 0.000

0.078 0.055 0.064 0.075 0.010 0.000 0.000 0.000 0.005 0.000

0.005 0.000 0.000 0.000 0.000 0.005 0.000 0.000 0.016 0.010

0.010 0.000 0.000 0.005 0.084 0.061 0.057 0.063 0.007 0.000

0.000 0.000 0.000 0.007 0.000 0.013 0.000 0.013 0.000 0.000

0.020 0.000 0.007 0.007 0.007 0.000 0.000 0.000 0.061 0.074

0.062 0.056

>Non-Methylation code 373

0.014 0.000 0.007 0.000 0.000 0.021 0.000 0.000 0.000 0.007

0.000 0.000 0.000 0.000 0.007 0.000 0.000 0.007 0.000 0.000

0.051 0.069 0.079 0.070 0.000 0.000 0.000 0.017 0.000 0.011

0.000 0.000 0.000 0.000 0.000 0.000 0.000 0.006 0.006 0.006

0.006 0.000 0.017 0.000 0.083 0.053 0.065 0.065 0.000 0.000

0.000 0.000 0.000 0.040 0.000 0.000 0.000 0.008 0.000 0.000

0.000 0.008 0.008 0.024 0.000 0.008 0.000 0.000 0.037 0.055

0.066 0.079

>Non-Methylation code 374

0.000 0.000 0.000 0.006 0.006 0.012 0.000 0.000 0.000 0.012

0.000 0.006 0.000 0.006 0.006 0.012 0.006 0.000 0.006 0.000

0.076 0.074 0.050 0.058 0.034 0.000 0.000 0.011 0.000 0.011

0.000 0.000 0.000 0.011 0.000 0.011 0.011 0.000 0.000 0.034

0.011 0.000 0.000 0.000 0.042 0.042 0.056 0.056 0.000 0.000

0.000 0.005 0.000 0.000 0.000 0.000 0.000 0.010 0.000 0.005

0.010 0.010 0.010 0.000 0.000 0.005 0.005 0.000 0.074 0.059

0.073 0.064

>Non-Methylation code 375

0.016 0.000 0.005 0.000 0.000 0.005 0.021 0.000 0.000 0.005

0.000 0.000 0.000 0.011 0.005 0.000 0.000 0.000 0.000 0.000

0.045 0.064 0.073 0.083 0.014 0.000 0.000 0.000 0.000 0.000

0.000 0.014 0.000 0.007 0.007 0.000 0.007 0.000 0.007 0.021

0.014 0.000 0.000 0.000 0.052 0.062 0.058 0.069 0.010 0.005

0.000 0.000 0.000 0.005 0.000 0.000 0.000 0.000 0.000 0.000

0.015 0.000 0.010 0.010 0.000 0.000 0.000 0.000 0.064 0.059

0.087 0.070

>Non-Methylation code 376

0.011 0.006 0.000 0.000 0.000 0.000 0.000 0.000 0.006 0.000

0.000 0.000 0.006 0.000 0.011 0.022 0.006 0.000 0.000 0.000

0.062 0.081 0.064 0.059 0.015 0.007 0.000 0.000 0.000 0.015

0.015 0.000 0.000 0.000 0.000 0.000 0.029 0.007 0.000 0.007

0.000 0.000 0.000 0.000 0.055 0.081 0.063 0.040 0.007 0.000

0.000 0.007 0.000 0.007 0.000 0.007 0.000 0.007 0.007 0.000

0.007 0.015 0.007 0.007 0.000 0.007 0.000 0.000 0.066 0.061

0.049 0.068

>Non-Methylation code 377

0.012 0.000 0.000 0.012 0.000 0.006 0.000 0.000 0.000 0.000

0.006 0.000 0.006 0.012 0.006 0.000 0.000 0.006 0.006 0.000

0.060 0.066 0.068 0.069 0.000 0.004 0.000 0.000 0.000 0.011

0.000 0.000 0.000 0.000 0.000 0.000 0.004 0.004 0.014 0.004

0.000 0.000 0.004 0.004 0.087 0.057 0.060 0.084 0.012 0.000

0.006 0.000 0.000 0.035 0.000 0.000 0.006 0.000 0.006 0.000

0.000 0.000 0.006 0.000 0.000 0.000 0.000 0.000 0.074 0.046

0.070 0.073

>Non-Methylation code 378

0.000 0.000 0.000 0.007 0.000 0.027 0.000 0.007 0.007 0.000

0.000 0.007 0.000 0.000 0.000 0.000 0.000 0.013 0.007 0.000

0.063 0.065 0.064 0.068 0.011 0.000 0.000 0.006 0.000 0.011

0.000 0.006 0.017 0.011 0.000 0.000 0.000 0.000 0.000 0.006

0.000 0.000 0.006 0.000 0.059 0.073 0.060 0.068 0.000 0.004

0.000 0.000 0.000 0.008 0.000 0.000 0.004 0.004 0.000 0.000

0.000 0.000 0.013 0.004 0.000 0.004 0.004 0.000 0.073 0.082

0.039 0.092

>Non-Methylation code 379

0.006 0.006 0.000 0.006 0.000 0.000 0.000 0.013 0.000 0.006

0.006 0.000 0.006 0.013 0.006 0.006 0.006 0.000 0.000 0.000

0.056 0.066 0.064 0.066 0.006 0.006 0.000 0.006 0.000 0.000

0.000 0.000 0.006 0.000 0.000 0.000 0.012 0.000 0.012 0.018

0.000 0.000 0.000 0.000 0.072 0.083 0.051 0.063 0.015 0.000

0.005 0.005 0.000 0.000 0.015 0.000 0.005 0.005 0.000 0.000

0.005 0.000 0.005 0.000 0.000 0.000 0.000 0.000 0.062 0.063

0.069 0.077

>Non-Methylation code 380

0.021 0.000 0.000 0.000 0.000 0.004 0.000 0.000 0.000 0.004

0.000 0.000 0.004 0.000 0.013 0.004 0.000 0.000 0.004 0.000

0.054 0.072 0.079 0.073 0.013 0.006 0.000 0.006 0.000 0.006

0.000 0.000 0.000 0.013 0.000 0.000 0.000 0.019 0.006 0.000

0.000 0.006 0.000 0.000 0.059 0.054 0.066 0.078 0.016 0.000

0.000 0.000 0.000 0.008 0.000 0.000 0.000 0.008 0.000 0.000

0.008 0.008 0.000 0.041 0.000 0.000 0.008 0.000 0.051 0.057

0.054 0.073

>Non-Methylation code 381

0.021 0.014 0.000 0.000 0.000 0.000 0.000 0.000 0.000 0.007

0.000 0.007 0.014 0.007 0.000 0.000 0.000 0.014 0.000 0.000

0.060 0.066 0.071 0.052 0.004 0.000 0.000 0.000 0.000 0.000

0.000 0.000 0.000 0.000 0.000 0.000 0.008 0.000 0.017 0.008

0.004 0.000 0.004 0.004 0.064 0.075 0.065 0.079 0.010 0.005

0.000 0.000 0.000 0.015 0.000 0.000 0.000 0.005 0.000 0.000

0.005 0.005 0.010 0.000 0.005 0.000 0.000 0.000 0.039 0.068

0.078 0.089

>Non-Methylation code 382

0.000 0.000 0.004 0.000 0.000 0.009 0.004 0.000 0.004 0.009

0.000 0.000 0.004 0.004 0.009 0.004 0.000 0.000 0.004 0.000

0.081 0.056 0.065 0.075 0.012 0.000 0.000 0.000 0.000 0.024

0.000 0.006 0.006 0.006 0.000 0.000 0.000 0.000 0.012 0.000

0.006 0.000 0.000 0.000 0.068 0.055 0.077 0.062 0.011 0.000

0.000 0.006 0.006 0.011 0.000 0.000 0.006 0.000 0.000 0.000

0.006 0.011 0.006 0.000 0.000 0.000 0.000 0.000 0.068 0.071

0.069 0.065

>Non-Methylation code 383

0.014 0.007 0.000 0.000 0.000 0.014 0.014 0.000 0.000 0.000

0.000 0.000 0.027 0.007 0.000 0.007 0.000 0.000 0.000 0.000

0.051 0.084 0.069 0.040 0.006 0.000 0.000 0.006 0.000 0.006

0.006 0.006 0.000 0.006 0.006 0.000 0.006 0.006 0.012 0.006

0.000 0.006 0.000 0.000 0.057 0.064 0.063 0.074 0.012 0.006

0.000 0.000 0.000 0.000 0.000 0.000 0.006 0.000 0.000 0.000

0.006 0.000 0.012 0.018 0.006 0.000 0.000 0.000 0.077 0.078

0.055 0.055

>Non-Methylation code 384

0.005 0.005 0.000 0.000 0.000 0.009 0.000 0.000 0.000 0.009

0.000 0.005 0.005 0.000 0.014 0.005 0.000 0.005 0.000 0.000

0.080 0.082 0.029 0.081 0.000 0.000 0.000 0.000 0.000 0.018

0.000 0.000 0.000 0.000 0.000 0.000 0.006 0.006 0.006 0.006

0.006 0.006 0.012 0.000 0.064 0.072 0.061 0.070 0.016 0.000

0.000 0.008 0.000 0.016 0.000 0.000 0.000 0.008 0.000 0.000

0.024 0.000 0.000 0.008 0.000 0.008 0.008 0.000 0.053 0.063

0.070 0.050

>Non-Methylation code 385

0.006 0.006 0.000 0.000 0.000 0.006 0.000 0.000 0.000 0.011

0.000 0.000 0.011 0.006 0.006 0.006 0.006 0.006 0.006 0.000

0.051 0.074 0.063 0.070 0.005 0.011 0.000 0.000 0.000 0.005

0.000 0.000 0.000 0.000 0.000 0.005 0.011 0.005 0.005 0.005

0.000 0.016 0.000 0.000 0.077 0.083 0.067 0.038 0.011 0.000

0.000 0.000 0.000 0.000 0.000 0.000 0.000 0.000 0.000 0.000

0.011 0.005 0.011 0.005 0.005 0.000 0.000 0.005 0.047 0.079

0.082 0.074

>Non-Methylation code 386

0.004 0.004 0.000 0.000 0.009 0.009 0.000 0.000 0.004 0.004

0.000 0.000 0.009 0.004 0.004 0.000 0.004 0.000 0.000 0.000

0.078 0.062 0.059 0.079 0.010 0.000 0.005 0.000 0.005 0.005

0.005 0.000 0.005 0.014 0.000 0.000 0.000 0.005 0.010 0.000

0.000 0.000 0.000 0.000 0.070 0.064 0.066 0.071 0.006 0.000

0.000 0.000 0.000 0.006 0.000 0.006 0.000 0.013 0.000 0.000

0.000 0.006 0.013 0.006 0.006 0.000 0.000 0.006 0.084 0.071

0.047 0.060

>Non-Methylation code 387

0.009 0.005 0.000 0.000 0.000 0.005 0.000 0.000 0.000 0.000

0.000 0.000 0.019 0.000 0.009 0.009 0.000 0.000 0.000 0.000

0.066 0.062 0.082 0.067 0.015 0.000 0.005 0.000 0.000 0.005

0.020 0.000 0.000 0.010 0.000 0.000 0.000 0.005 0.005 0.000

0.000 0.000 0.000 0.000 0.051 0.064 0.070 0.083 0.015 0.000

0.000 0.000 0.000 0.000 0.000 0.015 0.000 0.007 0.007 0.000

0.007 0.000 0.007 0.015 0.015 0.000 0.000 0.000 0.053 0.064

0.057 0.072

>Non-Methylation code 388

0.035 0.000 0.007 0.007 0.000 0.000 0.000 0.000 0.000 0.007

0.000 0.000 0.007 0.000 0.007 0.007 0.007 0.000 0.000 0.000

0.065 0.074 0.062 0.048 0.000 0.000 0.000 0.004 0.000 0.004

0.000 0.000 0.000 0.000 0.000 0.000 0.017 0.000 0.022 0.004

0.004 0.000 0.000 0.000 0.058 0.069 0.066 0.084 0.010 0.000

0.000 0.000 0.000 0.015 0.005 0.000 0.000 0.005 0.000 0.000

0.000 0.000 0.015 0.005 0.000 0.000 0.000 0.000 0.061 0.065

0.084 0.067

>Non-Methylation code 389

0.007 0.000 0.000 0.000 0.007 0.000 0.000 0.000 0.014 0.000

0.007 0.000 0.014 0.007 0.000 0.014 0.000 0.014 0.000 0.007

0.062 0.052 0.059 0.069 0.006 0.006 0.000 0.000 0.012 0.000

0.006 0.000 0.012 0.012 0.000 0.000 0.000 0.006 0.000 0.012

0.006 0.000 0.000 0.000 0.059 0.088 0.062 0.048 0.006 0.006

0.000 0.000 0.006 0.000 0.000 0.006 0.011 0.000 0.000 0.000

0.000 0.006 0.006 0.011 0.000 0.006 0.000 0.006 0.065 0.061

0.054 0.087

>Non-Methylation code 390

0.014 0.000 0.000 0.000 0.000 0.005 0.000 0.000 0.000 0.009

0.000 0.000 0.005 0.000 0.014 0.005 0.005 0.000 0.000 0.005

0.041 0.068 0.088 0.076 0.005 0.005 0.000 0.000 0.000 0.014

0.000 0.000 0.000 0.000 0.000 0.000 0.005 0.009 0.014 0.005

0.005 0.000 0.000 0.000 0.053 0.068 0.078 0.072 0.028 0.000

0.007 0.007 0.000 0.000 0.000 0.000 0.000 0.014 0.000 0.000

0.007 0.000 0.007 0.014 0.000 0.000 0.000 0.000 0.069 0.073

0.044 0.064

>Non-Methylation code 391

0.000 0.004 0.008 0.000 0.004 0.012 0.000 0.000 0.000 0.000

0.000 0.004 0.004 0.000 0.004 0.004 0.000 0.000 0.004 0.000

0.074 0.059 0.081 0.069 0.032 0.000 0.000 0.011 0.000 0.021

0.000 0.000 0.000 0.011 0.000 0.000 0.000 0.000 0.000 0.011

0.032 0.021 0.000 0.000 0.039 0.050 0.052 0.054 0.000 0.000

0.000 0.005 0.000 0.005 0.000 0.000 0.000 0.022 0.000 0.000

0.005 0.005 0.011 0.000 0.000 0.000 0.005 0.000 0.073 0.061

0.054 0.084

>Non-Methylation code 392

0.000 0.000 0.006 0.011 0.000 0.006 0.000 0.006 0.017 0.000

0.000 0.000 0.006 0.006 0.017 0.000 0.000 0.000 0.000 0.000

0.053 0.061 0.068 0.079 0.000 0.000 0.000 0.010 0.000 0.010

0.000 0.005 0.014 0.005 0.000 0.005 0.000 0.000 0.010 0.005

0.000 0.000 0.000 0.000 0.071 0.068 0.062 0.071 0.006 0.000

0.000 0.006 0.000 0.006 0.000 0.000 0.013 0.000 0.000 0.006

0.000 0.000 0.019 0.006 0.006 0.000 0.000 0.000 0.057 0.077

0.072 0.058

>Non-Methylation code 393

0.008 0.000 0.008 0.008 0.000 0.023 0.000 0.000 0.000 0.008

0.000 0.008 0.000 0.000 0.000 0.008 0.015 0.008 0.000 0.008

0.062 0.067 0.058 0.047 0.008 0.000 0.000 0.008 0.000 0.008

0.000 0.008 0.000 0.015 0.000 0.000 0.008 0.008 0.008 0.008

0.015 0.000 0.000 0.000 0.062 0.056 0.054 0.068 0.005 0.005

0.005 0.000 0.005 0.000 0.000 0.000 0.000 0.005 0.000 0.005

0.000 0.000 0.019 0.005 0.000 0.000 0.000 0.000 0.105 0.037

0.101 0.039

>Non-Methylation code 394

0.000 0.000 0.000 0.006 0.000 0.039 0.006 0.006 0.013 0.006

0.000 0.000 0.000 0.000 0.000 0.000 0.000 0.006 0.000 0.000

0.070 0.049 0.056 0.074 0.017 0.006 0.000 0.000 0.000 0.011

0.000 0.000 0.006 0.000 0.000 0.000 0.000 0.000 0.011 0.006

0.006 0.000 0.000 0.000 0.062 0.068 0.056 0.084 0.008 0.004

0.000 0.004 0.000 0.008 0.000 0.000 0.000 0.000 0.000 0.004

0.000 0.004 0.017 0.000 0.000 0.000 0.000 0.000 0.078 0.072

0.079 0.054

>Non-Methylation code 395

0.014 0.000 0.000 0.000 0.009 0.005 0.000 0.000 0.000 0.009

0.000 0.000 0.009 0.000 0.009 0.005 0.000 0.000 0.000 0.000

0.063 0.058 0.073 0.080 0.006 0.000 0.000 0.000 0.006 0.011

0.000 0.000 0.000 0.000 0.000 0.000 0.017 0.006 0.011 0.017

0.000 0.000 0.000 0.000 0.078 0.075 0.040 0.067 0.010 0.000

0.000 0.000 0.005 0.005 0.000 0.000 0.000 0.005 0.000 0.000

0.020 0.000 0.010 0.000 0.000 0.005 0.000 0.000 0.070 0.056

0.070 0.077

>Non-Methylation code 396

0.007 0.007 0.007 0.000 0.000 0.007 0.007 0.007 0.000 0.007

0.000 0.000 0.028 0.007 0.000 0.000 0.000 0.007 0.000 0.000

0.072 0.050 0.077 0.042 0.004 0.000 0.004 0.000 0.004 0.000

0.000 0.004 0.000 0.000 0.000 0.000 0.013 0.000 0.013 0.004

0.004 0.000 0.000 0.004 0.090 0.053 0.076 0.056 0.005 0.000

0.000 0.000 0.000 0.005 0.005 0.000 0.000 0.000 0.000 0.000

0.011 0.000 0.016 0.005 0.011 0.000 0.000 0.000 0.080 0.060

0.081 0.053

>Non-Methylation code 397

0.017 0.006 0.000 0.000 0.000 0.012 0.000 0.000 0.000 0.000

0.000 0.000 0.012 0.000 0.012 0.012 0.000 0.000 0.000 0.000

0.073 0.082 0.064 0.044 0.020 0.000 0.000 0.013 0.000 0.013

0.000 0.000 0.000 0.013 0.000 0.000 0.000 0.007 0.013 0.000

0.000 0.007 0.000 0.000 0.048 0.052 0.072 0.076 0.006 0.000

0.000 0.000 0.000 0.012 0.000 0.000 0.012 0.006 0.000 0.000

0.006 0.000 0.012 0.012 0.000 0.000 0.000 0.000 0.084 0.069

0.030 0.086

>Non-Methylation code 398

0.005 0.000 0.000 0.000 0.005 0.005 0.005 0.000 0.000 0.000

0.000 0.000 0.010 0.000 0.014 0.005 0.010 0.000 0.000 0.000

0.080 0.058 0.087 0.051 0.007 0.007 0.007 0.000 0.007 0.007

0.007 0.007 0.000 0.007 0.000 0.000 0.028 0.000 0.000 0.000

0.000 0.007 0.000 0.000 0.068 0.056 0.070 0.050 0.005 0.000

0.005 0.000 0.005 0.000 0.000 0.005 0.000 0.000 0.000 0.000

0.015 0.000 0.010 0.005 0.005 0.000 0.000 0.005 0.089 0.060

0.072 0.052

>Non-Methylation code 399

0.021 0.000 0.000 0.000 0.000 0.016 0.000 0.000 0.000 0.000

0.000 0.000 0.005 0.000 0.011 0.005 0.005 0.000 0.005 0.000

0.063 0.066 0.079 0.055 0.028 0.000 0.009 0.000 0.000 0.028

0.000 0.000 0.000 0.000 0.000 0.000 0.057 0.000 0.000 0.000

0.000 0.000 0.000 0.000 0.046 0.042 0.062 0.061 0.006 0.000

0.000 0.011 0.000 0.000 0.006 0.000 0.000 0.000 0.006 0.000

0.000 0.000 0.039 0.000 0.000 0.000 0.000 0.000 0.066 0.050

0.075 0.076

>Non-Methylation code 400

0.020 0.007 0.000 0.000 0.000 0.007 0.000 0.000 0.000 0.007

0.000 0.000 0.000 0.013 0.007 0.020 0.007 0.000 0.000 0.000

0.054 0.056 0.069 0.068 0.008 0.004 0.000 0.004 0.000 0.004

0.000 0.000 0.000 0.004 0.000 0.000 0.000 0.004 0.015 0.004

0.000 0.000 0.000 0.000 0.059 0.067 0.080 0.080 0.007 0.007

0.007 0.007 0.000 0.007 0.000 0.000 0.007 0.000 0.000 0.000

0.007 0.007 0.000 0.014 0.000 0.007 0.000 0.000 0.076 0.072

0.059 0.051

>Non-Methylation code 401

0.008 0.004 0.000 0.004 0.000 0.017 0.000 0.004 0.000 0.000

0.000 0.000 0.004 0.000 0.008 0.000 0.000 0.000 0.004 0.000

0.075 0.058 0.082 0.065 0.021 0.000 0.000 0.000 0.005 0.016

0.000 0.000 0.000 0.000 0.000 0.000 0.010 0.000 0.010 0.000

0.000 0.005 0.000 0.000 0.074 0.068 0.066 0.057 0.005 0.000

0.005 0.000 0.000 0.014 0.000 0.000 0.000 0.009 0.000 0.000

0.000 0.005 0.014 0.005 0.000 0.000 0.000 0.000 0.056 0.077

0.084 0.062

>Non-Methylation code 402

0.021 0.000 0.000 0.000 0.000 0.000 0.000 0.000 0.000 0.031

0.000 0.000 0.031 0.000 0.000 0.031 0.021 0.000 0.000 0.000

0.066 0.042 0.043 0.047 0.000 0.007 0.000 0.000 0.000 0.000

0.007 0.000 0.000 0.011 0.000 0.000 0.004 0.000 0.015 0.000

0.000 0.000 0.004 0.000 0.095 0.059 0.096 0.037 0.029 0.007

0.007 0.000 0.007 0.007 0.000 0.007 0.000 0.007 0.000 0.000

0.000 0.000 0.000 0.000 0.000 0.007 0.000 0.007 0.061 0.067

0.065 0.052

>Non-Methylation code 403

0.011 0.000 0.000 0.006 0.000 0.017 0.000 0.000 0.006 0.000

0.000 0.000 0.011 0.000 0.011 0.011 0.000 0.000 0.000 0.000

0.072 0.082 0.054 0.053 0.019 0.000 0.000 0.000 0.000 0.025

0.000 0.000 0.000 0.006 0.000 0.000 0.006 0.000 0.025 0.000

0.000 0.000 0.000 0.000 0.061 0.049 0.071 0.072 0.011 0.000

0.000 0.017 0.000 0.011 0.000 0.000 0.000 0.000 0.000 0.000

0.000 0.006 0.017 0.006 0.000 0.000 0.000 0.000 0.070 0.057

0.088 0.050

>Non-Methylation code 404

0.019 0.000 0.000 0.000 0.000 0.031 0.000 0.000 0.000 0.006

0.000 0.000 0.000 0.000 0.019 0.000 0.000 0.000 0.000 0.000

0.071 0.074 0.044 0.071 0.013 0.000 0.000 0.009 0.000 0.013

0.000 0.000 0.000 0.000 0.000 0.000 0.004 0.000 0.009 0.000

0.000 0.000 0.004 0.000 0.068 0.067 0.058 0.087 0.013 0.000

0.000 0.000 0.000 0.026 0.000 0.000 0.000 0.007 0.000 0.000

0.000 0.007 0.013 0.007 0.000 0.000 0.007 0.000 0.072 0.055

0.071 0.056

>Non-Methylation code 405

0.016 0.000 0.000 0.000 0.005 0.011 0.000 0.000 0.000 0.000

0.000 0.000 0.016 0.000 0.005 0.000 0.005 0.005 0.005 0.000

0.070 0.065 0.065 0.065 0.005 0.000 0.000 0.000 0.000 0.014

0.000 0.000 0.000 0.009 0.000 0.000 0.014 0.000 0.014 0.005

0.000 0.000 0.000 0.000 0.062 0.088 0.083 0.039 0.010 0.010

0.005 0.005 0.000 0.010 0.000 0.000 0.000 0.010 0.000 0.000

0.005 0.000 0.005 0.000 0.000 0.000 0.000 0.000 0.073 0.053

0.084 0.064

>Non-Methylation code 406

0.026 0.000 0.000 0.000 0.009 0.018 0.009 0.000 0.000 0.000

0.000 0.000 0.009 0.000 0.000 0.026 0.009 0.009 0.000 0.000

0.065 0.047 0.045 0.061 0.007 0.000 0.000 0.000 0.000 0.000

0.000 0.000 0.000 0.013 0.007 0.000 0.040 0.000 0.007 0.000

0.000 0.007 0.000 0.000 0.076 0.060 0.061 0.056 0.000 0.007

0.000 0.000 0.004 0.004 0.004 0.000 0.000 0.011 0.000 0.000

0.004 0.000 0.011 0.000 0.000 0.000 0.000 0.000 0.072 0.054

0.081 0.084

>Non-Methylation code 407

0.008 0.000 0.000 0.004 0.008 0.004 0.004 0.004 0.000 0.000

0.000 0.000 0.004 0.000 0.008 0.000 0.004 0.000 0.004 0.000

0.076 0.074 0.074 0.055 0.005 0.000 0.000 0.000 0.000 0.011

0.000 0.000 0.005 0.000 0.000 0.000 0.011 0.000 0.011 0.016

0.005 0.005 0.000 0.000 0.054 0.061 0.068 0.079 0.000 0.000

0.000 0.000 0.000 0.013 0.006 0.000 0.000 0.006 0.000 0.000

0.013 0.006 0.006 0.013 0.000 0.013 0.000 0.000 0.061 0.062

0.048 0.086

>Non-Methylation code 408

0.015 0.000 0.005 0.000 0.000 0.010 0.000 0.000 0.000 0.000

0.000 0.000 0.015 0.000 0.005 0.000 0.005 0.005 0.005 0.000

0.058 0.076 0.070 0.064 0.004 0.000 0.000 0.000 0.000 0.013

0.000 0.000 0.000 0.000 0.000 0.000 0.009 0.004 0.018 0.004

0.004 0.000 0.000 0.000 0.072 0.077 0.055 0.071 0.011 0.000

0.000 0.000 0.000 0.017 0.000 0.000 0.000 0.000 0.000 0.000

0.011 0.000 0.017 0.006 0.000 0.000 0.000 0.000 0.073 0.072

0.051 0.077

>Non-Methylation code 409

0.016 0.000 0.010 0.005 0.005 0.000 0.000 0.000 0.000 0.000

0.000 0.005 0.010 0.000 0.005 0.000 0.000 0.005 0.000 0.000

0.080 0.063 0.083 0.045 0.000 0.000 0.000 0.000 0.005 0.005

0.000 0.005 0.000 0.005 0.000 0.000 0.009 0.005 0.014 0.005

0.009 0.000 0.000 0.000 0.073 0.070 0.058 0.072 0.000 0.000

0.000 0.004 0.004 0.013 0.000 0.000 0.000 0.004 0.000 0.000

0.009 0.000 0.017 0.000 0.000 0.000 0.000 0.000 0.080 0.109

0.047 0.045

>Non-Methylation code 410

0.004 0.000 0.000 0.000 0.000 0.004 0.000 0.000 0.007 0.000

0.000 0.000 0.011 0.000 0.015 0.004 0.000 0.000 0.004 0.000

0.063 0.075 0.079 0.069 0.009 0.000 0.004 0.004 0.000 0.009

0.000 0.000 0.000 0.000 0.000 0.004 0.004 0.004 0.013 0.000

0.000 0.004 0.000 0.000 0.071 0.084 0.062 0.059 0.008 0.000

0.000 0.023 0.000 0.008 0.000 0.000 0.008 0.008 0.000 0.000

0.008 0.008 0.000 0.008 0.016 0.000 0.000 0.000 0.085 0.041

0.064 0.050

>Non-Methylation code 411

0.004 0.000 0.000 0.000 0.000 0.013 0.000 0.000 0.000 0.000

0.000 0.000 0.009 0.004 0.018 0.004 0.004 0.000 0.000 0.000

0.072 0.077 0.055 0.071 0.015 0.000 0.000 0.000 0.000 0.015

0.000 0.000 0.000 0.000 0.000 0.000 0.010 0.000 0.015 0.005

0.000 0.000 0.000 0.000 0.079 0.064 0.045 0.085 0.016 0.000

0.005 0.000 0.000 0.011 0.000 0.000 0.000 0.000 0.000 0.000

0.016 0.000 0.005 0.000 0.000 0.005 0.005 0.000 0.060 0.079

0.072 0.058

>Non-Methylation code 412

0.004 0.000 0.000 0.004 0.008 0.004 0.000 0.000 0.000 0.004

0.000 0.000 0.008 0.000 0.015 0.000 0.000 0.000 0.000 0.000

0.061 0.083 0.064 0.079 0.013 0.004 0.004 0.004 0.004 0.000

0.000 0.000 0.004 0.000 0.000 0.004 0.009 0.000 0.004 0.004

0.000 0.000 0.000 0.000 0.057 0.073 0.075 0.071 0.005 0.000

0.000 0.000 0.005 0.000 0.000 0.005 0.000 0.005 0.000 0.000

0.016 0.005 0.011 0.005 0.005 0.000 0.000 0.000 0.062 0.074

0.060 0.072

>Non-Methylation code 413

0.018 0.000 0.000 0.000 0.006 0.000 0.000 0.006 0.000 0.006

0.000 0.000 0.018 0.006 0.006 0.006 0.006 0.000 0.000 0.000

0.055 0.071 0.070 0.058 0.004 0.000 0.000 0.004 0.008 0.000

0.000 0.000 0.000 0.004 0.000 0.000 0.008 0.004 0.017 0.000

0.000 0.000 0.000 0.000 0.060 0.083 0.069 0.072 0.004 0.004

0.004 0.004 0.004 0.000 0.000 0.000 0.004 0.000 0.000 0.004

0.009 0.000 0.004 0.009 0.000 0.000 0.000 0.000 0.065 0.072

0.069 0.073

>Non-Methylation code 414

0.008 0.000 0.000 0.000 0.000 0.008 0.000 0.000 0.008 0.000

0.000 0.000 0.008 0.000 0.011 0.004 0.000 0.000 0.004 0.000

0.053 0.068 0.090 0.074 0.012 0.000 0.004 0.000 0.000 0.008

0.000 0.000 0.000 0.000 0.000 0.000 0.004 0.000 0.020 0.000

0.000 0.004 0.000 0.000 0.084 0.069 0.076 0.053 0.007 0.000

0.000 0.022 0.000 0.022 0.000 0.000 0.000 0.007 0.000 0.000

0.000 0.007 0.007 0.007 0.007 0.000 0.000 0.000 0.092 0.038

0.060 0.058

>Non-Methylation code 415

0.000 0.000 0.000 0.000 0.000 0.010 0.000 0.000 0.000 0.005

0.000 0.000 0.010 0.005 0.015 0.005 0.015 0.000 0.000 0.000

0.078 0.053 0.076 0.060 0.005 0.000 0.000 0.000 0.000 0.019

0.000 0.000 0.000 0.009 0.000 0.000 0.009 0.000 0.019 0.000

0.000 0.000 0.000 0.000 0.073 0.100 0.048 0.051 0.022 0.000

0.011 0.000 0.000 0.000 0.000 0.000 0.000 0.000 0.000 0.000

0.011 0.000 0.005 0.000 0.000 0.005 0.005 0.000 0.073 0.062

0.080 0.059

>Non-Methylation code 416

0.000 0.000 0.000 0.000 0.000 0.020 0.000 0.000 0.000 0.007

0.000 0.000 0.000 0.000 0.020 0.013 0.020 0.000 0.000 0.000

0.089 0.037 0.068 0.058 0.011 0.005 0.000 0.011 0.000 0.016

0.000 0.000 0.000 0.016 0.000 0.000 0.000 0.005 0.005 0.000

0.000 0.000 0.000 0.000 0.067 0.063 0.054 0.079 0.005 0.000

0.005 0.000 0.000 0.005 0.005 0.000 0.005 0.000 0.000 0.000

0.005 0.000 0.005 0.010 0.000 0.005 0.005 0.000 0.053 0.064

0.077 0.083

>Non-Methylation code 417

0.009 0.000 0.005 0.005 0.000 0.014 0.005 0.000 0.000 0.000

0.000 0.005 0.005 0.005 0.009 0.000 0.000 0.000 0.000 0.000

0.061 0.081 0.074 0.058 0.012 0.000 0.000 0.012 0.000 0.006

0.000 0.000 0.006 0.006 0.000 0.000 0.006 0.006 0.006 0.000

0.018 0.000 0.000 0.000 0.084 0.042 0.069 0.061 0.004 0.000

0.000 0.000 0.000 0.004 0.000 0.000 0.008 0.000 0.000 0.000

0.013 0.000 0.013 0.004 0.000 0.000 0.004 0.000 0.063 0.067

0.077 0.076

>Non-Methylation code 418

0.008 0.000 0.000 0.000 0.000 0.004 0.000 0.000 0.000 0.004

0.000 0.000 0.004 0.000 0.023 0.000 0.004 0.004 0.000 0.000

0.095 0.048 0.066 0.075 0.010 0.000 0.000 0.005 0.000 0.019

0.000 0.000 0.000 0.000 0.000 0.000 0.000 0.005 0.015 0.010

0.000 0.000 0.000 0.000 0.087 0.064 0.034 0.085 0.014 0.000

0.007 0.000 0.000 0.028 0.000 0.000 0.007 0.000 0.000 0.000

0.028 0.000 0.000 0.000 0.000 0.000 0.000 0.000 0.057 0.071

0.067 0.054

>Non-Methylation code 419

0.000 0.006 0.006 0.000 0.000 0.000 0.006 0.000 0.000 0.006

0.000 0.000 0.018 0.000 0.006 0.030 0.000 0.000 0.000 0.000

0.080 0.059 0.049 0.069 0.017 0.000 0.000 0.000 0.006 0.000

0.006 0.000 0.000 0.006 0.000 0.000 0.017 0.006 0.006 0.000

0.012 0.000 0.000 0.000 0.062 0.058 0.059 0.079 0.000 0.000

0.000 0.000 0.011 0.000 0.000 0.000 0.006 0.011 0.000 0.000

0.023 0.000 0.006 0.000 0.006 0.006 0.000 0.000 0.073 0.057

0.072 0.063

>Non-Methylation code 420

0.000 0.000 0.000 0.000 0.012 0.000 0.000 0.000 0.006 0.012

0.000 0.000 0.030 0.000 0.000 0.006 0.006 0.006 0.000 0.000

0.080 0.055 0.071 0.049 0.000 0.006 0.000 0.000 0.000 0.000

0.006 0.000 0.000 0.006 0.000 0.000 0.025 0.000 0.006 0.031

0.000 0.000 0.000 0.000 0.083 0.060 0.036 0.073 0.017 0.000

0.000 0.000 0.006 0.000 0.006 0.000 0.000 0.006 0.000 0.000

0.023 0.006 0.006 0.000 0.000 0.000 0.000 0.000 0.070 0.060

0.058 0.076

>Non-Methylation code 421

0.005 0.005 0.005 0.000 0.000 0.005 0.005 0.000 0.000 0.000

0.000 0.000 0.010 0.000 0.005 0.021 0.005 0.000 0.000 0.000

0.065 0.060 0.057 0.083 0.023 0.000 0.000 0.000 0.000 0.000

0.000 0.000 0.000 0.015 0.000 0.000 0.030 0.000 0.008 0.000

0.015 0.000 0.000 0.000 0.068 0.056 0.058 0.061 0.000 0.005

0.000 0.000 0.005 0.000 0.005 0.000 0.000 0.010 0.000 0.000

0.021 0.000 0.005 0.000 0.000 0.005 0.005 0.000 0.076 0.058

0.084 0.053

>Non-Methylation code 422

0.000 0.000 0.000 0.008 0.000 0.017 0.000 0.008 0.000 0.008

0.000 0.000 0.008 0.017 0.000 0.017 0.000 0.000 0.008 0.000

0.050 0.059 0.061 0.071 0.007 0.000 0.000 0.013 0.007 0.020

0.000 0.000 0.000 0.000 0.000 0.000 0.007 0.007 0.007 0.013

0.000 0.000 0.000 0.000 0.066 0.060 0.065 0.063 0.011 0.000

0.006 0.006 0.006 0.006 0.000 0.000 0.000 0.006 0.000 0.000

0.006 0.000 0.011 0.000 0.000 0.000 0.000 0.000 0.058 0.074

0.082 0.063

>Non-Methylation code 423

0.000 0.000 0.004 0.000 0.000 0.004 0.000 0.000 0.004 0.008

0.000 0.000 0.004 0.000 0.012 0.008 0.000 0.004 0.004 0.000

0.068 0.057 0.081 0.074 0.006 0.000 0.006 0.012 0.000 0.012

0.000 0.006 0.000 0.018 0.000 0.000 0.000 0.000 0.006 0.006

0.000 0.000 0.000 0.000 0.053 0.078 0.071 0.061 0.006 0.000

0.000 0.006 0.000 0.012 0.000 0.006 0.012 0.006 0.000 0.000

0.006 0.000 0.000 0.006 0.000 0.000 0.006 0.000 0.067 0.075

0.060 0.064

>Non-Methylation code 424

0.000 0.000 0.000 0.000 0.011 0.000 0.000 0.000 0.005 0.005

0.000 0.000 0.021 0.005 0.005 0.005 0.005 0.005 0.000 0.000

0.071 0.065 0.057 0.071 0.000 0.000 0.000 0.000 0.000 0.008

0.000 0.000 0.000 0.008 0.000 0.000 0.042 0.000 0.000 0.051

0.000 0.000 0.000 0.000 0.076 0.038 0.038 0.072 0.011 0.000

0.000 0.000 0.006 0.000 0.006 0.000 0.000 0.006 0.000 0.000

0.022 0.006 0.006 0.000 0.000 0.006 0.000 0.000 0.069 0.057

0.074 0.067

>Non-Methylation code 425

0.018 0.009 0.000 0.000 0.000 0.018 0.000 0.000 0.000 0.000

0.000 0.000 0.018 0.000 0.000 0.009 0.009 0.000 0.009 0.000

0.047 0.048 0.076 0.074 0.006 0.000 0.000 0.006 0.000 0.006

0.000 0.000 0.000 0.006 0.000 0.000 0.025 0.006 0.006 0.006

0.000 0.006 0.000 0.000 0.075 0.059 0.046 0.080 0.000 0.000

0.009 0.000 0.009 0.017 0.000 0.000 0.000 0.026 0.000 0.000

0.009 0.000 0.000 0.026 0.000 0.009 0.000 0.000 0.063 0.048

0.053 0.067

>Non-Methylation code 426

0.008 0.000 0.000 0.016 0.000 0.008 0.000 0.000 0.016 0.032

0.000 0.000 0.008 0.000 0.000 0.000 0.008 0.000 0.000 0.000

0.048 0.063 0.072 0.055 0.000 0.005 0.005 0.000 0.005 0.005

0.000 0.000 0.011 0.011 0.000 0.000 0.005 0.005 0.005 0.005

0.000 0.000 0.000 0.000 0.060 0.076 0.056 0.078 0.010 0.005

0.000 0.005 0.000 0.000 0.000 0.000 0.000 0.005 0.000 0.005

0.000 0.000 0.014 0.005 0.005 0.000 0.000 0.000 0.050 0.061

0.082 0.087

>Non-Methylation code 427

0.005 0.000 0.000 0.010 0.000 0.010 0.000 0.000 0.005 0.005

0.000 0.000 0.005 0.005 0.015 0.000 0.005 0.000 0.000 0.000

0.039 0.052 0.077 0.099 0.005 0.000 0.000 0.011 0.000 0.011

0.000 0.000 0.011 0.005 0.000 0.000 0.000 0.011 0.011 0.000

0.000 0.000 0.000 0.005 0.056 0.074 0.053 0.080 0.006 0.000

0.000 0.000 0.000 0.012 0.000 0.000 0.012 0.000 0.000 0.006

0.006 0.000 0.019 0.006 0.006 0.000 0.000 0.000 0.052 0.054

0.066 0.086

>Non-Methylation code 428

0.007 0.000 0.000 0.007 0.014 0.021 0.000 0.000 0.000 0.007

0.000 0.000 0.021 0.007 0.000 0.000 0.007 0.000 0.000 0.000

0.055 0.075 0.051 0.062 0.000 0.000 0.000 0.000 0.000 0.006

0.006 0.000 0.000 0.012 0.000 0.006 0.019 0.000 0.006 0.012

0.000 0.006 0.000 0.000 0.067 0.059 0.066 0.067 0.010 0.005

0.005 0.000 0.005 0.000 0.000 0.000 0.000 0.005 0.000 0.000

0.005 0.000 0.010 0.000 0.010 0.000 0.005 0.000 0.074 0.076

0.054 0.072

>Non-Methylation code 429

0.007 0.000 0.007 0.000 0.000 0.007 0.000 0.000 0.000 0.007

0.000 0.007 0.007 0.014 0.007 0.014 0.007 0.000 0.000 0.000

0.064 0.062 0.053 0.067 0.009 0.000 0.000 0.017 0.000 0.009

0.000 0.000 0.009 0.009 0.000 0.000 0.009 0.009 0.000 0.009

0.017 0.000 0.000 0.000 0.076 0.043 0.062 0.058 0.005 0.000

0.000 0.005 0.000 0.000 0.000 0.000 0.016 0.000 0.000 0.005

0.005 0.000 0.011 0.005 0.000 0.000 0.005 0.000 0.058 0.082

0.079 0.056

>Non-Methylation code 430

0.000 0.005 0.005 0.005 0.000 0.005 0.005 0.000 0.000 0.015

0.000 0.000 0.015 0.000 0.005 0.000 0.000 0.000 0.000 0.000

0.066 0.073 0.071 0.062 0.011 0.005 0.005 0.005 0.000 0.000

0.000 0.005 0.000 0.000 0.000 0.000 0.011 0.000 0.005 0.016

0.005 0.000 0.000 0.000 0.068 0.069 0.066 0.062 0.011 0.000

0.000 0.000 0.000 0.005 0.000 0.000 0.000 0.011 0.000 0.000

0.016 0.000 0.011 0.005 0.005 0.000 0.000 0.000 0.073 0.058

0.069 0.070

>Non-Methylation code 431

0.010 0.010 0.000 0.010 0.005 0.010 0.000 0.000 0.000 0.000

0.000 0.000 0.000 0.000 0.000 0.005 0.000 0.005 0.005 0.000

0.045 0.070 0.077 0.079 0.019 0.006 0.000 0.006 0.000 0.013

0.000 0.000 0.006 0.006 0.000 0.000 0.006 0.000 0.006 0.013

0.000 0.000 0.000 0.000 0.057 0.075 0.061 0.058 0.000 0.000

0.000 0.000 0.000 0.007 0.000 0.000 0.000 0.007 0.000 0.000

0.000 0.000 0.026 0.013 0.000 0.007 0.000 0.000 0.115 0.019

0.115 0.026

>Non-Methylation code 432

0.006 0.000 0.000 0.006 0.000 0.006 0.000 0.000 0.012 0.006

0.000 0.006 0.000 0.000 0.023 0.006 0.000 0.000 0.000 0.000

0.071 0.072 0.064 0.057 0.005 0.005 0.000 0.010 0.000 0.010

0.000 0.000 0.010 0.005 0.000 0.000 0.000 0.005 0.005 0.000

0.005 0.000 0.000 0.000 0.057 0.079 0.079 0.058 0.006 0.000

0.000 0.011 0.000 0.006 0.000 0.000 0.011 0.000 0.000 0.000

0.006 0.006 0.011 0.011 0.000 0.000 0.000 0.000 0.050 0.068

0.069 0.078

>Non-Methylation code 433

0.000 0.000 0.005 0.000 0.005 0.005 0.000 0.000 0.005 0.005

0.000 0.000 0.005 0.005 0.010 0.010 0.000 0.005 0.000 0.000

0.068 0.087 0.062 0.053 0.012 0.000 0.000 0.006 0.000 0.000

0.000 0.000 0.000 0.006 0.000 0.000 0.000 0.000 0.024 0.012

0.006 0.000 0.000 0.000 0.073 0.093 0.063 0.039 0.009 0.000

0.000 0.018 0.000 0.009 0.000 0.000 0.009 0.027 0.000 0.000

0.009 0.000 0.000 0.018 0.000 0.000 0.000 0.000 0.054 0.066

0.054 0.061

>Non-Methylation code 434

0.004 0.000 0.004 0.004 0.000 0.009 0.000 0.000 0.004 0.000

0.000 0.000 0.013 0.000 0.013 0.000 0.000 0.000 0.000 0.000

0.087 0.061 0.043 0.090 0.008 0.004 0.000 0.000 0.000 0.008

0.000 0.000 0.004 0.000 0.000 0.000 0.004 0.000 0.016 0.000

0.004 0.004 0.000 0.000 0.093 0.040 0.079 0.071 0.020 0.000

0.000 0.005 0.000 0.005 0.000 0.000 0.000 0.000 0.000 0.000

0.005 0.000 0.020 0.005 0.000 0.000 0.000 0.000 0.076 0.070

0.051 0.078

>Non-Methylation code 435

0.009 0.004 0.000 0.000 0.000 0.009 0.000 0.000 0.000 0.000

0.000 0.000 0.009 0.000 0.013 0.009 0.004 0.000 0.000 0.000

0.077 0.054 0.060 0.084 0.021 0.000 0.000 0.000 0.000 0.014

0.000 0.000 0.000 0.000 0.000 0.000 0.035 0.007 0.007 0.000

0.000 0.000 0.000 0.000 0.063 0.073 0.072 0.041 0.000 0.005

0.000 0.000 0.000 0.005 0.000 0.000 0.000 0.010 0.000 0.005

0.019 0.000 0.010 0.000 0.000 0.005 0.000 0.000 0.079 0.072

0.093 0.032

>Non-Methylation code 436

0.006 0.000 0.000 0.006 0.000 0.006 0.000 0.000 0.000 0.000

0.000 0.000 0.017 0.000 0.012 0.023 0.006 0.000 0.000 0.000

0.060 0.077 0.063 0.058 0.005 0.000 0.000 0.000 0.000 0.011

0.000 0.000 0.000 0.016 0.000 0.000 0.005 0.011 0.011 0.005

0.000 0.000 0.000 0.000 0.070 0.076 0.062 0.061 0.006 0.000

0.006 0.006 0.006 0.006 0.000 0.000 0.013 0.006 0.000 0.000

0.006 0.000 0.000 0.006 0.000 0.000 0.006 0.000 0.067 0.065

0.064 0.068

>Non-Methylation code 437

0.000 0.012 0.006 0.006 0.000 0.000 0.000 0.000 0.000 0.035

0.000 0.006 0.000 0.000 0.000 0.012 0.000 0.000 0.000 0.000

0.061 0.055 0.069 0.073 0.011 0.021 0.000 0.000 0.000 0.000

0.000 0.000 0.000 0.000 0.000 0.000 0.005 0.000 0.005 0.016

0.011 0.000 0.000 0.000 0.059 0.072 0.086 0.047 0.041 0.000

0.000 0.000 0.000 0.000 0.000 0.000 0.000 0.025 0.000 0.000

0.008 0.008 0.008 0.000 0.000 0.000 0.000 0.000 0.065 0.074

0.042 0.063

>Non-Methylation code 438

0.005 0.005 0.000 0.000 0.000 0.000 0.000 0.000 0.000 0.011

0.000 0.000 0.022 0.005 0.011 0.000 0.005 0.000 0.000 0.000

0.067 0.058 0.065 0.078 0.007 0.007 0.000 0.000 0.000 0.000

0.000 0.000 0.003 0.003 0.000 0.000 0.003 0.000 0.014 0.003

0.000 0.003 0.000 0.000 0.062 0.075 0.073 0.079 0.050 0.000

0.000 0.000 0.010 0.000 0.000 0.000 0.000 0.010 0.000 0.010

0.030 0.000 0.000 0.000 0.000 0.010 0.000 0.000 0.065 0.054

0.047 0.048

>Non-Methylation code 439

0.000 0.004 0.000 0.004 0.000 0.009 0.000 0.000 0.004 0.009

0.000 0.000 0.013 0.000 0.009 0.000 0.000 0.004 0.000 0.000

0.082 0.063 0.081 0.049 0.009 0.004 0.000 0.000 0.000 0.013

0.000 0.000 0.004 0.000 0.000 0.000 0.004 0.000 0.013 0.009

0.000 0.000 0.000 0.000 0.065 0.064 0.059 0.090 0.011 0.000

0.000 0.006 0.000 0.011 0.000 0.000 0.000 0.000 0.000 0.000

0.022 0.000 0.011 0.000 0.000 0.000 0.000 0.000 0.056 0.075

0.071 0.071

>Non-Methylation code 440

0.017 0.000 0.000 0.000 0.000 0.000 0.006 0.000 0.006 0.011

0.000 0.006 0.011 0.000 0.006 0.011 0.000 0.000 0.000 0.000

0.055 0.064 0.063 0.078 0.013 0.009 0.000 0.000 0.000 0.000

0.000 0.000 0.000 0.000 0.000 0.000 0.013 0.000 0.009 0.004

0.009 0.000 0.000 0.000 0.073 0.055 0.068 0.080 0.020 0.000

0.000 0.000 0.000 0.007 0.000 0.000 0.000 0.000 0.000 0.000

0.020 0.027 0.007 0.000 0.000 0.000 0.000 0.000 0.070 0.084

0.058 0.040

>Non-Methylation code 441

0.026 0.000 0.000 0.000 0.000 0.013 0.000 0.000 0.000 0.000

0.000 0.000 0.019 0.019 0.006 0.000 0.000 0.000 0.000 0.000

0.064 0.077 0.048 0.060 0.011 0.000 0.000 0.000 0.000 0.000

0.006 0.000 0.006 0.017 0.000 0.000 0.011 0.000 0.006 0.011

0.000 0.006 0.000 0.000 0.049 0.078 0.073 0.062 0.012 0.008

0.000 0.000 0.000 0.000 0.000 0.000 0.000 0.000 0.000 0.000

0.008 0.000 0.008 0.004 0.004 0.000 0.004 0.000 0.068 0.053

0.076 0.086

>Non-Methylation code 442

0.009 0.000 0.005 0.005 0.009 0.000 0.005 0.000 0.000 0.000

0.000 0.000 0.009 0.000 0.009 0.005 0.000 0.000 0.000 0.000

0.078 0.054 0.077 0.068 0.012 0.004 0.000 0.000 0.000 0.000

0.000 0.000 0.000 0.000 0.000 0.000 0.000 0.000 0.016 0.012

0.008 0.000 0.000 0.000 0.062 0.058 0.082 0.079 0.013 0.000

0.000 0.000 0.000 0.004 0.000 0.000 0.000 0.000 0.000 0.000

0.004 0.004 0.017 0.000 0.000 0.009 0.000 0.000 0.072 0.060

0.061 0.088

>Non-Methylation code 443

0.006 0.000 0.000 0.006 0.000 0.006 0.000 0.000 0.000 0.000

0.000 0.000 0.024 0.000 0.012 0.018 0.006 0.000 0.000 0.000

0.063 0.074 0.052 0.066 0.005 0.000 0.000 0.000 0.000 0.010

0.000 0.000 0.000 0.010 0.000 0.000 0.005 0.010 0.015 0.005

0.000 0.000 0.000 0.000 0.072 0.085 0.057 0.058 0.012 0.000

0.006 0.006 0.006 0.006 0.000 0.000 0.012 0.006 0.000 0.000

0.006 0.000 0.000 0.000 0.000 0.000 0.006 0.000 0.071 0.061

0.065 0.068

>Non-Methylation code 444

0.018 0.006 0.000 0.000 0.006 0.000 0.000 0.000 0.000 0.006

0.000 0.000 0.024 0.000 0.006 0.000 0.000 0.000 0.006 0.000

0.061 0.054 0.069 0.076 0.009 0.000 0.000 0.005 0.000 0.005

0.000 0.000 0.000 0.005 0.000 0.000 0.014 0.000 0.014 0.009

0.000 0.000 0.000 0.000 0.067 0.075 0.052 0.079 0.019 0.000

0.000 0.000 0.000 0.000 0.000 0.000 0.000 0.019 0.000 0.000

0.012 0.000 0.012 0.006 0.000 0.000 0.000 0.000 0.074 0.061

0.043 0.087

>Non-Methylation code 445

0.000 0.000 0.000 0.011 0.000 0.011 0.000 0.000 0.000 0.000

0.000 0.000 0.021 0.000 0.000 0.053 0.032 0.000 0.000 0.000

0.052 0.047 0.055 0.053 0.015 0.000 0.000 0.005 0.000 0.000

0.010 0.000 0.000 0.005 0.000 0.005 0.010 0.005 0.010 0.000

0.000 0.000 0.000 0.000 0.068 0.063 0.067 0.071 0.000 0.000

0.006 0.000 0.000 0.006 0.000 0.012 0.006 0.006 0.000 0.006

0.006 0.006 0.006 0.000 0.006 0.000 0.000 0.006 0.064 0.055

0.070 0.074

>Non-Methylation code 446

0.005 0.000 0.005 0.011 0.005 0.005 0.000 0.000 0.011 0.011

0.000 0.000 0.005 0.000 0.000 0.005 0.000 0.000 0.005 0.000

0.065 0.067 0.066 0.065 0.006 0.000 0.000 0.000 0.000 0.006

0.000 0.000 0.000 0.000 0.000 0.000 0.017 0.000 0.011 0.028

0.006 0.000 0.000 0.000 0.052 0.074 0.068 0.065 0.011 0.000

0.000 0.000 0.000 0.011 0.000 0.000 0.000 0.017 0.000 0.000

0.006 0.011 0.011 0.000 0.000 0.000 0.000 0.000 0.072 0.074

0.062 0.058

>Non-Methylation code 447

0.007 0.000 0.000 0.000 0.007 0.000 0.007 0.000 0.000 0.022

0.000 0.000 0.015 0.007 0.000 0.007 0.007 0.007 0.000 0.007

0.066 0.063 0.048 0.062 0.000 0.000 0.000 0.000 0.000 0.000

0.007 0.000 0.000 0.007 0.000 0.007 0.014 0.000 0.007 0.034

0.014 0.000 0.000 0.000 0.062 0.051 0.056 0.075 0.007 0.000

0.000 0.000 0.000 0.000 0.007 0.000 0.000 0.021 0.000 0.000

0.028 0.000 0.007 0.000 0.014 0.000 0.000 0.000 0.067 0.076

0.061 0.045

>Non-Methylation code 448

0.007 0.000 0.007 0.000 0.007 0.007 0.007 0.000 0.000 0.000

0.000 0.000 0.022 0.000 0.000 0.007 0.015 0.000 0.000 0.000

0.077 0.057 0.063 0.055 0.004 0.000 0.000 0.000 0.000 0.000

0.000 0.000 0.000 0.004 0.000 0.000 0.013 0.000 0.018 0.004

0.009 0.004 0.000 0.000 0.072 0.060 0.051 0.092 0.017 0.000

0.000 0.000 0.000 0.008 0.017 0.000 0.000 0.008 0.000 0.000

0.008 0.008 0.000 0.008 0.000 0.017 0.000 0.000 0.058 0.065

0.048 0.071

>Non-Methylation code 449

0.000 0.006 0.000 0.000 0.006 0.000 0.006 0.000 0.000 0.012

0.000 0.000 0.024 0.006 0.000 0.012 0.006 0.000 0.000 0.000

0.051 0.066 0.053 0.085 0.000 0.000 0.000 0.000 0.012 0.000

0.006 0.000 0.000 0.012 0.000 0.006 0.025 0.000 0.000 0.000

0.006 0.006 0.000 0.006 0.063 0.064 0.059 0.067 0.000 0.000

0.000 0.000 0.007 0.000 0.007 0.015 0.000 0.007 0.000 0.000

0.029 0.000 0.000 0.015 0.007 0.000 0.000 0.000 0.053 0.051

0.076 0.065

>Non-Methylation code 450

0.009 0.000 0.000 0.000 0.000 0.000 0.009 0.000 0.000 0.000

0.000 0.009 0.027 0.000 0.000 0.035 0.018 0.000 0.000 0.000

0.054 0.048 0.062 0.063 0.000 0.000 0.000 0.000 0.000 0.000

0.005 0.000 0.005 0.016 0.000 0.000 0.021 0.000 0.011 0.000

0.011 0.000 0.000 0.000 0.058 0.077 0.067 0.063 0.000 0.000

0.000 0.000 0.008 0.000 0.008 0.000 0.000 0.025 0.000 0.000

0.017 0.008 0.000 0.008 0.008 0.008 0.000 0.008 0.065 0.060

0.046 0.062

>Non-Methylation code 451

0.004 0.000 0.004 0.000 0.000 0.004 0.000 0.000 0.000 0.000

0.000 0.000 0.009 0.000 0.013 0.009 0.004 0.004 0.004 0.000

0.065 0.068 0.069 0.074 0.016 0.000 0.008 0.000 0.000 0.024

0.000 0.000 0.000 0.008 0.000 0.000 0.016 0.016 0.000 0.000

0.008 0.000 0.000 0.000 0.052 0.057 0.076 0.052 0.000 0.000

0.005 0.005 0.000 0.000 0.000 0.000 0.000 0.011 0.000 0.000

0.000 0.011 0.016 0.011 0.005 0.000 0.000 0.000 0.039 0.058

0.079 0.092

>Non-Methylation code 452

0.004 0.004 0.000 0.000 0.000 0.000 0.000 0.000 0.004 0.004

0.000 0.000 0.008 0.000 0.020 0.004 0.000 0.004 0.000 0.000

0.066 0.056 0.067 0.093 0.025 0.000 0.008 0.000 0.000 0.008

0.000 0.000 0.000 0.008 0.000 0.000 0.034 0.000 0.000 0.017

0.000 0.008 0.000 0.000 0.059 0.066 0.047 0.052 0.007 0.000

0.000 0.000 0.000 0.007 0.000 0.000 0.000 0.013 0.000 0.000

0.020 0.013 0.007 0.000 0.007 0.000 0.000 0.000 0.069 0.069

0.052 0.069

>Non-Methylation code 453

0.010 0.000 0.000 0.000 0.005 0.000 0.010 0.000 0.000 0.005

0.000 0.000 0.015 0.000 0.005 0.000 0.010 0.000 0.005 0.000

0.073 0.077 0.056 0.060 0.007 0.000 0.000 0.000 0.007 0.007

0.013 0.000 0.000 0.007 0.000 0.000 0.020 0.007 0.000 0.007

0.013 0.000 0.000 0.000 0.057 0.073 0.058 0.061 0.000 0.000

0.000 0.000 0.000 0.006 0.006 0.000 0.000 0.013 0.000 0.006

0.019 0.000 0.006 0.013 0.006 0.000 0.000 0.000 0.063 0.059

0.055 0.079

>Non-Methylation code 454

0.000 0.007 0.000 0.000 0.007 0.000 0.000 0.007 0.000 0.014

0.000 0.007 0.014 0.000 0.000 0.007 0.007 0.007 0.000 0.007

0.061 0.048 0.066 0.071 0.000 0.009 0.000 0.000 0.000 0.000

0.000 0.019 0.000 0.009 0.000 0.000 0.038 0.000 0.000 0.028

0.000 0.000 0.000 0.000 0.048 0.042 0.075 0.064 0.000 0.011

0.000 0.000 0.006 0.000 0.006 0.000 0.000 0.011 0.000 0.006

0.011 0.006 0.000 0.006 0.000 0.006 0.000 0.000 0.063 0.073

0.061 0.068

>Non-Methylation code 455

0.013 0.006 0.000 0.000 0.000 0.013 0.019 0.000 0.000 0.000

0.000 0.000 0.000 0.013 0.000 0.006 0.000 0.013 0.000 0.000

0.060 0.059 0.063 0.066 0.009 0.000 0.000 0.009 0.000 0.000

0.000 0.000 0.000 0.000 0.000 0.000 0.018 0.000 0.009 0.018

0.027 0.000 0.000 0.000 0.067 0.059 0.060 0.057 0.009 0.000

0.000 0.000 0.000 0.000 0.000 0.000 0.000 0.004 0.000 0.000

0.009 0.000 0.017 0.004 0.000 0.000 0.004 0.000 0.057 0.072

0.063 0.094

>Non-Methylation code 456

0.011 0.000 0.000 0.011 0.000 0.005 0.000 0.000 0.005 0.000

0.000 0.000 0.005 0.000 0.016 0.005 0.005 0.000 0.000 0.000

0.057 0.064 0.067 0.081 0.004 0.000 0.000 0.009 0.000 0.009

0.000 0.000 0.004 0.004 0.000 0.000 0.009 0.004 0.013 0.000

0.000 0.000 0.000 0.000 0.068 0.091 0.068 0.048 0.006 0.000

0.000 0.006 0.000 0.019 0.000 0.000 0.000 0.012 0.000 0.006

0.006 0.000 0.006 0.006 0.000 0.000 0.000 0.000 0.057 0.065

0.068 0.075

>Non-Methylation code 457

0.000 0.000 0.008 0.000 0.000 0.012 0.000 0.000 0.004 0.000

0.000 0.000 0.008 0.000 0.012 0.004 0.000 0.000 0.004 0.000

0.071 0.075 0.061 0.076 0.018 0.000 0.000 0.018 0.000 0.027

0.000 0.000 0.000 0.018 0.000 0.000 0.000 0.009 0.000 0.009

0.018 0.000 0.000 0.000 0.055 0.046 0.050 0.065 0.005 0.000

0.000 0.000 0.000 0.016 0.000 0.000 0.005 0.011 0.000 0.000

0.016 0.000 0.005 0.000 0.000 0.000 0.005 0.000 0.072 0.058

0.069 0.068

>Non-Methylation code 458

0.000 0.000 0.000 0.000 0.000 0.000 0.000 0.000 0.000 0.011

0.000 0.000 0.011 0.000 0.011 0.033 0.000 0.005 0.000 0.000

0.048 0.059 0.078 0.077 0.005 0.000 0.000 0.000 0.000 0.005

0.011 0.000 0.000 0.000 0.000 0.000 0.016 0.005 0.005 0.011

0.000 0.005 0.005 0.000 0.048 0.074 0.070 0.071 0.024 0.000

0.000 0.000 0.000 0.000 0.000 0.000 0.000 0.060 0.000 0.000

0.012 0.000 0.000 0.024 0.024 0.000 0.000 0.000 0.056 0.040

0.045 0.049

>Non-Methylation code 459

0.019 0.000 0.000 0.000 0.000 0.000 0.000 0.000 0.000 0.019

0.000 0.000 0.019 0.000 0.000 0.029 0.000 0.029 0.010 0.000

0.059 0.034 0.059 0.057 0.000 0.004 0.000 0.000 0.000 0.004

0.004 0.000 0.000 0.004 0.000 0.000 0.013 0.004 0.008 0.008

0.000 0.000 0.004 0.000 0.078 0.066 0.053 0.082 0.007 0.007

0.000 0.000 0.000 0.015 0.000 0.000 0.000 0.015 0.000 0.000

0.015 0.000 0.007 0.007 0.007 0.000 0.000 0.000 0.074 0.059

0.060 0.060

>Non-Methylation code 460

0.000 0.010 0.000 0.000 0.010 0.019 0.000 0.010 0.000 0.019

0.000 0.000 0.000 0.000 0.000 0.019 0.029 0.010 0.000 0.000

0.049 0.059 0.049 0.051 0.021 0.007 0.000 0.000 0.000 0.000

0.000 0.000 0.000 0.007 0.000 0.000 0.000 0.014 0.007 0.028

0.000 0.007 0.000 0.000 0.067 0.081 0.038 0.058 0.000 0.000

0.000 0.000 0.000 0.000 0.017 0.000 0.006 0.000 0.000 0.000

0.011 0.000 0.006 0.006 0.000 0.011 0.006 0.006 0.062 0.073

0.058 0.074

>Non-Methylation code 461

0.006 0.000 0.000 0.000 0.000 0.024 0.000 0.000 0.000 0.006

0.000 0.000 0.000 0.006 0.012 0.018 0.000 0.006 0.000 0.000

0.062 0.067 0.058 0.067 0.008 0.000 0.000 0.000 0.000 0.023

0.015 0.000 0.000 0.000 0.000 0.000 0.008 0.000 0.000 0.008

0.000 0.008 0.008 0.008 0.043 0.060 0.065 0.082 0.000 0.005

0.000 0.011 0.000 0.011 0.000 0.000 0.000 0.005 0.000 0.000

0.000 0.000 0.005 0.005 0.011 0.005 0.005 0.000 0.069 0.073

0.065 0.062

>Non-Methylation code 462

0.004 0.004 0.000 0.011 0.004 0.000 0.000 0.000 0.004 0.008

0.000 0.000 0.004 0.000 0.011 0.000 0.000 0.000 0.000 0.000

0.090 0.050 0.085 0.058 0.012 0.000 0.000 0.000 0.000 0.012

0.000 0.000 0.012 0.000 0.000 0.012 0.000 0.006 0.006 0.012

0.000 0.000 0.006 0.000 0.077 0.083 0.041 0.053 0.012 0.000

0.000 0.000 0.000 0.006 0.000 0.000 0.006 0.006 0.006 0.000

0.012 0.006 0.006 0.006 0.006 0.000 0.000 0.000 0.059 0.059

0.063 0.081

>Non-Methylation code 463

0.012 0.000 0.000 0.000 0.000 0.012 0.000 0.000 0.006 0.006

0.006 0.000 0.012 0.006 0.006 0.006 0.006 0.000 0.000 0.000

0.057 0.057 0.065 0.080 0.004 0.004 0.000 0.007 0.004 0.004

0.000 0.000 0.004 0.007 0.000 0.000 0.004 0.000 0.011 0.000

0.000 0.000 0.000 0.000 0.091 0.045 0.090 0.059 0.013 0.000

0.000 0.000 0.000 0.013 0.000 0.000 0.007 0.000 0.000 0.013

0.000 0.007 0.007 0.013 0.000 0.000 0.007 0.000 0.077 0.077

0.045 0.054

>Non-Methylation code 464

0.005 0.005 0.000 0.000 0.000 0.000 0.005 0.000 0.000 0.005

0.000 0.000 0.020 0.005 0.005 0.010 0.000 0.000 0.005 0.000

0.069 0.065 0.067 0.067 0.007 0.007 0.000 0.000 0.000 0.014

0.000 0.000 0.000 0.027 0.000 0.000 0.014 0.000 0.007 0.007

0.007 0.000 0.000 0.000 0.074 0.060 0.056 0.055 0.012 0.000

0.000 0.000 0.000 0.000 0.000 0.000 0.000 0.024 0.000 0.000

0.024 0.000 0.000 0.048 0.000 0.036 0.000 0.000 0.054 0.044

0.046 0.045

>Non-Methylation code 465

0.006 0.006 0.000 0.000 0.000 0.045 0.000 0.000 0.000 0.000

0.000 0.000 0.006 0.000 0.006 0.006 0.006 0.000 0.000 0.000

0.060 0.069 0.052 0.069 0.040 0.000 0.000 0.000 0.000 0.066

0.000 0.000 0.000 0.013 0.000 0.000 0.013 0.013 0.000 0.000

0.000 0.000 0.000 0.000 0.040 0.047 0.048 0.053 0.000 0.004

0.004 0.007 0.000 0.007 0.000 0.000 0.000 0.004 0.000 0.000

0.000 0.000 0.015 0.000 0.000 0.000 0.004 0.000 0.077 0.088

0.047 0.077

>Non-Methylation code 466

0.005 0.005 0.000 0.005 0.000 0.005 0.000 0.000 0.005 0.005

0.000 0.000 0.005 0.011 0.005 0.000 0.005 0.005 0.000 0.000

0.079 0.058 0.082 0.049 0.014 0.000 0.007 0.000 0.000 0.007

0.000 0.000 0.007 0.014 0.000 0.007 0.000 0.000 0.007 0.020

0.000 0.000 0.000 0.000 0.065 0.054 0.053 0.079 0.006 0.006

0.006 0.006 0.000 0.000 0.000 0.000 0.000 0.006 0.000 0.000

0.012 0.000 0.006 0.000 0.012 0.000 0.000 0.000 0.057 0.072

0.079 0.064

>Non-Methylation code 467

0.017 0.006 0.000 0.006 0.000 0.011 0.006 0.000 0.000 0.000

0.000 0.000 0.000 0.000 0.006 0.017 0.000 0.000 0.000 0.000

0.052 0.071 0.074 0.070 0.012 0.006 0.000 0.000 0.000 0.006

0.000 0.000 0.000 0.012 0.000 0.000 0.018 0.000 0.006 0.006

0.006 0.000 0.000 0.000 0.053 0.066 0.068 0.074 0.000 0.005

0.000 0.000 0.000 0.010 0.000 0.000 0.000 0.005 0.000 0.000

0.010 0.000 0.014 0.000 0.000 0.005 0.005 0.000 0.068 0.081

0.058 0.073

>Non-Methylation code 468

0.016 0.005 0.000 0.011 0.000 0.005 0.005 0.000 0.000 0.005

0.000 0.000 0.000 0.000 0.005 0.011 0.000 0.000 0.000 0.000

0.061 0.069 0.068 0.070 0.014 0.007 0.000 0.000 0.000 0.000

0.000 0.000 0.000 0.014 0.000 0.007 0.014 0.000 0.007 0.007

0.007 0.000 0.000 0.000 0.044 0.063 0.079 0.072 0.000 0.005

0.000 0.000 0.000 0.005 0.000 0.000 0.000 0.005 0.000 0.000

0.011 0.000 0.011 0.000 0.005 0.005 0.005 0.000 0.072 0.067

0.075 0.068

>Non-Methylation code 469

0.005 0.000 0.005 0.000 0.000 0.010 0.000 0.000 0.000 0.005

0.000 0.005 0.016 0.000 0.005 0.000 0.010 0.000 0.000 0.005

0.059 0.072 0.073 0.062 0.000 0.000 0.005 0.000 0.000 0.010

0.005 0.005 0.000 0.005 0.000 0.000 0.000 0.005 0.015 0.005

0.010 0.000 0.000 0.000 0.061 0.068 0.069 0.073 0.000 0.000

0.008 0.000 0.000 0.015 0.008 0.000 0.000 0.000 0.000 0.000

0.000 0.015 0.008 0.015 0.015 0.008 0.000 0.000 0.048 0.053

0.068 0.072

>Non-Methylation code 470

0.005 0.000 0.000 0.000 0.000 0.005 0.000 0.000 0.000 0.010

0.005 0.000 0.010 0.005 0.010 0.010 0.005 0.000 0.000 0.000

0.052 0.072 0.079 0.067 0.010 0.005 0.000 0.000 0.000 0.010

0.000 0.000 0.000 0.000 0.000 0.000 0.015 0.000 0.010 0.015

0.000 0.000 0.000 0.000 0.076 0.071 0.047 0.074 0.022 0.000

0.006 0.000 0.000 0.006 0.006 0.000 0.000 0.006 0.000 0.000

0.011 0.006 0.006 0.000 0.000 0.000 0.000 0.000 0.065 0.078

0.067 0.057

>Non-Methylation code 471

0.017 0.000 0.000 0.000 0.000 0.011 0.000 0.006 0.000 0.000

0.000 0.000 0.006 0.000 0.017 0.011 0.006 0.000 0.000 0.000

0.064 0.050 0.065 0.081 0.010 0.000 0.000 0.005 0.000 0.015

0.000 0.000 0.000 0.010 0.000 0.000 0.005 0.000 0.015 0.005

0.000 0.000 0.000 0.000 0.080 0.062 0.066 0.063 0.011 0.000

0.000 0.000 0.000 0.017 0.000 0.000 0.000 0.000 0.000 0.006

0.000 0.006 0.011 0.006 0.000 0.006 0.000 0.006 0.065 0.052

0.066 0.084

>Non-Methylation code 472

0.050 0.000 0.000 0.000 0.000 0.020 0.000 0.000 0.000 0.010

0.000 0.000 0.000 0.000 0.010 0.020 0.010 0.000 0.000 0.000

0.075 0.034 0.058 0.046 0.014 0.000 0.000 0.000 0.000 0.014

0.000 0.000 0.000 0.000 0.000 0.000 0.022 0.022 0.007 0.007

0.000 0.000 0.000 0.000 0.062 0.059 0.074 0.053 0.005 0.000

0.005 0.000 0.000 0.005 0.000 0.000 0.000 0.005 0.000 0.000

0.005 0.005 0.015 0.015 0.000 0.000 0.000 0.000 0.069 0.062

0.075 0.067

>Non-Methylation code 473

0.000 0.000 0.000 0.006 0.000 0.012 0.000 0.000 0.006 0.012

0.000 0.000 0.000 0.000 0.006 0.006 0.006 0.006 0.006 0.006

0.059 0.066 0.072 0.063 0.000 0.000 0.006 0.000 0.006 0.018

0.000 0.000 0.006 0.000 0.000 0.000 0.006 0.006 0.006 0.006

0.006 0.006 0.000 0.000 0.078 0.048 0.077 0.060 0.000 0.000

0.000 0.006 0.000 0.006 0.006 0.006 0.000 0.012 0.000 0.000

0.000 0.000 0.012 0.012 0.000 0.006 0.000 0.000 0.065 0.072

0.064 0.068

>Non-Methylation code 474

0.018 0.000 0.000 0.000 0.000 0.018 0.000 0.000 0.000 0.006

0.000 0.000 0.012 0.000 0.012 0.012 0.000 0.000 0.000 0.000

0.080 0.071 0.034 0.071 0.017 0.000 0.006 0.000 0.000 0.011

0.006 0.000 0.000 0.000 0.000 0.000 0.017 0.006 0.006 0.006

0.000 0.000 0.000 0.000 0.062 0.077 0.069 0.052 0.009 0.000

0.000 0.000 0.000 0.005 0.000 0.000 0.000 0.005 0.000 0.000

0.009 0.005 0.014 0.005 0.005 0.000 0.000 0.000 0.061 0.072

0.072 0.072

>Non-Methylation code 475

0.000 0.000 0.007 0.000 0.007 0.007 0.007 0.000 0.000 0.007

0.000 0.000 0.007 0.014 0.007 0.000 0.021 0.000 0.000 0.000

0.059 0.065 0.055 0.069 0.000 0.005 0.005 0.000 0.005 0.005

0.005 0.000 0.000 0.000 0.000 0.000 0.000 0.011 0.011 0.011

0.011 0.000 0.000 0.000 0.066 0.051 0.074 0.073 0.007 0.000

0.007 0.000 0.000 0.007 0.007 0.000 0.000 0.000 0.000 0.007

0.007 0.000 0.007 0.007 0.014 0.014 0.000 0.000 0.071 0.056

0.069 0.055

>Non-Methylation code 476

0.014 0.000 0.007 0.000 0.000 0.007 0.000 0.000 0.007 0.014

0.000 0.007 0.000 0.000 0.007 0.020 0.000 0.000 0.000 0.000

0.065 0.054 0.053 0.079 0.011 0.006 0.006 0.006 0.000 0.000

0.000 0.000 0.000 0.006 0.000 0.000 0.011 0.000 0.006 0.000

0.011 0.000 0.000 0.000 0.052 0.067 0.077 0.076 0.000 0.006

0.000 0.006 0.000 0.006 0.000 0.000 0.006 0.006 0.000 0.000

0.006 0.011 0.006 0.000 0.006 0.006 0.000 0.000 0.076 0.059

0.085 0.052

>Non-Methylation code 477

0.012 0.006 0.000 0.000 0.000 0.006 0.000 0.000 0.000 0.012

0.000 0.006 0.012 0.000 0.006 0.006 0.006 0.000 0.000 0.000

0.056 0.058 0.070 0.076 0.000 0.005 0.000 0.000 0.000 0.010

0.000 0.000 0.000 0.005 0.000 0.000 0.010 0.000 0.010 0.000

0.005 0.005 0.005 0.000 0.067 0.077 0.070 0.064 0.017 0.006

0.000 0.011 0.000 0.006 0.006 0.000 0.000 0.000 0.000 0.000

0.000 0.000 0.006 0.011 0.000 0.000 0.000 0.000 0.057 0.071

0.069 0.074

>Non-Methylation code 478

0.016 0.000 0.005 0.000 0.000 0.005 0.000 0.000 0.005 0.005

0.000 0.000 0.000 0.000 0.011 0.021 0.000 0.000 0.000 0.000

0.071 0.053 0.065 0.074 0.021 0.000 0.000 0.007 0.000 0.007

0.000 0.000 0.000 0.014 0.000 0.000 0.014 0.000 0.007 0.007

0.007 0.000 0.000 0.000 0.055 0.056 0.078 0.062 0.000 0.006

0.000 0.000 0.000 0.012 0.000 0.000 0.006 0.000 0.000 0.000

0.012 0.019 0.006 0.006 0.000 0.000 0.000 0.000 0.077 0.053

0.070 0.065

>Non-Methylation code 479

0.012 0.006 0.000 0.006 0.000 0.012 0.000 0.000 0.006 0.006

0.000 0.000 0.000 0.006 0.006 0.000 0.006 0.006 0.000 0.000

0.078 0.053 0.095 0.038 0.024 0.000 0.008 0.000 0.000 0.008

0.000 0.000 0.008 0.024 0.000 0.008 0.000 0.000 0.000 0.016

0.000 0.000 0.000 0.000 0.062 0.057 0.057 0.060 0.000 0.005

0.005 0.005 0.000 0.000 0.000 0.000 0.000 0.005 0.000 0.000

0.010 0.000 0.010 0.000 0.005 0.000 0.005 0.000 0.063 0.060

0.080 0.082

>Non-Methylation code 480

0.006 0.000 0.000 0.000 0.000 0.017 0.000 0.000 0.000 0.000

0.000 0.006 0.000 0.011 0.011 0.006 0.006 0.006 0.000 0.006

0.070 0.045 0.073 0.072 0.019 0.000 0.000 0.000 0.000 0.013

0.000 0.006 0.000 0.000 0.000 0.000 0.006 0.000 0.019 0.013

0.006 0.000 0.000 0.000 0.055 0.049 0.059 0.089 0.010 0.000

0.000 0.005 0.000 0.010 0.000 0.000 0.000 0.010 0.000 0.000

0.005 0.000 0.015 0.005 0.000 0.000 0.000 0.000 0.072 0.058

0.064 0.082

>Non-Methylation code 481

0.000 0.000 0.000 0.000 0.005 0.005 0.000 0.005 0.000 0.010

0.000 0.000 0.010 0.000 0.014 0.010 0.000 0.000 0.000 0.005

0.072 0.049 0.075 0.075 0.006 0.000 0.006 0.000 0.006 0.011

0.000 0.000 0.000 0.000 0.000 0.000 0.006 0.000 0.011 0.011

0.006 0.006 0.000 0.006 0.065 0.052 0.070 0.074 0.010 0.000

0.000 0.010 0.000 0.010 0.010 0.000 0.000 0.029 0.000 0.000

0.000 0.010 0.000 0.019 0.019 0.000 0.000 0.000 0.049 0.056

0.065 0.048

>Non-Methylation code 482

0.009 0.000 0.000 0.000 0.000 0.009 0.000 0.000 0.000 0.000

0.000 0.000 0.005 0.005 0.014 0.005 0.005 0.005 0.000 0.000

0.032 0.060 0.094 0.092 0.011 0.000 0.000 0.006 0.000 0.017

0.000 0.000 0.006 0.000 0.000 0.000 0.006 0.000 0.023 0.006

0.000 0.000 0.000 0.000 0.039 0.058 0.072 0.090 0.006 0.000

0.000 0.006 0.000 0.023 0.000 0.000 0.011 0.000 0.000 0.000

0.011 0.000 0.006 0.000 0.000 0.006 0.000 0.000 0.077 0.048

0.056 0.084

>Non-Methylation code 483

0.007 0.000 0.000 0.000 0.000 0.022 0.000 0.000 0.007 0.000

0.000 0.000 0.007 0.000 0.015 0.015 0.007 0.015 0.000 0.000

0.064 0.048 0.063 0.063 0.000 0.000 0.000 0.000 0.007 0.027

0.000 0.000 0.000 0.020 0.000 0.000 0.007 0.007 0.007 0.007

0.000 0.007 0.000 0.000 0.063 0.063 0.062 0.057 0.009 0.004

0.000 0.004 0.004 0.004 0.000 0.000 0.000 0.004 0.000 0.000

0.000 0.000 0.009 0.004 0.000 0.000 0.004 0.004 0.068 0.059

0.080 0.074

>Non-Methylation code 484

0.009 0.000 0.000 0.000 0.000 0.009 0.000 0.009 0.000 0.009

0.000 0.000 0.026 0.000 0.009 0.018 0.000 0.018 0.000 0.000

0.052 0.064 0.046 0.066 0.000 0.000 0.000 0.007 0.013 0.013

0.007 0.000 0.000 0.007 0.000 0.000 0.020 0.007 0.000 0.007

0.000 0.000 0.000 0.000 0.057 0.061 0.059 0.078 0.005 0.000

0.000 0.000 0.010 0.005 0.000 0.005 0.000 0.005 0.000 0.005

0.010 0.000 0.010 0.005 0.000 0.000 0.000 0.000 0.076 0.086

0.066 0.047

>Non-Methylation code 485

0.000 0.000 0.000 0.000 0.010 0.005 0.000 0.000 0.000 0.005

0.000 0.005 0.000 0.000 0.010 0.016 0.005 0.005 0.005 0.000

0.081 0.076 0.049 0.060 0.020 0.000 0.000 0.000 0.010 0.020

0.000 0.010 0.000 0.020 0.000 0.000 0.020 0.000 0.000 0.010

0.000 0.010 0.000 0.000 0.057 0.049 0.063 0.042 0.006 0.000

0.000 0.006 0.017 0.006 0.006 0.000 0.000 0.011 0.000 0.000

0.000 0.006 0.006 0.000 0.000 0.000 0.000 0.000 0.058 0.066

0.073 0.073

>Non-Methylation code 486

0.012 0.000 0.000 0.000 0.000 0.000 0.000 0.000 0.000 0.006

0.000 0.000 0.018 0.006 0.018 0.006 0.006 0.000 0.000 0.000

0.067 0.058 0.060 0.075 0.016 0.000 0.000 0.000 0.000 0.005

0.000 0.000 0.005 0.000 0.000 0.000 0.016 0.000 0.022 0.005

0.000 0.000 0.000 0.000 0.044 0.048 0.077 0.094 0.016 0.000

0.000 0.000 0.000 0.016 0.008 0.000 0.008 0.000 0.000 0.000

0.039 0.000 0.000 0.000 0.000 0.008 0.000 0.000 0.050 0.053

0.063 0.073

>Non-Methylation code 487

0.006 0.000 0.006 0.006 0.000 0.006 0.018 0.000 0.006 0.006

0.000 0.000 0.018 0.000 0.000 0.006 0.000 0.000 0.000 0.000

0.072 0.058 0.054 0.073 0.010 0.000 0.000 0.000 0.000 0.000

0.000 0.005 0.000 0.000 0.000 0.005 0.010 0.000 0.010 0.005

0.015 0.000 0.005 0.000 0.066 0.068 0.068 0.068 0.013 0.000

0.000 0.000 0.000 0.013 0.000 0.000 0.000 0.000 0.000 0.000

0.025 0.000 0.013 0.000 0.006 0.000 0.000 0.000 0.080 0.059

0.063 0.062

>Non-Methylation code 488

0.013 0.000 0.000 0.000 0.000 0.013 0.000 0.000 0.000 0.000

0.000 0.000 0.025 0.000 0.025 0.006 0.000 0.000 0.000 0.000

0.045 0.040 0.080 0.087 0.026 0.000 0.000 0.000 0.000 0.017

0.009 0.000 0.000 0.000 0.000 0.000 0.052 0.000 0.000 0.000

0.000 0.009 0.000 0.000 0.049 0.042 0.063 0.066 0.012 0.000

0.000 0.000 0.000 0.000 0.000 0.000 0.000 0.006 0.000 0.000

0.018 0.006 0.024 0.000 0.006 0.000 0.000 0.000 0.061 0.054

0.075 0.071

>Non-Methylation code 489

0.011 0.000 0.000 0.000 0.005 0.000 0.000 0.000 0.000 0.005

0.000 0.000 0.011 0.000 0.026 0.011 0.000 0.000 0.000 0.000

0.070 0.051 0.074 0.070 0.025 0.000 0.000 0.000 0.006 0.012

0.000 0.000 0.000 0.000 0.000 0.000 0.025 0.000 0.006 0.006

0.000 0.000 0.000 0.000 0.065 0.064 0.074 0.050 0.018 0.000

0.000 0.000 0.000 0.000 0.000 0.000 0.000 0.006 0.000 0.000

0.025 0.006 0.012 0.006 0.000 0.000 0.000 0.000 0.076 0.059

0.044 0.082

>Non-Methylation code 490

0.030 0.000 0.000 0.000 0.000 0.015 0.000 0.000 0.000 0.000

0.000 0.000 0.037 0.000 0.007 0.007 0.000 0.000 0.000 0.000

0.081 0.053 0.067 0.036 0.022 0.000 0.000 0.000 0.000 0.000

0.007 0.000 0.000 0.007 0.000 0.000 0.044 0.007 0.007 0.000

0.000 0.000 0.000 0.000 0.069 0.047 0.059 0.063 0.005 0.000

0.000 0.000 0.000 0.000 0.000 0.000 0.000 0.005 0.000 0.000

0.010 0.000 0.025 0.010 0.005 0.000 0.000 0.000 0.074 0.052

0.072 0.076

>Non-Methylation code 491

0.021 0.000 0.000 0.000 0.007 0.014 0.000 0.000 0.000 0.007

0.000 0.000 0.014 0.000 0.007 0.007 0.000 0.007 0.000 0.000

0.063 0.067 0.074 0.046 0.006 0.000 0.000 0.006 0.006 0.012

0.006 0.000 0.000 0.023 0.000 0.000 0.006 0.000 0.006 0.006

0.000 0.000 0.000 0.000 0.065 0.071 0.061 0.061 0.000 0.006

0.000 0.000 0.000 0.006 0.000 0.000 0.000 0.006 0.000 0.000

0.000 0.006 0.006 0.017 0.006 0.000 0.012 0.000 0.069 0.082

0.073 0.046

>Non-Methylation code 492

0.000 0.000 0.006 0.000 0.000 0.000 0.000 0.000 0.000 0.000

0.000 0.000 0.018 0.006 0.012 0.012 0.006 0.012 0.000 0.000

0.074 0.079 0.055 0.051 0.006 0.000 0.000 0.000 0.013 0.006

0.000 0.000 0.000 0.013 0.000 0.000 0.019 0.006 0.000 0.006

0.006 0.000 0.006 0.000 0.047 0.074 0.074 0.056 0.000 0.000

0.000 0.000 0.009 0.009 0.000 0.000 0.000 0.000 0.000 0.005

0.014 0.000 0.009 0.009 0.000 0.000 0.000 0.000 0.079 0.064

0.085 0.049

>Non-Methylation code 493

0.000 0.005 0.000 0.000 0.000 0.005 0.009 0.000 0.000 0.000

0.000 0.000 0.014 0.000 0.014 0.000 0.000 0.009 0.000 0.000

0.108 0.034 0.081 0.055 0.016 0.000 0.000 0.005 0.005 0.005

0.000 0.010 0.005 0.000 0.000 0.000 0.010 0.000 0.005 0.005

0.000 0.000 0.000 0.000 0.071 0.059 0.071 0.066 0.014 0.000

0.000 0.000 0.000 0.000 0.000 0.000 0.000 0.007 0.000 0.000

0.014 0.007 0.014 0.027 0.000 0.000 0.000 0.000 0.082 0.038

0.062 0.069

>Non-Methylation code 494

0.000 0.008 0.000 0.000 0.000 0.023 0.000 0.000 0.008 0.008

0.000 0.000 0.000 0.008 0.000 0.008 0.008 0.000 0.008 0.000

0.084 0.060 0.068 0.045 0.013 0.000 0.000 0.000 0.000 0.020

0.000 0.000 0.007 0.000 0.000 0.007 0.007 0.000 0.007 0.007

0.000 0.007 0.000 0.000 0.055 0.076 0.069 0.060 0.000 0.000

0.000 0.010 0.000 0.015 0.000 0.005 0.000 0.010 0.000 0.000

0.005 0.000 0.010 0.005 0.000 0.000 0.000 0.000 0.066 0.068

0.074 0.064

>Non-Methylation code 495

0.025 0.000 0.000 0.000 0.000 0.017 0.008 0.000 0.000 0.008

0.000 0.000 0.051 0.000 0.000 0.000 0.000 0.000 0.000 0.000

0.054 0.041 0.067 0.061 0.011 0.000 0.000 0.000 0.000 0.000

0.000 0.000 0.000 0.005 0.000 0.000 0.016 0.005 0.021 0.005

0.005 0.000 0.000 0.000 0.063 0.058 0.076 0.067 0.014 0.000

0.000 0.000 0.000 0.014 0.000 0.000 0.000 0.000 0.000 0.000

0.027 0.000 0.020 0.007 0.000 0.000 0.000 0.000 0.048 0.043

0.088 0.073

>Non-Methylation code 496

0.006 0.000 0.000 0.000 0.006 0.006 0.024 0.012 0.000 0.006

0.000 0.000 0.000 0.000 0.006 0.000 0.006 0.000 0.000 0.000

0.076 0.083 0.031 0.071 0.007 0.014 0.000 0.000 0.000 0.007

0.007 0.000 0.000 0.007 0.000 0.000 0.000 0.000 0.007 0.014

0.028 0.000 0.000 0.000 0.068 0.077 0.041 0.058 0.010 0.005

0.005 0.000 0.000 0.000 0.005 0.000 0.000 0.005 0.000 0.000

0.014 0.000 0.010 0.000 0.005 0.000 0.000 0.000 0.065 0.063

0.079 0.070

>Non-Methylation code 497

0.010 0.000 0.000 0.000 0.000 0.000 0.005 0.000 0.000 0.005

0.005 0.005 0.005 0.000 0.014 0.000 0.014 0.000 0.000 0.000

0.080 0.062 0.063 0.066 0.014 0.000 0.000 0.000 0.000 0.000

0.005 0.005 0.000 0.000 0.000 0.000 0.000 0.005 0.018 0.005

0.005 0.000 0.000 0.000 0.079 0.065 0.047 0.089 0.024 0.000

0.006 0.000 0.000 0.000 0.012 0.000 0.000 0.000 0.000 0.006

0.006 0.000 0.006 0.000 0.006 0.000 0.000 0.000 0.087 0.059

0.049 0.073

>Non-Methylation code 498

0.013 0.000 0.000 0.000 0.000 0.000 0.004 0.000 0.000 0.004

0.000 0.000 0.009 0.000 0.017 0.000 0.009 0.000 0.000 0.000

0.094 0.051 0.063 0.069 0.018 0.000 0.000 0.000 0.000 0.000

0.005 0.000 0.000 0.000 0.000 0.000 0.014 0.000 0.014 0.005

0.005 0.000 0.000 0.000 0.078 0.081 0.050 0.064 0.013 0.000

0.000 0.000 0.000 0.000 0.009 0.000 0.000 0.000 0.000 0.000

0.018 0.000 0.013 0.000 0.000 0.000 0.000 0.000 0.085 0.067

0.054 0.074

>Non-Methylation code 499

0.009 0.004 0.004 0.000 0.000 0.000 0.009 0.000 0.000 0.009

0.000 0.000 0.009 0.000 0.009 0.000 0.004 0.000 0.000 0.000

0.069 0.067 0.074 0.067 0.005 0.000 0.005 0.000 0.005 0.005

0.010 0.010 0.000 0.010 0.000 0.000 0.000 0.000 0.010 0.000

0.005 0.000 0.000 0.000 0.083 0.063 0.050 0.075 0.013 0.006

0.000 0.000 0.000 0.006 0.006 0.000 0.000 0.006 0.000 0.000

0.000 0.000 0.006 0.013 0.019 0.000 0.000 0.000 0.071 0.072

0.057 0.055

>Non-Methylation code 500

0.020 0.000 0.000 0.000 0.000 0.000 0.005 0.000 0.000 0.000

0.005 0.000 0.010 0.000 0.010 0.005 0.010 0.000 0.000 0.000

0.063 0.069 0.054 0.081 0.008 0.000 0.000 0.000 0.000 0.000

0.004 0.000 0.000 0.004 0.000 0.000 0.013 0.000 0.017 0.000

0.004 0.000 0.000 0.000 0.084 0.057 0.061 0.081 0.015 0.000

0.005 0.000 0.000 0.005 0.010 0.000 0.000 0.005 0.000 0.000

0.005 0.000 0.010 0.000 0.005 0.000 0.000 0.000 0.085 0.068

0.031 0.091

>Non-Methylation code 501

0.012 0.000 0.000 0.000 0.000 0.000 0.004 0.000 0.000 0.008

0.000 0.000 0.004 0.000 0.020 0.000 0.004 0.000 0.000 0.000

0.086 0.065 0.027 0.102 0.016 0.000 0.000 0.000 0.000 0.005

0.005 0.000 0.000 0.000 0.000 0.000 0.005 0.000 0.016 0.005

0.005 0.005 0.000 0.000 0.078 0.067 0.043 0.082 0.014 0.000

0.000 0.005 0.000 0.000 0.005 0.000 0.000 0.000 0.000 0.000

0.009 0.000 0.014 0.005 0.005 0.000 0.000 0.000 0.086 0.069

0.049 0.073

>Non-Methylation code 502

0.017 0.000 0.000 0.000 0.000 0.006 0.006 0.000 0.000 0.000

0.000 0.006 0.006 0.000 0.011 0.006 0.006 0.006 0.000 0.000

0.079 0.078 0.036 0.072 0.014 0.000 0.000 0.005 0.000 0.000

0.005 0.000 0.000 0.000 0.000 0.000 0.009 0.000 0.014 0.005

0.009 0.000 0.000 0.000 0.082 0.068 0.048 0.077 0.009 0.000

0.000 0.000 0.000 0.000 0.004 0.000 0.000 0.009 0.000 0.000

0.004 0.000 0.022 0.000 0.004 0.000 0.000 0.000 0.085 0.068

0.030 0.097

>Non-Methylation code 503

0.000 0.005 0.000 0.011 0.000 0.000 0.005 0.000 0.000 0.005

0.000 0.005 0.005 0.005 0.000 0.011 0.000 0.005 0.011 0.000

0.070 0.068 0.074 0.053 0.015 0.000 0.000 0.000 0.007 0.015

0.000 0.000 0.007 0.000 0.007 0.007 0.007 0.007 0.000 0.015

0.007 0.000 0.000 0.000 0.063 0.068 0.062 0.045 0.008 0.000

0.000 0.000 0.000 0.008 0.000 0.000 0.008 0.024 0.000 0.000

0.008 0.008 0.008 0.000 0.008 0.000 0.000 0.000 0.068 0.082

0.046 0.057

>Non-Methylation code 504

0.006 0.000 0.000 0.000 0.012 0.006 0.000 0.000 0.000 0.006

0.000 0.000 0.006 0.000 0.012 0.012 0.012 0.000 0.000 0.000

0.067 0.066 0.061 0.067 0.006 0.000 0.000 0.006 0.012 0.006

0.000 0.000 0.000 0.000 0.000 0.000 0.024 0.012 0.000 0.006

0.000 0.000 0.006 0.000 0.064 0.077 0.061 0.054 0.006 0.000

0.000 0.000 0.006 0.006 0.012 0.000 0.000 0.017 0.000 0.000

0.000 0.006 0.006 0.012 0.000 0.000 0.000 0.000 0.046 0.057

0.081 0.079

>Non-Methylation code 505

0.011 0.000 0.000 0.000 0.000 0.000 0.006 0.000 0.000 0.006

0.006 0.000 0.006 0.000 0.011 0.006 0.017 0.000 0.006 0.000

0.074 0.068 0.048 0.071 0.009 0.000 0.000 0.000 0.000 0.004

0.004 0.000 0.000 0.009 0.000 0.000 0.009 0.000 0.013 0.000

0.004 0.000 0.000 0.000 0.078 0.055 0.060 0.087 0.016 0.000

0.005 0.000 0.000 0.005 0.011 0.000 0.000 0.005 0.000 0.000

0.005 0.000 0.005 0.000 0.005 0.000 0.000 0.005 0.073 0.060

0.056 0.079

>Non-Methylation code 506

0.016 0.005 0.000 0.000 0.010 0.005 0.000 0.000 0.000 0.010

0.000 0.000 0.005 0.000 0.005 0.000 0.000 0.005 0.005 0.000

0.068 0.065 0.064 0.069 0.013 0.000 0.000 0.000 0.013 0.006

0.000 0.000 0.000 0.019 0.000 0.000 0.000 0.000 0.013 0.013

0.000 0.000 0.000 0.000 0.068 0.061 0.084 0.043 0.010 0.010

0.000 0.000 0.000 0.000 0.000 0.000 0.000 0.016 0.000 0.000

0.005 0.000 0.010 0.005 0.000 0.005 0.000 0.000 0.070 0.072

0.085 0.043

>Non-Methylation code 507

0.018 0.000 0.000 0.000 0.000 0.000 0.000 0.000 0.000 0.000

0.000 0.000 0.013 0.000 0.018 0.004 0.004 0.000 0.000 0.000

0.083 0.084 0.041 0.068 0.017 0.000 0.000 0.000 0.000 0.000

0.008 0.000 0.000 0.000 0.000 0.000 0.017 0.000 0.012 0.000

0.000 0.000 0.000 0.000 0.081 0.069 0.049 0.081 0.009 0.000

0.000 0.000 0.000 0.000 0.005 0.000 0.000 0.005 0.000 0.000

0.009 0.000 0.019 0.000 0.009 0.000 0.000 0.000 0.095 0.053

0.070 0.060

>Non-Methylation code 508

0.010 0.005 0.000 0.005 0.000 0.000 0.005 0.000 0.005 0.005

0.000 0.005 0.000 0.005 0.005 0.005 0.000 0.005 0.000 0.000

0.054 0.072 0.084 0.065 0.012 0.000 0.000 0.000 0.006 0.000

0.000 0.000 0.000 0.006 0.006 0.012 0.012 0.000 0.006 0.012

0.006 0.000 0.000 0.000 0.077 0.061 0.061 0.060 0.000 0.009

0.000 0.000 0.000 0.009 0.000 0.000 0.000 0.018 0.000 0.000

0.018 0.018 0.000 0.000 0.018 0.009 0.000 0.000 0.063 0.068

0.041 0.060

>Non-Methylation code 509

0.011 0.006 0.000 0.000 0.000 0.000 0.000 0.000 0.000 0.006

0.000 0.000 0.011 0.000 0.006 0.011 0.000 0.006 0.011 0.000

0.074 0.063 0.060 0.067 0.015 0.000 0.000 0.000 0.007 0.015

0.000 0.000 0.000 0.030 0.000 0.000 0.000 0.000 0.007 0.007

0.000 0.007 0.007 0.000 0.055 0.058 0.050 0.072 0.017 0.000

0.000 0.000 0.000 0.009 0.000 0.000 0.000 0.043 0.000 0.000

0.000 0.000 0.009 0.009 0.000 0.009 0.000 0.000 0.057 0.058

0.072 0.052

>Non-Methylation code 510

0.016 0.005 0.005 0.000 0.000 0.005 0.000 0.005 0.011 0.000

0.000 0.000 0.000 0.000 0.005 0.000 0.011 0.000 0.000 0.000

0.054 0.067 0.076 0.072 0.011 0.000 0.000 0.000 0.005 0.000

0.000 0.000 0.000 0.016 0.000 0.005 0.011 0.000 0.011 0.000

0.005 0.000 0.000 0.000 0.085 0.061 0.070 0.052 0.000 0.006

0.006 0.000 0.000 0.006 0.011 0.000 0.000 0.006 0.000 0.000

0.000 0.011 0.006 0.000 0.006 0.000 0.006 0.000 0.057 0.083

0.075 0.057

>Non-Methylation code 511

0.009 0.000 0.000 0.009 0.017 0.009 0.000 0.009 0.000 0.017

0.000 0.000 0.000 0.009 0.000 0.009 0.000 0.017 0.000 0.000

0.065 0.056 0.043 0.066 0.007 0.000 0.000 0.000 0.007 0.000

0.000 0.000 0.000 0.007 0.000 0.000 0.007 0.000 0.013 0.047

0.000 0.000 0.000 0.000 0.049 0.075 0.064 0.058 0.004 0.000

0.000 0.000 0.004 0.004 0.000 0.000 0.000 0.009 0.000 0.000

0.009 0.000 0.013 0.000 0.000 0.004 0.004 0.000 0.060 0.078

0.082 0.061

>Non-Methylation code 512

0.014 0.000 0.000 0.000 0.000 0.000 0.005 0.005 0.000 0.000

0.000 0.000 0.000 0.005 0.019 0.005 0.000 0.000 0.000 0.000

0.083 0.061 0.041 0.096 0.020 0.005 0.005 0.000 0.000 0.000

0.010 0.000 0.000 0.000 0.000 0.005 0.005 0.000 0.005 0.000

0.005 0.000 0.000 0.000 0.085 0.055 0.048 0.084 0.010 0.000

0.000 0.000 0.000 0.000 0.005 0.000 0.000 0.005 0.005 0.000

0.005 0.000 0.015 0.000 0.015 0.000 0.000 0.000 0.082 0.058

0.064 0.068

>Non-Methylation code 513

0.005 0.005 0.000 0.000 0.010 0.010 0.000 0.000 0.000 0.014

0.000 0.000 0.000 0.000 0.005 0.005 0.000 0.005 0.005 0.000

0.067 0.055 0.077 0.072 0.018 0.009 0.009 0.000 0.000 0.009

0.000 0.000 0.000 0.037 0.000 0.000 0.009 0.000 0.000 0.009

0.000 0.018 0.000 0.000 0.071 0.049 0.043 0.051 0.006 0.006

0.000 0.000 0.006 0.000 0.000 0.000 0.000 0.019 0.000 0.000

0.000 0.000 0.006 0.006 0.000 0.000 0.013 0.000 0.066 0.071

0.057 0.076

>Non-Methylation code 514

0.014 0.000 0.000 0.000 0.000 0.007 0.014 0.000 0.000 0.000

0.000 0.007 0.007 0.000 0.007 0.007 0.014 0.007 0.000 0.000

0.075 0.069 0.063 0.045 0.009 0.000 0.000 0.005 0.000 0.000

0.005 0.005 0.000 0.000 0.000 0.000 0.005 0.000 0.019 0.005

0.009 0.000 0.000 0.000 0.078 0.071 0.053 0.070 0.018 0.000

0.000 0.000 0.000 0.000 0.004 0.000 0.000 0.009 0.000 0.000

0.000 0.000 0.018 0.000 0.000 0.000 0.000 0.000 0.085 0.050

0.040 0.110

>Non-Methylation code 515

0.013 0.000 0.004 0.000 0.000 0.004 0.009 0.000 0.000 0.004

0.000 0.000 0.004 0.000 0.013 0.000 0.004 0.000 0.000 0.000

0.087 0.060 0.041 0.088 0.020 0.000 0.000 0.000 0.000 0.000

0.005 0.000 0.000 0.000 0.005 0.000 0.010 0.000 0.010 0.005

0.010 0.000 0.000 0.000 0.063 0.069 0.054 0.081 0.009 0.000

0.000 0.000 0.000 0.000 0.005 0.000 0.000 0.000 0.000 0.000

0.014 0.000 0.018 0.000 0.005 0.000 0.000 0.000 0.079 0.061

0.053 0.090

>Non-Methylation code 516

0.022 0.000 0.000 0.000 0.000 0.000 0.004 0.000 0.000 0.000

0.000 0.000 0.013 0.000 0.013 0.000 0.004 0.000 0.000 0.000

0.088 0.064 0.051 0.074 0.012 0.000 0.000 0.000 0.000 0.000

0.004 0.000 0.000 0.004 0.000 0.000 0.012 0.000 0.017 0.000

0.004 0.000 0.000 0.000 0.087 0.063 0.048 0.082 0.019 0.000

0.000 0.000 0.000 0.000 0.005 0.000 0.000 0.000 0.000 0.000

0.005 0.000 0.019 0.005 0.005 0.000 0.000 0.000 0.093 0.053

0.057 0.074

>Non-Methylation code 517

0.006 0.000 0.000 0.000 0.006 0.006 0.025 0.006 0.000 0.006

0.000 0.000 0.000 0.006 0.006 0.000 0.000 0.000 0.000 0.000

0.068 0.074 0.047 0.075 0.012 0.012 0.006 0.000 0.000 0.000

0.000 0.000 0.000 0.000 0.000 0.006 0.000 0.000 0.006 0.012

0.023 0.000 0.000 0.000 0.078 0.064 0.033 0.083 0.010 0.000

0.000 0.000 0.000 0.000 0.005 0.000 0.000 0.005 0.005 0.000

0.015 0.000 0.015 0.000 0.005 0.000 0.000 0.000 0.071 0.065

0.080 0.058

>Non-Methylation code 518

0.016 0.000 0.000 0.000 0.000 0.000 0.000 0.000 0.000 0.005

0.000 0.000 0.032 0.000 0.011 0.005 0.000 0.000 0.000 0.000

0.063 0.057 0.074 0.070 0.000 0.000 0.006 0.000 0.000 0.006

0.012 0.000 0.000 0.012 0.000 0.000 0.017 0.012 0.006 0.006

0.000 0.000 0.000 0.000 0.066 0.058 0.071 0.062 0.009 0.009

0.000 0.000 0.000 0.000 0.000 0.000 0.000 0.009 0.000 0.000

0.026 0.000 0.000 0.026 0.026 0.000 0.000 0.000 0.062 0.049

0.068 0.049

>Non-Methylation code 519

0.009 0.009 0.000 0.000 0.000 0.009 0.000 0.000 0.000 0.000

0.000 0.009 0.026 0.000 0.000 0.035 0.009 0.000 0.000 0.000

0.067 0.045 0.062 0.055 0.015 0.000 0.000 0.005 0.000 0.000

0.000 0.005 0.000 0.005 0.000 0.000 0.021 0.000 0.010 0.000

0.000 0.000 0.000 0.000 0.058 0.076 0.081 0.056 0.000 0.005

0.005 0.000 0.005 0.005 0.000 0.000 0.005 0.010 0.000 0.000

0.010 0.010 0.005 0.000 0.000 0.000 0.000 0.000 0.076 0.056

0.069 0.071

>Non-Methylation code 520

0.005 0.010 0.000 0.000 0.000 0.010 0.000 0.000 0.005 0.010

0.000 0.000 0.000 0.000 0.010 0.000 0.000 0.000 0.000 0.000

0.061 0.056 0.084 0.082 0.007 0.014 0.014 0.007 0.000 0.007

0.007 0.000 0.000 0.000 0.000 0.000 0.000 0.000 0.000 0.021

0.000 0.000 0.000 0.000 0.083 0.053 0.062 0.058 0.011 0.000

0.000 0.000 0.000 0.006 0.000 0.000 0.006 0.006 0.011 0.000

0.000 0.006 0.006 0.000 0.006 0.000 0.011 0.000 0.049 0.064

0.070 0.082

>Non-Methylation code 521

0.006 0.000 0.000 0.006 0.000 0.017 0.000 0.000 0.000 0.006

0.000 0.006 0.000 0.006 0.012 0.000 0.006 0.000 0.000 0.000

0.071 0.053 0.076 0.070 0.005 0.000 0.005 0.000 0.000 0.015

0.000 0.000 0.000 0.005 0.000 0.000 0.000 0.000 0.010 0.005

0.005 0.005 0.010 0.000 0.076 0.073 0.035 0.085 0.012 0.000

0.006 0.006 0.000 0.018 0.000 0.000 0.000 0.000 0.000 0.000

0.006 0.000 0.006 0.000 0.006 0.000 0.006 0.000 0.072 0.080

0.057 0.056

>Non-Methylation code 522

0.005 0.000 0.005 0.010 0.000 0.010 0.000 0.000 0.000 0.005

0.005 0.000 0.005 0.000 0.010 0.005 0.000 0.000 0.000 0.000

0.072 0.065 0.074 0.061 0.010 0.000 0.000 0.015 0.000 0.005

0.000 0.000 0.005 0.000 0.000 0.000 0.000 0.000 0.015 0.000

0.005 0.000 0.005 0.000 0.065 0.073 0.059 0.076 0.007 0.000

0.000 0.007 0.000 0.020 0.000 0.000 0.013 0.013 0.000 0.007

0.000 0.000 0.007 0.007 0.000 0.000 0.000 0.000 0.081 0.051

0.065 0.058
