# Supplementary material for: Benchmark data for identifying DNA methylation sites via pseudo trinucleotide composition
Source: Data Brief. 2015 May 7;4:87–9. doi: 10.1016/j.dib.2015.04.021 (PMC4510404; doi:10.1016/j.dib.2015.04.021)
Supplement: Supplementary file 1 — Supplementary data [file mmc1.zip › Supp3.doc]

**Supporting Information S3**. **The optimized benchmark dataset derived from the original benchmark dataset by the NCR and SMOTE treatments.** It contains 1,117 DNA methylation samples (including 330 hypothetical methylation samples created by SMOTE) and 1,117 non-methylation samples. See the title of Supporting Information S2 for further explanation.

**I. List of the 1,117 positive samples including 330 hypothetical samples**

>Hypothetical 1

0.077 0.000 0.077 0.000 0.001 0.001 0.001 0.000 0.001 0.077

0.000 0.000 0.077 0.001 0.000 0.077 0.077 0.001 0.001 0.001

0.006 0.006 0.004 0.005 0.001 0.000 0.077 0.001 0.001 0.001

0.000 0.000 0.001 0.000 0.001 0.000 0.077 0.000 0.001 0.001

0.001 0.077 0.077 0.000 0.005 0.006 0.004 0.005 0.000 0.000

0.000 0.000 0.000 0.000 0.000 0.000 0.000 0.001 0.000 0.077

0.000 0.001 0.001 0.000 0.077 0.001 0.001 0.000 0.006 0.004

0.003 0.007

>Hypothetical 2

0.001 0.001 0.001 0.000 0.001 0.000 0.000 0.000 0.001 0.091

0.000 0.000 0.091 0.091 0.000 0.000 0.001 0.000 0.000 0.000

0.008 0.006 0.006 0.005 0.000 0.091 0.091 0.001 0.000 0.001

0.000 0.000 0.001 0.001 0.001 0.000 0.000 0.001 0.001 0.001

0.000 0.001 0.000 0.000 0.008 0.006 0.006 0.004 0.091 0.001

0.000 0.000 0.000 0.000 0.001 0.091 0.001 0.000 0.000 0.000

0.090 0.001 0.001 0.091 0.091 0.001 0.000 0.001 0.008 0.008

0.005 0.005

>Hypothetical 3

0.001 0.000 0.001 0.000 0.000 0.083 0.000 0.001 0.001 0.000

0.000 0.001 0.000 0.000 0.000 0.083 0.001 0.083 0.000 0.000

0.006 0.006 0.006 0.006 0.001 0.000 0.000 0.000 0.083 0.001

0.083 0.000 0.001 0.001 0.002 0.083 0.001 0.000 0.001 0.001

0.083 0.000 0.000 0.000 0.005 0.008 0.005 0.003 0.000 0.001

0.001 0.001 0.083 0.000 0.001 0.000 0.000 0.000 0.000 0.000

0.001 0.001 0.001 0.000 0.083 0.083 0.083 0.000 0.006 0.008

0.004 0.004

>Hypothetical 4

0.000 0.000 0.000 0.059 0.000 0.001 0.000 0.000 0.002 0.001

0.002 0.000 0.000 0.059 0.000 0.001 0.000 0.001 0.059 0.000

0.005 0.005 0.004 0.001 0.057 0.001 0.001 0.001 0.000 0.058

0.001 0.000 0.000 0.001 0.000 0.001 0.000 0.001 0.059 0.058

0.000 0.058 0.000 0.000 0.006 0.006 0.002 0.004 0.059 0.001

0.001 0.059 0.000 0.059 0.000 0.001 0.001 0.059 0.000 0.001

0.059 0.059 0.059 0.059 0.000 0.000 0.000 0.000 0.004 0.005

0.004 0.004

>Hypothetical 5

0.000 0.000 0.001 0.059 0.000 0.000 0.000 0.059 0.059 0.000

0.000 0.000 0.058 0.000 0.001 0.001 0.059 0.059 0.000 0.000

0.006 0.003 0.005 0.003 0.000 0.059 0.001 0.001 0.000 0.001

0.059 0.001 0.058 0.002 0.000 0.001 0.058 0.001 0.000 0.001

0.059 0.000 0.000 0.000 0.005 0.000 0.005 0.003 0.059 0.000

0.000 0.000 0.000 0.000 0.059 0.002 0.001 0.001 0.000 0.001

0.059 0.001 0.058 0.001 0.001 0.001 0.001 0.059 0.002 0.003

0.004 0.005

>Hypothetical 6

0.071 0.000 0.000 0.071 0.000 0.071 0.071 0.001 0.001 0.001

0.000 0.001 0.000 0.001 0.071 0.001 0.001 0.001 0.001 0.000

0.004 0.005 0.005 0.004 0.000 0.071 0.000 0.000 0.000 0.000

0.000 0.000 0.000 0.071 0.000 0.071 0.000 0.000 0.002 0.001

0.000 0.000 0.000 0.000 0.008 0.004 0.007 0.002 0.000 0.071

0.001 0.002 0.000 0.000 0.000 0.000 0.000 0.002 0.000 0.001

0.071 0.001 0.001 0.001 0.071 0.071 0.071 0.000 0.007 0.001

0.007 0.002

>Hypothetical 7

0.000 0.050 0.001 0.001 0.000 0.001 0.050 0.050 0.000 0.004

0.002 0.000 0.049 0.000 0.050 0.001 0.050 0.001 0.050 0.000

0.001 0.003 0.002 0.003 0.050 0.001 0.000 0.000 0.001 0.050

0.000 0.001 0.001 0.000 0.000 0.001 0.050 0.000 0.050 0.000

0.050 0.000 0.000 0.050 0.005 0.002 0.004 0.004 0.000 0.000

0.000 0.000 0.000 0.000 0.050 0.000 0.000 0.001 0.050 0.050

0.050 0.000 0.001 0.001 0.050 0.050 0.000 0.000 0.003 0.003

0.004 0.004

>Hypothetical 8

0.000 0.000 0.111 0.000 0.000 0.002 0.111 0.001 0.000 0.003

0.000 0.000 0.001 0.000 0.001 0.001 0.001 0.001 0.000 0.001

0.008 0.007 0.006 0.006 0.002 0.000 0.000 0.000 0.001 0.001

0.000 0.000 0.000 0.001 0.000 0.000 0.002 0.111 0.111 0.002

0.001 0.111 0.001 0.001 0.007 0.008 0.004 0.007 0.000 0.000

0.111 0.000 0.111 0.001 0.001 0.001 0.111 0.001 0.000 0.000

0.002 0.000 0.001 0.001 0.000 0.001 0.000 0.001 0.008 0.008

0.006 0.009

>Hypothetical 9

0.001 0.091 0.091 0.000 0.000 0.001 0.000 0.000 0.000 0.001

0.000 0.000 0.001 0.091 0.091 0.002 0.001 0.091 0.001 0.002

0.006 0.007 0.003 0.006 0.001 0.000 0.091 0.000 0.000 0.000

0.001 0.001 0.000 0.001 0.000 0.000 0.001 0.000 0.001 0.091

0.001 0.001 0.001 0.000 0.007 0.006 0.004 0.007 0.000 0.000

0.000 0.091 0.000 0.002 0.000 0.001 0.000 0.001 0.000 0.000

0.001 0.091 0.001 0.001 0.091 0.001 0.000 0.001 0.006 0.007

0.005 0.005

>Hypothetical 10

0.089 0.000 0.001 0.091 0.001 0.002 0.000 0.000 0.000 0.090

0.000 0.000 0.001 0.000 0.002 0.000 0.000 0.000 0.091 0.000

0.006 0.010 0.007 0.005 0.002 0.000 0.001 0.001 0.000 0.004

0.000 0.000 0.091 0.001 0.000 0.000 0.001 0.001 0.090 0.001

0.001 0.001 0.090 0.000 0.003 0.004 0.005 0.004 0.000 0.000

0.000 0.001 0.000 0.089 0.000 0.000 0.000 0.091 0.000 0.000

0.090 0.001 0.002 0.000 0.001 0.000 0.000 0.000 0.009 0.007

0.008 0.003

>Hypothetical 11

0.002 0.000 0.001 0.001 0.000 0.003 0.000 0.000 0.125 0.125

0.000 0.001 0.001 0.001 0.124 0.001 0.001 0.001 0.000 0.000

0.007 0.006 0.006 0.009 0.000 0.000 0.000 0.000 0.000 0.125

0.000 0.000 0.000 0.000 0.000 0.000 0.000 0.001 0.003 0.000

0.002 0.000 0.125 0.000 0.011 0.007 0.011 0.007 0.124 0.001

0.001 0.001 0.000 0.001 0.000 0.000 0.000 0.000 0.000 0.000

0.001 0.001 0.002 0.000 0.000 0.124 0.000 0.000 0.010 0.014

0.005 0.005

>Hypothetical 12

0.001 0.000 0.001 0.001 0.000 0.001 0.083 0.000 0.083 0.083

0.000 0.083 0.001 0.000 0.000 0.001 0.083 0.083 0.000 0.000

0.006 0.006 0.003 0.006 0.001 0.000 0.000 0.001 0.000 0.001

0.000 0.000 0.001 0.001 0.000 0.001 0.001 0.001 0.083 0.083

0.001 0.000 0.000 0.000 0.006 0.005 0.004 0.006 0.000 0.000

0.083 0.083 0.000 0.001 0.000 0.000 0.000 0.001 0.000 0.000

0.000 0.083 0.002 0.000 0.001 0.000 0.000 0.000 0.006 0.005

0.007 0.005

>Hypothetical 13

0.066 0.000 0.001 0.001 0.000 0.001 0.000 0.001 0.001 0.001

0.000 0.066 0.001 0.001 0.066 0.066 0.001 0.066 0.001 0.000

0.002 0.004 0.005 0.005 0.000 0.001 0.066 0.066 0.066 0.001

0.000 0.000 0.001 0.001 0.000 0.000 0.001 0.066 0.001 0.000

0.000 0.000 0.066 0.001 0.003 0.004 0.004 0.006 0.001 0.066

0.001 0.066 0.066 0.000 0.001 0.000 0.000 0.000 0.001 0.000

0.000 0.000 0.001 0.001 0.066 0.000 0.000 0.000 0.005 0.005

0.003 0.005

>Hypothetical 14

0.001 0.000 0.000 0.000 0.062 0.002 0.062 0.001 0.062 0.001

0.000 0.000 0.001 0.062 0.001 0.001 0.000 0.062 0.062 0.062

0.005 0.006 0.004 0.002 0.002 0.000 0.000 0.000 0.001 0.001

0.000 0.000 0.062 0.000 0.062 0.000 0.001 0.000 0.062 0.062

0.000 0.000 0.001 0.000 0.004 0.005 0.003 0.004 0.000 0.001

0.000 0.000 0.000 0.001 0.001 0.000 0.000 0.062 0.000 0.000

0.062 0.001 0.000 0.002 0.000 0.062 0.062 0.000 0.004 0.004

0.005 0.004

>Hypothetical 15

0.000 0.077 0.001 0.000 0.000 0.002 0.001 0.077 0.000 0.001

0.000 0.000 0.077 0.000 0.077 0.002 0.002 0.001 0.077 0.001

0.004 0.004 0.004 0.005 0.001 0.000 0.000 0.000 0.000 0.001

0.077 0.000 0.000 0.001 0.000 0.000 0.000 0.001 0.000 0.001

0.002 0.001 0.000 0.077 0.005 0.005 0.004 0.005 0.077 0.000

0.000 0.000 0.000 0.000 0.001 0.000 0.000 0.000 0.077 0.077

0.000 0.000 0.001 0.000 0.077 0.077 0.000 0.000 0.005 0.006

0.005 0.006

>Hypothetical 16

0.003 0.000 0.000 0.000 0.000 0.005 0.000 0.003 0.000 0.008

0.000 0.000 0.003 0.000 0.250 0.003 0.003 0.003 0.000 0.003

0.015 0.015 0.009 0.012 0.003 0.000 0.000 0.000 0.002 0.250

0.002 0.000 0.002 0.002 0.000 0.000 0.003 0.000 0.000 0.003

0.002 0.000 0.002 0.002 0.019 0.017 0.013 0.014 0.000 0.000

0.000 0.000 0.000 0.001 0.001 0.001 0.000 0.001 0.000 0.000

0.003 0.000 0.003 0.003 0.001 0.250 0.000 0.001 0.019 0.018

0.010 0.020

>Hypothetical 17

0.000 0.000 0.062 0.000 0.062 0.062 0.000 0.000 0.000 0.000

0.000 0.001 0.062 0.001 0.062 0.001 0.000 0.000 0.000 0.000

0.004 0.004 0.004 0.004 0.001 0.000 0.062 0.062 0.000 0.000

0.000 0.000 0.001 0.062 0.000 0.000 0.001 0.062 0.000 0.002

0.002 0.001 0.000 0.000 0.003 0.004 0.002 0.003 0.062 0.000

0.001 0.062 0.000 0.062 0.000 0.000 0.000 0.062 0.000 0.001

0.001 0.002 0.000 0.001 0.062 0.000 0.062 0.000 0.004 0.004

0.004 0.003

>Hypothetical 18

0.055 0.000 0.000 0.055 0.000 0.055 0.055 0.000 0.000 0.055

0.000 0.001 0.000 0.001 0.055 0.001 0.000 0.055 0.000 0.000

0.004 0.004 0.004 0.003 0.002 0.000 0.000 0.000 0.055 0.056

0.000 0.000 0.001 0.001 0.000 0.000 0.001 0.000 0.055 0.002

0.001 0.000 0.000 0.000 0.003 0.003 0.002 0.004 0.055 0.055

0.001 0.055 0.001 0.055 0.000 0.000 0.055 0.000 0.000 0.001

0.000 0.002 0.000 0.055 0.000 0.001 0.000 0.055 0.002 0.003

0.004 0.005

>Hypothetical 19

0.000 0.077 0.000 0.000 0.001 0.002 0.077 0.002 0.001 0.003

0.000 0.000 0.000 0.000 0.077 0.000 0.002 0.077 0.077 0.000

0.003 0.006 0.003 0.004 0.000 0.001 0.000 0.000 0.001 0.000

0.000 0.000 0.000 0.001 0.000 0.000 0.000 0.000 0.001 0.002

0.077 0.077 0.000 0.076 0.005 0.005 0.005 0.005 0.000 0.000

0.001 0.001 0.000 0.001 0.000 0.001 0.000 0.001 0.077 0.077

0.000 0.000 0.000 0.001 0.077 0.077 0.000 0.000 0.006 0.004

0.006 0.005

>Hypothetical 20

0.000 0.001 0.091 0.000 0.001 0.002 0.091 0.001 0.000 0.091

0.000 0.000 0.000 0.002 0.000 0.002 0.001 0.091 0.000 0.091

0.005 0.006 0.005 0.005 0.090 0.000 0.000 0.000 0.000 0.001

0.000 0.000 0.000 0.001 0.000 0.001 0.002 0.001 0.001 0.001

0.090 0.002 0.000 0.000 0.007 0.003 0.005 0.006 0.000 0.000

0.091 0.000 0.001 0.000 0.000 0.001 0.091 0.000 0.000 0.000

0.001 0.000 0.001 0.091 0.000 0.001 0.000 0.001 0.005 0.005

0.006 0.008

>Hypothetical 21

0.071 0.001 0.000 0.000 0.071 0.001 0.001 0.001 0.000 0.071

0.000 0.070 0.001 0.001 0.001 0.000 0.001 0.001 0.000 0.000

0.004 0.003 0.006 0.004 0.001 0.071 0.000 0.000 0.001 0.002

0.001 0.071 0.000 0.070 0.000 0.000 0.001 0.001 0.000 0.001

0.071 0.000 0.071 0.000 0.002 0.006 0.004 0.006 0.071 0.000

0.000 0.000 0.071 0.001 0.001 0.000 0.071 0.001 0.000 0.001

0.001 0.071 0.000 0.000 0.001 0.001 0.000 0.000 0.007 0.002

0.002 0.007

>Hypothetical 22

0.001 0.000 0.000 0.000 0.000 0.000 0.001 0.071 0.000 0.002

0.000 0.000 0.001 0.000 0.001 0.000 0.071 0.071 0.071 0.000

0.003 0.006 0.005 0.005 0.001 0.001 0.000 0.000 0.000 0.001

0.071 0.001 0.000 0.002 0.000 0.001 0.001 0.000 0.071 0.001

0.001 0.071 0.000 0.071 0.004 0.003 0.005 0.004 0.001 0.001

0.071 0.000 0.000 0.000 0.071 0.000 0.000 0.002 0.071 0.071

0.001 0.000 0.000 0.000 0.000 0.071 0.000 0.000 0.004 0.004

0.006 0.004

>Hypothetical 23

0.000 0.083 0.083 0.000 0.000 0.000 0.000 0.000 0.083 0.001

0.000 0.000 0.000 0.000 0.001 0.001 0.083 0.000 0.083 0.000

0.004 0.006 0.006 0.006 0.001 0.000 0.001 0.000 0.000 0.083

0.000 0.000 0.000 0.000 0.083 0.000 0.002 0.083 0.000 0.000

0.000 0.000 0.000 0.000 0.004 0.007 0.006 0.006 0.001 0.000

0.000 0.083 0.000 0.001 0.000 0.001 0.000 0.001 0.000 0.083

0.004 0.000 0.001 0.001 0.001 0.000 0.083 0.000 0.006 0.004

0.004 0.006

>Hypothetical 24

0.001 0.000 0.111 0.000 0.001 0.001 0.111 0.000 0.000 0.001

0.000 0.001 0.001 0.111 0.001 0.001 0.001 0.001 0.000 0.000

0.008 0.007 0.008 0.006 0.001 0.000 0.000 0.000 0.000 0.000

0.000 0.001 0.000 0.111 0.111 0.111 0.001 0.001 0.001 0.002

0.000 0.001 0.001 0.000 0.008 0.007 0.009 0.005 0.001 0.111

0.000 0.000 0.001 0.001 0.001 0.000 0.001 0.001 0.000 0.000

0.001 0.001 0.000 0.000 0.111 0.001 0.000 0.000 0.008 0.009

0.005 0.008

>Hypothetical 25

0.001 0.000 0.001 0.001 0.001 0.077 0.000 0.000 0.000 0.001

0.000 0.000 0.001 0.076 0.001 0.000 0.000 0.000 0.077 0.000

0.005 0.004 0.007 0.006 0.001 0.076 0.000 0.076 0.001 0.000

0.000 0.001 0.000 0.077 0.000 0.077 0.077 0.001 0.002 0.001

0.000 0.077 0.000 0.000 0.008 0.004 0.006 0.003 0.002 0.000

0.000 0.000 0.000 0.004 0.000 0.000 0.000 0.001 0.000 0.000

0.004 0.077 0.076 0.003 0.077 0.000 0.000 0.000 0.001 0.003

0.004 0.005

>Hypothetical 26

0.002 0.001 0.001 0.000 0.059 0.001 0.000 0.000 0.059 0.002

0.059 0.000 0.059 0.000 0.059 0.000 0.002 0.000 0.000 0.059

0.004 0.003 0.003 0.003 0.001 0.059 0.000 0.059 0.059 0.000

0.000 0.000 0.059 0.000 0.000 0.000 0.001 0.000 0.000 0.000

0.000 0.000 0.001 0.000 0.005 0.004 0.004 0.002 0.059 0.000

0.000 0.000 0.000 0.001 0.001 0.000 0.000 0.001 0.000 0.059

0.000 0.000 0.000 0.059 0.059 0.059 0.000 0.059 0.005 0.003

0.005 0.004

>Hypothetical 27

0.071 0.000 0.001 0.000 0.000 0.000 0.001 0.000 0.071 0.000

0.000 0.071 0.000 0.000 0.001 0.000 0.000 0.000 0.071 0.000

0.007 0.005 0.005 0.004 0.002 0.001 0.071 0.000 0.000 0.003

0.000 0.000 0.071 0.002 0.071 0.000 0.071 0.001 0.071 0.071

0.004 0.000 0.071 0.000 0.003 0.004 0.004 0.002 0.001 0.000

0.000 0.071 0.000 0.000 0.001 0.000 0.000 0.000 0.000 0.000

0.001 0.000 0.001 0.000 0.071 0.000 0.001 0.000 0.006 0.007

0.005 0.004

>Hypothetical 28

0.000 0.000 0.071 0.071 0.000 0.001 0.000 0.071 0.071 0.071

0.000 0.000 0.001 0.071 0.000 0.001 0.000 0.000 0.001 0.000

0.005 0.004 0.005 0.006 0.000 0.071 0.000 0.000 0.000 0.002

0.000 0.000 0.071 0.000 0.000 0.000 0.001 0.000 0.000 0.000

0.071 0.000 0.000 0.071 0.005 0.005 0.003 0.006 0.001 0.000

0.071 0.000 0.000 0.001 0.001 0.000 0.000 0.000 0.001 0.001

0.071 0.001 0.071 0.001 0.000 0.001 0.001 0.000 0.005 0.005

0.002 0.005

>Hypothetical 29

0.071 0.000 0.000 0.000 0.000 0.001 0.000 0.000 0.071 0.000

0.000 0.000 0.000 0.000 0.001 0.071 0.000 0.000 0.000 0.000

0.008 0.005 0.001 0.008 0.001 0.000 0.000 0.002 0.001 0.002

0.071 0.000 0.000 0.071 0.000 0.000 0.002 0.071 0.071 0.071

0.000 0.071 0.000 0.000 0.004 0.005 0.005 0.003 0.000 0.001

0.000 0.000 0.071 0.001 0.071 0.071 0.000 0.000 0.000 0.001

0.001 0.001 0.000 0.001 0.000 0.000 0.071 0.000 0.006 0.003

0.004 0.005

>Hypothetical 30

0.001 0.000 0.100 0.100 0.000 0.001 0.001 0.000 0.001 0.001

0.000 0.000 0.001 0.000 0.100 0.000 0.100 0.100 0.001 0.001

0.007 0.006 0.007 0.006 0.002 0.000 0.100 0.100 0.000 0.001

0.000 0.001 0.100 0.001 0.000 0.000 0.001 0.001 0.001 0.001

0.002 0.000 0.000 0.001 0.006 0.006 0.005 0.007 0.100 0.000

0.000 0.001 0.000 0.001 0.000 0.000 0.000 0.001 0.000 0.000

0.002 0.000 0.001 0.000 0.000 0.000 0.000 0.000 0.004 0.007

0.008 0.009

>Hypothetical 31

0.000 0.001 0.062 0.000 0.000 0.001 0.000 0.000 0.000 0.001

0.000 0.000 0.062 0.000 0.000 0.000 0.001 0.062 0.002 0.062

0.007 0.004 0.002 0.003 0.003 0.000 0.062 0.000 0.062 0.003

0.000 0.062 0.000 0.062 0.062 0.000 0.002 0.000 0.062 0.002

0.000 0.000 0.001 0.000 0.002 0.004 0.004 0.002 0.001 0.062

0.000 0.000 0.062 0.001 0.001 0.000 0.000 0.004 0.000 0.000

0.000 0.000 0.000 0.062 0.062 0.000 0.062 0.000 0.002 0.004

0.005 0.004

>Hypothetical 32

0.062 0.001 0.001 0.000 0.001 0.000 0.001 0.000 0.000 0.062

0.000 0.000 0.062 0.000 0.062 0.062 0.000 0.001 0.002 0.000

0.006 0.004 0.003 0.005 0.003 0.062 0.000 0.000 0.000 0.003

0.062 0.000 0.000 0.062 0.000 0.000 0.002 0.062 0.062 0.003

0.002 0.000 0.000 0.000 0.001 0.003 0.003 0.003 0.062 0.000

0.000 0.062 0.062 0.001 0.000 0.000 0.000 0.004 0.000 0.000

0.001 0.000 0.000 0.062 0.000 0.000 0.062 0.000 0.003 0.005

0.004 0.004

>Hypothetical 33

0.004 0.000 0.083 0.083 0.000 0.002 0.000 0.001 0.000 0.003

0.000 0.083 0.002 0.000 0.083 0.001 0.001 0.000 0.000 0.000

0.003 0.005 0.002 0.004 0.000 0.000 0.000 0.083 0.000 0.001

0.000 0.083 0.000 0.002 0.000 0.000 0.005 0.083 0.083 0.001

0.083 0.001 0.001 0.000 0.005 0.006 0.005 0.004 0.000 0.000

0.000 0.083 0.000 0.001 0.000 0.000 0.083 0.000 0.000 0.000

0.001 0.000 0.000 0.000 0.000 0.000 0.000 0.000 0.005 0.005

0.007 0.007

>Hypothetical 34

0.059 0.058 0.000 0.000 0.000 0.001 0.000 0.000 0.000 0.000

0.000 0.000 0.058 0.001 0.001 0.058 0.059 0.000 0.001 0.000

0.005 0.006 0.004 0.002 0.001 0.000 0.001 0.000 0.000 0.005

0.059 0.000 0.059 0.059 0.001 0.000 0.059 0.000 0.058 0.003

0.001 0.000 0.000 0.000 0.002 0.002 0.002 0.002 0.002 0.000

0.000 0.059 0.000 0.001 0.059 0.000 0.000 0.058 0.000 0.000

0.001 0.000 0.001 0.059 0.001 0.059 0.059 0.000 0.004 0.004

0.004 0.003

>Hypothetical 35

0.001 0.000 0.001 0.000 0.000 0.006 0.066 0.000 0.000 0.066

0.001 0.000 0.000 0.000 0.000 0.003 0.000 0.066 0.000 0.066

0.003 0.004 0.002 0.002 0.004 0.000 0.000 0.000 0.000 0.001

0.000 0.000 0.000 0.066 0.000 0.000 0.001 0.066 0.001 0.066

0.067 0.066 0.000 0.000 0.004 0.004 0.003 0.006 0.000 0.000

0.000 0.000 0.066 0.001 0.000 0.000 0.066 0.066 0.000 0.000

0.066 0.001 0.001 0.066 0.000 0.000 0.001 0.000 0.004 0.006

0.006 0.003

>Hypothetical 36

0.111 0.000 0.001 0.000 0.000 0.005 0.000 0.000 0.000 0.000

0.111 0.000 0.000 0.000 0.002 0.007 0.111 0.000 0.000 0.000

0.006 0.006 0.006 0.007 0.013 0.000 0.000 0.000 0.000 0.003

0.000 0.000 0.000 0.000 0.000 0.000 0.111 0.000 0.001 0.000

0.001 0.002 0.000 0.000 0.004 0.004 0.005 0.004 0.000 0.000

0.111 0.111 0.000 0.001 0.111 0.000 0.000 0.000 0.000 0.000

0.000 0.006 0.004 0.000 0.000 0.001 0.111 0.000 0.004 0.008

0.004 0.009

>Hypothetical 37

0.100 0.099 0.000 0.000 0.000 0.002 0.000 0.000 0.000 0.001

0.000 0.000 0.099 0.003 0.001 0.000 0.001 0.001 0.001 0.000

0.007 0.004 0.006 0.007 0.002 0.000 0.000 0.000 0.000 0.004

0.100 0.000 0.100 0.100 0.000 0.000 0.001 0.000 0.001 0.006

0.000 0.000 0.000 0.000 0.006 0.005 0.006 0.005 0.002 0.000

0.000 0.000 0.000 0.001 0.000 0.000 0.000 0.000 0.000 0.000

0.000 0.000 0.001 0.100 0.100 0.000 0.100 0.000 0.007 0.003

0.008 0.010

>Hypothetical 38

0.004 0.000 0.111 0.000 0.000 0.006 0.000 0.000 0.000 0.000

0.111 0.000 0.003 0.000 0.000 0.003 0.111 0.001 0.000 0.000

0.006 0.005 0.003 0.006 0.001 0.000 0.000 0.001 0.000 0.001

0.001 0.000 0.000 0.002 0.000 0.000 0.001 0.000 0.001 0.001

0.111 0.000 0.000 0.000 0.009 0.005 0.007 0.009 0.000 0.000

0.111 0.111 0.000 0.002 0.111 0.000 0.000 0.001 0.000 0.000

0.001 0.111 0.001 0.001 0.001 0.000 0.001 0.000 0.009 0.007

0.008 0.005

>Hypothetical 39

0.001 0.000 0.000 0.000 0.000 0.083 0.001 0.000 0.000 0.001

0.000 0.000 0.004 0.000 0.000 0.083 0.001 0.000 0.000 0.000

0.005 0.006 0.005 0.005 0.083 0.001 0.083 0.083 0.000 0.001

0.002 0.000 0.000 0.002 0.000 0.000 0.001 0.001 0.083 0.001

0.001 0.083 0.000 0.000 0.006 0.004 0.004 0.005 0.002 0.083

0.000 0.000 0.000 0.083 0.001 0.000 0.083 0.000 0.000 0.000

0.001 0.001 0.001 0.002 0.002 0.000 0.083 0.000 0.005 0.006

0.003 0.006

>Hypothetical 40

0.110 0.111 0.001 0.000 0.000 0.002 0.000 0.000 0.002 0.005

0.000 0.000 0.002 0.003 0.110 0.001 0.000 0.001 0.000 0.000

0.008 0.007 0.003 0.006 0.001 0.002 0.111 0.000 0.001 0.110

0.000 0.000 0.000 0.001 0.000 0.000 0.001 0.000 0.000 0.001

0.000 0.111 0.001 0.000 0.008 0.011 0.005 0.009 0.004 0.000

0.000 0.000 0.000 0.000 0.000 0.000 0.000 0.001 0.000 0.000

0.001 0.111 0.002 0.002 0.111 0.000 0.000 0.000 0.003 0.011

0.008 0.005

>Hypothetical 41

0.062 0.001 0.000 0.000 0.000 0.001 0.001 0.000 0.000 0.062

0.062 0.062 0.001 0.062 0.001 0.000 0.001 0.000 0.000 0.062

0.004 0.003 0.005 0.005 0.003 0.062 0.000 0.000 0.000 0.001

0.001 0.062 0.001 0.001 0.000 0.000 0.000 0.000 0.001 0.062

0.001 0.062 0.000 0.000 0.005 0.004 0.005 0.001 0.062 0.000

0.000 0.000 0.000 0.000 0.001 0.000 0.000 0.062 0.000 0.001

0.002 0.062 0.000 0.062 0.001 0.001 0.001 0.062 0.004 0.006

0.004 0.003

>Hypothetical 42

0.001 0.000 0.000 0.000 0.000 0.002 0.066 0.000 0.066 0.001

0.000 0.000 0.003 0.000 0.066 0.066 0.001 0.000 0.001 0.000

0.004 0.004 0.003 0.004 0.066 0.000 0.000 0.067 0.066 0.001

0.066 0.000 0.000 0.001 0.000 0.066 0.002 0.066 0.001 0.000

0.066 0.000 0.000 0.000 0.004 0.004 0.003 0.006 0.001 0.000

0.000 0.000 0.000 0.000 0.000 0.000 0.066 0.001 0.000 0.001

0.001 0.000 0.001 0.000 0.066 0.066 0.001 0.000 0.005 0.004

0.005 0.004

>Hypothetical 43

0.003 0.001 0.000 0.001 0.000 0.001 0.000 0.000 0.000 0.001

0.000 0.000 0.166 0.001 0.001 0.000 0.000 0.000 0.001 0.000

0.013 0.012 0.010 0.011 0.004 0.166 0.000 0.000 0.000 0.001

0.000 0.000 0.001 0.001 0.000 0.000 0.001 0.166 0.002 0.166

0.000 0.000 0.002 0.000 0.014 0.014 0.007 0.011 0.166 0.000

0.000 0.000 0.000 0.005 0.000 0.000 0.000 0.002 0.000 0.000

0.004 0.000 0.001 0.001 0.000 0.000 0.000 0.000 0.010 0.010

0.010 0.013

>Hypothetical 44

0.002 0.167 0.000 0.167 0.000 0.001 0.001 0.000 0.001 0.167

0.000 0.000 0.001 0.000 0.001 0.167 0.000 0.000 0.002 0.000

0.011 0.014 0.012 0.009 0.002 0.000 0.000 0.000 0.000 0.005

0.000 0.000 0.000 0.001 0.000 0.000 0.003 0.000 0.001 0.001

0.001 0.000 0.000 0.000 0.010 0.010 0.011 0.010 0.003 0.001

0.000 0.001 0.000 0.001 0.000 0.000 0.000 0.003 0.000 0.000

0.002 0.000 0.001 0.000 0.000 0.000 0.166 0.000 0.011 0.012

0.010 0.011

>Hypothetical 45

0.077 0.001 0.000 0.000 0.000 0.077 0.001 0.000 0.000 0.077

0.000 0.000 0.001 0.000 0.001 0.077 0.000 0.000 0.000 0.000

0.004 0.006 0.006 0.005 0.002 0.001 0.000 0.000 0.000 0.000

0.001 0.000 0.000 0.076 0.000 0.000 0.000 0.077 0.001 0.001

0.002 0.000 0.077 0.000 0.005 0.004 0.004 0.006 0.000 0.000

0.000 0.077 0.000 0.077 0.000 0.000 0.000 0.001 0.000 0.077

0.002 0.000 0.000 0.077 0.000 0.000 0.077 0.000 0.006 0.004

0.006 0.005

>Hypothetical 46

0.059 0.000 0.000 0.000 0.059 0.001 0.059 0.059 0.001 0.000

0.000 0.059 0.001 0.000 0.059 0.000 0.000 0.000 0.001 0.000

0.005 0.003 0.003 0.005 0.000 0.000 0.000 0.000 0.000 0.001

0.059 0.059 0.000 0.000 0.000 0.059 0.000 0.000 0.000 0.000

0.059 0.000 0.000 0.000 0.003 0.004 0.004 0.005 0.001 0.000

0.000 0.001 0.059 0.000 0.000 0.059 0.059 0.001 0.000 0.001

0.059 0.059 0.000 0.000 0.000 0.059 0.001 0.000 0.005 0.003

0.004 0.004

>Hypothetical 47

0.000 0.000 0.000 0.000 0.000 0.083 0.000 0.000 0.000 0.001

0.083 0.000 0.001 0.000 0.002 0.000 0.000 0.000 0.083 0.000

0.004 0.007 0.007 0.006 0.002 0.001 0.001 0.000 0.000 0.083

0.001 0.000 0.000 0.083 0.000 0.000 0.002 0.083 0.000 0.000

0.000 0.000 0.083 0.000 0.005 0.005 0.006 0.005 0.001 0.000

0.000 0.083 0.000 0.083 0.000 0.000 0.000 0.001 0.000 0.083

0.003 0.000 0.001 0.000 0.002 0.000 0.083 0.000 0.004 0.006

0.007 0.005

>Hypothetical 48

0.004 0.000 0.000 0.000 0.000 0.001 0.000 0.000 0.000 0.200

0.000 0.000 0.006 0.000 0.002 0.002 0.000 0.000 0.000 0.000

0.010 0.012 0.016 0.014 0.004 0.199 0.199 0.000 0.000 0.000

0.001 0.000 0.000 0.001 0.000 0.000 0.010 0.000 0.002 0.000

0.000 0.000 0.000 0.000 0.012 0.013 0.016 0.009 0.200 0.000

0.000 0.000 0.000 0.000 0.000 0.000 0.000 0.001 0.000 0.000

0.003 0.000 0.004 0.001 0.000 0.001 0.000 0.000 0.010 0.016

0.018 0.013

>Hypothetical 49

0.005 0.000 0.000 0.000 0.000 0.001 0.000 0.000 0.000 0.166

0.000 0.000 0.006 0.000 0.001 0.003 0.000 0.166 0.000 0.000

0.009 0.009 0.013 0.010 0.001 0.000 0.000 0.000 0.000 0.166

0.002 0.000 0.000 0.002 0.000 0.000 0.007 0.000 0.002 0.165

0.000 0.000 0.000 0.000 0.012 0.016 0.015 0.002 0.164 0.000

0.000 0.000 0.000 0.000 0.000 0.000 0.000 0.001 0.000 0.000

0.002 0.000 0.003 0.001 0.001 0.001 0.000 0.000 0.007 0.013

0.017 0.012

>Hypothetical 50

0.166 0.000 0.000 0.000 0.000 0.001 0.000 0.000 0.000 0.001

0.000 0.000 0.003 0.000 0.003 0.001 0.000 0.001 0.000 0.000

0.010 0.014 0.013 0.010 0.005 0.000 0.000 0.000 0.000 0.001

0.000 0.000 0.000 0.167 0.000 0.000 0.004 0.000 0.002 0.002

0.000 0.000 0.000 0.000 0.008 0.008 0.011 0.013 0.003 0.000

0.000 0.000 0.000 0.167 0.000 0.000 0.000 0.001 0.000 0.000

0.006 0.000 0.002 0.167 0.000 0.167 0.000 0.000 0.012 0.012

0.013 0.006

>Hypothetical 51

0.008 0.000 0.000 0.000 0.000 0.002 0.000 0.000 0.000 0.000

0.000 0.000 0.010 0.000 0.002 0.004 0.000 0.000 0.000 0.000

0.017 0.012 0.013 0.016 0.001 0.000 0.000 0.000 0.000 0.001

0.250 0.000 0.000 0.001 0.000 0.000 0.011 0.000 0.003 0.000

0.000 0.000 0.000 0.000 0.016 0.019 0.020 0.011 0.000 0.000

0.000 0.000 0.000 0.001 0.000 0.000 0.000 0.250 0.000 0.000

0.006 0.000 0.005 0.001 0.250 0.001 0.000 0.000 0.019 0.023

0.015 0.013

>Hypothetical 52

0.001 0.000 0.000 0.000 0.000 0.001 0.000 0.000 0.000 0.250

0.000 0.000 0.007 0.000 0.004 0.250 0.000 0.001 0.000 0.000

0.018 0.020 0.016 0.015 0.007 0.000 0.000 0.000 0.000 0.002

0.000 0.000 0.000 0.000 0.000 0.000 0.012 0.000 0.001 0.001

0.000 0.000 0.000 0.000 0.019 0.015 0.013 0.013 0.001 0.000

0.000 0.000 0.000 0.002 0.000 0.000 0.000 0.250 0.000 0.000

0.013 0.000 0.003 0.000 0.000 0.000 0.000 0.000 0.019 0.021

0.017 0.008

>Hypothetical 53

0.003 0.000 0.000 0.000 0.091 0.001 0.000 0.000 0.000 0.091

0.000 0.000 0.004 0.091 0.001 0.001 0.000 0.000 0.000 0.000

0.006 0.005 0.006 0.005 0.000 0.000 0.000 0.000 0.091 0.000

0.000 0.000 0.000 0.000 0.000 0.091 0.004 0.000 0.001 0.091

0.000 0.000 0.000 0.000 0.008 0.006 0.007 0.004 0.000 0.000

0.000 0.000 0.000 0.001 0.000 0.000 0.000 0.091 0.000 0.000

0.003 0.091 0.001 0.091 0.091 0.000 0.000 0.000 0.007 0.006

0.005 0.006

>Hypothetical 54

0.001 0.125 0.125 0.000 0.000 0.001 0.000 0.000 0.000 0.000

0.000 0.000 0.004 0.125 0.002 0.125 0.000 0.001 0.000 0.000

0.010 0.010 0.007 0.007 0.001 0.000 0.000 0.000 0.000 0.001

0.000 0.000 0.000 0.000 0.000 0.000 0.003 0.125 0.002 0.001

0.125 0.000 0.000 0.000 0.010 0.009 0.006 0.009 0.003 0.000

0.000 0.000 0.000 0.002 0.000 0.000 0.000 0.000 0.000 0.000

0.005 0.125 0.001 0.000 0.000 0.000 0.000 0.000 0.007 0.007

0.007 0.009

>Hypothetical 55

0.001 0.000 0.000 0.000 0.000 0.001 0.000 0.000 0.000 0.000

0.000 0.000 0.003 0.125 0.001 0.001 0.125 0.000 0.000 0.000

0.009 0.009 0.008 0.008 0.002 0.000 0.000 0.000 0.000 0.001

0.000 0.000 0.000 0.000 0.000 0.000 0.004 0.125 0.002 0.000

0.000 0.000 0.000 0.000 0.009 0.007 0.009 0.009 0.000 0.125

0.125 0.000 0.000 0.001 0.000 0.000 0.000 0.001 0.000 0.000

0.003 0.125 0.002 0.125 0.000 0.001 0.000 0.000 0.010 0.010

0.005 0.009

>Hypothetical 56

0.001 0.000 0.000 0.062 0.000 0.001 0.000 0.000 0.000 0.000

0.000 0.000 0.001 0.000 0.001 0.062 0.062 0.062 0.000 0.062

0.005 0.003 0.005 0.004 0.001 0.000 0.000 0.062 0.000 0.001

0.000 0.062 0.000 0.000 0.000 0.062 0.002 0.000 0.001 0.062

0.000 0.000 0.000 0.000 0.004 0.005 0.002 0.005 0.001 0.000

0.000 0.062 0.000 0.001 0.062 0.062 0.000 0.062 0.000 0.062

0.002 0.000 0.001 0.001 0.000 0.062 0.000 0.000 0.004 0.004

0.004 0.005

>Hypothetical 57

0.001 0.000 0.000 0.000 0.090 0.002 0.000 0.000 0.000 0.090

0.000 0.000 0.004 0.090 0.001 0.002 0.000 0.000 0.000 0.000

0.006 0.006 0.003 0.007 0.091 0.000 0.000 0.000 0.090 0.002

0.000 0.000 0.000 0.000 0.000 0.091 0.004 0.000 0.002 0.090

0.000 0.000 0.000 0.000 0.007 0.003 0.007 0.009 0.001 0.000

0.000 0.000 0.000 0.002 0.000 0.000 0.000 0.001 0.000 0.000

0.001 0.090 0.001 0.090 0.090 0.000 0.000 0.000 0.007 0.005

0.006 0.007

>Hypothetical 58

0.008 0.199 0.199 0.000 0.000 0.002 0.000 0.000 0.000 0.003

0.000 0.000 0.003 0.000 0.004 0.002 0.003 0.000 0.000 0.000

0.001 0.013 0.019 0.011 0.002 0.003 0.000 0.000 0.000 0.000

0.000 0.000 0.000 0.000 0.000 0.000 0.009 0.002 0.002 0.000

0.199 0.000 0.000 0.000 0.015 0.019 0.006 0.011 0.199 0.002

0.002 0.001 0.000 0.000 0.000 0.000 0.000 0.000 0.000 0.000

0.005 0.001 0.000 0.002 0.000 0.000 0.000 0.000 0.010 0.013

0.016 0.013

>Hypothetical 59

0.001 0.000 0.000 0.000 0.000 0.000 0.000 0.000 0.000 0.077

0.000 0.077 0.002 0.000 0.001 0.077 0.077 0.000 0.000 0.000

0.006 0.006 0.004 0.005 0.001 0.000 0.001 0.077 0.000 0.001

0.000 0.000 0.000 0.077 0.000 0.000 0.002 0.001 0.001 0.001

0.000 0.077 0.077 0.000 0.005 0.006 0.005 0.003 0.001 0.000

0.000 0.077 0.000 0.077 0.000 0.000 0.000 0.000 0.000 0.077

0.001 0.000 0.001 0.077 0.000 0.000 0.000 0.000 0.005 0.004

0.005 0.007

>Hypothetical 60

0.006 0.000 0.000 0.000 0.000 0.002 0.000 0.000 0.000 0.000

0.000 0.000 0.008 0.002 0.004 0.002 0.002 0.000 0.000 0.000

0.023 0.024 0.018 0.019 0.003 0.000 0.000 0.000 0.000 0.001

0.000 0.000 0.000 0.333 0.000 0.000 0.005 0.002 0.006 0.000

0.000 0.333 0.000 0.000 0.023 0.020 0.024 0.028 0.004 0.002

0.002 0.000 0.000 0.002 0.000 0.000 0.000 0.000 0.000 0.000

0.007 0.002 0.002 0.002 0.000 0.000 0.000 0.000 0.021 0.021

0.020 0.027

>Hypothetical 61

0.001 0.000 0.000 0.050 0.000 0.000 0.000 0.050 0.050 0.050

0.050 0.000 0.002 0.000 0.050 0.000 0.000 0.000 0.050 0.000

0.003 0.003 0.002 0.004 0.001 0.050 0.050 0.000 0.000 0.000

0.000 0.000 0.050 0.000 0.000 0.000 0.003 0.001 0.000 0.000

0.001 0.050 0.050 0.000 0.004 0.003 0.002 0.003 0.000 0.000

0.050 0.050 0.000 0.000 0.000 0.000 0.000 0.050 0.000 0.050

0.001 0.000 0.001 0.050 0.050 0.050 0.000 0.000 0.005 0.003

0.002 0.005

>Hypothetical 62

0.000 0.000 0.000 0.001 0.000 0.001 0.200 0.000 0.000 0.000

0.000 0.000 0.003 0.001 0.005 0.000 0.000 0.000 0.000 0.000

0.017 0.014 0.011 0.014 0.002 0.001 0.001 0.000 0.000 0.002

0.000 0.000 0.000 0.000 0.200 0.000 0.003 0.000 0.002 0.001

0.000 0.000 0.000 0.000 0.016 0.015 0.011 0.014 0.004 0.000

0.000 0.200 0.000 0.002 0.000 0.000 0.000 0.000 0.000 0.000

0.005 0.002 0.001 0.000 0.002 0.000 0.200 0.000 0.014 0.013

0.011 0.014

>Hypothetical 63

0.002 0.001 0.001 0.000 0.000 0.000 0.000 0.000 0.000 0.000

0.000 0.000 0.002 0.083 0.000 0.001 0.000 0.083 0.000 0.000

0.008 0.005 0.002 0.007 0.002 0.000 0.083 0.000 0.000 0.001

0.000 0.000 0.000 0.000 0.000 0.000 0.004 0.001 0.083 0.083

0.001 0.083 0.000 0.000 0.006 0.006 0.002 0.005 0.001 0.000

0.000 0.083 0.000 0.083 0.000 0.000 0.000 0.083 0.000 0.000

0.002 0.000 0.002 0.083 0.083 0.000 0.000 0.000 0.008 0.009

0.003 0.004

>Hypothetical 64

0.002 0.000 0.050 0.000 0.000 0.000 0.000 0.050 0.000 0.050

0.001 0.000 0.001 0.000 0.000 0.000 0.000 0.050 0.000 0.050

0.003 0.003 0.003 0.004 0.050 0.000 0.050 0.000 0.050 0.001

0.000 0.050 0.000 0.050 0.000 0.050 0.001 0.000 0.001 0.000

0.050 0.000 0.000 0.000 0.003 0.005 0.003 0.003 0.000 0.000

0.000 0.001 0.000 0.001 0.000 0.050 0.000 0.000 0.000 0.050

0.050 0.050 0.001 0.050 0.050 0.000 0.050 0.000 0.004 0.004

0.002 0.004

>Hypothetical 65

0.000 0.000 0.000 0.002 0.000 0.001 0.000 0.000 0.000 0.077

0.000 0.000 0.077 0.077 0.002 0.077 0.000 0.077 0.000 0.000

0.004 0.006 0.006 0.006 0.002 0.000 0.000 0.001 0.077 0.000

0.077 0.077 0.001 0.000 0.001 0.001 0.000 0.000 0.000 0.001

0.000 0.000 0.077 0.000 0.004 0.005 0.005 0.005 0.000 0.000

0.000 0.000 0.000 0.000 0.000 0.000 0.000 0.000 0.000 0.000

0.001 0.000 0.002 0.076 0.077 0.077 0.000 0.000 0.004 0.007

0.006 0.006

>Hypothetical 66

0.001 0.001 0.001 0.001 0.000 0.001 0.000 0.000 0.001 0.000

0.001 0.001 0.000 0.001 0.090 0.001 0.000 0.091 0.000 0.000

0.007 0.007 0.005 0.004 0.001 0.001 0.091 0.000 0.000 0.090

0.000 0.000 0.000 0.000 0.000 0.000 0.001 0.000 0.001 0.091

0.001 0.000 0.000 0.000 0.004 0.007 0.006 0.007 0.000 0.000

0.000 0.001 0.000 0.090 0.001 0.000 0.000 0.091 0.000 0.000

0.001 0.091 0.003 0.091 0.091 0.001 0.000 0.000 0.009 0.006

0.007 0.004

>Hypothetical 67

0.100 0.100 0.001 0.000 0.000 0.001 0.000 0.000 0.000 0.000

0.000 0.000 0.001 0.000 0.002 0.100 0.000 0.000 0.000 0.000

0.007 0.006 0.007 0.009 0.001 0.000 0.001 0.000 0.000 0.005

0.100 0.000 0.001 0.001 0.000 0.000 0.002 0.000 0.099 0.000

0.001 0.000 0.000 0.000 0.008 0.003 0.006 0.006 0.002 0.000

0.000 0.100 0.000 0.000 0.000 0.000 0.000 0.100 0.000 0.000

0.001 0.000 0.001 0.100 0.000 0.100 0.000 0.000 0.009 0.005

0.009 0.006

>Hypothetical 68

0.001 0.000 0.000 0.000 0.000 0.055 0.000 0.055 0.000 0.055

0.055 0.055 0.000 0.000 0.001 0.000 0.000 0.055 0.000 0.000

0.004 0.004 0.003 0.005 0.001 0.055 0.000 0.056 0.000 0.000

0.000 0.000 0.000 0.056 0.000 0.000 0.001 0.000 0.001 0.056

0.056 0.000 0.056 0.000 0.005 0.004 0.004 0.003 0.001 0.056

0.000 0.000 0.000 0.000 0.056 0.000 0.000 0.001 0.000 0.056

0.001 0.056 0.000 0.000 0.000 0.056 0.000 0.000 0.003 0.004

0.003 0.004

>Hypothetical 69

0.000 0.000 0.000 0.000 0.000 0.001 0.053 0.000 0.053 0.000

0.000 0.000 0.053 0.053 0.053 0.000 0.000 0.000 0.053 0.000

0.003 0.004 0.003 0.003 0.001 0.000 0.000 0.000 0.053 0.053

0.053 0.053 0.053 0.000 0.053 0.000 0.000 0.000 0.000 0.001

0.000 0.000 0.001 0.000 0.005 0.003 0.004 0.003 0.053 0.000

0.000 0.000 0.053 0.001 0.000 0.000 0.053 0.000 0.053 0.000

0.000 0.000 0.000 0.053 0.000 0.000 0.053 0.000 0.003 0.004

0.004 0.004

>Hypothetical 70

0.002 0.000 0.001 0.000 0.000 0.167 0.000 0.000 0.000 0.000

0.000 0.000 0.005 0.000 0.002 0.001 0.001 0.000 0.000 0.000

0.012 0.011 0.010 0.012 0.001 0.000 0.001 0.000 0.000 0.001

0.167 0.000 0.000 0.001 0.000 0.000 0.004 0.001 0.002 0.000

0.001 0.000 0.000 0.000 0.009 0.014 0.012 0.009 0.002 0.000

0.000 0.000 0.000 0.001 0.000 0.000 0.000 0.167 0.000 0.001

0.004 0.001 0.001 0.167 0.001 0.167 0.000 0.000 0.013 0.007

0.015 0.008

>Hypothetical 71

0.200 0.000 0.001 0.001 0.000 0.001 0.000 0.000 0.000 0.001

0.000 0.000 0.005 0.001 0.002 0.200 0.001 0.000 0.000 0.000

0.013 0.015 0.013 0.012 0.002 0.000 0.000 0.000 0.000 0.001

0.000 0.000 0.000 0.000 0.000 0.001 0.004 0.000 0.002 0.001

0.001 0.000 0.000 0.000 0.017 0.009 0.018 0.010 0.003 0.000

0.001 0.000 0.000 0.001 0.000 0.000 0.000 0.200 0.000 0.000

0.008 0.000 0.001 0.000 0.001 0.200 0.000 0.000 0.014 0.012

0.012 0.015

>Hypothetical 72

0.110 0.111 0.111 0.000 0.000 0.001 0.000 0.000 0.000 0.000

0.000 0.001 0.001 0.111 0.003 0.001 0.001 0.000 0.000 0.000

0.009 0.006 0.010 0.005 0.003 0.000 0.001 0.001 0.000 0.002

0.000 0.000 0.000 0.000 0.000 0.000 0.001 0.111 0.001 0.111

0.001 0.000 0.000 0.000 0.008 0.006 0.006 0.008 0.111 0.000

0.001 0.001 0.000 0.111 0.000 0.000 0.000 0.000 0.000 0.000

0.006 0.001 0.001 0.000 0.001 0.000 0.000 0.000 0.006 0.008

0.007 0.006

>Hypothetical 73

0.123 0.000 0.000 0.000 0.000 0.003 0.000 0.125 0.001 0.000

0.000 0.001 0.002 0.125 0.002 0.002 0.125 0.125 0.000 0.000

0.013 0.010 0.005 0.005 0.004 0.000 0.000 0.002 0.000 0.006

0.000 0.000 0.000 0.000 0.000 0.000 0.003 0.000 0.124 0.001

0.002 0.000 0.000 0.000 0.008 0.003 0.006 0.008 0.000 0.000

0.000 0.002 0.000 0.001 0.124 0.000 0.000 0.000 0.000 0.000

0.002 0.001 0.001 0.000 0.000 0.001 0.000 0.000 0.008 0.009

0.010 0.008

>Hypothetical 74

0.000 0.077 0.077 0.000 0.077 0.001 0.000 0.000 0.000 0.000

0.000 0.000 0.000 0.000 0.001 0.000 0.000 0.000 0.000 0.000

0.005 0.008 0.005 0.003 0.001 0.000 0.077 0.001 0.000 0.002

0.000 0.000 0.000 0.001 0.000 0.000 0.001 0.077 0.000 0.001

0.077 0.077 0.001 0.000 0.006 0.004 0.004 0.005 0.001 0.000

0.000 0.000 0.000 0.001 0.000 0.000 0.000 0.077 0.000 0.000

0.001 0.077 0.001 0.077 0.077 0.001 0.077 0.000 0.005 0.005

0.006 0.004

>Hypothetical 75

0.076 0.076 0.000 0.001 0.076 0.003 0.000 0.076 0.000 0.001

0.076 0.076 0.002 0.000 0.000 0.002 0.001 0.000 0.001 0.000

0.005 0.003 0.006 0.004 0.001 0.077 0.000 0.001 0.000 0.001

0.000 0.000 0.000 0.000 0.000 0.000 0.000 0.000 0.002 0.076

0.077 0.001 0.077 0.000 0.006 0.005 0.006 0.004 0.001 0.077

0.000 0.000 0.000 0.002 0.001 0.000 0.001 0.001 0.000 0.000

0.001 0.001 0.000 0.001 0.000 0.077 0.000 0.000 0.006 0.006

0.004 0.003

>Hypothetical 76

0.083 0.000 0.083 0.001 0.000 0.000 0.000 0.000 0.000 0.001

0.000 0.083 0.001 0.000 0.001 0.082 0.000 0.000 0.001 0.000

0.006 0.006 0.008 0.006 0.001 0.000 0.001 0.083 0.000 0.001

0.000 0.000 0.000 0.083 0.000 0.000 0.001 0.000 0.000 0.001

0.083 0.000 0.001 0.000 0.005 0.005 0.005 0.007 0.002 0.083

0.000 0.001 0.000 0.003 0.000 0.001 0.083 0.001 0.000 0.000

0.002 0.001 0.083 0.000 0.001 0.083 0.000 0.000 0.004 0.006

0.001 0.006

>Hypothetical 77

0.001 0.001 0.000 0.001 0.111 0.001 0.000 0.000 0.001 0.001

0.000 0.000 0.001 0.001 0.001 0.001 0.111 0.111 0.000 0.000

0.007 0.007 0.008 0.008 0.000 0.000 0.000 0.001 0.111 0.000

0.000 0.000 0.000 0.001 0.000 0.000 0.001 0.111 0.001 0.001

0.000 0.000 0.001 0.000 0.008 0.007 0.008 0.007 0.001 0.111

0.000 0.000 0.000 0.004 0.000 0.000 0.001 0.002 0.000 0.111

0.111 0.000 0.001 0.002 0.000 0.001 0.000 0.000 0.008 0.008

0.008 0.003

>Hypothetical 78

0.000 0.067 0.000 0.000 0.000 0.001 0.000 0.000 0.000 0.001

0.000 0.067 0.001 0.000 0.067 0.000 0.067 0.000 0.000 0.067

0.005 0.004 0.005 0.004 0.001 0.067 0.000 0.000 0.000 0.000

0.067 0.000 0.000 0.001 0.000 0.000 0.067 0.066 0.001 0.001

0.067 0.000 0.000 0.000 0.004 0.004 0.005 0.005 0.000 0.000

0.000 0.000 0.000 0.001 0.000 0.000 0.001 0.000 0.000 0.067

0.001 0.067 0.000 0.000 0.067 0.067 0.000 0.000 0.005 0.004

0.004 0.006

>Hypothetical 79

0.000 0.013 0.000 0.000 0.000 0.026 0.000 0.000 0.007 0.000

0.000 0.000 0.013 0.007 0.000 0.013 0.007 0.000 0.000 0.000

0.051 0.048 0.072 0.077 0.028 0.000 0.000 0.000 0.000 0.011

0.006 0.000 0.011 0.006 0.000 0.000 0.006 0.000 0.006 0.000

0.000 0.000 0.000 0.000 0.069 0.063 0.045 0.084 0.000 0.000

0.000 0.000 0.000 0.005 0.005 0.000 0.005 0.009 0.000 0.000

0.009 0.000 0.009 0.000 0.005 0.005 0.005 0.000 0.073 0.068

0.059 0.077

>Hypothetical 80

0.001 0.003 0.000 0.200 0.000 0.004 0.000 0.000 0.000 0.200

0.000 0.000 0.003 0.002 0.001 0.003 0.002 0.000 0.200 0.000

0.009 0.009 0.015 0.015 0.008 0.000 0.200 0.000 0.000 0.002

0.002 0.000 0.002 0.002 0.000 0.000 0.001 0.000 0.002 0.000

0.000 0.000 0.000 0.000 0.012 0.012 0.013 0.011 0.000 0.000

0.000 0.000 0.000 0.000 0.002 0.000 0.000 0.003 0.000 0.000

0.002 0.000 0.001 0.002 0.001 0.002 0.002 0.000 0.015 0.016

0.009 0.014

>Hypothetical 81

0.000 0.083 0.000 0.000 0.000 0.002 0.001 0.083 0.000 0.001

0.000 0.000 0.001 0.000 0.001 0.001 0.001 0.001 0.001 0.000

0.006 0.007 0.003 0.005 0.002 0.002 0.000 0.000 0.000 0.003

0.001 0.000 0.000 0.000 0.000 0.000 0.001 0.001 0.083 0.001

0.001 0.083 0.000 0.083 0.004 0.004 0.005 0.005 0.003 0.000

0.083 0.000 0.000 0.001 0.001 0.000 0.001 0.001 0.083 0.083

0.000 0.000 0.001 0.083 0.083 0.083 0.000 0.000 0.006 0.005

0.005 0.005

>Hypothetical 82

0.001 0.083 0.083 0.001 0.000 0.000 0.003 0.001 0.000 0.001

0.000 0.083 0.083 0.000 0.083 0.000 0.002 0.083 0.001 0.000

0.006 0.003 0.007 0.003 0.083 0.001 0.000 0.000 0.083 0.001

0.001 0.000 0.000 0.001 0.000 0.001 0.001 0.000 0.000 0.083

0.002 0.000 0.000 0.001 0.005 0.004 0.006 0.005 0.000 0.000

0.000 0.000 0.000 0.001 0.003 0.001 0.000 0.000 0.001 0.000

0.083 0.083 0.001 0.001 0.002 0.000 0.000 0.000 0.007 0.004

0.006 0.003

>Hypothetical 83

0.002 0.100 0.100 0.100 0.000 0.000 0.000 0.000 0.100 0.005

0.000 0.100 0.005 0.000 0.000 0.002 0.000 0.000 0.000 0.000

0.005 0.005 0.003 0.007 0.100 0.001 0.000 0.000 0.000 0.000

0.000 0.000 0.000 0.001 0.000 0.000 0.004 0.001 0.001 0.003

0.100 0.000 0.000 0.000 0.006 0.006 0.004 0.008 0.001 0.000

0.000 0.000 0.000 0.100 0.000 0.000 0.000 0.000 0.000 0.000

0.005 0.100 0.001 0.002 0.000 0.000 0.000 0.000 0.006 0.006

0.005 0.008

>Hypothetical 84

0.003 0.001 0.000 0.001 0.000 0.000 0.143 0.000 0.000 0.002

0.000 0.000 0.001 0.001 0.143 0.000 0.000 0.000 0.001 0.000

0.008 0.012 0.007 0.011 0.001 0.000 0.000 0.000 0.000 0.142

0.000 0.000 0.001 0.001 0.143 0.000 0.002 0.000 0.001 0.000

0.000 0.000 0.002 0.000 0.008 0.013 0.006 0.013 0.000 0.000

0.000 0.142 0.000 0.004 0.000 0.000 0.000 0.003 0.000 0.000

0.005 0.000 0.142 0.002 0.000 0.000 0.001 0.000 0.007 0.009

0.008 0.009

>Hypothetical 85

0.004 0.000 0.000 0.000 0.000 0.002 0.002 0.000 0.002 0.000

0.000 0.000 0.004 0.000 0.004 0.000 0.000 0.000 0.006 0.000

0.032 0.042 0.030 0.038 0.008 0.000 0.000 0.000 0.000 0.016

0.000 0.000 0.000 0.004 0.000 0.000 0.012 0.000 0.499 0.004

0.004 0.000 0.004 0.000 0.024 0.030 0.030 0.031 0.009 0.003

0.000 0.003 0.000 0.003 0.000 0.000 0.000 0.009 0.000 0.000

0.006 0.000 0.003 0.000 0.000 0.000 0.000 0.000 0.033 0.034

0.031 0.034

>Hypothetical 86

0.001 0.004 0.000 0.000 0.000 0.001 0.002 0.000 0.000 0.000

0.002 0.000 0.006 0.000 0.142 0.142 0.000 0.002 0.000 0.000

0.006 0.007 0.005 0.012 0.003 0.001 0.000 0.000 0.000 0.142

0.001 0.000 0.000 0.001 0.000 0.000 0.142 0.000 0.001 0.001

0.001 0.000 0.000 0.000 0.010 0.010 0.013 0.005 0.002 0.000

0.000 0.000 0.000 0.143 0.000 0.000 0.000 0.001 0.001 0.000

0.001 0.000 0.002 0.000 0.000 0.142 0.000 0.000 0.012 0.006

0.013 0.010

>Hypothetical 87

0.001 0.000 0.000 0.000 0.000 0.083 0.000 0.000 0.083 0.001

0.001 0.083 0.002 0.000 0.001 0.083 0.000 0.000 0.000 0.000

0.007 0.006 0.005 0.006 0.001 0.003 0.000 0.001 0.000 0.083

0.000 0.000 0.000 0.000 0.000 0.000 0.005 0.001 0.000 0.083

0.083 0.000 0.000 0.000 0.004 0.005 0.004 0.006 0.002 0.000

0.000 0.083 0.000 0.083 0.001 0.000 0.001 0.001 0.000 0.000

0.001 0.083 0.001 0.001 0.000 0.083 0.000 0.000 0.008 0.006

0.007 0.002

>Hypothetical 88

0.002 0.000 0.000 0.002 0.000 0.001 0.000 0.000 0.071 0.002

0.000 0.000 0.071 0.001 0.071 0.071 0.001 0.071 0.001 0.000

0.004 0.001 0.007 0.003 0.000 0.071 0.001 0.000 0.000 0.001

0.001 0.000 0.001 0.071 0.000 0.000 0.001 0.000 0.070 0.071

0.000 0.000 0.001 0.000 0.004 0.004 0.005 0.007 0.070 0.000

0.001 0.071 0.071 0.002 0.000 0.000 0.000 0.071 0.001 0.000

0.001 0.000 0.001 0.003 0.001 0.000 0.000 0.000 0.003 0.005

0.003 0.006

>Hypothetical 89

0.012 0.000 0.006 0.000 0.012 0.006 0.012 0.000 0.000 0.006

0.000 0.000 0.006 0.012 0.000 0.000 0.000 0.006 0.000 0.000

0.056 0.067 0.071 0.060 0.008 0.000 0.000 0.000 0.000 0.000

0.000 0.000 0.000 0.008 0.008 0.008 0.008 0.000 0.008 0.038

0.015 0.000 0.000 0.000 0.063 0.062 0.064 0.045 0.004 0.004

0.000 0.000 0.000 0.004 0.000 0.000 0.000 0.008 0.000 0.000

0.013 0.000 0.013 0.000 0.004 0.000 0.000 0.000 0.089 0.072

0.040 0.083

>Hypothetical 90

0.001 0.000 0.001 0.001 0.000 0.001 0.001 0.000 0.000 0.001

0.000 0.076 0.001 0.002 0.077 0.077 0.000 0.001 0.000 0.000

0.004 0.006 0.004 0.005 0.001 0.000 0.000 0.000 0.000 0.000

0.000 0.077 0.001 0.000 0.001 0.001 0.002 0.076 0.000 0.003

0.001 0.077 0.077 0.000 0.004 0.003 0.005 0.006 0.000 0.000

0.000 0.000 0.077 0.000 0.076 0.000 0.077 0.000 0.000 0.001

0.001 0.077 0.001 0.000 0.001 0.077 0.001 0.000 0.007 0.006

0.004 0.005

>Hypothetical 91

0.059 0.000 0.000 0.059 0.058 0.059 0.059 0.059 0.000 0.000

0.000 0.001 0.001 0.000 0.000 0.001 0.002 0.001 0.001 0.000

0.003 0.004 0.005 0.003 0.059 0.000 0.000 0.001 0.000 0.001

0.000 0.000 0.000 0.001 0.000 0.059 0.000 0.001 0.059 0.059

0.000 0.000 0.000 0.000 0.004 0.004 0.001 0.005 0.001 0.000

0.000 0.000 0.059 0.059 0.000 0.059 0.001 0.001 0.001 0.001

0.000 0.059 0.000 0.000 0.059 0.059 0.000 0.001 0.006 0.001

0.004 0.004

>Hypothetical 92

0.001 0.000 0.000 0.000 0.000 0.066 0.001 0.002 0.001 0.066

0.001 0.066 0.002 0.000 0.066 0.001 0.000 0.066 0.001 0.000

0.004 0.004 0.003 0.005 0.001 0.001 0.066 0.000 0.001 0.000

0.001 0.066 0.000 0.001 0.000 0.066 0.000 0.066 0.001 0.000

0.001 0.000 0.066 0.066 0.006 0.006 0.002 0.004 0.000 0.000

0.001 0.000 0.000 0.066 0.000 0.000 0.066 0.000 0.000 0.001

0.000 0.001 0.000 0.000 0.000 0.000 0.066 0.001 0.005 0.004

0.005 0.004

>Hypothetical 93

0.077 0.000 0.000 0.000 0.001 0.000 0.001 0.000 0.000 0.003

0.000 0.001 0.076 0.001 0.001 0.000 0.000 0.000 0.077 0.077

0.007 0.005 0.004 0.004 0.076 0.000 0.000 0.000 0.000 0.002

0.000 0.001 0.000 0.076 0.000 0.001 0.076 0.076 0.001 0.003

0.001 0.000 0.000 0.002 0.005 0.006 0.005 0.002 0.002 0.077

0.000 0.002 0.000 0.077 0.000 0.002 0.000 0.076 0.000 0.000

0.002 0.000 0.000 0.077 0.001 0.000 0.000 0.000 0.006 0.004

0.005 0.005

>Hypothetical 94

0.000 0.000 0.000 0.002 0.000 0.071 0.071 0.000 0.071 0.001

0.000 0.000 0.000 0.000 0.001 0.071 0.000 0.000 0.000 0.000

0.004 0.004 0.005 0.006 0.071 0.001 0.000 0.071 0.071 0.001

0.071 0.000 0.000 0.000 0.000 0.001 0.000 0.000 0.000 0.000

0.071 0.000 0.071 0.000 0.005 0.004 0.005 0.006 0.002 0.000

0.000 0.001 0.000 0.071 0.000 0.001 0.071 0.000 0.000 0.000

0.071 0.000 0.001 0.000 0.000 0.000 0.000 0.000 0.005 0.005

0.005 0.003

>Hypothetical 95

0.000 0.000 0.059 0.000 0.001 0.059 0.000 0.000 0.000 0.001

0.000 0.059 0.001 0.000 0.059 0.000 0.059 0.059 0.000 0.000

0.003 0.005 0.004 0.003 0.001 0.000 0.001 0.059 0.000 0.000

0.000 0.000 0.000 0.059 0.000 0.000 0.001 0.059 0.000 0.001

0.000 0.000 0.059 0.000 0.004 0.003 0.004 0.005 0.000 0.059

0.000 0.059 0.059 0.059 0.000 0.000 0.000 0.001 0.001 0.059

0.000 0.000 0.000 0.000 0.059 0.000 0.000 0.000 0.004 0.003

0.003 0.004

>Hypothetical 96

0.000 0.000 0.066 0.066 0.000 0.001 0.000 0.001 0.066 0.000

0.000 0.001 0.001 0.000 0.000 0.001 0.000 0.001 0.000 0.000

0.004 0.005 0.004 0.004 0.066 0.000 0.000 0.002 0.001 0.066

0.000 0.001 0.066 0.001 0.066 0.000 0.001 0.001 0.066 0.001

0.000 0.001 0.000 0.000 0.003 0.004 0.003 0.004 0.000 0.066

0.000 0.000 0.002 0.066 0.066 0.000 0.000 0.000 0.000 0.001

0.000 0.066 0.001 0.001 0.000 0.066 0.001 0.066 0.005 0.004

0.004 0.004

>Hypothetical 97

0.000 0.000 0.000 0.001 0.000 0.002 0.077 0.000 0.000 0.076

0.000 0.000 0.077 0.002 0.001 0.076 0.000 0.077 0.000 0.000

0.002 0.005 0.007 0.007 0.002 0.000 0.077 0.001 0.077 0.001

0.001 0.000 0.000 0.076 0.000 0.001 0.076 0.077 0.001 0.001

0.077 0.000 0.001 0.000 0.005 0.005 0.004 0.007 0.002 0.000

0.000 0.000 0.000 0.003 0.000 0.000 0.000 0.000 0.000 0.000

0.077 0.000 0.001 0.000 0.001 0.000 0.000 0.000 0.005 0.005

0.005 0.004

>Hypothetical 98

0.002 0.000 0.000 0.091 0.000 0.002 0.000 0.000 0.091 0.003

0.000 0.000 0.000 0.000 0.001 0.003 0.001 0.091 0.091 0.000

0.007 0.004 0.004 0.004 0.000 0.001 0.000 0.000 0.000 0.001

0.001 0.000 0.091 0.001 0.000 0.000 0.001 0.001 0.000 0.000

0.000 0.091 0.000 0.000 0.006 0.007 0.005 0.006 0.002 0.091

0.000 0.001 0.001 0.091 0.001 0.000 0.091 0.000 0.000 0.000

0.091 0.000 0.000 0.000 0.000 0.000 0.000 0.000 0.006 0.005

0.005 0.009

>Hypothetical 99

0.001 0.000 0.067 0.000 0.001 0.000 0.000 0.067 0.067 0.000

0.000 0.000 0.067 0.000 0.000 0.000 0.000 0.067 0.000 0.067

0.004 0.004 0.004 0.006 0.001 0.000 0.066 0.001 0.067 0.001

0.000 0.067 0.000 0.002 0.000 0.067 0.001 0.001 0.000 0.003

0.001 0.000 0.000 0.000 0.003 0.003 0.003 0.003 0.000 0.000

0.000 0.000 0.000 0.000 0.000 0.066 0.000 0.001 0.000 0.067

0.000 0.000 0.001 0.000 0.067 0.000 0.067 0.000 0.004 0.005

0.004 0.005

>Hypothetical 100

0.000 0.000 0.000 0.001 0.002 0.000 0.000 0.000 0.077 0.002

0.000 0.000 0.076 0.000 0.000 0.002 0.001 0.000 0.000 0.000

0.006 0.006 0.004 0.003 0.002 0.000 0.076 0.001 0.000 0.001

0.001 0.000 0.001 0.077 0.000 0.077 0.076 0.001 0.000 0.006

0.000 0.077 0.000 0.000 0.003 0.004 0.004 0.003 0.001 0.000

0.000 0.000 0.077 0.000 0.000 0.001 0.000 0.001 0.000 0.000

0.001 0.077 0.001 0.077 0.076 0.001 0.077 0.000 0.004 0.005

0.004 0.008

>Hypothetical 101

0.052 0.000 0.000 0.052 0.001 0.052 0.000 0.052 0.001 0.000

0.000 0.052 0.000 0.000 0.001 0.001 0.052 0.000 0.000 0.000

0.003 0.004 0.003 0.004 0.000 0.000 0.052 0.052 0.000 0.001

0.052 0.052 0.052 0.001 0.000 0.052 0.000 0.000 0.001 0.001

0.000 0.000 0.000 0.000 0.005 0.001 0.005 0.003 0.002 0.000

0.052 0.001 0.001 0.000 0.000 0.000 0.000 0.001 0.000 0.052

0.052 0.000 0.000 0.052 0.052 0.001 0.000 0.052 0.003 0.003

0.004 0.004

>Hypothetical 102

0.001 0.000 0.001 0.000 0.000 0.077 0.001 0.077 0.077 0.001

0.077 0.000 0.077 0.001 0.000 0.002 0.001 0.001 0.001 0.000

0.005 0.004 0.005 0.004 0.001 0.000 0.000 0.000 0.077 0.000

0.001 0.000 0.000 0.000 0.001 0.000 0.002 0.000 0.077 0.077

0.001 0.000 0.001 0.000 0.005 0.003 0.006 0.005 0.077 0.000

0.000 0.001 0.000 0.001 0.077 0.000 0.077 0.001 0.000 0.000

0.001 0.000 0.001 0.000 0.001 0.000 0.077 0.000 0.005 0.005

0.004 0.006

>Hypothetical 103

0.001 0.000 0.000 0.000 0.000 0.003 0.000 0.000 0.000 0.000

0.000 0.000 0.001 0.090 0.002 0.091 0.091 0.091 0.091 0.000

0.007 0.007 0.003 0.007 0.007 0.000 0.000 0.000 0.000 0.009

0.000 0.000 0.000 0.004 0.000 0.000 0.002 0.000 0.091 0.000

0.000 0.000 0.000 0.000 0.003 0.003 0.002 0.001 0.001 0.000

0.000 0.091 0.000 0.001 0.091 0.000 0.000 0.091 0.000 0.000

0.001 0.000 0.001 0.001 0.000 0.091 0.001 0.000 0.009 0.004

0.006 0.006

>Hypothetical 104

0.049 0.000 0.000 0.000 0.012 0.049 0.000 0.000 0.000 0.024

0.000 0.000 0.012 0.000 0.000 0.012 0.000 0.000 0.000 0.000

0.054 0.051 0.045 0.025 0.009 0.000 0.000 0.000 0.000 0.009

0.000 0.000 0.000 0.004 0.000 0.000 0.013 0.000 0.013 0.004

0.000 0.000 0.004 0.000 0.087 0.051 0.060 0.077 0.006 0.000

0.000 0.000 0.000 0.029 0.000 0.000 0.000 0.011 0.000 0.000

0.011 0.000 0.011 0.000 0.000 0.000 0.000 0.000 0.075 0.082

0.035 0.072

>Hypothetical 105

0.003 0.000 0.000 0.000 0.002 0.005 0.166 0.000 0.166 0.004

0.000 0.002 0.001 0.000 0.001 0.002 0.000 0.002 0.166 0.000

0.007 0.010 0.010 0.008 0.002 0.000 0.000 0.000 0.000 0.001

0.000 0.000 0.000 0.002 0.000 0.000 0.003 0.000 0.001 0.001

0.001 0.000 0.001 0.000 0.014 0.010 0.010 0.009 0.167 0.001

0.000 0.166 0.000 0.002 0.000 0.000 0.000 0.001 0.000 0.000

0.002 0.001 0.002 0.000 0.000 0.000 0.000 0.000 0.014 0.014

0.009 0.011

>Hypothetical 106

0.000 0.000 0.000 0.000 0.001 0.001 0.090 0.000 0.000 0.090

0.000 0.001 0.090 0.000 0.002 0.090 0.000 0.001 0.000 0.000

0.002 0.006 0.012 0.007 0.001 0.000 0.001 0.000 0.090 0.004

0.000 0.000 0.000 0.001 0.000 0.000 0.001 0.090 0.001 0.000

0.001 0.000 0.000 0.000 0.004 0.007 0.005 0.005 0.002 0.001

0.000 0.001 0.000 0.001 0.000 0.000 0.000 0.091 0.000 0.000

0.090 0.002 0.000 0.089 0.090 0.000 0.000 0.000 0.009 0.006

0.005 0.007

>Hypothetical 107

0.067 0.001 0.000 0.001 0.000 0.066 0.000 0.000 0.000 0.065

0.000 0.000 0.067 0.001 0.001 0.066 0.001 0.065 0.000 0.000

0.006 0.005 0.006 0.006 0.000 0.066 0.000 0.000 0.000 0.000

0.000 0.000 0.000 0.066 0.000 0.000 0.000 0.000 0.002 0.066

0.000 0.001 0.066 0.000 0.002 0.004 0.007 0.006 0.001 0.066

0.001 0.000 0.066 0.002 0.000 0.000 0.000 0.001 0.000 0.000

0.001 0.000 0.001 0.001 0.001 0.066 0.066 0.000 0.003 0.005

0.004 0.003

>Hypothetical 108

0.005 0.000 0.000 0.000 0.000 0.110 0.001 0.000 0.000 0.111

0.000 0.000 0.000 0.110 0.003 0.111 0.000 0.000 0.111 0.000

0.017 0.001 0.015 0.000 0.109 0.000 0.000 0.000 0.000 0.001

0.000 0.000 0.000 0.110 0.001 0.000 0.000 0.000 0.003 0.000

0.000 0.000 0.000 0.000 0.010 0.008 0.009 0.007 0.003 0.000

0.000 0.000 0.000 0.008 0.000 0.000 0.000 0.000 0.000 0.000

0.110 0.000 0.000 0.000 0.000 0.000 0.001 0.000 0.006 0.006

0.009 0.005

>Hypothetical 109

0.001 0.000 0.000 0.001 0.000 0.002 0.000 0.000 0.000 0.001

0.000 0.000 0.002 0.000 0.003 0.002 0.000 0.000 0.001 0.000

0.019 0.013 0.018 0.020 0.005 0.000 0.001 0.000 0.000 0.003

0.000 0.000 0.000 0.000 0.000 0.000 0.001 0.000 0.004 0.000

0.000 0.249 0.000 0.000 0.019 0.018 0.013 0.020 0.000 0.000

0.000 0.005 0.250 0.005 0.000 0.000 0.000 0.001 0.000 0.000

0.005 0.000 0.001 0.250 0.001 0.000 0.000 0.000 0.018 0.015

0.017 0.015

>Hypothetical 110

0.333 0.000 0.000 0.001 0.000 0.002 0.000 0.000 0.000 0.333

0.000 0.000 0.002 0.000 0.005 0.002 0.000 0.000 0.002 0.000

0.018 0.023 0.027 0.027 0.006 0.000 0.002 0.000 0.000 0.006

0.000 0.000 0.000 0.000 0.000 0.000 0.004 0.000 0.004 0.000

0.000 0.004 0.000 0.000 0.023 0.025 0.014 0.024 0.000 0.000

0.000 0.004 0.002 0.004 0.000 0.000 0.000 0.004 0.000 0.000

0.004 0.000 0.002 0.002 0.002 0.000 0.000 0.000 0.021 0.026

0.021 0.023

>Hypothetical 111

0.090 0.091 0.000 0.000 0.000 0.001 0.000 0.001 0.001 0.000

0.001 0.000 0.091 0.000 0.002 0.091 0.000 0.000 0.001 0.000

0.006 0.005 0.009 0.006 0.000 0.001 0.001 0.000 0.000 0.002

0.000 0.000 0.001 0.090 0.000 0.000 0.090 0.000 0.091 0.000

0.000 0.001 0.001 0.000 0.007 0.007 0.005 0.007 0.090 0.090

0.001 0.001 0.000 0.001 0.000 0.000 0.000 0.000 0.000 0.001

0.000 0.000 0.000 0.001 0.001 0.001 0.090 0.000 0.005 0.006

0.008 0.004

>Hypothetical 112

0.002 0.111 0.111 0.000 0.000 0.111 0.001 0.000 0.000 0.001

0.000 0.000 0.002 0.111 0.001 0.111 0.001 0.000 0.001 0.000

0.007 0.008 0.007 0.008 0.001 0.000 0.000 0.000 0.000 0.001

0.000 0.000 0.000 0.000 0.000 0.000 0.005 0.111 0.001 0.111

0.001 0.000 0.001 0.000 0.009 0.006 0.007 0.007 0.001 0.000

0.001 0.000 0.000 0.001 0.000 0.000 0.000 0.001 0.000 0.000

0.004 0.111 0.001 0.000 0.000 0.000 0.000 0.000 0.008 0.005

0.009 0.008

>Hypothetical 113

0.058 0.001 0.000 0.000 0.000 0.000 0.002 0.001 0.000 0.001

0.000 0.059 0.059 0.059 0.058 0.001 0.058 0.001 0.000 0.001

0.004 0.005 0.002 0.003 0.001 0.000 0.001 0.001 0.000 0.058

0.059 0.001 0.059 0.000 0.000 0.000 0.000 0.000 0.058 0.001

0.001 0.001 0.000 0.001 0.003 0.004 0.002 0.004 0.059 0.000

0.059 0.058 0.000 0.058 0.058 0.001 0.000 0.002 0.001 0.001

0.001 0.001 0.000 0.001 0.059 0.000 0.000 0.000 0.004 0.006

0.002 0.002

>Hypothetical 114

0.001 0.056 0.056 0.000 0.056 0.001 0.000 0.056 0.056 0.000

0.000 0.000 0.000 0.001 0.056 0.056 0.056 0.001 0.001 0.000

0.002 0.004 0.003 0.004 0.056 0.000 0.000 0.000 0.000 0.001

0.000 0.000 0.000 0.000 0.055 0.000 0.056 0.000 0.001 0.001

0.000 0.000 0.000 0.000 0.004 0.005 0.004 0.001 0.001 0.000

0.056 0.055 0.000 0.000 0.056 0.000 0.000 0.056 0.000 0.000

0.056 0.055 0.001 0.000 0.000 0.000 0.000 0.000 0.005 0.005

0.004 0.002

>Hypothetical 115

0.001 0.000 0.000 0.000 0.000 0.000 0.000 0.000 0.000 0.066

0.000 0.000 0.066 0.000 0.001 0.001 0.000 0.067 0.000 0.000

0.006 0.006 0.004 0.004 0.001 0.000 0.000 0.067 0.066 0.000

0.067 0.000 0.000 0.000 0.000 0.001 0.067 0.001 0.000 0.067

0.000 0.001 0.001 0.001 0.002 0.004 0.004 0.006 0.000 0.067

0.000 0.001 0.067 0.001 0.000 0.001 0.001 0.000 0.000 0.067

0.000 0.067 0.001 0.001 0.000 0.067 0.067 0.001 0.005 0.006

0.005 0.001

>Hypothetical 116

0.000 0.001 0.001 0.000 0.000 0.001 0.000 0.001 0.002 0.071

0.071 0.000 0.000 0.071 0.000 0.001 0.071 0.000 0.000 0.000

0.007 0.004 0.003 0.005 0.002 0.071 0.000 0.000 0.000 0.071

0.000 0.001 0.002 0.001 0.000 0.001 0.000 0.000 0.000 0.071

0.000 0.000 0.000 0.000 0.005 0.005 0.004 0.003 0.071 0.000

0.071 0.000 0.000 0.071 0.071 0.000 0.000 0.001 0.000 0.001

0.071 0.002 0.001 0.071 0.001 0.000 0.002 0.000 0.004 0.003

0.004 0.008

>Hypothetical 117

0.066 0.000 0.002 0.000 0.000 0.001 0.001 0.000 0.000 0.000

0.000 0.066 0.000 0.001 0.000 0.066 0.065 0.065 0.001 0.001

0.004 0.003 0.007 0.006 0.001 0.000 0.000 0.000 0.001 0.001

0.066 0.001 0.001 0.066 0.000 0.000 0.000 0.000 0.067 0.066

0.002 0.000 0.001 0.000 0.005 0.003 0.003 0.006 0.066 0.066

0.000 0.000 0.001 0.001 0.066 0.000 0.000 0.001 0.000 0.000

0.001 0.066 0.001 0.001 0.066 0.000 0.000 0.000 0.005 0.007

0.006 0.001

>Hypothetical 118

0.071 0.000 0.000 0.000 0.071 0.001 0.000 0.071 0.000 0.001

0.000 0.000 0.001 0.001 0.001 0.071 0.000 0.000 0.000 0.000

0.005 0.005 0.006 0.003 0.071 0.000 0.001 0.000 0.071 0.000

0.071 0.000 0.000 0.000 0.000 0.000 0.071 0.000 0.071 0.001

0.000 0.000 0.000 0.000 0.004 0.004 0.005 0.005 0.000 0.000

0.000 0.000 0.071 0.000 0.000 0.071 0.000 0.071 0.000 0.000

0.000 0.071 0.000 0.001 0.002 0.000 0.000 0.000 0.005 0.005

0.004 0.005

>Hypothetical 119

0.001 0.125 0.001 0.000 0.000 0.001 0.000 0.002 0.002 0.002

0.000 0.001 0.125 0.001 0.125 0.002 0.000 0.000 0.000 0.000

0.007 0.007 0.008 0.010 0.001 0.000 0.000 0.000 0.000 0.000

0.000 0.125 0.001 0.001 0.000 0.125 0.001 0.001 0.002 0.002

0.002 0.000 0.000 0.001 0.008 0.009 0.005 0.009 0.125 0.001

0.001 0.000 0.000 0.000 0.000 0.000 0.001 0.002 0.000 0.001

0.001 0.001 0.001 0.000 0.001 0.000 0.125 0.000 0.009 0.008

0.009 0.009

>Hypothetical 120

0.000 0.000 0.000 0.000 0.059 0.000 0.000 0.059 0.000 0.059

0.000 0.000 0.059 0.000 0.001 0.059 0.000 0.000 0.000 0.000

0.004 0.005 0.004 0.004 0.001 0.000 0.000 0.059 0.058 0.059

0.059 0.000 0.000 0.059 0.000 0.000 0.059 0.000 0.001 0.000

0.000 0.001 0.000 0.000 0.002 0.005 0.006 0.004 0.001 0.059

0.000 0.000 0.059 0.001 0.001 0.000 0.000 0.001 0.000 0.059

0.000 0.001 0.001 0.002 0.001 0.059 0.059 0.000 0.004 0.004

0.003 0.004

>Hypothetical 121

0.083 0.000 0.000 0.001 0.083 0.001 0.083 0.000 0.000 0.001

0.000 0.001 0.000 0.001 0.001 0.002 0.000 0.001 0.000 0.083

0.005 0.005 0.005 0.005 0.000 0.001 0.001 0.001 0.000 0.083

0.001 0.083 0.001 0.083 0.000 0.000 0.083 0.000 0.000 0.000

0.000 0.001 0.000 0.000 0.006 0.008 0.005 0.004 0.001 0.000

0.000 0.000 0.083 0.000 0.083 0.002 0.001 0.000 0.000 0.000

0.083 0.000 0.001 0.001 0.000 0.001 0.000 0.001 0.002 0.008

0.006 0.006

>Hypothetical 122

0.000 0.000 0.000 0.001 0.001 0.001 0.001 0.001 0.091 0.000

0.000 0.000 0.001 0.000 0.000 0.001 0.091 0.000 0.001 0.000

0.005 0.004 0.008 0.006 0.001 0.000 0.000 0.000 0.091 0.001

0.091 0.001 0.000 0.003 0.000 0.000 0.002 0.000 0.091 0.004

0.001 0.001 0.000 0.000 0.004 0.005 0.004 0.003 0.091 0.001

0.091 0.001 0.000 0.091 0.001 0.000 0.000 0.002 0.000 0.000

0.000 0.091 0.001 0.001 0.091 0.000 0.000 0.000 0.007 0.006

0.007 0.005

>Hypothetical 123

0.111 0.000 0.001 0.111 0.000 0.004 0.001 0.000 0.000 0.001

0.000 0.000 0.002 0.000 0.111 0.001 0.001 0.000 0.000 0.000

0.006 0.006 0.006 0.008 0.111 0.001 0.002 0.000 0.000 0.001

0.000 0.001 0.111 0.001 0.000 0.111 0.001 0.000 0.000 0.000

0.000 0.001 0.001 0.000 0.007 0.008 0.007 0.007 0.001 0.001

0.000 0.000 0.000 0.001 0.000 0.000 0.111 0.001 0.000 0.000

0.001 0.000 0.001 0.111 0.001 0.000 0.000 0.001 0.008 0.006

0.008 0.008

>Hypothetical 124

0.000 0.000 0.000 0.000 0.000 0.000 0.062 0.001 0.000 0.001

0.000 0.000 0.062 0.000 0.001 0.000 0.000 0.062 0.062 0.062

0.005 0.006 0.003 0.003 0.000 0.000 0.001 0.001 0.062 0.062

0.000 0.062 0.001 0.062 0.000 0.001 0.001 0.001 0.000 0.002

0.062 0.062 0.000 0.000 0.004 0.004 0.003 0.004 0.062 0.062

0.000 0.000 0.000 0.000 0.000 0.000 0.001 0.000 0.000 0.000

0.000 0.062 0.001 0.062 0.001 0.000 0.000 0.000 0.003 0.004

0.005 0.004

>Hypothetical 125

0.058 0.000 0.000 0.001 0.000 0.058 0.000 0.058 0.000 0.001

0.059 0.001 0.000 0.000 0.001 0.059 0.002 0.001 0.059 0.000

0.003 0.003 0.005 0.004 0.001 0.000 0.000 0.001 0.059 0.059

0.000 0.000 0.000 0.001 0.059 0.000 0.059 0.001 0.001 0.001

0.001 0.000 0.059 0.000 0.005 0.003 0.004 0.003 0.059 0.000

0.001 0.000 0.000 0.058 0.058 0.000 0.000 0.000 0.000 0.059

0.001 0.000 0.001 0.001 0.000 0.000 0.059 0.000 0.004 0.004

0.004 0.004

>Hypothetical 126

0.124 0.000 0.002 0.125 0.000 0.125 0.000 0.000 0.002 0.000

0.000 0.000 0.001 0.000 0.001 0.001 0.000 0.000 0.000 0.000

0.005 0.012 0.010 0.008 0.124 0.000 0.000 0.000 0.000 0.001

0.001 0.000 0.001 0.001 0.000 0.001 0.001 0.124 0.001 0.000

0.003 0.001 0.000 0.000 0.008 0.011 0.007 0.006 0.000 0.000

0.000 0.002 0.000 0.124 0.000 0.000 0.001 0.001 0.000 0.000

0.124 0.004 0.002 0.001 0.003 0.000 0.000 0.000 0.005 0.007

0.010 0.008

>Hypothetical 127

0.002 0.000 0.000 0.001 0.000 0.001 0.000 0.000 0.000 0.002

0.000 0.000 0.002 0.001 0.001 0.001 0.001 0.000 0.000 0.000

0.011 0.012 0.017 0.012 0.200 0.001 0.001 0.000 0.000 0.000

0.001 0.000 0.002 0.001 0.000 0.000 0.005 0.000 0.002 0.002

0.000 0.002 0.000 0.000 0.010 0.015 0.013 0.014 0.001 0.001

0.000 0.000 0.001 0.001 0.000 0.000 0.000 0.200 0.000 0.001

0.004 0.001 0.001 0.200 0.200 0.000 0.000 0.000 0.014 0.017

0.014 0.010

>Hypothetical 128

0.000 0.001 0.001 0.000 0.000 0.000 0.001 0.000 0.001 0.091

0.091 0.000 0.002 0.091 0.000 0.000 0.091 0.001 0.000 0.000

0.005 0.006 0.006 0.007 0.000 0.091 0.000 0.000 0.000 0.000

0.000 0.000 0.000 0.091 0.000 0.000 0.001 0.000 0.001 0.091

0.000 0.000 0.000 0.000 0.006 0.006 0.007 0.006 0.091 0.000

0.091 0.001 0.000 0.001 0.091 0.000 0.000 0.001 0.000 0.000

0.000 0.001 0.001 0.001 0.001 0.000 0.000 0.000 0.004 0.006

0.007 0.008

>Hypothetical 129

0.071 0.000 0.001 0.001 0.000 0.001 0.000 0.000 0.000 0.071

0.000 0.071 0.001 0.001 0.000 0.001 0.001 0.000 0.000 0.071

0.003 0.005 0.005 0.004 0.001 0.071 0.000 0.000 0.000 0.000

0.000 0.000 0.000 0.000 0.000 0.000 0.001 0.071 0.001 0.000

0.071 0.000 0.000 0.000 0.004 0.004 0.007 0.006 0.071 0.000

0.000 0.000 0.000 0.001 0.000 0.000 0.000 0.071 0.000 0.000

0.001 0.071 0.000 0.071 0.071 0.071 0.000 0.000 0.005 0.004

0.006 0.005

>Hypothetical 130

0.001 0.000 0.001 0.001 0.000 0.000 0.111 0.000 0.000 0.000

0.000 0.001 0.001 0.000 0.001 0.000 0.001 0.000 0.000 0.000

0.007 0.007 0.007 0.009 0.110 0.000 0.000 0.001 0.000 0.001

0.000 0.001 0.111 0.002 0.111 0.111 0.001 0.001 0.001 0.001

0.001 0.000 0.000 0.000 0.007 0.007 0.008 0.007 0.111 0.000

0.001 0.001 0.000 0.001 0.001 0.000 0.001 0.000 0.000 0.000

0.000 0.111 0.002 0.001 0.000 0.000 0.111 0.000 0.006 0.009

0.008 0.007

>Hypothetical 131

0.005 0.000 0.000 0.000 0.000 0.000 0.000 0.000 0.002 0.001

0.000 0.000 0.001 0.090 0.090 0.006 0.000 0.090 0.000 0.002

0.004 0.003 0.005 0.005 0.004 0.000 0.000 0.000 0.090 0.000

0.000 0.002 0.000 0.004 0.000 0.002 0.006 0.000 0.089 0.090

0.000 0.000 0.000 0.000 0.005 0.008 0.090 0.004 0.089 0.002

0.000 0.000 0.000 0.090 0.000 0.001 0.000 0.000 0.000 0.000

0.001 0.002 0.001 0.090 0.000 0.000 0.000 0.000 0.002 0.007

0.009 0.009

>Hypothetical 132

0.002 0.083 0.083 0.000 0.000 0.001 0.083 0.000 0.000 0.000

0.000 0.083 0.002 0.000 0.001 0.001 0.083 0.083 0.000 0.000

0.003 0.007 0.005 0.005 0.001 0.000 0.001 0.001 0.083 0.000

0.000 0.000 0.000 0.001 0.000 0.000 0.001 0.000 0.001 0.000

0.083 0.001 0.000 0.083 0.005 0.005 0.007 0.006 0.000 0.001

0.000 0.000 0.000 0.001 0.083 0.000 0.001 0.000 0.083 0.000

0.001 0.001 0.000 0.000 0.001 0.000 0.001 0.000 0.007 0.003

0.006 0.006

>Hypothetical 133

0.000 0.000 0.000 0.000 0.000 0.001 0.002 0.000 0.000 0.076

0.000 0.076 0.002 0.000 0.000 0.077 0.001 0.000 0.001 0.000

0.004 0.005 0.005 0.007 0.077 0.076 0.000 0.001 0.001 0.001

0.001 0.000 0.000 0.076 0.000 0.000 0.001 0.076 0.001 0.076

0.077 0.000 0.076 0.000 0.006 0.006 0.004 0.006 0.077 0.000

0.000 0.000 0.000 0.000 0.001 0.000 0.001 0.000 0.001 0.000

0.001 0.076 0.001 0.001 0.001 0.000 0.000 0.000 0.007 0.004

0.004 0.006

>Hypothetical 134

0.000 0.000 0.000 0.000 0.000 0.001 0.001 0.000 0.001 0.001

0.001 0.001 0.001 0.000 0.001 0.002 0.001 0.001 0.000 0.000

0.010 0.009 0.005 0.008 0.001 0.000 0.000 0.125 0.002 0.002

0.125 0.000 0.000 0.000 0.001 0.125 0.003 0.125 0.000 0.001

0.001 0.000 0.001 0.000 0.008 0.006 0.006 0.010 0.001 0.001

0.000 0.002 0.000 0.001 0.001 0.000 0.125 0.000 0.000 0.000

0.001 0.000 0.001 0.001 0.125 0.125 0.000 0.000 0.011 0.007

0.008 0.008

>Hypothetical 135

0.067 0.000 0.001 0.001 0.001 0.001 0.066 0.001 0.067 0.000

0.000 0.000 0.001 0.000 0.000 0.001 0.000 0.000 0.067 0.067

0.005 0.004 0.004 0.003 0.001 0.001 0.000 0.000 0.001 0.066

0.000 0.001 0.000 0.003 0.000 0.000 0.001 0.066 0.066 0.001

0.066 0.000 0.000 0.066 0.004 0.004 0.002 0.005 0.000 0.000

0.000 0.067 0.000 0.067 0.000 0.000 0.000 0.001 0.000 0.000

0.000 0.000 0.001 0.067 0.067 0.000 0.001 0.000 0.005 0.005

0.005 0.004

>Hypothetical 136

0.001 0.000 0.083 0.000 0.001 0.083 0.083 0.000 0.000 0.001

0.000 0.002 0.001 0.000 0.000 0.002 0.001 0.083 0.000 0.083

0.005 0.005 0.005 0.005 0.001 0.001 0.000 0.000 0.000 0.083

0.000 0.001 0.000 0.002 0.000 0.000 0.002 0.001 0.083 0.001

0.001 0.083 0.001 0.000 0.005 0.004 0.006 0.004 0.001 0.000

0.083 0.000 0.001 0.083 0.000 0.000 0.001 0.083 0.000 0.000

0.001 0.003 0.000 0.001 0.000 0.000 0.000 0.000 0.004 0.007

0.006 0.004

>Hypothetical 137

0.071 0.001 0.000 0.000 0.000 0.070 0.001 0.001 0.000 0.000

0.000 0.000 0.001 0.071 0.000 0.000 0.001 0.001 0.000 0.000

0.005 0.002 0.006 0.006 0.000 0.071 0.000 0.071 0.000 0.000

0.001 0.000 0.000 0.071 0.000 0.071 0.001 0.000 0.001 0.001

0.001 0.000 0.000 0.002 0.005 0.006 0.006 0.002 0.071 0.000

0.001 0.000 0.000 0.071 0.002 0.000 0.000 0.071 0.001 0.001

0.001 0.071 0.071 0.071 0.000 0.001 0.000 0.000 0.004 0.004

0.004 0.007

>Hypothetical 138

0.111 0.000 0.000 0.000 0.001 0.001 0.000 0.000 0.001 0.001

0.001 0.111 0.001 0.000 0.001 0.000 0.002 0.000 0.111 0.002

0.006 0.007 0.008 0.007 0.000 0.001 0.111 0.001 0.001 0.001

0.001 0.000 0.001 0.001 0.111 0.000 0.001 0.000 0.000 0.001

0.001 0.000 0.111 0.000 0.007 0.005 0.009 0.007 0.001 0.000

0.000 0.111 0.000 0.000 0.001 0.000 0.000 0.001 0.000 0.001

0.000 0.111 0.001 0.001 0.000 0.001 0.000 0.001 0.007 0.007

0.008 0.007

>Hypothetical 139

0.002 0.001 0.071 0.001 0.001 0.001 0.001 0.071 0.000 0.001

0.000 0.000 0.000 0.001 0.071 0.001 0.001 0.000 0.000 0.071

0.003 0.003 0.005 0.005 0.071 0.000 0.001 0.071 0.000 0.000

0.000 0.000 0.071 0.001 0.000 0.001 0.000 0.000 0.001 0.001

0.070 0.000 0.000 0.001 0.006 0.005 0.005 0.005 0.000 0.001

0.000 0.000 0.001 0.001 0.071 0.000 0.071 0.000 0.000 0.071

0.071 0.071 0.001 0.000 0.001 0.001 0.000 0.000 0.005 0.004

0.005 0.006

>Hypothetical 140

0.000 0.001 0.000 0.001 0.000 0.076 0.000 0.002 0.000 0.076

0.000 0.000 0.000 0.000 0.001 0.076 0.076 0.076 0.000 0.001

0.005 0.007 0.007 0.005 0.002 0.000 0.000 0.002 0.000 0.076

0.000 0.000 0.002 0.001 0.000 0.000 0.076 0.001 0.076 0.001

0.004 0.001 0.000 0.000 0.005 0.003 0.002 0.004 0.000 0.000

0.077 0.000 0.077 0.077 0.000 0.000 0.001 0.002 0.000 0.002

0.001 0.001 0.001 0.077 0.000 0.000 0.000 0.001 0.004 0.006

0.004 0.005

>Hypothetical 141

0.001 0.001 0.001 0.111 0.001 0.002 0.000 0.000 0.001 0.111

0.000 0.000 0.001 0.000 0.111 0.002 0.001 0.000 0.111 0.000

0.007 0.007 0.007 0.007 0.001 0.000 0.000 0.000 0.000 0.111

0.000 0.000 0.000 0.001 0.000 0.000 0.001 0.000 0.001 0.000

0.001 0.000 0.001 0.000 0.010 0.008 0.005 0.008 0.000 0.001

0.000 0.111 0.000 0.002 0.001 0.001 0.000 0.002 0.000 0.000

0.111 0.001 0.001 0.001 0.111 0.000 0.000 0.000 0.008 0.006

0.009 0.006

>Hypothetical 142

0.076 0.000 0.000 0.000 0.077 0.001 0.001 0.001 0.000 0.002

0.000 0.077 0.077 0.001 0.000 0.000 0.000 0.077 0.000 0.001

0.007 0.004 0.006 0.004 0.000 0.001 0.001 0.000 0.001 0.001

0.000 0.001 0.001 0.076 0.000 0.000 0.077 0.000 0.077 0.001

0.000 0.000 0.077 0.000 0.005 0.007 0.004 0.004 0.002 0.077

0.001 0.000 0.000 0.076 0.000 0.000 0.000 0.001 0.000 0.000

0.001 0.077 0.001 0.000 0.001 0.000 0.000 0.000 0.007 0.007

0.002 0.006

>Hypothetical 143

0.001 0.000 0.000 0.000 0.055 0.000 0.055 0.000 0.000 0.000

0.000 0.055 0.000 0.001 0.055 0.000 0.000 0.000 0.055 0.000

0.003 0.004 0.003 0.003 0.055 0.000 0.000 0.000 0.055 0.055

0.000 0.055 0.000 0.000 0.055 0.000 0.000 0.000 0.001 0.000

0.000 0.000 0.000 0.000 0.005 0.002 0.004 0.004 0.055 0.000

0.000 0.000 0.055 0.001 0.000 0.000 0.055 0.000 0.055 0.000

0.055 0.000 0.000 0.001 0.000 0.055 0.055 0.000 0.003 0.003

0.005 0.004

>Hypothetical 144

0.001 0.002 0.000 0.066 0.000 0.001 0.001 0.000 0.000 0.001

0.000 0.001 0.000 0.000 0.000 0.066 0.066 0.066 0.000 0.066

0.008 0.001 0.007 0.001 0.001 0.001 0.000 0.067 0.000 0.001

0.000 0.067 0.000 0.000 0.000 0.067 0.000 0.000 0.000 0.067

0.001 0.001 0.001 0.000 0.004 0.005 0.005 0.004 0.000 0.001

0.000 0.066 0.000 0.001 0.066 0.066 0.000 0.000 0.000 0.066

0.001 0.001 0.001 0.000 0.000 0.066 0.000 0.000 0.006 0.004

0.003 0.004

>Hypothetical 145

0.000 0.001 0.000 0.083 0.083 0.000 0.000 0.000 0.000 0.001

0.000 0.001 0.001 0.083 0.000 0.083 0.000 0.001 0.000 0.000

0.008 0.005 0.006 0.003 0.001 0.000 0.083 0.000 0.001 0.000

0.001 0.083 0.083 0.001 0.000 0.000 0.001 0.000 0.083 0.001

0.002 0.000 0.000 0.001 0.006 0.003 0.005 0.005 0.000 0.000

0.083 0.000 0.083 0.000 0.001 0.000 0.000 0.083 0.001 0.000

0.002 0.000 0.001 0.001 0.000 0.000 0.000 0.000 0.004 0.005

0.006 0.007

>Hypothetical 146

0.002 0.000 0.000 0.062 0.000 0.062 0.001 0.062 0.062 0.001

0.000 0.062 0.002 0.000 0.062 0.001 0.002 0.000 0.000 0.001

0.004 0.002 0.003 0.004 0.062 0.000 0.000 0.000 0.001 0.062

0.001 0.062 0.062 0.000 0.001 0.000 0.001 0.001 0.001 0.062

0.000 0.000 0.000 0.000 0.003 0.003 0.005 0.006 0.001 0.001

0.000 0.000 0.062 0.062 0.001 0.000 0.062 0.001 0.000 0.001

0.001 0.062 0.000 0.001 0.000 0.000 0.000 0.000 0.006 0.002

0.005 0.004

>Hypothetical 147

0.002 0.000 0.000 0.000 0.000 0.090 0.000 0.001 0.000 0.090

0.000 0.000 0.090 0.001 0.001 0.000 0.000 0.001 0.000 0.000

0.003 0.004 0.009 0.011 0.003 0.090 0.000 0.000 0.000 0.090

0.000 0.000 0.000 0.000 0.000 0.000 0.004 0.090 0.001 0.003

0.000 0.000 0.000 0.000 0.005 0.005 0.007 0.005 0.000 0.090

0.090 0.090 0.000 0.000 0.002 0.000 0.000 0.000 0.000 0.000

0.002 0.000 0.003 0.090 0.000 0.000 0.000 0.000 0.006 0.008

0.007 0.005

>Hypothetical 148

0.111 0.001 0.000 0.000 0.000 0.000 0.111 0.000 0.111 0.001

0.000 0.000 0.000 0.000 0.002 0.003 0.000 0.000 0.000 0.000

0.009 0.009 0.007 0.006 0.002 0.002 0.000 0.000 0.000 0.003

0.000 0.000 0.000 0.111 0.000 0.000 0.000 0.001 0.001 0.111

0.111 0.001 0.000 0.000 0.009 0.005 0.007 0.007 0.002 0.111

0.000 0.002 0.001 0.111 0.000 0.000 0.000 0.000 0.000 0.000

0.000 0.001 0.001 0.001 0.000 0.000 0.000 0.000 0.007 0.009

0.007 0.006

>Hypothetical 149

0.006 0.006 0.000 0.006 0.000 0.024 0.000 0.000 0.006 0.000

0.000 0.000 0.006 0.000 0.012 0.012 0.000 0.000 0.000 0.000

0.062 0.056 0.054 0.085 0.007 0.000 0.000 0.015 0.000 0.022

0.000 0.000 0.000 0.007 0.000 0.000 0.000 0.007 0.015 0.000

0.000 0.007 0.000 0.000 0.071 0.049 0.057 0.074 0.006 0.000

0.000 0.011 0.000 0.006 0.000 0.000 0.011 0.006 0.000 0.000

0.000 0.000 0.006 0.006 0.000 0.000 0.011 0.000 0.063 0.066

0.058 0.083

>Hypothetical 150

0.059 0.000 0.000 0.000 0.059 0.002 0.000 0.059 0.001 0.000

0.000 0.000 0.058 0.001 0.000 0.000 0.000 0.059 0.001 0.000

0.003 0.004 0.003 0.005 0.001 0.000 0.000 0.059 0.058 0.002

0.059 0.000 0.001 0.000 0.000 0.000 0.059 0.000 0.000 0.000

0.000 0.001 0.001 0.000 0.003 0.003 0.003 0.005 0.001 0.000

0.000 0.002 0.059 0.002 0.000 0.000 0.000 0.059 0.000 0.059

0.001 0.059 0.000 0.000 0.059 0.059 0.059 0.000 0.003 0.005

0.004 0.004

>Hypothetical 151

0.001 0.000 0.000 0.000 0.000 0.001 0.000 0.066 0.001 0.000

0.066 0.066 0.000 0.000 0.067 0.001 0.000 0.000 0.002 0.000

0.004 0.005 0.003 0.005 0.000 0.000 0.067 0.001 0.000 0.002

0.000 0.000 0.000 0.067 0.000 0.000 0.001 0.000 0.001 0.001

0.067 0.000 0.067 0.000 0.004 0.006 0.004 0.004 0.066 0.067

0.000 0.000 0.000 0.002 0.067 0.000 0.001 0.001 0.000 0.067

0.066 0.001 0.001 0.001 0.000 0.067 0.001 0.000 0.004 0.005

0.003 0.005

>Hypothetical 152

0.083 0.000 0.000 0.083 0.001 0.001 0.000 0.000 0.083 0.002

0.000 0.000 0.083 0.000 0.001 0.001 0.001 0.001 0.083 0.000

0.005 0.004 0.005 0.006 0.083 0.000 0.000 0.000 0.001 0.000

0.000 0.000 0.083 0.000 0.000 0.000 0.000 0.000 0.001 0.001

0.000 0.083 0.000 0.000 0.005 0.005 0.006 0.008 0.000 0.000

0.001 0.000 0.001 0.001 0.000 0.001 0.083 0.083 0.000 0.000

0.000 0.000 0.000 0.000 0.000 0.001 0.083 0.000 0.005 0.006

0.006 0.005

>Hypothetical 153

0.002 0.002 0.000 0.000 0.000 0.083 0.001 0.083 0.000 0.002

0.000 0.000 0.001 0.083 0.083 0.082 0.001 0.002 0.000 0.000

0.006 0.004 0.004 0.005 0.000 0.000 0.000 0.000 0.001 0.082

0.083 0.000 0.000 0.002 0.000 0.000 0.001 0.000 0.001 0.001

0.001 0.000 0.001 0.000 0.006 0.004 0.006 0.005 0.000 0.001

0.000 0.000 0.001 0.000 0.083 0.000 0.000 0.001 0.000 0.000

0.001 0.083 0.000 0.000 0.083 0.083 0.000 0.000 0.008 0.005

0.007 0.004

>Hypothetical 154

0.090 0.000 0.000 0.000 0.091 0.001 0.000 0.000 0.091 0.002

0.001 0.001 0.090 0.000 0.003 0.000 0.000 0.002 0.000 0.000

0.006 0.003 0.007 0.007 0.002 0.002 0.001 0.001 0.000 0.091

0.000 0.091 0.000 0.001 0.000 0.000 0.091 0.000 0.091 0.000

0.001 0.000 0.001 0.000 0.004 0.008 0.006 0.007 0.001 0.001

0.000 0.000 0.000 0.000 0.000 0.000 0.091 0.000 0.000 0.001

0.001 0.001 0.000 0.001 0.000 0.000 0.091 0.000 0.007 0.004

0.008 0.005

>Hypothetical 155

0.003 0.000 0.090 0.090 0.000 0.090 0.000 0.001 0.000 0.001

0.000 0.001 0.000 0.001 0.001 0.000 0.001 0.000 0.000 0.000

0.006 0.005 0.004 0.008 0.000 0.000 0.000 0.090 0.000 0.001

0.001 0.090 0.001 0.090 0.000 0.000 0.001 0.090 0.003 0.000

0.000 0.000 0.000 0.000 0.006 0.006 0.006 0.007 0.001 0.000

0.000 0.001 0.000 0.001 0.001 0.000 0.090 0.001 0.000 0.000

0.090 0.000 0.090 0.000 0.001 0.000 0.000 0.000 0.006 0.006

0.005 0.007

>Hypothetical 156

0.000 0.000 0.000 0.000 0.071 0.000 0.071 0.000 0.000 0.001

0.000 0.000 0.000 0.071 0.001 0.001 0.000 0.000 0.071 0.000

0.005 0.005 0.004 0.005 0.001 0.000 0.001 0.000 0.001 0.071

0.000 0.001 0.071 0.001 0.000 0.001 0.001 0.000 0.071 0.071

0.000 0.000 0.071 0.000 0.006 0.004 0.003 0.004 0.000 0.000

0.000 0.000 0.071 0.071 0.000 0.001 0.000 0.071 0.000 0.000

0.001 0.001 0.000 0.000 0.000 0.071 0.000 0.000 0.005 0.006

0.005 0.003

>Hypothetical 157

0.002 0.000 0.083 0.083 0.000 0.001 0.001 0.000 0.001 0.001

0.000 0.083 0.083 0.000 0.002 0.001 0.083 0.001 0.000 0.000

0.006 0.004 0.004 0.008 0.001 0.000 0.001 0.000 0.000 0.000

0.001 0.083 0.001 0.000 0.000 0.000 0.001 0.001 0.083 0.001

0.001 0.000 0.000 0.000 0.006 0.006 0.005 0.004 0.000 0.001

0.083 0.000 0.000 0.083 0.083 0.001 0.083 0.000 0.000 0.000

0.002 0.000 0.001 0.000 0.001 0.000 0.000 0.000 0.006 0.008

0.004 0.005

>Hypothetical 158

0.001 0.001 0.000 0.001 0.001 0.125 0.000 0.000 0.000 0.001

0.000 0.000 0.124 0.125 0.001 0.002 0.000 0.000 0.000 0.000

0.010 0.009 0.011 0.006 0.001 0.002 0.001 0.001 0.000 0.124

0.000 0.000 0.002 0.000 0.000 0.000 0.001 0.000 0.124 0.002

0.000 0.001 0.000 0.000 0.009 0.009 0.006 0.009 0.123 0.000

0.000 0.000 0.000 0.124 0.000 0.000 0.001 0.001 0.000 0.000

0.001 0.001 0.001 0.003 0.001 0.004 0.000 0.000 0.010 0.010

0.004 0.008

>Hypothetical 159

0.001 0.000 0.000 0.000 0.001 0.001 0.001 0.000 0.001 0.000

0.000 0.001 0.143 0.001 0.001 0.001 0.142 0.000 0.001 0.000

0.007 0.011 0.012 0.010 0.002 0.000 0.000 0.142 0.001 0.001

0.001 0.002 0.002 0.000 0.001 0.000 0.000 0.000 0.001 0.001

0.143 0.000 0.000 0.000 0.009 0.013 0.009 0.005 0.001 0.000

0.000 0.000 0.001 0.000 0.142 0.000 0.001 0.000 0.001 0.000

0.001 0.000 0.001 0.000 0.143 0.001 0.001 0.000 0.010 0.010

0.011 0.010

>Hypothetical 160

0.001 0.000 0.071 0.071 0.000 0.001 0.001 0.001 0.001 0.000

0.001 0.000 0.001 0.000 0.071 0.001 0.071 0.071 0.001 0.000

0.004 0.006 0.003 0.004 0.000 0.000 0.071 0.071 0.000 0.001

0.000 0.000 0.001 0.071 0.000 0.000 0.001 0.000 0.001 0.071

0.071 0.000 0.000 0.000 0.005 0.006 0.004 0.005 0.000 0.071

0.000 0.000 0.000 0.001 0.001 0.000 0.001 0.000 0.000 0.001

0.000 0.001 0.071 0.000 0.071 0.000 0.001 0.000 0.002 0.004

0.007 0.007

>Hypothetical 161

0.001 0.000 0.001 0.083 0.000 0.083 0.083 0.000 0.001 0.083

0.000 0.001 0.000 0.001 0.001 0.001 0.002 0.083 0.000 0.001

0.006 0.006 0.005 0.004 0.001 0.000 0.000 0.001 0.083 0.000

0.000 0.001 0.000 0.083 0.000 0.000 0.001 0.001 0.000 0.001

0.001 0.000 0.000 0.000 0.006 0.005 0.005 0.006 0.083 0.083

0.001 0.001 0.000 0.083 0.001 0.000 0.001 0.001 0.000 0.001

0.001 0.001 0.001 0.001 0.000 0.000 0.000 0.083 0.005 0.006

0.005 0.005

>Hypothetical 162

0.001 0.000 0.000 0.001 0.083 0.001 0.083 0.001 0.000 0.000

0.000 0.001 0.001 0.001 0.001 0.001 0.002 0.000 0.000 0.000

0.004 0.003 0.005 0.009 0.083 0.000 0.001 0.001 0.000 0.083

0.000 0.000 0.001 0.001 0.000 0.000 0.000 0.000 0.002 0.083

0.083 0.000 0.000 0.000 0.005 0.008 0.005 0.005 0.000 0.000

0.001 0.001 0.083 0.083 0.000 0.083 0.083 0.000 0.000 0.001

0.000 0.083 0.001 0.001 0.002 0.001 0.000 0.001 0.007 0.003

0.005 0.006

>Hypothetical 163

0.110 0.000 0.000 0.001 0.111 0.000 0.000 0.000 0.000 0.111

0.000 0.000 0.002 0.000 0.111 0.003 0.003 0.001 0.000 0.001

0.006 0.007 0.006 0.008 0.111 0.000 0.000 0.001 0.111 0.110

0.000 0.001 0.000 0.000 0.000 0.001 0.003 0.000 0.002 0.001

0.000 0.000 0.000 0.000 0.011 0.002 0.009 0.008 0.000 0.000

0.000 0.001 0.000 0.000 0.001 0.001 0.000 0.001 0.000 0.001

0.000 0.111 0.001 0.001 0.000 0.001 0.000 0.000 0.006 0.008

0.010 0.007

>Hypothetical 164

0.200 0.000 0.000 0.000 0.000 0.001 0.000 0.000 0.000 0.000

0.000 0.002 0.008 0.000 0.002 0.002 0.002 0.000 0.000 0.000

0.017 0.006 0.017 0.011 0.002 0.000 0.000 0.000 0.000 0.198

0.002 0.002 0.000 0.199 0.000 0.000 0.001 0.002 0.005 0.000

0.000 0.001 0.000 0.000 0.015 0.010 0.017 0.013 0.002 0.000

0.000 0.002 0.000 0.198 0.000 0.000 0.000 0.000 0.000 0.002

0.002 0.000 0.002 0.002 0.002 0.000 0.000 0.000 0.009 0.019

0.013 0.013

>Hypothetical 165

0.017 0.000 0.000 0.000 0.006 0.000 0.000 0.000 0.000 0.000

0.000 0.006 0.017 0.006 0.011 0.000 0.011 0.000 0.000 0.000

0.056 0.092 0.061 0.051 0.006 0.000 0.000 0.000 0.000 0.006

0.000 0.000 0.000 0.006 0.000 0.000 0.034 0.000 0.011 0.006

0.006 0.000 0.000 0.000 0.067 0.075 0.055 0.064 0.009 0.000

0.005 0.000 0.005 0.000 0.005 0.000 0.000 0.000 0.000 0.000

0.014 0.005 0.014 0.000 0.000 0.000 0.000 0.000 0.068 0.082

0.069 0.058

>Hypothetical 166

0.002 0.000 0.000 0.000 0.002 0.091 0.091 0.000 0.091 0.000

0.000 0.001 0.001 0.001 0.001 0.000 0.002 0.000 0.090 0.000

0.005 0.008 0.004 0.005 0.000 0.000 0.000 0.000 0.000 0.090

0.000 0.000 0.000 0.001 0.000 0.000 0.005 0.000 0.001 0.002

0.001 0.000 0.000 0.000 0.006 0.007 0.004 0.007 0.091 0.091

0.001 0.091 0.001 0.091 0.001 0.000 0.000 0.091 0.000 0.000

0.001 0.001 0.002 0.000 0.000 0.000 0.000 0.000 0.006 0.009

0.006 0.005

>Hypothetical 167

0.001 0.000 0.000 0.000 0.001 0.111 0.004 0.000 0.001 0.002

0.000 0.000 0.001 0.111 0.110 0.111 0.001 0.000 0.000 0.000

0.008 0.008 0.005 0.008 0.110 0.000 0.000 0.000 0.002 0.111

0.001 0.000 0.000 0.001 0.000 0.000 0.001 0.111 0.000 0.000

0.003 0.000 0.000 0.000 0.007 0.010 0.008 0.006 0.000 0.111

0.001 0.000 0.000 0.001 0.000 0.000 0.000 0.001 0.000 0.000

0.001 0.001 0.001 0.003 0.001 0.000 0.000 0.000 0.008 0.006

0.004 0.009

>Hypothetical 168

0.000 0.000 0.000 0.000 0.000 0.077 0.000 0.000 0.077 0.001

0.000 0.077 0.002 0.000 0.000 0.001 0.002 0.077 0.000 0.000

0.006 0.004 0.005 0.005 0.001 0.000 0.000 0.000 0.001 0.001

0.000 0.000 0.000 0.003 0.000 0.077 0.003 0.001 0.077 0.001

0.077 0.077 0.000 0.000 0.003 0.003 0.004 0.005 0.001 0.077

0.000 0.000 0.000 0.077 0.001 0.000 0.001 0.001 0.000 0.000

0.001 0.077 0.000 0.001 0.077 0.000 0.000 0.001 0.006 0.005

0.005 0.004

>Hypothetical 169

0.000 0.000 0.001 0.076 0.076 0.002 0.000 0.000 0.000 0.001

0.000 0.000 0.001 0.000 0.001 0.076 0.000 0.000 0.000 0.000

0.006 0.007 0.004 0.004 0.005 0.000 0.000 0.076 0.000 0.001

0.076 0.000 0.076 0.001 0.000 0.000 0.000 0.076 0.001 0.001

0.001 0.000 0.001 0.000 0.006 0.008 0.002 0.002 0.002 0.000

0.000 0.077 0.000 0.000 0.001 0.077 0.077 0.076 0.000 0.000

0.002 0.000 0.002 0.000 0.000 0.077 0.000 0.000 0.004 0.005

0.004 0.008

>Hypothetical 170

0.000 0.000 0.000 0.053 0.000 0.001 0.000 0.053 0.053 0.053

0.053 0.000 0.000 0.000 0.001 0.000 0.000 0.000 0.000 0.000

0.003 0.004 0.004 0.005 0.002 0.053 0.053 0.001 0.000 0.002

0.001 0.000 0.053 0.000 0.000 0.000 0.053 0.001 0.053 0.053

0.000 0.053 0.053 0.000 0.003 0.004 0.002 0.003 0.000 0.000

0.053 0.053 0.000 0.053 0.000 0.000 0.000 0.000 0.000 0.053

0.000 0.000 0.000 0.000 0.000 0.053 0.001 0.000 0.002 0.004

0.005 0.005

>Hypothetical 171

0.001 0.001 0.000 0.000 0.000 0.000 0.001 0.000 0.077 0.002

0.000 0.000 0.002 0.001 0.077 0.000 0.000 0.077 0.077 0.077

0.005 0.005 0.004 0.005 0.001 0.001 0.077 0.000 0.000 0.077

0.001 0.077 0.000 0.000 0.000 0.000 0.001 0.000 0.000 0.002

0.001 0.077 0.000 0.000 0.004 0.005 0.004 0.006 0.001 0.077

0.000 0.000 0.000 0.000 0.000 0.000 0.000 0.000 0.000 0.000

0.003 0.077 0.000 0.000 0.001 0.077 0.000 0.000 0.004 0.005

0.006 0.006

>Hypothetical 172

0.000 0.000 0.000 0.000 0.000 0.083 0.001 0.000 0.000 0.001

0.083 0.000 0.003 0.083 0.001 0.001 0.083 0.001 0.000 0.000

0.006 0.006 0.002 0.006 0.000 0.083 0.001 0.000 0.001 0.083

0.000 0.000 0.000 0.001 0.000 0.000 0.002 0.001 0.000 0.001

0.001 0.000 0.000 0.000 0.005 0.005 0.008 0.003 0.083 0.000

0.083 0.000 0.000 0.083 0.083 0.000 0.000 0.002 0.000 0.000

0.003 0.083 0.001 0.002 0.001 0.000 0.000 0.000 0.004 0.004

0.007 0.006

>Hypothetical 173

0.001 0.000 0.000 0.000 0.000 0.000 0.000 0.000 0.091 0.091

0.000 0.091 0.090 0.000 0.002 0.000 0.000 0.000 0.091 0.000

0.004 0.006 0.008 0.008 0.001 0.000 0.000 0.000 0.000 0.001

0.000 0.000 0.091 0.000 0.091 0.000 0.091 0.000 0.001 0.001

0.001 0.001 0.091 0.000 0.006 0.005 0.007 0.006 0.090 0.000

0.000 0.001 0.000 0.002 0.000 0.000 0.000 0.001 0.000 0.000

0.000 0.001 0.001 0.001 0.000 0.000 0.000 0.000 0.007 0.005

0.006 0.007

>Hypothetical 174

0.001 0.000 0.100 0.000 0.000 0.001 0.000 0.000 0.100 0.000

0.000 0.000 0.001 0.001 0.001 0.100 0.000 0.100 0.001 0.000

0.007 0.008 0.007 0.007 0.001 0.001 0.000 0.001 0.100 0.001

0.000 0.000 0.000 0.001 0.000 0.001 0.001 0.000 0.000 0.100

0.100 0.001 0.100 0.000 0.007 0.007 0.005 0.008 0.001 0.000

0.000 0.100 0.000 0.001 0.000 0.000 0.000 0.001 0.000 0.000

0.001 0.001 0.002 0.001 0.001 0.000 0.000 0.000 0.007 0.007

0.005 0.006

>Hypothetical 175

0.004 0.000 0.000 0.005 0.000 0.499 0.003 0.000 0.000 0.003

0.000 0.000 0.001 0.000 0.004 0.003 0.000 0.000 0.005 0.000

0.042 0.025 0.038 0.035 0.002 0.000 0.000 0.000 0.000 0.005

0.000 0.000 0.003 0.003 0.003 0.000 0.004 0.000 0.002 0.003

0.000 0.003 0.003 0.003 0.030 0.031 0.037 0.033 0.004 0.000

0.000 0.000 0.000 0.008 0.000 0.000 0.000 0.009 0.000 0.000

0.003 0.000 0.004 0.008 0.004 0.000 0.000 0.000 0.026 0.033

0.030 0.040

>Hypothetical 176

0.002 0.053 0.000 0.000 0.000 0.001 0.053 0.053 0.000 0.001

0.001 0.000 0.001 0.053 0.053 0.001 0.052 0.053 0.001 0.000

0.003 0.003 0.004 0.003 0.001 0.000 0.000 0.000 0.053 0.002

0.053 0.000 0.000 0.003 0.000 0.000 0.052 0.002 0.000 0.052

0.053 0.000 0.001 0.000 0.002 0.002 0.002 0.003 0.000 0.000

0.000 0.001 0.000 0.000 0.052 0.000 0.000 0.053 0.000 0.000

0.052 0.053 0.000 0.001 0.053 0.053 0.001 0.000 0.004 0.003

0.003 0.005

>Hypothetical 177

0.001 0.003 0.000 0.000 0.000 0.002 0.003 0.000 0.000 0.165

0.000 0.000 0.002 0.000 0.000 0.000 0.000 0.001 0.000 0.000

0.008 0.011 0.012 0.013 0.005 0.000 0.001 0.000 0.001 0.000

0.000 0.000 0.000 0.165 0.000 0.000 0.002 0.166 0.002 0.165

0.002 0.000 0.000 0.000 0.013 0.011 0.012 0.010 0.001 0.000

0.000 0.000 0.000 0.001 0.000 0.000 0.000 0.003 0.000 0.000

0.000 0.001 0.002 0.166 0.001 0.000 0.000 0.000 0.011 0.010

0.012 0.014

>Hypothetical 178

0.167 0.000 0.000 0.000 0.000 0.166 0.000 0.000 0.000 0.004

0.000 0.000 0.002 0.001 0.003 0.003 0.000 0.000 0.000 0.000

0.010 0.011 0.010 0.012 0.001 0.004 0.000 0.000 0.002 0.001

0.002 0.000 0.000 0.166 0.000 0.000 0.004 0.000 0.166 0.002

0.000 0.003 0.000 0.000 0.009 0.010 0.012 0.009 0.004 0.000

0.000 0.002 0.002 0.165 0.000 0.000 0.000 0.000 0.000 0.000

0.003 0.002 0.000 0.000 0.002 0.000 0.000 0.000 0.010 0.010

0.011 0.011

>Hypothetical 179

0.058 0.058 0.000 0.001 0.058 0.002 0.001 0.001 0.000 0.058

0.000 0.000 0.058 0.000 0.000 0.006 0.001 0.058 0.000 0.000

0.003 0.003 0.003 0.002 0.058 0.000 0.000 0.000 0.058 0.002

0.000 0.002 0.000 0.001 0.000 0.000 0.002 0.000 0.058 0.002

0.000 0.008 0.058 0.000 0.002 0.001 0.003 0.001 0.058 0.000

0.000 0.002 0.002 0.058 0.001 0.000 0.000 0.058 0.000 0.000

0.058 0.000 0.000 0.058 0.000 0.058 0.001 0.001 0.005 0.002

0.005 0.004

>Hypothetical 180

0.110 0.001 0.001 0.000 0.001 0.111 0.000 0.000 0.001 0.001

0.000 0.000 0.000 0.111 0.000 0.004 0.000 0.001 0.000 0.000

0.007 0.007 0.008 0.007 0.000 0.000 0.000 0.000 0.001 0.111

0.001 0.000 0.000 0.001 0.000 0.001 0.111 0.000 0.000 0.001

0.001 0.003 0.111 0.000 0.007 0.004 0.012 0.005 0.110 0.001

0.000 0.001 0.002 0.110 0.000 0.000 0.000 0.002 0.000 0.000

0.000 0.001 0.000 0.000 0.001 0.001 0.001 0.000 0.009 0.007

0.007 0.007

>Hypothetical 181

0.001 0.000 0.077 0.077 0.000 0.001 0.001 0.000 0.001 0.000

0.000 0.001 0.000 0.000 0.000 0.001 0.000 0.077 0.000 0.000

0.006 0.004 0.006 0.003 0.000 0.077 0.000 0.077 0.001 0.001

0.077 0.001 0.000 0.076 0.000 0.001 0.001 0.001 0.001 0.077

0.000 0.001 0.000 0.000 0.004 0.007 0.007 0.003 0.000 0.000

0.000 0.001 0.001 0.001 0.000 0.001 0.000 0.077 0.000 0.000

0.000 0.000 0.000 0.077 0.077 0.000 0.000 0.077 0.007 0.007

0.004 0.004

>Hypothetical 182

0.000 0.000 0.071 0.000 0.001 0.000 0.000 0.000 0.001 0.000

0.000 0.000 0.071 0.000 0.000 0.000 0.071 0.071 0.000 0.000

0.006 0.006 0.004 0.004 0.001 0.071 0.000 0.071 0.000 0.001

0.001 0.001 0.000 0.071 0.000 0.001 0.000 0.000 0.001 0.002

0.001 0.000 0.000 0.000 0.006 0.004 0.005 0.002 0.071 0.000

0.000 0.000 0.000 0.000 0.071 0.000 0.001 0.071 0.000 0.071

0.001 0.000 0.001 0.071 0.000 0.000 0.000 0.071 0.004 0.007

0.006 0.003

>Hypothetical 183

0.000 0.000 0.001 0.001 0.091 0.091 0.000 0.001 0.001 0.091

0.091 0.000 0.002 0.000 0.001 0.000 0.001 0.001 0.000 0.091

0.005 0.007 0.005 0.006 0.000 0.000 0.000 0.000 0.091 0.091

0.000 0.000 0.001 0.001 0.000 0.000 0.001 0.000 0.091 0.000

0.000 0.000 0.000 0.000 0.005 0.006 0.007 0.006 0.001 0.000

0.000 0.000 0.000 0.091 0.001 0.000 0.001 0.001 0.000 0.001

0.001 0.000 0.001 0.001 0.001 0.091 0.000 0.001 0.007 0.006

0.006 0.005

>Hypothetical 184

0.058 0.059 0.059 0.059 0.000 0.001 0.001 0.001 0.000 0.000

0.000 0.000 0.001 0.000 0.000 0.000 0.058 0.001 0.000 0.000

0.004 0.004 0.004 0.003 0.001 0.000 0.000 0.000 0.000 0.059

0.001 0.001 0.000 0.001 0.059 0.001 0.059 0.059 0.058 0.000

0.000 0.059 0.000 0.001 0.004 0.003 0.005 0.003 0.000 0.059

0.000 0.000 0.000 0.059 0.059 0.000 0.000 0.000 0.000 0.000

0.000 0.058 0.001 0.000 0.001 0.059 0.000 0.000 0.003 0.005

0.003 0.005

>Hypothetical 185

0.001 0.100 0.000 0.001 0.000 0.100 0.001 0.000 0.001 0.002

0.000 0.000 0.000 0.100 0.100 0.003 0.000 0.000 0.100 0.001

0.006 0.007 0.006 0.005 0.100 0.000 0.000 0.000 0.001 0.100

0.000 0.001 0.000 0.000 0.000 0.001 0.000 0.001 0.002 0.000

0.001 0.000 0.100 0.001 0.008 0.008 0.004 0.008 0.000 0.000

0.000 0.000 0.001 0.001 0.000 0.001 0.001 0.100 0.000 0.000

0.000 0.000 0.001 0.001 0.000 0.001 0.000 0.000 0.007 0.005

0.008 0.007

>Hypothetical 186

0.062 0.000 0.000 0.000 0.001 0.001 0.001 0.000 0.001 0.062

0.000 0.062 0.000 0.000 0.000 0.001 0.062 0.001 0.000 0.000

0.003 0.004 0.005 0.005 0.001 0.000 0.000 0.001 0.000 0.000

0.001 0.000 0.000 0.001 0.001 0.000 0.062 0.062 0.000 0.001

0.062 0.062 0.062 0.001 0.003 0.006 0.004 0.003 0.062 0.000

0.000 0.001 0.001 0.062 0.062 0.001 0.062 0.000 0.000 0.000

0.062 0.000 0.001 0.001 0.001 0.062 0.000 0.000 0.006 0.005

0.001 0.006

>Hypothetical 187

0.002 0.000 0.000 0.000 0.001 0.090 0.000 0.001 0.000 0.091

0.000 0.000 0.001 0.001 0.000 0.006 0.000 0.000 0.091 0.000

0.004 0.005 0.005 0.006 0.001 0.000 0.001 0.000 0.001 0.091

0.001 0.000 0.000 0.000 0.000 0.000 0.002 0.000 0.000 0.091

0.000 0.000 0.091 0.000 0.006 0.006 0.005 0.008 0.091 0.091

0.000 0.091 0.002 0.001 0.000 0.001 0.001 0.002 0.000 0.000

0.000 0.000 0.001 0.000 0.002 0.091 0.000 0.000 0.004 0.005

0.008 0.007

>Hypothetical 188

0.071 0.000 0.071 0.071 0.000 0.002 0.000 0.000 0.000 0.000

0.000 0.000 0.002 0.001 0.002 0.001 0.000 0.071 0.000 0.071

0.004 0.007 0.005 0.003 0.001 0.000 0.001 0.071 0.000 0.001

0.000 0.000 0.001 0.000 0.000 0.001 0.001 0.000 0.071 0.071

0.071 0.000 0.000 0.000 0.005 0.004 0.005 0.006 0.071 0.071

0.000 0.000 0.001 0.071 0.000 0.000 0.000 0.071 0.000 0.000

0.000 0.001 0.001 0.001 0.002 0.000 0.000 0.000 0.003 0.006

0.006 0.003

>Hypothetical 189

0.165 0.001 0.000 0.000 0.002 0.002 0.166 0.005 0.000 0.003

0.000 0.000 0.001 0.000 0.000 0.000 0.003 0.003 0.002 0.000

0.012 0.008 0.008 0.008 0.002 0.000 0.000 0.000 0.002 0.002

0.002 0.000 0.000 0.001 0.000 0.000 0.001 0.002 0.166 0.010

0.166 0.002 0.166 0.000 0.009 0.014 0.005 0.008 0.000 0.001

0.000 0.000 0.001 0.001 0.002 0.000 0.000 0.000 0.001 0.001

0.001 0.000 0.001 0.001 0.000 0.000 0.000 0.000 0.007 0.014

0.011 0.015

>Hypothetical 190

0.001 0.000 0.000 0.000 0.001 0.071 0.000 0.071 0.071 0.001

0.000 0.001 0.071 0.000 0.000 0.004 0.002 0.002 0.000 0.000

0.004 0.004 0.003 0.003 0.000 0.071 0.000 0.000 0.071 0.071

0.000 0.000 0.000 0.000 0.000 0.000 0.001 0.000 0.001 0.001

0.000 0.000 0.000 0.000 0.004 0.006 0.005 0.004 0.000 0.001

0.071 0.071 0.000 0.071 0.001 0.071 0.000 0.001 0.000 0.000

0.071 0.001 0.000 0.071 0.001 0.001 0.000 0.000 0.005 0.004

0.004 0.006

>Hypothetical 191

0.002 0.000 0.082 0.001 0.083 0.083 0.000 0.001 0.000 0.003

0.000 0.001 0.000 0.000 0.001 0.083 0.000 0.000 0.083 0.083

0.007 0.005 0.003 0.006 0.001 0.000 0.000 0.000 0.000 0.083

0.000 0.083 0.000 0.002 0.000 0.001 0.083 0.000 0.000 0.001

0.000 0.000 0.000 0.000 0.006 0.006 0.005 0.006 0.000 0.000

0.000 0.000 0.083 0.000 0.001 0.000 0.001 0.082 0.000 0.000

0.001 0.000 0.001 0.001 0.001 0.001 0.000 0.000 0.007 0.007

0.003 0.006

>Hypothetical 192

0.001 0.000 0.001 0.000 0.000 0.090 0.000 0.002 0.000 0.003

0.000 0.001 0.000 0.000 0.090 0.002 0.091 0.091 0.000 0.001

0.005 0.005 0.005 0.005 0.001 0.000 0.090 0.090 0.001 0.000

0.000 0.000 0.000 0.000 0.000 0.000 0.000 0.000 0.000 0.000

0.002 0.000 0.002 0.000 0.008 0.004 0.005 0.007 0.090 0.091

0.000 0.001 0.000 0.002 0.002 0.000 0.001 0.001 0.000 0.000

0.001 0.000 0.000 0.091 0.091 0.001 0.000 0.000 0.004 0.002

0.007 0.011

>Hypothetical 193

0.000 0.000 0.000 0.000 0.001 0.000 0.000 0.001 0.001 0.001

0.000 0.066 0.066 0.066 0.001 0.001 0.000 0.066 0.000 0.000

0.003 0.005 0.007 0.004 0.001 0.000 0.000 0.001 0.001 0.066

0.000 0.066 0.066 0.000 0.000 0.066 0.000 0.066 0.001 0.000

0.000 0.000 0.066 0.002 0.004 0.004 0.004 0.006 0.066 0.000

0.001 0.000 0.001 0.000 0.000 0.001 0.001 0.001 0.000 0.000

0.001 0.000 0.000 0.066 0.067 0.000 0.066 0.000 0.004 0.004

0.005 0.005

>Hypothetical 194

0.071 0.001 0.000 0.000 0.000 0.001 0.000 0.001 0.000 0.071

0.000 0.001 0.000 0.071 0.001 0.071 0.001 0.000 0.000 0.000

0.007 0.004 0.003 0.006 0.000 0.001 0.000 0.001 0.001 0.001

0.000 0.000 0.000 0.001 0.001 0.000 0.000 0.071 0.071 0.071

0.000 0.001 0.071 0.000 0.004 0.005 0.004 0.005 0.001 0.001

0.000 0.000 0.001 0.071 0.000 0.000 0.001 0.071 0.000 0.001

0.071 0.000 0.001 0.000 0.071 0.071 0.000 0.001 0.005 0.006

0.006 0.005

>Hypothetical 195

0.077 0.077 0.001 0.000 0.001 0.001 0.001 0.000 0.077 0.001

0.000 0.000 0.000 0.077 0.076 0.001 0.077 0.002 0.000 0.001

0.004 0.005 0.005 0.005 0.076 0.000 0.000 0.000 0.001 0.000

0.000 0.001 0.000 0.077 0.001 0.077 0.000 0.000 0.000 0.002

0.001 0.000 0.000 0.000 0.004 0.005 0.005 0.006 0.000 0.001

0.077 0.077 0.001 0.001 0.000 0.000 0.000 0.001 0.000 0.000

0.076 0.000 0.001 0.000 0.000 0.000 0.001 0.000 0.006 0.006

0.005 0.005

>Hypothetical 196

0.002 0.000 0.000 0.000 0.000 0.001 0.125 0.000 0.125 0.000

0.000 0.000 0.001 0.000 0.001 0.003 0.000 0.000 0.001 0.000

0.010 0.008 0.007 0.009 0.001 0.000 0.001 0.001 0.000 0.003

0.000 0.000 0.000 0.002 0.000 0.000 0.005 0.000 0.000 0.125

0.125 0.000 0.125 0.000 0.008 0.007 0.007 0.008 0.001 0.125

0.000 0.125 0.000 0.001 0.000 0.001 0.000 0.005 0.000 0.000

0.001 0.000 0.001 0.001 0.000 0.001 0.000 0.000 0.005 0.008

0.009 0.009

>Hypothetical 197

0.002 0.000 0.000 0.000 0.000 0.000 0.000 0.000 0.000 0.100

0.000 0.000 0.100 0.100 0.001 0.008 0.000 0.000 0.001 0.000

0.008 0.005 0.004 0.006 0.000 0.000 0.000 0.000 0.000 0.001

0.000 0.000 0.000 0.001 0.000 0.000 0.006 0.000 0.001 0.100

0.000 0.000 0.100 0.000 0.010 0.002 0.007 0.005 0.099 0.100

0.000 0.100 0.001 0.001 0.000 0.000 0.000 0.005 0.000 0.000

0.001 0.099 0.001 0.000 0.000 0.003 0.000 0.000 0.005 0.006

0.006 0.008

>Hypothetical 198

0.066 0.000 0.000 0.000 0.001 0.001 0.001 0.000 0.000 0.004

0.000 0.000 0.000 0.066 0.066 0.066 0.000 0.004 0.000 0.001

0.004 0.003 0.003 0.003 0.000 0.067 0.000 0.000 0.000 0.067

0.066 0.001 0.000 0.067 0.001 0.000 0.001 0.000 0.066 0.012

0.000 0.001 0.000 0.000 0.000 0.002 0.002 0.003 0.066 0.000

0.000 0.066 0.066 0.000 0.000 0.066 0.000 0.001 0.000 0.000

0.002 0.000 0.002 0.066 0.000 0.000 0.000 0.000 0.007 0.002

0.007 0.003

>Hypothetical 199

0.001 0.062 0.000 0.000 0.000 0.000 0.000 0.000 0.000 0.062

0.000 0.001 0.000 0.001 0.000 0.062 0.062 0.001 0.000 0.062

0.004 0.005 0.003 0.004 0.000 0.062 0.000 0.000 0.001 0.062

0.062 0.000 0.000 0.002 0.001 0.001 0.062 0.062 0.000 0.001

0.000 0.000 0.000 0.000 0.004 0.004 0.005 0.003 0.062 0.001

0.000 0.000 0.000 0.000 0.000 0.000 0.000 0.062 0.000 0.062

0.001 0.062 0.000 0.000 0.000 0.062 0.000 0.000 0.006 0.002

0.005 0.005

>Hypothetical 200

0.000 0.076 0.001 0.002 0.000 0.076 0.000 0.000 0.001 0.002

0.000 0.000 0.076 0.001 0.001 0.002 0.000 0.000 0.000 0.000

0.006 0.003 0.005 0.004 0.001 0.001 0.000 0.076 0.000 0.077

0.000 0.000 0.000 0.076 0.000 0.001 0.076 0.000 0.002 0.001

0.001 0.000 0.076 0.000 0.006 0.011 0.002 0.003 0.000 0.000

0.000 0.002 0.000 0.076 0.000 0.000 0.076 0.076 0.000 0.000

0.000 0.000 0.002 0.076 0.001 0.000 0.000 0.000 0.006 0.009

0.006 0.002

>Hypothetical 201

0.000 0.000 0.000 0.001 0.000 0.000 0.000 0.000 0.000 0.000

0.000 0.077 0.077 0.077 0.001 0.077 0.000 0.000 0.000 0.000

0.006 0.007 0.006 0.002 0.001 0.000 0.001 0.001 0.000 0.077

0.000 0.000 0.001 0.001 0.000 0.000 0.077 0.077 0.000 0.001

0.001 0.000 0.000 0.000 0.005 0.003 0.005 0.005 0.001 0.001

0.077 0.077 0.000 0.077 0.000 0.000 0.000 0.000 0.000 0.000

0.077 0.077 0.002 0.001 0.001 0.000 0.000 0.000 0.006 0.009

0.004 0.003

>Hypothetical 202

0.000 0.001 0.001 0.001 0.000 0.071 0.071 0.001 0.000 0.071

0.000 0.001 0.071 0.000 0.000 0.000 0.001 0.000 0.000 0.071

0.004 0.007 0.004 0.004 0.070 0.000 0.000 0.000 0.000 0.000

0.001 0.071 0.001 0.000 0.001 0.000 0.000 0.000 0.001 0.071

0.001 0.001 0.000 0.071 0.006 0.005 0.003 0.006 0.000 0.000

0.001 0.071 0.000 0.001 0.000 0.000 0.001 0.000 0.001 0.000

0.071 0.001 0.000 0.071 0.071 0.000 0.001 0.000 0.003 0.006

0.005 0.006

>Hypothetical 203

0.001 0.062 0.000 0.000 0.062 0.001 0.062 0.000 0.000 0.001

0.000 0.000 0.000 0.062 0.000 0.062 0.000 0.000 0.000 0.000

0.002 0.005 0.005 0.005 0.062 0.000 0.000 0.001 0.000 0.000

0.000 0.000 0.000 0.001 0.000 0.062 0.001 0.062 0.000 0.000

0.001 0.062 0.000 0.062 0.005 0.004 0.003 0.004 0.000 0.000

0.000 0.000 0.062 0.062 0.000 0.000 0.000 0.062 0.000 0.000

0.000 0.000 0.002 0.000 0.000 0.062 0.000 0.062 0.004 0.004

0.004 0.004

>Hypothetical 204

0.002 0.000 0.001 0.001 0.000 0.002 0.000 0.000 0.000 0.003

0.000 0.001 0.001 0.000 0.001 0.000 0.001 0.000 0.000 0.000

0.007 0.009 0.011 0.010 0.143 0.000 0.001 0.002 0.000 0.143

0.000 0.000 0.001 0.001 0.000 0.000 0.002 0.143 0.001 0.002

0.002 0.000 0.000 0.000 0.011 0.007 0.008 0.011 0.001 0.001

0.000 0.143 0.000 0.143 0.001 0.000 0.001 0.000 0.000 0.000

0.002 0.001 0.003 0.143 0.001 0.000 0.000 0.000 0.008 0.008

0.008 0.013

>Hypothetical 205

0.090 0.001 0.000 0.000 0.000 0.000 0.002 0.000 0.001 0.089

0.000 0.000 0.000 0.001 0.002 0.090 0.001 0.000 0.000 0.000

0.004 0.005 0.008 0.010 0.002 0.090 0.001 0.000 0.090 0.001

0.000 0.000 0.000 0.003 0.000 0.001 0.090 0.000 0.001 0.000

0.002 0.000 0.090 0.000 0.004 0.006 0.005 0.006 0.090 0.000

0.001 0.001 0.000 0.091 0.000 0.000 0.000 0.001 0.000 0.000

0.003 0.000 0.091 0.002 0.002 0.000 0.000 0.000 0.007 0.003

0.005 0.007

>Hypothetical 206

0.000 0.000 0.001 0.001 0.000 0.001 0.091 0.000 0.001 0.091

0.000 0.000 0.000 0.001 0.001 0.001 0.000 0.001 0.000 0.001

0.004 0.005 0.006 0.007 0.001 0.000 0.091 0.001 0.000 0.091

0.091 0.000 0.001 0.001 0.000 0.000 0.091 0.091 0.001 0.001

0.001 0.001 0.000 0.000 0.005 0.006 0.007 0.006 0.000 0.000

0.000 0.000 0.000 0.000 0.000 0.091 0.001 0.001 0.000 0.000

0.000 0.000 0.001 0.091 0.091 0.000 0.000 0.000 0.007 0.007

0.005 0.007

>Hypothetical 207

0.001 0.000 0.000 0.001 0.000 0.001 0.000 0.000 0.001 0.002

0.000 0.000 0.143 0.001 0.002 0.003 0.000 0.000 0.143 0.000

0.008 0.010 0.010 0.010 0.143 0.143 0.000 0.001 0.000 0.002

0.000 0.000 0.000 0.001 0.000 0.002 0.002 0.000 0.143 0.002

0.000 0.002 0.000 0.001 0.008 0.009 0.008 0.010 0.001 0.000

0.000 0.001 0.002 0.001 0.000 0.000 0.000 0.001 0.000 0.000

0.001 0.143 0.001 0.001 0.002 0.000 0.000 0.000 0.010 0.011

0.008 0.009

>Hypothetical 208

0.001 0.000 0.000 0.001 0.000 0.001 0.111 0.000 0.001 0.001

0.000 0.000 0.111 0.001 0.003 0.001 0.000 0.000 0.111 0.000

0.007 0.007 0.007 0.008 0.111 0.000 0.000 0.001 0.000 0.002

0.000 0.000 0.111 0.001 0.111 0.002 0.002 0.000 0.111 0.002

0.000 0.002 0.111 0.001 0.007 0.007 0.006 0.007 0.001 0.000

0.000 0.001 0.001 0.001 0.000 0.000 0.000 0.001 0.000 0.000

0.001 0.000 0.001 0.001 0.002 0.000 0.000 0.000 0.007 0.008

0.006 0.008

>Hypothetical 209

0.001 0.000 0.000 0.001 0.000 0.001 0.143 0.000 0.143 0.001

0.000 0.000 0.142 0.001 0.002 0.003 0.000 0.000 0.000 0.000

0.009 0.010 0.010 0.010 0.007 0.000 0.000 0.000 0.000 0.002

0.000 0.000 0.000 0.142 0.000 0.000 0.000 0.000 0.003 0.001

0.142 0.002 0.000 0.000 0.004 0.009 0.010 0.010 0.001 0.143

0.000 0.002 0.000 0.001 0.000 0.000 0.000 0.001 0.000 0.000

0.000 0.001 0.002 0.001 0.000 0.000 0.000 0.000 0.011 0.011

0.008 0.010

>Hypothetical 210

0.001 0.001 0.000 0.001 0.000 0.002 0.001 0.000 0.000 0.000

0.001 0.000 0.091 0.000 0.000 0.000 0.001 0.000 0.091 0.000

0.005 0.006 0.008 0.005 0.001 0.000 0.091 0.000 0.000 0.001

0.000 0.000 0.000 0.000 0.000 0.000 0.091 0.000 0.001 0.000

0.000 0.091 0.000 0.000 0.005 0.007 0.006 0.008 0.000 0.000

0.001 0.091 0.091 0.001 0.001 0.001 0.001 0.091 0.000 0.000

0.000 0.000 0.001 0.091 0.091 0.000 0.000 0.000 0.007 0.009

0.005 0.006

>Hypothetical 211

0.000 0.077 0.000 0.000 0.000 0.077 0.000 0.077 0.000 0.000

0.000 0.000 0.077 0.077 0.002 0.001 0.001 0.077 0.000 0.000

0.006 0.006 0.005 0.004 0.001 0.000 0.000 0.000 0.077 0.001

0.077 0.001 0.000 0.001 0.000 0.000 0.001 0.077 0.001 0.000

0.000 0.000 0.000 0.000 0.004 0.005 0.004 0.006 0.001 0.000

0.000 0.000 0.001 0.077 0.001 0.000 0.000 0.001 0.000 0.077

0.000 0.001 0.000 0.000 0.077 0.000 0.000 0.000 0.006 0.005

0.004 0.006

>Hypothetical 212

0.001 0.071 0.000 0.001 0.001 0.000 0.001 0.071 0.000 0.001

0.000 0.000 0.071 0.001 0.000 0.001 0.071 0.000 0.071 0.000

0.005 0.005 0.004 0.005 0.000 0.000 0.000 0.000 0.001 0.071

0.000 0.000 0.000 0.000 0.000 0.001 0.071 0.000 0.001 0.002

0.001 0.071 0.000 0.000 0.005 0.006 0.004 0.003 0.001 0.000

0.071 0.000 0.000 0.001 0.071 0.001 0.000 0.001 0.071 0.071

0.002 0.000 0.001 0.001 0.001 0.071 0.000 0.000 0.003 0.005

0.004 0.005

>Hypothetical 213

0.000 0.000 0.000 0.001 0.071 0.003 0.000 0.000 0.001 0.001

0.071 0.000 0.001 0.000 0.071 0.001 0.001 0.000 0.000 0.071

0.004 0.004 0.004 0.005 0.000 0.071 0.000 0.001 0.071 0.001

0.071 0.000 0.001 0.071 0.000 0.000 0.001 0.000 0.000 0.000

0.071 0.000 0.000 0.000 0.005 0.003 0.005 0.006 0.071 0.000

0.000 0.000 0.000 0.000 0.000 0.000 0.000 0.071 0.000 0.000

0.000 0.000 0.001 0.071 0.000 0.001 0.000 0.071 0.005 0.004

0.005 0.005

>Hypothetical 214

0.001 0.000 0.000 0.001 0.125 0.001 0.001 0.000 0.000 0.002

0.000 0.001 0.125 0.000 0.002 0.001 0.001 0.001 0.000 0.000

0.007 0.009 0.008 0.008 0.125 0.000 0.125 0.000 0.001 0.004

0.000 0.000 0.001 0.001 0.000 0.000 0.001 0.000 0.001 0.125

0.002 0.000 0.000 0.000 0.007 0.010 0.006 0.008 0.000 0.001

0.000 0.001 0.000 0.001 0.000 0.000 0.001 0.125 0.000 0.000

0.001 0.124 0.001 0.001 0.000 0.000 0.000 0.001 0.010 0.008

0.009 0.009

>Hypothetical 215

0.000 0.000 0.000 0.001 0.001 0.000 0.001 0.000 0.083 0.001

0.000 0.083 0.001 0.000 0.000 0.000 0.083 0.001 0.000 0.083

0.006 0.005 0.005 0.006 0.001 0.000 0.001 0.083 0.001 0.083

0.000 0.001 0.001 0.001 0.000 0.000 0.001 0.083 0.001 0.001

0.000 0.000 0.000 0.000 0.006 0.006 0.003 0.006 0.000 0.000

0.000 0.083 0.001 0.000 0.083 0.000 0.083 0.000 0.000 0.083

0.001 0.001 0.000 0.002 0.000 0.000 0.000 0.000 0.005 0.006

0.004 0.007

>Hypothetical 216

0.000 0.000 0.001 0.000 0.001 0.000 0.000 0.076 0.000 0.001

0.000 0.000 0.001 0.001 0.000 0.077 0.000 0.077 0.000 0.000

0.007 0.003 0.005 0.006 0.000 0.001 0.000 0.001 0.001 0.000

0.077 0.077 0.001 0.000 0.000 0.077 0.000 0.001 0.000 0.003

0.002 0.000 0.000 0.077 0.006 0.004 0.004 0.004 0.000 0.000

0.000 0.001 0.077 0.000 0.000 0.077 0.000 0.000 0.000 0.077

0.001 0.000 0.001 0.002 0.076 0.001 0.000 0.077 0.006 0.003

0.005 0.006

>Hypothetical 217

0.002 0.000 0.000 0.100 0.001 0.001 0.000 0.000 0.000 0.002

0.000 0.000 0.001 0.001 0.001 0.100 0.000 0.000 0.000 0.000

0.004 0.007 0.006 0.009 0.002 0.100 0.099 0.100 0.000 0.000

0.000 0.000 0.100 0.001 0.000 0.000 0.000 0.001 0.001 0.000

0.000 0.000 0.001 0.001 0.009 0.007 0.006 0.007 0.100 0.000

0.000 0.000 0.001 0.002 0.000 0.100 0.000 0.004 0.000 0.000

0.002 0.000 0.100 0.001 0.001 0.001 0.000 0.000 0.005 0.006

0.004 0.008

>Hypothetical 218

0.100 0.001 0.000 0.100 0.001 0.001 0.000 0.000 0.100 0.001

0.000 0.000 0.002 0.000 0.001 0.001 0.001 0.001 0.100 0.000

0.007 0.007 0.004 0.008 0.002 0.000 0.000 0.001 0.001 0.100

0.000 0.000 0.100 0.000 0.000 0.000 0.001 0.001 0.001 0.000

0.000 0.000 0.000 0.000 0.005 0.008 0.007 0.008 0.001 0.100

0.000 0.000 0.000 0.100 0.000 0.000 0.100 0.001 0.000 0.000

0.001 0.001 0.001 0.001 0.000 0.000 0.000 0.000 0.006 0.007

0.008 0.008

>Hypothetical 219

0.001 0.000 0.000 0.000 0.077 0.001 0.000 0.000 0.000 0.000

0.000 0.000 0.001 0.001 0.077 0.003 0.077 0.077 0.001 0.000

0.004 0.004 0.005 0.004 0.001 0.077 0.077 0.000 0.077 0.001

0.000 0.000 0.000 0.000 0.000 0.000 0.000 0.000 0.001 0.000

0.000 0.000 0.000 0.000 0.004 0.006 0.006 0.006 0.001 0.001

0.077 0.001 0.077 0.000 0.000 0.077 0.000 0.001 0.000 0.000

0.077 0.001 0.001 0.077 0.000 0.001 0.000 0.000 0.005 0.004

0.005 0.006

>Hypothetical 220

0.002 0.000 0.000 0.000 0.000 0.124 0.000 0.000 0.000 0.001

0.000 0.000 0.002 0.125 0.001 0.004 0.001 0.000 0.001 0.000

0.009 0.009 0.007 0.006 0.002 0.001 0.000 0.000 0.000 0.001

0.000 0.000 0.000 0.125 0.000 0.000 0.002 0.000 0.002 0.125

0.000 0.000 0.125 0.000 0.009 0.006 0.010 0.009 0.003 0.125

0.000 0.001 0.000 0.001 0.001 0.000 0.000 0.001 0.000 0.000

0.000 0.003 0.001 0.000 0.000 0.124 0.000 0.000 0.005 0.008

0.008 0.011

>Hypothetical 221

0.002 0.001 0.077 0.000 0.000 0.076 0.000 0.077 0.076 0.001

0.000 0.001 0.077 0.000 0.001 0.001 0.001 0.000 0.000 0.001

0.007 0.005 0.004 0.006 0.002 0.001 0.000 0.000 0.000 0.001

0.001 0.076 0.076 0.076 0.000 0.076 0.001 0.003 0.001 0.076

0.002 0.000 0.000 0.000 0.006 0.004 0.005 0.003 0.001 0.000

0.000 0.000 0.000 0.000 0.000 0.000 0.000 0.002 0.000 0.001

0.001 0.077 0.000 0.002 0.001 0.001 0.076 0.000 0.004 0.004

0.007 0.004

>Hypothetical 222

0.002 0.077 0.000 0.001 0.077 0.076 0.000 0.000 0.000 0.001

0.000 0.001 0.077 0.000 0.001 0.001 0.000 0.077 0.000 0.000

0.005 0.004 0.006 0.004 0.001 0.000 0.000 0.077 0.076 0.001

0.000 0.000 0.000 0.001 0.000 0.000 0.077 0.077 0.002 0.000

0.001 0.077 0.000 0.001 0.005 0.005 0.007 0.004 0.001 0.001

0.000 0.000 0.000 0.002 0.001 0.000 0.000 0.076 0.000 0.000

0.000 0.001 0.000 0.000 0.001 0.000 0.077 0.000 0.005 0.004

0.006 0.004

>Hypothetical 223

0.001 0.000 0.000 0.001 0.000 0.001 0.091 0.000 0.091 0.001

0.000 0.000 0.001 0.001 0.001 0.001 0.000 0.001 0.000 0.000

0.005 0.006 0.006 0.006 0.001 0.000 0.000 0.000 0.091 0.000

0.091 0.000 0.000 0.000 0.000 0.091 0.001 0.091 0.000 0.001

0.091 0.000 0.000 0.000 0.006 0.007 0.006 0.007 0.001 0.000

0.000 0.000 0.000 0.000 0.000 0.000 0.091 0.000 0.000 0.000

0.001 0.000 0.002 0.091 0.000 0.091 0.000 0.000 0.008 0.006

0.007 0.004

>Hypothetical 224

0.002 0.000 0.125 0.000 0.000 0.002 0.000 0.000 0.001 0.000

0.000 0.000 0.125 0.000 0.002 0.002 0.000 0.001 0.000 0.000

0.010 0.011 0.002 0.010 0.002 0.125 0.000 0.000 0.001 0.001

0.000 0.000 0.000 0.125 0.000 0.000 0.001 0.000 0.002 0.001

0.125 0.000 0.001 0.000 0.009 0.009 0.005 0.011 0.002 0.000

0.000 0.001 0.001 0.002 0.000 0.000 0.000 0.001 0.000 0.000

0.125 0.001 0.001 0.124 0.000 0.000 0.000 0.000 0.005 0.008

0.011 0.011

>Hypothetical 225

0.000 0.000 0.000 0.000 0.001 0.003 0.000 0.000 0.000 0.142

0.000 0.000 0.001 0.001 0.000 0.003 0.001 0.000 0.000 0.000

0.015 0.010 0.004 0.008 0.000 0.000 0.000 0.000 0.001 0.143

0.000 0.000 0.000 0.001 0.000 0.000 0.002 0.000 0.002 0.142

0.000 0.001 0.000 0.000 0.010 0.007 0.010 0.014 0.001 0.000

0.000 0.000 0.002 0.001 0.000 0.000 0.000 0.002 0.000 0.000

0.143 0.142 0.001 0.001 0.000 0.142 0.000 0.000 0.006 0.007

0.013 0.013

>Hypothetical 226

0.001 0.000 0.000 0.142 0.000 0.006 0.000 0.000 0.000 0.001

0.000 0.000 0.142 0.003 0.001 0.001 0.000 0.000 0.001 0.000

0.010 0.011 0.010 0.005 0.005 0.000 0.142 0.000 0.000 0.007

0.000 0.000 0.000 0.002 0.000 0.000 0.142 0.000 0.002 0.000

0.000 0.000 0.000 0.000 0.012 0.008 0.001 0.012 0.002 0.000

0.000 0.001 0.000 0.001 0.000 0.000 0.000 0.142 0.000 0.000

0.001 0.000 0.002 0.000 0.142 0.000 0.001 0.000 0.011 0.011

0.007 0.013

>Hypothetical 227

0.002 0.000 0.000 0.001 0.077 0.002 0.000 0.077 0.000 0.076

0.000 0.000 0.000 0.001 0.001 0.001 0.000 0.076 0.000 0.077

0.006 0.004 0.006 0.004 0.001 0.077 0.000 0.001 0.076 0.001

0.000 0.000 0.000 0.000 0.000 0.000 0.001 0.000 0.001 0.002

0.077 0.000 0.000 0.077 0.006 0.007 0.004 0.004 0.002 0.000

0.077 0.000 0.000 0.002 0.000 0.000 0.000 0.001 0.000 0.000

0.000 0.000 0.002 0.076 0.000 0.076 0.000 0.000 0.005 0.003

0.006 0.006

>Hypothetical 228

0.002 0.071 0.000 0.071 0.000 0.001 0.071 0.000 0.000 0.001

0.071 0.000 0.001 0.000 0.002 0.071 0.071 0.000 0.000 0.000

0.004 0.005 0.006 0.005 0.001 0.071 0.000 0.001 0.000 0.001

0.071 0.000 0.000 0.071 0.000 0.000 0.000 0.001 0.001 0.001

0.071 0.000 0.000 0.000 0.005 0.004 0.006 0.003 0.001 0.000

0.071 0.001 0.000 0.001 0.071 0.071 0.000 0.000 0.000 0.000

0.001 0.000 0.001 0.001 0.000 0.000 0.000 0.000 0.006 0.006

0.003 0.004

>Hypothetical 229

0.100 0.000 0.000 0.000 0.100 0.002 0.000 0.000 0.001 0.001

0.000 0.000 0.001 0.000 0.001 0.001 0.100 0.000 0.100 0.000

0.004 0.006 0.007 0.009 0.001 0.000 0.000 0.002 0.001 0.001

0.001 0.100 0.000 0.000 0.000 0.001 0.001 0.001 0.100 0.001

0.001 0.000 0.001 0.000 0.007 0.006 0.005 0.007 0.001 0.000

0.000 0.000 0.100 0.001 0.100 0.000 0.002 0.000 0.100 0.000

0.001 0.000 0.001 0.001 0.001 0.001 0.000 0.000 0.006 0.006

0.006 0.008

>Hypothetical 230

0.001 0.000 0.000 0.125 0.000 0.002 0.000 0.000 0.001 0.000

0.000 0.000 0.002 0.000 0.001 0.003 0.001 0.001 0.124 0.000

0.007 0.007 0.008 0.010 0.124 0.000 0.125 0.000 0.000 0.001

0.001 0.000 0.000 0.001 0.000 0.000 0.001 0.001 0.001 0.002

0.000 0.001 0.000 0.000 0.008 0.006 0.006 0.012 0.002 0.000

0.000 0.000 0.001 0.000 0.001 0.001 0.000 0.124 0.000 0.000

0.000 0.000 0.001 0.125 0.125 0.000 0.001 0.000 0.011 0.009

0.010 0.006

>Hypothetical 231

0.062 0.000 0.000 0.000 0.000 0.000 0.001 0.000 0.000 0.000

0.062 0.062 0.001 0.062 0.001 0.062 0.062 0.001 0.000 0.062

0.007 0.003 0.002 0.007 0.001 0.062 0.000 0.000 0.001 0.001

0.000 0.000 0.000 0.000 0.000 0.000 0.000 0.000 0.000 0.062

0.062 0.062 0.000 0.000 0.005 0.006 0.006 0.001 0.000 0.000

0.000 0.062 0.000 0.002 0.000 0.000 0.000 0.062 0.000 0.000

0.002 0.062 0.000 0.002 0.001 0.001 0.000 0.062 0.003 0.003

0.003 0.004

>Hypothetical 232

0.001 0.000 0.000 0.000 0.000 0.055 0.055 0.000 0.000 0.000

0.000 0.000 0.001 0.055 0.055 0.001 0.000 0.055 0.000 0.055

0.003 0.005 0.004 0.004 0.002 0.000 0.055 0.000 0.001 0.055

0.000 0.055 0.055 0.000 0.000 0.055 0.001 0.001 0.001 0.001

0.055 0.000 0.000 0.000 0.002 0.005 0.005 0.003 0.000 0.000

0.000 0.001 0.001 0.055 0.000 0.055 0.000 0.000 0.000 0.000

0.001 0.000 0.001 0.055 0.055 0.055 0.000 0.000 0.003 0.006

0.003 0.005

>Hypothetical 233

0.001 0.111 0.000 0.000 0.000 0.002 0.000 0.000 0.000 0.001

0.000 0.000 0.001 0.001 0.002 0.000 0.000 0.000 0.111 0.000

0.008 0.011 0.005 0.006 0.002 0.000 0.001 0.000 0.000 0.001

0.000 0.000 0.000 0.000 0.000 0.000 0.001 0.111 0.001 0.001

0.000 0.111 0.000 0.000 0.008 0.007 0.009 0.007 0.002 0.000

0.000 0.111 0.111 0.002 0.000 0.000 0.000 0.111 0.000 0.000

0.001 0.001 0.001 0.111 0.001 0.001 0.001 0.000 0.005 0.008

0.009 0.008

>Hypothetical 234

0.013 0.000 0.000 0.013 0.000 0.019 0.000 0.000 0.000 0.006

0.000 0.000 0.000 0.013 0.006 0.013 0.000 0.000 0.000 0.000

0.078 0.046 0.058 0.069 0.008 0.000 0.000 0.000 0.000 0.008

0.000 0.000 0.008 0.000 0.000 0.000 0.012 0.000 0.008 0.004

0.000 0.000 0.004 0.000 0.043 0.068 0.086 0.082 0.013 0.000

0.000 0.000 0.000 0.004 0.000 0.000 0.000 0.004 0.000 0.000

0.004 0.000 0.021 0.000 0.000 0.004 0.000 0.000 0.094 0.067

0.071 0.051

>Hypothetical 235

0.000 0.000 0.000 0.002 0.000 0.001 0.066 0.066 0.000 0.001

0.000 0.066 0.000 0.001 0.000 0.000 0.000 0.000 0.000 0.000

0.006 0.002 0.004 0.005 0.001 0.000 0.000 0.000 0.066 0.000

0.000 0.066 0.000 0.000 0.000 0.066 0.000 0.066 0.001 0.000

0.066 0.066 0.000 0.000 0.004 0.004 0.007 0.006 0.001 0.000

0.001 0.000 0.066 0.001 0.066 0.066 0.066 0.000 0.000 0.066

0.000 0.000 0.002 0.000 0.000 0.001 0.000 0.000 0.004 0.005

0.005 0.005

>Hypothetical 236

0.143 0.000 0.000 0.000 0.002 0.143 0.000 0.000 0.001 0.002

0.000 0.000 0.003 0.001 0.142 0.001 0.001 0.001 0.000 0.000

0.011 0.008 0.009 0.007 0.142 0.001 0.000 0.000 0.000 0.142

0.000 0.000 0.000 0.001 0.000 0.000 0.004 0.000 0.001 0.007

0.000 0.000 0.000 0.000 0.012 0.007 0.004 0.011 0.001 0.000

0.000 0.000 0.001 0.142 0.001 0.000 0.000 0.001 0.000 0.000

0.002 0.001 0.001 0.000 0.000 0.001 0.000 0.000 0.011 0.008

0.009 0.011

>Hypothetical 237

0.000 0.001 0.001 0.000 0.000 0.000 0.001 0.000 0.077 0.077

0.000 0.077 0.000 0.000 0.000 0.002 0.001 0.077 0.000 0.000

0.006 0.004 0.004 0.006 0.005 0.000 0.000 0.000 0.077 0.077

0.000 0.000 0.000 0.003 0.000 0.077 0.005 0.000 0.077 0.000

0.003 0.076 0.000 0.000 0.002 0.003 0.003 0.004 0.000 0.000

0.000 0.000 0.000 0.077 0.001 0.000 0.000 0.001 0.000 0.000

0.001 0.077 0.000 0.000 0.077 0.000 0.001 0.000 0.007 0.004

0.008 0.003

>Hypothetical 238

0.001 0.000 0.001 0.001 0.066 0.001 0.066 0.000 0.066 0.001

0.000 0.000 0.000 0.066 0.001 0.000 0.000 0.000 0.066 0.066

0.003 0.006 0.005 0.005 0.002 0.000 0.001 0.000 0.066 0.001

0.000 0.001 0.001 0.002 0.066 0.000 0.000 0.000 0.066 0.066

0.000 0.000 0.000 0.000 0.004 0.004 0.004 0.004 0.001 0.000

0.000 0.000 0.000 0.066 0.000 0.000 0.000 0.001 0.000 0.000

0.066 0.066 0.002 0.000 0.001 0.066 0.000 0.000 0.005 0.005

0.004 0.005

>Hypothetical 239

0.001 0.000 0.000 0.000 0.000 0.000 0.083 0.000 0.083 0.000

0.000 0.000 0.000 0.083 0.002 0.000 0.001 0.000 0.000 0.000

0.007 0.005 0.006 0.007 0.002 0.000 0.083 0.000 0.000 0.000

0.000 0.000 0.000 0.083 0.000 0.083 0.001 0.000 0.001 0.000

0.083 0.000 0.000 0.000 0.005 0.005 0.005 0.009 0.001 0.000

0.000 0.083 0.000 0.000 0.000 0.000 0.000 0.083 0.000 0.000

0.001 0.000 0.001 0.083 0.083 0.000 0.000 0.000 0.007 0.005

0.005 0.007

>Hypothetical 240

0.000 0.062 0.000 0.000 0.000 0.000 0.000 0.001 0.000 0.001

0.000 0.000 0.062 0.000 0.000 0.000 0.000 0.062 0.062 0.000

0.003 0.003 0.005 0.005 0.062 0.000 0.000 0.001 0.062 0.001

0.062 0.000 0.000 0.062 0.000 0.000 0.001 0.062 0.000 0.005

0.002 0.062 0.000 0.000 0.002 0.002 0.003 0.004 0.001 0.062

0.000 0.000 0.062 0.062 0.001 0.000 0.000 0.000 0.062 0.000

0.001 0.000 0.000 0.062 0.000 0.000 0.000 0.000 0.004 0.004

0.004 0.005

>Hypothetical 241

0.001 0.001 0.000 0.000 0.000 0.001 0.077 0.000 0.000 0.077

0.000 0.000 0.002 0.000 0.000 0.002 0.002 0.077 0.000 0.000

0.006 0.004 0.005 0.003 0.002 0.000 0.000 0.000 0.077 0.000

0.000 0.000 0.000 0.077 0.000 0.000 0.001 0.001 0.001 0.077

0.077 0.000 0.077 0.000 0.005 0.007 0.005 0.005 0.000 0.077

0.000 0.000 0.077 0.077 0.000 0.000 0.000 0.001 0.000 0.000

0.001 0.000 0.001 0.077 0.000 0.000 0.000 0.000 0.006 0.004

0.006 0.005

>Hypothetical 242

0.066 0.000 0.001 0.067 0.001 0.000 0.000 0.000 0.001 0.003

0.000 0.000 0.001 0.067 0.066 0.000 0.067 0.000 0.000 0.000

0.003 0.005 0.005 0.004 0.000 0.001 0.067 0.000 0.000 0.000

0.067 0.000 0.067 0.067 0.000 0.000 0.000 0.000 0.000 0.001

0.000 0.067 0.000 0.000 0.003 0.005 0.006 0.005 0.003 0.067

0.000 0.000 0.067 0.001 0.000 0.000 0.000 0.001 0.000 0.067

0.001 0.001 0.001 0.000 0.067 0.001 0.000 0.000 0.004 0.004

0.004 0.004

>Hypothetical 243

0.091 0.001 0.001 0.001 0.091 0.000 0.000 0.000 0.001 0.003

0.000 0.001 0.091 0.000 0.091 0.000 0.000 0.000 0.000 0.000

0.006 0.005 0.006 0.007 0.001 0.002 0.000 0.000 0.091 0.000

0.000 0.091 0.000 0.091 0.000 0.000 0.000 0.091 0.001 0.002

0.001 0.000 0.000 0.000 0.005 0.008 0.007 0.004 0.003 0.000

0.000 0.091 0.090 0.002 0.000 0.000 0.000 0.002 0.000 0.000

0.001 0.002 0.000 0.000 0.000 0.000 0.000 0.000 0.006 0.007

0.003 0.007

>Hypothetical 244

0.003 0.000 0.000 0.000 0.166 0.000 0.000 0.000 0.000 0.004

0.000 0.000 0.003 0.000 0.001 0.001 0.000 0.000 0.000 0.000

0.014 0.010 0.006 0.014 0.003 0.001 0.000 0.000 0.001 0.166

0.000 0.000 0.000 0.001 0.000 0.000 0.004 0.000 0.001 0.166

0.000 0.000 0.001 0.000 0.010 0.008 0.011 0.014 0.002 0.000

0.000 0.001 0.000 0.001 0.000 0.000 0.000 0.001 0.000 0.000

0.002 0.166 0.003 0.001 0.000 0.166 0.000 0.000 0.012 0.012

0.009 0.014

>Hypothetical 245

0.001 0.000 0.111 0.001 0.000 0.000 0.000 0.000 0.000 0.001

0.000 0.000 0.001 0.111 0.002 0.000 0.000 0.000 0.000 0.000

0.007 0.008 0.007 0.010 0.003 0.000 0.000 0.000 0.000 0.110

0.000 0.000 0.000 0.003 0.000 0.000 0.001 0.110 0.002 0.001

0.110 0.000 0.000 0.000 0.008 0.008 0.005 0.008 0.003 0.001

0.000 0.000 0.000 0.111 0.000 0.000 0.000 0.001 0.000 0.000

0.003 0.111 0.111 0.000 0.000 0.000 0.001 0.000 0.006 0.006

0.008 0.008

>Hypothetical 246

0.001 0.001 0.143 0.000 0.000 0.001 0.143 0.000 0.001 0.001

0.000 0.001 0.000 0.000 0.001 0.001 0.000 0.001 0.000 0.000

0.009 0.012 0.011 0.009 0.003 0.000 0.000 0.000 0.001 0.001

0.000 0.143 0.000 0.001 0.000 0.001 0.002 0.143 0.001 0.000

0.001 0.002 0.000 0.000 0.010 0.009 0.008 0.009 0.000 0.001

0.000 0.143 0.001 0.001 0.000 0.000 0.000 0.002 0.000 0.000

0.002 0.002 0.001 0.143 0.001 0.000 0.000 0.000 0.009 0.010

0.010 0.010

>Hypothetical 247

0.001 0.000 0.000 0.100 0.001 0.001 0.000 0.000 0.100 0.100

0.000 0.000 0.000 0.100 0.001 0.001 0.001 0.001 0.000 0.000

0.007 0.006 0.007 0.008 0.002 0.000 0.000 0.100 0.001 0.001

0.000 0.000 0.000 0.001 0.000 0.000 0.003 0.001 0.001 0.100

0.000 0.000 0.100 0.000 0.007 0.007 0.006 0.005 0.100 0.001

0.000 0.000 0.000 0.000 0.000 0.000 0.100 0.001 0.000 0.001

0.001 0.000 0.001 0.000 0.000 0.000 0.000 0.000 0.009 0.007

0.008 0.004

>Hypothetical 248

0.001 0.000 0.000 0.000 0.111 0.002 0.000 0.000 0.000 0.000

0.000 0.000 0.000 0.000 0.002 0.001 0.111 0.111 0.000 0.000

0.009 0.007 0.006 0.009 0.002 0.000 0.000 0.001 0.111 0.003

0.000 0.000 0.000 0.111 0.000 0.000 0.003 0.111 0.001 0.000

0.000 0.000 0.000 0.000 0.005 0.008 0.008 0.008 0.000 0.000

0.000 0.001 0.000 0.001 0.000 0.000 0.000 0.001 0.000 0.111

0.002 0.000 0.001 0.111 0.000 0.001 0.000 0.000 0.008 0.009

0.009 0.005

>Hypothetical 249

0.000 0.001 0.000 0.000 0.000 0.001 0.000 0.077 0.000 0.001

0.077 0.000 0.002 0.000 0.000 0.000 0.000 0.001 0.077 0.000

0.007 0.007 0.006 0.002 0.000 0.000 0.077 0.000 0.000 0.001

0.000 0.000 0.000 0.000 0.000 0.000 0.000 0.000 0.002 0.001

0.000 0.077 0.077 0.000 0.006 0.005 0.004 0.007 0.003 0.000

0.077 0.001 0.000 0.002 0.000 0.000 0.000 0.077 0.000 0.077

0.003 0.000 0.001 0.077 0.077 0.077 0.000 0.000 0.004 0.006

0.004 0.005

>Hypothetical 250

0.001 0.000 0.000 0.111 0.000 0.002 0.000 0.000 0.001 0.002

0.000 0.001 0.002 0.111 0.110 0.000 0.000 0.000 0.001 0.000

0.007 0.006 0.009 0.008 0.110 0.111 0.001 0.000 0.000 0.001

0.000 0.000 0.001 0.000 0.001 0.000 0.111 0.000 0.001 0.000

0.000 0.000 0.001 0.000 0.008 0.008 0.006 0.010 0.002 0.000

0.000 0.004 0.000 0.004 0.000 0.000 0.000 0.002 0.000 0.000

0.110 0.001 0.111 0.002 0.003 0.000 0.000 0.000 0.006 0.004

0.005 0.006

>Hypothetical 251

0.091 0.091 0.001 0.000 0.001 0.000 0.001 0.000 0.000 0.091

0.000 0.091 0.091 0.001 0.001 0.000 0.000 0.000 0.000 0.001

0.006 0.006 0.007 0.006 0.000 0.000 0.001 0.000 0.091 0.002

0.000 0.002 0.001 0.001 0.000 0.001 0.091 0.000 0.001 0.000

0.002 0.091 0.000 0.000 0.006 0.004 0.005 0.006 0.001 0.091

0.000 0.000 0.091 0.001 0.000 0.001 0.000 0.001 0.000 0.000

0.001 0.001 0.001 0.001 0.001 0.001 0.000 0.000 0.006 0.005

0.005 0.005

>Hypothetical 252

0.000 0.001 0.001 0.111 0.001 0.001 0.000 0.000 0.111 0.001

0.000 0.111 0.111 0.001 0.001 0.002 0.000 0.000 0.000 0.000

0.008 0.008 0.007 0.006 0.002 0.000 0.000 0.001 0.111 0.000

0.000 0.002 0.000 0.001 0.000 0.000 0.001 0.001 0.001 0.002

0.111 0.001 0.000 0.000 0.008 0.008 0.007 0.004 0.111 0.000

0.000 0.001 0.111 0.000 0.000 0.000 0.000 0.002 0.000 0.000

0.001 0.001 0.001 0.001 0.000 0.000 0.001 0.000 0.009 0.009

0.005 0.007

>Hypothetical 253

0.000 0.000 0.083 0.000 0.083 0.083 0.001 0.000 0.001 0.083

0.000 0.000 0.000 0.000 0.002 0.000 0.000 0.000 0.001 0.000

0.004 0.007 0.006 0.006 0.000 0.000 0.002 0.000 0.001 0.002

0.000 0.001 0.000 0.001 0.083 0.000 0.083 0.000 0.083 0.001

0.003 0.001 0.000 0.000 0.003 0.006 0.005 0.003 0.002 0.001

0.000 0.000 0.083 0.083 0.000 0.000 0.000 0.001 0.000 0.000

0.083 0.002 0.001 0.003 0.001 0.001 0.083 0.000 0.004 0.005

0.004 0.005

>Hypothetical 254

0.143 0.143 0.000 0.000 0.000 0.002 0.001 0.001 0.000 0.001

0.000 0.000 0.000 0.143 0.002 0.001 0.001 0.001 0.000 0.000

0.011 0.012 0.007 0.009 0.001 0.001 0.000 0.001 0.143 0.001

0.002 0.000 0.000 0.143 0.000 0.000 0.142 0.001 0.000 0.005

0.001 0.000 0.000 0.000 0.007 0.008 0.009 0.009 0.001 0.001

0.000 0.000 0.000 0.001 0.000 0.000 0.000 0.001 0.000 0.001

0.003 0.000 0.001 0.001 0.001 0.000 0.000 0.000 0.008 0.010

0.011 0.009

>Hypothetical 255

0.001 0.001 0.071 0.001 0.000 0.000 0.001 0.000 0.000 0.071

0.000 0.071 0.071 0.001 0.000 0.003 0.071 0.000 0.000 0.000

0.003 0.004 0.005 0.005 0.001 0.001 0.071 0.071 0.000 0.001

0.000 0.000 0.000 0.000 0.000 0.001 0.002 0.000 0.001 0.001

0.000 0.071 0.071 0.000 0.004 0.005 0.006 0.005 0.000 0.001

0.000 0.071 0.000 0.001 0.000 0.001 0.000 0.001 0.000 0.071

0.001 0.000 0.001 0.071 0.071 0.001 0.000 0.000 0.004 0.007

0.002 0.007

>Hypothetical 256

0.002 0.000 0.000 0.001 0.000 0.001 0.001 0.000 0.000 0.001

0.000 0.000 0.142 0.000 0.001 0.002 0.001 0.001 0.000 0.000

0.009 0.008 0.012 0.006 0.002 0.001 0.142 0.000 0.000 0.000

0.142 0.000 0.000 0.006 0.000 0.002 0.003 0.000 0.000 0.002

0.002 0.000 0.000 0.000 0.006 0.006 0.011 0.008 0.142 0.002

0.001 0.000 0.000 0.000 0.000 0.000 0.000 0.001 0.000 0.000

0.001 0.000 0.001 0.142 0.142 0.001 0.001 0.000 0.007 0.009

0.014 0.012

>Hypothetical 257

0.002 0.000 0.001 0.083 0.000 0.001 0.000 0.000 0.001 0.003

0.000 0.001 0.083 0.000 0.083 0.002 0.083 0.000 0.000 0.000

0.005 0.005 0.004 0.005 0.083 0.001 0.001 0.001 0.000 0.083

0.000 0.000 0.000 0.000 0.000 0.000 0.001 0.000 0.000 0.083

0.001 0.000 0.001 0.083 0.005 0.005 0.007 0.007 0.000 0.001

0.000 0.001 0.000 0.001 0.083 0.000 0.001 0.001 0.000 0.000

0.083 0.000 0.001 0.083 0.001 0.001 0.000 0.000 0.007 0.004

0.008 0.002

>Hypothetical 258

0.001 0.000 0.000 0.100 0.000 0.000 0.000 0.000 0.000 0.001

0.000 0.000 0.001 0.000 0.001 0.000 0.000 0.000 0.000 0.000

0.005 0.007 0.008 0.007 0.001 0.001 0.100 0.000 0.000 0.001

0.100 0.000 0.100 0.001 0.000 0.000 0.001 0.000 0.001 0.002

0.000 0.100 0.000 0.000 0.008 0.007 0.005 0.007 0.002 0.100

0.001 0.000 0.100 0.001 0.001 0.000 0.000 0.000 0.000 0.100

0.001 0.001 0.001 0.001 0.100 0.000 0.000 0.000 0.007 0.008

0.006 0.006

>Hypothetical 259

0.002 0.001 0.000 0.000 0.142 0.001 0.000 0.000 0.000 0.000

0.000 0.000 0.003 0.142 0.001 0.001 0.142 0.001 0.000 0.000

0.010 0.011 0.008 0.010 0.005 0.000 0.001 0.000 0.142 0.001

0.001 0.000 0.000 0.001 0.000 0.000 0.001 0.001 0.000 0.000

0.000 0.142 0.000 0.001 0.008 0.013 0.009 0.006 0.000 0.000

0.000 0.000 0.142 0.001 0.000 0.000 0.000 0.000 0.001 0.000

0.002 0.001 0.002 0.002 0.001 0.000 0.000 0.000 0.008 0.011

0.010 0.009

>Hypothetical 260

0.001 0.000 0.000 0.000 0.000 0.001 0.000 0.000 0.071 0.001

0.001 0.001 0.071 0.001 0.071 0.002 0.001 0.071 0.000 0.001

0.002 0.004 0.005 0.005 0.001 0.000 0.000 0.000 0.000 0.071

0.071 0.000 0.000 0.071 0.000 0.000 0.001 0.071 0.001 0.001

0.000 0.000 0.000 0.000 0.004 0.004 0.004 0.007 0.001 0.000

0.000 0.000 0.071 0.000 0.071 0.071 0.000 0.001 0.000 0.000

0.071 0.000 0.001 0.000 0.000 0.000 0.071 0.000 0.005 0.004

0.005 0.005

>Hypothetical 261

0.000 0.000 0.000 0.062 0.000 0.001 0.000 0.062 0.062 0.000

0.062 0.000 0.062 0.001 0.000 0.000 0.000 0.000 0.062 0.000

0.004 0.003 0.004 0.005 0.001 0.062 0.062 0.000 0.000 0.001

0.001 0.001 0.062 0.001 0.000 0.000 0.000 0.000 0.001 0.001

0.000 0.062 0.062 0.000 0.004 0.005 0.004 0.003 0.001 0.000

0.062 0.062 0.000 0.001 0.000 0.000 0.000 0.001 0.000 0.062

0.000 0.000 0.000 0.000 0.000 0.062 0.000 0.001 0.004 0.005

0.003 0.004

>Hypothetical 262

0.071 0.000 0.071 0.001 0.071 0.000 0.071 0.000 0.000 0.000

0.000 0.000 0.000 0.071 0.001 0.001 0.001 0.000 0.001 0.000

0.006 0.007 0.005 0.002 0.071 0.000 0.000 0.001 0.000 0.002

0.000 0.000 0.071 0.000 0.071 0.000 0.071 0.000 0.002 0.071

0.071 0.000 0.000 0.000 0.008 0.004 0.004 0.005 0.071 0.000

0.001 0.001 0.000 0.000 0.000 0.000 0.000 0.000 0.000 0.000

0.071 0.000 0.000 0.001 0.000 0.000 0.000 0.000 0.005 0.005

0.004 0.007

>Hypothetical 263

0.001 0.000 0.000 0.111 0.000 0.000 0.000 0.000 0.000 0.001

0.000 0.001 0.000 0.000 0.001 0.000 0.001 0.000 0.000 0.000

0.009 0.009 0.004 0.008 0.111 0.000 0.001 0.002 0.000 0.111

0.000 0.000 0.111 0.002 0.000 0.111 0.002 0.111 0.000 0.000

0.002 0.001 0.001 0.000 0.006 0.009 0.009 0.006 0.000 0.000

0.000 0.001 0.000 0.001 0.000 0.000 0.111 0.111 0.000 0.001

0.001 0.000 0.001 0.002 0.001 0.000 0.000 0.000 0.008 0.007

0.007 0.008

>Hypothetical 264

0.001 0.000 0.001 0.110 0.000 0.001 0.000 0.000 0.000 0.002

0.000 0.001 0.000 0.000 0.001 0.001 0.000 0.001 0.000 0.000

0.009 0.008 0.005 0.009 0.000 0.000 0.001 0.001 0.001 0.110

0.000 0.111 0.000 0.001 0.000 0.000 0.001 0.001 0.111 0.111

0.002 0.000 0.001 0.000 0.005 0.008 0.009 0.007 0.000 0.001

0.111 0.000 0.001 0.000 0.111 0.000 0.111 0.000 0.000 0.001

0.001 0.000 0.000 0.001 0.001 0.000 0.000 0.000 0.009 0.006

0.006 0.010

>Hypothetical 265

0.076 0.000 0.001 0.002 0.001 0.077 0.000 0.000 0.000 0.000

0.077 0.000 0.001 0.001 0.076 0.077 0.002 0.000 0.001 0.000

0.004 0.005 0.006 0.005 0.076 0.000 0.000 0.001 0.001 0.077

0.000 0.000 0.000 0.077 0.000 0.001 0.001 0.000 0.001 0.001

0.001 0.000 0.000 0.000 0.005 0.005 0.006 0.005 0.076 0.000

0.001 0.000 0.000 0.001 0.076 0.000 0.000 0.002 0.000 0.001

0.076 0.076 0.001 0.001 0.001 0.002 0.000 0.000 0.006 0.004

0.004 0.007

>Hypothetical 266

0.001 0.000 0.000 0.000 0.001 0.071 0.000 0.000 0.000 0.001

0.000 0.071 0.002 0.001 0.000 0.001 0.071 0.071 0.000 0.000

0.004 0.005 0.005 0.005 0.001 0.000 0.000 0.000 0.001 0.000

0.000 0.071 0.000 0.000 0.000 0.000 0.002 0.071 0.001 0.000

0.071 0.071 0.071 0.000 0.006 0.006 0.005 0.003 0.000 0.000

0.000 0.000 0.071 0.071 0.071 0.000 0.071 0.000 0.000 0.000

0.002 0.000 0.001 0.001 0.001 0.000 0.000 0.000 0.005 0.007

0.002 0.005

>Hypothetical 267

0.004 0.000 0.000 0.000 0.003 0.332 0.000 0.000 0.000 0.003

0.000 0.000 0.005 0.000 0.009 0.004 0.000 0.000 0.000 0.000

0.018 0.016 0.025 0.026 0.005 0.000 0.000 0.000 0.002 0.004

0.000 0.000 0.000 0.000 0.000 0.000 0.005 0.000 0.003 0.002

0.000 0.000 0.000 0.000 0.024 0.024 0.025 0.015 0.004 0.000

0.000 0.000 0.000 0.333 0.000 0.000 0.000 0.002 0.000 0.000

0.007 0.003 0.003 0.003 0.000 0.002 0.000 0.000 0.021 0.024

0.018 0.025

>Hypothetical 268

0.002 0.000 0.000 0.000 0.000 0.125 0.000 0.000 0.000 0.001

0.000 0.000 0.003 0.001 0.001 0.001 0.125 0.001 0.000 0.000

0.008 0.009 0.007 0.009 0.001 0.000 0.000 0.000 0.001 0.125

0.000 0.000 0.000 0.001 0.000 0.000 0.002 0.125 0.003 0.001

0.000 0.000 0.000 0.000 0.008 0.007 0.010 0.008 0.002 0.125

0.125 0.000 0.001 0.001 0.000 0.000 0.000 0.000 0.000 0.000

0.002 0.125 0.001 0.001 0.000 0.000 0.000 0.000 0.008 0.008

0.010 0.008

>Hypothetical 269

0.002 0.124 0.124 0.000 0.001 0.002 0.000 0.000 0.000 0.000

0.000 0.000 0.003 0.124 0.002 0.001 0.000 0.000 0.000 0.000

0.009 0.008 0.012 0.005 0.002 0.000 0.000 0.000 0.000 0.124

0.000 0.000 0.000 0.001 0.000 0.000 0.002 0.001 0.002 0.001

0.124 0.001 0.000 0.000 0.009 0.009 0.005 0.012 0.002 0.000

0.000 0.000 0.001 0.125 0.000 0.000 0.000 0.001 0.000 0.000

0.001 0.125 0.003 0.003 0.000 0.000 0.000 0.000 0.006 0.006

0.011 0.009

>Hypothetical 270

0.001 0.000 0.000 0.001 0.000 0.001 0.077 0.000 0.000 0.076

0.000 0.000 0.000 0.000 0.003 0.000 0.000 0.000 0.000 0.000

0.003 0.004 0.005 0.007 0.000 0.000 0.000 0.077 0.077 0.002

0.077 0.000 0.001 0.001 0.000 0.077 0.001 0.077 0.001 0.077

0.077 0.001 0.077 0.000 0.005 0.004 0.004 0.007 0.001 0.000

0.000 0.000 0.000 0.001 0.000 0.000 0.076 0.000 0.000 0.000

0.000 0.001 0.001 0.000 0.077 0.000 0.000 0.000 0.001 0.005

0.009 0.008

>Hypothetical 271

0.000 0.000 0.083 0.002 0.001 0.083 0.083 0.001 0.000 0.002

0.000 0.000 0.000 0.001 0.001 0.083 0.000 0.001 0.083 0.001

0.005 0.006 0.006 0.003 0.083 0.001 0.000 0.000 0.001 0.000

0.000 0.083 0.000 0.000 0.000 0.000 0.083 0.000 0.001 0.001

0.000 0.000 0.083 0.000 0.006 0.007 0.006 0.005 0.001 0.000

0.001 0.000 0.083 0.001 0.000 0.000 0.000 0.083 0.000 0.000

0.000 0.000 0.001 0.002 0.000 0.001 0.000 0.000 0.007 0.007

0.005 0.003

>Hypothetical 272

0.001 0.000 0.000 0.124 0.000 0.003 0.000 0.000 0.001 0.000

0.000 0.000 0.001 0.124 0.002 0.002 0.001 0.002 0.000 0.000

0.010 0.005 0.010 0.006 0.124 0.000 0.000 0.001 0.001 0.003

0.000 0.000 0.125 0.004 0.000 0.000 0.001 0.001 0.001 0.000

0.000 0.001 0.000 0.000 0.008 0.009 0.006 0.008 0.125 0.001

0.000 0.001 0.001 0.125 0.000 0.000 0.000 0.000 0.000 0.000

0.125 0.000 0.000 0.002 0.000 0.000 0.001 0.001 0.009 0.007

0.010 0.010

>Hypothetical 273

0.166 0.000 0.002 0.002 0.000 0.001 0.005 0.000 0.000 0.003

0.000 0.000 0.003 0.000 0.165 0.002 0.000 0.166 0.000 0.000

0.012 0.006 0.010 0.011 0.000 0.000 0.000 0.000 0.001 0.166

0.000 0.001 0.000 0.000 0.000 0.000 0.001 0.000 0.001 0.001

0.004 0.000 0.001 0.000 0.011 0.011 0.013 0.012 0.167 0.000

0.000 0.000 0.000 0.002 0.000 0.000 0.000 0.000 0.000 0.000

0.007 0.000 0.001 0.002 0.000 0.002 0.000 0.000 0.010 0.014

0.009 0.010

>Hypothetical 274

0.002 0.100 0.000 0.000 0.000 0.002 0.000 0.000 0.000 0.000

0.000 0.000 0.003 0.000 0.001 0.002 0.000 0.001 0.000 0.000

0.006 0.008 0.006 0.005 0.099 0.000 0.100 0.002 0.000 0.002

0.001 0.000 0.000 0.001 0.000 0.000 0.004 0.001 0.100 0.001

0.000 0.000 0.000 0.100 0.007 0.005 0.006 0.006 0.000 0.000

0.000 0.000 0.001 0.100 0.000 0.001 0.000 0.000 0.100 0.000

0.001 0.100 0.001 0.001 0.100 0.000 0.000 0.000 0.006 0.007

0.008 0.007

>Hypothetical 275

0.001 0.000 0.100 0.000 0.001 0.001 0.000 0.001 0.100 0.002

0.000 0.000 0.002 0.000 0.001 0.002 0.000 0.001 0.000 0.100

0.006 0.006 0.007 0.005 0.001 0.000 0.100 0.001 0.001 0.001

0.001 0.100 0.000 0.002 0.000 0.100 0.001 0.000 0.001 0.001

0.100 0.000 0.000 0.000 0.008 0.005 0.006 0.007 0.000 0.001

0.000 0.000 0.001 0.000 0.000 0.100 0.000 0.001 0.000 0.001

0.100 0.001 0.001 0.001 0.001 0.001 0.001 0.000 0.007 0.008

0.007 0.006

>Hypothetical 276

0.071 0.001 0.000 0.000 0.000 0.001 0.000 0.000 0.001 0.071

0.000 0.000 0.071 0.001 0.001 0.000 0.001 0.071 0.001 0.000

0.006 0.007 0.005 0.004 0.002 0.071 0.000 0.000 0.071 0.001

0.000 0.000 0.001 0.071 0.000 0.001 0.001 0.000 0.000 0.000

0.000 0.001 0.071 0.000 0.003 0.005 0.005 0.006 0.071 0.071

0.000 0.001 0.071 0.002 0.001 0.001 0.000 0.001 0.000 0.000

0.001 0.000 0.000 0.000 0.000 0.071 0.071 0.000 0.004 0.005

0.005 0.004

>Hypothetical 277

0.001 0.000 0.000 0.000 0.001 0.002 0.000 0.002 0.001 0.091

0.000 0.001 0.001 0.000 0.091 0.091 0.000 0.000 0.000 0.000

0.005 0.005 0.007 0.006 0.000 0.000 0.091 0.000 0.000 0.001

0.091 0.001 0.000 0.000 0.000 0.000 0.001 0.000 0.001 0.002

0.000 0.091 0.000 0.000 0.008 0.003 0.008 0.006 0.000 0.091

0.000 0.000 0.000 0.001 0.001 0.000 0.000 0.000 0.000 0.000

0.001 0.091 0.000 0.091 0.091 0.001 0.000 0.000 0.007 0.004

0.007 0.008

>Hypothetical 278

0.083 0.000 0.000 0.083 0.000 0.001 0.001 0.000 0.000 0.083

0.000 0.000 0.001 0.000 0.001 0.083 0.000 0.000 0.000 0.000

0.008 0.002 0.007 0.007 0.000 0.000 0.000 0.000 0.001 0.002

0.000 0.002 0.001 0.083 0.000 0.001 0.002 0.000 0.000 0.083

0.000 0.083 0.000 0.000 0.006 0.005 0.007 0.004 0.000 0.083

0.000 0.001 0.000 0.001 0.000 0.001 0.083 0.083 0.000 0.000

0.001 0.000 0.001 0.002 0.000 0.000 0.083 0.000 0.007 0.002

0.007 0.006

>Hypothetical 279

0.002 0.000 0.000 0.000 0.000 0.001 0.000 0.000 0.000 0.001

0.000 0.000 0.004 0.001 0.001 0.005 0.000 0.167 0.000 0.000

0.013 0.008 0.010 0.009 0.167 0.001 0.000 0.000 0.167 0.001

0.001 0.000 0.000 0.001 0.000 0.000 0.002 0.000 0.002 0.167

0.000 0.001 0.000 0.000 0.015 0.007 0.015 0.010 0.003 0.000

0.000 0.001 0.001 0.167 0.000 0.002 0.000 0.000 0.000 0.000

0.002 0.000 0.001 0.001 0.000 0.000 0.001 0.000 0.013 0.010

0.011 0.011

>Hypothetical 280

0.002 0.000 0.100 0.001 0.001 0.001 0.000 0.002 0.000 0.000

0.000 0.000 0.100 0.000 0.000 0.001 0.100 0.000 0.001 0.000

0.009 0.005 0.008 0.005 0.001 0.000 0.100 0.000 0.000 0.001

0.000 0.000 0.000 0.001 0.000 0.000 0.002 0.001 0.001 0.005

0.100 0.001 0.000 0.000 0.007 0.003 0.005 0.006 0.099 0.001

0.000 0.000 0.000 0.000 0.002 0.000 0.000 0.001 0.000 0.100

0.001 0.100 0.001 0.000 0.100 0.002 0.000 0.000 0.010 0.003

0.010 0.006

>Hypothetical 281

0.001 0.000 0.000 0.000 0.000 0.000 0.066 0.000 0.066 0.001

0.000 0.000 0.001 0.000 0.001 0.000 0.000 0.001 0.066 0.066

0.003 0.006 0.005 0.003 0.000 0.001 0.000 0.001 0.001 0.066

0.000 0.066 0.000 0.001 0.000 0.000 0.000 0.001 0.066 0.001

0.066 0.001 0.000 0.066 0.005 0.004 0.003 0.004 0.000 0.067

0.000 0.067 0.000 0.067 0.000 0.000 0.000 0.066 0.000 0.000

0.001 0.000 0.001 0.004 0.067 0.000 0.000 0.000 0.003 0.004

0.005 0.005

>Hypothetical 282

0.001 0.000 0.055 0.000 0.000 0.000 0.000 0.000 0.055 0.000

0.000 0.055 0.000 0.055 0.001 0.055 0.001 0.000 0.000 0.055

0.006 0.003 0.004 0.004 0.001 0.000 0.000 0.055 0.000 0.055

0.002 0.055 0.055 0.000 0.000 0.000 0.055 0.000 0.001 0.000

0.055 0.000 0.000 0.000 0.005 0.002 0.004 0.005 0.001 0.001

0.000 0.055 0.000 0.000 0.001 0.000 0.055 0.055 0.000 0.000

0.000 0.055 0.000 0.055 0.002 0.000 0.000 0.000 0.006 0.003

0.002 0.005

>Hypothetical 283

0.001 0.000 0.000 0.066 0.001 0.066 0.000 0.000 0.000 0.002

0.000 0.000 0.066 0.000 0.001 0.001 0.066 0.066 0.000 0.001

0.005 0.005 0.008 0.000 0.001 0.002 0.000 0.066 0.000 0.067

0.000 0.000 0.000 0.001 0.000 0.000 0.000 0.000 0.067 0.000

0.001 0.001 0.000 0.000 0.006 0.005 0.004 0.003 0.002 0.000

0.066 0.066 0.001 0.000 0.000 0.000 0.000 0.001 0.001 0.066

0.000 0.000 0.000 0.066 0.066 0.001 0.066 0.000 0.004 0.004

0.005 0.004

>Hypothetical 284

0.001 0.001 0.000 0.077 0.000 0.077 0.077 0.000 0.000 0.001

0.000 0.000 0.001 0.000 0.077 0.000 0.077 0.001 0.001 0.000

0.005 0.004 0.004 0.007 0.001 0.000 0.000 0.077 0.001 0.001

0.000 0.000 0.000 0.002 0.000 0.000 0.077 0.077 0.001 0.001

0.077 0.000 0.001 0.000 0.004 0.005 0.003 0.006 0.000 0.077

0.000 0.000 0.000 0.000 0.077 0.000 0.000 0.002 0.000 0.000

0.000 0.077 0.001 0.000 0.000 0.001 0.000 0.000 0.006 0.006

0.006 0.002

>Hypothetical 285

0.002 0.000 0.000 0.000 0.002 0.001 0.125 0.000 0.125 0.002

0.000 0.000 0.125 0.000 0.001 0.001 0.000 0.001 0.001 0.000

0.008 0.008 0.007 0.009 0.002 0.000 0.000 0.000 0.000 0.002

0.000 0.000 0.000 0.005 0.000 0.000 0.001 0.000 0.001 0.001

0.125 0.001 0.125 0.000 0.007 0.006 0.006 0.008 0.001 0.001

0.000 0.125 0.000 0.125 0.000 0.000 0.000 0.002 0.000 0.000

0.001 0.000 0.001 0.002 0.000 0.001 0.001 0.000 0.008 0.008

0.008 0.010

>Hypothetical 286

0.004 0.000 0.071 0.071 0.000 0.003 0.000 0.000 0.000 0.005

0.070 0.000 0.002 0.000 0.000 0.001 0.000 0.003 0.000 0.000

0.005 0.001 0.002 0.002 0.001 0.000 0.000 0.000 0.071 0.000

0.071 0.071 0.071 0.003 0.000 0.071 0.000 0.000 0.001 0.001

0.000 0.071 0.001 0.000 0.003 0.006 0.005 0.005 0.001 0.001

0.071 0.000 0.001 0.001 0.000 0.071 0.000 0.000 0.000 0.000

0.000 0.000 0.071 0.000 0.000 0.001 0.001 0.071 0.007 0.004

0.004 0.005

>Hypothetical 287

0.001 0.002 0.000 0.001 0.000 0.000 0.001 0.000 0.000 0.090

0.000 0.000 0.000 0.000 0.002 0.090 0.090 0.000 0.090 0.000

0.008 0.004 0.007 0.008 0.001 0.000 0.000 0.002 0.000 0.000

0.000 0.000 0.090 0.000 0.000 0.090 0.090 0.001 0.002 0.001

0.001 0.002 0.000 0.000 0.004 0.007 0.005 0.006 0.000 0.000

0.000 0.000 0.000 0.091 0.090 0.090 0.001 0.000 0.000 0.000

0.001 0.000 0.001 0.001 0.000 0.000 0.001 0.000 0.005 0.009

0.006 0.007

>Hypothetical 288

0.000 0.001 0.001 0.000 0.001 0.001 0.125 0.000 0.000 0.001

0.000 0.001 0.125 0.001 0.000 0.002 0.001 0.125 0.000 0.000

0.007 0.008 0.009 0.010 0.003 0.000 0.000 0.000 0.000 0.125

0.001 0.001 0.000 0.001 0.000 0.000 0.000 0.125 0.001 0.125

0.001 0.000 0.002 0.000 0.009 0.007 0.010 0.007 0.000 0.000

0.001 0.001 0.000 0.001 0.000 0.000 0.000 0.001 0.001 0.000

0.000 0.001 0.001 0.001 0.000 0.000 0.000 0.125 0.006 0.009

0.008 0.011

>Hypothetical 289

0.002 0.000 0.000 0.001 0.000 0.000 0.100 0.001 0.000 0.001

0.000 0.000 0.001 0.001 0.002 0.000 0.100 0.000 0.000 0.000

0.004 0.006 0.007 0.009 0.100 0.001 0.000 0.001 0.000 0.002

0.000 0.000 0.000 0.100 0.000 0.100 0.001 0.000 0.001 0.001

0.100 0.100 0.000 0.000 0.006 0.007 0.007 0.008 0.002 0.000

0.001 0.001 0.000 0.000 0.100 0.100 0.000 0.000 0.000 0.000

0.000 0.000 0.002 0.000 0.001 0.001 0.000 0.000 0.006 0.006

0.007 0.009

>Hypothetical 290

0.001 0.000 0.090 0.000 0.000 0.091 0.000 0.090 0.090 0.000

0.000 0.000 0.090 0.000 0.002 0.001 0.001 0.000 0.001 0.000

0.007 0.006 0.008 0.005 0.003 0.000 0.000 0.000 0.000 0.005

0.000 0.000 0.000 0.004 0.000 0.000 0.002 0.000 0.090 0.090

0.091 0.000 0.001 0.000 0.003 0.005 0.004 0.005 0.000 0.002

0.000 0.000 0.000 0.000 0.001 0.000 0.000 0.001 0.000 0.000

0.001 0.091 0.091 0.001 0.000 0.001 0.000 0.000 0.003 0.009

0.006 0.007

>Hypothetical 291

0.000 0.062 0.062 0.000 0.062 0.001 0.000 0.000 0.000 0.000

0.000 0.062 0.000 0.062 0.001 0.001 0.000 0.000 0.000 0.000

0.005 0.004 0.004 0.004 0.001 0.000 0.000 0.000 0.000 0.001

0.062 0.062 0.000 0.001 0.000 0.000 0.062 0.000 0.000 0.000

0.062 0.000 0.062 0.000 0.006 0.002 0.005 0.004 0.001 0.000

0.062 0.062 0.000 0.000 0.000 0.000 0.000 0.001 0.062 0.000

0.000 0.062 0.000 0.062 0.000 0.000 0.000 0.000 0.004 0.004

0.004 0.004

>Hypothetical 292

0.090 0.089 0.000 0.001 0.002 0.001 0.000 0.001 0.001 0.002

0.090 0.000 0.001 0.090 0.001 0.000 0.000 0.000 0.000 0.000

0.005 0.009 0.006 0.006 0.090 0.000 0.001 0.001 0.001 0.001

0.000 0.000 0.091 0.000 0.000 0.000 0.001 0.000 0.001 0.002

0.000 0.000 0.000 0.000 0.007 0.005 0.006 0.006 0.000 0.090

0.000 0.003 0.000 0.091 0.090 0.001 0.000 0.001 0.000 0.001

0.000 0.000 0.001 0.090 0.000 0.002 0.000 0.000 0.007 0.005

0.008 0.004

>Hypothetical 293

0.002 0.000 0.000 0.000 0.000 0.091 0.000 0.000 0.000 0.000

0.000 0.000 0.001 0.091 0.002 0.000 0.000 0.091 0.000 0.000

0.008 0.008 0.003 0.006 0.002 0.000 0.091 0.091 0.091 0.000

0.001 0.000 0.000 0.091 0.000 0.000 0.002 0.000 0.001 0.091

0.000 0.000 0.000 0.000 0.007 0.006 0.005 0.007 0.001 0.091

0.000 0.000 0.000 0.000 0.000 0.000 0.000 0.000 0.000 0.000

0.001 0.000 0.002 0.091 0.001 0.000 0.000 0.000 0.009 0.005

0.005 0.006

>Hypothetical 294

0.075 0.076 0.000 0.000 0.076 0.001 0.000 0.001 0.000 0.076

0.076 0.000 0.000 0.000 0.002 0.000 0.001 0.076 0.001 0.000

0.003 0.009 0.009 0.005 0.000 0.000 0.000 0.000 0.000 0.004

0.000 0.000 0.000 0.077 0.000 0.000 0.000 0.000 0.001 0.076

0.000 0.000 0.077 0.000 0.005 0.003 0.007 0.007 0.001 0.077

0.001 0.001 0.000 0.003 0.000 0.000 0.000 0.001 0.000 0.000

0.076 0.001 0.077 0.000 0.000 0.001 0.000 0.000 0.006 0.002

0.005 0.006

>Hypothetical 295

0.000 0.000 0.001 0.001 0.000 0.001 0.066 0.000 0.000 0.066

0.000 0.000 0.066 0.000 0.002 0.001 0.066 0.000 0.000 0.000

0.002 0.004 0.005 0.007 0.001 0.000 0.000 0.001 0.000 0.001

0.066 0.000 0.001 0.000 0.001 0.066 0.000 0.001 0.066 0.000

0.066 0.066 0.001 0.000 0.003 0.004 0.004 0.006 0.066 0.001

0.000 0.000 0.000 0.001 0.066 0.066 0.001 0.000 0.000 0.000

0.000 0.001 0.001 0.066 0.066 0.000 0.000 0.000 0.004 0.003

0.007 0.005

>Hypothetical 296

0.005 0.000 0.000 0.006 0.000 0.017 0.000 0.000 0.000 0.000

0.000 0.000 0.025 0.000 0.006 0.000 0.006 0.006 0.000 0.000

0.061 0.059 0.068 0.074 0.005 0.000 0.005 0.005 0.000 0.005

0.016 0.000 0.005 0.005 0.000 0.000 0.005 0.000 0.005 0.011

0.000 0.000 0.000 0.000 0.066 0.071 0.055 0.073 0.010 0.000

0.000 0.000 0.000 0.005 0.000 0.005 0.000 0.000 0.000 0.005

0.000 0.000 0.015 0.000 0.016 0.000 0.005 0.000 0.069 0.079

0.049 0.075

>Hypothetical 297

0.004 0.000 0.000 0.000 0.000 0.007 0.000 0.083 0.000 0.000

0.000 0.083 0.001 0.000 0.001 0.000 0.083 0.000 0.000 0.000

0.005 0.004 0.002 0.004 0.002 0.000 0.000 0.083 0.000 0.003

0.083 0.000 0.000 0.001 0.000 0.000 0.001 0.000 0.001 0.000

0.083 0.083 0.000 0.000 0.006 0.007 0.001 0.006 0.001 0.000

0.000 0.000 0.000 0.002 0.083 0.000 0.083 0.000 0.000 0.000

0.000 0.000 0.002 0.083 0.083 0.000 0.000 0.000 0.008 0.005

0.007 0.004

>Hypothetical 298

0.000 0.000 0.125 0.000 0.001 0.000 0.000 0.125 0.002 0.002

0.002 0.000 0.000 0.000 0.001 0.000 0.001 0.000 0.000 0.000

0.005 0.010 0.012 0.007 0.000 0.001 0.000 0.000 0.001 0.125

0.000 0.000 0.000 0.001 0.000 0.000 0.124 0.001 0.000 0.002

0.125 0.001 0.001 0.000 0.009 0.010 0.006 0.010 0.001 0.000

0.002 0.001 0.000 0.001 0.125 0.000 0.000 0.000 0.000 0.000

0.001 0.125 0.000 0.002 0.000 0.001 0.000 0.001 0.006 0.009

0.009 0.009

>Hypothetical 299

0.001 0.000 0.001 0.000 0.001 0.001 0.001 0.000 0.001 0.143

0.000 0.000 0.001 0.000 0.001 0.002 0.001 0.001 0.000 0.000

0.008 0.010 0.009 0.011 0.001 0.000 0.000 0.000 0.001 0.001

0.001 0.000 0.001 0.003 0.001 0.000 0.001 0.143 0.001 0.143

0.001 0.000 0.000 0.000 0.008 0.009 0.010 0.009 0.001 0.001

0.000 0.001 0.001 0.143 0.000 0.001 0.000 0.000 0.000 0.000

0.143 0.001 0.001 0.001 0.000 0.000 0.001 0.143 0.007 0.012

0.011 0.010

>Hypothetical 300

0.002 0.002 0.000 0.000 0.077 0.077 0.000 0.000 0.000 0.000

0.000 0.077 0.000 0.077 0.000 0.000 0.000 0.000 0.001 0.000

0.004 0.006 0.004 0.006 0.000 0.001 0.000 0.000 0.000 0.001

0.000 0.077 0.000 0.077 0.000 0.001 0.001 0.001 0.000 0.000

0.000 0.001 0.077 0.000 0.007 0.002 0.007 0.005 0.000 0.000

0.077 0.077 0.000 0.000 0.000 0.001 0.000 0.001 0.001 0.001

0.077 0.077 0.002 0.077 0.000 0.001 0.000 0.000 0.004 0.002

0.006 0.007

>Hypothetical 301

0.077 0.001 0.001 0.000 0.000 0.076 0.000 0.000 0.000 0.076

0.000 0.001 0.076 0.001 0.001 0.001 0.076 0.076 0.000 0.000

0.008 0.004 0.008 0.005 0.002 0.000 0.000 0.000 0.000 0.002

0.000 0.000 0.000 0.001 0.001 0.000 0.077 0.076 0.001 0.002

0.002 0.000 0.000 0.000 0.006 0.003 0.003 0.003 0.001 0.001

0.000 0.000 0.000 0.077 0.000 0.000 0.000 0.000 0.000 0.000

0.076 0.001 0.001 0.076 0.000 0.000 0.001 0.077 0.005 0.004

0.005 0.009

>Hypothetical 302

0.002 0.001 0.000 0.000 0.000 0.000 0.001 0.000 0.000 0.143

0.002 0.001 0.003 0.000 0.001 0.001 0.001 0.000 0.000 0.000

0.008 0.010 0.009 0.009 0.001 0.000 0.000 0.000 0.000 0.143

0.001 0.001 0.000 0.143 0.000 0.143 0.002 0.000 0.001 0.000

0.001 0.001 0.001 0.000 0.011 0.009 0.011 0.009 0.001 0.000

0.001 0.143 0.000 0.001 0.001 0.143 0.000 0.000 0.001 0.000

0.002 0.000 0.001 0.001 0.001 0.001 0.000 0.001 0.006 0.010

0.013 0.010

>Hypothetical 303

0.006 0.002 0.000 0.083 0.000 0.004 0.083 0.000 0.082 0.000

0.002 0.000 0.081 0.000 0.000 0.003 0.002 0.083 0.000 0.000

0.003 0.004 0.003 0.004 0.002 0.000 0.000 0.001 0.000 0.001

0.000 0.000 0.000 0.000 0.000 0.000 0.001 0.083 0.000 0.083

0.083 0.000 0.001 0.000 0.009 0.007 0.003 0.004 0.083 0.001

0.001 0.000 0.000 0.001 0.001 0.000 0.083 0.001 0.000 0.000

0.083 0.001 0.001 0.000 0.000 0.000 0.000 0.000 0.009 0.005

0.004 0.006

>Hypothetical 304

0.002 0.000 0.000 0.000 0.000 0.197 0.003 0.000 0.000 0.001

0.000 0.000 0.002 0.000 0.003 0.000 0.001 0.000 0.000 0.000

0.016 0.014 0.010 0.018 0.003 0.000 0.000 0.200 0.000 0.198

0.000 0.000 0.000 0.001 0.000 0.000 0.001 0.000 0.005 0.001

0.003 0.000 0.000 0.000 0.020 0.012 0.010 0.014 0.003 0.000

0.000 0.000 0.000 0.196 0.001 0.000 0.000 0.000 0.000 0.000

0.003 0.000 0.003 0.001 0.000 0.000 0.000 0.000 0.016 0.014

0.011 0.018

>Hypothetical 305

0.002 0.001 0.000 0.000 0.000 0.002 0.002 0.000 0.000 0.125

0.000 0.000 0.002 0.000 0.125 0.001 0.001 0.000 0.001 0.000

0.008 0.008 0.006 0.008 0.001 0.000 0.000 0.000 0.000 0.001

0.001 0.000 0.000 0.125 0.000 0.125 0.001 0.001 0.002 0.000

0.001 0.001 0.000 0.000 0.010 0.006 0.007 0.011 0.000 0.001

0.000 0.125 0.000 0.002 0.001 0.125 0.001 0.001 0.000 0.000

0.002 0.000 0.000 0.125 0.001 0.000 0.000 0.000 0.008 0.007

0.010 0.010

>Hypothetical 306

0.012 0.018 0.000 0.000 0.000 0.012 0.000 0.000 0.000 0.000

0.006 0.000 0.000 0.006 0.006 0.000 0.000 0.000 0.000 0.000

0.071 0.052 0.074 0.075 0.012 0.008 0.000 0.000 0.000 0.000

0.000 0.000 0.004 0.000 0.000 0.000 0.000 0.000 0.008 0.000

0.000 0.000 0.008 0.000 0.084 0.064 0.075 0.068 0.006 0.011

0.000 0.000 0.000 0.011 0.006 0.000 0.000 0.000 0.000 0.000

0.000 0.000 0.011 0.006 0.000 0.000 0.000 0.000 0.083 0.051

0.094 0.053

>Hypothetical 307

0.002 0.001 0.001 0.000 0.000 0.002 0.000 0.000 0.001 0.000

0.000 0.000 0.001 0.111 0.111 0.000 0.000 0.000 0.001 0.000

0.008 0.009 0.005 0.007 0.000 0.000 0.001 0.001 0.110 0.001

0.111 0.000 0.000 0.001 0.000 0.001 0.002 0.000 0.001 0.001

0.001 0.001 0.000 0.000 0.006 0.008 0.009 0.006 0.110 0.111

0.000 0.001 0.000 0.002 0.000 0.000 0.001 0.001 0.001 0.000

0.001 0.000 0.002 0.110 0.110 0.000 0.001 0.000 0.008 0.007

0.010 0.006

>Hypothetical 308

0.001 0.000 0.000 0.000 0.000 0.001 0.000 0.000 0.000 0.001

0.000 0.000 0.004 0.091 0.001 0.001 0.001 0.090 0.000 0.000

0.006 0.007 0.007 0.001 0.002 0.000 0.091 0.091 0.090 0.001

0.091 0.000 0.000 0.091 0.000 0.000 0.003 0.002 0.001 0.091

0.000 0.000 0.000 0.000 0.007 0.007 0.007 0.003 0.002 0.091

0.000 0.000 0.000 0.000 0.000 0.000 0.000 0.001 0.000 0.001

0.003 0.001 0.001 0.000 0.090 0.000 0.000 0.000 0.009 0.007

0.007 0.001

>Hypothetical 309

0.006 0.001 0.000 0.000 0.001 0.000 0.000 0.000 0.000 0.003

0.001 0.077 0.000 0.000 0.000 0.000 0.076 0.001 0.076 0.000

0.005 0.002 0.002 0.006 0.077 0.001 0.000 0.000 0.000 0.076

0.000 0.000 0.000 0.000 0.000 0.000 0.001 0.077 0.001 0.003

0.077 0.077 0.000 0.000 0.006 0.005 0.005 0.006 0.000 0.001

0.000 0.000 0.000 0.000 0.077 0.000 0.077 0.000 0.000 0.000

0.001 0.000 0.002 0.000 0.000 0.001 0.077 0.077 0.005 0.009

0.006 0.002

>Hypothetical 310

0.003 0.000 0.001 0.000 0.000 0.001 0.000 0.143 0.143 0.143

0.000 0.000 0.001 0.000 0.002 0.001 0.000 0.000 0.000 0.000

0.010 0.010 0.011 0.008 0.001 0.000 0.000 0.002 0.000 0.002

0.000 0.000 0.000 0.001 0.000 0.000 0.002 0.000 0.001 0.142

0.001 0.000 0.000 0.000 0.012 0.009 0.011 0.007 0.002 0.000

0.000 0.001 0.000 0.002 0.143 0.000 0.000 0.001 0.000 0.000

0.002 0.143 0.001 0.002 0.000 0.000 0.000 0.000 0.012 0.006

0.011 0.009

>Hypothetical 311

0.001 0.059 0.059 0.000 0.000 0.002 0.000 0.000 0.059 0.000

0.000 0.000 0.001 0.000 0.001 0.000 0.000 0.059 0.059 0.000

0.005 0.005 0.001 0.004 0.001 0.000 0.059 0.000 0.000 0.002

0.000 0.000 0.000 0.000 0.000 0.059 0.059 0.000 0.001 0.059

0.059 0.059 0.000 0.000 0.005 0.005 0.004 0.003 0.002 0.000

0.000 0.059 0.000 0.003 0.000 0.000 0.059 0.059 0.059 0.000

0.001 0.000 0.001 0.001 0.000 0.000 0.059 0.000 0.005 0.004

0.003 0.001

>Hypothetical 312

0.001 0.000 0.001 0.000 0.001 0.000 0.001 0.000 0.000 0.200

0.000 0.000 0.004 0.000 0.002 0.000 0.200 0.000 0.000 0.000

0.015 0.011 0.013 0.018 0.001 0.001 0.000 0.000 0.000 0.000

0.000 0.000 0.000 0.200 0.000 0.000 0.001 0.000 0.004 0.001

0.001 0.001 0.000 0.000 0.014 0.013 0.014 0.017 0.004 0.000

0.000 0.000 0.000 0.001 0.200 0.000 0.000 0.000 0.000 0.000

0.001 0.000 0.003 0.001 0.000 0.001 0.000 0.000 0.012 0.011

0.011 0.022

>Hypothetical 313

0.001 0.000 0.110 0.000 0.000 0.002 0.000 0.000 0.110 0.110

0.000 0.001 0.109 0.000 0.003 0.000 0.001 0.000 0.000 0.000

0.006 0.014 0.008 0.004 0.002 0.000 0.000 0.002 0.000 0.001

0.001 0.000 0.000 0.000 0.000 0.000 0.111 0.000 0.000 0.110

0.001 0.001 0.000 0.000 0.007 0.010 0.008 0.005 0.000 0.000

0.000 0.000 0.000 0.002 0.002 0.000 0.001 0.000 0.000 0.000

0.110 0.111 0.001 0.004 0.001 0.000 0.000 0.000 0.005 0.007

0.007 0.008

>Hypothetical 314

0.002 0.000 0.000 0.077 0.000 0.005 0.077 0.000 0.077 0.002

0.000 0.000 0.002 0.000 0.000 0.000 0.001 0.077 0.000 0.000

0.003 0.003 0.004 0.004 0.002 0.000 0.000 0.000 0.000 0.000

0.000 0.000 0.000 0.077 0.000 0.000 0.001 0.077 0.000 0.077

0.077 0.000 0.001 0.000 0.006 0.005 0.006 0.004 0.077 0.000

0.000 0.077 0.000 0.001 0.000 0.000 0.077 0.000 0.000 0.000

0.001 0.000 0.002 0.077 0.000 0.000 0.000 0.000 0.005 0.006

0.006 0.005

>Hypothetical 315

0.001 0.000 0.142 0.002 0.142 0.003 0.001 0.000 0.003 0.000

0.000 0.000 0.006 0.000 0.142 0.000 0.000 0.002 0.000 0.000

0.006 0.010 0.008 0.009 0.002 0.142 0.000 0.000 0.000 0.000

0.000 0.000 0.000 0.001 0.000 0.000 0.002 0.001 0.004 0.001

0.001 0.142 0.000 0.000 0.003 0.009 0.013 0.012 0.000 0.000

0.000 0.001 0.000 0.001 0.000 0.000 0.001 0.000 0.000 0.000

0.001 0.000 0.003 0.001 0.000 0.142 0.000 0.000 0.002 0.007

0.014 0.016

>Hypothetical 316

0.003 0.001 0.001 0.000 0.000 0.003 0.001 0.000 0.077 0.000

0.000 0.000 0.076 0.000 0.077 0.000 0.000 0.001 0.077 0.000

0.005 0.003 0.004 0.005 0.002 0.000 0.077 0.077 0.000 0.001

0.000 0.001 0.000 0.001 0.000 0.077 0.002 0.000 0.000 0.077

0.001 0.077 0.001 0.000 0.005 0.004 0.004 0.006 0.000 0.000

0.000 0.077 0.000 0.001 0.000 0.000 0.077 0.000 0.077 0.000

0.000 0.000 0.002 0.000 0.000 0.000 0.000 0.000 0.006 0.007

0.004 0.006

>Hypothetical 317

0.083 0.000 0.083 0.083 0.000 0.001 0.001 0.000 0.000 0.000

0.000 0.000 0.001 0.000 0.002 0.083 0.000 0.001 0.000 0.000

0.007 0.003 0.010 0.004 0.004 0.000 0.001 0.082 0.000 0.004

0.000 0.000 0.083 0.083 0.001 0.000 0.001 0.083 0.001 0.001

0.000 0.000 0.082 0.000 0.006 0.006 0.004 0.002 0.001 0.083

0.000 0.000 0.000 0.000 0.000 0.000 0.083 0.000 0.000 0.000

0.002 0.000 0.001 0.000 0.001 0.000 0.001 0.000 0.008 0.003

0.008 0.003

>Hypothetical 318

0.003 0.000 0.000 0.001 0.002 0.003 0.000 0.000 0.000 0.000

0.000 0.000 0.143 0.000 0.001 0.000 0.001 0.000 0.001 0.000

0.011 0.010 0.007 0.009 0.143 0.001 0.000 0.002 0.000 0.002

0.001 0.000 0.000 0.143 0.000 0.000 0.143 0.002 0.001 0.002

0.000 0.000 0.000 0.000 0.010 0.009 0.010 0.009 0.143 0.000

0.000 0.000 0.000 0.003 0.000 0.001 0.001 0.001 0.000 0.001

0.143 0.000 0.002 0.001 0.000 0.000 0.000 0.000 0.010 0.012

0.009 0.008

>Hypothetical 319

0.002 0.100 0.001 0.000 0.000 0.001 0.100 0.001 0.001 0.001

0.000 0.000 0.002 0.000 0.001 0.000 0.000 0.100 0.000 0.000

0.008 0.005 0.006 0.007 0.100 0.000 0.000 0.000 0.000 0.002

0.000 0.100 0.000 0.100 0.000 0.000 0.003 0.000 0.001 0.001

0.001 0.000 0.100 0.000 0.007 0.006 0.007 0.006 0.001 0.000

0.000 0.000 0.000 0.001 0.000 0.000 0.000 0.100 0.000 0.000

0.001 0.000 0.002 0.000 0.000 0.000 0.100 0.000 0.008 0.006

0.007 0.008

>Hypothetical 320

0.002 0.001 0.000 0.000 0.000 0.002 0.000 0.125 0.000 0.000

0.001 0.001 0.000 0.000 0.124 0.000 0.002 0.000 0.002 0.000

0.007 0.008 0.010 0.007 0.002 0.000 0.000 0.124 0.000 0.003

0.125 0.000 0.000 0.001 0.000 0.000 0.002 0.001 0.001 0.000

0.001 0.001 0.001 0.000 0.008 0.008 0.007 0.008 0.125 0.000

0.000 0.000 0.000 0.002 0.000 0.000 0.001 0.001 0.000 0.000

0.000 0.000 0.001 0.124 0.125 0.000 0.001 0.001 0.010 0.008

0.009 0.010

>Hypothetical 321

0.003 0.067 0.000 0.001 0.000 0.066 0.066 0.000 0.000 0.001

0.000 0.000 0.000 0.001 0.001 0.000 0.067 0.001 0.067 0.000

0.005 0.004 0.003 0.003 0.067 0.000 0.000 0.000 0.000 0.000

0.067 0.000 0.000 0.000 0.000 0.000 0.002 0.067 0.001 0.001

0.067 0.067 0.000 0.000 0.005 0.005 0.003 0.005 0.001 0.000

0.000 0.000 0.000 0.001 0.067 0.000 0.067 0.067 0.000 0.000

0.000 0.000 0.001 0.000 0.067 0.000 0.000 0.000 0.004 0.006

0.005 0.004

>Hypothetical 322

0.001 0.000 0.000 0.000 0.002 0.002 0.006 0.004 0.000 0.001

0.000 0.000 0.249 0.000 0.002 0.000 0.001 0.000 0.000 0.000

0.018 0.018 0.011 0.019 0.004 0.002 0.000 0.000 0.000 0.002

0.002 0.000 0.000 0.002 0.000 0.000 0.250 0.000 0.000 0.004

0.009 0.000 0.000 0.000 0.018 0.017 0.009 0.013 0.002 0.002

0.002 0.000 0.000 0.000 0.003 0.000 0.000 0.002 0.000 0.000

0.004 0.000 0.000 0.250 0.002 0.000 0.000 0.000 0.015 0.014

0.022 0.015

>Hypothetical 323

0.001 0.090 0.000 0.001 0.000 0.000 0.001 0.000 0.000 0.001

0.090 0.000 0.000 0.000 0.001 0.001 0.090 0.000 0.001 0.000

0.005 0.007 0.007 0.007 0.001 0.000 0.000 0.000 0.000 0.003

0.000 0.000 0.000 0.001 0.000 0.000 0.000 0.090 0.001 0.001

0.002 0.090 0.001 0.000 0.007 0.005 0.005 0.005 0.002 0.000

0.000 0.001 0.000 0.002 0.090 0.000 0.090 0.090 0.000 0.000

0.000 0.002 0.001 0.000 0.000 0.000 0.090 0.090 0.000 0.007

0.009 0.008

>Hypothetical 324

0.007 0.000 0.000 0.000 0.000 0.000 0.003 0.000 0.000 0.002

0.000 0.000 0.001 0.000 0.005 0.000 0.002 0.000 0.000 0.000

0.031 0.011 0.019 0.030 0.001 0.000 0.000 0.000 0.000 0.002

0.002 0.000 0.000 0.002 0.000 0.002 0.002 0.000 0.005 0.333

0.004 0.002 0.000 0.000 0.027 0.022 0.017 0.022 0.004 0.000

0.000 0.002 0.000 0.000 0.002 0.002 0.000 0.000 0.000 0.000

0.332 0.000 0.005 0.004 0.002 0.000 0.000 0.000 0.021 0.020

0.015 0.036

>Hypothetical 325

0.001 0.050 0.000 0.000 0.000 0.000 0.050 0.000 0.000 0.000

0.049 0.050 0.050 0.000 0.001 0.050 0.050 0.001 0.000 0.000

0.004 0.003 0.003 0.005 0.001 0.000 0.000 0.000 0.001 0.000

0.000 0.050 0.000 0.000 0.000 0.000 0.050 0.000 0.001 0.000

0.050 0.050 0.050 0.000 0.003 0.003 0.003 0.004 0.001 0.000

0.050 0.000 0.000 0.050 0.050 0.000 0.000 0.000 0.050 0.000

0.050 0.000 0.001 0.001 0.001 0.050 0.000 0.050 0.003 0.003

0.004 0.004

>Hypothetical 326

0.001 0.000 0.066 0.000 0.000 0.001 0.001 0.000 0.000 0.001

0.000 0.000 0.066 0.000 0.000 0.001 0.001 0.066 0.066 0.000

0.004 0.006 0.005 0.003 0.001 0.000 0.000 0.066 0.000 0.066

0.001 0.000 0.000 0.000 0.000 0.066 0.001 0.000 0.001 0.066

0.000 0.066 0.000 0.000 0.006 0.005 0.003 0.005 0.001 0.000

0.001 0.067 0.000 0.000 0.001 0.001 0.067 0.000 0.067 0.000

0.067 0.001 0.001 0.000 0.001 0.000 0.067 0.000 0.006 0.004

0.004 0.004

>Hypothetical 327

0.004 0.000 0.000 0.123 0.000 0.005 0.003 0.000 0.000 0.004

0.000 0.000 0.002 0.124 0.124 0.002 0.003 0.004 0.000 0.000

0.005 0.003 0.005 0.006 0.003 0.000 0.000 0.000 0.000 0.001

0.000 0.000 0.000 0.002 0.000 0.000 0.002 0.002 0.125 0.001

0.002 0.000 0.124 0.000 0.007 0.005 0.011 0.007 0.123 0.124

0.000 0.000 0.000 0.001 0.000 0.000 0.000 0.000 0.000 0.000

0.005 0.001 0.001 0.003 0.000 0.000 0.000 0.001 0.013 0.011

0.006 0.002

>Hypothetical 328

0.071 0.000 0.000 0.000 0.001 0.001 0.000 0.001 0.000 0.000

0.071 0.071 0.001 0.000 0.001 0.001 0.071 0.001 0.071 0.000

0.004 0.004 0.005 0.006 0.000 0.000 0.000 0.000 0.001 0.000

0.001 0.000 0.000 0.002 0.000 0.000 0.071 0.071 0.001 0.001

0.071 0.002 0.071 0.000 0.006 0.004 0.004 0.005 0.000 0.000

0.000 0.000 0.001 0.071 0.071 0.000 0.071 0.001 0.000 0.000

0.000 0.000 0.000 0.001 0.000 0.000 0.000 0.071 0.005 0.005

0.005 0.005

>Hypothetical 329

0.002 0.050 0.000 0.000 0.000 0.000 0.050 0.000 0.000 0.001

0.050 0.050 0.001 0.001 0.000 0.050 0.050 0.000 0.000 0.000

0.003 0.003 0.003 0.003 0.050 0.000 0.000 0.000 0.000 0.000

0.050 0.050 0.000 0.002 0.000 0.000 0.000 0.000 0.050 0.000

0.050 0.050 0.050 0.000 0.003 0.003 0.003 0.003 0.000 0.001

0.050 0.000 0.000 0.050 0.050 0.000 0.000 0.000 0.050 0.000

0.000 0.000 0.000 0.001 0.001 0.050 0.000 0.050 0.003 0.004

0.004 0.003

>Hypothetical 330

0.066 0.066 0.000 0.001 0.000 0.001 0.066 0.000 0.000 0.000

0.000 0.000 0.001 0.001 0.001 0.000 0.066 0.000 0.000 0.001

0.004 0.006 0.006 0.003 0.067 0.001 0.001 0.000 0.000 0.000

0.001 0.001 0.000 0.001 0.000 0.000 0.066 0.066 0.000 0.000

0.066 0.066 0.000 0.000 0.005 0.003 0.004 0.007 0.066 0.066

0.000 0.000 0.000 0.002 0.066 0.002 0.066 0.001 0.000 0.000

0.066 0.000 0.001 0.001 0.002 0.000 0.000 0.000 0.004 0.004

0.002 0.006

>Methylation code ID 381

0.000 0.000 0.000 0.000 0.005 0.015 0.005 0.000 0.005 0.000

0.000 0.005 0.000 0.005 0.005 0.000 0.000 0.005 0.005 0.005

0.082 0.081 0.046 0.064 0.012 0.000 0.000 0.012 0.006 0.006

0.000 0.000 0.006 0.006 0.006 0.000 0.000 0.000 0.006 0.006

0.012 0.000 0.000 0.000 0.065 0.075 0.055 0.061 0.000 0.000

0.000 0.006 0.000 0.006 0.000 0.000 0.000 0.011 0.000 0.000

0.006 0.006 0.011 0.006 0.000 0.006 0.006 0.000 0.081 0.058

0.048 0.084

>Methylation code ID 381

0.010 0.005 0.005 0.005 0.005 0.005 0.000 0.000 0.010 0.000

0.000 0.000 0.000 0.000 0.005 0.005 0.010 0.000 0.000 0.000

0.075 0.068 0.070 0.059 0.006 0.000 0.000 0.006 0.000 0.006

0.000 0.000 0.006 0.011 0.006 0.000 0.006 0.006 0.011 0.006

0.000 0.006 0.000 0.000 0.074 0.065 0.069 0.054 0.000 0.005

0.000 0.000 0.000 0.005 0.005 0.000 0.005 0.005 0.000 0.000

0.000 0.011 0.011 0.000 0.000 0.005 0.000 0.005 0.086 0.078

0.052 0.057

>Methylation code ID 381

0.004 0.004 0.009 0.000 0.004 0.000 0.000 0.004 0.009 0.004

0.000 0.004 0.000 0.000 0.004 0.004 0.004 0.000 0.000 0.000

0.068 0.076 0.064 0.069 0.007 0.000 0.000 0.000 0.000 0.007

0.000 0.000 0.007 0.007 0.020 0.000 0.007 0.000 0.007 0.013

0.000 0.007 0.000 0.000 0.065 0.080 0.067 0.043 0.000 0.007

0.007 0.014 0.000 0.000 0.007 0.000 0.000 0.007 0.000 0.000

0.007 0.014 0.007 0.000 0.000 0.000 0.000 0.000 0.070 0.087

0.054 0.054

>Methylation code ID 381

0.007 0.000 0.000 0.000 0.000 0.007 0.000 0.000 0.013 0.007

0.020 0.000 0.000 0.000 0.007 0.013 0.000 0.007 0.000 0.000

0.070 0.075 0.067 0.042 0.000 0.006 0.006 0.013 0.000 0.000

0.006 0.000 0.000 0.006 0.000 0.006 0.006 0.006 0.006 0.000

0.000 0.000 0.000 0.000 0.068 0.080 0.054 0.067 0.004 0.004

0.009 0.004 0.000 0.000 0.000 0.009 0.009 0.004 0.000 0.004

0.000 0.000 0.004 0.000 0.000 0.000 0.000 0.000 0.067 0.080

0.065 0.069

>Methylation code ID 381

0.004 0.000 0.009 0.000 0.004 0.004 0.000 0.004 0.000 0.000

0.000 0.000 0.000 0.000 0.013 0.009 0.000 0.000 0.004 0.000

0.087 0.060 0.074 0.061 0.000 0.000 0.014 0.014 0.000 0.007

0.000 0.014 0.000 0.021 0.000 0.007 0.000 0.007 0.000 0.007

0.000 0.000 0.000 0.000 0.076 0.034 0.075 0.056 0.000 0.000

0.000 0.000 0.000 0.000 0.000 0.017 0.008 0.017 0.000 0.008

0.000 0.008 0.000 0.008 0.008 0.008 0.008 0.000 0.050 0.052

0.068 0.070

>Methylation code ID 381

0.000 0.000 0.000 0.000 0.000 0.000 0.000 0.014 0.007 0.007

0.000 0.014 0.000 0.007 0.000 0.014 0.007 0.007 0.007 0.000

0.058 0.067 0.067 0.057 0.005 0.000 0.000 0.000 0.005 0.005

0.000 0.005 0.000 0.000 0.000 0.000 0.005 0.000 0.020 0.010

0.005 0.000 0.000 0.000 0.096 0.051 0.090 0.036 0.000 0.000

0.013 0.020 0.000 0.000 0.000 0.000 0.000 0.020 0.000 0.007

0.000 0.007 0.007 0.007 0.000 0.000 0.000 0.000 0.091 0.028

0.094 0.040

>Methylation code ID 381

0.000 0.000 0.011 0.011 0.000 0.011 0.000 0.000 0.000 0.043

0.021 0.000 0.000 0.000 0.000 0.011 0.000 0.011 0.000 0.000

0.043 0.061 0.049 0.063 0.000 0.006 0.000 0.000 0.011 0.000

0.006 0.006 0.011 0.000 0.000 0.006 0.000 0.000 0.000 0.006

0.000 0.006 0.000 0.000 0.081 0.057 0.075 0.064 0.005 0.000

0.005 0.000 0.005 0.000 0.000 0.005 0.000 0.010 0.000 0.000

0.000 0.000 0.015 0.010 0.000 0.000 0.000 0.005 0.061 0.063

0.074 0.075

>Methylation code ID 776

0.000 0.000 0.000 0.000 0.000 0.016 0.000 0.008 0.000 0.024

0.000 0.000 0.008 0.000 0.008 0.008 0.008 0.008 0.000 0.008

0.076 0.061 0.052 0.051 0.014 0.000 0.000 0.000 0.007 0.007

0.000 0.000 0.000 0.007 0.000 0.000 0.014 0.000 0.000 0.021

0.007 0.000 0.007 0.007 0.064 0.072 0.041 0.066 0.000 0.000

0.000 0.000 0.000 0.005 0.005 0.005 0.000 0.005 0.000 0.000

0.015 0.000 0.010 0.005 0.000 0.005 0.000 0.005 0.073 0.069

0.050 0.082

>Methylation code ID 776

0.014 0.000 0.000 0.000 0.000 0.014 0.000 0.000 0.000 0.007

0.000 0.000 0.007 0.000 0.000 0.021 0.007 0.000 0.007 0.014

0.062 0.076 0.039 0.065 0.011 0.000 0.000 0.000 0.000 0.006

0.006 0.006 0.000 0.006 0.000 0.000 0.011 0.000 0.011 0.000

0.006 0.006 0.006 0.000 0.074 0.066 0.044 0.076 0.000 0.000

0.000 0.000 0.000 0.021 0.000 0.007 0.000 0.014 0.000 0.000

0.007 0.000 0.007 0.007 0.000 0.007 0.000 0.007 0.065 0.073

0.058 0.060

>Methylation code ID 776

0.000 0.004 0.004 0.000 0.004 0.012 0.000 0.000 0.000 0.000

0.000 0.000 0.008 0.004 0.012 0.000 0.000 0.000 0.000 0.000

0.069 0.090 0.068 0.057 0.016 0.000 0.008 0.008 0.000 0.024

0.000 0.000 0.000 0.008 0.000 0.000 0.008 0.008 0.000 0.008

0.008 0.008 0.000 0.000 0.050 0.063 0.054 0.061 0.006 0.000

0.000 0.006 0.000 0.006 0.000 0.000 0.000 0.006 0.000 0.000

0.006 0.006 0.012 0.006 0.006 0.000 0.006 0.000 0.077 0.070

0.074 0.049

>Methylation code ID 776

0.015 0.000 0.008 0.008 0.000 0.023 0.000 0.000 0.000 0.000

0.000 0.008 0.008 0.008 0.000 0.008 0.008 0.008 0.000 0.000

0.059 0.064 0.053 0.059 0.005 0.000 0.000 0.005 0.000 0.005

0.000 0.000 0.000 0.005 0.000 0.000 0.005 0.005 0.015 0.005

0.010 0.000 0.000 0.000 0.081 0.056 0.078 0.057 0.000 0.005

0.005 0.005 0.000 0.010 0.000 0.000 0.000 0.000 0.000 0.000

0.010 0.005 0.015 0.000 0.000 0.000 0.000 0.000 0.079 0.102

0.053 0.045

>Methylation code ID 776

0.006 0.000 0.012 0.012 0.000 0.012 0.000 0.000 0.000 0.000

0.000 0.000 0.012 0.000 0.006 0.012 0.000 0.000 0.000 0.000

0.073 0.073 0.044 0.071 0.007 0.000 0.000 0.014 0.000 0.007

0.000 0.000 0.007 0.007 0.000 0.007 0.007 0.014 0.000 0.000

0.014 0.000 0.000 0.000 0.069 0.060 0.055 0.069 0.000 0.000

0.000 0.000 0.000 0.005 0.000 0.000 0.005 0.010 0.000 0.000

0.005 0.000 0.020 0.005 0.005 0.000 0.000 0.000 0.069 0.062

0.080 0.065

>Methylation code ID 776

0.000 0.000 0.007 0.007 0.000 0.007 0.000 0.007 0.007 0.013

0.000 0.000 0.007 0.007 0.000 0.000 0.013 0.000 0.007 0.000

0.051 0.059 0.068 0.075 0.000 0.005 0.000 0.000 0.000 0.010

0.000 0.000 0.010 0.010 0.000 0.000 0.010 0.000 0.005 0.000

0.005 0.000 0.000 0.005 0.053 0.068 0.071 0.083 0.010 0.000

0.005 0.000 0.000 0.005 0.005 0.000 0.000 0.000 0.005 0.005

0.005 0.000 0.010 0.010 0.000 0.000 0.000 0.000 0.070 0.071

0.052 0.079

>Methylation code ID 776

0.019 0.000 0.000 0.006 0.012 0.019 0.000 0.000 0.000 0.000

0.000 0.000 0.000 0.000 0.006 0.000 0.006 0.000 0.006 0.000

0.076 0.073 0.048 0.061 0.000 0.005 0.000 0.011 0.000 0.016

0.005 0.000 0.000 0.000 0.000 0.000 0.000 0.011 0.005 0.016

0.000 0.000 0.000 0.000 0.070 0.063 0.069 0.061 0.000 0.000

0.000 0.000 0.000 0.024 0.000 0.006 0.006 0.006 0.000 0.006

0.000 0.000 0.012 0.006 0.000 0.000 0.000 0.000 0.071 0.082

0.061 0.052

>Methylation code ID 776

0.006 0.000 0.000 0.000 0.000 0.023 0.000 0.006 0.000 0.006

0.000 0.006 0.006 0.000 0.012 0.006 0.000 0.000 0.000 0.000

0.077 0.088 0.060 0.039 0.021 0.000 0.000 0.007 0.014 0.014

0.000 0.000 0.000 0.007 0.000 0.000 0.007 0.000 0.000 0.000

0.007 0.000 0.007 0.000 0.064 0.074 0.051 0.059 0.000 0.006

0.000 0.006 0.000 0.012 0.006 0.000 0.000 0.000 0.000 0.000

0.000 0.012 0.006 0.024 0.000 0.000 0.000 0.000 0.065 0.065

0.064 0.069

>Methylation code ID 776

0.006 0.000 0.000 0.000 0.000 0.026 0.000 0.006 0.006 0.000

0.000 0.000 0.013 0.000 0.006 0.000 0.006 0.000 0.000 0.000

0.073 0.059 0.078 0.051 0.006 0.000 0.000 0.006 0.000 0.013

0.000 0.000 0.006 0.019 0.000 0.006 0.006 0.000 0.000 0.000

0.000 0.000 0.006 0.006 0.068 0.071 0.061 0.058 0.000 0.005

0.005 0.005 0.000 0.005 0.000 0.005 0.005 0.000 0.000 0.000

0.000 0.000 0.010 0.010 0.005 0.000 0.000 0.005 0.076 0.071

0.070 0.057

>Methylation code ID 776

0.000 0.006 0.006 0.000 0.000 0.018 0.006 0.000 0.006 0.006

0.000 0.006 0.000 0.000 0.000 0.006 0.012 0.006 0.000 0.000

0.072 0.069 0.050 0.066 0.016 0.005 0.000 0.000 0.000 0.005

0.000 0.000 0.000 0.000 0.000 0.000 0.000 0.005 0.011 0.000

0.016 0.005 0.000 0.000 0.076 0.070 0.071 0.051 0.000 0.000

0.005 0.000 0.000 0.010 0.005 0.000 0.005 0.000 0.000 0.000

0.005 0.005 0.016 0.005 0.000 0.000 0.000 0.000 0.063 0.071

0.079 0.063

>Methylation code ID 776

0.000 0.000 0.004 0.000 0.004 0.008 0.008 0.000 0.004 0.000

0.000 0.000 0.004 0.004 0.012 0.004 0.000 0.000 0.000 0.000

0.066 0.075 0.075 0.063 0.000 0.000 0.007 0.000 0.000 0.022

0.007 0.000 0.007 0.007 0.000 0.000 0.000 0.000 0.000 0.015

0.022 0.007 0.000 0.000 0.058 0.047 0.062 0.069 0.013 0.000

0.000 0.000 0.000 0.007 0.000 0.000 0.000 0.007 0.000 0.000

0.000 0.007 0.013 0.007 0.013 0.007 0.000 0.000 0.062 0.058

0.071 0.070

>Methylation code ID 776

0.000 0.000 0.008 0.000 0.000 0.024 0.008 0.000 0.000 0.008

0.000 0.000 0.000 0.000 0.000 0.016 0.024 0.008 0.000 0.008

0.056 0.048 0.057 0.066 0.015 0.000 0.000 0.000 0.000 0.007

0.000 0.000 0.000 0.007 0.000 0.000 0.007 0.007 0.007 0.007

0.022 0.007 0.000 0.000 0.062 0.065 0.058 0.060 0.000 0.000

0.005 0.000 0.005 0.005 0.009 0.000 0.005 0.005 0.000 0.000

0.005 0.000 0.014 0.005 0.000 0.000 0.000 0.000 0.064 0.073

0.068 0.074

>Methylation code ID 776

0.010 0.000 0.000 0.000 0.000 0.021 0.000 0.010 0.000 0.031

0.000 0.000 0.010 0.000 0.000 0.010 0.010 0.010 0.000 0.010

0.063 0.060 0.037 0.048 0.012 0.000 0.000 0.000 0.006 0.000

0.006 0.000 0.006 0.006 0.000 0.000 0.012 0.000 0.000 0.012

0.006 0.000 0.006 0.006 0.074 0.070 0.052 0.059 0.000 0.000

0.000 0.000 0.000 0.005 0.005 0.005 0.000 0.005 0.000 0.000

0.011 0.000 0.011 0.011 0.005 0.000 0.000 0.005 0.075 0.073

0.040 0.081

>Methylation code ID 861

0.005 0.000 0.000 0.005 0.000 0.005 0.005 0.000 0.010 0.005

0.000 0.000 0.005 0.000 0.010 0.010 0.000 0.000 0.000 0.005

0.062 0.067 0.057 0.081 0.005 0.000 0.005 0.005 0.000 0.000

0.005 0.000 0.005 0.005 0.000 0.000 0.005 0.000 0.005 0.011

0.011 0.005 0.000 0.000 0.069 0.068 0.064 0.062 0.005 0.000

0.000 0.000 0.005 0.005 0.000 0.005 0.005 0.000 0.000 0.000

0.010 0.005 0.010 0.000 0.005 0.000 0.000 0.000 0.052 0.073

0.083 0.068

>Methylation code ID 861

0.032 0.000 0.000 0.000 0.000 0.021 0.011 0.000 0.000 0.021

0.000 0.000 0.011 0.000 0.000 0.011 0.011 0.021 0.000 0.000

0.055 0.043 0.046 0.051 0.013 0.000 0.000 0.000 0.000 0.007

0.000 0.000 0.000 0.007 0.000 0.000 0.020 0.007 0.007 0.013

0.007 0.000 0.000 0.000 0.066 0.053 0.069 0.066 0.000 0.000

0.000 0.000 0.000 0.011 0.000 0.000 0.000 0.000 0.000 0.000

0.023 0.006 0.011 0.011 0.000 0.000 0.000 0.006 0.083 0.083

0.058 0.041

>Methylation code ID 862

0.000 0.006 0.000 0.000 0.000 0.000 0.006 0.006 0.006 0.000

0.000 0.013 0.000 0.013 0.000 0.013 0.006 0.000 0.006 0.000

0.067 0.070 0.059 0.061 0.020 0.000 0.000 0.000 0.000 0.010

0.000 0.000 0.010 0.000 0.000 0.000 0.010 0.000 0.000 0.030

0.030 0.010 0.000 0.000 0.052 0.067 0.040 0.054 0.000 0.000

0.008 0.000 0.000 0.000 0.000 0.000 0.000 0.000 0.000 0.008

0.017 0.034 0.008 0.008 0.000 0.000 0.000 0.000 0.070 0.070

0.063 0.046

>Methylation code ID 862

0.000 0.006 0.000 0.000 0.000 0.000 0.000 0.006 0.006 0.000

0.000 0.012 0.000 0.012 0.000 0.018 0.006 0.000 0.006 0.000

0.060 0.071 0.067 0.064 0.019 0.000 0.000 0.000 0.000 0.010

0.000 0.000 0.010 0.010 0.000 0.000 0.019 0.000 0.000 0.029

0.019 0.000 0.000 0.000 0.050 0.059 0.041 0.067 0.000 0.000

0.007 0.000 0.007 0.000 0.000 0.000 0.000 0.000 0.000 0.007

0.007 0.029 0.007 0.000 0.000 0.007 0.000 0.000 0.050 0.057

0.073 0.081

>Methylation code ID 862

0.006 0.006 0.000 0.006 0.000 0.000 0.006 0.006 0.006 0.000

0.000 0.000 0.000 0.006 0.000 0.018 0.012 0.000 0.006 0.000

0.069 0.067 0.065 0.056 0.008 0.000 0.000 0.000 0.000 0.008

0.000 0.000 0.000 0.015 0.000 0.008 0.030 0.000 0.000 0.023

0.008 0.000 0.000 0.000 0.064 0.052 0.045 0.076 0.000 0.000

0.000 0.000 0.005 0.000 0.005 0.005 0.000 0.000 0.000 0.000

0.011 0.005 0.011 0.000 0.000 0.005 0.000 0.005 0.075 0.077

0.047 0.081

>Methylation code ID 862

0.014 0.007 0.000 0.007 0.000 0.000 0.007 0.007 0.007 0.000

0.000 0.000 0.014 0.000 0.000 0.021 0.007 0.000 0.000 0.000

0.069 0.049 0.062 0.062 0.007 0.000 0.000 0.000 0.000 0.000

0.007 0.000 0.000 0.014 0.000 0.007 0.028 0.007 0.000 0.014

0.007 0.000 0.000 0.000 0.064 0.059 0.056 0.063 0.000 0.000

0.000 0.000 0.005 0.000 0.005 0.005 0.000 0.000 0.000 0.000

0.010 0.005 0.010 0.005 0.005 0.005 0.000 0.005 0.074 0.082

0.052 0.066

>Methylation code ID 862

0.000 0.000 0.008 0.008 0.000 0.000 0.008 0.000 0.000 0.008

0.000 0.000 0.015 0.015 0.000 0.008 0.008 0.000 0.008 0.000

0.060 0.062 0.070 0.058 0.000 0.000 0.000 0.000 0.000 0.005

0.005 0.000 0.005 0.000 0.000 0.000 0.010 0.000 0.005 0.010

0.010 0.000 0.010 0.005 0.063 0.068 0.067 0.068 0.012 0.000

0.000 0.000 0.000 0.012 0.000 0.006 0.000 0.000 0.000 0.000

0.018 0.000 0.012 0.000 0.006 0.000 0.000 0.000 0.081 0.071

0.031 0.086

>Methylation code ID 862

0.000 0.000 0.000 0.000 0.009 0.017 0.000 0.017 0.009 0.026

0.000 0.000 0.009 0.000 0.000 0.000 0.017 0.000 0.000 0.000

0.053 0.066 0.052 0.059 0.005 0.005 0.000 0.000 0.005 0.005

0.005 0.000 0.000 0.005 0.000 0.000 0.005 0.000 0.011 0.016

0.000 0.000 0.000 0.000 0.069 0.072 0.069 0.060 0.006 0.000

0.011 0.006 0.000 0.006 0.006 0.006 0.000 0.006 0.000 0.000

0.006 0.000 0.006 0.011 0.000 0.000 0.000 0.000 0.071 0.054

0.068 0.071

>Methylation code ID 862

0.011 0.000 0.006 0.017 0.000 0.000 0.006 0.000 0.000 0.000

0.000 0.006 0.006 0.000 0.006 0.006 0.000 0.000 0.006 0.000

0.061 0.072 0.059 0.074 0.000 0.000 0.000 0.007 0.000 0.014

0.000 0.000 0.014 0.000 0.007 0.007 0.007 0.014 0.000 0.000

0.014 0.007 0.000 0.000 0.058 0.065 0.064 0.057 0.000 0.000

0.000 0.007 0.000 0.007 0.000 0.000 0.007 0.013 0.000 0.000

0.000 0.000 0.013 0.020 0.007 0.000 0.000 0.000 0.072 0.041

0.089 0.059

>Methylation code ID 862

0.000 0.008 0.000 0.000 0.008 0.023 0.000 0.008 0.000 0.000

0.000 0.000 0.000 0.015 0.000 0.023 0.015 0.000 0.000 0.000

0.059 0.058 0.059 0.058 0.000 0.000 0.000 0.000 0.000 0.007

0.000 0.000 0.000 0.007 0.000 0.007 0.020 0.007 0.007 0.013

0.000 0.020 0.000 0.000 0.074 0.045 0.060 0.068 0.000 0.000

0.000 0.000 0.010 0.005 0.005 0.005 0.000 0.005 0.000 0.000

0.010 0.000 0.010 0.000 0.000 0.005 0.000 0.005 0.061 0.059

0.070 0.084

>Methylation code ID 862

0.000 0.006 0.000 0.000 0.000 0.006 0.006 0.006 0.000 0.000

0.000 0.000 0.013 0.006 0.006 0.013 0.013 0.006 0.000 0.000

0.062 0.051 0.075 0.062 0.014 0.000 0.000 0.000 0.007 0.014

0.007 0.000 0.000 0.000 0.000 0.000 0.014 0.014 0.000 0.014

0.007 0.000 0.000 0.000 0.046 0.065 0.064 0.067 0.000 0.000

0.000 0.000 0.000 0.006 0.012 0.000 0.000 0.006 0.000 0.006

0.012 0.006 0.006 0.006 0.006 0.006 0.000 0.000 0.074 0.055

0.050 0.079

>Methylation code ID 862

0.011 0.005 0.000 0.000 0.000 0.000 0.011 0.000 0.000 0.016

0.000 0.000 0.011 0.000 0.011 0.000 0.000 0.000 0.000 0.000

0.048 0.072 0.073 0.075 0.007 0.007 0.000 0.000 0.000 0.014

0.000 0.007 0.000 0.014 0.000 0.007 0.014 0.000 0.000 0.014

0.007 0.000 0.000 0.000 0.062 0.055 0.067 0.059 0.012 0.006

0.000 0.000 0.000 0.000 0.000 0.000 0.000 0.019 0.000 0.000

0.012 0.000 0.006 0.006 0.006 0.000 0.000 0.000 0.062 0.063

0.075 0.065

>Methylation code ID 863

0.004 0.004 0.000 0.000 0.000 0.008 0.004 0.000 0.008 0.004

0.000 0.000 0.000 0.000 0.008 0.000 0.004 0.000 0.004 0.004

0.052 0.072 0.076 0.084 0.008 0.000 0.000 0.000 0.000 0.033

0.000 0.000 0.008 0.008 0.000 0.000 0.000 0.008 0.000 0.008

0.017 0.008 0.008 0.000 0.054 0.056 0.047 0.068 0.010 0.000

0.005 0.000 0.000 0.005 0.000 0.000 0.005 0.010 0.000 0.000

0.000 0.005 0.010 0.000 0.000 0.005 0.000 0.000 0.080 0.053

0.052 0.090

>Methylation code ID 863

0.007 0.000 0.000 0.000 0.000 0.022 0.000 0.000 0.007 0.007

0.000 0.000 0.007 0.007 0.007 0.007 0.015 0.007 0.000 0.000

0.069 0.046 0.057 0.067 0.006 0.000 0.006 0.006 0.000 0.006

0.006 0.000 0.006 0.012 0.000 0.000 0.000 0.006 0.006 0.006

0.000 0.006 0.000 0.000 0.066 0.052 0.064 0.079 0.008 0.004

0.000 0.000 0.000 0.004 0.000 0.000 0.008 0.000 0.000 0.004

0.000 0.000 0.008 0.000 0.004 0.000 0.004 0.004 0.056 0.078

0.080 0.072

>Methylation code ID 863

0.021 0.007 0.000 0.007 0.000 0.007 0.007 0.000 0.000 0.007

0.000 0.000 0.014 0.007 0.000 0.000 0.007 0.007 0.000 0.000

0.050 0.064 0.068 0.061 0.006 0.000 0.000 0.000 0.006 0.006

0.000 0.006 0.000 0.011 0.000 0.000 0.017 0.006 0.006 0.006

0.000 0.000 0.000 0.006 0.070 0.065 0.064 0.062 0.000 0.000

0.000 0.000 0.000 0.012 0.006 0.000 0.000 0.006 0.000 0.000

0.000 0.000 0.012 0.017 0.006 0.006 0.006 0.000 0.081 0.061

0.079 0.044

>Methylation code ID 863

0.005 0.000 0.000 0.005 0.000 0.005 0.000 0.000 0.000 0.009

0.000 0.000 0.005 0.005 0.014 0.014 0.000 0.000 0.000 0.000

0.051 0.072 0.074 0.076 0.010 0.005 0.010 0.005 0.000 0.000

0.000 0.000 0.005 0.000 0.000 0.000 0.020 0.000 0.005 0.005

0.000 0.000 0.000 0.000 0.047 0.077 0.074 0.068 0.008 0.000

0.000 0.000 0.000 0.008 0.000 0.008 0.000 0.008 0.000 0.000

0.038 0.000 0.008 0.008 0.008 0.000 0.000 0.000 0.069 0.054

0.052 0.068

>Methylation code ID 875

0.006 0.000 0.000 0.000 0.011 0.006 0.000 0.000 0.000 0.006

0.000 0.011 0.006 0.000 0.006 0.006 0.011 0.006 0.000 0.000

0.067 0.064 0.072 0.058 0.006 0.000 0.000 0.000 0.000 0.000

0.000 0.006 0.000 0.000 0.000 0.000 0.006 0.012 0.012 0.017

0.006 0.006 0.006 0.000 0.070 0.060 0.082 0.046 0.005 0.000

0.000 0.000 0.005 0.005 0.011 0.000 0.005 0.005 0.000 0.000

0.011 0.005 0.005 0.000 0.000 0.005 0.000 0.000 0.072 0.078

0.046 0.071

>Methylation code ID 875

0.008 0.000 0.004 0.004 0.004 0.004 0.000 0.000 0.000 0.008

0.000 0.000 0.012 0.000 0.008 0.000 0.000 0.000 0.000 0.000

0.064 0.063 0.076 0.077 0.009 0.000 0.000 0.000 0.004 0.004

0.000 0.004 0.000 0.000 0.000 0.000 0.004 0.004 0.017 0.009

0.000 0.000 0.000 0.000 0.087 0.064 0.069 0.058 0.020 0.000

0.000 0.000 0.000 0.031 0.000 0.000 0.000 0.010 0.000 0.000

0.031 0.000 0.000 0.031 0.000 0.000 0.000 0.000 0.038 0.054

0.055 0.065

>Methylation code ID 897

0.024 0.008 0.008 0.000 0.000 0.016 0.000 0.000 0.000 0.024

0.000 0.000 0.000 0.000 0.000 0.000 0.024 0.000 0.000 0.000

0.061 0.050 0.061 0.060 0.010 0.000 0.000 0.000 0.000 0.005

0.005 0.000 0.000 0.005 0.000 0.000 0.010 0.005 0.005 0.005

0.005 0.000 0.010 0.000 0.082 0.068 0.071 0.045 0.000 0.005

0.005 0.000 0.000 0.016 0.011 0.000 0.000 0.011 0.005 0.000

0.005 0.000 0.005 0.000 0.000 0.000 0.000 0.000 0.076 0.053

0.076 0.065

>Methylation code ID 897

0.000 0.004 0.009 0.000 0.000 0.009 0.009 0.000 0.000 0.004

0.004 0.000 0.009 0.000 0.009 0.000 0.000 0.000 0.000 0.000

0.085 0.064 0.070 0.059 0.018 0.009 0.000 0.000 0.000 0.027

0.000 0.000 0.000 0.018 0.000 0.000 0.000 0.009 0.000 0.000

0.037 0.000 0.000 0.000 0.058 0.059 0.053 0.044 0.012 0.000

0.000 0.000 0.000 0.012 0.006 0.000 0.000 0.006 0.000 0.000

0.012 0.006 0.006 0.006 0.000 0.000 0.006 0.000 0.073 0.076

0.062 0.054

>Methylation code ID 897

0.005 0.000 0.000 0.000 0.000 0.010 0.000 0.000 0.000 0.000

0.005 0.000 0.015 0.000 0.005 0.010 0.005 0.000 0.010 0.000

0.071 0.056 0.064 0.078 0.000 0.000 0.005 0.000 0.000 0.021

0.005 0.000 0.000 0.005 0.000 0.000 0.010 0.005 0.005 0.000

0.000 0.005 0.005 0.000 0.069 0.072 0.046 0.079 0.016 0.000

0.000 0.000 0.000 0.016 0.008 0.000 0.000 0.000 0.016 0.008

0.000 0.008 0.000 0.008 0.000 0.008 0.008 0.000 0.074 0.066

0.032 0.068

>Methylation code ID 897

0.007 0.000 0.000 0.007 0.007 0.028 0.000 0.000 0.000 0.000

0.007 0.007 0.007 0.007 0.000 0.000 0.000 0.007 0.000 0.000

0.058 0.067 0.064 0.059 0.009 0.004 0.000 0.000 0.000 0.009

0.000 0.000 0.000 0.000 0.004 0.000 0.004 0.004 0.009 0.009

0.000 0.000 0.004 0.000 0.074 0.057 0.070 0.078 0.000 0.000

0.005 0.000 0.000 0.018 0.000 0.000 0.000 0.000 0.000 0.000

0.009 0.005 0.009 0.000 0.000 0.005 0.005 0.000 0.080 0.074

0.041 0.085

>Methylation code ID 897

0.007 0.000 0.000 0.015 0.007 0.029 0.000 0.000 0.000 0.000

0.000 0.007 0.007 0.000 0.000 0.000 0.000 0.007 0.000 0.000

0.065 0.061 0.065 0.062 0.005 0.005 0.000 0.005 0.000 0.016

0.000 0.000 0.000 0.000 0.005 0.000 0.005 0.005 0.005 0.016

0.000 0.000 0.000 0.000 0.065 0.058 0.066 0.073 0.000 0.000

0.000 0.000 0.000 0.019 0.000 0.000 0.000 0.000 0.000 0.000

0.005 0.005 0.014 0.000 0.000 0.005 0.005 0.000 0.093 0.079

0.023 0.088

>Methylation code ID 897

0.000 0.000 0.000 0.000 0.000 0.016 0.000 0.000 0.000 0.000

0.000 0.000 0.008 0.000 0.016 0.000 0.004 0.004 0.000 0.000

0.091 0.065 0.030 0.098 0.008 0.000 0.000 0.023 0.008 0.023

0.000 0.000 0.000 0.000 0.000 0.000 0.023 0.000 0.000 0.000

0.000 0.000 0.000 0.000 0.058 0.069 0.066 0.057 0.006 0.006

0.000 0.006 0.000 0.012 0.000 0.000 0.000 0.000 0.000 0.006

0.012 0.006 0.006 0.012 0.000 0.000 0.000 0.000 0.083 0.052

0.058 0.067

>Methylation code ID 897

0.006 0.000 0.000 0.000 0.000 0.012 0.012 0.000 0.006 0.012

0.000 0.000 0.012 0.000 0.000 0.000 0.000 0.000 0.006 0.006

0.069 0.061 0.072 0.060 0.016 0.000 0.000 0.000 0.000 0.008

0.000 0.008 0.000 0.008 0.000 0.000 0.008 0.008 0.008 0.008

0.016 0.000 0.000 0.008 0.061 0.056 0.051 0.070 0.000 0.005

0.000 0.005 0.000 0.005 0.000 0.000 0.000 0.009 0.000 0.000

0.014 0.000 0.009 0.005 0.005 0.000 0.000 0.000 0.046 0.074

0.077 0.081

>Methylation code ID 897

0.000 0.000 0.006 0.000 0.006 0.017 0.006 0.006 0.006 0.000

0.000 0.006 0.006 0.000 0.000 0.000 0.000 0.000 0.000 0.006

0.078 0.073 0.059 0.063 0.013 0.006 0.000 0.000 0.000 0.006

0.000 0.000 0.000 0.006 0.006 0.000 0.000 0.000 0.006 0.013

0.019 0.000 0.000 0.000 0.063 0.063 0.060 0.071 0.000 0.004

0.004 0.004 0.000 0.000 0.000 0.000 0.000 0.004 0.000 0.000

0.009 0.004 0.013 0.000 0.000 0.004 0.004 0.000 0.040 0.056

0.093 0.092

>Methylation code ID 897

0.000 0.000 0.005 0.000 0.005 0.010 0.005 0.005 0.005 0.000

0.000 0.005 0.005 0.000 0.005 0.000 0.000 0.000 0.000 0.005

0.080 0.064 0.069 0.063 0.007 0.007 0.000 0.000 0.000 0.013

0.000 0.000 0.000 0.007 0.007 0.000 0.000 0.000 0.007 0.013

0.020 0.000 0.000 0.000 0.062 0.066 0.062 0.063 0.004 0.004

0.004 0.004 0.000 0.000 0.000 0.000 0.000 0.000 0.000 0.000

0.009 0.004 0.013 0.000 0.000 0.004 0.004 0.000 0.043 0.055

0.084 0.099

>Methylation code ID 897

0.013 0.013 0.000 0.000 0.000 0.013 0.000 0.000 0.000 0.000

0.007 0.000 0.000 0.000 0.000 0.013 0.020 0.000 0.007 0.000

0.065 0.062 0.064 0.057 0.009 0.000 0.000 0.000 0.000 0.005

0.000 0.000 0.000 0.000 0.000 0.000 0.005 0.009 0.018 0.000

0.000 0.009 0.005 0.000 0.058 0.069 0.076 0.070 0.000 0.000

0.006 0.000 0.006 0.012 0.006 0.000 0.000 0.000 0.000 0.006

0.000 0.000 0.012 0.000 0.000 0.000 0.000 0.006 0.083 0.068

0.068 0.058

>Methylation code ID 897

0.000 0.000 0.000 0.000 0.006 0.012 0.006 0.000 0.006 0.006

0.000 0.006 0.000 0.000 0.012 0.000 0.000 0.000 0.000 0.006

0.067 0.075 0.081 0.052 0.011 0.011 0.000 0.000 0.000 0.011

0.000 0.000 0.000 0.000 0.005 0.005 0.000 0.000 0.000 0.005

0.011 0.000 0.011 0.000 0.066 0.074 0.067 0.055 0.012 0.000

0.000 0.000 0.000 0.012 0.000 0.000 0.000 0.000 0.000 0.000

0.006 0.012 0.006 0.000 0.006 0.012 0.006 0.000 0.065 0.077

0.059 0.058

>Methylation code ID 897

0.017 0.006 0.000 0.000 0.000 0.017 0.000 0.000 0.000 0.000

0.006 0.006 0.000 0.000 0.000 0.000 0.011 0.000 0.011 0.000

0.058 0.075 0.074 0.053 0.012 0.000 0.000 0.000 0.000 0.018

0.000 0.000 0.000 0.006 0.000 0.000 0.012 0.006 0.006 0.000

0.006 0.006 0.006 0.000 0.062 0.070 0.062 0.060 0.000 0.000

0.000 0.000 0.000 0.016 0.005 0.000 0.005 0.005 0.000 0.000

0.000 0.000 0.011 0.000 0.000 0.000 0.005 0.005 0.071 0.064

0.068 0.076

>Methylation code ID 897

0.000 0.006 0.000 0.000 0.006 0.012 0.006 0.000 0.000 0.012

0.000 0.000 0.000 0.000 0.000 0.006 0.006 0.006 0.018 0.000

0.084 0.060 0.046 0.065 0.027 0.000 0.000 0.000 0.000 0.027

0.000 0.000 0.000 0.000 0.000 0.000 0.018 0.000 0.000 0.027

0.009 0.000 0.009 0.000 0.042 0.061 0.059 0.056 0.006 0.000

0.000 0.000 0.000 0.012 0.006 0.000 0.000 0.037 0.000 0.000

0.006 0.000 0.006 0.000 0.000 0.000 0.000 0.000 0.050 0.068

0.069 0.072

>Methylation code ID 897

0.000 0.005 0.005 0.000 0.005 0.011 0.005 0.000 0.000 0.005

0.000 0.000 0.000 0.000 0.000 0.005 0.005 0.005 0.016 0.000

0.087 0.058 0.049 0.069 0.031 0.000 0.000 0.000 0.000 0.031

0.000 0.000 0.000 0.000 0.000 0.000 0.021 0.000 0.000 0.031

0.021 0.000 0.000 0.000 0.037 0.054 0.051 0.055 0.006 0.000

0.000 0.000 0.000 0.013 0.006 0.000 0.000 0.038 0.000 0.000

0.006 0.000 0.006 0.000 0.000 0.000 0.000 0.000 0.050 0.071

0.064 0.073

>Methylation code ID 897

0.023 0.000 0.000 0.008 0.008 0.008 0.015 0.000 0.000 0.000

0.000 0.000 0.008 0.000 0.000 0.023 0.000 0.000 0.000 0.000

0.059 0.055 0.063 0.065 0.000 0.000 0.000 0.000 0.000 0.008

0.000 0.008 0.000 0.000 0.000 0.000 0.030 0.008 0.008 0.015

0.008 0.000 0.000 0.000 0.066 0.074 0.047 0.063 0.005 0.005

0.000 0.005 0.000 0.010 0.000 0.000 0.000 0.014 0.000 0.000

0.000 0.000 0.010 0.010 0.000 0.000 0.000 0.000 0.067 0.055

0.088 0.066

>Methylation code ID 897

0.022 0.000 0.000 0.007 0.007 0.007 0.007 0.000 0.000 0.007

0.000 0.000 0.000 0.000 0.000 0.022 0.000 0.000 0.007 0.000

0.056 0.067 0.063 0.059 0.000 0.000 0.000 0.000 0.000 0.013

0.000 0.000 0.000 0.000 0.000 0.000 0.027 0.000 0.007 0.013

0.007 0.000 0.007 0.000 0.062 0.063 0.068 0.067 0.010 0.005

0.000 0.005 0.000 0.010 0.000 0.000 0.000 0.020 0.000 0.000

0.000 0.000 0.010 0.000 0.000 0.000 0.000 0.000 0.068 0.047

0.081 0.079

>Methylation code ID 897

0.034 0.000 0.000 0.000 0.000 0.023 0.000 0.011 0.000 0.023

0.000 0.000 0.023 0.000 0.000 0.011 0.011 0.000 0.000 0.000

0.048 0.058 0.039 0.053 0.000 0.000 0.000 0.000 0.000 0.009

0.000 0.000 0.000 0.017 0.000 0.000 0.043 0.000 0.000 0.009

0.000 0.009 0.009 0.000 0.057 0.067 0.062 0.053 0.000 0.004

0.004 0.000 0.000 0.008 0.004 0.000 0.000 0.000 0.000 0.000

0.008 0.004 0.008 0.004 0.000 0.000 0.004 0.000 0.062 0.063

0.080 0.079

>Methylation code ID 906

0.028 0.000 0.000 0.000 0.000 0.028 0.000 0.000 0.000 0.000

0.000 0.000 0.014 0.000 0.007 0.000 0.014 0.000 0.000 0.000

0.061 0.049 0.063 0.070 0.013 0.000 0.000 0.013 0.000 0.006

0.000 0.000 0.000 0.006 0.000 0.000 0.025 0.013 0.006 0.000

0.000 0.000 0.000 0.000 0.061 0.071 0.060 0.058 0.000 0.000

0.000 0.000 0.000 0.010 0.010 0.000 0.000 0.000 0.000 0.000

0.010 0.000 0.015 0.015 0.000 0.000 0.000 0.000 0.075 0.092

0.059 0.047

>Methylation code ID 906

0.012 0.000 0.000 0.012 0.000 0.006 0.000 0.000 0.000 0.006

0.000 0.000 0.025 0.012 0.006 0.000 0.000 0.000 0.000 0.000

0.058 0.064 0.068 0.063 0.000 0.004 0.000 0.000 0.000 0.009

0.009 0.000 0.000 0.000 0.000 0.000 0.009 0.000 0.013 0.013

0.000 0.000 0.000 0.000 0.074 0.089 0.057 0.056 0.037 0.000

0.000 0.000 0.000 0.022 0.000 0.000 0.000 0.000 0.000 0.000

0.007 0.000 0.007 0.000 0.015 0.000 0.000 0.000 0.055 0.051

0.067 0.071

>Methylation code ID 906

0.036 0.007 0.000 0.000 0.000 0.029 0.000 0.000 0.000 0.000

0.000 0.000 0.000 0.000 0.007 0.000 0.007 0.000 0.000 0.000

0.055 0.050 0.066 0.077 0.016 0.000 0.000 0.011 0.000 0.005

0.000 0.000 0.000 0.005 0.000 0.000 0.016 0.005 0.005 0.000

0.000 0.000 0.005 0.000 0.064 0.073 0.067 0.059 0.000 0.004

0.000 0.000 0.000 0.009 0.004 0.000 0.000 0.004 0.000 0.000

0.004 0.000 0.013 0.013 0.000 0.000 0.000 0.000 0.069 0.086

0.071 0.056

>Methylation code ID 906

0.000 0.004 0.004 0.000 0.000 0.011 0.004 0.000 0.000 0.004

0.000 0.000 0.004 0.000 0.015 0.004 0.000 0.000 0.000 0.000

0.077 0.067 0.073 0.068 0.031 0.008 0.000 0.000 0.000 0.031

0.000 0.000 0.000 0.000 0.008 0.000 0.000 0.000 0.008 0.000

0.008 0.000 0.000 0.000 0.075 0.049 0.061 0.057 0.016 0.000

0.000 0.005 0.000 0.011 0.000 0.000 0.000 0.005 0.000 0.000

0.016 0.000 0.005 0.000 0.000 0.000 0.005 0.000 0.071 0.072

0.057 0.067

>Methylation code ID 906

0.041 0.010 0.000 0.000 0.000 0.041 0.000 0.000 0.000 0.000

0.010 0.000 0.000 0.000 0.000 0.010 0.010 0.000 0.000 0.000

0.051 0.053 0.047 0.059 0.022 0.000 0.000 0.005 0.000 0.005

0.000 0.000 0.000 0.005 0.000 0.000 0.016 0.000 0.005 0.000

0.000 0.000 0.005 0.000 0.069 0.071 0.059 0.069 0.000 0.004

0.004 0.004 0.000 0.008 0.004 0.000 0.000 0.004 0.000 0.000

0.000 0.000 0.016 0.004 0.000 0.000 0.000 0.000 0.072 0.061

0.066 0.086

>Methylation code ID 906

0.042 0.010 0.000 0.000 0.000 0.031 0.000 0.000 0.000 0.000

0.010 0.000 0.000 0.000 0.000 0.021 0.010 0.000 0.000 0.000

0.048 0.054 0.046 0.060 0.021 0.000 0.000 0.005 0.000 0.005

0.000 0.000 0.000 0.005 0.000 0.000 0.010 0.000 0.010 0.000

0.000 0.000 0.005 0.000 0.075 0.072 0.057 0.067 0.000 0.004

0.004 0.004 0.000 0.009 0.004 0.000 0.000 0.004 0.000 0.000

0.000 0.004 0.013 0.004 0.000 0.000 0.000 0.000 0.075 0.057

0.063 0.086

>Methylation code ID 906

0.000 0.000 0.004 0.004 0.000 0.013 0.004 0.000 0.000 0.000

0.000 0.000 0.000 0.009 0.013 0.000 0.000 0.000 0.009 0.000

0.076 0.077 0.070 0.054 0.023 0.000 0.012 0.000 0.000 0.058

0.000 0.000 0.000 0.000 0.012 0.000 0.000 0.000 0.000 0.035

0.012 0.000 0.000 0.000 0.048 0.049 0.041 0.045 0.025 0.000

0.000 0.000 0.000 0.017 0.000 0.000 0.000 0.000 0.000 0.000

0.017 0.000 0.008 0.000 0.008 0.000 0.000 0.000 0.067 0.069

0.059 0.061

>Methylation code ID 906

0.011 0.000 0.011 0.000 0.000 0.068 0.000 0.000 0.000 0.000

0.011 0.000 0.000 0.000 0.000 0.034 0.011 0.000 0.000 0.000

0.049 0.058 0.036 0.044 0.040 0.000 0.000 0.000 0.000 0.016

0.000 0.000 0.000 0.000 0.000 0.000 0.008 0.000 0.008 0.000

0.008 0.000 0.000 0.000 0.059 0.062 0.053 0.079 0.000 0.000

0.004 0.004 0.000 0.009 0.004 0.000 0.000 0.000 0.000 0.000

0.000 0.009 0.013 0.000 0.000 0.000 0.009 0.000 0.064 0.079

0.084 0.052

>Methylation code ID 906

0.000 0.000 0.011 0.000 0.000 0.091 0.000 0.000 0.000 0.000

0.000 0.000 0.000 0.000 0.000 0.034 0.011 0.000 0.000 0.000

0.039 0.053 0.045 0.048 0.088 0.000 0.000 0.000 0.000 0.013

0.000 0.000 0.000 0.000 0.000 0.000 0.000 0.000 0.013 0.000

0.013 0.013 0.000 0.000 0.046 0.052 0.037 0.059 0.000 0.000

0.000 0.000 0.000 0.010 0.000 0.000 0.000 0.000 0.000 0.000

0.000 0.015 0.025 0.000 0.000 0.000 0.010 0.000 0.062 0.080

0.066 0.066

>Methylation code ID 906

0.000 0.000 0.012 0.000 0.000 0.095 0.000 0.000 0.000 0.000

0.000 0.000 0.000 0.000 0.000 0.047 0.000 0.000 0.000 0.000

0.036 0.053 0.045 0.045 0.179 0.000 0.000 0.000 0.000 0.022

0.000 0.000 0.000 0.000 0.000 0.000 0.000 0.000 0.000 0.000

0.022 0.022 0.000 0.000 0.025 0.025 0.017 0.019 0.000 0.000

0.000 0.000 0.000 0.006 0.000 0.000 0.000 0.000 0.000 0.000

0.000 0.017 0.028 0.000 0.000 0.000 0.011 0.000 0.055 0.079

0.063 0.076

>Methylation code ID 906

0.000 0.000 0.010 0.000 0.000 0.059 0.000 0.000 0.000 0.000

0.000 0.000 0.000 0.000 0.010 0.049 0.000 0.000 0.000 0.000

0.047 0.062 0.044 0.053 0.167 0.000 0.000 0.000 0.000 0.042

0.000 0.000 0.000 0.000 0.000 0.000 0.000 0.000 0.000 0.000

0.021 0.021 0.000 0.000 0.023 0.018 0.022 0.019 0.000 0.000

0.000 0.000 0.000 0.007 0.000 0.000 0.000 0.000 0.000 0.000

0.000 0.035 0.028 0.000 0.000 0.000 0.007 0.000 0.050 0.072

0.067 0.069

>Methylation code ID 906

0.000 0.000 0.010 0.000 0.000 0.052 0.000 0.000 0.000 0.000

0.000 0.000 0.000 0.000 0.010 0.052 0.000 0.000 0.000 0.000

0.050 0.052 0.046 0.059 0.092 0.000 0.000 0.000 0.000 0.023

0.000 0.000 0.000 0.000 0.000 0.000 0.000 0.000 0.011 0.000

0.011 0.011 0.000 0.000 0.043 0.043 0.048 0.051 0.000 0.000

0.000 0.000 0.000 0.008 0.000 0.000 0.000 0.000 0.000 0.000

0.000 0.038 0.031 0.000 0.000 0.008 0.000 0.000 0.045 0.076

0.053 0.075

>Methylation code ID 906

0.000 0.000 0.000 0.000 0.000 0.021 0.000 0.000 0.000 0.007

0.000 0.000 0.000 0.021 0.007 0.007 0.007 0.007 0.007 0.000

0.065 0.045 0.068 0.072 0.027 0.000 0.000 0.000 0.000 0.027

0.000 0.000 0.000 0.000 0.000 0.000 0.009 0.000 0.009 0.036

0.000 0.000 0.000 0.000 0.058 0.053 0.061 0.053 0.014 0.000

0.000 0.005 0.000 0.010 0.005 0.000 0.000 0.005 0.000 0.000

0.005 0.000 0.010 0.000 0.000 0.005 0.000 0.000 0.072 0.048

0.067 0.090

>Methylation code ID 906

0.038 0.000 0.000 0.000 0.000 0.051 0.000 0.000 0.000 0.000

0.000 0.000 0.026 0.000 0.000 0.026 0.000 0.013 0.000 0.000

0.052 0.047 0.030 0.052 0.010 0.000 0.000 0.005 0.000 0.005

0.005 0.000 0.000 0.016 0.000 0.000 0.010 0.000 0.010 0.005

0.000 0.000 0.000 0.000 0.077 0.048 0.061 0.081 0.000 0.000

0.000 0.000 0.000 0.015 0.000 0.000 0.000 0.010 0.000 0.000

0.005 0.000 0.010 0.005 0.005 0.000 0.010 0.000 0.085 0.065

0.074 0.048

>Methylation code ID 906

0.024 0.008 0.000 0.000 0.000 0.024 0.016 0.000 0.000 0.000

0.000 0.000 0.016 0.000 0.000 0.008 0.000 0.008 0.000 0.000

0.069 0.050 0.047 0.064 0.015 0.000 0.000 0.008 0.000 0.008

0.008 0.000 0.000 0.015 0.008 0.000 0.023 0.000 0.000 0.008

0.008 0.000 0.000 0.000 0.068 0.052 0.066 0.049 0.000 0.005

0.000 0.000 0.000 0.005 0.000 0.000 0.000 0.009 0.000 0.000

0.009 0.000 0.009 0.009 0.005 0.000 0.005 0.000 0.071 0.079

0.073 0.053

>Methylation code ID 906

0.012 0.000 0.000 0.000 0.000 0.000 0.006 0.000 0.000 0.012

0.000 0.000 0.036 0.000 0.006 0.000 0.006 0.000 0.000 0.000

0.058 0.076 0.063 0.058 0.000 0.007 0.000 0.000 0.000 0.007

0.022 0.000 0.000 0.022 0.000 0.000 0.015 0.007 0.000 0.007

0.007 0.000 0.000 0.000 0.071 0.048 0.052 0.067 0.015 0.000

0.000 0.000 0.000 0.000 0.007 0.000 0.000 0.000 0.000 0.000

0.007 0.007 0.007 0.022 0.022 0.000 0.000 0.000 0.061 0.065

0.043 0.076

>Methylation code ID 906

0.000 0.000 0.008 0.000 0.000 0.016 0.000 0.000 0.008 0.023

0.000 0.000 0.008 0.016 0.000 0.008 0.000 0.008 0.000 0.000

0.062 0.061 0.050 0.066 0.008 0.008 0.000 0.000 0.004 0.000

0.000 0.000 0.000 0.004 0.000 0.000 0.004 0.000 0.008 0.008

0.004 0.000 0.004 0.000 0.070 0.082 0.057 0.074 0.021 0.000

0.000 0.007 0.000 0.014 0.000 0.000 0.000 0.007 0.000 0.000

0.007 0.000 0.014 0.014 0.000 0.000 0.000 0.000 0.049 0.075

0.064 0.061

>Methylation code ID 906

0.000 0.000 0.009 0.026 0.000 0.017 0.000 0.000 0.000 0.026

0.000 0.000 0.000 0.017 0.000 0.009 0.000 0.009 0.000 0.000

0.056 0.053 0.045 0.069 0.009 0.004 0.000 0.000 0.004 0.000

0.000 0.000 0.004 0.000 0.000 0.004 0.004 0.000 0.013 0.004

0.004 0.000 0.004 0.000 0.060 0.060 0.073 0.085 0.005 0.000

0.000 0.000 0.000 0.020 0.000 0.000 0.000 0.005 0.000 0.000

0.005 0.000 0.015 0.005 0.005 0.000 0.000 0.000 0.041 0.062

0.090 0.081

>Methylation code ID 957

0.000 0.006 0.000 0.000 0.000 0.018 0.006 0.000 0.000 0.000

0.000 0.000 0.012 0.006 0.006 0.012 0.012 0.000 0.000 0.000

0.054 0.050 0.076 0.075 0.026 0.000 0.000 0.000 0.000 0.013

0.007 0.000 0.007 0.007 0.000 0.000 0.007 0.000 0.013 0.000

0.007 0.000 0.000 0.000 0.066 0.060 0.068 0.054 0.000 0.000

0.000 0.005 0.000 0.005 0.005 0.000 0.000 0.005 0.000 0.005

0.016 0.000 0.005 0.000 0.005 0.005 0.005 0.000 0.066 0.077

0.063 0.063

>Methylation code ID 957

0.000 0.000 0.000 0.000 0.000 0.011 0.005 0.000 0.000 0.005

0.000 0.005 0.016 0.000 0.005 0.000 0.011 0.005 0.005 0.000

0.069 0.073 0.064 0.058 0.000 0.008 0.000 0.000 0.000 0.030

0.015 0.000 0.000 0.000 0.000 0.000 0.015 0.008 0.000 0.008

0.015 0.000 0.000 0.000 0.063 0.051 0.066 0.055 0.018 0.000

0.006 0.000 0.000 0.006 0.006 0.000 0.006 0.006 0.000 0.000

0.006 0.000 0.012 0.000 0.006 0.000 0.000 0.000 0.071 0.062

0.070 0.058

>Methylation code ID 957

0.006 0.006 0.006 0.000 0.000 0.000 0.012 0.006 0.000 0.000

0.000 0.006 0.006 0.000 0.000 0.000 0.012 0.000 0.006 0.000

0.063 0.062 0.060 0.081 0.006 0.012 0.000 0.000 0.000 0.006

0.006 0.000 0.000 0.006 0.000 0.000 0.012 0.000 0.006 0.000

0.023 0.000 0.000 0.000 0.076 0.047 0.053 0.081 0.000 0.006

0.000 0.006 0.000 0.006 0.017 0.000 0.000 0.000 0.000 0.000

0.012 0.000 0.006 0.000 0.006 0.000 0.000 0.000 0.059 0.058

0.067 0.091

>Methylation code ID 957

0.013 0.000 0.000 0.000 0.000 0.006 0.000 0.000 0.000 0.006

0.000 0.000 0.044 0.000 0.006 0.006 0.000 0.000 0.000 0.000

0.066 0.060 0.049 0.076 0.018 0.000 0.000 0.000 0.000 0.000

0.000 0.000 0.000 0.036 0.000 0.000 0.054 0.000 0.000 0.009

0.000 0.000 0.000 0.000 0.057 0.053 0.037 0.069 0.000 0.006

0.000 0.000 0.000 0.000 0.000 0.000 0.000 0.006 0.000 0.000

0.029 0.000 0.011 0.017 0.000 0.000 0.000 0.000 0.074 0.074

0.034 0.082

>Methylation code ID 957

0.006 0.000 0.000 0.000 0.000 0.006 0.000 0.000 0.000 0.006

0.000 0.000 0.042 0.000 0.006 0.006 0.000 0.000 0.006 0.000

0.073 0.068 0.054 0.060 0.018 0.000 0.000 0.000 0.000 0.009

0.000 0.000 0.000 0.026 0.000 0.000 0.053 0.000 0.000 0.009

0.000 0.000 0.000 0.000 0.050 0.059 0.037 0.074 0.005 0.000

0.000 0.000 0.000 0.000 0.000 0.000 0.000 0.011 0.000 0.000

0.022 0.000 0.011 0.016 0.000 0.000 0.000 0.000 0.076 0.070

0.042 0.081

>Methylation code ID 957

0.007 0.000 0.000 0.000 0.000 0.021 0.000 0.000 0.000 0.014

0.000 0.000 0.035 0.000 0.000 0.000 0.007 0.000 0.007 0.000

0.055 0.058 0.054 0.074 0.000 0.000 0.000 0.006 0.000 0.013

0.000 0.000 0.000 0.013 0.000 0.000 0.025 0.000 0.006 0.006

0.000 0.000 0.006 0.000 0.066 0.064 0.048 0.080 0.015 0.000

0.000 0.000 0.000 0.005 0.000 0.000 0.000 0.005 0.000 0.005

0.010 0.000 0.010 0.005 0.000 0.000 0.005 0.000 0.076 0.065

0.065 0.068

>Methylation code ID 957

0.020 0.005 0.000 0.005 0.000 0.005 0.000 0.000 0.000 0.010

0.000 0.000 0.000 0.005 0.005 0.005 0.000 0.000 0.005 0.000

0.073 0.069 0.058 0.067 0.019 0.000 0.000 0.000 0.000 0.005

0.000 0.000 0.005 0.005 0.000 0.000 0.009 0.000 0.009 0.000

0.000 0.000 0.009 0.000 0.083 0.079 0.043 0.068 0.000 0.000

0.000 0.000 0.000 0.025 0.000 0.000 0.000 0.013 0.000 0.000

0.025 0.000 0.006 0.006 0.000 0.000 0.000 0.000 0.062 0.059

0.058 0.079

>Methylation code ID 957

0.013 0.000 0.000 0.000 0.000 0.004 0.004 0.000 0.004 0.000

0.000 0.000 0.009 0.000 0.009 0.000 0.000 0.000 0.013 0.000

0.064 0.084 0.069 0.060 0.014 0.000 0.000 0.000 0.000 0.027

0.000 0.000 0.000 0.007 0.000 0.000 0.020 0.000 0.007 0.007

0.007 0.000 0.000 0.000 0.060 0.061 0.065 0.059 0.017 0.006

0.000 0.006 0.000 0.006 0.000 0.000 0.000 0.017 0.000 0.000

0.012 0.000 0.006 0.000 0.000 0.000 0.000 0.000 0.066 0.069

0.062 0.067

>Methylation code ID 957

0.014 0.000 0.000 0.000 0.000 0.021 0.007 0.000 0.000 0.007

0.000 0.000 0.021 0.000 0.007 0.000 0.014 0.000 0.000 0.000

0.071 0.053 0.050 0.069 0.011 0.005 0.005 0.000 0.000 0.005

0.000 0.000 0.000 0.021 0.000 0.000 0.011 0.000 0.005 0.000

0.005 0.000 0.000 0.000 0.062 0.070 0.059 0.073 0.014 0.000

0.000 0.000 0.000 0.000 0.009 0.000 0.000 0.000 0.000 0.000

0.005 0.000 0.009 0.000 0.005 0.000 0.014 0.000 0.058 0.085

0.065 0.071

>Methylation code ID 957

0.022 0.000 0.000 0.000 0.000 0.022 0.007 0.000 0.000 0.000

0.000 0.000 0.022 0.000 0.007 0.000 0.015 0.000 0.000 0.000

0.059 0.061 0.049 0.069 0.012 0.000 0.006 0.000 0.000 0.006

0.006 0.000 0.000 0.023 0.000 0.000 0.012 0.000 0.006 0.000

0.006 0.000 0.000 0.000 0.065 0.070 0.062 0.061 0.008 0.004

0.000 0.000 0.000 0.000 0.008 0.000 0.000 0.000 0.000 0.000

0.004 0.000 0.008 0.000 0.004 0.000 0.013 0.000 0.060 0.078

0.070 0.075

>Methylation code ID 957

0.000 0.011 0.000 0.000 0.000 0.000 0.016 0.000 0.000 0.000

0.005 0.000 0.011 0.000 0.011 0.000 0.005 0.005 0.005 0.000

0.055 0.070 0.072 0.068 0.025 0.006 0.000 0.000 0.000 0.006

0.012 0.000 0.000 0.000 0.000 0.000 0.000 0.000 0.006 0.006

0.019 0.000 0.000 0.000 0.068 0.057 0.053 0.075 0.006 0.000

0.006 0.000 0.000 0.000 0.006 0.000 0.000 0.011 0.006 0.000

0.023 0.000 0.006 0.000 0.006 0.000 0.000 0.000 0.077 0.053

0.071 0.064

>Methylation code ID 957

0.006 0.013 0.000 0.000 0.000 0.000 0.019 0.000 0.000 0.000

0.006 0.000 0.019 0.000 0.000 0.000 0.006 0.006 0.006 0.000

0.061 0.062 0.062 0.066 0.011 0.006 0.000 0.000 0.000 0.006

0.011 0.000 0.000 0.000 0.000 0.000 0.011 0.000 0.006 0.006

0.017 0.000 0.000 0.000 0.073 0.068 0.049 0.069 0.010 0.000

0.005 0.000 0.000 0.000 0.005 0.000 0.000 0.010 0.005 0.000

0.010 0.000 0.010 0.000 0.005 0.000 0.000 0.000 0.078 0.052

0.065 0.079

>Methylation code ID 957

0.013 0.000 0.000 0.000 0.000 0.000 0.004 0.000 0.000 0.004

0.004 0.000 0.018 0.000 0.013 0.000 0.000 0.000 0.000 0.000

0.083 0.064 0.080 0.049 0.011 0.011 0.000 0.000 0.000 0.000

0.006 0.000 0.000 0.000 0.006 0.000 0.028 0.000 0.006 0.000

0.000 0.006 0.000 0.000 0.060 0.067 0.075 0.058 0.016 0.005

0.000 0.000 0.000 0.000 0.005 0.000 0.000 0.000 0.000 0.000

0.016 0.000 0.011 0.005 0.005 0.000 0.000 0.000 0.085 0.064

0.072 0.049

>Methylation code ID 957

0.011 0.005 0.000 0.000 0.000 0.000 0.005 0.000 0.000 0.000

0.005 0.000 0.026 0.000 0.011 0.000 0.000 0.005 0.000 0.000

0.063 0.069 0.080 0.053 0.010 0.005 0.000 0.000 0.000 0.005

0.005 0.000 0.000 0.000 0.000 0.000 0.020 0.000 0.010 0.005

0.005 0.000 0.000 0.000 0.077 0.074 0.083 0.035 0.015 0.000

0.000 0.000 0.000 0.000 0.000 0.000 0.000 0.000 0.005 0.000

0.025 0.000 0.015 0.000 0.000 0.000 0.000 0.000 0.093 0.067

0.069 0.046

>Methylation code ID 957

0.010 0.000 0.000 0.000 0.000 0.005 0.005 0.000 0.000 0.000

0.000 0.000 0.026 0.000 0.010 0.005 0.005 0.000 0.000 0.000

0.076 0.068 0.076 0.044 0.009 0.000 0.000 0.000 0.000 0.005

0.000 0.000 0.000 0.000 0.005 0.000 0.028 0.000 0.014 0.000

0.000 0.000 0.000 0.000 0.081 0.072 0.074 0.046 0.018 0.006

0.000 0.000 0.000 0.000 0.000 0.000 0.000 0.000 0.000 0.000

0.029 0.000 0.012 0.000 0.000 0.006 0.000 0.000 0.075 0.069

0.077 0.043

>Methylation code ID 957

0.015 0.000 0.000 0.000 0.000 0.005 0.000 0.000 0.000 0.000

0.005 0.000 0.030 0.000 0.010 0.000 0.000 0.000 0.000 0.000

0.079 0.068 0.069 0.051 0.016 0.005 0.000 0.000 0.000 0.000

0.000 0.000 0.000 0.005 0.000 0.000 0.027 0.005 0.011 0.000

0.000 0.000 0.000 0.000 0.072 0.063 0.086 0.043 0.011 0.000

0.000 0.000 0.000 0.006 0.006 0.000 0.000 0.000 0.000 0.000

0.028 0.000 0.011 0.006 0.000 0.000 0.000 0.000 0.083 0.071

0.076 0.036

>Methylation code ID 957

0.016 0.005 0.000 0.000 0.000 0.000 0.000 0.000 0.000 0.005

0.000 0.000 0.021 0.005 0.010 0.005 0.000 0.000 0.000 0.000

0.070 0.062 0.085 0.049 0.017 0.000 0.000 0.000 0.000 0.006

0.000 0.000 0.000 0.006 0.000 0.000 0.029 0.000 0.012 0.006

0.000 0.000 0.000 0.000 0.080 0.071 0.074 0.033 0.018 0.000

0.000 0.000 0.000 0.006 0.000 0.000 0.000 0.000 0.000 0.000

0.030 0.000 0.012 0.000 0.000 0.000 0.000 0.000 0.083 0.081

0.068 0.035

>Methylation code ID 957

0.012 0.000 0.000 0.000 0.000 0.006 0.000 0.000 0.000 0.006

0.000 0.000 0.030 0.000 0.012 0.006 0.006 0.000 0.000 0.000

0.070 0.072 0.073 0.041 0.017 0.000 0.000 0.000 0.000 0.006

0.000 0.000 0.000 0.000 0.000 0.000 0.023 0.011 0.011 0.000

0.000 0.000 0.000 0.000 0.078 0.069 0.073 0.045 0.017 0.000

0.000 0.000 0.000 0.000 0.000 0.000 0.000 0.006 0.000 0.006

0.017 0.006 0.012 0.006 0.000 0.000 0.000 0.000 0.081 0.072

0.074 0.037

>Methylation code ID 957

0.012 0.006 0.006 0.000 0.000 0.006 0.006 0.000 0.000 0.006

0.000 0.006 0.000 0.006 0.006 0.012 0.000 0.006 0.000 0.000

0.079 0.047 0.075 0.057 0.018 0.000 0.000 0.000 0.000 0.018

0.000 0.000 0.000 0.009 0.009 0.000 0.009 0.000 0.009 0.018

0.018 0.000 0.000 0.000 0.073 0.047 0.053 0.054 0.011 0.005

0.000 0.000 0.000 0.005 0.000 0.000 0.000 0.000 0.000 0.000

0.005 0.011 0.011 0.000 0.000 0.000 0.005 0.000 0.074 0.064

0.060 0.083

>Methylation code ID 957

0.007 0.007 0.007 0.000 0.000 0.007 0.007 0.000 0.000 0.021

0.000 0.007 0.000 0.000 0.000 0.014 0.000 0.007 0.000 0.000

0.066 0.056 0.075 0.054 0.013 0.006 0.006 0.000 0.000 0.006

0.000 0.000 0.000 0.006 0.006 0.000 0.000 0.000 0.006 0.006

0.013 0.000 0.000 0.006 0.065 0.067 0.062 0.065 0.006 0.006

0.000 0.000 0.000 0.000 0.000 0.000 0.000 0.000 0.000 0.000

0.006 0.011 0.011 0.000 0.006 0.000 0.006 0.000 0.074 0.085

0.051 0.073

>Methylation code ID 957

0.005 0.005 0.000 0.000 0.000 0.000 0.000 0.000 0.000 0.000

0.000 0.000 0.005 0.010 0.010 0.000 0.010 0.000 0.005 0.000

0.068 0.082 0.055 0.077 0.007 0.007 0.007 0.000 0.000 0.007

0.007 0.000 0.000 0.014 0.000 0.007 0.000 0.007 0.000 0.014

0.000 0.007 0.000 0.000 0.067 0.052 0.077 0.054 0.013 0.006

0.006 0.000 0.000 0.006 0.000 0.000 0.000 0.000 0.006 0.000

0.000 0.000 0.006 0.006 0.013 0.000 0.000 0.006 0.065 0.067

0.063 0.068

>Methylation code ID 957

0.015 0.000 0.000 0.015 0.000 0.007 0.000 0.000 0.000 0.022

0.000 0.000 0.000 0.000 0.007 0.000 0.007 0.000 0.007 0.000

0.070 0.043 0.073 0.067 0.000 0.004 0.004 0.000 0.000 0.013

0.004 0.000 0.009 0.000 0.000 0.000 0.009 0.000 0.000 0.000

0.000 0.000 0.009 0.000 0.082 0.064 0.055 0.080 0.007 0.000

0.007 0.000 0.000 0.014 0.000 0.000 0.000 0.000 0.007 0.000

0.007 0.000 0.007 0.014 0.007 0.007 0.000 0.000 0.051 0.061

0.064 0.079

>Methylation code ID 957

0.000 0.005 0.005 0.005 0.000 0.009 0.000 0.000 0.009 0.000

0.000 0.000 0.009 0.000 0.000 0.000 0.005 0.000 0.009 0.000

0.083 0.061 0.055 0.080 0.006 0.000 0.006 0.000 0.000 0.017

0.000 0.000 0.006 0.000 0.006 0.000 0.006 0.006 0.006 0.012

0.000 0.000 0.000 0.000 0.053 0.069 0.075 0.066 0.007 0.000

0.000 0.014 0.000 0.007 0.000 0.000 0.000 0.007 0.000 0.007

0.000 0.000 0.007 0.000 0.007 0.000 0.007 0.000 0.080 0.063

0.068 0.061

>Methylation code ID 1000

0.032 0.000 0.000 0.000 0.000 0.024 0.000 0.000 0.000 0.024

0.000 0.000 0.016 0.000 0.008 0.000 0.000 0.000 0.000 0.000

0.065 0.057 0.049 0.059 0.014 0.000 0.000 0.000 0.000 0.014

0.000 0.000 0.000 0.000 0.000 0.000 0.084 0.000 0.000 0.028

0.000 0.000 0.000 0.000 0.047 0.041 0.040 0.066 0.000 0.008

0.000 0.000 0.000 0.004 0.000 0.000 0.000 0.000 0.000 0.000

0.020 0.000 0.016 0.000 0.000 0.000 0.000 0.000 0.067 0.070

0.064 0.084

>Methylation code ID 1000

0.007 0.000 0.007 0.014 0.000 0.014 0.000 0.007 0.007 0.000

0.000 0.007 0.000 0.000 0.000 0.014 0.000 0.000 0.000 0.007

0.059 0.073 0.063 0.055 0.015 0.005 0.000 0.000 0.000 0.000

0.000 0.005 0.000 0.000 0.000 0.000 0.010 0.000 0.015 0.005

0.010 0.000 0.000 0.000 0.049 0.067 0.068 0.085 0.000 0.000

0.000 0.010 0.000 0.000 0.005 0.000 0.000 0.005 0.000 0.000

0.000 0.000 0.029 0.000 0.000 0.005 0.000 0.000 0.078 0.053

0.065 0.084

>Methylation code ID 1017

0.016 0.011 0.011 0.000 0.000 0.000 0.005 0.000 0.000 0.000

0.000 0.000 0.005 0.000 0.000 0.005 0.005 0.000 0.011 0.000

0.053 0.072 0.066 0.072 0.008 0.000 0.000 0.000 0.000 0.016

0.000 0.000 0.000 0.024 0.000 0.000 0.016 0.000 0.008 0.000

0.024 0.000 0.000 0.000 0.048 0.054 0.067 0.068 0.005 0.005

0.005 0.000 0.005 0.000 0.000 0.000 0.000 0.009 0.000 0.000

0.005 0.000 0.009 0.000 0.000 0.000 0.000 0.000 0.052 0.081

0.085 0.073

>Methylation code ID 1017

0.017 0.011 0.011 0.000 0.000 0.000 0.006 0.000 0.000 0.000

0.000 0.000 0.011 0.000 0.000 0.006 0.006 0.000 0.006 0.000

0.062 0.068 0.064 0.066 0.007 0.000 0.000 0.000 0.000 0.007

0.000 0.000 0.000 0.022 0.000 0.000 0.022 0.000 0.007 0.000

0.022 0.000 0.000 0.000 0.051 0.068 0.066 0.059 0.004 0.004

0.004 0.000 0.004 0.000 0.000 0.000 0.000 0.009 0.000 0.000

0.009 0.000 0.009 0.000 0.000 0.000 0.000 0.000 0.051 0.075

0.082 0.081

>Methylation code ID 1017

0.004 0.000 0.000 0.000 0.000 0.000 0.000 0.000 0.000 0.009

0.000 0.000 0.013 0.000 0.017 0.004 0.000 0.000 0.000 0.000

0.053 0.084 0.079 0.071 0.024 0.012 0.012 0.000 0.000 0.000

0.006 0.000 0.000 0.006 0.000 0.000 0.018 0.000 0.000 0.000

0.000 0.000 0.000 0.000 0.061 0.063 0.067 0.063 0.007 0.000

0.000 0.000 0.000 0.000 0.000 0.000 0.000 0.007 0.000 0.000

0.026 0.000 0.007 0.007 0.020 0.000 0.000 0.000 0.049 0.072

0.076 0.066

>Methylation code ID 1017

0.027 0.007 0.007 0.000 0.000 0.000 0.007 0.000 0.000 0.007

0.000 0.000 0.034 0.000 0.000 0.000 0.000 0.000 0.000 0.000

0.052 0.057 0.076 0.060 0.000 0.000 0.000 0.000 0.000 0.000

0.000 0.000 0.000 0.005 0.000 0.000 0.024 0.000 0.014 0.005

0.009 0.000 0.000 0.000 0.050 0.079 0.081 0.066 0.014 0.000

0.000 0.000 0.000 0.000 0.000 0.000 0.000 0.005 0.000 0.000

0.014 0.000 0.014 0.005 0.000 0.000 0.000 0.000 0.046 0.066

0.085 0.086

>Methylation code ID 1017

0.018 0.006 0.006 0.000 0.000 0.000 0.006 0.000 0.000 0.006

0.000 0.000 0.030 0.000 0.006 0.000 0.000 0.000 0.000 0.000

0.059 0.057 0.073 0.067 0.000 0.000 0.000 0.000 0.000 0.000

0.000 0.000 0.000 0.005 0.000 0.000 0.018 0.000 0.014 0.005

0.009 0.005 0.000 0.000 0.052 0.086 0.079 0.061 0.016 0.000

0.000 0.000 0.000 0.000 0.000 0.000 0.000 0.005 0.000 0.000

0.021 0.000 0.010 0.005 0.000 0.000 0.000 0.000 0.043 0.065

0.081 0.087

>Methylation code ID 1017

0.015 0.000 0.000 0.000 0.000 0.005 0.000 0.000 0.000 0.000

0.000 0.000 0.026 0.000 0.010 0.010 0.000 0.000 0.000 0.000

0.050 0.064 0.081 0.072 0.019 0.000 0.000 0.000 0.000 0.000

0.006 0.000 0.000 0.006 0.000 0.000 0.044 0.000 0.006 0.000

0.000 0.000 0.000 0.000 0.061 0.064 0.076 0.050 0.000 0.000

0.000 0.000 0.000 0.000 0.000 0.000 0.000 0.004 0.000 0.000

0.017 0.000 0.017 0.004 0.004 0.004 0.000 0.000 0.049 0.079

0.089 0.065

>Methylation code ID 1017

0.017 0.000 0.000 0.000 0.000 0.006 0.000 0.000 0.000 0.000

0.000 0.000 0.028 0.000 0.011 0.011 0.000 0.000 0.000 0.000

0.056 0.069 0.077 0.059 0.017 0.000 0.000 0.000 0.000 0.000

0.006 0.000 0.000 0.006 0.000 0.000 0.034 0.000 0.011 0.000

0.000 0.000 0.000 0.000 0.063 0.073 0.082 0.041 0.000 0.000

0.000 0.000 0.000 0.000 0.000 0.000 0.000 0.005 0.000 0.000

0.023 0.000 0.014 0.005 0.005 0.005 0.000 0.000 0.058 0.075

0.088 0.056

>Methylation code ID 1017

0.000 0.000 0.000 0.000 0.000 0.004 0.000 0.000 0.000 0.004

0.000 0.000 0.020 0.000 0.016 0.004 0.000 0.004 0.000 0.000

0.060 0.083 0.076 0.061 0.027 0.000 0.000 0.000 0.000 0.007

0.000 0.000 0.000 0.000 0.000 0.000 0.027 0.000 0.013 0.013

0.000 0.000 0.000 0.000 0.051 0.050 0.069 0.076 0.019 0.000

0.000 0.000 0.000 0.000 0.000 0.000 0.000 0.006 0.000 0.000

0.038 0.000 0.013 0.000 0.000 0.000 0.000 0.000 0.075 0.072

0.074 0.037

>Methylation code ID 1017

0.030 0.000 0.000 0.000 0.000 0.008 0.000 0.000 0.000 0.000

0.000 0.000 0.038 0.000 0.008 0.015 0.000 0.000 0.000 0.000

0.067 0.048 0.055 0.065 0.005 0.000 0.000 0.000 0.000 0.005

0.000 0.000 0.000 0.005 0.000 0.000 0.043 0.000 0.011 0.000

0.000 0.000 0.000 0.000 0.066 0.074 0.079 0.045 0.000 0.000

0.000 0.000 0.000 0.005 0.000 0.000 0.000 0.000 0.000 0.000

0.023 0.000 0.018 0.005 0.000 0.005 0.000 0.000 0.075 0.091

0.060 0.052

>Methylation code ID 1017

0.004 0.000 0.000 0.000 0.000 0.004 0.000 0.000 0.000 0.000

0.000 0.000 0.026 0.000 0.018 0.000 0.000 0.004 0.000 0.000

0.069 0.081 0.066 0.061 0.029 0.000 0.000 0.000 0.000 0.007

0.000 0.000 0.000 0.000 0.000 0.000 0.043 0.000 0.007 0.007

0.000 0.000 0.000 0.000 0.071 0.058 0.054 0.056 0.006 0.000

0.000 0.000 0.000 0.006 0.000 0.000 0.000 0.000 0.000 0.000

0.050 0.000 0.012 0.000 0.000 0.000 0.000 0.000 0.077 0.082

0.069 0.032

>Methylation code ID 1017

0.027 0.000 0.000 0.000 0.000 0.007 0.000 0.000 0.000 0.000

0.000 0.000 0.040 0.000 0.007 0.007 0.000 0.000 0.000 0.000

0.070 0.054 0.069 0.052 0.005 0.000 0.000 0.000 0.000 0.005

0.000 0.000 0.000 0.000 0.000 0.000 0.040 0.000 0.015 0.000

0.000 0.000 0.000 0.000 0.087 0.066 0.074 0.042 0.005 0.000

0.000 0.000 0.000 0.011 0.000 0.000 0.000 0.000 0.000 0.000

0.033 0.000 0.011 0.000 0.000 0.005 0.000 0.000 0.077 0.071

0.054 0.065

>Methylation code ID 1017

0.005 0.000 0.000 0.000 0.000 0.005 0.000 0.000 0.000 0.000

0.000 0.000 0.034 0.000 0.015 0.000 0.000 0.005 0.000 0.000

0.076 0.078 0.060 0.055 0.009 0.000 0.000 0.000 0.000 0.009

0.000 0.000 0.000 0.000 0.000 0.000 0.023 0.000 0.014 0.005

0.000 0.000 0.000 0.000 0.081 0.073 0.047 0.072 0.020 0.000

0.000 0.000 0.000 0.013 0.000 0.000 0.000 0.000 0.000 0.000

0.040 0.000 0.007 0.000 0.000 0.000 0.000 0.000 0.060 0.060

0.059 0.074

>Methylation code ID 1017

0.010 0.000 0.000 0.000 0.000 0.010 0.000 0.000 0.000 0.000

0.000 0.000 0.025 0.000 0.010 0.010 0.000 0.000 0.000 0.000

0.070 0.072 0.063 0.062 0.014 0.000 0.000 0.000 0.000 0.005

0.000 0.000 0.000 0.000 0.000 0.000 0.028 0.000 0.014 0.000

0.000 0.000 0.000 0.000 0.070 0.055 0.070 0.078 0.005 0.000

0.000 0.000 0.000 0.005 0.000 0.000 0.000 0.005 0.000 0.000

0.025 0.000 0.015 0.000 0.000 0.005 0.000 0.000 0.075 0.075

0.047 0.077

>Methylation code ID 1017

0.013 0.000 0.000 0.000 0.000 0.009 0.000 0.000 0.000 0.000

0.000 0.000 0.022 0.000 0.013 0.000 0.000 0.000 0.000 0.000

0.076 0.054 0.081 0.064 0.010 0.000 0.000 0.000 0.000 0.010

0.000 0.000 0.000 0.005 0.000 0.000 0.026 0.000 0.010 0.000

0.000 0.005 0.000 0.000 0.069 0.066 0.049 0.081 0.011 0.000

0.000 0.000 0.000 0.011 0.000 0.000 0.000 0.000 0.000 0.000

0.023 0.000 0.011 0.011 0.000 0.000 0.000 0.000 0.061 0.065

0.066 0.073

>Methylation code ID 1017

0.013 0.000 0.000 0.000 0.000 0.013 0.000 0.000 0.000 0.000

0.000 0.000 0.032 0.000 0.006 0.019 0.000 0.000 0.000 0.000

0.063 0.063 0.053 0.071 0.004 0.000 0.000 0.000 0.000 0.009

0.000 0.000 0.000 0.000 0.000 0.000 0.031 0.000 0.013 0.000

0.000 0.000 0.000 0.000 0.076 0.053 0.076 0.071 0.011 0.000

0.000 0.000 0.000 0.011 0.000 0.000 0.000 0.011 0.000 0.000

0.017 0.000 0.011 0.000 0.000 0.006 0.000 0.000 0.076 0.069

0.049 0.073

>Methylation code ID 1017

0.018 0.000 0.000 0.000 0.000 0.012 0.000 0.000 0.000 0.012

0.000 0.000 0.018 0.000 0.012 0.000 0.000 0.006 0.000 0.000

0.080 0.065 0.034 0.078 0.007 0.000 0.000 0.000 0.000 0.014

0.000 0.000 0.000 0.000 0.000 0.000 0.036 0.000 0.007 0.022

0.000 0.000 0.000 0.000 0.070 0.075 0.038 0.064 0.005 0.005

0.000 0.000 0.000 0.009 0.000 0.000 0.000 0.000 0.000 0.000

0.023 0.000 0.014 0.000 0.000 0.000 0.000 0.000 0.083 0.054

0.077 0.065

>Methylation code ID 1017

0.044 0.000 0.000 0.000 0.000 0.026 0.000 0.000 0.000 0.018

0.000 0.000 0.018 0.000 0.009 0.000 0.000 0.000 0.000 0.000

0.052 0.054 0.051 0.063 0.009 0.000 0.000 0.000 0.000 0.009

0.000 0.000 0.000 0.000 0.000 0.000 0.052 0.000 0.009 0.017

0.000 0.000 0.000 0.000 0.058 0.054 0.046 0.080 0.000 0.008

0.000 0.000 0.000 0.004 0.000 0.000 0.000 0.000 0.000 0.000

0.015 0.000 0.019 0.000 0.000 0.000 0.000 0.000 0.085 0.071

0.062 0.069

>Methylation code ID 1017

0.040 0.010 0.000 0.000 0.000 0.030 0.000 0.000 0.000 0.030

0.000 0.000 0.020 0.000 0.000 0.000 0.000 0.000 0.000 0.000

0.047 0.056 0.048 0.055 0.009 0.000 0.000 0.000 0.000 0.009

0.000 0.000 0.000 0.000 0.000 0.000 0.043 0.000 0.009 0.017

0.000 0.000 0.000 0.000 0.070 0.050 0.053 0.074 0.000 0.008

0.000 0.000 0.000 0.004 0.000 0.000 0.000 0.000 0.000 0.000

0.016 0.000 0.016 0.000 0.000 0.000 0.000 0.000 0.070 0.074

0.067 0.077

>Methylation code ID 1017

0.031 0.000 0.000 0.000 0.000 0.023 0.000 0.000 0.000 0.015

0.000 0.000 0.015 0.000 0.008 0.000 0.008 0.000 0.000 0.000

0.061 0.067 0.054 0.052 0.008 0.000 0.000 0.000 0.000 0.008

0.000 0.000 0.000 0.000 0.000 0.000 0.051 0.000 0.008 0.008

0.000 0.000 0.000 0.000 0.071 0.065 0.052 0.061 0.004 0.008

0.004 0.000 0.000 0.004 0.000 0.000 0.000 0.000 0.000 0.000

0.016 0.000 0.012 0.000 0.000 0.000 0.000 0.000 0.066 0.070

0.058 0.092

>Methylation code ID 1017

0.028 0.000 0.000 0.000 0.000 0.007 0.000 0.000 0.000 0.007

0.000 0.000 0.014 0.000 0.014 0.007 0.007 0.000 0.000 0.000

0.041 0.065 0.074 0.070 0.015 0.007 0.000 0.000 0.000 0.007

0.000 0.000 0.000 0.000 0.000 0.000 0.037 0.007 0.007 0.000

0.000 0.000 0.000 0.000 0.074 0.082 0.037 0.060 0.000 0.005

0.005 0.005 0.000 0.005 0.000 0.000 0.000 0.000 0.000 0.000

0.021 0.005 0.010 0.005 0.000 0.000 0.000 0.000 0.069 0.073

0.064 0.065

>Methylation code ID 1017

0.011 0.005 0.005 0.000 0.000 0.005 0.000 0.000 0.000 0.000

0.000 0.000 0.021 0.005 0.011 0.000 0.000 0.000 0.000 0.000

0.074 0.077 0.050 0.068 0.014 0.000 0.007 0.000 0.000 0.007

0.000 0.000 0.000 0.000 0.000 0.000 0.028 0.007 0.007 0.007

0.007 0.000 0.000 0.000 0.061 0.080 0.066 0.042 0.010 0.005

0.000 0.000 0.000 0.000 0.000 0.000 0.000 0.005 0.000 0.000

0.015 0.000 0.015 0.000 0.005 0.000 0.000 0.000 0.066 0.059

0.065 0.088

>Methylation code ID 1017

0.019 0.000 0.000 0.000 0.000 0.006 0.000 0.000 0.000 0.000

0.000 0.000 0.025 0.006 0.012 0.006 0.006 0.000 0.000 0.000

0.070 0.070 0.055 0.057 0.009 0.000 0.000 0.000 0.000 0.004

0.000 0.000 0.000 0.000 0.000 0.000 0.017 0.004 0.017 0.000

0.000 0.000 0.000 0.000 0.069 0.059 0.069 0.085 0.011 0.005

0.005 0.000 0.000 0.005 0.000 0.000 0.000 0.000 0.000 0.000

0.021 0.005 0.005 0.005 0.000 0.000 0.000 0.000 0.064 0.064

0.060 0.081

>Methylation code ID 1017

0.011 0.005 0.005 0.000 0.000 0.005 0.000 0.000 0.000 0.000

0.000 0.000 0.021 0.005 0.005 0.005 0.000 0.000 0.000 0.000

0.064 0.064 0.060 0.081 0.022 0.000 0.000 0.000 0.000 0.007

0.000 0.000 0.000 0.000 0.000 0.000 0.030 0.007 0.007 0.007

0.007 0.000 0.000 0.000 0.073 0.066 0.046 0.059 0.005 0.000

0.000 0.000 0.000 0.005 0.000 0.000 0.000 0.000 0.000 0.000

0.019 0.005 0.019 0.000 0.000 0.000 0.000 0.000 0.076 0.064

0.063 0.080

>Methylation code ID 1017

0.005 0.000 0.000 0.005 0.000 0.005 0.000 0.000 0.000 0.000

0.000 0.000 0.014 0.005 0.023 0.000 0.000 0.000 0.000 0.000

0.087 0.065 0.062 0.064 0.010 0.005 0.005 0.000 0.000 0.010

0.000 0.000 0.000 0.000 0.000 0.000 0.014 0.000 0.010 0.005

0.000 0.000 0.000 0.000 0.079 0.074 0.054 0.068 0.021 0.000

0.000 0.000 0.000 0.014 0.000 0.000 0.000 0.000 0.000 0.000

0.021 0.007 0.007 0.000 0.007 0.000 0.000 0.000 0.069 0.063

0.056 0.069

>Methylation code ID 1017

0.015 0.005 0.005 0.000 0.000 0.005 0.000 0.000 0.000 0.000

0.000 0.000 0.015 0.000 0.010 0.005 0.000 0.000 0.000 0.000

0.074 0.063 0.052 0.084 0.019 0.000 0.000 0.000 0.000 0.013

0.000 0.000 0.000 0.000 0.000 0.000 0.026 0.006 0.006 0.000

0.006 0.000 0.000 0.000 0.072 0.069 0.047 0.067 0.005 0.000

0.000 0.005 0.000 0.009 0.000 0.000 0.000 0.000 0.000 0.000

0.014 0.005 0.019 0.000 0.000 0.000 0.000 0.000 0.087 0.079

0.048 0.064

>Methylation code ID 1017

0.024 0.000 0.000 0.000 0.000 0.006 0.000 0.000 0.006 0.000

0.006 0.000 0.018 0.000 0.006 0.006 0.000 0.000 0.000 0.000

0.066 0.058 0.058 0.078 0.005 0.000 0.000 0.000 0.000 0.010

0.000 0.000 0.000 0.000 0.000 0.000 0.019 0.005 0.015 0.000

0.000 0.000 0.005 0.000 0.070 0.082 0.058 0.064 0.005 0.000

0.000 0.009 0.000 0.014 0.005 0.000 0.000 0.000 0.000 0.000

0.005 0.005 0.014 0.000 0.000 0.000 0.000 0.000 0.077 0.084

0.051 0.067

>Methylation code ID 1017

0.004 0.000 0.000 0.021 0.000 0.009 0.004 0.000 0.000 0.000

0.000 0.000 0.000 0.000 0.017 0.000 0.000 0.000 0.000 0.000

0.060 0.075 0.065 0.078 0.023 0.000 0.000 0.006 0.000 0.006

0.000 0.000 0.011 0.000 0.006 0.006 0.006 0.000 0.006 0.006

0.000 0.000 0.000 0.000 0.055 0.069 0.071 0.065 0.004 0.000

0.000 0.000 0.000 0.008 0.000 0.000 0.000 0.000 0.000 0.000

0.012 0.004 0.017 0.000 0.000 0.000 0.004 0.000 0.057 0.080

0.072 0.074

>Methylation code ID 1017

0.017 0.006 0.000 0.006 0.000 0.006 0.000 0.006 0.012 0.000

0.006 0.006 0.000 0.000 0.000 0.012 0.000 0.000 0.000 0.000

0.064 0.073 0.074 0.048 0.013 0.000 0.000 0.000 0.000 0.000

0.000 0.000 0.000 0.000 0.000 0.000 0.009 0.000 0.017 0.004

0.004 0.004 0.004 0.000 0.057 0.080 0.069 0.070 0.000 0.000

0.000 0.022 0.000 0.005 0.011 0.000 0.000 0.005 0.000 0.000

0.000 0.000 0.016 0.000 0.000 0.000 0.000 0.000 0.057 0.071

0.057 0.089

>Methylation code ID 1017

0.014 0.007 0.000 0.007 0.000 0.007 0.000 0.014 0.014 0.000

0.000 0.000 0.000 0.000 0.000 0.021 0.000 0.000 0.000 0.000

0.061 0.074 0.060 0.056 0.014 0.005 0.000 0.000 0.005 0.000

0.000 0.000 0.000 0.000 0.000 0.000 0.009 0.000 0.019 0.005

0.000 0.005 0.000 0.000 0.050 0.077 0.070 0.076 0.000 0.000

0.005 0.016 0.000 0.000 0.005 0.000 0.000 0.005 0.000 0.000

0.000 0.000 0.021 0.000 0.000 0.005 0.000 0.000 0.071 0.061

0.052 0.093

>Methylation code ID 1017

0.007 0.007 0.007 0.007 0.000 0.013 0.000 0.000 0.007 0.000

0.007 0.007 0.000 0.007 0.000 0.013 0.000 0.000 0.000 0.000

0.068 0.070 0.064 0.053 0.014 0.010 0.000 0.000 0.000 0.000

0.000 0.000 0.000 0.000 0.000 0.000 0.010 0.000 0.014 0.000

0.010 0.005 0.000 0.000 0.055 0.076 0.067 0.073 0.000 0.000

0.000 0.012 0.000 0.000 0.006 0.000 0.000 0.000 0.000 0.000

0.006 0.000 0.029 0.000 0.000 0.006 0.000 0.000 0.089 0.063

0.072 0.053

>Methylation code ID 1017

0.000 0.007 0.007 0.000 0.000 0.020 0.000 0.014 0.014 0.000

0.000 0.000 0.000 0.007 0.000 0.014 0.000 0.000 0.000 0.000

0.055 0.067 0.069 0.060 0.014 0.005 0.000 0.000 0.005 0.000

0.000 0.000 0.000 0.000 0.000 0.000 0.005 0.000 0.014 0.005

0.005 0.009 0.000 0.000 0.041 0.066 0.083 0.082 0.000 0.000

0.000 0.009 0.000 0.005 0.005 0.000 0.000 0.005 0.000 0.000

0.005 0.000 0.018 0.000 0.000 0.005 0.000 0.000 0.069 0.067

0.050 0.096

>Methylation code ID 1017

0.007 0.000 0.007 0.000 0.000 0.020 0.000 0.007 0.013 0.007

0.000 0.000 0.020 0.007 0.000 0.000 0.000 0.000 0.000 0.000

0.055 0.059 0.064 0.069 0.000 0.000 0.000 0.000 0.000 0.009

0.000 0.000 0.000 0.000 0.000 0.000 0.014 0.000 0.019 0.009

0.005 0.005 0.000 0.000 0.052 0.065 0.080 0.076 0.010 0.000

0.000 0.010 0.000 0.005 0.005 0.000 0.000 0.000 0.000 0.000

0.005 0.000 0.019 0.005 0.000 0.000 0.000 0.000 0.074 0.052

0.071 0.079

>Methylation code ID 1017

0.009 0.000 0.000 0.009 0.000 0.009 0.004 0.000 0.000 0.000

0.000 0.000 0.004 0.000 0.017 0.004 0.000 0.000 0.000 0.000

0.076 0.058 0.061 0.082 0.013 0.000 0.007 0.000 0.000 0.020

0.000 0.007 0.013 0.007 0.000 0.000 0.020 0.000 0.000 0.000

0.000 0.000 0.000 0.000 0.053 0.067 0.064 0.062 0.000 0.000

0.000 0.000 0.000 0.010 0.000 0.000 0.000 0.000 0.000 0.000

0.015 0.000 0.015 0.010 0.005 0.005 0.000 0.000 0.057 0.073

0.073 0.069

>Methylation code ID 1017

0.012 0.000 0.006 0.000 0.000 0.018 0.000 0.006 0.006 0.006

0.000 0.000 0.018 0.000 0.006 0.000 0.000 0.000 0.000 0.000

0.064 0.068 0.059 0.063 0.000 0.000 0.000 0.000 0.000 0.016

0.000 0.000 0.000 0.000 0.000 0.000 0.022 0.000 0.011 0.011

0.005 0.005 0.000 0.000 0.068 0.065 0.061 0.069 0.009 0.000

0.000 0.000 0.000 0.009 0.004 0.000 0.000 0.000 0.000 0.000

0.009 0.000 0.017 0.004 0.000 0.000 0.000 0.000 0.076 0.063

0.068 0.076

>Methylation code ID 1017

0.018 0.000 0.006 0.000 0.000 0.012 0.000 0.006 0.006 0.006

0.000 0.000 0.018 0.000 0.006 0.000 0.000 0.000 0.000 0.000

0.076 0.053 0.055 0.071 0.000 0.000 0.000 0.000 0.000 0.016

0.000 0.000 0.000 0.000 0.000 0.000 0.027 0.000 0.011 0.011

0.005 0.000 0.000 0.000 0.069 0.063 0.069 0.062 0.008 0.000

0.000 0.000 0.000 0.008 0.004 0.000 0.000 0.000 0.000 0.000

0.008 0.004 0.017 0.000 0.000 0.000 0.000 0.000 0.077 0.060

0.068 0.079

>Methylation code ID 1017

0.017 0.000 0.006 0.000 0.000 0.017 0.000 0.006 0.006 0.006

0.000 0.000 0.012 0.000 0.006 0.000 0.000 0.000 0.000 0.000

0.076 0.047 0.067 0.068 0.005 0.000 0.000 0.000 0.000 0.016

0.000 0.000 0.000 0.000 0.000 0.000 0.022 0.000 0.011 0.011

0.005 0.000 0.000 0.000 0.079 0.062 0.054 0.067 0.009 0.000

0.000 0.000 0.000 0.014 0.005 0.000 0.000 0.000 0.000 0.000

0.005 0.005 0.018 0.000 0.000 0.000 0.000 0.000 0.081 0.052

0.075 0.070

>Methylation code ID 1017

0.011 0.000 0.000 0.000 0.000 0.016 0.000 0.000 0.000 0.000

0.000 0.000 0.022 0.000 0.011 0.000 0.011 0.000 0.000 0.000

0.073 0.056 0.068 0.065 0.008 0.000 0.000 0.000 0.000 0.012

0.000 0.000 0.000 0.000 0.000 0.000 0.008 0.004 0.019 0.000

0.000 0.000 0.000 0.000 0.073 0.064 0.065 0.081 0.011 0.000

0.011 0.000 0.000 0.022 0.000 0.000 0.005 0.000 0.000 0.000

0.011 0.000 0.005 0.000 0.000 0.000 0.000 0.000 0.084 0.047

0.073 0.064

>Methylation code ID 1017

0.016 0.000 0.011 0.000 0.000 0.021 0.000 0.000 0.005 0.005

0.000 0.000 0.005 0.000 0.005 0.000 0.000 0.000 0.000 0.000

0.077 0.045 0.079 0.063 0.014 0.000 0.000 0.000 0.000 0.009

0.000 0.000 0.000 0.000 0.000 0.000 0.014 0.000 0.009 0.000

0.009 0.000 0.005 0.000 0.073 0.063 0.063 0.074 0.000 0.000

0.000 0.000 0.000 0.017 0.000 0.000 0.000 0.000 0.000 0.000

0.013 0.004 0.017 0.000 0.000 0.000 0.000 0.000 0.072 0.062

0.059 0.089

>Methylation code ID 1017

0.018 0.000 0.006 0.000 0.000 0.030 0.000 0.000 0.006 0.006

0.000 0.000 0.006 0.000 0.006 0.000 0.000 0.000 0.000 0.000

0.079 0.041 0.073 0.064 0.013 0.000 0.000 0.000 0.000 0.013

0.000 0.000 0.000 0.000 0.000 0.000 0.013 0.000 0.009 0.000

0.004 0.000 0.004 0.000 0.077 0.056 0.072 0.070 0.000 0.000

0.000 0.000 0.000 0.023 0.000 0.000 0.000 0.000 0.000 0.000

0.014 0.005 0.014 0.000 0.000 0.000 0.000 0.000 0.069 0.053

0.064 0.093

>Methylation code ID 1017

0.013 0.000 0.006 0.000 0.000 0.026 0.000 0.000 0.006 0.006

0.000 0.000 0.013 0.000 0.006 0.000 0.006 0.000 0.000 0.000

0.076 0.033 0.075 0.066 0.013 0.000 0.000 0.000 0.000 0.009

0.004 0.000 0.000 0.000 0.000 0.000 0.013 0.000 0.009 0.000

0.004 0.000 0.004 0.000 0.080 0.065 0.071 0.061 0.000 0.000

0.004 0.000 0.000 0.018 0.000 0.000 0.000 0.000 0.000 0.000

0.009 0.004 0.013 0.000 0.004 0.000 0.000 0.000 0.070 0.054

0.066 0.090

>Methylation code ID 1017

0.000 0.000 0.004 0.000 0.000 0.016 0.000 0.000 0.000 0.000

0.000 0.000 0.008 0.004 0.016 0.000 0.004 0.000 0.000 0.000

0.068 0.055 0.069 0.090 0.015 0.000 0.007 0.000 0.000 0.037

0.000 0.000 0.007 0.007 0.000 0.000 0.015 0.000 0.000 0.000

0.007 0.000 0.000 0.000 0.074 0.040 0.063 0.060 0.014 0.000

0.000 0.000 0.000 0.005 0.005 0.000 0.000 0.000 0.000 0.000

0.014 0.000 0.009 0.000 0.005 0.000 0.005 0.000 0.082 0.057

0.076 0.064

>Methylation code ID 1017

0.022 0.000 0.000 0.000 0.000 0.056 0.000 0.000 0.000 0.022

0.000 0.000 0.022 0.000 0.000 0.000 0.011 0.000 0.000 0.000

0.046 0.047 0.050 0.056 0.019 0.005 0.000 0.000 0.000 0.005

0.005 0.000 0.000 0.000 0.000 0.000 0.014 0.000 0.005 0.000

0.000 0.000 0.009 0.000 0.074 0.063 0.076 0.060 0.000 0.000

0.004 0.000 0.000 0.012 0.000 0.000 0.000 0.000 0.000 0.000

0.008 0.000 0.021 0.000 0.004 0.000 0.000 0.000 0.058 0.069

0.083 0.074

>Methylation code ID 1017

0.022 0.000 0.000 0.000 0.000 0.056 0.000 0.000 0.000 0.022

0.000 0.000 0.022 0.000 0.000 0.000 0.011 0.000 0.000 0.000

0.046 0.047 0.050 0.056 0.017 0.004 0.000 0.000 0.000 0.004

0.004 0.000 0.000 0.000 0.000 0.000 0.009 0.000 0.009 0.000

0.000 0.000 0.009 0.000 0.072 0.066 0.074 0.065 0.004 0.000

0.004 0.000 0.000 0.013 0.000 0.000 0.000 0.000 0.000 0.000

0.009 0.000 0.017 0.000 0.004 0.000 0.000 0.000 0.061 0.062

0.076 0.082

>Methylation code ID 1017

0.014 0.000 0.000 0.000 0.000 0.021 0.000 0.007 0.000 0.007

0.000 0.000 0.021 0.000 0.007 0.000 0.007 0.000 0.000 0.000

0.059 0.069 0.062 0.059 0.017 0.004 0.000 0.000 0.000 0.000

0.004 0.000 0.000 0.000 0.000 0.000 0.008 0.000 0.013 0.000

0.000 0.000 0.004 0.000 0.061 0.072 0.062 0.087 0.014 0.000

0.005 0.000 0.000 0.005 0.000 0.000 0.000 0.000 0.000 0.000

0.005 0.000 0.023 0.000 0.005 0.000 0.000 0.000 0.068 0.081

0.079 0.051

>Methylation code ID 1017

0.012 0.000 0.004 0.000 0.000 0.004 0.000 0.000 0.000 0.000

0.000 0.000 0.008 0.000 0.024 0.000 0.000 0.000 0.000 0.000

0.072 0.068 0.085 0.056 0.012 0.000 0.006 0.000 0.000 0.019

0.000 0.006 0.000 0.012 0.000 0.000 0.012 0.000 0.006 0.000

0.000 0.000 0.000 0.000 0.062 0.062 0.063 0.072 0.018 0.004

0.000 0.000 0.000 0.000 0.000 0.000 0.000 0.000 0.000 0.000

0.004 0.000 0.013 0.000 0.004 0.000 0.004 0.000 0.065 0.069

0.066 0.085

>Methylation code ID 1017

0.012 0.000 0.004 0.000 0.000 0.004 0.000 0.000 0.000 0.000

0.000 0.000 0.008 0.000 0.020 0.000 0.000 0.004 0.000 0.000

0.072 0.077 0.078 0.055 0.012 0.000 0.006 0.000 0.000 0.012

0.000 0.006 0.000 0.012 0.000 0.000 0.012 0.000 0.006 0.006

0.000 0.000 0.000 0.000 0.062 0.065 0.062 0.072 0.012 0.008

0.000 0.000 0.000 0.000 0.000 0.000 0.000 0.000 0.000 0.000

0.004 0.000 0.012 0.000 0.004 0.000 0.004 0.000 0.063 0.077

0.057 0.092

>Methylation code ID 1017

0.011 0.008 0.000 0.000 0.000 0.000 0.000 0.000 0.000 0.000

0.000 0.000 0.004 0.004 0.011 0.000 0.004 0.000 0.004 0.000

0.066 0.074 0.058 0.089 0.012 0.000 0.004 0.000 0.000 0.004

0.000 0.000 0.004 0.000 0.000 0.000 0.008 0.000 0.016 0.000

0.000 0.004 0.000 0.000 0.079 0.075 0.067 0.062 0.012 0.000

0.006 0.000 0.000 0.006 0.000 0.006 0.000 0.012 0.000 0.000

0.012 0.000 0.006 0.006 0.000 0.000 0.000 0.000 0.064 0.066

0.065 0.071

>Methylation code ID 1017

0.012 0.000 0.006 0.000 0.000 0.006 0.000 0.006 0.000 0.012

0.000 0.000 0.012 0.000 0.006 0.012 0.000 0.000 0.000 0.000

0.061 0.065 0.066 0.069 0.008 0.008 0.000 0.000 0.000 0.000

0.000 0.000 0.000 0.000 0.000 0.000 0.004 0.004 0.012 0.000

0.004 0.000 0.004 0.000 0.070 0.071 0.060 0.090 0.013 0.000

0.004 0.000 0.000 0.004 0.000 0.000 0.004 0.000 0.000 0.000

0.009 0.000 0.013 0.000 0.000 0.004 0.000 0.000 0.077 0.072

0.076 0.056

>Methylation code ID 1017

0.012 0.004 0.004 0.000 0.000 0.004 0.000 0.000 0.004 0.000

0.000 0.000 0.008 0.000 0.012 0.000 0.000 0.004 0.000 0.000

0.080 0.068 0.067 0.067 0.018 0.000 0.006 0.000 0.000 0.000

0.000 0.006 0.000 0.012 0.000 0.000 0.012 0.000 0.006 0.012

0.000 0.000 0.000 0.000 0.061 0.066 0.062 0.074 0.004 0.009

0.000 0.000 0.000 0.000 0.000 0.000 0.000 0.000 0.000 0.000

0.004 0.004 0.013 0.000 0.004 0.000 0.004 0.000 0.069 0.076

0.060 0.085

>Methylation code ID 1019

0.008 0.008 0.000 0.000 0.008 0.024 0.000 0.008 0.000 0.008

0.008 0.008 0.000 0.000 0.000 0.008 0.000 0.000 0.000 0.000

0.061 0.068 0.056 0.061 0.015 0.005 0.000 0.000 0.000 0.000

0.000 0.000 0.000 0.000 0.000 0.000 0.005 0.000 0.005 0.010

0.005 0.000 0.015 0.000 0.087 0.065 0.073 0.051 0.000 0.004

0.000 0.000 0.000 0.013 0.004 0.000 0.000 0.004 0.000 0.000

0.004 0.004 0.009 0.000 0.000 0.004 0.000 0.000 0.054 0.073

0.080 0.078

>Methylation code ID 1019

0.009 0.009 0.000 0.000 0.009 0.026 0.000 0.000 0.000 0.009

0.009 0.009 0.000 0.000 0.000 0.009 0.000 0.000 0.000 0.000

0.065 0.070 0.049 0.063 0.014 0.010 0.000 0.000 0.000 0.000

0.000 0.000 0.000 0.000 0.000 0.000 0.005 0.000 0.005 0.005

0.005 0.000 0.014 0.000 0.084 0.060 0.077 0.056 0.000 0.004

0.000 0.000 0.000 0.013 0.000 0.000 0.000 0.004 0.000 0.000

0.004 0.004 0.013 0.000 0.000 0.004 0.000 0.000 0.050 0.074

0.080 0.082

>Methylation code ID 1019

0.006 0.000 0.000 0.000 0.011 0.011 0.000 0.000 0.000 0.006

0.000 0.006 0.006 0.000 0.006 0.000 0.000 0.000 0.006 0.006

0.075 0.051 0.070 0.076 0.018 0.006 0.000 0.000 0.000 0.006

0.000 0.000 0.000 0.000 0.000 0.000 0.012 0.000 0.000 0.012

0.012 0.000 0.012 0.000 0.079 0.046 0.072 0.056 0.005 0.000

0.000 0.000 0.000 0.010 0.000 0.000 0.000 0.010 0.000 0.000

0.010 0.005 0.015 0.000 0.000 0.000 0.000 0.000 0.054 0.071

0.075 0.079

>Methylation code ID 1019

0.026 0.013 0.000 0.000 0.000 0.006 0.000 0.000 0.000 0.000

0.000 0.000 0.006 0.000 0.000 0.006 0.013 0.000 0.013 0.000

0.082 0.038 0.074 0.056 0.007 0.000 0.000 0.000 0.000 0.013

0.000 0.000 0.000 0.020 0.000 0.000 0.020 0.007 0.007 0.000

0.000 0.000 0.000 0.000 0.064 0.055 0.069 0.073 0.000 0.005

0.000 0.000 0.005 0.010 0.000 0.000 0.000 0.010 0.000 0.005

0.005 0.000 0.005 0.000 0.000 0.000 0.005 0.005 0.072 0.053

0.064 0.090

>Methylation code ID 1019

0.000 0.005 0.000 0.005 0.000 0.014 0.000 0.000 0.005 0.009

0.000 0.005 0.005 0.000 0.005 0.000 0.000 0.000 0.000 0.009

0.057 0.071 0.067 0.077 0.018 0.006 0.000 0.006 0.000 0.000

0.000 0.000 0.000 0.000 0.006 0.000 0.006 0.000 0.006 0.012

0.012 0.000 0.000 0.000 0.064 0.070 0.062 0.065 0.005 0.000

0.000 0.005 0.000 0.000 0.000 0.000 0.000 0.005 0.000 0.000

0.015 0.005 0.010 0.000 0.000 0.000 0.005 0.000 0.049 0.077

0.073 0.085

>Methylation code ID 1019

0.013 0.000 0.000 0.006 0.000 0.006 0.000 0.000 0.000 0.006

0.006 0.000 0.019 0.000 0.006 0.013 0.000 0.000 0.000 0.000

0.070 0.066 0.062 0.059 0.018 0.000 0.000 0.000 0.000 0.000

0.000 0.000 0.000 0.000 0.000 0.000 0.030 0.000 0.012 0.006

0.000 0.000 0.006 0.000 0.048 0.070 0.064 0.079 0.000 0.000

0.000 0.006 0.000 0.017 0.000 0.000 0.000 0.006 0.000 0.000

0.028 0.000 0.011 0.000 0.000 0.000 0.000 0.000 0.073 0.078

0.033 0.082

>Methylation code ID 1019

0.010 0.000 0.005 0.000 0.000 0.000 0.000 0.000 0.000 0.000

0.000 0.000 0.030 0.000 0.010 0.005 0.005 0.000 0.000 0.000

0.072 0.064 0.061 0.072 0.005 0.000 0.005 0.000 0.000 0.005

0.000 0.000 0.000 0.005 0.000 0.000 0.026 0.005 0.010 0.000

0.005 0.000 0.000 0.000 0.057 0.080 0.074 0.055 0.013 0.000

0.000 0.000 0.000 0.007 0.000 0.000 0.000 0.000 0.000 0.007

0.027 0.007 0.007 0.000 0.007 0.000 0.000 0.000 0.075 0.048

0.087 0.050

>Methylation code ID 1019

0.000 0.000 0.005 0.005 0.000 0.005 0.000 0.000 0.000 0.005

0.000 0.000 0.027 0.005 0.011 0.000 0.005 0.000 0.000 0.000

0.065 0.074 0.064 0.061 0.011 0.000 0.000 0.000 0.000 0.006

0.000 0.000 0.000 0.000 0.000 0.006 0.022 0.000 0.011 0.006

0.006 0.000 0.000 0.000 0.082 0.044 0.088 0.053 0.012 0.000

0.006 0.000 0.000 0.006 0.000 0.000 0.000 0.000 0.000 0.000

0.037 0.000 0.006 0.000 0.006 0.000 0.000 0.000 0.068 0.060

0.057 0.075

>Methylation code ID 1019

0.000 0.000 0.000 0.000 0.000 0.010 0.000 0.000 0.000 0.000

0.000 0.005 0.015 0.000 0.019 0.005 0.005 0.000 0.000 0.000

0.076 0.058 0.079 0.062 0.027 0.000 0.007 0.007 0.000 0.013

0.000 0.000 0.000 0.000 0.000 0.000 0.020 0.000 0.007 0.000

0.007 0.000 0.000 0.000 0.075 0.058 0.049 0.065 0.000 0.000

0.007 0.007 0.000 0.000 0.000 0.000 0.000 0.000 0.000 0.000

0.039 0.007 0.013 0.000 0.007 0.000 0.000 0.000 0.061 0.067

0.060 0.067

>Methylation code ID 1019

0.027 0.000 0.007 0.007 0.000 0.013 0.000 0.000 0.000 0.000

0.000 0.000 0.020 0.000 0.007 0.000 0.007 0.000 0.000 0.000

0.075 0.058 0.049 0.065 0.000 0.000 0.006 0.006 0.000 0.006

0.000 0.000 0.000 0.000 0.000 0.000 0.034 0.006 0.011 0.000

0.006 0.000 0.000 0.000 0.067 0.074 0.057 0.062 0.000 0.000

0.000 0.000 0.000 0.010 0.000 0.000 0.000 0.000 0.000 0.005

0.015 0.000 0.019 0.005 0.005 0.000 0.000 0.000 0.076 0.058

0.079 0.062

>Methylation code ID 1019

0.024 0.000 0.000 0.008 0.000 0.016 0.000 0.000 0.000 0.000

0.000 0.000 0.024 0.000 0.008 0.008 0.008 0.000 0.000 0.000

0.061 0.055 0.050 0.071 0.000 0.000 0.005 0.010 0.000 0.005

0.000 0.000 0.000 0.000 0.000 0.000 0.024 0.005 0.010 0.000

0.000 0.005 0.000 0.000 0.060 0.071 0.064 0.076 0.000 0.000

0.000 0.000 0.000 0.011 0.000 0.000 0.005 0.000 0.000 0.005

0.011 0.000 0.016 0.005 0.005 0.000 0.000 0.000 0.075 0.065

0.066 0.069

>Methylation code ID 1019

0.030 0.000 0.000 0.010 0.000 0.020 0.000 0.000 0.000 0.000

0.000 0.000 0.040 0.000 0.000 0.010 0.010 0.000 0.000 0.000

0.057 0.047 0.051 0.059 0.000 0.000 0.000 0.009 0.000 0.005

0.000 0.000 0.000 0.000 0.000 0.000 0.023 0.005 0.014 0.000

0.000 0.005 0.000 0.000 0.065 0.073 0.070 0.066 0.000 0.000

0.000 0.000 0.000 0.011 0.000 0.000 0.005 0.000 0.000 0.005

0.011 0.000 0.016 0.011 0.000 0.000 0.000 0.000 0.077 0.063

0.063 0.070

>Methylation code ID 1019

0.000 0.000 0.000 0.000 0.000 0.016 0.000 0.000 0.005 0.000

0.000 0.005 0.011 0.000 0.016 0.011 0.000 0.000 0.000 0.000

0.075 0.070 0.059 0.065 0.029 0.000 0.000 0.010 0.000 0.029

0.000 0.000 0.000 0.000 0.000 0.000 0.029 0.000 0.000 0.010

0.010 0.000 0.000 0.000 0.064 0.040 0.050 0.062 0.000 0.000

0.000 0.010 0.000 0.005 0.000 0.000 0.000 0.000 0.000 0.000

0.019 0.005 0.014 0.000 0.000 0.005 0.000 0.000 0.062 0.076

0.074 0.064

>Methylation code ID 1019

0.006 0.000 0.000 0.000 0.000 0.018 0.000 0.000 0.006 0.006

0.000 0.000 0.006 0.006 0.018 0.006 0.000 0.000 0.000 0.000

0.062 0.100 0.065 0.036 0.016 0.000 0.000 0.008 0.000 0.024

0.000 0.000 0.000 0.008 0.000 0.000 0.016 0.000 0.000 0.016

0.000 0.000 0.008 0.000 0.072 0.056 0.051 0.060 0.010 0.000

0.000 0.005 0.000 0.010 0.000 0.000 0.000 0.000 0.000 0.000

0.010 0.000 0.015 0.000 0.000 0.010 0.000 0.000 0.070 0.070

0.077 0.056

>Methylation code ID 1019

0.000 0.000 0.000 0.007 0.000 0.029 0.000 0.000 0.000 0.007

0.000 0.000 0.015 0.000 0.000 0.015 0.007 0.000 0.007 0.000

0.065 0.052 0.069 0.059 0.014 0.000 0.000 0.005 0.000 0.009

0.000 0.000 0.000 0.000 0.000 0.000 0.005 0.000 0.014 0.000

0.000 0.009 0.005 0.000 0.081 0.064 0.074 0.053 0.006 0.000

0.000 0.000 0.000 0.019 0.006 0.000 0.006 0.006 0.000 0.000

0.006 0.006 0.006 0.006 0.000 0.000 0.000 0.000 0.067 0.072

0.065 0.059

>Methylation code ID 1019

0.012 0.000 0.000 0.000 0.000 0.019 0.006 0.000 0.000 0.006

0.000 0.000 0.019 0.006 0.006 0.006 0.000 0.000 0.000 0.000

0.060 0.060 0.061 0.072 0.000 0.000 0.000 0.000 0.000 0.019

0.000 0.000 0.000 0.005 0.000 0.000 0.009 0.000 0.014 0.005

0.005 0.000 0.005 0.000 0.094 0.039 0.089 0.051 0.024 0.000

0.000 0.000 0.000 0.018 0.000 0.000 0.000 0.000 0.000 0.000

0.006 0.000 0.006 0.000 0.000 0.012 0.006 0.000 0.064 0.058

0.066 0.073

>Methylation code ID 1019

0.018 0.000 0.000 0.000 0.000 0.024 0.000 0.000 0.000 0.000

0.000 0.000 0.018 0.000 0.006 0.000 0.000 0.006 0.006 0.000

0.072 0.056 0.063 0.065 0.015 0.000 0.000 0.000 0.000 0.022

0.015 0.000 0.000 0.007 0.000 0.000 0.022 0.000 0.000 0.007

0.000 0.007 0.000 0.000 0.057 0.061 0.060 0.058 0.000 0.000

0.000 0.000 0.000 0.013 0.000 0.000 0.000 0.004 0.000 0.000

0.009 0.000 0.013 0.004 0.004 0.000 0.004 0.000 0.088 0.042

0.090 0.059

>Methylation code ID 1019

0.021 0.000 0.000 0.000 0.000 0.021 0.000 0.000 0.000 0.007

0.000 0.000 0.021 0.000 0.007 0.000 0.000 0.007 0.007 0.000

0.072 0.059 0.055 0.057 0.006 0.006 0.000 0.000 0.000 0.017

0.012 0.000 0.000 0.006 0.000 0.000 0.012 0.000 0.006 0.006

0.000 0.006 0.000 0.000 0.065 0.065 0.058 0.070 0.005 0.000

0.000 0.000 0.000 0.014 0.000 0.000 0.000 0.000 0.000 0.000

0.005 0.000 0.014 0.005 0.009 0.000 0.005 0.000 0.088 0.045

0.088 0.058

>Methylation code ID 1019

0.013 0.000 0.000 0.000 0.000 0.004 0.000 0.000 0.000 0.004

0.000 0.000 0.004 0.000 0.013 0.004 0.004 0.000 0.009 0.000

0.077 0.063 0.073 0.063 0.018 0.000 0.000 0.000 0.000 0.036

0.000 0.000 0.000 0.027 0.000 0.000 0.027 0.000 0.000 0.000

0.000 0.000 0.009 0.000 0.048 0.057 0.052 0.059 0.005 0.010

0.000 0.000 0.000 0.005 0.005 0.000 0.000 0.005 0.000 0.000

0.010 0.000 0.005 0.005 0.000 0.005 0.000 0.000 0.050 0.081

0.069 0.076

>Methylation code ID 1019

0.007 0.000 0.000 0.000 0.000 0.021 0.000 0.000 0.000 0.021

0.000 0.000 0.021 0.000 0.007 0.000 0.000 0.007 0.007 0.000

0.062 0.065 0.053 0.064 0.011 0.011 0.000 0.000 0.000 0.005

0.005 0.000 0.000 0.005 0.000 0.000 0.005 0.000 0.005 0.011

0.000 0.005 0.000 0.000 0.055 0.080 0.074 0.059 0.015 0.000

0.000 0.000 0.000 0.005 0.000 0.000 0.000 0.005 0.000 0.000

0.005 0.000 0.010 0.005 0.005 0.000 0.010 0.000 0.067 0.076

0.079 0.051

>Methylation code ID 1019

0.006 0.000 0.000 0.000 0.000 0.012 0.006 0.000 0.000 0.023

0.000 0.000 0.006 0.000 0.006 0.006 0.000 0.006 0.006 0.000

0.072 0.058 0.061 0.066 0.018 0.018 0.000 0.000 0.000 0.009

0.000 0.000 0.000 0.009 0.000 0.000 0.018 0.000 0.000 0.027

0.009 0.000 0.000 0.000 0.053 0.059 0.065 0.049 0.010 0.000

0.000 0.000 0.000 0.005 0.000 0.000 0.000 0.010 0.000 0.000

0.020 0.000 0.010 0.000 0.000 0.000 0.005 0.000 0.063 0.086

0.075 0.050

>Methylation code ID 1019

0.029 0.010 0.000 0.000 0.000 0.010 0.000 0.000 0.000 0.010

0.000 0.000 0.020 0.000 0.000 0.029 0.010 0.000 0.000 0.000

0.061 0.055 0.062 0.038 0.005 0.000 0.000 0.000 0.000 0.005

0.000 0.000 0.000 0.010 0.000 0.000 0.020 0.005 0.010 0.005

0.000 0.000 0.005 0.000 0.064 0.076 0.073 0.057 0.005 0.000

0.000 0.000 0.000 0.010 0.005 0.000 0.000 0.010 0.000 0.000

0.005 0.000 0.010 0.005 0.000 0.005 0.005 0.000 0.074 0.065

0.059 0.073

>Methylation code ID 1019

0.025 0.008 0.000 0.000 0.000 0.000 0.000 0.000 0.000 0.017

0.008 0.000 0.017 0.000 0.000 0.017 0.008 0.008 0.000 0.000

0.060 0.054 0.049 0.061 0.006 0.006 0.000 0.000 0.000 0.000

0.000 0.000 0.000 0.006 0.000 0.000 0.023 0.011 0.006 0.011

0.000 0.000 0.006 0.000 0.054 0.062 0.075 0.069 0.000 0.000

0.000 0.000 0.000 0.005 0.010 0.000 0.000 0.005 0.000 0.000

0.010 0.000 0.010 0.010 0.000 0.005 0.000 0.000 0.078 0.054

0.065 0.079

>Methylation code ID 1019

0.000 0.004 0.000 0.000 0.000 0.016 0.000 0.000 0.000 0.000

0.000 0.000 0.008 0.000 0.016 0.000 0.000 0.000 0.000 0.000

0.051 0.100 0.088 0.049 0.033 0.008 0.008 0.000 0.000 0.033

0.000 0.000 0.000 0.008 0.000 0.000 0.000 0.000 0.008 0.000

0.000 0.000 0.000 0.000 0.060 0.047 0.060 0.068 0.011 0.000

0.000 0.006 0.000 0.017 0.000 0.000 0.000 0.000 0.006 0.000

0.011 0.006 0.006 0.000 0.000 0.000 0.006 0.000 0.077 0.056

0.072 0.061

>Methylation code ID 1019

0.039 0.000 0.008 0.008 0.000 0.031 0.000 0.000 0.000 0.000

0.000 0.000 0.000 0.000 0.008 0.000 0.000 0.000 0.000 0.000

0.066 0.062 0.041 0.071 0.010 0.000 0.000 0.005 0.000 0.015

0.000 0.000 0.000 0.005 0.005 0.000 0.010 0.005 0.010 0.000

0.000 0.000 0.000 0.000 0.086 0.054 0.070 0.056 0.000 0.004

0.000 0.000 0.000 0.016 0.000 0.000 0.000 0.000 0.000 0.000

0.004 0.000 0.020 0.000 0.000 0.000 0.000 0.000 0.057 0.093

0.081 0.059

>Methylation code ID 1019

0.010 0.000 0.000 0.000 0.000 0.010 0.000 0.000 0.000 0.005

0.005 0.000 0.019 0.000 0.015 0.000 0.000 0.000 0.000 0.000

0.084 0.070 0.073 0.043 0.000 0.008 0.004 0.000 0.000 0.016

0.000 0.000 0.000 0.000 0.000 0.000 0.012 0.000 0.012 0.000

0.000 0.000 0.000 0.000 0.057 0.091 0.074 0.060 0.030 0.000

0.006 0.006 0.000 0.012 0.000 0.000 0.000 0.000 0.000 0.000

0.006 0.006 0.006 0.000 0.000 0.000 0.000 0.000 0.059 0.062

0.063 0.078

>Methylation code ID 1019

0.028 0.000 0.006 0.006 0.000 0.011 0.000 0.000 0.000 0.000

0.000 0.000 0.006 0.006 0.006 0.000 0.006 0.000 0.000 0.000

0.057 0.065 0.062 0.075 0.010 0.000 0.000 0.000 0.000 0.005

0.000 0.000 0.000 0.010 0.005 0.000 0.019 0.000 0.014 0.000

0.000 0.000 0.000 0.000 0.082 0.074 0.072 0.043 0.000 0.009

0.004 0.000 0.000 0.013 0.000 0.000 0.000 0.000 0.000 0.000

0.013 0.000 0.013 0.000 0.000 0.000 0.000 0.000 0.062 0.100

0.068 0.052

>Methylation code ID 1019

0.004 0.000 0.000 0.004 0.000 0.004 0.000 0.000 0.004 0.017

0.000 0.000 0.008 0.000 0.008 0.000 0.000 0.000 0.004 0.000

0.067 0.068 0.078 0.066 0.004 0.004 0.009 0.000 0.000 0.004

0.000 0.000 0.000 0.000 0.000 0.000 0.013 0.000 0.004 0.009

0.000 0.000 0.009 0.000 0.062 0.065 0.070 0.081 0.018 0.000

0.000 0.009 0.000 0.027 0.000 0.009 0.000 0.009 0.000 0.000

0.018 0.009 0.000 0.000 0.009 0.000 0.000 0.000 0.060 0.068

0.044 0.056

>Methylation code ID 1019

0.004 0.000 0.008 0.000 0.000 0.004 0.000 0.000 0.000 0.000

0.000 0.000 0.008 0.000 0.008 0.008 0.000 0.000 0.011 0.000

0.071 0.076 0.070 0.067 0.005 0.000 0.000 0.005 0.000 0.026

0.000 0.005 0.000 0.005 0.000 0.000 0.005 0.000 0.010 0.000

0.005 0.000 0.000 0.000 0.054 0.085 0.068 0.058 0.010 0.000

0.000 0.010 0.000 0.005 0.000 0.000 0.005 0.015 0.000 0.000

0.000 0.000 0.005 0.000 0.000 0.000 0.005 0.000 0.059 0.056

0.082 0.079

>Methylation code ID 1019

0.010 0.000 0.000 0.010 0.000 0.010 0.000 0.000 0.010 0.005

0.000 0.000 0.000 0.005 0.005 0.000 0.000 0.000 0.005 0.000

0.070 0.058 0.075 0.072 0.004 0.000 0.004 0.004 0.000 0.004

0.000 0.000 0.000 0.000 0.000 0.000 0.008 0.000 0.012 0.008

0.000 0.000 0.008 0.000 0.073 0.079 0.076 0.055 0.010 0.000

0.000 0.005 0.000 0.021 0.000 0.005 0.005 0.005 0.000 0.000

0.000 0.000 0.010 0.000 0.000 0.000 0.000 0.000 0.059 0.081

0.062 0.069

>Methylation code ID 1019

0.016 0.000 0.000 0.010 0.000 0.005 0.000 0.000 0.010 0.005

0.000 0.000 0.000 0.005 0.005 0.000 0.000 0.000 0.005 0.000

0.069 0.063 0.074 0.065 0.000 0.000 0.004 0.004 0.000 0.004

0.000 0.000 0.000 0.000 0.000 0.000 0.011 0.000 0.011 0.008

0.000 0.000 0.008 0.000 0.081 0.070 0.077 0.056 0.010 0.000

0.000 0.005 0.000 0.026 0.000 0.005 0.005 0.000 0.000 0.000

0.000 0.000 0.010 0.000 0.000 0.000 0.000 0.000 0.054 0.088

0.061 0.069

>Methylation code ID 1019

0.011 0.006 0.000 0.006 0.000 0.011 0.000 0.000 0.006 0.006

0.000 0.000 0.006 0.006 0.006 0.006 0.000 0.000 0.000 0.000

0.062 0.061 0.068 0.075 0.005 0.000 0.000 0.009 0.000 0.005

0.000 0.000 0.000 0.009 0.000 0.000 0.009 0.000 0.009 0.005

0.000 0.000 0.009 0.000 0.073 0.066 0.071 0.063 0.007 0.000

0.000 0.000 0.000 0.028 0.000 0.000 0.007 0.014 0.000 0.000

0.000 0.000 0.007 0.014 0.000 0.007 0.000 0.000 0.070 0.073

0.074 0.030

>Methylation code ID 1019

0.006 0.000 0.000 0.006 0.000 0.012 0.000 0.000 0.006 0.012

0.000 0.000 0.019 0.000 0.000 0.006 0.000 0.006 0.006 0.000

0.072 0.055 0.066 0.060 0.011 0.000 0.000 0.000 0.000 0.005

0.000 0.000 0.005 0.016 0.000 0.000 0.000 0.000 0.011 0.011

0.000 0.005 0.000 0.000 0.056 0.061 0.078 0.074 0.005 0.005

0.000 0.000 0.000 0.010 0.000 0.000 0.010 0.005 0.000 0.000

0.010 0.000 0.005 0.005 0.000 0.000 0.005 0.000 0.067 0.061

0.065 0.081

>Methylation code ID 1019

0.000 0.013 0.000 0.000 0.000 0.026 0.000 0.000 0.007 0.000

0.000 0.000 0.013 0.007 0.000 0.013 0.007 0.000 0.000 0.000

0.051 0.048 0.072 0.077 0.028 0.000 0.000 0.000 0.000 0.011

0.006 0.000 0.011 0.006 0.000 0.000 0.006 0.000 0.006 0.000

0.000 0.000 0.000 0.000 0.069 0.063 0.045 0.084 0.000 0.000

0.000 0.000 0.000 0.005 0.005 0.000 0.005 0.009 0.000 0.000

0.009 0.000 0.009 0.000 0.005 0.005 0.005 0.000 0.073 0.068

0.059 0.077

>Methylation code ID 1027

0.006 0.012 0.000 0.000 0.000 0.018 0.000 0.000 0.000 0.000

0.000 0.000 0.012 0.006 0.006 0.012 0.006 0.000 0.000 0.000

0.053 0.049 0.075 0.077 0.033 0.000 0.000 0.000 0.000 0.013

0.007 0.000 0.007 0.007 0.000 0.000 0.007 0.000 0.013 0.000

0.000 0.000 0.000 0.000 0.062 0.063 0.061 0.062 0.000 0.000

0.000 0.006 0.000 0.006 0.006 0.000 0.000 0.011 0.000 0.000

0.011 0.000 0.006 0.006 0.006 0.006 0.006 0.000 0.073 0.075

0.050 0.068

>Methylation code ID 1027

0.000 0.000 0.000 0.000 0.000 0.012 0.006 0.000 0.000 0.012

0.000 0.000 0.012 0.000 0.006 0.006 0.012 0.006 0.006 0.000

0.072 0.068 0.053 0.064 0.015 0.015 0.000 0.000 0.000 0.023

0.008 0.000 0.000 0.000 0.000 0.000 0.015 0.008 0.000 0.008

0.008 0.000 0.000 0.000 0.058 0.057 0.064 0.057 0.025 0.000

0.000 0.000 0.000 0.006 0.012 0.000 0.006 0.006 0.000 0.000

0.006 0.000 0.012 0.000 0.000 0.000 0.000 0.000 0.066 0.064

0.059 0.069

>Methylation code ID 1027

0.000 0.007 0.000 0.000 0.000 0.020 0.007 0.000 0.000 0.013

0.000 0.000 0.007 0.000 0.007 0.007 0.013 0.007 0.000 0.000

0.081 0.056 0.055 0.057 0.015 0.015 0.000 0.000 0.000 0.023

0.008 0.000 0.000 0.000 0.000 0.000 0.008 0.008 0.000 0.008

0.008 0.000 0.000 0.000 0.066 0.068 0.052 0.056 0.015 0.000

0.000 0.000 0.000 0.005 0.010 0.005 0.005 0.000 0.000 0.000

0.000 0.000 0.010 0.000 0.000 0.000 0.005 0.000 0.064 0.069

0.067 0.078

>Methylation code ID 1027

0.006 0.006 0.000 0.000 0.000 0.000 0.017 0.006 0.000 0.000

0.000 0.000 0.006 0.000 0.006 0.000 0.011 0.000 0.006 0.000

0.059 0.064 0.070 0.079 0.006 0.012 0.000 0.000 0.000 0.012

0.006 0.000 0.000 0.006 0.000 0.000 0.012 0.000 0.006 0.000

0.017 0.000 0.000 0.000 0.069 0.050 0.061 0.078 0.006 0.006

0.000 0.000 0.000 0.006 0.018 0.000 0.000 0.000 0.000 0.000

0.012 0.000 0.006 0.000 0.006 0.000 0.000 0.000 0.063 0.065

0.060 0.084

>Methylation code ID 1027

0.006 0.006 0.000 0.000 0.000 0.000 0.019 0.006 0.000 0.000

0.000 0.006 0.006 0.000 0.000 0.000 0.012 0.000 0.006 0.000

0.062 0.056 0.062 0.085 0.006 0.012 0.000 0.000 0.000 0.006

0.006 0.000 0.000 0.006 0.000 0.000 0.012 0.000 0.006 0.000

0.023 0.000 0.000 0.000 0.076 0.047 0.053 0.081 0.000 0.006

0.000 0.006 0.000 0.006 0.017 0.000 0.000 0.000 0.000 0.000

0.012 0.000 0.006 0.000 0.006 0.000 0.000 0.000 0.059 0.058

0.067 0.091

>Methylation code ID 1027

0.008 0.000 0.000 0.008 0.000 0.000 0.032 0.008 0.000 0.008

0.000 0.000 0.000 0.000 0.000 0.000 0.024 0.000 0.008 0.000

0.071 0.042 0.084 0.040 0.000 0.007 0.000 0.000 0.000 0.007

0.014 0.000 0.000 0.007 0.000 0.014 0.007 0.000 0.000 0.000

0.028 0.000 0.000 0.007 0.064 0.046 0.067 0.065 0.000 0.000

0.000 0.000 0.000 0.008 0.032 0.008 0.000 0.000 0.008 0.000

0.000 0.000 0.008 0.008 0.024 0.000 0.000 0.000 0.079 0.046

0.077 0.037

>Methylation code ID 1027

0.019 0.000 0.000 0.000 0.000 0.000 0.000 0.000 0.000 0.046

0.000 0.000 0.037 0.000 0.000 0.019 0.000 0.000 0.000 0.000

0.055 0.055 0.035 0.068 0.000 0.007 0.000 0.000 0.000 0.000

0.000 0.000 0.000 0.007 0.000 0.000 0.036 0.007 0.007 0.029

0.000 0.000 0.000 0.000 0.062 0.062 0.043 0.072 0.013 0.000

0.000 0.000 0.000 0.000 0.000 0.000 0.000 0.006 0.000 0.000

0.039 0.000 0.006 0.013 0.000 0.000 0.000 0.000 0.062 0.059

0.051 0.083

>Methylation code ID 1027

0.006 0.000 0.000 0.000 0.000 0.000 0.000 0.000 0.000 0.006

0.000 0.000 0.043 0.000 0.006 0.012 0.000 0.000 0.006 0.000

0.068 0.070 0.054 0.062 0.018 0.000 0.000 0.000 0.000 0.009

0.000 0.000 0.000 0.037 0.000 0.000 0.046 0.000 0.000 0.009

0.000 0.000 0.000 0.000 0.052 0.062 0.030 0.069 0.007 0.000

0.000 0.000 0.000 0.000 0.000 0.000 0.000 0.014 0.000 0.000

0.020 0.007 0.007 0.027 0.000 0.000 0.000 0.000 0.062 0.064

0.049 0.077

>Methylation code ID 1027

0.020 0.005 0.000 0.005 0.000 0.005 0.000 0.000 0.000 0.010

0.000 0.000 0.005 0.005 0.005 0.000 0.000 0.000 0.005 0.000

0.072 0.070 0.063 0.065 0.009 0.000 0.000 0.000 0.000 0.004

0.000 0.000 0.004 0.004 0.000 0.000 0.009 0.000 0.013 0.000

0.000 0.000 0.009 0.000 0.066 0.086 0.045 0.083 0.008 0.000

0.000 0.000 0.000 0.032 0.000 0.000 0.000 0.016 0.000 0.000

0.024 0.000 0.000 0.008 0.000 0.000 0.008 0.000 0.052 0.064

0.058 0.062

>Methylation code ID 1027

0.009 0.000 0.000 0.000 0.000 0.004 0.004 0.000 0.004 0.000

0.000 0.000 0.009 0.000 0.009 0.000 0.000 0.000 0.013 0.000

0.064 0.083 0.061 0.074 0.016 0.000 0.000 0.000 0.000 0.031

0.000 0.000 0.000 0.008 0.000 0.000 0.023 0.000 0.000 0.008

0.008 0.000 0.008 0.000 0.049 0.061 0.061 0.061 0.017 0.006

0.000 0.006 0.000 0.006 0.000 0.000 0.000 0.017 0.000 0.000

0.012 0.000 0.006 0.000 0.000 0.000 0.000 0.000 0.066 0.069

0.062 0.067

>Methylation code ID 1027

0.016 0.000 0.000 0.000 0.000 0.024 0.000 0.000 0.000 0.008

0.000 0.008 0.024 0.000 0.000 0.000 0.016 0.000 0.008 0.000

0.059 0.055 0.047 0.069 0.011 0.005 0.000 0.005 0.000 0.005

0.000 0.000 0.000 0.022 0.000 0.000 0.011 0.000 0.005 0.000

0.005 0.000 0.000 0.000 0.061 0.069 0.061 0.072 0.010 0.000

0.000 0.000 0.000 0.000 0.010 0.000 0.005 0.000 0.000 0.000

0.005 0.000 0.010 0.000 0.000 0.000 0.014 0.000 0.069 0.085

0.058 0.068

>Methylation code ID 1027

0.024 0.000 0.000 0.000 0.000 0.024 0.000 0.000 0.000 0.000

0.000 0.008 0.024 0.000 0.000 0.000 0.016 0.000 0.008 0.000

0.059 0.059 0.045 0.066 0.012 0.000 0.000 0.006 0.000 0.006

0.006 0.000 0.000 0.023 0.000 0.000 0.012 0.000 0.006 0.000

0.006 0.000 0.000 0.000 0.063 0.068 0.066 0.060 0.008 0.004

0.000 0.000 0.000 0.000 0.008 0.000 0.004 0.000 0.000 0.000

0.004 0.000 0.008 0.000 0.000 0.000 0.013 0.000 0.066 0.087

0.062 0.066

>Methylation code ID 1027

0.006 0.012 0.000 0.000 0.000 0.000 0.017 0.000 0.006 0.000

0.006 0.000 0.017 0.000 0.000 0.000 0.000 0.006 0.006 0.000

0.064 0.068 0.063 0.063 0.011 0.006 0.000 0.000 0.000 0.006

0.006 0.000 0.000 0.000 0.000 0.006 0.011 0.000 0.006 0.006

0.017 0.000 0.000 0.000 0.071 0.065 0.055 0.070 0.010 0.000

0.000 0.005 0.000 0.000 0.005 0.000 0.000 0.010 0.005 0.000

0.010 0.000 0.010 0.000 0.005 0.000 0.000 0.000 0.077 0.051

0.068 0.078

>Methylation code ID 1027

0.009 0.000 0.000 0.000 0.000 0.005 0.005 0.000 0.000 0.009

0.005 0.000 0.018 0.000 0.009 0.000 0.000 0.000 0.000 0.000

0.083 0.052 0.078 0.061 0.008 0.023 0.000 0.000 0.000 0.008

0.008 0.000 0.000 0.000 0.008 0.000 0.038 0.000 0.000 0.000

0.000 0.008 0.000 0.000 0.062 0.060 0.055 0.057 0.021 0.005

0.000 0.000 0.000 0.000 0.005 0.000 0.000 0.000 0.000 0.000

0.011 0.000 0.011 0.005 0.005 0.000 0.000 0.000 0.085 0.059

0.074 0.052

>Methylation code ID 1027

0.016 0.016 0.000 0.000 0.000 0.008 0.008 0.000 0.000 0.000

0.008 0.000 0.040 0.000 0.000 0.000 0.000 0.008 0.000 0.000

0.057 0.051 0.052 0.070 0.015 0.005 0.000 0.000 0.000 0.000

0.005 0.000 0.000 0.005 0.000 0.000 0.015 0.000 0.010 0.005

0.005 0.000 0.000 0.000 0.079 0.067 0.084 0.039 0.009 0.000

0.000 0.000 0.000 0.005 0.000 0.000 0.000 0.005 0.005 0.000

0.018 0.000 0.014 0.000 0.000 0.000 0.000 0.000 0.081 0.057

0.072 0.069

>Methylation code ID 1027

0.009 0.000 0.000 0.000 0.000 0.004 0.000 0.000 0.000 0.009

0.004 0.000 0.018 0.000 0.013 0.000 0.000 0.000 0.000 0.000

0.081 0.072 0.058 0.066 0.018 0.018 0.000 0.009 0.000 0.009

0.000 0.000 0.000 0.000 0.000 0.000 0.046 0.009 0.000 0.000

0.000 0.000 0.000 0.000 0.055 0.054 0.047 0.067 0.015 0.000

0.000 0.000 0.000 0.000 0.005 0.000 0.005 0.005 0.000 0.000

0.015 0.000 0.010 0.005 0.000 0.000 0.000 0.000 0.079 0.073

0.081 0.039

>Methylation code ID 1027

0.010 0.005 0.005 0.000 0.000 0.005 0.005 0.000 0.005 0.005

0.000 0.000 0.005 0.000 0.005 0.010 0.000 0.005 0.000 0.000

0.072 0.055 0.078 0.061 0.010 0.000 0.000 0.000 0.000 0.010

0.000 0.000 0.000 0.005 0.005 0.000 0.005 0.000 0.015 0.005

0.005 0.000 0.000 0.000 0.083 0.076 0.044 0.070 0.010 0.005

0.000 0.005 0.000 0.005 0.000 0.000 0.000 0.000 0.000 0.000

0.005 0.005 0.010 0.000 0.000 0.000 0.005 0.000 0.075 0.066

0.058 0.085

>Methylation code ID 1027

0.007 0.007 0.007 0.000 0.000 0.007 0.007 0.000 0.007 0.020

0.000 0.000 0.000 0.000 0.000 0.013 0.000 0.007 0.000 0.000

0.066 0.055 0.077 0.055 0.010 0.005 0.005 0.000 0.000 0.005

0.000 0.000 0.000 0.005 0.005 0.000 0.000 0.000 0.010 0.005

0.005 0.000 0.000 0.005 0.073 0.075 0.062 0.063 0.005 0.005

0.000 0.005 0.000 0.000 0.000 0.000 0.000 0.000 0.000 0.000

0.005 0.005 0.011 0.000 0.005 0.000 0.005 0.000 0.076 0.085

0.050 0.075

>Methylation code ID 1027

0.005 0.005 0.000 0.005 0.000 0.000 0.000 0.000 0.000 0.000

0.000 0.000 0.005 0.005 0.010 0.000 0.010 0.000 0.005 0.000

0.070 0.082 0.053 0.078 0.007 0.007 0.007 0.000 0.000 0.007

0.007 0.000 0.007 0.014 0.000 0.000 0.000 0.007 0.000 0.014

0.000 0.007 0.000 0.000 0.068 0.048 0.080 0.056 0.010 0.005

0.005 0.000 0.000 0.005 0.000 0.000 0.000 0.000 0.005 0.000

0.000 0.000 0.010 0.005 0.005 0.000 0.000 0.005 0.077 0.065

0.066 0.068

>Methylation code ID 1027

0.016 0.000 0.000 0.016 0.008 0.008 0.000 0.000 0.000 0.016

0.000 0.000 0.000 0.008 0.000 0.000 0.008 0.000 0.008 0.000

0.059 0.043 0.076 0.065 0.000 0.000 0.005 0.000 0.005 0.010

0.005 0.000 0.010 0.000 0.000 0.000 0.010 0.000 0.000 0.005

0.000 0.000 0.010 0.000 0.075 0.051 0.072 0.078 0.000 0.000

0.007 0.000 0.000 0.015 0.000 0.000 0.000 0.000 0.007 0.000

0.007 0.000 0.007 0.022 0.007 0.007 0.000 0.000 0.057 0.062

0.055 0.079

>Methylation code ID 1066

0.012 0.000 0.006 0.000 0.012 0.006 0.012 0.000 0.000 0.006

0.000 0.000 0.006 0.012 0.000 0.000 0.000 0.006 0.000 0.000

0.056 0.067 0.071 0.060 0.008 0.000 0.000 0.000 0.000 0.000

0.000 0.000 0.000 0.008 0.008 0.008 0.008 0.000 0.008 0.038

0.015 0.000 0.000 0.000 0.063 0.062 0.064 0.045 0.004 0.004

0.000 0.000 0.000 0.004 0.000 0.000 0.000 0.008 0.000 0.000

0.013 0.000 0.013 0.000 0.004 0.000 0.000 0.000 0.089 0.072

0.040 0.083

>Methylation code ID 1066

0.006 0.000 0.006 0.006 0.006 0.013 0.006 0.000 0.000 0.006

0.000 0.000 0.006 0.013 0.000 0.000 0.006 0.006 0.000 0.000

0.061 0.068 0.063 0.059 0.007 0.000 0.000 0.000 0.000 0.000

0.000 0.000 0.007 0.000 0.007 0.007 0.015 0.000 0.007 0.029

0.007 0.000 0.000 0.000 0.061 0.050 0.070 0.064 0.005 0.000

0.000 0.000 0.000 0.005 0.000 0.000 0.000 0.005 0.000 0.005

0.015 0.000 0.010 0.000 0.005 0.000 0.005 0.000 0.082 0.078

0.048 0.069

>Methylation code ID 1066

0.000 0.000 0.000 0.000 0.000 0.000 0.000 0.000 0.006 0.000

0.000 0.012 0.012 0.000 0.006 0.006 0.018 0.006 0.006 0.000

0.064 0.073 0.073 0.051 0.000 0.000 0.000 0.007 0.000 0.014

0.007 0.007 0.007 0.014 0.000 0.000 0.007 0.014 0.000 0.007

0.007 0.000 0.000 0.000 0.075 0.063 0.041 0.066 0.006 0.000

0.000 0.000 0.000 0.000 0.000 0.000 0.013 0.006 0.006 0.006

0.006 0.000 0.006 0.000 0.000 0.000 0.000 0.013 0.086 0.047

0.073 0.064

>Methylation code ID 1066

0.007 0.000 0.000 0.000 0.000 0.000 0.007 0.020 0.007 0.000

0.007 0.000 0.020 0.007 0.000 0.007 0.000 0.000 0.007 0.000

0.056 0.066 0.060 0.063 0.005 0.005 0.000 0.000 0.005 0.005

0.011 0.000 0.005 0.011 0.000 0.000 0.000 0.000 0.005 0.005

0.005 0.000 0.000 0.000 0.077 0.082 0.042 0.068 0.006 0.000

0.006 0.000 0.000 0.000 0.000 0.000 0.000 0.000 0.000 0.013

0.006 0.006 0.006 0.006 0.013 0.000 0.000 0.006 0.068 0.064

0.065 0.067

>Methylation code ID 1066

0.000 0.000 0.000 0.000 0.006 0.006 0.012 0.000 0.006 0.025

0.000 0.006 0.000 0.006 0.006 0.000 0.000 0.000 0.000 0.000

0.080 0.061 0.061 0.057 0.000 0.000 0.000 0.000 0.000 0.016

0.000 0.008 0.000 0.000 0.000 0.008 0.000 0.000 0.008 0.023

0.016 0.000 0.000 0.016 0.066 0.067 0.061 0.045 0.012 0.000

0.000 0.018 0.000 0.000 0.000 0.012 0.000 0.000 0.000 0.000

0.018 0.000 0.006 0.000 0.006 0.000 0.000 0.000 0.062 0.060

0.068 0.069

>Methylation code ID 1066

0.005 0.000 0.000 0.019 0.000 0.000 0.000 0.000 0.000 0.014

0.000 0.000 0.005 0.005 0.014 0.000 0.000 0.000 0.000 0.000

0.054 0.058 0.075 0.085 0.000 0.009 0.000 0.000 0.000 0.013

0.000 0.000 0.004 0.004 0.000 0.009 0.004 0.004 0.004 0.004

0.000 0.000 0.000 0.000 0.070 0.058 0.068 0.079 0.018 0.000

0.000 0.012 0.000 0.000 0.000 0.006 0.000 0.000 0.000 0.000

0.000 0.000 0.012 0.006 0.006 0.006 0.000 0.000 0.074 0.073

0.071 0.049

>Methylation code ID 1066

0.000 0.009 0.000 0.005 0.000 0.014 0.000 0.000 0.000 0.005

0.000 0.009 0.009 0.000 0.005 0.005 0.000 0.000 0.000 0.000

0.074 0.063 0.057 0.079 0.018 0.000 0.000 0.012 0.000 0.000

0.000 0.006 0.000 0.000 0.000 0.000 0.000 0.000 0.012 0.006

0.006 0.006 0.000 0.000 0.057 0.070 0.077 0.065 0.005 0.000

0.000 0.010 0.000 0.000 0.000 0.000 0.000 0.015 0.000 0.000

0.005 0.005 0.015 0.000 0.000 0.000 0.000 0.000 0.044 0.056

0.089 0.091

>Methylation code ID 1066

0.019 0.000 0.000 0.006 0.000 0.000 0.000 0.006 0.000 0.013

0.000 0.000 0.006 0.006 0.019 0.000 0.000 0.000 0.000 0.000

0.050 0.056 0.064 0.087 0.000 0.009 0.000 0.005 0.000 0.014

0.005 0.000 0.000 0.000 0.000 0.000 0.009 0.000 0.009 0.009

0.000 0.000 0.000 0.000 0.063 0.066 0.068 0.075 0.015 0.000

0.005 0.005 0.000 0.000 0.000 0.000 0.000 0.000 0.000 0.000

0.000 0.000 0.015 0.005 0.005 0.005 0.000 0.000 0.059 0.063

0.065 0.090

>Methylation code ID 1066

0.024 0.000 0.000 0.008 0.000 0.000 0.008 0.008 0.000 0.016

0.000 0.000 0.008 0.008 0.000 0.016 0.000 0.000 0.000 0.000

0.059 0.061 0.048 0.068 0.004 0.009 0.000 0.004 0.000 0.000

0.004 0.000 0.000 0.000 0.000 0.000 0.013 0.000 0.009 0.009

0.004 0.000 0.000 0.000 0.065 0.059 0.071 0.082 0.016 0.000

0.005 0.000 0.000 0.000 0.000 0.000 0.000 0.005 0.000 0.000

0.000 0.005 0.016 0.005 0.005 0.000 0.000 0.000 0.055 0.063

0.065 0.093

>Methylation code ID 1066

0.015 0.000 0.005 0.000 0.000 0.000 0.000 0.000 0.000 0.005

0.000 0.000 0.005 0.005 0.015 0.005 0.005 0.000 0.000 0.000

0.057 0.063 0.064 0.090 0.025 0.000 0.000 0.008 0.000 0.000

0.008 0.008 0.000 0.016 0.000 0.000 0.008 0.008 0.000 0.016

0.000 0.000 0.000 0.000 0.058 0.063 0.058 0.056 0.005 0.005

0.000 0.005 0.000 0.000 0.005 0.000 0.000 0.000 0.000 0.000

0.014 0.000 0.009 0.009 0.005 0.000 0.000 0.000 0.061 0.055

0.075 0.088

>Methylation code ID 1066

0.013 0.000 0.006 0.000 0.000 0.000 0.000 0.006 0.013 0.000

0.000 0.006 0.000 0.000 0.000 0.000 0.006 0.006 0.006 0.006

0.054 0.056 0.077 0.077 0.000 0.000 0.000 0.006 0.000 0.006

0.000 0.006 0.013 0.000 0.000 0.006 0.006 0.006 0.006 0.006

0.013 0.000 0.000 0.000 0.064 0.062 0.066 0.066 0.000 0.000

0.000 0.000 0.000 0.007 0.013 0.007 0.013 0.007 0.000 0.000

0.000 0.000 0.013 0.007 0.000 0.000 0.000 0.000 0.100 0.035

0.104 0.029

>Methylation code ID 1116

0.007 0.007 0.000 0.000 0.014 0.000 0.000 0.000 0.000 0.014

0.000 0.000 0.014 0.007 0.000 0.007 0.000 0.000 0.000 0.007

0.060 0.076 0.070 0.053 0.009 0.005 0.009 0.000 0.005 0.000

0.000 0.000 0.000 0.000 0.000 0.005 0.009 0.000 0.005 0.009

0.005 0.000 0.000 0.000 0.069 0.057 0.071 0.078 0.007 0.000

0.000 0.000 0.000 0.000 0.000 0.007 0.000 0.022 0.015 0.000

0.007 0.007 0.007 0.007 0.000 0.007 0.000 0.000 0.063 0.053

0.052 0.077

>Methylation code ID 1116

0.005 0.005 0.000 0.000 0.005 0.010 0.000 0.005 0.000 0.000

0.000 0.005 0.005 0.000 0.005 0.010 0.005 0.005 0.000 0.000

0.067 0.071 0.066 0.062 0.000 0.000 0.000 0.022 0.007 0.000

0.000 0.007 0.000 0.014 0.000 0.000 0.007 0.014 0.000 0.014

0.000 0.007 0.000 0.000 0.059 0.065 0.054 0.061 0.000 0.000

0.000 0.000 0.016 0.000 0.000 0.000 0.005 0.005 0.000 0.010

0.000 0.000 0.010 0.010 0.000 0.000 0.005 0.000 0.083 0.070

0.059 0.058

>Methylation code ID 1116

0.015 0.000 0.005 0.005 0.000 0.010 0.000 0.000 0.000 0.005

0.000 0.005 0.000 0.000 0.005 0.005 0.000 0.000 0.010 0.000

0.058 0.074 0.071 0.068 0.013 0.000 0.000 0.000 0.000 0.025

0.000 0.006 0.000 0.006 0.000 0.000 0.006 0.000 0.006 0.006

0.006 0.000 0.006 0.000 0.077 0.064 0.061 0.049 0.007 0.000

0.000 0.007 0.000 0.020 0.000 0.000 0.000 0.007 0.000 0.000

0.000 0.013 0.013 0.000 0.000 0.000 0.000 0.000 0.038 0.079

0.075 0.076

>Methylation code ID 1116

0.015 0.000 0.000 0.005 0.000 0.010 0.005 0.000 0.000 0.005

0.000 0.005 0.000 0.000 0.005 0.005 0.000 0.000 0.010 0.000

0.054 0.075 0.073 0.066 0.012 0.000 0.000 0.000 0.000 0.025

0.000 0.000 0.000 0.006 0.000 0.000 0.006 0.000 0.006 0.006

0.012 0.000 0.006 0.000 0.073 0.061 0.061 0.058 0.012 0.000

0.000 0.006 0.000 0.018 0.000 0.000 0.000 0.000 0.000 0.000

0.000 0.012 0.012 0.000 0.000 0.000 0.000 0.000 0.034 0.070

0.086 0.084

>Methylation code ID 1116

0.006 0.000 0.000 0.006 0.000 0.017 0.000 0.000 0.000 0.000

0.000 0.000 0.006 0.017 0.011 0.000 0.000 0.000 0.000 0.000

0.041 0.067 0.080 0.083 0.015 0.000 0.000 0.005 0.000 0.010

0.005 0.000 0.000 0.000 0.000 0.005 0.000 0.000 0.010 0.010

0.000 0.000 0.005 0.000 0.062 0.062 0.064 0.080 0.022 0.000

0.000 0.000 0.000 0.022 0.000 0.000 0.000 0.007 0.000 0.000

0.007 0.000 0.007 0.007 0.015 0.000 0.000 0.000 0.065 0.060

0.067 0.053

>Methylation code ID 1116

0.017 0.000 0.000 0.000 0.000 0.017 0.000 0.000 0.000 0.026

0.000 0.000 0.009 0.000 0.009 0.026 0.009 0.000 0.000 0.000

0.076 0.050 0.050 0.046 0.006 0.006 0.000 0.000 0.000 0.012

0.006 0.000 0.000 0.012 0.000 0.000 0.006 0.006 0.006 0.006

0.000 0.000 0.000 0.000 0.064 0.071 0.063 0.068 0.020 0.000

0.000 0.005 0.010 0.000 0.005 0.000 0.000 0.005 0.000 0.000

0.000 0.000 0.005 0.005 0.000 0.000 0.005 0.000 0.067 0.058

0.057 0.091

>Methylation code ID 1116

0.015 0.000 0.000 0.005 0.010 0.005 0.005 0.000 0.000 0.005

0.000 0.000 0.000 0.000 0.005 0.005 0.005 0.000 0.005 0.000

0.063 0.062 0.059 0.083 0.021 0.000 0.000 0.010 0.000 0.010

0.000 0.000 0.000 0.031 0.000 0.000 0.010 0.010 0.000 0.031

0.010 0.000 0.000 0.000 0.053 0.049 0.051 0.046 0.005 0.005

0.000 0.000 0.000 0.005 0.005 0.000 0.000 0.011 0.000 0.000

0.005 0.005 0.011 0.005 0.000 0.000 0.000 0.000 0.062 0.082

0.066 0.065

>Methylation code ID 1116

0.000 0.000 0.000 0.007 0.014 0.007 0.000 0.000 0.000 0.014

0.000 0.000 0.000 0.007 0.007 0.021 0.007 0.000 0.000 0.000

0.069 0.078 0.054 0.049 0.019 0.000 0.000 0.009 0.000 0.009

0.009 0.000 0.009 0.000 0.000 0.000 0.000 0.009 0.000 0.047

0.000 0.000 0.000 0.000 0.048 0.060 0.060 0.053 0.010 0.000

0.000 0.005 0.000 0.000 0.000 0.005 0.005 0.010 0.000 0.000

0.010 0.000 0.010 0.000 0.000 0.005 0.000 0.000 0.057 0.068

0.061 0.084

>Methylation code ID 1116

0.000 0.000 0.000 0.000 0.015 0.000 0.000 0.000 0.010 0.005

0.000 0.000 0.005 0.005 0.010 0.010 0.000 0.005 0.000 0.000

0.062 0.071 0.054 0.082 0.000 0.005 0.000 0.000 0.005 0.009

0.000 0.000 0.000 0.009 0.000 0.000 0.005 0.000 0.014 0.014

0.000 0.000 0.000 0.000 0.088 0.043 0.080 0.061 0.020 0.000

0.000 0.013 0.013 0.000 0.000 0.000 0.000 0.007 0.000 0.000

0.000 0.000 0.007 0.000 0.000 0.013 0.000 0.000 0.065 0.057

0.071 0.067

>Methylation code ID 1116

0.017 0.000 0.008 0.000 0.000 0.000 0.008 0.000 0.000 0.008

0.000 0.000 0.000 0.008 0.000 0.025 0.008 0.008 0.008 0.000

0.063 0.057 0.062 0.050 0.012 0.000 0.000 0.000 0.000 0.006

0.006 0.000 0.006 0.000 0.006 0.000 0.018 0.000 0.000 0.000

0.006 0.000 0.012 0.000 0.068 0.048 0.074 0.070 0.000 0.000

0.000 0.006 0.000 0.011 0.000 0.000 0.000 0.006 0.000 0.006

0.011 0.006 0.011 0.006 0.006 0.000 0.000 0.000 0.060 0.066

0.061 0.079

>Methylation code ID 1116

0.019 0.000 0.000 0.000 0.000 0.009 0.000 0.000 0.000 0.000

0.000 0.000 0.009 0.000 0.014 0.000 0.005 0.000 0.000 0.000

0.084 0.055 0.069 0.069 0.008 0.004 0.004 0.004 0.000 0.004

0.000 0.000 0.000 0.008 0.000 0.000 0.008 0.004 0.008 0.000

0.000 0.000 0.000 0.000 0.078 0.071 0.044 0.085 0.011 0.005

0.000 0.000 0.000 0.011 0.005 0.000 0.000 0.000 0.000 0.000

0.005 0.000 0.011 0.011 0.005 0.000 0.000 0.000 0.065 0.071

0.053 0.082

>Methylation code ID 1116

0.010 0.005 0.000 0.000 0.000 0.010 0.005 0.000 0.000 0.000

0.000 0.000 0.005 0.000 0.010 0.015 0.005 0.000 0.000 0.000

0.070 0.067 0.052 0.078 0.019 0.000 0.000 0.000 0.000 0.010

0.000 0.000 0.000 0.005 0.000 0.000 0.005 0.000 0.014 0.000

0.005 0.000 0.000 0.000 0.082 0.057 0.057 0.081 0.009 0.005

0.005 0.005 0.000 0.005 0.000 0.000 0.000 0.009 0.000 0.000

0.005 0.005 0.009 0.000 0.000 0.000 0.000 0.000 0.080 0.065

0.046 0.088

>Methylation code ID 1116

0.009 0.004 0.004 0.004 0.000 0.004 0.000 0.000 0.000 0.009

0.000 0.000 0.004 0.004 0.009 0.004 0.000 0.000 0.000 0.000

0.075 0.066 0.052 0.081 0.011 0.006 0.000 0.000 0.000 0.011

0.006 0.000 0.000 0.006 0.000 0.000 0.006 0.000 0.006 0.017

0.006 0.000 0.000 0.000 0.070 0.062 0.060 0.069 0.017 0.000

0.000 0.000 0.000 0.006 0.000 0.000 0.000 0.006 0.000 0.000

0.006 0.000 0.017 0.000 0.006 0.000 0.000 0.000 0.081 0.050

0.067 0.080

>Methylation code ID 1116

0.013 0.000 0.000 0.019 0.000 0.019 0.000 0.000 0.000 0.013

0.000 0.000 0.006 0.000 0.000 0.006 0.000 0.000 0.006 0.000

0.047 0.070 0.075 0.059 0.005 0.005 0.000 0.000 0.000 0.010

0.005 0.000 0.005 0.005 0.000 0.000 0.005 0.000 0.010 0.010

0.000 0.000 0.000 0.000 0.063 0.072 0.091 0.046 0.010 0.000

0.000 0.000 0.000 0.016 0.000 0.000 0.000 0.005 0.000 0.000

0.000 0.000 0.010 0.005 0.000 0.000 0.005 0.000 0.071 0.056

0.086 0.068

>Methylation code ID 1116

0.013 0.000 0.000 0.007 0.000 0.013 0.000 0.000 0.000 0.000

0.000 0.007 0.000 0.000 0.013 0.013 0.020 0.000 0.000 0.000

0.069 0.051 0.050 0.076 0.004 0.000 0.000 0.004 0.000 0.009

0.000 0.004 0.000 0.009 0.000 0.000 0.009 0.000 0.017 0.000

0.000 0.000 0.000 0.000 0.071 0.072 0.063 0.070 0.000 0.000

0.004 0.009 0.000 0.009 0.004 0.000 0.000 0.000 0.000 0.004

0.000 0.000 0.009 0.004 0.000 0.004 0.004 0.000 0.073 0.069

0.075 0.065

>Methylation code ID 1116

0.000 0.000 0.000 0.011 0.000 0.022 0.000 0.000 0.000 0.000

0.000 0.006 0.006 0.006 0.011 0.006 0.006 0.000 0.000 0.000

0.064 0.059 0.053 0.084 0.019 0.000 0.000 0.000 0.000 0.019

0.000 0.006 0.006 0.006 0.000 0.006 0.000 0.006 0.006 0.000

0.000 0.006 0.000 0.000 0.069 0.068 0.051 0.061 0.005 0.000

0.000 0.009 0.000 0.005 0.005 0.000 0.005 0.000 0.000 0.000

0.000 0.000 0.014 0.009 0.000 0.000 0.005 0.000 0.069 0.054

0.076 0.079

>Methylation code ID 1128

0.004 0.007 0.000 0.000 0.000 0.014 0.000 0.000 0.000 0.000

0.000 0.000 0.004 0.000 0.011 0.004 0.000 0.000 0.004 0.000

0.075 0.062 0.070 0.079 0.006 0.000 0.012 0.006 0.000 0.025

0.000 0.000 0.000 0.006 0.000 0.000 0.000 0.006 0.006 0.000

0.000 0.006 0.000 0.000 0.080 0.062 0.073 0.044 0.010 0.000

0.000 0.005 0.000 0.005 0.000 0.005 0.000 0.005 0.000 0.000

0.000 0.000 0.010 0.000 0.005 0.000 0.010 0.000 0.052 0.066

0.086 0.075

>Methylation code ID 1128

0.006 0.000 0.012 0.006 0.000 0.029 0.000 0.000 0.000 0.006

0.000 0.000 0.000 0.006 0.006 0.000 0.000 0.000 0.000 0.000

0.080 0.054 0.068 0.060 0.008 0.000 0.000 0.004 0.000 0.008

0.000 0.004 0.000 0.000 0.000 0.000 0.000 0.000 0.012 0.000

0.004 0.000 0.008 0.000 0.050 0.079 0.081 0.073 0.004 0.004

0.000 0.000 0.000 0.021 0.000 0.000 0.000 0.000 0.000 0.000

0.004 0.000 0.013 0.004 0.000 0.000 0.000 0.000 0.068 0.055

0.081 0.078

>Methylation code ID 1128

0.008 0.000 0.000 0.000 0.000 0.008 0.000 0.004 0.000 0.004

0.000 0.000 0.000 0.000 0.013 0.000 0.004 0.000 0.008 0.000

0.047 0.084 0.087 0.065 0.005 0.005 0.000 0.000 0.000 0.028

0.000 0.000 0.000 0.000 0.000 0.000 0.005 0.000 0.009 0.005

0.000 0.000 0.000 0.000 0.068 0.048 0.080 0.081 0.006 0.000

0.006 0.006 0.000 0.025 0.000 0.000 0.000 0.006 0.000 0.000

0.000 0.006 0.006 0.000 0.000 0.006 0.000 0.000 0.070 0.055

0.070 0.070

>Methylation code ID 1128

0.006 0.000 0.006 0.006 0.000 0.023 0.000 0.000 0.006 0.006

0.000 0.000 0.000 0.006 0.006 0.000 0.000 0.006 0.000 0.000

0.073 0.058 0.070 0.065 0.008 0.000 0.000 0.000 0.000 0.008

0.000 0.000 0.000 0.004 0.000 0.000 0.000 0.000 0.017 0.000

0.004 0.000 0.008 0.000 0.055 0.093 0.072 0.062 0.005 0.005

0.000 0.000 0.000 0.029 0.000 0.000 0.000 0.000 0.000 0.000

0.005 0.000 0.010 0.000 0.000 0.000 0.000 0.000 0.070 0.049

0.076 0.086

>Methylation code ID 1128

0.023 0.000 0.000 0.000 0.000 0.093 0.000 0.000 0.000 0.000

0.000 0.000 0.012 0.000 0.012 0.000 0.000 0.000 0.000 0.000

0.040 0.046 0.051 0.057 0.025 0.000 0.000 0.006 0.000 0.019

0.000 0.000 0.006 0.000 0.000 0.000 0.000 0.013 0.006 0.000

0.000 0.006 0.000 0.000 0.065 0.060 0.058 0.067 0.005 0.000

0.000 0.000 0.000 0.010 0.000 0.000 0.000 0.005 0.000 0.000

0.000 0.000 0.042 0.000 0.000 0.000 0.000 0.000 0.057 0.090

0.067 0.058

>Methylation code ID 1128

0.077 0.000 0.000 0.000 0.000 0.231 0.000 0.000 0.000 0.000

0.000 0.000 0.000 0.000 0.000 0.000 0.000 0.000 0.000 0.000

0.007 0.004 0.007 0.007 0.023 0.000 0.000 0.006 0.000 0.017

0.000 0.000 0.006 0.000 0.000 0.000 0.000 0.006 0.011 0.000

0.000 0.006 0.000 0.000 0.080 0.042 0.065 0.073 0.000 0.000

0.000 0.000 0.000 0.019 0.000 0.000 0.000 0.005 0.000 0.000

0.000 0.000 0.034 0.000 0.000 0.000 0.000 0.000 0.040 0.070

0.078 0.088

>Methylation code ID 1128

0.077 0.000 0.000 0.000 0.000 0.230 0.000 0.000 0.000 0.000

0.000 0.000 0.000 0.000 0.000 0.000 0.000 0.000 0.000 0.000

0.008 0.004 0.008 0.006 0.028 0.000 0.000 0.000 0.000 0.017

0.000 0.000 0.006 0.000 0.000 0.000 0.000 0.006 0.011 0.000

0.000 0.006 0.000 0.000 0.087 0.035 0.074 0.065 0.000 0.000

0.000 0.000 0.000 0.025 0.000 0.000 0.000 0.005 0.000 0.000

0.000 0.000 0.030 0.000 0.000 0.000 0.000 0.000 0.028 0.058

0.080 0.108

>Methylation code ID 1128

0.069 0.000 0.000 0.000 0.000 0.184 0.000 0.000 0.000 0.023

0.000 0.000 0.000 0.000 0.000 0.000 0.000 0.000 0.000 0.000

0.017 0.010 0.017 0.013 0.025 0.000 0.000 0.000 0.000 0.010

0.000 0.000 0.005 0.000 0.000 0.000 0.000 0.005 0.010 0.000

0.000 0.005 0.005 0.000 0.084 0.038 0.077 0.070 0.000 0.000

0.000 0.000 0.000 0.022 0.000 0.000 0.000 0.006 0.000 0.000

0.006 0.000 0.033 0.000 0.000 0.000 0.000 0.000 0.033 0.052

0.076 0.106

>Methylation code ID 1128

0.000 0.000 0.000 0.000 0.000 0.040 0.000 0.000 0.000 0.000

0.000 0.000 0.007 0.000 0.040 0.000 0.000 0.000 0.000 0.000

0.028 0.049 0.069 0.100 0.082 0.000 0.000 0.000 0.000 0.163

0.000 0.000 0.000 0.020 0.000 0.000 0.000 0.000 0.000 0.000

0.000 0.000 0.000 0.000 0.021 0.018 0.015 0.014 0.025 0.000

0.000 0.000 0.000 0.010 0.000 0.000 0.000 0.000 0.000 0.000

0.000 0.005 0.015 0.000 0.000 0.000 0.005 0.000 0.081 0.049

0.065 0.079

>Methylation code ID 1128

0.000 0.000 0.000 0.000 0.000 0.030 0.000 0.000 0.000 0.000

0.000 0.000 0.010 0.000 0.025 0.000 0.000 0.000 0.000 0.000

0.047 0.056 0.072 0.093 0.076 0.000 0.000 0.000 0.000 0.134

0.000 0.000 0.000 0.038 0.000 0.000 0.000 0.000 0.000 0.000

0.000 0.000 0.000 0.000 0.023 0.024 0.024 0.013 0.022 0.000

0.000 0.000 0.000 0.009 0.000 0.000 0.000 0.000 0.000 0.000

0.004 0.000 0.013 0.000 0.000 0.000 0.004 0.000 0.078 0.058

0.062 0.083

>Methylation code ID 1128

0.005 0.000 0.000 0.000 0.000 0.030 0.000 0.000 0.000 0.005

0.000 0.000 0.010 0.000 0.015 0.000 0.000 0.000 0.000 0.000

0.076 0.070 0.045 0.077 0.058 0.000 0.000 0.000 0.000 0.073

0.000 0.000 0.000 0.029 0.000 0.000 0.015 0.000 0.000 0.015

0.000 0.000 0.000 0.000 0.039 0.043 0.037 0.025 0.009 0.000

0.000 0.000 0.000 0.013 0.000 0.000 0.000 0.000 0.000 0.000

0.009 0.000 0.013 0.004 0.000 0.000 0.004 0.000 0.090 0.049

0.065 0.076

>Methylation code ID 1128

0.049 0.000 0.000 0.000 0.012 0.049 0.000 0.000 0.000 0.024

0.000 0.000 0.012 0.000 0.000 0.012 0.000 0.000 0.000 0.000

0.054 0.051 0.045 0.025 0.009 0.000 0.000 0.000 0.000 0.009

0.000 0.000 0.000 0.004 0.000 0.000 0.013 0.000 0.013 0.004

0.000 0.000 0.004 0.000 0.087 0.051 0.060 0.077 0.006 0.000

0.000 0.000 0.000 0.029 0.000 0.000 0.000 0.011 0.000 0.000

0.011 0.000 0.011 0.000 0.000 0.000 0.000 0.000 0.075 0.082

0.035 0.072

>Methylation code ID 1128

0.015 0.000 0.000 0.000 0.008 0.023 0.000 0.000 0.000 0.015

0.000 0.008 0.008 0.000 0.008 0.008 0.000 0.008 0.000 0.000

0.049 0.069 0.065 0.052 0.012 0.000 0.000 0.000 0.000 0.012

0.000 0.000 0.000 0.012 0.000 0.000 0.018 0.000 0.006 0.006

0.006 0.000 0.006 0.000 0.075 0.062 0.061 0.056 0.005 0.005

0.000 0.000 0.000 0.010 0.000 0.000 0.000 0.010 0.000 0.000

0.010 0.005 0.010 0.000 0.000 0.000 0.000 0.000 0.078 0.079

0.055 0.066

>Methylation code ID 1128

0.007 0.000 0.000 0.000 0.000 0.027 0.000 0.000 0.000 0.013

0.000 0.000 0.020 0.000 0.007 0.007 0.007 0.000 0.000 0.000

0.057 0.070 0.071 0.049 0.015 0.005 0.000 0.005 0.000 0.005

0.000 0.000 0.000 0.005 0.000 0.000 0.005 0.010 0.010 0.000

0.000 0.000 0.000 0.000 0.076 0.063 0.073 0.062 0.004 0.000

0.000 0.000 0.004 0.004 0.000 0.000 0.000 0.000 0.000 0.004

0.008 0.000 0.016 0.000 0.000 0.008 0.000 0.000 0.046 0.075

0.092 0.073

>Methylation code ID 1128

0.004 0.000 0.000 0.000 0.004 0.004 0.000 0.000 0.000 0.000

0.000 0.004 0.007 0.000 0.019 0.000 0.000 0.007 0.000 0.000

0.042 0.069 0.095 0.078 0.006 0.000 0.006 0.000 0.000 0.025

0.000 0.000 0.000 0.012 0.000 0.000 0.012 0.000 0.006 0.006

0.006 0.000 0.000 0.000 0.056 0.074 0.067 0.055 0.018 0.006

0.000 0.006 0.000 0.006 0.000 0.000 0.000 0.006 0.000 0.000

0.006 0.012 0.006 0.000 0.000 0.000 0.000 0.000 0.076 0.060

0.063 0.069

>Methylation code ID 1128

0.010 0.005 0.000 0.005 0.000 0.005 0.000 0.000 0.000 0.005

0.000 0.000 0.005 0.010 0.010 0.000 0.005 0.000 0.000 0.000

0.078 0.057 0.067 0.069 0.004 0.000 0.000 0.000 0.004 0.004

0.000 0.000 0.000 0.000 0.000 0.004 0.004 0.000 0.021 0.000

0.000 0.007 0.000 0.000 0.040 0.069 0.095 0.082 0.013 0.000

0.007 0.000 0.000 0.020 0.000 0.000 0.000 0.013 0.000 0.000

0.007 0.000 0.007 0.007 0.007 0.000 0.000 0.000 0.060 0.072

0.064 0.056

>Methylation code ID 1128

0.032 0.000 0.006 0.000 0.000 0.019 0.000 0.000 0.000 0.000

0.000 0.000 0.006 0.000 0.006 0.006 0.006 0.000 0.000 0.000

0.054 0.072 0.065 0.058 0.010 0.000 0.000 0.005 0.000 0.005

0.000 0.000 0.000 0.000 0.000 0.000 0.014 0.010 0.014 0.000

0.005 0.000 0.000 0.000 0.093 0.046 0.068 0.063 0.004 0.000

0.000 0.000 0.000 0.008 0.000 0.000 0.000 0.000 0.000 0.000

0.004 0.000 0.027 0.000 0.000 0.004 0.000 0.000 0.056 0.057

0.088 0.085

>Methylation code ID 1128

0.035 0.000 0.007 0.000 0.000 0.021 0.000 0.000 0.000 0.000

0.007 0.000 0.007 0.000 0.007 0.007 0.000 0.000 0.000 0.000

0.064 0.078 0.054 0.046 0.009 0.000 0.000 0.004 0.000 0.004

0.000 0.000 0.000 0.000 0.000 0.000 0.013 0.004 0.013 0.000

0.004 0.000 0.004 0.000 0.083 0.049 0.079 0.067 0.004 0.000

0.000 0.000 0.000 0.013 0.004 0.000 0.000 0.000 0.000 0.000

0.004 0.000 0.021 0.000 0.000 0.004 0.000 0.000 0.063 0.043

0.090 0.085

>Methylation code ID 1128

0.004 0.000 0.000 0.000 0.000 0.012 0.004 0.000 0.000 0.000

0.000 0.000 0.004 0.000 0.024 0.000 0.000 0.004 0.000 0.000

0.068 0.045 0.094 0.075 0.030 0.000 0.007 0.000 0.000 0.030

0.000 0.000 0.000 0.000 0.007 0.000 0.007 0.000 0.007 0.007

0.000 0.000 0.000 0.000 0.066 0.067 0.053 0.049 0.010 0.000

0.000 0.005 0.000 0.005 0.000 0.000 0.000 0.000 0.000 0.000

0.015 0.005 0.010 0.000 0.005 0.000 0.005 0.000 0.083 0.043

0.086 0.061

>Methylation code ID 1128

0.025 0.000 0.006 0.000 0.000 0.025 0.000 0.000 0.000 0.000

0.006 0.000 0.006 0.000 0.013 0.000 0.000 0.000 0.000 0.000

0.062 0.069 0.058 0.062 0.014 0.000 0.000 0.005 0.000 0.009

0.000 0.000 0.000 0.000 0.000 0.000 0.009 0.005 0.009 0.000

0.005 0.000 0.005 0.000 0.074 0.044 0.088 0.068 0.009 0.000

0.000 0.000 0.000 0.014 0.005 0.000 0.000 0.000 0.000 0.000

0.000 0.000 0.028 0.000 0.000 0.000 0.000 0.000 0.087 0.040

0.092 0.059

>Methylation code ID 1128

0.008 0.000 0.000 0.000 0.000 0.013 0.004 0.000 0.000 0.000

0.000 0.000 0.000 0.000 0.029 0.000 0.000 0.000 0.000 0.000

0.090 0.036 0.094 0.059 0.030 0.000 0.007 0.000 0.000 0.037

0.000 0.000 0.000 0.000 0.007 0.000 0.007 0.000 0.007 0.000

0.000 0.000 0.000 0.000 0.073 0.060 0.045 0.059 0.014 0.000

0.000 0.005 0.000 0.005 0.000 0.000 0.000 0.000 0.000 0.000

0.010 0.005 0.010 0.000 0.005 0.000 0.005 0.000 0.073 0.046

0.087 0.071

>Methylation code ID 1128

0.028 0.000 0.006 0.000 0.000 0.022 0.000 0.000 0.000 0.000

0.006 0.000 0.000 0.000 0.011 0.000 0.000 0.000 0.000 0.000

0.073 0.048 0.062 0.078 0.014 0.000 0.000 0.000 0.000 0.014

0.000 0.000 0.000 0.000 0.000 0.000 0.009 0.000 0.014 0.000

0.005 0.000 0.005 0.000 0.083 0.043 0.076 0.072 0.009 0.000

0.000 0.000 0.000 0.018 0.005 0.000 0.000 0.000 0.000 0.000

0.000 0.000 0.023 0.000 0.000 0.000 0.000 0.000 0.125 0.014

0.122 0.018

>Methylation code ID 1128

0.014 0.000 0.000 0.000 0.000 0.019 0.000 0.000 0.000 0.000

0.000 0.000 0.010 0.000 0.014 0.000 0.000 0.000 0.005 0.000

0.078 0.054 0.069 0.070 0.018 0.000 0.000 0.000 0.000 0.018

0.004 0.000 0.000 0.000 0.000 0.000 0.000 0.000 0.018 0.000

0.000 0.000 0.000 0.000 0.109 0.026 0.116 0.025 0.022 0.000

0.000 0.000 0.000 0.022 0.000 0.000 0.000 0.000 0.005 0.000

0.000 0.000 0.016 0.000 0.000 0.000 0.000 0.000 0.078 0.055

0.052 0.084

>Methylation code ID 1128

0.020 0.000 0.000 0.000 0.000 0.020 0.000 0.000 0.000 0.000

0.005 0.000 0.000 0.000 0.020 0.000 0.000 0.000 0.000 0.000

0.070 0.063 0.062 0.074 0.016 0.000 0.000 0.000 0.000 0.026

0.000 0.000 0.000 0.000 0.000 0.000 0.010 0.000 0.010 0.000

0.000 0.000 0.005 0.000 0.072 0.059 0.077 0.058 0.019 0.000

0.000 0.000 0.000 0.014 0.005 0.000 0.000 0.000 0.000 0.000

0.000 0.000 0.019 0.000 0.000 0.000 0.000 0.000 0.104 0.028

0.117 0.027

>Methylation code ID 1128

0.015 0.000 0.000 0.005 0.000 0.026 0.000 0.000 0.000 0.000

0.000 0.000 0.005 0.000 0.010 0.000 0.000 0.000 0.005 0.000

0.064 0.065 0.071 0.066 0.019 0.000 0.000 0.000 0.000 0.014

0.005 0.000 0.000 0.000 0.000 0.000 0.000 0.000 0.019 0.000

0.000 0.000 0.000 0.000 0.101 0.035 0.115 0.026 0.010 0.000

0.000 0.000 0.000 0.020 0.000 0.000 0.000 0.000 0.005 0.000

0.000 0.000 0.020 0.000 0.000 0.000 0.000 0.000 0.072 0.063

0.063 0.081

>Methylation code ID 1128

0.009 0.000 0.000 0.000 0.000 0.023 0.000 0.000 0.000 0.000

0.005 0.000 0.000 0.000 0.018 0.000 0.000 0.000 0.000 0.000

0.079 0.071 0.057 0.072 0.019 0.000 0.000 0.006 0.000 0.037

0.000 0.000 0.000 0.000 0.000 0.000 0.006 0.000 0.006 0.000

0.000 0.000 0.006 0.000 0.069 0.060 0.066 0.058 0.020 0.000

0.000 0.000 0.000 0.010 0.005 0.000 0.000 0.000 0.000 0.000

0.000 0.000 0.020 0.000 0.000 0.000 0.000 0.000 0.102 0.038

0.110 0.029

>Methylation code ID 1128

0.024 0.000 0.000 0.000 0.000 0.010 0.005 0.000 0.000 0.000

0.000 0.000 0.000 0.000 0.019 0.000 0.000 0.000 0.000 0.000

0.110 0.036 0.102 0.027 0.004 0.000 0.000 0.000 0.000 0.022

0.000 0.000 0.000 0.000 0.004 0.000 0.000 0.000 0.022 0.000

0.000 0.000 0.000 0.000 0.086 0.069 0.055 0.070 0.020 0.000

0.000 0.007 0.000 0.041 0.000 0.000 0.000 0.000 0.000 0.000

0.000 0.000 0.007 0.000 0.000 0.000 0.007 0.000 0.063 0.062

0.067 0.060

>Methylation code ID 1128

0.020 0.000 0.000 0.007 0.000 0.046 0.000 0.000 0.000 0.000

0.000 0.000 0.000 0.000 0.007 0.000 0.000 0.000 0.007 0.000

0.062 0.066 0.065 0.055 0.032 0.000 0.000 0.000 0.000 0.011

0.000 0.000 0.000 0.000 0.000 0.000 0.000 0.000 0.021 0.000

0.000 0.000 0.000 0.000 0.099 0.047 0.103 0.021 0.005 0.000

0.000 0.000 0.000 0.018 0.000 0.000 0.000 0.005 0.000 0.000

0.000 0.000 0.023 0.000 0.000 0.000 0.000 0.000 0.097 0.056

0.061 0.069

>Methylation code ID 1128

0.011 0.000 0.000 0.005 0.000 0.037 0.000 0.000 0.000 0.000

0.000 0.000 0.000 0.000 0.011 0.000 0.000 0.000 0.005 0.000

0.065 0.070 0.070 0.058 0.033 0.000 0.000 0.000 0.000 0.016

0.000 0.000 0.000 0.000 0.000 0.000 0.000 0.000 0.016 0.000

0.000 0.000 0.000 0.000 0.088 0.049 0.089 0.041 0.009 0.000

0.000 0.000 0.000 0.013 0.000 0.000 0.000 0.004 0.000 0.000

0.000 0.000 0.022 0.000 0.000 0.000 0.000 0.000 0.085 0.069

0.045 0.085

>Methylation code ID 1128

0.004 0.000 0.000 0.004 0.000 0.031 0.000 0.000 0.000 0.000

0.000 0.000 0.000 0.000 0.013 0.000 0.000 0.000 0.004 0.000

0.089 0.055 0.072 0.060 0.039 0.000 0.000 0.000 0.000 0.039

0.000 0.000 0.000 0.000 0.000 0.000 0.000 0.000 0.016 0.000

0.000 0.000 0.000 0.000 0.064 0.071 0.076 0.030 0.010 0.000

0.000 0.000 0.000 0.021 0.000 0.000 0.000 0.005 0.000 0.000

0.005 0.000 0.016 0.000 0.000 0.000 0.000 0.000 0.065 0.070

0.058 0.082

>Methylation code ID 1128

0.065 0.000 0.000 0.000 0.000 0.054 0.000 0.000 0.000 0.000

0.000 0.000 0.000 0.000 0.011 0.000 0.000 0.000 0.000 0.000

0.060 0.066 0.035 0.042 0.013 0.000 0.000 0.000 0.000 0.025

0.000 0.000 0.000 0.006 0.000 0.000 0.013 0.000 0.019 0.000

0.000 0.000 0.000 0.000 0.070 0.056 0.048 0.084 0.000 0.000

0.000 0.005 0.000 0.028 0.000 0.000 0.000 0.000 0.000 0.000

0.000 0.000 0.019 0.000 0.000 0.000 0.005 0.000 0.081 0.057

0.071 0.069

>Methylation code ID 1128

0.065 0.000 0.000 0.000 0.000 0.078 0.000 0.000 0.000 0.000

0.000 0.000 0.000 0.000 0.013 0.000 0.000 0.000 0.000 0.000

0.039 0.044 0.043 0.051 0.013 0.000 0.000 0.000 0.000 0.033

0.000 0.000 0.000 0.007 0.000 0.000 0.013 0.000 0.013 0.000

0.000 0.000 0.000 0.000 0.074 0.065 0.050 0.065 0.000 0.000

0.000 0.004 0.000 0.024 0.000 0.000 0.000 0.000 0.000 0.000

0.000 0.000 0.016 0.000 0.000 0.000 0.004 0.000 0.077 0.069

0.075 0.065

>Methylation code ID 1128

0.058 0.000 0.000 0.000 0.000 0.081 0.000 0.000 0.000 0.000

0.000 0.000 0.000 0.000 0.012 0.000 0.000 0.000 0.000 0.000

0.064 0.038 0.037 0.044 0.019 0.000 0.000 0.000 0.000 0.031

0.000 0.000 0.000 0.006 0.000 0.000 0.012 0.000 0.012 0.000

0.000 0.000 0.000 0.000 0.069 0.075 0.046 0.063 0.004 0.000

0.000 0.000 0.000 0.019 0.000 0.000 0.000 0.000 0.000 0.000

0.000 0.000 0.019 0.000 0.000 0.000 0.004 0.000 0.080 0.065

0.076 0.067

>Methylation code ID 1128

0.039 0.000 0.000 0.000 0.000 0.069 0.000 0.000 0.000 0.000

0.000 0.000 0.010 0.000 0.010 0.000 0.000 0.000 0.000 0.000

0.062 0.059 0.037 0.048 0.020 0.000 0.000 0.000 0.000 0.033

0.000 0.000 0.000 0.007 0.000 0.000 0.013 0.000 0.013 0.000

0.000 0.000 0.000 0.000 0.073 0.079 0.028 0.068 0.008 0.000

0.000 0.000 0.000 0.017 0.000 0.000 0.000 0.000 0.000 0.000

0.000 0.000 0.021 0.000 0.000 0.000 0.004 0.000 0.089 0.057

0.081 0.056

>Methylation code ID 1128

0.008 0.000 0.000 0.004 0.000 0.021 0.000 0.000 0.000 0.000

0.000 0.000 0.004 0.000 0.017 0.000 0.000 0.000 0.000 0.000

0.078 0.063 0.071 0.068 0.027 0.000 0.000 0.000 0.000 0.034

0.000 0.000 0.000 0.000 0.000 0.000 0.007 0.000 0.014 0.007

0.000 0.000 0.000 0.000 0.079 0.063 0.066 0.036 0.018 0.000

0.000 0.000 0.000 0.030 0.000 0.000 0.000 0.000 0.000 0.000

0.012 0.000 0.012 0.000 0.000 0.000 0.000 0.000 0.078 0.086

0.019 0.078

>Methylation code ID 1128

0.016 0.000 0.000 0.000 0.000 0.027 0.000 0.000 0.000 0.000

0.000 0.000 0.016 0.000 0.011 0.000 0.000 0.000 0.000 0.000

0.075 0.080 0.034 0.074 0.009 0.000 0.000 0.005 0.000 0.018

0.000 0.000 0.000 0.005 0.000 0.000 0.005 0.000 0.018 0.000

0.000 0.000 0.000 0.000 0.074 0.073 0.069 0.059 0.021 0.000

0.000 0.000 0.000 0.035 0.000 0.000 0.000 0.000 0.000 0.000

0.007 0.000 0.014 0.007 0.000 0.000 0.000 0.000 0.080 0.064

0.069 0.037

>Methylation code ID 1128

0.021 0.000 0.000 0.000 0.000 0.035 0.000 0.000 0.000 0.000

0.000 0.000 0.007 0.000 0.014 0.007 0.000 0.000 0.000 0.000

0.080 0.064 0.069 0.037 0.016 0.000 0.000 0.005 0.000 0.026

0.000 0.000 0.000 0.000 0.000 0.000 0.010 0.000 0.010 0.000

0.000 0.000 0.000 0.000 0.072 0.079 0.038 0.077 0.010 0.000

0.000 0.005 0.000 0.020 0.000 0.000 0.000 0.005 0.000 0.000

0.005 0.000 0.015 0.000 0.000 0.000 0.000 0.000 0.068 0.080

0.077 0.049

>Methylation code ID 1128

0.009 0.000 0.000 0.004 0.000 0.013 0.000 0.000 0.000 0.004

0.000 0.000 0.004 0.000 0.018 0.004 0.000 0.000 0.000 0.000

0.059 0.072 0.085 0.061 0.024 0.000 0.000 0.000 0.000 0.024

0.000 0.000 0.000 0.000 0.000 0.000 0.006 0.000 0.012 0.006

0.000 0.000 0.000 0.000 0.069 0.073 0.081 0.038 0.011 0.000

0.000 0.005 0.000 0.022 0.000 0.000 0.000 0.000 0.000 0.000

0.016 0.000 0.011 0.000 0.000 0.000 0.000 0.000 0.077 0.078

0.046 0.067

>Methylation code ID 1128

0.011 0.000 0.000 0.005 0.000 0.027 0.000 0.000 0.000 0.000

0.000 0.000 0.016 0.000 0.011 0.000 0.000 0.000 0.000 0.000

0.082 0.079 0.040 0.061 0.014 0.000 0.000 0.005 0.000 0.014

0.000 0.000 0.000 0.005 0.000 0.000 0.005 0.000 0.014 0.005

0.000 0.000 0.000 0.000 0.077 0.053 0.074 0.066 0.018 0.000

0.000 0.000 0.000 0.018 0.000 0.000 0.000 0.000 0.000 0.000

0.012 0.000 0.012 0.006 0.000 0.000 0.000 0.000 0.076 0.080

0.070 0.040

>Methylation code ID 1128

0.022 0.000 0.005 0.000 0.000 0.011 0.000 0.000 0.000 0.000

0.000 0.000 0.011 0.000 0.011 0.005 0.000 0.000 0.000 0.000

0.073 0.068 0.070 0.059 0.005 0.000 0.000 0.010 0.000 0.015

0.000 0.000 0.000 0.005 0.000 0.000 0.015 0.000 0.010 0.000

0.005 0.000 0.000 0.000 0.079 0.061 0.076 0.052 0.011 0.000

0.000 0.005 0.000 0.011 0.000 0.000 0.000 0.005 0.000 0.000

0.011 0.000 0.011 0.011 0.000 0.000 0.000 0.000 0.080 0.049

0.075 0.066

>Methylation code ID 1128

0.020 0.000 0.005 0.000 0.000 0.010 0.000 0.000 0.000 0.000

0.000 0.000 0.005 0.000 0.015 0.005 0.000 0.000 0.000 0.000

0.074 0.078 0.054 0.066 0.006 0.000 0.000 0.017 0.000 0.017

0.000 0.000 0.000 0.006 0.000 0.000 0.017 0.000 0.006 0.000

0.006 0.000 0.000 0.000 0.066 0.064 0.069 0.062 0.005 0.000

0.000 0.005 0.000 0.009 0.000 0.000 0.000 0.005 0.000 0.000

0.009 0.000 0.009 0.009 0.000 0.000 0.005 0.000 0.076 0.048

0.076 0.078

>Methylation code ID 1128

0.004 0.000 0.000 0.004 0.000 0.008 0.000 0.000 0.000 0.004

0.000 0.000 0.008 0.000 0.012 0.008 0.000 0.000 0.004 0.000

0.073 0.054 0.074 0.079 0.019 0.000 0.005 0.000 0.000 0.014

0.000 0.000 0.000 0.000 0.000 0.000 0.005 0.000 0.014 0.000

0.000 0.000 0.000 0.000 0.075 0.072 0.050 0.078 0.000 0.000

0.000 0.017 0.000 0.017 0.000 0.000 0.000 0.006 0.000 0.000

0.017 0.000 0.006 0.000 0.006 0.000 0.000 0.000 0.069 0.064

0.067 0.063

>Methylation code ID 1128

0.000 0.000 0.000 0.016 0.005 0.016 0.000 0.000 0.000 0.005

0.000 0.000 0.016 0.000 0.005 0.000 0.005 0.000 0.000 0.000

0.071 0.062 0.065 0.066 0.004 0.000 0.000 0.004 0.000 0.008

0.000 0.000 0.000 0.004 0.000 0.000 0.004 0.000 0.012 0.012

0.000 0.000 0.004 0.000 0.079 0.061 0.068 0.073 0.017 0.000

0.006 0.000 0.000 0.017 0.000 0.000 0.000 0.000 0.000 0.000

0.006 0.000 0.011 0.000 0.000 0.006 0.000 0.000 0.073 0.085

0.039 0.076

>Methylation code ID 1128

0.000 0.000 0.000 0.004 0.000 0.007 0.000 0.000 0.000 0.000

0.000 0.000 0.007 0.000 0.015 0.007 0.000 0.000 0.007 0.000

0.055 0.068 0.081 0.082 0.017 0.000 0.006 0.000 0.000 0.017

0.000 0.000 0.000 0.000 0.000 0.000 0.011 0.000 0.011 0.000

0.000 0.011 0.000 0.000 0.069 0.076 0.043 0.072 0.000 0.000

0.000 0.011 0.005 0.011 0.000 0.000 0.000 0.011 0.000 0.000

0.011 0.000 0.005 0.005 0.005 0.000 0.000 0.000 0.062 0.077

0.062 0.068

>Methylation code ID 1128

0.020 0.000 0.007 0.000 0.000 0.027 0.000 0.000 0.000 0.000

0.000 0.000 0.013 0.000 0.007 0.000 0.000 0.013 0.000 0.000

0.066 0.052 0.053 0.077 0.005 0.000 0.000 0.009 0.005 0.009

0.000 0.000 0.000 0.005 0.000 0.000 0.009 0.000 0.009 0.005

0.005 0.000 0.000 0.000 0.067 0.073 0.067 0.065 0.000 0.000

0.000 0.004 0.000 0.012 0.000 0.000 0.000 0.000 0.000 0.000

0.004 0.000 0.020 0.004 0.000 0.000 0.004 0.000 0.065 0.059

0.079 0.083

>Methylation code ID 1128

0.019 0.000 0.006 0.000 0.000 0.019 0.000 0.000 0.006 0.000

0.000 0.000 0.012 0.000 0.006 0.000 0.000 0.012 0.000 0.000

0.055 0.060 0.059 0.079 0.005 0.000 0.000 0.009 0.005 0.005

0.000 0.000 0.000 0.005 0.000 0.005 0.009 0.000 0.009 0.005

0.005 0.000 0.000 0.000 0.071 0.078 0.068 0.056 0.000 0.000

0.000 0.000 0.000 0.012 0.000 0.000 0.004 0.000 0.000 0.000

0.004 0.000 0.020 0.004 0.000 0.000 0.004 0.000 0.064 0.055

0.087 0.078

>Methylation code ID 1128

0.020 0.007 0.007 0.000 0.000 0.020 0.000 0.000 0.007 0.000

0.000 0.000 0.013 0.000 0.000 0.000 0.000 0.013 0.000 0.000

0.061 0.057 0.053 0.075 0.005 0.000 0.000 0.005 0.005 0.005

0.000 0.000 0.000 0.005 0.000 0.005 0.009 0.000 0.009 0.005

0.005 0.005 0.000 0.000 0.071 0.075 0.074 0.052 0.000 0.000

0.000 0.000 0.000 0.013 0.000 0.000 0.004 0.000 0.004 0.000

0.004 0.000 0.017 0.004 0.000 0.000 0.004 0.000 0.063 0.055

0.091 0.072

>Methylation code ID 1128

0.016 0.005 0.005 0.000 0.000 0.010 0.000 0.000 0.005 0.000

0.000 0.000 0.010 0.000 0.005 0.000 0.000 0.005 0.005 0.000

0.070 0.072 0.051 0.072 0.005 0.000 0.005 0.005 0.000 0.005

0.000 0.000 0.000 0.005 0.000 0.005 0.010 0.000 0.010 0.005

0.005 0.005 0.000 0.000 0.063 0.068 0.078 0.061 0.000 0.000

0.000 0.004 0.000 0.009 0.000 0.000 0.004 0.004 0.004 0.000

0.004 0.000 0.017 0.000 0.000 0.000 0.004 0.000 0.066 0.062

0.086 0.068

>Methylation code ID 1128

0.000 0.000 0.000 0.004 0.000 0.008 0.000 0.004 0.004 0.004

0.004 0.000 0.004 0.000 0.016 0.000 0.000 0.000 0.004 0.000

0.068 0.059 0.081 0.073 0.015 0.005 0.005 0.000 0.000 0.010

0.000 0.000 0.005 0.000 0.000 0.000 0.005 0.000 0.005 0.005

0.000 0.005 0.005 0.000 0.072 0.076 0.051 0.070 0.006 0.000

0.006 0.006 0.000 0.006 0.000 0.000 0.000 0.006 0.000 0.006

0.011 0.000 0.006 0.006 0.006 0.006 0.000 0.000 0.059 0.064

0.081 0.061

>Methylation code ID 1261

0.015 0.000 0.000 0.000 0.000 0.000 0.005 0.000 0.000 0.005

0.000 0.000 0.020 0.000 0.010 0.000 0.005 0.000 0.005 0.000

0.067 0.067 0.062 0.071 0.013 0.000 0.000 0.000 0.000 0.006

0.000 0.000 0.000 0.000 0.000 0.000 0.044 0.000 0.006 0.000

0.006 0.000 0.006 0.000 0.077 0.055 0.059 0.060 0.005 0.000

0.005 0.000 0.000 0.005 0.000 0.000 0.000 0.005 0.000 0.000

0.030 0.000 0.010 0.000 0.000 0.000 0.000 0.000 0.074 0.048

0.076 0.075

>Methylation code ID 1261

0.041 0.000 0.000 0.007 0.000 0.007 0.000 0.000 0.000 0.007

0.000 0.000 0.007 0.000 0.007 0.007 0.007 0.000 0.000 0.000

0.062 0.072 0.053 0.058 0.006 0.000 0.000 0.000 0.000 0.006

0.000 0.000 0.006 0.000 0.000 0.000 0.018 0.018 0.012 0.000

0.000 0.000 0.000 0.000 0.077 0.048 0.074 0.068 0.011 0.005

0.005 0.005 0.000 0.000 0.000 0.000 0.000 0.000 0.000 0.000

0.000 0.000 0.021 0.016 0.000 0.000 0.000 0.000 0.093 0.031

0.098 0.047

>Methylation code ID 1329

0.000 0.015 0.000 0.000 0.000 0.000 0.023 0.008 0.000 0.008

0.000 0.000 0.000 0.000 0.000 0.015 0.000 0.008 0.000 0.008

0.068 0.073 0.043 0.065 0.008 0.000 0.008 0.008 0.000 0.000

0.000 0.016 0.000 0.000 0.000 0.000 0.008 0.000 0.000 0.016

0.016 0.008 0.000 0.008 0.057 0.066 0.044 0.071 0.000 0.000

0.000 0.000 0.000 0.000 0.000 0.007 0.000 0.020 0.007 0.007

0.014 0.007 0.007 0.014 0.000 0.000 0.000 0.000 0.070 0.086

0.050 0.046

>Methylation code ID 1329

0.016 0.000 0.000 0.000 0.000 0.016 0.000 0.000 0.000 0.016

0.000 0.008 0.000 0.008 0.000 0.000 0.000 0.016 0.016 0.008

0.060 0.063 0.045 0.065 0.000 0.005 0.000 0.005 0.000 0.010

0.000 0.005 0.000 0.005 0.000 0.000 0.000 0.000 0.010 0.010

0.005 0.005 0.000 0.005 0.074 0.073 0.069 0.051 0.000 0.006

0.000 0.000 0.006 0.012 0.000 0.006 0.000 0.000 0.000 0.000

0.000 0.000 0.012 0.012 0.000 0.006 0.000 0.000 0.076 0.063

0.087 0.049

>Methylation code ID 1329

0.000 0.006 0.000 0.000 0.006 0.012 0.000 0.006 0.000 0.000

0.000 0.000 0.000 0.000 0.012 0.012 0.000 0.006 0.000 0.000

0.076 0.063 0.087 0.049 0.016 0.000 0.000 0.000 0.000 0.016

0.000 0.000 0.000 0.016 0.000 0.000 0.000 0.008 0.000 0.000

0.000 0.016 0.024 0.008 0.057 0.069 0.039 0.064 0.000 0.005

0.000 0.005 0.000 0.011 0.000 0.000 0.000 0.005 0.000 0.000

0.000 0.000 0.011 0.011 0.005 0.005 0.000 0.005 0.075 0.069

0.070 0.055

>Methylation code ID 1329

0.015 0.000 0.000 0.000 0.000 0.015 0.000 0.000 0.000 0.008

0.000 0.008 0.000 0.015 0.000 0.000 0.000 0.015 0.015 0.008

0.042 0.069 0.058 0.066 0.000 0.000 0.000 0.005 0.000 0.011

0.000 0.005 0.005 0.005 0.000 0.000 0.000 0.000 0.011 0.011

0.005 0.005 0.000 0.005 0.074 0.083 0.079 0.027 0.011 0.005

0.000 0.000 0.005 0.005 0.000 0.005 0.005 0.000 0.000 0.000

0.000 0.000 0.011 0.005 0.000 0.000 0.000 0.000 0.084 0.084

0.067 0.045

>Methylation code ID 1329

0.010 0.005 0.000 0.000 0.005 0.005 0.000 0.005 0.005 0.000

0.000 0.000 0.000 0.000 0.010 0.010 0.000 0.000 0.000 0.000

0.083 0.080 0.063 0.053 0.014 0.000 0.000 0.000 0.000 0.007

0.000 0.000 0.000 0.007 0.000 0.007 0.000 0.014 0.007 0.000

0.000 0.014 0.014 0.007 0.039 0.061 0.061 0.084 0.000 0.000

0.000 0.006 0.000 0.012 0.000 0.006 0.006 0.006 0.000 0.000

0.000 0.000 0.012 0.012 0.006 0.000 0.000 0.006 0.078 0.091

0.070 0.022

>Methylation code ID 1329

0.005 0.005 0.005 0.000 0.000 0.009 0.000 0.005 0.014 0.000

0.000 0.000 0.005 0.000 0.005 0.009 0.000 0.000 0.000 0.000

0.078 0.064 0.062 0.068 0.020 0.000 0.000 0.000 0.000 0.000

0.000 0.007 0.013 0.007 0.000 0.007 0.007 0.000 0.007 0.007

0.000 0.000 0.000 0.000 0.072 0.073 0.056 0.060 0.006 0.000

0.000 0.000 0.000 0.000 0.000 0.000 0.000 0.006 0.000 0.006

0.000 0.019 0.012 0.000 0.006 0.000 0.012 0.000 0.064 0.061

0.057 0.083

>Methylation code ID 1329

0.005 0.005 0.005 0.005 0.000 0.009 0.000 0.005 0.014 0.000

0.000 0.000 0.009 0.000 0.000 0.005 0.000 0.000 0.000 0.000

0.068 0.064 0.057 0.084 0.019 0.000 0.000 0.000 0.000 0.000

0.000 0.006 0.013 0.006 0.000 0.006 0.013 0.000 0.000 0.013

0.000 0.000 0.000 0.000 0.063 0.068 0.063 0.061 0.006 0.000

0.000 0.000 0.000 0.000 0.000 0.000 0.000 0.006 0.000 0.006

0.000 0.017 0.017 0.000 0.006 0.000 0.006 0.000 0.076 0.049

0.046 0.100

>Methylation code ID 1329

0.000 0.000 0.014 0.000 0.007 0.007 0.007 0.000 0.000 0.014

0.000 0.000 0.000 0.007 0.000 0.014 0.000 0.000 0.007 0.007

0.061 0.055 0.070 0.067 0.006 0.000 0.000 0.000 0.006 0.006

0.000 0.006 0.006 0.000 0.000 0.000 0.006 0.000 0.006 0.006

0.019 0.000 0.006 0.000 0.066 0.065 0.057 0.069 0.000 0.000

0.000 0.000 0.005 0.011 0.000 0.000 0.000 0.016 0.000 0.000

0.011 0.000 0.011 0.005 0.000 0.005 0.000 0.000 0.071 0.081

0.071 0.045

>Methylation code ID 1329

0.000 0.000 0.000 0.000 0.000 0.010 0.000 0.000 0.000 0.016

0.000 0.005 0.010 0.010 0.010 0.000 0.000 0.005 0.000 0.000

0.064 0.073 0.079 0.049 0.000 0.000 0.013 0.000 0.000 0.006

0.000 0.006 0.006 0.006 0.000 0.006 0.000 0.006 0.000 0.013

0.000 0.000 0.006 0.006 0.057 0.053 0.071 0.075 0.006 0.000

0.000 0.000 0.000 0.006 0.000 0.000 0.006 0.000 0.000 0.000

0.006 0.000 0.006 0.013 0.025 0.000 0.006 0.000 0.064 0.068

0.054 0.072

>Methylation code ID 1329

0.006 0.000 0.006 0.000 0.000 0.006 0.000 0.013 0.013 0.013

0.000 0.006 0.000 0.006 0.000 0.013 0.000 0.000 0.000 0.000

0.054 0.059 0.060 0.076 0.006 0.000 0.000 0.000 0.000 0.000

0.000 0.000 0.006 0.006 0.000 0.000 0.006 0.006 0.012 0.018

0.012 0.000 0.000 0.006 0.068 0.074 0.042 0.072 0.000 0.005

0.005 0.000 0.000 0.000 0.000 0.000 0.005 0.016 0.000 0.005

0.005 0.010 0.005 0.000 0.005 0.000 0.000 0.000 0.069 0.062

0.069 0.071

>Methylation code ID 1329

0.004 0.004 0.000 0.000 0.000 0.000 0.000 0.004 0.004 0.000

0.000 0.000 0.000 0.000 0.012 0.004 0.004 0.012 0.000 0.004

0.055 0.069 0.091 0.067 0.006 0.000 0.000 0.000 0.000 0.013

0.006 0.000 0.000 0.006 0.000 0.000 0.006 0.000 0.013 0.013

0.006 0.000 0.000 0.000 0.070 0.081 0.062 0.052 0.005 0.009

0.005 0.000 0.000 0.005 0.005 0.000 0.005 0.005 0.000 0.000

0.000 0.005 0.005 0.000 0.005 0.005 0.000 0.000 0.068 0.068

0.067 0.074

>Methylation code ID 1329

0.006 0.000 0.000 0.000 0.000 0.012 0.006 0.000 0.000 0.006

0.000 0.000 0.006 0.006 0.012 0.012 0.006 0.000 0.000 0.000

0.067 0.076 0.065 0.054 0.004 0.004 0.004 0.000 0.000 0.004

0.004 0.000 0.004 0.004 0.000 0.000 0.000 0.004 0.009 0.004

0.004 0.004 0.000 0.000 0.063 0.072 0.067 0.075 0.009 0.000

0.000 0.000 0.000 0.000 0.000 0.004 0.004 0.000 0.000 0.000

0.000 0.000 0.013 0.004 0.004 0.009 0.000 0.004 0.051 0.070

0.085 0.073

>Methylation code ID 1329

0.006 0.000 0.000 0.000 0.000 0.011 0.006 0.000 0.000 0.006

0.000 0.000 0.006 0.006 0.011 0.017 0.006 0.000 0.000 0.000

0.064 0.072 0.061 0.062 0.004 0.004 0.004 0.000 0.000 0.004

0.004 0.000 0.000 0.004 0.000 0.000 0.004 0.004 0.009 0.004

0.004 0.004 0.000 0.000 0.062 0.084 0.067 0.063 0.010 0.000

0.000 0.000 0.000 0.000 0.000 0.005 0.000 0.000 0.000 0.000

0.000 0.000 0.014 0.005 0.005 0.014 0.000 0.005 0.051 0.073

0.084 0.069

>Methylation code ID 1329

0.004 0.004 0.000 0.004 0.000 0.004 0.004 0.000 0.000 0.004

0.000 0.000 0.004 0.004 0.009 0.004 0.004 0.004 0.000 0.000

0.065 0.081 0.071 0.059 0.009 0.000 0.000 0.000 0.000 0.000

0.000 0.004 0.004 0.004 0.000 0.000 0.000 0.000 0.013 0.004

0.000 0.013 0.000 0.004 0.046 0.074 0.083 0.072 0.008 0.000

0.000 0.000 0.000 0.016 0.008 0.000 0.000 0.008 0.000 0.000

0.000 0.008 0.008 0.023 0.008 0.000 0.000 0.000 0.063 0.071

0.054 0.059

>Methylation code ID 1329

0.009 0.000 0.000 0.000 0.000 0.000 0.000 0.009 0.004 0.004

0.000 0.000 0.000 0.000 0.013 0.004 0.000 0.009 0.000 0.004

0.049 0.083 0.070 0.073 0.007 0.000 0.000 0.000 0.000 0.014

0.007 0.000 0.000 0.007 0.000 0.007 0.000 0.007 0.007 0.022

0.007 0.000 0.000 0.000 0.067 0.068 0.047 0.064 0.005 0.000

0.005 0.005 0.000 0.000 0.005 0.000 0.000 0.005 0.000 0.000

0.005 0.000 0.010 0.005 0.010 0.005 0.000 0.000 0.052 0.069

0.073 0.077

>Methylation code ID 1329

0.000 0.000 0.000 0.007 0.000 0.007 0.000 0.000 0.000 0.007

0.000 0.007 0.000 0.007 0.013 0.020 0.007 0.007 0.000 0.000

0.068 0.065 0.062 0.061 0.006 0.006 0.006 0.006 0.000 0.000

0.006 0.000 0.006 0.000 0.000 0.000 0.006 0.000 0.006 0.006

0.006 0.006 0.000 0.000 0.068 0.088 0.057 0.056 0.013 0.000

0.000 0.000 0.000 0.000 0.000 0.013 0.006 0.006 0.000 0.000

0.000 0.006 0.006 0.006 0.000 0.006 0.000 0.006 0.045 0.081

0.067 0.070

>Methylation code ID 1345

0.006 0.000 0.000 0.006 0.006 0.006 0.012 0.006 0.000 0.006

0.000 0.000 0.012 0.000 0.000 0.012 0.000 0.000 0.006 0.000

0.060 0.053 0.080 0.065 0.011 0.000 0.000 0.000 0.000 0.011

0.000 0.011 0.000 0.022 0.000 0.000 0.022 0.000 0.000 0.033

0.011 0.011 0.000 0.000 0.050 0.059 0.052 0.042 0.000 0.005

0.000 0.005 0.000 0.000 0.005 0.000 0.000 0.015 0.000 0.000

0.005 0.000 0.010 0.015 0.000 0.000 0.000 0.000 0.073 0.067

0.070 0.063

>Methylation code ID 1345

0.010 0.000 0.000 0.005 0.000 0.000 0.000 0.000 0.005 0.010

0.005 0.000 0.000 0.000 0.010 0.000 0.005 0.005 0.005 0.005

0.081 0.054 0.082 0.051 0.005 0.000 0.000 0.005 0.000 0.005

0.005 0.000 0.000 0.000 0.000 0.000 0.000 0.005 0.010 0.005

0.005 0.000 0.010 0.005 0.075 0.064 0.063 0.069 0.012 0.000

0.000 0.000 0.000 0.012 0.006 0.006 0.006 0.000 0.000 0.006

0.000 0.000 0.006 0.006 0.006 0.000 0.000 0.000 0.069 0.057

0.065 0.076

>Methylation code ID 1345

0.005 0.000 0.005 0.005 0.000 0.005 0.005 0.000 0.000 0.000

0.000 0.000 0.000 0.005 0.009 0.000 0.005 0.000 0.009 0.005

0.076 0.070 0.058 0.072 0.006 0.000 0.000 0.000 0.000 0.018

0.006 0.006 0.006 0.000 0.000 0.006 0.000 0.000 0.006 0.006

0.012 0.000 0.000 0.000 0.060 0.056 0.068 0.079 0.010 0.000

0.000 0.005 0.000 0.005 0.000 0.000 0.000 0.005 0.005 0.000

0.000 0.000 0.010 0.000 0.005 0.005 0.005 0.005 0.071 0.059

0.091 0.050

>Methylation code ID 1345

0.007 0.000 0.000 0.000 0.000 0.026 0.007 0.007 0.000 0.000

0.000 0.007 0.000 0.000 0.007 0.007 0.013 0.000 0.000 0.000

0.063 0.059 0.063 0.070 0.011 0.000 0.000 0.006 0.000 0.011

0.000 0.000 0.000 0.006 0.006 0.000 0.000 0.006 0.006 0.000

0.006 0.006 0.006 0.006 0.070 0.053 0.089 0.048 0.004 0.000

0.004 0.000 0.000 0.004 0.004 0.000 0.000 0.000 0.000 0.000

0.000 0.004 0.013 0.000 0.004 0.000 0.009 0.004 0.079 0.057

0.063 0.080

>Methylation code ID 1345

0.000 0.000 0.009 0.000 0.000 0.027 0.009 0.000 0.000 0.009

0.000 0.000 0.018 0.000 0.000 0.009 0.009 0.000 0.000 0.000

0.060 0.059 0.052 0.071 0.000 0.006 0.011 0.006 0.000 0.006

0.000 0.006 0.000 0.006 0.000 0.000 0.011 0.006 0.000 0.000

0.006 0.006 0.006 0.000 0.063 0.067 0.063 0.068 0.005 0.005

0.000 0.000 0.000 0.005 0.000 0.000 0.000 0.010 0.000 0.000

0.005 0.000 0.010 0.000 0.010 0.000 0.000 0.005 0.071 0.056

0.077 0.071

>Methylation code ID 1345

0.006 0.000 0.000 0.006 0.000 0.000 0.000 0.012 0.006 0.012

0.000 0.000 0.000 0.006 0.012 0.006 0.000 0.000 0.000 0.000

0.073 0.088 0.055 0.049 0.000 0.000 0.016 0.008 0.000 0.000

0.000 0.000 0.008 0.000 0.000 0.016 0.008 0.008 0.000 0.024

0.000 0.000 0.000 0.000 0.057 0.072 0.049 0.066 0.000 0.000

0.000 0.000 0.000 0.000 0.006 0.006 0.012 0.006 0.000 0.006

0.000 0.000 0.012 0.000 0.018 0.006 0.000 0.000 0.046 0.071

0.080 0.065

>Methylation code ID 1345

0.000 0.000 0.000 0.006 0.000 0.000 0.006 0.000 0.006 0.006

0.000 0.006 0.006 0.000 0.013 0.000 0.019 0.006 0.000 0.000

0.049 0.064 0.076 0.066 0.007 0.000 0.000 0.007 0.000 0.000

0.000 0.000 0.007 0.007 0.000 0.000 0.000 0.014 0.014 0.007

0.014 0.000 0.000 0.000 0.076 0.063 0.064 0.053 0.000 0.000

0.016 0.005 0.000 0.000 0.000 0.000 0.005 0.000 0.000 0.000

0.011 0.005 0.011 0.011 0.000 0.000 0.000 0.000 0.056 0.074

0.069 0.071

>Methylation code ID 1345

0.000 0.000 0.014 0.005 0.000 0.005 0.000 0.000 0.009 0.000

0.000 0.000 0.009 0.000 0.009 0.009 0.000 0.000 0.000 0.000

0.056 0.086 0.063 0.067 0.000 0.000 0.000 0.007 0.000 0.007

0.007 0.000 0.007 0.007 0.000 0.007 0.007 0.000 0.007 0.000

0.020 0.007 0.000 0.000 0.065 0.075 0.056 0.058 0.000 0.000

0.000 0.008 0.000 0.000 0.000 0.000 0.008 0.008 0.000 0.000

0.000 0.016 0.016 0.008 0.016 0.000 0.000 0.000 0.053 0.058

0.078 0.065

>Methylation code ID 1345

0.000 0.000 0.005 0.000 0.000 0.005 0.000 0.000 0.005 0.016

0.000 0.000 0.005 0.005 0.010 0.005 0.005 0.005 0.000 0.000

0.048 0.069 0.078 0.071 0.000 0.005 0.005 0.005 0.000 0.005

0.005 0.000 0.005 0.005 0.000 0.000 0.005 0.000 0.005 0.016

0.005 0.000 0.000 0.000 0.062 0.078 0.067 0.058 0.005 0.000

0.000 0.005 0.000 0.005 0.005 0.005 0.005 0.000 0.000 0.000

0.015 0.000 0.005 0.000 0.005 0.000 0.005 0.000 0.059 0.061

0.071 0.084

>Methylation code ID 1376

0.010 0.000 0.000 0.005 0.000 0.005 0.000 0.000 0.000 0.010

0.000 0.000 0.010 0.005 0.010 0.005 0.005 0.000 0.000 0.000

0.055 0.067 0.082 0.063 0.006 0.006 0.006 0.000 0.000 0.000

0.006 0.000 0.006 0.006 0.000 0.000 0.022 0.000 0.006 0.006

0.000 0.006 0.000 0.000 0.055 0.070 0.066 0.070 0.005 0.005

0.000 0.000 0.005 0.005 0.000 0.000 0.000 0.000 0.000 0.005

0.021 0.005 0.005 0.000 0.005 0.000 0.000 0.000 0.063 0.080

0.073 0.054

>Methylation code ID 1376

0.005 0.005 0.005 0.000 0.000 0.005 0.005 0.000 0.005 0.000

0.000 0.000 0.020 0.000 0.005 0.005 0.000 0.005 0.000 0.000

0.054 0.070 0.072 0.074 0.005 0.000 0.000 0.000 0.005 0.005

0.000 0.005 0.000 0.000 0.000 0.005 0.014 0.005 0.009 0.000

0.005 0.000 0.000 0.000 0.070 0.065 0.077 0.064 0.000 0.000

0.000 0.006 0.000 0.011 0.000 0.000 0.000 0.011 0.000 0.000

0.006 0.006 0.011 0.011 0.006 0.000 0.000 0.000 0.047 0.063

0.075 0.081

>Methylation code ID 1376

0.000 0.006 0.006 0.006 0.000 0.013 0.000 0.000 0.000 0.000

0.000 0.000 0.006 0.006 0.006 0.013 0.013 0.000 0.000 0.000

0.051 0.071 0.074 0.063 0.008 0.000 0.000 0.000 0.000 0.004

0.004 0.000 0.004 0.000 0.004 0.000 0.008 0.000 0.013 0.000

0.000 0.004 0.000 0.000 0.058 0.059 0.091 0.075 0.000 0.005

0.005 0.000 0.005 0.010 0.000 0.005 0.000 0.000 0.000 0.005

0.015 0.000 0.005 0.000 0.000 0.000 0.000 0.000 0.070 0.057

0.079 0.072

>Methylation code ID 1376

0.005 0.005 0.010 0.000 0.000 0.010 0.000 0.005 0.000 0.000

0.000 0.005 0.010 0.000 0.005 0.000 0.005 0.000 0.000 0.000

0.074 0.061 0.070 0.068 0.000 0.007 0.007 0.007 0.000 0.007

0.000 0.000 0.000 0.007 0.000 0.000 0.015 0.007 0.000 0.007

0.022 0.000 0.000 0.000 0.051 0.062 0.071 0.062 0.005 0.000

0.000 0.000 0.000 0.005 0.010 0.000 0.005 0.000 0.005 0.000

0.005 0.000 0.015 0.000 0.000 0.000 0.000 0.000 0.058 0.054

0.085 0.085

>Methylation code ID 1376

0.005 0.005 0.010 0.000 0.000 0.005 0.000 0.010 0.000 0.005

0.000 0.000 0.005 0.000 0.010 0.000 0.005 0.000 0.000 0.000

0.090 0.058 0.060 0.068 0.007 0.015 0.007 0.000 0.000 0.007

0.000 0.000 0.000 0.015 0.000 0.000 0.015 0.000 0.000 0.007

0.015 0.000 0.000 0.000 0.064 0.079 0.054 0.049 0.011 0.000

0.000 0.000 0.000 0.005 0.011 0.000 0.000 0.005 0.005 0.000

0.005 0.000 0.011 0.000 0.000 0.000 0.000 0.000 0.066 0.061

0.071 0.080

>Methylation code ID 1376

0.005 0.005 0.005 0.005 0.000 0.005 0.000 0.000 0.000 0.011

0.000 0.000 0.005 0.000 0.005 0.000 0.011 0.000 0.005 0.000

0.069 0.068 0.062 0.069 0.010 0.005 0.000 0.005 0.000 0.005

0.010 0.000 0.000 0.005 0.005 0.000 0.000 0.000 0.010 0.000

0.000 0.000 0.000 0.000 0.064 0.081 0.066 0.065 0.005 0.005

0.011 0.000 0.000 0.005 0.000 0.005 0.000 0.005 0.000 0.000

0.000 0.005 0.011 0.000 0.005 0.000 0.000 0.000 0.073 0.073

0.065 0.062

>Methylation code ID 1376

0.005 0.005 0.000 0.009 0.000 0.005 0.005 0.005 0.000 0.005

0.000 0.000 0.000 0.000 0.009 0.009 0.000 0.005 0.000 0.000

0.060 0.067 0.065 0.082 0.005 0.000 0.000 0.005 0.010 0.010

0.000 0.005 0.000 0.000 0.000 0.000 0.005 0.005 0.010 0.000

0.000 0.000 0.000 0.000 0.076 0.068 0.067 0.069 0.000 0.000

0.000 0.009 0.004 0.009 0.004 0.000 0.004 0.004 0.000 0.000

0.000 0.000 0.009 0.004 0.000 0.000 0.004 0.000 0.065 0.069

0.065 0.083

>Methylation code ID 1376

0.000 0.000 0.005 0.009 0.005 0.009 0.000 0.005 0.000 0.005

0.000 0.000 0.000 0.000 0.009 0.005 0.000 0.009 0.000 0.000

0.067 0.075 0.060 0.072 0.015 0.000 0.000 0.007 0.015 0.007

0.000 0.000 0.000 0.000 0.000 0.000 0.007 0.000 0.007 0.015

0.007 0.000 0.000 0.000 0.075 0.045 0.062 0.071 0.000 0.000

0.000 0.005 0.005 0.009 0.005 0.000 0.000 0.000 0.000 0.000

0.005 0.000 0.018 0.005 0.000 0.005 0.000 0.000 0.073 0.060

0.083 0.062

>Methylation code ID 1376

0.000 0.000 0.004 0.004 0.004 0.009 0.000 0.004 0.000 0.004

0.000 0.000 0.000 0.000 0.013 0.004 0.000 0.009 0.000 0.000

0.059 0.080 0.066 0.073 0.019 0.000 0.000 0.010 0.019 0.019

0.000 0.000 0.000 0.000 0.000 0.000 0.010 0.000 0.000 0.019

0.010 0.000 0.000 0.000 0.083 0.033 0.050 0.061 0.000 0.000

0.000 0.005 0.005 0.005 0.005 0.000 0.005 0.000 0.000 0.000

0.005 0.000 0.018 0.005 0.000 0.005 0.000 0.000 0.077 0.053

0.092 0.057

>Methylation code ID 1376

0.000 0.000 0.004 0.004 0.004 0.008 0.000 0.000 0.000 0.008

0.000 0.004 0.000 0.000 0.012 0.000 0.004 0.004 0.000 0.000

0.061 0.079 0.072 0.068 0.026 0.000 0.000 0.013 0.000 0.026

0.000 0.000 0.000 0.000 0.000 0.000 0.013 0.000 0.000 0.026

0.026 0.000 0.000 0.000 0.064 0.043 0.044 0.051 0.000 0.000

0.005 0.009 0.000 0.005 0.000 0.000 0.005 0.000 0.000 0.000

0.009 0.000 0.019 0.000 0.000 0.005 0.000 0.000 0.078 0.057

0.083 0.060

>Methylation code ID 1376

0.018 0.000 0.000 0.009 0.000 0.018 0.000 0.000 0.000 0.000

0.000 0.000 0.009 0.000 0.009 0.018 0.018 0.000 0.000 0.000

0.068 0.047 0.060 0.057 0.000 0.000 0.005 0.005 0.000 0.009

0.000 0.000 0.005 0.000 0.000 0.000 0.014 0.000 0.018 0.000

0.000 0.005 0.000 0.000 0.078 0.052 0.079 0.064 0.000 0.000

0.005 0.005 0.005 0.010 0.000 0.000 0.000 0.005 0.000 0.005

0.000 0.005 0.010 0.000 0.005 0.005 0.000 0.000 0.050 0.075

0.073 0.078

>Methylation code ID 1376

0.005 0.000 0.005 0.011 0.000 0.005 0.000 0.000 0.000 0.000

0.000 0.005 0.011 0.000 0.011 0.000 0.011 0.000 0.000 0.000

0.063 0.067 0.062 0.077 0.000 0.000 0.000 0.011 0.000 0.011

0.000 0.006 0.000 0.011 0.000 0.000 0.011 0.006 0.006 0.006

0.006 0.000 0.000 0.000 0.059 0.065 0.073 0.064 0.000 0.000

0.006 0.006 0.000 0.006 0.006 0.000 0.006 0.000 0.000 0.000

0.006 0.000 0.017 0.006 0.000 0.000 0.000 0.000 0.057 0.081

0.072 0.066

>Methylation code ID 1376

0.033 0.000 0.000 0.000 0.000 0.000 0.000 0.000 0.008 0.008

0.000 0.000 0.017 0.000 0.000 0.033 0.000 0.000 0.000 0.008

0.058 0.053 0.053 0.062 0.025 0.000 0.000 0.000 0.000 0.000

0.000 0.008 0.000 0.025 0.000 0.008 0.042 0.000 0.000 0.000

0.000 0.000 0.000 0.000 0.063 0.070 0.035 0.055 0.000 0.009

0.000 0.000 0.004 0.000 0.000 0.004 0.000 0.000 0.000 0.000

0.013 0.013 0.009 0.000 0.000 0.000 0.000 0.000 0.042 0.070

0.091 0.079

>Methylation code ID 1376

0.017 0.000 0.000 0.000 0.000 0.012 0.000 0.000 0.000 0.006

0.000 0.000 0.017 0.000 0.012 0.012 0.000 0.000 0.000 0.000

0.052 0.081 0.067 0.058 0.016 0.000 0.005 0.005 0.000 0.000

0.005 0.000 0.000 0.011 0.000 0.000 0.011 0.000 0.005 0.005

0.000 0.005 0.000 0.000 0.060 0.053 0.080 0.071 0.000 0.006

0.000 0.000 0.000 0.006 0.000 0.000 0.006 0.000 0.000 0.000

0.011 0.011 0.006 0.006 0.011 0.000 0.006 0.000 0.079 0.042

0.076 0.070

>Methylation code ID 1376

0.020 0.000 0.005 0.005 0.000 0.000 0.005 0.000 0.000 0.005

0.000 0.000 0.005 0.000 0.005 0.005 0.000 0.005 0.005 0.000

0.064 0.060 0.070 0.073 0.000 0.000 0.000 0.000 0.000 0.011

0.000 0.000 0.006 0.006 0.000 0.000 0.017 0.006 0.006 0.006

0.011 0.000 0.006 0.000 0.072 0.054 0.058 0.075 0.005 0.005

0.000 0.000 0.000 0.015 0.000 0.000 0.000 0.010 0.000 0.000

0.010 0.000 0.010 0.005 0.000 0.000 0.000 0.000 0.046 0.072

0.075 0.082

>Methylation code ID 1437

0.006 0.000 0.000 0.006 0.000 0.011 0.000 0.000 0.000 0.000

0.000 0.000 0.011 0.006 0.006 0.006 0.000 0.000 0.006 0.011

0.066 0.073 0.068 0.060 0.004 0.004 0.008 0.000 0.000 0.004

0.008 0.008 0.000 0.008 0.000 0.000 0.000 0.000 0.008 0.000

0.000 0.000 0.000 0.000 0.077 0.050 0.057 0.095 0.000 0.000

0.000 0.000 0.000 0.024 0.000 0.016 0.000 0.008 0.000 0.000

0.000 0.000 0.008 0.008 0.016 0.000 0.000 0.000 0.062 0.055

0.050 0.085

>Methylation code ID 1437

0.006 0.000 0.000 0.000 0.006 0.006 0.013 0.000 0.000 0.000

0.000 0.000 0.019 0.000 0.000 0.006 0.013 0.000 0.006 0.000

0.054 0.064 0.065 0.075 0.005 0.000 0.000 0.010 0.005 0.005

0.010 0.005 0.000 0.000 0.000 0.000 0.015 0.000 0.005 0.000

0.005 0.000 0.000 0.000 0.073 0.067 0.061 0.067 0.000 0.000

0.000 0.000 0.006 0.000 0.013 0.000 0.006 0.000 0.006 0.000

0.013 0.000 0.006 0.013 0.006 0.000 0.000 0.000 0.072 0.063

0.060 0.068

>Methylation code ID 1437

0.000 0.000 0.000 0.000 0.000 0.007 0.007 0.000 0.007 0.007

0.007 0.007 0.007 0.000 0.007 0.014 0.007 0.007 0.000 0.000

0.075 0.069 0.042 0.065 0.007 0.000 0.000 0.000 0.015 0.015

0.000 0.000 0.000 0.000 0.007 0.000 0.022 0.000 0.000 0.007

0.007 0.000 0.007 0.000 0.066 0.052 0.046 0.080 0.005 0.005

0.000 0.015 0.000 0.005 0.010 0.000 0.000 0.000 0.000 0.000

0.010 0.000 0.005 0.005 0.000 0.000 0.000 0.000 0.084 0.058

0.065 0.064

>Methylation code ID 1437

0.009 0.000 0.000 0.000 0.000 0.000 0.009 0.009 0.009 0.000

0.000 0.009 0.009 0.009 0.000 0.036 0.009 0.000 0.000 0.000

0.056 0.046 0.060 0.064 0.000 0.006 0.000 0.000 0.000 0.000

0.006 0.006 0.000 0.006 0.000 0.000 0.024 0.006 0.006 0.012

0.006 0.000 0.000 0.000 0.056 0.060 0.061 0.078 0.000 0.005

0.000 0.005 0.005 0.000 0.005 0.005 0.000 0.000 0.000 0.005

0.010 0.005 0.005 0.000 0.000 0.010 0.000 0.000 0.073 0.069

0.059 0.073

>Methylation code ID 1437

0.000 0.000 0.007 0.007 0.014 0.014 0.000 0.007 0.000 0.007

0.000 0.000 0.014 0.000 0.000 0.014 0.000 0.000 0.000 0.000

0.073 0.064 0.060 0.053 0.017 0.009 0.000 0.000 0.009 0.000

0.000 0.009 0.000 0.035 0.000 0.000 0.009 0.000 0.000 0.017

0.000 0.000 0.000 0.000 0.063 0.059 0.035 0.073 0.005 0.005

0.000 0.000 0.005 0.000 0.000 0.000 0.000 0.009 0.000 0.000

0.005 0.000 0.009 0.000 0.000 0.005 0.009 0.005 0.067 0.071

0.072 0.068

>Methylation code ID 1437

0.006 0.000 0.000 0.000 0.006 0.006 0.000 0.000 0.012 0.012

0.012 0.000 0.006 0.000 0.006 0.000 0.006 0.000 0.000 0.000

0.049 0.070 0.085 0.059 0.000 0.005 0.000 0.000 0.005 0.005

0.000 0.000 0.000 0.005 0.000 0.000 0.005 0.005 0.005 0.015

0.000 0.005 0.005 0.000 0.069 0.074 0.053 0.076 0.006 0.000

0.012 0.006 0.000 0.006 0.000 0.000 0.000 0.000 0.000 0.000

0.006 0.000 0.006 0.012 0.000 0.006 0.000 0.006 0.058 0.067

0.069 0.075

>Methylation code ID 1437

0.010 0.000 0.000 0.000 0.000 0.010 0.000 0.000 0.015 0.005

0.005 0.000 0.000 0.000 0.010 0.000 0.000 0.005 0.000 0.005

0.066 0.076 0.047 0.080 0.006 0.000 0.000 0.006 0.006 0.012

0.000 0.000 0.017 0.000 0.000 0.000 0.006 0.006 0.000 0.006

0.006 0.000 0.006 0.000 0.056 0.060 0.075 0.068 0.000 0.000

0.000 0.000 0.000 0.018 0.000 0.000 0.018 0.006 0.000 0.006

0.000 0.006 0.012 0.006 0.000 0.000 0.000 0.000 0.075 0.040

0.101 0.045

>Methylation code ID 1440

0.007 0.000 0.000 0.000 0.007 0.000 0.000 0.000 0.000 0.014

0.000 0.021 0.007 0.000 0.000 0.021 0.014 0.000 0.000 0.000

0.062 0.055 0.063 0.062 0.015 0.008 0.000 0.000 0.000 0.000

0.000 0.008 0.000 0.015 0.000 0.000 0.015 0.008 0.000 0.008

0.015 0.000 0.008 0.000 0.059 0.050 0.069 0.055 0.006 0.000

0.000 0.000 0.012 0.000 0.006 0.000 0.006 0.000 0.000 0.000

0.006 0.023 0.006 0.006 0.000 0.000 0.000 0.000 0.053 0.078

0.073 0.059

>Methylation code ID 1440

0.006 0.000 0.006 0.000 0.000 0.000 0.006 0.000 0.000 0.013

0.006 0.013 0.006 0.000 0.006 0.013 0.000 0.006 0.000 0.000

0.068 0.060 0.054 0.069 0.006 0.012 0.000 0.000 0.000 0.000

0.006 0.018 0.000 0.006 0.000 0.000 0.006 0.006 0.000 0.000

0.006 0.006 0.006 0.000 0.073 0.058 0.072 0.053 0.012 0.000

0.000 0.000 0.006 0.006 0.000 0.000 0.006 0.000 0.000 0.000

0.006 0.006 0.006 0.006 0.006 0.000 0.000 0.006 0.063 0.077

0.069 0.061

>Methylation code ID 1440

0.000 0.005 0.000 0.000 0.000 0.000 0.005 0.010 0.000 0.005

0.000 0.000 0.016 0.000 0.005 0.000 0.010 0.005 0.005 0.000

0.070 0.051 0.071 0.073 0.006 0.000 0.000 0.000 0.000 0.006

0.006 0.000 0.000 0.000 0.000 0.000 0.011 0.000 0.011 0.006

0.006 0.006 0.000 0.011 0.066 0.075 0.069 0.055 0.006 0.000

0.006 0.000 0.000 0.000 0.011 0.000 0.000 0.000 0.006 0.006

0.011 0.000 0.006 0.006 0.006 0.006 0.000 0.000 0.064 0.067

0.059 0.076

>Methylation code ID 1440

0.006 0.000 0.006 0.000 0.000 0.000 0.006 0.000 0.000 0.000

0.000 0.000 0.011 0.000 0.011 0.006 0.006 0.006 0.000 0.011

0.056 0.078 0.074 0.059 0.006 0.000 0.000 0.000 0.000 0.000

0.011 0.000 0.000 0.000 0.017 0.006 0.011 0.000 0.006 0.006

0.006 0.006 0.000 0.000 0.059 0.068 0.063 0.069 0.000 0.006

0.000 0.000 0.000 0.000 0.006 0.012 0.000 0.006 0.000 0.000

0.018 0.000 0.006 0.000 0.006 0.006 0.000 0.000 0.073 0.048

0.064 0.082

>Methylation code ID 1440

0.012 0.006 0.000 0.000 0.000 0.000 0.006 0.000 0.000 0.000

0.012 0.006 0.018 0.000 0.006 0.006 0.006 0.000 0.000 0.000

0.060 0.068 0.060 0.068 0.005 0.000 0.000 0.000 0.000 0.000

0.009 0.005 0.000 0.000 0.000 0.000 0.014 0.000 0.009 0.000

0.005 0.005 0.005 0.000 0.077 0.065 0.075 0.062 0.005 0.000

0.005 0.000 0.000 0.005 0.005 0.000 0.000 0.000 0.005 0.000

0.010 0.000 0.005 0.005 0.005 0.005 0.000 0.005 0.044 0.072

0.083 0.072

>Methylation code ID 1440

0.004 0.000 0.004 0.000 0.008 0.004 0.004 0.000 0.000 0.000

0.004 0.004 0.008 0.000 0.008 0.000 0.000 0.004 0.000 0.000

0.049 0.082 0.087 0.060 0.013 0.006 0.000 0.000 0.006 0.006

0.006 0.006 0.000 0.006 0.013 0.000 0.013 0.000 0.000 0.006

0.000 0.000 0.000 0.000 0.045 0.070 0.067 0.068 0.005 0.000

0.000 0.005 0.010 0.000 0.005 0.000 0.000 0.000 0.000 0.000

0.015 0.000 0.005 0.000 0.005 0.000 0.005 0.000 0.058 0.072

0.067 0.083

>Methylation code ID 1440

0.011 0.006 0.000 0.006 0.006 0.006 0.000 0.006 0.006 0.006

0.011 0.000 0.011 0.000 0.000 0.000 0.000 0.000 0.000 0.000

0.042 0.068 0.074 0.075 0.004 0.000 0.000 0.004 0.008 0.000

0.004 0.000 0.004 0.000 0.000 0.004 0.008 0.000 0.004 0.000

0.000 0.000 0.008 0.000 0.055 0.076 0.078 0.073 0.004 0.000

0.004 0.000 0.009 0.009 0.004 0.000 0.004 0.000 0.004 0.004

0.000 0.000 0.009 0.000 0.000 0.000 0.000 0.000 0.049 0.082

0.084 0.065

>Methylation code ID 1440

0.000 0.000 0.000 0.000 0.006 0.012 0.000 0.000 0.006 0.012

0.006 0.000 0.006 0.000 0.006 0.000 0.012 0.000 0.000 0.012

0.058 0.062 0.070 0.064 0.000 0.005 0.000 0.005 0.005 0.011

0.005 0.000 0.005 0.005 0.000 0.000 0.005 0.000 0.005 0.005

0.011 0.000 0.000 0.000 0.069 0.052 0.076 0.067 0.012 0.000

0.000 0.000 0.000 0.006 0.006 0.000 0.000 0.012 0.000 0.006

0.000 0.000 0.006 0.006 0.006 0.006 0.000 0.006 0.062 0.062

0.072 0.064

>Methylation code ID 1440

0.013 0.007 0.000 0.007 0.007 0.013 0.007 0.000 0.000 0.007

0.000 0.000 0.000 0.007 0.000 0.013 0.007 0.000 0.000 0.000

0.054 0.051 0.072 0.070 0.006 0.000 0.006 0.000 0.000 0.000

0.000 0.000 0.000 0.012 0.000 0.006 0.000 0.006 0.012 0.012

0.006 0.006 0.000 0.006 0.077 0.059 0.062 0.061 0.000 0.005

0.000 0.000 0.005 0.010 0.000 0.000 0.000 0.010 0.000 0.000

0.000 0.000 0.010 0.000 0.010 0.005 0.000 0.005 0.069 0.063

0.066 0.074

>Methylation code ID 1440

0.013 0.006 0.000 0.006 0.000 0.013 0.000 0.000 0.006 0.019

0.000 0.006 0.000 0.000 0.000 0.006 0.000 0.000 0.000 0.000

0.065 0.065 0.062 0.063 0.013 0.000 0.006 0.000 0.000 0.006

0.000 0.000 0.000 0.006 0.006 0.013 0.000 0.013 0.006 0.000

0.000 0.000 0.000 0.013 0.049 0.066 0.066 0.070 0.000 0.005

0.000 0.005 0.000 0.005 0.000 0.010 0.005 0.005 0.000 0.000

0.000 0.000 0.010 0.000 0.010 0.000 0.000 0.000 0.083 0.052

0.086 0.058

>Methylation code ID 1440

0.000 0.005 0.005 0.005 0.000 0.005 0.005 0.016 0.000 0.000

0.000 0.000 0.000 0.000 0.005 0.005 0.005 0.000 0.000 0.010

0.060 0.066 0.072 0.067 0.020 0.000 0.000 0.010 0.000 0.000

0.000 0.000 0.010 0.010 0.000 0.000 0.000 0.010 0.000 0.010

0.040 0.010 0.000 0.000 0.062 0.051 0.045 0.054 0.000 0.000

0.000 0.000 0.000 0.000 0.006 0.000 0.006 0.018 0.000 0.012

0.006 0.006 0.012 0.000 0.000 0.000 0.000 0.006 0.060 0.075

0.062 0.066

>Methylation code ID 1440

0.007 0.007 0.007 0.000 0.007 0.013 0.000 0.000 0.007 0.000

0.000 0.000 0.007 0.000 0.000 0.013 0.007 0.000 0.000 0.000

0.063 0.064 0.068 0.066 0.013 0.000 0.004 0.000 0.000 0.000

0.000 0.000 0.000 0.009 0.000 0.000 0.009 0.000 0.013 0.000

0.004 0.000 0.004 0.000 0.085 0.074 0.047 0.070 0.000 0.006

0.000 0.000 0.000 0.019 0.006 0.006 0.000 0.013 0.000 0.000

0.000 0.006 0.006 0.006 0.000 0.000 0.000 0.000 0.074 0.056

0.075 0.059

>Methylation code ID 1440

0.000 0.000 0.000 0.000 0.000 0.020 0.007 0.007 0.000 0.020

0.000 0.000 0.000 0.007 0.007 0.007 0.000 0.000 0.000 0.007

0.073 0.060 0.072 0.050 0.006 0.006 0.006 0.000 0.006 0.012

0.000 0.006 0.006 0.000 0.000 0.000 0.006 0.000 0.000 0.012

0.006 0.000 0.000 0.000 0.070 0.078 0.055 0.056 0.014 0.000

0.005 0.000 0.000 0.000 0.000 0.000 0.000 0.009 0.000 0.000

0.009 0.000 0.014 0.000 0.005 0.000 0.000 0.000 0.084 0.084

0.039 0.070

>Methylation code ID 1440

0.000 0.000 0.017 0.000 0.000 0.012 0.000 0.006 0.006 0.023

0.000 0.000 0.000 0.000 0.000 0.006 0.000 0.000 0.000 0.006

0.074 0.064 0.053 0.067 0.000 0.000 0.009 0.000 0.009 0.000

0.000 0.028 0.000 0.009 0.000 0.000 0.009 0.000 0.000 0.028

0.009 0.009 0.000 0.000 0.062 0.055 0.059 0.045 0.005 0.000

0.000 0.000 0.000 0.000 0.000 0.000 0.000 0.005 0.000 0.000

0.000 0.000 0.026 0.010 0.005 0.000 0.000 0.005 0.076 0.059

0.060 0.081

>Methylation code ID 1440

0.000 0.000 0.021 0.005 0.000 0.005 0.000 0.010 0.005 0.015

0.000 0.000 0.000 0.000 0.000 0.000 0.000 0.000 0.000 0.005

0.083 0.056 0.049 0.078 0.000 0.000 0.000 0.000 0.000 0.000

0.000 0.048 0.000 0.012 0.000 0.012 0.000 0.000 0.000 0.048

0.012 0.012 0.000 0.000 0.049 0.049 0.041 0.050 0.005 0.000

0.000 0.000 0.000 0.000 0.000 0.000 0.000 0.000 0.000 0.000

0.000 0.000 0.027 0.011 0.000 0.000 0.000 0.011 0.083 0.059

0.036 0.100

>Methylation code ID 1440

0.013 0.000 0.007 0.007 0.000 0.007 0.000 0.007 0.007 0.000

0.007 0.000 0.007 0.013 0.000 0.007 0.007 0.000 0.000 0.000

0.054 0.066 0.062 0.064 0.000 0.000 0.000 0.000 0.000 0.000

0.005 0.000 0.005 0.005 0.000 0.005 0.005 0.005 0.015 0.005

0.005 0.000 0.005 0.000 0.089 0.051 0.066 0.066 0.000 0.000

0.006 0.000 0.000 0.019 0.006 0.006 0.000 0.000 0.000 0.006

0.000 0.006 0.006 0.013 0.006 0.000 0.000 0.000 0.050 0.052

0.080 0.074

>Methylation code ID 1440

0.038 0.000 0.000 0.000 0.010 0.010 0.000 0.010 0.010 0.010

0.010 0.000 0.000 0.000 0.000 0.000 0.010 0.010 0.000 0.000

0.066 0.046 0.055 0.053 0.000 0.005 0.000 0.000 0.000 0.000

0.000 0.000 0.000 0.005 0.000 0.000 0.014 0.000 0.009 0.019

0.000 0.000 0.005 0.000 0.082 0.077 0.070 0.048 0.000 0.004

0.004 0.000 0.000 0.009 0.004 0.000 0.000 0.000 0.000 0.000

0.004 0.004 0.018 0.000 0.000 0.004 0.000 0.000 0.055 0.079

0.078 0.067

>Methylation code ID 1440

0.000 0.004 0.004 0.000 0.000 0.008 0.004 0.000 0.000 0.004

0.000 0.000 0.004 0.004 0.016 0.000 0.000 0.004 0.000 0.000

0.060 0.082 0.081 0.058 0.033 0.008 0.000 0.000 0.008 0.008

0.000 0.008 0.008 0.008 0.008 0.000 0.000 0.000 0.000 0.000

0.000 0.008 0.000 0.000 0.063 0.042 0.058 0.072 0.000 0.005

0.000 0.000 0.000 0.000 0.000 0.000 0.000 0.005 0.000 0.000

0.010 0.000 0.010 0.021 0.000 0.000 0.005 0.000 0.085 0.071

0.066 0.055

>Methylation code ID 1440

0.049 0.010 0.000 0.000 0.010 0.010 0.000 0.000 0.000 0.019

0.010 0.000 0.000 0.000 0.000 0.000 0.000 0.010 0.000 0.000

0.061 0.047 0.044 0.064 0.005 0.005 0.000 0.000 0.000 0.000

0.000 0.000 0.000 0.005 0.000 0.000 0.010 0.000 0.010 0.020

0.000 0.000 0.005 0.000 0.071 0.065 0.065 0.071 0.000 0.004

0.000 0.000 0.000 0.008 0.000 0.000 0.000 0.004 0.000 0.000

0.008 0.000 0.020 0.000 0.000 0.004 0.000 0.000 0.065 0.094

0.076 0.050

>Methylation code ID 1440

0.059 0.010 0.000 0.000 0.010 0.010 0.000 0.000 0.000 0.020

0.000 0.000 0.000 0.000 0.000 0.000 0.000 0.010 0.000 0.000

0.056 0.044 0.058 0.058 0.006 0.006 0.000 0.000 0.000 0.000

0.000 0.000 0.000 0.006 0.000 0.000 0.011 0.006 0.011 0.023

0.000 0.000 0.000 0.000 0.075 0.065 0.060 0.066 0.000 0.004

0.000 0.000 0.000 0.008 0.000 0.000 0.000 0.004 0.000 0.000

0.008 0.000 0.020 0.000 0.000 0.004 0.000 0.000 0.067 0.091

0.063 0.063

>Methylation code ID 1440

0.051 0.010 0.000 0.000 0.010 0.000 0.000 0.000 0.000 0.010

0.000 0.000 0.000 0.000 0.000 0.010 0.000 0.020 0.000 0.000

0.050 0.045 0.054 0.073 0.005 0.005 0.000 0.000 0.000 0.000

0.000 0.000 0.000 0.010 0.000 0.000 0.005 0.005 0.016 0.016

0.000 0.000 0.005 0.000 0.070 0.078 0.056 0.061 0.000 0.000

0.000 0.000 0.000 0.015 0.000 0.000 0.000 0.005 0.000 0.000

0.005 0.000 0.020 0.005 0.000 0.010 0.000 0.000 0.062 0.073

0.075 0.063

>Methylation code ID 1440

0.000 0.005 0.000 0.000 0.000 0.014 0.000 0.000 0.000 0.005

0.000 0.000 0.005 0.000 0.018 0.005 0.000 0.009 0.000 0.000

0.063 0.071 0.075 0.065 0.059 0.010 0.000 0.000 0.010 0.000

0.000 0.000 0.000 0.010 0.000 0.000 0.000 0.000 0.000 0.010

0.000 0.020 0.000 0.000 0.050 0.046 0.051 0.068 0.006 0.000

0.000 0.000 0.000 0.000 0.000 0.000 0.000 0.011 0.000 0.000

0.006 0.006 0.017 0.017 0.000 0.000 0.006 0.000 0.068 0.086

0.052 0.058

>Methylation code ID 1440

0.005 0.011 0.000 0.000 0.000 0.022 0.000 0.000 0.000 0.005

0.000 0.000 0.005 0.000 0.011 0.005 0.000 0.005 0.000 0.000

0.082 0.054 0.060 0.067 0.070 0.010 0.000 0.000 0.000 0.000

0.010 0.000 0.000 0.010 0.000 0.000 0.000 0.000 0.000 0.010

0.000 0.010 0.000 0.000 0.069 0.058 0.035 0.053 0.005 0.000

0.000 0.000 0.000 0.000 0.000 0.000 0.000 0.010 0.005 0.000

0.000 0.005 0.024 0.005 0.000 0.000 0.005 0.000 0.080 0.067

0.066 0.062

>Methylation code ID 1440

0.005 0.010 0.000 0.000 0.000 0.020 0.000 0.000 0.000 0.005

0.000 0.000 0.005 0.000 0.010 0.005 0.000 0.005 0.000 0.000

0.075 0.056 0.070 0.067 0.053 0.009 0.009 0.000 0.000 0.000

0.009 0.000 0.000 0.009 0.000 0.000 0.000 0.000 0.000 0.009

0.000 0.009 0.000 0.000 0.062 0.062 0.042 0.063 0.005 0.000

0.000 0.000 0.000 0.000 0.000 0.000 0.000 0.005 0.005 0.000

0.005 0.005 0.023 0.005 0.000 0.000 0.005 0.000 0.087 0.068

0.063 0.061

>Methylation code ID 1440

0.005 0.010 0.000 0.000 0.000 0.020 0.000 0.000 0.000 0.005

0.000 0.000 0.000 0.005 0.010 0.005 0.000 0.005 0.000 0.000

0.067 0.055 0.074 0.074 0.048 0.000 0.008 0.000 0.000 0.000

0.008 0.000 0.000 0.000 0.000 0.008 0.000 0.000 0.000 0.008

0.000 0.008 0.000 0.008 0.066 0.066 0.046 0.059 0.000 0.000

0.000 0.000 0.000 0.000 0.000 0.005 0.000 0.005 0.005 0.000

0.000 0.010 0.026 0.000 0.005 0.000 0.005 0.000 0.081 0.069

0.066 0.056

>Methylation code ID 1440

0.010 0.010 0.000 0.000 0.000 0.015 0.000 0.000 0.000 0.005

0.000 0.000 0.005 0.005 0.005 0.005 0.000 0.005 0.000 0.000

0.060 0.053 0.077 0.079 0.030 0.000 0.007 0.000 0.000 0.000

0.007 0.000 0.000 0.000 0.000 0.007 0.000 0.015 0.000 0.007

0.000 0.007 0.000 0.007 0.063 0.066 0.049 0.067 0.000 0.000

0.000 0.000 0.000 0.000 0.000 0.005 0.000 0.005 0.005 0.000

0.005 0.000 0.020 0.005 0.010 0.000 0.005 0.000 0.081 0.052

0.071 0.070

>Methylation code ID 1443

0.006 0.017 0.000 0.000 0.000 0.011 0.006 0.000 0.000 0.006

0.000 0.006 0.011 0.000 0.006 0.000 0.000 0.000 0.000 0.000

0.102 0.035 0.089 0.038 0.011 0.005 0.000 0.000 0.000 0.011

0.000 0.000 0.000 0.000 0.000 0.000 0.011 0.000 0.005 0.000

0.011 0.005 0.005 0.000 0.068 0.057 0.080 0.063 0.006 0.006

0.000 0.000 0.000 0.006 0.000 0.000 0.000 0.006 0.000 0.000

0.012 0.006 0.012 0.006 0.000 0.000 0.000 0.000 0.084 0.066

0.055 0.067

>Methylation code ID 1443

0.011 0.005 0.000 0.000 0.000 0.005 0.000 0.000 0.000 0.005

0.000 0.000 0.011 0.005 0.011 0.005 0.000 0.000 0.000 0.000

0.087 0.057 0.055 0.076 0.006 0.011 0.000 0.000 0.000 0.011

0.011 0.000 0.000 0.006 0.000 0.006 0.011 0.000 0.006 0.000

0.000 0.000 0.000 0.000 0.091 0.036 0.090 0.048 0.011 0.006

0.000 0.000 0.000 0.011 0.000 0.000 0.000 0.000 0.000 0.000

0.011 0.000 0.006 0.000 0.011 0.000 0.006 0.000 0.071 0.053

0.084 0.064

>Methylation code ID 1443

0.026 0.013 0.000 0.000 0.000 0.013 0.007 0.000 0.000 0.000

0.000 0.007 0.007 0.007 0.000 0.000 0.000 0.000 0.000 0.000

0.083 0.038 0.075 0.059 0.004 0.000 0.000 0.000 0.000 0.009

0.000 0.000 0.000 0.000 0.000 0.000 0.013 0.000 0.013 0.000

0.009 0.000 0.004 0.000 0.078 0.057 0.078 0.068 0.005 0.005

0.000 0.000 0.000 0.014 0.000 0.000 0.000 0.000 0.000 0.000

0.009 0.000 0.014 0.000 0.000 0.000 0.000 0.000 0.094 0.048

0.064 0.081

>Methylation code ID 1443

0.025 0.025 0.000 0.000 0.000 0.013 0.006 0.000 0.000 0.000

0.000 0.000 0.000 0.006 0.000 0.000 0.000 0.000 0.000 0.000

0.075 0.047 0.070 0.067 0.009 0.000 0.000 0.000 0.000 0.009

0.000 0.000 0.000 0.000 0.000 0.000 0.009 0.000 0.013 0.000

0.004 0.004 0.004 0.000 0.065 0.053 0.083 0.081 0.005 0.005

0.000 0.000 0.000 0.016 0.000 0.000 0.000 0.011 0.000 0.000

0.005 0.000 0.011 0.000 0.000 0.000 0.000 0.000 0.078 0.046

0.078 0.076

>Methylation code ID 1443

0.005 0.005 0.005 0.000 0.000 0.000 0.005 0.000 0.000 0.011

0.000 0.005 0.011 0.000 0.005 0.000 0.005 0.011 0.000 0.000

0.089 0.061 0.071 0.042 0.015 0.000 0.000 0.000 0.008 0.000

0.008 0.000 0.000 0.015 0.000 0.000 0.015 0.000 0.000 0.008

0.023 0.000 0.000 0.008 0.068 0.046 0.063 0.057 0.000 0.000

0.000 0.000 0.000 0.000 0.006 0.000 0.000 0.000 0.006 0.000

0.023 0.006 0.012 0.012 0.000 0.000 0.000 0.000 0.052 0.066

0.071 0.081

>Methylation code ID 1443

0.015 0.000 0.000 0.000 0.008 0.000 0.008 0.000 0.000 0.008

0.000 0.000 0.023 0.000 0.000 0.008 0.015 0.000 0.000 0.008

0.069 0.057 0.056 0.059 0.000 0.000 0.000 0.000 0.005 0.000

0.009 0.000 0.000 0.000 0.005 0.005 0.014 0.005 0.009 0.005

0.005 0.000 0.000 0.000 0.060 0.058 0.079 0.077 0.006 0.006

0.000 0.000 0.000 0.000 0.006 0.006 0.000 0.006 0.000 0.006

0.011 0.000 0.006 0.006 0.006 0.006 0.000 0.000 0.078 0.052

0.079 0.059

>Methylation code ID 1443

0.005 0.005 0.000 0.000 0.000 0.000 0.005 0.005 0.000 0.005

0.005 0.005 0.016 0.000 0.005 0.005 0.000 0.005 0.000 0.000

0.075 0.052 0.077 0.059 0.014 0.007 0.000 0.000 0.007 0.000

0.000 0.000 0.000 0.014 0.000 0.000 0.021 0.000 0.000 0.000

0.014 0.000 0.000 0.007 0.061 0.075 0.060 0.053 0.005 0.000

0.000 0.000 0.005 0.000 0.005 0.000 0.000 0.000 0.000 0.005

0.015 0.005 0.010 0.005 0.005 0.000 0.000 0.000 0.065 0.054

0.082 0.074

>Methylation code ID 1443

0.019 0.000 0.000 0.000 0.000 0.019 0.006 0.006 0.000 0.000

0.000 0.006 0.006 0.006 0.006 0.000 0.000 0.000 0.000 0.000

0.071 0.066 0.047 0.071 0.025 0.000 0.000 0.000 0.000 0.000

0.000 0.000 0.000 0.000 0.000 0.000 0.019 0.006 0.006 0.006

0.013 0.000 0.006 0.000 0.065 0.054 0.067 0.065 0.000 0.000

0.005 0.000 0.000 0.005 0.000 0.000 0.000 0.000 0.000 0.000

0.014 0.005 0.023 0.005 0.000 0.000 0.000 0.000 0.069 0.086

0.062 0.060

>Methylation code ID 1443

0.017 0.000 0.000 0.000 0.000 0.017 0.012 0.000 0.000 0.000

0.000 0.006 0.006 0.000 0.012 0.000 0.000 0.000 0.000 0.000

0.051 0.067 0.065 0.081 0.037 0.000 0.000 0.000 0.000 0.000

0.000 0.000 0.000 0.000 0.000 0.000 0.022 0.007 0.000 0.000

0.022 0.000 0.007 0.000 0.064 0.046 0.057 0.071 0.000 0.000

0.000 0.000 0.000 0.005 0.000 0.000 0.000 0.000 0.000 0.000

0.019 0.005 0.023 0.005 0.000 0.000 0.000 0.000 0.054 0.069

0.065 0.090

>Methylation code ID 1443

0.019 0.000 0.000 0.000 0.000 0.019 0.012 0.000 0.000 0.006

0.000 0.006 0.006 0.000 0.006 0.000 0.000 0.000 0.000 0.000

0.054 0.062 0.074 0.069 0.027 0.007 0.000 0.000 0.000 0.000

0.000 0.000 0.000 0.000 0.000 0.000 0.020 0.007 0.000 0.000

0.020 0.000 0.007 0.000 0.067 0.048 0.060 0.071 0.000 0.000

0.000 0.000 0.000 0.005 0.000 0.000 0.000 0.000 0.000 0.000

0.025 0.005 0.020 0.005 0.000 0.000 0.000 0.000 0.057 0.066

0.053 0.099

>Methylation code ID 1443

0.022 0.007 0.000 0.000 0.000 0.000 0.000 0.000 0.000 0.000

0.000 0.000 0.029 0.007 0.000 0.007 0.014 0.000 0.007 0.000

0.066 0.058 0.053 0.063 0.005 0.000 0.000 0.000 0.000 0.005

0.005 0.000 0.000 0.000 0.000 0.000 0.018 0.005 0.018 0.005

0.000 0.000 0.000 0.000 0.043 0.072 0.073 0.086 0.019 0.000

0.000 0.000 0.000 0.012 0.012 0.000 0.000 0.012 0.000 0.000

0.000 0.000 0.006 0.000 0.006 0.000 0.000 0.000 0.039 0.064

0.081 0.082

>Methylation code ID 1443

0.016 0.000 0.000 0.005 0.000 0.005 0.011 0.000 0.000 0.011

0.000 0.000 0.000 0.000 0.011 0.005 0.005 0.000 0.000 0.000

0.061 0.068 0.072 0.062 0.034 0.009 0.000 0.000 0.000 0.000

0.000 0.000 0.000 0.000 0.000 0.000 0.034 0.000 0.000 0.017

0.017 0.000 0.000 0.000 0.054 0.056 0.058 0.054 0.011 0.000

0.000 0.000 0.000 0.000 0.005 0.000 0.000 0.000 0.000 0.000

0.022 0.005 0.022 0.000 0.000 0.000 0.000 0.000 0.060 0.046

0.075 0.087

>Methylation code ID 1443

0.016 0.000 0.000 0.005 0.000 0.000 0.011 0.005 0.000 0.011

0.000 0.000 0.000 0.000 0.011 0.005 0.005 0.000 0.000 0.000

0.056 0.077 0.065 0.065 0.026 0.009 0.000 0.000 0.000 0.000

0.000 0.000 0.000 0.000 0.000 0.000 0.035 0.000 0.000 0.026

0.018 0.000 0.000 0.000 0.059 0.056 0.059 0.044 0.010 0.000

0.000 0.000 0.000 0.000 0.010 0.000 0.000 0.000 0.000 0.000

0.015 0.005 0.020 0.000 0.000 0.000 0.000 0.000 0.062 0.052

0.078 0.080

>Methylation code ID 1443

0.022 0.000 0.000 0.006 0.000 0.000 0.000 0.006 0.000 0.011

0.000 0.000 0.000 0.000 0.011 0.006 0.011 0.000 0.000 0.000

0.053 0.066 0.070 0.073 0.027 0.009 0.000 0.000 0.000 0.000

0.000 0.000 0.000 0.000 0.000 0.000 0.054 0.000 0.000 0.027

0.000 0.000 0.000 0.000 0.065 0.048 0.051 0.052 0.010 0.000

0.000 0.000 0.000 0.000 0.015 0.000 0.000 0.000 0.000 0.000

0.005 0.005 0.025 0.000 0.000 0.000 0.000 0.000 0.074 0.047

0.089 0.063

>Methylation code ID 1443

0.028 0.009 0.000 0.000 0.000 0.000 0.000 0.000 0.000 0.000

0.000 0.000 0.055 0.000 0.000 0.028 0.000 0.000 0.000 0.000

0.060 0.052 0.050 0.053 0.009 0.000 0.000 0.000 0.000 0.000

0.014 0.000 0.000 0.000 0.000 0.000 0.009 0.005 0.023 0.000

0.000 0.000 0.000 0.000 0.070 0.055 0.086 0.063 0.022 0.000

0.000 0.006 0.000 0.000 0.000 0.006 0.000 0.006 0.000 0.000

0.000 0.000 0.011 0.006 0.011 0.000 0.000 0.000 0.049 0.066

0.078 0.074

>Methylation code ID 1443

0.019 0.000 0.000 0.005 0.000 0.000 0.000 0.005 0.000 0.005

0.000 0.000 0.000 0.000 0.014 0.005 0.010 0.000 0.000 0.000

0.043 0.066 0.082 0.081 0.038 0.000 0.000 0.000 0.000 0.000

0.000 0.000 0.000 0.000 0.000 0.000 0.056 0.000 0.000 0.028

0.000 0.000 0.000 0.000 0.046 0.056 0.054 0.055 0.005 0.000

0.000 0.000 0.000 0.000 0.015 0.000 0.000 0.000 0.000 0.000

0.010 0.005 0.026 0.000 0.000 0.000 0.000 0.000 0.072 0.048

0.083 0.069

>Methylation code ID 1443

0.021 0.000 0.000 0.004 0.000 0.000 0.000 0.004 0.000 0.000

0.000 0.000 0.000 0.000 0.017 0.004 0.004 0.000 0.000 0.000

0.044 0.065 0.090 0.080 0.050 0.000 0.000 0.000 0.000 0.000

0.000 0.000 0.000 0.000 0.000 0.000 0.060 0.000 0.000 0.020

0.000 0.000 0.000 0.000 0.046 0.056 0.054 0.049 0.005 0.000

0.000 0.000 0.000 0.000 0.009 0.000 0.000 0.000 0.000 0.000

0.014 0.005 0.024 0.000 0.000 0.000 0.000 0.000 0.072 0.045

0.096 0.063

>Methylation code ID 1443

0.037 0.000 0.000 0.000 0.000 0.000 0.000 0.000 0.000 0.000

0.000 0.000 0.037 0.000 0.007 0.015 0.000 0.000 0.000 0.000

0.067 0.051 0.056 0.063 0.004 0.000 0.000 0.000 0.000 0.004

0.008 0.000 0.000 0.000 0.000 0.000 0.017 0.000 0.021 0.000

0.000 0.000 0.000 0.000 0.080 0.060 0.069 0.070 0.017 0.000

0.000 0.004 0.000 0.000 0.000 0.004 0.000 0.000 0.000 0.000

0.000 0.004 0.017 0.000 0.004 0.000 0.000 0.000 0.047 0.057

0.092 0.087

>Methylation code ID 1443

0.028 0.000 0.000 0.000 0.000 0.000 0.000 0.000 0.000 0.000

0.000 0.000 0.042 0.000 0.007 0.014 0.000 0.000 0.000 0.000

0.062 0.069 0.049 0.062 0.004 0.000 0.000 0.000 0.000 0.004

0.008 0.000 0.000 0.000 0.000 0.000 0.017 0.000 0.021 0.000

0.000 0.000 0.000 0.000 0.067 0.070 0.073 0.068 0.021 0.000

0.000 0.000 0.000 0.000 0.000 0.004 0.000 0.000 0.000 0.000

0.000 0.004 0.017 0.000 0.004 0.000 0.000 0.000 0.041 0.057

0.087 0.098

>Methylation code ID 1443

0.027 0.000 0.000 0.000 0.000 0.000 0.000 0.000 0.000 0.000

0.000 0.000 0.040 0.000 0.007 0.013 0.000 0.000 0.000 0.000

0.062 0.065 0.060 0.059 0.000 0.000 0.000 0.000 0.000 0.004

0.009 0.000 0.000 0.000 0.000 0.000 0.018 0.004 0.022 0.000

0.000 0.000 0.000 0.000 0.065 0.073 0.068 0.070 0.024 0.000

0.000 0.000 0.000 0.000 0.000 0.005 0.000 0.000 0.000 0.000

0.000 0.005 0.015 0.000 0.005 0.005 0.000 0.000 0.034 0.051

0.088 0.102

>Methylation code ID 1443

0.024 0.000 0.000 0.000 0.000 0.000 0.000 0.005 0.000 0.000

0.000 0.000 0.000 0.005 0.014 0.005 0.005 0.005 0.000 0.000

0.035 0.051 0.086 0.098 0.021 0.000 0.000 0.000 0.000 0.000

0.000 0.000 0.000 0.000 0.000 0.000 0.042 0.000 0.007 0.014

0.000 0.000 0.000 0.000 0.062 0.067 0.060 0.059 0.000 0.000

0.000 0.000 0.000 0.005 0.010 0.000 0.000 0.000 0.000 0.000

0.019 0.005 0.019 0.000 0.000 0.000 0.000 0.000 0.065 0.080

0.068 0.062

>Methylation code ID 1443

0.025 0.000 0.000 0.006 0.000 0.000 0.000 0.000 0.000 0.006

0.000 0.000 0.000 0.006 0.018 0.006 0.006 0.006 0.000 0.000

0.055 0.052 0.079 0.068 0.018 0.000 0.000 0.000 0.000 0.000

0.000 0.000 0.000 0.000 0.000 0.000 0.030 0.000 0.006 0.012

0.000 0.000 0.006 0.000 0.061 0.059 0.061 0.081 0.005 0.005

0.000 0.000 0.000 0.005 0.000 0.000 0.000 0.000 0.000 0.000

0.018 0.005 0.018 0.000 0.000 0.000 0.000 0.000 0.079 0.087

0.061 0.053

>Methylation code ID 1443

0.034 0.000 0.000 0.007 0.000 0.000 0.000 0.000 0.000 0.007

0.000 0.000 0.000 0.007 0.020 0.007 0.000 0.007 0.000 0.000

0.050 0.052 0.072 0.073 0.016 0.000 0.000 0.000 0.000 0.000

0.000 0.000 0.000 0.000 0.000 0.000 0.021 0.000 0.010 0.010

0.000 0.000 0.005 0.000 0.064 0.058 0.065 0.083 0.004 0.004

0.000 0.000 0.000 0.009 0.000 0.000 0.000 0.000 0.000 0.000

0.013 0.004 0.017 0.000 0.000 0.000 0.000 0.000 0.076 0.096

0.053 0.056

>Methylation code ID 1443

0.036 0.000 0.000 0.015 0.000 0.000 0.000 0.000 0.000 0.007

0.000 0.000 0.007 0.007 0.007 0.007 0.000 0.007 0.000 0.000

0.074 0.064 0.049 0.052 0.000 0.000 0.000 0.000 0.000 0.005

0.000 0.000 0.000 0.000 0.000 0.000 0.019 0.000 0.014 0.014

0.000 0.000 0.005 0.000 0.079 0.068 0.050 0.078 0.014 0.005

0.000 0.000 0.000 0.014 0.000 0.000 0.000 0.000 0.000 0.000

0.005 0.005 0.014 0.000 0.000 0.000 0.000 0.000 0.060 0.079

0.070 0.067

>Methylation code ID 1443

0.016 0.011 0.000 0.000 0.000 0.016 0.000 0.000 0.000 0.000

0.000 0.000 0.000 0.005 0.016 0.000 0.000 0.005 0.000 0.000

0.064 0.067 0.065 0.067 0.024 0.000 0.000 0.012 0.006 0.000

0.000 0.000 0.000 0.006 0.000 0.000 0.006 0.000 0.006 0.006

0.000 0.006 0.000 0.000 0.062 0.078 0.064 0.057 0.000 0.005

0.000 0.000 0.000 0.005 0.000 0.000 0.000 0.005 0.000 0.000

0.010 0.000 0.010 0.014 0.000 0.000 0.005 0.000 0.075 0.072

0.053 0.080

>Methylation code ID 1443

0.000 0.004 0.000 0.000 0.000 0.004 0.000 0.000 0.000 0.004

0.000 0.000 0.004 0.000 0.013 0.017 0.000 0.000 0.004 0.000

0.077 0.083 0.064 0.059 0.019 0.013 0.000 0.000 0.000 0.025

0.000 0.000 0.000 0.000 0.000 0.000 0.006 0.006 0.006 0.000

0.000 0.006 0.000 0.000 0.076 0.050 0.066 0.060 0.018 0.000

0.000 0.012 0.006 0.000 0.000 0.000 0.000 0.006 0.000 0.000

0.006 0.006 0.006 0.006 0.000 0.000 0.000 0.000 0.064 0.081

0.064 0.059

>Methylation code ID 1443

0.006 0.006 0.000 0.006 0.000 0.024 0.000 0.000 0.006 0.000

0.000 0.000 0.006 0.000 0.012 0.012 0.000 0.000 0.000 0.000

0.062 0.056 0.054 0.085 0.007 0.000 0.000 0.015 0.000 0.022

0.000 0.000 0.000 0.007 0.000 0.000 0.000 0.007 0.015 0.000

0.000 0.007 0.000 0.000 0.071 0.049 0.057 0.074 0.006 0.000

0.000 0.011 0.000 0.006 0.000 0.000 0.011 0.006 0.000 0.000

0.000 0.000 0.006 0.006 0.000 0.000 0.011 0.000 0.063 0.066

0.058 0.083

>Methylation code ID 1443

0.000 0.000 0.000 0.000 0.000 0.028 0.000 0.000 0.014 0.007

0.000 0.000 0.000 0.007 0.007 0.007 0.000 0.000 0.007 0.000

0.059 0.071 0.055 0.070 0.013 0.000 0.000 0.000 0.000 0.025

0.000 0.000 0.006 0.006 0.000 0.000 0.000 0.000 0.006 0.006

0.000 0.006 0.013 0.000 0.063 0.055 0.051 0.082 0.006 0.006

0.000 0.018 0.000 0.018 0.000 0.000 0.000 0.000 0.000 0.000

0.006 0.000 0.006 0.006 0.000 0.000 0.000 0.000 0.055 0.079

0.073 0.060

>Methylation code ID 1443

0.012 0.000 0.000 0.000 0.000 0.018 0.000 0.000 0.006 0.006

0.000 0.000 0.000 0.000 0.006 0.006 0.000 0.006 0.018 0.000

0.060 0.068 0.057 0.072 0.006 0.006 0.000 0.017 0.000 0.017

0.000 0.000 0.000 0.000 0.000 0.000 0.006 0.000 0.006 0.012

0.000 0.000 0.000 0.000 0.058 0.081 0.065 0.060 0.000 0.000

0.000 0.000 0.000 0.022 0.000 0.000 0.014 0.007 0.000 0.000

0.000 0.007 0.007 0.007 0.000 0.000 0.007 0.000 0.061 0.070

0.058 0.072

>Methylation code ID 1443

0.011 0.000 0.000 0.022 0.000 0.067 0.000 0.000 0.000 0.011

0.000 0.000 0.011 0.000 0.000 0.011 0.000 0.011 0.000 0.000

0.040 0.048 0.045 0.056 0.016 0.000 0.000 0.000 0.000 0.010

0.000 0.000 0.010 0.005 0.000 0.000 0.000 0.000 0.010 0.000

0.000 0.005 0.005 0.000 0.053 0.081 0.063 0.075 0.004 0.000

0.000 0.000 0.000 0.012 0.000 0.000 0.004 0.000 0.000 0.000

0.000 0.000 0.016 0.004 0.000 0.000 0.008 0.000 0.066 0.090

0.067 0.063

>Methylation code ID 1443

0.007 0.000 0.000 0.000 0.000 0.051 0.000 0.000 0.000 0.007

0.000 0.000 0.007 0.000 0.015 0.000 0.000 0.007 0.000 0.000

0.078 0.035 0.062 0.064 0.016 0.000 0.005 0.000 0.000 0.016

0.000 0.000 0.000 0.005 0.000 0.000 0.000 0.000 0.011 0.000

0.000 0.005 0.005 0.000 0.052 0.082 0.069 0.067 0.010 0.000

0.000 0.000 0.000 0.020 0.000 0.000 0.000 0.000 0.000 0.000

0.000 0.000 0.015 0.005 0.005 0.000 0.005 0.000 0.065 0.084

0.065 0.061

>Methylation code ID 1443

0.009 0.000 0.000 0.000 0.000 0.019 0.000 0.005 0.000 0.000

0.000 0.000 0.000 0.000 0.014 0.000 0.005 0.000 0.009 0.000

0.069 0.082 0.062 0.061 0.006 0.000 0.000 0.000 0.006 0.040

0.000 0.000 0.000 0.000 0.000 0.000 0.006 0.000 0.011 0.000

0.000 0.006 0.000 0.000 0.077 0.042 0.073 0.066 0.016 0.000

0.011 0.000 0.000 0.016 0.000 0.000 0.000 0.005 0.000 0.000

0.000 0.000 0.011 0.000 0.000 0.000 0.000 0.000 0.040 0.084

0.077 0.073

>Methylation code ID 1443

0.010 0.000 0.010 0.000 0.000 0.015 0.000 0.000 0.000 0.005

0.000 0.000 0.000 0.000 0.010 0.005 0.005 0.000 0.000 0.000

0.042 0.085 0.076 0.070 0.009 0.000 0.000 0.000 0.000 0.013

0.000 0.004 0.000 0.000 0.000 0.000 0.000 0.000 0.017 0.000

0.004 0.000 0.009 0.000 0.064 0.076 0.057 0.081 0.005 0.000

0.005 0.000 0.005 0.025 0.000 0.000 0.000 0.000 0.000 0.000

0.005 0.000 0.010 0.000 0.000 0.005 0.000 0.000 0.080 0.044

0.079 0.070

>Methylation code ID 1443

0.005 0.000 0.005 0.000 0.005 0.023 0.000 0.005 0.000 0.000

0.000 0.000 0.005 0.000 0.009 0.005 0.000 0.000 0.000 0.000

0.083 0.052 0.064 0.073 0.000 0.000 0.012 0.000 0.000 0.018

0.000 0.000 0.000 0.006 0.000 0.000 0.000 0.000 0.012 0.006

0.006 0.006 0.000 0.000 0.066 0.069 0.064 0.066 0.004 0.000

0.004 0.000 0.004 0.012 0.000 0.004 0.000 0.000 0.000 0.000

0.000 0.000 0.012 0.000 0.004 0.000 0.004 0.000 0.054 0.070

0.064 0.097

>Methylation code ID 1443

0.004 0.000 0.004 0.000 0.004 0.022 0.000 0.004 0.000 0.000

0.000 0.000 0.000 0.000 0.013 0.004 0.000 0.000 0.000 0.000

0.087 0.045 0.071 0.072 0.000 0.000 0.015 0.000 0.000 0.023

0.000 0.000 0.000 0.008 0.000 0.000 0.000 0.000 0.008 0.008

0.008 0.008 0.000 0.000 0.063 0.066 0.063 0.065 0.004 0.000

0.004 0.000 0.004 0.008 0.000 0.004 0.000 0.000 0.000 0.000

0.000 0.000 0.012 0.004 0.004 0.000 0.004 0.000 0.054 0.073

0.069 0.089

>Methylation code ID 1443

0.000 0.003 0.003 0.000 0.003 0.003 0.000 0.003 0.000 0.003

0.000 0.000 0.000 0.000 0.013 0.007 0.000 0.000 0.003 0.000

0.066 0.073 0.070 0.081 0.005 0.005 0.005 0.000 0.005 0.016

0.000 0.005 0.000 0.000 0.000 0.000 0.005 0.000 0.005 0.005

0.000 0.011 0.000 0.000 0.070 0.064 0.061 0.069 0.000 0.000

0.007 0.000 0.007 0.007 0.000 0.000 0.000 0.007 0.000 0.000

0.000 0.000 0.007 0.014 0.007 0.014 0.000 0.000 0.064 0.053

0.068 0.077

>Methylation code ID 1443

0.000 0.000 0.000 0.000 0.008 0.008 0.000 0.000 0.000 0.024

0.000 0.000 0.000 0.000 0.008 0.016 0.008 0.016 0.000 0.000

0.063 0.052 0.065 0.067 0.000 0.004 0.004 0.000 0.007 0.004

0.000 0.000 0.000 0.004 0.000 0.000 0.000 0.000 0.014 0.007

0.000 0.000 0.004 0.000 0.063 0.063 0.072 0.088 0.006 0.006

0.006 0.000 0.006 0.011 0.000 0.006 0.000 0.000 0.000 0.000

0.006 0.000 0.006 0.006 0.000 0.011 0.000 0.000 0.064 0.069

0.066 0.066

>Methylation code ID 1443

0.005 0.005 0.000 0.000 0.005 0.016 0.000 0.005 0.000 0.000

0.000 0.000 0.005 0.000 0.005 0.011 0.000 0.011 0.000 0.000

0.061 0.066 0.069 0.068 0.008 0.000 0.000 0.000 0.008 0.008

0.008 0.000 0.000 0.023 0.000 0.000 0.000 0.000 0.008 0.008

0.000 0.015 0.000 0.000 0.071 0.058 0.055 0.065 0.000 0.004

0.004 0.000 0.008 0.004 0.000 0.000 0.000 0.008 0.000 0.000

0.000 0.000 0.008 0.008 0.000 0.000 0.004 0.000 0.068 0.067

0.073 0.075

>Methylation code ID 1443

0.014 0.014 0.000 0.000 0.000 0.000 0.007 0.000 0.000 0.014

0.000 0.000 0.014 0.000 0.000 0.000 0.014 0.014 0.000 0.000

0.067 0.052 0.062 0.062 0.007 0.000 0.000 0.000 0.007 0.000

0.000 0.000 0.000 0.015 0.000 0.000 0.015 0.007 0.007 0.015

0.007 0.000 0.007 0.000 0.059 0.054 0.069 0.065 0.000 0.005

0.000 0.000 0.005 0.005 0.005 0.000 0.000 0.010 0.000 0.005

0.015 0.000 0.005 0.005 0.000 0.000 0.000 0.000 0.083 0.058

0.066 0.066

>Methylation code ID 1445

0.000 0.000 0.000 0.000 0.000 0.014 0.000 0.007 0.000 0.014

0.007 0.007 0.000 0.000 0.021 0.000 0.000 0.014 0.000 0.000

0.061 0.039 0.076 0.073 0.017 0.017 0.006 0.006 0.000 0.000

0.000 0.000 0.000 0.006 0.000 0.000 0.000 0.000 0.000 0.006

0.006 0.000 0.006 0.000 0.052 0.073 0.068 0.072 0.011 0.005

0.000 0.000 0.000 0.005 0.005 0.000 0.000 0.000 0.000 0.005

0.011 0.005 0.005 0.005 0.000 0.005 0.000 0.000 0.079 0.042

0.086 0.062

>Methylation code ID 1445

0.024 0.018 0.006 0.000 0.000 0.000 0.000 0.000 0.000 0.006

0.000 0.000 0.000 0.000 0.000 0.006 0.006 0.000 0.006 0.000

0.054 0.070 0.059 0.077 0.010 0.005 0.000 0.000 0.000 0.005

0.005 0.000 0.000 0.000 0.000 0.005 0.015 0.005 0.005 0.000

0.005 0.005 0.000 0.000 0.082 0.038 0.087 0.059 0.000 0.000

0.000 0.000 0.000 0.007 0.000 0.007 0.000 0.014 0.007 0.007

0.000 0.000 0.027 0.000 0.000 0.014 0.000 0.000 0.056 0.040

0.072 0.085

>Methylation code ID 1445

0.037 0.007 0.007 0.000 0.000 0.000 0.000 0.000 0.000 0.007

0.000 0.000 0.000 0.000 0.000 0.007 0.015 0.000 0.007 0.000

0.054 0.059 0.057 0.074 0.006 0.006 0.000 0.000 0.000 0.006

0.006 0.000 0.000 0.006 0.000 0.006 0.022 0.006 0.006 0.000

0.006 0.000 0.000 0.000 0.096 0.035 0.090 0.040 0.000 0.005

0.000 0.000 0.000 0.005 0.005 0.005 0.000 0.000 0.005 0.005

0.000 0.000 0.020 0.000 0.000 0.010 0.000 0.000 0.055 0.056

0.078 0.084

>Methylation code ID 1445

0.038 0.006 0.006 0.000 0.000 0.000 0.000 0.000 0.000 0.006

0.000 0.000 0.000 0.000 0.006 0.006 0.013 0.000 0.000 0.000

0.059 0.061 0.049 0.082 0.005 0.005 0.000 0.000 0.000 0.005

0.005 0.000 0.000 0.005 0.000 0.000 0.021 0.005 0.011 0.000

0.005 0.000 0.000 0.000 0.101 0.040 0.078 0.046 0.000 0.005

0.000 0.005 0.000 0.005 0.005 0.000 0.000 0.000 0.005 0.005

0.000 0.000 0.023 0.000 0.000 0.005 0.000 0.000 0.077 0.065

0.063 0.072

>Methylation code ID 1445

0.038 0.006 0.006 0.000 0.000 0.000 0.000 0.000 0.000 0.000

0.000 0.000 0.000 0.006 0.006 0.006 0.013 0.000 0.000 0.000

0.063 0.065 0.053 0.071 0.005 0.000 0.000 0.000 0.000 0.005

0.005 0.000 0.005 0.005 0.000 0.000 0.021 0.005 0.011 0.000

0.005 0.000 0.000 0.000 0.091 0.040 0.082 0.050 0.005 0.005

0.000 0.005 0.000 0.005 0.005 0.000 0.000 0.000 0.005 0.005

0.000 0.000 0.023 0.000 0.000 0.000 0.000 0.000 0.080 0.064

0.069 0.065

>Methylation code ID 1445

0.036 0.006 0.006 0.000 0.000 0.000 0.000 0.000 0.000 0.000

0.000 0.000 0.000 0.006 0.006 0.000 0.018 0.000 0.000 0.000

0.059 0.069 0.054 0.074 0.006 0.000 0.000 0.000 0.000 0.006

0.000 0.000 0.006 0.006 0.000 0.000 0.017 0.006 0.022 0.000

0.006 0.000 0.000 0.000 0.067 0.045 0.072 0.078 0.005 0.005

0.005 0.005 0.000 0.005 0.005 0.000 0.000 0.000 0.000 0.005

0.000 0.000 0.020 0.005 0.000 0.000 0.000 0.000 0.065 0.073

0.054 0.081

>Methylation code ID 1445

0.005 0.005 0.005 0.010 0.000 0.005 0.005 0.000 0.000 0.000

0.000 0.005 0.000 0.000 0.019 0.005 0.000 0.000 0.000 0.000

0.067 0.070 0.054 0.081 0.037 0.000 0.006 0.000 0.000 0.000

0.000 0.000 0.000 0.000 0.000 0.006 0.000 0.006 0.006 0.000

0.018 0.000 0.000 0.000 0.061 0.066 0.056 0.071 0.000 0.000

0.000 0.000 0.000 0.006 0.000 0.000 0.006 0.006 0.000 0.000

0.017 0.006 0.023 0.000 0.006 0.000 0.000 0.000 0.066 0.044

0.074 0.080

>Methylation code ID 1445

0.004 0.004 0.004 0.013 0.000 0.004 0.009 0.000 0.000 0.000

0.000 0.000 0.000 0.000 0.013 0.004 0.000 0.000 0.000 0.000

0.076 0.078 0.047 0.076 0.035 0.000 0.000 0.000 0.000 0.000

0.000 0.000 0.000 0.000 0.000 0.006 0.000 0.006 0.012 0.000

0.018 0.000 0.000 0.000 0.065 0.067 0.048 0.077 0.000 0.000

0.000 0.000 0.000 0.005 0.000 0.000 0.005 0.005 0.000 0.000

0.014 0.000 0.024 0.000 0.005 0.000 0.000 0.000 0.066 0.066

0.070 0.073

>Methylation code ID 1445

0.004 0.004 0.004 0.013 0.000 0.009 0.009 0.000 0.000 0.000

0.000 0.000 0.000 0.000 0.009 0.004 0.000 0.000 0.000 0.000

0.075 0.073 0.055 0.072 0.035 0.000 0.000 0.000 0.000 0.000

0.000 0.000 0.000 0.000 0.000 0.006 0.000 0.006 0.012 0.000

0.017 0.000 0.000 0.000 0.077 0.067 0.050 0.065 0.000 0.000

0.000 0.000 0.000 0.009 0.000 0.000 0.005 0.005 0.000 0.000

0.009 0.000 0.023 0.000 0.005 0.000 0.000 0.000 0.072 0.068

0.056 0.082

>Methylation code ID 1445

0.005 0.005 0.005 0.014 0.000 0.009 0.005 0.000 0.000 0.000

0.000 0.000 0.000 0.000 0.009 0.005 0.005 0.000 0.000 0.000

0.073 0.073 0.059 0.069 0.034 0.000 0.000 0.000 0.000 0.000

0.000 0.000 0.000 0.000 0.000 0.006 0.006 0.006 0.011 0.000

0.011 0.000 0.000 0.000 0.075 0.077 0.040 0.068 0.000 0.000

0.000 0.000 0.000 0.009 0.005 0.000 0.005 0.000 0.000 0.000

0.009 0.000 0.024 0.000 0.005 0.000 0.000 0.000 0.077 0.062

0.062 0.076

>Methylation code ID 1445

0.022 0.000 0.000 0.000 0.000 0.000 0.000 0.005 0.000 0.005

0.000 0.005 0.005 0.005 0.011 0.000 0.011 0.000 0.000 0.000

0.063 0.061 0.054 0.084 0.000 0.000 0.000 0.000 0.000 0.010

0.005 0.000 0.005 0.000 0.000 0.000 0.010 0.000 0.020 0.005

0.005 0.000 0.000 0.000 0.065 0.067 0.069 0.074 0.006 0.000

0.006 0.011 0.000 0.011 0.011 0.000 0.000 0.006 0.000 0.000

0.000 0.000 0.006 0.006 0.006 0.000 0.000 0.000 0.062 0.072

0.064 0.069

>Methylation code ID 1445

0.005 0.000 0.010 0.010 0.000 0.010 0.010 0.000 0.000 0.005

0.000 0.000 0.000 0.000 0.005 0.005 0.005 0.000 0.000 0.000

0.065 0.070 0.061 0.070 0.025 0.000 0.000 0.000 0.000 0.000

0.000 0.006 0.000 0.006 0.006 0.006 0.006 0.006 0.006 0.000

0.012 0.000 0.000 0.000 0.065 0.053 0.057 0.078 0.000 0.000

0.000 0.000 0.000 0.006 0.006 0.000 0.006 0.000 0.000 0.000

0.012 0.000 0.023 0.006 0.006 0.000 0.000 0.000 0.069 0.064

0.065 0.070

>Methylation code ID 1445

0.018 0.009 0.018 0.000 0.000 0.009 0.000 0.000 0.000 0.009

0.009 0.000 0.009 0.000 0.000 0.018 0.000 0.009 0.000 0.000

0.044 0.059 0.054 0.070 0.006 0.000 0.000 0.006 0.006 0.000

0.000 0.012 0.000 0.006 0.000 0.000 0.018 0.000 0.006 0.000

0.000 0.000 0.006 0.000 0.061 0.064 0.072 0.070 0.000 0.005

0.005 0.000 0.005 0.005 0.000 0.000 0.000 0.010 0.000 0.000

0.005 0.000 0.010 0.005 0.000 0.005 0.000 0.000 0.069 0.073

0.086 0.049

>Methylation code ID 1445

0.014 0.000 0.007 0.000 0.000 0.000 0.000 0.014 0.000 0.021

0.000 0.007 0.021 0.000 0.000 0.000 0.007 0.000 0.000 0.000

0.058 0.060 0.057 0.067 0.000 0.000 0.000 0.000 0.000 0.000

0.000 0.006 0.000 0.006 0.000 0.000 0.012 0.012 0.012 0.006

0.006 0.000 0.006 0.000 0.067 0.068 0.071 0.063 0.000 0.005

0.016 0.000 0.000 0.000 0.000 0.000 0.005 0.005 0.000 0.000

0.011 0.000 0.005 0.011 0.000 0.005 0.000 0.000 0.056 0.071

0.058 0.085

>Methylation code ID 1445

0.000 0.000 0.005 0.000 0.000 0.005 0.000 0.005 0.005 0.010

0.000 0.000 0.005 0.000 0.010 0.010 0.000 0.005 0.000 0.005

0.064 0.069 0.059 0.074 0.014 0.000 0.014 0.000 0.007 0.000

0.000 0.007 0.000 0.014 0.000 0.007 0.014 0.000 0.000 0.000

0.007 0.000 0.000 0.000 0.081 0.058 0.048 0.061 0.000 0.000

0.000 0.000 0.000 0.000 0.000 0.012 0.000 0.000 0.000 0.006

0.012 0.012 0.006 0.006 0.006 0.000 0.006 0.000 0.065 0.082

0.074 0.047

>Methylation code ID 1445

0.013 0.000 0.013 0.000 0.006 0.000 0.000 0.013 0.006 0.019

0.000 0.000 0.000 0.000 0.000 0.000 0.006 0.000 0.000 0.000

0.073 0.052 0.064 0.068 0.000 0.000 0.000 0.000 0.000 0.000

0.000 0.014 0.000 0.007 0.000 0.007 0.007 0.007 0.007 0.029

0.000 0.000 0.007 0.000 0.075 0.066 0.045 0.060 0.005 0.000

0.005 0.000 0.000 0.005 0.000 0.005 0.000 0.005 0.000 0.000

0.000 0.005 0.011 0.005 0.000 0.005 0.000 0.011 0.060 0.063

0.063 0.082

>Methylation code ID 1445

0.000 0.000 0.012 0.006 0.000 0.000 0.000 0.024 0.012 0.006

0.000 0.000 0.000 0.000 0.000 0.000 0.006 0.006 0.000 0.000

0.074 0.071 0.039 0.078 0.000 0.000 0.000 0.000 0.000 0.000

0.000 0.014 0.000 0.007 0.000 0.000 0.007 0.000 0.014 0.041

0.000 0.000 0.000 0.000 0.072 0.038 0.072 0.068 0.005 0.000

0.009 0.000 0.000 0.000 0.000 0.000 0.005 0.005 0.000 0.000

0.000 0.005 0.014 0.000 0.000 0.000 0.000 0.014 0.070 0.064

0.067 0.076

>Methylation code ID 1445

0.000 0.000 0.007 0.007 0.000 0.000 0.000 0.026 0.013 0.007

0.000 0.000 0.000 0.000 0.000 0.007 0.007 0.007 0.000 0.000

0.075 0.071 0.034 0.075 0.000 0.000 0.000 0.000 0.000 0.000

0.000 0.006 0.000 0.006 0.000 0.000 0.012 0.000 0.012 0.037

0.000 0.000 0.000 0.000 0.066 0.047 0.070 0.077 0.005 0.000

0.009 0.000 0.000 0.000 0.000 0.000 0.005 0.005 0.000 0.000

0.000 0.005 0.014 0.000 0.000 0.005 0.000 0.009 0.075 0.065

0.067 0.071

>Methylation code ID 1445

0.006 0.000 0.006 0.012 0.000 0.006 0.006 0.012 0.006 0.000

0.000 0.000 0.006 0.000 0.000 0.006 0.000 0.006 0.000 0.000

0.066 0.071 0.062 0.060 0.000 0.000 0.000 0.005 0.000 0.000

0.000 0.005 0.000 0.000 0.000 0.000 0.009 0.000 0.019 0.014

0.005 0.000 0.000 0.000 0.070 0.060 0.075 0.072 0.004 0.000

0.009 0.000 0.000 0.004 0.000 0.000 0.004 0.004 0.000 0.000

0.000 0.000 0.018 0.004 0.000 0.004 0.000 0.000 0.076 0.064

0.049 0.092

>Methylation code ID 1445

0.007 0.000 0.007 0.007 0.000 0.007 0.007 0.007 0.007 0.007

0.000 0.000 0.007 0.007 0.000 0.007 0.000 0.000 0.000 0.000

0.067 0.064 0.071 0.057 0.000 0.008 0.000 0.004 0.000 0.000

0.000 0.004 0.000 0.000 0.000 0.000 0.008 0.000 0.016 0.004

0.004 0.000 0.000 0.000 0.069 0.080 0.063 0.073 0.010 0.000

0.005 0.000 0.000 0.010 0.000 0.000 0.005 0.005 0.000 0.000

0.000 0.000 0.015 0.005 0.000 0.005 0.000 0.000 0.064 0.056

0.052 0.099

>Methylation code ID 1445

0.016 0.000 0.000 0.000 0.000 0.011 0.005 0.000 0.005 0.005

0.000 0.000 0.000 0.000 0.016 0.005 0.000 0.005 0.000 0.000

0.066 0.056 0.050 0.092 0.007 0.000 0.007 0.007 0.000 0.007

0.007 0.000 0.007 0.007 0.000 0.000 0.014 0.007 0.000 0.007

0.007 0.000 0.000 0.000 0.066 0.066 0.068 0.052 0.000 0.009

0.000 0.004 0.000 0.000 0.000 0.004 0.000 0.000 0.000 0.000

0.013 0.000 0.013 0.004 0.004 0.000 0.000 0.000 0.070 0.088

0.060 0.062

>Methylation code ID 1445

0.006 0.000 0.000 0.000 0.000 0.006 0.000 0.006 0.006 0.006

0.000 0.000 0.000 0.006 0.011 0.011 0.006 0.000 0.000 0.006

0.077 0.077 0.037 0.075 0.016 0.000 0.000 0.008 0.000 0.008

0.000 0.008 0.000 0.016 0.000 0.008 0.000 0.016 0.000 0.016

0.000 0.000 0.000 0.008 0.058 0.046 0.069 0.057 0.005 0.000

0.000 0.000 0.000 0.000 0.000 0.005 0.010 0.005 0.000 0.000

0.000 0.005 0.010 0.000 0.005 0.005 0.000 0.005 0.037 0.069

0.090 0.081

>Methylation code ID 1445

0.014 0.014 0.000 0.000 0.007 0.000 0.000 0.007 0.007 0.021

0.000 0.000 0.007 0.000 0.000 0.000 0.000 0.000 0.000 0.000

0.052 0.081 0.063 0.060 0.013 0.007 0.000 0.000 0.007 0.000

0.000 0.000 0.000 0.013 0.000 0.007 0.007 0.007 0.000 0.020

0.000 0.000 0.000 0.007 0.058 0.060 0.069 0.061 0.000 0.000

0.000 0.000 0.000 0.000 0.007 0.014 0.000 0.014 0.000 0.000

0.007 0.000 0.007 0.014 0.000 0.014 0.000 0.000 0.063 0.065

0.051 0.076

>Methylation code ID 1445

0.005 0.005 0.000 0.005 0.005 0.000 0.000 0.000 0.000 0.005

0.000 0.000 0.000 0.000 0.015 0.010 0.000 0.000 0.000 0.000

0.085 0.069 0.075 0.053 0.010 0.010 0.005 0.005 0.000 0.000

0.000 0.000 0.010 0.000 0.000 0.000 0.010 0.000 0.000 0.010

0.000 0.005 0.000 0.000 0.075 0.072 0.049 0.072 0.000 0.000

0.000 0.000 0.000 0.000 0.000 0.000 0.007 0.007 0.000 0.000

0.013 0.007 0.007 0.013 0.007 0.020 0.000 0.000 0.074 0.074

0.041 0.064

>Methylation code ID 1445

0.000 0.009 0.000 0.005 0.000 0.005 0.005 0.000 0.009 0.000

0.000 0.000 0.009 0.000 0.005 0.005 0.000 0.009 0.000 0.000

0.077 0.074 0.055 0.068 0.000 0.000 0.000 0.000 0.000 0.007

0.000 0.000 0.007 0.000 0.007 0.000 0.013 0.000 0.007 0.013

0.000 0.020 0.000 0.007 0.077 0.076 0.040 0.063 0.009 0.005

0.000 0.005 0.005 0.000 0.000 0.000 0.000 0.000 0.000 0.000

0.005 0.000 0.014 0.000 0.005 0.000 0.000 0.000 0.087 0.070

0.038 0.092

>Methylation code ID 1445

0.004 0.000 0.000 0.000 0.009 0.004 0.009 0.000 0.004 0.000

0.000 0.004 0.004 0.004 0.004 0.004 0.000 0.000 0.004 0.000

0.051 0.075 0.080 0.071 0.012 0.000 0.000 0.000 0.006 0.006

0.006 0.012 0.012 0.000 0.006 0.000 0.006 0.000 0.006 0.006

0.000 0.000 0.000 0.000 0.066 0.085 0.060 0.044 0.005 0.000

0.000 0.000 0.009 0.000 0.000 0.000 0.009 0.000 0.005 0.000

0.009 0.000 0.005 0.005 0.000 0.005 0.005 0.000 0.068 0.067

0.071 0.072

>Methylation code ID 1445

0.013 0.000 0.000 0.000 0.000 0.013 0.007 0.013 0.013 0.000

0.007 0.000 0.007 0.000 0.000 0.007 0.000 0.000 0.007 0.000

0.060 0.083 0.055 0.050 0.004 0.000 0.000 0.000 0.004 0.008

0.000 0.000 0.008 0.000 0.004 0.000 0.008 0.000 0.008 0.004

0.000 0.000 0.004 0.000 0.071 0.073 0.065 0.071 0.005 0.000

0.000 0.000 0.000 0.010 0.010 0.000 0.005 0.000 0.000 0.005

0.000 0.005 0.005 0.005 0.000 0.000 0.005 0.000 0.047 0.072

0.080 0.078

>Methylation code ID 1445

0.010 0.005 0.005 0.000 0.000 0.010 0.000 0.010 0.000 0.005

0.005 0.000 0.000 0.000 0.005 0.000 0.000 0.005 0.005 0.000

0.073 0.060 0.061 0.077 0.005 0.000 0.005 0.000 0.000 0.011

0.000 0.000 0.000 0.000 0.000 0.000 0.005 0.000 0.005 0.005

0.005 0.000 0.005 0.011 0.070 0.073 0.074 0.057 0.000 0.000

0.000 0.000 0.000 0.017 0.017 0.006 0.000 0.000 0.006 0.000

0.000 0.000 0.006 0.000 0.006 0.000 0.006 0.000 0.049 0.069

0.088 0.066

>Methylation code ID 1445

0.005 0.000 0.005 0.000 0.000 0.010 0.000 0.000 0.000 0.000

0.000 0.000 0.000 0.000 0.010 0.005 0.005 0.000 0.005 0.010

0.076 0.070 0.067 0.063 0.000 0.000 0.000 0.000 0.000 0.016

0.011 0.005 0.000 0.000 0.005 0.005 0.000 0.000 0.005 0.000

0.005 0.005 0.005 0.000 0.056 0.069 0.082 0.062 0.005 0.005

0.005 0.000 0.000 0.009 0.000 0.005 0.000 0.005 0.005 0.000

0.000 0.000 0.005 0.000 0.005 0.005 0.005 0.000 0.073 0.064

0.062 0.078

>Methylation code ID 1447

0.006 0.000 0.006 0.000 0.000 0.000 0.000 0.000 0.006 0.000

0.000 0.006 0.000 0.006 0.013 0.006 0.019 0.000 0.000 0.006

0.073 0.074 0.056 0.054 0.005 0.000 0.000 0.011 0.000 0.005

0.000 0.005 0.005 0.000 0.000 0.000 0.011 0.005 0.005 0.005

0.011 0.000 0.000 0.000 0.071 0.057 0.060 0.074 0.000 0.000

0.006 0.006 0.000 0.000 0.006 0.000 0.013 0.006 0.000 0.006

0.006 0.006 0.006 0.006 0.000 0.000 0.000 0.000 0.064 0.071

0.065 0.063

>Methylation code ID 1447

0.000 0.000 0.005 0.005 0.000 0.000 0.005 0.000 0.010 0.005

0.000 0.005 0.005 0.005 0.010 0.010 0.000 0.000 0.000 0.000

0.062 0.075 0.060 0.069 0.005 0.000 0.005 0.000 0.000 0.000

0.000 0.000 0.005 0.000 0.000 0.005 0.005 0.000 0.011 0.005

0.016 0.005 0.000 0.005 0.069 0.068 0.058 0.069 0.006 0.000

0.000 0.006 0.000 0.006 0.000 0.006 0.006 0.000 0.000 0.000

0.018 0.006 0.006 0.000 0.012 0.000 0.000 0.000 0.072 0.049

0.060 0.082

>Methylation code ID 1447

0.006 0.000 0.000 0.006 0.000 0.006 0.000 0.006 0.006 0.000

0.000 0.006 0.011 0.006 0.006 0.006 0.011 0.000 0.000 0.000

0.065 0.053 0.061 0.082 0.000 0.000 0.005 0.005 0.000 0.000

0.005 0.005 0.010 0.005 0.000 0.005 0.005 0.000 0.014 0.005

0.000 0.000 0.000 0.000 0.067 0.084 0.065 0.055 0.000 0.000

0.006 0.006 0.000 0.000 0.000 0.000 0.000 0.000 0.000 0.006

0.006 0.000 0.012 0.006 0.018 0.006 0.000 0.006 0.071 0.059

0.068 0.064

>Methylation code ID 1447

0.000 0.000 0.000 0.006 0.000 0.000 0.000 0.000 0.000 0.000

0.000 0.000 0.019 0.000 0.006 0.019 0.019 0.006 0.000 0.006

0.055 0.062 0.063 0.072 0.005 0.000 0.000 0.005 0.000 0.000

0.000 0.005 0.000 0.000 0.000 0.005 0.024 0.000 0.014 0.005

0.000 0.000 0.000 0.000 0.091 0.037 0.080 0.064 0.005 0.000

0.000 0.005 0.000 0.000 0.005 0.005 0.000 0.005 0.000 0.005

0.010 0.000 0.010 0.005 0.000 0.005 0.000 0.000 0.060 0.071

0.073 0.069

>Methylation code ID 1447

0.009 0.000 0.000 0.000 0.000 0.000 0.005 0.005 0.000 0.005

0.000 0.005 0.009 0.000 0.014 0.005 0.000 0.005 0.000 0.000

0.062 0.066 0.077 0.067 0.006 0.000 0.000 0.006 0.000 0.000

0.000 0.000 0.000 0.000 0.000 0.000 0.017 0.000 0.011 0.011

0.011 0.006 0.000 0.006 0.057 0.078 0.065 0.062 0.005 0.000

0.000 0.000 0.000 0.005 0.000 0.005 0.000 0.000 0.000 0.005

0.026 0.000 0.010 0.005 0.000 0.000 0.000 0.000 0.079 0.038

0.087 0.067

>Methylation code ID 1447

0.010 0.000 0.000 0.000 0.000 0.000 0.005 0.005 0.000 0.000

0.000 0.000 0.014 0.005 0.014 0.005 0.000 0.005 0.000 0.000

0.073 0.059 0.065 0.074 0.005 0.000 0.000 0.005 0.000 0.000

0.000 0.000 0.000 0.000 0.000 0.005 0.021 0.000 0.010 0.010

0.005 0.005 0.000 0.000 0.054 0.086 0.072 0.054 0.005 0.000

0.000 0.000 0.000 0.005 0.000 0.000 0.000 0.000 0.000 0.005

0.032 0.000 0.011 0.005 0.000 0.000 0.000 0.000 0.076 0.043

0.080 0.071

>Methylation code ID 1447

0.005 0.000 0.000 0.000 0.000 0.005 0.000 0.000 0.000 0.000

0.000 0.005 0.032 0.000 0.011 0.005 0.005 0.000 0.000 0.000

0.082 0.040 0.083 0.058 0.010 0.000 0.000 0.000 0.000 0.000

0.005 0.005 0.000 0.000 0.000 0.000 0.015 0.005 0.020 0.000

0.000 0.005 0.000 0.000 0.072 0.056 0.070 0.071 0.011 0.000

0.000 0.005 0.000 0.000 0.000 0.000 0.000 0.000 0.000 0.005

0.016 0.000 0.011 0.011 0.005 0.000 0.000 0.000 0.052 0.083

0.067 0.067

>Methylation code ID 1447

0.006 0.000 0.000 0.000 0.000 0.006 0.000 0.000 0.000 0.000

0.000 0.006 0.040 0.000 0.012 0.000 0.006 0.000 0.000 0.000

0.084 0.043 0.076 0.056 0.010 0.000 0.000 0.000 0.000 0.000

0.005 0.005 0.000 0.000 0.000 0.000 0.021 0.005 0.016 0.000

0.000 0.005 0.000 0.000 0.076 0.067 0.065 0.058 0.016 0.000

0.000 0.000 0.000 0.000 0.000 0.000 0.000 0.000 0.000 0.005

0.016 0.000 0.011 0.011 0.005 0.000 0.000 0.000 0.050 0.088

0.068 0.061

>Methylation code ID 1447

0.017 0.000 0.000 0.000 0.006 0.000 0.000 0.000 0.000 0.000

0.000 0.006 0.017 0.006 0.011 0.000 0.011 0.000 0.000 0.000

0.056 0.092 0.061 0.051 0.006 0.000 0.000 0.000 0.000 0.006

0.000 0.000 0.000 0.006 0.000 0.000 0.034 0.000 0.011 0.006

0.006 0.000 0.000 0.000 0.067 0.075 0.055 0.064 0.009 0.000

0.005 0.000 0.005 0.000 0.005 0.000 0.000 0.000 0.000 0.000

0.014 0.005 0.014 0.000 0.000 0.000 0.000 0.000 0.068 0.082

0.069 0.058

>Methylation code ID 1447

0.018 0.000 0.000 0.000 0.012 0.000 0.000 0.000 0.000 0.000

0.000 0.006 0.012 0.006 0.012 0.000 0.012 0.000 0.000 0.000

0.059 0.087 0.053 0.057 0.007 0.000 0.000 0.000 0.000 0.007

0.000 0.000 0.000 0.007 0.000 0.000 0.039 0.000 0.007 0.013

0.007 0.000 0.000 0.000 0.061 0.071 0.050 0.067 0.005 0.000

0.005 0.000 0.005 0.000 0.005 0.000 0.000 0.005 0.000 0.000

0.014 0.005 0.014 0.000 0.000 0.000 0.000 0.000 0.067 0.088

0.063 0.059

>Methylation code ID 1447

0.012 0.000 0.000 0.000 0.006 0.000 0.018 0.000 0.006 0.012

0.000 0.000 0.018 0.000 0.000 0.000 0.006 0.000 0.000 0.000

0.069 0.073 0.047 0.064 0.000 0.000 0.000 0.000 0.009 0.000

0.005 0.000 0.000 0.005 0.000 0.000 0.014 0.000 0.009 0.000

0.014 0.000 0.000 0.000 0.064 0.075 0.070 0.070 0.006 0.000

0.006 0.000 0.000 0.006 0.000 0.000 0.000 0.006 0.000 0.000

0.013 0.006 0.006 0.019 0.006 0.000 0.000 0.000 0.068 0.061

0.046 0.080

>Methylation code ID 1447

0.006 0.006 0.000 0.000 0.000 0.000 0.000 0.000 0.000 0.012

0.000 0.000 0.018 0.000 0.006 0.012 0.018 0.000 0.000 0.000

0.070 0.053 0.069 0.064 0.009 0.000 0.000 0.000 0.009 0.009

0.000 0.000 0.000 0.037 0.000 0.000 0.037 0.009 0.000 0.009

0.000 0.000 0.000 0.000 0.049 0.047 0.057 0.061 0.006 0.000

0.000 0.000 0.006 0.000 0.011 0.000 0.006 0.011 0.000 0.000

0.011 0.000 0.006 0.006 0.000 0.000 0.000 0.006 0.077 0.066

0.066 0.058

>Methylation code ID 1447

0.020 0.000 0.000 0.000 0.000 0.007 0.007 0.000 0.000 0.007

0.000 0.000 0.027 0.007 0.007 0.000 0.007 0.000 0.000 0.000

0.058 0.072 0.062 0.054 0.000 0.005 0.000 0.005 0.000 0.005

0.005 0.000 0.000 0.014 0.000 0.000 0.005 0.005 0.009 0.005

0.005 0.000 0.000 0.000 0.069 0.076 0.052 0.075 0.028 0.000

0.000 0.000 0.000 0.007 0.000 0.000 0.000 0.000 0.000 0.007

0.000 0.000 0.007 0.021 0.007 0.000 0.007 0.000 0.068 0.076

0.059 0.047

>Methylation code ID 1447

0.000 0.000 0.006 0.000 0.000 0.017 0.000 0.000 0.000 0.017

0.000 0.000 0.006 0.006 0.011 0.006 0.006 0.000 0.000 0.000

0.071 0.084 0.052 0.053 0.041 0.000 0.000 0.000 0.000 0.008

0.000 0.000 0.000 0.008 0.000 0.000 0.000 0.000 0.008 0.025

0.008 0.000 0.008 0.000 0.065 0.083 0.039 0.038 0.017 0.000

0.000 0.000 0.000 0.000 0.006 0.000 0.000 0.000 0.000 0.000

0.023 0.000 0.017 0.000 0.000 0.000 0.000 0.000 0.054 0.064

0.059 0.095

>Methylation code ID 1447

0.015 0.000 0.008 0.000 0.000 0.030 0.000 0.000 0.000 0.023

0.000 0.000 0.000 0.008 0.008 0.000 0.008 0.000 0.000 0.000

0.060 0.066 0.060 0.050 0.034 0.000 0.000 0.000 0.000 0.000

0.000 0.000 0.000 0.007 0.000 0.000 0.000 0.000 0.014 0.020

0.007 0.000 0.007 0.000 0.053 0.067 0.059 0.066 0.005 0.000

0.000 0.000 0.000 0.009 0.005 0.000 0.000 0.000 0.000 0.000

0.014 0.000 0.018 0.000 0.000 0.000 0.000 0.000 0.066 0.069

0.063 0.086

>Methylation code ID 1447

0.024 0.000 0.008 0.000 0.000 0.033 0.000 0.000 0.000 0.016

0.000 0.000 0.000 0.008 0.008 0.000 0.008 0.000 0.000 0.000

0.044 0.066 0.065 0.052 0.030 0.000 0.000 0.000 0.000 0.000

0.000 0.000 0.000 0.006 0.000 0.000 0.000 0.000 0.018 0.012

0.006 0.000 0.006 0.000 0.045 0.060 0.072 0.078 0.005 0.000

0.000 0.000 0.000 0.014 0.005 0.000 0.000 0.000 0.000 0.000

0.009 0.000 0.018 0.000 0.000 0.000 0.000 0.000 0.067 0.081

0.050 0.084

>Methylation code ID 1447

0.031 0.000 0.020 0.000 0.000 0.061 0.000 0.000 0.000 0.010

0.000 0.000 0.000 0.000 0.000 0.000 0.010 0.000 0.000 0.000

0.048 0.058 0.047 0.048 0.024 0.000 0.000 0.000 0.000 0.000

0.000 0.000 0.000 0.005 0.005 0.000 0.000 0.000 0.015 0.000

0.005 0.000 0.005 0.000 0.049 0.068 0.084 0.074 0.000 0.000

0.000 0.000 0.000 0.018 0.005 0.000 0.000 0.000 0.000 0.000

0.005 0.000 0.018 0.000 0.000 0.000 0.005 0.000 0.066 0.082

0.068 0.068

>Methylation code ID 1447

0.000 0.000 0.000 0.000 0.000 0.017 0.004 0.000 0.000 0.000

0.000 0.000 0.004 0.000 0.017 0.000 0.000 0.000 0.008 0.000

0.071 0.073 0.069 0.070 0.031 0.000 0.021 0.000 0.000 0.062

0.000 0.000 0.000 0.010 0.000 0.000 0.000 0.000 0.000 0.000

0.010 0.000 0.000 0.000 0.059 0.044 0.047 0.049 0.019 0.000

0.000 0.000 0.000 0.000 0.000 0.000 0.000 0.005 0.005 0.000

0.000 0.000 0.015 0.000 0.005 0.000 0.005 0.000 0.048 0.067

0.087 0.078

>Methylation code ID 1447

0.000 0.000 0.000 0.000 0.000 0.016 0.000 0.000 0.000 0.000

0.000 0.000 0.004 0.000 0.020 0.000 0.000 0.000 0.008 0.000

0.074 0.065 0.074 0.073 0.042 0.000 0.021 0.000 0.000 0.063

0.000 0.000 0.000 0.010 0.000 0.000 0.000 0.000 0.000 0.000

0.000 0.000 0.000 0.000 0.053 0.056 0.044 0.045 0.022 0.000

0.000 0.000 0.000 0.000 0.000 0.000 0.000 0.000 0.004 0.000

0.000 0.000 0.013 0.000 0.004 0.000 0.004 0.000 0.045 0.061

0.093 0.085

>Methylation code ID 1447

0.004 0.000 0.000 0.000 0.000 0.014 0.000 0.000 0.000 0.000

0.000 0.000 0.004 0.000 0.018 0.000 0.000 0.000 0.007 0.000

0.076 0.064 0.076 0.071 0.047 0.000 0.012 0.000 0.000 0.070

0.000 0.000 0.000 0.023 0.000 0.000 0.000 0.000 0.000 0.000

0.000 0.000 0.000 0.000 0.046 0.051 0.049 0.036 0.021 0.000

0.000 0.000 0.000 0.000 0.000 0.000 0.000 0.000 0.004 0.000

0.000 0.000 0.017 0.000 0.000 0.000 0.004 0.000 0.051 0.064

0.095 0.077

>Methylation code ID 1447

0.055 0.000 0.011 0.000 0.000 0.055 0.000 0.000 0.000 0.022

0.000 0.000 0.000 0.000 0.000 0.000 0.000 0.000 0.000 0.000

0.042 0.048 0.047 0.053 0.018 0.000 0.000 0.000 0.000 0.000

0.000 0.000 0.000 0.004 0.004 0.000 0.000 0.000 0.018 0.004

0.000 0.000 0.004 0.000 0.057 0.074 0.101 0.048 0.004 0.000

0.000 0.000 0.000 0.015 0.000 0.000 0.000 0.000 0.000 0.000

0.000 0.000 0.015 0.000 0.000 0.000 0.012 0.000 0.090 0.043

0.094 0.060

>Methylation code ID 1447

0.056 0.000 0.011 0.000 0.000 0.067 0.000 0.000 0.000 0.011

0.000 0.000 0.000 0.000 0.000 0.000 0.000 0.000 0.000 0.000

0.038 0.045 0.049 0.056 0.022 0.000 0.000 0.000 0.000 0.000

0.000 0.000 0.000 0.006 0.006 0.000 0.000 0.000 0.022 0.006

0.000 0.000 0.000 0.000 0.050 0.064 0.096 0.063 0.004 0.000

0.000 0.000 0.000 0.012 0.000 0.000 0.000 0.000 0.000 0.000

0.000 0.000 0.020 0.000 0.000 0.000 0.012 0.000 0.083 0.045

0.096 0.062

>Methylation code ID 1447

0.004 0.000 0.000 0.000 0.000 0.012 0.000 0.000 0.000 0.000

0.000 0.000 0.000 0.000 0.020 0.000 0.000 0.000 0.012 0.000

0.083 0.045 0.096 0.062 0.038 0.000 0.010 0.010 0.000 0.058

0.000 0.000 0.000 0.010 0.000 0.000 0.000 0.000 0.000 0.000

0.000 0.000 0.000 0.000 0.043 0.050 0.054 0.061 0.025 0.000

0.000 0.000 0.000 0.000 0.000 0.000 0.000 0.006 0.006 0.000

0.000 0.000 0.019 0.006 0.000 0.000 0.000 0.000 0.056 0.072

0.089 0.055

>Methylation code ID 1447

0.029 0.000 0.010 0.010 0.000 0.049 0.000 0.000 0.000 0.010

0.000 0.000 0.000 0.010 0.000 0.000 0.000 0.000 0.000 0.000

0.055 0.056 0.049 0.056 0.020 0.000 0.000 0.000 0.000 0.000

0.000 0.000 0.000 0.005 0.005 0.000 0.000 0.000 0.015 0.010

0.000 0.000 0.005 0.000 0.054 0.071 0.081 0.068 0.008 0.000

0.000 0.000 0.000 0.008 0.000 0.000 0.000 0.000 0.000 0.000

0.000 0.000 0.021 0.000 0.000 0.000 0.008 0.000 0.081 0.053

0.094 0.060

>Methylation code ID 1447

0.032 0.000 0.011 0.011 0.000 0.042 0.000 0.000 0.000 0.011

0.000 0.000 0.000 0.011 0.000 0.000 0.000 0.000 0.000 0.000

0.046 0.061 0.054 0.057 0.014 0.000 0.000 0.000 0.000 0.000

0.000 0.000 0.000 0.005 0.005 0.000 0.000 0.000 0.014 0.009

0.000 0.000 0.009 0.000 0.049 0.081 0.077 0.071 0.009 0.000

0.000 0.000 0.000 0.013 0.000 0.000 0.000 0.000 0.000 0.000

0.000 0.000 0.017 0.000 0.000 0.000 0.009 0.000 0.071 0.054

0.082 0.078

>Methylation code ID 1447

0.004 0.000 0.000 0.000 0.000 0.017 0.000 0.000 0.000 0.000

0.000 0.000 0.004 0.000 0.022 0.000 0.000 0.000 0.004 0.000

0.050 0.067 0.069 0.095 0.039 0.000 0.000 0.010 0.000 0.039

0.010 0.000 0.000 0.000 0.000 0.000 0.000 0.010 0.000 0.000

0.000 0.000 0.000 0.000 0.053 0.074 0.043 0.057 0.009 0.000

0.000 0.000 0.000 0.000 0.000 0.000 0.000 0.004 0.000 0.000

0.009 0.000 0.009 0.004 0.004 0.000 0.009 0.000 0.039 0.067

0.087 0.092

>Methylation code ID 1447

0.024 0.008 0.000 0.008 0.000 0.024 0.008 0.000 0.000 0.000

0.000 0.000 0.000 0.008 0.000 0.000 0.000 0.008 0.000 0.000

0.066 0.067 0.051 0.060 0.016 0.000 0.000 0.000 0.000 0.005

0.000 0.000 0.000 0.005 0.000 0.000 0.011 0.000 0.011 0.005

0.005 0.000 0.011 0.000 0.049 0.066 0.074 0.076 0.005 0.000

0.000 0.000 0.000 0.024 0.000 0.000 0.000 0.005 0.000 0.000

0.005 0.000 0.014 0.000 0.000 0.000 0.005 0.000 0.062 0.061

0.059 0.094

>Methylation code ID 1447

0.035 0.009 0.000 0.000 0.000 0.026 0.009 0.000 0.000 0.000

0.000 0.000 0.000 0.009 0.000 0.000 0.000 0.009 0.000 0.000

0.067 0.063 0.043 0.064 0.017 0.000 0.000 0.000 0.000 0.006

0.000 0.000 0.000 0.006 0.000 0.000 0.017 0.000 0.011 0.000

0.006 0.000 0.011 0.000 0.046 0.065 0.074 0.075 0.000 0.000

0.000 0.000 0.000 0.021 0.000 0.000 0.000 0.004 0.000 0.000

0.004 0.000 0.017 0.000 0.000 0.000 0.004 0.000 0.066 0.066

0.052 0.098

>Methylation code ID 1447

0.033 0.008 0.008 0.000 0.000 0.025 0.008 0.000 0.000 0.000

0.000 0.000 0.000 0.000 0.000 0.000 0.000 0.008 0.000 0.000

0.069 0.052 0.052 0.069 0.018 0.000 0.000 0.000 0.000 0.006

0.000 0.006 0.000 0.006 0.000 0.000 0.018 0.000 0.006 0.000

0.006 0.000 0.012 0.000 0.066 0.057 0.063 0.068 0.000 0.000

0.000 0.000 0.000 0.015 0.000 0.000 0.000 0.004 0.000 0.000

0.004 0.000 0.019 0.000 0.000 0.000 0.004 0.000 0.070 0.082

0.064 0.071

>Methylation code ID 1447

0.018 0.006 0.006 0.000 0.000 0.018 0.006 0.000 0.000 0.012

0.000 0.000 0.000 0.000 0.006 0.000 0.000 0.006 0.000 0.000

0.079 0.050 0.066 0.061 0.018 0.006 0.000 0.000 0.000 0.006

0.000 0.006 0.000 0.012 0.000 0.000 0.012 0.000 0.006 0.000

0.006 0.006 0.000 0.000 0.062 0.068 0.057 0.069 0.004 0.000

0.000 0.000 0.000 0.008 0.000 0.000 0.000 0.004 0.000 0.000

0.000 0.000 0.021 0.008 0.000 0.000 0.004 0.000 0.067 0.072

0.064 0.080

>Methylation code ID 1447

0.018 0.006 0.006 0.000 0.000 0.018 0.000 0.000 0.006 0.012

0.000 0.000 0.000 0.000 0.006 0.000 0.000 0.006 0.000 0.000

0.082 0.043 0.069 0.061 0.016 0.005 0.000 0.000 0.000 0.005

0.000 0.005 0.000 0.011 0.000 0.005 0.011 0.000 0.005 0.000

0.000 0.005 0.000 0.000 0.059 0.067 0.065 0.071 0.005 0.000

0.000 0.000 0.000 0.009 0.000 0.000 0.000 0.005 0.000 0.000

0.000 0.000 0.018 0.009 0.000 0.000 0.005 0.000 0.073 0.066

0.071 0.073

>Methylation code ID 1447

0.005 0.000 0.000 0.000 0.000 0.009 0.000 0.000 0.000 0.005

0.000 0.000 0.000 0.000 0.018 0.009 0.005 0.000 0.005 0.000

0.072 0.066 0.069 0.072 0.011 0.006 0.006 0.000 0.000 0.017

0.000 0.000 0.006 0.017 0.000 0.000 0.000 0.000 0.006 0.000

0.000 0.006 0.000 0.000 0.087 0.033 0.083 0.057 0.017 0.006

0.000 0.000 0.000 0.006 0.000 0.006 0.000 0.012 0.000 0.006

0.006 0.000 0.006 0.000 0.000 0.006 0.000 0.000 0.059 0.069

0.065 0.071

>Methylation code ID 1447

0.005 0.005 0.000 0.000 0.000 0.005 0.000 0.005 0.000 0.016

0.000 0.005 0.005 0.000 0.005 0.005 0.000 0.005 0.000 0.000

0.063 0.067 0.062 0.076 0.005 0.005 0.005 0.000 0.000 0.010

0.000 0.000 0.000 0.000 0.000 0.000 0.005 0.000 0.010 0.010

0.005 0.000 0.005 0.000 0.053 0.075 0.081 0.064 0.012 0.000

0.006 0.000 0.000 0.006 0.000 0.000 0.006 0.018 0.000 0.000

0.000 0.000 0.006 0.000 0.006 0.012 0.000 0.000 0.074 0.033

0.090 0.065

>Methylation code ID 1447

0.013 0.000 0.000 0.000 0.000 0.013 0.006 0.000 0.000 0.006

0.000 0.000 0.013 0.000 0.006 0.006 0.013 0.000 0.000 0.000

0.060 0.066 0.065 0.065 0.018 0.006 0.000 0.000 0.000 0.000

0.012 0.000 0.000 0.000 0.000 0.000 0.012 0.018 0.000 0.000

0.006 0.000 0.006 0.000 0.056 0.055 0.059 0.084 0.005 0.000

0.000 0.000 0.000 0.005 0.005 0.000 0.000 0.000 0.000 0.005

0.014 0.000 0.014 0.005 0.005 0.000 0.000 0.000 0.085 0.055

0.073 0.064

>Methylation code ID 1447

0.010 0.000 0.000 0.000 0.000 0.015 0.005 0.000 0.000 0.005

0.000 0.000 0.010 0.000 0.010 0.000 0.005 0.000 0.000 0.000

0.068 0.058 0.074 0.071 0.023 0.006 0.000 0.000 0.000 0.006

0.012 0.000 0.000 0.000 0.000 0.000 0.012 0.006 0.000 0.000

0.006 0.000 0.006 0.000 0.063 0.060 0.051 0.085 0.004 0.000

0.000 0.000 0.000 0.004 0.004 0.000 0.000 0.000 0.000 0.000

0.013 0.000 0.018 0.000 0.009 0.000 0.000 0.000 0.080 0.056

0.074 0.070

>Methylation code ID 1447

0.019 0.006 0.000 0.000 0.000 0.013 0.013 0.000 0.000 0.000

0.000 0.000 0.013 0.000 0.000 0.006 0.006 0.000 0.006 0.000

0.070 0.062 0.049 0.070 0.010 0.000 0.000 0.000 0.000 0.005

0.005 0.000 0.000 0.000 0.000 0.000 0.005 0.005 0.015 0.000

0.010 0.005 0.000 0.000 0.080 0.051 0.055 0.086 0.005 0.005

0.000 0.000 0.000 0.010 0.005 0.000 0.005 0.005 0.000 0.000

0.010 0.000 0.005 0.000 0.005 0.000 0.000 0.000 0.065 0.055

0.077 0.081

>Methylation code ID 1447

0.004 0.004 0.000 0.000 0.000 0.009 0.004 0.000 0.004 0.004

0.000 0.000 0.009 0.000 0.009 0.000 0.004 0.000 0.000 0.000

0.056 0.064 0.078 0.082 0.012 0.006 0.006 0.000 0.000 0.012

0.012 0.000 0.000 0.000 0.000 0.000 0.012 0.000 0.000 0.006

0.006 0.000 0.006 0.000 0.063 0.065 0.045 0.082 0.011 0.000

0.000 0.000 0.000 0.006 0.006 0.000 0.000 0.000 0.000 0.000

0.006 0.006 0.011 0.000 0.011 0.006 0.000 0.000 0.077 0.060

0.052 0.082

>Methylation code ID 1470

0.006 0.006 0.000 0.000 0.000 0.000 0.012 0.000 0.000 0.025

0.000 0.000 0.019 0.006 0.000 0.006 0.000 0.000 0.000 0.000

0.058 0.070 0.059 0.066 0.007 0.007 0.000 0.000 0.007 0.000

0.013 0.000 0.000 0.007 0.000 0.000 0.013 0.000 0.000 0.020

0.013 0.000 0.000 0.000 0.054 0.061 0.057 0.076 0.013 0.000

0.000 0.000 0.000 0.000 0.000 0.000 0.000 0.006 0.000 0.000

0.031 0.000 0.006 0.006 0.013 0.000 0.000 0.000 0.055 0.063

0.068 0.072

>Methylation code ID 1470

0.007 0.000 0.007 0.000 0.007 0.000 0.007 0.007 0.000 0.014

0.000 0.000 0.014 0.007 0.000 0.014 0.007 0.000 0.000 0.000

0.055 0.059 0.075 0.055 0.000 0.000 0.000 0.000 0.006 0.000

0.000 0.000 0.000 0.006 0.000 0.000 0.026 0.000 0.006 0.026

0.013 0.000 0.000 0.000 0.064 0.070 0.056 0.060 0.005 0.000

0.000 0.000 0.005 0.000 0.010 0.000 0.000 0.005 0.000 0.000

0.015 0.000 0.010 0.005 0.000 0.005 0.000 0.000 0.069 0.072

0.063 0.070

>Methylation code ID 1470

0.006 0.000 0.000 0.000 0.000 0.000 0.006 0.000 0.000 0.012

0.000 0.000 0.024 0.000 0.012 0.012 0.000 0.006 0.000 0.000

0.060 0.073 0.050 0.074 0.006 0.000 0.006 0.000 0.006 0.000

0.000 0.000 0.000 0.012 0.000 0.000 0.018 0.006 0.006 0.012

0.006 0.000 0.000 0.000 0.064 0.066 0.075 0.051 0.007 0.000

0.000 0.000 0.000 0.000 0.000 0.000 0.000 0.013 0.000 0.000

0.027 0.000 0.007 0.020 0.007 0.000 0.000 0.000 0.055 0.058

0.075 0.065

>Methylation code ID 1470

0.013 0.000 0.004 0.004 0.000 0.009 0.000 0.000 0.000 0.000

0.000 0.000 0.004 0.000 0.013 0.004 0.000 0.004 0.000 0.000

0.088 0.057 0.059 0.072 0.005 0.000 0.000 0.009 0.000 0.009

0.005 0.000 0.000 0.009 0.000 0.000 0.005 0.000 0.009 0.005

0.005 0.000 0.000 0.000 0.078 0.047 0.065 0.084 0.006 0.000

0.000 0.000 0.000 0.018 0.000 0.000 0.000 0.006 0.000 0.000

0.006 0.000 0.018 0.006 0.006 0.000 0.000 0.000 0.055 0.077

0.074 0.061

>Methylation code ID 1470

0.007 0.004 0.004 0.000 0.000 0.007 0.000 0.000 0.000 0.000

0.000 0.000 0.000 0.004 0.015 0.004 0.000 0.004 0.000 0.000

0.053 0.062 0.087 0.082 0.010 0.000 0.005 0.000 0.000 0.015

0.000 0.000 0.000 0.000 0.000 0.000 0.000 0.000 0.015 0.010

0.005 0.005 0.000 0.000 0.072 0.060 0.068 0.069 0.000 0.000

0.000 0.014 0.000 0.028 0.000 0.000 0.000 0.007 0.000 0.000

0.000 0.007 0.007 0.007 0.007 0.000 0.000 0.000 0.073 0.049

0.059 0.077

>Methylation code ID 1470

0.009 0.009 0.005 0.000 0.000 0.005 0.000 0.000 0.000 0.005

0.000 0.005 0.009 0.005 0.005 0.000 0.000 0.005 0.000 0.000

0.071 0.069 0.055 0.079 0.013 0.000 0.000 0.000 0.000 0.006

0.000 0.000 0.000 0.000 0.000 0.000 0.006 0.006 0.013 0.019

0.013 0.006 0.000 0.000 0.077 0.051 0.058 0.064 0.006 0.000

0.000 0.000 0.000 0.012 0.000 0.000 0.000 0.006 0.000 0.000

0.012 0.012 0.012 0.006 0.000 0.000 0.000 0.000 0.062 0.057

0.056 0.092

>Methylation code ID 1470

0.005 0.000 0.000 0.000 0.000 0.015 0.000 0.000 0.000 0.005

0.000 0.000 0.010 0.010 0.010 0.000 0.000 0.000 0.005 0.000

0.069 0.073 0.063 0.069 0.010 0.010 0.000 0.010 0.000 0.005

0.000 0.000 0.000 0.005 0.000 0.005 0.010 0.000 0.005 0.000

0.000 0.005 0.000 0.000 0.068 0.072 0.052 0.077 0.013 0.000

0.000 0.000 0.000 0.007 0.000 0.000 0.000 0.007 0.000 0.000

0.007 0.007 0.020 0.013 0.007 0.000 0.000 0.000 0.070 0.074

0.055 0.056

>Methylation code ID 1470

0.005 0.000 0.000 0.011 0.000 0.011 0.000 0.000 0.000 0.011

0.000 0.000 0.011 0.000 0.011 0.000 0.005 0.000 0.000 0.000

0.051 0.082 0.070 0.067 0.009 0.000 0.009 0.000 0.004 0.004

0.000 0.000 0.000 0.004 0.000 0.000 0.004 0.004 0.013 0.004

0.000 0.000 0.000 0.000 0.061 0.078 0.069 0.070 0.000 0.000

0.004 0.000 0.000 0.009 0.000 0.000 0.000 0.004 0.000 0.000

0.009 0.000 0.009 0.004 0.009 0.000 0.004 0.000 0.067 0.069

0.070 0.074

>Methylation code ID 1470

0.008 0.000 0.008 0.000 0.004 0.004 0.000 0.000 0.004 0.004

0.004 0.000 0.004 0.000 0.013 0.000 0.000 0.000 0.000 0.000

0.068 0.065 0.072 0.072 0.006 0.000 0.006 0.000 0.000 0.011

0.000 0.000 0.000 0.006 0.000 0.000 0.006 0.000 0.006 0.011

0.011 0.000 0.006 0.000 0.060 0.069 0.067 0.071 0.005 0.000

0.000 0.010 0.000 0.010 0.005 0.000 0.000 0.005 0.000 0.000

0.005 0.000 0.010 0.000 0.005 0.000 0.000 0.000 0.054 0.076

0.068 0.079

>Methylation code ID 1470

0.009 0.000 0.000 0.009 0.000 0.000 0.004 0.000 0.000 0.004

0.000 0.000 0.004 0.000 0.009 0.004 0.000 0.000 0.009 0.000

0.083 0.050 0.077 0.070 0.005 0.000 0.000 0.000 0.000 0.011

0.000 0.000 0.005 0.005 0.005 0.000 0.011 0.000 0.005 0.005

0.000 0.005 0.005 0.005 0.062 0.063 0.071 0.068 0.008 0.000

0.000 0.000 0.000 0.015 0.000 0.000 0.000 0.015 0.000 0.000

0.008 0.000 0.008 0.015 0.008 0.000 0.000 0.000 0.054 0.065

0.060 0.079

>Methylation code ID 1470

0.025 0.000 0.000 0.000 0.000 0.016 0.000 0.000 0.000 0.008

0.008 0.000 0.025 0.000 0.000 0.016 0.000 0.000 0.008 0.000

0.051 0.048 0.069 0.057 0.011 0.000 0.000 0.000 0.000 0.033

0.000 0.000 0.000 0.044 0.000 0.000 0.000 0.033 0.000 0.000

0.000 0.000 0.011 0.000 0.041 0.050 0.047 0.064 0.005 0.000

0.000 0.011 0.000 0.005 0.000 0.000 0.000 0.000 0.000 0.005

0.000 0.000 0.005 0.022 0.000 0.000 0.011 0.000 0.070 0.053

0.060 0.085

>Methylation code ID 1470

0.032 0.000 0.006 0.000 0.000 0.006 0.000 0.000 0.000 0.000

0.006 0.000 0.019 0.000 0.006 0.000 0.006 0.000 0.000 0.000

0.065 0.074 0.053 0.059 0.007 0.000 0.000 0.000 0.000 0.007

0.000 0.000 0.000 0.033 0.000 0.000 0.000 0.020 0.007 0.000

0.007 0.000 0.007 0.000 0.058 0.065 0.056 0.068 0.005 0.005

0.000 0.000 0.000 0.011 0.005 0.000 0.000 0.000 0.000 0.005

0.000 0.000 0.011 0.016 0.000 0.000 0.000 0.000 0.043 0.078

0.074 0.080

>Methylation code ID 1470

0.006 0.012 0.000 0.000 0.000 0.006 0.012 0.000 0.000 0.000

0.000 0.000 0.018 0.000 0.006 0.006 0.000 0.006 0.000 0.000

0.052 0.070 0.064 0.077 0.019 0.000 0.005 0.000 0.005 0.000

0.000 0.000 0.000 0.000 0.000 0.000 0.015 0.000 0.010 0.000

0.010 0.000 0.000 0.000 0.073 0.068 0.074 0.055 0.005 0.000

0.000 0.000 0.000 0.005 0.000 0.000 0.000 0.016 0.000 0.000

0.011 0.005 0.011 0.005 0.005 0.000 0.000 0.000 0.061 0.060

0.072 0.075

>Methylation code ID 1470

0.005 0.000 0.000 0.000 0.000 0.000 0.000 0.000 0.000 0.015

0.000 0.000 0.015 0.005 0.015 0.010 0.000 0.000 0.000 0.000

0.066 0.062 0.070 0.069 0.006 0.013 0.000 0.000 0.006 0.006

0.006 0.000 0.000 0.000 0.000 0.000 0.026 0.000 0.000 0.006

0.000 0.013 0.000 0.000 0.060 0.063 0.062 0.064 0.018 0.000

0.000 0.006 0.006 0.000 0.000 0.000 0.000 0.000 0.000 0.000

0.018 0.006 0.006 0.006 0.006 0.000 0.000 0.000 0.060 0.064

0.066 0.070

>Methylation code ID 1470

0.000 0.000 0.000 0.009 0.000 0.017 0.009 0.009 0.000 0.000

0.000 0.000 0.000 0.000 0.000 0.052 0.009 0.000 0.009 0.000

0.056 0.053 0.050 0.062 0.000 0.000 0.000 0.000 0.000 0.026

0.000 0.013 0.000 0.026 0.000 0.000 0.013 0.000 0.000 0.026

0.000 0.065 0.000 0.000 0.047 0.034 0.045 0.039 0.000 0.000

0.000 0.013 0.013 0.000 0.007 0.000 0.000 0.007 0.000 0.000

0.000 0.000 0.007 0.000 0.000 0.000 0.007 0.007 0.082 0.058

0.079 0.056

>Methylation code ID 1470

0.000 0.005 0.000 0.005 0.010 0.000 0.000 0.005 0.000 0.010

0.000 0.000 0.000 0.005 0.005 0.000 0.010 0.005 0.005 0.000

0.062 0.072 0.073 0.061 0.000 0.006 0.006 0.000 0.006 0.006

0.000 0.000 0.000 0.006 0.000 0.000 0.000 0.000 0.006 0.034

0.000 0.006 0.000 0.000 0.070 0.061 0.056 0.073 0.007 0.000

0.000 0.000 0.007 0.000 0.015 0.000 0.000 0.007 0.000 0.000

0.000 0.000 0.007 0.007 0.007 0.007 0.000 0.000 0.075 0.040

0.084 0.068

>Methylation code ID 1470

0.000 0.005 0.005 0.000 0.005 0.005 0.000 0.000 0.005 0.005

0.000 0.000 0.000 0.000 0.005 0.026 0.000 0.005 0.000 0.000

0.069 0.072 0.054 0.072 0.007 0.000 0.000 0.000 0.007 0.000

0.007 0.000 0.000 0.007 0.000 0.007 0.000 0.000 0.007 0.007

0.007 0.015 0.000 0.000 0.069 0.049 0.082 0.059 0.000 0.006

0.000 0.006 0.011 0.000 0.000 0.000 0.000 0.011 0.000 0.000

0.000 0.006 0.006 0.000 0.006 0.006 0.006 0.000 0.064 0.066

0.071 0.071

>Methylation code ID 1470

0.006 0.006 0.000 0.000 0.000 0.006 0.000 0.006 0.006 0.006

0.000 0.006 0.000 0.000 0.006 0.006 0.006 0.011 0.000 0.000

0.080 0.056 0.067 0.064 0.018 0.000 0.000 0.009 0.000 0.000

0.000 0.000 0.000 0.018 0.000 0.009 0.000 0.009 0.000 0.009

0.009 0.009 0.009 0.000 0.055 0.068 0.065 0.047 0.000 0.005

0.000 0.000 0.000 0.005 0.000 0.000 0.005 0.000 0.005 0.000

0.005 0.010 0.010 0.010 0.000 0.000 0.000 0.005 0.062 0.064

0.070 0.076

>Methylation code ID 3162

0.000 0.006 0.000 0.006 0.012 0.006 0.000 0.006 0.000 0.012

0.000 0.000 0.012 0.006 0.000 0.000 0.000 0.000 0.000 0.012

0.064 0.061 0.055 0.077 0.000 0.005 0.005 0.000 0.010 0.000

0.000 0.000 0.000 0.005 0.000 0.005 0.005 0.000 0.005 0.005

0.010 0.005 0.000 0.005 0.059 0.076 0.071 0.064 0.007 0.000

0.000 0.000 0.000 0.000 0.007 0.013 0.000 0.000 0.007 0.000

0.007 0.000 0.007 0.013 0.000 0.013 0.000 0.000 0.067 0.045

0.075 0.073

>Methylation code ID 3162

0.006 0.000 0.000 0.000 0.000 0.006 0.006 0.006 0.013 0.000

0.000 0.006 0.006 0.000 0.006 0.013 0.006 0.000 0.000 0.000

0.074 0.058 0.074 0.052 0.000 0.000 0.000 0.000 0.005 0.005

0.000 0.005 0.009 0.000 0.000 0.005 0.009 0.005 0.009 0.000

0.005 0.005 0.000 0.000 0.058 0.080 0.084 0.052 0.005 0.000

0.000 0.005 0.010 0.005 0.005 0.005 0.005 0.000 0.000 0.005

0.005 0.000 0.005 0.000 0.000 0.000 0.000 0.000 0.083 0.079

0.057 0.058

>Methylation code ID 3162

0.005 0.000 0.000 0.005 0.009 0.005 0.005 0.005 0.009 0.000

0.000 0.005 0.005 0.000 0.005 0.000 0.000 0.000 0.000 0.000

0.080 0.080 0.061 0.056 0.006 0.000 0.000 0.000 0.000 0.006

0.006 0.006 0.006 0.000 0.000 0.006 0.006 0.000 0.006 0.019

0.006 0.000 0.000 0.000 0.075 0.063 0.074 0.044 0.000 0.000

0.000 0.000 0.005 0.005 0.000 0.005 0.010 0.000 0.000 0.000

0.010 0.005 0.010 0.000 0.005 0.005 0.000 0.000 0.060 0.083

0.083 0.049

>Methylation code ID 3162

0.000 0.000 0.006 0.006 0.000 0.000 0.000 0.013 0.006 0.000

0.000 0.000 0.019 0.000 0.006 0.000 0.006 0.006 0.000 0.000

0.060 0.073 0.061 0.068 0.000 0.005 0.000 0.005 0.000 0.000

0.005 0.000 0.015 0.015 0.000 0.000 0.010 0.000 0.000 0.005

0.005 0.000 0.000 0.000 0.058 0.070 0.072 0.068 0.006 0.000

0.000 0.000 0.000 0.000 0.006 0.000 0.006 0.006 0.000 0.006

0.006 0.000 0.012 0.006 0.006 0.000 0.000 0.006 0.076 0.062

0.070 0.058

>Methylation code ID 3162

0.000 0.000 0.000 0.000 0.005 0.011 0.005 0.011 0.005 0.000

0.000 0.000 0.011 0.000 0.005 0.005 0.000 0.005 0.000 0.000

0.075 0.068 0.072 0.054 0.014 0.000 0.000 0.007 0.000 0.000

0.007 0.007 0.007 0.014 0.000 0.007 0.000 0.000 0.000 0.007

0.000 0.000 0.000 0.007 0.074 0.060 0.073 0.049 0.000 0.000

0.000 0.000 0.005 0.000 0.000 0.005 0.005 0.005 0.000 0.005

0.005 0.000 0.015 0.000 0.010 0.000 0.000 0.005 0.064 0.081

0.056 0.071

>Methylation code ID 3162

0.016 0.000 0.000 0.008 0.000 0.000 0.008 0.000 0.008 0.016

0.000 0.000 0.000 0.000 0.000 0.024 0.000 0.000 0.000 0.008

0.065 0.069 0.061 0.051 0.000 0.000 0.000 0.000 0.004 0.000

0.000 0.004 0.004 0.004 0.000 0.004 0.004 0.004 0.018 0.000

0.004 0.000 0.000 0.004 0.078 0.080 0.044 0.074 0.000 0.000

0.000 0.000 0.011 0.011 0.000 0.011 0.005 0.000 0.000 0.000

0.005 0.000 0.005 0.005 0.000 0.011 0.000 0.000 0.070 0.054

0.076 0.068

>Methylation code ID 3162

0.000 0.000 0.000 0.000 0.010 0.010 0.005 0.000 0.005 0.000

0.000 0.005 0.005 0.000 0.005 0.010 0.000 0.010 0.000 0.000

0.059 0.063 0.079 0.069 0.012 0.000 0.000 0.006 0.000 0.000

0.006 0.006 0.000 0.012 0.006 0.000 0.000 0.000 0.006 0.019

0.000 0.000 0.000 0.006 0.059 0.075 0.070 0.049 0.000 0.000

0.000 0.005 0.009 0.000 0.000 0.005 0.000 0.005 0.000 0.000

0.005 0.005 0.014 0.005 0.005 0.000 0.000 0.000 0.086 0.076

0.032 0.084

>Methylation code ID 3162

0.019 0.000 0.000 0.000 0.010 0.000 0.000 0.010 0.000 0.000

0.000 0.000 0.010 0.010 0.000 0.057 0.000 0.000 0.000 0.000

0.047 0.059 0.053 0.061 0.010 0.000 0.005 0.000 0.005 0.000

0.010 0.000 0.000 0.005 0.000 0.000 0.020 0.000 0.005 0.000

0.000 0.000 0.000 0.000 0.063 0.067 0.060 0.082 0.000 0.000

0.000 0.000 0.013 0.006 0.000 0.006 0.006 0.019 0.000 0.000

0.000 0.006 0.006 0.000 0.013 0.000 0.000 0.000 0.050 0.053

0.079 0.076

>Methylation code ID 3162

0.020 0.000 0.000 0.000 0.007 0.000 0.000 0.007 0.000 0.000

0.000 0.000 0.013 0.000 0.007 0.020 0.000 0.013 0.000 0.000

0.069 0.055 0.057 0.067 0.006 0.000 0.000 0.000 0.006 0.006

0.006 0.000 0.000 0.012 0.000 0.000 0.025 0.006 0.000 0.000

0.000 0.000 0.000 0.012 0.067 0.056 0.055 0.074 0.000 0.004

0.004 0.000 0.009 0.000 0.000 0.000 0.000 0.004 0.000 0.000

0.004 0.004 0.009 0.004 0.009 0.000 0.000 0.000 0.050 0.069

0.086 0.075

>Methylation code ID 3164

0.000 0.005 0.000 0.010 0.000 0.005 0.000 0.000 0.005 0.010

0.000 0.010 0.000 0.000 0.005 0.010 0.000 0.005 0.000 0.000

0.086 0.071 0.046 0.064 0.011 0.000 0.000 0.000 0.011 0.005

0.011 0.000 0.011 0.000 0.000 0.000 0.000 0.000 0.000 0.005

0.011 0.000 0.005 0.000 0.058 0.072 0.061 0.073 0.000 0.000

0.000 0.000 0.000 0.012 0.000 0.012 0.012 0.006 0.000 0.000

0.000 0.000 0.012 0.012 0.000 0.000 0.000 0.000 0.042 0.074

0.086 0.066

>Methylation code ID 3167

0.000 0.000 0.000 0.000 0.000 0.018 0.000 0.000 0.006 0.000

0.000 0.000 0.012 0.012 0.018 0.012 0.000 0.000 0.000 0.000

0.052 0.080 0.067 0.056 0.011 0.000 0.011 0.000 0.000 0.006

0.000 0.000 0.011 0.006 0.000 0.006 0.011 0.000 0.000 0.000

0.000 0.006 0.000 0.000 0.064 0.064 0.068 0.071 0.000 0.000

0.000 0.005 0.005 0.000 0.000 0.000 0.005 0.000 0.000 0.000

0.005 0.005 0.010 0.005 0.016 0.000 0.005 0.000 0.059 0.085

0.068 0.058

>Methylation code ID 3168

0.000 0.000 0.010 0.005 0.000 0.000 0.000 0.000 0.000 0.000

0.000 0.000 0.010 0.000 0.010 0.000 0.019 0.005 0.005 0.000

0.068 0.072 0.070 0.061 0.000 0.000 0.000 0.000 0.000 0.022

0.000 0.000 0.007 0.007 0.000 0.000 0.029 0.007 0.000 0.000

0.014 0.000 0.000 0.000 0.071 0.056 0.074 0.046 0.010 0.000

0.005 0.000 0.000 0.000 0.005 0.000 0.005 0.005 0.000 0.005

0.000 0.000 0.020 0.000 0.000 0.000 0.000 0.005 0.055 0.078

0.073 0.069

>Methylation code ID 3168

0.000 0.000 0.000 0.000 0.007 0.000 0.020 0.000 0.000 0.020

0.000 0.007 0.007 0.000 0.000 0.000 0.000 0.020 0.000 0.000

0.047 0.072 0.077 0.055 0.000 0.008 0.000 0.008 0.000 0.000

0.000 0.008 0.000 0.000 0.008 0.000 0.008 0.000 0.000 0.033

0.017 0.000 0.000 0.017 0.057 0.060 0.044 0.064 0.006 0.006

0.000 0.000 0.000 0.000 0.000 0.006 0.000 0.017 0.000 0.000

0.012 0.006 0.006 0.006 0.006 0.000 0.000 0.000 0.072 0.050

0.070 0.071

>Methylation code ID 3169

0.000 0.008 0.000 0.000 0.008 0.008 0.000 0.016 0.000 0.016

0.000 0.000 0.008 0.000 0.000 0.000 0.016 0.016 0.008 0.000

0.072 0.050 0.055 0.054 0.008 0.000 0.000 0.000 0.008 0.008

0.008 0.000 0.000 0.008 0.000 0.000 0.008 0.008 0.000 0.041

0.000 0.008 0.000 0.000 0.056 0.069 0.046 0.054 0.000 0.005

0.000 0.000 0.005 0.005 0.009 0.000 0.000 0.005 0.005 0.005

0.009 0.000 0.005 0.005 0.000 0.000 0.000 0.000 0.062 0.074

0.064 0.079

>Methylation code ID 3169

0.010 0.010 0.000 0.000 0.000 0.000 0.005 0.000 0.000 0.005

0.000 0.000 0.005 0.005 0.005 0.000 0.005 0.005 0.005 0.005

0.067 0.046 0.072 0.082 0.010 0.000 0.000 0.000 0.000 0.010

0.020 0.000 0.000 0.020 0.000 0.000 0.000 0.000 0.000 0.031

0.020 0.020 0.000 0.000 0.057 0.046 0.057 0.040 0.006 0.000

0.000 0.000 0.000 0.006 0.006 0.006 0.000 0.006 0.006 0.000

0.011 0.000 0.011 0.011 0.000 0.000 0.000 0.000 0.055 0.075

0.070 0.067

>Methylation code ID 3169

0.011 0.000 0.000 0.000 0.011 0.000 0.000 0.000 0.000 0.021

0.000 0.011 0.000 0.000 0.000 0.042 0.021 0.021 0.000 0.000

0.056 0.060 0.040 0.040 0.005 0.000 0.000 0.000 0.000 0.000

0.005 0.000 0.000 0.005 0.000 0.000 0.010 0.000 0.014 0.019

0.005 0.000 0.000 0.000 0.061 0.085 0.071 0.054 0.006 0.006

0.000 0.000 0.000 0.000 0.012 0.000 0.000 0.018 0.000 0.000

0.000 0.012 0.006 0.000 0.006 0.006 0.000 0.000 0.069 0.056

0.053 0.082

>Methylation code ID 3169

0.011 0.006 0.000 0.000 0.000 0.000 0.011 0.000 0.000 0.017

0.000 0.000 0.000 0.011 0.006 0.000 0.006 0.006 0.000 0.000

0.066 0.054 0.059 0.081 0.009 0.000 0.000 0.000 0.009 0.000

0.000 0.000 0.000 0.017 0.000 0.009 0.000 0.000 0.009 0.035

0.017 0.009 0.000 0.000 0.061 0.073 0.047 0.040 0.005 0.000

0.000 0.000 0.000 0.000 0.005 0.000 0.000 0.000 0.000 0.000

0.010 0.000 0.016 0.021 0.005 0.000 0.000 0.000 0.065 0.078

0.071 0.057

>Methylation code ID 3169

0.012 0.000 0.000 0.006 0.000 0.000 0.006 0.006 0.000 0.024

0.000 0.006 0.000 0.006 0.006 0.006 0.000 0.000 0.000 0.000

0.076 0.058 0.051 0.070 0.011 0.000 0.000 0.000 0.006 0.000

0.000 0.000 0.006 0.011 0.000 0.006 0.000 0.000 0.006 0.011

0.011 0.000 0.006 0.000 0.068 0.067 0.058 0.069 0.000 0.000

0.000 0.000 0.000 0.006 0.006 0.000 0.006 0.000 0.000 0.000

0.012 0.000 0.012 0.012 0.006 0.006 0.000 0.000 0.077 0.081

0.054 0.057

>Methylation code ID 3169

0.007 0.000 0.007 0.007 0.000 0.000 0.000 0.014 0.000 0.022

0.000 0.007 0.000 0.000 0.000 0.014 0.000 0.000 0.000 0.007

0.064 0.067 0.067 0.049 0.013 0.000 0.000 0.000 0.006 0.000

0.000 0.000 0.006 0.006 0.000 0.000 0.000 0.000 0.006 0.006

0.019 0.000 0.013 0.000 0.082 0.047 0.058 0.071 0.000 0.000

0.000 0.005 0.000 0.010 0.010 0.000 0.005 0.005 0.000 0.000

0.005 0.000 0.010 0.000 0.000 0.005 0.000 0.000 0.059 0.063

0.070 0.088

>Methylation code ID 3169

0.006 0.006 0.006 0.000 0.006 0.006 0.000 0.000 0.000 0.013

0.000 0.006 0.000 0.006 0.000 0.013 0.006 0.000 0.000 0.000

0.054 0.063 0.068 0.074 0.023 0.000 0.000 0.000 0.000 0.000

0.006 0.006 0.000 0.006 0.000 0.000 0.006 0.000 0.006 0.000

0.006 0.000 0.012 0.000 0.071 0.059 0.076 0.059 0.000 0.000

0.005 0.005 0.000 0.011 0.000 0.000 0.000 0.005 0.005 0.000

0.005 0.005 0.011 0.005 0.000 0.000 0.000 0.000 0.054 0.072

0.067 0.081

>Methylation code ID 3170

0.006 0.006 0.000 0.000 0.006 0.006 0.000 0.000 0.006 0.018

0.000 0.000 0.000 0.000 0.006 0.006 0.000 0.018 0.000 0.000

0.071 0.060 0.060 0.065 0.005 0.005 0.000 0.000 0.005 0.000

0.000 0.000 0.005 0.010 0.000 0.000 0.005 0.000 0.005 0.000

0.000 0.000 0.000 0.014 0.079 0.064 0.069 0.069 0.006 0.006

0.000 0.000 0.000 0.006 0.000 0.012 0.000 0.000 0.006 0.000

0.006 0.000 0.006 0.012 0.000 0.000 0.006 0.000 0.048 0.075

0.078 0.069

>Methylation code ID 3170

0.006 0.000 0.006 0.006 0.000 0.012 0.000 0.000 0.000 0.012

0.000 0.000 0.000 0.012 0.006 0.006 0.000 0.012 0.000 0.000

0.060 0.067 0.073 0.057 0.000 0.000 0.000 0.000 0.000 0.007

0.000 0.000 0.007 0.007 0.000 0.007 0.015 0.000 0.000 0.015

0.007 0.007 0.000 0.015 0.061 0.059 0.048 0.078 0.008 0.004

0.000 0.000 0.000 0.000 0.000 0.008 0.000 0.000 0.000 0.000

0.004 0.000 0.016 0.000 0.004 0.000 0.000 0.000 0.061 0.070

0.057 0.100

>Methylation code ID 3170

0.010 0.000 0.005 0.000 0.005 0.005 0.000 0.000 0.005 0.000

0.000 0.005 0.005 0.000 0.005 0.010 0.000 0.010 0.000 0.000

0.072 0.062 0.063 0.071 0.011 0.000 0.000 0.000 0.000 0.000

0.000 0.006 0.000 0.000 0.000 0.000 0.011 0.000 0.017 0.017

0.006 0.000 0.000 0.000 0.082 0.060 0.059 0.067 0.000 0.004

0.000 0.004 0.004 0.008 0.000 0.000 0.000 0.004 0.000 0.000

0.004 0.008 0.013 0.000 0.000 0.000 0.000 0.000 0.059 0.088

0.084 0.051

>Methylation code ID 3171

0.004 0.004 0.000 0.000 0.000 0.013 0.000 0.000 0.004 0.000

0.000 0.013 0.004 0.000 0.004 0.000 0.000 0.000 0.004 0.004

0.055 0.068 0.075 0.081 0.014 0.000 0.000 0.014 0.000 0.014

0.000 0.007 0.007 0.007 0.000 0.000 0.007 0.000 0.000 0.000

0.020 0.000 0.000 0.000 0.047 0.060 0.068 0.069 0.000 0.000

0.000 0.000 0.000 0.006 0.000 0.000 0.012 0.012 0.000 0.000

0.006 0.006 0.006 0.006 0.000 0.000 0.006 0.000 0.036 0.060

0.082 0.093

>Methylation code ID 3171

0.012 0.006 0.000 0.000 0.000 0.025 0.000 0.000 0.006 0.006

0.000 0.006 0.000 0.000 0.006 0.006 0.000 0.000 0.000 0.000

0.063 0.065 0.063 0.069 0.011 0.006 0.006 0.017 0.000 0.006

0.000 0.000 0.006 0.011 0.000 0.000 0.000 0.006 0.000 0.000

0.006 0.000 0.000 0.000 0.045 0.062 0.078 0.076 0.000 0.000

0.000 0.000 0.000 0.000 0.000 0.005 0.010 0.000 0.000 0.000

0.005 0.000 0.016 0.010 0.000 0.000 0.005 0.000 0.073 0.056

0.079 0.072

>Methylation code ID 3171

0.005 0.005 0.016 0.005 0.000 0.000 0.000 0.005 0.005 0.011

0.000 0.005 0.000 0.005 0.000 0.005 0.000 0.000 0.000 0.000

0.053 0.075 0.079 0.058 0.000 0.007 0.000 0.000 0.000 0.000

0.000 0.014 0.000 0.007 0.007 0.000 0.007 0.007 0.007 0.021

0.007 0.000 0.000 0.000 0.080 0.050 0.059 0.059 0.016 0.000

0.000 0.000 0.000 0.008 0.000 0.000 0.008 0.008 0.000 0.000

0.000 0.000 0.008 0.008 0.000 0.008 0.000 0.008 0.069 0.086

0.045 0.064

>Methylation code ID 3171

0.005 0.000 0.005 0.010 0.000 0.000 0.000 0.005 0.000 0.005

0.005 0.005 0.000 0.000 0.010 0.005 0.000 0.000 0.000 0.005

0.063 0.067 0.071 0.073 0.000 0.000 0.005 0.005 0.005 0.005

0.000 0.000 0.011 0.005 0.000 0.000 0.005 0.000 0.005 0.000

0.016 0.000 0.000 0.000 0.073 0.070 0.058 0.069 0.000 0.000

0.000 0.006 0.000 0.006 0.006 0.006 0.006 0.006 0.000 0.006

0.000 0.000 0.012 0.006 0.000 0.000 0.000 0.000 0.046 0.059

0.087 0.082

>Methylation code ID 3171

0.000 0.000 0.000 0.000 0.005 0.005 0.000 0.005 0.005 0.016

0.000 0.000 0.000 0.000 0.016 0.005 0.000 0.000 0.000 0.000

0.054 0.074 0.093 0.052 0.012 0.000 0.006 0.012 0.006 0.000

0.000 0.000 0.000 0.006 0.000 0.000 0.000 0.000 0.006 0.006

0.000 0.000 0.000 0.017 0.059 0.056 0.066 0.083 0.000 0.000

0.005 0.000 0.005 0.005 0.000 0.005 0.010 0.005 0.000 0.000

0.010 0.000 0.005 0.000 0.010 0.000 0.000 0.000 0.065 0.062

0.070 0.076

>Methylation code ID 3171

0.011 0.000 0.005 0.005 0.005 0.000 0.000 0.000 0.000 0.005

0.000 0.000 0.000 0.000 0.011 0.005 0.000 0.000 0.000 0.016

0.073 0.063 0.057 0.074 0.000 0.000 0.005 0.000 0.005 0.011

0.000 0.011 0.005 0.000 0.000 0.000 0.011 0.000 0.005 0.000

0.011 0.000 0.000 0.000 0.061 0.071 0.062 0.074 0.000 0.000

0.000 0.000 0.006 0.006 0.000 0.006 0.006 0.019 0.000 0.000

0.000 0.000 0.012 0.000 0.000 0.006 0.000 0.000 0.052 0.076

0.082 0.061

>Methylation code ID 3171

0.000 0.000 0.008 0.000 0.008 0.004 0.004 0.000 0.000 0.004

0.000 0.008 0.000 0.000 0.004 0.004 0.000 0.004 0.004 0.000

0.071 0.057 0.082 0.069 0.000 0.000 0.000 0.000 0.009 0.018

0.000 0.018 0.000 0.000 0.009 0.000 0.000 0.000 0.009 0.018

0.018 0.009 0.000 0.000 0.047 0.047 0.062 0.068 0.005 0.000

0.000 0.000 0.011 0.000 0.000 0.000 0.000 0.011 0.000 0.000

0.000 0.005 0.011 0.000 0.000 0.000 0.005 0.000 0.031 0.074

0.094 0.085

>Methylation code ID 3171

0.011 0.005 0.000 0.011 0.005 0.005 0.000 0.000 0.000 0.005

0.005 0.000 0.000 0.005 0.011 0.000 0.000 0.000 0.000 0.000

0.051 0.069 0.073 0.075 0.004 0.004 0.004 0.000 0.004 0.004

0.004 0.000 0.004 0.000 0.000 0.004 0.000 0.000 0.008 0.004

0.000 0.004 0.004 0.000 0.080 0.065 0.074 0.063 0.000 0.000

0.000 0.000 0.006 0.024 0.000 0.000 0.000 0.000 0.000 0.006

0.000 0.000 0.012 0.006 0.012 0.000 0.000 0.000 0.070 0.064

0.047 0.086

>Methylation code ID 3171

0.000 0.005 0.000 0.000 0.005 0.010 0.000 0.005 0.000 0.005

0.000 0.005 0.005 0.000 0.010 0.000 0.010 0.000 0.000 0.000

0.078 0.058 0.048 0.089 0.006 0.006 0.000 0.012 0.012 0.006

0.000 0.000 0.000 0.012 0.006 0.000 0.006 0.000 0.000 0.000

0.000 0.006 0.000 0.006 0.062 0.067 0.057 0.070 0.005 0.005

0.000 0.000 0.009 0.005 0.000 0.000 0.005 0.005 0.000 0.005

0.005 0.000 0.005 0.005 0.000 0.000 0.000 0.005 0.064 0.075

0.066 0.073

>Methylation code ID 3173

0.000 0.000 0.006 0.000 0.006 0.013 0.013 0.000 0.000 0.006

0.000 0.000 0.006 0.000 0.000 0.006 0.000 0.019 0.000 0.006

0.054 0.058 0.062 0.078 0.000 0.000 0.006 0.000 0.006 0.006

0.000 0.006 0.000 0.006 0.006 0.000 0.006 0.000 0.006 0.019

0.013 0.000 0.000 0.000 0.049 0.062 0.063 0.077 0.000 0.005

0.000 0.000 0.005 0.009 0.000 0.000 0.000 0.009 0.000 0.000

0.005 0.000 0.009 0.005 0.005 0.000 0.005 0.000 0.074 0.068

0.062 0.074

>Methylation code ID 3173

0.012 0.000 0.000 0.000 0.000 0.006 0.000 0.000 0.000 0.000

0.000 0.000 0.006 0.000 0.006 0.024 0.000 0.000 0.012 0.000

0.078 0.064 0.054 0.071 0.008 0.000 0.008 0.008 0.000 0.023

0.000 0.000 0.000 0.016 0.000 0.000 0.039 0.000 0.000 0.000

0.000 0.000 0.000 0.000 0.060 0.054 0.058 0.061 0.007 0.000

0.000 0.000 0.000 0.007 0.000 0.007 0.000 0.035 0.000 0.000

0.007 0.000 0.007 0.007 0.000 0.007 0.000 0.000 0.045 0.062

0.070 0.073

>Methylation code ID 3173

0.008 0.000 0.008 0.008 0.000 0.024 0.000 0.000 0.000 0.008

0.000 0.000 0.049 0.000 0.000 0.000 0.000 0.000 0.000 0.000

0.066 0.056 0.045 0.061 0.005 0.000 0.000 0.000 0.005 0.005

0.000 0.005 0.000 0.021 0.000 0.000 0.005 0.000 0.011 0.005

0.000 0.005 0.000 0.000 0.051 0.064 0.070 0.080 0.014 0.000

0.000 0.000 0.000 0.007 0.000 0.000 0.000 0.000 0.000 0.000

0.007 0.000 0.007 0.028 0.000 0.000 0.007 0.000 0.069 0.070

0.052 0.071

>Methylation code ID 3173

0.016 0.000 0.000 0.000 0.000 0.008 0.000 0.000 0.000 0.000

0.000 0.000 0.000 0.000 0.008 0.064 0.000 0.000 0.008 0.000

0.072 0.050 0.049 0.059 0.006 0.000 0.000 0.000 0.000 0.012

0.000 0.000 0.000 0.006 0.000 0.000 0.043 0.000 0.012 0.000

0.000 0.000 0.000 0.000 0.082 0.042 0.069 0.062 0.000 0.000

0.000 0.000 0.007 0.007 0.000 0.000 0.000 0.036 0.000 0.000

0.007 0.000 0.007 0.000 0.000 0.022 0.000 0.000 0.053 0.057

0.062 0.075

>Methylation code ID 3173

0.000 0.000 0.000 0.000 0.010 0.010 0.010 0.000 0.000 0.038

0.000 0.000 0.010 0.000 0.000 0.000 0.000 0.038 0.000 0.010

0.066 0.049 0.046 0.048 0.000 0.000 0.000 0.000 0.000 0.000

0.000 0.013 0.000 0.000 0.013 0.000 0.013 0.000 0.000 0.118

0.000 0.013 0.000 0.000 0.030 0.038 0.047 0.049 0.000 0.000

0.000 0.000 0.000 0.000 0.000 0.000 0.000 0.009 0.000 0.000

0.021 0.000 0.017 0.000 0.000 0.000 0.004 0.000 0.093 0.041

0.097 0.051

>Methylation code ID 3175

0.013 0.000 0.000 0.000 0.006 0.000 0.006 0.006 0.000 0.000

0.000 0.013 0.000 0.013 0.006 0.000 0.000 0.019 0.000 0.000

0.063 0.077 0.049 0.061 0.007 0.000 0.000 0.000 0.007 0.000

0.000 0.000 0.000 0.022 0.015 0.007 0.000 0.000 0.007 0.015

0.007 0.000 0.000 0.000 0.059 0.062 0.076 0.048 0.000 0.013

0.000 0.000 0.004 0.004 0.000 0.000 0.004 0.000 0.000 0.000

0.009 0.000 0.004 0.004 0.004 0.000 0.000 0.000 0.088 0.039

0.081 0.078

>Methylation code ID 3175

0.012 0.000 0.000 0.006 0.006 0.000 0.000 0.000 0.006 0.006

0.000 0.006 0.000 0.006 0.006 0.006 0.000 0.012 0.000 0.000

0.059 0.058 0.080 0.067 0.010 0.005 0.000 0.000 0.005 0.000

0.000 0.000 0.000 0.005 0.005 0.005 0.000 0.000 0.015 0.010

0.000 0.000 0.000 0.000 0.069 0.087 0.058 0.059 0.000 0.004

0.000 0.009 0.004 0.004 0.000 0.000 0.000 0.000 0.000 0.000

0.004 0.000 0.013 0.000 0.004 0.004 0.000 0.000 0.072 0.070

0.072 0.072

>Methylation code ID 3175

0.007 0.000 0.007 0.015 0.000 0.000 0.000 0.000 0.007 0.015

0.000 0.000 0.000 0.007 0.007 0.015 0.000 0.000 0.000 0.000

0.073 0.049 0.069 0.062 0.011 0.006 0.000 0.000 0.000 0.000

0.000 0.000 0.000 0.000 0.000 0.006 0.000 0.000 0.017 0.006

0.006 0.000 0.000 0.000 0.077 0.102 0.051 0.052 0.005 0.000

0.000 0.016 0.000 0.011 0.000 0.000 0.000 0.000 0.000 0.000

0.000 0.000 0.016 0.000 0.005 0.005 0.000 0.000 0.072 0.094

0.077 0.031

>Methylation code ID 3175

0.005 0.000 0.005 0.015 0.000 0.010 0.000 0.000 0.000 0.000

0.000 0.000 0.000 0.000 0.015 0.000 0.005 0.005 0.000 0.000

0.075 0.086 0.077 0.037 0.007 0.000 0.007 0.015 0.000 0.000

0.000 0.000 0.007 0.015 0.000 0.000 0.000 0.000 0.007 0.015

0.007 0.000 0.000 0.000 0.072 0.047 0.069 0.064 0.013 0.006

0.000 0.000 0.000 0.000 0.000 0.000 0.000 0.000 0.000 0.000

0.000 0.000 0.019 0.006 0.006 0.000 0.000 0.000 0.079 0.113

0.055 0.036

>Methylation code ID 3175

0.007 0.007 0.007 0.007 0.000 0.000 0.000 0.000 0.000 0.014

0.000 0.000 0.000 0.000 0.000 0.014 0.014 0.007 0.007 0.000

0.058 0.064 0.059 0.066 0.019 0.000 0.000 0.000 0.000 0.009

0.009 0.000 0.000 0.000 0.000 0.000 0.000 0.009 0.009 0.009

0.009 0.000 0.000 0.009 0.088 0.047 0.059 0.055 0.005 0.000

0.005 0.011 0.000 0.011 0.000 0.000 0.000 0.000 0.011 0.005

0.000 0.000 0.011 0.000 0.000 0.000 0.000 0.000 0.071 0.064

0.085 0.055

>Methylation code ID 3175

0.005 0.005 0.010 0.010 0.000 0.005 0.000 0.005 0.005 0.000

0.000 0.005 0.005 0.000 0.000 0.000 0.005 0.000 0.005 0.000

0.061 0.082 0.065 0.059 0.000 0.000 0.000 0.000 0.000 0.006

0.006 0.000 0.006 0.006 0.006 0.000 0.006 0.006 0.012 0.000

0.012 0.006 0.000 0.000 0.076 0.063 0.046 0.079 0.000 0.005

0.005 0.000 0.000 0.010 0.000 0.000 0.005 0.005 0.005 0.000

0.005 0.005 0.005 0.000 0.000 0.000 0.005 0.000 0.044 0.078

0.074 0.080

>Methylation code ID 3175

0.005 0.005 0.000 0.015 0.005 0.000 0.010 0.005 0.005 0.000

0.000 0.000 0.005 0.010 0.000 0.000 0.000 0.000 0.000 0.000

0.067 0.068 0.059 0.074 0.000 0.000 0.000 0.000 0.000 0.000

0.007 0.000 0.007 0.007 0.000 0.015 0.000 0.007 0.015 0.007

0.015 0.000 0.000 0.007 0.065 0.073 0.044 0.061 0.007 0.000

0.000 0.000 0.000 0.007 0.000 0.007 0.000 0.000 0.007 0.007

0.007 0.000 0.013 0.000 0.013 0.000 0.000 0.000 0.058 0.070

0.079 0.061

>Methylation code ID 21046

0.023 0.000 0.000 0.000 0.008 0.015 0.000 0.000 0.000 0.015

0.000 0.000 0.015 0.000 0.000 0.000 0.000 0.008 0.015 0.000

0.058 0.066 0.057 0.053 0.014 0.007 0.000 0.000 0.014 0.014

0.000 0.000 0.000 0.020 0.000 0.000 0.014 0.000 0.000 0.007

0.000 0.000 0.000 0.000 0.060 0.062 0.070 0.053 0.005 0.005

0.000 0.000 0.000 0.005 0.000 0.000 0.000 0.015 0.000 0.000

0.005 0.000 0.015 0.010 0.000 0.000 0.000 0.000 0.068 0.080

0.058 0.067

>Methylation code ID 21127

0.011 0.000 0.005 0.005 0.000 0.016 0.000 0.000 0.000 0.011

0.000 0.005 0.005 0.000 0.005 0.000 0.005 0.000 0.000 0.000

0.040 0.067 0.077 0.080 0.000 0.000 0.006 0.018 0.000 0.000

0.000 0.000 0.006 0.012 0.000 0.000 0.012 0.000 0.006 0.006

0.012 0.000 0.000 0.000 0.075 0.067 0.056 0.060 0.006 0.006

0.000 0.000 0.000 0.000 0.006 0.000 0.006 0.000 0.000 0.000

0.000 0.006 0.028 0.000 0.006 0.000 0.006 0.000 0.068 0.065

0.066 0.068

>Methylation code ID 21127

0.012 0.000 0.006 0.006 0.000 0.012 0.000 0.000 0.000 0.018

0.000 0.006 0.006 0.000 0.006 0.000 0.006 0.000 0.000 0.000

0.046 0.066 0.076 0.069 0.000 0.000 0.006 0.011 0.000 0.000

0.000 0.000 0.006 0.006 0.000 0.000 0.017 0.000 0.006 0.011

0.011 0.000 0.000 0.000 0.075 0.052 0.054 0.079 0.006 0.006

0.000 0.000 0.000 0.000 0.006 0.000 0.006 0.000 0.000 0.000

0.012 0.006 0.023 0.000 0.006 0.000 0.000 0.000 0.057 0.057

0.055 0.093

>Methylation code ID 21127

0.004 0.004 0.000 0.000 0.000 0.000 0.009 0.000 0.004 0.000

0.000 0.000 0.009 0.004 0.018 0.000 0.004 0.000 0.000 0.000

0.060 0.056 0.066 0.093 0.021 0.000 0.007 0.000 0.000 0.007

0.000 0.000 0.000 0.021 0.000 0.007 0.007 0.000 0.007 0.000

0.014 0.000 0.000 0.000 0.052 0.063 0.058 0.069 0.000 0.000

0.006 0.006 0.000 0.000 0.000 0.000 0.000 0.006 0.000 0.000

0.025 0.000 0.006 0.012 0.012 0.000 0.000 0.000 0.071 0.052

0.055 0.081

>Methylation code ID 21127

0.010 0.005 0.000 0.000 0.000 0.005 0.010 0.000 0.000 0.010

0.000 0.000 0.015 0.000 0.005 0.005 0.000 0.000 0.000 0.000

0.055 0.065 0.072 0.076 0.012 0.000 0.000 0.000 0.000 0.000

0.000 0.000 0.000 0.012 0.000 0.000 0.012 0.000 0.012 0.006

0.012 0.006 0.000 0.006 0.075 0.065 0.039 0.078 0.005 0.000

0.000 0.000 0.005 0.000 0.000 0.000 0.000 0.005 0.000 0.000

0.019 0.000 0.014 0.005 0.000 0.005 0.000 0.000 0.078 0.077

0.039 0.084

>Methylation code ID 21127

0.005 0.005 0.000 0.000 0.000 0.005 0.005 0.000 0.000 0.015

0.000 0.000 0.015 0.000 0.010 0.005 0.000 0.000 0.000 0.000

0.064 0.077 0.066 0.061 0.005 0.000 0.000 0.005 0.000 0.000

0.000 0.000 0.000 0.005 0.000 0.000 0.010 0.000 0.010 0.010

0.005 0.010 0.000 0.005 0.073 0.069 0.053 0.071 0.005 0.000

0.000 0.000 0.005 0.000 0.000 0.000 0.000 0.005 0.000 0.000

0.016 0.000 0.011 0.005 0.005 0.005 0.000 0.000 0.072 0.079

0.043 0.081

>Methylation code ID 21127

0.006 0.000 0.006 0.006 0.000 0.006 0.000 0.000 0.012 0.000

0.000 0.000 0.000 0.012 0.012 0.006 0.000 0.006 0.000 0.006

0.050 0.057 0.067 0.080 0.006 0.000 0.000 0.006 0.000 0.000

0.000 0.000 0.006 0.012 0.000 0.000 0.000 0.000 0.012 0.012

0.012 0.006 0.000 0.000 0.059 0.069 0.074 0.061 0.004 0.004

0.000 0.004 0.000 0.004 0.000 0.000 0.008 0.008 0.000 0.000

0.004 0.000 0.008 0.000 0.000 0.000 0.004 0.000 0.078 0.082

0.052 0.073

>Methylation code ID 21127

0.005 0.000 0.000 0.005 0.000 0.005 0.000 0.000 0.005 0.011

0.000 0.000 0.000 0.005 0.016 0.016 0.000 0.000 0.000 0.000

0.055 0.068 0.068 0.072 0.000 0.000 0.000 0.007 0.000 0.013

0.000 0.000 0.000 0.007 0.000 0.013 0.013 0.000 0.000 0.013

0.000 0.013 0.000 0.007 0.056 0.065 0.058 0.068 0.007 0.000

0.000 0.007 0.013 0.007 0.000 0.000 0.000 0.007 0.000 0.000

0.007 0.000 0.007 0.007 0.013 0.000 0.000 0.000 0.069 0.079

0.053 0.061

>Methylation code ID 21127

0.006 0.000 0.000 0.006 0.000 0.006 0.000 0.000 0.006 0.011

0.000 0.000 0.000 0.006 0.022 0.011 0.000 0.000 0.000 0.000

0.065 0.059 0.067 0.071 0.000 0.000 0.000 0.007 0.000 0.013

0.000 0.000 0.000 0.007 0.000 0.013 0.013 0.000 0.000 0.013

0.000 0.013 0.000 0.007 0.061 0.060 0.059 0.067 0.007 0.000

0.000 0.007 0.007 0.007 0.000 0.000 0.000 0.007 0.000 0.000

0.007 0.000 0.007 0.007 0.020 0.000 0.000 0.000 0.059 0.074

0.059 0.070

>Methylation code ID 21127

0.020 0.000 0.000 0.000 0.014 0.014 0.000 0.000 0.007 0.014

0.000 0.000 0.007 0.000 0.000 0.000 0.007 0.000 0.000 0.000

0.060 0.066 0.068 0.058 0.000 0.000 0.000 0.010 0.005 0.000

0.000 0.000 0.000 0.005 0.000 0.000 0.005 0.000 0.020 0.020

0.000 0.000 0.000 0.000 0.059 0.062 0.066 0.080 0.006 0.000

0.000 0.000 0.000 0.006 0.000 0.000 0.006 0.006 0.000 0.006

0.000 0.000 0.018 0.012 0.000 0.012 0.000 0.000 0.056 0.097

0.075 0.033

>Methylation code ID 21135

0.009 0.004 0.000 0.000 0.004 0.009 0.000 0.000 0.000 0.004

0.000 0.000 0.009 0.004 0.009 0.004 0.000 0.000 0.000 0.000

0.072 0.052 0.071 0.082 0.018 0.006 0.000 0.000 0.000 0.006

0.000 0.000 0.000 0.012 0.000 0.006 0.012 0.000 0.006 0.006

0.000 0.006 0.000 0.000 0.073 0.058 0.070 0.053 0.013 0.004

0.000 0.000 0.004 0.000 0.000 0.000 0.000 0.009 0.000 0.000

0.000 0.000 0.013 0.004 0.004 0.000 0.000 0.000 0.072 0.067

0.069 0.073

>Methylation code ID 21135

0.020 0.000 0.000 0.000 0.000 0.007 0.000 0.000 0.000 0.013

0.000 0.007 0.013 0.000 0.007 0.013 0.000 0.007 0.000 0.000

0.069 0.065 0.066 0.047 0.008 0.004 0.000 0.000 0.004 0.000

0.000 0.000 0.000 0.008 0.000 0.000 0.008 0.000 0.012 0.004

0.004 0.000 0.000 0.000 0.065 0.065 0.063 0.089 0.009 0.005

0.000 0.000 0.005 0.009 0.000 0.000 0.000 0.005 0.000 0.000

0.009 0.000 0.009 0.005 0.000 0.000 0.000 0.000 0.074 0.047

0.069 0.087

>Methylation code ID 21135

0.008 0.004 0.000 0.000 0.004 0.008 0.000 0.000 0.000 0.004

0.000 0.000 0.008 0.000 0.012 0.004 0.000 0.000 0.000 0.000

0.074 0.052 0.067 0.088 0.028 0.000 0.000 0.000 0.000 0.007

0.000 0.000 0.000 0.014 0.000 0.000 0.014 0.000 0.007 0.014

0.000 0.007 0.000 0.000 0.066 0.065 0.063 0.047 0.008 0.004

0.000 0.000 0.004 0.000 0.000 0.000 0.000 0.008 0.000 0.000

0.008 0.000 0.012 0.004 0.000 0.000 0.000 0.000 0.067 0.060

0.065 0.093

>Methylation code ID 21135

0.015 0.000 0.000 0.000 0.000 0.008 0.000 0.000 0.000 0.015

0.000 0.000 0.008 0.000 0.000 0.023 0.008 0.008 0.015 0.000

0.068 0.037 0.071 0.056 0.006 0.006 0.000 0.000 0.006 0.018

0.000 0.000 0.000 0.006 0.000 0.000 0.024 0.000 0.006 0.006

0.000 0.000 0.000 0.000 0.053 0.066 0.060 0.078 0.009 0.000

0.005 0.005 0.005 0.000 0.000 0.000 0.000 0.009 0.000 0.000

0.000 0.000 0.014 0.005 0.000 0.000 0.000 0.000 0.059 0.077

0.073 0.072

>Methylation code ID 21135

0.009 0.000 0.000 0.004 0.000 0.004 0.000 0.000 0.000 0.004

0.000 0.000 0.000 0.004 0.013 0.013 0.000 0.000 0.004 0.000

0.063 0.073 0.072 0.068 0.040 0.000 0.000 0.000 0.000 0.016

0.000 0.000 0.000 0.000 0.000 0.000 0.008 0.000 0.016 0.008

0.000 0.008 0.000 0.000 0.038 0.065 0.067 0.068 0.010 0.000

0.000 0.010 0.000 0.005 0.000 0.000 0.000 0.010 0.000 0.000

0.005 0.005 0.010 0.005 0.000 0.000 0.000 0.000 0.073 0.075

0.056 0.067

>Methylation code ID 21135

0.005 0.005 0.000 0.010 0.000 0.015 0.005 0.000 0.000 0.000

0.005 0.000 0.000 0.000 0.005 0.005 0.010 0.000 0.000 0.000

0.054 0.066 0.084 0.062 0.009 0.004 0.000 0.004 0.000 0.009

0.004 0.000 0.000 0.004 0.000 0.000 0.000 0.004 0.013 0.000

0.004 0.000 0.000 0.000 0.060 0.073 0.061 0.083 0.000 0.000

0.010 0.000 0.000 0.015 0.005 0.005 0.005 0.000 0.000 0.000

0.005 0.000 0.010 0.000 0.000 0.000 0.000 0.000 0.070 0.092

0.054 0.063

>Methylation code ID 21139

0.005 0.000 0.005 0.000 0.000 0.000 0.005 0.000 0.005 0.005

0.000 0.016 0.000 0.000 0.005 0.016 0.000 0.000 0.000 0.005

0.063 0.055 0.077 0.069 0.000 0.000 0.000 0.000 0.000 0.007

0.000 0.021 0.000 0.007 0.000 0.000 0.021 0.000 0.007 0.000

0.021 0.000 0.000 0.000 0.059 0.063 0.071 0.058 0.000 0.000

0.000 0.006 0.006 0.000 0.000 0.000 0.000 0.012 0.000 0.000

0.000 0.017 0.012 0.006 0.000 0.006 0.000 0.000 0.058 0.069

0.070 0.073

>Methylation code ID 21139

0.005 0.000 0.000 0.000 0.005 0.000 0.005 0.000 0.000 0.000

0.000 0.005 0.000 0.000 0.021 0.011 0.011 0.000 0.000 0.005

0.071 0.076 0.062 0.056 0.012 0.000 0.000 0.000 0.000 0.012

0.000 0.012 0.000 0.006 0.000 0.000 0.019 0.000 0.006 0.006

0.006 0.000 0.000 0.000 0.056 0.065 0.059 0.072 0.006 0.000

0.000 0.000 0.006 0.000 0.011 0.000 0.000 0.011 0.000 0.000

0.006 0.011 0.006 0.006 0.000 0.006 0.000 0.000 0.076 0.059

0.055 0.075

>Methylation code ID 21139

0.005 0.000 0.000 0.005 0.005 0.000 0.010 0.000 0.000 0.010

0.000 0.000 0.005 0.010 0.005 0.005 0.000 0.005 0.000 0.000

0.072 0.059 0.060 0.075 0.005 0.000 0.000 0.000 0.005 0.000

0.005 0.000 0.000 0.000 0.000 0.005 0.000 0.000 0.016 0.016

0.011 0.000 0.000 0.005 0.073 0.084 0.057 0.048 0.013 0.000

0.000 0.000 0.000 0.007 0.000 0.013 0.000 0.007 0.000 0.000

0.020 0.000 0.007 0.007 0.007 0.000 0.000 0.000 0.054 0.066

0.060 0.075

>Methylation code ID 21139

0.019 0.000 0.000 0.000 0.000 0.006 0.000 0.013 0.000 0.006

0.000 0.000 0.019 0.000 0.006 0.006 0.006 0.000 0.000 0.000

0.053 0.064 0.060 0.073 0.005 0.000 0.000 0.005 0.005 0.000

0.009 0.000 0.000 0.009 0.000 0.000 0.005 0.009 0.009 0.005

0.000 0.000 0.000 0.000 0.068 0.058 0.065 0.083 0.006 0.000

0.000 0.000 0.006 0.000 0.006 0.000 0.000 0.000 0.000 0.006

0.000 0.000 0.017 0.011 0.011 0.000 0.000 0.006 0.072 0.085

0.056 0.052

>Methylation code ID 21139

0.022 0.000 0.000 0.000 0.000 0.007 0.000 0.000 0.000 0.007

0.007 0.000 0.015 0.000 0.007 0.015 0.007 0.000 0.000 0.000

0.060 0.068 0.058 0.059 0.004 0.004 0.004 0.004 0.004 0.000

0.008 0.000 0.000 0.004 0.000 0.000 0.008 0.004 0.008 0.000

0.000 0.000 0.000 0.000 0.081 0.066 0.064 0.068 0.005 0.000

0.005 0.000 0.005 0.000 0.000 0.005 0.000 0.000 0.000 0.000

0.000 0.000 0.015 0.010 0.010 0.000 0.000 0.000 0.065 0.081

0.066 0.065

>Methylation code ID 21139

0.034 0.000 0.011 0.000 0.000 0.011 0.000 0.023 0.000 0.000

0.011 0.000 0.000 0.000 0.000 0.023 0.011 0.011 0.000 0.000

0.045 0.043 0.059 0.050 0.000 0.005 0.005 0.005 0.000 0.000

0.000 0.000 0.000 0.010 0.000 0.000 0.010 0.005 0.010 0.005

0.005 0.000 0.000 0.000 0.073 0.056 0.075 0.070 0.000 0.005

0.005 0.000 0.000 0.005 0.005 0.005 0.000 0.000 0.000 0.005

0.000 0.000 0.014 0.005 0.000 0.000 0.000 0.005 0.067 0.062

0.079 0.073

>Methylation code ID 21139

0.000 0.005 0.000 0.000 0.000 0.010 0.005 0.005 0.000 0.000

0.000 0.005 0.010 0.000 0.010 0.000 0.000 0.005 0.000 0.005

0.072 0.052 0.066 0.086 0.008 0.000 0.008 0.000 0.008 0.008

0.008 0.017 0.000 0.008 0.000 0.000 0.000 0.000 0.000 0.008

0.008 0.008 0.000 0.000 0.064 0.052 0.073 0.051 0.000 0.000

0.006 0.000 0.000 0.006 0.000 0.000 0.000 0.012 0.000 0.000

0.006 0.000 0.006 0.012 0.012 0.000 0.006 0.000 0.063 0.065

0.066 0.071

>Methylation code ID 21139

0.005 0.000 0.022 0.005 0.005 0.005 0.005 0.000 0.000 0.005

0.000 0.000 0.000 0.000 0.000 0.000 0.005 0.000 0.005 0.000

0.072 0.058 0.070 0.068 0.000 0.000 0.000 0.000 0.000 0.011

0.000 0.011 0.006 0.006 0.000 0.000 0.000 0.000 0.006 0.006

0.017 0.000 0.011 0.000 0.073 0.066 0.064 0.058 0.000 0.005

0.000 0.000 0.000 0.019 0.000 0.000 0.000 0.005 0.000 0.000

0.009 0.000 0.014 0.000 0.000 0.005 0.000 0.000 0.070 0.067

0.060 0.080

>Methylation code ID 21139

0.000 0.000 0.015 0.005 0.010 0.010 0.005 0.000 0.000 0.005

0.000 0.000 0.000 0.000 0.000 0.000 0.005 0.000 0.005 0.000

0.067 0.051 0.076 0.080 0.007 0.000 0.000 0.000 0.000 0.014

0.000 0.014 0.007 0.007 0.000 0.000 0.000 0.000 0.000 0.014

0.014 0.000 0.014 0.000 0.063 0.061 0.064 0.055 0.000 0.000

0.000 0.000 0.004 0.013 0.004 0.000 0.000 0.004 0.000 0.000

0.004 0.000 0.013 0.000 0.000 0.004 0.004 0.000 0.068 0.066

0.075 0.073

>Methylation code ID 21139

0.000 0.000 0.010 0.005 0.000 0.005 0.000 0.000 0.000 0.000

0.005 0.000 0.000 0.005 0.010 0.000 0.010 0.000 0.010 0.000

0.070 0.062 0.061 0.079 0.000 0.000 0.000 0.007 0.000 0.022

0.000 0.000 0.007 0.007 0.000 0.000 0.000 0.007 0.007 0.007

0.015 0.007 0.000 0.000 0.074 0.046 0.065 0.060 0.005 0.000

0.010 0.000 0.000 0.010 0.005 0.000 0.005 0.005 0.000 0.000

0.000 0.000 0.010 0.005 0.000 0.000 0.000 0.000 0.044 0.070

0.081 0.085

>Methylation code ID 21139

0.000 0.000 0.000 0.008 0.000 0.032 0.000 0.000 0.016 0.008

0.000 0.000 0.008 0.000 0.000 0.016 0.016 0.000 0.000 0.000

0.053 0.058 0.054 0.063 0.000 0.000 0.000 0.011 0.000 0.011

0.000 0.000 0.011 0.000 0.000 0.000 0.011 0.006 0.006 0.006

0.000 0.000 0.000 0.006 0.073 0.048 0.070 0.076 0.000 0.000

0.000 0.005 0.000 0.005 0.005 0.000 0.005 0.000 0.000 0.005

0.000 0.000 0.016 0.000 0.005 0.010 0.000 0.000 0.069 0.063

0.072 0.072

>Methylation code ID 21139

0.005 0.000 0.000 0.005 0.000 0.005 0.005 0.000 0.000 0.011

0.000 0.005 0.000 0.000 0.016 0.005 0.005 0.005 0.000 0.000

0.052 0.072 0.072 0.069 0.000 0.000 0.000 0.000 0.008 0.031

0.000 0.000 0.008 0.008 0.000 0.000 0.008 0.000 0.008 0.000

0.016 0.000 0.000 0.000 0.058 0.076 0.053 0.060 0.005 0.005

0.000 0.010 0.000 0.010 0.000 0.000 0.005 0.000 0.000 0.000

0.010 0.000 0.005 0.005 0.000 0.000 0.000 0.005 0.077 0.061

0.068 0.069

>Methylation code ID 21139

0.005 0.005 0.005 0.015 0.000 0.005 0.000 0.000 0.005 0.005

0.000 0.000 0.005 0.000 0.005 0.005 0.005 0.000 0.000 0.000

0.076 0.062 0.071 0.059 0.006 0.000 0.000 0.006 0.000 0.006

0.006 0.000 0.000 0.022 0.000 0.006 0.000 0.000 0.011 0.006

0.006 0.000 0.000 0.000 0.059 0.068 0.074 0.059 0.000 0.000

0.007 0.000 0.007 0.014 0.000 0.000 0.007 0.000 0.000 0.000

0.000 0.000 0.007 0.007 0.014 0.000 0.000 0.000 0.061 0.075

0.065 0.071

>Methylation code ID 21139

0.005 0.000 0.005 0.005 0.011 0.000 0.005 0.000 0.000 0.005

0.000 0.000 0.005 0.005 0.005 0.005 0.005 0.005 0.000 0.000

0.071 0.064 0.062 0.065 0.006 0.000 0.006 0.000 0.006 0.000

0.000 0.006 0.006 0.012 0.000 0.000 0.012 0.000 0.006 0.012

0.006 0.000 0.000 0.000 0.070 0.068 0.047 0.070 0.000 0.000

0.006 0.000 0.006 0.000 0.000 0.000 0.000 0.006 0.000 0.000

0.012 0.012 0.006 0.018 0.000 0.000 0.000 0.000 0.064 0.072

0.055 0.077

>Methylation code ID 21139

0.000 0.000 0.005 0.000 0.010 0.005 0.005 0.000 0.005 0.010

0.000 0.000 0.010 0.005 0.005 0.000 0.000 0.000 0.000 0.000

0.087 0.054 0.066 0.067 0.008 0.008 0.000 0.008 0.008 0.000

0.000 0.000 0.008 0.008 0.000 0.000 0.000 0.008 0.000 0.023

0.016 0.000 0.000 0.000 0.077 0.052 0.058 0.052 0.000 0.000

0.000 0.005 0.000 0.000 0.000 0.000 0.005 0.005 0.000 0.000

0.010 0.000 0.010 0.015 0.000 0.010 0.000 0.000 0.071 0.058

0.065 0.079

>Methylation code ID 21139

0.000 0.000 0.000 0.005 0.010 0.005 0.000 0.000 0.000 0.000

0.000 0.000 0.005 0.005 0.005 0.000 0.000 0.010 0.010 0.005

0.060 0.070 0.077 0.064 0.000 0.000 0.014 0.000 0.007 0.021

0.000 0.007 0.000 0.000 0.000 0.000 0.007 0.000 0.000 0.021

0.000 0.000 0.000 0.007 0.049 0.067 0.074 0.061 0.007 0.000

0.000 0.000 0.007 0.007 0.000 0.007 0.000 0.000 0.007 0.000

0.007 0.000 0.007 0.007 0.000 0.007 0.000 0.000 0.056 0.069

0.072 0.073

>Methylation code ID 21139

0.000 0.000 0.013 0.000 0.013 0.000 0.000 0.000 0.000 0.006

0.000 0.000 0.013 0.000 0.000 0.019 0.000 0.006 0.000 0.006

0.047 0.072 0.063 0.073 0.000 0.006 0.000 0.000 0.012 0.000

0.006 0.006 0.000 0.006 0.006 0.000 0.006 0.000 0.006 0.012

0.006 0.000 0.000 0.000 0.070 0.069 0.060 0.063 0.000 0.006

0.000 0.000 0.011 0.000 0.000 0.006 0.000 0.006 0.000 0.000

0.000 0.006 0.006 0.006 0.000 0.017 0.000 0.000 0.048 0.068

0.073 0.084

>Methylation code ID 21148

0.014 0.000 0.000 0.000 0.000 0.000 0.000 0.000 0.000 0.009

0.000 0.000 0.005 0.009 0.009 0.005 0.000 0.000 0.005 0.005

0.069 0.063 0.065 0.076 0.000 0.006 0.000 0.000 0.006 0.011

0.000 0.000 0.000 0.011 0.000 0.000 0.017 0.000 0.006 0.006

0.006 0.006 0.000 0.000 0.071 0.056 0.055 0.077 0.015 0.000

0.000 0.000 0.005 0.005 0.000 0.000 0.000 0.010 0.000 0.000

0.005 0.005 0.010 0.005 0.000 0.000 0.000 0.000 0.047 0.072

0.069 0.085

>Methylation code ID 21148

0.015 0.000 0.000 0.000 0.005 0.005 0.000 0.000 0.000 0.015

0.000 0.000 0.005 0.005 0.010 0.005 0.000 0.000 0.000 0.000

0.047 0.071 0.065 0.087 0.013 0.000 0.000 0.000 0.000 0.000

0.000 0.000 0.000 0.004 0.000 0.000 0.009 0.004 0.009 0.004

0.000 0.000 0.009 0.004 0.071 0.070 0.066 0.069 0.000 0.000

0.000 0.000 0.008 0.017 0.000 0.000 0.000 0.025 0.000 0.000

0.025 0.000 0.000 0.008 0.008 0.008 0.000 0.000 0.058 0.058

0.042 0.076

>Methylation code ID 21148

0.000 0.006 0.000 0.000 0.006 0.012 0.000 0.000 0.000 0.012

0.000 0.000 0.018 0.000 0.006 0.006 0.006 0.006 0.000 0.000

0.068 0.064 0.050 0.075 0.014 0.000 0.000 0.005 0.010 0.000

0.000 0.000 0.000 0.005 0.000 0.000 0.005 0.005 0.010 0.005

0.000 0.005 0.000 0.000 0.051 0.076 0.069 0.075 0.010 0.000

0.000 0.000 0.000 0.000 0.000 0.000 0.000 0.010 0.000 0.000

0.010 0.005 0.010 0.010 0.000 0.000 0.005 0.000 0.060 0.064

0.073 0.077

>Methylation code ID 21148

0.007 0.000 0.000 0.000 0.000 0.014 0.007 0.007 0.000 0.000

0.000 0.000 0.014 0.014 0.007 0.000 0.014 0.007 0.000 0.000

0.068 0.045 0.073 0.059 0.010 0.000 0.000 0.000 0.000 0.005

0.000 0.000 0.000 0.000 0.000 0.000 0.005 0.005 0.020 0.015

0.005 0.000 0.000 0.000 0.075 0.073 0.064 0.057 0.018 0.000

0.009 0.000 0.000 0.000 0.005 0.000 0.000 0.000 0.000 0.000

0.009 0.000 0.014 0.000 0.000 0.000 0.000 0.000 0.073 0.055

0.086 0.064

>Methylation code ID 21148

0.018 0.000 0.000 0.000 0.000 0.004 0.004 0.000 0.000 0.000

0.000 0.000 0.013 0.000 0.018 0.000 0.000 0.000 0.000 0.000

0.077 0.056 0.083 0.059 0.006 0.000 0.000 0.000 0.000 0.012

0.006 0.006 0.000 0.000 0.000 0.000 0.018 0.012 0.006 0.000

0.000 0.006 0.000 0.000 0.078 0.048 0.088 0.046 0.017 0.006

0.000 0.000 0.000 0.000 0.000 0.000 0.000 0.000 0.000 0.000

0.006 0.000 0.017 0.017 0.006 0.000 0.000 0.000 0.088 0.080

0.073 0.025

>Methylation code ID 21148

0.009 0.005 0.000 0.005 0.000 0.014 0.000 0.000 0.000 0.005

0.000 0.000 0.000 0.000 0.009 0.005 0.000 0.009 0.000 0.000

0.074 0.057 0.062 0.079 0.027 0.000 0.000 0.000 0.000 0.018

0.000 0.000 0.000 0.009 0.000 0.000 0.009 0.009 0.009 0.018

0.000 0.000 0.000 0.000 0.055 0.056 0.047 0.077 0.005 0.000

0.000 0.005 0.000 0.005 0.000 0.000 0.000 0.005 0.000 0.000

0.005 0.005 0.010 0.005 0.000 0.000 0.010 0.000 0.071 0.075

0.061 0.074

>Methylation code ID 21148

0.016 0.000 0.000 0.000 0.000 0.016 0.000 0.000 0.000 0.008

0.000 0.000 0.008 0.008 0.000 0.039 0.000 0.000 0.008 0.000

0.054 0.056 0.062 0.059 0.016 0.000 0.000 0.000 0.000 0.008

0.000 0.000 0.000 0.004 0.000 0.000 0.004 0.000 0.012 0.004

0.000 0.000 0.004 0.000 0.052 0.078 0.074 0.078 0.013 0.007

0.000 0.007 0.000 0.007 0.000 0.000 0.000 0.007 0.000 0.000

0.000 0.013 0.007 0.000 0.000 0.013 0.000 0.000 0.068 0.055

0.065 0.071

>Methylation code ID 21148

0.013 0.006 0.000 0.006 0.000 0.006 0.000 0.000 0.000 0.013

0.000 0.000 0.000 0.019 0.006 0.000 0.000 0.006 0.000 0.000

0.059 0.054 0.066 0.078 0.016 0.000 0.000 0.000 0.000 0.008

0.000 0.000 0.000 0.008 0.000 0.000 0.008 0.008 0.000 0.049

0.000 0.000 0.008 0.000 0.051 0.055 0.052 0.070 0.022 0.000

0.000 0.000 0.000 0.004 0.000 0.000 0.000 0.000 0.000 0.000

0.004 0.000 0.013 0.004 0.000 0.000 0.004 0.000 0.046 0.075

0.081 0.078

>Methylation code ID 21148

0.014 0.000 0.000 0.000 0.000 0.000 0.000 0.000 0.000 0.007

0.000 0.000 0.014 0.000 0.007 0.034 0.007 0.000 0.007 0.000

0.065 0.066 0.060 0.053 0.018 0.005 0.000 0.000 0.000 0.009

0.000 0.000 0.000 0.000 0.000 0.000 0.014 0.000 0.014 0.000

0.000 0.000 0.000 0.000 0.068 0.056 0.078 0.072 0.021 0.000

0.000 0.007 0.000 0.007 0.007 0.000 0.000 0.007 0.000 0.000

0.000 0.021 0.007 0.000 0.000 0.000 0.000 0.000 0.047 0.061

0.063 0.083

>Methylation code ID 21148

0.017 0.000 0.000 0.006 0.000 0.006 0.017 0.000 0.000 0.006

0.000 0.000 0.000 0.017 0.006 0.000 0.000 0.000 0.000 0.000

0.045 0.065 0.068 0.083 0.016 0.000 0.000 0.000 0.000 0.000

0.000 0.016 0.000 0.008 0.000 0.000 0.008 0.000 0.008 0.039

0.008 0.000 0.000 0.000 0.054 0.062 0.049 0.068 0.020 0.005

0.000 0.000 0.000 0.005 0.000 0.000 0.000 0.000 0.000 0.000

0.015 0.000 0.010 0.005 0.000 0.000 0.000 0.000 0.067 0.057

0.083 0.066

>Methylation code ID 21152

0.015 0.010 0.000 0.000 0.000 0.000 0.000 0.000 0.000 0.005

0.000 0.005 0.000 0.000 0.010 0.010 0.005 0.000 0.000 0.005

0.082 0.062 0.053 0.073 0.020 0.007 0.000 0.000 0.000 0.007

0.007 0.000 0.000 0.000 0.000 0.000 0.007 0.020 0.007 0.000

0.013 0.000 0.000 0.000 0.073 0.065 0.058 0.051 0.007 0.000

0.000 0.000 0.000 0.000 0.000 0.000 0.000 0.020 0.000 0.007

0.007 0.007 0.007 0.013 0.007 0.007 0.000 0.000 0.057 0.053

0.075 0.068

>Methylation code ID 21152

0.019 0.000 0.000 0.006 0.000 0.000 0.000 0.000 0.000 0.012

0.000 0.006 0.006 0.000 0.012 0.012 0.006 0.000 0.000 0.000

0.066 0.059 0.064 0.065 0.014 0.000 0.000 0.000 0.000 0.005

0.000 0.000 0.000 0.009 0.000 0.000 0.000 0.000 0.019 0.005

0.005 0.000 0.000 0.005 0.057 0.072 0.079 0.064 0.012 0.006

0.000 0.000 0.000 0.012 0.006 0.000 0.000 0.000 0.000 0.000

0.006 0.012 0.006 0.006 0.006 0.000 0.000 0.000 0.065 0.060

0.076 0.061

>Methylation code ID 21152

0.039 0.000 0.007 0.007 0.000 0.000 0.000 0.000 0.000 0.000

0.000 0.000 0.007 0.000 0.007 0.007 0.007 0.000 0.000 0.000

0.062 0.074 0.055 0.064 0.000 0.000 0.000 0.004 0.000 0.004

0.000 0.000 0.000 0.004 0.000 0.000 0.017 0.000 0.021 0.000

0.004 0.000 0.000 0.000 0.059 0.074 0.073 0.073 0.006 0.000

0.000 0.000 0.000 0.017 0.006 0.000 0.000 0.006 0.000 0.000

0.000 0.000 0.017 0.011 0.000 0.000 0.000 0.000 0.051 0.069

0.081 0.072

>Methylation code ID 21152

0.047 0.000 0.012 0.000 0.000 0.000 0.000 0.000 0.000 0.024

0.000 0.000 0.024 0.000 0.000 0.012 0.012 0.000 0.000 0.000

0.039 0.067 0.052 0.045 0.000 0.005 0.000 0.005 0.000 0.000

0.000 0.000 0.000 0.000 0.000 0.000 0.024 0.005 0.010 0.010

0.005 0.000 0.000 0.000 0.060 0.073 0.054 0.084 0.012 0.000

0.000 0.000 0.000 0.000 0.006 0.000 0.000 0.006 0.000 0.000

0.012 0.000 0.018 0.012 0.000 0.000 0.000 0.000 0.064 0.069

0.085 0.050

>Methylation code ID 21166

0.006 0.000 0.000 0.012 0.000 0.012 0.000 0.000 0.000 0.012

0.000 0.000 0.006 0.006 0.006 0.012 0.000 0.006 0.000 0.000

0.057 0.063 0.068 0.068 0.009 0.004 0.004 0.004 0.000 0.004

0.000 0.000 0.004 0.000 0.000 0.000 0.009 0.000 0.004 0.009

0.000 0.000 0.004 0.000 0.064 0.074 0.063 0.077 0.009 0.000

0.000 0.005 0.000 0.005 0.000 0.000 0.000 0.000 0.000 0.000

0.009 0.005 0.019 0.000 0.005 0.000 0.000 0.000 0.091 0.061

0.077 0.048

>Methylation code ID 21166

0.011 0.000 0.000 0.000 0.000 0.017 0.000 0.000 0.006 0.006

0.000 0.000 0.000 0.006 0.011 0.011 0.000 0.006 0.000 0.000

0.076 0.083 0.022 0.079 0.013 0.000 0.000 0.000 0.004 0.008

0.000 0.000 0.000 0.000 0.000 0.000 0.004 0.000 0.013 0.008

0.000 0.000 0.004 0.000 0.074 0.075 0.043 0.087 0.015 0.000

0.000 0.005 0.005 0.015 0.000 0.000 0.000 0.005 0.000 0.000

0.000 0.005 0.010 0.000 0.000 0.000 0.000 0.000 0.043 0.067

0.081 0.081

>Methylation code ID 21166

0.000 0.007 0.014 0.000 0.000 0.007 0.007 0.007 0.000 0.000

0.000 0.000 0.021 0.007 0.000 0.000 0.000 0.014 0.000 0.000

0.067 0.044 0.080 0.058 0.005 0.005 0.005 0.000 0.000 0.000

0.000 0.005 0.000 0.000 0.000 0.000 0.009 0.000 0.009 0.005

0.009 0.000 0.000 0.005 0.090 0.042 0.078 0.067 0.006 0.000

0.000 0.000 0.000 0.006 0.006 0.000 0.000 0.000 0.000 0.000

0.006 0.000 0.022 0.006 0.011 0.000 0.000 0.000 0.069 0.078

0.090 0.036

>Methylation code ID 21166

0.007 0.007 0.007 0.000 0.007 0.007 0.007 0.007 0.000 0.007

0.000 0.000 0.028 0.000 0.000 0.000 0.000 0.007 0.000 0.000

0.068 0.056 0.070 0.050 0.005 0.000 0.005 0.000 0.005 0.000

0.000 0.005 0.000 0.000 0.000 0.000 0.014 0.000 0.009 0.009

0.005 0.000 0.000 0.005 0.088 0.061 0.076 0.047 0.005 0.000

0.000 0.000 0.005 0.000 0.005 0.000 0.000 0.000 0.000 0.000

0.010 0.000 0.016 0.005 0.010 0.000 0.000 0.000 0.086 0.048

0.095 0.046

>Methylation code ID 21166

0.007 0.007 0.000 0.000 0.014 0.007 0.007 0.007 0.000 0.014

0.000 0.000 0.021 0.000 0.000 0.000 0.000 0.007 0.000 0.000

0.064 0.047 0.077 0.053 0.005 0.000 0.005 0.000 0.005 0.000

0.000 0.000 0.000 0.000 0.000 0.000 0.016 0.000 0.005 0.022

0.005 0.000 0.000 0.005 0.071 0.062 0.073 0.056 0.005 0.000

0.000 0.000 0.005 0.000 0.005 0.000 0.000 0.005 0.000 0.000

0.015 0.000 0.015 0.000 0.010 0.000 0.000 0.000 0.077 0.058

0.083 0.056

>Methylation code ID 21166

0.006 0.000 0.000 0.000 0.006 0.011 0.000 0.000 0.000 0.000

0.000 0.000 0.011 0.006 0.011 0.017 0.006 0.000 0.000 0.000

0.084 0.065 0.047 0.063 0.008 0.000 0.000 0.000 0.004 0.004

0.000 0.000 0.000 0.004 0.000 0.000 0.017 0.000 0.013 0.000

0.000 0.004 0.000 0.000 0.070 0.059 0.072 0.077 0.010 0.000

0.000 0.000 0.010 0.005 0.000 0.000 0.000 0.010 0.000 0.000

0.010 0.000 0.010 0.005 0.000 0.000 0.000 0.000 0.053 0.062

0.076 0.086

>Methylation code ID 21206

0.005 0.005 0.000 0.005 0.000 0.011 0.000 0.000 0.011 0.000

0.000 0.000 0.011 0.005 0.005 0.005 0.000 0.000 0.000 0.000

0.084 0.067 0.051 0.067 0.014 0.005 0.000 0.000 0.000 0.009

0.000 0.000 0.000 0.005 0.000 0.000 0.000 0.000 0.023 0.005

0.000 0.000 0.000 0.000 0.063 0.063 0.085 0.064 0.016 0.005

0.000 0.011 0.000 0.011 0.000 0.000 0.000 0.000 0.000 0.000

0.000 0.005 0.011 0.000 0.000 0.000 0.000 0.000 0.068 0.053

0.072 0.081

>Methylation code ID 21206

0.020 0.007 0.007 0.007 0.000 0.000 0.000 0.000 0.000 0.000

0.000 0.000 0.007 0.007 0.007 0.007 0.000 0.007 0.000 0.000

0.079 0.053 0.076 0.053 0.006 0.006 0.000 0.000 0.000 0.000

0.000 0.000 0.000 0.006 0.000 0.000 0.012 0.012 0.006 0.012

0.006 0.000 0.006 0.000 0.074 0.057 0.071 0.059 0.008 0.008

0.000 0.000 0.000 0.008 0.000 0.000 0.000 0.004 0.000 0.000

0.004 0.000 0.012 0.004 0.000 0.000 0.000 0.000 0.052 0.073

0.074 0.087

>Methylation code ID 21206

0.007 0.000 0.000 0.007 0.000 0.000 0.000 0.000 0.000 0.000

0.000 0.000 0.015 0.015 0.007 0.015 0.007 0.000 0.007 0.000

0.069 0.048 0.071 0.064 0.004 0.004 0.000 0.000 0.000 0.008

0.000 0.000 0.000 0.004 0.000 0.000 0.004 0.008 0.012 0.004

0.000 0.000 0.004 0.000 0.056 0.083 0.073 0.070 0.011 0.000

0.006 0.006 0.000 0.011 0.000 0.000 0.000 0.000 0.000 0.000

0.006 0.000 0.011 0.006 0.000 0.006 0.000 0.000 0.084 0.039

0.084 0.064

>Methylation code ID 21206

0.028 0.000 0.000 0.000 0.000 0.028 0.000 0.000 0.000 0.007

0.000 0.000 0.000 0.000 0.014 0.000 0.000 0.000 0.000 0.000

0.078 0.066 0.028 0.084 0.009 0.000 0.000 0.009 0.000 0.009

0.000 0.000 0.000 0.000 0.000 0.000 0.009 0.000 0.009 0.000

0.000 0.000 0.009 0.000 0.073 0.072 0.056 0.079 0.006 0.000

0.000 0.000 0.000 0.022 0.000 0.000 0.000 0.006 0.000 0.000

0.000 0.011 0.011 0.006 0.000 0.000 0.006 0.000 0.072 0.070

0.071 0.054

>Methylation code ID 21206

0.006 0.000 0.000 0.000 0.000 0.028 0.000 0.000 0.000 0.006

0.000 0.000 0.000 0.011 0.011 0.006 0.000 0.000 0.006 0.000

0.070 0.067 0.070 0.054 0.028 0.000 0.000 0.000 0.000 0.035

0.000 0.000 0.000 0.007 0.000 0.000 0.000 0.000 0.014 0.000

0.000 0.000 0.000 0.000 0.080 0.064 0.027 0.080 0.010 0.000

0.000 0.010 0.000 0.010 0.000 0.000 0.000 0.000 0.000 0.000

0.010 0.000 0.010 0.000 0.000 0.000 0.005 0.000 0.073 0.072

0.055 0.079

>Methylation code ID 21206

0.004 0.000 0.000 0.004 0.000 0.016 0.000 0.004 0.000 0.000

0.000 0.000 0.004 0.000 0.012 0.000 0.000 0.000 0.008 0.000

0.078 0.068 0.068 0.068 0.028 0.000 0.000 0.000 0.007 0.035

0.000 0.000 0.000 0.007 0.000 0.000 0.000 0.000 0.007 0.007

0.000 0.000 0.000 0.000 0.062 0.062 0.053 0.065 0.014 0.000

0.007 0.000 0.000 0.027 0.000 0.000 0.000 0.014 0.000 0.000

0.000 0.007 0.007 0.000 0.000 0.000 0.000 0.000 0.066 0.056

0.064 0.072

>Methylation code ID 21206

0.008 0.004 0.004 0.004 0.000 0.012 0.000 0.004 0.000 0.000

0.000 0.000 0.004 0.000 0.008 0.000 0.000 0.000 0.004 0.000

0.077 0.066 0.074 0.062 0.016 0.000 0.000 0.000 0.005 0.016

0.000 0.000 0.000 0.000 0.000 0.000 0.010 0.000 0.010 0.000

0.005 0.005 0.000 0.000 0.065 0.070 0.068 0.063 0.005 0.000

0.005 0.000 0.000 0.014 0.000 0.000 0.000 0.009 0.000 0.000

0.000 0.005 0.014 0.005 0.000 0.000 0.000 0.000 0.053 0.087

0.089 0.047

>Methylation code ID 21206

0.004 0.004 0.004 0.004 0.000 0.012 0.000 0.004 0.000 0.004

0.000 0.000 0.004 0.000 0.008 0.000 0.000 0.000 0.004 0.000

0.083 0.058 0.085 0.056 0.017 0.000 0.000 0.000 0.006 0.017

0.000 0.000 0.000 0.000 0.000 0.000 0.011 0.000 0.006 0.000

0.006 0.006 0.006 0.000 0.062 0.072 0.061 0.064 0.005 0.000

0.005 0.000 0.000 0.009 0.000 0.000 0.000 0.009 0.000 0.000

0.005 0.005 0.014 0.005 0.000 0.000 0.000 0.000 0.059 0.082

0.087 0.050

>Methylation code ID 21206

0.009 0.009 0.005 0.005 0.000 0.009 0.005 0.000 0.000 0.009

0.000 0.000 0.005 0.000 0.005 0.000 0.000 0.000 0.000 0.000

0.073 0.054 0.083 0.064 0.016 0.000 0.000 0.000 0.005 0.005

0.000 0.000 0.000 0.000 0.000 0.000 0.016 0.000 0.005 0.000

0.011 0.005 0.005 0.000 0.072 0.071 0.066 0.055 0.005 0.000

0.000 0.000 0.000 0.010 0.000 0.000 0.000 0.010 0.000 0.000

0.015 0.000 0.015 0.005 0.000 0.000 0.000 0.000 0.067 0.083

0.077 0.044

>Methylation code ID 21206

0.006 0.006 0.000 0.006 0.000 0.006 0.006 0.000 0.000 0.006

0.000 0.000 0.018 0.000 0.006 0.006 0.000 0.012 0.000 0.000

0.066 0.055 0.066 0.067 0.011 0.000 0.000 0.000 0.011 0.006

0.000 0.000 0.000 0.006 0.000 0.000 0.006 0.006 0.011 0.011

0.006 0.000 0.000 0.000 0.068 0.053 0.063 0.077 0.000 0.000

0.000 0.000 0.005 0.010 0.000 0.000 0.000 0.000 0.000 0.000

0.020 0.000 0.005 0.010 0.000 0.000 0.005 0.000 0.062 0.053

0.077 0.087

>Methylation code ID 21206

0.014 0.000 0.000 0.007 0.000 0.021 0.000 0.000 0.000 0.000

0.000 0.000 0.014 0.000 0.007 0.021 0.000 0.007 0.000 0.000

0.062 0.057 0.062 0.060 0.022 0.000 0.000 0.000 0.000 0.022

0.000 0.000 0.000 0.007 0.000 0.000 0.007 0.007 0.015 0.015

0.000 0.000 0.000 0.000 0.065 0.037 0.065 0.069 0.005 0.000

0.000 0.009 0.000 0.014 0.000 0.000 0.000 0.000 0.000 0.000

0.005 0.005 0.009 0.005 0.000 0.000 0.005 0.000 0.065 0.058

0.078 0.075

>Methylation code ID 21206

0.013 0.000 0.000 0.007 0.000 0.020 0.000 0.000 0.000 0.000

0.000 0.000 0.020 0.000 0.007 0.013 0.000 0.007 0.000 0.000

0.061 0.073 0.063 0.051 0.020 0.000 0.000 0.000 0.000 0.020

0.000 0.000 0.000 0.014 0.000 0.000 0.007 0.000 0.014 0.014

0.000 0.000 0.000 0.000 0.059 0.055 0.065 0.065 0.005 0.000

0.000 0.009 0.000 0.009 0.000 0.000 0.000 0.000 0.000 0.000

0.005 0.005 0.014 0.005 0.000 0.000 0.005 0.000 0.073 0.059

0.088 0.059

>Methylation code ID 21206

0.012 0.000 0.000 0.018 0.000 0.018 0.000 0.000 0.000 0.000

0.000 0.000 0.000 0.012 0.012 0.006 0.000 0.000 0.000 0.000

0.073 0.061 0.076 0.048 0.010 0.000 0.000 0.005 0.000 0.010

0.000 0.000 0.005 0.000 0.000 0.000 0.010 0.000 0.010 0.015

0.000 0.000 0.000 0.000 0.074 0.083 0.057 0.054 0.016 0.000

0.000 0.000 0.000 0.016 0.000 0.000 0.000 0.005 0.000 0.000

0.005 0.000 0.022 0.000 0.000 0.000 0.000 0.000 0.068 0.060

0.077 0.062

>Methylation code ID 21206

0.019 0.000 0.000 0.000 0.000 0.014 0.000 0.000 0.000 0.005

0.000 0.000 0.005 0.000 0.019 0.000 0.000 0.000 0.000 0.000

0.059 0.065 0.080 0.068 0.011 0.000 0.000 0.011 0.000 0.016

0.000 0.000 0.000 0.000 0.000 0.000 0.000 0.011 0.016 0.005

0.000 0.000 0.000 0.000 0.062 0.064 0.077 0.062 0.011 0.000

0.000 0.005 0.000 0.011 0.000 0.000 0.005 0.000 0.000 0.000

0.011 0.000 0.011 0.011 0.000 0.000 0.000 0.000 0.076 0.086

0.050 0.058

>Methylation code ID 21210

0.005 0.000 0.005 0.000 0.005 0.005 0.005 0.000 0.005 0.000

0.000 0.000 0.005 0.000 0.005 0.011 0.005 0.011 0.000 0.000

0.061 0.073 0.060 0.070 0.006 0.000 0.000 0.000 0.006 0.006

0.000 0.000 0.006 0.019 0.006 0.000 0.006 0.000 0.013 0.000

0.006 0.000 0.000 0.000 0.044 0.069 0.071 0.073 0.004 0.004

0.000 0.004 0.008 0.000 0.000 0.000 0.000 0.000 0.000 0.004

0.000 0.004 0.008 0.008 0.000 0.000 0.004 0.000 0.053 0.080

0.080 0.068

>Methylation code ID 21210

0.005 0.000 0.000 0.016 0.005 0.000 0.000 0.000 0.000 0.000

0.000 0.005 0.000 0.005 0.011 0.011 0.005 0.000 0.005 0.000

0.061 0.067 0.060 0.074 0.010 0.000 0.005 0.000 0.000 0.005

0.000 0.000 0.015 0.000 0.000 0.000 0.005 0.000 0.010 0.005

0.005 0.005 0.000 0.000 0.062 0.070 0.069 0.069 0.000 0.000

0.000 0.000 0.000 0.011 0.005 0.000 0.011 0.011 0.000 0.000

0.005 0.000 0.005 0.005 0.005 0.000 0.000 0.000 0.039 0.060

0.080 0.094

>Methylation code ID 21210

0.000 0.000 0.000 0.000 0.000 0.015 0.005 0.000 0.010 0.010

0.000 0.000 0.010 0.000 0.005 0.010 0.000 0.000 0.000 0.000

0.042 0.058 0.074 0.093 0.006 0.000 0.000 0.017 0.006 0.006

0.006 0.000 0.000 0.000 0.000 0.006 0.006 0.006 0.000 0.006

0.006 0.000 0.006 0.000 0.071 0.062 0.053 0.073 0.005 0.000

0.000 0.000 0.000 0.005 0.000 0.000 0.016 0.000 0.000 0.000

0.011 0.000 0.011 0.005 0.005 0.005 0.000 0.000 0.065 0.062

0.057 0.084

>Methylation code ID 21210

0.005 0.000 0.000 0.000 0.000 0.011 0.000 0.000 0.005 0.000

0.000 0.000 0.011 0.000 0.011 0.016 0.005 0.005 0.000 0.000

0.054 0.061 0.068 0.079 0.000 0.000 0.000 0.000 0.000 0.011

0.005 0.000 0.000 0.005 0.000 0.000 0.011 0.005 0.011 0.011

0.000 0.011 0.000 0.000 0.066 0.059 0.049 0.090 0.009 0.000

0.000 0.004 0.009 0.004 0.004 0.004 0.000 0.000 0.000 0.000

0.004 0.000 0.009 0.000 0.000 0.000 0.004 0.000 0.077 0.076

0.072 0.056

>Methylation code ID 21210

0.008 0.000 0.000 0.004 0.008 0.008 0.004 0.004 0.000 0.000

0.000 0.000 0.004 0.000 0.008 0.000 0.000 0.000 0.004 0.000

0.075 0.081 0.068 0.057 0.006 0.000 0.000 0.000 0.000 0.019

0.000 0.000 0.006 0.000 0.000 0.000 0.013 0.000 0.006 0.019

0.006 0.006 0.000 0.000 0.063 0.054 0.061 0.071 0.000 0.000

0.000 0.000 0.000 0.005 0.005 0.000 0.000 0.005 0.000 0.000

0.011 0.005 0.011 0.011 0.000 0.011 0.000 0.000 0.066 0.058

0.051 0.093

>Methylation code ID 21210

0.000 0.000 0.000 0.000 0.000 0.010 0.005 0.000 0.000 0.005

0.000 0.000 0.010 0.005 0.010 0.010 0.000 0.010 0.000 0.000

0.073 0.054 0.050 0.092 0.012 0.000 0.000 0.000 0.008 0.008

0.004 0.004 0.000 0.000 0.000 0.000 0.004 0.000 0.008 0.000

0.000 0.000 0.004 0.000 0.072 0.078 0.076 0.053 0.008 0.000

0.000 0.000 0.000 0.023 0.000 0.000 0.000 0.000 0.000 0.000

0.015 0.000 0.008 0.023 0.008 0.008 0.000 0.000 0.061 0.053

0.059 0.069

>Methylation code ID 21210

0.010 0.000 0.000 0.000 0.010 0.010 0.005 0.005 0.000 0.005

0.005 0.000 0.005 0.000 0.010 0.000 0.000 0.000 0.000 0.000

0.072 0.069 0.071 0.056 0.005 0.005 0.000 0.005 0.000 0.009

0.000 0.000 0.000 0.000 0.000 0.000 0.005 0.000 0.009 0.014

0.005 0.000 0.005 0.000 0.067 0.065 0.077 0.064 0.011 0.000

0.000 0.000 0.000 0.011 0.006 0.000 0.000 0.000 0.000 0.006

0.006 0.006 0.011 0.000 0.000 0.011 0.000 0.000 0.083 0.054

0.056 0.073

>Methylation code ID 21210

0.009 0.000 0.000 0.000 0.000 0.009 0.005 0.000 0.000 0.000

0.000 0.005 0.000 0.005 0.019 0.000 0.000 0.009 0.000 0.000

0.071 0.059 0.057 0.086 0.018 0.000 0.000 0.000 0.012 0.018

0.000 0.006 0.000 0.006 0.006 0.000 0.006 0.000 0.006 0.000

0.000 0.000 0.000 0.000 0.069 0.064 0.058 0.062 0.005 0.005

0.000 0.005 0.000 0.009 0.000 0.000 0.000 0.000 0.000 0.000

0.005 0.000 0.009 0.014 0.000 0.000 0.005 0.000 0.056 0.062

0.083 0.076

>Methylation code ID 21210

0.023 0.008 0.000 0.008 0.008 0.023 0.000 0.000 0.000 0.008

0.008 0.000 0.008 0.000 0.000 0.000 0.000 0.008 0.000 0.000

0.070 0.056 0.043 0.064 0.009 0.004 0.000 0.004 0.000 0.004

0.004 0.000 0.000 0.000 0.000 0.000 0.004 0.004 0.009 0.004

0.000 0.000 0.004 0.000 0.065 0.070 0.071 0.075 0.010 0.000

0.000 0.000 0.000 0.015 0.000 0.000 0.000 0.000 0.005 0.005

0.000 0.000 0.024 0.000 0.000 0.000 0.000 0.000 0.070 0.074

0.049 0.082

>Methylation code ID 21210

0.024 0.008 0.000 0.008 0.000 0.024 0.000 0.000 0.000 0.008

0.008 0.000 0.008 0.000 0.000 0.000 0.008 0.008 0.000 0.000

0.064 0.055 0.045 0.067 0.009 0.004 0.000 0.004 0.000 0.004

0.009 0.000 0.000 0.000 0.000 0.000 0.004 0.004 0.009 0.000

0.000 0.000 0.004 0.000 0.070 0.065 0.072 0.073 0.011 0.000

0.000 0.000 0.000 0.016 0.000 0.000 0.000 0.000 0.005 0.011

0.000 0.000 0.021 0.000 0.000 0.000 0.000 0.000 0.068 0.070

0.040 0.091

>Methylation code ID 21210

0.012 0.006 0.000 0.006 0.000 0.012 0.006 0.000 0.000 0.006

0.006 0.000 0.006 0.000 0.006 0.000 0.006 0.006 0.000 0.000

0.066 0.061 0.053 0.075 0.011 0.005 0.000 0.005 0.000 0.000

0.011 0.000 0.000 0.000 0.000 0.000 0.005 0.005 0.005 0.000

0.005 0.005 0.005 0.000 0.064 0.075 0.072 0.059 0.005 0.000

0.000 0.000 0.000 0.010 0.000 0.000 0.000 0.000 0.005 0.010

0.000 0.000 0.021 0.005 0.005 0.000 0.000 0.000 0.069 0.066

0.044 0.093

>Methylation code ID 21210

0.013 0.000 0.000 0.000 0.000 0.000 0.019 0.000 0.000 0.000

0.000 0.000 0.006 0.006 0.006 0.006 0.006 0.013 0.000 0.000

0.067 0.067 0.073 0.049 0.000 0.000 0.000 0.000 0.005 0.005

0.005 0.000 0.000 0.005 0.010 0.005 0.000 0.000 0.020 0.005

0.005 0.000 0.000 0.000 0.065 0.067 0.061 0.076 0.016 0.010

0.000 0.005 0.000 0.000 0.005 0.005 0.000 0.000 0.000 0.000

0.000 0.000 0.005 0.005 0.005 0.005 0.000 0.000 0.064 0.066

0.068 0.073

>Methylation code ID 21210

0.008 0.004 0.000 0.004 0.004 0.000 0.004 0.004 0.000 0.000

0.000 0.000 0.000 0.000 0.008 0.004 0.004 0.004 0.004 0.000

0.057 0.076 0.067 0.078 0.000 0.000 0.006 0.000 0.006 0.006

0.012 0.000 0.000 0.000 0.000 0.000 0.006 0.006 0.006 0.006

0.006 0.012 0.000 0.000 0.068 0.068 0.065 0.057 0.006 0.000

0.000 0.000 0.006 0.006 0.006 0.000 0.000 0.012 0.006 0.006

0.000 0.000 0.012 0.006 0.006 0.000 0.000 0.000 0.056 0.054

0.074 0.078

>Methylation code ID 21210

0.005 0.000 0.005 0.000 0.005 0.005 0.005 0.000 0.005 0.000

0.000 0.000 0.005 0.000 0.005 0.011 0.005 0.011 0.000 0.000

0.058 0.067 0.064 0.074 0.007 0.000 0.000 0.000 0.007 0.007

0.007 0.000 0.007 0.021 0.007 0.000 0.007 0.000 0.007 0.000

0.007 0.000 0.000 0.000 0.055 0.061 0.070 0.065 0.004 0.004

0.000 0.004 0.009 0.000 0.000 0.004 0.000 0.000 0.000 0.000

0.000 0.004 0.009 0.009 0.000 0.000 0.004 0.000 0.049 0.086

0.078 0.068

>Methylation code ID 21226

0.008 0.004 0.004 0.004 0.004 0.000 0.000 0.000 0.004 0.000

0.000 0.004 0.008 0.000 0.004 0.008 0.000 0.000 0.000 0.000

0.060 0.077 0.074 0.067 0.018 0.000 0.000 0.000 0.006 0.000

0.000 0.006 0.000 0.006 0.000 0.000 0.012 0.006 0.012 0.006

0.006 0.000 0.000 0.000 0.057 0.069 0.074 0.057 0.005 0.000

0.000 0.005 0.010 0.000 0.000 0.000 0.000 0.005 0.000 0.000

0.010 0.005 0.015 0.000 0.000 0.000 0.000 0.000 0.063 0.085

0.055 0.076

>Methylation code ID 21226

0.018 0.000 0.000 0.000 0.006 0.000 0.000 0.000 0.000 0.006

0.000 0.000 0.012 0.006 0.006 0.006 0.006 0.006 0.006 0.000

0.059 0.080 0.067 0.051 0.005 0.000 0.000 0.000 0.005 0.010

0.000 0.000 0.000 0.005 0.000 0.000 0.010 0.005 0.014 0.005

0.000 0.000 0.000 0.000 0.057 0.086 0.069 0.065 0.010 0.005

0.005 0.000 0.000 0.005 0.000 0.000 0.005 0.000 0.000 0.000

0.010 0.000 0.005 0.010 0.000 0.000 0.000 0.000 0.057 0.072

0.079 0.069

>Methylation code ID 21226

0.005 0.000 0.000 0.000 0.000 0.014 0.000 0.000 0.000 0.005

0.000 0.000 0.009 0.005 0.019 0.005 0.000 0.000 0.000 0.000

0.070 0.096 0.051 0.056 0.015 0.005 0.005 0.000 0.000 0.010

0.000 0.000 0.000 0.000 0.000 0.000 0.010 0.000 0.010 0.005

0.000 0.000 0.000 0.000 0.075 0.060 0.078 0.060 0.016 0.000

0.000 0.000 0.000 0.011 0.000 0.000 0.000 0.000 0.000 0.000

0.011 0.005 0.005 0.000 0.005 0.005 0.005 0.000 0.051 0.074

0.074 0.067

>Methylation code ID 21226

0.011 0.000 0.005 0.000 0.000 0.011 0.000 0.000 0.000 0.000

0.000 0.000 0.027 0.000 0.005 0.000 0.000 0.005 0.005 0.000

0.064 0.086 0.060 0.051 0.004 0.000 0.000 0.000 0.000 0.013

0.000 0.000 0.000 0.004 0.000 0.000 0.008 0.000 0.017 0.004

0.004 0.000 0.000 0.000 0.072 0.069 0.064 0.074 0.018 0.000

0.000 0.000 0.000 0.018 0.000 0.000 0.000 0.000 0.000 0.000

0.012 0.000 0.012 0.006 0.000 0.000 0.000 0.000 0.085 0.066

0.036 0.079

>Methylation code ID 21226

0.011 0.000 0.000 0.000 0.000 0.022 0.000 0.000 0.000 0.000

0.000 0.000 0.011 0.005 0.011 0.005 0.000 0.000 0.000 0.000

0.077 0.063 0.036 0.092 0.005 0.000 0.005 0.000 0.000 0.016

0.000 0.000 0.005 0.000 0.000 0.000 0.022 0.000 0.005 0.000

0.000 0.005 0.005 0.000 0.066 0.074 0.062 0.060 0.005 0.000

0.000 0.000 0.000 0.014 0.000 0.000 0.000 0.005 0.000 0.000

0.005 0.000 0.019 0.005 0.005 0.000 0.000 0.000 0.079 0.074

0.054 0.070

>Methylation code ID 21226

0.013 0.000 0.006 0.000 0.000 0.019 0.000 0.000 0.006 0.000

0.000 0.000 0.026 0.000 0.000 0.000 0.000 0.006 0.006 0.000

0.060 0.080 0.057 0.053 0.008 0.000 0.000 0.000 0.000 0.008

0.000 0.000 0.000 0.004 0.000 0.000 0.004 0.000 0.020 0.004

0.004 0.000 0.000 0.000 0.088 0.063 0.045 0.085 0.010 0.000

0.000 0.000 0.000 0.020 0.000 0.000 0.000 0.000 0.000 0.000

0.005 0.005 0.015 0.005 0.000 0.000 0.000 0.000 0.082 0.065

0.039 0.087

>Methylation code ID 21226

0.010 0.000 0.000 0.000 0.000 0.024 0.000 0.000 0.000 0.000

0.000 0.000 0.005 0.005 0.014 0.005 0.000 0.000 0.000 0.000

0.089 0.061 0.040 0.081 0.014 0.000 0.007 0.000 0.000 0.028

0.000 0.000 0.007 0.000 0.000 0.000 0.028 0.000 0.000 0.000

0.000 0.007 0.000 0.000 0.060 0.073 0.060 0.048 0.008 0.000

0.000 0.000 0.000 0.004 0.000 0.000 0.000 0.004 0.000 0.000

0.004 0.000 0.021 0.004 0.004 0.000 0.000 0.000 0.090 0.055

0.048 0.091

>Methylation code ID 21226

0.010 0.000 0.005 0.000 0.000 0.010 0.000 0.000 0.010 0.000

0.000 0.000 0.020 0.000 0.010 0.000 0.000 0.000 0.000 0.000

0.049 0.066 0.079 0.075 0.008 0.000 0.000 0.000 0.000 0.004

0.000 0.000 0.000 0.004 0.000 0.000 0.004 0.000 0.023 0.000

0.004 0.004 0.000 0.000 0.095 0.048 0.064 0.077 0.012 0.000

0.000 0.006 0.000 0.017 0.000 0.000 0.000 0.000 0.000 0.000

0.000 0.012 0.012 0.012 0.000 0.000 0.000 0.000 0.084 0.048

0.044 0.088

>Methylation code ID 21226

0.011 0.000 0.000 0.006 0.000 0.022 0.000 0.000 0.000 0.000

0.000 0.000 0.000 0.011 0.011 0.011 0.000 0.000 0.000 0.000

0.085 0.045 0.048 0.083 0.013 0.000 0.004 0.000 0.000 0.009

0.000 0.000 0.009 0.000 0.000 0.000 0.013 0.000 0.009 0.000

0.000 0.000 0.000 0.000 0.047 0.070 0.086 0.071 0.008 0.000

0.000 0.000 0.000 0.004 0.000 0.000 0.000 0.004 0.000 0.000

0.004 0.000 0.021 0.000 0.004 0.004 0.000 0.000 0.098 0.053

0.059 0.074

>Methylation code ID 21226

0.013 0.000 0.000 0.013 0.000 0.019 0.000 0.000 0.000 0.006

0.000 0.000 0.000 0.013 0.006 0.013 0.000 0.000 0.000 0.000

0.078 0.046 0.058 0.069 0.008 0.000 0.000 0.000 0.000 0.008

0.000 0.000 0.008 0.000 0.000 0.000 0.012 0.000 0.008 0.004

0.000 0.000 0.004 0.000 0.043 0.068 0.086 0.082 0.013 0.000

0.000 0.000 0.000 0.004 0.000 0.000 0.000 0.004 0.000 0.000

0.004 0.000 0.021 0.000 0.000 0.004 0.000 0.000 0.094 0.067

0.071 0.051

>Methylation code ID 21226

0.012 0.000 0.004 0.000 0.000 0.008 0.000 0.000 0.000 0.000

0.000 0.000 0.004 0.000 0.020 0.000 0.000 0.004 0.000 0.000

0.089 0.067 0.084 0.042 0.007 0.000 0.000 0.020 0.000 0.020

0.000 0.000 0.000 0.007 0.000 0.000 0.000 0.013 0.007 0.007

0.007 0.000 0.000 0.000 0.090 0.043 0.060 0.053 0.008 0.000

0.000 0.000 0.000 0.008 0.000 0.000 0.008 0.000 0.000 0.000

0.004 0.000 0.012 0.004 0.000 0.000 0.004 0.000 0.046 0.065

0.094 0.079

>Methylation code ID 21226

0.006 0.000 0.000 0.019 0.000 0.013 0.000 0.000 0.006 0.006

0.000 0.000 0.006 0.006 0.006 0.006 0.006 0.000 0.000 0.000

0.080 0.046 0.064 0.062 0.008 0.000 0.000 0.000 0.000 0.004

0.000 0.000 0.008 0.000 0.000 0.000 0.008 0.000 0.015 0.004

0.000 0.000 0.004 0.000 0.056 0.067 0.091 0.070 0.009 0.000

0.004 0.004 0.000 0.009 0.000 0.000 0.000 0.000 0.000 0.000

0.004 0.000 0.018 0.000 0.000 0.004 0.000 0.000 0.074 0.077

0.063 0.067

>Methylation code ID 21251

0.000 0.000 0.000 0.000 0.012 0.000 0.000 0.000 0.006 0.012

0.000 0.000 0.024 0.006 0.000 0.006 0.006 0.006 0.000 0.000

0.076 0.062 0.065 0.050 0.000 0.007 0.000 0.000 0.000 0.000

0.000 0.000 0.000 0.007 0.000 0.000 0.026 0.000 0.007 0.040

0.000 0.000 0.000 0.000 0.083 0.057 0.033 0.074 0.011 0.000

0.000 0.000 0.006 0.000 0.006 0.000 0.000 0.006 0.000 0.000

0.022 0.006 0.006 0.000 0.000 0.006 0.000 0.000 0.072 0.058

0.062 0.074

>Methylation code ID 21251

0.005 0.010 0.005 0.000 0.000 0.005 0.005 0.000 0.000 0.000

0.000 0.000 0.005 0.000 0.005 0.020 0.005 0.000 0.000 0.000

0.067 0.063 0.064 0.075 0.042 0.000 0.000 0.000 0.000 0.000

0.000 0.000 0.000 0.021 0.000 0.000 0.042 0.000 0.000 0.000

0.021 0.000 0.000 0.000 0.046 0.052 0.050 0.058 0.000 0.005

0.000 0.000 0.005 0.000 0.005 0.000 0.000 0.014 0.000 0.000

0.014 0.005 0.005 0.000 0.000 0.000 0.005 0.000 0.077 0.057

0.088 0.053

>Methylation code ID 21251

0.026 0.000 0.000 0.000 0.000 0.000 0.000 0.000 0.000 0.007

0.000 0.000 0.013 0.000 0.013 0.000 0.013 0.000 0.000 0.000

0.054 0.076 0.055 0.076 0.000 0.009 0.000 0.000 0.000 0.009

0.005 0.000 0.000 0.009 0.000 0.000 0.005 0.009 0.009 0.000

0.000 0.000 0.005 0.000 0.064 0.077 0.073 0.059 0.012 0.006

0.006 0.006 0.000 0.012 0.000 0.000 0.000 0.000 0.000 0.000

0.000 0.000 0.006 0.012 0.006 0.000 0.000 0.000 0.062 0.070

0.065 0.073

>Methylation code ID 21251

0.006 0.000 0.011 0.006 0.000 0.006 0.000 0.006 0.000 0.000

0.000 0.000 0.000 0.000 0.011 0.017 0.000 0.000 0.000 0.000

0.077 0.067 0.057 0.070 0.015 0.000 0.000 0.000 0.005 0.005

0.000 0.000 0.000 0.005 0.000 0.000 0.005 0.000 0.010 0.000

0.010 0.000 0.005 0.000 0.055 0.080 0.069 0.067 0.000 0.005

0.005 0.000 0.005 0.014 0.000 0.000 0.000 0.005 0.000 0.000

0.005 0.009 0.009 0.000 0.000 0.000 0.000 0.000 0.056 0.076

0.072 0.075

>Methylation code ID 21251

0.005 0.000 0.011 0.005 0.000 0.005 0.000 0.005 0.000 0.005

0.000 0.000 0.000 0.000 0.011 0.011 0.000 0.000 0.000 0.000

0.066 0.061 0.072 0.074 0.011 0.000 0.000 0.000 0.006 0.006

0.000 0.000 0.000 0.006 0.000 0.000 0.006 0.000 0.011 0.006

0.011 0.000 0.006 0.000 0.060 0.094 0.059 0.054 0.000 0.005

0.005 0.000 0.005 0.014 0.000 0.000 0.000 0.005 0.000 0.000

0.000 0.009 0.009 0.005 0.000 0.000 0.000 0.000 0.060 0.076

0.069 0.072

>Methylation code ID 21251

0.006 0.000 0.006 0.012 0.000 0.012 0.000 0.006 0.000 0.018

0.000 0.000 0.000 0.000 0.006 0.006 0.000 0.000 0.000 0.000

0.053 0.078 0.071 0.061 0.006 0.000 0.000 0.006 0.000 0.012

0.000 0.006 0.012 0.006 0.000 0.000 0.006 0.000 0.000 0.006

0.000 0.000 0.006 0.000 0.067 0.075 0.060 0.064 0.000 0.000

0.004 0.000 0.000 0.004 0.000 0.000 0.004 0.009 0.000 0.000

0.004 0.000 0.013 0.004 0.000 0.004 0.004 0.000 0.065 0.057

0.084 0.076

>Methylation code ID 21251

0.005 0.000 0.005 0.005 0.000 0.015 0.000 0.000 0.000 0.005

0.000 0.005 0.000 0.000 0.015 0.000 0.000 0.005 0.000 0.000

0.059 0.083 0.062 0.069 0.024 0.000 0.008 0.008 0.000 0.008

0.000 0.008 0.008 0.016 0.000 0.000 0.000 0.000 0.000 0.000

0.008 0.000 0.000 0.000 0.065 0.069 0.054 0.057 0.005 0.000

0.000 0.005 0.000 0.000 0.000 0.000 0.000 0.010 0.000 0.000

0.000 0.000 0.019 0.005 0.005 0.000 0.005 0.000 0.075 0.070

0.055 0.081

>Methylation code ID 21251

0.027 0.000 0.009 0.000 0.000 0.018 0.000 0.000 0.009 0.027

0.000 0.000 0.000 0.000 0.000 0.000 0.009 0.000 0.000 0.000

0.056 0.052 0.053 0.074 0.005 0.000 0.000 0.005 0.000 0.000

0.000 0.000 0.000 0.005 0.000 0.000 0.000 0.000 0.014 0.005

0.005 0.005 0.009 0.000 0.062 0.065 0.065 0.090 0.005 0.000

0.000 0.000 0.000 0.021 0.000 0.000 0.000 0.005 0.000 0.005

0.000 0.000 0.016 0.005 0.000 0.005 0.000 0.000 0.070 0.082

0.049 0.069

>Methylation code ID 21259

0.000 0.011 0.000 0.000 0.000 0.011 0.006 0.000 0.000 0.000

0.006 0.000 0.000 0.000 0.006 0.000 0.006 0.000 0.006 0.006

0.070 0.078 0.064 0.065 0.000 0.016 0.000 0.000 0.000 0.011

0.000 0.000 0.000 0.000 0.000 0.000 0.000 0.000 0.005 0.000

0.011 0.005 0.005 0.000 0.087 0.058 0.061 0.075 0.010 0.010

0.000 0.000 0.000 0.005 0.005 0.000 0.000 0.000 0.000 0.000

0.000 0.000 0.005 0.000 0.000 0.005 0.005 0.005 0.081 0.070

0.077 0.055

>Methylation code ID 21259

0.005 0.019 0.000 0.000 0.000 0.009 0.000 0.000 0.000 0.000

0.000 0.000 0.000 0.000 0.009 0.000 0.005 0.000 0.005 0.000

0.094 0.059 0.071 0.057 0.015 0.015 0.000 0.000 0.000 0.022

0.015 0.000 0.000 0.000 0.000 0.000 0.000 0.000 0.000 0.000

0.000 0.007 0.000 0.000 0.085 0.056 0.058 0.062 0.006 0.006

0.000 0.000 0.000 0.006 0.000 0.000 0.000 0.000 0.006 0.000

0.000 0.000 0.006 0.000 0.006 0.000 0.012 0.006 0.067 0.066

0.065 0.083

>Methylation code ID 21259

0.012 0.012 0.000 0.000 0.000 0.012 0.012 0.000 0.000 0.000

0.000 0.000 0.000 0.000 0.006 0.000 0.000 0.006 0.000 0.000

0.079 0.071 0.058 0.065 0.005 0.005 0.000 0.000 0.000 0.000

0.000 0.000 0.000 0.000 0.005 0.000 0.000 0.000 0.011 0.000

0.005 0.000 0.011 0.005 0.070 0.080 0.066 0.069 0.009 0.019

0.000 0.000 0.000 0.009 0.000 0.000 0.000 0.000 0.000 0.000

0.000 0.000 0.009 0.000 0.005 0.000 0.000 0.000 0.097 0.062

0.058 0.064

>Methylation code ID 21259

0.016 0.011 0.000 0.000 0.000 0.011 0.005 0.000 0.000 0.000

0.000 0.000 0.000 0.000 0.005 0.000 0.000 0.000 0.005 0.000

0.082 0.081 0.057 0.059 0.005 0.010 0.000 0.000 0.000 0.005

0.005 0.000 0.000 0.000 0.000 0.000 0.000 0.000 0.010 0.000

0.005 0.000 0.005 0.000 0.078 0.075 0.071 0.066 0.009 0.019

0.000 0.000 0.000 0.005 0.000 0.000 0.000 0.000 0.005 0.000

0.000 0.000 0.009 0.000 0.000 0.000 0.000 0.000 0.083 0.096

0.043 0.064

>Methylation code ID 21259

0.009 0.018 0.000 0.000 0.000 0.009 0.000 0.000 0.000 0.000

0.004 0.000 0.000 0.000 0.009 0.000 0.000 0.000 0.000 0.000

0.081 0.097 0.048 0.058 0.017 0.011 0.000 0.000 0.000 0.006

0.006 0.000 0.000 0.000 0.000 0.000 0.000 0.000 0.006 0.000

0.000 0.000 0.006 0.000 0.088 0.077 0.064 0.053 0.005 0.010

0.000 0.000 0.000 0.005 0.005 0.000 0.000 0.000 0.000 0.000

0.000 0.000 0.010 0.000 0.005 0.000 0.005 0.000 0.078 0.075

0.071 0.066

>Methylation code ID 21259

0.004 0.013 0.000 0.000 0.000 0.004 0.004 0.000 0.000 0.000

0.000 0.000 0.000 0.000 0.009 0.000 0.004 0.000 0.004 0.000

0.076 0.074 0.062 0.079 0.014 0.014 0.000 0.000 0.000 0.009

0.000 0.000 0.000 0.000 0.005 0.000 0.000 0.000 0.009 0.000

0.000 0.000 0.000 0.000 0.079 0.085 0.049 0.069 0.012 0.012

0.000 0.000 0.000 0.006 0.006 0.000 0.000 0.000 0.000 0.000

0.000 0.000 0.006 0.000 0.000 0.000 0.006 0.000 0.083 0.083

0.059 0.061

>Methylation code ID 21259

0.005 0.009 0.000 0.000 0.000 0.009 0.005 0.000 0.000 0.000

0.005 0.000 0.000 0.000 0.014 0.000 0.000 0.000 0.000 0.000

0.081 0.076 0.065 0.065 0.024 0.018 0.000 0.000 0.000 0.006

0.006 0.000 0.000 0.000 0.006 0.000 0.000 0.000 0.000 0.000

0.000 0.000 0.006 0.000 0.065 0.082 0.062 0.057 0.009 0.009

0.000 0.000 0.000 0.005 0.005 0.000 0.000 0.000 0.000 0.000

0.000 0.000 0.009 0.000 0.005 0.000 0.005 0.000 0.084 0.067

0.074 0.062

>Methylation code ID 21259

0.016 0.016 0.000 0.000 0.000 0.005 0.005 0.000 0.000 0.000

0.005 0.000 0.000 0.000 0.005 0.000 0.000 0.000 0.005 0.000

0.064 0.072 0.069 0.070 0.009 0.009 0.000 0.000 0.000 0.005

0.005 0.000 0.000 0.000 0.000 0.000 0.000 0.000 0.009 0.000

0.005 0.000 0.005 0.000 0.075 0.071 0.072 0.070 0.011 0.011

0.000 0.000 0.000 0.011 0.005 0.000 0.000 0.000 0.005 0.000

0.000 0.000 0.011 0.000 0.000 0.000 0.000 0.000 0.089 0.081

0.058 0.051

>Methylation code ID 21259

0.016 0.010 0.000 0.000 0.000 0.005 0.010 0.000 0.000 0.000

0.000 0.000 0.000 0.000 0.005 0.000 0.000 0.000 0.005 0.000

0.079 0.065 0.060 0.078 0.009 0.013 0.000 0.000 0.000 0.004

0.004 0.000 0.000 0.000 0.004 0.000 0.000 0.000 0.009 0.000

0.004 0.000 0.000 0.000 0.082 0.057 0.071 0.074 0.014 0.010

0.000 0.000 0.000 0.005 0.000 0.000 0.000 0.000 0.005 0.000

0.000 0.000 0.014 0.000 0.000 0.000 0.000 0.000 0.093 0.082

0.047 0.063

>Methylation code ID 21259

0.013 0.008 0.000 0.000 0.000 0.004 0.000 0.000 0.000 0.000

0.004 0.000 0.000 0.000 0.013 0.000 0.000 0.000 0.004 0.000

0.088 0.084 0.053 0.062 0.015 0.010 0.000 0.000 0.000 0.005

0.010 0.000 0.000 0.000 0.000 0.000 0.000 0.000 0.005 0.000

0.000 0.000 0.005 0.000 0.084 0.063 0.075 0.061 0.009 0.013

0.000 0.000 0.000 0.004 0.004 0.000 0.000 0.000 0.004 0.000

0.000 0.000 0.009 0.000 0.004 0.000 0.000 0.000 0.082 0.057

0.071 0.074

>Methylation code ID 21259

0.008 0.013 0.000 0.000 0.000 0.008 0.004 0.000 0.000 0.000

0.004 0.000 0.000 0.000 0.008 0.000 0.004 0.000 0.000 0.000

0.080 0.060 0.066 0.076 0.017 0.004 0.000 0.000 0.000 0.004

0.000 0.000 0.000 0.000 0.004 0.000 0.000 0.000 0.013 0.000

0.000 0.000 0.004 0.000 0.082 0.078 0.061 0.064 0.011 0.011

0.000 0.000 0.000 0.005 0.011 0.000 0.000 0.000 0.000 0.000

0.000 0.000 0.005 0.000 0.000 0.000 0.005 0.000 0.084 0.070

0.073 0.057

>Methylation code ID 21259

0.010 0.005 0.000 0.000 0.000 0.005 0.010 0.000 0.000 0.000

0.000 0.000 0.000 0.000 0.010 0.005 0.000 0.000 0.005 0.000

0.094 0.059 0.093 0.036 0.005 0.016 0.000 0.000 0.000 0.011

0.000 0.000 0.000 0.000 0.005 0.000 0.000 0.005 0.005 0.000

0.005 0.000 0.000 0.000 0.076 0.064 0.070 0.070 0.016 0.008

0.000 0.000 0.000 0.004 0.000 0.000 0.004 0.000 0.000 0.000

0.000 0.000 0.008 0.000 0.000 0.000 0.004 0.000 0.080 0.075

0.049 0.085

>Methylation code ID 21259

0.012 0.018 0.000 0.000 0.000 0.012 0.000 0.000 0.000 0.000

0.006 0.000 0.000 0.006 0.006 0.000 0.000 0.000 0.000 0.000

0.071 0.052 0.074 0.075 0.012 0.008 0.000 0.000 0.000 0.000

0.000 0.000 0.004 0.000 0.000 0.000 0.000 0.000 0.008 0.000

0.000 0.000 0.008 0.000 0.084 0.064 0.075 0.068 0.006 0.011

0.000 0.000 0.000 0.011 0.006 0.000 0.000 0.000 0.000 0.000

0.000 0.000 0.011 0.006 0.000 0.000 0.000 0.000 0.083 0.051

0.094 0.053

>Methylation code ID 21259

0.007 0.022 0.000 0.000 0.000 0.015 0.007 0.000 0.000 0.000

0.000 0.000 0.000 0.000 0.000 0.000 0.000 0.000 0.007 0.000

0.094 0.045 0.090 0.047 0.012 0.025 0.000 0.000 0.000 0.006

0.006 0.000 0.000 0.000 0.000 0.000 0.000 0.000 0.000 0.000

0.006 0.000 0.006 0.000 0.086 0.058 0.058 0.069 0.005 0.015

0.000 0.000 0.000 0.010 0.000 0.000 0.000 0.000 0.005 0.000

0.000 0.000 0.010 0.000 0.000 0.000 0.000 0.000 0.091 0.093

0.034 0.071

>Methylation code ID 21259

0.006 0.019 0.000 0.000 0.000 0.000 0.000 0.000 0.000 0.006

0.000 0.000 0.000 0.006 0.006 0.000 0.006 0.000 0.006 0.006

0.086 0.044 0.083 0.057 0.018 0.024 0.000 0.000 0.000 0.006

0.012 0.000 0.000 0.000 0.000 0.000 0.000 0.000 0.000 0.006

0.006 0.000 0.000 0.000 0.085 0.054 0.069 0.054 0.012 0.006

0.000 0.000 0.000 0.006 0.006 0.000 0.000 0.000 0.006 0.000

0.000 0.000 0.012 0.000 0.006 0.006 0.000 0.000 0.076 0.079

0.077 0.043

>Methylation code ID 21259

0.005 0.005 0.005 0.000 0.000 0.005 0.000 0.000 0.000 0.005

0.000 0.000 0.000 0.005 0.015 0.000 0.010 0.000 0.000 0.005

0.088 0.040 0.084 0.063 0.013 0.013 0.000 0.013 0.000 0.006

0.006 0.000 0.000 0.000 0.000 0.000 0.006 0.006 0.000 0.006

0.013 0.000 0.000 0.000 0.063 0.069 0.086 0.034 0.010 0.000

0.000 0.000 0.000 0.005 0.005 0.000 0.005 0.000 0.000 0.005

0.005 0.000 0.015 0.000 0.005 0.005 0.000 0.000 0.062 0.075

0.075 0.064

>Methylation code ID 21259

0.006 0.006 0.000 0.012 0.006 0.006 0.006 0.000 0.000 0.000

0.000 0.000 0.019 0.006 0.000 0.006 0.006 0.000 0.000 0.000

0.061 0.080 0.071 0.041 0.009 0.000 0.000 0.000 0.000 0.005

0.005 0.000 0.005 0.000 0.000 0.005 0.009 0.000 0.009 0.009

0.005 0.000 0.000 0.000 0.061 0.071 0.079 0.061 0.010 0.000

0.005 0.000 0.000 0.005 0.000 0.000 0.000 0.010 0.000 0.000

0.000 0.005 0.015 0.000 0.010 0.000 0.000 0.000 0.082 0.041

0.086 0.064

>Methylation code ID 21285

0.007 0.004 0.000 0.000 0.000 0.004 0.000 0.000 0.000 0.000

0.000 0.000 0.004 0.000 0.018 0.000 0.007 0.004 0.000 0.000

0.077 0.056 0.068 0.085 0.022 0.000 0.000 0.000 0.000 0.004

0.000 0.000 0.000 0.000 0.000 0.000 0.009 0.000 0.013 0.004

0.000 0.004 0.000 0.000 0.062 0.059 0.057 0.098 0.009 0.000

0.005 0.000 0.000 0.000 0.005 0.000 0.000 0.000 0.000 0.000

0.018 0.000 0.014 0.000 0.000 0.000 0.000 0.000 0.081 0.055

0.061 0.086

>Methylation code ID 21285

0.008 0.000 0.004 0.000 0.004 0.000 0.004 0.000 0.000 0.000

0.000 0.000 0.016 0.000 0.012 0.000 0.000 0.000 0.000 0.000

0.077 0.057 0.062 0.088 0.007 0.004 0.000 0.000 0.000 0.000

0.000 0.000 0.000 0.000 0.000 0.000 0.004 0.000 0.018 0.004

0.007 0.004 0.000 0.000 0.075 0.063 0.066 0.081 0.018 0.000

0.000 0.000 0.000 0.004 0.000 0.000 0.000 0.000 0.000 0.000

0.009 0.000 0.013 0.004 0.000 0.004 0.000 0.000 0.063 0.058

0.056 0.103

>Methylation code ID 21285

0.008 0.000 0.004 0.000 0.004 0.000 0.004 0.000 0.000 0.000

0.000 0.000 0.013 0.000 0.013 0.004 0.000 0.000 0.000 0.000

0.073 0.061 0.068 0.081 0.011 0.004 0.000 0.000 0.000 0.000

0.000 0.000 0.000 0.000 0.000 0.000 0.004 0.000 0.015 0.004

0.008 0.004 0.000 0.000 0.068 0.065 0.080 0.072 0.014 0.000

0.000 0.000 0.000 0.005 0.000 0.000 0.000 0.000 0.000 0.000

0.009 0.005 0.014 0.005 0.000 0.005 0.000 0.000 0.065 0.060

0.062 0.092

>Methylation code ID 21285

0.012 0.000 0.000 0.000 0.000 0.004 0.000 0.000 0.000 0.000

0.000 0.000 0.008 0.004 0.016 0.000 0.000 0.008 0.000 0.000

0.070 0.067 0.061 0.083 0.010 0.000 0.005 0.005 0.010 0.000

0.005 0.000 0.000 0.005 0.000 0.000 0.005 0.000 0.010 0.005

0.000 0.000 0.000 0.000 0.073 0.061 0.071 0.068 0.013 0.004

0.000 0.000 0.000 0.000 0.000 0.000 0.000 0.000 0.000 0.000

0.000 0.000 0.013 0.013 0.009 0.000 0.000 0.000 0.057 0.065

0.078 0.079

>Methylation code ID 21285

0.009 0.000 0.000 0.000 0.000 0.004 0.004 0.000 0.000 0.000

0.000 0.000 0.009 0.004 0.017 0.000 0.000 0.009 0.000 0.000

0.068 0.076 0.058 0.075 0.011 0.000 0.005 0.005 0.011 0.000

0.005 0.005 0.000 0.005 0.000 0.000 0.005 0.000 0.005 0.005

0.000 0.000 0.000 0.000 0.064 0.068 0.070 0.067 0.010 0.005

0.000 0.000 0.000 0.000 0.000 0.000 0.000 0.000 0.000 0.000

0.000 0.000 0.014 0.019 0.010 0.000 0.000 0.000 0.048 0.069

0.083 0.076

>Methylation code ID 21285

0.006 0.000 0.006 0.006 0.012 0.000 0.006 0.006 0.000 0.006

0.000 0.006 0.006 0.000 0.000 0.006 0.006 0.000 0.000 0.000

0.056 0.069 0.064 0.071 0.006 0.006 0.000 0.000 0.000 0.000

0.000 0.000 0.000 0.000 0.000 0.000 0.006 0.000 0.012 0.029

0.018 0.000 0.000 0.000 0.045 0.070 0.077 0.065 0.005 0.000

0.000 0.000 0.000 0.005 0.011 0.000 0.000 0.000 0.000 0.000

0.011 0.011 0.011 0.000 0.000 0.011 0.000 0.000 0.061 0.070

0.069 0.070

>Methylation code ID 21285

0.000 0.000 0.000 0.006 0.006 0.006 0.000 0.012 0.006 0.012

0.000 0.006 0.000 0.006 0.006 0.000 0.006 0.000 0.000 0.006

0.055 0.052 0.073 0.077 0.000 0.000 0.000 0.012 0.000 0.012

0.000 0.000 0.000 0.000 0.000 0.000 0.012 0.000 0.000 0.085

0.024 0.000 0.000 0.000 0.039 0.038 0.050 0.061 0.011 0.000

0.000 0.006 0.000 0.000 0.011 0.000 0.000 0.006 0.000 0.006

0.011 0.006 0.006 0.000 0.000 0.006 0.000 0.000 0.059 0.067

0.062 0.077

>Methylation code ID 21285

0.009 0.009 0.000 0.009 0.000 0.009 0.000 0.000 0.000 0.000

0.000 0.000 0.027 0.000 0.000 0.027 0.018 0.000 0.000 0.000

0.065 0.040 0.063 0.056 0.018 0.000 0.000 0.005 0.000 0.000

0.000 0.000 0.000 0.005 0.000 0.005 0.009 0.005 0.014 0.000

0.000 0.000 0.000 0.000 0.073 0.079 0.052 0.070 0.005 0.000

0.005 0.000 0.000 0.005 0.000 0.005 0.005 0.010 0.000 0.000

0.005 0.005 0.010 0.000 0.000 0.000 0.000 0.005 0.077 0.054

0.087 0.057

>Methylation code ID 21285

0.004 0.000 0.004 0.000 0.000 0.004 0.000 0.004 0.004 0.009

0.000 0.004 0.009 0.004 0.009 0.000 0.000 0.000 0.000 0.000

0.074 0.051 0.093 0.057 0.009 0.009 0.000 0.009 0.000 0.009

0.000 0.000 0.000 0.000 0.000 0.000 0.027 0.000 0.000 0.027

0.018 0.000 0.000 0.000 0.067 0.034 0.074 0.048 0.021 0.000

0.000 0.005 0.000 0.000 0.000 0.000 0.000 0.000 0.000 0.005

0.016 0.005 0.011 0.000 0.000 0.000 0.000 0.000 0.075 0.088

0.049 0.057

>Methylation code ID 21285

0.009 0.017 0.000 0.000 0.000 0.009 0.000 0.000 0.000 0.000

0.000 0.000 0.026 0.000 0.000 0.017 0.026 0.000 0.000 0.000

0.072 0.048 0.064 0.047 0.025 0.000 0.000 0.005 0.000 0.000

0.000 0.000 0.000 0.000 0.000 0.000 0.015 0.010 0.010 0.000

0.000 0.000 0.000 0.000 0.064 0.083 0.060 0.062 0.005 0.000

0.005 0.000 0.000 0.000 0.005 0.000 0.005 0.010 0.000 0.005

0.010 0.005 0.010 0.000 0.000 0.000 0.000 0.000 0.080 0.053

0.080 0.062

>Methylation code ID 21285

0.005 0.000 0.005 0.000 0.000 0.000 0.005 0.000 0.005 0.009

0.000 0.005 0.009 0.005 0.009 0.005 0.000 0.000 0.000 0.000

0.080 0.052 0.079 0.061 0.016 0.016 0.000 0.000 0.000 0.008

0.000 0.000 0.000 0.000 0.000 0.000 0.024 0.000 0.000 0.016

0.024 0.000 0.000 0.000 0.069 0.054 0.062 0.046 0.027 0.000

0.000 0.000 0.000 0.000 0.000 0.000 0.000 0.000 0.000 0.000

0.016 0.011 0.011 0.000 0.000 0.000 0.000 0.000 0.065 0.086

0.061 0.057

>Methylation code ID 21285

0.025 0.000 0.000 0.000 0.000 0.006 0.000 0.000 0.000 0.000

0.000 0.000 0.019 0.025 0.006 0.000 0.000 0.000 0.000 0.000

0.068 0.075 0.052 0.057 0.011 0.000 0.000 0.000 0.000 0.000

0.006 0.000 0.006 0.017 0.000 0.006 0.011 0.000 0.006 0.011

0.000 0.000 0.000 0.000 0.058 0.075 0.060 0.068 0.014 0.009

0.000 0.000 0.000 0.000 0.000 0.000 0.000 0.000 0.000 0.000

0.009 0.000 0.009 0.005 0.009 0.000 0.000 0.000 0.071 0.058

0.070 0.080

>Methylation code ID 21285

0.025 0.000 0.000 0.000 0.000 0.012 0.006 0.000 0.000 0.000

0.000 0.000 0.012 0.018 0.006 0.000 0.000 0.000 0.000 0.000

0.071 0.080 0.042 0.061 0.011 0.000 0.000 0.000 0.000 0.000

0.005 0.005 0.005 0.016 0.000 0.000 0.005 0.000 0.005 0.011

0.000 0.005 0.000 0.000 0.043 0.069 0.077 0.074 0.012 0.008

0.000 0.000 0.000 0.000 0.000 0.000 0.000 0.000 0.000 0.000

0.004 0.000 0.008 0.008 0.004 0.000 0.004 0.000 0.071 0.049

0.074 0.091

>Methylation code ID 21285

0.013 0.004 0.000 0.000 0.000 0.000 0.000 0.000 0.000 0.000

0.000 0.000 0.004 0.000 0.009 0.013 0.009 0.000 0.004 0.000

0.071 0.053 0.077 0.076 0.015 0.000 0.000 0.000 0.000 0.010

0.005 0.000 0.000 0.000 0.000 0.000 0.005 0.015 0.015 0.000

0.000 0.000 0.000 0.000 0.058 0.077 0.057 0.075 0.011 0.000

0.005 0.000 0.000 0.000 0.000 0.005 0.005 0.011 0.000 0.000

0.005 0.000 0.005 0.011 0.000 0.005 0.000 0.000 0.043 0.073

0.080 0.071

>Methylation code ID 21285

0.006 0.000 0.006 0.000 0.000 0.000 0.000 0.006 0.011 0.011

0.000 0.000 0.011 0.000 0.006 0.000 0.000 0.006 0.000 0.000

0.052 0.085 0.073 0.062 0.005 0.010 0.000 0.000 0.000 0.000

0.000 0.000 0.000 0.000 0.000 0.000 0.010 0.000 0.005 0.020

0.005 0.000 0.010 0.000 0.063 0.070 0.076 0.061 0.020 0.000

0.000 0.000 0.000 0.010 0.005 0.000 0.000 0.000 0.000 0.000

0.005 0.010 0.010 0.000 0.000 0.000 0.000 0.000 0.044 0.076

0.081 0.072

>Methylation code ID 21285

0.000 0.000 0.005 0.000 0.005 0.000 0.000 0.005 0.010 0.010

0.000 0.000 0.010 0.000 0.005 0.000 0.000 0.005 0.000 0.000

0.058 0.074 0.070 0.075 0.006 0.011 0.000 0.000 0.000 0.000

0.000 0.000 0.000 0.000 0.000 0.000 0.011 0.000 0.000 0.028

0.006 0.000 0.011 0.000 0.051 0.069 0.080 0.061 0.015 0.000

0.000 0.000 0.000 0.010 0.005 0.000 0.000 0.000 0.000 0.000

0.005 0.010 0.010 0.000 0.000 0.005 0.000 0.000 0.046 0.074

0.081 0.073

>Methylation code ID 21285

0.000 0.000 0.007 0.000 0.007 0.007 0.000 0.000 0.007 0.033

0.000 0.000 0.013 0.000 0.000 0.007 0.000 0.000 0.000 0.000

0.047 0.075 0.076 0.057 0.004 0.018 0.000 0.000 0.000 0.000

0.000 0.000 0.000 0.000 0.000 0.000 0.009 0.000 0.004 0.013

0.004 0.000 0.004 0.000 0.051 0.068 0.081 0.075 0.035 0.000

0.000 0.000 0.000 0.007 0.000 0.000 0.000 0.007 0.000 0.000

0.014 0.007 0.007 0.000 0.000 0.007 0.000 0.000 0.063 0.065

0.063 0.058

>Methylation code ID 21285

0.026 0.000 0.000 0.000 0.000 0.007 0.000 0.000 0.000 0.013

0.000 0.000 0.013 0.013 0.007 0.000 0.000 0.007 0.000 0.000

0.065 0.062 0.065 0.056 0.000 0.006 0.006 0.000 0.006 0.006

0.000 0.000 0.006 0.028 0.000 0.006 0.006 0.000 0.000 0.006

0.000 0.000 0.000 0.000 0.060 0.068 0.070 0.061 0.005 0.014

0.000 0.000 0.000 0.000 0.000 0.000 0.000 0.000 0.000 0.000

0.005 0.000 0.005 0.014 0.009 0.000 0.005 0.000 0.053 0.074

0.082 0.068

>Methylation code ID 21285

0.000 0.010 0.005 0.010 0.000 0.000 0.000 0.000 0.005 0.026

0.000 0.005 0.000 0.000 0.000 0.005 0.000 0.000 0.000 0.000

0.061 0.059 0.069 0.076 0.011 0.017 0.000 0.000 0.000 0.000

0.000 0.000 0.000 0.000 0.000 0.000 0.006 0.000 0.011 0.017

0.011 0.000 0.000 0.000 0.055 0.081 0.076 0.049 0.021 0.000

0.000 0.000 0.000 0.014 0.000 0.000 0.000 0.014 0.000 0.000

0.007 0.014 0.007 0.000 0.000 0.000 0.000 0.000 0.063 0.076

0.040 0.077

>Methylation code ID 21285

0.005 0.005 0.005 0.010 0.005 0.005 0.000 0.000 0.010 0.010

0.000 0.000 0.005 0.000 0.000 0.005 0.000 0.000 0.000 0.000

0.061 0.070 0.068 0.068 0.011 0.000 0.000 0.000 0.000 0.000

0.000 0.000 0.000 0.000 0.000 0.000 0.016 0.000 0.011 0.027

0.005 0.000 0.000 0.000 0.048 0.072 0.068 0.075 0.010 0.000

0.000 0.000 0.000 0.010 0.000 0.000 0.000 0.010 0.000 0.000

0.005 0.010 0.015 0.000 0.000 0.000 0.000 0.000 0.065 0.081

0.058 0.068

>Methylation code ID 21285

0.018 0.000 0.006 0.006 0.006 0.006 0.000 0.000 0.006 0.006

0.000 0.000 0.012 0.000 0.000 0.000 0.000 0.000 0.006 0.000

0.064 0.062 0.062 0.074 0.006 0.000 0.000 0.006 0.000 0.006

0.000 0.000 0.000 0.000 0.000 0.000 0.025 0.000 0.012 0.019

0.006 0.000 0.000 0.000 0.065 0.071 0.047 0.071 0.005 0.000

0.000 0.000 0.000 0.005 0.000 0.000 0.000 0.010 0.000 0.000

0.010 0.005 0.015 0.005 0.000 0.000 0.000 0.000 0.080 0.070

0.050 0.078

>Methylation code ID 21285

0.018 0.000 0.000 0.000 0.006 0.000 0.000 0.000 0.000 0.006

0.000 0.000 0.030 0.000 0.006 0.000 0.000 0.000 0.006 0.000

0.064 0.064 0.071 0.062 0.005 0.000 0.000 0.005 0.000 0.005

0.000 0.000 0.000 0.005 0.000 0.000 0.020 0.000 0.015 0.010

0.000 0.000 0.000 0.000 0.073 0.069 0.039 0.088 0.017 0.000

0.000 0.000 0.000 0.000 0.000 0.000 0.000 0.012 0.000 0.000

0.017 0.000 0.012 0.006 0.000 0.000 0.000 0.000 0.072 0.071

0.040 0.086

>Methylation code ID 21285

0.018 0.000 0.000 0.000 0.000 0.000 0.000 0.000 0.000 0.018

0.000 0.000 0.018 0.000 0.012 0.006 0.000 0.000 0.000 0.000

0.076 0.061 0.044 0.080 0.017 0.006 0.000 0.000 0.006 0.000

0.000 0.000 0.000 0.006 0.000 0.000 0.023 0.000 0.006 0.000

0.000 0.000 0.006 0.000 0.065 0.054 0.068 0.077 0.010 0.000

0.000 0.005 0.000 0.005 0.000 0.000 0.000 0.005 0.000 0.000

0.014 0.000 0.014 0.005 0.000 0.000 0.000 0.000 0.069 0.074

0.054 0.080

>Methylation code ID 21285

0.008 0.004 0.000 0.004 0.000 0.004 0.000 0.000 0.000 0.004

0.000 0.000 0.012 0.000 0.012 0.004 0.000 0.000 0.000 0.000

0.064 0.069 0.062 0.085 0.027 0.000 0.000 0.000 0.000 0.000

0.000 0.000 0.000 0.013 0.000 0.000 0.020 0.000 0.013 0.007

0.000 0.000 0.000 0.000 0.071 0.070 0.046 0.066 0.018 0.006

0.000 0.000 0.000 0.000 0.000 0.000 0.000 0.006 0.000 0.000

0.024 0.000 0.006 0.000 0.000 0.000 0.006 0.000 0.064 0.057

0.067 0.078

>Methylation code ID 21285

0.031 0.000 0.000 0.000 0.000 0.000 0.000 0.000 0.000 0.016

0.000 0.000 0.023 0.000 0.008 0.008 0.000 0.008 0.000 0.000

0.063 0.072 0.054 0.051 0.012 0.006 0.000 0.000 0.000 0.000

0.000 0.000 0.000 0.006 0.000 0.000 0.023 0.006 0.006 0.000

0.000 0.000 0.006 0.000 0.055 0.065 0.071 0.078 0.008 0.004

0.000 0.004 0.000 0.004 0.000 0.000 0.000 0.004 0.000 0.000

0.008 0.000 0.012 0.004 0.000 0.000 0.000 0.000 0.064 0.065

0.063 0.093

>Methylation code ID 21285

0.031 0.000 0.000 0.000 0.000 0.000 0.000 0.000 0.000 0.008

0.000 0.008 0.023 0.000 0.008 0.008 0.000 0.008 0.000 0.000

0.067 0.058 0.058 0.058 0.013 0.007 0.000 0.000 0.000 0.000

0.000 0.000 0.000 0.007 0.000 0.000 0.026 0.007 0.007 0.000

0.007 0.000 0.000 0.000 0.053 0.064 0.076 0.069 0.008 0.004

0.000 0.004 0.000 0.000 0.000 0.000 0.004 0.004 0.000 0.000

0.008 0.000 0.012 0.004 0.000 0.000 0.000 0.000 0.061 0.073

0.066 0.086

>Methylation code ID 21285

0.026 0.000 0.000 0.000 0.007 0.000 0.000 0.000 0.000 0.007

0.000 0.007 0.013 0.000 0.007 0.000 0.007 0.013 0.000 0.000

0.056 0.066 0.060 0.065 0.000 0.006 0.000 0.000 0.006 0.006

0.000 0.000 0.000 0.011 0.000 0.000 0.017 0.011 0.006 0.000

0.006 0.000 0.000 0.000 0.055 0.066 0.071 0.073 0.004 0.008

0.000 0.000 0.000 0.000 0.000 0.000 0.004 0.004 0.000 0.004

0.004 0.000 0.008 0.008 0.000 0.004 0.000 0.000 0.059 0.083

0.080 0.062

>Methylation code ID 21285

0.004 0.008 0.000 0.000 0.000 0.004 0.000 0.000 0.004 0.004

0.000 0.004 0.004 0.000 0.008 0.008 0.000 0.004 0.000 0.000

0.059 0.082 0.075 0.065 0.019 0.000 0.000 0.000 0.006 0.006

0.000 0.000 0.000 0.006 0.000 0.006 0.012 0.000 0.006 0.000

0.006 0.012 0.000 0.000 0.069 0.063 0.058 0.063 0.000 0.006

0.000 0.000 0.006 0.006 0.000 0.000 0.000 0.012 0.000 0.000

0.012 0.012 0.006 0.000 0.006 0.000 0.000 0.000 0.058 0.068

0.071 0.072

>Methylation code ID 21285

0.015 0.000 0.000 0.000 0.007 0.007 0.000 0.000 0.000 0.007

0.000 0.000 0.022 0.007 0.007 0.000 0.007 0.000 0.000 0.000

0.060 0.069 0.062 0.060 0.000 0.009 0.000 0.000 0.000 0.004

0.000 0.000 0.004 0.009 0.000 0.004 0.009 0.000 0.009 0.004

0.000 0.004 0.000 0.000 0.075 0.082 0.080 0.039 0.010 0.000

0.000 0.000 0.005 0.010 0.000 0.000 0.000 0.000 0.000 0.005

0.005 0.000 0.010 0.005 0.005 0.005 0.000 0.000 0.071 0.053

0.074 0.075

>Methylation code ID 21285

0.018 0.000 0.000 0.000 0.006 0.006 0.000 0.000 0.000 0.006

0.000 0.000 0.018 0.006 0.012 0.000 0.000 0.000 0.000 0.000

0.057 0.073 0.062 0.068 0.000 0.009 0.000 0.000 0.000 0.005

0.000 0.000 0.000 0.009 0.000 0.005 0.009 0.000 0.009 0.005

0.000 0.009 0.000 0.000 0.088 0.071 0.077 0.038 0.010 0.005

0.000 0.000 0.005 0.010 0.000 0.000 0.000 0.000 0.000 0.000

0.005 0.000 0.010 0.005 0.005 0.005 0.000 0.000 0.071 0.051

0.071 0.083

>Methylation code ID 21285

0.009 0.005 0.000 0.000 0.005 0.009 0.000 0.000 0.000 0.000

0.000 0.000 0.005 0.000 0.009 0.009 0.005 0.005 0.000 0.000

0.070 0.056 0.067 0.080 0.021 0.000 0.000 0.000 0.007 0.007

0.000 0.000 0.000 0.007 0.000 0.000 0.028 0.007 0.007 0.000

0.000 0.000 0.000 0.000 0.066 0.067 0.062 0.054 0.000 0.010

0.000 0.000 0.000 0.005 0.000 0.000 0.000 0.010 0.000 0.005

0.010 0.000 0.010 0.005 0.000 0.005 0.000 0.000 0.087 0.074

0.077 0.038

>Methylation code ID 21285

0.009 0.004 0.000 0.000 0.000 0.013 0.000 0.000 0.000 0.000

0.000 0.000 0.004 0.000 0.017 0.009 0.000 0.000 0.000 0.000

0.075 0.063 0.058 0.081 0.019 0.000 0.000 0.006 0.000 0.019

0.000 0.000 0.000 0.000 0.000 0.000 0.026 0.000 0.006 0.000

0.000 0.000 0.000 0.000 0.053 0.071 0.069 0.063 0.000 0.005

0.000 0.005 0.000 0.010 0.000 0.000 0.000 0.010 0.000 0.000

0.014 0.000 0.010 0.000 0.000 0.005 0.000 0.000 0.078 0.076

0.081 0.040

>Methylation code ID 21285

0.000 0.004 0.000 0.004 0.000 0.009 0.000 0.000 0.004 0.009

0.000 0.000 0.013 0.000 0.009 0.000 0.000 0.004 0.000 0.000

0.077 0.073 0.081 0.046 0.009 0.005 0.000 0.000 0.000 0.014

0.000 0.000 0.005 0.000 0.000 0.000 0.005 0.000 0.014 0.009

0.000 0.000 0.000 0.000 0.075 0.068 0.049 0.082 0.020 0.000

0.000 0.007 0.000 0.013 0.000 0.000 0.000 0.000 0.000 0.000

0.027 0.000 0.007 0.000 0.000 0.000 0.000 0.000 0.055 0.075

0.068 0.061

>Methylation code ID 21285

0.016 0.000 0.000 0.005 0.000 0.011 0.000 0.000 0.000 0.000

0.000 0.000 0.021 0.000 0.011 0.000 0.000 0.000 0.000 0.000

0.064 0.088 0.072 0.046 0.000 0.004 0.000 0.004 0.000 0.009

0.000 0.000 0.004 0.009 0.000 0.000 0.013 0.000 0.009 0.000

0.000 0.004 0.000 0.000 0.070 0.071 0.068 0.066 0.010 0.005

0.000 0.000 0.000 0.010 0.000 0.000 0.005 0.000 0.000 0.000

0.005 0.000 0.014 0.010 0.000 0.000 0.000 0.000 0.073 0.058

0.064 0.081

>Methylation code ID 21285

0.020 0.000 0.000 0.005 0.000 0.010 0.000 0.000 0.000 0.000

0.000 0.000 0.015 0.000 0.010 0.005 0.000 0.000 0.000 0.000

0.068 0.071 0.062 0.066 0.004 0.000 0.000 0.004 0.000 0.009

0.000 0.000 0.004 0.000 0.000 0.000 0.021 0.000 0.013 0.000

0.000 0.000 0.000 0.000 0.080 0.060 0.044 0.094 0.004 0.000

0.000 0.000 0.000 0.008 0.000 0.000 0.004 0.000 0.000 0.000

0.008 0.000 0.021 0.000 0.000 0.004 0.000 0.000 0.091 0.053

0.058 0.083

>Methylation code ID 21285

0.008 0.000 0.000 0.000 0.000 0.008 0.000 0.000 0.004 0.000

0.000 0.000 0.008 0.000 0.019 0.000 0.000 0.004 0.000 0.000

0.094 0.048 0.064 0.077 0.019 0.000 0.000 0.005 0.000 0.009

0.000 0.000 0.000 0.000 0.000 0.000 0.009 0.000 0.014 0.005

0.000 0.000 0.000 0.000 0.075 0.061 0.067 0.070 0.004 0.000

0.000 0.004 0.000 0.009 0.000 0.000 0.004 0.000 0.000 0.000

0.018 0.000 0.013 0.000 0.000 0.000 0.000 0.000 0.085 0.060

0.043 0.092

>Methylation code ID 21285

0.022 0.000 0.000 0.004 0.000 0.004 0.000 0.000 0.000 0.000

0.000 0.000 0.004 0.000 0.018 0.004 0.000 0.000 0.000 0.000

0.078 0.073 0.044 0.081 0.004 0.000 0.004 0.000 0.000 0.009

0.004 0.000 0.004 0.000 0.000 0.000 0.013 0.000 0.013 0.000

0.000 0.000 0.000 0.000 0.086 0.061 0.044 0.091 0.008 0.004

0.000 0.000 0.000 0.008 0.000 0.000 0.004 0.000 0.000 0.000

0.004 0.000 0.012 0.000 0.004 0.004 0.000 0.000 0.089 0.042

0.074 0.079

>Methylation code ID 21285

0.008 0.004 0.000 0.000 0.000 0.008 0.000 0.000 0.004 0.000

0.000 0.000 0.004 0.000 0.012 0.000 0.008 0.004 0.000 0.000

0.090 0.040 0.071 0.081 0.021 0.000 0.000 0.004 0.000 0.000

0.000 0.000 0.000 0.000 0.000 0.000 0.008 0.000 0.017 0.004

0.000 0.000 0.000 0.000 0.073 0.070 0.054 0.081 0.005 0.000

0.005 0.000 0.000 0.005 0.005 0.000 0.005 0.000 0.000 0.000

0.014 0.000 0.014 0.000 0.000 0.000 0.000 0.000 0.086 0.067

0.045 0.085

>Methylation code ID 21285

0.004 0.000 0.004 0.000 0.000 0.004 0.004 0.000 0.004 0.000

0.000 0.000 0.017 0.000 0.013 0.000 0.000 0.000 0.000 0.000

0.084 0.065 0.054 0.078 0.007 0.004 0.000 0.000 0.000 0.004

0.000 0.000 0.004 0.000 0.000 0.000 0.004 0.000 0.015 0.000

0.007 0.004 0.000 0.000 0.084 0.052 0.062 0.088 0.022 0.000

0.000 0.000 0.000 0.000 0.000 0.000 0.000 0.000 0.000 0.000

0.009 0.000 0.018 0.004 0.000 0.000 0.000 0.000 0.070 0.070

0.053 0.086

>Methylation code ID 21285

0.020 0.000 0.000 0.000 0.000 0.004 0.000 0.000 0.000 0.000

0.000 0.000 0.008 0.000 0.016 0.004 0.000 0.000 0.000 0.000

0.067 0.063 0.060 0.090 0.009 0.000 0.004 0.000 0.000 0.004

0.004 0.000 0.000 0.000 0.000 0.000 0.017 0.000 0.013 0.000

0.000 0.000 0.000 0.000 0.079 0.063 0.059 0.082 0.008 0.004

0.000 0.000 0.000 0.004 0.000 0.000 0.000 0.000 0.000 0.000

0.004 0.000 0.016 0.000 0.008 0.004 0.000 0.000 0.085 0.055

0.059 0.087

>Methylation code ID 21352

0.005 0.000 0.000 0.000 0.000 0.015 0.000 0.000 0.005 0.010

0.000 0.005 0.015 0.000 0.005 0.000 0.000 0.000 0.005 0.000

0.073 0.056 0.071 0.068 0.000 0.000 0.008 0.000 0.000 0.012

0.000 0.000 0.004 0.000 0.004 0.000 0.004 0.000 0.012 0.004

0.000 0.000 0.004 0.000 0.076 0.074 0.054 0.079 0.020 0.000

0.000 0.020 0.000 0.029 0.000 0.000 0.000 0.010 0.000 0.000

0.000 0.010 0.000 0.010 0.020 0.000 0.000 0.000 0.057 0.048

0.052 0.059

>Methylation code ID 21354

0.000 0.000 0.008 0.000 0.004 0.004 0.004 0.000 0.004 0.000

0.000 0.000 0.000 0.004 0.013 0.004 0.000 0.004 0.000 0.004

0.066 0.070 0.073 0.069 0.007 0.000 0.007 0.000 0.000 0.014

0.000 0.014 0.007 0.007 0.000 0.007 0.000 0.000 0.007 0.000

0.014 0.000 0.000 0.000 0.069 0.051 0.058 0.069 0.008 0.000

0.000 0.000 0.000 0.008 0.000 0.008 0.000 0.008 0.000 0.000

0.008 0.008 0.008 0.008 0.008 0.008 0.000 0.000 0.065 0.062

0.062 0.062

>Methylation code ID 21354

0.005 0.005 0.010 0.000 0.010 0.005 0.000 0.000 0.000 0.005

0.000 0.000 0.000 0.005 0.005 0.015 0.000 0.000 0.000 0.000

0.070 0.074 0.068 0.057 0.021 0.000 0.000 0.007 0.000 0.000

0.000 0.014 0.000 0.007 0.000 0.000 0.007 0.007 0.007 0.014

0.000 0.007 0.000 0.000 0.067 0.073 0.063 0.039 0.000 0.000

0.000 0.005 0.000 0.000 0.000 0.000 0.000 0.016 0.000 0.000

0.005 0.005 0.011 0.011 0.000 0.000 0.005 0.000 0.075 0.085

0.046 0.067

>Methylation code ID 21354

0.000 0.000 0.000 0.000 0.000 0.000 0.015 0.000 0.005 0.000

0.000 0.000 0.005 0.000 0.015 0.005 0.000 0.010 0.005 0.005

0.049 0.076 0.071 0.072 0.000 0.000 0.017 0.000 0.008 0.017

0.000 0.008 0.000 0.008 0.000 0.000 0.000 0.000 0.000 0.017

0.025 0.008 0.000 0.000 0.040 0.070 0.064 0.050 0.015 0.008

0.000 0.000 0.000 0.000 0.000 0.000 0.000 0.008 0.000 0.000

0.000 0.015 0.008 0.023 0.008 0.008 0.000 0.000 0.061 0.064

0.050 0.066

>Methylation code ID 21357

0.008 0.008 0.000 0.008 0.000 0.017 0.008 0.000 0.000 0.000

0.000 0.000 0.000 0.008 0.000 0.025 0.008 0.008 0.000 0.000

0.057 0.057 0.056 0.063 0.010 0.005 0.000 0.000 0.000 0.005

0.000 0.000 0.000 0.005 0.000 0.005 0.015 0.000 0.005 0.005

0.005 0.000 0.000 0.005 0.058 0.067 0.073 0.068 0.000 0.000

0.000 0.000 0.000 0.011 0.005 0.005 0.000 0.005 0.000 0.000

0.000 0.000 0.016 0.005 0.005 0.005 0.000 0.000 0.077 0.072

0.043 0.082

>Methylation code ID 21357

0.000 0.000 0.000 0.000 0.000 0.011 0.005 0.005 0.000 0.011

0.000 0.000 0.005 0.000 0.011 0.005 0.005 0.005 0.000 0.000

0.072 0.082 0.049 0.066 0.008 0.008 0.000 0.008 0.000 0.008

0.016 0.000 0.000 0.000 0.000 0.000 0.000 0.008 0.000 0.032

0.008 0.000 0.000 0.000 0.049 0.058 0.064 0.066 0.006 0.006

0.000 0.000 0.000 0.006 0.000 0.000 0.000 0.006 0.000 0.006

0.022 0.000 0.006 0.006 0.006 0.000 0.000 0.000 0.055 0.071

0.077 0.064

>Methylation code ID 21357

0.013 0.006 0.000 0.006 0.000 0.006 0.006 0.000 0.000 0.000

0.000 0.000 0.000 0.006 0.006 0.025 0.000 0.000 0.000 0.000

0.059 0.064 0.060 0.075 0.005 0.005 0.000 0.000 0.000 0.005

0.000 0.000 0.000 0.005 0.000 0.005 0.022 0.000 0.005 0.005

0.005 0.000 0.000 0.000 0.052 0.070 0.079 0.067 0.000 0.006

0.000 0.000 0.000 0.011 0.000 0.006 0.000 0.011 0.000 0.000

0.011 0.000 0.011 0.000 0.000 0.006 0.000 0.000 0.065 0.081

0.043 0.084

>Methylation code ID 21357

0.014 0.007 0.000 0.000 0.000 0.000 0.000 0.000 0.000 0.014

0.000 0.007 0.014 0.000 0.007 0.007 0.007 0.000 0.000 0.000

0.044 0.063 0.079 0.072 0.000 0.005 0.005 0.000 0.000 0.005

0.000 0.000 0.000 0.005 0.000 0.000 0.009 0.000 0.009 0.000

0.005 0.005 0.005 0.000 0.067 0.070 0.079 0.065 0.011 0.006

0.000 0.011 0.000 0.006 0.006 0.000 0.000 0.006 0.000 0.000

0.000 0.000 0.006 0.011 0.000 0.000 0.000 0.000 0.059 0.065

0.071 0.075

>Methylation code ID 21357

0.012 0.000 0.000 0.006 0.000 0.006 0.006 0.000 0.000 0.006

0.000 0.000 0.000 0.000 0.012 0.012 0.006 0.006 0.000 0.000

0.060 0.067 0.084 0.052 0.019 0.010 0.000 0.000 0.000 0.000

0.000 0.000 0.000 0.029 0.000 0.010 0.019 0.000 0.000 0.010

0.010 0.000 0.000 0.000 0.047 0.048 0.074 0.057 0.000 0.009

0.005 0.000 0.000 0.000 0.000 0.000 0.000 0.005 0.000 0.000

0.014 0.000 0.005 0.000 0.005 0.005 0.005 0.000 0.049 0.067

0.088 0.077

>Methylation code ID 21357

0.024 0.000 0.008 0.000 0.000 0.008 0.000 0.000 0.008 0.024

0.000 0.008 0.000 0.000 0.000 0.016 0.000 0.000 0.000 0.000

0.062 0.057 0.057 0.060 0.000 0.005 0.005 0.005 0.000 0.000

0.000 0.000 0.000 0.005 0.000 0.000 0.009 0.000 0.009 0.000

0.009 0.000 0.005 0.000 0.063 0.065 0.078 0.076 0.006 0.006

0.000 0.006 0.000 0.012 0.000 0.000 0.006 0.006 0.000 0.000

0.000 0.006 0.006 0.000 0.006 0.006 0.000 0.000 0.080 0.056

0.093 0.038

>Methylation code ID 21357

0.030 0.000 0.000 0.008 0.000 0.008 0.000 0.000 0.000 0.015

0.000 0.000 0.008 0.000 0.008 0.008 0.008 0.000 0.000 0.000

0.057 0.064 0.063 0.058 0.000 0.007 0.000 0.000 0.000 0.013

0.000 0.000 0.007 0.000 0.000 0.000 0.020 0.027 0.000 0.007

0.000 0.000 0.000 0.000 0.065 0.059 0.066 0.064 0.011 0.000

0.005 0.000 0.000 0.005 0.000 0.000 0.005 0.005 0.000 0.000

0.000 0.000 0.011 0.022 0.000 0.000 0.000 0.000 0.074 0.046

0.074 0.074

>Methylation code ID 21357

0.038 0.000 0.000 0.009 0.000 0.019 0.000 0.000 0.000 0.009

0.000 0.000 0.000 0.000 0.009 0.019 0.009 0.000 0.000 0.000

0.044 0.042 0.064 0.069 0.016 0.000 0.000 0.000 0.000 0.016

0.000 0.000 0.008 0.000 0.000 0.000 0.024 0.024 0.000 0.008

0.000 0.000 0.000 0.000 0.063 0.064 0.061 0.051 0.005 0.000

0.005 0.000 0.000 0.005 0.000 0.000 0.000 0.005 0.000 0.000

0.000 0.005 0.015 0.021 0.000 0.000 0.000 0.000 0.060 0.051

0.080 0.081

>Methylation code ID 21357

0.025 0.000 0.000 0.000 0.000 0.019 0.000 0.000 0.000 0.006

0.000 0.000 0.006 0.000 0.013 0.013 0.000 0.000 0.000 0.000

0.080 0.059 0.039 0.074 0.018 0.000 0.006 0.000 0.000 0.012

0.000 0.000 0.000 0.000 0.000 0.000 0.018 0.012 0.006 0.006

0.000 0.000 0.000 0.000 0.066 0.077 0.066 0.045 0.010 0.000

0.000 0.000 0.000 0.005 0.000 0.000 0.000 0.005 0.000 0.000

0.010 0.005 0.014 0.010 0.000 0.000 0.000 0.000 0.062 0.070

0.073 0.071

>Methylation code ID 21357

0.017 0.000 0.006 0.000 0.000 0.011 0.006 0.000 0.000 0.000

0.000 0.000 0.017 0.006 0.006 0.006 0.000 0.000 0.000 0.000

0.062 0.077 0.069 0.052 0.009 0.000 0.000 0.000 0.000 0.004

0.000 0.000 0.000 0.004 0.004 0.000 0.009 0.004 0.013 0.004

0.004 0.000 0.000 0.000 0.058 0.077 0.072 0.069 0.012 0.000

0.000 0.000 0.000 0.018 0.000 0.000 0.000 0.006 0.000 0.000

0.012 0.000 0.012 0.012 0.000 0.000 0.000 0.000 0.081 0.071

0.033 0.075

>Methylation code ID 21357

0.010 0.000 0.000 0.000 0.000 0.005 0.000 0.000 0.000 0.010

0.005 0.000 0.010 0.005 0.010 0.005 0.005 0.000 0.000 0.000

0.049 0.070 0.081 0.071 0.010 0.005 0.000 0.000 0.000 0.010

0.000 0.000 0.000 0.005 0.000 0.000 0.010 0.000 0.010 0.015

0.000 0.000 0.000 0.000 0.075 0.071 0.050 0.072 0.023 0.000

0.006 0.000 0.000 0.006 0.006 0.000 0.000 0.000 0.000 0.000

0.012 0.006 0.006 0.006 0.000 0.000 0.000 0.000 0.067 0.078

0.060 0.059

>Methylation code ID 21357

0.021 0.000 0.005 0.000 0.000 0.005 0.005 0.000 0.000 0.005

0.000 0.000 0.011 0.011 0.005 0.000 0.000 0.000 0.000 0.000

0.065 0.078 0.065 0.056 0.005 0.000 0.000 0.000 0.000 0.005

0.000 0.000 0.000 0.005 0.005 0.000 0.010 0.005 0.010 0.016

0.005 0.000 0.000 0.000 0.053 0.076 0.071 0.066 0.010 0.005

0.000 0.000 0.000 0.010 0.000 0.000 0.000 0.000 0.000 0.000

0.016 0.000 0.010 0.010 0.000 0.000 0.000 0.000 0.079 0.075

0.049 0.068

>Methylation code ID 21357

0.010 0.005 0.000 0.000 0.000 0.010 0.000 0.000 0.000 0.000

0.000 0.000 0.015 0.000 0.010 0.010 0.000 0.005 0.000 0.000

0.077 0.072 0.052 0.066 0.022 0.000 0.005 0.000 0.000 0.005

0.005 0.000 0.000 0.005 0.000 0.000 0.011 0.005 0.005 0.000

0.000 0.000 0.000 0.005 0.062 0.077 0.068 0.056 0.006 0.000

0.000 0.000 0.000 0.006 0.000 0.000 0.000 0.006 0.006 0.000

0.011 0.006 0.011 0.011 0.006 0.000 0.000 0.000 0.055 0.071

0.071 0.071

>Methylation code ID 21357

0.009 0.000 0.000 0.000 0.000 0.018 0.000 0.000 0.000 0.009

0.009 0.009 0.000 0.018 0.000 0.027 0.009 0.000 0.000 0.009

0.034 0.052 0.067 0.066 0.009 0.004 0.000 0.000 0.000 0.000

0.000 0.004 0.000 0.000 0.000 0.000 0.009 0.000 0.013 0.009

0.004 0.004 0.000 0.000 0.058 0.057 0.061 0.103 0.010 0.000

0.000 0.005 0.000 0.005 0.000 0.000 0.000 0.016 0.000 0.000

0.000 0.005 0.010 0.005 0.000 0.000 0.000 0.005 0.071 0.060

0.067 0.073

>Methylation code ID 21357

0.015 0.000 0.000 0.005 0.000 0.005 0.000 0.000 0.000 0.015

0.000 0.000 0.000 0.005 0.010 0.005 0.000 0.000 0.000 0.005

0.068 0.056 0.062 0.084 0.007 0.000 0.000 0.000 0.000 0.015

0.000 0.000 0.000 0.007 0.000 0.007 0.000 0.015 0.007 0.022

0.007 0.000 0.000 0.007 0.036 0.056 0.068 0.078 0.009 0.000

0.000 0.000 0.000 0.000 0.000 0.005 0.000 0.000 0.000 0.000

0.009 0.000 0.014 0.009 0.005 0.005 0.000 0.000 0.057 0.063

0.062 0.095

>Methylation code ID 21357

0.007 0.000 0.000 0.000 0.000 0.014 0.000 0.000 0.000 0.000

0.000 0.007 0.000 0.014 0.007 0.021 0.007 0.007 0.000 0.007

0.049 0.055 0.066 0.072 0.010 0.000 0.000 0.000 0.000 0.005

0.000 0.005 0.000 0.000 0.000 0.000 0.010 0.000 0.015 0.010

0.005 0.005 0.000 0.000 0.057 0.064 0.060 0.089 0.010 0.000

0.000 0.005 0.000 0.010 0.000 0.000 0.000 0.010 0.000 0.000

0.000 0.005 0.010 0.005 0.000 0.000 0.000 0.005 0.068 0.055

0.061 0.087

>Methylation code ID 21357

0.018 0.000 0.000 0.000 0.000 0.012 0.000 0.006 0.000 0.006

0.000 0.000 0.006 0.000 0.012 0.012 0.006 0.000 0.000 0.000

0.075 0.057 0.052 0.069 0.011 0.000 0.000 0.000 0.000 0.016

0.000 0.000 0.000 0.011 0.000 0.000 0.005 0.000 0.016 0.005

0.000 0.000 0.000 0.000 0.079 0.063 0.063 0.064 0.011 0.000

0.000 0.000 0.000 0.017 0.000 0.000 0.000 0.006 0.000 0.000

0.000 0.006 0.011 0.006 0.000 0.006 0.000 0.006 0.061 0.048

0.077 0.080

>Methylation code ID 21357

0.021 0.000 0.000 0.000 0.000 0.014 0.007 0.014 0.000 0.007

0.000 0.000 0.007 0.000 0.007 0.014 0.000 0.000 0.000 0.000

0.063 0.071 0.049 0.061 0.011 0.000 0.000 0.000 0.000 0.011

0.000 0.000 0.000 0.011 0.000 0.000 0.006 0.000 0.011 0.006

0.006 0.000 0.000 0.006 0.062 0.074 0.061 0.069 0.005 0.000

0.005 0.000 0.000 0.010 0.000 0.000 0.000 0.005 0.000 0.000

0.000 0.005 0.010 0.005 0.005 0.005 0.000 0.005 0.068 0.053

0.072 0.078

>Methylation code ID 21357

0.005 0.000 0.005 0.000 0.000 0.010 0.000 0.000 0.000 0.005

0.000 0.000 0.000 0.010 0.010 0.005 0.005 0.005 0.000 0.005

0.066 0.054 0.071 0.077 0.012 0.000 0.000 0.000 0.000 0.012

0.006 0.012 0.000 0.006 0.000 0.000 0.006 0.000 0.012 0.012

0.000 0.000 0.000 0.000 0.064 0.078 0.061 0.051 0.014 0.000

0.000 0.000 0.000 0.014 0.000 0.000 0.000 0.014 0.000 0.000

0.007 0.000 0.007 0.007 0.007 0.000 0.000 0.007 0.059 0.073

0.063 0.062

>Methylation code ID 21357

0.012 0.000 0.000 0.000 0.000 0.018 0.000 0.000 0.006 0.006

0.000 0.000 0.006 0.000 0.006 0.006 0.006 0.000 0.000 0.006

0.063 0.074 0.062 0.062 0.005 0.000 0.005 0.000 0.000 0.005

0.000 0.000 0.005 0.005 0.000 0.000 0.000 0.010 0.010 0.000

0.005 0.010 0.000 0.005 0.075 0.051 0.072 0.068 0.007 0.000

0.000 0.000 0.000 0.014 0.007 0.014 0.000 0.007 0.000 0.000

0.000 0.000 0.014 0.014 0.000 0.000 0.000 0.000 0.063 0.081

0.062 0.053

>Methylation code ID 21357

0.000 0.000 0.000 0.013 0.000 0.006 0.000 0.000 0.000 0.006

0.000 0.000 0.006 0.000 0.006 0.006 0.013 0.006 0.006 0.000

0.071 0.081 0.068 0.044 0.000 0.000 0.000 0.010 0.000 0.015

0.000 0.000 0.000 0.000 0.000 0.005 0.005 0.000 0.015 0.010

0.000 0.000 0.000 0.000 0.088 0.054 0.059 0.071 0.000 0.000

0.006 0.006 0.000 0.006 0.000 0.000 0.000 0.006 0.000 0.006

0.006 0.000 0.006 0.006 0.006 0.000 0.006 0.000 0.072 0.071

0.049 0.082

>Methylation code ID 21357

0.005 0.000 0.005 0.000 0.000 0.005 0.000 0.000 0.000 0.011

0.000 0.005 0.005 0.000 0.011 0.005 0.005 0.000 0.000 0.000

0.084 0.080 0.035 0.075 0.000 0.000 0.007 0.014 0.000 0.000

0.000 0.000 0.000 0.014 0.000 0.000 0.014 0.000 0.000 0.000

0.014 0.007 0.007 0.000 0.056 0.074 0.074 0.054 0.000 0.000

0.000 0.010 0.000 0.005 0.000 0.000 0.000 0.000 0.000 0.005

0.010 0.000 0.010 0.016 0.005 0.000 0.000 0.000 0.070 0.059

0.069 0.072

>Methylation code ID 21357

0.005 0.000 0.005 0.000 0.000 0.005 0.000 0.000 0.000 0.010

0.000 0.005 0.005 0.000 0.010 0.005 0.005 0.005 0.000 0.000

0.076 0.068 0.050 0.078 0.000 0.000 0.006 0.012 0.006 0.000

0.000 0.000 0.000 0.012 0.000 0.000 0.012 0.006 0.000 0.000

0.012 0.000 0.006 0.000 0.053 0.071 0.076 0.064 0.000 0.005

0.000 0.005 0.005 0.005 0.000 0.000 0.000 0.000 0.000 0.005

0.009 0.000 0.009 0.009 0.005 0.000 0.000 0.000 0.073 0.063

0.060 0.082

>Methylation code ID 21357

0.000 0.000 0.006 0.012 0.006 0.000 0.000 0.000 0.000 0.006

0.000 0.000 0.012 0.006 0.000 0.000 0.012 0.000 0.006 0.000

0.050 0.070 0.080 0.066 0.000 0.004 0.000 0.004 0.004 0.004

0.000 0.000 0.000 0.000 0.000 0.004 0.009 0.000 0.009 0.013

0.004 0.000 0.000 0.000 0.071 0.068 0.066 0.071 0.006 0.000

0.006 0.000 0.000 0.006 0.000 0.000 0.000 0.013 0.000 0.006

0.000 0.000 0.006 0.006 0.006 0.013 0.000 0.000 0.072 0.063

0.055 0.073

>Methylation code ID 21357

0.012 0.000 0.006 0.000 0.000 0.006 0.006 0.000 0.000 0.000

0.000 0.006 0.006 0.000 0.006 0.006 0.012 0.012 0.000 0.000

0.067 0.067 0.070 0.051 0.000 0.000 0.007 0.000 0.007 0.007

0.007 0.000 0.000 0.000 0.000 0.000 0.007 0.013 0.013 0.000

0.020 0.000 0.000 0.000 0.053 0.071 0.058 0.072 0.000 0.000

0.006 0.000 0.006 0.006 0.006 0.000 0.000 0.000 0.000 0.000

0.000 0.012 0.012 0.012 0.012 0.000 0.000 0.000 0.059 0.049

0.076 0.074

>Methylation code ID 21357

0.000 0.000 0.006 0.000 0.000 0.006 0.012 0.000 0.000 0.000

0.000 0.000 0.000 0.012 0.012 0.006 0.018 0.006 0.000 0.000

0.061 0.051 0.073 0.070 0.005 0.000 0.005 0.000 0.000 0.005

0.005 0.000 0.000 0.005 0.000 0.005 0.010 0.000 0.005 0.000

0.016 0.000 0.000 0.005 0.066 0.064 0.068 0.068 0.000 0.000

0.006 0.000 0.000 0.006 0.012 0.006 0.000 0.000 0.000 0.000

0.000 0.006 0.012 0.006 0.017 0.000 0.000 0.000 0.047 0.069

0.063 0.085

>Methylation code ID 21357

0.000 0.000 0.006 0.000 0.000 0.006 0.006 0.000 0.000 0.000

0.000 0.000 0.000 0.013 0.013 0.013 0.019 0.006 0.000 0.000

0.062 0.047 0.071 0.070 0.005 0.000 0.005 0.000 0.000 0.005

0.005 0.000 0.000 0.005 0.000 0.005 0.015 0.000 0.005 0.000

0.010 0.000 0.000 0.005 0.062 0.068 0.071 0.067 0.000 0.000

0.006 0.000 0.000 0.006 0.006 0.006 0.000 0.006 0.000 0.000

0.000 0.006 0.012 0.006 0.018 0.000 0.000 0.000 0.045 0.063

0.070 0.085

>Methylation code ID 21362

0.012 0.000 0.000 0.000 0.012 0.000 0.000 0.000 0.000 0.012

0.000 0.000 0.018 0.006 0.006 0.012 0.000 0.000 0.000 0.000

0.061 0.065 0.069 0.063 0.009 0.000 0.000 0.000 0.009 0.000

0.000 0.000 0.000 0.000 0.000 0.004 0.018 0.000 0.009 0.009

0.000 0.000 0.000 0.000 0.078 0.073 0.077 0.048 0.006 0.000

0.000 0.000 0.000 0.000 0.000 0.000 0.000 0.006 0.000 0.000

0.018 0.006 0.012 0.012 0.006 0.006 0.000 0.000 0.071 0.088

0.036 0.067

>Methylation code ID 21362

0.011 0.000 0.000 0.000 0.006 0.000 0.000 0.000 0.000 0.006

0.000 0.000 0.017 0.000 0.023 0.011 0.000 0.000 0.000 0.000

0.058 0.056 0.072 0.073 0.015 0.000 0.000 0.000 0.005 0.010

0.000 0.000 0.000 0.000 0.000 0.000 0.020 0.000 0.010 0.005

0.000 0.000 0.000 0.000 0.072 0.067 0.074 0.056 0.011 0.000

0.000 0.000 0.000 0.000 0.000 0.000 0.000 0.005 0.000 0.000

0.022 0.005 0.011 0.005 0.000 0.005 0.000 0.000 0.067 0.073

0.052 0.076

>Methylation code ID 21362

0.016 0.000 0.000 0.000 0.000 0.000 0.000 0.000 0.000 0.005

0.000 0.000 0.021 0.005 0.011 0.005 0.000 0.005 0.000 0.000

0.067 0.071 0.053 0.073 0.010 0.000 0.000 0.000 0.005 0.000

0.000 0.000 0.000 0.005 0.000 0.000 0.016 0.000 0.021 0.010

0.000 0.000 0.000 0.000 0.066 0.055 0.076 0.069 0.016 0.000

0.000 0.000 0.005 0.011 0.000 0.000 0.000 0.000 0.000 0.000

0.016 0.000 0.011 0.005 0.000 0.000 0.000 0.000 0.065 0.063

0.077 0.063

>Methylation code ID 21362

0.016 0.000 0.000 0.000 0.005 0.010 0.000 0.000 0.000 0.000

0.000 0.000 0.021 0.000 0.010 0.005 0.000 0.000 0.000 0.000

0.066 0.063 0.078 0.059 0.017 0.000 0.000 0.000 0.000 0.000

0.000 0.000 0.000 0.006 0.000 0.000 0.022 0.006 0.011 0.006

0.000 0.006 0.000 0.000 0.071 0.069 0.043 0.077 0.011 0.000

0.000 0.000 0.005 0.000 0.000 0.000 0.000 0.005 0.000 0.000

0.011 0.000 0.022 0.011 0.000 0.000 0.000 0.000 0.060 0.054

0.078 0.076

>Methylation code ID 21362

0.013 0.000 0.000 0.007 0.000 0.013 0.007 0.000 0.007 0.000

0.000 0.000 0.033 0.000 0.000 0.000 0.000 0.007 0.000 0.000

0.056 0.056 0.063 0.074 0.011 0.000 0.000 0.000 0.000 0.000

0.000 0.000 0.000 0.006 0.000 0.000 0.017 0.006 0.017 0.006

0.006 0.000 0.000 0.000 0.058 0.064 0.074 0.071 0.010 0.000

0.000 0.000 0.000 0.005 0.000 0.000 0.005 0.000 0.000 0.000

0.015 0.000 0.020 0.005 0.000 0.000 0.000 0.000 0.037 0.050

0.084 0.103

>Methylation code ID 21362

0.006 0.000 0.000 0.006 0.000 0.013 0.006 0.000 0.013 0.000

0.000 0.000 0.031 0.000 0.000 0.000 0.000 0.006 0.000 0.000

0.058 0.063 0.057 0.073 0.011 0.000 0.000 0.000 0.000 0.000

0.000 0.000 0.000 0.006 0.000 0.000 0.011 0.006 0.022 0.006

0.006 0.000 0.000 0.000 0.043 0.063 0.079 0.082 0.009 0.000

0.000 0.005 0.000 0.005 0.000 0.000 0.005 0.000 0.000 0.000

0.009 0.000 0.019 0.005 0.000 0.000 0.000 0.000 0.037 0.052

0.080 0.108

>Methylation code ID 21362

0.006 0.000 0.000 0.006 0.000 0.018 0.006 0.000 0.012 0.000

0.000 0.000 0.023 0.000 0.000 0.000 0.000 0.006 0.000 0.000

0.063 0.065 0.050 0.080 0.011 0.000 0.000 0.000 0.000 0.006

0.000 0.000 0.000 0.006 0.000 0.000 0.011 0.006 0.017 0.006

0.006 0.000 0.000 0.000 0.044 0.063 0.085 0.076 0.005 0.000

0.000 0.005 0.000 0.010 0.000 0.000 0.005 0.000 0.000 0.000

0.010 0.000 0.019 0.005 0.000 0.000 0.000 0.000 0.041 0.052

0.080 0.104

>Methylation code ID 21362

0.011 0.000 0.000 0.006 0.000 0.017 0.000 0.000 0.006 0.000

0.000 0.000 0.006 0.000 0.023 0.006 0.000 0.000 0.000 0.000

0.039 0.058 0.072 0.090 0.005 0.000 0.000 0.005 0.000 0.021

0.000 0.000 0.010 0.005 0.000 0.000 0.010 0.000 0.005 0.000

0.000 0.005 0.000 0.000 0.071 0.057 0.052 0.085 0.009 0.000

0.000 0.000 0.000 0.009 0.000 0.000 0.000 0.000 0.000 0.000

0.005 0.005 0.014 0.005 0.000 0.005 0.000 0.000 0.033 0.060

0.096 0.092

>Methylation code ID 21362

0.005 0.000 0.000 0.000 0.000 0.011 0.000 0.000 0.000 0.000

0.000 0.000 0.011 0.000 0.016 0.011 0.005 0.005 0.000 0.000

0.040 0.066 0.084 0.079 0.015 0.000 0.000 0.008 0.000 0.023

0.000 0.000 0.008 0.023 0.000 0.000 0.000 0.008 0.008 0.008

0.000 0.000 0.000 0.000 0.040 0.069 0.067 0.059 0.000 0.000

0.000 0.005 0.000 0.011 0.000 0.000 0.011 0.005 0.000 0.000

0.005 0.000 0.005 0.011 0.000 0.005 0.000 0.005 0.076 0.053

0.053 0.087

>Methylation code ID 21362

0.005 0.000 0.005 0.000 0.005 0.005 0.000 0.000 0.000 0.000

0.005 0.000 0.005 0.000 0.014 0.009 0.005 0.000 0.000 0.005

0.062 0.063 0.063 0.085 0.008 0.008 0.000 0.008 0.000 0.016

0.008 0.000 0.000 0.024 0.000 0.000 0.000 0.008 0.000 0.008

0.016 0.000 0.000 0.000 0.052 0.061 0.068 0.050 0.000 0.000

0.005 0.005 0.005 0.000 0.000 0.005 0.000 0.005 0.000 0.000

0.010 0.000 0.010 0.010 0.000 0.000 0.000 0.005 0.064 0.049

0.083 0.077

>Methylation code ID 21362

0.008 0.008 0.000 0.008 0.000 0.016 0.008 0.000 0.000 0.024

0.000 0.000 0.000 0.000 0.000 0.016 0.016 0.000 0.000 0.000

0.060 0.059 0.065 0.046 0.000 0.000 0.005 0.005 0.005 0.000

0.000 0.005 0.000 0.005 0.000 0.000 0.014 0.000 0.009 0.005

0.000 0.005 0.000 0.005 0.067 0.050 0.077 0.080 0.000 0.000

0.004 0.000 0.009 0.004 0.000 0.000 0.000 0.000 0.004 0.000

0.004 0.000 0.013 0.004 0.004 0.000 0.000 0.004 0.054 0.066

0.072 0.090

>Methylation code ID 21362

0.008 0.008 0.000 0.008 0.000 0.015 0.008 0.000 0.000 0.023

0.000 0.000 0.000 0.000 0.000 0.015 0.015 0.000 0.000 0.000

0.061 0.062 0.065 0.045 0.000 0.004 0.004 0.004 0.004 0.000

0.000 0.004 0.000 0.004 0.000 0.000 0.013 0.000 0.009 0.004

0.000 0.004 0.000 0.000 0.068 0.052 0.078 0.078 0.004 0.000

0.004 0.000 0.008 0.004 0.000 0.000 0.000 0.000 0.004 0.000

0.004 0.000 0.012 0.004 0.000 0.000 0.000 0.004 0.056 0.061

0.069 0.100

>Methylation code ID 21362

0.011 0.000 0.005 0.011 0.011 0.000 0.000 0.000 0.000 0.000

0.000 0.000 0.005 0.005 0.005 0.000 0.005 0.005 0.000 0.000

0.062 0.068 0.076 0.061 0.000 0.004 0.000 0.000 0.000 0.004

0.000 0.000 0.000 0.018 0.000 0.000 0.009 0.000 0.013 0.004

0.004 0.000 0.000 0.000 0.040 0.073 0.086 0.076 0.000 0.010

0.005 0.000 0.000 0.014 0.000 0.000 0.000 0.000 0.000 0.000

0.005 0.005 0.010 0.005 0.000 0.005 0.000 0.000 0.059 0.076

0.054 0.087

>Methylation code ID 21362

0.006 0.000 0.006 0.011 0.006 0.006 0.000 0.000 0.000 0.006

0.000 0.006 0.000 0.006 0.006 0.000 0.006 0.006 0.000 0.000

0.059 0.068 0.071 0.069 0.000 0.006 0.000 0.000 0.000 0.012

0.000 0.006 0.000 0.012 0.000 0.000 0.012 0.000 0.017 0.000

0.006 0.000 0.000 0.000 0.041 0.065 0.079 0.079 0.000 0.004

0.004 0.004 0.004 0.013 0.000 0.000 0.000 0.000 0.000 0.000

0.000 0.004 0.008 0.000 0.000 0.004 0.004 0.000 0.068 0.066

0.068 0.080

>Methylation code ID 21362

0.000 0.000 0.000 0.004 0.009 0.009 0.000 0.004 0.004 0.009

0.000 0.000 0.004 0.000 0.009 0.000 0.000 0.000 0.000 0.000

0.065 0.074 0.073 0.068 0.000 0.004 0.004 0.004 0.004 0.008

0.000 0.000 0.000 0.000 0.000 0.000 0.004 0.000 0.008 0.013

0.000 0.000 0.004 0.000 0.078 0.061 0.070 0.070 0.005 0.000

0.000 0.015 0.000 0.005 0.000 0.005 0.000 0.005 0.000 0.005

0.000 0.000 0.010 0.000 0.000 0.010 0.000 0.000 0.081 0.054

0.090 0.046

>Methylation code ID 21362

0.000 0.004 0.000 0.004 0.008 0.008 0.000 0.004 0.004 0.000

0.000 0.000 0.004 0.000 0.008 0.000 0.004 0.000 0.000 0.000

0.072 0.084 0.075 0.056 0.000 0.000 0.005 0.005 0.005 0.009

0.000 0.000 0.000 0.000 0.000 0.000 0.005 0.000 0.014 0.014

0.000 0.005 0.000 0.000 0.081 0.074 0.073 0.045 0.005 0.000

0.000 0.015 0.000 0.000 0.000 0.005 0.000 0.010 0.000 0.005

0.000 0.000 0.010 0.000 0.000 0.005 0.000 0.005 0.072 0.051

0.092 0.061

>Methylation code ID 21362

0.000 0.000 0.000 0.019 0.006 0.000 0.000 0.006 0.000 0.019

0.000 0.000 0.000 0.006 0.006 0.000 0.000 0.013 0.000 0.006

0.064 0.069 0.076 0.042 0.000 0.008 0.000 0.000 0.011 0.004

0.000 0.000 0.004 0.000 0.000 0.000 0.000 0.000 0.008 0.008

0.004 0.000 0.000 0.004 0.072 0.079 0.068 0.065 0.006 0.000

0.006 0.000 0.000 0.012 0.000 0.000 0.000 0.000 0.000 0.000

0.006 0.000 0.012 0.018 0.000 0.012 0.000 0.000 0.078 0.082

0.066 0.036

>Methylation code ID 21362

0.007 0.000 0.000 0.000 0.000 0.020 0.000 0.000 0.007 0.000

0.000 0.000 0.007 0.000 0.013 0.013 0.007 0.013 0.000 0.000

0.077 0.044 0.076 0.050 0.000 0.000 0.000 0.007 0.007 0.020

0.000 0.000 0.000 0.020 0.000 0.000 0.007 0.007 0.007 0.007

0.000 0.007 0.000 0.000 0.068 0.073 0.048 0.059 0.004 0.004

0.000 0.004 0.004 0.004 0.000 0.000 0.000 0.004 0.000 0.000

0.000 0.000 0.009 0.009 0.000 0.000 0.004 0.004 0.073 0.058

0.078 0.073

>Methylation code ID 21362

0.006 0.000 0.000 0.006 0.000 0.031 0.006 0.000 0.000 0.006

0.000 0.000 0.006 0.000 0.006 0.006 0.000 0.006 0.000 0.000

0.061 0.062 0.060 0.071 0.010 0.000 0.000 0.010 0.000 0.010

0.000 0.000 0.000 0.005 0.000 0.000 0.000 0.000 0.010 0.005

0.005 0.000 0.005 0.005 0.075 0.068 0.060 0.063 0.005 0.000

0.000 0.000 0.000 0.020 0.000 0.000 0.005 0.000 0.000 0.000

0.000 0.000 0.015 0.005 0.005 0.005 0.000 0.000 0.047 0.052

0.097 0.077

>Methylation code ID 21362

0.006 0.000 0.000 0.006 0.000 0.030 0.006 0.000 0.000 0.006

0.000 0.000 0.006 0.000 0.006 0.006 0.000 0.006 0.000 0.000

0.063 0.065 0.059 0.069 0.011 0.000 0.000 0.011 0.000 0.011

0.000 0.000 0.000 0.005 0.000 0.000 0.000 0.000 0.011 0.011

0.005 0.000 0.005 0.000 0.067 0.068 0.072 0.058 0.005 0.000

0.000 0.000 0.000 0.018 0.000 0.000 0.005 0.000 0.000 0.000

0.000 0.000 0.018 0.005 0.000 0.005 0.000 0.000 0.054 0.060

0.101 0.064

>Methylation code ID 21362

0.008 0.000 0.000 0.000 0.000 0.019 0.000 0.000 0.004 0.000

0.000 0.000 0.000 0.000 0.019 0.000 0.000 0.000 0.000 0.000

0.053 0.078 0.098 0.055 0.006 0.000 0.000 0.012 0.000 0.036

0.006 0.000 0.000 0.000 0.000 0.000 0.006 0.000 0.006 0.000

0.000 0.006 0.000 0.000 0.065 0.063 0.064 0.063 0.012 0.000

0.000 0.012 0.000 0.018 0.000 0.000 0.000 0.000 0.000 0.000

0.000 0.000 0.012 0.012 0.006 0.000 0.000 0.000 0.065 0.054

0.071 0.069

>Methylation code ID 21362

0.008 0.000 0.000 0.016 0.000 0.039 0.008 0.000 0.000 0.000

0.000 0.000 0.008 0.000 0.000 0.000 0.008 0.008 0.000 0.000

0.049 0.067 0.063 0.060 0.011 0.000 0.000 0.005 0.000 0.011

0.005 0.000 0.005 0.000 0.000 0.000 0.000 0.000 0.011 0.016

0.005 0.000 0.000 0.000 0.059 0.061 0.073 0.071 0.009 0.000

0.000 0.000 0.000 0.013 0.000 0.004 0.000 0.000 0.000 0.004

0.000 0.000 0.021 0.000 0.000 0.000 0.000 0.000 0.044 0.082

0.088 0.068

>Methylation code ID 21362

0.006 0.000 0.000 0.013 0.000 0.025 0.006 0.000 0.000 0.000

0.000 0.000 0.006 0.000 0.006 0.000 0.006 0.006 0.000 0.000

0.050 0.067 0.070 0.071 0.010 0.000 0.000 0.005 0.000 0.010

0.005 0.000 0.005 0.000 0.000 0.000 0.000 0.000 0.010 0.015

0.005 0.000 0.000 0.000 0.059 0.061 0.079 0.068 0.014 0.000

0.000 0.000 0.000 0.014 0.000 0.005 0.000 0.000 0.000 0.005

0.000 0.000 0.018 0.000 0.000 0.000 0.000 0.000 0.049 0.094

0.079 0.056

>Methylation code ID 21362

0.013 0.000 0.000 0.000 0.000 0.013 0.000 0.004 0.000 0.000

0.000 0.004 0.000 0.000 0.017 0.000 0.004 0.000 0.000 0.000

0.065 0.091 0.066 0.055 0.011 0.000 0.000 0.011 0.000 0.011

0.006 0.000 0.000 0.000 0.000 0.000 0.011 0.000 0.006 0.000

0.006 0.006 0.000 0.000 0.064 0.078 0.069 0.055 0.006 0.000

0.000 0.000 0.000 0.011 0.011 0.000 0.006 0.000 0.000 0.000

0.000 0.000 0.011 0.017 0.006 0.000 0.000 0.000 0.061 0.062

0.067 0.077

>Methylation code ID 21362

0.005 0.000 0.005 0.000 0.000 0.005 0.016 0.000 0.005 0.005

0.000 0.000 0.005 0.000 0.005 0.011 0.005 0.000 0.000 0.000

0.062 0.066 0.065 0.072 0.009 0.000 0.000 0.000 0.000 0.005

0.000 0.005 0.000 0.000 0.000 0.005 0.000 0.000 0.018 0.000

0.014 0.000 0.005 0.000 0.062 0.084 0.059 0.069 0.006 0.000

0.000 0.006 0.000 0.013 0.000 0.000 0.000 0.000 0.000 0.000

0.025 0.000 0.006 0.000 0.006 0.006 0.000 0.000 0.059 0.058

0.073 0.073

>Methylation code ID 21362

0.006 0.000 0.000 0.006 0.000 0.018 0.000 0.000 0.000 0.000

0.000 0.000 0.024 0.000 0.006 0.000 0.006 0.006 0.000 0.000

0.061 0.059 0.067 0.073 0.005 0.000 0.005 0.005 0.000 0.005

0.016 0.000 0.005 0.005 0.000 0.000 0.005 0.000 0.005 0.011

0.000 0.000 0.000 0.000 0.066 0.071 0.054 0.073 0.010 0.000

0.000 0.000 0.000 0.005 0.000 0.005 0.000 0.000 0.000 0.005

0.000 0.000 0.015 0.000 0.015 0.000 0.005 0.000 0.069 0.079

0.050 0.074

>Methylation code ID 21362

0.000 0.000 0.006 0.006 0.000 0.006 0.019 0.000 0.000 0.013

0.000 0.000 0.025 0.000 0.000 0.006 0.000 0.000 0.000 0.000

0.075 0.053 0.059 0.064 0.005 0.000 0.000 0.000 0.005 0.000

0.000 0.005 0.000 0.000 0.000 0.000 0.014 0.000 0.009 0.005

0.014 0.000 0.005 0.000 0.070 0.068 0.065 0.071 0.007 0.000

0.000 0.000 0.000 0.013 0.000 0.000 0.000 0.000 0.000 0.000

0.039 0.000 0.007 0.007 0.000 0.007 0.000 0.000 0.062 0.074

0.054 0.065

>Methylation code ID 21362

0.014 0.000 0.000 0.000 0.000 0.014 0.000 0.000 0.000 0.000

0.000 0.000 0.028 0.000 0.007 0.014 0.000 0.007 0.000 0.000

0.064 0.075 0.060 0.050 0.000 0.000 0.000 0.014 0.000 0.014

0.007 0.000 0.000 0.007 0.000 0.000 0.035 0.007 0.000 0.007

0.000 0.000 0.000 0.000 0.071 0.056 0.060 0.057 0.004 0.000

0.000 0.000 0.004 0.000 0.000 0.004 0.000 0.004 0.000 0.000

0.013 0.000 0.013 0.009 0.000 0.000 0.000 0.000 0.060 0.072

0.078 0.071

>Methylation code ID 21362

0.008 0.000 0.000 0.000 0.008 0.008 0.000 0.008 0.000 0.015

0.000 0.000 0.015 0.000 0.008 0.015 0.000 0.008 0.000 0.000

0.061 0.063 0.066 0.052 0.006 0.000 0.000 0.006 0.013 0.006

0.006 0.000 0.000 0.019 0.000 0.000 0.006 0.000 0.006 0.006

0.000 0.000 0.000 0.000 0.077 0.051 0.058 0.072 0.000 0.006

0.000 0.000 0.006 0.000 0.000 0.000 0.000 0.006 0.000 0.006

0.000 0.006 0.006 0.011 0.006 0.006 0.006 0.000 0.068 0.078

0.068 0.057

>Methylation code ID 21362

0.000 0.007 0.000 0.000 0.000 0.013 0.000 0.000 0.007 0.007

0.000 0.000 0.000 0.007 0.007 0.007 0.007 0.000 0.007 0.000

0.074 0.079 0.065 0.050 0.019 0.000 0.000 0.000 0.000 0.013

0.000 0.000 0.006 0.000 0.000 0.006 0.006 0.000 0.006 0.006

0.000 0.006 0.000 0.000 0.047 0.067 0.069 0.082 0.000 0.000

0.000 0.011 0.000 0.017 0.006 0.006 0.000 0.011 0.000 0.000

0.006 0.000 0.006 0.006 0.000 0.000 0.000 0.000 0.064 0.068

0.072 0.062

>Methylation code ID 21362

0.014 0.000 0.000 0.000 0.007 0.021 0.000 0.014 0.007 0.000

0.000 0.007 0.014 0.000 0.000 0.000 0.000 0.000 0.000 0.000

0.055 0.056 0.079 0.061 0.005 0.000 0.000 0.005 0.000 0.010

0.000 0.005 0.000 0.005 0.000 0.000 0.010 0.000 0.010 0.015

0.000 0.000 0.000 0.000 0.080 0.037 0.083 0.069 0.000 0.000

0.000 0.000 0.000 0.011 0.011 0.000 0.005 0.000 0.000 0.000

0.011 0.000 0.005 0.000 0.000 0.005 0.005 0.000 0.081 0.040

0.073 0.085

>Methylation code ID 21362

0.000 0.000 0.000 0.000 0.000 0.011 0.011 0.000 0.005 0.000

0.000 0.000 0.016 0.000 0.005 0.000 0.000 0.005 0.005 0.000

0.085 0.038 0.076 0.077 0.006 0.000 0.000 0.000 0.006 0.017

0.000 0.012 0.006 0.000 0.000 0.006 0.012 0.000 0.006 0.000

0.000 0.000 0.000 0.000 0.063 0.064 0.079 0.058 0.005 0.000

0.000 0.005 0.000 0.010 0.000 0.005 0.000 0.000 0.000 0.000

0.010 0.000 0.010 0.015 0.000 0.000 0.000 0.000 0.081 0.037

0.080 0.074

>Methylation code ID 21362

0.014 0.000 0.000 0.000 0.000 0.007 0.000 0.000 0.000 0.007

0.000 0.000 0.021 0.007 0.007 0.028 0.000 0.000 0.000 0.000

0.077 0.048 0.062 0.054 0.000 0.004 0.000 0.000 0.000 0.004

0.008 0.000 0.000 0.004 0.000 0.000 0.012 0.000 0.012 0.000

0.000 0.008 0.000 0.000 0.087 0.042 0.088 0.062 0.016 0.000

0.000 0.005 0.005 0.000 0.000 0.011 0.000 0.000 0.000 0.000

0.011 0.000 0.005 0.005 0.000 0.000 0.005 0.000 0.075 0.058

0.069 0.066

>Methylation code ID 21362

0.016 0.000 0.000 0.005 0.005 0.005 0.000 0.010 0.000 0.000

0.000 0.000 0.010 0.000 0.005 0.005 0.000 0.000 0.005 0.000

0.080 0.055 0.070 0.061 0.007 0.000 0.000 0.000 0.000 0.007

0.000 0.000 0.000 0.007 0.000 0.000 0.022 0.007 0.007 0.029

0.000 0.007 0.000 0.000 0.063 0.052 0.063 0.060 0.000 0.005

0.000 0.000 0.000 0.005 0.010 0.000 0.000 0.005 0.000 0.000

0.014 0.000 0.010 0.000 0.000 0.010 0.000 0.000 0.088 0.035

0.096 0.057

>Methylation code ID 21368

0.012 0.000 0.000 0.000 0.000 0.006 0.000 0.000 0.000 0.017

0.000 0.000 0.017 0.000 0.012 0.000 0.000 0.012 0.000 0.000

0.052 0.083 0.070 0.052 0.007 0.007 0.000 0.007 0.007 0.000

0.000 0.000 0.000 0.015 0.000 0.000 0.007 0.015 0.000 0.015

0.000 0.007 0.000 0.000 0.066 0.063 0.049 0.067 0.000 0.000

0.000 0.000 0.000 0.000 0.000 0.000 0.000 0.000 0.000 0.000

0.019 0.000 0.013 0.044 0.000 0.000 0.000 0.000 0.044 0.060

0.073 0.082

>Methylation code ID 21368

0.011 0.000 0.000 0.000 0.005 0.000 0.000 0.000 0.000 0.011

0.005 0.011 0.011 0.000 0.005 0.005 0.005 0.000 0.000 0.000

0.074 0.057 0.071 0.060 0.000 0.008 0.000 0.000 0.000 0.008

0.017 0.000 0.000 0.008 0.000 0.000 0.017 0.008 0.000 0.000

0.017 0.008 0.000 0.000 0.058 0.077 0.056 0.050 0.010 0.005

0.005 0.005 0.000 0.000 0.005 0.000 0.005 0.005 0.000 0.000

0.000 0.005 0.005 0.000 0.005 0.000 0.000 0.000 0.050 0.077

0.084 0.066

>Methylation code ID 21368

0.020 0.010 0.005 0.000 0.000 0.005 0.000 0.005 0.005 0.000

0.000 0.000 0.000 0.000 0.005 0.000 0.010 0.000 0.000 0.000

0.066 0.075 0.074 0.055 0.017 0.000 0.000 0.000 0.006 0.000

0.006 0.000 0.000 0.017 0.000 0.000 0.012 0.000 0.006 0.000

0.006 0.000 0.000 0.000 0.074 0.069 0.061 0.060 0.000 0.005

0.005 0.000 0.000 0.000 0.011 0.000 0.000 0.011 0.000 0.000

0.000 0.005 0.011 0.000 0.005 0.000 0.005 0.000 0.073 0.075

0.078 0.050

>Methylation code ID 21368

0.009 0.009 0.000 0.000 0.000 0.009 0.005 0.005 0.005 0.005

0.000 0.000 0.000 0.000 0.005 0.000 0.009 0.000 0.000 0.000

0.071 0.076 0.064 0.062 0.015 0.005 0.005 0.000 0.005 0.000

0.010 0.000 0.000 0.005 0.000 0.000 0.005 0.000 0.005 0.000

0.005 0.000 0.000 0.000 0.073 0.077 0.059 0.064 0.005 0.000

0.005 0.000 0.000 0.000 0.005 0.005 0.000 0.009 0.000 0.000

0.000 0.005 0.014 0.000 0.005 0.000 0.005 0.000 0.080 0.066

0.083 0.048

>Methylation code ID 21368

0.014 0.005 0.005 0.000 0.000 0.000 0.010 0.000 0.000 0.005

0.000 0.000 0.005 0.000 0.010 0.000 0.005 0.000 0.000 0.000

0.071 0.072 0.060 0.072 0.005 0.000 0.005 0.000 0.000 0.005

0.005 0.009 0.000 0.005 0.000 0.000 0.000 0.005 0.014 0.000

0.005 0.000 0.005 0.000 0.083 0.060 0.076 0.056 0.011 0.005

0.000 0.000 0.000 0.011 0.005 0.000 0.005 0.005 0.000 0.000

0.000 0.000 0.005 0.005 0.011 0.000 0.000 0.000 0.070 0.085

0.060 0.053

>Methylation code ID 21368

0.016 0.005 0.000 0.000 0.000 0.010 0.005 0.000 0.005 0.005

0.000 0.000 0.000 0.000 0.005 0.005 0.010 0.000 0.000 0.000

0.068 0.082 0.065 0.051 0.013 0.004 0.004 0.000 0.000 0.000

0.009 0.000 0.000 0.004 0.000 0.000 0.009 0.000 0.009 0.000

0.004 0.000 0.000 0.000 0.077 0.072 0.060 0.066 0.005 0.000

0.005 0.000 0.000 0.005 0.005 0.010 0.000 0.005 0.000 0.000

0.000 0.005 0.015 0.000 0.005 0.000 0.000 0.000 0.079 0.062

0.071 0.060

>Methylation code ID 21368

0.005 0.000 0.000 0.000 0.005 0.005 0.021 0.010 0.000 0.005

0.000 0.000 0.000 0.000 0.010 0.000 0.005 0.000 0.000 0.000

0.075 0.071 0.044 0.077 0.014 0.007 0.000 0.000 0.000 0.007

0.007 0.000 0.000 0.007 0.000 0.000 0.000 0.000 0.007 0.014

0.028 0.000 0.000 0.000 0.077 0.067 0.043 0.058 0.011 0.005

0.005 0.000 0.000 0.000 0.011 0.000 0.000 0.005 0.000 0.000

0.016 0.000 0.005 0.000 0.005 0.000 0.000 0.000 0.067 0.060

0.080 0.063

>Methylation code ID 21368

0.006 0.000 0.000 0.000 0.006 0.006 0.026 0.006 0.000 0.006

0.000 0.000 0.000 0.006 0.006 0.000 0.000 0.000 0.000 0.000

0.072 0.075 0.053 0.062 0.011 0.011 0.006 0.000 0.000 0.000

0.000 0.000 0.000 0.000 0.000 0.006 0.000 0.000 0.006 0.011

0.022 0.000 0.000 0.000 0.083 0.060 0.036 0.083 0.015 0.000

0.000 0.000 0.000 0.000 0.000 0.000 0.000 0.005 0.005 0.000

0.015 0.000 0.015 0.000 0.005 0.000 0.000 0.000 0.080 0.062

0.078 0.051

>Methylation code ID 21368

0.009 0.009 0.005 0.000 0.000 0.000 0.005 0.000 0.000 0.000

0.000 0.005 0.005 0.000 0.005 0.005 0.014 0.000 0.000 0.000

0.084 0.061 0.036 0.091 0.014 0.000 0.000 0.000 0.000 0.000

0.000 0.000 0.000 0.005 0.005 0.000 0.009 0.000 0.018 0.000

0.009 0.000 0.000 0.000 0.085 0.063 0.072 0.053 0.012 0.000

0.000 0.000 0.000 0.000 0.018 0.000 0.000 0.006 0.000 0.000

0.000 0.006 0.012 0.006 0.000 0.000 0.000 0.000 0.065 0.058

0.065 0.086

>Methylation code ID 21368

0.017 0.000 0.000 0.000 0.000 0.000 0.017 0.000 0.000 0.006

0.000 0.000 0.000 0.006 0.011 0.006 0.000 0.000 0.000 0.000

0.074 0.055 0.057 0.087 0.009 0.009 0.005 0.000 0.000 0.000

0.009 0.000 0.000 0.000 0.000 0.005 0.005 0.000 0.005 0.000

0.014 0.000 0.000 0.000 0.084 0.061 0.043 0.084 0.015 0.000

0.000 0.000 0.000 0.000 0.000 0.000 0.000 0.005 0.005 0.000

0.005 0.000 0.019 0.000 0.010 0.000 0.000 0.000 0.089 0.068

0.064 0.055

>Methylation code ID 21368

0.014 0.000 0.000 0.000 0.000 0.000 0.000 0.000 0.000 0.005

0.005 0.000 0.005 0.000 0.018 0.000 0.014 0.000 0.000 0.000

0.091 0.063 0.060 0.060 0.016 0.000 0.000 0.000 0.000 0.000

0.016 0.000 0.000 0.000 0.000 0.000 0.000 0.005 0.016 0.005

0.000 0.000 0.000 0.000 0.080 0.046 0.063 0.085 0.010 0.005

0.005 0.000 0.000 0.000 0.010 0.000 0.000 0.000 0.000 0.005

0.005 0.000 0.005 0.000 0.015 0.000 0.000 0.000 0.085 0.059

0.048 0.079

>Methylation code ID 21368

0.016 0.000 0.000 0.000 0.000 0.000 0.011 0.000 0.000 0.000

0.000 0.000 0.000 0.005 0.021 0.005 0.000 0.000 0.000 0.000

0.079 0.058 0.054 0.085 0.019 0.005 0.005 0.000 0.000 0.000

0.010 0.000 0.000 0.000 0.000 0.005 0.005 0.000 0.005 0.000

0.010 0.000 0.000 0.000 0.087 0.055 0.049 0.080 0.010 0.000

0.000 0.000 0.000 0.000 0.000 0.000 0.000 0.005 0.005 0.000

0.005 0.000 0.019 0.000 0.014 0.000 0.000 0.000 0.086 0.064

0.064 0.062

>Methylation code ID 21368

0.020 0.000 0.005 0.000 0.000 0.000 0.010 0.000 0.000 0.000

0.000 0.005 0.005 0.000 0.010 0.000 0.005 0.000 0.000 0.000

0.085 0.063 0.048 0.077 0.005 0.000 0.000 0.000 0.000 0.000

0.005 0.000 0.000 0.005 0.005 0.005 0.005 0.000 0.014 0.000

0.014 0.005 0.000 0.000 0.084 0.066 0.052 0.069 0.014 0.000

0.000 0.000 0.000 0.000 0.005 0.005 0.000 0.000 0.000 0.000

0.000 0.000 0.019 0.005 0.005 0.000 0.000 0.000 0.078 0.065

0.048 0.092

>Methylation code ID 21368

0.013 0.000 0.000 0.000 0.000 0.000 0.004 0.004 0.000 0.000

0.000 0.000 0.000 0.000 0.018 0.009 0.004 0.000 0.000 0.000

0.073 0.062 0.053 0.093 0.020 0.000 0.005 0.000 0.000 0.000

0.010 0.000 0.000 0.005 0.000 0.000 0.005 0.000 0.010 0.000

0.005 0.000 0.000 0.000 0.089 0.058 0.045 0.080 0.005 0.000

0.000 0.000 0.000 0.000 0.005 0.000 0.000 0.005 0.000 0.005

0.005 0.000 0.016 0.000 0.016 0.005 0.000 0.000 0.091 0.061

0.054 0.064

>Methylation code ID 21368

0.019 0.000 0.000 0.000 0.000 0.000 0.010 0.000 0.000 0.005

0.000 0.000 0.005 0.000 0.014 0.000 0.005 0.000 0.000 0.000

0.090 0.044 0.054 0.087 0.005 0.000 0.000 0.000 0.000 0.005

0.005 0.000 0.000 0.005 0.000 0.005 0.005 0.000 0.016 0.000

0.011 0.005 0.000 0.000 0.084 0.066 0.054 0.067 0.014 0.000

0.000 0.005 0.000 0.000 0.005 0.005 0.000 0.000 0.000 0.000

0.000 0.000 0.014 0.009 0.005 0.000 0.000 0.000 0.068 0.063

0.048 0.099

>Methylation code ID 21368

0.014 0.000 0.000 0.000 0.000 0.000 0.009 0.000 0.000 0.005

0.000 0.000 0.005 0.000 0.019 0.000 0.005 0.000 0.000 0.000

0.077 0.055 0.050 0.094 0.011 0.000 0.000 0.000 0.000 0.006

0.006 0.000 0.000 0.006 0.000 0.006 0.006 0.000 0.011 0.000

0.011 0.006 0.000 0.000 0.075 0.072 0.048 0.071 0.015 0.000

0.000 0.005 0.000 0.000 0.005 0.005 0.000 0.000 0.000 0.000

0.000 0.000 0.015 0.010 0.005 0.000 0.000 0.000 0.068 0.068

0.051 0.088

>Methylation code ID 21368

0.013 0.000 0.000 0.000 0.000 0.006 0.013 0.000 0.000 0.006

0.000 0.006 0.006 0.000 0.006 0.000 0.013 0.006 0.000 0.000

0.071 0.078 0.060 0.048 0.009 0.000 0.000 0.005 0.000 0.000

0.000 0.005 0.000 0.000 0.000 0.000 0.005 0.000 0.018 0.009

0.009 0.000 0.000 0.000 0.079 0.069 0.055 0.070 0.019 0.000

0.000 0.000 0.000 0.000 0.009 0.000 0.000 0.005 0.000 0.000

0.000 0.000 0.019 0.000 0.000 0.000 0.000 0.000 0.085 0.051

0.036 0.109

>Methylation code ID 21368

0.019 0.000 0.000 0.000 0.000 0.006 0.012 0.000 0.000 0.000

0.000 0.006 0.006 0.000 0.006 0.006 0.006 0.006 0.000 0.000

0.074 0.066 0.052 0.067 0.009 0.000 0.000 0.004 0.000 0.000

0.004 0.004 0.000 0.000 0.000 0.000 0.004 0.000 0.018 0.004

0.009 0.000 0.000 0.000 0.070 0.064 0.066 0.074 0.013 0.000

0.000 0.000 0.000 0.000 0.000 0.000 0.000 0.009 0.000 0.000

0.000 0.000 0.022 0.000 0.004 0.000 0.000 0.000 0.079 0.062

0.034 0.110

>Methylation code ID 21368

0.016 0.000 0.000 0.000 0.000 0.000 0.000 0.000 0.000 0.008

0.000 0.000 0.000 0.000 0.020 0.000 0.004 0.000 0.000 0.000

0.083 0.058 0.032 0.112 0.018 0.000 0.000 0.000 0.000 0.006

0.012 0.000 0.000 0.000 0.000 0.006 0.012 0.000 0.006 0.006

0.000 0.006 0.000 0.000 0.069 0.076 0.051 0.066 0.010 0.000

0.000 0.005 0.000 0.000 0.005 0.005 0.000 0.000 0.000 0.000

0.005 0.000 0.015 0.005 0.010 0.000 0.000 0.000 0.078 0.057

0.064 0.074

>Methylation code ID 21368

0.017 0.000 0.000 0.000 0.000 0.006 0.011 0.000 0.000 0.000

0.000 0.006 0.006 0.000 0.011 0.006 0.000 0.006 0.000 0.000

0.073 0.080 0.044 0.070 0.015 0.000 0.000 0.005 0.000 0.000

0.005 0.005 0.000 0.000 0.000 0.000 0.010 0.000 0.010 0.005

0.010 0.000 0.000 0.000 0.078 0.063 0.059 0.069 0.009 0.000

0.000 0.000 0.000 0.000 0.000 0.000 0.000 0.009 0.000 0.000

0.005 0.000 0.023 0.000 0.005 0.000 0.000 0.000 0.086 0.066

0.025 0.105

>Methylation code ID 21368

0.017 0.000 0.000 0.004 0.000 0.000 0.004 0.000 0.000 0.000

0.000 0.000 0.009 0.000 0.013 0.004 0.004 0.000 0.000 0.000

0.089 0.062 0.046 0.079 0.012 0.000 0.000 0.000 0.000 0.000

0.004 0.000 0.000 0.008 0.000 0.000 0.004 0.000 0.019 0.000

0.004 0.000 0.000 0.000 0.081 0.067 0.038 0.097 0.016 0.000

0.000 0.000 0.000 0.005 0.005 0.000 0.000 0.000 0.000 0.000

0.005 0.000 0.016 0.005 0.005 0.000 0.000 0.000 0.079 0.069

0.038 0.087

>Methylation code ID 21368

0.020 0.000 0.000 0.000 0.000 0.005 0.005 0.000 0.000 0.000

0.000 0.000 0.005 0.000 0.015 0.005 0.005 0.000 0.000 0.000

0.084 0.063 0.049 0.079 0.017 0.000 0.000 0.004 0.000 0.000

0.004 0.000 0.000 0.000 0.000 0.000 0.013 0.000 0.013 0.000

0.004 0.000 0.000 0.000 0.088 0.071 0.040 0.078 0.013 0.000

0.000 0.000 0.000 0.000 0.004 0.000 0.000 0.004 0.000 0.000

0.004 0.000 0.021 0.000 0.004 0.000 0.000 0.000 0.080 0.064

0.042 0.096

>Methylation code ID 21368

0.012 0.000 0.000 0.000 0.000 0.000 0.004 0.000 0.000 0.004

0.000 0.000 0.008 0.000 0.020 0.000 0.004 0.000 0.000 0.000

0.086 0.062 0.045 0.088 0.018 0.000 0.000 0.000 0.000 0.005

0.005 0.000 0.000 0.000 0.000 0.000 0.005 0.000 0.018 0.005

0.005 0.000 0.000 0.000 0.090 0.058 0.054 0.071 0.018 0.000

0.000 0.000 0.000 0.000 0.005 0.000 0.000 0.000 0.000 0.000

0.014 0.000 0.014 0.000 0.005 0.000 0.000 0.000 0.090 0.068

0.040 0.080

>Methylation code ID 21368

0.018 0.000 0.000 0.000 0.000 0.000 0.004 0.000 0.000 0.000

0.000 0.000 0.013 0.000 0.013 0.004 0.004 0.000 0.000 0.000

0.079 0.068 0.051 0.077 0.013 0.000 0.000 0.000 0.000 0.000

0.008 0.000 0.000 0.004 0.000 0.000 0.013 0.000 0.013 0.000

0.004 0.000 0.000 0.000 0.083 0.070 0.043 0.083 0.014 0.000

0.000 0.000 0.000 0.000 0.005 0.000 0.000 0.005 0.000 0.000

0.005 0.000 0.019 0.005 0.005 0.000 0.000 0.000 0.097 0.054

0.069 0.058

>Methylation code ID 21368

0.013 0.000 0.000 0.000 0.000 0.000 0.009 0.000 0.000 0.004

0.000 0.000 0.013 0.000 0.013 0.000 0.004 0.000 0.000 0.000

0.079 0.072 0.044 0.084 0.013 0.000 0.000 0.000 0.000 0.000

0.000 0.000 0.000 0.004 0.000 0.000 0.004 0.000 0.021 0.004

0.008 0.000 0.000 0.000 0.090 0.064 0.057 0.067 0.018 0.000

0.000 0.000 0.000 0.000 0.005 0.000 0.000 0.000 0.000 0.000

0.014 0.000 0.014 0.005 0.000 0.000 0.000 0.000 0.079 0.069

0.059 0.071

>Methylation code ID 21368

0.022 0.000 0.000 0.000 0.000 0.000 0.004 0.000 0.000 0.000

0.000 0.000 0.013 0.000 0.013 0.004 0.000 0.000 0.000 0.000

0.086 0.067 0.055 0.069 0.012 0.000 0.000 0.000 0.000 0.000

0.008 0.000 0.000 0.004 0.000 0.000 0.012 0.000 0.012 0.000

0.004 0.000 0.000 0.000 0.077 0.068 0.055 0.080 0.009 0.000

0.000 0.000 0.000 0.000 0.000 0.000 0.000 0.004 0.000 0.000

0.004 0.000 0.022 0.004 0.009 0.000 0.000 0.000 0.093 0.056

0.061 0.070

>Methylation code ID 21368

0.012 0.000 0.000 0.000 0.000 0.000 0.000 0.000 0.000 0.004

0.000 0.000 0.004 0.000 0.020 0.004 0.008 0.000 0.000 0.000

0.096 0.052 0.056 0.076 0.022 0.000 0.000 0.000 0.000 0.000

0.004 0.000 0.000 0.000 0.000 0.000 0.013 0.000 0.013 0.004

0.000 0.000 0.000 0.000 0.083 0.076 0.046 0.072 0.013 0.000

0.000 0.000 0.000 0.000 0.009 0.000 0.000 0.000 0.000 0.000

0.013 0.000 0.013 0.000 0.004 0.000 0.000 0.000 0.080 0.060

0.058 0.083

>Methylation code ID 21368

0.022 0.000 0.000 0.000 0.000 0.000 0.000 0.000 0.000 0.000

0.000 0.000 0.013 0.000 0.017 0.004 0.000 0.000 0.000 0.000

0.086 0.081 0.039 0.072 0.016 0.000 0.000 0.000 0.000 0.000

0.008 0.000 0.000 0.000 0.000 0.000 0.016 0.000 0.012 0.000

0.000 0.000 0.000 0.000 0.079 0.067 0.054 0.080 0.013 0.000

0.000 0.000 0.000 0.000 0.000 0.000 0.000 0.004 0.000 0.000

0.009 0.000 0.018 0.000 0.009 0.000 0.000 0.000 0.089 0.057

0.063 0.071

>Methylation code ID 21368

0.012 0.000 0.000 0.000 0.000 0.000 0.008 0.000 0.000 0.000

0.000 0.000 0.017 0.000 0.012 0.000 0.004 0.000 0.000 0.000

0.086 0.064 0.053 0.076 0.013 0.000 0.000 0.000 0.000 0.000

0.009 0.000 0.000 0.004 0.000 0.000 0.004 0.000 0.018 0.000

0.009 0.000 0.000 0.000 0.093 0.048 0.059 0.076 0.019 0.000

0.000 0.000 0.000 0.000 0.005 0.000 0.000 0.000 0.000 0.000

0.010 0.000 0.015 0.005 0.005 0.000 0.000 0.000 0.080 0.073

0.051 0.070

>Methylation code ID 21368

0.018 0.000 0.000 0.000 0.000 0.000 0.005 0.000 0.000 0.000

0.005 0.000 0.009 0.000 0.014 0.005 0.005 0.000 0.000 0.000

0.078 0.069 0.058 0.070 0.013 0.000 0.000 0.000 0.000 0.000

0.009 0.000 0.000 0.000 0.000 0.000 0.013 0.000 0.013 0.000

0.004 0.000 0.000 0.000 0.086 0.066 0.045 0.084 0.014 0.000

0.000 0.000 0.000 0.000 0.009 0.000 0.000 0.005 0.000 0.000

0.005 0.000 0.014 0.000 0.009 0.000 0.000 0.000 0.091 0.052

0.049 0.084

>Methylation code ID 21368

0.013 0.000 0.004 0.000 0.000 0.000 0.009 0.000 0.000 0.004

0.000 0.000 0.004 0.000 0.013 0.000 0.009 0.000 0.000 0.000

0.089 0.055 0.050 0.082 0.014 0.000 0.000 0.000 0.000 0.000

0.005 0.000 0.000 0.000 0.005 0.000 0.009 0.000 0.014 0.005

0.009 0.000 0.000 0.000 0.067 0.072 0.055 0.080 0.014 0.000

0.000 0.000 0.000 0.000 0.005 0.000 0.000 0.000 0.000 0.000

0.014 0.000 0.014 0.000 0.005 0.000 0.000 0.000 0.087 0.066

0.042 0.087

>Methylation code ID 21368

0.008 0.000 0.000 0.000 0.000 0.000 0.004 0.000 0.000 0.004

0.000 0.000 0.013 0.000 0.017 0.000 0.004 0.000 0.000 0.000

0.084 0.057 0.061 0.081 0.014 0.000 0.005 0.000 0.000 0.005

0.009 0.000 0.000 0.009 0.000 0.000 0.005 0.000 0.009 0.000

0.005 0.000 0.000 0.000 0.082 0.071 0.035 0.084 0.016 0.000

0.000 0.000 0.000 0.000 0.005 0.000 0.000 0.000 0.005 0.000

0.011 0.000 0.011 0.005 0.011 0.000 0.000 0.000 0.065 0.075

0.058 0.070

>Methylation code ID 21368

0.015 0.000 0.005 0.000 0.000 0.005 0.010 0.000 0.000 0.010

0.000 0.000 0.005 0.000 0.010 0.000 0.005 0.000 0.000 0.000

0.074 0.073 0.042 0.081 0.016 0.000 0.000 0.000 0.000 0.000

0.005 0.000 0.000 0.005 0.005 0.000 0.005 0.000 0.011 0.005

0.011 0.000 0.005 0.000 0.072 0.075 0.044 0.073 0.009 0.000

0.000 0.000 0.000 0.005 0.005 0.000 0.000 0.005 0.000 0.000

0.009 0.000 0.014 0.000 0.005 0.000 0.000 0.000 0.084 0.058

0.061 0.077

>Methylation code ID 21368

0.017 0.000 0.006 0.000 0.000 0.006 0.011 0.000 0.000 0.011

0.000 0.000 0.006 0.000 0.006 0.000 0.000 0.000 0.000 0.006

0.069 0.062 0.052 0.081 0.011 0.005 0.000 0.000 0.000 0.000

0.005 0.000 0.000 0.005 0.005 0.005 0.005 0.000 0.005 0.000

0.016 0.000 0.005 0.000 0.070 0.063 0.064 0.066 0.005 0.000

0.000 0.000 0.000 0.005 0.000 0.000 0.000 0.011 0.000 0.000

0.011 0.000 0.016 0.000 0.005 0.005 0.000 0.000 0.075 0.056

0.068 0.077

>Methylation code ID 21368

0.006 0.017 0.000 0.000 0.000 0.006 0.000 0.000 0.000 0.012

0.000 0.006 0.006 0.000 0.000 0.006 0.006 0.006 0.006 0.000

0.073 0.068 0.064 0.053 0.013 0.000 0.000 0.000 0.013 0.013

0.000 0.000 0.000 0.019 0.006 0.000 0.006 0.000 0.006 0.000

0.000 0.006 0.000 0.000 0.066 0.060 0.063 0.063 0.012 0.000

0.000 0.000 0.006 0.000 0.000 0.000 0.000 0.012 0.000 0.000

0.000 0.000 0.012 0.006 0.000 0.000 0.006 0.006 0.080 0.063

0.086 0.044

>Methylation code ID 21368

0.006 0.000 0.000 0.000 0.006 0.000 0.000 0.000 0.000 0.018

0.000 0.000 0.000 0.000 0.012 0.012 0.000 0.000 0.006 0.006

0.076 0.075 0.095 0.022 0.005 0.015 0.000 0.000 0.000 0.005

0.000 0.000 0.000 0.010 0.000 0.005 0.005 0.000 0.005 0.005

0.005 0.005 0.000 0.000 0.085 0.065 0.057 0.060 0.019 0.000

0.000 0.000 0.013 0.006 0.000 0.000 0.000 0.013 0.006 0.000

0.006 0.000 0.006 0.000 0.000 0.006 0.000 0.000 0.062 0.064

0.063 0.067

>Methylation code ID 21368

0.011 0.011 0.000 0.000 0.000 0.005 0.000 0.000 0.000 0.016

0.000 0.005 0.005 0.000 0.005 0.005 0.000 0.005 0.000 0.000

0.071 0.070 0.076 0.049 0.015 0.005 0.000 0.000 0.010 0.005

0.000 0.000 0.000 0.010 0.005 0.000 0.005 0.000 0.005 0.000

0.000 0.000 0.005 0.000 0.064 0.063 0.058 0.081 0.006 0.000

0.000 0.000 0.006 0.006 0.000 0.000 0.000 0.018 0.000 0.000

0.000 0.000 0.012 0.012 0.000 0.000 0.006 0.000 0.077 0.060

0.085 0.044

>Methylation code ID 21368

0.016 0.005 0.000 0.000 0.011 0.000 0.000 0.000 0.000 0.011

0.000 0.000 0.011 0.000 0.005 0.000 0.000 0.005 0.005 0.000

0.073 0.066 0.049 0.076 0.012 0.000 0.000 0.000 0.012 0.006

0.000 0.000 0.000 0.025 0.000 0.000 0.000 0.000 0.012 0.012

0.000 0.000 0.000 0.000 0.068 0.060 0.080 0.044 0.011 0.005

0.000 0.000 0.000 0.000 0.000 0.000 0.000 0.016 0.000 0.000

0.005 0.000 0.011 0.011 0.000 0.005 0.000 0.000 0.075 0.067

0.080 0.048

>Methylation code ID 21368

0.018 0.006 0.000 0.000 0.006 0.000 0.000 0.000 0.000 0.012

0.000 0.000 0.012 0.000 0.000 0.006 0.000 0.006 0.012 0.000

0.061 0.051 0.050 0.092 0.016 0.000 0.000 0.000 0.008 0.016

0.000 0.000 0.000 0.032 0.000 0.000 0.000 0.000 0.008 0.016

0.000 0.000 0.008 0.000 0.057 0.057 0.047 0.069 0.006 0.000

0.000 0.000 0.000 0.006 0.000 0.000 0.000 0.024 0.000 0.000

0.006 0.000 0.012 0.006 0.000 0.006 0.000 0.000 0.072 0.083

0.070 0.041

>Methylation code ID 21368

0.017 0.000 0.000 0.000 0.012 0.006 0.000 0.000 0.000 0.012

0.000 0.000 0.000 0.000 0.006 0.006 0.000 0.006 0.012 0.000

0.067 0.068 0.054 0.069 0.020 0.000 0.000 0.000 0.000 0.020

0.000 0.000 0.000 0.039 0.000 0.000 0.010 0.000 0.010 0.010

0.000 0.010 0.000 0.000 0.054 0.046 0.053 0.064 0.006 0.006

0.000 0.000 0.000 0.000 0.000 0.000 0.000 0.017 0.000 0.000

0.006 0.000 0.006 0.011 0.000 0.006 0.006 0.000 0.065 0.065

0.067 0.076

>Methylation code ID 21368

0.027 0.000 0.000 0.000 0.000 0.027 0.000 0.000 0.000 0.054

0.000 0.000 0.014 0.000 0.000 0.014 0.000 0.027 0.000 0.000

0.054 0.034 0.037 0.045 0.005 0.005 0.000 0.000 0.005 0.000

0.000 0.000 0.000 0.022 0.000 0.000 0.000 0.000 0.005 0.011

0.000 0.000 0.005 0.000 0.059 0.073 0.074 0.067 0.010 0.005

0.000 0.000 0.010 0.005 0.000 0.000 0.000 0.005 0.000 0.000

0.000 0.000 0.005 0.005 0.000 0.005 0.010 0.000 0.077 0.058

0.068 0.071

>Methylation code ID 21368

0.008 0.000 0.000 0.008 0.016 0.008 0.000 0.008 0.000 0.016

0.000 0.000 0.000 0.008 0.000 0.008 0.000 0.016 0.008 0.000

0.063 0.065 0.045 0.059 0.007 0.000 0.000 0.000 0.007 0.007

0.000 0.000 0.000 0.007 0.000 0.000 0.007 0.000 0.014 0.042

0.000 0.000 0.000 0.000 0.054 0.065 0.062 0.062 0.004 0.000

0.004 0.000 0.004 0.004 0.000 0.000 0.000 0.004 0.000 0.000

0.008 0.000 0.012 0.000 0.000 0.004 0.004 0.000 0.063 0.078

0.082 0.064

>Methylation code ID 21523

0.004 0.012 0.000 0.004 0.000 0.008 0.004 0.000 0.004 0.000

0.000 0.000 0.000 0.004 0.012 0.000 0.000 0.000 0.000 0.000

0.080 0.058 0.072 0.070 0.014 0.000 0.000 0.014 0.000 0.007

0.000 0.000 0.000 0.000 0.000 0.000 0.000 0.007 0.014 0.007

0.007 0.014 0.000 0.000 0.043 0.072 0.063 0.073 0.000 0.000

0.000 0.006 0.000 0.006 0.000 0.000 0.006 0.006 0.000 0.000

0.006 0.000 0.011 0.006 0.000 0.000 0.006 0.000 0.059 0.088

0.069 0.068

>Methylation code ID 21523

0.005 0.010 0.005 0.000 0.000 0.019 0.005 0.000 0.005 0.000

0.000 0.000 0.000 0.000 0.000 0.010 0.000 0.000 0.005 0.000

0.077 0.057 0.061 0.075 0.020 0.000 0.000 0.007 0.000 0.020

0.000 0.000 0.007 0.013 0.007 0.000 0.000 0.000 0.007 0.000

0.007 0.000 0.000 0.000 0.060 0.066 0.060 0.061 0.007 0.000

0.000 0.000 0.000 0.007 0.000 0.000 0.007 0.020 0.000 0.000

0.000 0.000 0.020 0.000 0.000 0.000 0.000 0.000 0.062 0.088

0.071 0.052

>Methylation code ID 21523

0.011 0.000 0.005 0.005 0.000 0.005 0.000 0.000 0.000 0.000

0.000 0.000 0.000 0.000 0.021 0.011 0.000 0.000 0.000 0.000

0.051 0.054 0.069 0.100 0.012 0.000 0.000 0.012 0.000 0.012

0.000 0.000 0.006 0.006 0.006 0.000 0.006 0.006 0.000 0.000

0.000 0.000 0.012 0.000 0.058 0.063 0.055 0.077 0.004 0.009

0.000 0.004 0.000 0.009 0.000 0.000 0.009 0.000 0.000 0.000

0.000 0.004 0.013 0.000 0.000 0.000 0.000 0.000 0.062 0.052

0.078 0.088

**II. List of 1,117 negative samples**

>Non-Methylation code ID 1066

0.000 0.005 0.000 0.000 0.011 0.000 0.000 0.011 0.005 0.000

0.000 0.000 0.000 0.005 0.011 0.011 0.000 0.000 0.000 0.000

0.090 0.046 0.073 0.065 0.005 0.000 0.005 0.005 0.005 0.005

0.005 0.000 0.000 0.000 0.000 0.005 0.000 0.000 0.009 0.009

0.000 0.005 0.000 0.005 0.060 0.064 0.066 0.084 0.000 0.000

0.013 0.007 0.007 0.000 0.000 0.007 0.007 0.013 0.013 0.000

0.000 0.000 0.000 0.007 0.007 0.000 0.000 0.000 0.051 0.065

0.077 0.062

>Non-Methylation code ID 1066

0.006 0.000 0.006 0.006 0.006 0.011 0.000 0.000 0.000 0.022

0.000 0.006 0.000 0.000 0.006 0.000 0.000 0.000 0.000 0.000

0.079 0.053 0.076 0.058 0.000 0.000 0.000 0.000 0.000 0.000

0.000 0.009 0.000 0.009 0.009 0.019 0.000 0.000 0.000 0.019

0.000 0.028 0.009 0.000 0.054 0.064 0.067 0.046 0.000 0.000

0.000 0.000 0.009 0.005 0.000 0.005 0.000 0.005 0.000 0.000

0.005 0.000 0.014 0.000 0.005 0.000 0.005 0.000 0.082 0.067

0.054 0.081

>Non-Methylation code ID 1116

0.007 0.000 0.007 0.007 0.007 0.007 0.007 0.000 0.000 0.007

0.000 0.007 0.000 0.007 0.000 0.000 0.000 0.000 0.007 0.000

0.055 0.074 0.073 0.063 0.000 0.000 0.007 0.020 0.000 0.007

0.000 0.020 0.000 0.014 0.000 0.007 0.000 0.000 0.000 0.014

0.000 0.000 0.000 0.000 0.059 0.057 0.074 0.055 0.006 0.000

0.000 0.000 0.000 0.000 0.000 0.000 0.006 0.013 0.013 0.000

0.000 0.000 0.013 0.019 0.000 0.006 0.000 0.000 0.078 0.081

0.037 0.062

>Non-Methylation code ID 1116

0.000 0.000 0.006 0.018 0.000 0.006 0.000 0.018 0.000 0.006

0.000 0.006 0.000 0.000 0.006 0.012 0.000 0.000 0.000 0.000

0.053 0.063 0.069 0.070 0.006 0.000 0.000 0.000 0.000 0.006

0.000 0.000 0.006 0.012 0.012 0.000 0.000 0.000 0.012 0.018

0.000 0.006 0.000 0.000 0.076 0.079 0.044 0.057 0.008 0.000

0.008 0.008 0.000 0.008 0.008 0.000 0.000 0.008 0.000 0.008

0.000 0.008 0.000 0.000 0.000 0.000 0.008 0.000 0.061 0.064

0.077 0.063

>Non-Methylation code ID 1116

0.006 0.000 0.000 0.000 0.013 0.000 0.006 0.013 0.013 0.006

0.006 0.000 0.000 0.000 0.000 0.013 0.000 0.000 0.000 0.000

0.068 0.061 0.058 0.069 0.000 0.000 0.000 0.000 0.011 0.000

0.000 0.011 0.000 0.032 0.000 0.011 0.000 0.000 0.000 0.053

0.000 0.011 0.000 0.000 0.057 0.051 0.042 0.056 0.007 0.007

0.000 0.007 0.007 0.000 0.007 0.007 0.000 0.013 0.000 0.000

0.007 0.000 0.000 0.000 0.000 0.000 0.000 0.013 0.072 0.071

0.050 0.067

>Non-Methylation code ID 1116

0.006 0.006 0.000 0.006 0.006 0.000 0.006 0.006 0.000 0.013

0.000 0.000 0.006 0.000 0.000 0.000 0.000 0.006 0.000 0.013

0.068 0.071 0.055 0.062 0.007 0.000 0.000 0.000 0.014 0.000

0.007 0.014 0.014 0.007 0.007 0.000 0.000 0.000 0.000 0.007

0.000 0.000 0.000 0.000 0.066 0.061 0.058 0.074 0.000 0.000

0.000 0.000 0.012 0.000 0.000 0.012 0.000 0.035 0.000 0.012

0.000 0.000 0.000 0.058 0.000 0.000 0.000 0.000 0.059 0.052

0.048 0.046

>Non-Methylation code ID 1116

0.006 0.000 0.000 0.000 0.000 0.000 0.000 0.000 0.006 0.019

0.006 0.000 0.000 0.000 0.012 0.019 0.000 0.006 0.000 0.000

0.077 0.072 0.053 0.058 0.007 0.000 0.013 0.000 0.007 0.007

0.007 0.000 0.000 0.007 0.000 0.007 0.000 0.007 0.000 0.000

0.000 0.000 0.007 0.000 0.058 0.080 0.067 0.063 0.000 0.000

0.000 0.025 0.000 0.008 0.000 0.034 0.000 0.017 0.000 0.000

0.000 0.000 0.000 0.017 0.000 0.000 0.000 0.000 0.058 0.047

0.065 0.063

>Non-Methylation code ID 1116

0.000 0.000 0.007 0.021 0.000 0.007 0.000 0.014 0.000 0.021

0.000 0.007 0.000 0.007 0.000 0.007 0.000 0.000 0.000 0.000

0.070 0.043 0.070 0.060 0.000 0.000 0.000 0.000 0.000 0.000

0.000 0.007 0.007 0.020 0.000 0.007 0.000 0.007 0.007 0.007

0.007 0.007 0.007 0.000 0.070 0.061 0.059 0.064 0.005 0.000

0.010 0.000 0.005 0.000 0.000 0.005 0.000 0.000 0.000 0.000

0.000 0.000 0.014 0.005 0.000 0.000 0.005 0.000 0.098 0.055

0.078 0.055

>Non-Methylation code ID 1116

0.008 0.000 0.000 0.000 0.000 0.015 0.000 0.008 0.000 0.023

0.000 0.000 0.008 0.000 0.008 0.008 0.008 0.008 0.000 0.008

0.066 0.064 0.051 0.053 0.013 0.000 0.000 0.000 0.007 0.007

0.007 0.000 0.000 0.007 0.000 0.000 0.013 0.000 0.000 0.013

0.007 0.000 0.007 0.007 0.070 0.083 0.040 0.053 0.000 0.000

0.000 0.000 0.000 0.005 0.005 0.005 0.000 0.005 0.000 0.000

0.015 0.000 0.010 0.005 0.005 0.000 0.000 0.005 0.073 0.069

0.053 0.077

>Non-Methylation code ID 1116

0.000 0.000 0.000 0.000 0.000 0.015 0.000 0.008 0.000 0.023

0.000 0.000 0.008 0.000 0.008 0.008 0.008 0.008 0.000 0.008

0.070 0.077 0.050 0.045 0.014 0.000 0.000 0.000 0.000 0.007

0.000 0.000 0.000 0.007 0.000 0.000 0.014 0.000 0.000 0.021

0.007 0.000 0.007 0.014 0.067 0.069 0.043 0.064 0.005 0.000

0.000 0.000 0.000 0.005 0.005 0.005 0.000 0.005 0.000 0.000

0.016 0.000 0.010 0.000 0.000 0.005 0.000 0.005 0.075 0.069

0.046 0.082

>Non-Methylation code ID 1116

0.000 0.005 0.000 0.000 0.000 0.016 0.000 0.000 0.010 0.016

0.000 0.005 0.005 0.000 0.000 0.000 0.005 0.000 0.000 0.005

0.062 0.076 0.069 0.058 0.009 0.005 0.005 0.000 0.000 0.005

0.000 0.005 0.005 0.000 0.000 0.000 0.000 0.005 0.009 0.005

0.005 0.000 0.000 0.005 0.083 0.072 0.083 0.036 0.000 0.000

0.007 0.000 0.000 0.013 0.000 0.007 0.007 0.000 0.000 0.000

0.013 0.007 0.007 0.000 0.007 0.000 0.000 0.000 0.072 0.057

0.065 0.073

>Non-Methylation code ID 1116

0.005 0.000 0.000 0.000 0.005 0.005 0.010 0.000 0.005 0.005

0.000 0.000 0.010 0.000 0.005 0.005 0.005 0.000 0.000 0.005

0.066 0.073 0.059 0.069 0.000 0.000 0.006 0.000 0.000 0.000

0.006 0.006 0.000 0.006 0.000 0.000 0.006 0.000 0.012 0.012

0.012 0.006 0.000 0.006 0.079 0.066 0.045 0.067 0.009 0.000

0.000 0.000 0.000 0.009 0.000 0.009 0.000 0.017 0.000 0.000

0.009 0.000 0.009 0.009 0.017 0.000 0.000 0.009 0.055 0.065

0.047 0.073

>Non-Methylation code ID 1116

0.009 0.005 0.005 0.000 0.000 0.005 0.000 0.005 0.005 0.000

0.000 0.000 0.000 0.005 0.009 0.005 0.005 0.000 0.000 0.005

0.083 0.072 0.083 0.036 0.000 0.000 0.006 0.000 0.000 0.012

0.000 0.006 0.006 0.000 0.000 0.000 0.012 0.006 0.012 0.000

0.006 0.000 0.000 0.000 0.062 0.063 0.075 0.070 0.000 0.005

0.000 0.000 0.000 0.016 0.000 0.000 0.011 0.011 0.000 0.005

0.005 0.000 0.000 0.000 0.005 0.000 0.000 0.005 0.060 0.077

0.073 0.058

>Non-Methylation code ID 1116

0.000 0.000 0.000 0.000 0.000 0.022 0.000 0.011 0.005 0.011

0.000 0.005 0.000 0.000 0.005 0.005 0.000 0.000 0.005 0.000

0.066 0.077 0.040 0.079 0.019 0.000 0.000 0.000 0.012 0.019

0.000 0.000 0.000 0.006 0.000 0.000 0.000 0.000 0.006 0.000

0.006 0.000 0.000 0.006 0.061 0.068 0.070 0.059 0.007 0.000

0.000 0.007 0.000 0.014 0.007 0.000 0.000 0.000 0.007 0.000

0.000 0.014 0.007 0.014 0.000 0.000 0.007 0.000 0.068 0.060

0.064 0.059

>Non-Methylation code ID 1116

0.018 0.000 0.000 0.000 0.006 0.000 0.000 0.000 0.000 0.012

0.000 0.000 0.006 0.000 0.006 0.006 0.006 0.012 0.006 0.000

0.065 0.062 0.059 0.070 0.000 0.000 0.000 0.000 0.000 0.015

0.000 0.000 0.000 0.007 0.000 0.000 0.022 0.007 0.007 0.029

0.000 0.000 0.000 0.000 0.059 0.050 0.065 0.071 0.000 0.000

0.000 0.000 0.000 0.026 0.000 0.000 0.000 0.026 0.000 0.000

0.017 0.009 0.000 0.017 0.000 0.000 0.000 0.009 0.058 0.059

0.055 0.058

>Non-Methylation code ID 1116

0.014 0.000 0.000 0.000 0.000 0.007 0.000 0.000 0.000 0.007

0.000 0.000 0.014 0.000 0.000 0.021 0.007 0.000 0.007 0.014

0.067 0.069 0.043 0.064 0.005 0.000 0.000 0.000 0.000 0.005

0.005 0.005 0.000 0.005 0.000 0.000 0.015 0.000 0.010 0.000

0.005 0.005 0.000 0.005 0.074 0.067 0.045 0.082 0.000 0.000

0.000 0.000 0.000 0.017 0.000 0.008 0.000 0.025 0.000 0.000

0.000 0.000 0.008 0.008 0.008 0.008 0.000 0.008 0.071 0.079

0.049 0.043

>Non-Methylation code ID 1116

0.015 0.000 0.007 0.007 0.000 0.022 0.000 0.000 0.000 0.000

0.000 0.007 0.015 0.015 0.000 0.000 0.007 0.000 0.000 0.000

0.064 0.074 0.058 0.041 0.005 0.000 0.000 0.005 0.000 0.005

0.000 0.000 0.000 0.005 0.000 0.000 0.005 0.005 0.018 0.005

0.009 0.000 0.000 0.000 0.079 0.063 0.069 0.064 0.006 0.000

0.006 0.006 0.000 0.011 0.000 0.000 0.000 0.000 0.000 0.000

0.017 0.000 0.017 0.000 0.000 0.000 0.000 0.000 0.072 0.101

0.053 0.044

>Non-Methylation code ID 1116

0.007 0.000 0.000 0.000 0.000 0.021 0.000 0.000 0.000 0.007

0.000 0.000 0.014 0.007 0.000 0.021 0.000 0.000 0.007 0.007

0.064 0.062 0.054 0.062 0.019 0.000 0.000 0.000 0.000 0.006

0.000 0.000 0.000 0.012 0.000 0.000 0.012 0.000 0.006 0.000

0.006 0.012 0.006 0.000 0.068 0.062 0.063 0.058 0.000 0.000

0.000 0.000 0.000 0.015 0.000 0.000 0.000 0.015 0.000 0.000

0.015 0.007 0.007 0.015 0.000 0.007 0.000 0.000 0.066 0.066

0.060 0.061

>Non-Methylation code ID 1116

0.017 0.000 0.000 0.000 0.000 0.006 0.000 0.000 0.000 0.006

0.000 0.000 0.022 0.000 0.011 0.011 0.000 0.000 0.000 0.000

0.080 0.070 0.043 0.069 0.015 0.000 0.000 0.000 0.000 0.007

0.000 0.000 0.000 0.022 0.000 0.000 0.036 0.000 0.007 0.007

0.000 0.000 0.000 0.000 0.072 0.052 0.052 0.062 0.005 0.000

0.000 0.000 0.000 0.005 0.000 0.000 0.000 0.005 0.000 0.000

0.019 0.000 0.014 0.010 0.000 0.000 0.000 0.000 0.066 0.075

0.095 0.040

>Non-Methylation code ID 1116

0.012 0.000 0.000 0.004 0.000 0.000 0.004 0.000 0.000 0.000

0.000 0.000 0.008 0.000 0.016 0.008 0.000 0.000 0.000 0.000

0.062 0.078 0.076 0.065 0.012 0.000 0.000 0.006 0.000 0.012

0.000 0.000 0.000 0.006 0.000 0.000 0.018 0.006 0.006 0.006

0.006 0.000 0.000 0.000 0.065 0.074 0.070 0.044 0.011 0.000

0.000 0.006 0.000 0.006 0.000 0.000 0.000 0.000 0.000 0.000

0.022 0.000 0.011 0.011 0.000 0.000 0.000 0.000 0.083 0.071

0.039 0.073

>Non-Methylation code ID 1116

0.008 0.000 0.000 0.000 0.004 0.004 0.000 0.000 0.000 0.004

0.000 0.000 0.016 0.000 0.012 0.004 0.000 0.000 0.000 0.000

0.083 0.071 0.058 0.069 0.005 0.005 0.005 0.000 0.000 0.010

0.000 0.000 0.000 0.000 0.000 0.000 0.025 0.000 0.010 0.005

0.000 0.000 0.000 0.000 0.069 0.056 0.088 0.056 0.006 0.000

0.000 0.006 0.000 0.012 0.000 0.006 0.000 0.006 0.000 0.000

0.030 0.000 0.006 0.000 0.000 0.000 0.000 0.000 0.064 0.073

0.054 0.071

>Non-Methylation code ID 1116

0.007 0.000 0.000 0.007 0.007 0.000 0.000 0.000 0.000 0.007

0.000 0.007 0.021 0.007 0.000 0.000 0.014 0.007 0.000 0.000

0.062 0.065 0.055 0.067 0.000 0.005 0.000 0.000 0.005 0.000

0.000 0.000 0.000 0.000 0.000 0.000 0.015 0.005 0.015 0.015

0.005 0.000 0.000 0.000 0.079 0.058 0.064 0.068 0.012 0.000

0.000 0.000 0.000 0.006 0.012 0.000 0.000 0.000 0.000 0.000

0.006 0.006 0.012 0.012 0.000 0.006 0.000 0.000 0.066 0.066

0.054 0.077

>Non-Methylation code ID 1116

0.016 0.000 0.000 0.000 0.000 0.008 0.000 0.000 0.008 0.016

0.000 0.000 0.016 0.008 0.000 0.000 0.008 0.016 0.000 0.008

0.057 0.056 0.055 0.064 0.000 0.000 0.000 0.000 0.000 0.007

0.000 0.007 0.000 0.013 0.000 0.000 0.007 0.013 0.013 0.020

0.000 0.000 0.000 0.000 0.067 0.065 0.061 0.061 0.006 0.000

0.000 0.006 0.000 0.006 0.000 0.000 0.000 0.000 0.000 0.000

0.011 0.000 0.011 0.022 0.000 0.000 0.000 0.006 0.073 0.066

0.067 0.061

>Non-Methylation code ID 1116

0.012 0.000 0.000 0.006 0.000 0.006 0.000 0.000 0.000 0.000

0.000 0.000 0.025 0.006 0.006 0.012 0.006 0.000 0.000 0.000

0.066 0.058 0.071 0.058 0.004 0.000 0.000 0.000 0.000 0.008

0.000 0.000 0.000 0.004 0.000 0.000 0.017 0.004 0.017 0.000

0.000 0.000 0.000 0.000 0.089 0.072 0.054 0.064 0.019 0.000

0.000 0.006 0.000 0.012 0.006 0.000 0.000 0.006 0.000 0.000

0.012 0.000 0.006 0.006 0.000 0.000 0.000 0.000 0.066 0.073

0.057 0.063

>Non-Methylation code ID 1116

0.021 0.000 0.000 0.007 0.000 0.014 0.000 0.000 0.000 0.000

0.000 0.000 0.021 0.000 0.007 0.014 0.000 0.007 0.000 0.000

0.078 0.045 0.059 0.060 0.009 0.000 0.000 0.004 0.000 0.004

0.000 0.000 0.000 0.000 0.000 0.000 0.013 0.000 0.017 0.009

0.000 0.000 0.000 0.000 0.060 0.067 0.083 0.068 0.006 0.000

0.000 0.000 0.000 0.025 0.000 0.000 0.000 0.006 0.000 0.000

0.019 0.006 0.006 0.006 0.000 0.000 0.000 0.000 0.068 0.059

0.069 0.062

>Non-Methylation code ID 1116

0.000 0.005 0.005 0.000 0.000 0.011 0.005 0.000 0.000 0.000

0.000 0.000 0.011 0.005 0.005 0.011 0.005 0.005 0.000 0.000

0.066 0.054 0.082 0.062 0.010 0.000 0.000 0.005 0.000 0.005

0.000 0.005 0.000 0.005 0.000 0.000 0.010 0.000 0.014 0.000

0.005 0.000 0.000 0.000 0.053 0.084 0.071 0.069 0.008 0.000

0.000 0.000 0.004 0.004 0.004 0.000 0.000 0.004 0.000 0.000

0.008 0.000 0.013 0.004 0.000 0.000 0.000 0.000 0.087 0.068

0.046 0.082

>Non-Methylation code ID 1116

0.000 0.000 0.005 0.000 0.000 0.005 0.000 0.000 0.000 0.011

0.000 0.000 0.016 0.000 0.011 0.016 0.000 0.005 0.000 0.000

0.061 0.077 0.067 0.058 0.010 0.000 0.000 0.000 0.000 0.005

0.000 0.000 0.000 0.005 0.005 0.000 0.010 0.000 0.015 0.015

0.000 0.000 0.000 0.000 0.082 0.067 0.043 0.074 0.016 0.000

0.000 0.000 0.000 0.000 0.000 0.000 0.000 0.011 0.000 0.000

0.016 0.000 0.011 0.005 0.000 0.000 0.005 0.000 0.057 0.077

0.074 0.060

>Non-Methylation code ID 1116

0.000 0.000 0.000 0.000 0.005 0.005 0.000 0.005 0.005 0.000

0.000 0.000 0.010 0.010 0.010 0.000 0.005 0.000 0.000 0.005

0.051 0.068 0.081 0.075 0.005 0.000 0.000 0.005 0.000 0.005

0.000 0.005 0.000 0.005 0.000 0.000 0.005 0.000 0.014 0.014

0.000 0.000 0.000 0.005 0.055 0.079 0.072 0.065 0.007 0.000

0.007 0.007 0.000 0.000 0.007 0.000 0.007 0.007 0.000 0.000

0.007 0.000 0.000 0.014 0.007 0.014 0.000 0.000 0.062 0.074

0.051 0.062

>Non-Methylation code ID 1116

0.012 0.000 0.006 0.006 0.000 0.006 0.000 0.000 0.006 0.006

0.000 0.000 0.006 0.000 0.006 0.012 0.000 0.012 0.000 0.000

0.077 0.070 0.053 0.055 0.000 0.000 0.000 0.000 0.006 0.006

0.000 0.000 0.013 0.006 0.000 0.000 0.013 0.013 0.000 0.000

0.006 0.006 0.000 0.006 0.061 0.066 0.052 0.076 0.000 0.000

0.000 0.000 0.000 0.006 0.000 0.006 0.000 0.006 0.000 0.000

0.000 0.006 0.017 0.028 0.000 0.000 0.000 0.000 0.053 0.081

0.086 0.046

>Non-Methylation code ID 1116

0.017 0.000 0.000 0.000 0.000 0.017 0.000 0.000 0.000 0.000

0.000 0.000 0.058 0.000 0.008 0.008 0.000 0.000 0.000 0.000

0.061 0.048 0.060 0.056 0.000 0.000 0.005 0.000 0.000 0.005

0.005 0.000 0.000 0.005 0.000 0.000 0.019 0.005 0.014 0.000

0.000 0.000 0.000 0.000 0.074 0.065 0.089 0.049 0.011 0.000

0.000 0.006 0.006 0.006 0.000 0.000 0.000 0.000 0.000 0.000

0.017 0.000 0.006 0.000 0.011 0.000 0.006 0.000 0.071 0.060

0.067 0.067

>Non-Methylation code ID 1116

0.017 0.000 0.000 0.006 0.000 0.006 0.000 0.000 0.000 0.000

0.000 0.000 0.017 0.000 0.011 0.011 0.000 0.006 0.000 0.000

0.076 0.067 0.051 0.065 0.012 0.000 0.000 0.004 0.000 0.000

0.000 0.000 0.000 0.000 0.000 0.000 0.012 0.000 0.016 0.008

0.000 0.000 0.000 0.000 0.069 0.066 0.079 0.066 0.012 0.000

0.000 0.000 0.000 0.018 0.000 0.000 0.000 0.000 0.000 0.000

0.018 0.006 0.012 0.006 0.000 0.000 0.000 0.000 0.073 0.074

0.063 0.051

>Non-Methylation code ID 1116

0.004 0.000 0.000 0.000 0.004 0.004 0.000 0.000 0.000 0.000

0.000 0.000 0.016 0.000 0.016 0.008 0.000 0.000 0.000 0.000

0.071 0.076 0.087 0.047 0.016 0.000 0.005 0.000 0.000 0.005

0.000 0.000 0.000 0.005 0.000 0.000 0.022 0.000 0.011 0.005

0.000 0.000 0.000 0.000 0.079 0.063 0.049 0.072 0.014 0.000

0.000 0.000 0.000 0.007 0.000 0.007 0.000 0.014 0.000 0.000

0.035 0.000 0.007 0.000 0.000 0.000 0.000 0.000 0.071 0.069

0.051 0.058

>Non-Methylation code ID 1116

0.004 0.000 0.000 0.000 0.000 0.009 0.000 0.000 0.000 0.000

0.000 0.000 0.017 0.004 0.017 0.004 0.000 0.000 0.000 0.000

0.095 0.071 0.055 0.056 0.024 0.000 0.000 0.006 0.000 0.012

0.006 0.000 0.000 0.006 0.000 0.000 0.012 0.000 0.006 0.006

0.000 0.000 0.000 0.000 0.065 0.070 0.055 0.064 0.014 0.000

0.000 0.007 0.000 0.007 0.000 0.000 0.000 0.000 0.000 0.000

0.020 0.007 0.007 0.014 0.007 0.000 0.000 0.000 0.067 0.056

0.072 0.056

>Non-Methylation code ID 1116

0.000 0.005 0.000 0.000 0.000 0.000 0.000 0.000 0.000 0.005

0.000 0.005 0.005 0.005 0.018 0.009 0.009 0.000 0.000 0.000

0.071 0.068 0.057 0.077 0.029 0.000 0.000 0.007 0.000 0.007

0.000 0.000 0.000 0.007 0.000 0.000 0.007 0.007 0.007 0.014

0.007 0.000 0.000 0.000 0.057 0.069 0.052 0.062 0.009 0.000

0.000 0.000 0.000 0.000 0.000 0.000 0.000 0.000 0.000 0.017

0.017 0.034 0.000 0.009 0.000 0.009 0.000 0.000 0.046 0.059

0.067 0.067

>Non-Methylation code ID 1116

0.000 0.000 0.005 0.005 0.000 0.000 0.005 0.000 0.000 0.009

0.000 0.000 0.018 0.005 0.014 0.000 0.000 0.000 0.000 0.000

0.071 0.058 0.089 0.057 0.011 0.000 0.000 0.005 0.005 0.000

0.000 0.000 0.000 0.000 0.000 0.000 0.016 0.000 0.011 0.005

0.011 0.000 0.000 0.000 0.069 0.068 0.074 0.056 0.014 0.000

0.000 0.000 0.000 0.000 0.000 0.000 0.000 0.000 0.000 0.000

0.049 0.000 0.014 0.007 0.000 0.000 0.000 0.000 0.061 0.076

0.072 0.040

>Non-Methylation code ID 1116

0.011 0.000 0.000 0.000 0.000 0.017 0.000 0.000 0.000 0.006

0.000 0.000 0.017 0.006 0.011 0.006 0.000 0.000 0.000 0.000

0.071 0.070 0.074 0.047 0.017 0.000 0.000 0.006 0.000 0.006

0.000 0.000 0.000 0.000 0.000 0.000 0.017 0.000 0.011 0.011

0.000 0.006 0.000 0.000 0.081 0.065 0.045 0.071 0.013 0.000

0.000 0.004 0.000 0.000 0.000 0.000 0.000 0.000 0.000 0.000

0.009 0.000 0.018 0.009 0.000 0.000 0.000 0.000 0.065 0.069

0.076 0.070

>Non-Methylation code ID 1116

0.010 0.000 0.000 0.000 0.000 0.005 0.000 0.005 0.000 0.000

0.000 0.000 0.026 0.000 0.010 0.005 0.005 0.000 0.000 0.000

0.046 0.069 0.074 0.078 0.005 0.000 0.000 0.000 0.000 0.009

0.005 0.000 0.000 0.009 0.000 0.000 0.014 0.005 0.009 0.005

0.000 0.000 0.000 0.000 0.062 0.080 0.065 0.066 0.006 0.000

0.000 0.006 0.000 0.006 0.012 0.000 0.000 0.000 0.000 0.000

0.012 0.000 0.006 0.012 0.006 0.006 0.000 0.000 0.068 0.076

0.049 0.068

>Non-Methylation code ID 1116

0.014 0.000 0.000 0.000 0.000 0.014 0.000 0.000 0.000 0.007

0.007 0.000 0.007 0.000 0.007 0.028 0.000 0.007 0.000 0.000

0.065 0.058 0.051 0.067 0.004 0.000 0.000 0.004 0.000 0.004

0.000 0.000 0.000 0.004 0.000 0.000 0.012 0.000 0.012 0.008

0.000 0.000 0.004 0.000 0.055 0.082 0.080 0.067 0.000 0.000

0.007 0.000 0.000 0.021 0.000 0.000 0.000 0.021 0.000 0.000

0.007 0.000 0.007 0.014 0.000 0.007 0.000 0.000 0.077 0.050

0.060 0.063

>Non-Methylation code ID 1116

0.007 0.000 0.000 0.007 0.007 0.007 0.000 0.000 0.000 0.021

0.000 0.000 0.035 0.000 0.000 0.007 0.000 0.000 0.000 0.000

0.075 0.050 0.070 0.048 0.000 0.004 0.000 0.000 0.000 0.000

0.000 0.000 0.000 0.004 0.000 0.000 0.013 0.000 0.018 0.009

0.000 0.004 0.000 0.000 0.051 0.069 0.076 0.085 0.022 0.000

0.000 0.000 0.005 0.005 0.000 0.000 0.000 0.005 0.000 0.000

0.011 0.000 0.011 0.005 0.000 0.000 0.000 0.000 0.069 0.069

0.073 0.056

>Non-Methylation code ID 1116

0.005 0.000 0.000 0.000 0.000 0.022 0.005 0.000 0.000 0.011

0.000 0.000 0.016 0.000 0.005 0.005 0.000 0.000 0.000 0.000

0.057 0.067 0.068 0.071 0.018 0.000 0.000 0.006 0.000 0.000

0.012 0.000 0.000 0.006 0.000 0.000 0.018 0.000 0.000 0.000

0.006 0.006 0.006 0.000 0.063 0.070 0.061 0.062 0.000 0.000

0.000 0.000 0.000 0.004 0.000 0.004 0.000 0.000 0.000 0.000

0.018 0.000 0.013 0.004 0.004 0.000 0.004 0.000 0.065 0.081

0.068 0.067

>Non-Methylation code ID 1116

0.004 0.004 0.000 0.000 0.000 0.000 0.004 0.000 0.000 0.004

0.000 0.000 0.013 0.000 0.013 0.008 0.000 0.004 0.000 0.000

0.058 0.073 0.068 0.079 0.023 0.000 0.000 0.000 0.006 0.000

0.006 0.000 0.000 0.006 0.000 0.000 0.012 0.006 0.006 0.006

0.006 0.000 0.000 0.000 0.065 0.067 0.052 0.073 0.000 0.000

0.000 0.000 0.007 0.007 0.000 0.000 0.000 0.013 0.000 0.000

0.033 0.000 0.007 0.007 0.007 0.000 0.000 0.000 0.066 0.060

0.075 0.052

>Non-Methylation code ID 1116

0.011 0.000 0.000 0.000 0.005 0.000 0.000 0.000 0.000 0.011

0.000 0.000 0.016 0.000 0.011 0.005 0.005 0.000 0.000 0.000

0.058 0.071 0.085 0.054 0.004 0.004 0.000 0.000 0.000 0.004

0.004 0.000 0.000 0.009 0.000 0.000 0.009 0.000 0.013 0.009

0.000 0.000 0.000 0.000 0.080 0.066 0.056 0.074 0.016 0.008

0.000 0.000 0.000 0.008 0.008 0.000 0.000 0.008 0.000 0.000

0.016 0.008 0.000 0.008 0.008 0.008 0.000 0.000 0.050 0.056

0.054 0.080

>Non-Methylation code ID 1116

0.022 0.000 0.000 0.007 0.000 0.007 0.007 0.000 0.000 0.007

0.000 0.000 0.015 0.000 0.007 0.007 0.007 0.007 0.000 0.000

0.063 0.058 0.054 0.063 0.013 0.000 0.000 0.007 0.000 0.000

0.000 0.000 0.000 0.000 0.000 0.000 0.020 0.013 0.007 0.013

0.007 0.000 0.000 0.000 0.066 0.055 0.073 0.059 0.005 0.000

0.000 0.000 0.000 0.005 0.000 0.000 0.000 0.000 0.000 0.000

0.021 0.005 0.016 0.010 0.000 0.000 0.000 0.000 0.087 0.090

0.046 0.048

>Non-Methylation code ID 1116

0.020 0.000 0.000 0.000 0.005 0.005 0.005 0.000 0.000 0.005

0.000 0.000 0.010 0.000 0.010 0.005 0.000 0.000 0.000 0.000

0.066 0.070 0.072 0.060 0.007 0.000 0.000 0.007 0.007 0.007

0.000 0.000 0.000 0.014 0.000 0.000 0.035 0.000 0.000 0.007

0.007 0.000 0.000 0.000 0.070 0.056 0.067 0.049 0.000 0.004

0.000 0.000 0.000 0.000 0.000 0.000 0.000 0.004 0.000 0.000

0.013 0.000 0.018 0.009 0.000 0.004 0.000 0.000 0.051 0.069

0.076 0.085

>Non-Methylation code ID 1116

0.011 0.000 0.000 0.000 0.000 0.005 0.000 0.005 0.000 0.000

0.000 0.000 0.027 0.000 0.011 0.005 0.005 0.000 0.000 0.000

0.050 0.073 0.077 0.063 0.000 0.000 0.000 0.000 0.000 0.010

0.005 0.000 0.000 0.010 0.000 0.000 0.015 0.005 0.010 0.005

0.000 0.000 0.000 0.000 0.060 0.072 0.068 0.074 0.006 0.000

0.000 0.006 0.000 0.006 0.006 0.000 0.000 0.000 0.000 0.000

0.012 0.000 0.012 0.012 0.006 0.006 0.000 0.000 0.073 0.077

0.039 0.076

>Non-Methylation code ID 1116

0.000 0.000 0.000 0.000 0.006 0.006 0.000 0.000 0.000 0.011

0.000 0.000 0.028 0.000 0.011 0.006 0.006 0.000 0.000 0.000

0.057 0.061 0.080 0.062 0.005 0.005 0.000 0.000 0.000 0.005

0.005 0.000 0.000 0.005 0.000 0.000 0.014 0.000 0.009 0.009

0.000 0.005 0.000 0.000 0.063 0.074 0.060 0.077 0.019 0.000

0.000 0.000 0.006 0.000 0.006 0.000 0.000 0.006 0.000 0.000

0.013 0.006 0.006 0.006 0.006 0.000 0.000 0.000 0.072 0.056

0.051 0.078

>Non-Methylation code ID 1116

0.008 0.000 0.000 0.000 0.004 0.004 0.000 0.000 0.000 0.000

0.000 0.000 0.016 0.000 0.016 0.004 0.000 0.000 0.000 0.000

0.083 0.077 0.067 0.055 0.011 0.000 0.005 0.000 0.000 0.011

0.000 0.000 0.000 0.000 0.000 0.000 0.027 0.000 0.011 0.005

0.000 0.000 0.000 0.000 0.079 0.061 0.073 0.051 0.006 0.000

0.000 0.006 0.000 0.012 0.000 0.006 0.000 0.006 0.000 0.000

0.030 0.000 0.006 0.000 0.000 0.000 0.000 0.000 0.068 0.071

0.048 0.074

>Non-Methylation code ID 1116

0.006 0.000 0.006 0.000 0.000 0.023 0.000 0.000 0.000 0.006

0.000 0.000 0.017 0.006 0.006 0.006 0.000 0.000 0.000 0.000

0.069 0.054 0.072 0.064 0.014 0.000 0.000 0.007 0.000 0.014

0.000 0.000 0.000 0.000 0.007 0.000 0.021 0.000 0.007 0.014

0.000 0.007 0.000 0.000 0.076 0.057 0.058 0.053 0.010 0.000

0.000 0.005 0.000 0.005 0.000 0.000 0.000 0.000 0.000 0.000

0.014 0.000 0.010 0.010 0.000 0.000 0.005 0.000 0.061 0.058

0.079 0.078

>Non-Methylation code ID 1116

0.005 0.000 0.000 0.005 0.000 0.005 0.011 0.000 0.000 0.000

0.000 0.000 0.011 0.000 0.005 0.011 0.005 0.005 0.005 0.000

0.069 0.074 0.049 0.072 0.010 0.000 0.000 0.000 0.000 0.005

0.000 0.005 0.000 0.000 0.000 0.000 0.026 0.000 0.010 0.005

0.005 0.000 0.000 0.000 0.046 0.069 0.074 0.078 0.005 0.000

0.000 0.000 0.000 0.010 0.005 0.000 0.000 0.010 0.000 0.000

0.015 0.000 0.010 0.005 0.000 0.000 0.000 0.000 0.063 0.082

0.064 0.065

>Non-Methylation code ID 1116

0.012 0.000 0.000 0.006 0.000 0.006 0.000 0.006 0.000 0.012

0.000 0.006 0.006 0.000 0.012 0.006 0.006 0.000 0.000 0.000

0.080 0.043 0.071 0.063 0.006 0.000 0.000 0.000 0.006 0.012

0.006 0.000 0.000 0.006 0.000 0.000 0.006 0.012 0.006 0.006

0.006 0.000 0.000 0.000 0.063 0.057 0.061 0.079 0.000 0.000

0.007 0.000 0.000 0.007 0.007 0.000 0.000 0.007 0.000 0.000

0.007 0.007 0.007 0.027 0.007 0.000 0.000 0.000 0.069 0.060

0.060 0.061

>Non-Methylation code ID 1116

0.005 0.000 0.000 0.000 0.005 0.015 0.005 0.000 0.010 0.000

0.000 0.000 0.000 0.000 0.010 0.005 0.005 0.000 0.000 0.000

0.053 0.065 0.074 0.081 0.012 0.000 0.006 0.000 0.000 0.012

0.000 0.000 0.006 0.000 0.000 0.000 0.012 0.006 0.006 0.012

0.006 0.000 0.000 0.000 0.083 0.060 0.056 0.057 0.005 0.000

0.005 0.005 0.000 0.005 0.000 0.005 0.000 0.009 0.000 0.000

0.000 0.009 0.014 0.000 0.000 0.000 0.000 0.000 0.075 0.055

0.081 0.067

>Non-Methylation code ID 1116

0.005 0.000 0.000 0.005 0.000 0.009 0.005 0.000 0.000 0.000

0.000 0.000 0.009 0.000 0.009 0.009 0.000 0.005 0.005 0.000

0.080 0.079 0.037 0.076 0.019 0.000 0.000 0.000 0.000 0.013

0.000 0.006 0.000 0.000 0.000 0.000 0.025 0.000 0.013 0.006

0.000 0.000 0.000 0.000 0.048 0.072 0.067 0.064 0.000 0.000

0.000 0.000 0.000 0.020 0.000 0.000 0.000 0.013 0.000 0.000

0.020 0.007 0.007 0.007 0.000 0.000 0.000 0.000 0.059 0.071

0.061 0.071

>Non-Methylation code ID 1116

0.014 0.000 0.000 0.000 0.005 0.005 0.000 0.000 0.000 0.005

0.000 0.000 0.014 0.000 0.014 0.005 0.000 0.000 0.000 0.000

0.087 0.079 0.082 0.024 0.006 0.000 0.006 0.006 0.006 0.006

0.000 0.000 0.000 0.012 0.000 0.000 0.018 0.006 0.006 0.006

0.000 0.000 0.000 0.000 0.063 0.068 0.062 0.063 0.000 0.000

0.000 0.005 0.005 0.000 0.000 0.000 0.000 0.000 0.000 0.000

0.009 0.000 0.014 0.009 0.005 0.005 0.000 0.000 0.041 0.065

0.086 0.092

>Non-Methylation code ID 1116

0.000 0.000 0.005 0.000 0.000 0.005 0.005 0.000 0.000 0.005

0.000 0.000 0.019 0.005 0.014 0.000 0.000 0.000 0.000 0.000

0.076 0.063 0.087 0.049 0.009 0.000 0.000 0.005 0.005 0.005

0.000 0.000 0.000 0.000 0.000 0.000 0.014 0.000 0.009 0.000

0.009 0.000 0.005 0.000 0.072 0.063 0.071 0.065 0.018 0.000

0.000 0.000 0.000 0.018 0.000 0.000 0.000 0.000 0.000 0.000

0.054 0.000 0.009 0.009 0.000 0.000 0.000 0.000 0.044 0.053

0.067 0.063

>Non-Methylation code ID 1116

0.008 0.000 0.000 0.000 0.004 0.008 0.004 0.000 0.000 0.004

0.000 0.000 0.011 0.000 0.011 0.000 0.000 0.000 0.000 0.000

0.081 0.064 0.059 0.079 0.005 0.005 0.005 0.000 0.000 0.010

0.000 0.000 0.000 0.000 0.000 0.000 0.015 0.005 0.005 0.005

0.005 0.005 0.000 0.000 0.062 0.051 0.083 0.071 0.010 0.000

0.000 0.005 0.000 0.005 0.000 0.005 0.000 0.000 0.000 0.000

0.016 0.000 0.016 0.000 0.000 0.000 0.000 0.000 0.061 0.068

0.065 0.081

>Non-Methylation code ID 1116

0.006 0.000 0.000 0.006 0.000 0.011 0.000 0.006 0.000 0.006

0.000 0.000 0.034 0.000 0.006 0.000 0.000 0.000 0.000 0.000

0.068 0.067 0.050 0.074 0.008 0.000 0.000 0.000 0.004 0.004

0.000 0.000 0.000 0.000 0.000 0.000 0.016 0.000 0.016 0.004

0.000 0.000 0.000 0.000 0.073 0.078 0.076 0.054 0.012 0.000

0.006 0.000 0.000 0.006 0.000 0.000 0.000 0.000 0.000 0.000

0.029 0.000 0.012 0.006 0.000 0.000 0.000 0.000 0.082 0.051

0.073 0.056

>Non-Methylation code ID 1116

0.006 0.000 0.000 0.006 0.000 0.006 0.006 0.000 0.012 0.006

0.000 0.000 0.006 0.000 0.000 0.018 0.006 0.000 0.000 0.006

0.068 0.065 0.050 0.071 0.000 0.000 0.000 0.005 0.000 0.000

0.005 0.000 0.005 0.005 0.000 0.000 0.010 0.000 0.010 0.010

0.010 0.005 0.000 0.000 0.065 0.067 0.065 0.072 0.005 0.000

0.005 0.000 0.005 0.005 0.000 0.005 0.005 0.000 0.000 0.000

0.010 0.010 0.010 0.000 0.000 0.000 0.000 0.000 0.054 0.080

0.075 0.062

>Non-Methylation code ID 1116

0.011 0.000 0.000 0.000 0.000 0.017 0.000 0.000 0.000 0.006

0.000 0.000 0.011 0.006 0.011 0.011 0.000 0.000 0.000 0.000

0.081 0.067 0.062 0.051 0.026 0.000 0.000 0.007 0.000 0.007

0.000 0.000 0.000 0.000 0.000 0.000 0.020 0.000 0.007 0.013

0.000 0.007 0.000 0.000 0.072 0.059 0.060 0.057 0.009 0.000

0.000 0.004 0.000 0.000 0.000 0.000 0.000 0.000 0.000 0.000

0.013 0.000 0.018 0.009 0.000 0.000 0.000 0.000 0.068 0.077

0.079 0.058

>Non-Methylation code ID 1116

0.009 0.000 0.000 0.005 0.005 0.005 0.000 0.000 0.000 0.000

0.000 0.000 0.014 0.000 0.009 0.005 0.005 0.000 0.005 0.000

0.075 0.064 0.069 0.065 0.016 0.000 0.000 0.000 0.000 0.016

0.000 0.000 0.000 0.000 0.000 0.000 0.057 0.000 0.008 0.008

0.000 0.000 0.000 0.000 0.046 0.054 0.064 0.064 0.000 0.000

0.000 0.000 0.000 0.005 0.005 0.000 0.000 0.005 0.000 0.000

0.021 0.005 0.016 0.000 0.000 0.000 0.000 0.000 0.074 0.066

0.085 0.050

>Non-Methylation code ID 1116

0.004 0.000 0.000 0.000 0.000 0.013 0.000 0.000 0.000 0.013

0.000 0.000 0.013 0.000 0.013 0.000 0.000 0.000 0.000 0.000

0.080 0.060 0.053 0.084 0.016 0.000 0.005 0.005 0.000 0.005

0.005 0.000 0.000 0.005 0.000 0.000 0.011 0.000 0.005 0.005

0.000 0.000 0.005 0.000 0.066 0.080 0.061 0.057 0.000 0.000

0.000 0.006 0.000 0.006 0.000 0.000 0.000 0.000 0.000 0.000

0.024 0.006 0.006 0.006 0.012 0.000 0.006 0.000 0.070 0.058

0.067 0.067

>Non-Methylation code ID 1116

0.000 0.000 0.000 0.000 0.000 0.006 0.006 0.000 0.000 0.017

0.000 0.000 0.023 0.006 0.012 0.000 0.000 0.000 0.000 0.000

0.069 0.077 0.076 0.041 0.009 0.000 0.000 0.000 0.005 0.005

0.000 0.000 0.000 0.000 0.000 0.000 0.014 0.000 0.009 0.009

0.005 0.000 0.005 0.000 0.073 0.070 0.073 0.057 0.022 0.000

0.000 0.000 0.000 0.022 0.000 0.000 0.000 0.000 0.000 0.000

0.076 0.000 0.000 0.011 0.000 0.000 0.000 0.000 0.034 0.053

0.058 0.058

>Non-Methylation code ID 1116

0.000 0.005 0.000 0.000 0.005 0.000 0.000 0.000 0.000 0.000

0.000 0.005 0.009 0.005 0.014 0.014 0.005 0.000 0.000 0.000

0.078 0.058 0.064 0.072 0.027 0.000 0.000 0.000 0.000 0.007

0.007 0.000 0.000 0.000 0.000 0.000 0.007 0.007 0.007 0.014

0.007 0.007 0.000 0.000 0.052 0.062 0.053 0.079 0.008 0.000

0.000 0.000 0.008 0.000 0.000 0.000 0.000 0.000 0.000 0.008

0.023 0.023 0.000 0.000 0.008 0.008 0.000 0.000 0.055 0.065

0.063 0.064

>Non-Methylation code ID 1116

0.016 0.000 0.000 0.008 0.000 0.000 0.000 0.000 0.000 0.016

0.000 0.008 0.016 0.024 0.000 0.008 0.008 0.000 0.000 0.000

0.047 0.052 0.059 0.070 0.000 0.000 0.000 0.000 0.000 0.000

0.006 0.000 0.000 0.006 0.000 0.006 0.006 0.013 0.019 0.013

0.006 0.000 0.000 0.000 0.063 0.070 0.054 0.070 0.016 0.000

0.000 0.008 0.000 0.000 0.008 0.000 0.000 0.008 0.000 0.000

0.008 0.000 0.008 0.024 0.016 0.000 0.000 0.000 0.060 0.061

0.060 0.057

>Non-Methylation code ID 1116

0.011 0.000 0.000 0.006 0.000 0.011 0.000 0.006 0.000 0.006

0.000 0.000 0.029 0.000 0.006 0.000 0.000 0.000 0.000 0.000

0.062 0.069 0.055 0.073 0.008 0.000 0.000 0.000 0.004 0.004

0.000 0.000 0.000 0.004 0.000 0.000 0.016 0.000 0.012 0.004

0.000 0.000 0.000 0.000 0.083 0.071 0.058 0.069 0.006 0.006

0.006 0.000 0.000 0.012 0.000 0.000 0.000 0.000 0.000 0.000

0.031 0.000 0.006 0.006 0.000 0.000 0.000 0.000 0.069 0.052

0.090 0.049

>Non-Methylation code ID 1116

0.023 0.000 0.000 0.000 0.000 0.008 0.000 0.000 0.000 0.000

0.000 0.000 0.053 0.000 0.008 0.008 0.000 0.000 0.000 0.000

0.060 0.066 0.058 0.049 0.000 0.000 0.004 0.000 0.000 0.004

0.004 0.000 0.000 0.009 0.000 0.000 0.017 0.004 0.013 0.000

0.000 0.000 0.000 0.000 0.070 0.066 0.093 0.048 0.012 0.000

0.000 0.006 0.006 0.006 0.000 0.000 0.000 0.000 0.000 0.000

0.018 0.000 0.006 0.006 0.012 0.000 0.000 0.000 0.064 0.066

0.070 0.061

>Non-Methylation code ID 1116

0.000 0.000 0.000 0.000 0.000 0.011 0.006 0.000 0.000 0.017

0.000 0.000 0.011 0.006 0.011 0.006 0.000 0.000 0.000 0.000

0.063 0.081 0.071 0.049 0.009 0.000 0.000 0.000 0.005 0.009

0.005 0.000 0.000 0.000 0.000 0.000 0.005 0.000 0.009 0.009

0.005 0.000 0.005 0.000 0.075 0.069 0.044 0.086 0.032 0.000

0.000 0.000 0.000 0.022 0.000 0.011 0.000 0.000 0.000 0.000

0.054 0.000 0.000 0.011 0.000 0.000 0.000 0.000 0.049 0.051

0.050 0.053

>Non-Methylation code ID 1116

0.006 0.000 0.000 0.000 0.006 0.012 0.006 0.000 0.000 0.006

0.000 0.000 0.006 0.012 0.006 0.006 0.006 0.000 0.000 0.000

0.063 0.057 0.061 0.079 0.000 0.000 0.006 0.000 0.000 0.013

0.006 0.000 0.000 0.006 0.000 0.000 0.006 0.006 0.006 0.026

0.006 0.000 0.000 0.000 0.066 0.066 0.062 0.056 0.013 0.000

0.000 0.006 0.000 0.006 0.000 0.006 0.000 0.013 0.000 0.000

0.006 0.000 0.013 0.006 0.006 0.000 0.000 0.000 0.083 0.038

0.074 0.062

>Non-Methylation code ID 1116

0.005 0.005 0.000 0.000 0.005 0.005 0.000 0.000 0.000 0.000

0.000 0.000 0.010 0.005 0.010 0.016 0.005 0.000 0.000 0.000

0.079 0.076 0.055 0.056 0.010 0.000 0.000 0.005 0.000 0.005

0.010 0.000 0.000 0.000 0.000 0.000 0.005 0.005 0.010 0.010

0.000 0.005 0.000 0.000 0.067 0.083 0.056 0.059 0.000 0.000

0.000 0.008 0.008 0.000 0.000 0.000 0.000 0.008 0.000 0.008

0.015 0.008 0.000 0.008 0.015 0.008 0.000 0.000 0.069 0.059

0.046 0.075

>Non-Methylation code ID 1116

0.014 0.007 0.000 0.000 0.000 0.014 0.007 0.000 0.000 0.007

0.000 0.000 0.014 0.007 0.000 0.007 0.007 0.007 0.000 0.000

0.057 0.051 0.051 0.079 0.011 0.000 0.000 0.000 0.000 0.005

0.000 0.000 0.000 0.011 0.000 0.000 0.016 0.000 0.011 0.005

0.005 0.000 0.000 0.000 0.048 0.064 0.088 0.070 0.005 0.005

0.000 0.000 0.000 0.005 0.005 0.000 0.000 0.010 0.000 0.000

0.010 0.000 0.014 0.005 0.000 0.000 0.000 0.000 0.083 0.064

0.058 0.073

>Non-Methylation code ID 1116

0.009 0.000 0.000 0.000 0.004 0.004 0.000 0.000 0.000 0.000

0.000 0.000 0.009 0.000 0.013 0.009 0.004 0.000 0.004 0.000

0.074 0.074 0.056 0.070 0.031 0.000 0.000 0.000 0.000 0.021

0.000 0.000 0.000 0.000 0.000 0.000 0.072 0.000 0.000 0.010

0.000 0.000 0.000 0.000 0.038 0.055 0.054 0.053 0.000 0.000

0.000 0.000 0.000 0.006 0.006 0.000 0.000 0.018 0.000 0.000

0.018 0.006 0.012 0.000 0.000 0.000 0.000 0.000 0.057 0.082

0.082 0.045

>Non-Methylation code ID 1116

0.006 0.006 0.000 0.006 0.006 0.006 0.006 0.000 0.000 0.012

0.000 0.000 0.000 0.000 0.006 0.018 0.000 0.000 0.000 0.000

0.059 0.076 0.071 0.056 0.005 0.005 0.005 0.000 0.000 0.005

0.005 0.005 0.000 0.005 0.000 0.010 0.005 0.000 0.005 0.010

0.000 0.000 0.000 0.000 0.085 0.051 0.064 0.069 0.000 0.000

0.000 0.000 0.011 0.022 0.000 0.022 0.000 0.022 0.000 0.000

0.011 0.000 0.000 0.000 0.022 0.011 0.000 0.000 0.047 0.054

0.049 0.063

>Non-Methylation code ID 1116

0.000 0.000 0.000 0.000 0.000 0.017 0.000 0.006 0.006 0.012

0.000 0.000 0.000 0.012 0.012 0.000 0.012 0.000 0.000 0.000

0.044 0.063 0.067 0.085 0.004 0.000 0.000 0.004 0.004 0.013

0.004 0.000 0.000 0.000 0.000 0.000 0.004 0.000 0.013 0.004

0.000 0.000 0.000 0.000 0.076 0.071 0.075 0.058 0.006 0.000

0.012 0.006 0.000 0.012 0.006 0.000 0.006 0.000 0.000 0.000

0.000 0.006 0.006 0.006 0.006 0.000 0.000 0.000 0.064 0.062

0.067 0.069

>Non-Methylation code ID 1116

0.000 0.000 0.008 0.000 0.000 0.000 0.008 0.008 0.008 0.000

0.000 0.016 0.000 0.016 0.000 0.016 0.016 0.000 0.000 0.000

0.057 0.067 0.054 0.056 0.008 0.000 0.000 0.000 0.000 0.000

0.000 0.000 0.008 0.008 0.000 0.000 0.008 0.000 0.008 0.016

0.032 0.008 0.000 0.000 0.074 0.082 0.025 0.057 0.000 0.000

0.008 0.008 0.000 0.000 0.000 0.000 0.000 0.000 0.000 0.008

0.015 0.031 0.008 0.008 0.000 0.000 0.000 0.000 0.063 0.067

0.070 0.050

>Non-Methylation code ID 1116

0.000 0.000 0.000 0.000 0.000 0.012 0.000 0.000 0.012 0.000

0.006 0.006 0.006 0.018 0.000 0.000 0.006 0.006 0.006 0.000

0.066 0.059 0.063 0.069 0.000 0.000 0.000 0.000 0.000 0.012

0.000 0.000 0.006 0.006 0.000 0.006 0.000 0.000 0.018 0.012

0.006 0.006 0.000 0.000 0.089 0.063 0.070 0.040 0.012 0.000

0.006 0.018 0.000 0.006 0.006 0.000 0.000 0.006 0.000 0.000

0.006 0.000 0.000 0.006 0.000 0.000 0.006 0.000 0.064 0.066

0.055 0.075

>Non-Methylation code ID 1116

0.007 0.000 0.007 0.007 0.000 0.007 0.000 0.000 0.000 0.007

0.000 0.000 0.000 0.022 0.007 0.007 0.007 0.000 0.007 0.000

0.078 0.050 0.068 0.049 0.000 0.006 0.006 0.006 0.000 0.012

0.000 0.000 0.006 0.006 0.000 0.006 0.006 0.006 0.000 0.012

0.006 0.000 0.000 0.000 0.068 0.056 0.068 0.064 0.010 0.005

0.000 0.000 0.000 0.000 0.005 0.000 0.005 0.005 0.000 0.000

0.000 0.000 0.010 0.000 0.010 0.005 0.000 0.000 0.070 0.064

0.058 0.085

>Non-Methylation code ID 1116

0.000 0.000 0.000 0.000 0.000 0.006 0.012 0.000 0.000 0.006

0.000 0.006 0.012 0.006 0.006 0.012 0.006 0.006 0.000 0.000

0.073 0.058 0.052 0.073 0.000 0.006 0.000 0.000 0.000 0.006

0.006 0.006 0.000 0.000 0.000 0.000 0.013 0.006 0.006 0.013

0.013 0.006 0.000 0.000 0.062 0.051 0.075 0.062 0.015 0.000

0.000 0.000 0.007 0.015 0.007 0.000 0.000 0.000 0.000 0.000

0.015 0.015 0.000 0.007 0.007 0.000 0.000 0.000 0.048 0.063

0.065 0.069

>Non-Methylation code ID 1116

0.005 0.000 0.005 0.005 0.000 0.005 0.011 0.000 0.005 0.000

0.000 0.005 0.000 0.000 0.016 0.005 0.000 0.000 0.000 0.000

0.054 0.071 0.062 0.082 0.000 0.000 0.007 0.007 0.000 0.021

0.000 0.000 0.014 0.000 0.007 0.000 0.000 0.007 0.000 0.000

0.021 0.007 0.000 0.000 0.063 0.059 0.060 0.059 0.000 0.000

0.000 0.015 0.000 0.007 0.000 0.000 0.015 0.007 0.000 0.000

0.000 0.007 0.007 0.015 0.007 0.000 0.000 0.000 0.061 0.051

0.071 0.070

>Non-Methylation code ID 1116

0.027 0.000 0.000 0.000 0.000 0.000 0.005 0.000 0.000 0.000

0.000 0.000 0.011 0.005 0.011 0.005 0.005 0.000 0.000 0.000

0.083 0.043 0.089 0.048 0.000 0.000 0.000 0.000 0.000 0.012

0.018 0.000 0.000 0.012 0.000 0.000 0.012 0.006 0.006 0.000

0.006 0.006 0.000 0.000 0.061 0.048 0.074 0.071 0.000 0.010

0.000 0.000 0.000 0.030 0.010 0.000 0.000 0.000 0.000 0.000

0.000 0.020 0.000 0.020 0.020 0.000 0.000 0.000 0.059 0.042

0.050 0.072

>Non-Methylation code ID 1128

0.013 0.000 0.000 0.006 0.000 0.006 0.006 0.006 0.000 0.000

0.000 0.000 0.013 0.000 0.006 0.019 0.006 0.000 0.000 0.000

0.073 0.053 0.056 0.068 0.000 0.000 0.007 0.000 0.000 0.000

0.007 0.000 0.000 0.015 0.000 0.007 0.030 0.007 0.000 0.007

0.007 0.000 0.000 0.000 0.070 0.062 0.056 0.057 0.000 0.000

0.000 0.000 0.005 0.000 0.005 0.005 0.000 0.000 0.000 0.000

0.014 0.000 0.010 0.005 0.005 0.000 0.005 0.005 0.079 0.071

0.052 0.075

>Non-Methylation code ID 1128

0.000 0.000 0.006 0.000 0.006 0.000 0.000 0.000 0.000 0.000

0.000 0.006 0.013 0.026 0.006 0.000 0.000 0.006 0.000 0.000

0.054 0.058 0.071 0.079 0.000 0.006 0.000 0.000 0.000 0.000

0.000 0.006 0.006 0.000 0.000 0.012 0.000 0.012 0.000 0.018

0.006 0.000 0.006 0.000 0.060 0.071 0.067 0.064 0.022 0.000

0.000 0.000 0.000 0.011 0.000 0.000 0.011 0.011 0.000 0.000

0.011 0.000 0.000 0.033 0.022 0.000 0.000 0.000 0.049 0.057

0.040 0.067

>Non-Methylation code ID 1128

0.000 0.008 0.000 0.000 0.000 0.008 0.008 0.008 0.000 0.000

0.000 0.000 0.015 0.008 0.000 0.023 0.015 0.008 0.000 0.000

0.056 0.047 0.064 0.065 0.014 0.000 0.000 0.000 0.007 0.007

0.007 0.000 0.000 0.000 0.000 0.000 0.021 0.014 0.000 0.014

0.007 0.000 0.000 0.000 0.050 0.065 0.064 0.064 0.000 0.000

0.000 0.000 0.000 0.000 0.013 0.000 0.000 0.007 0.000 0.007

0.013 0.007 0.007 0.007 0.007 0.013 0.000 0.000 0.078 0.054

0.061 0.061

>Non-Methylation code ID 1128

0.005 0.000 0.000 0.000 0.005 0.000 0.000 0.005 0.000 0.010

0.000 0.005 0.010 0.000 0.015 0.000 0.000 0.005 0.000 0.000

0.052 0.065 0.070 0.084 0.005 0.005 0.000 0.005 0.000 0.005

0.005 0.000 0.000 0.021 0.005 0.000 0.000 0.000 0.005 0.005

0.000 0.000 0.000 0.005 0.055 0.064 0.069 0.078 0.007 0.007

0.000 0.007 0.007 0.007 0.007 0.007 0.007 0.000 0.000 0.000

0.007 0.000 0.000 0.007 0.007 0.000 0.000 0.000 0.074 0.066

0.066 0.048

>Non-Methylation code ID 1128

0.000 0.000 0.000 0.000 0.000 0.008 0.000 0.000 0.000 0.008

0.000 0.000 0.042 0.025 0.000 0.008 0.017 0.000 0.000 0.000

0.040 0.046 0.061 0.077 0.000 0.004 0.000 0.000 0.000 0.000

0.009 0.000 0.004 0.004 0.000 0.004 0.013 0.004 0.009 0.000

0.000 0.004 0.000 0.000 0.072 0.066 0.076 0.061 0.012 0.000

0.006 0.000 0.006 0.000 0.000 0.000 0.000 0.000 0.000 0.006

0.019 0.000 0.006 0.000 0.019 0.000 0.000 0.000 0.063 0.063

0.059 0.073

>Non-Methylation code ID 1128

0.016 0.005 0.000 0.000 0.000 0.000 0.000 0.000 0.000 0.016

0.000 0.000 0.005 0.005 0.010 0.005 0.005 0.000 0.000 0.000

0.070 0.058 0.075 0.063 0.012 0.006 0.000 0.000 0.000 0.006

0.012 0.000 0.000 0.018 0.000 0.000 0.012 0.000 0.006 0.000

0.000 0.000 0.000 0.000 0.056 0.074 0.073 0.058 0.008 0.008

0.000 0.000 0.000 0.016 0.000 0.000 0.000 0.016 0.000 0.008

0.016 0.000 0.000 0.008 0.016 0.000 0.000 0.000 0.057 0.049

0.061 0.070

>Non-Methylation code ID 1261

0.000 0.006 0.006 0.012 0.000 0.012 0.000 0.006 0.000 0.006

0.000 0.006 0.006 0.006 0.000 0.006 0.006 0.000 0.000 0.000

0.061 0.059 0.073 0.064 0.010 0.005 0.000 0.000 0.000 0.000

0.005 0.000 0.005 0.005 0.000 0.000 0.000 0.000 0.010 0.000

0.010 0.005 0.000 0.000 0.063 0.065 0.069 0.079 0.007 0.000

0.007 0.000 0.000 0.007 0.000 0.000 0.000 0.007 0.000 0.000

0.000 0.014 0.014 0.007 0.007 0.000 0.007 0.000 0.086 0.041

0.080 0.051

>Non-Methylation code ID 1261

0.000 0.000 0.000 0.000 0.000 0.000 0.005 0.005 0.005 0.000

0.000 0.000 0.015 0.000 0.005 0.010 0.010 0.000 0.005 0.005

0.070 0.076 0.063 0.057 0.012 0.000 0.000 0.000 0.000 0.006

0.006 0.006 0.000 0.000 0.000 0.000 0.018 0.000 0.012 0.006

0.006 0.000 0.000 0.000 0.079 0.070 0.040 0.075 0.000 0.000

0.009 0.009 0.000 0.000 0.009 0.000 0.000 0.009 0.000 0.000

0.026 0.017 0.000 0.009 0.009 0.000 0.000 0.000 0.060 0.070

0.053 0.054

>Non-Methylation code ID 1261

0.000 0.000 0.000 0.000 0.000 0.000 0.005 0.005 0.005 0.000

0.000 0.000 0.014 0.000 0.010 0.005 0.010 0.000 0.005 0.005

0.075 0.084 0.051 0.061 0.011 0.000 0.000 0.005 0.000 0.005

0.005 0.005 0.000 0.000 0.000 0.000 0.016 0.000 0.011 0.005

0.005 0.000 0.000 0.000 0.076 0.072 0.043 0.072 0.000 0.000

0.008 0.000 0.000 0.000 0.008 0.000 0.000 0.008 0.000 0.008

0.025 0.017 0.000 0.008 0.008 0.000 0.000 0.000 0.066 0.071

0.052 0.053

>Non-Methylation code ID 1261

0.005 0.000 0.005 0.005 0.000 0.005 0.005 0.005 0.011 0.005

0.000 0.011 0.000 0.005 0.005 0.000 0.000 0.000 0.000 0.000

0.078 0.067 0.052 0.068 0.008 0.000 0.000 0.000 0.000 0.008

0.000 0.000 0.008 0.000 0.008 0.008 0.000 0.008 0.008 0.016

0.024 0.000 0.000 0.000 0.057 0.057 0.071 0.054 0.005 0.005

0.000 0.010 0.000 0.000 0.005 0.000 0.000 0.000 0.000 0.000

0.010 0.010 0.010 0.000 0.005 0.000 0.000 0.000 0.070 0.077

0.059 0.064

>Non-Methylation code ID 1261

0.000 0.000 0.000 0.000 0.008 0.016 0.000 0.008 0.000 0.016

0.000 0.000 0.008 0.000 0.008 0.008 0.016 0.008 0.000 0.000

0.058 0.058 0.055 0.067 0.006 0.006 0.000 0.006 0.000 0.006

0.006 0.000 0.000 0.011 0.000 0.000 0.006 0.000 0.006 0.017

0.000 0.000 0.000 0.000 0.056 0.070 0.069 0.070 0.005 0.005

0.005 0.000 0.000 0.005 0.000 0.005 0.000 0.005 0.000 0.011

0.005 0.005 0.005 0.005 0.000 0.000 0.000 0.000 0.083 0.056

0.059 0.071

>Non-Methylation code ID 1261

0.000 0.000 0.000 0.000 0.000 0.032 0.011 0.000 0.000 0.021

0.000 0.000 0.011 0.021 0.000 0.011 0.021 0.000 0.000 0.000

0.042 0.043 0.047 0.074 0.015 0.005 0.000 0.000 0.000 0.000

0.000 0.000 0.000 0.005 0.000 0.000 0.010 0.005 0.010 0.010

0.005 0.000 0.000 0.000 0.089 0.035 0.083 0.058 0.005 0.000

0.000 0.000 0.000 0.011 0.011 0.000 0.000 0.005 0.000 0.000

0.022 0.000 0.005 0.000 0.000 0.005 0.000 0.000 0.048 0.055

0.084 0.081

>Non-Methylation code ID 1261

0.000 0.000 0.007 0.000 0.000 0.000 0.007 0.007 0.007 0.000

0.000 0.015 0.000 0.015 0.000 0.015 0.007 0.000 0.007 0.000

0.054 0.062 0.064 0.063 0.008 0.000 0.000 0.000 0.000 0.008

0.000 0.000 0.008 0.000 0.000 0.000 0.008 0.000 0.008 0.017

0.033 0.008 0.000 0.000 0.076 0.069 0.030 0.060 0.000 0.000

0.008 0.008 0.000 0.000 0.000 0.000 0.000 0.000 0.000 0.008

0.015 0.031 0.008 0.008 0.000 0.000 0.000 0.000 0.063 0.067

0.070 0.050

>Non-Methylation code ID 1261

0.011 0.000 0.006 0.000 0.006 0.000 0.000 0.000 0.000 0.000

0.000 0.006 0.017 0.000 0.006 0.000 0.017 0.000 0.000 0.006

0.059 0.068 0.062 0.073 0.000 0.000 0.000 0.000 0.000 0.009

0.000 0.000 0.000 0.009 0.000 0.000 0.044 0.018 0.000 0.009

0.026 0.000 0.000 0.000 0.040 0.051 0.053 0.074 0.000 0.005

0.000 0.000 0.000 0.000 0.010 0.000 0.000 0.005 0.000 0.005

0.015 0.005 0.010 0.000 0.000 0.005 0.000 0.000 0.076 0.073

0.075 0.050

>Non-Methylation code ID 1261

0.000 0.005 0.000 0.000 0.000 0.010 0.005 0.000 0.005 0.005

0.000 0.015 0.005 0.000 0.010 0.000 0.000 0.005 0.000 0.000

0.069 0.080 0.047 0.072 0.022 0.000 0.000 0.007 0.000 0.007

0.000 0.007 0.000 0.007 0.000 0.007 0.007 0.000 0.000 0.000

0.022 0.000 0.007 0.000 0.051 0.058 0.055 0.073 0.000 0.000

0.000 0.000 0.000 0.016 0.000 0.000 0.008 0.008 0.000 0.000

0.016 0.024 0.000 0.000 0.008 0.000 0.008 0.000 0.071 0.050

0.067 0.059

>Non-Methylation code ID 1261

0.000 0.005 0.000 0.000 0.000 0.005 0.010 0.000 0.005 0.000

0.000 0.015 0.015 0.000 0.005 0.000 0.000 0.005 0.000 0.000

0.066 0.059 0.066 0.077 0.025 0.000 0.000 0.000 0.000 0.000

0.000 0.008 0.000 0.008 0.000 0.008 0.025 0.000 0.000 0.000

0.034 0.000 0.000 0.000 0.050 0.047 0.052 0.074 0.000 0.000

0.000 0.000 0.000 0.000 0.000 0.000 0.000 0.010 0.000 0.000

0.052 0.031 0.000 0.000 0.010 0.000 0.010 0.000 0.041 0.040

0.057 0.080

>Non-Methylation code ID 1261

0.000 0.000 0.000 0.000 0.000 0.006 0.006 0.000 0.000 0.006

0.000 0.006 0.019 0.006 0.006 0.013 0.000 0.013 0.000 0.000

0.076 0.049 0.064 0.062 0.000 0.000 0.000 0.000 0.006 0.006

0.006 0.006 0.000 0.006 0.000 0.000 0.011 0.000 0.006 0.011

0.006 0.006 0.000 0.006 0.053 0.060 0.072 0.075 0.000 0.007

0.000 0.000 0.007 0.015 0.000 0.007 0.000 0.000 0.000 0.000

0.015 0.015 0.000 0.015 0.007 0.000 0.000 0.000 0.057 0.055

0.069 0.064

>Non-Methylation code ID 1261

0.012 0.000 0.006 0.000 0.006 0.000 0.000 0.000 0.000 0.000

0.000 0.006 0.018 0.000 0.006 0.000 0.024 0.000 0.000 0.000

0.065 0.060 0.056 0.075 0.000 0.000 0.000 0.000 0.000 0.008

0.000 0.000 0.000 0.008 0.000 0.000 0.042 0.025 0.000 0.008

0.017 0.000 0.000 0.000 0.040 0.046 0.061 0.077 0.000 0.005

0.000 0.000 0.000 0.000 0.010 0.000 0.005 0.000 0.000 0.005

0.014 0.005 0.010 0.000 0.000 0.005 0.000 0.000 0.077 0.069

0.068 0.062

>Non-Methylation code ID 1261

0.005 0.000 0.000 0.005 0.000 0.009 0.005 0.000 0.005 0.000

0.000 0.005 0.005 0.000 0.019 0.000 0.000 0.000 0.000 0.000

0.070 0.063 0.074 0.070 0.006 0.000 0.013 0.006 0.000 0.019

0.000 0.000 0.013 0.000 0.000 0.000 0.000 0.013 0.000 0.000

0.013 0.000 0.000 0.000 0.062 0.067 0.060 0.062 0.000 0.000

0.000 0.013 0.000 0.006 0.000 0.000 0.006 0.006 0.000 0.000

0.000 0.013 0.013 0.006 0.013 0.000 0.000 0.000 0.054 0.070

0.070 0.062

>Non-Methylation code ID 1261

0.005 0.000 0.000 0.000 0.000 0.011 0.011 0.000 0.000 0.005

0.000 0.000 0.022 0.000 0.005 0.000 0.000 0.005 0.000 0.000

0.048 0.055 0.084 0.081 0.000 0.000 0.000 0.000 0.000 0.031

0.000 0.000 0.000 0.021 0.000 0.010 0.010 0.021 0.000 0.010

0.021 0.000 0.000 0.000 0.051 0.046 0.053 0.060 0.016 0.005

0.000 0.000 0.000 0.000 0.000 0.000 0.000 0.005 0.000 0.000

0.011 0.005 0.011 0.011 0.000 0.000 0.000 0.000 0.095 0.033

0.079 0.061

>Non-Methylation code ID 1261

0.000 0.000 0.004 0.000 0.008 0.000 0.004 0.000 0.000 0.004

0.000 0.000 0.012 0.000 0.008 0.000 0.004 0.004 0.000 0.004

0.061 0.072 0.066 0.081 0.000 0.008 0.000 0.000 0.000 0.016

0.000 0.008 0.000 0.000 0.000 0.000 0.016 0.016 0.000 0.023

0.016 0.000 0.000 0.000 0.048 0.063 0.057 0.063 0.000 0.000

0.000 0.000 0.000 0.007 0.007 0.000 0.000 0.007 0.000 0.000

0.027 0.007 0.007 0.007 0.000 0.014 0.000 0.000 0.064 0.062

0.055 0.071

>Non-Methylation code ID 1261

0.000 0.000 0.005 0.010 0.000 0.010 0.005 0.005 0.005 0.005

0.000 0.010 0.000 0.005 0.005 0.000 0.000 0.000 0.000 0.000

0.065 0.072 0.063 0.069 0.016 0.000 0.000 0.000 0.000 0.000

0.000 0.000 0.016 0.000 0.000 0.008 0.000 0.000 0.008 0.008

0.032 0.000 0.000 0.000 0.062 0.087 0.063 0.034 0.000 0.000

0.000 0.000 0.000 0.005 0.005 0.000 0.000 0.000 0.000 0.000

0.010 0.014 0.014 0.000 0.005 0.000 0.005 0.000 0.074 0.070

0.078 0.054

>Non-Methylation code ID 1261

0.005 0.000 0.000 0.000 0.005 0.000 0.000 0.005 0.000 0.011

0.000 0.005 0.016 0.000 0.011 0.005 0.000 0.000 0.000 0.005

0.063 0.071 0.071 0.058 0.000 0.006 0.000 0.000 0.000 0.000

0.006 0.006 0.000 0.006 0.000 0.000 0.006 0.006 0.006 0.013

0.006 0.006 0.000 0.000 0.060 0.078 0.059 0.065 0.015 0.000

0.007 0.000 0.007 0.000 0.000 0.000 0.000 0.007 0.000 0.000

0.022 0.007 0.000 0.015 0.007 0.000 0.000 0.000 0.061 0.064

0.061 0.060

>Non-Methylation code ID 1261

0.010 0.005 0.000 0.000 0.000 0.010 0.000 0.000 0.000 0.010

0.000 0.000 0.005 0.005 0.005 0.010 0.000 0.000 0.005 0.000

0.055 0.070 0.077 0.065 0.024 0.000 0.000 0.008 0.000 0.016

0.008 0.000 0.008 0.016 0.000 0.000 0.000 0.000 0.000 0.016

0.000 0.008 0.000 0.000 0.058 0.054 0.061 0.057 0.000 0.000

0.000 0.000 0.000 0.005 0.000 0.000 0.011 0.005 0.000 0.000

0.000 0.005 0.011 0.011 0.005 0.000 0.005 0.000 0.062 0.058

0.063 0.089

>Non-Methylation code ID 1261

0.032 0.000 0.000 0.008 0.000 0.016 0.008 0.000 0.000 0.024

0.000 0.000 0.008 0.000 0.000 0.008 0.000 0.000 0.000 0.000

0.055 0.062 0.068 0.046 0.000 0.000 0.000 0.000 0.000 0.010

0.000 0.000 0.005 0.010 0.000 0.000 0.000 0.010 0.016 0.005

0.005 0.000 0.005 0.000 0.059 0.062 0.070 0.074 0.016 0.000

0.000 0.000 0.000 0.016 0.000 0.000 0.000 0.005 0.000 0.000

0.005 0.000 0.011 0.011 0.000 0.000 0.000 0.000 0.065 0.066

0.063 0.075

>Non-Methylation code ID 1261

0.000 0.007 0.000 0.000 0.000 0.000 0.000 0.000 0.000 0.030

0.000 0.000 0.022 0.007 0.000 0.007 0.022 0.000 0.000 0.000

0.040 0.065 0.058 0.074 0.006 0.006 0.000 0.000 0.006 0.000

0.006 0.000 0.006 0.006 0.000 0.000 0.023 0.000 0.006 0.006

0.000 0.000 0.000 0.000 0.058 0.063 0.063 0.079 0.012 0.000

0.006 0.000 0.006 0.000 0.012 0.000 0.000 0.012 0.000 0.000

0.012 0.000 0.000 0.006 0.006 0.000 0.000 0.000 0.062 0.056

0.074 0.068

>Non-Methylation code ID 1261

0.006 0.000 0.006 0.000 0.006 0.000 0.011 0.000 0.006 0.017

0.000 0.000 0.011 0.000 0.000 0.011 0.000 0.000 0.000 0.000

0.063 0.065 0.068 0.065 0.000 0.008 0.000 0.000 0.000 0.000

0.000 0.000 0.000 0.031 0.000 0.000 0.008 0.008 0.000 0.016

0.023 0.000 0.008 0.000 0.042 0.071 0.058 0.062 0.007 0.007

0.000 0.000 0.007 0.000 0.000 0.000 0.007 0.007 0.000 0.000

0.029 0.000 0.000 0.007 0.000 0.007 0.000 0.000 0.067 0.059

0.058 0.070

>Non-Methylation code ID 1261

0.000 0.008 0.000 0.000 0.000 0.008 0.000 0.000 0.000 0.031

0.000 0.000 0.023 0.000 0.000 0.008 0.023 0.000 0.000 0.000

0.047 0.073 0.057 0.054 0.006 0.006 0.000 0.000 0.000 0.006

0.006 0.000 0.000 0.006 0.000 0.000 0.024 0.000 0.006 0.006

0.000 0.000 0.006 0.000 0.059 0.072 0.059 0.071 0.013 0.000

0.006 0.000 0.006 0.006 0.013 0.000 0.000 0.013 0.000 0.000

0.013 0.000 0.000 0.000 0.006 0.000 0.000 0.000 0.058 0.044

0.075 0.080

>Non-Methylation code ID 1261

0.009 0.005 0.000 0.000 0.005 0.005 0.005 0.000 0.009 0.005

0.005 0.000 0.000 0.000 0.009 0.000 0.000 0.000 0.000 0.000

0.043 0.069 0.080 0.086 0.000 0.005 0.000 0.005 0.000 0.015

0.000 0.000 0.005 0.005 0.000 0.000 0.000 0.005 0.005 0.005

0.005 0.005 0.005 0.000 0.079 0.064 0.053 0.071 0.006 0.000

0.006 0.006 0.006 0.006 0.000 0.000 0.006 0.006 0.000 0.000

0.006 0.006 0.006 0.006 0.000 0.000 0.000 0.000 0.063 0.067

0.067 0.073

>Non-Methylation code ID 1261

0.006 0.000 0.000 0.000 0.000 0.006 0.000 0.000 0.006 0.000

0.000 0.006 0.006 0.006 0.013 0.013 0.019 0.000 0.000 0.000

0.077 0.056 0.042 0.076 0.005 0.000 0.000 0.011 0.000 0.005

0.005 0.000 0.000 0.000 0.000 0.000 0.011 0.016 0.011 0.000

0.005 0.000 0.000 0.000 0.066 0.080 0.058 0.059 0.006 0.000

0.006 0.006 0.000 0.000 0.006 0.000 0.018 0.006 0.000 0.006

0.000 0.006 0.012 0.000 0.000 0.000 0.000 0.000 0.074 0.087

0.048 0.054

>Non-Methylation code ID 1261

0.006 0.006 0.000 0.017 0.000 0.006 0.000 0.000 0.011 0.000

0.000 0.000 0.000 0.017 0.006 0.000 0.006 0.000 0.000 0.000

0.063 0.076 0.066 0.056 0.010 0.000 0.000 0.000 0.000 0.005

0.000 0.000 0.005 0.000 0.000 0.005 0.005 0.005 0.015 0.015

0.000 0.000 0.000 0.000 0.080 0.071 0.036 0.083 0.012 0.000

0.000 0.012 0.000 0.012 0.006 0.000 0.000 0.000 0.000 0.000

0.006 0.006 0.012 0.000 0.000 0.000 0.000 0.000 0.076 0.079

0.065 0.049

>Non-Methylation code ID 1261

0.000 0.000 0.000 0.000 0.000 0.010 0.005 0.000 0.000 0.005

0.000 0.000 0.005 0.000 0.010 0.015 0.005 0.005 0.005 0.000

0.075 0.066 0.079 0.050 0.021 0.007 0.000 0.007 0.000 0.007

0.007 0.000 0.000 0.014 0.000 0.000 0.007 0.007 0.000 0.000

0.007 0.007 0.000 0.000 0.048 0.054 0.072 0.068 0.006 0.000

0.000 0.000 0.006 0.006 0.000 0.000 0.000 0.012 0.000 0.000

0.017 0.000 0.006 0.012 0.000 0.000 0.000 0.006 0.066 0.071

0.069 0.057

>Non-Methylation code ID 1261

0.010 0.000 0.000 0.000 0.010 0.010 0.000 0.000 0.005 0.000

0.000 0.000 0.005 0.005 0.010 0.010 0.000 0.000 0.000 0.000

0.079 0.073 0.052 0.064 0.006 0.000 0.000 0.006 0.000 0.017

0.006 0.000 0.000 0.000 0.000 0.000 0.006 0.006 0.011 0.011

0.000 0.000 0.000 0.000 0.074 0.066 0.082 0.044 0.005 0.005

0.000 0.010 0.000 0.005 0.000 0.000 0.005 0.005 0.000 0.000

0.000 0.010 0.010 0.000 0.005 0.000 0.000 0.000 0.068 0.067

0.064 0.072

>Non-Methylation code ID 1261

0.005 0.000 0.005 0.005 0.010 0.005 0.000 0.000 0.000 0.005

0.000 0.000 0.010 0.005 0.005 0.005 0.000 0.000 0.000 0.000

0.067 0.059 0.071 0.074 0.010 0.005 0.000 0.000 0.005 0.005

0.010 0.000 0.005 0.005 0.005 0.000 0.000 0.000 0.005 0.005

0.000 0.000 0.000 0.000 0.052 0.074 0.070 0.077 0.000 0.005

0.000 0.005 0.000 0.011 0.000 0.000 0.000 0.005 0.000 0.000

0.005 0.005 0.005 0.005 0.005 0.005 0.005 0.000 0.072 0.069

0.070 0.059

>Non-Methylation code ID 1261

0.017 0.000 0.006 0.000 0.006 0.000 0.012 0.000 0.000 0.012

0.000 0.000 0.012 0.000 0.000 0.006 0.006 0.000 0.000 0.000

0.059 0.055 0.071 0.074 0.000 0.007 0.000 0.000 0.000 0.000

0.000 0.000 0.000 0.030 0.000 0.000 0.022 0.007 0.000 0.007

0.022 0.000 0.000 0.000 0.040 0.065 0.058 0.074 0.007 0.007

0.000 0.000 0.007 0.000 0.007 0.000 0.000 0.007 0.000 0.000

0.028 0.000 0.007 0.007 0.000 0.000 0.000 0.000 0.056 0.053

0.064 0.082

>Non-Methylation code ID 1261

0.006 0.006 0.000 0.000 0.006 0.000 0.006 0.000 0.006 0.006

0.000 0.000 0.023 0.000 0.006 0.006 0.000 0.000 0.000 0.000

0.058 0.063 0.063 0.079 0.011 0.000 0.005 0.000 0.005 0.000

0.011 0.000 0.005 0.011 0.000 0.000 0.011 0.000 0.000 0.005

0.005 0.000 0.000 0.000 0.060 0.059 0.078 0.065 0.000 0.008

0.000 0.000 0.000 0.000 0.000 0.000 0.000 0.032 0.000 0.000

0.016 0.008 0.000 0.008 0.024 0.000 0.000 0.000 0.044 0.064

0.056 0.073

>Non-Methylation code ID 1261

0.006 0.011 0.006 0.000 0.000 0.000 0.006 0.000 0.006 0.000

0.000 0.000 0.000 0.000 0.006 0.017 0.006 0.006 0.000 0.000

0.063 0.079 0.070 0.054 0.004 0.000 0.000 0.000 0.004 0.004

0.004 0.000 0.000 0.004 0.000 0.000 0.009 0.004 0.009 0.000

0.009 0.004 0.000 0.000 0.052 0.076 0.082 0.065 0.000 0.006

0.000 0.006 0.006 0.006 0.000 0.006 0.000 0.025 0.000 0.000

0.006 0.006 0.006 0.000 0.000 0.000 0.000 0.000 0.077 0.063

0.057 0.062

>Non-Methylation code ID 1261

0.016 0.000 0.000 0.000 0.005 0.000 0.000 0.005 0.005 0.005

0.000 0.005 0.005 0.005 0.005 0.005 0.000 0.000 0.000 0.005

0.075 0.065 0.061 0.061 0.000 0.000 0.000 0.000 0.000 0.006

0.000 0.000 0.000 0.006 0.000 0.000 0.006 0.006 0.012 0.019

0.012 0.006 0.006 0.000 0.083 0.061 0.054 0.055 0.007 0.007

0.000 0.014 0.000 0.000 0.007 0.000 0.000 0.007 0.000 0.000

0.014 0.014 0.000 0.007 0.000 0.007 0.000 0.000 0.061 0.070

0.069 0.049

>Non-Methylation code ID 1261

0.013 0.006 0.000 0.000 0.006 0.006 0.000 0.000 0.006 0.013

0.000 0.000 0.013 0.000 0.000 0.006 0.000 0.006 0.000 0.000

0.063 0.058 0.062 0.073 0.005 0.000 0.005 0.000 0.005 0.000

0.005 0.000 0.005 0.011 0.000 0.000 0.005 0.000 0.005 0.011

0.000 0.000 0.005 0.000 0.073 0.068 0.056 0.071 0.007 0.015

0.000 0.000 0.000 0.000 0.000 0.000 0.000 0.022 0.000 0.000

0.007 0.007 0.000 0.007 0.015 0.000 0.007 0.000 0.061 0.081

0.050 0.054

>Non-Methylation code ID 1261

0.000 0.000 0.000 0.000 0.000 0.011 0.000 0.000 0.000 0.006

0.000 0.000 0.000 0.000 0.017 0.017 0.011 0.006 0.006 0.000

0.080 0.060 0.077 0.045 0.020 0.007 0.000 0.013 0.000 0.007

0.007 0.000 0.000 0.007 0.000 0.000 0.013 0.007 0.000 0.000

0.000 0.007 0.000 0.000 0.054 0.065 0.070 0.058 0.006 0.000

0.000 0.000 0.006 0.006 0.000 0.006 0.000 0.006 0.000 0.006

0.012 0.006 0.006 0.006 0.000 0.000 0.000 0.006 0.076 0.063

0.071 0.053

>Non-Methylation code ID 1261

0.005 0.005 0.000 0.015 0.000 0.005 0.000 0.000 0.010 0.000

0.000 0.000 0.000 0.010 0.010 0.000 0.005 0.000 0.000 0.000

0.063 0.076 0.062 0.067 0.010 0.000 0.000 0.000 0.000 0.010

0.000 0.000 0.005 0.000 0.000 0.000 0.005 0.005 0.015 0.015

0.000 0.000 0.000 0.000 0.086 0.070 0.034 0.078 0.006 0.000

0.000 0.012 0.000 0.018 0.006 0.000 0.000 0.000 0.000 0.000

0.006 0.006 0.012 0.000 0.000 0.000 0.000 0.000 0.088 0.065

0.073 0.042

>Non-Methylation code ID 1261

0.010 0.000 0.005 0.000 0.005 0.000 0.005 0.000 0.005 0.010

0.000 0.000 0.015 0.005 0.005 0.000 0.000 0.000 0.000 0.000

0.070 0.073 0.052 0.073 0.000 0.000 0.000 0.000 0.006 0.006

0.000 0.006 0.000 0.011 0.000 0.011 0.011 0.000 0.006 0.006

0.006 0.000 0.000 0.006 0.068 0.069 0.073 0.053 0.007 0.007

0.000 0.000 0.000 0.000 0.000 0.007 0.000 0.000 0.000 0.000

0.007 0.007 0.007 0.014 0.014 0.007 0.000 0.000 0.063 0.066

0.062 0.066

>Non-Methylation code ID 1261

0.000 0.000 0.005 0.015 0.000 0.005 0.005 0.005 0.010 0.005

0.000 0.005 0.000 0.005 0.005 0.000 0.000 0.000 0.000 0.000

0.071 0.069 0.059 0.068 0.008 0.000 0.000 0.000 0.000 0.000

0.000 0.000 0.016 0.000 0.000 0.008 0.000 0.000 0.016 0.016

0.024 0.000 0.000 0.000 0.065 0.062 0.064 0.055 0.005 0.000

0.000 0.005 0.000 0.005 0.005 0.000 0.000 0.000 0.000 0.000

0.011 0.011 0.016 0.000 0.005 0.000 0.000 0.000 0.069 0.067

0.070 0.063

>Non-Methylation code ID 1261

0.005 0.000 0.005 0.000 0.005 0.005 0.000 0.000 0.005 0.005

0.000 0.000 0.005 0.005 0.010 0.005 0.000 0.005 0.000 0.000

0.074 0.058 0.057 0.085 0.010 0.005 0.000 0.000 0.005 0.010

0.005 0.000 0.005 0.005 0.005 0.000 0.000 0.000 0.005 0.000

0.000 0.000 0.000 0.005 0.056 0.077 0.066 0.074 0.000 0.007

0.000 0.007 0.000 0.014 0.000 0.000 0.007 0.007 0.000 0.000

0.000 0.007 0.000 0.007 0.014 0.007 0.007 0.000 0.066 0.068

0.042 0.073

>Non-Methylation code ID 1261

0.020 0.007 0.007 0.007 0.000 0.007 0.007 0.000 0.000 0.013

0.000 0.000 0.013 0.000 0.000 0.000 0.007 0.000 0.000 0.000

0.053 0.058 0.072 0.063 0.006 0.000 0.000 0.000 0.000 0.006

0.000 0.000 0.000 0.019 0.000 0.000 0.019 0.000 0.006 0.006

0.012 0.000 0.000 0.006 0.064 0.072 0.062 0.054 0.000 0.000

0.000 0.000 0.000 0.010 0.005 0.000 0.000 0.005 0.000 0.000

0.010 0.000 0.010 0.005 0.005 0.000 0.005 0.000 0.075 0.073

0.074 0.056

>Non-Methylation code ID 1261

0.006 0.000 0.000 0.000 0.000 0.006 0.012 0.000 0.000 0.012

0.000 0.000 0.006 0.006 0.012 0.012 0.000 0.006 0.000 0.000

0.057 0.072 0.052 0.075 0.017 0.000 0.000 0.000 0.006 0.011

0.000 0.000 0.000 0.000 0.000 0.000 0.011 0.000 0.006 0.006

0.011 0.000 0.006 0.000 0.076 0.062 0.066 0.055 0.014 0.000

0.000 0.000 0.000 0.014 0.000 0.000 0.000 0.007 0.000 0.000

0.014 0.021 0.007 0.007 0.000 0.000 0.000 0.000 0.050 0.053

0.070 0.076

>Non-Methylation code ID 1261

0.000 0.000 0.000 0.000 0.000 0.005 0.009 0.000 0.009 0.005

0.000 0.000 0.009 0.000 0.014 0.005 0.000 0.005 0.000 0.000

0.092 0.062 0.054 0.065 0.012 0.000 0.000 0.000 0.012 0.012

0.000 0.000 0.006 0.006 0.000 0.006 0.006 0.000 0.006 0.000

0.012 0.000 0.000 0.000 0.066 0.050 0.069 0.071 0.007 0.000

0.000 0.000 0.000 0.000 0.000 0.000 0.000 0.000 0.000 0.000

0.007 0.021 0.014 0.021 0.007 0.007 0.000 0.000 0.059 0.076

0.071 0.042

>Non-Methylation code ID 1261

0.009 0.000 0.000 0.004 0.000 0.009 0.009 0.000 0.004 0.000

0.000 0.000 0.013 0.000 0.009 0.000 0.000 0.000 0.000 0.000

0.076 0.065 0.050 0.085 0.011 0.000 0.000 0.005 0.000 0.000

0.005 0.000 0.000 0.005 0.000 0.005 0.011 0.000 0.011 0.000

0.011 0.005 0.000 0.000 0.052 0.064 0.059 0.090 0.000 0.000

0.000 0.005 0.000 0.005 0.000 0.000 0.000 0.000 0.000 0.000

0.014 0.000 0.019 0.005 0.009 0.000 0.000 0.000 0.059 0.073

0.060 0.085

>Non-Methylation code ID 1261

0.004 0.000 0.000 0.000 0.000 0.013 0.004 0.000 0.000 0.009

0.000 0.000 0.009 0.000 0.013 0.000 0.004 0.000 0.000 0.000

0.073 0.083 0.057 0.064 0.013 0.000 0.006 0.013 0.000 0.006

0.000 0.006 0.000 0.006 0.000 0.000 0.006 0.006 0.006 0.013

0.000 0.000 0.000 0.000 0.058 0.069 0.063 0.063 0.006 0.000

0.000 0.000 0.000 0.006 0.006 0.000 0.006 0.000 0.000 0.000

0.017 0.000 0.017 0.006 0.006 0.000 0.000 0.000 0.055 0.079

0.060 0.071

>Non-Methylation code ID 1261

0.022 0.000 0.000 0.006 0.006 0.006 0.000 0.000 0.000 0.000

0.000 0.006 0.011 0.006 0.006 0.006 0.000 0.000 0.000 0.000

0.065 0.052 0.065 0.079 0.005 0.000 0.000 0.000 0.000 0.005

0.000 0.000 0.000 0.000 0.000 0.000 0.020 0.010 0.015 0.005

0.005 0.000 0.000 0.000 0.064 0.070 0.069 0.065 0.005 0.000

0.000 0.005 0.000 0.010 0.000 0.000 0.000 0.010 0.000 0.000

0.005 0.005 0.014 0.005 0.000 0.000 0.000 0.000 0.059 0.091

0.066 0.061

>Non-Methylation code ID 1261

0.005 0.000 0.005 0.009 0.000 0.000 0.000 0.005 0.005 0.005

0.000 0.000 0.005 0.000 0.014 0.005 0.005 0.000 0.000 0.000

0.069 0.067 0.073 0.065 0.000 0.000 0.000 0.000 0.006 0.006

0.000 0.000 0.006 0.006 0.000 0.000 0.017 0.000 0.006 0.006

0.006 0.011 0.000 0.000 0.079 0.075 0.054 0.057 0.009 0.000

0.005 0.000 0.009 0.000 0.000 0.000 0.005 0.000 0.000 0.005

0.005 0.000 0.014 0.000 0.000 0.000 0.000 0.000 0.084 0.100

0.065 0.034

>Non-Methylation code ID 1261

0.021 0.000 0.000 0.005 0.000 0.005 0.000 0.005 0.000 0.005

0.000 0.000 0.005 0.005 0.011 0.000 0.005 0.000 0.000 0.000

0.061 0.060 0.073 0.071 0.005 0.005 0.000 0.000 0.000 0.009

0.000 0.000 0.005 0.009 0.000 0.000 0.014 0.000 0.009 0.005

0.000 0.000 0.000 0.000 0.058 0.061 0.073 0.083 0.005 0.000

0.000 0.000 0.000 0.016 0.000 0.000 0.000 0.000 0.000 0.005

0.011 0.005 0.011 0.005 0.000 0.000 0.000 0.005 0.072 0.064

0.069 0.063

>Non-Methylation code ID 1261

0.000 0.000 0.000 0.000 0.006 0.006 0.000 0.000 0.006 0.006

0.000 0.000 0.017 0.000 0.006 0.006 0.006 0.011 0.000 0.000

0.079 0.075 0.054 0.057 0.009 0.000 0.004 0.000 0.013 0.000

0.000 0.000 0.004 0.000 0.000 0.004 0.004 0.000 0.013 0.000

0.000 0.000 0.000 0.000 0.082 0.102 0.067 0.030 0.005 0.000

0.005 0.010 0.000 0.000 0.000 0.005 0.000 0.005 0.000 0.000

0.005 0.000 0.015 0.005 0.005 0.000 0.000 0.000 0.066 0.068

0.070 0.068

>Non-Methylation code ID 1261

0.009 0.000 0.000 0.004 0.000 0.009 0.009 0.000 0.004 0.000

0.000 0.000 0.009 0.000 0.009 0.004 0.000 0.000 0.000 0.000

0.068 0.070 0.054 0.085 0.014 0.000 0.000 0.007 0.000 0.000

0.000 0.000 0.000 0.000 0.000 0.007 0.034 0.000 0.007 0.000

0.014 0.007 0.000 0.000 0.052 0.069 0.054 0.070 0.000 0.000

0.000 0.005 0.000 0.000 0.000 0.000 0.000 0.005 0.000 0.000

0.019 0.000 0.023 0.005 0.000 0.000 0.000 0.000 0.068 0.079

0.047 0.083

>Non-Methylation code ID 1329

0.004 0.004 0.009 0.004 0.000 0.000 0.000 0.004 0.004 0.000

0.000 0.000 0.013 0.000 0.009 0.004 0.000 0.000 0.000 0.000

0.058 0.073 0.068 0.078 0.005 0.000 0.000 0.000 0.005 0.005

0.000 0.005 0.000 0.000 0.000 0.000 0.027 0.000 0.005 0.005

0.005 0.005 0.000 0.000 0.072 0.054 0.062 0.074 0.005 0.000

0.005 0.005 0.005 0.000 0.000 0.000 0.000 0.005 0.000 0.000

0.010 0.005 0.014 0.005 0.000 0.000 0.000 0.000 0.078 0.089

0.047 0.061

>Non-Methylation code ID 1329

0.009 0.000 0.000 0.000 0.005 0.014 0.000 0.000 0.000 0.000

0.000 0.000 0.005 0.005 0.014 0.009 0.000 0.000 0.000 0.000

0.078 0.051 0.078 0.067 0.020 0.000 0.000 0.000 0.000 0.029

0.000 0.000 0.000 0.010 0.000 0.000 0.039 0.000 0.000 0.020

0.000 0.000 0.000 0.000 0.042 0.064 0.057 0.053 0.008 0.000

0.000 0.000 0.004 0.004 0.000 0.000 0.000 0.008 0.000 0.000

0.008 0.000 0.013 0.000 0.000 0.000 0.004 0.000 0.081 0.054

0.085 0.064

>Non-Methylation code ID 1329

0.000 0.000 0.000 0.005 0.000 0.005 0.000 0.000 0.000 0.005

0.000 0.000 0.018 0.000 0.018 0.005 0.005 0.000 0.000 0.000

0.058 0.076 0.054 0.087 0.009 0.000 0.000 0.004 0.000 0.009

0.009 0.000 0.004 0.000 0.000 0.000 0.009 0.000 0.009 0.004

0.000 0.000 0.000 0.000 0.076 0.073 0.044 0.083 0.013 0.000

0.000 0.006 0.000 0.000 0.000 0.000 0.000 0.000 0.000 0.006

0.019 0.000 0.013 0.000 0.013 0.006 0.000 0.000 0.053 0.060

0.056 0.087

>Non-Methylation code ID 1329

0.009 0.000 0.000 0.000 0.000 0.009 0.000 0.000 0.000 0.000

0.000 0.005 0.014 0.005 0.009 0.005 0.000 0.000 0.000 0.005

0.061 0.052 0.085 0.073 0.016 0.000 0.000 0.000 0.000 0.005

0.000 0.005 0.000 0.005 0.000 0.000 0.005 0.016 0.011 0.000

0.005 0.000 0.000 0.000 0.073 0.054 0.070 0.066 0.000 0.005

0.000 0.000 0.000 0.010 0.000 0.000 0.010 0.005 0.000 0.000

0.021 0.000 0.005 0.005 0.000 0.000 0.000 0.000 0.080 0.051

0.067 0.075

>Non-Methylation code ID 1329

0.022 0.000 0.000 0.000 0.000 0.000 0.000 0.005 0.000 0.005

0.000 0.000 0.005 0.016 0.011 0.000 0.005 0.000 0.000 0.000

0.072 0.071 0.066 0.054 0.000 0.005 0.000 0.000 0.000 0.010

0.000 0.000 0.010 0.010 0.000 0.000 0.019 0.000 0.005 0.005

0.000 0.000 0.000 0.000 0.080 0.048 0.065 0.077 0.011 0.000

0.000 0.000 0.000 0.011 0.000 0.000 0.000 0.000 0.000 0.006

0.017 0.006 0.006 0.006 0.000 0.000 0.000 0.006 0.058 0.046

0.084 0.080

>Non-Methylation code ID 1329

0.010 0.000 0.005 0.005 0.000 0.010 0.000 0.000 0.005 0.005

0.000 0.000 0.000 0.000 0.010 0.010 0.000 0.000 0.000 0.000

0.063 0.073 0.065 0.070 0.010 0.000 0.005 0.000 0.000 0.010

0.000 0.000 0.000 0.000 0.000 0.000 0.010 0.005 0.015 0.000

0.005 0.000 0.000 0.000 0.092 0.071 0.058 0.053 0.005 0.000

0.000 0.009 0.000 0.009 0.000 0.000 0.000 0.009 0.000 0.000

0.005 0.005 0.014 0.000 0.000 0.000 0.000 0.000 0.076 0.063

0.066 0.074

>Non-Methylation code ID 1329

0.009 0.000 0.000 0.000 0.004 0.004 0.000 0.004 0.000 0.000

0.000 0.000 0.004 0.004 0.017 0.009 0.000 0.000 0.000 0.000

0.087 0.064 0.069 0.058 0.019 0.000 0.000 0.000 0.000 0.028

0.000 0.000 0.000 0.009 0.000 0.000 0.037 0.000 0.000 0.028

0.000 0.000 0.000 0.000 0.035 0.057 0.055 0.064 0.009 0.000

0.004 0.000 0.004 0.004 0.000 0.000 0.000 0.009 0.000 0.000

0.013 0.000 0.009 0.000 0.000 0.000 0.000 0.000 0.066 0.062

0.076 0.077

>Non-Methylation code ID 1329

0.000 0.006 0.000 0.000 0.000 0.006 0.006 0.000 0.000 0.006

0.000 0.000 0.000 0.012 0.012 0.019 0.000 0.006 0.000 0.000

0.054 0.072 0.061 0.072 0.011 0.005 0.011 0.000 0.000 0.000

0.000 0.000 0.000 0.000 0.000 0.005 0.011 0.000 0.005 0.011

0.005 0.005 0.000 0.000 0.057 0.069 0.050 0.087 0.000 0.000

0.000 0.006 0.000 0.000 0.000 0.006 0.000 0.006 0.000 0.000

0.017 0.000 0.012 0.006 0.012 0.000 0.000 0.000 0.074 0.075

0.044 0.077

>Non-Methylation code ID 1329

0.000 0.005 0.000 0.009 0.000 0.005 0.000 0.000 0.005 0.000

0.000 0.000 0.005 0.009 0.014 0.000 0.009 0.000 0.000 0.000

0.047 0.066 0.075 0.085 0.009 0.000 0.000 0.000 0.000 0.014

0.000 0.000 0.005 0.005 0.000 0.000 0.005 0.000 0.014 0.009

0.000 0.000 0.000 0.000 0.089 0.076 0.032 0.075 0.013 0.000

0.006 0.013 0.000 0.013 0.006 0.000 0.000 0.000 0.000 0.000

0.006 0.006 0.006 0.000 0.000 0.000 0.000 0.000 0.070 0.056

0.082 0.056

>Non-Methylation code ID 1329

0.009 0.000 0.000 0.000 0.000 0.014 0.005 0.000 0.000 0.009

0.000 0.000 0.005 0.000 0.014 0.000 0.005 0.000 0.000 0.000

0.074 0.096 0.045 0.059 0.012 0.000 0.006 0.012 0.000 0.006

0.006 0.006 0.000 0.000 0.000 0.000 0.006 0.006 0.006 0.012

0.000 0.000 0.000 0.000 0.057 0.059 0.068 0.070 0.005 0.000

0.000 0.000 0.000 0.005 0.005 0.000 0.005 0.000 0.000 0.000

0.011 0.000 0.016 0.005 0.011 0.000 0.000 0.000 0.054 0.082

0.064 0.068

>Non-Methylation code ID 1329

0.019 0.006 0.000 0.000 0.000 0.000 0.000 0.000 0.000 0.000

0.000 0.006 0.006 0.000 0.006 0.019 0.012 0.006 0.000 0.000

0.063 0.076 0.052 0.062 0.005 0.000 0.000 0.005 0.000 0.000

0.000 0.000 0.000 0.000 0.000 0.000 0.025 0.000 0.015 0.005

0.005 0.000 0.000 0.000 0.072 0.071 0.035 0.097 0.000 0.008

0.000 0.000 0.000 0.008 0.016 0.000 0.000 0.008 0.000 0.000

0.008 0.024 0.000 0.008 0.000 0.008 0.000 0.000 0.061 0.071

0.049 0.066

>Non-Methylation code ID 1329

0.011 0.000 0.005 0.005 0.000 0.005 0.000 0.005 0.000 0.005

0.000 0.000 0.011 0.000 0.011 0.011 0.000 0.000 0.000 0.000

0.066 0.076 0.071 0.050 0.011 0.000 0.000 0.000 0.005 0.005

0.005 0.000 0.000 0.000 0.000 0.000 0.022 0.000 0.011 0.005

0.005 0.000 0.000 0.000 0.074 0.074 0.049 0.065 0.005 0.000

0.000 0.000 0.000 0.011 0.005 0.000 0.000 0.011 0.000 0.000

0.011 0.000 0.011 0.005 0.005 0.000 0.000 0.000 0.071 0.069

0.070 0.060

>Non-Methylation code ID 1329

0.000 0.011 0.000 0.000 0.000 0.000 0.006 0.006 0.000 0.006

0.000 0.000 0.006 0.011 0.006 0.006 0.006 0.000 0.006 0.000

0.067 0.064 0.076 0.059 0.015 0.008 0.000 0.000 0.000 0.008

0.000 0.000 0.000 0.008 0.000 0.008 0.015 0.008 0.000 0.015

0.008 0.008 0.000 0.000 0.059 0.065 0.065 0.046 0.007 0.000

0.000 0.000 0.000 0.000 0.000 0.000 0.000 0.014 0.000 0.007

0.020 0.000 0.007 0.007 0.000 0.007 0.000 0.007 0.057 0.064

0.069 0.069

>Non-Methylation code ID 1329

0.010 0.005 0.010 0.005 0.000 0.000 0.000 0.000 0.005 0.000

0.000 0.000 0.020 0.000 0.005 0.005 0.000 0.000 0.000 0.000

0.047 0.077 0.074 0.068 0.006 0.000 0.000 0.000 0.000 0.006

0.000 0.006 0.000 0.006 0.000 0.000 0.030 0.000 0.012 0.006

0.006 0.000 0.000 0.000 0.070 0.053 0.059 0.072 0.005 0.000

0.000 0.005 0.000 0.005 0.000 0.000 0.000 0.010 0.000 0.000

0.005 0.005 0.015 0.010 0.000 0.000 0.000 0.000 0.052 0.074

0.079 0.070

>Non-Methylation code ID 1329

0.012 0.000 0.006 0.012 0.000 0.006 0.000 0.000 0.000 0.006

0.000 0.000 0.006 0.000 0.012 0.006 0.012 0.000 0.000 0.000

0.039 0.068 0.073 0.075 0.005 0.000 0.000 0.005 0.000 0.005

0.000 0.005 0.005 0.000 0.000 0.000 0.019 0.005 0.010 0.005

0.000 0.000 0.000 0.000 0.072 0.071 0.047 0.080 0.000 0.000

0.000 0.005 0.000 0.010 0.005 0.000 0.000 0.005 0.000 0.005

0.005 0.000 0.015 0.010 0.000 0.000 0.000 0.000 0.062 0.066

0.069 0.076

>Non-Methylation code ID 1329

0.005 0.000 0.000 0.000 0.005 0.005 0.010 0.000 0.005 0.005

0.000 0.000 0.010 0.000 0.010 0.005 0.000 0.005 0.000 0.000

0.080 0.072 0.038 0.080 0.006 0.000 0.000 0.000 0.012 0.006

0.000 0.000 0.000 0.012 0.000 0.006 0.006 0.000 0.006 0.006

0.012 0.006 0.000 0.000 0.065 0.062 0.073 0.059 0.006 0.000

0.000 0.000 0.000 0.000 0.000 0.000 0.000 0.000 0.000 0.000

0.006 0.013 0.013 0.019 0.006 0.006 0.006 0.000 0.078 0.051

0.070 0.056

>Non-Methylation code ID 1329

0.005 0.000 0.000 0.000 0.005 0.015 0.000 0.000 0.010 0.000

0.000 0.000 0.000 0.000 0.015 0.005 0.005 0.000 0.000 0.000

0.064 0.060 0.070 0.079 0.013 0.000 0.007 0.000 0.000 0.020

0.000 0.000 0.007 0.007 0.000 0.000 0.007 0.007 0.007 0.013

0.000 0.000 0.000 0.000 0.070 0.059 0.063 0.056 0.005 0.000

0.005 0.005 0.000 0.005 0.000 0.005 0.000 0.000 0.000 0.000

0.000 0.015 0.015 0.000 0.000 0.005 0.000 0.000 0.082 0.064

0.075 0.053

>Non-Methylation code ID 1329

0.026 0.000 0.007 0.000 0.000 0.000 0.007 0.000 0.000 0.000

0.000 0.000 0.020 0.000 0.007 0.020 0.000 0.000 0.000 0.000

0.060 0.053 0.069 0.066 0.010 0.000 0.000 0.000 0.000 0.000

0.000 0.000 0.000 0.019 0.000 0.000 0.048 0.010 0.000 0.000

0.019 0.019 0.000 0.000 0.051 0.047 0.055 0.056 0.000 0.005

0.000 0.000 0.000 0.000 0.000 0.000 0.005 0.005 0.000 0.000

0.014 0.000 0.018 0.005 0.000 0.000 0.000 0.000 0.077 0.077

0.041 0.087

>Non-Methylation code ID 1329

0.009 0.000 0.005 0.005 0.005 0.005 0.000 0.000 0.000 0.009

0.000 0.000 0.014 0.000 0.005 0.005 0.000 0.000 0.000 0.000

0.055 0.068 0.075 0.075 0.004 0.000 0.000 0.000 0.004 0.004

0.000 0.004 0.000 0.000 0.000 0.000 0.009 0.004 0.017 0.009

0.000 0.000 0.000 0.000 0.093 0.062 0.066 0.057 0.015 0.000

0.000 0.000 0.000 0.023 0.000 0.000 0.000 0.008 0.000 0.000

0.023 0.000 0.008 0.015 0.000 0.000 0.000 0.000 0.059 0.054

0.059 0.069

>Non-Methylation code ID 1329

0.009 0.000 0.000 0.004 0.000 0.009 0.009 0.000 0.004 0.000

0.000 0.000 0.009 0.000 0.009 0.004 0.000 0.000 0.000 0.000

0.073 0.070 0.051 0.084 0.012 0.000 0.000 0.006 0.000 0.000

0.000 0.000 0.000 0.000 0.000 0.006 0.025 0.000 0.012 0.000

0.012 0.006 0.000 0.000 0.051 0.063 0.057 0.081 0.000 0.000

0.000 0.005 0.000 0.000 0.000 0.000 0.000 0.005 0.000 0.000

0.020 0.000 0.020 0.005 0.005 0.000 0.000 0.000 0.064 0.081

0.046 0.083

>Non-Methylation code ID 1329

0.026 0.000 0.005 0.000 0.000 0.005 0.010 0.000 0.000 0.000

0.000 0.000 0.010 0.000 0.005 0.005 0.000 0.000 0.000 0.000

0.061 0.065 0.064 0.076 0.009 0.000 0.000 0.000 0.000 0.000

0.000 0.000 0.000 0.009 0.000 0.000 0.066 0.000 0.000 0.000

0.028 0.009 0.000 0.000 0.058 0.054 0.037 0.062 0.000 0.004

0.000 0.000 0.000 0.000 0.000 0.000 0.000 0.004 0.000 0.000

0.017 0.000 0.021 0.004 0.000 0.000 0.000 0.000 0.068 0.071

0.051 0.093

>Non-Methylation code ID 1329

0.006 0.000 0.000 0.000 0.000 0.000 0.000 0.000 0.000 0.000

0.000 0.000 0.006 0.019 0.013 0.019 0.006 0.013 0.000 0.000

0.066 0.064 0.068 0.052 0.005 0.000 0.000 0.000 0.005 0.000

0.009 0.000 0.005 0.005 0.000 0.000 0.009 0.000 0.014 0.005

0.000 0.005 0.000 0.000 0.084 0.063 0.047 0.082 0.007 0.000

0.000 0.000 0.013 0.013 0.000 0.000 0.000 0.007 0.000 0.007

0.007 0.000 0.007 0.007 0.013 0.000 0.000 0.000 0.074 0.051

0.071 0.059

>Non-Methylation code ID 1329

0.009 0.000 0.000 0.005 0.000 0.009 0.000 0.000 0.000 0.005

0.000 0.000 0.000 0.005 0.018 0.009 0.000 0.000 0.000 0.000

0.062 0.095 0.057 0.059 0.030 0.000 0.000 0.006 0.000 0.006

0.000 0.000 0.000 0.006 0.000 0.006 0.000 0.006 0.012 0.006

0.000 0.000 0.000 0.000 0.061 0.053 0.066 0.075 0.005 0.005

0.000 0.000 0.000 0.005 0.000 0.000 0.000 0.005 0.000 0.000

0.009 0.009 0.014 0.000 0.005 0.000 0.000 0.000 0.058 0.074

0.082 0.065

>Non-Methylation code ID 1329

0.006 0.000 0.006 0.011 0.000 0.006 0.000 0.000 0.000 0.006

0.000 0.000 0.011 0.000 0.011 0.006 0.011 0.000 0.000 0.000

0.051 0.075 0.066 0.068 0.005 0.000 0.000 0.005 0.000 0.005

0.000 0.005 0.005 0.000 0.000 0.000 0.014 0.005 0.014 0.005

0.000 0.000 0.000 0.000 0.073 0.075 0.044 0.079 0.005 0.000

0.000 0.005 0.000 0.005 0.005 0.000 0.000 0.005 0.000 0.005

0.005 0.000 0.014 0.010 0.000 0.000 0.000 0.000 0.061 0.074

0.070 0.071

>Non-Methylation code ID 1329

0.012 0.000 0.006 0.000 0.000 0.000 0.000 0.000 0.000 0.000

0.000 0.000 0.006 0.024 0.012 0.012 0.000 0.006 0.000 0.000

0.061 0.077 0.071 0.047 0.000 0.000 0.000 0.000 0.000 0.005

0.010 0.000 0.010 0.000 0.000 0.000 0.005 0.000 0.020 0.005

0.005 0.005 0.000 0.000 0.087 0.062 0.058 0.062 0.024 0.000

0.000 0.000 0.008 0.016 0.000 0.000 0.008 0.008 0.000 0.000

0.016 0.000 0.000 0.008 0.008 0.000 0.000 0.000 0.064 0.051

0.070 0.054

>Non-Methylation code ID 1329

0.000 0.005 0.010 0.005 0.000 0.000 0.000 0.005 0.005 0.000

0.000 0.000 0.019 0.000 0.005 0.010 0.000 0.000 0.000 0.000

0.051 0.075 0.074 0.070 0.006 0.000 0.000 0.000 0.006 0.006

0.000 0.006 0.000 0.006 0.000 0.000 0.030 0.000 0.006 0.006

0.006 0.000 0.000 0.000 0.078 0.065 0.042 0.069 0.005 0.000

0.005 0.005 0.005 0.000 0.000 0.000 0.000 0.005 0.000 0.000

0.005 0.005 0.015 0.010 0.000 0.000 0.000 0.000 0.071 0.083

0.055 0.066

>Non-Methylation code ID 1329

0.000 0.004 0.000 0.000 0.000 0.000 0.000 0.000 0.004 0.004

0.000 0.000 0.016 0.000 0.020 0.004 0.000 0.000 0.000 0.000

0.081 0.070 0.041 0.089 0.022 0.000 0.006 0.000 0.000 0.006

0.011 0.000 0.000 0.000 0.000 0.000 0.011 0.000 0.006 0.011

0.000 0.000 0.000 0.000 0.066 0.062 0.070 0.063 0.010 0.000

0.000 0.000 0.000 0.000 0.000 0.000 0.000 0.010 0.000 0.000

0.061 0.000 0.000 0.000 0.031 0.010 0.000 0.000 0.055 0.049

0.044 0.063

>Non-Methylation code ID 1329

0.022 0.000 0.000 0.000 0.006 0.006 0.000 0.000 0.000 0.000

0.000 0.006 0.011 0.006 0.006 0.011 0.000 0.000 0.000 0.000

0.057 0.051 0.077 0.076 0.005 0.000 0.000 0.000 0.000 0.005

0.000 0.000 0.000 0.005 0.000 0.000 0.022 0.011 0.011 0.005

0.005 0.000 0.000 0.000 0.069 0.059 0.069 0.067 0.005 0.000

0.000 0.005 0.000 0.005 0.000 0.000 0.000 0.010 0.000 0.000

0.005 0.005 0.015 0.010 0.000 0.000 0.000 0.000 0.074 0.078

0.065 0.054

>Non-Methylation code ID 1329

0.012 0.000 0.006 0.012 0.006 0.006 0.000 0.000 0.000 0.006

0.000 0.000 0.006 0.000 0.006 0.006 0.012 0.000 0.000 0.000

0.045 0.062 0.074 0.074 0.005 0.000 0.000 0.005 0.000 0.000

0.000 0.005 0.005 0.000 0.000 0.000 0.019 0.005 0.010 0.010

0.000 0.000 0.000 0.000 0.071 0.072 0.044 0.083 0.000 0.000

0.000 0.000 0.000 0.011 0.006 0.000 0.000 0.011 0.000 0.006

0.006 0.000 0.017 0.011 0.000 0.000 0.000 0.000 0.060 0.064

0.066 0.076

>Non-Methylation code ID 1329

0.006 0.000 0.006 0.006 0.000 0.011 0.000 0.000 0.006 0.006

0.000 0.000 0.000 0.000 0.011 0.017 0.000 0.000 0.000 0.000

0.056 0.067 0.072 0.070 0.011 0.000 0.000 0.000 0.000 0.011

0.000 0.000 0.000 0.000 0.000 0.000 0.016 0.000 0.016 0.000

0.005 0.000 0.000 0.000 0.097 0.079 0.044 0.056 0.004 0.000

0.000 0.009 0.000 0.004 0.000 0.000 0.000 0.009 0.000 0.000

0.004 0.004 0.013 0.000 0.000 0.000 0.004 0.000 0.077 0.077

0.058 0.068

>Non-Methylation code ID 1345

0.000 0.007 0.000 0.000 0.000 0.007 0.014 0.000 0.000 0.007

0.000 0.000 0.000 0.021 0.007 0.014 0.000 0.007 0.000 0.000

0.063 0.071 0.047 0.068 0.011 0.006 0.006 0.000 0.000 0.000

0.000 0.000 0.000 0.000 0.000 0.006 0.006 0.000 0.006 0.017

0.011 0.006 0.000 0.000 0.061 0.070 0.053 0.077 0.005 0.000

0.000 0.005 0.000 0.000 0.000 0.000 0.000 0.005 0.000 0.000

0.015 0.000 0.015 0.005 0.005 0.000 0.000 0.000 0.081 0.072

0.036 0.088

>Non-Methylation code ID 1345

0.011 0.005 0.011 0.000 0.000 0.000 0.000 0.000 0.000 0.000

0.000 0.000 0.016 0.000 0.011 0.011 0.005 0.000 0.000 0.000

0.056 0.076 0.063 0.070 0.006 0.000 0.000 0.006 0.000 0.006

0.000 0.006 0.000 0.006 0.000 0.000 0.029 0.000 0.012 0.000

0.006 0.000 0.000 0.000 0.077 0.064 0.045 0.073 0.000 0.000

0.000 0.000 0.000 0.006 0.000 0.000 0.000 0.011 0.000 0.000

0.006 0.011 0.011 0.017 0.000 0.006 0.000 0.000 0.043 0.074

0.078 0.070

>Non-Methylation code ID 1345

0.007 0.007 0.000 0.000 0.000 0.007 0.000 0.000 0.000 0.007

0.000 0.000 0.029 0.000 0.000 0.007 0.000 0.007 0.014 0.000

0.072 0.080 0.035 0.061 0.004 0.000 0.004 0.000 0.000 0.013

0.004 0.000 0.000 0.004 0.000 0.000 0.009 0.000 0.009 0.004

0.000 0.000 0.004 0.000 0.064 0.063 0.083 0.066 0.026 0.000

0.000 0.000 0.000 0.039 0.000 0.013 0.000 0.039 0.000 0.000

0.013 0.000 0.000 0.000 0.013 0.000 0.000 0.000 0.040 0.059

0.047 0.043

>Non-Methylation code ID 1345

0.006 0.000 0.000 0.000 0.000 0.011 0.011 0.006 0.006 0.006

0.000 0.000 0.011 0.000 0.000 0.000 0.000 0.000 0.006 0.006

0.071 0.064 0.073 0.057 0.017 0.000 0.000 0.000 0.000 0.008

0.000 0.008 0.000 0.008 0.000 0.000 0.008 0.008 0.008 0.017

0.017 0.000 0.000 0.000 0.075 0.057 0.040 0.060 0.000 0.005

0.005 0.005 0.000 0.005 0.000 0.000 0.000 0.009 0.000 0.000

0.014 0.000 0.009 0.005 0.000 0.000 0.000 0.000 0.046 0.073

0.078 0.082

>Non-Methylation code ID 1345

0.000 0.000 0.000 0.000 0.000 0.007 0.000 0.013 0.000 0.000

0.000 0.000 0.020 0.007 0.007 0.013 0.013 0.000 0.000 0.007

0.061 0.064 0.049 0.073 0.000 0.000 0.000 0.007 0.000 0.007

0.000 0.000 0.000 0.007 0.000 0.000 0.034 0.007 0.007 0.014

0.007 0.000 0.000 0.000 0.048 0.057 0.065 0.074 0.006 0.000

0.000 0.000 0.000 0.000 0.012 0.000 0.000 0.019 0.000 0.012

0.019 0.000 0.000 0.000 0.000 0.000 0.006 0.000 0.086 0.052

0.059 0.062

>Non-Methylation code ID 1345

0.028 0.000 0.000 0.000 0.000 0.057 0.000 0.014 0.000 0.071

0.000 0.000 0.000 0.000 0.000 0.014 0.000 0.000 0.000 0.000

0.039 0.042 0.032 0.037 0.010 0.010 0.000 0.005 0.000 0.005

0.005 0.000 0.000 0.010 0.000 0.000 0.005 0.000 0.000 0.005

0.000 0.000 0.010 0.000 0.064 0.081 0.068 0.055 0.006 0.000

0.006 0.000 0.000 0.012 0.000 0.000 0.000 0.006 0.000 0.000

0.012 0.000 0.006 0.006 0.006 0.006 0.006 0.000 0.061 0.060

0.066 0.074

>Non-Methylation code ID 1345

0.000 0.004 0.009 0.000 0.000 0.009 0.009 0.000 0.000 0.000

0.004 0.000 0.009 0.004 0.009 0.000 0.000 0.000 0.000 0.000

0.084 0.065 0.073 0.055 0.020 0.010 0.000 0.000 0.000 0.031

0.000 0.000 0.000 0.010 0.000 0.000 0.000 0.010 0.000 0.010

0.041 0.000 0.000 0.000 0.066 0.051 0.042 0.042 0.016 0.000

0.000 0.000 0.000 0.005 0.005 0.000 0.000 0.005 0.000 0.000

0.011 0.005 0.011 0.005 0.000 0.000 0.000 0.000 0.076 0.079

0.054 0.061

>Non-Methylation code ID 1345

0.000 0.000 0.000 0.000 0.000 0.006 0.000 0.000 0.000 0.013

0.000 0.000 0.025 0.000 0.006 0.006 0.000 0.006 0.006 0.000

0.069 0.074 0.065 0.057 0.004 0.004 0.009 0.000 0.000 0.004

0.000 0.000 0.000 0.004 0.000 0.000 0.009 0.004 0.009 0.004

0.000 0.000 0.000 0.000 0.058 0.068 0.078 0.076 0.018 0.000

0.000 0.009 0.000 0.009 0.000 0.009 0.000 0.009 0.000 0.000

0.018 0.000 0.000 0.009 0.009 0.000 0.009 0.000 0.050 0.065

0.044 0.073

>Non-Methylation code ID 1345

0.005 0.000 0.005 0.000 0.000 0.016 0.005 0.005 0.005 0.000

0.000 0.000 0.011 0.000 0.000 0.000 0.000 0.000 0.005 0.005

0.067 0.069 0.073 0.059 0.024 0.000 0.000 0.000 0.000 0.008

0.000 0.000 0.000 0.008 0.000 0.000 0.008 0.008 0.008 0.008

0.024 0.000 0.000 0.000 0.065 0.069 0.051 0.053 0.000 0.004

0.004 0.004 0.000 0.004 0.000 0.000 0.000 0.008 0.000 0.000

0.008 0.000 0.016 0.000 0.000 0.000 0.000 0.000 0.042 0.077

0.083 0.084

>Non-Methylation code ID 1345

0.041 0.000 0.000 0.000 0.000 0.041 0.000 0.014 0.000 0.068

0.000 0.000 0.000 0.000 0.000 0.014 0.000 0.000 0.000 0.000

0.043 0.043 0.027 0.043 0.005 0.011 0.000 0.000 0.000 0.011

0.005 0.000 0.000 0.011 0.000 0.000 0.011 0.000 0.000 0.005

0.000 0.000 0.011 0.000 0.062 0.080 0.057 0.066 0.007 0.000

0.007 0.000 0.000 0.020 0.000 0.000 0.000 0.007 0.000 0.000

0.013 0.000 0.000 0.000 0.007 0.007 0.013 0.000 0.077 0.055

0.057 0.065

>Non-Methylation code ID 1345

0.006 0.000 0.006 0.000 0.000 0.019 0.000 0.000 0.000 0.006

0.000 0.000 0.012 0.000 0.000 0.006 0.006 0.006 0.012 0.000

0.073 0.052 0.063 0.065 0.041 0.000 0.000 0.000 0.000 0.041

0.000 0.014 0.000 0.068 0.000 0.000 0.000 0.000 0.000 0.014

0.000 0.000 0.000 0.000 0.043 0.043 0.027 0.043 0.005 0.011

0.000 0.000 0.000 0.011 0.005 0.000 0.000 0.011 0.000 0.000

0.005 0.000 0.000 0.005 0.000 0.000 0.011 0.000 0.061 0.080

0.059 0.068

>Non-Methylation code ID 1345

0.006 0.000 0.000 0.000 0.000 0.017 0.006 0.006 0.006 0.006

0.000 0.000 0.012 0.000 0.000 0.000 0.000 0.000 0.006 0.006

0.068 0.067 0.070 0.058 0.025 0.000 0.000 0.000 0.000 0.008

0.000 0.000 0.000 0.008 0.000 0.000 0.008 0.008 0.008 0.016

0.016 0.000 0.000 0.000 0.071 0.073 0.037 0.054 0.000 0.004

0.004 0.004 0.000 0.004 0.000 0.000 0.000 0.008 0.000 0.000

0.012 0.000 0.012 0.000 0.000 0.000 0.000 0.000 0.046 0.072

0.088 0.079

>Non-Methylation code ID 1345

0.000 0.000 0.006 0.000 0.006 0.011 0.006 0.006 0.006 0.000

0.000 0.006 0.000 0.000 0.006 0.000 0.000 0.000 0.000 0.006

0.081 0.069 0.072 0.055 0.007 0.007 0.000 0.000 0.000 0.013

0.000 0.000 0.000 0.000 0.007 0.000 0.000 0.000 0.007 0.013

0.020 0.000 0.007 0.000 0.052 0.061 0.065 0.075 0.004 0.000

0.004 0.004 0.000 0.000 0.000 0.000 0.000 0.000 0.000 0.000

0.004 0.004 0.018 0.000 0.000 0.009 0.004 0.000 0.050 0.065

0.084 0.082

>Non-Methylation code ID 1345

0.004 0.004 0.009 0.000 0.000 0.004 0.000 0.000 0.000 0.004

0.000 0.000 0.009 0.004 0.009 0.004 0.000 0.000 0.000 0.000

0.058 0.068 0.078 0.076 0.016 0.000 0.000 0.008 0.000 0.008

0.000 0.008 0.000 0.008 0.000 0.000 0.024 0.000 0.000 0.008

0.008 0.000 0.008 0.000 0.058 0.062 0.047 0.070 0.000 0.000

0.000 0.000 0.000 0.007 0.000 0.000 0.000 0.013 0.000 0.000

0.020 0.000 0.007 0.007 0.000 0.007 0.007 0.000 0.068 0.081

0.072 0.045

>Non-Methylation code ID 1345

0.010 0.000 0.000 0.000 0.000 0.010 0.005 0.000 0.000 0.005

0.000 0.000 0.010 0.005 0.005 0.005 0.000 0.000 0.010 0.000

0.078 0.064 0.067 0.059 0.000 0.004 0.009 0.000 0.000 0.013

0.009 0.000 0.000 0.004 0.004 0.000 0.004 0.000 0.009 0.000

0.000 0.000 0.000 0.000 0.085 0.055 0.078 0.058 0.029 0.010

0.000 0.000 0.000 0.029 0.000 0.000 0.000 0.019 0.000 0.000

0.000 0.000 0.000 0.000 0.029 0.000 0.000 0.000 0.058 0.051

0.053 0.058

>Non-Methylation code ID 1345

0.005 0.005 0.005 0.000 0.000 0.011 0.005 0.000 0.000 0.000

0.005 0.000 0.005 0.011 0.005 0.005 0.000 0.000 0.005 0.000

0.080 0.066 0.056 0.063 0.013 0.000 0.000 0.000 0.000 0.020

0.000 0.000 0.000 0.000 0.000 0.000 0.013 0.007 0.007 0.007

0.013 0.000 0.007 0.000 0.066 0.058 0.061 0.063 0.006 0.000

0.000 0.000 0.000 0.018 0.006 0.000 0.000 0.006 0.000 0.000

0.018 0.006 0.006 0.000 0.000 0.006 0.000 0.000 0.072 0.080

0.046 0.063

>Non-Methylation code ID 1345

0.015 0.000 0.000 0.007 0.000 0.007 0.000 0.007 0.000 0.000

0.000 0.000 0.022 0.000 0.000 0.007 0.007 0.000 0.015 0.000

0.067 0.050 0.050 0.078 0.000 0.000 0.000 0.000 0.000 0.011

0.000 0.000 0.000 0.017 0.000 0.000 0.017 0.000 0.006 0.006

0.000 0.006 0.006 0.000 0.061 0.066 0.077 0.061 0.005 0.005

0.005 0.000 0.000 0.005 0.000 0.000 0.000 0.010 0.000 0.000

0.005 0.005 0.010 0.005 0.000 0.000 0.000 0.000 0.060 0.070

0.076 0.070

>Non-Methylation code ID 1345

0.000 0.004 0.004 0.000 0.000 0.009 0.009 0.000 0.000 0.000

0.004 0.000 0.009 0.004 0.009 0.004 0.000 0.000 0.000 0.000

0.083 0.060 0.076 0.056 0.019 0.010 0.000 0.000 0.000 0.029

0.000 0.000 0.000 0.010 0.000 0.000 0.010 0.010 0.000 0.010

0.029 0.000 0.000 0.000 0.058 0.052 0.047 0.051 0.016 0.000

0.000 0.000 0.000 0.005 0.000 0.000 0.000 0.005 0.000 0.000

0.011 0.005 0.011 0.005 0.000 0.005 0.000 0.000 0.070 0.089

0.054 0.055

>Non-Methylation code ID 1345

0.000 0.005 0.000 0.005 0.000 0.014 0.000 0.000 0.000 0.014

0.000 0.000 0.000 0.000 0.009 0.009 0.005 0.000 0.000 0.000

0.067 0.054 0.074 0.076 0.025 0.000 0.000 0.008 0.008 0.016

0.008 0.000 0.000 0.000 0.000 0.000 0.008 0.000 0.000 0.025

0.000 0.000 0.000 0.000 0.065 0.053 0.054 0.063 0.000 0.000

0.008 0.000 0.000 0.008 0.000 0.008 0.000 0.000 0.000 0.000

0.032 0.008 0.008 0.008 0.000 0.000 0.000 0.000 0.071 0.069

0.049 0.063

>Non-Methylation code ID 1345

0.006 0.000 0.000 0.000 0.000 0.012 0.012 0.000 0.000 0.018

0.000 0.006 0.012 0.000 0.000 0.000 0.000 0.000 0.006 0.006

0.077 0.056 0.078 0.046 0.016 0.000 0.000 0.000 0.000 0.008

0.000 0.016 0.000 0.000 0.000 0.000 0.016 0.008 0.000 0.016

0.016 0.000 0.000 0.008 0.055 0.059 0.049 0.066 0.000 0.000

0.000 0.000 0.000 0.006 0.000 0.000 0.000 0.012 0.000 0.000

0.018 0.006 0.012 0.012 0.006 0.000 0.000 0.000 0.052 0.079

0.066 0.065

>Non-Methylation code ID 1345

0.017 0.006 0.000 0.000 0.000 0.011 0.000 0.000 0.000 0.000

0.000 0.006 0.000 0.000 0.000 0.006 0.017 0.000 0.011 0.000

0.058 0.075 0.076 0.050 0.007 0.000 0.000 0.000 0.000 0.022

0.000 0.000 0.000 0.022 0.000 0.000 0.014 0.007 0.007 0.000

0.007 0.007 0.000 0.000 0.068 0.082 0.039 0.050 0.000 0.000

0.000 0.000 0.006 0.011 0.011 0.000 0.006 0.006 0.000 0.000

0.000 0.000 0.006 0.000 0.000 0.000 0.006 0.006 0.065 0.066

0.072 0.074

>Non-Methylation code ID 1345

0.021 0.000 0.000 0.000 0.000 0.032 0.000 0.000 0.000 0.011

0.000 0.000 0.021 0.011 0.000 0.011 0.032 0.000 0.000 0.000

0.059 0.039 0.044 0.054 0.005 0.000 0.000 0.000 0.000 0.010

0.000 0.000 0.000 0.010 0.000 0.000 0.016 0.005 0.010 0.005

0.000 0.005 0.000 0.000 0.071 0.073 0.050 0.072 0.005 0.005

0.005 0.000 0.000 0.010 0.010 0.000 0.000 0.000 0.000 0.000

0.005 0.005 0.005 0.005 0.000 0.000 0.005 0.000 0.079 0.057

0.074 0.063

>Non-Methylation code ID 1345

0.006 0.000 0.000 0.000 0.000 0.011 0.017 0.000 0.000 0.011

0.000 0.006 0.011 0.000 0.000 0.000 0.000 0.000 0.006 0.006

0.070 0.057 0.077 0.055 0.017 0.000 0.000 0.000 0.000 0.008

0.000 0.017 0.000 0.000 0.000 0.000 0.017 0.008 0.000 0.008

0.025 0.000 0.000 0.008 0.057 0.057 0.050 0.061 0.000 0.000

0.000 0.000 0.000 0.006 0.000 0.000 0.000 0.011 0.000 0.000

0.017 0.006 0.011 0.011 0.006 0.000 0.000 0.000 0.053 0.082

0.072 0.059

>Non-Methylation code ID 1345

0.007 0.007 0.000 0.007 0.000 0.014 0.000 0.000 0.000 0.014

0.000 0.000 0.000 0.000 0.007 0.028 0.007 0.000 0.000 0.000

0.052 0.063 0.070 0.057 0.025 0.000 0.000 0.000 0.008 0.008

0.008 0.000 0.000 0.025 0.000 0.000 0.008 0.000 0.000 0.017

0.000 0.000 0.000 0.000 0.059 0.047 0.058 0.070 0.000 0.007

0.007 0.000 0.000 0.000 0.000 0.007 0.000 0.007 0.000 0.000

0.013 0.007 0.000 0.007 0.000 0.000 0.013 0.000 0.077 0.062

0.051 0.077

>Non-Methylation code ID 1345

0.027 0.009 0.000 0.000 0.000 0.027 0.000 0.000 0.000 0.018

0.000 0.000 0.000 0.000 0.000 0.000 0.036 0.000 0.000 0.000

0.058 0.051 0.049 0.058 0.011 0.000 0.000 0.000 0.000 0.011

0.005 0.000 0.000 0.005 0.000 0.000 0.016 0.005 0.005 0.005

0.000 0.000 0.005 0.000 0.076 0.073 0.063 0.051 0.000 0.005

0.009 0.000 0.000 0.009 0.009 0.000 0.000 0.005 0.005 0.000

0.005 0.000 0.009 0.000 0.000 0.000 0.000 0.000 0.087 0.058

0.076 0.058

>Non-Methylation code ID 1345

0.025 0.000 0.000 0.008 0.008 0.016 0.008 0.000 0.000 0.000

0.000 0.000 0.008 0.000 0.000 0.025 0.000 0.000 0.000 0.000

0.065 0.053 0.054 0.063 0.000 0.000 0.007 0.000 0.000 0.007

0.000 0.007 0.000 0.000 0.000 0.000 0.029 0.007 0.007 0.015

0.000 0.000 0.000 0.000 0.076 0.063 0.051 0.063 0.000 0.005

0.000 0.005 0.000 0.010 0.000 0.000 0.000 0.015 0.000 0.000

0.000 0.000 0.010 0.010 0.005 0.000 0.000 0.000 0.068 0.056

0.077 0.074

>Non-Methylation code ID 1376

0.005 0.000 0.000 0.000 0.000 0.010 0.000 0.000 0.000 0.010

0.000 0.000 0.016 0.005 0.010 0.005 0.000 0.005 0.000 0.000

0.071 0.073 0.050 0.072 0.005 0.005 0.005 0.000 0.000 0.009

0.014 0.000 0.000 0.000 0.000 0.000 0.005 0.005 0.005 0.005

0.000 0.000 0.005 0.000 0.081 0.060 0.072 0.060 0.022 0.000

0.000 0.000 0.000 0.034 0.000 0.000 0.000 0.000 0.000 0.000

0.022 0.011 0.000 0.011 0.034 0.000 0.000 0.000 0.059 0.039

0.044 0.056

>Non-Methylation code ID 1376

0.024 0.012 0.000 0.000 0.000 0.061 0.000 0.000 0.000 0.000

0.012 0.000 0.000 0.000 0.000 0.024 0.012 0.000 0.000 0.000

0.041 0.052 0.046 0.048 0.029 0.000 0.000 0.000 0.000 0.014

0.000 0.000 0.000 0.000 0.000 0.000 0.014 0.000 0.007 0.000

0.000 0.000 0.007 0.000 0.078 0.073 0.046 0.064 0.000 0.000

0.005 0.005 0.000 0.009 0.005 0.000 0.000 0.000 0.000 0.000

0.000 0.005 0.018 0.000 0.000 0.000 0.009 0.000 0.081 0.068

0.067 0.063

>Non-Methylation code ID 1376

0.023 0.011 0.000 0.000 0.000 0.057 0.000 0.000 0.000 0.000

0.011 0.000 0.000 0.000 0.000 0.023 0.011 0.000 0.000 0.000

0.056 0.049 0.042 0.049 0.037 0.000 0.000 0.000 0.000 0.007

0.000 0.000 0.000 0.000 0.000 0.000 0.015 0.000 0.007 0.000

0.000 0.000 0.007 0.000 0.081 0.067 0.053 0.058 0.000 0.000

0.004 0.004 0.000 0.009 0.004 0.000 0.000 0.004 0.000 0.000

0.000 0.004 0.018 0.000 0.000 0.000 0.004 0.000 0.091 0.056

0.070 0.063

>Non-Methylation code ID 1376

0.007 0.007 0.000 0.000 0.004 0.000 0.004 0.000 0.000 0.004

0.000 0.000 0.004 0.000 0.011 0.004 0.000 0.000 0.004 0.000

0.081 0.088 0.044 0.073 0.027 0.000 0.000 0.009 0.000 0.027

0.009 0.000 0.000 0.009 0.000 0.000 0.009 0.000 0.000 0.018

0.009 0.000 0.000 0.000 0.048 0.065 0.062 0.041 0.000 0.000

0.000 0.000 0.000 0.017 0.000 0.000 0.008 0.025 0.000 0.000

0.017 0.017 0.000 0.000 0.008 0.000 0.000 0.000 0.053 0.063

0.053 0.072

>Non-Methylation code ID 1376

0.016 0.000 0.000 0.000 0.000 0.033 0.000 0.000 0.000 0.000

0.000 0.000 0.008 0.000 0.008 0.033 0.000 0.000 0.000 0.000

0.054 0.046 0.058 0.077 0.019 0.000 0.000 0.005 0.000 0.010

0.005 0.000 0.000 0.005 0.000 0.000 0.005 0.000 0.010 0.000

0.000 0.005 0.000 0.000 0.072 0.047 0.065 0.087 0.000 0.000

0.000 0.000 0.000 0.023 0.000 0.000 0.000 0.008 0.000 0.000

0.000 0.023 0.008 0.000 0.008 0.008 0.008 0.000 0.059 0.050

0.074 0.066

>Non-Methylation code ID 1376

0.008 0.004 0.000 0.000 0.004 0.000 0.000 0.000 0.000 0.004

0.000 0.000 0.004 0.000 0.011 0.008 0.004 0.000 0.004 0.000

0.068 0.071 0.059 0.085 0.011 0.000 0.000 0.005 0.000 0.016

0.000 0.000 0.000 0.005 0.000 0.000 0.005 0.000 0.016 0.011

0.000 0.000 0.000 0.000 0.049 0.066 0.082 0.066 0.000 0.000

0.008 0.008 0.000 0.016 0.000 0.000 0.008 0.025 0.000 0.000

0.000 0.016 0.000 0.008 0.000 0.008 0.000 0.000 0.065 0.049

0.051 0.070

>Non-Methylation code ID 1376

0.006 0.006 0.000 0.000 0.000 0.006 0.025 0.000 0.000 0.000

0.000 0.000 0.013 0.006 0.000 0.013 0.000 0.006 0.000 0.000

0.079 0.048 0.063 0.061 0.014 0.000 0.000 0.000 0.000 0.000

0.007 0.000 0.000 0.007 0.007 0.000 0.014 0.000 0.007 0.014

0.022 0.000 0.000 0.000 0.069 0.059 0.055 0.057 0.007 0.007

0.000 0.000 0.000 0.000 0.000 0.000 0.000 0.020 0.000 0.000

0.027 0.000 0.007 0.007 0.007 0.000 0.000 0.000 0.061 0.064

0.062 0.064

>Non-Methylation code ID 1376

0.016 0.008 0.000 0.016 0.000 0.016 0.000 0.000 0.000 0.016

0.000 0.000 0.008 0.000 0.000 0.024 0.000 0.000 0.000 0.000

0.056 0.049 0.062 0.062 0.011 0.000 0.000 0.000 0.000 0.006

0.000 0.000 0.000 0.000 0.000 0.006 0.028 0.000 0.006 0.006

0.000 0.000 0.006 0.000 0.083 0.051 0.057 0.077 0.005 0.000

0.000 0.000 0.000 0.010 0.000 0.000 0.000 0.010 0.000 0.000

0.005 0.000 0.015 0.005 0.005 0.005 0.000 0.000 0.074 0.048

0.072 0.081

>Non-Methylation code ID 1376

0.005 0.000 0.000 0.000 0.000 0.009 0.000 0.000 0.000 0.023

0.000 0.000 0.000 0.000 0.014 0.000 0.005 0.005 0.000 0.000

0.070 0.049 0.078 0.076 0.007 0.007 0.000 0.015 0.000 0.015

0.000 0.000 0.000 0.000 0.000 0.000 0.007 0.007 0.000 0.037

0.000 0.000 0.000 0.000 0.058 0.049 0.060 0.071 0.011 0.000

0.000 0.000 0.000 0.006 0.006 0.000 0.000 0.000 0.000 0.000

0.028 0.000 0.011 0.006 0.000 0.000 0.000 0.000 0.070 0.065

0.058 0.073

>Non-Methylation code ID 1376

0.020 0.000 0.000 0.010 0.000 0.010 0.000 0.000 0.000 0.020

0.000 0.000 0.020 0.000 0.000 0.030 0.010 0.000 0.000 0.000

0.043 0.051 0.056 0.060 0.004 0.004 0.000 0.000 0.000 0.000

0.004 0.000 0.000 0.004 0.000 0.004 0.018 0.000 0.009 0.000

0.000 0.000 0.004 0.000 0.088 0.067 0.053 0.072 0.007 0.000

0.000 0.000 0.000 0.020 0.007 0.000 0.000 0.013 0.000 0.000

0.007 0.000 0.007 0.007 0.013 0.000 0.000 0.000 0.061 0.068

0.076 0.049

>Non-Methylation code ID 1376

0.013 0.006 0.000 0.000 0.000 0.000 0.006 0.000 0.000 0.013

0.000 0.000 0.025 0.000 0.006 0.006 0.006 0.000 0.000 0.000

0.055 0.069 0.057 0.070 0.006 0.006 0.000 0.000 0.000 0.006

0.026 0.000 0.000 0.006 0.000 0.000 0.013 0.006 0.000 0.006

0.006 0.000 0.000 0.000 0.080 0.046 0.067 0.057 0.007 0.000

0.000 0.000 0.000 0.000 0.007 0.000 0.000 0.000 0.007 0.000

0.015 0.007 0.007 0.015 0.022 0.000 0.000 0.000 0.066 0.065

0.052 0.062

>Non-Methylation code ID 1376

0.000 0.004 0.000 0.000 0.000 0.009 0.009 0.000 0.000 0.000

0.000 0.000 0.009 0.000 0.013 0.013 0.000 0.000 0.000 0.000

0.072 0.097 0.058 0.049 0.036 0.000 0.000 0.000 0.000 0.029

0.000 0.000 0.000 0.000 0.000 0.000 0.007 0.000 0.007 0.000

0.014 0.000 0.000 0.000 0.059 0.051 0.063 0.066 0.016 0.000

0.000 0.016 0.000 0.008 0.000 0.000 0.000 0.008 0.000 0.000

0.031 0.016 0.000 0.000 0.000 0.000 0.000 0.000 0.055 0.067

0.064 0.054

>Non-Methylation code ID 1376

0.000 0.000 0.000 0.000 0.000 0.004 0.000 0.004 0.000 0.004

0.000 0.000 0.016 0.000 0.020 0.000 0.000 0.000 0.000 0.000

0.080 0.064 0.062 0.079 0.009 0.000 0.000 0.000 0.005 0.018

0.000 0.000 0.005 0.000 0.000 0.000 0.005 0.005 0.009 0.000

0.000 0.000 0.000 0.005 0.083 0.051 0.079 0.062 0.042 0.000

0.000 0.000 0.000 0.008 0.000 0.008 0.008 0.000 0.000 0.008

0.008 0.008 0.000 0.008 0.000 0.000 0.000 0.000 0.062 0.055

0.047 0.068

>Non-Methylation code ID 1376

0.015 0.000 0.000 0.000 0.000 0.005 0.005 0.000 0.000 0.000

0.000 0.000 0.025 0.000 0.010 0.005 0.000 0.000 0.000 0.000

0.071 0.073 0.055 0.068 0.005 0.000 0.000 0.000 0.000 0.009

0.000 0.000 0.000 0.023 0.000 0.000 0.000 0.000 0.014 0.000

0.005 0.005 0.000 0.000 0.070 0.049 0.078 0.076 0.008 0.008

0.000 0.008 0.000 0.016 0.000 0.000 0.000 0.000 0.000 0.000

0.008 0.008 0.000 0.040 0.000 0.000 0.000 0.000 0.058 0.045

0.061 0.073

>Non-Methylation code ID 1376

0.013 0.000 0.000 0.000 0.000 0.000 0.007 0.000 0.000 0.000

0.007 0.000 0.013 0.007 0.007 0.013 0.020 0.000 0.000 0.000

0.064 0.060 0.048 0.077 0.012 0.006 0.000 0.000 0.000 0.000

0.006 0.000 0.000 0.012 0.000 0.000 0.031 0.000 0.006 0.000

0.006 0.000 0.000 0.000 0.060 0.070 0.056 0.066 0.007 0.007

0.000 0.000 0.000 0.007 0.021 0.000 0.000 0.007 0.000 0.000

0.014 0.007 0.000 0.007 0.007 0.000 0.000 0.000 0.076 0.050

0.061 0.063

>Non-Methylation code ID 1376

0.010 0.000 0.000 0.000 0.000 0.049 0.000 0.000 0.000 0.000

0.000 0.000 0.000 0.000 0.010 0.049 0.000 0.000 0.000 0.000

0.055 0.050 0.061 0.049 0.059 0.000 0.000 0.000 0.000 0.007

0.000 0.000 0.000 0.007 0.000 0.000 0.000 0.000 0.015 0.000

0.000 0.007 0.000 0.000 0.080 0.026 0.073 0.058 0.000 0.000

0.000 0.000 0.000 0.018 0.000 0.000 0.000 0.009 0.000 0.000

0.000 0.036 0.027 0.000 0.000 0.009 0.000 0.000 0.046 0.048

0.051 0.091

>Non-Methylation code ID 1376

0.000 0.000 0.000 0.000 0.000 0.015 0.000 0.000 0.000 0.010

0.000 0.000 0.005 0.000 0.010 0.005 0.005 0.005 0.010 0.000

0.084 0.062 0.072 0.051 0.038 0.000 0.000 0.000 0.000 0.051

0.000 0.000 0.000 0.000 0.000 0.000 0.026 0.000 0.000 0.026

0.000 0.013 0.000 0.000 0.052 0.047 0.030 0.052 0.011 0.000

0.000 0.005 0.000 0.005 0.005 0.000 0.000 0.011 0.000 0.000

0.011 0.000 0.011 0.005 0.000 0.000 0.000 0.000 0.077 0.048

0.064 0.080

>Non-Methylation code ID 1376

0.010 0.000 0.000 0.000 0.000 0.019 0.000 0.000 0.000 0.005

0.000 0.000 0.005 0.000 0.014 0.005 0.005 0.000 0.000 0.000

0.040 0.061 0.090 0.080 0.000 0.000 0.009 0.026 0.000 0.017

0.000 0.000 0.000 0.026 0.000 0.000 0.000 0.017 0.000 0.009

0.000 0.009 0.000 0.000 0.056 0.053 0.045 0.069 0.009 0.004

0.000 0.000 0.004 0.000 0.000 0.000 0.000 0.000 0.000 0.004

0.004 0.000 0.013 0.004 0.004 0.000 0.004 0.000 0.060 0.060

0.075 0.085

>Non-Methylation code ID 1376

0.007 0.000 0.000 0.000 0.000 0.000 0.007 0.000 0.000 0.007

0.007 0.000 0.014 0.007 0.007 0.014 0.021 0.000 0.000 0.000

0.063 0.068 0.051 0.060 0.013 0.006 0.000 0.000 0.000 0.000

0.006 0.000 0.000 0.013 0.000 0.000 0.025 0.000 0.006 0.006

0.006 0.000 0.000 0.000 0.055 0.069 0.057 0.070 0.007 0.007

0.000 0.000 0.000 0.000 0.027 0.000 0.000 0.007 0.000 0.000

0.014 0.007 0.000 0.007 0.007 0.000 0.000 0.000 0.083 0.047

0.066 0.055

>Non-Methylation code ID 1376

0.016 0.000 0.000 0.000 0.005 0.016 0.000 0.000 0.005 0.000

0.000 0.000 0.000 0.005 0.011 0.005 0.000 0.000 0.000 0.005

0.063 0.067 0.078 0.057 0.023 0.000 0.006 0.000 0.000 0.000

0.000 0.006 0.006 0.000 0.000 0.006 0.006 0.012 0.006 0.006

0.000 0.000 0.000 0.000 0.065 0.081 0.039 0.073 0.000 0.000

0.000 0.000 0.000 0.005 0.000 0.009 0.000 0.005 0.000 0.000

0.009 0.000 0.018 0.005 0.000 0.000 0.000 0.000 0.075 0.074

0.074 0.059

>Non-Methylation code ID 1437

0.016 0.000 0.000 0.000 0.000 0.011 0.005 0.000 0.000 0.000

0.000 0.000 0.011 0.005 0.011 0.011 0.000 0.000 0.000 0.000

0.058 0.076 0.053 0.076 0.017 0.000 0.000 0.000 0.000 0.017

0.000 0.000 0.000 0.000 0.000 0.000 0.017 0.000 0.017 0.000

0.006 0.000 0.000 0.000 0.086 0.042 0.069 0.065 0.006 0.000

0.000 0.006 0.000 0.017 0.000 0.000 0.000 0.000 0.000 0.000

0.017 0.011 0.011 0.000 0.000 0.000 0.000 0.000 0.082 0.043

0.081 0.061

>Non-Methylation code ID 1437

0.004 0.007 0.000 0.000 0.004 0.000 0.004 0.000 0.000 0.004

0.000 0.000 0.007 0.000 0.011 0.004 0.000 0.000 0.004 0.000

0.086 0.082 0.049 0.070 0.025 0.000 0.000 0.008 0.000 0.025

0.008 0.000 0.000 0.000 0.000 0.000 0.017 0.000 0.000 0.017

0.008 0.000 0.000 0.000 0.058 0.065 0.058 0.043 0.008 0.000

0.000 0.000 0.000 0.008 0.000 0.000 0.008 0.024 0.000 0.000

0.016 0.016 0.000 0.000 0.008 0.000 0.000 0.000 0.052 0.060

0.057 0.077

>Non-Methylation code ID 1437

0.006 0.000 0.000 0.000 0.000 0.006 0.006 0.000 0.000 0.017

0.000 0.000 0.011 0.006 0.011 0.000 0.006 0.006 0.000 0.000

0.089 0.034 0.083 0.054 0.006 0.006 0.000 0.006 0.000 0.006

0.000 0.000 0.000 0.000 0.000 0.000 0.013 0.006 0.006 0.025

0.006 0.000 0.000 0.000 0.070 0.059 0.050 0.073 0.017 0.000

0.000 0.000 0.000 0.006 0.006 0.000 0.000 0.000 0.000 0.000

0.028 0.000 0.006 0.006 0.000 0.000 0.000 0.000 0.047 0.070

0.070 0.078

>Non-Methylation code ID 1437

0.007 0.007 0.000 0.000 0.000 0.007 0.020 0.000 0.000 0.013

0.000 0.000 0.013 0.007 0.000 0.007 0.007 0.000 0.000 0.000

0.075 0.050 0.057 0.064 0.013 0.000 0.000 0.000 0.000 0.000

0.007 0.000 0.000 0.000 0.000 0.000 0.013 0.007 0.007 0.020

0.020 0.000 0.000 0.000 0.061 0.062 0.045 0.078 0.013 0.000

0.000 0.000 0.000 0.000 0.007 0.000 0.000 0.013 0.000 0.000

0.033 0.000 0.007 0.000 0.007 0.000 0.000 0.000 0.064 0.067

0.061 0.061

>Non-Methylation code ID 1437

0.000 0.000 0.000 0.000 0.000 0.015 0.000 0.000 0.000 0.005

0.000 0.000 0.005 0.005 0.010 0.005 0.005 0.005 0.010 0.000

0.076 0.065 0.072 0.054 0.028 0.000 0.000 0.000 0.000 0.038

0.000 0.000 0.000 0.000 0.000 0.000 0.019 0.000 0.009 0.009

0.000 0.009 0.000 0.000 0.059 0.050 0.042 0.068 0.010 0.000

0.000 0.005 0.000 0.010 0.005 0.000 0.000 0.010 0.000 0.000

0.010 0.000 0.010 0.000 0.000 0.000 0.000 0.000 0.084 0.044

0.071 0.074

>Non-Methylation code ID 1437

0.000 0.000 0.004 0.004 0.000 0.008 0.004 0.000 0.000 0.004

0.000 0.000 0.000 0.004 0.016 0.000 0.000 0.000 0.008 0.000

0.083 0.062 0.072 0.063 0.024 0.012 0.000 0.000 0.000 0.061

0.000 0.000 0.000 0.000 0.012 0.000 0.000 0.000 0.000 0.024

0.012 0.000 0.000 0.000 0.041 0.052 0.046 0.048 0.031 0.000

0.000 0.000 0.000 0.008 0.000 0.000 0.000 0.000 0.000 0.000

0.016 0.000 0.008 0.000 0.000 0.000 0.008 0.000 0.087 0.058

0.052 0.066

>Non-Methylation code ID 1437

0.000 0.008 0.000 0.000 0.000 0.000 0.004 0.000 0.000 0.004

0.000 0.004 0.013 0.000 0.008 0.004 0.000 0.000 0.004 0.000

0.085 0.081 0.061 0.055 0.021 0.000 0.000 0.007 0.000 0.014

0.007 0.000 0.000 0.000 0.000 0.000 0.007 0.000 0.007 0.014

0.014 0.000 0.000 0.000 0.056 0.070 0.063 0.052 0.017 0.000

0.000 0.008 0.000 0.000 0.000 0.000 0.008 0.025 0.000 0.000

0.017 0.008 0.000 0.000 0.008 0.000 0.000 0.000 0.054 0.059

0.064 0.064

>Non-Methylation code ID 1437

0.005 0.000 0.000 0.000 0.000 0.009 0.009 0.000 0.000 0.000

0.000 0.000 0.009 0.000 0.014 0.014 0.000 0.000 0.000 0.000

0.068 0.086 0.061 0.058 0.028 0.000 0.000 0.000 0.000 0.028

0.000 0.000 0.000 0.000 0.000 0.000 0.014 0.000 0.007 0.000

0.014 0.000 0.000 0.000 0.061 0.049 0.063 0.070 0.014 0.000

0.000 0.014 0.000 0.007 0.000 0.000 0.000 0.000 0.000 0.000

0.027 0.014 0.007 0.000 0.000 0.000 0.000 0.000 0.058 0.071

0.063 0.060

>Non-Methylation code ID 1437

0.018 0.000 0.000 0.018 0.000 0.018 0.000 0.000 0.000 0.018

0.000 0.000 0.009 0.000 0.000 0.027 0.009 0.000 0.000 0.000

0.051 0.056 0.057 0.054 0.005 0.000 0.000 0.000 0.000 0.005

0.005 0.000 0.000 0.000 0.000 0.005 0.026 0.000 0.005 0.005

0.000 0.000 0.005 0.000 0.084 0.053 0.060 0.073 0.005 0.000

0.000 0.000 0.000 0.011 0.005 0.000 0.000 0.011 0.000 0.000

0.005 0.000 0.011 0.005 0.005 0.005 0.000 0.000 0.073 0.059

0.065 0.072

>Non-Methylation code ID 1437

0.017 0.000 0.000 0.000 0.000 0.000 0.009 0.000 0.000 0.017

0.009 0.000 0.026 0.000 0.000 0.009 0.026 0.000 0.000 0.000

0.062 0.056 0.052 0.051 0.000 0.005 0.000 0.000 0.000 0.000

0.000 0.000 0.000 0.016 0.000 0.000 0.027 0.000 0.005 0.005

0.005 0.000 0.005 0.000 0.070 0.065 0.060 0.070 0.007 0.007

0.000 0.000 0.000 0.007 0.027 0.000 0.000 0.000 0.000 0.000

0.013 0.000 0.000 0.013 0.000 0.007 0.000 0.000 0.074 0.053

0.060 0.066

>Non-Methylation code ID 1437

0.018 0.000 0.000 0.000 0.000 0.009 0.000 0.000 0.000 0.026

0.000 0.000 0.044 0.000 0.000 0.018 0.000 0.000 0.000 0.000

0.051 0.059 0.041 0.068 0.005 0.000 0.000 0.000 0.000 0.000

0.000 0.000 0.000 0.010 0.000 0.000 0.025 0.000 0.010 0.015

0.000 0.000 0.000 0.000 0.073 0.063 0.043 0.089 0.006 0.000

0.000 0.000 0.000 0.006 0.000 0.000 0.000 0.006 0.000 0.000

0.038 0.000 0.006 0.006 0.000 0.000 0.006 0.000 0.065 0.068

0.058 0.066

>Non-Methylation code ID 1437

0.000 0.007 0.000 0.000 0.000 0.007 0.021 0.000 0.000 0.007

0.000 0.014 0.014 0.000 0.000 0.000 0.021 0.000 0.000 0.000

0.059 0.049 0.060 0.075 0.000 0.000 0.000 0.000 0.000 0.008

0.024 0.000 0.000 0.000 0.008 0.000 0.008 0.000 0.008 0.008

0.032 0.008 0.000 0.000 0.076 0.047 0.070 0.036 0.008 0.000

0.000 0.008 0.000 0.000 0.025 0.000 0.000 0.008 0.000 0.000

0.000 0.008 0.000 0.008 0.025 0.000 0.008 0.000 0.068 0.041

0.056 0.067

>Non-Methylation code ID 1437

0.000 0.013 0.000 0.000 0.000 0.000 0.000 0.000 0.000 0.007

0.000 0.000 0.040 0.000 0.007 0.020 0.000 0.000 0.000 0.000

0.066 0.070 0.050 0.062 0.007 0.000 0.000 0.000 0.000 0.007

0.000 0.000 0.000 0.007 0.000 0.000 0.046 0.000 0.007 0.007

0.000 0.007 0.000 0.000 0.064 0.062 0.065 0.057 0.019 0.000

0.000 0.000 0.000 0.000 0.000 0.000 0.000 0.039 0.000 0.000

0.039 0.000 0.000 0.019 0.000 0.000 0.000 0.000 0.058 0.043

0.053 0.062

>Non-Methylation code ID 1437

0.009 0.000 0.005 0.000 0.000 0.005 0.005 0.005 0.005 0.000

0.000 0.000 0.005 0.000 0.009 0.000 0.005 0.000 0.005 0.000

0.066 0.072 0.075 0.065 0.000 0.006 0.000 0.000 0.000 0.017

0.006 0.000 0.000 0.006 0.000 0.006 0.011 0.000 0.006 0.000

0.011 0.006 0.000 0.000 0.073 0.068 0.058 0.060 0.000 0.009

0.000 0.000 0.000 0.027 0.018 0.000 0.000 0.000 0.000 0.000

0.009 0.009 0.000 0.009 0.018 0.000 0.000 0.000 0.066 0.052

0.063 0.055

>Non-Methylation code ID 1437

0.000 0.005 0.000 0.005 0.000 0.016 0.005 0.000 0.000 0.000

0.000 0.000 0.011 0.005 0.005 0.011 0.000 0.005 0.000 0.000

0.067 0.055 0.076 0.067 0.023 0.000 0.000 0.000 0.000 0.012

0.000 0.000 0.000 0.006 0.000 0.000 0.006 0.000 0.017 0.000

0.006 0.000 0.000 0.000 0.060 0.061 0.068 0.076 0.000 0.000

0.000 0.005 0.000 0.015 0.000 0.000 0.005 0.005 0.000 0.000

0.015 0.000 0.005 0.000 0.000 0.005 0.005 0.000 0.062 0.085

0.067 0.061

>Non-Methylation code ID 1437

0.018 0.000 0.000 0.000 0.000 0.018 0.000 0.000 0.000 0.036

0.000 0.000 0.036 0.000 0.000 0.009 0.000 0.000 0.000 0.000

0.054 0.055 0.033 0.074 0.009 0.000 0.000 0.000 0.000 0.000

0.000 0.000 0.000 0.005 0.000 0.000 0.014 0.000 0.014 0.014

0.000 0.000 0.005 0.000 0.087 0.070 0.044 0.073 0.007 0.000

0.000 0.000 0.000 0.013 0.000 0.000 0.000 0.007 0.000 0.000

0.039 0.000 0.007 0.000 0.000 0.000 0.007 0.000 0.061 0.071

0.056 0.067

>Non-Methylation code ID 1437

0.009 0.000 0.000 0.000 0.000 0.000 0.000 0.000 0.000 0.005

0.000 0.000 0.019 0.000 0.014 0.014 0.000 0.000 0.000 0.000

0.087 0.071 0.036 0.077 0.006 0.000 0.000 0.000 0.000 0.012

0.000 0.000 0.000 0.006 0.000 0.000 0.043 0.000 0.006 0.000

0.000 0.000 0.006 0.000 0.072 0.070 0.051 0.060 0.020 0.000

0.000 0.000 0.000 0.020 0.000 0.000 0.000 0.029 0.000 0.000

0.039 0.000 0.000 0.010 0.000 0.000 0.000 0.000 0.055 0.052

0.032 0.077

>Non-Methylation code ID 1437

0.017 0.000 0.000 0.008 0.000 0.017 0.000 0.000 0.000 0.025

0.000 0.000 0.033 0.000 0.000 0.008 0.000 0.000 0.000 0.000

0.057 0.058 0.040 0.070 0.009 0.000 0.000 0.000 0.000 0.000

0.000 0.000 0.000 0.005 0.000 0.005 0.014 0.000 0.014 0.009

0.000 0.000 0.005 0.000 0.087 0.071 0.047 0.069 0.008 0.000

0.000 0.000 0.000 0.023 0.000 0.000 0.000 0.008 0.000 0.000

0.046 0.000 0.000 0.000 0.000 0.000 0.008 0.000 0.058 0.062

0.050 0.071

>Non-Methylation code ID 1437

0.000 0.015 0.000 0.000 0.000 0.007 0.000 0.000 0.000 0.015

0.000 0.007 0.045 0.000 0.000 0.007 0.000 0.000 0.000 0.000

0.061 0.057 0.052 0.067 0.008 0.000 0.000 0.000 0.000 0.008

0.008 0.000 0.000 0.008 0.008 0.000 0.041 0.000 0.000 0.016

0.000 0.008 0.000 0.000 0.059 0.053 0.057 0.059 0.009 0.000

0.000 0.000 0.000 0.000 0.000 0.000 0.000 0.028 0.000 0.000

0.037 0.000 0.000 0.019 0.009 0.000 0.009 0.000 0.069 0.037

0.057 0.060

>Non-Methylation code ID 1437

0.013 0.000 0.000 0.000 0.000 0.019 0.000 0.000 0.000 0.013

0.000 0.000 0.019 0.000 0.006 0.006 0.006 0.000 0.000 0.000

0.056 0.059 0.060 0.077 0.015 0.005 0.000 0.005 0.000 0.005

0.000 0.000 0.000 0.015 0.000 0.000 0.010 0.000 0.005 0.000

0.000 0.000 0.005 0.000 0.066 0.067 0.067 0.069 0.014 0.000

0.000 0.000 0.000 0.005 0.005 0.000 0.005 0.000 0.000 0.000

0.009 0.000 0.009 0.000 0.000 0.000 0.009 0.000 0.065 0.080

0.063 0.069

>Non-Methylation code ID 1437

0.006 0.011 0.000 0.000 0.000 0.000 0.011 0.000 0.000 0.000

0.006 0.000 0.028 0.000 0.006 0.000 0.000 0.006 0.000 0.000

0.066 0.069 0.058 0.067 0.016 0.005 0.000 0.000 0.000 0.000

0.005 0.000 0.000 0.000 0.000 0.000 0.016 0.000 0.010 0.005

0.010 0.000 0.000 0.000 0.084 0.069 0.068 0.046 0.015 0.000

0.000 0.000 0.000 0.000 0.005 0.000 0.000 0.005 0.005 0.000

0.020 0.000 0.010 0.000 0.000 0.000 0.000 0.000 0.081 0.059

0.077 0.057

>Non-Methylation code ID 1437

0.000 0.000 0.000 0.007 0.000 0.007 0.027 0.000 0.000 0.000

0.000 0.000 0.014 0.000 0.007 0.000 0.014 0.000 0.000 0.000

0.064 0.058 0.083 0.053 0.007 0.007 0.000 0.000 0.000 0.000

0.021 0.000 0.000 0.000 0.000 0.007 0.007 0.000 0.000 0.000

0.028 0.000 0.007 0.000 0.067 0.053 0.062 0.067 0.007 0.007

0.000 0.000 0.000 0.007 0.013 0.000 0.000 0.000 0.000 0.000

0.013 0.000 0.007 0.000 0.026 0.000 0.000 0.000 0.071 0.049

0.068 0.066

>Non-Methylation code ID 1437

0.008 0.000 0.000 0.000 0.000 0.000 0.000 0.000 0.000 0.025

0.000 0.000 0.034 0.008 0.000 0.017 0.008 0.000 0.008 0.000

0.071 0.040 0.052 0.059 0.000 0.015 0.000 0.000 0.000 0.007

0.000 0.000 0.000 0.015 0.000 0.007 0.045 0.000 0.000 0.007

0.000 0.000 0.000 0.000 0.061 0.057 0.052 0.067 0.009 0.000

0.000 0.000 0.000 0.009 0.009 0.000 0.000 0.009 0.009 0.000

0.035 0.000 0.000 0.018 0.000 0.009 0.000 0.000 0.056 0.058

0.054 0.060

>Non-Methylation code ID 1437

0.000 0.010 0.000 0.000 0.000 0.000 0.015 0.000 0.000 0.000

0.005 0.000 0.005 0.000 0.010 0.000 0.005 0.005 0.010 0.000

0.063 0.080 0.071 0.055 0.029 0.007 0.000 0.000 0.000 0.015

0.015 0.000 0.000 0.000 0.000 0.000 0.000 0.000 0.000 0.007

0.022 0.000 0.000 0.000 0.061 0.057 0.050 0.071 0.012 0.000

0.006 0.000 0.000 0.000 0.006 0.000 0.000 0.012 0.006 0.000

0.018 0.000 0.006 0.000 0.006 0.000 0.000 0.000 0.076 0.058

0.056 0.072

>Non-Methylation code ID 1437

0.000 0.014 0.000 0.000 0.000 0.007 0.014 0.000 0.000 0.007

0.000 0.007 0.027 0.000 0.000 0.007 0.007 0.000 0.000 0.000

0.056 0.060 0.054 0.075 0.008 0.000 0.000 0.000 0.000 0.008

0.017 0.000 0.000 0.008 0.008 0.000 0.025 0.000 0.000 0.008

0.017 0.008 0.000 0.000 0.049 0.055 0.069 0.050 0.009 0.000

0.000 0.000 0.000 0.000 0.009 0.000 0.000 0.026 0.000 0.000

0.017 0.009 0.000 0.009 0.017 0.000 0.009 0.000 0.063 0.044

0.056 0.066

>Non-Methylation code ID 1437

0.000 0.007 0.000 0.000 0.000 0.007 0.021 0.000 0.000 0.007

0.000 0.014 0.007 0.000 0.000 0.000 0.028 0.000 0.000 0.000

0.060 0.052 0.064 0.067 0.008 0.000 0.000 0.000 0.000 0.008

0.030 0.000 0.000 0.000 0.008 0.000 0.000 0.000 0.008 0.008

0.030 0.000 0.000 0.000 0.081 0.042 0.082 0.029 0.009 0.000

0.000 0.009 0.000 0.000 0.035 0.000 0.000 0.000 0.000 0.000

0.000 0.009 0.000 0.000 0.035 0.000 0.009 0.000 0.072 0.045

0.067 0.045

>Non-Methylation code ID 1437

0.008 0.008 0.000 0.000 0.000 0.000 0.032 0.000 0.000 0.000

0.000 0.008 0.008 0.000 0.000 0.000 0.032 0.000 0.000 0.000

0.070 0.045 0.064 0.058 0.007 0.007 0.000 0.000 0.000 0.000

0.020 0.000 0.000 0.000 0.000 0.000 0.013 0.000 0.007 0.000

0.033 0.000 0.000 0.000 0.072 0.043 0.072 0.062 0.000 0.000

0.000 0.008 0.000 0.000 0.030 0.000 0.000 0.000 0.000 0.000

0.015 0.000 0.008 0.000 0.023 0.000 0.000 0.000 0.069 0.058

0.073 0.050

>Non-Methylation code ID 1437

0.015 0.000 0.000 0.000 0.000 0.015 0.000 0.000 0.005 0.010

0.000 0.000 0.005 0.000 0.010 0.000 0.005 0.000 0.000 0.000

0.079 0.057 0.055 0.078 0.000 0.000 0.000 0.010 0.000 0.010

0.000 0.000 0.000 0.010 0.000 0.000 0.010 0.000 0.010 0.005

0.000 0.000 0.005 0.000 0.070 0.061 0.066 0.074 0.017 0.011

0.000 0.006 0.000 0.006 0.000 0.000 0.000 0.000 0.000 0.006

0.006 0.006 0.000 0.006 0.000 0.000 0.006 0.000 0.060 0.071

0.068 0.066

>Non-Methylation code ID 1437

0.009 0.000 0.000 0.000 0.000 0.000 0.000 0.000 0.000 0.026

0.000 0.000 0.026 0.009 0.000 0.017 0.017 0.000 0.009 0.000

0.072 0.043 0.057 0.050 0.000 0.014 0.000 0.000 0.000 0.007

0.007 0.000 0.000 0.014 0.000 0.007 0.034 0.000 0.000 0.007

0.000 0.000 0.000 0.000 0.055 0.063 0.058 0.069 0.009 0.000

0.000 0.000 0.000 0.009 0.009 0.000 0.000 0.009 0.009 0.000

0.037 0.000 0.000 0.009 0.009 0.009 0.000 0.000 0.052 0.060

0.059 0.052

>Non-Methylation code ID 1437

0.007 0.000 0.007 0.000 0.000 0.014 0.000 0.000 0.000 0.000

0.007 0.000 0.007 0.000 0.007 0.020 0.007 0.007 0.000 0.000

0.050 0.060 0.064 0.077 0.015 0.000 0.000 0.015 0.000 0.007

0.000 0.000 0.000 0.022 0.000 0.000 0.000 0.000 0.007 0.000

0.007 0.000 0.007 0.000 0.070 0.043 0.073 0.067 0.000 0.005

0.005 0.000 0.000 0.009 0.005 0.000 0.009 0.000 0.000 0.000

0.009 0.000 0.000 0.000 0.000 0.000 0.009 0.000 0.081 0.068

0.055 0.078

>Non-Methylation code ID 1437

0.015 0.010 0.000 0.005 0.000 0.005 0.000 0.000 0.000 0.000

0.000 0.005 0.005 0.005 0.000 0.005 0.000 0.000 0.010 0.000

0.060 0.069 0.068 0.071 0.015 0.000 0.000 0.000 0.000 0.015

0.000 0.000 0.005 0.010 0.000 0.000 0.005 0.000 0.010 0.000

0.005 0.000 0.000 0.000 0.079 0.057 0.055 0.078 0.000 0.000

0.000 0.013 0.000 0.013 0.000 0.000 0.000 0.013 0.000 0.000

0.013 0.000 0.006 0.006 0.000 0.000 0.006 0.000 0.069 0.063

0.061 0.070

>Non-Methylation code ID 1437

0.008 0.000 0.000 0.000 0.000 0.008 0.017 0.000 0.000 0.008

0.008 0.000 0.033 0.000 0.000 0.008 0.008 0.008 0.000 0.000

0.052 0.060 0.067 0.046 0.009 0.000 0.000 0.000 0.000 0.000

0.000 0.000 0.000 0.026 0.000 0.000 0.026 0.009 0.000 0.017

0.017 0.000 0.009 0.000 0.072 0.043 0.057 0.050 0.000 0.015

0.000 0.000 0.000 0.008 0.008 0.000 0.000 0.008 0.000 0.008

0.038 0.000 0.000 0.008 0.000 0.000 0.000 0.000 0.055 0.065

0.052 0.071

>Non-Methylation code ID 1437

0.006 0.006 0.000 0.000 0.000 0.006 0.019 0.000 0.000 0.000

0.000 0.000 0.012 0.000 0.006 0.000 0.025 0.000 0.000 0.000

0.070 0.045 0.067 0.071 0.000 0.000 0.000 0.007 0.000 0.007

0.026 0.000 0.000 0.000 0.000 0.000 0.013 0.000 0.007 0.000

0.020 0.000 0.000 0.000 0.064 0.055 0.082 0.055 0.009 0.009

0.000 0.000 0.000 0.000 0.026 0.000 0.000 0.000 0.000 0.009

0.009 0.000 0.000 0.000 0.034 0.000 0.000 0.000 0.070 0.049

0.064 0.056

>Non-Methylation code ID 1437

0.000 0.006 0.000 0.000 0.000 0.011 0.017 0.000 0.000 0.000

0.000 0.000 0.011 0.000 0.006 0.000 0.011 0.000 0.000 0.000

0.061 0.065 0.064 0.080 0.005 0.005 0.005 0.000 0.000 0.000

0.011 0.005 0.000 0.000 0.000 0.000 0.005 0.000 0.005 0.000

0.011 0.000 0.005 0.000 0.064 0.068 0.067 0.075 0.007 0.015

0.000 0.000 0.000 0.015 0.007 0.000 0.000 0.007 0.000 0.000

0.015 0.000 0.000 0.000 0.022 0.000 0.000 0.000 0.074 0.053

0.054 0.065

>Non-Methylation code ID 1437

0.010 0.000 0.005 0.000 0.000 0.000 0.005 0.000 0.000 0.015

0.000 0.000 0.015 0.000 0.010 0.000 0.005 0.000 0.000 0.000

0.074 0.057 0.064 0.072 0.005 0.005 0.000 0.000 0.000 0.000

0.014 0.000 0.000 0.000 0.005 0.000 0.005 0.000 0.009 0.000

0.005 0.005 0.009 0.000 0.066 0.083 0.074 0.052 0.026 0.009

0.000 0.000 0.000 0.017 0.009 0.000 0.000 0.000 0.000 0.000

0.009 0.000 0.000 0.009 0.026 0.000 0.000 0.000 0.058 0.065

0.046 0.063

>Non-Methylation code ID 1437

0.004 0.004 0.000 0.000 0.000 0.000 0.012 0.000 0.000 0.000

0.004 0.000 0.004 0.000 0.008 0.000 0.004 0.000 0.012 0.000

0.062 0.083 0.068 0.067 0.024 0.008 0.000 0.000 0.000 0.024

0.008 0.000 0.000 0.000 0.000 0.000 0.016 0.000 0.000 0.000

0.024 0.000 0.000 0.000 0.061 0.060 0.044 0.063 0.018 0.000

0.006 0.000 0.000 0.000 0.006 0.000 0.000 0.018 0.000 0.000

0.012 0.000 0.006 0.000 0.006 0.000 0.000 0.000 0.075 0.059

0.051 0.074

>Non-Methylation code ID 1437

0.000 0.006 0.000 0.000 0.000 0.017 0.006 0.000 0.000 0.000

0.000 0.000 0.011 0.006 0.006 0.011 0.006 0.006 0.000 0.000

0.065 0.049 0.073 0.072 0.029 0.000 0.000 0.000 0.000 0.015

0.007 0.000 0.007 0.007 0.000 0.000 0.007 0.000 0.007 0.000

0.007 0.000 0.000 0.000 0.073 0.057 0.054 0.063 0.000 0.000

0.000 0.005 0.000 0.005 0.005 0.000 0.005 0.005 0.000 0.000

0.015 0.000 0.005 0.000 0.005 0.005 0.005 0.000 0.065 0.080

0.066 0.063

>Non-Methylation code ID 1437

0.024 0.000 0.000 0.000 0.000 0.012 0.000 0.000 0.012 0.000

0.006 0.000 0.012 0.000 0.006 0.000 0.000 0.000 0.000 0.000

0.059 0.066 0.068 0.068 0.004 0.004 0.000 0.000 0.000 0.009

0.000 0.000 0.000 0.000 0.000 0.000 0.017 0.000 0.017 0.000

0.000 0.000 0.004 0.000 0.060 0.065 0.071 0.082 0.009 0.000

0.000 0.009 0.000 0.014 0.005 0.000 0.000 0.000 0.000 0.000

0.005 0.000 0.014 0.000 0.000 0.000 0.000 0.000 0.072 0.086

0.055 0.065

>Non-Methylation code ID 1437

0.016 0.000 0.000 0.000 0.000 0.010 0.000 0.000 0.000 0.000

0.000 0.000 0.031 0.000 0.010 0.000 0.000 0.000 0.000 0.000

0.069 0.061 0.063 0.073 0.014 0.000 0.000 0.000 0.000 0.005

0.000 0.000 0.000 0.005 0.000 0.000 0.023 0.000 0.014 0.000

0.000 0.000 0.000 0.000 0.082 0.072 0.047 0.072 0.011 0.000

0.000 0.000 0.000 0.011 0.000 0.000 0.000 0.000 0.000 0.000

0.032 0.000 0.011 0.000 0.000 0.000 0.000 0.000 0.076 0.073

0.062 0.057

>Non-Methylation code ID 1437

0.014 0.000 0.005 0.000 0.000 0.010 0.000 0.000 0.000 0.000

0.000 0.000 0.014 0.000 0.014 0.000 0.000 0.000 0.000 0.000

0.052 0.065 0.073 0.086 0.018 0.006 0.000 0.000 0.000 0.012

0.000 0.000 0.000 0.000 0.000 0.000 0.030 0.000 0.006 0.000

0.006 0.000 0.000 0.000 0.067 0.070 0.047 0.070 0.009 0.000

0.000 0.000 0.000 0.009 0.000 0.000 0.000 0.000 0.000 0.000

0.013 0.000 0.022 0.000 0.000 0.000 0.000 0.000 0.093 0.075

0.048 0.066

>Non-Methylation code ID 1437

0.004 0.000 0.000 0.000 0.000 0.004 0.000 0.000 0.000 0.000

0.000 0.000 0.025 0.000 0.017 0.000 0.000 0.004 0.000 0.000

0.074 0.097 0.064 0.044 0.031 0.000 0.000 0.000 0.000 0.008

0.000 0.000 0.000 0.000 0.000 0.000 0.047 0.000 0.008 0.008

0.000 0.000 0.000 0.000 0.063 0.051 0.059 0.059 0.006 0.000

0.000 0.000 0.000 0.000 0.000 0.000 0.000 0.000 0.000 0.000

0.046 0.000 0.017 0.000 0.000 0.000 0.000 0.000 0.076 0.086

0.093 0.009

>Non-Methylation code ID 1437

0.020 0.007 0.000 0.000 0.000 0.013 0.000 0.000 0.000 0.000

0.000 0.000 0.026 0.000 0.007 0.000 0.007 0.000 0.000 0.000

0.063 0.072 0.055 0.064 0.012 0.000 0.000 0.004 0.000 0.004

0.000 0.000 0.000 0.000 0.000 0.000 0.012 0.000 0.020 0.000

0.000 0.000 0.000 0.000 0.080 0.072 0.054 0.077 0.010 0.000

0.005 0.000 0.000 0.005 0.000 0.000 0.000 0.000 0.000 0.000

0.015 0.000 0.020 0.000 0.000 0.000 0.000 0.000 0.054 0.064

0.070 0.091

>Non-Methylation code ID 1437

0.000 0.005 0.005 0.000 0.005 0.005 0.000 0.005 0.000 0.000

0.005 0.000 0.000 0.005 0.005 0.005 0.005 0.000 0.005 0.000

0.061 0.063 0.066 0.082 0.012 0.004 0.000 0.000 0.000 0.004

0.000 0.000 0.000 0.000 0.000 0.000 0.000 0.000 0.012 0.008

0.004 0.000 0.008 0.000 0.087 0.050 0.080 0.066 0.006 0.000

0.006 0.000 0.000 0.019 0.012 0.000 0.000 0.006 0.000 0.000

0.006 0.000 0.006 0.000 0.000 0.006 0.000 0.000 0.068 0.054

0.064 0.079

>Non-Methylation code ID 1437

0.004 0.000 0.000 0.000 0.000 0.004 0.000 0.000 0.000 0.000

0.000 0.000 0.025 0.000 0.017 0.000 0.000 0.004 0.000 0.000

0.063 0.084 0.075 0.057 0.027 0.000 0.000 0.000 0.000 0.007

0.000 0.000 0.000 0.000 0.000 0.000 0.033 0.000 0.013 0.007

0.000 0.000 0.000 0.000 0.050 0.057 0.071 0.068 0.017 0.000

0.000 0.000 0.000 0.000 0.000 0.000 0.000 0.000 0.000 0.000

0.033 0.000 0.017 0.000 0.000 0.000 0.000 0.000 0.089 0.088

0.077 0.013

>Non-Methylation code ID 1437

0.020 0.000 0.013 0.000 0.000 0.026 0.000 0.000 0.007 0.000

0.000 0.000 0.020 0.000 0.000 0.000 0.000 0.000 0.000 0.000

0.075 0.054 0.066 0.054 0.009 0.000 0.000 0.000 0.000 0.009

0.000 0.000 0.000 0.000 0.000 0.000 0.018 0.000 0.014 0.000

0.009 0.000 0.000 0.000 0.077 0.047 0.078 0.073 0.008 0.000

0.000 0.000 0.000 0.013 0.000 0.000 0.000 0.000 0.000 0.000

0.004 0.004 0.021 0.000 0.000 0.000 0.000 0.000 0.069 0.059

0.068 0.086

>Non-Methylation code ID 1437

0.016 0.000 0.000 0.000 0.005 0.011 0.000 0.000 0.000 0.005

0.000 0.005 0.000 0.000 0.011 0.000 0.000 0.000 0.005 0.000

0.069 0.072 0.050 0.083 0.025 0.006 0.000 0.000 0.000 0.006

0.000 0.000 0.000 0.000 0.000 0.000 0.019 0.000 0.000 0.006

0.006 0.000 0.013 0.000 0.075 0.054 0.061 0.062 0.005 0.000

0.000 0.000 0.000 0.009 0.000 0.000 0.000 0.005 0.000 0.000

0.005 0.005 0.023 0.000 0.000 0.000 0.000 0.000 0.062 0.075

0.060 0.086

>Non-Methylation code ID 1437

0.019 0.000 0.000 0.000 0.000 0.005 0.005 0.000 0.000 0.000

0.000 0.005 0.009 0.000 0.009 0.000 0.000 0.000 0.005 0.000

0.080 0.077 0.047 0.072 0.019 0.006 0.000 0.000 0.000 0.006

0.000 0.000 0.000 0.000 0.000 0.000 0.032 0.000 0.006 0.000

0.013 0.000 0.000 0.000 0.066 0.082 0.053 0.048 0.009 0.000

0.000 0.000 0.000 0.000 0.000 0.000 0.000 0.000 0.000 0.000

0.014 0.005 0.024 0.000 0.000 0.000 0.000 0.000 0.084 0.060

0.058 0.078

>Non-Methylation code ID 1437

0.016 0.000 0.000 0.000 0.000 0.005 0.000 0.000 0.000 0.000

0.000 0.000 0.031 0.000 0.010 0.005 0.000 0.000 0.000 0.000

0.050 0.070 0.078 0.068 0.016 0.000 0.000 0.000 0.000 0.000

0.005 0.000 0.000 0.000 0.000 0.000 0.038 0.000 0.011 0.000

0.000 0.000 0.000 0.000 0.074 0.078 0.086 0.025 0.004 0.000

0.000 0.000 0.000 0.000 0.000 0.000 0.000 0.000 0.000 0.000

0.021 0.000 0.017 0.000 0.004 0.004 0.000 0.000 0.051 0.079

0.090 0.062

>Non-Methylation code ID 1437

0.000 0.000 0.006 0.006 0.000 0.012 0.000 0.006 0.012 0.006

0.000 0.000 0.006 0.000 0.000 0.012 0.000 0.000 0.000 0.006

0.044 0.064 0.079 0.074 0.013 0.004 0.000 0.000 0.000 0.000

0.000 0.004 0.000 0.000 0.000 0.000 0.009 0.000 0.013 0.009

0.004 0.000 0.000 0.000 0.039 0.066 0.089 0.085 0.000 0.000

0.000 0.010 0.000 0.005 0.000 0.000 0.000 0.005 0.000 0.000

0.010 0.000 0.020 0.000 0.000 0.005 0.000 0.000 0.058 0.051

0.085 0.084

>Non-Methylation code ID 1437

0.000 0.004 0.004 0.000 0.000 0.004 0.000 0.000 0.000 0.000

0.000 0.000 0.020 0.000 0.016 0.000 0.000 0.000 0.000 0.000

0.041 0.078 0.085 0.082 0.024 0.000 0.000 0.005 0.000 0.005

0.000 0.000 0.000 0.005 0.000 0.000 0.010 0.000 0.010 0.000

0.005 0.000 0.000 0.000 0.060 0.072 0.072 0.067 0.012 0.000

0.000 0.000 0.000 0.000 0.000 0.000 0.000 0.000 0.000 0.000

0.086 0.000 0.012 0.000 0.000 0.000 0.000 0.000 0.046 0.053

0.061 0.062

>Non-Methylation code ID 1437

0.000 0.000 0.005 0.005 0.005 0.000 0.000 0.005 0.000 0.000

0.005 0.000 0.005 0.005 0.009 0.000 0.009 0.000 0.005 0.000

0.064 0.064 0.072 0.077 0.008 0.004 0.000 0.000 0.000 0.004

0.000 0.000 0.000 0.000 0.000 0.000 0.004 0.000 0.015 0.008

0.004 0.000 0.004 0.000 0.082 0.049 0.060 0.093 0.011 0.000

0.011 0.000 0.000 0.011 0.011 0.000 0.000 0.011 0.000 0.000

0.006 0.000 0.006 0.000 0.000 0.000 0.000 0.000 0.068 0.056

0.067 0.075

>Non-Methylation code ID 1437

0.009 0.000 0.000 0.000 0.000 0.009 0.000 0.000 0.000 0.000

0.000 0.000 0.018 0.000 0.014 0.000 0.009 0.000 0.000 0.000

0.077 0.047 0.078 0.073 0.008 0.000 0.000 0.000 0.000 0.012

0.000 0.000 0.000 0.000 0.000 0.000 0.008 0.004 0.019 0.000

0.000 0.000 0.000 0.000 0.073 0.064 0.065 0.081 0.014 0.000

0.014 0.000 0.000 0.028 0.000 0.000 0.007 0.000 0.000 0.000

0.021 0.000 0.000 0.000 0.000 0.000 0.000 0.000 0.077 0.059

0.068 0.047

>Non-Methylation code ID 1437

0.005 0.000 0.005 0.000 0.000 0.000 0.000 0.000 0.000 0.000

0.000 0.000 0.032 0.000 0.016 0.000 0.000 0.000 0.000 0.000

0.036 0.061 0.086 0.090 0.010 0.000 0.005 0.000 0.000 0.005

0.000 0.000 0.000 0.000 0.000 0.000 0.026 0.000 0.010 0.000

0.005 0.000 0.000 0.000 0.039 0.073 0.083 0.077 0.015 0.000

0.000 0.005 0.000 0.000 0.000 0.000 0.000 0.005 0.000 0.000

0.015 0.000 0.015 0.000 0.005 0.000 0.000 0.000 0.074 0.071

0.079 0.050

>Non-Methylation code ID 1437

0.000 0.008 0.000 0.000 0.000 0.004 0.000 0.000 0.000 0.000

0.000 0.000 0.023 0.000 0.015 0.000 0.000 0.000 0.000 0.000

0.074 0.070 0.060 0.079 0.032 0.000 0.000 0.000 0.000 0.024

0.000 0.000 0.000 0.024 0.000 0.000 0.016 0.000 0.008 0.000

0.000 0.000 0.000 0.000 0.065 0.057 0.049 0.059 0.014 0.000

0.000 0.000 0.000 0.014 0.000 0.000 0.000 0.000 0.000 0.000

0.084 0.000 0.000 0.028 0.000 0.000 0.000 0.000 0.047 0.041

0.040 0.066

>Non-Methylation code ID 1437

0.019 0.000 0.000 0.000 0.000 0.013 0.000 0.000 0.000 0.000

0.000 0.000 0.038 0.000 0.006 0.006 0.000 0.000 0.000 0.000

0.062 0.058 0.061 0.071 0.010 0.000 0.000 0.000 0.000 0.005

0.000 0.000 0.000 0.000 0.000 0.000 0.035 0.000 0.015 0.000

0.000 0.000 0.000 0.000 0.076 0.079 0.060 0.054 0.005 0.000

0.000 0.000 0.000 0.010 0.000 0.000 0.000 0.000 0.000 0.000

0.029 0.000 0.015 0.000 0.000 0.000 0.000 0.000 0.087 0.066

0.045 0.076

>Non-Methylation code ID 1437

0.000 0.007 0.004 0.000 0.000 0.000 0.000 0.000 0.000 0.000

0.000 0.000 0.018 0.000 0.018 0.000 0.000 0.000 0.000 0.000

0.059 0.071 0.060 0.096 0.033 0.000 0.000 0.000 0.000 0.013

0.000 0.000 0.000 0.013 0.000 0.000 0.013 0.000 0.007 0.000

0.007 0.000 0.000 0.000 0.054 0.056 0.064 0.074 0.019 0.000

0.000 0.000 0.000 0.000 0.000 0.000 0.000 0.000 0.000 0.000

0.132 0.000 0.000 0.019 0.000 0.000 0.000 0.000 0.039 0.044

0.037 0.044

>Non-Methylation code ID 1437

0.005 0.000 0.000 0.000 0.000 0.009 0.000 0.000 0.000 0.000

0.000 0.000 0.027 0.000 0.014 0.000 0.000 0.005 0.000 0.000

0.084 0.074 0.045 0.071 0.019 0.000 0.000 0.000 0.000 0.013

0.000 0.000 0.000 0.000 0.000 0.000 0.038 0.000 0.006 0.006

0.000 0.000 0.000 0.000 0.062 0.058 0.061 0.071 0.011 0.000

0.000 0.000 0.000 0.006 0.000 0.000 0.000 0.000 0.000 0.000

0.040 0.000 0.011 0.000 0.000 0.000 0.000 0.000 0.073 0.076

0.071 0.045

>Non-Methylation code ID 1437

0.008 0.000 0.000 0.008 0.000 0.008 0.004 0.000 0.000 0.000

0.000 0.000 0.008 0.000 0.017 0.000 0.000 0.000 0.000 0.000

0.073 0.060 0.065 0.081 0.013 0.000 0.007 0.000 0.000 0.020

0.000 0.007 0.013 0.007 0.000 0.000 0.020 0.000 0.000 0.000

0.000 0.000 0.000 0.000 0.053 0.067 0.064 0.062 0.005 0.000

0.000 0.000 0.000 0.010 0.000 0.000 0.000 0.000 0.000 0.000

0.015 0.000 0.015 0.010 0.005 0.000 0.000 0.000 0.057 0.072

0.073 0.069

>Non-Methylation code ID 1437

0.017 0.000 0.000 0.000 0.000 0.006 0.000 0.000 0.000 0.000

0.000 0.000 0.034 0.000 0.011 0.006 0.000 0.000 0.000 0.000

0.056 0.076 0.073 0.055 0.015 0.000 0.000 0.000 0.000 0.000

0.005 0.000 0.000 0.000 0.000 0.000 0.030 0.000 0.015 0.000

0.000 0.000 0.000 0.000 0.075 0.085 0.090 0.019 0.005 0.000

0.000 0.000 0.000 0.000 0.000 0.000 0.000 0.000 0.000 0.000

0.028 0.000 0.014 0.000 0.005 0.005 0.000 0.000 0.060 0.075

0.088 0.053

>Non-Methylation code ID 1437

0.025 0.000 0.000 0.000 0.000 0.005 0.005 0.000 0.000 0.000

0.000 0.000 0.010 0.000 0.005 0.000 0.005 0.000 0.010 0.000

0.071 0.049 0.079 0.069 0.000 0.000 0.004 0.000 0.000 0.011

0.000 0.000 0.000 0.000 0.000 0.000 0.011 0.000 0.019 0.000

0.004 0.000 0.000 0.000 0.069 0.066 0.062 0.086 0.000 0.000

0.010 0.000 0.000 0.059 0.000 0.000 0.000 0.010 0.000 0.000

0.020 0.000 0.000 0.000 0.010 0.000 0.000 0.000 0.070 0.045

0.060 0.051

>Non-Methylation code ID 1437

0.014 0.000 0.000 0.000 0.000 0.005 0.005 0.000 0.000 0.000

0.000 0.000 0.014 0.000 0.009 0.000 0.005 0.000 0.009 0.000

0.090 0.033 0.085 0.067 0.000 0.000 0.004 0.000 0.000 0.017

0.000 0.000 0.000 0.000 0.000 0.000 0.012 0.004 0.012 0.000

0.004 0.000 0.000 0.000 0.068 0.054 0.063 0.095 0.008 0.000

0.008 0.000 0.000 0.031 0.000 0.000 0.008 0.008 0.000 0.000

0.015 0.000 0.000 0.000 0.008 0.000 0.000 0.000 0.071 0.047

0.072 0.059

>Non-Methylation code ID 1437

0.014 0.000 0.007 0.000 0.000 0.035 0.000 0.000 0.007 0.007

0.000 0.000 0.014 0.000 0.000 0.000 0.000 0.000 0.000 0.000

0.067 0.046 0.076 0.059 0.013 0.000 0.000 0.000 0.000 0.009

0.000 0.000 0.000 0.000 0.000 0.000 0.013 0.000 0.009 0.000

0.004 0.000 0.009 0.000 0.085 0.038 0.079 0.073 0.000 0.000

0.000 0.000 0.000 0.023 0.000 0.000 0.000 0.000 0.000 0.000

0.014 0.005 0.014 0.000 0.000 0.000 0.000 0.000 0.069 0.053

0.064 0.093

>Non-Methylation code ID 1437

0.004 0.000 0.004 0.000 0.000 0.004 0.000 0.000 0.000 0.000

0.000 0.000 0.026 0.000 0.013 0.000 0.000 0.000 0.000 0.000

0.034 0.053 0.087 0.106 0.017 0.000 0.000 0.000 0.000 0.006

0.000 0.000 0.000 0.000 0.000 0.000 0.022 0.000 0.017 0.000

0.006 0.000 0.000 0.000 0.057 0.066 0.060 0.084 0.014 0.000

0.000 0.005 0.000 0.005 0.000 0.000 0.000 0.000 0.000 0.000

0.018 0.000 0.014 0.000 0.000 0.000 0.000 0.000 0.080 0.067

0.084 0.047

>Non-Methylation code ID 1437

0.010 0.000 0.000 0.000 0.000 0.005 0.000 0.000 0.000 0.000

0.000 0.000 0.035 0.000 0.015 0.000 0.000 0.000 0.000 0.000

0.076 0.079 0.076 0.038 0.005 0.000 0.000 0.000 0.000 0.009

0.000 0.000 0.000 0.000 0.000 0.000 0.027 0.000 0.014 0.000

0.000 0.005 0.000 0.000 0.084 0.074 0.045 0.071 0.019 0.000

0.000 0.000 0.000 0.013 0.000 0.000 0.000 0.000 0.000 0.000

0.032 0.000 0.006 0.006 0.000 0.000 0.000 0.000 0.059 0.056

0.064 0.075

>Non-Methylation code ID 1437

0.014 0.000 0.000 0.000 0.000 0.005 0.000 0.000 0.000 0.005

0.000 0.000 0.023 0.000 0.014 0.000 0.000 0.000 0.000 0.000

0.082 0.072 0.047 0.072 0.010 0.000 0.000 0.000 0.000 0.010

0.000 0.000 0.000 0.000 0.000 0.000 0.030 0.000 0.010 0.005

0.000 0.000 0.000 0.000 0.074 0.069 0.062 0.063 0.016 0.000

0.000 0.000 0.000 0.005 0.000 0.000 0.000 0.000 0.000 0.000

0.032 0.000 0.011 0.000 0.000 0.000 0.000 0.000 0.067 0.064

0.066 0.073

>Non-Methylation code ID 1437

0.019 0.000 0.000 0.000 0.000 0.012 0.000 0.000 0.000 0.000

0.000 0.000 0.037 0.000 0.006 0.006 0.000 0.000 0.000 0.000

0.062 0.054 0.066 0.070 0.010 0.000 0.000 0.000 0.000 0.005

0.000 0.000 0.000 0.000 0.000 0.000 0.035 0.000 0.015 0.000

0.000 0.000 0.000 0.000 0.076 0.079 0.076 0.038 0.005 0.000

0.000 0.000 0.000 0.010 0.000 0.000 0.000 0.000 0.000 0.000

0.024 0.000 0.014 0.000 0.000 0.005 0.000 0.000 0.077 0.076

0.049 0.074

>Non-Methylation code ID 1440

0.004 0.000 0.000 0.000 0.000 0.000 0.000 0.000 0.000 0.000

0.000 0.000 0.025 0.000 0.016 0.000 0.004 0.004 0.000 0.000

0.059 0.079 0.084 0.058 0.016 0.000 0.000 0.000 0.000 0.005

0.000 0.000 0.000 0.000 0.000 0.000 0.031 0.000 0.010 0.005

0.000 0.000 0.000 0.000 0.050 0.070 0.078 0.068 0.012 0.000

0.000 0.000 0.000 0.000 0.006 0.000 0.000 0.000 0.000 0.000

0.041 0.000 0.012 0.000 0.000 0.000 0.000 0.000 0.078 0.083

0.077 0.024

>Non-Methylation code ID 1440

0.019 0.000 0.000 0.000 0.000 0.000 0.005 0.000 0.000 0.000

0.000 0.000 0.024 0.000 0.014 0.000 0.000 0.000 0.000 0.000

0.079 0.074 0.075 0.044 0.009 0.000 0.000 0.000 0.000 0.000

0.000 0.000 0.000 0.000 0.000 0.000 0.027 0.000 0.013 0.000

0.004 0.004 0.000 0.000 0.055 0.085 0.080 0.056 0.014 0.000

0.000 0.000 0.000 0.000 0.000 0.000 0.000 0.000 0.000 0.000

0.029 0.000 0.014 0.000 0.000 0.000 0.000 0.000 0.059 0.077

0.083 0.056

>Non-Methylation code ID 1440

0.004 0.004 0.000 0.000 0.000 0.008 0.000 0.000 0.000 0.000

0.000 0.000 0.015 0.000 0.019 0.000 0.000 0.000 0.000 0.000

0.083 0.057 0.076 0.067 0.039 0.000 0.000 0.000 0.000 0.023

0.000 0.000 0.000 0.015 0.000 0.000 0.015 0.000 0.008 0.000

0.000 0.000 0.000 0.000 0.066 0.062 0.047 0.058 0.008 0.000

0.000 0.000 0.000 0.008 0.000 0.000 0.000 0.000 0.000 0.000

0.051 0.000 0.008 0.017 0.000 0.000 0.000 0.000 0.062 0.055

0.055 0.068

>Non-Methylation code ID 1440

0.017 0.004 0.000 0.000 0.000 0.004 0.004 0.000 0.000 0.000

0.000 0.000 0.009 0.000 0.009 0.000 0.000 0.000 0.009 0.000

0.060 0.070 0.073 0.073 0.013 0.000 0.004 0.000 0.000 0.008

0.000 0.000 0.000 0.000 0.000 0.000 0.013 0.000 0.013 0.000

0.004 0.000 0.000 0.000 0.056 0.074 0.088 0.061 0.007 0.000

0.000 0.000 0.000 0.021 0.000 0.000 0.000 0.014 0.000 0.000

0.021 0.000 0.007 0.000 0.007 0.000 0.000 0.000 0.058 0.072

0.065 0.062

>Non-Methylation code ID 1440

0.013 0.000 0.000 0.000 0.000 0.000 0.000 0.000 0.000 0.000

0.000 0.000 0.026 0.000 0.017 0.000 0.000 0.000 0.000 0.000

0.063 0.068 0.084 0.063 0.019 0.000 0.000 0.000 0.000 0.000

0.005 0.000 0.000 0.000 0.000 0.000 0.024 0.000 0.014 0.000

0.000 0.000 0.000 0.000 0.079 0.074 0.075 0.044 0.010 0.000

0.000 0.000 0.000 0.000 0.000 0.000 0.000 0.000 0.000 0.000

0.029 0.000 0.015 0.000 0.005 0.000 0.000 0.000 0.058 0.081

0.074 0.063

>Non-Methylation code ID 1440

0.000 0.000 0.000 0.000 0.000 0.004 0.000 0.000 0.000 0.004

0.000 0.000 0.020 0.000 0.016 0.004 0.000 0.004 0.000 0.000

0.069 0.081 0.071 0.060 0.031 0.000 0.000 0.000 0.000 0.008

0.000 0.000 0.000 0.000 0.000 0.000 0.038 0.000 0.008 0.015

0.000 0.000 0.000 0.000 0.062 0.047 0.056 0.068 0.012 0.000

0.000 0.000 0.000 0.000 0.000 0.000 0.000 0.006 0.000 0.000

0.043 0.000 0.012 0.000 0.000 0.000 0.000 0.000 0.072 0.085

0.072 0.031

>Non-Methylation code ID 1440

0.019 0.000 0.000 0.000 0.000 0.013 0.000 0.000 0.000 0.000

0.000 0.000 0.038 0.000 0.006 0.006 0.000 0.000 0.000 0.000

0.062 0.058 0.061 0.071 0.005 0.000 0.000 0.000 0.000 0.005

0.000 0.000 0.000 0.000 0.000 0.000 0.034 0.000 0.015 0.000

0.000 0.005 0.000 0.000 0.076 0.078 0.060 0.055 0.005 0.000

0.000 0.000 0.000 0.010 0.000 0.000 0.000 0.000 0.000 0.000

0.024 0.000 0.015 0.005 0.000 0.000 0.000 0.000 0.084 0.064

0.051 0.076

>Non-Methylation code ID 1440

0.000 0.004 0.004 0.000 0.000 0.009 0.000 0.000 0.000 0.000

0.000 0.000 0.017 0.004 0.013 0.004 0.000 0.000 0.000 0.000

0.055 0.082 0.074 0.065 0.029 0.000 0.000 0.000 0.000 0.014

0.000 0.000 0.000 0.007 0.000 0.000 0.014 0.000 0.014 0.000

0.007 0.000 0.000 0.000 0.041 0.067 0.072 0.067 0.016 0.000

0.000 0.000 0.000 0.008 0.000 0.000 0.000 0.000 0.000 0.000

0.040 0.008 0.008 0.000 0.000 0.000 0.000 0.000 0.068 0.080

0.040 0.066

>Non-Methylation code ID 1440

0.004 0.000 0.000 0.017 0.000 0.012 0.004 0.000 0.000 0.000

0.000 0.000 0.000 0.000 0.017 0.000 0.000 0.000 0.000 0.000

0.069 0.074 0.055 0.081 0.031 0.000 0.000 0.000 0.000 0.006

0.000 0.000 0.012 0.000 0.006 0.000 0.006 0.000 0.006 0.006

0.000 0.000 0.000 0.000 0.061 0.069 0.066 0.063 0.005 0.000

0.000 0.000 0.000 0.010 0.000 0.000 0.000 0.000 0.000 0.000

0.015 0.005 0.015 0.000 0.000 0.000 0.005 0.000 0.056 0.068

0.077 0.079

>Non-Methylation code ID 1440

0.031 0.000 0.000 0.000 0.000 0.008 0.000 0.000 0.000 0.000

0.000 0.000 0.038 0.000 0.008 0.015 0.000 0.000 0.000 0.000

0.062 0.047 0.056 0.068 0.011 0.000 0.000 0.000 0.000 0.000

0.000 0.000 0.000 0.006 0.000 0.000 0.046 0.000 0.011 0.000

0.000 0.000 0.000 0.000 0.067 0.078 0.083 0.032 0.000 0.000

0.000 0.000 0.000 0.004 0.000 0.000 0.000 0.004 0.000 0.000

0.017 0.000 0.017 0.004 0.000 0.004 0.000 0.000 0.073 0.087

0.067 0.054

>Non-Methylation code ID 1440

0.005 0.000 0.000 0.000 0.000 0.000 0.000 0.000 0.000 0.005

0.000 0.000 0.020 0.000 0.015 0.005 0.010 0.000 0.000 0.000

0.042 0.071 0.080 0.081 0.012 0.000 0.000 0.000 0.000 0.000

0.000 0.000 0.000 0.004 0.000 0.000 0.012 0.000 0.016 0.004

0.000 0.000 0.000 0.000 0.041 0.069 0.086 0.088 0.029 0.007

0.007 0.000 0.000 0.000 0.007 0.000 0.000 0.007 0.000 0.000

0.029 0.000 0.000 0.000 0.000 0.000 0.000 0.000 0.054 0.060

0.074 0.059

>Non-Methylation code ID 1440

0.004 0.000 0.000 0.000 0.000 0.012 0.000 0.000 0.000 0.000

0.000 0.000 0.008 0.004 0.024 0.000 0.000 0.000 0.000 0.000

0.075 0.066 0.055 0.084 0.015 0.000 0.010 0.000 0.000 0.020

0.000 0.000 0.005 0.000 0.000 0.000 0.010 0.000 0.005 0.000

0.000 0.000 0.000 0.000 0.080 0.049 0.073 0.065 0.012 0.000

0.000 0.000 0.000 0.019 0.000 0.000 0.000 0.000 0.000 0.000

0.025 0.000 0.006 0.000 0.012 0.000 0.000 0.000 0.068 0.053

0.059 0.078

>Non-Methylation code ID 1440

0.008 0.000 0.000 0.000 0.000 0.012 0.000 0.000 0.000 0.000

0.000 0.000 0.004 0.004 0.024 0.000 0.000 0.000 0.000 0.000

0.075 0.054 0.075 0.078 0.016 0.000 0.010 0.000 0.000 0.021

0.000 0.000 0.005 0.000 0.000 0.000 0.010 0.000 0.005 0.000

0.000 0.000 0.000 0.000 0.082 0.045 0.071 0.068 0.006 0.000

0.000 0.000 0.000 0.017 0.000 0.000 0.000 0.000 0.000 0.000

0.023 0.000 0.012 0.000 0.012 0.000 0.000 0.000 0.077 0.061

0.057 0.068

>Non-Methylation code ID 1440

0.017 0.004 0.000 0.000 0.000 0.000 0.000 0.000 0.000 0.000

0.000 0.000 0.009 0.000 0.013 0.000 0.004 0.000 0.004 0.000

0.064 0.067 0.064 0.088 0.012 0.000 0.004 0.000 0.000 0.004

0.000 0.000 0.000 0.000 0.000 0.000 0.008 0.000 0.024 0.000

0.000 0.000 0.000 0.000 0.072 0.068 0.085 0.056 0.014 0.000

0.007 0.000 0.000 0.021 0.000 0.007 0.000 0.007 0.000 0.000

0.014 0.000 0.007 0.000 0.000 0.000 0.000 0.000 0.058 0.071

0.070 0.059

>Non-Methylation code ID 1440

0.007 0.007 0.000 0.000 0.007 0.020 0.000 0.000 0.000 0.007

0.000 0.007 0.000 0.000 0.007 0.000 0.000 0.000 0.007 0.000

0.068 0.059 0.052 0.088 0.024 0.006 0.000 0.000 0.000 0.006

0.000 0.000 0.000 0.000 0.000 0.000 0.006 0.000 0.000 0.006

0.006 0.000 0.018 0.000 0.077 0.053 0.078 0.052 0.004 0.004

0.000 0.000 0.000 0.013 0.000 0.000 0.000 0.004 0.000 0.000

0.004 0.004 0.013 0.000 0.000 0.000 0.000 0.000 0.057 0.078

0.082 0.070

>Non-Methylation code ID 1440

0.005 0.010 0.000 0.000 0.000 0.005 0.005 0.000 0.000 0.005

0.000 0.000 0.010 0.000 0.005 0.010 0.000 0.005 0.000 0.000

0.049 0.082 0.070 0.072 0.013 0.000 0.000 0.000 0.000 0.004

0.000 0.000 0.000 0.004 0.000 0.000 0.004 0.000 0.013 0.004

0.004 0.000 0.009 0.000 0.077 0.063 0.073 0.063 0.022 0.000

0.000 0.000 0.000 0.043 0.000 0.000 0.000 0.032 0.000 0.000

0.032 0.000 0.000 0.000 0.000 0.000 0.000 0.000 0.037 0.046

0.056 0.065

>Non-Methylation code ID 1440

0.009 0.000 0.000 0.005 0.000 0.009 0.000 0.000 0.000 0.000

0.000 0.000 0.014 0.000 0.014 0.000 0.000 0.009 0.000 0.000

0.063 0.075 0.082 0.053 0.006 0.000 0.000 0.000 0.000 0.018

0.000 0.000 0.006 0.006 0.000 0.000 0.006 0.006 0.018 0.006

0.000 0.000 0.000 0.000 0.062 0.100 0.065 0.036 0.017 0.000

0.000 0.008 0.000 0.025 0.000 0.000 0.000 0.000 0.000 0.000

0.017 0.000 0.000 0.017 0.000 0.000 0.008 0.000 0.071 0.055

0.052 0.062

>Non-Methylation code ID 1440

0.000 0.000 0.000 0.000 0.000 0.005 0.000 0.000 0.000 0.000

0.000 0.005 0.020 0.000 0.020 0.005 0.005 0.000 0.000 0.000

0.092 0.046 0.072 0.063 0.025 0.000 0.006 0.006 0.000 0.006

0.000 0.000 0.000 0.000 0.000 0.000 0.025 0.000 0.006 0.000

0.006 0.000 0.000 0.000 0.069 0.058 0.064 0.060 0.000 0.000

0.007 0.007 0.000 0.000 0.000 0.000 0.000 0.007 0.000 0.000

0.042 0.007 0.007 0.000 0.007 0.000 0.000 0.000 0.068 0.060

0.058 0.063

>Non-Methylation code ID 1440

0.000 0.004 0.000 0.000 0.004 0.004 0.000 0.000 0.004 0.009

0.000 0.004 0.004 0.000 0.004 0.000 0.000 0.000 0.004 0.009

0.077 0.063 0.058 0.082 0.017 0.012 0.000 0.000 0.000 0.006

0.000 0.000 0.000 0.000 0.000 0.000 0.006 0.000 0.006 0.006

0.017 0.000 0.006 0.000 0.068 0.060 0.078 0.052 0.008 0.000

0.000 0.000 0.000 0.008 0.000 0.000 0.000 0.023 0.000 0.000

0.015 0.015 0.008 0.000 0.000 0.000 0.000 0.000 0.068 0.066

0.057 0.065

>Non-Methylation code ID 1440

0.000 0.000 0.000 0.000 0.000 0.025 0.000 0.000 0.000 0.005

0.000 0.000 0.005 0.000 0.015 0.005 0.005 0.000 0.005 0.000

0.088 0.037 0.091 0.054 0.030 0.000 0.000 0.000 0.000 0.018

0.000 0.000 0.000 0.000 0.000 0.000 0.006 0.000 0.006 0.000

0.000 0.012 0.006 0.000 0.062 0.057 0.063 0.073 0.013 0.000

0.000 0.000 0.000 0.020 0.007 0.000 0.000 0.007 0.000 0.000

0.013 0.007 0.007 0.007 0.000 0.000 0.000 0.000 0.064 0.059

0.059 0.072

>Non-Methylation code ID 1440

0.005 0.000 0.000 0.000 0.000 0.000 0.010 0.000 0.000 0.005

0.000 0.000 0.010 0.000 0.010 0.010 0.000 0.005 0.005 0.000

0.076 0.059 0.074 0.066 0.017 0.009 0.000 0.000 0.000 0.009

0.000 0.000 0.000 0.017 0.009 0.000 0.017 0.000 0.000 0.017

0.009 0.009 0.000 0.000 0.066 0.045 0.045 0.064 0.006 0.006

0.000 0.000 0.000 0.006 0.000 0.000 0.000 0.006 0.000 0.000

0.025 0.006 0.006 0.013 0.000 0.000 0.000 0.000 0.061 0.053

0.068 0.076

>Non-Methylation code ID 1440

0.010 0.000 0.005 0.000 0.000 0.000 0.000 0.000 0.000 0.000

0.000 0.000 0.030 0.000 0.010 0.010 0.000 0.000 0.000 0.000

0.073 0.074 0.062 0.059 0.005 0.000 0.005 0.000 0.000 0.005

0.000 0.000 0.000 0.005 0.000 0.000 0.023 0.005 0.009 0.000

0.005 0.000 0.000 0.000 0.049 0.073 0.079 0.074 0.013 0.000

0.000 0.000 0.000 0.007 0.000 0.000 0.000 0.000 0.000 0.000

0.027 0.013 0.007 0.000 0.007 0.000 0.000 0.000 0.064 0.057

0.081 0.058

>Non-Methylation code ID 1440

0.000 0.000 0.000 0.000 0.000 0.021 0.000 0.000 0.000 0.005

0.000 0.000 0.011 0.000 0.011 0.011 0.005 0.000 0.005 0.000

0.077 0.047 0.070 0.071 0.015 0.000 0.000 0.000 0.000 0.015

0.000 0.000 0.000 0.000 0.000 0.000 0.005 0.000 0.010 0.000

0.000 0.015 0.005 0.000 0.078 0.062 0.068 0.061 0.013 0.000

0.000 0.000 0.000 0.019 0.006 0.000 0.000 0.006 0.000 0.000

0.006 0.006 0.006 0.013 0.000 0.000 0.000 0.000 0.062 0.071

0.068 0.056

>Non-Methylation code ID 1440

0.000 0.008 0.004 0.000 0.000 0.008 0.000 0.000 0.000 0.000

0.000 0.000 0.012 0.000 0.012 0.004 0.000 0.000 0.004 0.000

0.066 0.096 0.063 0.057 0.022 0.000 0.005 0.005 0.000 0.011

0.000 0.000 0.000 0.000 0.000 0.000 0.011 0.005 0.005 0.000

0.005 0.000 0.000 0.000 0.064 0.071 0.056 0.072 0.011 0.000

0.000 0.000 0.000 0.000 0.000 0.000 0.000 0.017 0.006 0.000

0.022 0.000 0.011 0.000 0.000 0.000 0.000 0.000 0.077 0.086

0.069 0.036

>Non-Methylation code ID 1440

0.005 0.010 0.000 0.000 0.000 0.005 0.005 0.000 0.000 0.005

0.000 0.000 0.015 0.000 0.005 0.005 0.000 0.005 0.000 0.000

0.047 0.075 0.075 0.078 0.013 0.000 0.000 0.000 0.000 0.004

0.000 0.000 0.000 0.004 0.000 0.000 0.004 0.000 0.013 0.004

0.004 0.000 0.009 0.000 0.069 0.081 0.079 0.046 0.019 0.000

0.000 0.000 0.000 0.028 0.000 0.000 0.000 0.028 0.000 0.000

0.028 0.000 0.000 0.000 0.000 0.000 0.009 0.000 0.063 0.054

0.046 0.059

>Non-Methylation code ID 1440

0.006 0.006 0.000 0.000 0.000 0.006 0.000 0.000 0.000 0.006

0.000 0.000 0.022 0.006 0.006 0.011 0.000 0.000 0.006 0.000

0.062 0.060 0.068 0.071 0.005 0.000 0.000 0.000 0.000 0.005

0.010 0.000 0.000 0.005 0.000 0.000 0.010 0.000 0.010 0.010

0.000 0.005 0.000 0.000 0.073 0.063 0.060 0.080 0.018 0.009

0.000 0.000 0.000 0.000 0.000 0.000 0.000 0.018 0.009 0.000

0.018 0.000 0.000 0.018 0.009 0.009 0.000 0.000 0.063 0.046

0.048 0.067

>Non-Methylation code ID 1440

0.005 0.000 0.000 0.000 0.000 0.005 0.000 0.000 0.000 0.000

0.000 0.000 0.032 0.000 0.019 0.000 0.000 0.000 0.000 0.000

0.061 0.084 0.054 0.073 0.018 0.000 0.000 0.000 0.000 0.012

0.000 0.000 0.000 0.006 0.000 0.000 0.024 0.012 0.006 0.000

0.000 0.000 0.000 0.000 0.065 0.067 0.056 0.067 0.021 0.000

0.000 0.000 0.000 0.000 0.000 0.000 0.000 0.000 0.000 0.000

0.043 0.000 0.007 0.014 0.000 0.000 0.000 0.000 0.061 0.067

0.066 0.054

>Non-Methylation code ID 1440

0.000 0.000 0.000 0.005 0.000 0.010 0.000 0.000 0.000 0.005

0.000 0.000 0.036 0.000 0.010 0.000 0.000 0.000 0.000 0.000

0.068 0.071 0.039 0.088 0.006 0.000 0.000 0.006 0.000 0.006

0.000 0.000 0.000 0.006 0.000 0.000 0.024 0.006 0.006 0.012

0.000 0.000 0.000 0.000 0.066 0.057 0.066 0.074 0.027 0.000

0.000 0.000 0.000 0.000 0.000 0.000 0.000 0.000 0.000 0.000

0.054 0.000 0.009 0.009 0.000 0.000 0.000 0.000 0.055 0.051

0.057 0.072

>Non-Methylation code ID 1440

0.013 0.000 0.000 0.000 0.000 0.013 0.000 0.000 0.000 0.007

0.000 0.000 0.026 0.013 0.007 0.000 0.000 0.000 0.000 0.000

0.067 0.050 0.069 0.069 0.015 0.000 0.005 0.000 0.000 0.000

0.000 0.000 0.000 0.000 0.000 0.000 0.026 0.000 0.010 0.010

0.000 0.000 0.000 0.000 0.070 0.060 0.063 0.074 0.005 0.000

0.000 0.000 0.000 0.005 0.000 0.000 0.000 0.005 0.000 0.000

0.031 0.000 0.010 0.000 0.005 0.000 0.000 0.000 0.040 0.076

0.073 0.082

>Non-Methylation code ID 1440

0.023 0.000 0.000 0.000 0.000 0.000 0.000 0.000 0.000 0.000

0.000 0.000 0.054 0.000 0.008 0.015 0.000 0.000 0.000 0.000

0.066 0.050 0.047 0.070 0.005 0.000 0.000 0.000 0.000 0.005

0.000 0.000 0.000 0.005 0.000 0.000 0.035 0.000 0.015 0.000

0.000 0.000 0.000 0.000 0.070 0.076 0.043 0.080 0.012 0.000

0.000 0.000 0.000 0.006 0.000 0.000 0.000 0.006 0.000 0.000

0.024 0.012 0.006 0.006 0.000 0.000 0.000 0.000 0.068 0.060

0.065 0.069

>Non-Methylation code ID 1440

0.000 0.004 0.000 0.000 0.004 0.004 0.000 0.000 0.004 0.004

0.000 0.009 0.004 0.000 0.004 0.000 0.000 0.000 0.004 0.009

0.076 0.063 0.068 0.073 0.018 0.012 0.000 0.000 0.000 0.006

0.000 0.000 0.000 0.000 0.006 0.000 0.006 0.000 0.006 0.006

0.018 0.000 0.000 0.000 0.075 0.057 0.069 0.053 0.008 0.000

0.000 0.008 0.000 0.000 0.000 0.000 0.000 0.023 0.000 0.000

0.015 0.015 0.008 0.000 0.000 0.000 0.000 0.000 0.065 0.053

0.066 0.073

>Non-Methylation code ID 1443

0.024 0.000 0.006 0.000 0.000 0.006 0.000 0.000 0.000 0.000

0.000 0.000 0.030 0.000 0.006 0.000 0.006 0.000 0.000 0.000

0.067 0.058 0.061 0.070 0.000 0.000 0.007 0.007 0.000 0.000

0.000 0.000 0.000 0.007 0.000 0.000 0.046 0.007 0.007 0.000

0.007 0.000 0.000 0.000 0.069 0.062 0.052 0.065 0.000 0.000

0.000 0.000 0.000 0.006 0.000 0.000 0.000 0.000 0.000 0.006

0.024 0.000 0.018 0.006 0.006 0.000 0.000 0.000 0.098 0.044

0.091 0.037

>Non-Methylation code ID 1443

0.006 0.006 0.000 0.000 0.000 0.006 0.000 0.000 0.000 0.011

0.000 0.000 0.023 0.006 0.006 0.011 0.000 0.000 0.000 0.000

0.057 0.063 0.066 0.072 0.005 0.000 0.000 0.000 0.000 0.000

0.010 0.000 0.000 0.005 0.000 0.000 0.010 0.000 0.010 0.010

0.000 0.005 0.005 0.000 0.076 0.059 0.074 0.066 0.018 0.009

0.000 0.000 0.000 0.009 0.000 0.000 0.000 0.009 0.009 0.000

0.018 0.000 0.000 0.018 0.009 0.009 0.000 0.000 0.058 0.048

0.049 0.068

>Non-Methylation code ID 1443

0.021 0.000 0.000 0.000 0.000 0.000 0.000 0.000 0.000 0.000

0.000 0.000 0.042 0.000 0.014 0.007 0.000 0.000 0.000 0.000

0.043 0.060 0.066 0.079 0.000 0.000 0.000 0.005 0.000 0.010

0.000 0.000 0.000 0.005 0.000 0.000 0.036 0.000 0.010 0.000

0.000 0.000 0.000 0.000 0.068 0.071 0.039 0.088 0.006 0.000

0.000 0.006 0.000 0.006 0.000 0.000 0.000 0.006 0.000 0.000

0.025 0.000 0.006 0.013 0.000 0.000 0.000 0.000 0.065 0.058

0.065 0.076

>Non-Methylation code ID 1443

0.000 0.000 0.000 0.000 0.000 0.010 0.005 0.000 0.000 0.010

0.000 0.000 0.010 0.000 0.010 0.000 0.010 0.005 0.005 0.000

0.079 0.067 0.057 0.067 0.008 0.015 0.000 0.000 0.000 0.030

0.008 0.000 0.000 0.000 0.000 0.000 0.015 0.008 0.000 0.008

0.008 0.000 0.000 0.000 0.058 0.057 0.064 0.056 0.024 0.000

0.000 0.000 0.000 0.012 0.012 0.000 0.006 0.006 0.000 0.000

0.006 0.000 0.006 0.000 0.000 0.000 0.000 0.000 0.073 0.063

0.038 0.088

>Non-Methylation code ID 1443

0.008 0.004 0.000 0.004 0.000 0.000 0.000 0.000 0.004 0.004

0.000 0.000 0.000 0.004 0.017 0.004 0.000 0.004 0.000 0.000

0.089 0.060 0.073 0.057 0.005 0.000 0.000 0.005 0.000 0.009

0.000 0.000 0.000 0.000 0.000 0.000 0.009 0.005 0.014 0.005

0.000 0.005 0.000 0.000 0.071 0.067 0.069 0.068 0.000 0.000

0.000 0.006 0.000 0.012 0.000 0.000 0.012 0.006 0.000 0.000

0.006 0.006 0.006 0.012 0.000 0.006 0.000 0.000 0.077 0.047

0.073 0.065

>Non-Methylation code ID 1443

0.000 0.012 0.000 0.000 0.000 0.017 0.000 0.000 0.000 0.000

0.000 0.000 0.012 0.006 0.006 0.012 0.006 0.006 0.000 0.000

0.062 0.046 0.076 0.073 0.036 0.000 0.000 0.000 0.000 0.014

0.007 0.000 0.007 0.007 0.000 0.000 0.007 0.000 0.007 0.000

0.000 0.000 0.000 0.000 0.068 0.061 0.046 0.072 0.000 0.000

0.000 0.005 0.000 0.005 0.005 0.000 0.005 0.010 0.000 0.000

0.010 0.000 0.005 0.000 0.005 0.005 0.005 0.000 0.071 0.074

0.057 0.070

>Non-Methylation code ID 1443

0.008 0.004 0.000 0.000 0.000 0.000 0.008 0.000 0.004 0.000

0.000 0.000 0.004 0.000 0.008 0.000 0.000 0.000 0.013 0.000

0.066 0.087 0.062 0.066 0.024 0.000 0.000 0.000 0.000 0.024

0.000 0.000 0.000 0.000 0.000 0.008 0.024 0.000 0.000 0.000

0.016 0.000 0.008 0.000 0.059 0.059 0.045 0.066 0.013 0.000

0.000 0.006 0.000 0.006 0.000 0.000 0.000 0.026 0.000 0.000

0.013 0.000 0.006 0.000 0.006 0.000 0.000 0.000 0.062 0.073

0.064 0.057

>Non-Methylation code ID 1443

0.000 0.000 0.006 0.000 0.000 0.017 0.000 0.000 0.006 0.000

0.006 0.000 0.006 0.000 0.006 0.017 0.000 0.006 0.000 0.000

0.053 0.063 0.065 0.082 0.008 0.000 0.000 0.016 0.008 0.008

0.000 0.000 0.000 0.008 0.000 0.008 0.000 0.008 0.000 0.000

0.008 0.000 0.008 0.000 0.066 0.057 0.064 0.069 0.000 0.000

0.005 0.005 0.005 0.005 0.000 0.000 0.010 0.000 0.000 0.000

0.010 0.000 0.000 0.005 0.000 0.000 0.010 0.000 0.078 0.056

0.070 0.073

>Non-Methylation code ID 1443

0.000 0.000 0.000 0.007 0.000 0.014 0.007 0.000 0.000 0.028

0.000 0.000 0.021 0.000 0.000 0.007 0.000 0.000 0.007 0.000

0.065 0.065 0.038 0.077 0.005 0.000 0.000 0.000 0.000 0.010

0.000 0.000 0.000 0.005 0.000 0.005 0.005 0.000 0.010 0.015

0.005 0.000 0.005 0.000 0.076 0.066 0.063 0.064 0.016 0.000

0.000 0.000 0.000 0.016 0.000 0.000 0.000 0.016 0.000 0.000

0.032 0.000 0.000 0.000 0.008 0.000 0.008 0.000 0.048 0.064

0.054 0.072

>Non-Methylation code ID 1443

0.017 0.000 0.000 0.009 0.000 0.017 0.000 0.000 0.000 0.035

0.000 0.000 0.026 0.000 0.000 0.009 0.000 0.000 0.000 0.000

0.060 0.061 0.034 0.066 0.005 0.000 0.000 0.000 0.000 0.005

0.000 0.000 0.000 0.005 0.000 0.005 0.010 0.005 0.010 0.015

0.000 0.000 0.005 0.000 0.080 0.069 0.061 0.060 0.016 0.000

0.000 0.000 0.000 0.016 0.000 0.000 0.000 0.008 0.000 0.000

0.040 0.000 0.000 0.008 0.000 0.000 0.008 0.000 0.049 0.070

0.051 0.068

>Non-Methylation code ID 1443

0.006 0.000 0.000 0.006 0.000 0.012 0.000 0.000 0.000 0.006

0.000 0.006 0.000 0.006 0.006 0.006 0.006 0.000 0.012 0.006

0.083 0.070 0.051 0.050 0.015 0.000 0.000 0.000 0.000 0.031

0.000 0.000 0.008 0.015 0.000 0.000 0.000 0.000 0.008 0.000

0.015 0.000 0.000 0.000 0.071 0.066 0.039 0.066 0.000 0.000

0.000 0.012 0.000 0.018 0.006 0.000 0.000 0.012 0.000 0.000

0.012 0.000 0.000 0.000 0.000 0.006 0.006 0.000 0.056 0.067

0.070 0.068

>Non-Methylation code ID 1443

0.000 0.000 0.000 0.000 0.000 0.022 0.000 0.000 0.000 0.022

0.000 0.011 0.000 0.011 0.000 0.033 0.011 0.022 0.000 0.000

0.038 0.044 0.056 0.065 0.012 0.006 0.006 0.006 0.006 0.006

0.000 0.000 0.006 0.012 0.000 0.000 0.000 0.000 0.000 0.006

0.006 0.000 0.000 0.006 0.065 0.058 0.074 0.061 0.000 0.000

0.000 0.000 0.006 0.006 0.006 0.000 0.012 0.000 0.000 0.000

0.006 0.006 0.000 0.000 0.012 0.000 0.012 0.000 0.059 0.064

0.067 0.076

>Non-Methylation code ID 1443

0.000 0.007 0.000 0.000 0.000 0.007 0.014 0.000 0.000 0.007

0.000 0.014 0.007 0.000 0.000 0.000 0.028 0.000 0.000 0.007

0.062 0.054 0.064 0.063 0.007 0.000 0.000 0.000 0.000 0.007

0.028 0.007 0.000 0.000 0.007 0.000 0.000 0.000 0.007 0.007

0.021 0.000 0.000 0.000 0.079 0.042 0.087 0.035 0.008 0.000

0.000 0.008 0.000 0.000 0.033 0.008 0.000 0.000 0.000 0.000

0.000 0.000 0.000 0.000 0.025 0.000 0.008 0.000 0.072 0.043

0.084 0.043

>Non-Methylation code ID 1443

0.000 0.007 0.000 0.000 0.000 0.000 0.000 0.000 0.000 0.007

0.000 0.000 0.044 0.007 0.007 0.022 0.000 0.000 0.000 0.000

0.062 0.061 0.045 0.070 0.012 0.000 0.000 0.000 0.000 0.000

0.000 0.000 0.000 0.012 0.000 0.000 0.037 0.000 0.006 0.012

0.000 0.000 0.000 0.000 0.065 0.057 0.051 0.081 0.019 0.000

0.000 0.000 0.000 0.000 0.000 0.000 0.000 0.038 0.000 0.000

0.038 0.000 0.000 0.019 0.000 0.000 0.000 0.000 0.054 0.049

0.044 0.072

>Non-Methylation code ID 1443

0.020 0.005 0.000 0.000 0.000 0.000 0.005 0.000 0.000 0.000

0.000 0.000 0.015 0.000 0.010 0.005 0.005 0.000 0.000 0.000

0.082 0.069 0.071 0.047 0.008 0.000 0.000 0.000 0.000 0.004

0.004 0.000 0.000 0.004 0.004 0.000 0.017 0.000 0.013 0.000

0.000 0.000 0.000 0.000 0.082 0.058 0.072 0.066 0.009 0.017

0.000 0.000 0.000 0.009 0.009 0.000 0.000 0.000 0.009 0.000

0.043 0.000 0.000 0.000 0.000 0.009 0.000 0.000 0.057 0.055

0.052 0.064

>Non-Methylation code ID 1443

0.007 0.000 0.000 0.000 0.000 0.000 0.007 0.000 0.000 0.013

0.000 0.000 0.026 0.000 0.007 0.020 0.000 0.007 0.000 0.000

0.072 0.048 0.051 0.076 0.019 0.000 0.000 0.000 0.000 0.000

0.000 0.000 0.000 0.039 0.000 0.000 0.029 0.000 0.000 0.029

0.010 0.000 0.000 0.000 0.055 0.040 0.051 0.061 0.000 0.018

0.000 0.000 0.000 0.000 0.000 0.000 0.000 0.018 0.000 0.000

0.044 0.009 0.000 0.018 0.000 0.000 0.000 0.000 0.059 0.062

0.051 0.056

>Non-Methylation code ID 1443

0.005 0.005 0.005 0.000 0.005 0.005 0.000 0.000 0.005 0.005

0.000 0.000 0.000 0.000 0.005 0.005 0.011 0.000 0.000 0.005

0.065 0.061 0.078 0.067 0.000 0.006 0.000 0.000 0.006 0.006

0.006 0.000 0.006 0.000 0.000 0.000 0.006 0.006 0.006 0.000

0.011 0.000 0.006 0.000 0.062 0.059 0.073 0.078 0.009 0.009

0.000 0.000 0.000 0.009 0.000 0.000 0.000 0.018 0.000 0.009

0.000 0.009 0.000 0.009 0.009 0.018 0.000 0.000 0.047 0.049

0.063 0.074

>Non-Methylation code ID 1443

0.015 0.000 0.000 0.000 0.000 0.023 0.000 0.000 0.000 0.015

0.000 0.000 0.023 0.000 0.000 0.008 0.008 0.000 0.008 0.000

0.048 0.062 0.059 0.066 0.015 0.005 0.000 0.005 0.000 0.005

0.000 0.000 0.000 0.015 0.000 0.000 0.010 0.000 0.005 0.000

0.000 0.000 0.005 0.000 0.066 0.067 0.067 0.069 0.009 0.000

0.000 0.000 0.000 0.005 0.005 0.000 0.005 0.000 0.000 0.000

0.009 0.000 0.009 0.000 0.000 0.000 0.009 0.000 0.064 0.079

0.053 0.086

>Non-Methylation code ID 1443

0.006 0.000 0.000 0.000 0.000 0.000 0.000 0.000 0.000 0.013

0.000 0.000 0.025 0.006 0.006 0.025 0.000 0.000 0.000 0.000

0.063 0.059 0.051 0.078 0.006 0.000 0.000 0.000 0.000 0.000

0.000 0.000 0.000 0.006 0.000 0.000 0.043 0.000 0.006 0.012

0.000 0.000 0.006 0.000 0.068 0.070 0.054 0.062 0.020 0.000

0.000 0.000 0.000 0.010 0.000 0.000 0.000 0.041 0.000 0.000

0.041 0.000 0.000 0.010 0.000 0.000 0.000 0.000 0.050 0.059

0.034 0.067

>Non-Methylation code ID 1443

0.000 0.000 0.000 0.012 0.000 0.006 0.000 0.000 0.006 0.006

0.000 0.000 0.006 0.006 0.006 0.012 0.006 0.006 0.006 0.000

0.069 0.048 0.076 0.062 0.009 0.005 0.000 0.005 0.000 0.005

0.000 0.000 0.009 0.005 0.000 0.000 0.000 0.005 0.014 0.005

0.000 0.000 0.000 0.000 0.089 0.047 0.097 0.041 0.005 0.000

0.000 0.005 0.000 0.010 0.000 0.000 0.000 0.000 0.000 0.005

0.010 0.005 0.010 0.005 0.000 0.005 0.000 0.000 0.058 0.082

0.064 0.070

>Non-Methylation code ID 1443

0.020 0.005 0.000 0.000 0.000 0.000 0.005 0.000 0.000 0.000

0.000 0.000 0.010 0.000 0.010 0.005 0.010 0.000 0.000 0.000

0.083 0.064 0.070 0.049 0.009 0.000 0.000 0.000 0.000 0.005

0.005 0.000 0.000 0.009 0.005 0.000 0.018 0.000 0.009 0.000

0.000 0.000 0.000 0.000 0.083 0.052 0.078 0.061 0.008 0.024

0.000 0.000 0.000 0.008 0.008 0.000 0.000 0.000 0.008 0.000

0.032 0.000 0.000 0.000 0.000 0.008 0.000 0.000 0.060 0.064

0.057 0.056

>Non-Methylation code ID 1443

0.012 0.000 0.000 0.000 0.000 0.000 0.000 0.000 0.000 0.012

0.000 0.000 0.037 0.000 0.006 0.012 0.000 0.000 0.000 0.000

0.065 0.057 0.051 0.081 0.018 0.000 0.000 0.000 0.000 0.000

0.000 0.000 0.000 0.045 0.000 0.000 0.036 0.000 0.000 0.018

0.000 0.000 0.000 0.000 0.058 0.045 0.043 0.069 0.000 0.008

0.000 0.000 0.000 0.000 0.000 0.000 0.000 0.008 0.000 0.000

0.038 0.008 0.008 0.023 0.000 0.000 0.000 0.000 0.058 0.064

0.046 0.075

>Non-Methylation code ID 1443

0.011 0.000 0.000 0.005 0.000 0.005 0.000 0.000 0.000 0.005

0.000 0.000 0.011 0.005 0.011 0.005 0.000 0.005 0.000 0.000

0.064 0.073 0.078 0.054 0.000 0.000 0.000 0.005 0.000 0.009

0.000 0.000 0.009 0.005 0.000 0.000 0.009 0.005 0.005 0.009

0.000 0.000 0.005 0.000 0.071 0.057 0.070 0.074 0.009 0.004

0.000 0.004 0.000 0.000 0.000 0.000 0.004 0.004 0.000 0.000

0.004 0.000 0.013 0.004 0.000 0.004 0.000 0.000 0.083 0.073

0.058 0.068

>Non-Methylation code ID 1443

0.007 0.000 0.000 0.000 0.000 0.000 0.000 0.000 0.000 0.013

0.000 0.000 0.033 0.000 0.007 0.020 0.000 0.007 0.000 0.000

0.073 0.058 0.042 0.074 0.019 0.000 0.000 0.000 0.000 0.000

0.000 0.000 0.000 0.047 0.000 0.000 0.028 0.000 0.000 0.028

0.000 0.000 0.000 0.000 0.053 0.042 0.046 0.069 0.000 0.018

0.000 0.000 0.000 0.000 0.000 0.000 0.000 0.009 0.000 0.000

0.054 0.009 0.000 0.018 0.000 0.000 0.000 0.000 0.056 0.064

0.047 0.058

>Non-Methylation code ID 1443

0.008 0.000 0.000 0.000 0.000 0.008 0.016 0.008 0.000 0.016

0.008 0.000 0.024 0.000 0.000 0.008 0.000 0.008 0.000 0.000

0.067 0.050 0.058 0.056 0.010 0.000 0.000 0.000 0.000 0.000

0.000 0.010 0.000 0.030 0.000 0.000 0.020 0.000 0.000 0.030

0.010 0.000 0.010 0.000 0.073 0.033 0.052 0.054 0.000 0.015

0.000 0.000 0.000 0.008 0.000 0.000 0.000 0.008 0.000 0.008

0.038 0.000 0.000 0.008 0.000 0.000 0.000 0.008 0.059 0.064

0.055 0.065

>Non-Methylation code ID 1443

0.008 0.000 0.000 0.000 0.000 0.008 0.008 0.000 0.000 0.016

0.008 0.000 0.032 0.000 0.000 0.016 0.000 0.008 0.000 0.000

0.072 0.045 0.044 0.067 0.009 0.000 0.000 0.000 0.000 0.000

0.000 0.000 0.000 0.035 0.000 0.000 0.027 0.000 0.000 0.027

0.009 0.000 0.009 0.000 0.066 0.042 0.045 0.065 0.000 0.018

0.000 0.000 0.000 0.009 0.000 0.000 0.000 0.018 0.000 0.000

0.054 0.000 0.000 0.009 0.000 0.000 0.000 0.000 0.064 0.055

0.053 0.054

>Non-Methylation code ID 1443

0.007 0.022 0.000 0.000 0.000 0.007 0.015 0.000 0.000 0.000

0.007 0.000 0.029 0.000 0.000 0.000 0.000 0.007 0.000 0.000

0.065 0.063 0.051 0.060 0.020 0.005 0.000 0.000 0.000 0.000

0.005 0.000 0.000 0.000 0.000 0.000 0.010 0.000 0.010 0.005

0.010 0.000 0.000 0.000 0.083 0.064 0.070 0.049 0.010 0.000

0.000 0.000 0.000 0.005 0.005 0.000 0.000 0.010 0.005 0.000

0.015 0.000 0.010 0.000 0.000 0.000 0.000 0.000 0.086 0.056

0.081 0.051

>Non-Methylation code ID 1443

0.015 0.000 0.000 0.000 0.000 0.023 0.000 0.000 0.000 0.015

0.000 0.000 0.031 0.000 0.000 0.000 0.008 0.000 0.008 0.000

0.049 0.065 0.051 0.068 0.000 0.000 0.000 0.007 0.000 0.013

0.007 0.000 0.000 0.020 0.000 0.000 0.020 0.000 0.000 0.007

0.000 0.007 0.007 0.000 0.063 0.071 0.046 0.067 0.005 0.000

0.000 0.000 0.000 0.010 0.000 0.000 0.000 0.005 0.000 0.005

0.005 0.000 0.010 0.010 0.005 0.000 0.005 0.000 0.070 0.070

0.068 0.063

>Non-Methylation code ID 1443

0.000 0.000 0.007 0.000 0.000 0.014 0.000 0.000 0.000 0.000

0.007 0.000 0.007 0.000 0.007 0.029 0.007 0.007 0.000 0.000

0.056 0.062 0.056 0.074 0.016 0.000 0.000 0.016 0.008 0.008

0.000 0.000 0.000 0.016 0.000 0.000 0.000 0.008 0.000 0.000

0.008 0.000 0.008 0.000 0.059 0.043 0.076 0.065 0.000 0.000

0.005 0.000 0.005 0.005 0.005 0.000 0.010 0.000 0.000 0.000

0.010 0.000 0.000 0.005 0.000 0.000 0.010 0.000 0.074 0.054

0.074 0.075

>Non-Methylation code ID 1445

0.009 0.000 0.000 0.000 0.000 0.004 0.000 0.000 0.004 0.004

0.000 0.000 0.009 0.000 0.013 0.000 0.000 0.000 0.009 0.000

0.066 0.086 0.045 0.083 0.016 0.000 0.000 0.000 0.000 0.031

0.000 0.000 0.000 0.016 0.000 0.000 0.023 0.000 0.000 0.008

0.000 0.000 0.008 0.000 0.049 0.060 0.060 0.063 0.021 0.005

0.000 0.005 0.000 0.005 0.000 0.000 0.000 0.010 0.000 0.000

0.005 0.000 0.005 0.000 0.000 0.000 0.005 0.000 0.072 0.069

0.065 0.065

>Non-Methylation code ID 1445

0.013 0.000 0.000 0.000 0.000 0.013 0.000 0.000 0.000 0.006

0.000 0.000 0.031 0.000 0.006 0.006 0.000 0.000 0.006 0.000

0.049 0.071 0.058 0.073 0.017 0.000 0.000 0.009 0.000 0.017

0.000 0.000 0.000 0.035 0.000 0.000 0.026 0.000 0.000 0.009

0.000 0.000 0.000 0.000 0.060 0.061 0.034 0.066 0.005 0.000

0.000 0.000 0.000 0.005 0.000 0.000 0.000 0.005 0.000 0.000

0.011 0.005 0.011 0.016 0.000 0.000 0.005 0.000 0.078 0.069

0.064 0.059

>Non-Methylation code ID 1445

0.013 0.000 0.000 0.000 0.000 0.009 0.000 0.000 0.000 0.009

0.004 0.000 0.009 0.000 0.013 0.000 0.000 0.000 0.000 0.000

0.085 0.067 0.057 0.067 0.018 0.006 0.000 0.006 0.000 0.006

0.000 0.000 0.000 0.000 0.000 0.000 0.018 0.006 0.006 0.000

0.000 0.000 0.006 0.000 0.068 0.065 0.053 0.077 0.012 0.000

0.006 0.000 0.000 0.006 0.000 0.000 0.006 0.006 0.000 0.000

0.017 0.000 0.006 0.012 0.000 0.000 0.000 0.000 0.067 0.064

0.079 0.055

>Non-Methylation code ID 1445

0.000 0.011 0.000 0.006 0.000 0.017 0.000 0.000 0.000 0.000

0.000 0.000 0.011 0.000 0.006 0.011 0.006 0.006 0.000 0.000

0.064 0.050 0.075 0.072 0.031 0.000 0.000 0.000 0.000 0.012

0.006 0.000 0.000 0.006 0.000 0.000 0.006 0.000 0.012 0.000

0.000 0.000 0.000 0.000 0.070 0.060 0.058 0.071 0.000 0.000

0.000 0.005 0.000 0.010 0.005 0.000 0.005 0.010 0.000 0.000

0.010 0.000 0.005 0.000 0.000 0.005 0.005 0.000 0.073 0.075

0.058 0.068

>Non-Methylation code ID 1445

0.000 0.010 0.000 0.000 0.000 0.000 0.015 0.000 0.005 0.000

0.005 0.000 0.010 0.000 0.010 0.000 0.000 0.005 0.005 0.000

0.061 0.079 0.067 0.059 0.025 0.006 0.000 0.000 0.000 0.006

0.006 0.000 0.000 0.000 0.000 0.006 0.000 0.000 0.006 0.006

0.019 0.000 0.000 0.000 0.066 0.056 0.057 0.073 0.006 0.000

0.000 0.006 0.000 0.000 0.006 0.000 0.000 0.012 0.006 0.000

0.023 0.000 0.006 0.000 0.006 0.000 0.000 0.000 0.075 0.051

0.074 0.062

>Non-Methylation code ID 1445

0.015 0.000 0.000 0.007 0.000 0.007 0.015 0.007 0.000 0.015

0.000 0.000 0.007 0.000 0.000 0.022 0.000 0.000 0.000 0.000

0.065 0.054 0.056 0.063 0.014 0.005 0.000 0.000 0.000 0.000

0.005 0.000 0.000 0.000 0.000 0.000 0.009 0.000 0.009 0.005

0.009 0.000 0.005 0.000 0.072 0.048 0.074 0.078 0.012 0.000

0.006 0.000 0.000 0.006 0.000 0.000 0.000 0.006 0.000 0.000

0.012 0.012 0.012 0.000 0.006 0.000 0.000 0.000 0.074 0.045

0.071 0.069

>Non-Methylation code ID 1445

0.006 0.000 0.000 0.000 0.000 0.006 0.000 0.006 0.000 0.000

0.006 0.000 0.011 0.006 0.017 0.011 0.000 0.000 0.000 0.000

0.041 0.064 0.082 0.080 0.004 0.004 0.000 0.004 0.004 0.008

0.000 0.000 0.000 0.004 0.000 0.000 0.012 0.000 0.008 0.004

0.000 0.000 0.000 0.000 0.064 0.077 0.067 0.071 0.012 0.000

0.006 0.000 0.000 0.006 0.000 0.000 0.000 0.006 0.000 0.006

0.012 0.000 0.006 0.006 0.000 0.012 0.000 0.000 0.061 0.063

0.059 0.079

>Non-Methylation code ID 1445

0.023 0.005 0.000 0.000 0.009 0.000 0.005 0.000 0.000 0.000

0.000 0.000 0.005 0.005 0.005 0.000 0.000 0.005 0.000 0.000

0.059 0.073 0.073 0.069 0.012 0.000 0.000 0.000 0.000 0.000

0.006 0.000 0.000 0.012 0.000 0.000 0.000 0.000 0.018 0.025

0.006 0.000 0.000 0.000 0.064 0.069 0.062 0.058 0.004 0.004

0.000 0.000 0.000 0.009 0.000 0.000 0.000 0.009 0.000 0.000

0.004 0.000 0.013 0.000 0.004 0.004 0.000 0.000 0.082 0.058

0.053 0.089

>Non-Methylation code ID 1445

0.005 0.005 0.000 0.000 0.000 0.005 0.000 0.000 0.000 0.005

0.000 0.005 0.014 0.000 0.009 0.000 0.005 0.000 0.005 0.000

0.080 0.070 0.049 0.081 0.006 0.000 0.006 0.006 0.006 0.013

0.006 0.000 0.000 0.006 0.000 0.000 0.006 0.013 0.000 0.000

0.006 0.006 0.000 0.000 0.061 0.068 0.063 0.059 0.008 0.000

0.000 0.000 0.000 0.000 0.000 0.000 0.008 0.000 0.008 0.008

0.008 0.000 0.008 0.033 0.008 0.000 0.000 0.000 0.054 0.049

0.068 0.070

>Non-Methylation code ID 1445

0.007 0.000 0.000 0.000 0.007 0.007 0.007 0.007 0.000 0.015

0.000 0.000 0.022 0.000 0.000 0.022 0.000 0.000 0.000 0.000

0.071 0.053 0.061 0.054 0.017 0.000 0.000 0.000 0.017 0.000

0.000 0.000 0.000 0.017 0.000 0.000 0.026 0.009 0.000 0.009

0.009 0.000 0.000 0.000 0.069 0.047 0.056 0.060 0.000 0.000

0.000 0.000 0.005 0.000 0.000 0.000 0.005 0.011 0.000 0.005

0.016 0.000 0.005 0.016 0.000 0.000 0.000 0.000 0.037 0.065

0.078 0.090

>Non-Methylation code ID 1445

0.004 0.004 0.000 0.004 0.000 0.000 0.004 0.000 0.000 0.000

0.000 0.000 0.012 0.000 0.008 0.008 0.004 0.000 0.004 0.000

0.061 0.057 0.071 0.090 0.011 0.000 0.005 0.000 0.000 0.005

0.000 0.000 0.000 0.005 0.000 0.000 0.005 0.005 0.016 0.005

0.005 0.000 0.000 0.000 0.066 0.058 0.055 0.089 0.027 0.000

0.000 0.009 0.000 0.000 0.009 0.009 0.000 0.018 0.000 0.000

0.009 0.000 0.000 0.018 0.000 0.000 0.000 0.000 0.057 0.063

0.060 0.054

>Non-Methylation code ID 1445

0.009 0.000 0.004 0.000 0.004 0.004 0.000 0.000 0.000 0.000

0.000 0.004 0.009 0.004 0.009 0.004 0.000 0.004 0.000 0.000

0.066 0.071 0.072 0.069 0.011 0.000 0.000 0.000 0.000 0.005

0.000 0.005 0.000 0.005 0.005 0.000 0.011 0.000 0.011 0.016

0.000 0.000 0.000 0.000 0.041 0.071 0.074 0.078 0.005 0.005

0.000 0.005 0.005 0.005 0.000 0.000 0.000 0.000 0.000 0.000

0.019 0.000 0.009 0.000 0.000 0.005 0.000 0.000 0.069 0.071

0.063 0.074

>Non-Methylation code ID 1445

0.000 0.005 0.005 0.000 0.000 0.005 0.000 0.000 0.009 0.009

0.000 0.000 0.000 0.000 0.014 0.005 0.000 0.000 0.000 0.000

0.082 0.054 0.093 0.052 0.033 0.011 0.000 0.000 0.000 0.022

0.000 0.011 0.000 0.011 0.000 0.000 0.000 0.000 0.000 0.033

0.000 0.011 0.000 0.000 0.050 0.060 0.046 0.046 0.016 0.000

0.000 0.008 0.000 0.016 0.000 0.000 0.000 0.000 0.000 0.000

0.008 0.016 0.008 0.016 0.000 0.000 0.000 0.000 0.060 0.060

0.061 0.063

>Non-Methylation code ID 1445

0.014 0.000 0.000 0.000 0.014 0.000 0.000 0.007 0.007 0.007

0.000 0.000 0.014 0.007 0.000 0.007 0.007 0.000 0.000 0.000

0.064 0.059 0.056 0.067 0.000 0.000 0.000 0.000 0.005 0.000

0.000 0.000 0.005 0.011 0.000 0.005 0.016 0.000 0.005 0.016

0.000 0.000 0.000 0.000 0.039 0.067 0.077 0.086 0.007 0.000

0.000 0.000 0.007 0.007 0.007 0.007 0.007 0.014 0.000 0.000

0.014 0.000 0.000 0.014 0.000 0.000 0.000 0.000 0.069 0.061

0.054 0.065

>Non-Methylation code ID 1445

0.000 0.006 0.000 0.000 0.006 0.012 0.012 0.000 0.000 0.024

0.000 0.000 0.000 0.000 0.006 0.000 0.006 0.000 0.000 0.000

0.076 0.074 0.053 0.057 0.009 0.000 0.000 0.000 0.000 0.017

0.000 0.000 0.000 0.000 0.000 0.009 0.000 0.000 0.009 0.017

0.017 0.000 0.000 0.017 0.069 0.065 0.055 0.051 0.012 0.000

0.000 0.006 0.000 0.000 0.006 0.012 0.000 0.000 0.000 0.000

0.018 0.000 0.006 0.000 0.006 0.000 0.006 0.000 0.062 0.078

0.062 0.060

>Non-Methylation code ID 1445

0.014 0.000 0.005 0.010 0.000 0.000 0.000 0.000 0.000 0.000

0.000 0.000 0.000 0.000 0.014 0.005 0.005 0.005 0.000 0.000

0.061 0.059 0.066 0.089 0.019 0.000 0.000 0.006 0.000 0.000

0.000 0.006 0.000 0.013 0.000 0.000 0.006 0.006 0.019 0.000

0.000 0.000 0.000 0.000 0.050 0.056 0.064 0.087 0.000 0.006

0.000 0.006 0.000 0.017 0.006 0.000 0.000 0.000 0.000 0.000

0.011 0.000 0.011 0.011 0.000 0.000 0.000 0.000 0.068 0.058

0.073 0.066

>Non-Methylation code ID 1445

0.013 0.000 0.006 0.000 0.013 0.013 0.006 0.000 0.000 0.006

0.000 0.000 0.006 0.013 0.000 0.000 0.000 0.006 0.000 0.000

0.058 0.067 0.064 0.061 0.008 0.000 0.000 0.000 0.000 0.000

0.000 0.000 0.000 0.008 0.008 0.008 0.008 0.000 0.008 0.041

0.008 0.000 0.000 0.000 0.068 0.064 0.053 0.050 0.004 0.004

0.000 0.000 0.000 0.004 0.000 0.000 0.000 0.004 0.000 0.000

0.012 0.000 0.012 0.000 0.004 0.000 0.004 0.000 0.079 0.071

0.050 0.084

>Non-Methylation code ID 1445

0.008 0.000 0.000 0.000 0.000 0.016 0.000 0.008 0.000 0.000

0.000 0.008 0.000 0.000 0.008 0.024 0.008 0.000 0.000 0.008

0.057 0.080 0.061 0.048 0.011 0.000 0.000 0.022 0.000 0.000

0.000 0.006 0.000 0.011 0.000 0.000 0.011 0.000 0.006 0.000

0.006 0.000 0.000 0.000 0.064 0.059 0.064 0.074 0.000 0.005

0.000 0.000 0.005 0.005 0.005 0.000 0.005 0.021 0.000 0.005

0.000 0.005 0.005 0.000 0.000 0.000 0.000 0.000 0.081 0.051

0.075 0.063

>Non-Methylation code ID 1445

0.019 0.000 0.006 0.000 0.013 0.006 0.006 0.000 0.000 0.006

0.000 0.000 0.006 0.013 0.000 0.000 0.000 0.006 0.000 0.000

0.064 0.065 0.064 0.058 0.007 0.000 0.000 0.000 0.000 0.000

0.007 0.000 0.000 0.007 0.000 0.007 0.007 0.000 0.007 0.037

0.015 0.000 0.000 0.000 0.059 0.062 0.063 0.053 0.004 0.004

0.000 0.000 0.000 0.004 0.000 0.000 0.000 0.008 0.000 0.000

0.013 0.000 0.013 0.000 0.004 0.000 0.000 0.000 0.086 0.072

0.045 0.080

>Non-Methylation code ID 1445

0.016 0.000 0.000 0.000 0.000 0.000 0.005 0.000 0.000 0.005

0.005 0.000 0.005 0.000 0.011 0.016 0.005 0.000 0.000 0.000

0.047 0.071 0.070 0.076 0.004 0.004 0.000 0.000 0.000 0.004

0.000 0.000 0.000 0.009 0.000 0.000 0.013 0.000 0.013 0.000

0.004 0.004 0.000 0.000 0.081 0.055 0.064 0.075 0.013 0.004

0.004 0.000 0.004 0.000 0.004 0.000 0.000 0.000 0.000 0.000

0.004 0.004 0.008 0.000 0.000 0.004 0.000 0.000 0.060 0.069

0.078 0.077

>Non-Methylation code ID 1445

0.009 0.004 0.000 0.000 0.000 0.000 0.004 0.000 0.000 0.000

0.000 0.000 0.013 0.000 0.009 0.004 0.009 0.000 0.004 0.000

0.066 0.057 0.066 0.088 0.011 0.000 0.006 0.000 0.000 0.006

0.000 0.000 0.000 0.006 0.000 0.000 0.011 0.011 0.017 0.000

0.006 0.000 0.000 0.000 0.066 0.052 0.063 0.080 0.015 0.000

0.000 0.008 0.000 0.008 0.015 0.008 0.000 0.015 0.000 0.000

0.008 0.000 0.000 0.015 0.000 0.000 0.000 0.000 0.063 0.055

0.056 0.068

>Non-Methylation code ID 1445

0.007 0.000 0.000 0.000 0.000 0.007 0.014 0.007 0.000 0.014

0.000 0.000 0.007 0.000 0.007 0.021 0.000 0.000 0.000 0.000

0.059 0.052 0.069 0.070 0.016 0.005 0.005 0.000 0.000 0.000

0.005 0.000 0.000 0.000 0.000 0.000 0.005 0.000 0.005 0.005

0.011 0.000 0.005 0.000 0.068 0.055 0.071 0.076 0.012 0.000

0.000 0.006 0.000 0.006 0.000 0.000 0.000 0.006 0.000 0.000

0.012 0.012 0.012 0.000 0.006 0.000 0.000 0.000 0.065 0.054

0.071 0.072

>Non-Methylation code ID 1445

0.014 0.000 0.000 0.000 0.000 0.000 0.007 0.000 0.000 0.014

0.000 0.000 0.000 0.000 0.014 0.034 0.007 0.000 0.000 0.000

0.071 0.069 0.057 0.048 0.004 0.004 0.000 0.000 0.000 0.004

0.000 0.000 0.000 0.012 0.000 0.000 0.008 0.000 0.012 0.000

0.004 0.004 0.000 0.000 0.081 0.052 0.065 0.083 0.021 0.005

0.000 0.000 0.011 0.000 0.005 0.000 0.000 0.005 0.000 0.000

0.000 0.005 0.005 0.000 0.000 0.005 0.000 0.000 0.053 0.068

0.077 0.072

>Non-Methylation code ID 1445

0.005 0.005 0.000 0.000 0.000 0.005 0.005 0.005 0.010 0.010

0.000 0.000 0.000 0.005 0.005 0.005 0.005 0.000 0.000 0.000

0.081 0.073 0.045 0.069 0.006 0.000 0.006 0.000 0.000 0.000

0.000 0.000 0.012 0.000 0.000 0.006 0.006 0.006 0.006 0.000

0.006 0.000 0.000 0.012 0.072 0.058 0.072 0.068 0.007 0.000

0.000 0.000 0.000 0.000 0.007 0.007 0.014 0.000 0.007 0.007

0.007 0.007 0.000 0.007 0.007 0.000 0.007 0.000 0.062 0.063

0.055 0.069

>Non-Methylation code ID 1445

0.007 0.000 0.000 0.000 0.000 0.007 0.000 0.000 0.007 0.014

0.000 0.000 0.029 0.000 0.007 0.014 0.000 0.000 0.000 0.000

0.045 0.069 0.067 0.066 0.007 0.007 0.000 0.007 0.000 0.013

0.007 0.000 0.000 0.013 0.000 0.000 0.020 0.000 0.000 0.013

0.000 0.000 0.000 0.000 0.061 0.063 0.066 0.058 0.014 0.007

0.000 0.000 0.000 0.000 0.000 0.000 0.007 0.007 0.000 0.000

0.021 0.000 0.007 0.007 0.007 0.000 0.000 0.000 0.038 0.069

0.077 0.073

>Non-Methylation code ID 1445

0.000 0.004 0.004 0.004 0.004 0.004 0.000 0.000 0.004 0.008

0.000 0.000 0.004 0.000 0.012 0.004 0.000 0.000 0.000 0.000

0.085 0.063 0.089 0.045 0.031 0.000 0.000 0.000 0.000 0.021

0.000 0.010 0.000 0.010 0.000 0.010 0.010 0.000 0.000 0.031

0.000 0.010 0.000 0.000 0.047 0.055 0.045 0.050 0.007 0.000

0.000 0.000 0.000 0.013 0.000 0.007 0.000 0.000 0.000 0.000

0.013 0.013 0.007 0.013 0.000 0.000 0.000 0.000 0.046 0.065

0.078 0.070

>Non-Methylation code ID 1447

0.017 0.006 0.006 0.000 0.011 0.006 0.006 0.000 0.000 0.006

0.000 0.000 0.006 0.006 0.000 0.000 0.000 0.006 0.000 0.000

0.062 0.063 0.077 0.058 0.015 0.000 0.000 0.000 0.000 0.000

0.008 0.000 0.000 0.008 0.000 0.000 0.008 0.000 0.008 0.039

0.015 0.000 0.000 0.000 0.064 0.055 0.062 0.051 0.004 0.004

0.000 0.000 0.000 0.004 0.000 0.000 0.000 0.008 0.000 0.000

0.008 0.000 0.016 0.000 0.004 0.000 0.000 0.000 0.080 0.078

0.043 0.083

>Non-Methylation code ID 1447

0.000 0.000 0.000 0.011 0.000 0.005 0.000 0.000 0.005 0.011

0.005 0.000 0.011 0.005 0.005 0.000 0.011 0.000 0.000 0.000

0.071 0.077 0.037 0.078 0.004 0.008 0.000 0.004 0.000 0.000

0.000 0.000 0.000 0.000 0.000 0.000 0.008 0.004 0.012 0.008

0.000 0.000 0.004 0.000 0.085 0.064 0.055 0.077 0.020 0.000

0.010 0.000 0.000 0.005 0.005 0.000 0.005 0.000 0.000 0.000

0.010 0.000 0.005 0.000 0.000 0.000 0.000 0.000 0.051 0.079

0.071 0.072

>Non-Methylation code ID 1447

0.000 0.000 0.000 0.006 0.000 0.006 0.006 0.000 0.000 0.000

0.000 0.006 0.006 0.012 0.006 0.019 0.000 0.006 0.000 0.000

0.062 0.063 0.054 0.080 0.006 0.000 0.000 0.000 0.000 0.006

0.006 0.012 0.000 0.006 0.000 0.000 0.000 0.006 0.006 0.024

0.000 0.000 0.006 0.000 0.062 0.061 0.053 0.080 0.017 0.009

0.000 0.000 0.009 0.017 0.000 0.009 0.000 0.000 0.000 0.000

0.009 0.009 0.000 0.017 0.000 0.009 0.000 0.000 0.049 0.049

0.060 0.071

>Non-Methylation code ID 1447

0.005 0.009 0.000 0.005 0.009 0.000 0.000 0.000 0.000 0.014

0.000 0.005 0.005 0.000 0.005 0.000 0.000 0.000 0.000 0.005

0.071 0.063 0.062 0.076 0.009 0.004 0.000 0.000 0.009 0.004

0.000 0.000 0.000 0.000 0.000 0.000 0.004 0.000 0.009 0.004

0.009 0.000 0.000 0.000 0.069 0.073 0.071 0.069 0.007 0.000

0.000 0.007 0.000 0.000 0.000 0.000 0.000 0.021 0.000 0.000

0.000 0.007 0.014 0.014 0.000 0.007 0.000 0.000 0.059 0.055

0.065 0.078

>Non-Methylation code ID 1447

0.014 0.000 0.009 0.000 0.000 0.009 0.005 0.000 0.005 0.005

0.000 0.000 0.005 0.000 0.009 0.000 0.000 0.000 0.000 0.000

0.071 0.074 0.063 0.065 0.000 0.000 0.000 0.011 0.006 0.006

0.000 0.000 0.006 0.006 0.006 0.000 0.011 0.000 0.006 0.000

0.011 0.000 0.000 0.000 0.076 0.066 0.034 0.089 0.004 0.004

0.000 0.004 0.000 0.000 0.000 0.000 0.000 0.000 0.000 0.000

0.004 0.004 0.012 0.008 0.000 0.000 0.008 0.000 0.088 0.066

0.049 0.084

>Non-Methylation code ID 1447

0.009 0.005 0.000 0.005 0.000 0.000 0.005 0.000 0.000 0.000

0.000 0.000 0.009 0.005 0.014 0.009 0.000 0.000 0.000 0.000

0.072 0.057 0.069 0.074 0.021 0.000 0.005 0.000 0.000 0.000

0.005 0.005 0.005 0.000 0.000 0.000 0.016 0.000 0.005 0.000

0.000 0.000 0.000 0.000 0.047 0.067 0.079 0.077 0.000 0.006

0.000 0.000 0.000 0.006 0.000 0.000 0.006 0.006 0.000 0.000

0.017 0.006 0.006 0.006 0.012 0.000 0.000 0.000 0.075 0.086

0.029 0.074

>Non-Methylation code ID 1447

0.005 0.000 0.000 0.005 0.005 0.010 0.005 0.000 0.000 0.010

0.000 0.000 0.005 0.000 0.010 0.005 0.000 0.000 0.000 0.000

0.066 0.077 0.056 0.074 0.000 0.000 0.000 0.015 0.015 0.015

0.000 0.000 0.000 0.030 0.000 0.008 0.000 0.000 0.000 0.000

0.008 0.008 0.000 0.000 0.059 0.063 0.064 0.049 0.000 0.000

0.000 0.006 0.006 0.006 0.000 0.000 0.006 0.006 0.000 0.000

0.000 0.000 0.006 0.018 0.006 0.000 0.006 0.000 0.061 0.059

0.076 0.073

>Non-Methylation code ID 1447

0.005 0.000 0.000 0.015 0.000 0.010 0.000 0.005 0.000 0.000

0.000 0.000 0.000 0.000 0.010 0.000 0.000 0.005 0.000 0.005

0.069 0.054 0.068 0.087 0.000 0.000 0.006 0.017 0.000 0.006

0.000 0.000 0.011 0.006 0.000 0.000 0.000 0.006 0.011 0.006

0.006 0.000 0.000 0.000 0.069 0.057 0.061 0.072 0.000 0.000

0.000 0.000 0.000 0.011 0.000 0.005 0.005 0.000 0.000 0.005

0.000 0.005 0.016 0.005 0.000 0.000 0.005 0.000 0.082 0.070

0.062 0.060

>Non-Methylation code ID 1447

0.040 0.000 0.000 0.000 0.000 0.007 0.000 0.007 0.007 0.000

0.000 0.000 0.013 0.000 0.007 0.000 0.000 0.000 0.000 0.000

0.065 0.062 0.074 0.051 0.000 0.005 0.000 0.005 0.000 0.005

0.000 0.000 0.005 0.010 0.000 0.000 0.015 0.010 0.005 0.005

0.000 0.000 0.000 0.000 0.065 0.083 0.056 0.063 0.006 0.006

0.000 0.000 0.000 0.006 0.006 0.000 0.000 0.000 0.000 0.000

0.006 0.006 0.006 0.023 0.000 0.000 0.006 0.000 0.061 0.072

0.050 0.082

>Non-Methylation code ID 1447

0.000 0.009 0.000 0.009 0.004 0.000 0.000 0.004 0.004 0.000

0.000 0.009 0.004 0.000 0.004 0.004 0.000 0.000 0.000 0.000

0.085 0.070 0.057 0.069 0.006 0.000 0.000 0.006 0.000 0.000

0.000 0.006 0.006 0.012 0.000 0.018 0.006 0.000 0.000 0.006

0.006 0.006 0.000 0.000 0.066 0.055 0.063 0.072 0.000 0.000

0.000 0.000 0.005 0.000 0.000 0.005 0.005 0.010 0.005 0.000

0.010 0.000 0.010 0.000 0.000 0.000 0.000 0.005 0.070 0.083

0.077 0.048

>Non-Methylation code ID 1447

0.000 0.000 0.000 0.007 0.000 0.015 0.000 0.007 0.007 0.015

0.000 0.000 0.000 0.007 0.000 0.000 0.015 0.000 0.007 0.000

0.061 0.071 0.078 0.041 0.000 0.000 0.000 0.000 0.000 0.010

0.000 0.000 0.000 0.005 0.000 0.000 0.005 0.000 0.015 0.020

0.000 0.005 0.005 0.000 0.083 0.046 0.087 0.052 0.008 0.000

0.023 0.000 0.000 0.015 0.000 0.000 0.000 0.015 0.000 0.000

0.008 0.008 0.000 0.008 0.000 0.000 0.000 0.000 0.064 0.054

0.075 0.056

>Non-Methylation code ID 1447

0.013 0.000 0.013 0.000 0.000 0.000 0.000 0.007 0.000 0.000

0.000 0.013 0.007 0.000 0.000 0.013 0.000 0.007 0.000 0.007

0.070 0.063 0.047 0.073 0.000 0.000 0.000 0.008 0.000 0.000

0.000 0.008 0.000 0.008 0.024 0.000 0.008 0.008 0.008 0.008

0.008 0.008 0.000 0.000 0.067 0.060 0.046 0.062 0.000 0.011

0.000 0.000 0.006 0.000 0.006 0.000 0.011 0.006 0.000 0.000

0.006 0.006 0.000 0.006 0.000 0.000 0.000 0.000 0.067 0.069

0.075 0.066

>Non-Methylation code ID 1447

0.029 0.000 0.000 0.010 0.000 0.000 0.000 0.000 0.000 0.029

0.000 0.000 0.010 0.010 0.000 0.029 0.010 0.000 0.000 0.000

0.057 0.052 0.051 0.046 0.005 0.005 0.000 0.000 0.000 0.000

0.005 0.000 0.005 0.009 0.000 0.000 0.009 0.005 0.009 0.005

0.000 0.000 0.000 0.000 0.062 0.081 0.074 0.063 0.012 0.000

0.000 0.006 0.012 0.006 0.006 0.000 0.000 0.006 0.000 0.000

0.000 0.000 0.006 0.012 0.006 0.000 0.000 0.000 0.062 0.054

0.063 0.079

>Non-Methylation code ID 1447

0.010 0.005 0.000 0.000 0.000 0.000 0.000 0.000 0.005 0.000

0.000 0.005 0.005 0.005 0.010 0.010 0.010 0.000 0.000 0.000

0.084 0.046 0.075 0.060 0.010 0.000 0.000 0.005 0.000 0.000

0.000 0.000 0.000 0.005 0.000 0.000 0.020 0.005 0.010 0.005

0.005 0.000 0.000 0.000 0.069 0.088 0.045 0.066 0.006 0.006

0.006 0.000 0.000 0.000 0.000 0.000 0.006 0.013 0.000 0.006

0.013 0.013 0.000 0.000 0.000 0.006 0.000 0.000 0.066 0.062

0.060 0.068

>Non-Methylation code ID 1447

0.000 0.000 0.000 0.000 0.000 0.005 0.000 0.000 0.000 0.005

0.000 0.000 0.000 0.000 0.016 0.031 0.000 0.005 0.005 0.000

0.084 0.050 0.086 0.046 0.026 0.000 0.017 0.000 0.000 0.026

0.000 0.000 0.000 0.017 0.000 0.000 0.009 0.000 0.000 0.009

0.000 0.000 0.000 0.000 0.066 0.047 0.063 0.055 0.000 0.000

0.000 0.019 0.000 0.010 0.000 0.010 0.000 0.019 0.000 0.000

0.000 0.019 0.000 0.000 0.010 0.000 0.010 0.000 0.061 0.066

0.060 0.050

>Non-Methylation code ID 1447

0.000 0.000 0.007 0.007 0.013 0.013 0.000 0.000 0.000 0.020

0.000 0.007 0.000 0.000 0.000 0.007 0.007 0.007 0.000 0.000

0.067 0.063 0.057 0.060 0.000 0.000 0.000 0.006 0.006 0.006

0.000 0.006 0.006 0.000 0.000 0.000 0.000 0.006 0.006 0.017

0.006 0.000 0.006 0.000 0.066 0.059 0.066 0.072 0.005 0.000

0.000 0.005 0.005 0.009 0.005 0.000 0.000 0.009 0.000 0.000

0.005 0.000 0.014 0.000 0.000 0.000 0.000 0.000 0.072 0.074

0.051 0.082

>Non-Methylation code ID 1447

0.000 0.000 0.000 0.000 0.000 0.023 0.000 0.000 0.023 0.000

0.008 0.000 0.000 0.000 0.000 0.023 0.008 0.000 0.008 0.000

0.075 0.067 0.075 0.026 0.008 0.000 0.000 0.000 0.000 0.016

0.000 0.000 0.000 0.008 0.000 0.000 0.023 0.000 0.000 0.023

0.000 0.008 0.008 0.000 0.071 0.064 0.056 0.049 0.000 0.000

0.007 0.007 0.000 0.015 0.007 0.000 0.000 0.007 0.000 0.000

0.000 0.015 0.000 0.000 0.000 0.015 0.007 0.000 0.065 0.060

0.061 0.067

>Non-Methylation code ID 1447

0.006 0.000 0.000 0.006 0.022 0.000 0.000 0.000 0.017 0.011

0.000 0.000 0.000 0.000 0.000 0.006 0.000 0.000 0.000 0.000

0.055 0.063 0.081 0.067 0.000 0.005 0.000 0.000 0.014 0.000

0.000 0.000 0.010 0.005 0.000 0.005 0.005 0.005 0.005 0.005

0.000 0.000 0.000 0.000 0.063 0.080 0.075 0.059 0.000 0.000

0.000 0.000 0.012 0.006 0.000 0.006 0.012 0.006 0.000 0.000

0.006 0.000 0.006 0.012 0.000 0.000 0.000 0.000 0.069 0.074

0.061 0.064

>Non-Methylation code ID 1447

0.000 0.000 0.005 0.000 0.000 0.014 0.000 0.005 0.000 0.000

0.000 0.005 0.000 0.000 0.009 0.005 0.000 0.005 0.009 0.000

0.076 0.075 0.070 0.056 0.024 0.000 0.000 0.016 0.000 0.024

0.000 0.000 0.000 0.000 0.000 0.000 0.000 0.000 0.000 0.016

0.016 0.000 0.008 0.000 0.052 0.069 0.056 0.053 0.000 0.000

0.000 0.005 0.000 0.010 0.005 0.000 0.005 0.005 0.000 0.000

0.005 0.000 0.019 0.000 0.000 0.000 0.000 0.000 0.067 0.075

0.055 0.083

>Non-Methylation code ID 1447

0.006 0.000 0.000 0.012 0.006 0.000 0.000 0.006 0.000 0.012

0.000 0.000 0.006 0.006 0.006 0.000 0.012 0.006 0.000 0.000

0.070 0.058 0.067 0.062 0.000 0.000 0.000 0.005 0.010 0.000

0.005 0.000 0.010 0.005 0.000 0.005 0.005 0.000 0.005 0.010

0.000 0.000 0.005 0.000 0.070 0.061 0.066 0.068 0.000 0.006

0.000 0.000 0.000 0.013 0.000 0.006 0.000 0.000 0.000 0.013

0.013 0.006 0.000 0.013 0.006 0.000 0.000 0.000 0.045 0.064

0.076 0.071

>Non-Methylation code ID 1447

0.013 0.006 0.006 0.006 0.000 0.019 0.000 0.006 0.000 0.000

0.000 0.000 0.006 0.000 0.000 0.006 0.000 0.006 0.006 0.000

0.070 0.049 0.066 0.063 0.000 0.000 0.000 0.006 0.000 0.012

0.000 0.006 0.000 0.006 0.000 0.000 0.006 0.006 0.012 0.012

0.000 0.000 0.000 0.000 0.066 0.076 0.069 0.055 0.007 0.000

0.000 0.000 0.000 0.015 0.007 0.000 0.000 0.015 0.000 0.000

0.000 0.000 0.007 0.015 0.000 0.000 0.007 0.000 0.059 0.058

0.068 0.074

>Non-Methylation code ID 1447

0.000 0.000 0.005 0.005 0.000 0.011 0.000 0.000 0.000 0.011

0.000 0.000 0.000 0.005 0.011 0.005 0.005 0.000 0.011 0.000

0.088 0.048 0.068 0.059 0.011 0.000 0.000 0.005 0.000 0.016

0.000 0.000 0.005 0.000 0.000 0.000 0.000 0.000 0.005 0.005

0.005 0.005 0.000 0.011 0.047 0.082 0.077 0.057 0.016 0.000

0.000 0.000 0.000 0.023 0.008 0.000 0.008 0.008 0.008 0.000

0.000 0.000 0.008 0.000 0.008 0.000 0.000 0.000 0.084 0.048

0.080 0.036

>Non-Methylation code ID 1447

0.019 0.000 0.000 0.005 0.010 0.005 0.005 0.000 0.000 0.005

0.000 0.000 0.000 0.000 0.005 0.005 0.000 0.000 0.005 0.000

0.060 0.063 0.063 0.085 0.016 0.000 0.000 0.000 0.000 0.016

0.000 0.000 0.000 0.025 0.000 0.000 0.008 0.000 0.008 0.025

0.008 0.000 0.000 0.000 0.073 0.062 0.048 0.044 0.007 0.000

0.000 0.000 0.000 0.013 0.000 0.000 0.000 0.013 0.000 0.000

0.007 0.007 0.013 0.007 0.000 0.000 0.000 0.000 0.069 0.078

0.057 0.062

>Non-Methylation code ID 1447

0.019 0.000 0.000 0.006 0.012 0.000 0.000 0.000 0.000 0.012

0.000 0.000 0.006 0.000 0.006 0.000 0.000 0.012 0.000 0.000

0.070 0.059 0.063 0.067 0.000 0.000 0.000 0.000 0.015 0.000

0.005 0.000 0.000 0.005 0.000 0.000 0.005 0.005 0.010 0.010

0.000 0.005 0.000 0.000 0.071 0.074 0.053 0.075 0.000 0.005

0.000 0.000 0.005 0.011 0.000 0.000 0.000 0.011 0.000 0.000

0.005 0.000 0.011 0.011 0.005 0.000 0.000 0.000 0.084 0.051

0.068 0.065

>Non-Methylation code ID 1447

0.009 0.013 0.000 0.004 0.000 0.004 0.000 0.000 0.000 0.000

0.000 0.000 0.013 0.004 0.009 0.000 0.000 0.000 0.000 0.000

0.066 0.063 0.060 0.089 0.014 0.000 0.005 0.000 0.000 0.005

0.010 0.000 0.000 0.000 0.000 0.000 0.005 0.000 0.010 0.010

0.000 0.005 0.000 0.000 0.073 0.084 0.040 0.074 0.018 0.000

0.000 0.000 0.000 0.006 0.000 0.000 0.000 0.006 0.006 0.000

0.006 0.000 0.012 0.000 0.012 0.000 0.000 0.000 0.075 0.067

0.039 0.086

>Non-Methylation code ID 1447

0.006 0.000 0.000 0.000 0.000 0.013 0.006 0.019 0.000 0.000

0.000 0.000 0.000 0.006 0.006 0.026 0.000 0.000 0.000 0.000

0.062 0.054 0.052 0.082 0.017 0.000 0.000 0.009 0.009 0.017

0.000 0.009 0.000 0.000 0.000 0.000 0.009 0.009 0.000 0.026

0.000 0.009 0.000 0.000 0.045 0.056 0.062 0.060 0.000 0.000

0.000 0.006 0.000 0.006 0.006 0.000 0.000 0.000 0.000 0.012

0.006 0.012 0.006 0.012 0.000 0.006 0.000 0.000 0.065 0.066

0.055 0.074

>Non-Methylation code ID 1447

0.009 0.005 0.009 0.000 0.009 0.000 0.000 0.000 0.000 0.000

0.000 0.000 0.005 0.000 0.005 0.009 0.005 0.000 0.000 0.000

0.066 0.066 0.069 0.076 0.006 0.000 0.000 0.000 0.000 0.000

0.000 0.006 0.000 0.024 0.006 0.000 0.000 0.006 0.012 0.012

0.000 0.006 0.000 0.000 0.059 0.056 0.056 0.083 0.007 0.007

0.000 0.000 0.022 0.000 0.000 0.000 0.000 0.022 0.000 0.000

0.007 0.000 0.000 0.007 0.000 0.000 0.000 0.007 0.066 0.074

0.056 0.055

>Non-Methylation code ID 1447

0.000 0.006 0.006 0.012 0.000 0.006 0.000 0.000 0.006 0.018

0.000 0.000 0.000 0.000 0.000 0.000 0.000 0.000 0.000 0.012

0.073 0.062 0.062 0.071 0.000 0.000 0.006 0.006 0.006 0.000

0.000 0.000 0.006 0.000 0.012 0.006 0.000 0.000 0.006 0.006

0.006 0.000 0.000 0.012 0.073 0.069 0.044 0.075 0.000 0.000

0.000 0.000 0.000 0.000 0.000 0.014 0.007 0.014 0.007 0.000

0.000 0.000 0.007 0.000 0.007 0.007 0.007 0.000 0.064 0.056

0.060 0.083

>Non-Methylation code ID 1447

0.022 0.007 0.000 0.000 0.000 0.007 0.000 0.000 0.000 0.007

0.000 0.000 0.007 0.000 0.000 0.022 0.015 0.000 0.000 0.007

0.053 0.064 0.042 0.078 0.010 0.000 0.000 0.000 0.000 0.000

0.000 0.000 0.000 0.019 0.000 0.000 0.005 0.000 0.019 0.000

0.005 0.000 0.005 0.000 0.044 0.073 0.074 0.081 0.000 0.000

0.007 0.000 0.007 0.021 0.000 0.000 0.000 0.028 0.000 0.000

0.000 0.000 0.007 0.014 0.000 0.000 0.000 0.000 0.065 0.071

0.048 0.064

>Non-Methylation code ID 1447

0.010 0.000 0.000 0.000 0.000 0.005 0.005 0.000 0.000 0.015

0.000 0.000 0.005 0.005 0.010 0.000 0.005 0.000 0.005 0.000

0.047 0.066 0.073 0.081 0.000 0.000 0.000 0.000 0.000 0.027

0.000 0.007 0.000 0.027 0.000 0.007 0.000 0.000 0.007 0.014

0.000 0.000 0.000 0.000 0.056 0.069 0.046 0.074 0.026 0.009

0.000 0.000 0.000 0.009 0.000 0.000 0.000 0.009 0.000 0.000

0.009 0.000 0.000 0.026 0.009 0.000 0.000 0.009 0.060 0.070

0.035 0.065

>Non-Methylation code ID 1447

0.005 0.000 0.000 0.005 0.000 0.015 0.000 0.000 0.000 0.005

0.000 0.000 0.000 0.010 0.010 0.005 0.005 0.000 0.005 0.000

0.084 0.046 0.062 0.074 0.009 0.000 0.000 0.009 0.000 0.009

0.000 0.000 0.009 0.000 0.000 0.000 0.000 0.000 0.009 0.004

0.000 0.004 0.000 0.004 0.053 0.070 0.080 0.073 0.018 0.000

0.000 0.000 0.000 0.012 0.006 0.000 0.006 0.006 0.000 0.000

0.000 0.000 0.012 0.006 0.000 0.000 0.000 0.000 0.088 0.060

0.069 0.049

>Non-Methylation code ID 1447

0.000 0.005 0.005 0.000 0.005 0.016 0.000 0.000 0.000 0.005

0.005 0.000 0.000 0.000 0.005 0.016 0.000 0.000 0.005 0.000

0.067 0.073 0.063 0.061 0.024 0.000 0.012 0.000 0.000 0.024

0.000 0.000 0.000 0.012 0.012 0.000 0.000 0.012 0.000 0.012

0.000 0.024 0.000 0.000 0.054 0.043 0.062 0.040 0.005 0.000

0.000 0.005 0.010 0.005 0.000 0.000 0.000 0.010 0.000 0.005

0.000 0.000 0.010 0.005 0.000 0.000 0.005 0.000 0.079 0.076

0.074 0.048

>Non-Methylation code ID 1447

0.027 0.000 0.000 0.018 0.000 0.027 0.000 0.000 0.000 0.000

0.000 0.000 0.000 0.000 0.000 0.018 0.018 0.000 0.009 0.000

0.061 0.058 0.054 0.046 0.000 0.000 0.000 0.004 0.000 0.012

0.004 0.000 0.004 0.004 0.000 0.000 0.008 0.000 0.016 0.000

0.000 0.000 0.000 0.000 0.063 0.069 0.057 0.091 0.000 0.000

0.005 0.000 0.000 0.016 0.000 0.005 0.000 0.000 0.000 0.005

0.000 0.000 0.011 0.005 0.000 0.005 0.005 0.000 0.065 0.075

0.068 0.068

>Non-Methylation code ID 1447

0.012 0.000 0.000 0.000 0.000 0.000 0.000 0.000 0.012 0.006

0.000 0.000 0.012 0.006 0.012 0.012 0.000 0.006 0.000 0.000

0.056 0.053 0.063 0.086 0.005 0.000 0.000 0.005 0.005 0.005

0.000 0.000 0.000 0.005 0.000 0.000 0.000 0.010 0.020 0.005

0.000 0.000 0.000 0.000 0.086 0.048 0.067 0.071 0.015 0.000

0.000 0.015 0.000 0.008 0.000 0.000 0.015 0.008 0.000 0.000

0.000 0.000 0.000 0.030 0.000 0.000 0.000 0.000 0.069 0.038

0.060 0.075

>Non-Methylation code ID 1447

0.000 0.000 0.007 0.007 0.014 0.014 0.000 0.000 0.000 0.027

0.000 0.007 0.000 0.000 0.000 0.000 0.007 0.007 0.000 0.000

0.063 0.060 0.064 0.058 0.000 0.000 0.000 0.006 0.006 0.006

0.000 0.006 0.006 0.006 0.000 0.000 0.000 0.000 0.006 0.017

0.006 0.000 0.006 0.000 0.061 0.059 0.075 0.071 0.005 0.000

0.000 0.005 0.005 0.010 0.005 0.000 0.000 0.010 0.000 0.000

0.005 0.000 0.010 0.005 0.000 0.000 0.000 0.000 0.066 0.077

0.056 0.074

>Non-Methylation code ID 1447

0.011 0.000 0.000 0.011 0.005 0.005 0.000 0.000 0.000 0.011

0.000 0.000 0.000 0.000 0.011 0.000 0.011 0.000 0.000 0.000

0.061 0.067 0.070 0.071 0.000 0.000 0.000 0.011 0.000 0.000

0.006 0.000 0.011 0.000 0.000 0.000 0.006 0.006 0.011 0.011

0.000 0.000 0.006 0.000 0.070 0.076 0.060 0.058 0.000 0.006

0.000 0.006 0.000 0.011 0.000 0.006 0.000 0.000 0.000 0.011

0.011 0.006 0.006 0.006 0.000 0.000 0.000 0.000 0.071 0.059

0.075 0.061

>Non-Methylation code ID 1447

0.007 0.000 0.000 0.007 0.000 0.000 0.000 0.007 0.007 0.013

0.000 0.013 0.007 0.000 0.000 0.007 0.000 0.007 0.000 0.007

0.070 0.065 0.061 0.057 0.000 0.005 0.000 0.000 0.005 0.000

0.000 0.005 0.005 0.005 0.000 0.000 0.010 0.000 0.010 0.010

0.010 0.000 0.000 0.000 0.072 0.080 0.068 0.048 0.000 0.000

0.000 0.010 0.005 0.000 0.000 0.000 0.000 0.005 0.000 0.005

0.010 0.000 0.010 0.005 0.000 0.005 0.000 0.000 0.081 0.072

0.055 0.069

>Non-Methylation code ID 1447

0.009 0.000 0.000 0.009 0.000 0.009 0.009 0.000 0.018 0.009

0.000 0.000 0.000 0.000 0.000 0.045 0.000 0.000 0.000 0.000

0.073 0.039 0.058 0.057 0.012 0.000 0.000 0.006 0.000 0.000

0.000 0.006 0.012 0.012 0.000 0.000 0.006 0.000 0.006 0.012

0.000 0.006 0.000 0.000 0.056 0.067 0.063 0.071 0.006 0.000

0.000 0.006 0.011 0.000 0.000 0.000 0.000 0.006 0.000 0.000

0.000 0.011 0.011 0.011 0.000 0.000 0.000 0.000 0.063 0.081

0.055 0.072

>Non-Methylation code ID 1447

0.020 0.000 0.000 0.000 0.000 0.013 0.007 0.000 0.007 0.000

0.000 0.000 0.020 0.000 0.000 0.000 0.000 0.000 0.013 0.000

0.070 0.052 0.066 0.066 0.000 0.000 0.000 0.010 0.000 0.010

0.000 0.000 0.000 0.010 0.000 0.005 0.005 0.005 0.010 0.005

0.005 0.000 0.000 0.000 0.058 0.069 0.054 0.085 0.023 0.000

0.000 0.000 0.000 0.000 0.000 0.000 0.000 0.012 0.000 0.000

0.000 0.012 0.000 0.047 0.012 0.012 0.012 0.000 0.057 0.064

0.045 0.039

>Non-Methylation code ID 1447

0.012 0.000 0.000 0.000 0.000 0.006 0.000 0.017 0.000 0.000

0.000 0.000 0.000 0.006 0.012 0.017 0.000 0.006 0.000 0.000

0.077 0.062 0.054 0.065 0.007 0.000 0.000 0.014 0.014 0.007

0.000 0.000 0.000 0.007 0.000 0.000 0.007 0.014 0.000 0.021

0.000 0.000 0.000 0.000 0.046 0.068 0.069 0.060 0.000 0.000

0.000 0.000 0.006 0.006 0.006 0.000 0.000 0.000 0.000 0.013

0.006 0.006 0.006 0.019 0.000 0.006 0.000 0.000 0.064 0.065

0.062 0.065

>Non-Methylation code ID 1447

0.006 0.000 0.000 0.000 0.000 0.023 0.006 0.006 0.000 0.000

0.000 0.006 0.000 0.006 0.006 0.012 0.000 0.000 0.006 0.000

0.073 0.068 0.045 0.071 0.044 0.000 0.000 0.000 0.000 0.015

0.000 0.007 0.000 0.000 0.000 0.000 0.007 0.000 0.007 0.007

0.007 0.000 0.000 0.000 0.053 0.061 0.061 0.062 0.000 0.000

0.000 0.005 0.000 0.005 0.005 0.000 0.000 0.005 0.000 0.000

0.011 0.011 0.016 0.005 0.000 0.000 0.000 0.000 0.057 0.074

0.057 0.082

>Non-Methylation code ID 1447

0.012 0.000 0.000 0.000 0.000 0.018 0.000 0.000 0.006 0.000

0.000 0.000 0.006 0.000 0.006 0.006 0.006 0.006 0.000 0.012

0.049 0.089 0.068 0.051 0.014 0.000 0.000 0.000 0.007 0.021

0.007 0.000 0.007 0.007 0.007 0.000 0.007 0.007 0.000 0.000

0.007 0.000 0.000 0.000 0.082 0.051 0.056 0.053 0.000 0.000

0.007 0.007 0.000 0.007 0.000 0.000 0.000 0.014 0.000 0.000

0.000 0.007 0.000 0.021 0.007 0.000 0.014 0.000 0.064 0.067

0.071 0.046

>Non-Methylation code ID 1470

0.000 0.000 0.000 0.007 0.007 0.014 0.000 0.007 0.000 0.007

0.000 0.000 0.000 0.000 0.007 0.000 0.007 0.022 0.000 0.007

0.068 0.059 0.052 0.069 0.000 0.000 0.006 0.018 0.006 0.000

0.000 0.000 0.000 0.018 0.000 0.000 0.000 0.000 0.006 0.006

0.006 0.000 0.000 0.006 0.063 0.053 0.067 0.080 0.000 0.004

0.000 0.000 0.004 0.004 0.000 0.008 0.000 0.000 0.000 0.008

0.000 0.004 0.008 0.004 0.000 0.000 0.004 0.000 0.079 0.055

0.072 0.078

>Non-Methylation code ID 1470

0.005 0.000 0.000 0.005 0.011 0.000 0.000 0.000 0.000 0.005

0.000 0.000 0.000 0.011 0.011 0.016 0.000 0.000 0.000 0.000

0.069 0.078 0.054 0.067 0.015 0.000 0.000 0.008 0.000 0.008

0.008 0.000 0.015 0.008 0.000 0.000 0.000 0.000 0.000 0.039

0.000 0.000 0.000 0.000 0.070 0.047 0.051 0.064 0.013 0.000

0.000 0.000 0.000 0.000 0.000 0.007 0.013 0.013 0.000 0.000

0.007 0.000 0.007 0.013 0.000 0.007 0.000 0.000 0.056 0.067

0.057 0.072

>Non-Methylation code ID 1470

0.005 0.000 0.000 0.005 0.014 0.009 0.000 0.000 0.024 0.000

0.000 0.005 0.000 0.000 0.000 0.000 0.000 0.000 0.000 0.000

0.047 0.064 0.086 0.075 0.000 0.000 0.000 0.009 0.009 0.000

0.000 0.000 0.009 0.005 0.000 0.005 0.000 0.005 0.014 0.000

0.005 0.000 0.000 0.000 0.035 0.070 0.097 0.072 0.000 0.000

0.000 0.017 0.006 0.000 0.000 0.006 0.011 0.006 0.000 0.000

0.000 0.000 0.011 0.006 0.000 0.000 0.000 0.000 0.056 0.076

0.071 0.070

>Non-Methylation code ID 1470

0.000 0.000 0.000 0.000 0.008 0.008 0.000 0.008 0.000 0.000

0.000 0.000 0.000 0.008 0.008 0.032 0.008 0.000 0.008 0.000

0.065 0.068 0.054 0.058 0.004 0.004 0.000 0.004 0.009 0.009

0.004 0.000 0.000 0.009 0.000 0.000 0.004 0.004 0.004 0.000

0.000 0.000 0.000 0.000 0.074 0.062 0.063 0.077 0.000 0.000

0.006 0.000 0.012 0.000 0.000 0.000 0.000 0.018 0.000 0.006

0.000 0.000 0.006 0.006 0.006 0.012 0.000 0.000 0.078 0.061

0.053 0.068

>Non-Methylation code ID 1470

0.000 0.000 0.000 0.000 0.000 0.018 0.000 0.006 0.000 0.012

0.000 0.006 0.000 0.006 0.006 0.018 0.000 0.000 0.006 0.000

0.065 0.071 0.055 0.065 0.040 0.000 0.000 0.000 0.000 0.016

0.000 0.000 0.000 0.000 0.000 0.000 0.008 0.000 0.008 0.016

0.008 0.000 0.000 0.008 0.047 0.058 0.057 0.068 0.005 0.000

0.000 0.005 0.000 0.005 0.005 0.000 0.000 0.015 0.000 0.000

0.005 0.010 0.010 0.000 0.000 0.000 0.000 0.000 0.045 0.065

0.073 0.092

>Non-Methylation code ID 1470

0.000 0.000 0.000 0.005 0.005 0.000 0.000 0.005 0.000 0.000

0.000 0.010 0.005 0.000 0.010 0.005 0.000 0.010 0.000 0.005

0.058 0.081 0.072 0.063 0.000 0.000 0.009 0.009 0.000 0.000

0.000 0.000 0.009 0.009 0.009 0.000 0.009 0.000 0.000 0.017

0.017 0.009 0.000 0.000 0.067 0.068 0.062 0.041 0.000 0.010

0.000 0.000 0.000 0.000 0.005 0.000 0.016 0.016 0.000 0.000

0.005 0.000 0.000 0.000 0.005 0.000 0.000 0.000 0.074 0.066

0.074 0.062

>Non-Methylation code ID 1470

0.007 0.000 0.000 0.015 0.007 0.000 0.000 0.000 0.000 0.007

0.007 0.007 0.007 0.007 0.000 0.007 0.015 0.000 0.000 0.000

0.065 0.068 0.039 0.072 0.000 0.004 0.000 0.000 0.000 0.000

0.000 0.000 0.000 0.004 0.000 0.000 0.016 0.000 0.012 0.004

0.004 0.000 0.008 0.000 0.084 0.086 0.036 0.075 0.005 0.005

0.015 0.000 0.000 0.010 0.000 0.000 0.000 0.010 0.000 0.000

0.005 0.005 0.005 0.000 0.000 0.000 0.000 0.000 0.076 0.067

0.067 0.063

>Non-Methylation code ID 1470

0.006 0.000 0.000 0.000 0.000 0.000 0.000 0.000 0.000 0.006

0.000 0.000 0.000 0.006 0.018 0.031 0.006 0.000 0.006 0.000

0.088 0.042 0.080 0.044 0.023 0.000 0.015 0.000 0.000 0.015

0.000 0.000 0.008 0.008 0.000 0.000 0.023 0.000 0.000 0.008

0.000 0.000 0.000 0.000 0.075 0.047 0.063 0.050 0.000 0.000

0.000 0.014 0.000 0.000 0.000 0.007 0.000 0.014 0.000 0.007

0.007 0.014 0.007 0.000 0.007 0.000 0.000 0.000 0.063 0.066

0.062 0.064

>Non-Methylation code ID 1470

0.006 0.000 0.000 0.000 0.006 0.000 0.006 0.000 0.006 0.019

0.000 0.000 0.013 0.000 0.000 0.000 0.000 0.013 0.000 0.006

0.059 0.057 0.071 0.069 0.000 0.000 0.000 0.000 0.025 0.000

0.000 0.006 0.000 0.000 0.000 0.006 0.006 0.013 0.000 0.006

0.006 0.000 0.013 0.000 0.066 0.076 0.050 0.059 0.000 0.000

0.000 0.000 0.000 0.013 0.000 0.006 0.006 0.006 0.000 0.000

0.000 0.000 0.013 0.031 0.000 0.000 0.000 0.000 0.078 0.079

0.056 0.044

>Non-Methylation code ID 1470

0.000 0.000 0.005 0.000 0.000 0.019 0.000 0.005 0.000 0.000

0.000 0.000 0.000 0.000 0.010 0.005 0.000 0.005 0.010 0.000

0.082 0.076 0.065 0.053 0.023 0.000 0.000 0.015 0.000 0.030

0.000 0.000 0.000 0.000 0.000 0.000 0.000 0.000 0.000 0.015

0.008 0.000 0.008 0.000 0.047 0.065 0.066 0.057 0.005 0.000

0.000 0.000 0.000 0.010 0.005 0.000 0.005 0.005 0.000 0.000

0.005 0.000 0.020 0.000 0.000 0.000 0.000 0.000 0.063 0.067

0.061 0.088

>Non-Methylation code ID 1470

0.000 0.000 0.000 0.000 0.007 0.007 0.000 0.007 0.013 0.007

0.000 0.007 0.000 0.007 0.007 0.013 0.000 0.000 0.000 0.000

0.063 0.094 0.062 0.042 0.004 0.004 0.000 0.008 0.004 0.000

0.000 0.004 0.000 0.000 0.000 0.000 0.000 0.004 0.013 0.000

0.000 0.000 0.000 0.004 0.075 0.056 0.079 0.077 0.000 0.000

0.006 0.006 0.006 0.011 0.000 0.000 0.017 0.000 0.000 0.000

0.000 0.006 0.006 0.000 0.006 0.006 0.000 0.000 0.068 0.068

0.050 0.082

>Non-Methylation code ID 1470

0.000 0.000 0.000 0.000 0.019 0.000 0.000 0.006 0.000 0.000

0.000 0.006 0.000 0.006 0.006 0.019 0.006 0.000 0.006 0.000

0.060 0.071 0.059 0.069 0.000 0.000 0.000 0.000 0.000 0.013

0.000 0.007 0.013 0.007 0.000 0.000 0.000 0.000 0.007 0.027

0.000 0.013 0.000 0.000 0.070 0.074 0.036 0.067 0.000 0.000

0.000 0.006 0.006 0.000 0.006 0.000 0.012 0.017 0.000 0.000

0.006 0.000 0.000 0.006 0.000 0.006 0.000 0.006 0.063 0.067

0.068 0.066

>Non-Methylation code ID 1470

0.019 0.000 0.000 0.000 0.000 0.009 0.009 0.000 0.019 0.009

0.000 0.000 0.000 0.000 0.000 0.047 0.000 0.000 0.000 0.000

0.066 0.046 0.052 0.056 0.011 0.000 0.000 0.006 0.000 0.000

0.000 0.006 0.006 0.011 0.000 0.000 0.011 0.000 0.006 0.011

0.000 0.006 0.000 0.000 0.061 0.065 0.060 0.073 0.006 0.000

0.000 0.006 0.013 0.000 0.000 0.000 0.000 0.013 0.000 0.000

0.000 0.013 0.006 0.013 0.000 0.000 0.000 0.000 0.067 0.075

0.056 0.065

>Non-Methylation code ID 1470

0.019 0.000 0.000 0.019 0.000 0.009 0.000 0.000 0.000 0.019

0.000 0.000 0.009 0.000 0.000 0.028 0.009 0.009 0.000 0.000

0.064 0.053 0.051 0.042 0.005 0.000 0.005 0.000 0.000 0.000

0.000 0.000 0.000 0.015 0.000 0.000 0.010 0.000 0.015 0.005

0.000 0.005 0.000 0.000 0.082 0.051 0.080 0.064 0.007 0.000

0.000 0.000 0.000 0.013 0.007 0.000 0.000 0.007 0.000 0.000

0.000 0.007 0.013 0.007 0.007 0.000 0.000 0.000 0.074 0.080

0.037 0.076

>Non-Methylation code ID 1470

0.013 0.000 0.009 0.000 0.000 0.009 0.004 0.000 0.004 0.000

0.000 0.000 0.009 0.000 0.009 0.000 0.000 0.000 0.000 0.000

0.060 0.079 0.067 0.069 0.000 0.000 0.000 0.012 0.000 0.006

0.000 0.000 0.006 0.012 0.006 0.000 0.012 0.000 0.006 0.000

0.012 0.000 0.000 0.000 0.072 0.076 0.036 0.080 0.004 0.004

0.000 0.004 0.000 0.000 0.000 0.000 0.000 0.000 0.000 0.000

0.008 0.004 0.012 0.004 0.000 0.000 0.008 0.000 0.089 0.065

0.048 0.082

>Non-Methylation code ID 1470

0.000 0.004 0.000 0.009 0.004 0.000 0.000 0.000 0.000 0.004

0.000 0.004 0.009 0.000 0.009 0.004 0.000 0.004 0.000 0.000

0.077 0.069 0.066 0.067 0.007 0.000 0.000 0.007 0.000 0.000

0.000 0.007 0.007 0.013 0.000 0.013 0.007 0.000 0.000 0.007

0.000 0.007 0.000 0.007 0.070 0.065 0.061 0.057 0.000 0.005

0.000 0.000 0.005 0.000 0.000 0.005 0.005 0.005 0.000 0.000

0.010 0.000 0.010 0.005 0.010 0.000 0.000 0.000 0.066 0.084

0.072 0.051

>Non-Methylation code ID 1470

0.005 0.005 0.000 0.000 0.000 0.025 0.000 0.000 0.000 0.010

0.000 0.000 0.010 0.000 0.010 0.000 0.000 0.000 0.000 0.000

0.071 0.076 0.051 0.070 0.035 0.000 0.000 0.000 0.012 0.046

0.000 0.000 0.000 0.023 0.000 0.000 0.012 0.000 0.000 0.012

0.000 0.012 0.000 0.000 0.057 0.056 0.045 0.026 0.010 0.000

0.000 0.000 0.000 0.010 0.000 0.000 0.000 0.005 0.000 0.000

0.015 0.000 0.010 0.005 0.000 0.000 0.005 0.000 0.087 0.059

0.054 0.072

>Non-Methylation code ID 1470

0.014 0.000 0.000 0.005 0.000 0.014 0.000 0.000 0.000 0.005

0.000 0.000 0.005 0.000 0.014 0.005 0.000 0.000 0.000 0.000

0.086 0.051 0.071 0.065 0.023 0.000 0.006 0.000 0.000 0.012

0.000 0.000 0.000 0.000 0.000 0.000 0.012 0.000 0.012 0.006

0.000 0.000 0.000 0.000 0.083 0.064 0.074 0.041 0.011 0.000

0.000 0.005 0.000 0.016 0.000 0.000 0.000 0.000 0.000 0.000

0.016 0.000 0.016 0.000 0.000 0.000 0.000 0.000 0.079 0.078

0.056 0.056

>Non-Methylation code ID 1470

0.004 0.000 0.000 0.000 0.000 0.004 0.000 0.000 0.000 0.000

0.000 0.004 0.004 0.000 0.027 0.000 0.000 0.008 0.000 0.000

0.051 0.069 0.089 0.074 0.026 0.000 0.006 0.000 0.000 0.019

0.000 0.000 0.000 0.006 0.000 0.000 0.006 0.000 0.006 0.006

0.006 0.000 0.000 0.000 0.057 0.075 0.067 0.050 0.012 0.000

0.000 0.006 0.000 0.006 0.000 0.000 0.000 0.000 0.000 0.000

0.012 0.012 0.012 0.000 0.006 0.000 0.000 0.000 0.087 0.060

0.058 0.063

>Non-Methylation code ID 1470

0.012 0.000 0.000 0.000 0.000 0.006 0.000 0.000 0.000 0.012

0.000 0.000 0.029 0.000 0.006 0.000 0.006 0.000 0.000 0.006

0.064 0.047 0.066 0.081 0.011 0.000 0.000 0.000 0.000 0.000

0.000 0.000 0.000 0.000 0.000 0.000 0.032 0.000 0.011 0.011

0.005 0.000 0.000 0.000 0.058 0.060 0.082 0.064 0.011 0.000

0.000 0.000 0.000 0.000 0.000 0.000 0.000 0.006 0.000 0.000

0.034 0.000 0.017 0.000 0.000 0.000 0.000 0.000 0.079 0.043

0.067 0.076

>Non-Methylation code ID 1470

0.010 0.000 0.005 0.000 0.000 0.000 0.000 0.000 0.000 0.005

0.000 0.000 0.000 0.005 0.020 0.020 0.000 0.000 0.000 0.000

0.088 0.032 0.108 0.039 0.031 0.000 0.000 0.006 0.006 0.006

0.000 0.000 0.000 0.006 0.000 0.000 0.006 0.000 0.006 0.006

0.006 0.000 0.000 0.000 0.063 0.076 0.053 0.062 0.005 0.000

0.000 0.000 0.000 0.005 0.000 0.000 0.005 0.000 0.000 0.000

0.011 0.016 0.011 0.005 0.000 0.005 0.000 0.000 0.061 0.058

0.074 0.075

>Non-Methylation code ID 1470

0.005 0.000 0.000 0.000 0.000 0.009 0.000 0.000 0.000 0.000

0.000 0.000 0.009 0.005 0.014 0.005 0.014 0.000 0.000 0.000

0.075 0.066 0.056 0.075 0.030 0.000 0.000 0.000 0.000 0.000

0.005 0.000 0.000 0.000 0.000 0.000 0.015 0.000 0.015 0.000

0.000 0.000 0.000 0.000 0.075 0.060 0.047 0.086 0.010 0.000

0.000 0.005 0.000 0.000 0.010 0.000 0.000 0.000 0.000 0.000

0.015 0.000 0.015 0.000 0.005 0.000 0.000 0.000 0.082 0.053

0.057 0.082

>Non-Methylation code ID 1470

0.006 0.000 0.000 0.000 0.000 0.000 0.000 0.000 0.000 0.000

0.000 0.006 0.012 0.006 0.023 0.006 0.000 0.000 0.000 0.000

0.068 0.057 0.073 0.079 0.016 0.016 0.000 0.008 0.000 0.008

0.000 0.000 0.000 0.000 0.000 0.000 0.024 0.000 0.000 0.008

0.008 0.008 0.000 0.000 0.060 0.083 0.061 0.036 0.017 0.000

0.000 0.006 0.000 0.000 0.000 0.000 0.000 0.006 0.000 0.000

0.011 0.006 0.011 0.011 0.000 0.000 0.000 0.000 0.072 0.078

0.042 0.073

>Non-Methylation code ID 1470

0.032 0.000 0.000 0.000 0.000 0.008 0.000 0.000 0.000 0.000

0.000 0.000 0.024 0.000 0.008 0.016 0.000 0.008 0.000 0.000

0.062 0.077 0.054 0.046 0.000 0.005 0.000 0.000 0.000 0.009

0.000 0.000 0.000 0.000 0.000 0.000 0.023 0.005 0.014 0.005

0.000 0.000 0.000 0.000 0.086 0.080 0.034 0.074 0.004 0.000

0.000 0.000 0.000 0.009 0.000 0.000 0.000 0.000 0.000 0.000

0.009 0.000 0.018 0.004 0.000 0.009 0.000 0.000 0.060 0.081

0.052 0.087

>Non-Methylation code ID 1470

0.006 0.000 0.000 0.000 0.000 0.006 0.000 0.000 0.000 0.000

0.000 0.000 0.050 0.000 0.006 0.000 0.006 0.000 0.006 0.000

0.071 0.051 0.074 0.055 0.005 0.000 0.005 0.000 0.000 0.005

0.000 0.000 0.000 0.009 0.000 0.000 0.028 0.000 0.009 0.000

0.000 0.000 0.000 0.000 0.068 0.051 0.070 0.083 0.018 0.000

0.000 0.000 0.000 0.000 0.006 0.000 0.000 0.000 0.000 0.000

0.030 0.000 0.006 0.000 0.006 0.000 0.006 0.000 0.078 0.055

0.060 0.066

>Non-Methylation code ID 1470

0.043 0.011 0.000 0.000 0.000 0.000 0.000 0.000 0.000 0.000

0.000 0.000 0.032 0.000 0.000 0.022 0.000 0.022 0.000 0.000

0.048 0.065 0.044 0.047 0.005 0.005 0.000 0.000 0.000 0.000

0.000 0.000 0.000 0.000 0.000 0.000 0.023 0.005 0.014 0.009

0.000 0.000 0.000 0.000 0.079 0.078 0.049 0.068 0.008 0.000

0.000 0.000 0.000 0.000 0.000 0.000 0.000 0.000 0.000 0.000

0.004 0.000 0.025 0.004 0.000 0.008 0.000 0.000 0.064 0.086

0.045 0.088

>Non-Methylation code ID 1470

0.025 0.000 0.000 0.000 0.000 0.000 0.000 0.000 0.000 0.000

0.000 0.000 0.015 0.000 0.015 0.005 0.005 0.000 0.000 0.000

0.064 0.063 0.054 0.088 0.016 0.000 0.000 0.005 0.000 0.000

0.011 0.000 0.000 0.005 0.000 0.000 0.016 0.005 0.011 0.000

0.000 0.000 0.000 0.000 0.085 0.045 0.063 0.072 0.000 0.000

0.000 0.000 0.000 0.012 0.006 0.000 0.006 0.000 0.000 0.000

0.012 0.006 0.012 0.006 0.012 0.000 0.000 0.000 0.078 0.069

0.061 0.053

>Non-Methylation code ID 1470

0.011 0.000 0.000 0.000 0.000 0.000 0.000 0.000 0.000 0.000

0.000 0.000 0.032 0.000 0.011 0.011 0.005 0.000 0.000 0.000

0.058 0.060 0.082 0.064 0.010 0.000 0.000 0.000 0.000 0.000

0.000 0.000 0.000 0.005 0.000 0.000 0.029 0.000 0.020 0.000

0.000 0.000 0.000 0.000 0.068 0.050 0.072 0.079 0.012 0.000

0.000 0.000 0.000 0.006 0.000 0.000 0.000 0.012 0.000 0.000

0.030 0.000 0.006 0.000 0.000 0.000 0.000 0.006 0.065 0.045

0.068 0.082

>Non-Methylation code ID 1470

0.000 0.000 0.000 0.000 0.000 0.000 0.000 0.000 0.005 0.000

0.000 0.000 0.025 0.000 0.015 0.015 0.005 0.000 0.000 0.000

0.092 0.040 0.077 0.059 0.021 0.000 0.000 0.000 0.000 0.007

0.000 0.000 0.000 0.000 0.000 0.000 0.043 0.007 0.007 0.007

0.000 0.000 0.000 0.000 0.073 0.058 0.055 0.055 0.020 0.000

0.000 0.000 0.000 0.000 0.007 0.000 0.007 0.013 0.000 0.000

0.026 0.007 0.000 0.000 0.000 0.000 0.000 0.000 0.066 0.054

0.058 0.078

>Non-Methylation code ID 1470

0.014 0.000 0.000 0.000 0.000 0.014 0.000 0.000 0.000 0.000

0.000 0.000 0.042 0.000 0.000 0.007 0.007 0.000 0.007 0.000

0.064 0.049 0.070 0.059 0.007 0.000 0.007 0.000 0.000 0.007

0.007 0.000 0.000 0.007 0.000 0.000 0.049 0.000 0.007 0.000

0.000 0.000 0.000 0.000 0.059 0.063 0.049 0.071 0.005 0.000

0.000 0.000 0.000 0.000 0.005 0.000 0.000 0.009 0.000 0.000

0.009 0.000 0.014 0.000 0.009 0.000 0.005 0.000 0.097 0.045

0.081 0.054

>Non-Methylation code ID 1470

0.009 0.000 0.000 0.000 0.000 0.009 0.000 0.000 0.000 0.000

0.000 0.000 0.014 0.000 0.014 0.000 0.014 0.000 0.000 0.000

0.076 0.072 0.056 0.070 0.030 0.000 0.000 0.000 0.000 0.000

0.006 0.000 0.000 0.012 0.000 0.000 0.018 0.000 0.012 0.000

0.000 0.000 0.000 0.000 0.079 0.045 0.063 0.068 0.005 0.000

0.000 0.000 0.000 0.000 0.010 0.000 0.000 0.000 0.000 0.000

0.014 0.000 0.014 0.005 0.005 0.000 0.000 0.005 0.072 0.054

0.067 0.083

>Non-Methylation code ID 1470

0.018 0.000 0.000 0.000 0.000 0.006 0.000 0.000 0.000 0.012

0.000 0.000 0.030 0.000 0.006 0.000 0.000 0.000 0.000 0.006

0.062 0.046 0.066 0.080 0.011 0.000 0.000 0.000 0.000 0.000

0.000 0.000 0.000 0.000 0.000 0.000 0.027 0.005 0.011 0.011

0.005 0.000 0.000 0.000 0.067 0.064 0.072 0.060 0.011 0.000

0.000 0.000 0.000 0.000 0.000 0.000 0.000 0.005 0.000 0.000

0.027 0.000 0.016 0.005 0.000 0.000 0.000 0.000 0.058 0.067

0.068 0.074

>Non-Methylation code ID 1470

0.016 0.016 0.000 0.008 0.000 0.008 0.000 0.000 0.000 0.000

0.000 0.000 0.024 0.000 0.000 0.008 0.008 0.008 0.000 0.000

0.066 0.088 0.051 0.031 0.009 0.005 0.000 0.005 0.000 0.000

0.000 0.000 0.000 0.005 0.000 0.000 0.014 0.005 0.009 0.009

0.000 0.000 0.000 0.000 0.067 0.069 0.055 0.081 0.006 0.000

0.000 0.000 0.000 0.000 0.000 0.000 0.000 0.000 0.000 0.006

0.012 0.000 0.018 0.006 0.000 0.006 0.000 0.000 0.051 0.057

0.065 0.107

>Non-Methylation code ID 1470

0.000 0.000 0.000 0.000 0.000 0.011 0.005 0.000 0.005 0.000

0.000 0.000 0.011 0.005 0.011 0.005 0.011 0.000 0.005 0.000

0.077 0.073 0.062 0.053 0.024 0.000 0.000 0.000 0.000 0.005

0.000 0.000 0.000 0.000 0.000 0.000 0.010 0.000 0.014 0.005

0.005 0.000 0.000 0.000 0.061 0.062 0.049 0.099 0.018 0.000

0.000 0.006 0.000 0.000 0.012 0.000 0.000 0.006 0.000 0.000

0.018 0.006 0.006 0.000 0.000 0.000 0.000 0.000 0.082 0.051

0.055 0.074

>Non-Methylation code ID 1470

0.005 0.000 0.000 0.000 0.000 0.000 0.005 0.000 0.005 0.010

0.000 0.000 0.020 0.000 0.010 0.005 0.005 0.000 0.000 0.000

0.072 0.047 0.070 0.079 0.011 0.000 0.000 0.000 0.000 0.000

0.000 0.000 0.000 0.000 0.000 0.000 0.027 0.000 0.011 0.016

0.005 0.000 0.000 0.000 0.073 0.055 0.065 0.070 0.017 0.000

0.000 0.000 0.000 0.009 0.009 0.000 0.000 0.009 0.000 0.000

0.052 0.009 0.000 0.000 0.000 0.000 0.000 0.000 0.066 0.069

0.054 0.040

>Non-Methylation code ID 1470

0.005 0.000 0.000 0.000 0.000 0.000 0.005 0.000 0.005 0.000

0.000 0.000 0.011 0.005 0.005 0.016 0.011 0.000 0.005 0.000

0.061 0.056 0.076 0.070 0.021 0.000 0.000 0.000 0.000 0.007

0.000 0.000 0.000 0.007 0.000 0.000 0.028 0.000 0.014 0.007

0.007 0.000 0.000 0.000 0.048 0.061 0.060 0.072 0.011 0.000

0.000 0.011 0.005 0.000 0.005 0.000 0.000 0.005 0.000 0.000

0.016 0.005 0.005 0.000 0.000 0.000 0.000 0.000 0.078 0.063

0.073 0.056

>Non-Methylation code ID 1470

0.006 0.000 0.000 0.000 0.000 0.006 0.000 0.000 0.000 0.000

0.000 0.000 0.040 0.000 0.011 0.006 0.006 0.000 0.000 0.000

0.068 0.055 0.073 0.064 0.011 0.000 0.005 0.000 0.000 0.000

0.000 0.000 0.000 0.011 0.000 0.000 0.032 0.000 0.011 0.000

0.000 0.000 0.000 0.000 0.080 0.045 0.064 0.075 0.011 0.000

0.000 0.000 0.000 0.000 0.000 0.000 0.000 0.006 0.000 0.000

0.028 0.000 0.006 0.000 0.006 0.000 0.006 0.006 0.067 0.049

0.071 0.078

>Non-Methylation code ID 3162

0.005 0.000 0.000 0.000 0.000 0.000 0.009 0.000 0.000 0.000

0.000 0.000 0.014 0.000 0.014 0.009 0.005 0.000 0.000 0.005

0.069 0.055 0.063 0.087 0.009 0.000 0.000 0.000 0.000 0.009

0.000 0.000 0.000 0.000 0.000 0.000 0.014 0.000 0.014 0.000

0.014 0.000 0.000 0.000 0.076 0.072 0.056 0.070 0.036 0.000

0.000 0.000 0.000 0.000 0.007 0.000 0.000 0.014 0.000 0.000

0.022 0.000 0.007 0.000 0.000 0.000 0.000 0.000 0.077 0.056

0.056 0.059

>Non-Methylation code ID 3162

0.006 0.000 0.000 0.000 0.000 0.006 0.000 0.000 0.000 0.000

0.000 0.000 0.040 0.000 0.011 0.000 0.011 0.000 0.000 0.000

0.063 0.057 0.073 0.067 0.010 0.000 0.005 0.000 0.000 0.000

0.000 0.000 0.000 0.010 0.000 0.000 0.030 0.000 0.010 0.000

0.000 0.000 0.000 0.000 0.077 0.048 0.071 0.071 0.012 0.000

0.000 0.000 0.000 0.000 0.000 0.000 0.000 0.000 0.000 0.000

0.035 0.000 0.006 0.000 0.006 0.000 0.006 0.006 0.076 0.054

0.072 0.060

>Non-Methylation code ID 3162

0.005 0.000 0.005 0.000 0.000 0.005 0.000 0.000 0.000 0.005

0.000 0.000 0.033 0.000 0.009 0.000 0.000 0.000 0.000 0.000

0.069 0.048 0.070 0.084 0.017 0.000 0.000 0.000 0.000 0.000

0.006 0.000 0.000 0.006 0.000 0.000 0.029 0.000 0.006 0.000

0.006 0.000 0.006 0.000 0.075 0.059 0.061 0.065 0.007 0.000

0.000 0.000 0.000 0.007 0.000 0.000 0.000 0.000 0.000 0.000

0.048 0.000 0.007 0.000 0.007 0.000 0.007 0.000 0.073 0.056

0.061 0.062

>Non-Methylation code ID 3162

0.021 0.000 0.000 0.000 0.000 0.007 0.000 0.000 0.000 0.007

0.000 0.000 0.028 0.000 0.014 0.007 0.007 0.000 0.000 0.000

0.048 0.061 0.060 0.072 0.010 0.000 0.000 0.010 0.005 0.000

0.005 0.000 0.000 0.005 0.000 0.000 0.020 0.005 0.005 0.000

0.000 0.000 0.000 0.000 0.079 0.058 0.079 0.051 0.006 0.000

0.000 0.000 0.000 0.000 0.006 0.000 0.006 0.000 0.000 0.000

0.011 0.006 0.006 0.017 0.006 0.000 0.006 0.000 0.062 0.058

0.074 0.071

>Non-Methylation code ID 3162

0.005 0.000 0.005 0.000 0.000 0.005 0.000 0.000 0.000 0.009

0.000 0.000 0.028 0.000 0.009 0.000 0.000 0.000 0.000 0.000

0.068 0.051 0.070 0.083 0.016 0.000 0.000 0.000 0.000 0.000

0.005 0.000 0.000 0.000 0.000 0.000 0.027 0.000 0.005 0.000

0.005 0.000 0.005 0.005 0.076 0.060 0.061 0.067 0.007 0.000

0.000 0.000 0.000 0.007 0.000 0.000 0.000 0.000 0.000 0.000

0.060 0.000 0.007 0.000 0.007 0.000 0.000 0.000 0.070 0.052

0.073 0.048

>Non-Methylation code ID 3162

0.012 0.000 0.000 0.000 0.000 0.006 0.000 0.000 0.000 0.000

0.000 0.000 0.031 0.000 0.006 0.006 0.012 0.000 0.006 0.000

0.062 0.062 0.071 0.058 0.019 0.000 0.000 0.000 0.000 0.009

0.009 0.000 0.000 0.009 0.000 0.000 0.075 0.000 0.000 0.000

0.000 0.000 0.000 0.000 0.062 0.064 0.032 0.053 0.004 0.000

0.000 0.000 0.000 0.000 0.009 0.000 0.000 0.009 0.000 0.000

0.013 0.000 0.013 0.000 0.004 0.000 0.000 0.000 0.088 0.046

0.078 0.068

>Non-Methylation code ID 3162

0.000 0.000 0.000 0.000 0.000 0.000 0.005 0.000 0.005 0.010

0.000 0.000 0.024 0.000 0.010 0.005 0.005 0.000 0.000 0.000

0.079 0.042 0.079 0.071 0.011 0.000 0.000 0.000 0.000 0.000

0.000 0.000 0.000 0.000 0.000 0.000 0.028 0.000 0.011 0.017

0.006 0.000 0.000 0.000 0.066 0.053 0.073 0.068 0.018 0.000

0.000 0.000 0.000 0.000 0.009 0.000 0.000 0.009 0.000 0.000

0.062 0.009 0.000 0.000 0.000 0.000 0.000 0.000 0.067 0.062

0.067 0.031

>Non-Methylation code ID 3162

0.019 0.000 0.000 0.000 0.000 0.000 0.006 0.000 0.006 0.012

0.000 0.000 0.025 0.006 0.000 0.000 0.006 0.000 0.000 0.000

0.067 0.053 0.056 0.078 0.000 0.000 0.000 0.000 0.000 0.000

0.000 0.000 0.005 0.000 0.000 0.000 0.025 0.000 0.015 0.015

0.005 0.000 0.000 0.000 0.092 0.040 0.077 0.059 0.016 0.000

0.000 0.000 0.000 0.008 0.000 0.000 0.000 0.000 0.000 0.000

0.047 0.008 0.008 0.008 0.000 0.000 0.000 0.000 0.060 0.062

0.056 0.062

>Non-Methylation code ID 3164

0.011 0.005 0.000 0.005 0.000 0.005 0.000 0.000 0.000 0.000

0.000 0.000 0.005 0.000 0.016 0.016 0.005 0.000 0.000 0.000

0.079 0.046 0.091 0.047 0.029 0.000 0.000 0.012 0.000 0.006

0.000 0.000 0.000 0.006 0.000 0.000 0.006 0.000 0.012 0.006

0.000 0.000 0.000 0.000 0.062 0.074 0.064 0.057 0.007 0.000

0.000 0.000 0.000 0.007 0.000 0.000 0.007 0.000 0.000 0.007

0.007 0.022 0.014 0.000 0.000 0.000 0.000 0.000 0.066 0.061

0.074 0.060

>Non-Methylation code ID 3164

0.011 0.000 0.000 0.006 0.006 0.000 0.000 0.000 0.000 0.006

0.000 0.000 0.011 0.000 0.011 0.017 0.006 0.000 0.000 0.000

0.063 0.081 0.057 0.060 0.018 0.006 0.000 0.000 0.000 0.006

0.000 0.000 0.006 0.006 0.000 0.000 0.012 0.006 0.006 0.006

0.000 0.006 0.000 0.000 0.070 0.054 0.064 0.069 0.014 0.000

0.007 0.000 0.000 0.000 0.000 0.000 0.000 0.014 0.000 0.000

0.007 0.007 0.014 0.014 0.000 0.000 0.000 0.000 0.069 0.037

0.075 0.075

>Non-Methylation code ID 3164

0.021 0.000 0.000 0.000 0.000 0.007 0.000 0.000 0.000 0.000

0.000 0.000 0.035 0.007 0.007 0.014 0.000 0.000 0.000 0.000

0.069 0.069 0.053 0.052 0.016 0.000 0.000 0.000 0.000 0.000

0.005 0.000 0.005 0.011 0.000 0.000 0.021 0.005 0.005 0.000

0.000 0.000 0.000 0.000 0.076 0.057 0.056 0.074 0.000 0.000

0.000 0.000 0.000 0.000 0.000 0.000 0.006 0.000 0.000 0.000

0.023 0.000 0.011 0.017 0.006 0.006 0.000 0.000 0.095 0.033

0.077 0.060

>Non-Methylation code ID 3164

0.009 0.004 0.000 0.004 0.000 0.000 0.000 0.000 0.000 0.000

0.000 0.000 0.013 0.004 0.013 0.009 0.000 0.000 0.000 0.000

0.069 0.077 0.062 0.069 0.004 0.000 0.000 0.000 0.000 0.000

0.000 0.000 0.000 0.000 0.000 0.004 0.008 0.000 0.021 0.004

0.000 0.008 0.000 0.000 0.057 0.070 0.062 0.095 0.020 0.010

0.000 0.000 0.000 0.010 0.000 0.000 0.000 0.000 0.000 0.000

0.031 0.000 0.000 0.010 0.010 0.020 0.000 0.000 0.060 0.079

0.041 0.041

>Non-Methylation code ID 3164

0.016 0.000 0.000 0.000 0.000 0.000 0.005 0.000 0.000 0.000

0.000 0.000 0.027 0.000 0.005 0.000 0.005 0.000 0.005 0.005

0.076 0.060 0.061 0.067 0.007 0.000 0.000 0.000 0.000 0.007

0.000 0.000 0.000 0.000 0.000 0.000 0.054 0.000 0.007 0.000

0.013 0.000 0.000 0.000 0.068 0.049 0.071 0.057 0.005 0.000

0.005 0.000 0.000 0.000 0.000 0.000 0.000 0.010 0.000 0.000

0.030 0.000 0.010 0.000 0.000 0.000 0.000 0.000 0.069 0.051

0.073 0.082

>Non-Methylation code ID 3164

0.005 0.000 0.000 0.000 0.000 0.010 0.000 0.000 0.005 0.000

0.000 0.000 0.010 0.005 0.014 0.005 0.010 0.000 0.000 0.000

0.071 0.067 0.060 0.072 0.031 0.000 0.000 0.000 0.000 0.000

0.000 0.000 0.000 0.000 0.000 0.000 0.016 0.000 0.016 0.005

0.000 0.000 0.000 0.000 0.070 0.064 0.050 0.081 0.011 0.000

0.000 0.005 0.000 0.000 0.011 0.000 0.000 0.000 0.000 0.000

0.016 0.005 0.016 0.000 0.000 0.000 0.000 0.000 0.081 0.051

0.058 0.081

>Non-Methylation code ID 3164

0.004 0.000 0.000 0.000 0.000 0.000 0.000 0.000 0.000 0.000

0.000 0.004 0.008 0.000 0.021 0.004 0.000 0.008 0.000 0.000

0.057 0.070 0.062 0.095 0.019 0.009 0.000 0.000 0.000 0.009

0.000 0.000 0.000 0.000 0.000 0.000 0.028 0.000 0.000 0.019

0.009 0.019 0.000 0.000 0.058 0.076 0.037 0.048 0.005 0.005

0.000 0.005 0.000 0.000 0.000 0.000 0.000 0.000 0.000 0.000

0.014 0.005 0.014 0.009 0.000 0.000 0.000 0.000 0.072 0.078

0.053 0.076

>Non-Methylation code ID 3164

0.005 0.000 0.000 0.010 0.005 0.000 0.005 0.000 0.000 0.005

0.000 0.000 0.020 0.005 0.005 0.000 0.005 0.000 0.000 0.000

0.077 0.056 0.082 0.054 0.005 0.000 0.000 0.000 0.000 0.000

0.005 0.000 0.005 0.000 0.000 0.000 0.020 0.000 0.005 0.015

0.005 0.000 0.005 0.000 0.059 0.066 0.082 0.061 0.029 0.000

0.000 0.000 0.000 0.010 0.010 0.000 0.000 0.010 0.000 0.000

0.038 0.000 0.000 0.010 0.010 0.000 0.000 0.000 0.051 0.053

0.057 0.058

>Non-Methylation code ID 3164

0.006 0.006 0.000 0.000 0.000 0.012 0.006 0.000 0.000 0.006

0.000 0.000 0.030 0.000 0.006 0.000 0.006 0.000 0.000 0.000

0.086 0.051 0.074 0.044 0.005 0.000 0.005 0.000 0.000 0.010

0.000 0.000 0.000 0.010 0.000 0.000 0.014 0.000 0.010 0.005

0.005 0.000 0.000 0.000 0.064 0.054 0.064 0.089 0.019 0.000

0.000 0.000 0.000 0.006 0.006 0.000 0.000 0.000 0.006 0.000

0.019 0.000 0.006 0.013 0.000 0.000 0.000 0.000 0.058 0.069

0.069 0.061

>Non-Methylation code ID 3164

0.010 0.000 0.000 0.000 0.000 0.010 0.005 0.000 0.000 0.000

0.000 0.000 0.016 0.000 0.010 0.000 0.010 0.000 0.000 0.000

0.076 0.064 0.064 0.067 0.027 0.000 0.000 0.007 0.000 0.000

0.007 0.000 0.000 0.014 0.000 0.000 0.020 0.000 0.007 0.000

0.007 0.000 0.000 0.000 0.061 0.052 0.064 0.068 0.005 0.000

0.000 0.000 0.000 0.000 0.005 0.000 0.000 0.005 0.000 0.000

0.011 0.000 0.016 0.011 0.005 0.000 0.000 0.005 0.067 0.054

0.060 0.088

>Non-Methylation code ID 3164

0.014 0.000 0.000 0.000 0.000 0.007 0.007 0.000 0.000 0.000

0.000 0.000 0.041 0.007 0.007 0.007 0.000 0.000 0.000 0.000

0.060 0.064 0.058 0.063 0.019 0.000 0.000 0.000 0.000 0.000

0.006 0.000 0.006 0.012 0.000 0.000 0.025 0.006 0.000 0.000

0.006 0.000 0.000 0.000 0.067 0.053 0.056 0.078 0.000 0.000

0.000 0.000 0.000 0.000 0.000 0.000 0.006 0.000 0.000 0.000

0.028 0.000 0.011 0.017 0.006 0.000 0.000 0.000 0.087 0.043

0.070 0.067

>Non-Methylation code ID 3164

0.018 0.000 0.000 0.000 0.000 0.006 0.000 0.000 0.000 0.000

0.000 0.000 0.024 0.000 0.006 0.012 0.006 0.000 0.006 0.000

0.057 0.068 0.083 0.049 0.018 0.000 0.000 0.000 0.000 0.009

0.009 0.000 0.000 0.009 0.000 0.000 0.072 0.000 0.000 0.000

0.000 0.000 0.000 0.000 0.064 0.048 0.055 0.049 0.000 0.000

0.000 0.000 0.005 0.000 0.005 0.000 0.000 0.009 0.000 0.000

0.019 0.000 0.014 0.000 0.005 0.000 0.000 0.000 0.102 0.035

0.089 0.051

>Non-Methylation code ID 3164

0.012 0.012 0.000 0.006 0.000 0.006 0.000 0.000 0.000 0.000

0.000 0.000 0.012 0.000 0.006 0.012 0.006 0.006 0.000 0.000

0.058 0.075 0.067 0.053 0.029 0.000 0.000 0.006 0.000 0.000

0.000 0.000 0.000 0.006 0.000 0.000 0.012 0.000 0.012 0.012

0.000 0.000 0.000 0.000 0.068 0.074 0.045 0.070 0.007 0.000

0.000 0.000 0.000 0.000 0.000 0.000 0.000 0.000 0.000 0.007

0.007 0.014 0.028 0.000 0.000 0.000 0.000 0.000 0.073 0.057

0.062 0.080

>Non-Methylation code ID 3164

0.013 0.000 0.000 0.000 0.000 0.000 0.000 0.000 0.000 0.000

0.000 0.006 0.006 0.013 0.025 0.000 0.000 0.000 0.000 0.000

0.070 0.055 0.075 0.071 0.012 0.012 0.000 0.006 0.000 0.006

0.000 0.000 0.000 0.000 0.000 0.000 0.012 0.000 0.006 0.012

0.006 0.006 0.000 0.000 0.058 0.075 0.067 0.053 0.034 0.000

0.000 0.007 0.000 0.000 0.000 0.000 0.000 0.007 0.000 0.000

0.007 0.000 0.014 0.014 0.000 0.000 0.000 0.000 0.062 0.081

0.049 0.060

>Non-Methylation code ID 3164

0.005 0.000 0.000 0.000 0.000 0.009 0.000 0.000 0.000 0.000

0.000 0.000 0.014 0.005 0.014 0.000 0.014 0.000 0.000 0.000

0.076 0.076 0.048 0.072 0.028 0.000 0.000 0.000 0.000 0.000

0.006 0.000 0.000 0.006 0.000 0.000 0.017 0.000 0.017 0.000

0.000 0.000 0.000 0.000 0.074 0.052 0.058 0.077 0.010 0.000

0.000 0.000 0.000 0.000 0.010 0.000 0.000 0.000 0.000 0.000

0.015 0.000 0.015 0.000 0.005 0.000 0.000 0.005 0.077 0.055

0.061 0.083

>Non-Methylation code ID 3164

0.000 0.004 0.000 0.000 0.000 0.009 0.000 0.000 0.000 0.000

0.000 0.000 0.021 0.000 0.013 0.004 0.004 0.000 0.000 0.000

0.085 0.083 0.033 0.076 0.009 0.000 0.004 0.000 0.000 0.009

0.000 0.000 0.000 0.000 0.000 0.000 0.009 0.000 0.022 0.000

0.000 0.004 0.000 0.000 0.067 0.079 0.048 0.082 0.026 0.000

0.000 0.000 0.000 0.007 0.007 0.000 0.000 0.000 0.007 0.000

0.013 0.000 0.007 0.007 0.000 0.007 0.000 0.000 0.061 0.074

0.071 0.049

>Non-Methylation code ID 3167

0.000 0.000 0.000 0.000 0.004 0.000 0.004 0.000 0.000 0.008

0.000 0.000 0.016 0.000 0.016 0.000 0.004 0.000 0.000 0.000

0.098 0.043 0.088 0.050 0.018 0.000 0.000 0.000 0.000 0.006

0.000 0.000 0.000 0.000 0.000 0.000 0.024 0.000 0.006 0.012

0.006 0.000 0.006 0.000 0.057 0.068 0.083 0.049 0.020 0.000

0.000 0.000 0.000 0.000 0.010 0.000 0.000 0.010 0.000 0.000

0.079 0.000 0.000 0.000 0.000 0.000 0.000 0.000 0.063 0.045

0.061 0.045

>Non-Methylation code ID 3167

0.010 0.000 0.000 0.010 0.005 0.000 0.005 0.000 0.000 0.005

0.000 0.000 0.020 0.005 0.005 0.000 0.000 0.000 0.000 0.000

0.079 0.058 0.079 0.051 0.005 0.000 0.000 0.000 0.000 0.000

0.005 0.000 0.005 0.000 0.000 0.000 0.016 0.005 0.005 0.016

0.005 0.000 0.005 0.000 0.062 0.067 0.073 0.064 0.026 0.000

0.000 0.000 0.000 0.009 0.000 0.000 0.000 0.009 0.000 0.000

0.034 0.000 0.009 0.009 0.009 0.000 0.000 0.000 0.058 0.049

0.056 0.069

>Non-Methylation code ID 3167

0.007 0.000 0.000 0.007 0.007 0.007 0.000 0.000 0.000 0.007

0.000 0.014 0.014 0.007 0.000 0.000 0.007 0.007 0.000 0.000

0.066 0.048 0.063 0.070 0.006 0.000 0.000 0.000 0.000 0.000

0.006 0.000 0.000 0.006 0.012 0.006 0.006 0.006 0.006 0.006

0.000 0.000 0.012 0.006 0.064 0.068 0.058 0.064 0.000 0.011

0.000 0.000 0.000 0.011 0.005 0.011 0.005 0.000 0.000 0.000

0.005 0.005 0.000 0.000 0.005 0.000 0.005 0.000 0.053 0.062

0.071 0.084

>Non-Methylation code ID 3167

0.000 0.000 0.000 0.000 0.000 0.000 0.000 0.006 0.000 0.019

0.006 0.006 0.019 0.006 0.006 0.013 0.000 0.000 0.000 0.000

0.069 0.084 0.049 0.050 0.000 0.008 0.000 0.000 0.000 0.000

0.023 0.008 0.000 0.008 0.000 0.000 0.008 0.000 0.000 0.015

0.000 0.008 0.000 0.008 0.065 0.071 0.054 0.060 0.008 0.000

0.008 0.008 0.000 0.000 0.000 0.015 0.000 0.000 0.000 0.000

0.015 0.000 0.000 0.015 0.015 0.000 0.000 0.008 0.057 0.061

0.048 0.078

>Non-Methylation code ID 3167

0.000 0.014 0.000 0.000 0.000 0.007 0.007 0.007 0.000 0.000

0.000 0.000 0.000 0.000 0.000 0.014 0.007 0.014 0.000 0.007

0.069 0.063 0.057 0.065 0.007 0.000 0.007 0.007 0.007 0.007

0.000 0.007 0.000 0.000 0.000 0.000 0.007 0.000 0.007 0.014

0.007 0.007 0.000 0.000 0.060 0.061 0.057 0.073 0.000 0.000

0.000 0.000 0.000 0.007 0.000 0.007 0.000 0.027 0.000 0.007

0.007 0.007 0.007 0.000 0.000 0.000 0.007 0.007 0.076 0.090

0.039 0.048

>Non-Methylation code ID 3167

0.000 0.000 0.000 0.000 0.007 0.000 0.015 0.000 0.000 0.022

0.000 0.007 0.007 0.000 0.000 0.015 0.000 0.000 0.000 0.007

0.054 0.062 0.060 0.076 0.000 0.000 0.014 0.000 0.007 0.000

0.000 0.014 0.000 0.000 0.000 0.000 0.014 0.000 0.000 0.007

0.014 0.007 0.000 0.007 0.065 0.077 0.046 0.061 0.000 0.000

0.000 0.008 0.008 0.000 0.000 0.016 0.000 0.016 0.008 0.000

0.016 0.000 0.000 0.024 0.000 0.000 0.000 0.000 0.068 0.069

0.035 0.064

>Non-Methylation code ID 3167

0.000 0.000 0.000 0.000 0.000 0.013 0.007 0.013 0.007 0.007

0.000 0.007 0.020 0.000 0.000 0.007 0.007 0.000 0.000 0.000

0.064 0.051 0.058 0.073 0.007 0.000 0.000 0.007 0.007 0.000

0.000 0.000 0.000 0.007 0.000 0.000 0.027 0.000 0.007 0.000

0.014 0.007 0.000 0.000 0.062 0.051 0.059 0.079 0.000 0.000

0.000 0.005 0.000 0.000 0.005 0.000 0.005 0.000 0.000 0.005

0.020 0.000 0.005 0.005 0.000 0.000 0.005 0.005 0.069 0.073

0.059 0.072

>Non-Methylation code ID 3167

0.000 0.000 0.015 0.000 0.007 0.000 0.000 0.015 0.000 0.000

0.000 0.000 0.007 0.000 0.000 0.007 0.015 0.015 0.000 0.007

0.063 0.081 0.041 0.061 0.000 0.000 0.000 0.008 0.008 0.000

0.000 0.016 0.000 0.016 0.008 0.000 0.016 0.000 0.000 0.024

0.000 0.000 0.000 0.000 0.068 0.069 0.035 0.064 0.000 0.000

0.000 0.000 0.008 0.000 0.016 0.000 0.000 0.024 0.000 0.008

0.008 0.000 0.000 0.008 0.000 0.000 0.000 0.008 0.055 0.062

0.059 0.077

>Non-Methylation code ID 3167

0.000 0.000 0.000 0.008 0.008 0.000 0.000 0.016 0.000 0.016

0.008 0.000 0.016 0.000 0.000 0.024 0.000 0.000 0.000 0.000

0.068 0.069 0.035 0.064 0.000 0.000 0.000 0.000 0.007 0.000

0.015 0.000 0.000 0.022 0.000 0.007 0.007 0.000 0.000 0.015

0.000 0.000 0.000 0.007 0.054 0.062 0.060 0.076 0.000 0.000

0.016 0.000 0.008 0.000 0.000 0.016 0.000 0.000 0.000 0.000

0.008 0.000 0.000 0.008 0.016 0.008 0.000 0.008 0.065 0.075

0.045 0.062

>Non-Methylation code ID 3167

0.013 0.000 0.000 0.000 0.000 0.019 0.006 0.000 0.000 0.000

0.000 0.006 0.006 0.006 0.006 0.000 0.013 0.006 0.000 0.000

0.067 0.077 0.055 0.053 0.000 0.000 0.006 0.000 0.000 0.019

0.000 0.000 0.000 0.013 0.006 0.000 0.006 0.013 0.006 0.006

0.006 0.000 0.000 0.000 0.067 0.055 0.057 0.071 0.000 0.005

0.000 0.000 0.000 0.021 0.010 0.000 0.005 0.000 0.000 0.000

0.000 0.000 0.005 0.005 0.005 0.000 0.000 0.000 0.068 0.069

0.056 0.083

>Non-Methylation code ID 3167

0.000 0.000 0.013 0.000 0.000 0.000 0.000 0.013 0.007 0.007

0.000 0.007 0.000 0.007 0.000 0.013 0.000 0.000 0.007 0.007

0.059 0.054 0.070 0.072 0.000 0.000 0.000 0.000 0.000 0.006

0.000 0.000 0.006 0.000 0.000 0.000 0.006 0.000 0.011 0.017

0.022 0.000 0.006 0.000 0.075 0.067 0.046 0.074 0.000 0.000

0.006 0.000 0.000 0.012 0.000 0.000 0.000 0.012 0.000 0.006

0.012 0.012 0.006 0.000 0.000 0.006 0.000 0.000 0.063 0.067

0.070 0.063

>Non-Methylation code ID 3168

0.000 0.006 0.000 0.000 0.006 0.018 0.006 0.000 0.012 0.000

0.000 0.000 0.006 0.000 0.000 0.018 0.006 0.000 0.000 0.000

0.068 0.066 0.068 0.055 0.008 0.000 0.000 0.000 0.000 0.017

0.008 0.000 0.000 0.000 0.000 0.008 0.017 0.008 0.000 0.017

0.008 0.017 0.000 0.000 0.073 0.068 0.048 0.037 0.000 0.000

0.000 0.000 0.000 0.007 0.000 0.000 0.000 0.007 0.007 0.000

0.000 0.021 0.007 0.007 0.007 0.014 0.000 0.007 0.061 0.058

0.045 0.084

>Non-Methylation code ID 3168

0.000 0.000 0.014 0.000 0.000 0.007 0.000 0.014 0.007 0.014

0.000 0.007 0.000 0.007 0.000 0.021 0.000 0.000 0.000 0.000

0.055 0.055 0.057 0.077 0.006 0.000 0.000 0.000 0.000 0.000

0.000 0.000 0.006 0.006 0.000 0.000 0.006 0.000 0.012 0.018

0.018 0.000 0.000 0.006 0.078 0.072 0.034 0.071 0.000 0.000

0.006 0.000 0.000 0.006 0.000 0.000 0.000 0.025 0.000 0.006

0.006 0.013 0.006 0.000 0.006 0.000 0.000 0.000 0.061 0.060

0.081 0.056

>Non-Methylation code ID 3168

0.005 0.000 0.005 0.005 0.000 0.000 0.005 0.000 0.000 0.005

0.000 0.000 0.005 0.000 0.009 0.005 0.009 0.005 0.005 0.000

0.057 0.066 0.073 0.077 0.009 0.000 0.000 0.000 0.000 0.005

0.000 0.009 0.005 0.005 0.000 0.000 0.000 0.000 0.014 0.005

0.000 0.005 0.000 0.005 0.051 0.084 0.065 0.075 0.008 0.000

0.000 0.000 0.000 0.016 0.008 0.000 0.000 0.008 0.000 0.008

0.000 0.008 0.008 0.016 0.008 0.000 0.000 0.000 0.069 0.074

0.050 0.054

>Non-Methylation code ID 3168

0.000 0.010 0.000 0.000 0.000 0.014 0.005 0.010 0.005 0.000

0.000 0.000 0.005 0.005 0.000 0.000 0.005 0.000 0.005 0.000

0.055 0.063 0.067 0.086 0.007 0.000 0.000 0.007 0.007 0.015

0.000 0.000 0.000 0.015 0.000 0.015 0.007 0.000 0.000 0.000

0.007 0.007 0.000 0.000 0.063 0.046 0.067 0.069 0.007 0.000

0.000 0.000 0.000 0.000 0.000 0.000 0.000 0.007 0.015 0.007

0.007 0.007 0.000 0.007 0.000 0.000 0.022 0.007 0.052 0.052

0.077 0.063

>Non-Methylation code ID 3168

0.000 0.000 0.007 0.000 0.007 0.000 0.000 0.015 0.000 0.000

0.000 0.000 0.015 0.000 0.000 0.015 0.015 0.007 0.000 0.007

0.059 0.072 0.045 0.067 0.000 0.000 0.000 0.006 0.006 0.000

0.000 0.006 0.000 0.013 0.006 0.000 0.019 0.000 0.006 0.019

0.000 0.000 0.000 0.000 0.081 0.075 0.043 0.054 0.000 0.000

0.000 0.000 0.008 0.000 0.023 0.000 0.000 0.015 0.000 0.000

0.008 0.000 0.000 0.015 0.000 0.008 0.000 0.008 0.060 0.070

0.048 0.071

>Non-Methylation code ID 3168

0.000 0.000 0.005 0.000 0.000 0.011 0.000 0.000 0.000 0.011

0.000 0.005 0.011 0.011 0.011 0.000 0.000 0.005 0.000 0.000

0.068 0.066 0.069 0.060 0.000 0.000 0.014 0.000 0.000 0.007

0.000 0.014 0.007 0.007 0.000 0.007 0.000 0.007 0.000 0.014

0.000 0.000 0.000 0.007 0.063 0.063 0.061 0.065 0.000 0.000

0.000 0.000 0.000 0.000 0.000 0.000 0.006 0.000 0.000 0.000

0.006 0.000 0.012 0.018 0.024 0.000 0.006 0.000 0.078 0.070

0.036 0.078

>Non-Methylation code ID 3168

0.007 0.000 0.000 0.000 0.000 0.013 0.000 0.007 0.007 0.007

0.000 0.007 0.026 0.000 0.000 0.007 0.007 0.000 0.000 0.000

0.064 0.052 0.059 0.073 0.006 0.000 0.000 0.006 0.000 0.000

0.000 0.000 0.000 0.013 0.000 0.000 0.019 0.006 0.013 0.000

0.006 0.006 0.000 0.000 0.052 0.061 0.062 0.084 0.006 0.000

0.000 0.006 0.000 0.000 0.006 0.000 0.011 0.000 0.000 0.000

0.022 0.000 0.000 0.000 0.000 0.000 0.011 0.006 0.069 0.052

0.071 0.073

>Non-Methylation code ID 3168

0.007 0.000 0.000 0.007 0.007 0.013 0.007 0.000 0.000 0.007

0.000 0.013 0.007 0.000 0.000 0.000 0.007 0.007 0.000 0.000

0.070 0.054 0.054 0.077 0.008 0.000 0.000 0.000 0.000 0.000

0.000 0.000 0.000 0.008 0.017 0.008 0.000 0.017 0.000 0.008

0.008 0.008 0.017 0.008 0.051 0.061 0.065 0.047 0.000 0.010

0.000 0.000 0.000 0.010 0.005 0.010 0.005 0.000 0.000 0.000

0.005 0.005 0.000 0.005 0.000 0.000 0.005 0.000 0.052 0.073

0.072 0.077

>Non-Methylation code ID 3168

0.007 0.000 0.000 0.007 0.007 0.000 0.000 0.000 0.000 0.014

0.000 0.000 0.021 0.000 0.007 0.000 0.014 0.007 0.000 0.000

0.066 0.053 0.069 0.063 0.000 0.000 0.000 0.005 0.000 0.000

0.005 0.000 0.005 0.000 0.000 0.005 0.021 0.005 0.000 0.005

0.000 0.000 0.011 0.005 0.061 0.064 0.070 0.069 0.000 0.000

0.000 0.000 0.000 0.015 0.007 0.015 0.007 0.007 0.000 0.000

0.022 0.000 0.000 0.007 0.007 0.000 0.000 0.000 0.063 0.054

0.059 0.069

>Non-Methylation code ID 3168

0.012 0.000 0.000 0.000 0.000 0.000 0.000 0.006 0.012 0.006

0.000 0.006 0.018 0.000 0.000 0.012 0.006 0.000 0.000 0.000

0.062 0.061 0.068 0.065 0.005 0.000 0.000 0.000 0.000 0.000

0.000 0.000 0.000 0.005 0.000 0.005 0.016 0.011 0.016 0.000

0.005 0.000 0.000 0.000 0.068 0.054 0.049 0.097 0.005 0.005

0.005 0.005 0.000 0.000 0.005 0.005 0.014 0.000 0.000 0.000

0.014 0.000 0.000 0.000 0.000 0.000 0.000 0.000 0.071 0.067

0.064 0.074

>Non-Methylation code ID 3168

0.006 0.000 0.000 0.006 0.000 0.000 0.000 0.012 0.006 0.012

0.000 0.000 0.000 0.000 0.012 0.006 0.000 0.006 0.000 0.000

0.085 0.082 0.045 0.056 0.000 0.000 0.019 0.010 0.000 0.000

0.000 0.000 0.000 0.000 0.000 0.019 0.010 0.010 0.000 0.029

0.000 0.000 0.000 0.000 0.055 0.066 0.049 0.066 0.000 0.000

0.000 0.000 0.000 0.000 0.006 0.006 0.018 0.006 0.000 0.006

0.000 0.000 0.012 0.000 0.012 0.006 0.000 0.000 0.056 0.076

0.071 0.059

>Non-Methylation code ID 3168

0.000 0.006 0.006 0.000 0.000 0.006 0.013 0.006 0.000 0.006

0.000 0.000 0.006 0.000 0.000 0.019 0.013 0.000 0.000 0.000

0.063 0.066 0.060 0.061 0.000 0.000 0.000 0.000 0.000 0.010

0.000 0.030 0.000 0.020 0.000 0.000 0.020 0.010 0.000 0.020

0.000 0.020 0.000 0.000 0.054 0.044 0.047 0.060 0.008 0.000

0.000 0.000 0.008 0.000 0.008 0.000 0.000 0.016 0.000 0.000

0.008 0.000 0.008 0.016 0.000 0.000 0.000 0.008 0.061 0.070

0.061 0.063

>Non-Methylation code ID 3168

0.005 0.000 0.005 0.000 0.005 0.000 0.005 0.000 0.000 0.010

0.000 0.000 0.005 0.000 0.010 0.010 0.000 0.000 0.005 0.005

0.071 0.062 0.071 0.066 0.000 0.007 0.000 0.000 0.000 0.020

0.013 0.007 0.000 0.007 0.000 0.000 0.007 0.000 0.000 0.013

0.013 0.000 0.000 0.000 0.057 0.058 0.063 0.071 0.000 0.000

0.000 0.009 0.000 0.018 0.000 0.018 0.000 0.009 0.000 0.000

0.018 0.009 0.000 0.018 0.000 0.009 0.000 0.000 0.063 0.040

0.057 0.065

>Non-Methylation code ID 3168

0.000 0.000 0.000 0.008 0.000 0.015 0.000 0.015 0.000 0.008

0.000 0.000 0.015 0.008 0.000 0.008 0.008 0.008 0.008 0.000

0.070 0.042 0.066 0.058 0.004 0.000 0.004 0.000 0.004 0.004

0.000 0.000 0.000 0.009 0.000 0.000 0.004 0.000 0.013 0.009

0.000 0.000 0.004 0.000 0.077 0.065 0.059 0.077 0.008 0.008

0.000 0.000 0.000 0.025 0.016 0.000 0.000 0.008 0.000 0.000

0.008 0.000 0.000 0.008 0.008 0.000 0.000 0.000 0.058 0.055

0.058 0.072

>Non-Methylation code ID 3168

0.000 0.000 0.008 0.000 0.000 0.025 0.008 0.000 0.000 0.000

0.000 0.000 0.008 0.000 0.000 0.017 0.017 0.008 0.000 0.000

0.047 0.061 0.064 0.069 0.000 0.006 0.006 0.006 0.000 0.000

0.000 0.006 0.000 0.012 0.000 0.000 0.012 0.006 0.006 0.000

0.006 0.006 0.000 0.000 0.050 0.059 0.085 0.070 0.004 0.004

0.000 0.000 0.004 0.004 0.000 0.004 0.000 0.004 0.000 0.000

0.004 0.000 0.012 0.004 0.000 0.000 0.000 0.004 0.077 0.078

0.055 0.074

>Non-Methylation code ID 3168

0.000 0.000 0.008 0.000 0.000 0.000 0.000 0.000 0.008 0.008

0.000 0.016 0.000 0.008 0.008 0.016 0.008 0.000 0.000 0.000

0.076 0.063 0.063 0.053 0.000 0.005 0.005 0.000 0.000 0.000

0.005 0.005 0.016 0.000 0.000 0.005 0.005 0.000 0.011 0.000

0.011 0.000 0.000 0.000 0.074 0.070 0.080 0.040 0.007 0.000

0.007 0.000 0.000 0.000 0.000 0.013 0.007 0.013 0.000 0.000

0.000 0.007 0.007 0.000 0.007 0.007 0.000 0.000 0.069 0.074

0.041 0.075

>Non-Methylation code ID 3168

0.000 0.000 0.007 0.000 0.000 0.007 0.000 0.000 0.007 0.014

0.000 0.007 0.000 0.000 0.007 0.000 0.022 0.000 0.000 0.000

0.079 0.047 0.061 0.074 0.000 0.005 0.005 0.000 0.000 0.005

0.000 0.005 0.005 0.005 0.000 0.005 0.011 0.000 0.005 0.000

0.005 0.000 0.000 0.000 0.076 0.062 0.088 0.049 0.000 0.000

0.019 0.000 0.000 0.006 0.000 0.000 0.000 0.006 0.000 0.000

0.000 0.013 0.006 0.000 0.006 0.006 0.006 0.000 0.086 0.056

0.049 0.073

>Non-Methylation code ID 3169

0.009 0.009 0.000 0.000 0.000 0.009 0.000 0.009 0.000 0.009

0.000 0.000 0.019 0.000 0.000 0.028 0.009 0.009 0.000 0.000

0.058 0.056 0.058 0.049 0.009 0.005 0.000 0.005 0.000 0.000

0.005 0.000 0.000 0.014 0.000 0.000 0.005 0.000 0.009 0.009

0.000 0.000 0.000 0.000 0.074 0.073 0.067 0.059 0.006 0.000

0.000 0.006 0.006 0.006 0.013 0.006 0.000 0.013 0.000 0.000

0.000 0.000 0.000 0.013 0.000 0.000 0.006 0.000 0.072 0.051

0.080 0.053

>Non-Methylation code ID 3169

0.000 0.005 0.000 0.005 0.000 0.005 0.005 0.000 0.005 0.011

0.000 0.000 0.005 0.000 0.005 0.016 0.005 0.000 0.000 0.000

0.065 0.074 0.063 0.060 0.005 0.000 0.000 0.005 0.000 0.005

0.005 0.005 0.005 0.000 0.000 0.000 0.014 0.000 0.005 0.009

0.000 0.000 0.005 0.000 0.060 0.064 0.068 0.080 0.006 0.000

0.006 0.000 0.000 0.006 0.000 0.000 0.006 0.013 0.000 0.000

0.006 0.006 0.006 0.006 0.006 0.006 0.000 0.000 0.041 0.070

0.079 0.067

>Non-Methylation code ID 3169

0.005 0.000 0.000 0.000 0.005 0.010 0.005 0.005 0.005 0.000

0.000 0.000 0.010 0.000 0.005 0.010 0.000 0.000 0.005 0.000

0.065 0.057 0.075 0.071 0.007 0.000 0.007 0.000 0.000 0.007

0.000 0.000 0.000 0.014 0.000 0.000 0.014 0.000 0.007 0.014

0.007 0.014 0.000 0.000 0.049 0.067 0.072 0.053 0.000 0.006

0.000 0.006 0.000 0.006 0.006 0.000 0.000 0.018 0.000 0.000

0.000 0.000 0.006 0.018 0.006 0.000 0.000 0.000 0.070 0.067

0.064 0.061

>Non-Methylation code ID 3169

0.000 0.005 0.000 0.005 0.005 0.000 0.000 0.005 0.000 0.000

0.000 0.005 0.000 0.005 0.005 0.005 0.010 0.000 0.005 0.005

0.048 0.064 0.077 0.083 0.013 0.000 0.000 0.007 0.000 0.007

0.000 0.007 0.007 0.007 0.000 0.007 0.000 0.000 0.007 0.013

0.007 0.000 0.000 0.000 0.067 0.056 0.060 0.070 0.006 0.000

0.000 0.000 0.006 0.000 0.006 0.000 0.006 0.006 0.006 0.006

0.000 0.013 0.006 0.000 0.000 0.006 0.000 0.006 0.075 0.050

0.080 0.053

>Non-Methylation code ID 3169

0.000 0.005 0.010 0.000 0.010 0.000 0.000 0.005 0.000 0.000

0.000 0.005 0.000 0.000 0.005 0.015 0.005 0.000 0.000 0.000

0.066 0.062 0.067 0.077 0.029 0.000 0.000 0.010 0.000 0.000

0.000 0.010 0.000 0.020 0.000 0.000 0.000 0.000 0.000 0.020

0.020 0.000 0.010 0.000 0.048 0.055 0.064 0.048 0.000 0.006

0.000 0.000 0.013 0.006 0.006 0.000 0.006 0.013 0.000 0.006

0.000 0.019 0.000 0.000 0.000 0.000 0.000 0.000 0.066 0.066

0.062 0.064

>Non-Methylation code ID 3169

0.008 0.000 0.004 0.000 0.000 0.004 0.004 0.000 0.000 0.000

0.000 0.000 0.000 0.000 0.011 0.004 0.004 0.000 0.008 0.004

0.075 0.060 0.068 0.081 0.006 0.000 0.000 0.000 0.000 0.026

0.013 0.006 0.000 0.006 0.000 0.000 0.000 0.000 0.006 0.000

0.013 0.000 0.000 0.000 0.069 0.055 0.067 0.065 0.006 0.000

0.000 0.006 0.000 0.012 0.000 0.006 0.000 0.006 0.000 0.000

0.000 0.006 0.006 0.000 0.006 0.006 0.006 0.006 0.072 0.059

0.087 0.045

>Non-Methylation code ID 3169

0.008 0.000 0.004 0.000 0.000 0.004 0.004 0.000 0.000 0.008

0.000 0.000 0.000 0.000 0.012 0.008 0.000 0.000 0.004 0.000

0.076 0.057 0.063 0.086 0.008 0.008 0.000 0.000 0.000 0.023

0.015 0.000 0.000 0.008 0.000 0.000 0.008 0.000 0.000 0.008

0.015 0.000 0.000 0.000 0.060 0.055 0.064 0.061 0.000 0.000

0.000 0.007 0.000 0.014 0.000 0.007 0.000 0.007 0.000 0.000

0.007 0.007 0.007 0.007 0.007 0.007 0.007 0.000 0.066 0.059

0.069 0.059

>Non-Methylation code ID 3169

0.000 0.000 0.000 0.008 0.000 0.015 0.000 0.015 0.000 0.015

0.000 0.000 0.015 0.008 0.000 0.008 0.000 0.008 0.008 0.000

0.062 0.047 0.061 0.064 0.005 0.000 0.005 0.000 0.005 0.005

0.000 0.000 0.000 0.009 0.000 0.000 0.005 0.000 0.009 0.009

0.000 0.000 0.005 0.005 0.072 0.063 0.067 0.073 0.008 0.008

0.000 0.000 0.000 0.023 0.015 0.000 0.000 0.008 0.000 0.000

0.008 0.000 0.000 0.015 0.008 0.000 0.000 0.000 0.058 0.056

0.066 0.063

>Non-Methylation code ID 3169

0.056 0.000 0.000 0.000 0.000 0.011 0.000 0.000 0.000 0.011

0.000 0.000 0.034 0.000 0.000 0.034 0.000 0.000 0.000 0.000

0.047 0.044 0.037 0.059 0.022 0.000 0.000 0.000 0.000 0.000

0.007 0.000 0.000 0.022 0.000 0.000 0.036 0.000 0.007 0.000

0.000 0.000 0.000 0.000 0.065 0.056 0.059 0.060 0.000 0.009

0.000 0.000 0.000 0.000 0.000 0.000 0.000 0.000 0.000 0.000

0.013 0.009 0.013 0.000 0.004 0.000 0.004 0.000 0.056 0.067

0.088 0.070

>Non-Methylation code ID 3169

0.004 0.000 0.000 0.004 0.004 0.000 0.000 0.004 0.000 0.000

0.000 0.004 0.009 0.009 0.013 0.000 0.004 0.000 0.000 0.000

0.046 0.071 0.080 0.079 0.013 0.000 0.000 0.006 0.000 0.000

0.000 0.006 0.006 0.006 0.000 0.000 0.013 0.006 0.000 0.019

0.000 0.000 0.000 0.006 0.059 0.052 0.060 0.078 0.013 0.000

0.000 0.000 0.000 0.000 0.006 0.006 0.006 0.000 0.000 0.006

0.019 0.000 0.006 0.006 0.000 0.000 0.000 0.000 0.062 0.074

0.051 0.075

>Non-Methylation code ID 3169

0.014 0.000 0.000 0.000 0.000 0.005 0.000 0.000 0.000 0.010

0.000 0.000 0.010 0.005 0.010 0.005 0.000 0.000 0.005 0.000

0.062 0.061 0.075 0.074 0.006 0.006 0.006 0.000 0.000 0.006

0.000 0.000 0.000 0.006 0.000 0.000 0.032 0.000 0.006 0.006

0.000 0.006 0.000 0.000 0.062 0.070 0.054 0.065 0.006 0.006

0.000 0.000 0.006 0.006 0.000 0.000 0.000 0.006 0.000 0.000

0.023 0.006 0.006 0.000 0.006 0.000 0.000 0.000 0.070 0.088

0.073 0.034

>Non-Methylation code ID 3169

0.000 0.005 0.000 0.000 0.000 0.005 0.000 0.000 0.005 0.000

0.000 0.000 0.015 0.010 0.005 0.005 0.010 0.000 0.005 0.000

0.077 0.042 0.073 0.074 0.011 0.000 0.000 0.000 0.000 0.011

0.000 0.000 0.000 0.011 0.000 0.000 0.016 0.000 0.011 0.011

0.000 0.000 0.000 0.000 0.050 0.071 0.073 0.070 0.016 0.000

0.005 0.005 0.000 0.000 0.005 0.000 0.000 0.005 0.000 0.000

0.011 0.000 0.005 0.005 0.000 0.005 0.000 0.000 0.057 0.053

0.084 0.074

>Non-Methylation code ID 3169

0.005 0.000 0.000 0.000 0.005 0.005 0.000 0.005 0.000 0.000

0.000 0.005 0.018 0.005 0.009 0.000 0.005 0.000 0.000 0.000

0.072 0.066 0.073 0.063 0.000 0.000 0.000 0.005 0.000 0.011

0.000 0.000 0.000 0.011 0.000 0.000 0.011 0.005 0.011 0.011

0.005 0.000 0.000 0.000 0.056 0.057 0.073 0.078 0.006 0.006

0.006 0.000 0.000 0.000 0.006 0.000 0.006 0.000 0.000 0.000

0.022 0.000 0.006 0.006 0.000 0.006 0.000 0.000 0.057 0.071

0.073 0.067

>Non-Methylation code ID 3169

0.030 0.000 0.000 0.007 0.000 0.000 0.000 0.007 0.007 0.007

0.000 0.000 0.007 0.000 0.000 0.022 0.000 0.000 0.000 0.007

0.056 0.053 0.055 0.073 0.015 0.000 0.000 0.000 0.000 0.000

0.000 0.007 0.000 0.015 0.000 0.007 0.036 0.000 0.007 0.000

0.000 0.000 0.000 0.000 0.068 0.073 0.034 0.072 0.000 0.008

0.000 0.004 0.004 0.000 0.000 0.004 0.000 0.000 0.000 0.000

0.008 0.008 0.012 0.000 0.000 0.000 0.000 0.000 0.039 0.069

0.088 0.090

>Non-Methylation code ID 3169

0.005 0.005 0.000 0.015 0.000 0.005 0.000 0.000 0.000 0.005

0.000 0.000 0.005 0.000 0.010 0.000 0.005 0.000 0.000 0.000

0.070 0.072 0.064 0.073 0.000 0.000 0.005 0.010 0.000 0.010

0.000 0.000 0.005 0.000 0.000 0.000 0.010 0.005 0.010 0.005

0.000 0.000 0.000 0.000 0.048 0.069 0.082 0.076 0.009 0.000

0.000 0.000 0.000 0.018 0.000 0.000 0.009 0.018 0.000 0.009

0.000 0.009 0.009 0.000 0.009 0.000 0.000 0.000 0.046 0.069

0.063 0.068

>Non-Methylation code ID 3169

0.005 0.000 0.005 0.005 0.000 0.011 0.000 0.000 0.000 0.000

0.000 0.005 0.011 0.000 0.011 0.000 0.011 0.000 0.000 0.000

0.054 0.074 0.059 0.082 0.006 0.000 0.000 0.011 0.000 0.011

0.000 0.006 0.000 0.011 0.000 0.000 0.011 0.006 0.006 0.000

0.006 0.000 0.000 0.000 0.062 0.062 0.072 0.063 0.000 0.000

0.005 0.005 0.000 0.005 0.005 0.000 0.005 0.005 0.000 0.000

0.005 0.000 0.011 0.005 0.000 0.000 0.000 0.000 0.068 0.074

0.071 0.066

>Non-Methylation code ID 3169

0.000 0.008 0.000 0.000 0.000 0.004 0.000 0.000 0.004 0.000

0.000 0.000 0.012 0.004 0.012 0.000 0.004 0.000 0.004 0.000

0.064 0.059 0.083 0.074 0.041 0.000 0.000 0.000 0.000 0.017

0.000 0.000 0.000 0.008 0.000 0.000 0.025 0.000 0.008 0.008

0.000 0.000 0.000 0.000 0.054 0.057 0.052 0.063 0.014 0.000

0.000 0.007 0.000 0.000 0.007 0.000 0.000 0.021 0.000 0.000

0.027 0.000 0.007 0.000 0.000 0.000 0.000 0.000 0.067 0.057

0.067 0.059

>Non-Methylation code ID 3170

0.013 0.000 0.000 0.006 0.000 0.000 0.000 0.006 0.006 0.006

0.000 0.000 0.013 0.006 0.000 0.019 0.000 0.000 0.000 0.006

0.059 0.052 0.060 0.078 0.013 0.000 0.000 0.000 0.000 0.000

0.006 0.006 0.006 0.000 0.000 0.006 0.025 0.000 0.006 0.006

0.000 0.000 0.000 0.000 0.060 0.075 0.048 0.076 0.000 0.000

0.000 0.005 0.005 0.000 0.000 0.005 0.000 0.000 0.000 0.005

0.010 0.010 0.014 0.000 0.005 0.000 0.000 0.000 0.048 0.075

0.083 0.071

>Non-Methylation code ID 3170

0.000 0.000 0.000 0.005 0.000 0.011 0.000 0.000 0.000 0.011

0.000 0.000 0.011 0.005 0.011 0.011 0.005 0.000 0.000 0.000

0.056 0.057 0.073 0.078 0.005 0.005 0.005 0.000 0.000 0.005

0.005 0.000 0.005 0.000 0.000 0.000 0.020 0.000 0.005 0.005

0.000 0.005 0.000 0.000 0.054 0.070 0.072 0.074 0.005 0.000

0.000 0.000 0.005 0.005 0.000 0.005 0.000 0.000 0.000 0.005

0.014 0.005 0.009 0.000 0.005 0.000 0.000 0.000 0.070 0.065

0.077 0.064

>Non-Methylation code ID 3170

0.011 0.000 0.000 0.005 0.011 0.005 0.000 0.000 0.000 0.000

0.000 0.000 0.005 0.000 0.011 0.005 0.005 0.000 0.000 0.000

0.079 0.059 0.057 0.078 0.000 0.000 0.000 0.008 0.004 0.008

0.004 0.000 0.004 0.000 0.000 0.000 0.000 0.000 0.016 0.004

0.000 0.004 0.000 0.000 0.060 0.077 0.081 0.063 0.000 0.000

0.005 0.010 0.000 0.005 0.000 0.005 0.000 0.005 0.000 0.000

0.000 0.000 0.010 0.010 0.000 0.010 0.000 0.000 0.062 0.074

0.068 0.066

>Non-Methylation code ID 3170

0.009 0.004 0.004 0.000 0.000 0.004 0.004 0.000 0.000 0.000

0.000 0.000 0.000 0.000 0.017 0.000 0.004 0.004 0.000 0.000

0.075 0.108 0.046 0.053 0.007 0.000 0.000 0.015 0.000 0.022

0.007 0.015 0.000 0.000 0.000 0.000 0.015 0.000 0.000 0.007

0.000 0.000 0.000 0.000 0.052 0.064 0.063 0.066 0.007 0.007

0.000 0.000 0.000 0.014 0.007 0.000 0.007 0.007 0.007 0.000

0.007 0.014 0.000 0.007 0.000 0.000 0.000 0.000 0.052 0.069

0.063 0.064

>Non-Methylation code ID 3170

0.016 0.000 0.000 0.000 0.000 0.000 0.016 0.000 0.000 0.008

0.000 0.000 0.024 0.000 0.000 0.016 0.024 0.000 0.000 0.000

0.049 0.045 0.063 0.075 0.008 0.000 0.000 0.000 0.000 0.000

0.008 0.000 0.000 0.008 0.008 0.000 0.054 0.000 0.000 0.008

0.008 0.000 0.000 0.000 0.065 0.053 0.060 0.056 0.000 0.018

0.000 0.000 0.000 0.000 0.018 0.009 0.000 0.000 0.000 0.000

0.055 0.009 0.000 0.000 0.000 0.000 0.000 0.000 0.050 0.050

0.059 0.063

>Non-Methylation code ID 3170

0.000 0.009 0.000 0.000 0.000 0.000 0.000 0.000 0.000 0.017

0.009 0.000 0.061 0.000 0.000 0.009 0.009 0.000 0.000 0.000

0.067 0.053 0.049 0.051 0.000 0.018 0.000 0.000 0.000 0.000

0.018 0.000 0.000 0.000 0.000 0.000 0.061 0.000 0.000 0.009

0.000 0.009 0.000 0.000 0.059 0.034 0.071 0.056 0.015 0.000

0.000 0.000 0.000 0.000 0.015 0.000 0.000 0.008 0.000 0.000

0.031 0.000 0.000 0.008 0.015 0.000 0.000 0.000 0.054 0.048

0.062 0.078

>Non-Methylation code ID 3170

0.015 0.007 0.000 0.000 0.007 0.007 0.000 0.015 0.000 0.015

0.000 0.000 0.000 0.007 0.000 0.007 0.015 0.000 0.000 0.000

0.044 0.067 0.070 0.055 0.012 0.006 0.000 0.000 0.006 0.000

0.000 0.000 0.000 0.000 0.000 0.000 0.006 0.017 0.006 0.012

0.000 0.000 0.000 0.000 0.069 0.060 0.071 0.070 0.000 0.000

0.000 0.006 0.000 0.000 0.006 0.000 0.000 0.006 0.000 0.019

0.013 0.000 0.000 0.013 0.000 0.006 0.006 0.000 0.060 0.068

0.064 0.067

>Non-Methylation code ID 3170

0.019 0.006 0.006 0.013 0.006 0.006 0.000 0.000 0.000 0.000

0.000 0.000 0.013 0.000 0.000 0.006 0.000 0.006 0.000 0.000

0.065 0.063 0.063 0.059 0.006 0.000 0.000 0.006 0.000 0.000

0.000 0.000 0.006 0.024 0.000 0.000 0.006 0.006 0.006 0.012

0.006 0.000 0.000 0.000 0.062 0.084 0.069 0.039 0.000 0.000

0.000 0.000 0.005 0.005 0.000 0.000 0.000 0.010 0.000 0.000

0.005 0.000 0.015 0.010 0.000 0.000 0.005 0.000 0.066 0.074

0.082 0.058

>Non-Methylation code ID 3170

0.000 0.000 0.000 0.006 0.000 0.012 0.006 0.006 0.006 0.006

0.000 0.012 0.006 0.000 0.000 0.006 0.000 0.000 0.006 0.000

0.060 0.063 0.069 0.071 0.017 0.006 0.000 0.006 0.000 0.006

0.000 0.006 0.000 0.006 0.000 0.000 0.000 0.000 0.011 0.006

0.011 0.000 0.000 0.000 0.067 0.076 0.076 0.043 0.007 0.000

0.000 0.000 0.000 0.015 0.007 0.000 0.000 0.007 0.000 0.000

0.007 0.022 0.007 0.007 0.000 0.000 0.000 0.000 0.079 0.048

0.069 0.057

>Non-Methylation code ID 3170

0.006 0.006 0.000 0.000 0.000 0.006 0.006 0.000 0.006 0.011

0.006 0.000 0.006 0.011 0.006 0.006 0.000 0.000 0.000 0.000

0.074 0.072 0.066 0.049 0.010 0.005 0.000 0.000 0.000 0.005

0.005 0.000 0.000 0.000 0.000 0.000 0.000 0.000 0.019 0.000

0.005 0.005 0.000 0.000 0.074 0.111 0.045 0.051 0.007 0.000

0.000 0.014 0.000 0.014 0.007 0.007 0.007 0.000 0.000 0.000

0.014 0.000 0.000 0.007 0.000 0.000 0.000 0.000 0.054 0.075

0.058 0.067

>Non-Methylation code ID 3170

0.000 0.006 0.000 0.006 0.006 0.012 0.000 0.006 0.006 0.006

0.000 0.006 0.012 0.000 0.000 0.006 0.000 0.000 0.000 0.000

0.077 0.054 0.076 0.056 0.021 0.000 0.000 0.005 0.010 0.000

0.000 0.005 0.000 0.010 0.000 0.000 0.000 0.000 0.010 0.005

0.000 0.000 0.000 0.000 0.065 0.067 0.085 0.049 0.000 0.000

0.000 0.000 0.007 0.013 0.007 0.000 0.000 0.007 0.000 0.000

0.007 0.013 0.007 0.013 0.000 0.000 0.000 0.000 0.080 0.058

0.078 0.044

>Non-Methylation code ID 3170

0.008 0.000 0.000 0.000 0.025 0.017 0.008 0.000 0.000 0.008

0.008 0.000 0.000 0.008 0.000 0.000 0.000 0.000 0.000 0.008

0.056 0.045 0.062 0.078 0.006 0.011 0.000 0.000 0.000 0.000

0.000 0.000 0.000 0.006 0.011 0.000 0.000 0.000 0.006 0.022

0.000 0.006 0.006 0.000 0.071 0.047 0.067 0.075 0.008 0.015

0.000 0.000 0.000 0.015 0.000 0.000 0.000 0.023 0.000 0.000

0.015 0.000 0.000 0.000 0.000 0.008 0.000 0.008 0.081 0.055

0.069 0.036

>Non-Methylation code ID 3170

0.000 0.000 0.000 0.000 0.000 0.000 0.000 0.000 0.000 0.010

0.000 0.000 0.005 0.000 0.019 0.005 0.010 0.000 0.005 0.000

0.072 0.070 0.081 0.058 0.013 0.000 0.013 0.013 0.000 0.013

0.006 0.000 0.000 0.006 0.000 0.000 0.006 0.000 0.000 0.013

0.000 0.000 0.000 0.000 0.063 0.072 0.046 0.072 0.000 0.000

0.000 0.013 0.000 0.000 0.006 0.000 0.006 0.013 0.006 0.006

0.013 0.000 0.000 0.000 0.013 0.000 0.000 0.000 0.065 0.078

0.065 0.047

>Non-Methylation code ID 3170

0.005 0.000 0.000 0.005 0.005 0.005 0.005 0.005 0.000 0.000

0.000 0.000 0.015 0.000 0.010 0.000 0.010 0.000 0.000 0.000

0.071 0.067 0.049 0.082 0.005 0.000 0.000 0.005 0.005 0.005

0.005 0.000 0.000 0.005 0.000 0.000 0.020 0.000 0.005 0.005

0.005 0.000 0.000 0.000 0.057 0.067 0.078 0.066 0.000 0.000

0.007 0.007 0.007 0.000 0.014 0.000 0.000 0.000 0.000 0.000

0.022 0.007 0.000 0.007 0.007 0.000 0.000 0.000 0.072 0.055

0.063 0.063

>Non-Methylation code ID 3170

0.006 0.000 0.011 0.006 0.006 0.006 0.006 0.000 0.000 0.011

0.000 0.000 0.006 0.000 0.006 0.006 0.000 0.000 0.000 0.000

0.074 0.083 0.049 0.060 0.000 0.000 0.000 0.005 0.000 0.005

0.005 0.000 0.000 0.005 0.005 0.005 0.011 0.000 0.005 0.005

0.011 0.000 0.005 0.000 0.064 0.065 0.064 0.071 0.000 0.006

0.000 0.000 0.000 0.006 0.000 0.000 0.000 0.006 0.000 0.000

0.011 0.006 0.011 0.000 0.011 0.006 0.000 0.000 0.062 0.082

0.065 0.064

>Non-Methylation code ID 3171

0.000 0.005 0.000 0.000 0.005 0.000 0.000 0.000 0.000 0.015

0.000 0.000 0.010 0.000 0.005 0.005 0.000 0.005 0.010 0.005

0.074 0.067 0.059 0.066 0.008 0.000 0.000 0.008 0.017 0.017

0.000 0.008 0.000 0.008 0.000 0.000 0.008 0.000 0.000 0.025

0.000 0.000 0.000 0.000 0.065 0.069 0.037 0.063 0.019 0.000

0.000 0.000 0.010 0.000 0.000 0.000 0.000 0.039 0.000 0.000

0.019 0.000 0.000 0.029 0.000 0.000 0.000 0.000 0.057 0.047

0.055 0.058

>Non-Methylation code ID 3171

0.000 0.005 0.000 0.000 0.005 0.000 0.005 0.010 0.000 0.000

0.000 0.005 0.016 0.005 0.005 0.000 0.000 0.010 0.000 0.000

0.074 0.063 0.060 0.070 0.008 0.000 0.000 0.000 0.016 0.000

0.008 0.008 0.008 0.000 0.000 0.000 0.008 0.008 0.000 0.024

0.008 0.000 0.000 0.000 0.074 0.041 0.056 0.068 0.000 0.009

0.000 0.000 0.000 0.000 0.009 0.000 0.000 0.009 0.000 0.009

0.026 0.009 0.000 0.035 0.000 0.000 0.000 0.000 0.043 0.051

0.066 0.070

>Non-Methylation code ID 3171

0.000 0.000 0.000 0.000 0.007 0.015 0.000 0.007 0.000 0.015

0.000 0.000 0.007 0.007 0.007 0.007 0.000 0.007 0.000 0.000

0.069 0.077 0.049 0.059 0.005 0.000 0.005 0.016 0.005 0.000

0.000 0.000 0.000 0.000 0.000 0.000 0.000 0.000 0.011 0.011

0.000 0.000 0.000 0.011 0.070 0.063 0.065 0.070 0.004 0.000

0.004 0.000 0.000 0.004 0.000 0.004 0.009 0.009 0.000 0.000

0.004 0.000 0.009 0.000 0.004 0.000 0.000 0.000 0.058 0.077

0.086 0.059

>Non-Methylation code ID 3171

0.000 0.000 0.000 0.000 0.007 0.014 0.000 0.007 0.000 0.014

0.014 0.000 0.007 0.000 0.007 0.000 0.007 0.007 0.000 0.000

0.057 0.064 0.066 0.064 0.000 0.007 0.007 0.000 0.007 0.007

0.000 0.000 0.000 0.007 0.000 0.000 0.007 0.007 0.000 0.013

0.000 0.007 0.007 0.000 0.075 0.071 0.070 0.043 0.000 0.005

0.005 0.005 0.000 0.005 0.005 0.000 0.000 0.000 0.000 0.000

0.005 0.000 0.009 0.005 0.005 0.005 0.000 0.005 0.066 0.064

0.070 0.077

>Non-Methylation code ID 3171

0.007 0.007 0.000 0.007 0.000 0.000 0.007 0.000 0.000 0.022

0.007 0.000 0.029 0.000 0.000 0.000 0.007 0.000 0.000 0.000

0.065 0.057 0.055 0.062 0.000 0.013 0.000 0.000 0.000 0.000

0.013 0.006 0.000 0.000 0.000 0.006 0.025 0.000 0.000 0.006

0.000 0.006 0.006 0.000 0.068 0.049 0.069 0.065 0.009 0.000

0.000 0.000 0.000 0.009 0.017 0.000 0.000 0.009 0.000 0.000

0.017 0.000 0.000 0.017 0.026 0.000 0.000 0.000 0.051 0.050

0.065 0.063

>Non-Methylation code ID 3171

0.004 0.000 0.000 0.000 0.004 0.009 0.000 0.000 0.000 0.000

0.000 0.000 0.004 0.004 0.018 0.004 0.004 0.000 0.000 0.000

0.075 0.064 0.073 0.070 0.011 0.000 0.006 0.011 0.000 0.011

0.000 0.000 0.000 0.000 0.000 0.000 0.006 0.017 0.006 0.006

0.000 0.000 0.000 0.000 0.059 0.060 0.064 0.077 0.000 0.000

0.000 0.005 0.000 0.005 0.005 0.000 0.016 0.000 0.005 0.000

0.010 0.000 0.005 0.005 0.000 0.005 0.000 0.000 0.071 0.076

0.053 0.071

>Non-Methylation code ID 3171

0.005 0.000 0.000 0.000 0.000 0.009 0.000 0.000 0.000 0.005

0.000 0.000 0.005 0.005 0.014 0.005 0.005 0.000 0.000 0.005

0.056 0.069 0.075 0.078 0.011 0.000 0.006 0.011 0.006 0.006

0.000 0.000 0.000 0.000 0.000 0.000 0.006 0.017 0.006 0.000

0.006 0.000 0.000 0.000 0.069 0.063 0.063 0.065 0.000 0.000

0.000 0.005 0.005 0.005 0.005 0.000 0.010 0.000 0.005 0.000

0.015 0.000 0.005 0.005 0.000 0.000 0.000 0.000 0.071 0.073

0.064 0.066

>Non-Methylation code ID 3171

0.000 0.000 0.000 0.000 0.000 0.006 0.006 0.006 0.000 0.006

0.000 0.000 0.024 0.000 0.006 0.012 0.012 0.000 0.000 0.000

0.055 0.068 0.071 0.061 0.006 0.006 0.000 0.006 0.006 0.000

0.006 0.000 0.000 0.013 0.000 0.000 0.025 0.006 0.000 0.000

0.006 0.000 0.000 0.000 0.065 0.065 0.063 0.059 0.000 0.000

0.000 0.000 0.000 0.000 0.012 0.006 0.006 0.000 0.000 0.006

0.018 0.000 0.000 0.012 0.000 0.006 0.006 0.000 0.060 0.059

0.061 0.082

>Non-Methylation code ID 3171

0.010 0.000 0.000 0.005 0.000 0.005 0.000 0.000 0.000 0.005

0.000 0.000 0.010 0.000 0.010 0.005 0.005 0.000 0.000 0.010

0.065 0.076 0.051 0.078 0.006 0.000 0.000 0.006 0.000 0.006

0.012 0.012 0.000 0.018 0.000 0.000 0.012 0.000 0.006 0.000

0.000 0.000 0.000 0.000 0.057 0.061 0.072 0.066 0.000 0.000

0.008 0.008 0.000 0.000 0.000 0.016 0.000 0.008 0.000 0.000

0.008 0.008 0.000 0.024 0.000 0.000 0.008 0.000 0.051 0.058

0.051 0.086

>Non-Methylation code ID 3171

0.000 0.005 0.000 0.005 0.005 0.000 0.005 0.005 0.000 0.000

0.000 0.005 0.014 0.005 0.005 0.000 0.000 0.009 0.000 0.000

0.071 0.066 0.058 0.076 0.009 0.000 0.000 0.000 0.000 0.000

0.009 0.009 0.009 0.000 0.000 0.009 0.009 0.009 0.000 0.036

0.009 0.000 0.000 0.000 0.056 0.046 0.060 0.064 0.000 0.007

0.000 0.000 0.000 0.000 0.007 0.007 0.000 0.007 0.000 0.000

0.026 0.007 0.007 0.013 0.000 0.000 0.000 0.000 0.050 0.060

0.064 0.080

>Non-Methylation code ID 3171

0.008 0.000 0.000 0.000 0.000 0.024 0.008 0.000 0.008 0.008

0.008 0.000 0.008 0.016 0.000 0.008 0.000 0.000 0.000 0.000

0.057 0.066 0.050 0.062 0.009 0.005 0.005 0.000 0.000 0.000

0.005 0.000 0.000 0.000 0.000 0.000 0.000 0.000 0.023 0.000

0.005 0.000 0.000 0.000 0.081 0.092 0.033 0.078 0.008 0.000

0.000 0.015 0.000 0.031 0.008 0.015 0.000 0.000 0.000 0.000

0.015 0.000 0.000 0.000 0.000 0.000 0.000 0.000 0.055 0.063

0.061 0.062

>Non-Methylation code ID 3171

0.006 0.000 0.000 0.000 0.000 0.006 0.000 0.000 0.030 0.012

0.000 0.000 0.006 0.000 0.006 0.006 0.000 0.000 0.000 0.000

0.058 0.059 0.089 0.055 0.000 0.000 0.000 0.005 0.005 0.000

0.000 0.000 0.010 0.010 0.000 0.005 0.005 0.000 0.010 0.010

0.000 0.005 0.000 0.000 0.073 0.062 0.082 0.051 0.007 0.007

0.000 0.007 0.000 0.007 0.000 0.000 0.021 0.014 0.000 0.000

0.007 0.000 0.000 0.007 0.000 0.000 0.000 0.000 0.053 0.065

0.057 0.082

>Non-Methylation code ID 3171

0.008 0.008 0.000 0.000 0.000 0.000 0.008 0.008 0.000 0.017

0.000 0.000 0.042 0.008 0.000 0.008 0.000 0.000 0.000 0.000

0.047 0.055 0.064 0.058 0.008 0.000 0.000 0.000 0.000 0.000

0.000 0.000 0.000 0.024 0.000 0.000 0.032 0.000 0.000 0.016

0.008 0.000 0.008 0.008 0.062 0.049 0.052 0.068 0.009 0.000

0.000 0.000 0.000 0.009 0.009 0.009 0.000 0.000 0.000 0.000

0.051 0.000 0.000 0.017 0.000 0.000 0.000 0.000 0.062 0.056

0.063 0.050

>Non-Methylation code ID 3171

0.006 0.006 0.000 0.000 0.000 0.013 0.000 0.006 0.000 0.013

0.000 0.000 0.006 0.013 0.006 0.006 0.000 0.000 0.000 0.000

0.080 0.052 0.069 0.057 0.010 0.005 0.000 0.000 0.000 0.010

0.000 0.000 0.010 0.016 0.000 0.000 0.005 0.000 0.005 0.005

0.000 0.000 0.000 0.000 0.087 0.059 0.083 0.037 0.015 0.000

0.007 0.000 0.000 0.000 0.000 0.000 0.000 0.015 0.000 0.000

0.015 0.000 0.007 0.015 0.000 0.007 0.000 0.000 0.075 0.070

0.052 0.055

>Non-Methylation code ID 3173

0.000 0.000 0.007 0.007 0.007 0.000 0.013 0.000 0.000 0.000

0.000 0.000 0.026 0.007 0.000 0.007 0.007 0.000 0.000 0.000

0.064 0.063 0.065 0.062 0.005 0.000 0.000 0.005 0.005 0.000

0.005 0.005 0.000 0.000 0.000 0.000 0.021 0.000 0.010 0.000

0.010 0.000 0.000 0.000 0.068 0.079 0.050 0.069 0.000 0.000

0.000 0.005 0.005 0.005 0.005 0.000 0.000 0.005 0.000 0.000

0.022 0.000 0.005 0.005 0.005 0.000 0.000 0.000 0.061 0.072

0.068 0.066

>Non-Methylation code ID 3173

0.012 0.000 0.006 0.000 0.000 0.000 0.000 0.000 0.000 0.012

0.000 0.000 0.012 0.000 0.012 0.012 0.000 0.006 0.000 0.000

0.074 0.068 0.059 0.063 0.006 0.006 0.000 0.000 0.000 0.013

0.000 0.006 0.000 0.013 0.000 0.000 0.006 0.013 0.006 0.006

0.000 0.000 0.000 0.000 0.080 0.052 0.069 0.057 0.011 0.005

0.000 0.000 0.000 0.011 0.000 0.000 0.011 0.011 0.000 0.000

0.005 0.000 0.005 0.005 0.000 0.000 0.000 0.000 0.092 0.061

0.078 0.038

>Non-Methylation code ID 3173

0.000 0.000 0.000 0.005 0.005 0.005 0.005 0.000 0.000 0.005

0.000 0.000 0.019 0.000 0.009 0.005 0.005 0.000 0.000 0.000

0.060 0.070 0.073 0.070 0.000 0.000 0.006 0.006 0.006 0.006

0.012 0.000 0.000 0.000 0.000 0.000 0.025 0.000 0.000 0.006

0.006 0.000 0.000 0.000 0.063 0.062 0.070 0.064 0.006 0.000

0.000 0.006 0.006 0.000 0.006 0.006 0.000 0.000 0.000 0.000

0.023 0.000 0.006 0.000 0.012 0.000 0.000 0.000 0.067 0.079

0.057 0.060

>Non-Methylation code ID 3173

0.000 0.005 0.000 0.000 0.005 0.000 0.005 0.010 0.000 0.000

0.000 0.005 0.015 0.005 0.005 0.000 0.000 0.010 0.000 0.000

0.075 0.060 0.062 0.072 0.009 0.000 0.000 0.000 0.009 0.000

0.009 0.009 0.009 0.000 0.000 0.000 0.009 0.009 0.000 0.034

0.009 0.000 0.000 0.000 0.059 0.047 0.060 0.065 0.000 0.009

0.000 0.000 0.000 0.000 0.009 0.009 0.000 0.009 0.000 0.000

0.034 0.009 0.000 0.026 0.000 0.000 0.000 0.000 0.044 0.056

0.065 0.065

>Non-Methylation code ID 3173

0.000 0.005 0.005 0.000 0.000 0.000 0.000 0.000 0.000 0.016

0.000 0.000 0.010 0.000 0.010 0.000 0.005 0.005 0.000 0.000

0.071 0.075 0.070 0.060 0.000 0.000 0.000 0.008 0.000 0.008

0.008 0.008 0.000 0.016 0.000 0.000 0.000 0.008 0.008 0.008

0.000 0.008 0.000 0.000 0.073 0.049 0.052 0.081 0.005 0.005

0.000 0.011 0.005 0.000 0.000 0.000 0.000 0.005 0.000 0.005

0.005 0.000 0.005 0.011 0.005 0.000 0.000 0.000 0.071 0.058

0.070 0.070

>Non-Methylation code ID 3173

0.000 0.000 0.006 0.006 0.006 0.006 0.000 0.000 0.000 0.006

0.000 0.000 0.012 0.006 0.006 0.012 0.000 0.006 0.006 0.000

0.056 0.074 0.065 0.061 0.000 0.005 0.000 0.005 0.000 0.010

0.000 0.005 0.000 0.010 0.000 0.005 0.005 0.000 0.005 0.010

0.000 0.005 0.000 0.000 0.057 0.064 0.071 0.074 0.005 0.000

0.000 0.005 0.005 0.005 0.000 0.000 0.000 0.005 0.000 0.000

0.005 0.000 0.005 0.011 0.005 0.005 0.005 0.000 0.053 0.062

0.077 0.076

>Non-Methylation code ID 3173

0.000 0.000 0.007 0.000 0.000 0.013 0.000 0.013 0.000 0.007

0.000 0.000 0.000 0.007 0.007 0.013 0.007 0.000 0.007 0.000

0.071 0.066 0.041 0.076 0.005 0.000 0.000 0.005 0.000 0.011

0.000 0.000 0.000 0.005 0.000 0.000 0.005 0.005 0.011 0.005

0.005 0.000 0.000 0.011 0.064 0.065 0.066 0.069 0.011 0.000

0.006 0.000 0.000 0.006 0.011 0.011 0.000 0.011 0.000 0.000

0.000 0.000 0.011 0.000 0.000 0.000 0.000 0.000 0.072 0.047

0.057 0.088

>Non-Methylation code ID 3173

0.017 0.000 0.000 0.000 0.006 0.000 0.006 0.000 0.000 0.006

0.000 0.000 0.006 0.000 0.006 0.000 0.006 0.000 0.000 0.011

0.065 0.086 0.059 0.061 0.000 0.000 0.007 0.007 0.000 0.000

0.000 0.007 0.000 0.015 0.007 0.000 0.022 0.000 0.000 0.007

0.007 0.000 0.007 0.000 0.063 0.065 0.057 0.061 0.000 0.015

0.000 0.000 0.008 0.008 0.008 0.000 0.000 0.023 0.000 0.000

0.008 0.008 0.000 0.008 0.008 0.000 0.000 0.000 0.063 0.050

0.056 0.073

>Non-Methylation code ID 3173

0.006 0.006 0.000 0.013 0.006 0.000 0.000 0.000 0.013 0.000

0.000 0.006 0.006 0.000 0.000 0.013 0.006 0.000 0.000 0.000

0.063 0.077 0.057 0.059 0.000 0.005 0.000 0.000 0.000 0.000

0.000 0.000 0.005 0.014 0.000 0.000 0.009 0.000 0.014 0.000

0.005 0.005 0.000 0.000 0.077 0.084 0.062 0.055 0.000 0.000

0.000 0.009 0.000 0.018 0.009 0.000 0.009 0.018 0.000 0.000

0.000 0.000 0.000 0.009 0.000 0.009 0.000 0.000 0.062 0.065

0.062 0.064

>Non-Methylation code ID 3173

0.005 0.000 0.000 0.005 0.005 0.005 0.000 0.005 0.000 0.005

0.000 0.000 0.015 0.000 0.010 0.000 0.010 0.000 0.000 0.000

0.066 0.075 0.044 0.083 0.005 0.000 0.000 0.005 0.005 0.005

0.005 0.000 0.000 0.010 0.000 0.000 0.020 0.000 0.005 0.005

0.000 0.000 0.000 0.000 0.057 0.064 0.071 0.078 0.000 0.000

0.007 0.007 0.007 0.000 0.013 0.000 0.000 0.000 0.000 0.000

0.013 0.007 0.000 0.007 0.007 0.000 0.007 0.000 0.061 0.060

0.066 0.072

>Non-Methylation code ID 3173

0.011 0.000 0.006 0.011 0.000 0.011 0.000 0.000 0.000 0.000

0.000 0.000 0.006 0.017 0.006 0.006 0.000 0.000 0.000 0.000

0.059 0.060 0.064 0.077 0.000 0.000 0.000 0.005 0.000 0.005

0.005 0.000 0.015 0.005 0.005 0.000 0.010 0.000 0.005 0.005

0.000 0.005 0.000 0.000 0.068 0.081 0.052 0.067 0.005 0.000

0.000 0.000 0.005 0.009 0.000 0.000 0.000 0.000 0.000 0.000

0.005 0.005 0.019 0.000 0.005 0.000 0.000 0.000 0.076 0.060

0.069 0.076

>Non-Methylation code ID 3173

0.000 0.000 0.000 0.000 0.000 0.021 0.000 0.014 0.000 0.007

0.000 0.000 0.000 0.000 0.007 0.014 0.014 0.000 0.007 0.000

0.065 0.054 0.046 0.085 0.006 0.000 0.000 0.006 0.000 0.017

0.000 0.000 0.000 0.006 0.000 0.000 0.011 0.006 0.006 0.006

0.000 0.000 0.000 0.011 0.061 0.073 0.064 0.061 0.005 0.000

0.010 0.000 0.000 0.000 0.010 0.010 0.000 0.015 0.000 0.000

0.000 0.000 0.010 0.000 0.000 0.000 0.000 0.000 0.076 0.051

0.058 0.089

>Non-Methylation code ID 3173

0.000 0.007 0.007 0.000 0.007 0.007 0.000 0.007 0.000 0.007

0.000 0.000 0.000 0.007 0.000 0.014 0.000 0.007 0.007 0.000

0.073 0.070 0.073 0.042 0.000 0.004 0.004 0.004 0.004 0.004

0.004 0.000 0.004 0.000 0.000 0.000 0.004 0.000 0.004 0.004

0.004 0.004 0.000 0.004 0.077 0.066 0.065 0.067 0.000 0.000

0.000 0.000 0.007 0.007 0.000 0.007 0.000 0.014 0.014 0.007

0.007 0.000 0.007 0.000 0.000 0.007 0.000 0.000 0.059 0.068

0.063 0.067

>Non-Methylation code ID 3173

0.000 0.004 0.000 0.000 0.004 0.004 0.000 0.000 0.000 0.013

0.000 0.004 0.004 0.000 0.009 0.009 0.000 0.000 0.004 0.000

0.074 0.084 0.065 0.054 0.013 0.006 0.000 0.013 0.006 0.006

0.000 0.006 0.000 0.000 0.000 0.000 0.013 0.000 0.000 0.013

0.000 0.006 0.000 0.000 0.061 0.054 0.075 0.062 0.008 0.000

0.000 0.008 0.000 0.000 0.000 0.000 0.008 0.030 0.000 0.000

0.000 0.008 0.008 0.023 0.000 0.000 0.000 0.000 0.058 0.083

0.063 0.038

>Non-Methylation code ID 3173

0.005 0.000 0.005 0.016 0.005 0.005 0.000 0.000 0.000 0.000

0.000 0.000 0.000 0.000 0.011 0.011 0.000 0.000 0.000 0.005

0.064 0.074 0.065 0.067 0.005 0.000 0.005 0.000 0.000 0.005

0.000 0.000 0.009 0.009 0.005 0.000 0.005 0.000 0.009 0.000

0.005 0.000 0.000 0.000 0.056 0.081 0.080 0.062 0.000 0.000

0.000 0.000 0.006 0.012 0.000 0.006 0.000 0.006 0.000 0.000

0.006 0.006 0.006 0.006 0.000 0.006 0.006 0.000 0.064 0.076

0.060 0.065

>Non-Methylation code ID 3175

0.017 0.000 0.000 0.000 0.008 0.000 0.008 0.008 0.000 0.025

0.000 0.000 0.017 0.008 0.000 0.017 0.000 0.000 0.000 0.000

0.060 0.064 0.063 0.036 0.000 0.000 0.000 0.000 0.007 0.000

0.000 0.000 0.000 0.021 0.000 0.000 0.021 0.000 0.000 0.014

0.007 0.000 0.014 0.007 0.062 0.053 0.055 0.074 0.008 0.000

0.000 0.000 0.008 0.017 0.008 0.008 0.000 0.008 0.000 0.000

0.025 0.000 0.000 0.017 0.000 0.000 0.000 0.000 0.058 0.073

0.055 0.049

>Non-Methylation code ID 3175

0.000 0.000 0.000 0.005 0.005 0.015 0.000 0.000 0.015 0.000

0.000 0.005 0.000 0.005 0.015 0.000 0.000 0.000 0.000 0.000

0.090 0.039 0.091 0.047 0.010 0.000 0.000 0.005 0.000 0.010

0.000 0.000 0.014 0.005 0.005 0.000 0.000 0.000 0.010 0.000

0.000 0.005 0.000 0.000 0.072 0.082 0.039 0.078 0.007 0.000

0.000 0.007 0.000 0.013 0.000 0.000 0.020 0.000 0.000 0.000

0.007 0.007 0.007 0.000 0.000 0.000 0.007 0.000 0.051 0.052

0.076 0.083

>Non-Methylation code ID 3175

0.004 0.004 0.000 0.000 0.004 0.000 0.000 0.000 0.000 0.008

0.000 0.000 0.008 0.000 0.008 0.000 0.000 0.004 0.008 0.004

0.069 0.068 0.073 0.070 0.000 0.000 0.007 0.007 0.014 0.014

0.000 0.007 0.000 0.007 0.000 0.000 0.014 0.000 0.000 0.014

0.000 0.000 0.000 0.000 0.073 0.064 0.060 0.053 0.019 0.009

0.000 0.000 0.009 0.000 0.000 0.000 0.000 0.037 0.000 0.000

0.009 0.000 0.000 0.019 0.000 0.000 0.000 0.000 0.068 0.050

0.038 0.074

>Non-Methylation code ID 3175

0.005 0.005 0.000 0.010 0.000 0.000 0.000 0.000 0.000 0.010

0.000 0.005 0.005 0.000 0.010 0.000 0.005 0.005 0.000 0.000

0.075 0.064 0.080 0.054 0.000 0.000 0.000 0.000 0.000 0.012

0.006 0.000 0.006 0.006 0.000 0.000 0.006 0.000 0.012 0.006

0.006 0.006 0.000 0.000 0.070 0.081 0.063 0.052 0.000 0.000

0.000 0.000 0.000 0.013 0.006 0.006 0.013 0.006 0.000 0.000

0.000 0.000 0.006 0.006 0.000 0.006 0.000 0.000 0.068 0.079

0.071 0.051

>Non-Methylation code ID 3175

0.010 0.005 0.000 0.000 0.000 0.010 0.000 0.000 0.010 0.016

0.000 0.000 0.005 0.000 0.005 0.005 0.000 0.000 0.000 0.000

0.087 0.059 0.083 0.037 0.013 0.000 0.007 0.000 0.007 0.000

0.000 0.000 0.000 0.013 0.000 0.000 0.013 0.000 0.007 0.013

0.000 0.007 0.000 0.000 0.073 0.065 0.068 0.049 0.007 0.007

0.000 0.000 0.000 0.007 0.000 0.007 0.000 0.014 0.000 0.000

0.007 0.014 0.007 0.007 0.000 0.000 0.000 0.000 0.084 0.055

0.056 0.062

>Non-Methylation code ID 3175

0.005 0.000 0.000 0.005 0.005 0.000 0.000 0.000 0.010 0.010

0.000 0.000 0.005 0.000 0.010 0.010 0.000 0.005 0.000 0.000

0.075 0.065 0.075 0.050 0.006 0.006 0.000 0.006 0.000 0.006

0.000 0.000 0.019 0.013 0.000 0.000 0.006 0.006 0.000 0.006

0.000 0.000 0.000 0.000 0.053 0.062 0.063 0.078 0.006 0.000

0.000 0.000 0.000 0.006 0.000 0.000 0.025 0.013 0.000 0.000

0.006 0.000 0.006 0.006 0.000 0.000 0.000 0.000 0.061 0.063

0.094 0.045

>Non-Methylation code ID 3175

0.007 0.007 0.000 0.007 0.007 0.007 0.000 0.007 0.000 0.007

0.000 0.000 0.013 0.000 0.000 0.020 0.000 0.007 0.000 0.000

0.052 0.058 0.069 0.068 0.016 0.000 0.000 0.008 0.000 0.000

0.000 0.000 0.000 0.033 0.000 0.000 0.000 0.008 0.008 0.024

0.000 0.000 0.000 0.000 0.055 0.077 0.062 0.042 0.000 0.005

0.000 0.000 0.005 0.005 0.000 0.000 0.000 0.015 0.000 0.005

0.010 0.000 0.005 0.005 0.000 0.000 0.005 0.000 0.068 0.077

0.057 0.073

>Non-Methylation code ID 3175

0.000 0.007 0.000 0.000 0.013 0.007 0.000 0.007 0.000 0.000

0.007 0.000 0.013 0.007 0.000 0.007 0.007 0.007 0.007 0.000

0.078 0.051 0.057 0.061 0.009 0.004 0.000 0.004 0.009 0.004

0.000 0.000 0.004 0.000 0.000 0.000 0.004 0.004 0.004 0.009

0.000 0.000 0.000 0.000 0.079 0.046 0.074 0.077 0.000 0.000

0.000 0.000 0.000 0.008 0.008 0.000 0.000 0.017 0.000 0.017

0.008 0.000 0.000 0.025 0.000 0.008 0.000 0.000 0.062 0.051

0.067 0.062

>Non-Methylation code ID 21046

0.016 0.000 0.000 0.000 0.000 0.000 0.008 0.000 0.000 0.016

0.000 0.000 0.024 0.000 0.000 0.016 0.016 0.000 0.000 0.008

0.048 0.055 0.053 0.072 0.008 0.000 0.000 0.000 0.000 0.000

0.008 0.008 0.000 0.008 0.008 0.000 0.046 0.000 0.000 0.015

0.000 0.000 0.000 0.000 0.058 0.060 0.061 0.054 0.000 0.020

0.000 0.000 0.000 0.000 0.010 0.010 0.000 0.010 0.000 0.000

0.059 0.010 0.000 0.000 0.000 0.000 0.000 0.000 0.047 0.057

0.065 0.047

>Non-Methylation code ID 21046

0.010 0.000 0.000 0.005 0.010 0.000 0.000 0.000 0.000 0.010

0.000 0.000 0.005 0.000 0.010 0.010 0.000 0.000 0.005 0.000

0.065 0.067 0.080 0.055 0.000 0.000 0.000 0.000 0.007 0.020

0.007 0.000 0.000 0.013 0.000 0.000 0.007 0.000 0.007 0.013

0.000 0.007 0.000 0.000 0.082 0.054 0.073 0.044 0.000 0.006

0.000 0.012 0.006 0.006 0.000 0.006 0.006 0.006 0.000 0.000

0.012 0.000 0.000 0.006 0.000 0.000 0.000 0.000 0.080 0.056

0.079 0.052

>Non-Methylation code ID 21046

0.000 0.000 0.008 0.000 0.008 0.015 0.008 0.000 0.000 0.015

0.000 0.000 0.008 0.000 0.000 0.023 0.000 0.008 0.000 0.000

0.061 0.062 0.062 0.057 0.000 0.006 0.000 0.006 0.006 0.006

0.000 0.012 0.006 0.012 0.000 0.000 0.012 0.000 0.000 0.006

0.000 0.000 0.000 0.000 0.077 0.057 0.080 0.050 0.012 0.000

0.000 0.000 0.017 0.000 0.000 0.000 0.000 0.012 0.000 0.000

0.006 0.000 0.006 0.012 0.000 0.000 0.006 0.000 0.061 0.065

0.074 0.063

>Non-Methylation code ID 21046

0.000 0.009 0.000 0.004 0.000 0.009 0.000 0.000 0.004 0.004

0.000 0.000 0.000 0.000 0.013 0.000 0.009 0.000 0.000 0.000

0.101 0.056 0.088 0.035 0.026 0.007 0.000 0.007 0.000 0.013

0.000 0.000 0.007 0.013 0.000 0.007 0.000 0.000 0.000 0.007

0.000 0.000 0.000 0.000 0.055 0.059 0.064 0.070 0.015 0.000

0.007 0.000 0.000 0.007 0.000 0.000 0.000 0.015 0.007 0.000

0.000 0.022 0.007 0.000 0.000 0.000 0.000 0.007 0.046 0.074

0.069 0.057

>Non-Methylation code ID 21046

0.000 0.000 0.006 0.000 0.012 0.000 0.006 0.000 0.006 0.000

0.000 0.006 0.000 0.024 0.006 0.006 0.000 0.000 0.000 0.006

0.065 0.079 0.068 0.041 0.007 0.000 0.000 0.000 0.007 0.000

0.000 0.007 0.000 0.000 0.000 0.021 0.007 0.000 0.000 0.021

0.021 0.000 0.000 0.000 0.066 0.053 0.062 0.061 0.010 0.000

0.000 0.000 0.000 0.000 0.000 0.010 0.000 0.031 0.000 0.000

0.021 0.021 0.000 0.010 0.021 0.000 0.000 0.000 0.050 0.059

0.059 0.040

>Non-Methylation code ID 21046

0.013 0.000 0.007 0.000 0.000 0.000 0.000 0.000 0.000 0.013

0.007 0.007 0.000 0.013 0.007 0.013 0.000 0.000 0.000 0.007

0.067 0.057 0.068 0.054 0.000 0.009 0.000 0.000 0.000 0.004

0.000 0.000 0.004 0.004 0.000 0.000 0.000 0.000 0.013 0.000

0.013 0.004 0.000 0.000 0.077 0.066 0.089 0.049 0.025 0.008

0.000 0.008 0.000 0.008 0.000 0.000 0.000 0.017 0.000 0.008

0.000 0.008 0.000 0.017 0.000 0.000 0.000 0.000 0.046 0.053

0.069 0.065

>Non-Methylation code ID 21046

0.006 0.000 0.006 0.000 0.000 0.000 0.000 0.000 0.000 0.011

0.000 0.006 0.000 0.006 0.011 0.011 0.006 0.000 0.000 0.011

0.068 0.059 0.063 0.070 0.000 0.005 0.000 0.000 0.005 0.010

0.000 0.000 0.000 0.010 0.000 0.000 0.000 0.000 0.010 0.000

0.019 0.005 0.000 0.000 0.065 0.072 0.067 0.067 0.016 0.000

0.000 0.008 0.008 0.016 0.008 0.000 0.000 0.016 0.000 0.000

0.000 0.008 0.000 0.016 0.000 0.000 0.000 0.000 0.052 0.049

0.071 0.068

>Non-Methylation code ID 21046

0.016 0.000 0.000 0.000 0.000 0.008 0.000 0.008 0.008 0.008

0.000 0.016 0.000 0.000 0.000 0.024 0.000 0.008 0.000 0.000

0.069 0.056 0.039 0.073 0.004 0.000 0.004 0.000 0.000 0.000

0.000 0.004 0.000 0.004 0.000 0.000 0.004 0.000 0.017 0.004

0.004 0.000 0.000 0.004 0.081 0.047 0.073 0.080 0.000 0.005

0.005 0.005 0.000 0.010 0.000 0.010 0.000 0.010 0.000 0.000

0.000 0.010 0.005 0.000 0.000 0.000 0.000 0.000 0.074 0.055

0.074 0.069

>Non-Methylation code ID 21046

0.008 0.000 0.000 0.000 0.008 0.033 0.000 0.016 0.000 0.000

0.000 0.008 0.000 0.000 0.008 0.016 0.008 0.000 0.000 0.000

0.058 0.044 0.059 0.065 0.017 0.000 0.008 0.008 0.000 0.017

0.000 0.000 0.000 0.008 0.000 0.000 0.008 0.017 0.000 0.017

0.008 0.000 0.000 0.000 0.047 0.056 0.056 0.064 0.000 0.000

0.005 0.000 0.000 0.005 0.005 0.000 0.005 0.005 0.000 0.005

0.000 0.005 0.015 0.000 0.005 0.000 0.005 0.000 0.090 0.049

0.074 0.062

>Non-Methylation code ID 21046

0.020 0.000 0.007 0.007 0.000 0.007 0.000 0.007 0.007 0.007

0.007 0.000 0.007 0.007 0.000 0.000 0.007 0.000 0.000 0.000

0.052 0.060 0.065 0.071 0.000 0.000 0.000 0.000 0.000 0.000

0.005 0.000 0.005 0.005 0.000 0.000 0.010 0.000 0.015 0.010

0.005 0.000 0.005 0.000 0.093 0.061 0.072 0.049 0.000 0.006

0.006 0.000 0.000 0.017 0.006 0.000 0.000 0.000 0.000 0.006

0.006 0.006 0.006 0.006 0.006 0.000 0.000 0.000 0.051 0.053

0.085 0.077

>Non-Methylation code ID 21046

0.000 0.000 0.004 0.000 0.000 0.004 0.004 0.000 0.004 0.004

0.000 0.004 0.004 0.004 0.013 0.000 0.004 0.000 0.004 0.000

0.090 0.048 0.079 0.059 0.008 0.000 0.000 0.000 0.000 0.032

0.008 0.016 0.000 0.000 0.000 0.008 0.000 0.000 0.008 0.016

0.008 0.000 0.000 0.000 0.053 0.052 0.062 0.062 0.018 0.000

0.009 0.009 0.000 0.018 0.000 0.000 0.000 0.000 0.000 0.000

0.009 0.018 0.000 0.018 0.009 0.000 0.000 0.000 0.047 0.057

0.057 0.064

>Non-Methylation code ID 21046

0.000 0.006 0.012 0.000 0.000 0.000 0.006 0.025 0.000 0.000

0.000 0.000 0.000 0.000 0.000 0.006 0.006 0.000 0.000 0.012

0.055 0.057 0.069 0.078 0.012 0.000 0.000 0.000 0.000 0.000

0.000 0.000 0.012 0.012 0.000 0.000 0.000 0.012 0.000 0.012

0.058 0.012 0.000 0.012 0.060 0.049 0.046 0.039 0.000 0.000

0.000 0.000 0.000 0.000 0.005 0.005 0.005 0.016 0.000 0.005

0.005 0.000 0.010 0.000 0.000 0.000 0.000 0.010 0.058 0.066

0.067 0.079

>Non-Methylation code ID 21046

0.006 0.013 0.000 0.000 0.000 0.000 0.000 0.019 0.000 0.006

0.000 0.000 0.006 0.006 0.000 0.006 0.013 0.000 0.006 0.000

0.065 0.059 0.078 0.049 0.010 0.000 0.000 0.000 0.005 0.005

0.000 0.000 0.005 0.000 0.000 0.000 0.005 0.010 0.010 0.005

0.000 0.000 0.000 0.005 0.062 0.084 0.070 0.057 0.008 0.000

0.008 0.000 0.000 0.000 0.008 0.000 0.000 0.016 0.008 0.023

0.000 0.000 0.000 0.016 0.000 0.008 0.000 0.000 0.062 0.058

0.053 0.067

>Non-Methylation code ID 21046

0.007 0.000 0.000 0.000 0.007 0.000 0.000 0.007 0.000 0.007

0.000 0.021 0.007 0.000 0.000 0.021 0.014 0.000 0.000 0.000

0.065 0.055 0.069 0.055 0.018 0.009 0.000 0.000 0.000 0.000

0.000 0.009 0.000 0.027 0.000 0.000 0.018 0.009 0.000 0.009

0.018 0.000 0.000 0.000 0.054 0.045 0.063 0.055 0.000 0.000

0.000 0.000 0.012 0.000 0.006 0.000 0.006 0.000 0.000 0.000

0.006 0.024 0.006 0.006 0.000 0.000 0.000 0.006 0.054 0.079

0.074 0.053

>Non-Methylation code ID 21046

0.009 0.000 0.000 0.000 0.009 0.034 0.000 0.017 0.000 0.000

0.000 0.000 0.000 0.000 0.009 0.026 0.009 0.000 0.000 0.000

0.064 0.038 0.060 0.059 0.014 0.000 0.007 0.007 0.000 0.014

0.000 0.000 0.000 0.007 0.000 0.000 0.007 0.014 0.007 0.014

0.000 0.000 0.000 0.000 0.052 0.060 0.059 0.073 0.000 0.000

0.005 0.000 0.000 0.005 0.005 0.000 0.000 0.005 0.000 0.005

0.000 0.005 0.014 0.000 0.005 0.005 0.005 0.000 0.087 0.053

0.077 0.060

>Non-Methylation code ID 21046

0.019 0.006 0.000 0.006 0.000 0.012 0.000 0.000 0.006 0.019

0.000 0.006 0.000 0.000 0.000 0.006 0.000 0.000 0.000 0.000

0.062 0.063 0.060 0.067 0.013 0.000 0.006 0.000 0.000 0.006

0.000 0.000 0.000 0.006 0.006 0.006 0.000 0.019 0.006 0.000

0.000 0.000 0.000 0.013 0.047 0.070 0.066 0.068 0.000 0.005

0.000 0.005 0.000 0.005 0.000 0.005 0.005 0.005 0.000 0.000

0.000 0.000 0.014 0.000 0.009 0.000 0.000 0.000 0.088 0.053

0.087 0.054

>Non-Methylation code ID 21046

0.000 0.005 0.000 0.000 0.000 0.000 0.005 0.009 0.000 0.000

0.000 0.000 0.014 0.000 0.009 0.000 0.005 0.005 0.005 0.000

0.072 0.059 0.069 0.077 0.011 0.000 0.005 0.000 0.000 0.005

0.005 0.000 0.000 0.000 0.000 0.000 0.011 0.000 0.005 0.005

0.005 0.005 0.000 0.005 0.044 0.072 0.084 0.069 0.013 0.000

0.000 0.000 0.000 0.000 0.013 0.000 0.000 0.000 0.013 0.007

0.013 0.000 0.007 0.007 0.007 0.000 0.000 0.000 0.066 0.069

0.057 0.063

>Non-Methylation code ID 21046

0.006 0.006 0.013 0.000 0.000 0.000 0.006 0.013 0.000 0.006

0.000 0.000 0.000 0.000 0.000 0.006 0.006 0.000 0.000 0.013

0.064 0.060 0.066 0.068 0.009 0.000 0.000 0.000 0.000 0.000

0.000 0.000 0.009 0.009 0.000 0.000 0.000 0.009 0.009 0.009

0.044 0.009 0.000 0.009 0.066 0.067 0.037 0.048 0.000 0.005

0.000 0.000 0.000 0.000 0.005 0.005 0.005 0.015 0.000 0.000

0.010 0.000 0.010 0.000 0.000 0.000 0.000 0.005 0.062 0.075

0.059 0.077

>Non-Methylation code ID 21046

0.009 0.000 0.000 0.000 0.009 0.034 0.000 0.017 0.009 0.000

0.000 0.000 0.000 0.000 0.000 0.017 0.009 0.009 0.000 0.000

0.067 0.043 0.054 0.058 0.012 0.000 0.006 0.000 0.000 0.012

0.000 0.000 0.000 0.006 0.000 0.000 0.006 0.006 0.012 0.018

0.000 0.000 0.000 0.000 0.068 0.048 0.070 0.071 0.000 0.000

0.005 0.000 0.000 0.010 0.005 0.005 0.000 0.000 0.000 0.005

0.000 0.010 0.010 0.000 0.000 0.005 0.005 0.000 0.061 0.055

0.082 0.077

>Non-Methylation code ID 21046

0.000 0.010 0.000 0.000 0.000 0.000 0.005 0.010 0.000 0.005

0.000 0.000 0.010 0.000 0.005 0.000 0.010 0.005 0.005 0.000

0.081 0.053 0.078 0.055 0.012 0.000 0.000 0.000 0.000 0.006

0.000 0.000 0.000 0.000 0.000 0.000 0.012 0.000 0.012 0.006

0.006 0.006 0.000 0.012 0.061 0.086 0.070 0.047 0.000 0.000

0.006 0.000 0.000 0.000 0.011 0.000 0.000 0.006 0.006 0.006

0.011 0.000 0.006 0.006 0.006 0.006 0.000 0.000 0.070 0.057

0.057 0.082

>Non-Methylation code ID 21046

0.012 0.000 0.000 0.000 0.000 0.000 0.012 0.000 0.000 0.000

0.018 0.006 0.012 0.000 0.006 0.006 0.006 0.000 0.000 0.000

0.063 0.066 0.057 0.071 0.000 0.005 0.000 0.000 0.000 0.000

0.005 0.009 0.000 0.000 0.000 0.000 0.014 0.000 0.009 0.000

0.005 0.005 0.005 0.000 0.072 0.059 0.069 0.077 0.006 0.000

0.006 0.000 0.000 0.006 0.006 0.000 0.000 0.000 0.000 0.000

0.012 0.000 0.006 0.006 0.006 0.006 0.000 0.006 0.050 0.073

0.086 0.059

>Non-Methylation code ID 21046

0.006 0.006 0.000 0.000 0.006 0.013 0.000 0.000 0.006 0.013

0.000 0.000 0.000 0.000 0.000 0.013 0.006 0.000 0.000 0.006

0.061 0.071 0.065 0.059 0.021 0.005 0.000 0.000 0.000 0.000

0.000 0.005 0.000 0.005 0.000 0.000 0.011 0.000 0.011 0.000

0.000 0.000 0.005 0.000 0.071 0.071 0.062 0.065 0.005 0.005

0.000 0.000 0.005 0.010 0.005 0.000 0.005 0.015 0.000 0.000

0.000 0.005 0.005 0.000 0.000 0.000 0.000 0.000 0.088 0.052

0.081 0.051

>Non-Methylation code ID 21046

0.000 0.000 0.000 0.000 0.000 0.005 0.000 0.005 0.000 0.005

0.000 0.000 0.000 0.000 0.019 0.010 0.015 0.005 0.000 0.000

0.047 0.060 0.082 0.082 0.006 0.000 0.013 0.000 0.000 0.013

0.000 0.000 0.000 0.006 0.000 0.000 0.006 0.013 0.006 0.006

0.000 0.006 0.000 0.000 0.074 0.053 0.062 0.067 0.000 0.000

0.012 0.006 0.006 0.000 0.000 0.006 0.006 0.000 0.000 0.006

0.000 0.012 0.000 0.006 0.006 0.000 0.006 0.000 0.073 0.057

0.069 0.061

>Non-Methylation code ID 21046

0.012 0.000 0.000 0.000 0.000 0.018 0.000 0.000 0.006 0.006

0.000 0.000 0.000 0.000 0.006 0.012 0.000 0.006 0.012 0.000

0.060 0.070 0.069 0.059 0.006 0.006 0.000 0.017 0.000 0.012

0.000 0.000 0.000 0.006 0.000 0.000 0.006 0.000 0.006 0.012

0.000 0.000 0.000 0.000 0.056 0.074 0.074 0.060 0.000 0.000

0.000 0.000 0.000 0.013 0.000 0.000 0.013 0.006 0.000 0.000

0.000 0.006 0.006 0.006 0.000 0.006 0.006 0.000 0.063 0.076

0.058 0.071

>Non-Methylation code ID 21127

0.018 0.000 0.000 0.000 0.006 0.012 0.000 0.006 0.006 0.000

0.000 0.006 0.006 0.006 0.006 0.000 0.006 0.000 0.000 0.000

0.075 0.062 0.065 0.053 0.016 0.000 0.000 0.000 0.000 0.000

0.000 0.000 0.000 0.005 0.000 0.000 0.016 0.005 0.016 0.005

0.005 0.000 0.000 0.000 0.050 0.053 0.089 0.071 0.005 0.000

0.010 0.000 0.000 0.000 0.000 0.000 0.000 0.000 0.000 0.000

0.010 0.005 0.021 0.005 0.000 0.000 0.000 0.000 0.075 0.062

0.078 0.061

>Non-Methylation code ID 21127

0.005 0.016 0.000 0.000 0.000 0.005 0.005 0.000 0.000 0.011

0.000 0.005 0.011 0.000 0.005 0.000 0.000 0.000 0.000 0.000

0.098 0.049 0.076 0.045 0.011 0.011 0.000 0.000 0.000 0.006

0.000 0.000 0.000 0.000 0.000 0.000 0.011 0.000 0.006 0.000

0.011 0.006 0.006 0.000 0.076 0.067 0.069 0.055 0.007 0.007

0.000 0.000 0.000 0.007 0.000 0.000 0.000 0.007 0.000 0.000

0.015 0.007 0.007 0.007 0.000 0.000 0.000 0.000 0.084 0.052

0.072 0.058

>Non-Methylation code ID 21127

0.000 0.000 0.000 0.000 0.000 0.000 0.006 0.006 0.000 0.006

0.006 0.006 0.025 0.000 0.006 0.006 0.006 0.006 0.000 0.000

0.071 0.055 0.064 0.062 0.006 0.006 0.000 0.000 0.006 0.000

0.013 0.000 0.000 0.006 0.000 0.000 0.019 0.000 0.000 0.000

0.013 0.000 0.000 0.006 0.054 0.074 0.060 0.068 0.006 0.000

0.000 0.000 0.006 0.000 0.012 0.000 0.000 0.000 0.000 0.006

0.018 0.000 0.006 0.000 0.012 0.000 0.000 0.000 0.076 0.054

0.072 0.066

>Non-Methylation code ID 21127

0.017 0.000 0.000 0.004 0.000 0.000 0.000 0.004 0.000 0.000

0.000 0.000 0.000 0.004 0.017 0.004 0.004 0.000 0.000 0.000

0.048 0.069 0.091 0.072 0.038 0.000 0.000 0.000 0.000 0.000

0.000 0.000 0.000 0.000 0.000 0.000 0.038 0.000 0.008 0.015

0.000 0.000 0.000 0.000 0.055 0.061 0.058 0.060 0.005 0.000

0.000 0.000 0.000 0.000 0.010 0.000 0.000 0.000 0.000 0.000

0.020 0.005 0.020 0.000 0.000 0.000 0.000 0.000 0.081 0.046

0.083 0.064

>Non-Methylation code ID 21127

0.010 0.000 0.000 0.000 0.000 0.000 0.010 0.000 0.000 0.000

0.000 0.000 0.020 0.005 0.020 0.000 0.000 0.000 0.000 0.000

0.061 0.050 0.075 0.081 0.016 0.000 0.000 0.005 0.000 0.000

0.011 0.005 0.000 0.011 0.000 0.000 0.000 0.000 0.011 0.005

0.005 0.000 0.000 0.000 0.056 0.077 0.065 0.065 0.028 0.009

0.000 0.000 0.000 0.000 0.000 0.000 0.000 0.000 0.000 0.000

0.037 0.000 0.000 0.019 0.019 0.000 0.000 0.000 0.055 0.057

0.065 0.044

>Non-Methylation code ID 21127

0.000 0.000 0.007 0.000 0.000 0.000 0.007 0.007 0.000 0.007

0.007 0.007 0.014 0.007 0.000 0.007 0.007 0.007 0.007 0.000

0.069 0.054 0.065 0.054 0.000 0.006 0.000 0.000 0.006 0.006

0.011 0.006 0.000 0.006 0.000 0.000 0.006 0.000 0.006 0.000

0.011 0.000 0.000 0.006 0.059 0.082 0.060 0.066 0.008 0.000

0.000 0.000 0.008 0.008 0.016 0.000 0.000 0.008 0.000 0.008

0.008 0.000 0.000 0.000 0.023 0.000 0.000 0.000 0.079 0.046

0.061 0.062

>Non-Methylation code ID 21127

0.008 0.000 0.000 0.000 0.000 0.008 0.000 0.000 0.000 0.000

0.000 0.000 0.013 0.000 0.013 0.000 0.004 0.000 0.004 0.000

0.074 0.059 0.072 0.079 0.005 0.005 0.000 0.000 0.000 0.014

0.000 0.000 0.000 0.009 0.000 0.000 0.005 0.000 0.014 0.000

0.000 0.000 0.000 0.000 0.084 0.053 0.065 0.080 0.028 0.021

0.000 0.000 0.000 0.014 0.007 0.000 0.000 0.000 0.000 0.000

0.000 0.007 0.000 0.000 0.000 0.000 0.000 0.000 0.083 0.043

0.065 0.065

>Non-Methylation code ID 21127

0.016 0.000 0.000 0.004 0.000 0.000 0.000 0.004 0.000 0.000

0.000 0.000 0.000 0.004 0.016 0.004 0.004 0.000 0.000 0.000

0.048 0.062 0.088 0.084 0.037 0.000 0.000 0.000 0.000 0.000

0.000 0.000 0.000 0.000 0.000 0.000 0.037 0.000 0.007 0.015

0.000 0.000 0.000 0.000 0.067 0.051 0.056 0.063 0.005 0.000

0.000 0.000 0.000 0.005 0.010 0.000 0.000 0.000 0.000 0.000

0.019 0.000 0.019 0.000 0.000 0.000 0.000 0.000 0.078 0.055

0.079 0.065

>Non-Methylation code ID 21127

0.005 0.000 0.000 0.000 0.005 0.000 0.005 0.000 0.000 0.000

0.005 0.005 0.014 0.005 0.009 0.005 0.005 0.000 0.000 0.000

0.063 0.060 0.077 0.074 0.005 0.005 0.000 0.000 0.000 0.000

0.005 0.005 0.000 0.005 0.005 0.005 0.016 0.000 0.005 0.005

0.000 0.005 0.000 0.000 0.075 0.052 0.077 0.059 0.015 0.008

0.000 0.000 0.008 0.000 0.000 0.000 0.000 0.008 0.000 0.000

0.023 0.000 0.000 0.000 0.015 0.000 0.000 0.008 0.055 0.075

0.064 0.055

>Non-Methylation code ID 21127

0.020 0.000 0.000 0.000 0.000 0.013 0.000 0.007 0.007 0.000

0.000 0.007 0.007 0.007 0.007 0.000 0.007 0.000 0.000 0.000

0.069 0.063 0.065 0.057 0.016 0.000 0.000 0.000 0.000 0.000

0.000 0.000 0.000 0.000 0.000 0.000 0.016 0.005 0.016 0.005

0.005 0.000 0.005 0.000 0.046 0.051 0.081 0.084 0.005 0.000

0.010 0.000 0.000 0.000 0.000 0.000 0.000 0.000 0.000 0.000

0.010 0.005 0.024 0.005 0.000 0.000 0.000 0.000 0.069 0.066

0.070 0.071

>Non-Methylation code ID 21127

0.011 0.005 0.005 0.000 0.000 0.000 0.005 0.000 0.000 0.011

0.000 0.005 0.011 0.000 0.005 0.000 0.005 0.005 0.000 0.000

0.078 0.067 0.069 0.049 0.009 0.000 0.000 0.000 0.009 0.009

0.009 0.000 0.000 0.035 0.000 0.000 0.009 0.000 0.000 0.009

0.026 0.000 0.000 0.000 0.059 0.045 0.056 0.058 0.007 0.013

0.000 0.000 0.000 0.000 0.007 0.000 0.000 0.000 0.007 0.000

0.013 0.007 0.007 0.013 0.000 0.000 0.000 0.000 0.052 0.072

0.062 0.075

>Non-Methylation code ID 21127

0.006 0.000 0.000 0.000 0.006 0.000 0.011 0.000 0.000 0.000

0.000 0.006 0.022 0.000 0.006 0.000 0.011 0.000 0.000 0.000

0.070 0.049 0.082 0.065 0.000 0.000 0.000 0.000 0.000 0.000

0.006 0.006 0.000 0.006 0.006 0.006 0.025 0.000 0.006 0.006

0.006 0.006 0.000 0.000 0.071 0.055 0.064 0.062 0.007 0.007

0.000 0.000 0.007 0.000 0.007 0.000 0.000 0.007 0.000 0.000

0.020 0.000 0.000 0.000 0.013 0.000 0.000 0.007 0.054 0.080

0.061 0.064

>Non-Methylation code ID 21127

0.007 0.007 0.000 0.000 0.007 0.000 0.007 0.000 0.000 0.020

0.000 0.000 0.000 0.000 0.007 0.007 0.000 0.020 0.000 0.000

0.079 0.049 0.057 0.067 0.000 0.005 0.000 0.000 0.010 0.005

0.000 0.000 0.000 0.010 0.000 0.000 0.000 0.000 0.005 0.015

0.005 0.000 0.005 0.000 0.060 0.061 0.084 0.068 0.005 0.010

0.000 0.000 0.005 0.005 0.000 0.000 0.000 0.005 0.000 0.000

0.005 0.000 0.005 0.010 0.000 0.010 0.000 0.000 0.064 0.073

0.061 0.072

>Non-Methylation code ID 21127

0.009 0.000 0.004 0.000 0.000 0.009 0.000 0.000 0.000 0.004

0.000 0.000 0.009 0.000 0.009 0.000 0.004 0.004 0.004 0.000

0.068 0.049 0.077 0.082 0.006 0.000 0.000 0.000 0.006 0.019

0.000 0.000 0.000 0.019 0.000 0.000 0.006 0.000 0.012 0.000

0.006 0.000 0.000 0.000 0.086 0.031 0.082 0.060 0.021 0.021

0.000 0.000 0.000 0.014 0.007 0.000 0.000 0.000 0.000 0.000

0.007 0.007 0.000 0.000 0.000 0.000 0.000 0.000 0.066 0.055

0.060 0.073

>Non-Methylation code ID 21127

0.006 0.000 0.000 0.000 0.006 0.006 0.012 0.000 0.000 0.000

0.000 0.006 0.012 0.000 0.006 0.000 0.018 0.000 0.000 0.000

0.082 0.042 0.070 0.068 0.000 0.000 0.008 0.000 0.000 0.000

0.008 0.008 0.000 0.008 0.008 0.008 0.023 0.008 0.000 0.008

0.008 0.008 0.000 0.000 0.061 0.058 0.061 0.053 0.000 0.007

0.000 0.000 0.007 0.000 0.013 0.000 0.000 0.007 0.000 0.000

0.013 0.000 0.007 0.000 0.013 0.000 0.000 0.007 0.057 0.082

0.055 0.067

>Non-Methylation code ID 21135

0.000 0.000 0.000 0.000 0.000 0.004 0.000 0.000 0.000 0.000

0.000 0.000 0.022 0.004 0.022 0.004 0.000 0.000 0.000 0.000

0.060 0.074 0.063 0.080 0.017 0.000 0.000 0.000 0.000 0.017

0.012 0.000 0.000 0.000 0.000 0.006 0.006 0.000 0.012 0.000

0.000 0.000 0.000 0.000 0.051 0.067 0.065 0.081 0.032 0.000

0.000 0.000 0.000 0.000 0.000 0.000 0.000 0.000 0.000 0.000

0.024 0.008 0.000 0.000 0.024 0.000 0.008 0.000 0.066 0.046

0.063 0.062

>Non-Methylation code ID 21135

0.013 0.000 0.013 0.000 0.007 0.000 0.000 0.007 0.007 0.020

0.000 0.000 0.007 0.000 0.000 0.000 0.007 0.000 0.000 0.000

0.078 0.048 0.067 0.061 0.000 0.000 0.000 0.000 0.000 0.000

0.000 0.012 0.000 0.006 0.000 0.006 0.012 0.006 0.006 0.018

0.000 0.000 0.006 0.000 0.068 0.077 0.069 0.047 0.006 0.000

0.006 0.000 0.000 0.006 0.000 0.006 0.000 0.013 0.000 0.000

0.000 0.006 0.006 0.013 0.000 0.006 0.000 0.006 0.063 0.061

0.065 0.068

>Non-Methylation code ID 21135

0.000 0.004 0.000 0.004 0.000 0.000 0.000 0.004 0.000 0.000

0.000 0.000 0.009 0.000 0.017 0.009 0.004 0.000 0.000 0.000

0.066 0.085 0.070 0.060 0.009 0.000 0.005 0.000 0.000 0.009

0.000 0.000 0.005 0.009 0.000 0.000 0.000 0.000 0.014 0.005

0.000 0.005 0.000 0.000 0.065 0.058 0.046 0.103 0.007 0.000

0.007 0.007 0.000 0.007 0.007 0.007 0.007 0.007 0.000 0.000

0.007 0.007 0.000 0.007 0.000 0.000 0.000 0.000 0.067 0.064

0.071 0.057

>Non-Methylation code ID 21135

0.012 0.000 0.006 0.000 0.000 0.012 0.006 0.012 0.012 0.000

0.006 0.000 0.006 0.000 0.000 0.000 0.000 0.000 0.006 0.000

0.066 0.081 0.058 0.050 0.004 0.000 0.000 0.000 0.004 0.009

0.000 0.000 0.009 0.000 0.004 0.000 0.004 0.000 0.009 0.004

0.004 0.000 0.004 0.000 0.070 0.071 0.068 0.068 0.005 0.000

0.000 0.000 0.000 0.015 0.010 0.000 0.005 0.000 0.000 0.005

0.000 0.000 0.005 0.005 0.000 0.000 0.005 0.000 0.043 0.067

0.087 0.081

>Non-Methylation code ID 21135

0.000 0.011 0.000 0.006 0.006 0.000 0.000 0.006 0.006 0.006

0.000 0.000 0.000 0.000 0.006 0.011 0.000 0.000 0.000 0.000

0.076 0.092 0.071 0.039 0.018 0.012 0.006 0.000 0.000 0.000

0.000 0.000 0.012 0.006 0.000 0.000 0.006 0.000 0.000 0.018

0.000 0.000 0.000 0.000 0.077 0.067 0.047 0.065 0.000 0.000

0.000 0.000 0.000 0.000 0.008 0.000 0.000 0.008 0.000 0.000

0.008 0.008 0.008 0.008 0.008 0.032 0.000 0.000 0.070 0.073

0.060 0.043

>Non-Methylation code ID 21135

0.000 0.008 0.000 0.004 0.000 0.000 0.000 0.004 0.000 0.000

0.000 0.004 0.008 0.004 0.012 0.004 0.004 0.000 0.000 0.000

0.065 0.087 0.068 0.061 0.022 0.000 0.000 0.000 0.000 0.011

0.006 0.006 0.006 0.006 0.000 0.000 0.000 0.000 0.011 0.006

0.000 0.000 0.000 0.000 0.063 0.060 0.072 0.067 0.008 0.000

0.008 0.008 0.000 0.000 0.008 0.000 0.008 0.008 0.000 0.000

0.016 0.008 0.000 0.000 0.008 0.000 0.000 0.000 0.066 0.062

0.057 0.069

>Non-Methylation code ID 21135

0.011 0.005 0.011 0.000 0.000 0.005 0.000 0.000 0.005 0.005

0.000 0.000 0.011 0.000 0.005 0.005 0.000 0.000 0.000 0.000

0.049 0.071 0.066 0.084 0.007 0.000 0.007 0.007 0.000 0.000

0.000 0.014 0.000 0.014 0.000 0.007 0.029 0.000 0.000 0.000

0.000 0.000 0.000 0.000 0.065 0.065 0.064 0.053 0.000 0.005

0.000 0.000 0.000 0.000 0.000 0.005 0.000 0.005 0.000 0.000

0.011 0.005 0.011 0.011 0.005 0.000 0.000 0.000 0.073 0.064

0.077 0.059

>Non-Methylation code ID 21135

0.005 0.000 0.005 0.000 0.000 0.005 0.000 0.005 0.000 0.011

0.000 0.000 0.000 0.005 0.011 0.011 0.000 0.005 0.000 0.005

0.062 0.066 0.067 0.068 0.013 0.000 0.013 0.000 0.007 0.000

0.000 0.007 0.007 0.020 0.000 0.000 0.007 0.000 0.000 0.000

0.007 0.000 0.000 0.000 0.078 0.048 0.067 0.061 0.000 0.000

0.000 0.000 0.000 0.000 0.000 0.012 0.000 0.000 0.000 0.006

0.012 0.006 0.006 0.019 0.000 0.000 0.006 0.000 0.067 0.078

0.073 0.047

>Non-Methylation code ID 21135

0.000 0.004 0.004 0.000 0.000 0.004 0.000 0.004 0.000 0.009

0.000 0.000 0.000 0.000 0.009 0.009 0.004 0.004 0.004 0.000

0.076 0.055 0.068 0.077 0.011 0.000 0.005 0.000 0.000 0.011

0.000 0.000 0.000 0.000 0.000 0.000 0.005 0.000 0.005 0.000

0.005 0.000 0.005 0.011 0.068 0.066 0.074 0.067 0.006 0.000

0.000 0.000 0.000 0.006 0.006 0.006 0.000 0.000 0.006 0.006

0.000 0.013 0.006 0.000 0.006 0.006 0.006 0.000 0.065 0.065

0.070 0.057

>Non-Methylation code ID 21135

0.011 0.006 0.011 0.000 0.000 0.006 0.000 0.000 0.006 0.000

0.000 0.000 0.011 0.000 0.006 0.011 0.000 0.000 0.000 0.000

0.052 0.061 0.062 0.090 0.007 0.000 0.007 0.007 0.000 0.000

0.000 0.015 0.000 0.007 0.000 0.007 0.037 0.000 0.000 0.000

0.000 0.000 0.000 0.000 0.070 0.055 0.064 0.055 0.000 0.005

0.000 0.000 0.000 0.000 0.000 0.005 0.000 0.011 0.000 0.000

0.005 0.005 0.011 0.011 0.005 0.000 0.000 0.000 0.070 0.074

0.079 0.052

>Non-Methylation code ID 21135

0.000 0.000 0.000 0.000 0.004 0.004 0.004 0.000 0.000 0.000

0.000 0.000 0.009 0.000 0.018 0.004 0.004 0.000 0.004 0.000

0.072 0.075 0.065 0.068 0.000 0.000 0.005 0.010 0.005 0.015

0.010 0.000 0.000 0.005 0.000 0.000 0.000 0.000 0.005 0.000

0.005 0.005 0.000 0.000 0.066 0.076 0.069 0.057 0.020 0.000

0.000 0.000 0.000 0.000 0.000 0.007 0.000 0.007 0.007 0.007

0.007 0.000 0.007 0.007 0.007 0.007 0.000 0.000 0.064 0.055

0.051 0.084

>Non-Methylation code ID 21135

0.012 0.018 0.000 0.000 0.006 0.000 0.000 0.006 0.006 0.006

0.000 0.000 0.006 0.000 0.000 0.012 0.000 0.000 0.000 0.000

0.066 0.089 0.068 0.037 0.035 0.000 0.000 0.000 0.009 0.000

0.000 0.000 0.000 0.017 0.000 0.000 0.017 0.009 0.000 0.026

0.000 0.000 0.000 0.000 0.051 0.064 0.060 0.046 0.000 0.000

0.000 0.000 0.000 0.000 0.007 0.000 0.000 0.022 0.000 0.000

0.007 0.007 0.007 0.015 0.000 0.015 0.000 0.000 0.066 0.078

0.060 0.050

>Non-Methylation code ID 21135

0.011 0.011 0.000 0.006 0.000 0.006 0.000 0.000 0.000 0.006

0.000 0.006 0.006 0.011 0.006 0.006 0.000 0.000 0.000 0.000

0.080 0.055 0.062 0.061 0.016 0.000 0.000 0.000 0.000 0.005

0.005 0.005 0.010 0.000 0.000 0.000 0.000 0.005 0.010 0.000

0.000 0.005 0.000 0.000 0.044 0.078 0.091 0.057 0.007 0.000

0.000 0.000 0.000 0.007 0.000 0.000 0.007 0.007 0.000 0.000

0.007 0.007 0.014 0.007 0.007 0.000 0.000 0.000 0.084 0.089

0.022 0.071

>Non-Methylation code ID 21135

0.013 0.000 0.006 0.000 0.000 0.013 0.006 0.013 0.006 0.000

0.006 0.000 0.006 0.000 0.000 0.000 0.000 0.006 0.006 0.000

0.078 0.077 0.048 0.046 0.004 0.000 0.000 0.000 0.004 0.008

0.000 0.000 0.004 0.000 0.004 0.000 0.004 0.000 0.008 0.004

0.004 0.000 0.004 0.004 0.070 0.066 0.072 0.070 0.005 0.000

0.000 0.000 0.000 0.014 0.009 0.000 0.000 0.000 0.000 0.005

0.000 0.000 0.009 0.005 0.000 0.000 0.005 0.000 0.051 0.072

0.088 0.070

>Non-Methylation code ID 21135

0.006 0.000 0.006 0.000 0.000 0.000 0.000 0.000 0.006 0.006

0.000 0.000 0.000 0.006 0.011 0.000 0.006 0.017 0.000 0.006

0.044 0.065 0.082 0.076 0.006 0.000 0.000 0.000 0.000 0.006

0.000 0.006 0.000 0.006 0.006 0.000 0.000 0.006 0.013 0.013

0.000 0.000 0.000 0.006 0.075 0.083 0.043 0.062 0.013 0.006

0.000 0.006 0.000 0.006 0.000 0.006 0.000 0.013 0.000 0.000

0.000 0.006 0.006 0.006 0.000 0.000 0.000 0.006 0.071 0.051

0.076 0.058

>Non-Methylation code ID 21135

0.000 0.000 0.010 0.000 0.000 0.005 0.000 0.005 0.005 0.010

0.000 0.000 0.005 0.000 0.010 0.010 0.000 0.005 0.000 0.000

0.058 0.073 0.067 0.069 0.013 0.000 0.013 0.000 0.007 0.000

0.000 0.007 0.000 0.013 0.000 0.007 0.013 0.000 0.000 0.000

0.007 0.000 0.000 0.000 0.070 0.062 0.057 0.064 0.000 0.000

0.000 0.000 0.000 0.000 0.000 0.006 0.000 0.000 0.000 0.006

0.012 0.012 0.006 0.012 0.006 0.000 0.006 0.000 0.065 0.076

0.074 0.050

>Non-Methylation code ID 21135

0.018 0.000 0.000 0.000 0.006 0.006 0.000 0.000 0.000 0.000

0.000 0.006 0.000 0.000 0.006 0.012 0.018 0.000 0.006 0.000

0.067 0.073 0.066 0.048 0.014 0.000 0.000 0.000 0.000 0.007

0.000 0.000 0.000 0.007 0.000 0.000 0.041 0.007 0.007 0.000

0.007 0.000 0.000 0.000 0.069 0.059 0.066 0.051 0.000 0.005

0.005 0.005 0.000 0.000 0.005 0.000 0.000 0.020 0.000 0.000

0.005 0.000 0.010 0.005 0.000 0.000 0.000 0.000 0.085 0.065

0.059 0.065

>Non-Methylation code ID 21135

0.006 0.000 0.006 0.000 0.000 0.006 0.000 0.000 0.000 0.000

0.000 0.000 0.025 0.006 0.006 0.019 0.006 0.000 0.000 0.000

0.063 0.061 0.055 0.071 0.012 0.000 0.000 0.000 0.000 0.000

0.012 0.000 0.006 0.012 0.000 0.000 0.023 0.000 0.006 0.000

0.006 0.000 0.000 0.000 0.075 0.061 0.065 0.056 0.000 0.000

0.000 0.000 0.005 0.000 0.005 0.000 0.000 0.005 0.000 0.000

0.020 0.000 0.010 0.000 0.010 0.000 0.000 0.000 0.072 0.070

0.074 0.061

>Non-Methylation code ID 21135

0.004 0.000 0.000 0.000 0.000 0.009 0.004 0.000 0.000 0.000

0.000 0.000 0.013 0.000 0.013 0.000 0.009 0.004 0.000 0.000

0.079 0.055 0.065 0.078 0.005 0.000 0.000 0.000 0.000 0.015

0.005 0.000 0.000 0.010 0.000 0.000 0.010 0.000 0.010 0.000

0.005 0.000 0.000 0.000 0.069 0.071 0.075 0.057 0.017 0.006

0.000 0.000 0.000 0.006 0.012 0.000 0.000 0.000 0.000 0.000

0.012 0.000 0.000 0.000 0.006 0.000 0.012 0.000 0.061 0.065

0.057 0.081

>Non-Methylation code ID 21135

0.004 0.000 0.000 0.000 0.000 0.021 0.000 0.000 0.000 0.004

0.000 0.000 0.004 0.000 0.017 0.000 0.000 0.000 0.004 0.000

0.066 0.066 0.051 0.095 0.035 0.009 0.000 0.000 0.000 0.026

0.009 0.000 0.000 0.000 0.000 0.000 0.000 0.009 0.000 0.000

0.000 0.009 0.000 0.000 0.067 0.063 0.043 0.064 0.018 0.000

0.000 0.000 0.000 0.006 0.000 0.000 0.000 0.006 0.000 0.000

0.012 0.000 0.012 0.000 0.006 0.000 0.012 0.000 0.045 0.064

0.075 0.077

>Non-Methylation code ID 21135

0.018 0.006 0.000 0.000 0.000 0.006 0.006 0.000 0.000 0.000

0.000 0.000 0.012 0.018 0.000 0.000 0.006 0.000 0.006 0.000

0.058 0.053 0.066 0.076 0.004 0.000 0.000 0.000 0.000 0.004

0.004 0.000 0.000 0.000 0.000 0.004 0.013 0.000 0.013 0.004

0.004 0.004 0.000 0.000 0.083 0.053 0.080 0.061 0.014 0.000

0.000 0.000 0.000 0.014 0.007 0.000 0.000 0.007 0.000 0.000

0.014 0.000 0.007 0.007 0.007 0.000 0.000 0.000 0.061 0.065

0.068 0.065

>Non-Methylation code ID 21135

0.008 0.008 0.000 0.000 0.008 0.000 0.000 0.000 0.000 0.016

0.000 0.008 0.000 0.000 0.000 0.024 0.024 0.000 0.008 0.000

0.066 0.045 0.061 0.056 0.006 0.000 0.000 0.000 0.006 0.006

0.000 0.000 0.000 0.023 0.000 0.000 0.018 0.000 0.006 0.006

0.006 0.000 0.000 0.000 0.058 0.050 0.078 0.071 0.000 0.007

0.007 0.007 0.007 0.000 0.007 0.000 0.000 0.027 0.000 0.000

0.000 0.000 0.000 0.013 0.000 0.000 0.000 0.007 0.082 0.049

0.066 0.056

>Non-Methylation code ID 21135

0.000 0.017 0.000 0.000 0.000 0.000 0.000 0.000 0.000 0.025

0.000 0.008 0.000 0.008 0.000 0.033 0.000 0.008 0.000 0.000

0.055 0.058 0.058 0.062 0.006 0.011 0.006 0.000 0.000 0.000

0.000 0.000 0.000 0.000 0.000 0.006 0.017 0.000 0.006 0.006

0.006 0.000 0.000 0.000 0.068 0.078 0.048 0.076 0.000 0.000

0.000 0.000 0.000 0.000 0.000 0.009 0.009 0.036 0.000 0.000

0.009 0.000 0.009 0.000 0.009 0.027 0.000 0.000 0.073 0.030

0.065 0.056

>Non-Methylation code ID 21135

0.011 0.000 0.000 0.000 0.011 0.000 0.006 0.000 0.000 0.006

0.000 0.000 0.011 0.006 0.006 0.000 0.011 0.000 0.000 0.000

0.051 0.072 0.080 0.062 0.005 0.000 0.005 0.000 0.000 0.000

0.000 0.000 0.000 0.005 0.000 0.000 0.022 0.000 0.011 0.016

0.005 0.000 0.000 0.000 0.074 0.073 0.051 0.064 0.006 0.000

0.006 0.000 0.006 0.000 0.006 0.000 0.000 0.012 0.000 0.000

0.024 0.000 0.006 0.000 0.006 0.000 0.000 0.000 0.062 0.071

0.056 0.072

>Non-Methylation code ID 21135

0.007 0.000 0.000 0.000 0.000 0.007 0.000 0.000 0.000 0.007

0.000 0.000 0.033 0.000 0.007 0.013 0.013 0.000 0.000 0.000

0.056 0.067 0.052 0.074 0.004 0.000 0.004 0.000 0.004 0.000

0.004 0.000 0.000 0.009 0.000 0.000 0.013 0.004 0.013 0.000

0.000 0.000 0.000 0.000 0.068 0.080 0.067 0.061 0.021 0.000

0.000 0.000 0.014 0.000 0.007 0.000 0.000 0.000 0.000 0.007

0.007 0.007 0.007 0.000 0.014 0.000 0.000 0.000 0.064 0.077

0.042 0.068

>Non-Methylation code ID 21135

0.000 0.006 0.006 0.006 0.006 0.000 0.011 0.000 0.000 0.017

0.000 0.000 0.000 0.000 0.000 0.017 0.000 0.000 0.000 0.006

0.079 0.050 0.067 0.064 0.017 0.000 0.000 0.000 0.008 0.000

0.000 0.000 0.000 0.017 0.000 0.008 0.000 0.000 0.000 0.017

0.033 0.000 0.008 0.000 0.064 0.050 0.067 0.045 0.000 0.000

0.000 0.000 0.006 0.006 0.000 0.000 0.000 0.018 0.000 0.000

0.018 0.006 0.006 0.006 0.006 0.000 0.000 0.000 0.057 0.053

0.080 0.073

>Non-Methylation code ID 21135

0.012 0.000 0.000 0.000 0.012 0.000 0.006 0.000 0.000 0.006

0.000 0.006 0.006 0.006 0.006 0.000 0.012 0.000 0.000 0.000

0.054 0.081 0.069 0.059 0.005 0.000 0.005 0.000 0.000 0.000

0.000 0.000 0.000 0.005 0.000 0.000 0.016 0.000 0.011 0.016

0.011 0.000 0.000 0.000 0.075 0.064 0.059 0.064 0.006 0.000

0.006 0.000 0.006 0.000 0.006 0.000 0.000 0.011 0.000 0.000

0.017 0.000 0.011 0.000 0.006 0.000 0.000 0.000 0.055 0.081

0.060 0.071

>Non-Methylation code ID 21135

0.000 0.000 0.005 0.005 0.000 0.005 0.005 0.000 0.000 0.015

0.000 0.000 0.005 0.005 0.010 0.005 0.005 0.000 0.000 0.000

0.068 0.077 0.054 0.072 0.021 0.000 0.000 0.000 0.000 0.007

0.000 0.000 0.000 0.007 0.000 0.007 0.000 0.000 0.007 0.021

0.014 0.000 0.007 0.000 0.061 0.074 0.058 0.048 0.023 0.000

0.000 0.000 0.000 0.008 0.008 0.000 0.000 0.000 0.000 0.000

0.023 0.008 0.008 0.000 0.008 0.000 0.000 0.000 0.055 0.074

0.061 0.059

>Non-Methylation code ID 21135

0.000 0.006 0.006 0.006 0.006 0.000 0.006 0.000 0.000 0.032

0.000 0.000 0.000 0.000 0.000 0.013 0.000 0.000 0.000 0.006

0.079 0.050 0.066 0.055 0.008 0.008 0.000 0.000 0.008 0.000

0.000 0.000 0.000 0.016 0.000 0.008 0.000 0.000 0.000 0.024

0.024 0.000 0.008 0.000 0.066 0.045 0.061 0.056 0.006 0.000

0.000 0.000 0.006 0.006 0.000 0.000 0.000 0.025 0.000 0.000

0.019 0.000 0.006 0.006 0.000 0.000 0.000 0.000 0.059 0.048

0.082 0.070

>Non-Methylation code ID 21135

0.015 0.000 0.007 0.000 0.000 0.015 0.007 0.000 0.007 0.000

0.000 0.000 0.000 0.007 0.000 0.015 0.007 0.000 0.007 0.000

0.059 0.065 0.056 0.066 0.014 0.000 0.000 0.000 0.000 0.007

0.000 0.000 0.007 0.000 0.000 0.000 0.000 0.014 0.014 0.000

0.014 0.007 0.000 0.000 0.068 0.066 0.036 0.084 0.000 0.004

0.000 0.004 0.000 0.008 0.004 0.000 0.008 0.004 0.000 0.000

0.004 0.000 0.008 0.004 0.000 0.000 0.000 0.000 0.056 0.068

0.074 0.086

>Non-Methylation code ID 21135

0.006 0.000 0.006 0.000 0.000 0.006 0.000 0.000 0.000 0.006

0.000 0.000 0.018 0.006 0.006 0.018 0.006 0.000 0.000 0.000

0.067 0.061 0.048 0.078 0.012 0.000 0.000 0.000 0.006 0.000

0.018 0.000 0.006 0.012 0.000 0.000 0.018 0.000 0.000 0.000

0.006 0.000 0.000 0.000 0.069 0.073 0.047 0.064 0.000 0.000

0.000 0.000 0.010 0.000 0.005 0.000 0.000 0.005 0.000 0.000

0.014 0.000 0.010 0.000 0.010 0.000 0.000 0.000 0.064 0.070

0.075 0.071

>Non-Methylation code ID 21135

0.007 0.007 0.000 0.000 0.000 0.000 0.000 0.000 0.000 0.014

0.000 0.000 0.014 0.000 0.007 0.014 0.028 0.000 0.000 0.000

0.075 0.041 0.065 0.063 0.008 0.000 0.000 0.000 0.008 0.008

0.000 0.000 0.000 0.034 0.000 0.000 0.034 0.008 0.000 0.008

0.000 0.000 0.000 0.000 0.040 0.050 0.073 0.060 0.006 0.000

0.000 0.000 0.006 0.000 0.012 0.000 0.006 0.012 0.000 0.000

0.006 0.000 0.006 0.012 0.000 0.000 0.000 0.006 0.079 0.059

0.077 0.049

>Non-Methylation code ID 21135

0.005 0.005 0.000 0.000 0.000 0.000 0.000 0.000 0.000 0.005

0.000 0.000 0.020 0.000 0.010 0.005 0.015 0.000 0.000 0.000

0.072 0.057 0.070 0.070 0.014 0.000 0.000 0.000 0.000 0.007

0.000 0.000 0.000 0.021 0.000 0.000 0.028 0.007 0.007 0.007

0.000 0.000 0.000 0.000 0.064 0.055 0.049 0.074 0.011 0.000

0.000 0.000 0.000 0.000 0.011 0.000 0.005 0.011 0.000 0.000

0.016 0.000 0.005 0.000 0.000 0.000 0.000 0.005 0.070 0.067

0.078 0.055

>Non-Methylation code ID 21135

0.024 0.000 0.000 0.000 0.000 0.000 0.000 0.000 0.000 0.006

0.006 0.000 0.000 0.000 0.018 0.012 0.000 0.000 0.000 0.000

0.056 0.072 0.087 0.051 0.007 0.000 0.000 0.000 0.000 0.007

0.000 0.000 0.000 0.000 0.000 0.000 0.000 0.000 0.019 0.000

0.000 0.000 0.011 0.000 0.088 0.053 0.089 0.058 0.031 0.000

0.010 0.010 0.000 0.062 0.000 0.000 0.000 0.010 0.000 0.000

0.000 0.000 0.000 0.000 0.000 0.000 0.000 0.000 0.046 0.054

0.060 0.049

>Non-Methylation code ID 21135

0.000 0.000 0.005 0.005 0.000 0.010 0.005 0.000 0.000 0.010

0.000 0.000 0.005 0.005 0.010 0.005 0.005 0.000 0.000 0.000

0.073 0.078 0.054 0.061 0.032 0.000 0.000 0.000 0.000 0.008

0.000 0.000 0.000 0.008 0.000 0.008 0.000 0.000 0.008 0.024

0.016 0.000 0.000 0.000 0.060 0.077 0.052 0.039 0.020 0.000

0.000 0.000 0.000 0.000 0.007 0.000 0.000 0.000 0.000 0.000

0.020 0.007 0.013 0.000 0.007 0.000 0.000 0.000 0.055 0.059

0.069 0.076

>Non-Methylation code ID 21135

0.000 0.000 0.000 0.000 0.009 0.000 0.005 0.000 0.000 0.005

0.000 0.000 0.014 0.000 0.009 0.000 0.014 0.000 0.000 0.000

0.061 0.071 0.076 0.069 0.006 0.000 0.006 0.000 0.000 0.006

0.000 0.000 0.000 0.006 0.000 0.000 0.018 0.006 0.006 0.018

0.006 0.000 0.000 0.000 0.067 0.061 0.048 0.078 0.013 0.000

0.000 0.000 0.007 0.000 0.013 0.000 0.007 0.013 0.000 0.000

0.020 0.000 0.000 0.000 0.007 0.000 0.000 0.000 0.065 0.074

0.053 0.061

>Non-Methylation code ID 21135

0.023 0.006 0.000 0.000 0.000 0.000 0.012 0.000 0.000 0.000

0.000 0.000 0.012 0.012 0.000 0.000 0.006 0.000 0.006 0.000

0.062 0.056 0.055 0.085 0.004 0.000 0.000 0.000 0.000 0.004

0.004 0.000 0.000 0.000 0.000 0.000 0.013 0.000 0.017 0.004

0.008 0.000 0.000 0.000 0.083 0.056 0.070 0.069 0.011 0.000

0.000 0.000 0.000 0.011 0.006 0.000 0.000 0.006 0.000 0.000

0.011 0.000 0.011 0.000 0.006 0.000 0.000 0.000 0.060 0.063

0.073 0.075

>Non-Methylation code ID 21135

0.005 0.000 0.000 0.000 0.000 0.005 0.000 0.000 0.000 0.016

0.000 0.000 0.005 0.016 0.011 0.005 0.005 0.000 0.000 0.000

0.063 0.069 0.068 0.063 0.005 0.011 0.000 0.000 0.000 0.005

0.005 0.000 0.000 0.000 0.000 0.000 0.011 0.000 0.011 0.011

0.000 0.005 0.000 0.000 0.050 0.069 0.075 0.077 0.030 0.000

0.006 0.000 0.006 0.006 0.000 0.000 0.000 0.000 0.000 0.000

0.012 0.000 0.006 0.000 0.006 0.000 0.000 0.000 0.070 0.073

0.062 0.057

>Non-Methylation code ID 21135

0.015 0.000 0.000 0.005 0.005 0.000 0.000 0.000 0.000 0.000

0.000 0.000 0.015 0.005 0.010 0.005 0.005 0.000 0.000 0.000

0.060 0.064 0.075 0.069 0.005 0.000 0.000 0.000 0.000 0.005

0.000 0.000 0.000 0.016 0.000 0.000 0.016 0.005 0.011 0.011

0.000 0.000 0.000 0.000 0.063 0.069 0.075 0.057 0.007 0.014

0.000 0.000 0.007 0.007 0.007 0.000 0.000 0.000 0.000 0.000

0.027 0.000 0.000 0.007 0.000 0.007 0.000 0.000 0.071 0.070

0.055 0.056

>Non-Methylation code ID 21135

0.017 0.000 0.000 0.006 0.006 0.000 0.000 0.000 0.000 0.000

0.000 0.000 0.023 0.006 0.006 0.006 0.006 0.000 0.000 0.000

0.059 0.062 0.074 0.063 0.005 0.000 0.000 0.000 0.000 0.000

0.000 0.000 0.000 0.015 0.000 0.000 0.015 0.005 0.015 0.010

0.000 0.000 0.000 0.000 0.066 0.062 0.070 0.069 0.007 0.013

0.000 0.000 0.007 0.007 0.007 0.000 0.000 0.000 0.000 0.000

0.027 0.000 0.000 0.007 0.000 0.007 0.000 0.000 0.062 0.065

0.061 0.064

>Non-Methylation code ID 21135

0.005 0.011 0.000 0.000 0.000 0.005 0.005 0.000 0.000 0.000

0.000 0.000 0.011 0.000 0.011 0.011 0.000 0.005 0.000 0.000

0.050 0.069 0.075 0.077 0.028 0.000 0.006 0.000 0.006 0.006

0.000 0.000 0.000 0.000 0.000 0.000 0.017 0.000 0.006 0.000

0.006 0.000 0.000 0.000 0.068 0.068 0.069 0.055 0.006 0.000

0.000 0.000 0.000 0.006 0.000 0.000 0.000 0.017 0.000 0.000

0.006 0.011 0.011 0.006 0.006 0.000 0.000 0.000 0.066 0.069

0.072 0.060

>Non-Methylation code ID 21135

0.006 0.000 0.000 0.006 0.006 0.000 0.012 0.000 0.000 0.006

0.000 0.000 0.000 0.000 0.006 0.006 0.006 0.006 0.000 0.006

0.072 0.044 0.082 0.067 0.000 0.005 0.000 0.005 0.010 0.000

0.000 0.005 0.000 0.010 0.000 0.000 0.000 0.005 0.005 0.005

0.010 0.005 0.000 0.000 0.065 0.072 0.074 0.055 0.000 0.000

0.007 0.000 0.007 0.000 0.000 0.000 0.000 0.007 0.000 0.000

0.000 0.000 0.007 0.042 0.000 0.014 0.000 0.000 0.068 0.063

0.063 0.054

>Non-Methylation code ID 21135

0.013 0.000 0.000 0.000 0.000 0.006 0.000 0.000 0.000 0.006

0.000 0.000 0.006 0.006 0.019 0.019 0.006 0.000 0.000 0.000

0.069 0.072 0.052 0.060 0.005 0.000 0.000 0.000 0.000 0.015

0.000 0.000 0.000 0.005 0.000 0.000 0.010 0.010 0.010 0.000

0.000 0.000 0.005 0.000 0.069 0.073 0.063 0.069 0.010 0.010

0.000 0.010 0.000 0.000 0.000 0.000 0.000 0.005 0.000 0.005

0.010 0.000 0.005 0.000 0.000 0.005 0.000 0.000 0.072 0.071

0.049 0.079

>Non-Methylation code ID 21135

0.005 0.005 0.000 0.000 0.005 0.000 0.005 0.000 0.000 0.005

0.000 0.000 0.022 0.005 0.005 0.000 0.011 0.000 0.000 0.000

0.077 0.062 0.061 0.062 0.004 0.000 0.000 0.000 0.000 0.000

0.004 0.000 0.000 0.009 0.000 0.000 0.009 0.000 0.018 0.004

0.004 0.004 0.000 0.000 0.053 0.068 0.091 0.064 0.017 0.000

0.000 0.000 0.000 0.008 0.008 0.008 0.000 0.008 0.000 0.000

0.025 0.000 0.000 0.017 0.000 0.000 0.000 0.008 0.043 0.067

0.063 0.060

>Non-Methylation code ID 21135

0.005 0.000 0.000 0.000 0.000 0.000 0.005 0.000 0.000 0.010

0.000 0.000 0.010 0.000 0.019 0.005 0.005 0.005 0.000 0.000

0.069 0.061 0.087 0.054 0.015 0.000 0.000 0.000 0.000 0.015

0.015 0.007 0.000 0.007 0.000 0.000 0.015 0.000 0.000 0.015

0.000 0.000 0.000 0.007 0.051 0.063 0.069 0.056 0.006 0.006

0.000 0.000 0.006 0.006 0.006 0.000 0.000 0.000 0.000 0.000

0.026 0.006 0.000 0.000 0.013 0.000 0.000 0.000 0.074 0.064

0.066 0.052

>Non-Methylation code ID 21135

0.000 0.005 0.000 0.000 0.000 0.000 0.005 0.000 0.010 0.005

0.005 0.000 0.015 0.005 0.005 0.005 0.000 0.000 0.000 0.005

0.069 0.073 0.056 0.070 0.005 0.010 0.000 0.000 0.000 0.005

0.005 0.005 0.005 0.000 0.000 0.000 0.010 0.000 0.005 0.010

0.005 0.000 0.000 0.000 0.080 0.061 0.057 0.069 0.028 0.000

0.000 0.009 0.000 0.000 0.000 0.000 0.000 0.009 0.000 0.009

0.009 0.009 0.000 0.000 0.009 0.019 0.000 0.000 0.064 0.050

0.063 0.053

>Non-Methylation code ID 21135

0.005 0.000 0.000 0.005 0.010 0.000 0.000 0.000 0.000 0.000

0.000 0.000 0.005 0.000 0.014 0.005 0.005 0.000 0.005 0.000

0.075 0.052 0.074 0.080 0.010 0.000 0.000 0.000 0.000 0.030

0.010 0.000 0.000 0.010 0.000 0.000 0.010 0.000 0.000 0.050

0.000 0.010 0.000 0.000 0.036 0.051 0.054 0.061 0.000 0.008

0.000 0.008 0.000 0.008 0.000 0.008 0.000 0.008 0.000 0.000

0.016 0.008 0.000 0.000 0.000 0.024 0.000 0.008 0.058 0.050

0.054 0.076

>Non-Methylation code ID 21135

0.007 0.000 0.000 0.000 0.007 0.000 0.007 0.000 0.000 0.007

0.000 0.000 0.021 0.000 0.000 0.028 0.014 0.000 0.000 0.000

0.061 0.065 0.055 0.060 0.005 0.000 0.000 0.000 0.005 0.000

0.009 0.000 0.000 0.005 0.000 0.000 0.018 0.000 0.009 0.005

0.005 0.000 0.000 0.000 0.062 0.070 0.078 0.065 0.007 0.000

0.007 0.000 0.000 0.000 0.007 0.007 0.000 0.022 0.000 0.000

0.015 0.007 0.000 0.007 0.007 0.000 0.000 0.000 0.051 0.053

0.069 0.070

>Non-Methylation code ID 21135

0.015 0.000 0.000 0.000 0.000 0.022 0.000 0.000 0.000 0.022

0.000 0.000 0.007 0.000 0.007 0.007 0.007 0.000 0.000 0.000

0.051 0.060 0.064 0.071 0.004 0.000 0.000 0.009 0.000 0.000

0.000 0.000 0.000 0.004 0.000 0.000 0.004 0.000 0.013 0.009

0.000 0.000 0.009 0.000 0.093 0.040 0.086 0.062 0.016 0.000

0.000 0.000 0.000 0.016 0.000 0.000 0.000 0.008 0.000 0.000

0.016 0.000 0.000 0.016 0.000 0.008 0.008 0.008 0.066 0.037

0.068 0.064

>Non-Methylation code ID 21139

0.006 0.006 0.000 0.000 0.006 0.018 0.000 0.000 0.000 0.006

0.000 0.000 0.006 0.000 0.006 0.006 0.012 0.000 0.000 0.000

0.060 0.082 0.060 0.059 0.015 0.000 0.000 0.005 0.000 0.005

0.000 0.000 0.000 0.005 0.000 0.000 0.010 0.000 0.015 0.005

0.000 0.005 0.000 0.000 0.088 0.056 0.071 0.055 0.005 0.000

0.000 0.000 0.000 0.010 0.005 0.000 0.000 0.015 0.000 0.000

0.005 0.000 0.010 0.005 0.000 0.000 0.000 0.005 0.078 0.050

0.067 0.079

>Non-Methylation code ID 21139

0.004 0.000 0.004 0.000 0.000 0.012 0.000 0.000 0.000 0.004

0.000 0.000 0.008 0.004 0.012 0.000 0.000 0.000 0.004 0.000

0.069 0.071 0.068 0.075 0.013 0.007 0.000 0.013 0.000 0.013

0.000 0.000 0.000 0.007 0.000 0.007 0.013 0.000 0.000 0.000

0.007 0.007 0.000 0.000 0.054 0.070 0.058 0.066 0.005 0.000

0.000 0.000 0.000 0.005 0.000 0.000 0.000 0.005 0.000 0.000

0.010 0.005 0.016 0.010 0.005 0.000 0.000 0.000 0.061 0.073

0.071 0.066

>Non-Methylation code ID 21139

0.006 0.000 0.000 0.012 0.012 0.000 0.006 0.000 0.000 0.006

0.000 0.000 0.000 0.000 0.006 0.000 0.000 0.000 0.006 0.006

0.079 0.062 0.076 0.056 0.000 0.000 0.000 0.009 0.000 0.017

0.009 0.009 0.000 0.000 0.000 0.000 0.000 0.000 0.000 0.052

0.009 0.000 0.009 0.000 0.056 0.053 0.050 0.062 0.000 0.000

0.000 0.000 0.000 0.028 0.000 0.014 0.000 0.028 0.000 0.000

0.014 0.000 0.000 0.014 0.000 0.070 0.000 0.000 0.045 0.037

0.044 0.039

>Non-Methylation code ID 21139

0.015 0.000 0.000 0.000 0.000 0.007 0.000 0.000 0.000 0.007

0.000 0.000 0.037 0.000 0.007 0.015 0.000 0.007 0.000 0.000

0.042 0.063 0.063 0.069 0.011 0.000 0.000 0.000 0.005 0.000

0.000 0.000 0.000 0.011 0.000 0.000 0.022 0.011 0.005 0.005

0.000 0.000 0.000 0.000 0.069 0.074 0.062 0.057 0.005 0.000

0.000 0.000 0.000 0.000 0.000 0.000 0.000 0.011 0.000 0.000

0.011 0.000 0.016 0.022 0.000 0.000 0.000 0.000 0.051 0.069

0.082 0.066

>Non-Methylation code ID 21139

0.004 0.000 0.000 0.000 0.004 0.000 0.009 0.000 0.000 0.004

0.000 0.000 0.013 0.000 0.013 0.004 0.004 0.000 0.000 0.000

0.069 0.060 0.071 0.078 0.014 0.000 0.007 0.000 0.000 0.000

0.007 0.007 0.000 0.014 0.000 0.000 0.014 0.007 0.000 0.014

0.007 0.000 0.000 0.000 0.051 0.054 0.070 0.068 0.000 0.000

0.000 0.000 0.008 0.000 0.008 0.000 0.000 0.008 0.000 0.000

0.039 0.000 0.000 0.016 0.016 0.000 0.000 0.000 0.061 0.067

0.060 0.052

>Non-Methylation code ID 21139

0.005 0.000 0.000 0.000 0.000 0.000 0.005 0.000 0.000 0.009

0.000 0.000 0.009 0.000 0.014 0.014 0.005 0.000 0.000 0.000

0.038 0.063 0.086 0.087 0.016 0.000 0.000 0.000 0.000 0.008

0.008 0.008 0.000 0.008 0.000 0.000 0.032 0.000 0.000 0.016

0.000 0.008 0.000 0.000 0.040 0.067 0.069 0.054 0.006 0.000

0.000 0.000 0.006 0.000 0.006 0.000 0.000 0.006 0.000 0.000

0.024 0.006 0.006 0.006 0.006 0.000 0.000 0.000 0.083 0.063

0.062 0.054

>Non-Methylation code ID 21139

0.004 0.000 0.004 0.000 0.000 0.008 0.000 0.000 0.000 0.008

0.000 0.000 0.012 0.000 0.008 0.000 0.004 0.000 0.004 0.000

0.067 0.069 0.074 0.073 0.006 0.006 0.000 0.012 0.000 0.012

0.000 0.000 0.000 0.006 0.000 0.000 0.012 0.000 0.012 0.000

0.006 0.000 0.000 0.000 0.043 0.068 0.065 0.086 0.016 0.000

0.005 0.000 0.000 0.005 0.000 0.000 0.000 0.005 0.000 0.000

0.005 0.005 0.016 0.005 0.000 0.000 0.000 0.000 0.062 0.078

0.067 0.063

>Non-Methylation code ID 21139

0.004 0.000 0.000 0.009 0.000 0.013 0.000 0.000 0.000 0.009

0.000 0.000 0.004 0.000 0.013 0.000 0.004 0.000 0.000 0.000

0.091 0.044 0.070 0.071 0.009 0.000 0.004 0.000 0.000 0.013

0.000 0.000 0.000 0.004 0.000 0.000 0.000 0.000 0.017 0.004

0.000 0.000 0.000 0.000 0.048 0.076 0.086 0.071 0.009 0.000

0.004 0.004 0.000 0.013 0.000 0.000 0.000 0.000 0.000 0.000

0.000 0.000 0.013 0.004 0.004 0.000 0.000 0.000 0.068 0.060

0.070 0.081

>Non-Methylation code ID 21139

0.000 0.010 0.000 0.000 0.000 0.005 0.005 0.005 0.005 0.000

0.000 0.000 0.010 0.000 0.005 0.010 0.005 0.005 0.000 0.000

0.084 0.059 0.068 0.058 0.026 0.000 0.000 0.009 0.000 0.000

0.000 0.000 0.000 0.009 0.000 0.009 0.009 0.009 0.000 0.009

0.009 0.017 0.000 0.000 0.065 0.049 0.060 0.054 0.000 0.000

0.000 0.000 0.000 0.000 0.005 0.000 0.011 0.005 0.005 0.000

0.016 0.005 0.005 0.005 0.000 0.000 0.000 0.005 0.068 0.072

0.061 0.068

>Non-Methylation code ID 21139

0.020 0.000 0.000 0.000 0.000 0.020 0.000 0.000 0.000 0.039

0.000 0.000 0.000 0.029 0.000 0.000 0.000 0.000 0.010 0.000

0.040 0.049 0.060 0.067 0.000 0.000 0.000 0.010 0.000 0.005

0.000 0.000 0.000 0.000 0.000 0.005 0.000 0.000 0.014 0.019

0.000 0.000 0.010 0.000 0.068 0.061 0.065 0.077 0.032 0.000

0.000 0.000 0.000 0.032 0.000 0.000 0.000 0.011 0.011 0.000

0.021 0.000 0.000 0.021 0.000 0.000 0.000 0.000 0.039 0.048

0.064 0.056

>Non-Methylation code ID 21139

0.028 0.000 0.000 0.000 0.000 0.009 0.000 0.000 0.000 0.009

0.000 0.000 0.046 0.000 0.000 0.018 0.000 0.009 0.000 0.000

0.031 0.055 0.067 0.061 0.005 0.000 0.000 0.000 0.005 0.000

0.005 0.000 0.000 0.010 0.000 0.000 0.021 0.010 0.005 0.005

0.000 0.000 0.000 0.000 0.072 0.071 0.064 0.058 0.005 0.000

0.000 0.000 0.000 0.000 0.000 0.000 0.000 0.011 0.000 0.000

0.005 0.000 0.022 0.022 0.000 0.000 0.000 0.000 0.047 0.064

0.077 0.080

>Non-Methylation code ID 21139

0.006 0.000 0.000 0.000 0.000 0.000 0.000 0.000 0.000 0.013

0.000 0.000 0.026 0.000 0.006 0.026 0.006 0.000 0.000 0.000

0.055 0.061 0.074 0.060 0.007 0.000 0.000 0.000 0.000 0.000

0.007 0.000 0.000 0.013 0.000 0.000 0.033 0.000 0.007 0.013

0.000 0.007 0.000 0.000 0.058 0.062 0.062 0.067 0.006 0.000

0.000 0.000 0.006 0.000 0.000 0.000 0.000 0.013 0.000 0.000

0.019 0.006 0.006 0.013 0.006 0.000 0.000 0.000 0.067 0.069

0.075 0.045

>Non-Methylation code ID 21139

0.000 0.004 0.000 0.000 0.009 0.004 0.000 0.000 0.000 0.004

0.000 0.000 0.004 0.004 0.009 0.004 0.013 0.000 0.000 0.000

0.058 0.072 0.080 0.066 0.020 0.000 0.000 0.000 0.007 0.000

0.000 0.000 0.000 0.000 0.000 0.000 0.013 0.007 0.007 0.020

0.000 0.013 0.000 0.000 0.057 0.062 0.060 0.067 0.006 0.000

0.000 0.000 0.006 0.000 0.006 0.000 0.000 0.012 0.000 0.000

0.024 0.006 0.006 0.000 0.000 0.000 0.000 0.006 0.063 0.057

0.063 0.080

>Non-Methylation code ID 21139

0.024 0.000 0.012 0.000 0.000 0.000 0.000 0.012 0.000 0.012

0.000 0.000 0.000 0.012 0.000 0.072 0.000 0.000 0.000 0.000

0.045 0.059 0.032 0.052 0.009 0.000 0.005 0.000 0.000 0.000

0.014 0.000 0.000 0.000 0.000 0.000 0.009 0.000 0.009 0.000

0.005 0.000 0.000 0.005 0.070 0.073 0.066 0.069 0.000 0.000

0.006 0.000 0.000 0.006 0.000 0.018 0.006 0.018 0.000 0.000

0.000 0.006 0.006 0.000 0.006 0.000 0.000 0.000 0.051 0.067

0.079 0.065

>Non-Methylation code ID 21139

0.000 0.000 0.000 0.000 0.000 0.005 0.000 0.005 0.005 0.020

0.000 0.000 0.005 0.000 0.015 0.010 0.000 0.000 0.000 0.000

0.077 0.082 0.049 0.060 0.000 0.006 0.006 0.006 0.000 0.011

0.000 0.000 0.000 0.000 0.000 0.006 0.006 0.006 0.006 0.011

0.000 0.000 0.000 0.006 0.073 0.061 0.070 0.062 0.014 0.000

0.007 0.007 0.000 0.000 0.000 0.007 0.014 0.014 0.000 0.000

0.000 0.000 0.000 0.000 0.014 0.007 0.000 0.000 0.073 0.065

0.035 0.079

>Non-Methylation code ID 21139

0.005 0.009 0.005 0.000 0.000 0.000 0.000 0.000 0.000 0.000

0.000 0.000 0.009 0.000 0.014 0.014 0.005 0.000 0.000 0.000

0.060 0.053 0.079 0.081 0.008 0.000 0.004 0.000 0.000 0.008

0.004 0.004 0.000 0.004 0.000 0.000 0.000 0.004 0.012 0.000

0.000 0.004 0.000 0.000 0.051 0.070 0.071 0.090 0.009 0.000

0.000 0.009 0.000 0.018 0.000 0.000 0.000 0.027 0.000 0.009

0.009 0.000 0.000 0.000 0.018 0.009 0.000 0.000 0.055 0.067

0.055 0.049

>Non-Methylation code ID 21139

0.000 0.000 0.006 0.006 0.000 0.013 0.000 0.000 0.019 0.006

0.006 0.013 0.000 0.000 0.000 0.000 0.013 0.000 0.000 0.000

0.063 0.062 0.057 0.070 0.005 0.005 0.000 0.000 0.000 0.005

0.000 0.009 0.014 0.005 0.000 0.000 0.000 0.000 0.009 0.000

0.005 0.000 0.000 0.000 0.065 0.094 0.057 0.061 0.000 0.000

0.000 0.011 0.000 0.011 0.006 0.000 0.006 0.000 0.000 0.006

0.000 0.000 0.011 0.000 0.000 0.006 0.000 0.006 0.073 0.080

0.048 0.072

>Non-Methylation code ID 21139

0.000 0.000 0.000 0.000 0.000 0.005 0.000 0.005 0.005 0.016

0.000 0.000 0.005 0.000 0.016 0.016 0.000 0.000 0.000 0.000

0.068 0.085 0.065 0.047 0.000 0.005 0.000 0.005 0.000 0.010

0.000 0.000 0.000 0.005 0.000 0.005 0.000 0.005 0.010 0.010

0.000 0.005 0.000 0.005 0.082 0.069 0.051 0.066 0.012 0.000

0.006 0.000 0.012 0.006 0.000 0.000 0.012 0.012 0.000 0.000

0.000 0.000 0.000 0.000 0.012 0.000 0.000 0.000 0.059 0.074

0.051 0.074

>Non-Methylation code ID 21139

0.008 0.000 0.000 0.000 0.000 0.015 0.000 0.000 0.000 0.000

0.000 0.015 0.000 0.008 0.008 0.000 0.015 0.008 0.000 0.008

0.073 0.084 0.059 0.034 0.010 0.000 0.005 0.000 0.000 0.000

0.005 0.000 0.000 0.000 0.000 0.005 0.005 0.000 0.010 0.010

0.015 0.000 0.000 0.000 0.067 0.056 0.077 0.067 0.005 0.000

0.000 0.005 0.000 0.005 0.009 0.014 0.000 0.000 0.000 0.000

0.005 0.005 0.009 0.000 0.000 0.000 0.000 0.000 0.050 0.073

0.082 0.072

>Non-Methylation code ID 21139

0.005 0.005 0.000 0.000 0.000 0.010 0.000 0.000 0.024 0.000

0.000 0.010 0.000 0.000 0.010 0.000 0.000 0.000 0.000 0.000

0.074 0.063 0.073 0.061 0.016 0.000 0.000 0.000 0.000 0.011

0.000 0.005 0.011 0.000 0.000 0.005 0.000 0.005 0.011 0.000

0.005 0.000 0.000 0.000 0.079 0.084 0.056 0.045 0.000 0.000

0.000 0.012 0.000 0.012 0.000 0.000 0.012 0.000 0.006 0.000

0.006 0.012 0.006 0.000 0.000 0.000 0.000 0.000 0.063 0.081

0.067 0.053

>Non-Methylation code ID 21139

0.000 0.000 0.000 0.000 0.000 0.006 0.000 0.011 0.011 0.011

0.000 0.000 0.000 0.000 0.011 0.011 0.006 0.000 0.000 0.000

0.062 0.069 0.056 0.080 0.006 0.006 0.000 0.012 0.000 0.000

0.000 0.000 0.006 0.018 0.000 0.006 0.000 0.000 0.000 0.012

0.000 0.006 0.000 0.000 0.078 0.064 0.063 0.055 0.006 0.000

0.000 0.000 0.011 0.000 0.011 0.000 0.011 0.000 0.000 0.006

0.000 0.006 0.000 0.006 0.006 0.000 0.006 0.000 0.070 0.073

0.049 0.073

>Non-Methylation code ID 21139

0.000 0.007 0.007 0.000 0.007 0.000 0.007 0.015 0.000 0.007

0.000 0.000 0.000 0.007 0.000 0.015 0.015 0.007 0.000 0.000

0.064 0.060 0.061 0.051 0.015 0.000 0.000 0.000 0.000 0.000

0.000 0.000 0.000 0.000 0.007 0.007 0.015 0.000 0.007 0.022

0.007 0.000 0.000 0.000 0.072 0.072 0.060 0.048 0.000 0.007

0.007 0.000 0.000 0.000 0.014 0.000 0.000 0.014 0.000 0.007

0.014 0.007 0.000 0.000 0.007 0.000 0.000 0.000 0.078 0.054

0.047 0.080

>Non-Methylation code ID 21139

0.005 0.014 0.000 0.000 0.000 0.000 0.000 0.000 0.000 0.000

0.005 0.000 0.009 0.000 0.009 0.014 0.005 0.000 0.000 0.000

0.060 0.068 0.085 0.060 0.008 0.004 0.004 0.000 0.000 0.004

0.004 0.000 0.000 0.004 0.000 0.000 0.000 0.004 0.012 0.000

0.000 0.008 0.000 0.000 0.054 0.068 0.079 0.082 0.017 0.000

0.000 0.008 0.000 0.008 0.000 0.000 0.000 0.025 0.000 0.008

0.008 0.000 0.000 0.000 0.008 0.008 0.000 0.008 0.053 0.065

0.061 0.052

>Non-Methylation code ID 21139

0.000 0.000 0.006 0.006 0.000 0.012 0.000 0.000 0.018 0.006

0.006 0.012 0.000 0.000 0.006 0.000 0.006 0.000 0.000 0.000

0.057 0.058 0.072 0.067 0.010 0.005 0.000 0.000 0.000 0.005

0.000 0.010 0.014 0.000 0.000 0.000 0.000 0.000 0.010 0.000

0.005 0.000 0.000 0.000 0.076 0.100 0.051 0.049 0.000 0.000

0.000 0.011 0.000 0.011 0.006 0.000 0.006 0.000 0.000 0.006

0.000 0.006 0.011 0.000 0.000 0.006 0.000 0.000 0.071 0.086

0.044 0.069

>Non-Methylation code ID 21139

0.000 0.000 0.000 0.005 0.000 0.011 0.000 0.000 0.000 0.005

0.000 0.011 0.000 0.005 0.011 0.011 0.000 0.005 0.000 0.005

0.075 0.070 0.049 0.068 0.012 0.000 0.006 0.000 0.012 0.006

0.000 0.000 0.012 0.012 0.000 0.000 0.000 0.000 0.000 0.000

0.018 0.000 0.000 0.000 0.061 0.075 0.050 0.071 0.000 0.000

0.000 0.000 0.000 0.006 0.000 0.006 0.006 0.011 0.000 0.000

0.006 0.000 0.017 0.017 0.000 0.000 0.000 0.000 0.069 0.093

0.072 0.033

>Non-Methylation code ID 21139

0.006 0.000 0.000 0.000 0.011 0.006 0.011 0.000 0.017 0.006

0.000 0.000 0.000 0.000 0.000 0.000 0.011 0.000 0.006 0.000

0.062 0.078 0.051 0.070 0.000 0.000 0.000 0.000 0.000 0.010

0.000 0.010 0.010 0.005 0.000 0.000 0.000 0.000 0.015 0.015

0.000 0.000 0.000 0.000 0.054 0.092 0.079 0.044 0.000 0.000

0.000 0.012 0.000 0.006 0.000 0.000 0.000 0.012 0.000 0.012

0.000 0.006 0.006 0.012 0.000 0.006 0.000 0.000 0.080 0.074

0.039 0.067

>Non-Methylation code ID 21139

0.008 0.004 0.004 0.004 0.000 0.004 0.000 0.000 0.000 0.004

0.000 0.004 0.000 0.000 0.008 0.004 0.000 0.008 0.000 0.000

0.061 0.063 0.078 0.078 0.014 0.000 0.000 0.007 0.000 0.007

0.000 0.007 0.000 0.021 0.000 0.000 0.007 0.000 0.007 0.000

0.007 0.000 0.000 0.007 0.059 0.060 0.065 0.064 0.000 0.015

0.000 0.005 0.000 0.000 0.000 0.000 0.000 0.000 0.005 0.000

0.010 0.000 0.010 0.010 0.000 0.000 0.000 0.000 0.066 0.064

0.088 0.061

>Non-Methylation code ID 21139

0.000 0.000 0.000 0.000 0.000 0.005 0.000 0.005 0.005 0.005

0.000 0.000 0.005 0.000 0.028 0.005 0.005 0.000 0.000 0.000

0.092 0.040 0.093 0.046 0.008 0.000 0.000 0.000 0.000 0.055

0.000 0.000 0.000 0.000 0.000 0.000 0.016 0.000 0.008 0.016

0.000 0.000 0.000 0.000 0.065 0.072 0.045 0.049 0.018 0.000

0.012 0.012 0.000 0.000 0.000 0.000 0.000 0.000 0.000 0.000

0.006 0.018 0.006 0.000 0.000 0.000 0.000 0.000 0.062 0.066

0.056 0.079

>Non-Methylation code ID 21139

0.005 0.000 0.000 0.000 0.000 0.005 0.000 0.005 0.005 0.005

0.000 0.000 0.005 0.000 0.019 0.005 0.005 0.000 0.000 0.000

0.107 0.023 0.116 0.029 0.006 0.000 0.006 0.000 0.000 0.024

0.006 0.000 0.000 0.000 0.000 0.000 0.012 0.000 0.012 0.012

0.000 0.000 0.000 0.000 0.062 0.045 0.073 0.074 0.013 0.000

0.013 0.007 0.000 0.007 0.000 0.007 0.000 0.000 0.000 0.000

0.007 0.020 0.007 0.000 0.000 0.000 0.000 0.000 0.081 0.066

0.055 0.052

>Non-Methylation code ID 21139

0.000 0.000 0.004 0.000 0.000 0.021 0.004 0.000 0.000 0.000

0.000 0.004 0.000 0.004 0.017 0.000 0.000 0.000 0.000 0.000

0.083 0.056 0.086 0.053 0.000 0.000 0.014 0.027 0.000 0.014

0.000 0.007 0.007 0.000 0.007 0.000 0.000 0.000 0.000 0.000

0.007 0.000 0.000 0.000 0.051 0.053 0.075 0.072 0.000 0.000

0.000 0.000 0.000 0.010 0.000 0.000 0.010 0.000 0.000 0.000

0.005 0.000 0.010 0.000 0.010 0.000 0.005 0.000 0.054 0.072

0.070 0.087

>Non-Methylation code ID 21139

0.000 0.000 0.037 0.037 0.000 0.009 0.000 0.009 0.000 0.000

0.009 0.000 0.000 0.000 0.000 0.000 0.009 0.000 0.000 0.000

0.050 0.045 0.075 0.052 0.000 0.000 0.000 0.000 0.000 0.006

0.000 0.000 0.006 0.000 0.000 0.000 0.006 0.000 0.017 0.000

0.023 0.000 0.006 0.000 0.081 0.069 0.060 0.060 0.000 0.000

0.005 0.000 0.000 0.024 0.005 0.000 0.000 0.000 0.000 0.005

0.000 0.000 0.019 0.000 0.000 0.000 0.000 0.000 0.118 0.018

0.112 0.029

>Non-Methylation code ID 21139

0.000 0.000 0.000 0.000 0.000 0.005 0.000 0.005 0.005 0.005

0.000 0.000 0.005 0.000 0.030 0.005 0.005 0.000 0.000 0.000

0.083 0.042 0.084 0.060 0.007 0.000 0.000 0.000 0.000 0.051

0.000 0.000 0.000 0.000 0.000 0.000 0.014 0.000 0.007 0.014

0.000 0.000 0.000 0.000 0.058 0.066 0.063 0.053 0.018 0.000

0.012 0.018 0.000 0.000 0.000 0.000 0.000 0.000 0.000 0.000

0.006 0.012 0.006 0.000 0.000 0.000 0.000 0.000 0.065 0.067

0.052 0.077

>Non-Methylation code ID 21139

0.000 0.000 0.000 0.000 0.000 0.017 0.000 0.000 0.006 0.000

0.000 0.000 0.011 0.011 0.017 0.011 0.000 0.000 0.000 0.000

0.054 0.074 0.071 0.062 0.011 0.000 0.011 0.006 0.000 0.000

0.000 0.000 0.011 0.006 0.000 0.006 0.011 0.000 0.000 0.000

0.000 0.006 0.000 0.000 0.064 0.068 0.060 0.072 0.000 0.000

0.000 0.000 0.005 0.000 0.000 0.000 0.010 0.000 0.000 0.000

0.005 0.005 0.010 0.005 0.015 0.000 0.005 0.000 0.059 0.083

0.073 0.057

>Non-Methylation code ID 21139

0.000 0.000 0.013 0.006 0.000 0.006 0.000 0.006 0.006 0.000

0.006 0.006 0.006 0.006 0.000 0.006 0.000 0.006 0.000 0.000

0.068 0.060 0.074 0.054 0.000 0.005 0.000 0.005 0.005 0.000

0.000 0.000 0.009 0.000 0.000 0.000 0.009 0.000 0.005 0.000

0.014 0.000 0.005 0.000 0.067 0.072 0.071 0.067 0.000 0.000

0.005 0.000 0.000 0.005 0.005 0.000 0.005 0.000 0.000 0.000

0.005 0.005 0.023 0.000 0.000 0.005 0.000 0.000 0.061 0.085

0.086 0.047

>Non-Methylation code ID 21139

0.006 0.000 0.006 0.006 0.000 0.006 0.000 0.006 0.006 0.000

0.006 0.006 0.012 0.000 0.000 0.006 0.000 0.006 0.000 0.000

0.058 0.062 0.081 0.058 0.000 0.005 0.000 0.005 0.005 0.000

0.000 0.000 0.005 0.000 0.000 0.000 0.009 0.000 0.014 0.000

0.009 0.000 0.005 0.000 0.067 0.089 0.061 0.060 0.000 0.000

0.005 0.000 0.000 0.010 0.000 0.000 0.005 0.000 0.000 0.000

0.005 0.005 0.019 0.005 0.000 0.005 0.000 0.000 0.060 0.102

0.079 0.035

>Non-Methylation code ID 21139

0.005 0.000 0.005 0.000 0.000 0.000 0.000 0.005 0.005 0.011

0.000 0.000 0.011 0.005 0.005 0.000 0.000 0.000 0.005 0.000

0.075 0.071 0.064 0.063 0.000 0.000 0.000 0.000 0.000 0.012

0.000 0.000 0.006 0.012 0.000 0.006 0.000 0.000 0.012 0.012

0.006 0.000 0.000 0.012 0.054 0.065 0.059 0.077 0.000 0.000

0.000 0.006 0.006 0.006 0.000 0.000 0.000 0.000 0.006 0.000

0.012 0.000 0.006 0.000 0.012 0.012 0.000 0.000 0.062 0.074

0.067 0.065

>Non-Methylation code ID 21148

0.000 0.000 0.000 0.000 0.000 0.005 0.000 0.000 0.000 0.005

0.000 0.005 0.015 0.000 0.005 0.005 0.015 0.000 0.010 0.000

0.067 0.082 0.078 0.043 0.008 0.000 0.000 0.000 0.008 0.024

0.000 0.000 0.000 0.008 0.008 0.000 0.040 0.008 0.000 0.000

0.000 0.000 0.000 0.000 0.084 0.028 0.073 0.046 0.006 0.000

0.006 0.006 0.000 0.000 0.006 0.000 0.000 0.012 0.000 0.006

0.018 0.000 0.000 0.006 0.000 0.000 0.006 0.000 0.075 0.060

0.070 0.058

>Non-Methylation code ID 21148

0.000 0.000 0.000 0.010 0.000 0.000 0.000 0.010 0.000 0.000

0.010 0.000 0.010 0.000 0.000 0.049 0.020 0.000 0.000 0.020

0.055 0.055 0.043 0.053 0.000 0.005 0.000 0.000 0.000 0.000

0.000 0.005 0.000 0.014 0.000 0.000 0.014 0.005 0.009 0.005

0.005 0.000 0.000 0.000 0.079 0.065 0.065 0.064 0.000 0.000

0.000 0.000 0.008 0.000 0.024 0.000 0.000 0.008 0.000 0.008

0.000 0.000 0.000 0.008 0.000 0.032 0.000 0.000 0.050 0.078

0.070 0.049

>Non-Methylation code ID 21148

0.010 0.000 0.005 0.000 0.000 0.000 0.005 0.000 0.005 0.010

0.000 0.005 0.010 0.000 0.016 0.000 0.000 0.000 0.000 0.000

0.053 0.075 0.065 0.073 0.000 0.000 0.005 0.005 0.000 0.000

0.000 0.000 0.000 0.000 0.000 0.000 0.014 0.000 0.009 0.000

0.014 0.005 0.009 0.000 0.074 0.073 0.073 0.055 0.000 0.000

0.000 0.000 0.000 0.027 0.000 0.000 0.009 0.000 0.000 0.000

0.045 0.009 0.000 0.000 0.009 0.000 0.000 0.000 0.076 0.038

0.085 0.036

>Non-Methylation code ID 21148

0.008 0.000 0.000 0.000 0.008 0.023 0.000 0.000 0.008 0.000

0.000 0.000 0.000 0.000 0.000 0.008 0.008 0.016 0.008 0.008

0.053 0.061 0.076 0.051 0.000 0.006 0.000 0.006 0.000 0.006

0.000 0.000 0.000 0.006 0.006 0.000 0.006 0.000 0.006 0.012

0.000 0.012 0.000 0.006 0.054 0.074 0.065 0.067 0.000 0.004

0.000 0.000 0.000 0.004 0.000 0.004 0.000 0.004 0.000 0.000

0.000 0.000 0.017 0.000 0.000 0.004 0.004 0.000 0.073 0.102

0.051 0.064

>Non-Methylation code ID 21148

0.000 0.000 0.005 0.000 0.000 0.005 0.000 0.000 0.000 0.000

0.000 0.005 0.014 0.000 0.005 0.000 0.014 0.005 0.009 0.000

0.072 0.078 0.071 0.051 0.009 0.000 0.000 0.000 0.000 0.028

0.000 0.000 0.000 0.000 0.009 0.000 0.047 0.009 0.000 0.000

0.009 0.000 0.000 0.000 0.081 0.031 0.082 0.026 0.011 0.000

0.005 0.000 0.000 0.000 0.005 0.000 0.000 0.011 0.000 0.005

0.011 0.000 0.011 0.000 0.000 0.000 0.005 0.000 0.064 0.080

0.066 0.060

>Non-Methylation code ID 21148

0.010 0.000 0.005 0.000 0.000 0.000 0.005 0.000 0.000 0.010

0.000 0.005 0.010 0.000 0.014 0.000 0.000 0.000 0.005 0.000

0.057 0.071 0.074 0.069 0.000 0.000 0.005 0.005 0.000 0.005

0.000 0.000 0.000 0.000 0.000 0.000 0.014 0.000 0.005 0.000

0.014 0.005 0.009 0.000 0.073 0.073 0.067 0.059 0.011 0.000

0.000 0.000 0.000 0.032 0.000 0.000 0.000 0.000 0.000 0.000

0.054 0.011 0.000 0.000 0.011 0.000 0.000 0.000 0.088 0.024

0.089 0.015

>Non-Methylation code ID 21148

0.004 0.009 0.000 0.000 0.000 0.000 0.000 0.000 0.004 0.004

0.000 0.004 0.009 0.004 0.004 0.004 0.004 0.000 0.000 0.004

0.061 0.056 0.082 0.078 0.011 0.000 0.000 0.000 0.000 0.006

0.000 0.006 0.000 0.011 0.000 0.006 0.006 0.006 0.011 0.000

0.006 0.000 0.000 0.006 0.059 0.059 0.067 0.076 0.000 0.000

0.000 0.016 0.008 0.008 0.008 0.000 0.000 0.024 0.000 0.000

0.008 0.000 0.000 0.000 0.016 0.000 0.000 0.000 0.051 0.059

0.070 0.065

>Non-Methylation code ID 21148

0.005 0.010 0.000 0.000 0.000 0.000 0.000 0.000 0.005 0.000

0.005 0.005 0.005 0.000 0.005 0.015 0.005 0.000 0.000 0.005

0.056 0.058 0.066 0.088 0.010 0.005 0.000 0.000 0.000 0.005

0.000 0.005 0.000 0.010 0.000 0.000 0.005 0.005 0.015 0.000

0.005 0.000 0.000 0.000 0.069 0.049 0.070 0.080 0.000 0.000

0.000 0.014 0.007 0.007 0.007 0.000 0.000 0.021 0.000 0.007

0.007 0.000 0.000 0.000 0.000 0.014 0.000 0.000 0.052 0.070

0.070 0.059

>Non-Methylation code ID 21148

0.000 0.000 0.000 0.014 0.007 0.007 0.000 0.000 0.000 0.021

0.000 0.000 0.021 0.000 0.000 0.000 0.014 0.000 0.000 0.000

0.050 0.068 0.058 0.072 0.004 0.009 0.000 0.000 0.000 0.000

0.000 0.000 0.004 0.009 0.000 0.004 0.013 0.004 0.004 0.000

0.000 0.000 0.000 0.004 0.066 0.052 0.075 0.081 0.011 0.000

0.000 0.000 0.000 0.006 0.000 0.000 0.000 0.017 0.000 0.006

0.000 0.000 0.011 0.006 0.006 0.000 0.000 0.006 0.053 0.063

0.061 0.090

>Non-Methylation code ID 21148

0.000 0.000 0.000 0.011 0.006 0.006 0.000 0.000 0.000 0.000

0.006 0.000 0.011 0.000 0.006 0.000 0.011 0.011 0.000 0.000

0.063 0.071 0.064 0.069 0.005 0.000 0.005 0.000 0.000 0.000

0.000 0.000 0.005 0.010 0.000 0.000 0.010 0.005 0.010 0.000

0.000 0.000 0.005 0.000 0.084 0.054 0.068 0.069 0.000 0.000

0.000 0.000 0.000 0.014 0.000 0.000 0.007 0.014 0.000 0.007

0.000 0.000 0.014 0.014 0.007 0.000 0.000 0.007 0.052 0.063

0.061 0.076

>Non-Methylation code ID 21148

0.000 0.000 0.000 0.000 0.000 0.005 0.000 0.000 0.000 0.005

0.000 0.005 0.014 0.000 0.005 0.000 0.014 0.000 0.014 0.000

0.071 0.078 0.074 0.050 0.008 0.000 0.000 0.000 0.008 0.033

0.000 0.000 0.000 0.000 0.008 0.000 0.041 0.008 0.000 0.000

0.000 0.000 0.000 0.000 0.079 0.027 0.079 0.041 0.012 0.000

0.006 0.006 0.000 0.000 0.006 0.000 0.000 0.018 0.000 0.006

0.012 0.000 0.000 0.000 0.000 0.000 0.006 0.000 0.074 0.067

0.066 0.054

>Non-Methylation code ID 21148

0.005 0.000 0.000 0.000 0.000 0.005 0.000 0.000 0.005 0.011

0.000 0.005 0.000 0.000 0.011 0.011 0.011 0.000 0.000 0.005

0.056 0.063 0.064 0.081 0.000 0.000 0.000 0.012 0.006 0.006

0.000 0.000 0.000 0.006 0.000 0.000 0.018 0.000 0.006 0.000

0.012 0.006 0.000 0.000 0.067 0.071 0.065 0.060 0.006 0.006

0.006 0.000 0.000 0.000 0.000 0.000 0.006 0.011 0.000 0.006

0.011 0.006 0.006 0.000 0.000 0.000 0.000 0.000 0.080 0.056

0.062 0.073

>Non-Methylation code ID 21148

0.004 0.009 0.000 0.000 0.000 0.000 0.000 0.000 0.004 0.000

0.004 0.004 0.009 0.004 0.004 0.004 0.004 0.000 0.000 0.004

0.060 0.054 0.075 0.087 0.011 0.006 0.000 0.000 0.000 0.006

0.000 0.006 0.000 0.011 0.000 0.006 0.006 0.006 0.011 0.000

0.006 0.000 0.000 0.000 0.063 0.059 0.064 0.073 0.000 0.000

0.000 0.015 0.008 0.008 0.008 0.000 0.000 0.023 0.000 0.008

0.008 0.000 0.000 0.000 0.008 0.000 0.000 0.000 0.052 0.064

0.069 0.065

>Non-Methylation code ID 21148

0.000 0.004 0.004 0.000 0.000 0.004 0.000 0.000 0.004 0.004

0.000 0.004 0.000 0.000 0.019 0.000 0.000 0.004 0.000 0.004

0.055 0.090 0.081 0.056 0.007 0.000 0.007 0.007 0.007 0.013

0.000 0.000 0.000 0.000 0.000 0.000 0.000 0.000 0.007 0.000

0.020 0.007 0.007 0.000 0.050 0.074 0.071 0.059 0.000 0.007

0.000 0.007 0.000 0.014 0.000 0.000 0.007 0.014 0.000 0.000

0.014 0.000 0.000 0.007 0.007 0.000 0.000 0.000 0.049 0.070

0.068 0.071

>Non-Methylation code ID 21148

0.000 0.000 0.000 0.006 0.000 0.006 0.000 0.006 0.000 0.006

0.000 0.006 0.012 0.000 0.000 0.012 0.006 0.000 0.012 0.006

0.061 0.076 0.070 0.049 0.006 0.000 0.000 0.000 0.006 0.011

0.000 0.000 0.000 0.011 0.006 0.000 0.017 0.000 0.006 0.006

0.006 0.000 0.000 0.000 0.070 0.055 0.067 0.070 0.000 0.000

0.000 0.008 0.000 0.000 0.015 0.000 0.000 0.015 0.000 0.000

0.015 0.000 0.000 0.008 0.000 0.015 0.008 0.000 0.069 0.060

0.068 0.051

>Non-Methylation code ID 21148

0.009 0.000 0.009 0.000 0.000 0.000 0.004 0.000 0.004 0.004

0.000 0.004 0.004 0.000 0.013 0.000 0.000 0.000 0.000 0.004

0.048 0.073 0.077 0.077 0.000 0.000 0.005 0.005 0.000 0.000

0.000 0.000 0.000 0.000 0.000 0.000 0.010 0.000 0.010 0.000

0.025 0.005 0.005 0.000 0.075 0.074 0.060 0.061 0.000 0.000

0.000 0.000 0.000 0.025 0.000 0.000 0.008 0.008 0.000 0.000

0.034 0.008 0.000 0.000 0.008 0.000 0.000 0.000 0.075 0.055

0.076 0.035

>Non-Methylation code ID 21148

0.006 0.006 0.006 0.000 0.006 0.006 0.000 0.000 0.000 0.006

0.000 0.006 0.000 0.006 0.000 0.019 0.006 0.000 0.000 0.000

0.054 0.060 0.064 0.080 0.025 0.000 0.000 0.000 0.000 0.000

0.006 0.006 0.000 0.006 0.000 0.000 0.012 0.000 0.006 0.000

0.006 0.000 0.006 0.000 0.066 0.071 0.065 0.057 0.000 0.000

0.005 0.005 0.000 0.005 0.000 0.000 0.000 0.011 0.005 0.000

0.005 0.005 0.011 0.005 0.000 0.000 0.000 0.000 0.050 0.071

0.064 0.089

>Non-Methylation code ID 21148

0.000 0.000 0.000 0.000 0.000 0.013 0.000 0.007 0.000 0.013

0.013 0.007 0.013 0.007 0.007 0.007 0.000 0.000 0.000 0.000

0.062 0.061 0.053 0.070 0.005 0.010 0.000 0.000 0.000 0.005

0.005 0.000 0.000 0.000 0.000 0.005 0.010 0.000 0.000 0.000

0.005 0.005 0.010 0.005 0.067 0.067 0.059 0.077 0.007 0.000

0.000 0.000 0.000 0.015 0.022 0.007 0.000 0.000 0.000 0.000

0.000 0.007 0.000 0.000 0.015 0.015 0.000 0.000 0.051 0.052

0.069 0.072

>Non-Methylation code ID 21148

0.000 0.000 0.000 0.000 0.005 0.011 0.011 0.005 0.000 0.005

0.005 0.005 0.011 0.005 0.005 0.000 0.000 0.000 0.000 0.000

0.066 0.073 0.054 0.071 0.006 0.000 0.000 0.000 0.006 0.006

0.000 0.006 0.000 0.006 0.000 0.006 0.006 0.000 0.000 0.000

0.013 0.006 0.013 0.006 0.072 0.064 0.043 0.072 0.007 0.000

0.000 0.000 0.007 0.015 0.015 0.007 0.000 0.000 0.000 0.000

0.007 0.007 0.000 0.022 0.000 0.000 0.000 0.000 0.055 0.057

0.061 0.072

>Non-Methylation code ID 21148

0.008 0.000 0.000 0.000 0.008 0.000 0.000 0.000 0.000 0.015

0.000 0.008 0.000 0.000 0.008 0.031 0.015 0.000 0.008 0.000

0.073 0.074 0.044 0.043 0.005 0.000 0.000 0.000 0.000 0.005

0.005 0.000 0.000 0.000 0.000 0.000 0.010 0.000 0.014 0.014

0.005 0.005 0.000 0.000 0.065 0.082 0.068 0.056 0.013 0.000

0.000 0.000 0.000 0.000 0.007 0.000 0.000 0.027 0.000 0.000

0.000 0.013 0.007 0.000 0.007 0.007 0.000 0.000 0.062 0.052

0.056 0.083

>Non-Methylation code ID 21148

0.006 0.000 0.000 0.000 0.006 0.000 0.000 0.000 0.006 0.013

0.000 0.006 0.000 0.000 0.006 0.019 0.013 0.000 0.006 0.000

0.077 0.068 0.056 0.050 0.005 0.000 0.000 0.000 0.000 0.005

0.005 0.000 0.005 0.000 0.000 0.000 0.010 0.000 0.010 0.015

0.005 0.005 0.000 0.000 0.072 0.089 0.058 0.049 0.013 0.000

0.000 0.006 0.000 0.000 0.006 0.000 0.000 0.025 0.000 0.006

0.000 0.013 0.006 0.000 0.000 0.000 0.000 0.000 0.073 0.057

0.057 0.071

>Non-Methylation code ID 21148

0.008 0.000 0.000 0.000 0.008 0.000 0.000 0.000 0.000 0.025

0.000 0.017 0.008 0.000 0.000 0.017 0.000 0.000 0.000 0.008

0.057 0.057 0.048 0.080 0.000 0.017 0.000 0.000 0.000 0.000

0.006 0.011 0.000 0.006 0.000 0.000 0.011 0.000 0.000 0.006

0.006 0.000 0.006 0.000 0.066 0.063 0.065 0.072 0.006 0.000

0.000 0.000 0.013 0.006 0.000 0.000 0.006 0.013 0.000 0.000

0.000 0.000 0.006 0.000 0.006 0.013 0.000 0.000 0.065 0.071

0.043 0.083

>Non-Methylation code ID 21148

0.000 0.007 0.013 0.000 0.000 0.007 0.000 0.007 0.000 0.007

0.000 0.007 0.000 0.007 0.000 0.013 0.007 0.000 0.000 0.007

0.061 0.063 0.064 0.066 0.023 0.000 0.000 0.000 0.006 0.000

0.006 0.006 0.000 0.000 0.000 0.000 0.000 0.000 0.006 0.000

0.017 0.000 0.006 0.000 0.065 0.059 0.074 0.067 0.000 0.000

0.005 0.005 0.000 0.005 0.005 0.000 0.000 0.005 0.005 0.000

0.005 0.005 0.016 0.005 0.000 0.000 0.000 0.000 0.053 0.069

0.070 0.077

>Non-Methylation code ID 21148

0.006 0.000 0.000 0.000 0.006 0.011 0.000 0.000 0.006 0.011

0.000 0.000 0.006 0.000 0.006 0.000 0.006 0.011 0.000 0.000

0.062 0.064 0.051 0.088 0.009 0.000 0.000 0.000 0.000 0.000

0.000 0.000 0.000 0.034 0.000 0.017 0.009 0.000 0.000 0.017

0.000 0.000 0.000 0.009 0.057 0.060 0.046 0.077 0.000 0.018

0.000 0.000 0.000 0.000 0.006 0.006 0.000 0.006 0.000 0.000

0.012 0.000 0.000 0.000 0.006 0.000 0.012 0.000 0.073 0.067

0.056 0.070

>Non-Methylation code ID 21148

0.000 0.016 0.000 0.000 0.000 0.005 0.005 0.011 0.000 0.005

0.000 0.000 0.005 0.000 0.000 0.005 0.005 0.000 0.005 0.000

0.065 0.059 0.065 0.078 0.013 0.000 0.000 0.000 0.013 0.006

0.000 0.000 0.006 0.013 0.000 0.000 0.000 0.000 0.006 0.000

0.006 0.013 0.000 0.000 0.068 0.066 0.044 0.078 0.000 0.000

0.000 0.000 0.009 0.000 0.000 0.000 0.000 0.027 0.000 0.018

0.009 0.000 0.000 0.009 0.000 0.000 0.009 0.009 0.057 0.055

0.047 0.084

>Non-Methylation code ID 21148

0.000 0.000 0.000 0.000 0.005 0.005 0.000 0.000 0.005 0.016

0.000 0.000 0.005 0.000 0.016 0.000 0.005 0.000 0.000 0.000

0.059 0.074 0.056 0.086 0.008 0.000 0.008 0.000 0.008 0.008

0.000 0.000 0.000 0.016 0.000 0.016 0.008 0.000 0.000 0.023

0.000 0.000 0.000 0.000 0.070 0.062 0.039 0.069 0.016 0.000

0.000 0.000 0.000 0.000 0.008 0.008 0.000 0.016 0.000 0.000

0.016 0.000 0.000 0.000 0.016 0.000 0.008 0.000 0.063 0.052

0.065 0.067

>Non-Methylation code ID 21148

0.006 0.000 0.006 0.006 0.012 0.000 0.006 0.000 0.000 0.000

0.006 0.006 0.000 0.000 0.000 0.019 0.006 0.000 0.000 0.000

0.059 0.061 0.072 0.067 0.000 0.006 0.000 0.000 0.012 0.000

0.000 0.000 0.006 0.012 0.012 0.000 0.006 0.000 0.006 0.006

0.006 0.006 0.000 0.000 0.070 0.062 0.055 0.071 0.008 0.008

0.000 0.000 0.015 0.000 0.000 0.000 0.008 0.015 0.000 0.000

0.000 0.008 0.000 0.000 0.000 0.008 0.000 0.008 0.085 0.068

0.059 0.044

>Non-Methylation code ID 21148

0.000 0.007 0.000 0.000 0.007 0.004 0.000 0.000 0.000 0.004

0.000 0.000 0.007 0.000 0.011 0.004 0.000 0.000 0.000 0.004

0.069 0.075 0.070 0.073 0.016 0.000 0.005 0.000 0.016 0.000

0.000 0.005 0.000 0.005 0.000 0.000 0.005 0.000 0.005 0.000

0.000 0.011 0.000 0.000 0.070 0.068 0.062 0.065 0.000 0.000

0.000 0.000 0.018 0.000 0.000 0.009 0.000 0.018 0.000 0.000

0.027 0.000 0.000 0.027 0.000 0.000 0.000 0.000 0.074 0.069

0.041 0.049

>Non-Methylation code ID 21148

0.000 0.000 0.000 0.013 0.009 0.000 0.000 0.004 0.000 0.004

0.004 0.000 0.009 0.000 0.009 0.004 0.000 0.000 0.000 0.000

0.072 0.075 0.073 0.057 0.005 0.000 0.000 0.000 0.010 0.000

0.000 0.000 0.005 0.005 0.000 0.005 0.005 0.005 0.005 0.005

0.000 0.010 0.000 0.000 0.064 0.077 0.065 0.067 0.000 0.000

0.000 0.005 0.010 0.000 0.000 0.000 0.000 0.000 0.000 0.005

0.010 0.000 0.010 0.010 0.005 0.000 0.000 0.000 0.056 0.081

0.078 0.064

>Non-Methylation code ID 21148

0.000 0.000 0.006 0.012 0.000 0.006 0.000 0.012 0.006 0.018

0.000 0.006 0.000 0.000 0.000 0.012 0.000 0.000 0.000 0.000

0.076 0.053 0.057 0.069 0.006 0.000 0.000 0.000 0.000 0.000

0.000 0.006 0.000 0.006 0.000 0.006 0.000 0.000 0.011 0.017

0.006 0.011 0.006 0.000 0.079 0.076 0.036 0.071 0.000 0.000

0.007 0.007 0.000 0.007 0.000 0.000 0.000 0.000 0.000 0.000

0.007 0.014 0.014 0.007 0.007 0.000 0.000 0.000 0.056 0.067

0.073 0.067

>Non-Methylation code ID 21148

0.004 0.009 0.000 0.004 0.000 0.009 0.000 0.000 0.004 0.000

0.000 0.000 0.000 0.000 0.009 0.009 0.000 0.004 0.000 0.000

0.060 0.060 0.066 0.096 0.012 0.000 0.012 0.006 0.000 0.006

0.000 0.000 0.012 0.012 0.000 0.000 0.000 0.006 0.000 0.000

0.000 0.006 0.000 0.000 0.067 0.053 0.063 0.077 0.000 0.000

0.000 0.000 0.000 0.000 0.000 0.008 0.025 0.008 0.008 0.000

0.000 0.000 0.000 0.008 0.000 0.000 0.017 0.000 0.078 0.077

0.057 0.047

>Non-Methylation code ID 21148

0.005 0.009 0.000 0.005 0.005 0.005 0.000 0.000 0.000 0.000

0.000 0.005 0.009 0.000 0.005 0.014 0.000 0.000 0.000 0.000

0.075 0.053 0.089 0.057 0.006 0.000 0.000 0.006 0.006 0.000

0.006 0.000 0.006 0.012 0.000 0.000 0.012 0.000 0.000 0.000

0.006 0.012 0.000 0.000 0.069 0.059 0.059 0.072 0.000 0.000

0.000 0.000 0.015 0.000 0.000 0.000 0.015 0.023 0.000 0.000

0.000 0.008 0.008 0.000 0.008 0.000 0.008 0.000 0.055 0.049

0.060 0.084

>Non-Methylation code ID 21148

0.005 0.009 0.000 0.000 0.005 0.005 0.000 0.005 0.000 0.005

0.000 0.005 0.009 0.000 0.005 0.009 0.000 0.000 0.000 0.000

0.073 0.054 0.092 0.053 0.006 0.006 0.000 0.006 0.012 0.000

0.006 0.000 0.000 0.012 0.000 0.000 0.012 0.000 0.000 0.000

0.006 0.006 0.000 0.000 0.070 0.053 0.068 0.070 0.008 0.000

0.000 0.000 0.023 0.000 0.000 0.000 0.008 0.023 0.000 0.000

0.000 0.008 0.008 0.000 0.008 0.000 0.008 0.000 0.051 0.048

0.068 0.075

>Non-Methylation code ID 21148

0.014 0.000 0.000 0.000 0.014 0.000 0.000 0.000 0.007 0.014

0.000 0.007 0.007 0.007 0.000 0.000 0.000 0.007 0.000 0.000

0.051 0.083 0.066 0.056 0.000 0.000 0.005 0.005 0.009 0.000

0.005 0.000 0.000 0.000 0.000 0.005 0.005 0.000 0.009 0.009

0.005 0.000 0.000 0.000 0.060 0.080 0.080 0.058 0.000 0.000

0.000 0.011 0.011 0.000 0.000 0.000 0.000 0.000 0.011 0.000

0.011 0.000 0.011 0.005 0.005 0.000 0.000 0.000 0.067 0.070

0.071 0.059

>Non-Methylation code ID 21152

0.004 0.004 0.004 0.004 0.004 0.009 0.000 0.000 0.000 0.004

0.000 0.000 0.004 0.009 0.009 0.000 0.000 0.000 0.000 0.000

0.051 0.088 0.079 0.057 0.005 0.000 0.005 0.000 0.005 0.005

0.000 0.000 0.005 0.000 0.000 0.005 0.005 0.000 0.005 0.011

0.005 0.011 0.000 0.000 0.080 0.052 0.067 0.066 0.011 0.000

0.000 0.000 0.000 0.000 0.000 0.005 0.000 0.000 0.000 0.000

0.005 0.000 0.021 0.011 0.005 0.000 0.000 0.000 0.075 0.065

0.047 0.088

>Non-Methylation code ID 21152

0.000 0.000 0.000 0.006 0.000 0.000 0.006 0.006 0.006 0.006

0.000 0.006 0.000 0.000 0.000 0.012 0.006 0.006 0.012 0.000

0.072 0.058 0.066 0.065 0.000 0.006 0.000 0.000 0.000 0.011

0.006 0.000 0.000 0.006 0.000 0.000 0.000 0.000 0.011 0.011

0.011 0.006 0.000 0.000 0.058 0.072 0.072 0.063 0.006 0.000

0.006 0.012 0.000 0.012 0.000 0.006 0.000 0.006 0.000 0.000

0.000 0.006 0.006 0.000 0.000 0.000 0.000 0.006 0.062 0.062

0.082 0.063

>Non-Methylation code ID 21152

0.000 0.000 0.000 0.000 0.000 0.005 0.000 0.000 0.005 0.016

0.005 0.000 0.010 0.010 0.010 0.000 0.000 0.000 0.000 0.000

0.044 0.069 0.082 0.076 0.000 0.000 0.005 0.000 0.000 0.010

0.000 0.000 0.000 0.000 0.000 0.014 0.005 0.000 0.010 0.005

0.000 0.000 0.005 0.005 0.068 0.067 0.067 0.075 0.017 0.000

0.000 0.017 0.000 0.009 0.009 0.009 0.000 0.000 0.000 0.000

0.009 0.000 0.000 0.000 0.034 0.000 0.000 0.000 0.065 0.058

0.059 0.049

>Non-Methylation code ID 21152

0.000 0.000 0.000 0.000 0.008 0.024 0.000 0.000 0.000 0.000

0.000 0.008 0.000 0.000 0.008 0.016 0.016 0.008 0.000 0.000

0.069 0.054 0.067 0.055 0.005 0.005 0.000 0.005 0.010 0.005

0.000 0.000 0.000 0.010 0.005 0.000 0.000 0.005 0.010 0.000

0.000 0.000 0.000 0.000 0.048 0.079 0.074 0.075 0.000 0.004

0.004 0.000 0.008 0.004 0.004 0.000 0.004 0.004 0.000 0.000

0.000 0.000 0.004 0.004 0.000 0.004 0.004 0.000 0.075 0.053

0.084 0.071

>Non-Methylation code ID 21152

0.007 0.000 0.000 0.000 0.020 0.000 0.007 0.007 0.007 0.007

0.000 0.007 0.000 0.000 0.000 0.007 0.007 0.007 0.000 0.007

0.064 0.057 0.055 0.072 0.000 0.000 0.000 0.000 0.015 0.000

0.007 0.007 0.000 0.015 0.015 0.007 0.007 0.000 0.000 0.007

0.000 0.000 0.000 0.007 0.050 0.062 0.065 0.066 0.000 0.006

0.000 0.000 0.006 0.000 0.000 0.017 0.006 0.000 0.000 0.000

0.000 0.006 0.006 0.011 0.000 0.000 0.000 0.011 0.074 0.073

0.070 0.047

>Non-Methylation code ID 21152

0.000 0.000 0.015 0.005 0.000 0.000 0.000 0.010 0.005 0.010

0.005 0.005 0.000 0.000 0.005 0.005 0.000 0.000 0.000 0.000

0.063 0.070 0.066 0.071 0.000 0.007 0.000 0.007 0.007 0.000

0.000 0.007 0.007 0.007 0.007 0.000 0.000 0.007 0.000 0.013

0.013 0.000 0.000 0.000 0.063 0.062 0.059 0.071 0.007 0.000

0.000 0.007 0.000 0.000 0.007 0.000 0.007 0.000 0.000 0.007

0.000 0.000 0.014 0.007 0.000 0.007 0.000 0.000 0.078 0.071

0.055 0.065

>Non-Methylation code ID 21152

0.000 0.008 0.000 0.008 0.008 0.000 0.000 0.016 0.000 0.008

0.008 0.000 0.000 0.008 0.000 0.024 0.008 0.000 0.000 0.000

0.064 0.045 0.056 0.074 0.012 0.000 0.000 0.006 0.000 0.000

0.006 0.000 0.006 0.000 0.000 0.006 0.000 0.000 0.006 0.012

0.000 0.006 0.000 0.006 0.077 0.086 0.048 0.055 0.000 0.000

0.011 0.000 0.000 0.000 0.000 0.011 0.006 0.011 0.006 0.006

0.000 0.006 0.006 0.006 0.000 0.000 0.000 0.000 0.068 0.062

0.067 0.071

>Non-Methylation code ID 21152

0.000 0.006 0.000 0.000 0.011 0.000 0.000 0.017 0.006 0.000

0.000 0.000 0.000 0.006 0.006 0.006 0.000 0.006 0.000 0.011

0.069 0.073 0.059 0.061 0.007 0.000 0.000 0.000 0.021 0.000

0.000 0.007 0.007 0.014 0.000 0.007 0.000 0.000 0.000 0.007

0.007 0.007 0.000 0.007 0.061 0.056 0.052 0.072 0.000 0.007

0.000 0.000 0.013 0.000 0.007 0.007 0.000 0.007 0.007 0.007

0.007 0.000 0.000 0.007 0.000 0.000 0.000 0.007 0.049 0.065

0.081 0.064

>Non-Methylation code ID 21152

0.005 0.005 0.000 0.000 0.010 0.005 0.000 0.005 0.005 0.010

0.000 0.005 0.005 0.000 0.005 0.000 0.000 0.000 0.000 0.005

0.071 0.072 0.057 0.066 0.000 0.006 0.000 0.000 0.012 0.012

0.000 0.006 0.000 0.006 0.000 0.006 0.006 0.000 0.006 0.000

0.006 0.000 0.000 0.000 0.057 0.067 0.068 0.076 0.007 0.007

0.000 0.014 0.014 0.000 0.000 0.000 0.000 0.007 0.007 0.000

0.007 0.000 0.000 0.007 0.000 0.007 0.000 0.007 0.066 0.068

0.047 0.069

>Non-Methylation code ID 21152

0.000 0.000 0.000 0.000 0.000 0.000 0.006 0.000 0.000 0.012

0.012 0.006 0.012 0.006 0.006 0.006 0.000 0.000 0.000 0.006

0.060 0.071 0.074 0.055 0.000 0.005 0.005 0.000 0.000 0.000

0.000 0.016 0.000 0.000 0.000 0.010 0.005 0.005 0.005 0.005

0.000 0.000 0.000 0.005 0.074 0.073 0.073 0.051 0.007 0.000

0.000 0.007 0.007 0.000 0.007 0.007 0.007 0.000 0.000 0.000

0.007 0.000 0.000 0.007 0.014 0.007 0.000 0.007 0.070 0.056

0.064 0.060

>Non-Methylation code ID 21152

0.011 0.005 0.005 0.016 0.000 0.005 0.000 0.005 0.005 0.011

0.000 0.000 0.000 0.005 0.000 0.000 0.000 0.000 0.000 0.000

0.041 0.061 0.081 0.080 0.000 0.000 0.000 0.000 0.000 0.000

0.000 0.005 0.010 0.005 0.000 0.000 0.005 0.000 0.010 0.015

0.000 0.000 0.005 0.000 0.074 0.053 0.084 0.065 0.013 0.007

0.000 0.000 0.000 0.020 0.000 0.000 0.007 0.007 0.000 0.000

0.000 0.000 0.007 0.007 0.000 0.000 0.000 0.007 0.051 0.061

0.079 0.071

>Non-Methylation code ID 21152

0.005 0.005 0.000 0.000 0.010 0.005 0.000 0.005 0.005 0.005

0.000 0.005 0.005 0.000 0.005 0.005 0.000 0.000 0.000 0.005

0.067 0.074 0.062 0.065 0.000 0.005 0.000 0.000 0.011 0.011

0.000 0.005 0.000 0.005 0.000 0.005 0.005 0.000 0.011 0.000

0.005 0.000 0.000 0.000 0.064 0.062 0.059 0.084 0.007 0.007

0.000 0.014 0.014 0.007 0.000 0.000 0.000 0.007 0.007 0.000

0.007 0.000 0.000 0.000 0.000 0.007 0.000 0.007 0.067 0.067

0.057 0.060

>Non-Methylation code ID 21152

0.000 0.004 0.000 0.004 0.012 0.004 0.004 0.000 0.000 0.008

0.000 0.000 0.000 0.000 0.008 0.000 0.004 0.004 0.000 0.000

0.049 0.068 0.075 0.091 0.010 0.000 0.000 0.000 0.005 0.010

0.000 0.000 0.000 0.005 0.000 0.000 0.000 0.000 0.010 0.015

0.005 0.000 0.005 0.000 0.065 0.070 0.082 0.050 0.000 0.000

0.008 0.000 0.008 0.016 0.000 0.000 0.000 0.024 0.000 0.000

0.024 0.000 0.000 0.000 0.000 0.008 0.000 0.000 0.055 0.060

0.070 0.060

>Non-Methylation code ID 21152

0.000 0.000 0.000 0.000 0.008 0.000 0.008 0.000 0.000 0.041

0.000 0.000 0.008 0.000 0.008 0.000 0.000 0.033 0.000 0.000

0.054 0.057 0.055 0.061 0.014 0.000 0.000 0.000 0.000 0.000

0.000 0.000 0.000 0.000 0.014 0.000 0.014 0.000 0.000 0.140

0.000 0.000 0.000 0.000 0.033 0.036 0.040 0.042 0.000 0.000

0.000 0.000 0.000 0.000 0.000 0.000 0.000 0.005 0.000 0.000

0.032 0.000 0.018 0.000 0.000 0.000 0.000 0.000 0.099 0.038

0.093 0.048

>Non-Methylation code ID 21152

0.012 0.000 0.000 0.000 0.000 0.000 0.000 0.000 0.000 0.000

0.000 0.000 0.012 0.000 0.000 0.124 0.000 0.000 0.012 0.000

0.047 0.040 0.041 0.043 0.000 0.000 0.000 0.000 0.000 0.005

0.000 0.000 0.000 0.009 0.000 0.000 0.033 0.000 0.014 0.000

0.000 0.000 0.000 0.000 0.084 0.047 0.089 0.053 0.000 0.000

0.000 0.000 0.017 0.000 0.000 0.000 0.000 0.033 0.000 0.000

0.008 0.000 0.008 0.000 0.000 0.033 0.000 0.000 0.074 0.068

0.040 0.051

>Non-Methylation code ID 21152

0.000 0.005 0.000 0.005 0.009 0.005 0.014 0.000 0.000 0.005

0.000 0.000 0.005 0.000 0.005 0.000 0.000 0.005 0.000 0.005

0.069 0.065 0.069 0.069 0.014 0.000 0.000 0.000 0.000 0.000

0.000 0.007 0.000 0.007 0.007 0.000 0.007 0.000 0.007 0.021

0.014 0.007 0.000 0.000 0.053 0.055 0.061 0.073 0.000 0.000

0.000 0.000 0.000 0.009 0.000 0.000 0.000 0.036 0.000 0.000

0.036 0.000 0.000 0.009 0.000 0.000 0.009 0.000 0.050 0.064

0.071 0.050

>Non-Methylation code ID 21152

0.000 0.004 0.000 0.004 0.012 0.000 0.004 0.000 0.000 0.008

0.000 0.000 0.004 0.000 0.008 0.000 0.004 0.004 0.000 0.000

0.050 0.070 0.082 0.081 0.010 0.000 0.000 0.000 0.005 0.005

0.000 0.000 0.000 0.005 0.000 0.000 0.005 0.000 0.010 0.015

0.005 0.000 0.005 0.000 0.060 0.069 0.071 0.067 0.000 0.000

0.008 0.000 0.008 0.008 0.000 0.000 0.000 0.025 0.000 0.000

0.033 0.000 0.000 0.000 0.000 0.008 0.000 0.000 0.055 0.068

0.067 0.053

>Non-Methylation code ID 21152

0.000 0.000 0.000 0.000 0.015 0.000 0.000 0.000 0.000 0.038

0.000 0.000 0.008 0.000 0.008 0.000 0.000 0.030 0.000 0.000

0.067 0.063 0.059 0.045 0.012 0.000 0.000 0.000 0.000 0.000

0.000 0.000 0.000 0.000 0.000 0.000 0.012 0.000 0.000 0.124

0.000 0.000 0.012 0.000 0.047 0.040 0.041 0.043 0.000 0.000

0.000 0.000 0.000 0.005 0.000 0.000 0.000 0.010 0.000 0.000

0.030 0.000 0.015 0.000 0.000 0.000 0.000 0.000 0.076 0.050

0.090 0.057

>Non-Methylation code ID 21152

0.000 0.000 0.000 0.000 0.014 0.000 0.000 0.000 0.000 0.043

0.000 0.000 0.007 0.000 0.007 0.000 0.000 0.022 0.000 0.000

0.059 0.056 0.058 0.067 0.012 0.000 0.000 0.000 0.000 0.000

0.000 0.000 0.000 0.000 0.000 0.000 0.012 0.000 0.000 0.120

0.000 0.000 0.012 0.000 0.052 0.039 0.039 0.047 0.000 0.000

0.000 0.000 0.000 0.005 0.000 0.000 0.000 0.011 0.000 0.000

0.038 0.000 0.011 0.000 0.000 0.000 0.000 0.000 0.075 0.045

0.084 0.064

>Non-Methylation code ID 21152

0.000 0.000 0.000 0.000 0.006 0.006 0.000 0.000 0.000 0.029

0.000 0.000 0.006 0.000 0.012 0.000 0.000 0.017 0.000 0.000

0.056 0.061 0.065 0.076 0.016 0.000 0.000 0.000 0.000 0.008

0.000 0.000 0.000 0.000 0.000 0.000 0.000 0.000 0.008 0.064

0.000 0.000 0.008 0.000 0.072 0.050 0.049 0.059 0.007 0.000

0.000 0.000 0.000 0.014 0.000 0.000 0.000 0.007 0.000 0.000

0.041 0.000 0.014 0.000 0.000 0.000 0.000 0.000 0.075 0.048

0.058 0.072

>Non-Methylation code ID 21152

0.000 0.000 0.000 0.000 0.000 0.000 0.000 0.000 0.000 0.008

0.000 0.000 0.024 0.000 0.016 0.000 0.000 0.000 0.004 0.000

0.096 0.038 0.101 0.046 0.000 0.000 0.000 0.000 0.010 0.010

0.010 0.000 0.000 0.038 0.000 0.000 0.010 0.000 0.000 0.000

0.000 0.038 0.000 0.010 0.066 0.049 0.046 0.048 0.000 0.000

0.000 0.000 0.000 0.000 0.000 0.000 0.000 0.000 0.014 0.000

0.014 0.000 0.000 0.130 0.000 0.014 0.000 0.000 0.033 0.039

0.043 0.045

>Non-Methylation code ID 21152

0.000 0.000 0.000 0.000 0.000 0.000 0.000 0.013 0.000 0.000

0.013 0.000 0.013 0.000 0.000 0.100 0.013 0.013 0.000 0.000

0.035 0.040 0.048 0.048 0.000 0.000 0.000 0.000 0.000 0.000

0.000 0.000 0.000 0.009 0.000 0.000 0.026 0.000 0.013 0.004

0.000 0.000 0.004 0.000 0.087 0.051 0.096 0.044 0.000 0.000

0.000 0.000 0.009 0.009 0.018 0.000 0.000 0.027 0.000 0.000

0.009 0.000 0.000 0.000 0.000 0.027 0.000 0.009 0.067 0.062

0.043 0.055

>Non-Methylation code ID 21152

0.007 0.000 0.000 0.000 0.007 0.015 0.015 0.000 0.000 0.015

0.000 0.000 0.000 0.000 0.000 0.007 0.000 0.022 0.000 0.007

0.049 0.048 0.056 0.085 0.000 0.000 0.007 0.000 0.007 0.007

0.000 0.007 0.000 0.007 0.007 0.000 0.014 0.000 0.000 0.020

0.007 0.000 0.007 0.000 0.057 0.052 0.064 0.072 0.000 0.006

0.000 0.000 0.006 0.011 0.000 0.000 0.000 0.017 0.000 0.000

0.006 0.000 0.006 0.006 0.006 0.000 0.006 0.000 0.069 0.069

0.050 0.078

>Non-Methylation code ID 21152

0.006 0.006 0.000 0.000 0.006 0.000 0.000 0.000 0.000 0.006

0.012 0.006 0.000 0.000 0.012 0.012 0.000 0.000 0.000 0.000

0.064 0.068 0.054 0.079 0.000 0.008 0.000 0.008 0.004 0.004

0.000 0.000 0.004 0.000 0.000 0.000 0.004 0.000 0.008 0.000

0.004 0.004 0.000 0.000 0.086 0.053 0.080 0.067 0.006 0.000

0.000 0.006 0.006 0.000 0.000 0.000 0.006 0.006 0.000 0.013

0.000 0.006 0.006 0.000 0.000 0.013 0.000 0.000 0.067 0.064

0.071 0.061

>Non-Methylation code ID 21152

0.000 0.000 0.004 0.004 0.000 0.004 0.004 0.004 0.004 0.000

0.000 0.000 0.004 0.004 0.009 0.000 0.000 0.000 0.009 0.000

0.075 0.084 0.069 0.052 0.000 0.000 0.018 0.006 0.000 0.012

0.000 0.000 0.012 0.000 0.000 0.006 0.000 0.006 0.000 0.000

0.012 0.000 0.000 0.006 0.070 0.078 0.048 0.062 0.007 0.000

0.000 0.000 0.000 0.000 0.000 0.000 0.007 0.000 0.015 0.007

0.000 0.007 0.015 0.000 0.022 0.000 0.000 0.000 0.073 0.090

0.053 0.037

>Non-Methylation code ID 21152

0.006 0.011 0.006 0.011 0.000 0.000 0.000 0.006 0.000 0.000

0.000 0.000 0.011 0.000 0.000 0.000 0.006 0.006 0.011 0.000

0.055 0.080 0.077 0.047 0.006 0.000 0.000 0.000 0.000 0.011

0.011 0.000 0.006 0.006 0.000 0.000 0.006 0.006 0.006 0.000

0.006 0.006 0.000 0.006 0.080 0.055 0.052 0.072 0.000 0.006

0.000 0.000 0.000 0.017 0.000 0.000 0.000 0.006 0.017 0.006

0.011 0.000 0.006 0.000 0.000 0.000 0.000 0.000 0.060 0.059

0.070 0.077

>Non-Methylation code ID 21152

0.000 0.000 0.000 0.000 0.000 0.000 0.006 0.000 0.000 0.006

0.000 0.000 0.033 0.000 0.011 0.017 0.000 0.000 0.000 0.000

0.065 0.064 0.067 0.065 0.010 0.005 0.000 0.000 0.000 0.000

0.000 0.005 0.000 0.005 0.000 0.000 0.031 0.000 0.010 0.000

0.000 0.000 0.000 0.000 0.069 0.049 0.074 0.074 0.032 0.000

0.000 0.000 0.011 0.000 0.000 0.000 0.000 0.022 0.000 0.000

0.065 0.000 0.000 0.000 0.000 0.000 0.000 0.000 0.092 0.006

0.094 0.012

>Non-Methylation code ID 21152

0.005 0.005 0.005 0.005 0.005 0.009 0.000 0.000 0.000 0.000

0.000 0.005 0.005 0.000 0.005 0.005 0.000 0.000 0.000 0.005

0.069 0.063 0.077 0.070 0.006 0.000 0.000 0.000 0.006 0.012

0.000 0.000 0.000 0.000 0.000 0.006 0.012 0.000 0.006 0.006

0.018 0.006 0.000 0.000 0.042 0.070 0.067 0.076 0.000 0.000

0.000 0.000 0.000 0.029 0.000 0.000 0.000 0.039 0.000 0.000

0.000 0.019 0.010 0.000 0.010 0.010 0.000 0.000 0.065 0.045

0.060 0.047

>Non-Methylation code ID 21152

0.007 0.007 0.000 0.000 0.007 0.014 0.000 0.000 0.000 0.021

0.007 0.000 0.014 0.000 0.000 0.014 0.000 0.000 0.000 0.000

0.054 0.053 0.079 0.056 0.005 0.005 0.000 0.000 0.000 0.011

0.000 0.000 0.000 0.011 0.000 0.000 0.005 0.005 0.011 0.011

0.000 0.000 0.005 0.000 0.074 0.068 0.052 0.071 0.018 0.000

0.006 0.000 0.006 0.006 0.000 0.000 0.000 0.012 0.000 0.000

0.012 0.000 0.006 0.000 0.000 0.000 0.006 0.000 0.056 0.071

0.071 0.062

>Non-Methylation code ID 21152

0.013 0.006 0.000 0.000 0.006 0.013 0.000 0.000 0.000 0.019

0.000 0.000 0.013 0.000 0.006 0.006 0.000 0.000 0.000 0.000

0.060 0.080 0.064 0.049 0.012 0.004 0.000 0.000 0.000 0.004

0.000 0.000 0.000 0.004 0.000 0.000 0.008 0.000 0.012 0.008

0.000 0.000 0.000 0.000 0.059 0.075 0.069 0.076 0.021 0.000

0.000 0.000 0.000 0.007 0.000 0.000 0.000 0.014 0.000 0.000

0.021 0.000 0.007 0.000 0.000 0.007 0.007 0.000 0.068 0.068

0.061 0.053

>Non-Methylation code ID 21152

0.000 0.000 0.000 0.007 0.000 0.007 0.000 0.000 0.000 0.013

0.000 0.020 0.000 0.000 0.007 0.007 0.020 0.007 0.000 0.000

0.057 0.066 0.054 0.071 0.000 0.009 0.000 0.000 0.000 0.018

0.009 0.000 0.000 0.018 0.000 0.000 0.000 0.009 0.000 0.018

0.027 0.009 0.000 0.000 0.060 0.056 0.046 0.055 0.006 0.000

0.006 0.000 0.000 0.006 0.006 0.000 0.000 0.000 0.000 0.000

0.000 0.013 0.013 0.000 0.006 0.000 0.000 0.006 0.069 0.066

0.076 0.058

>Non-Methylation code ID 21152

0.009 0.004 0.000 0.000 0.000 0.000 0.000 0.000 0.000 0.000

0.000 0.000 0.027 0.000 0.013 0.004 0.000 0.000 0.000 0.000

0.074 0.056 0.071 0.075 0.047 0.000 0.000 0.000 0.000 0.000

0.000 0.000 0.000 0.012 0.000 0.000 0.095 0.000 0.000 0.000

0.000 0.000 0.000 0.000 0.065 0.018 0.067 0.030 0.000 0.000

0.000 0.000 0.000 0.000 0.000 0.000 0.000 0.011 0.000 0.000

0.039 0.000 0.011 0.006 0.000 0.000 0.000 0.000 0.061 0.057

0.069 0.078

>Non-Methylation code ID 21152

0.014 0.007 0.000 0.000 0.007 0.014 0.000 0.000 0.000 0.021

0.007 0.000 0.014 0.000 0.000 0.007 0.000 0.000 0.000 0.000

0.057 0.054 0.080 0.050 0.005 0.005 0.000 0.000 0.000 0.010

0.000 0.000 0.000 0.010 0.000 0.000 0.005 0.000 0.015 0.010

0.000 0.000 0.005 0.000 0.067 0.069 0.068 0.064 0.023 0.000

0.008 0.000 0.008 0.015 0.000 0.000 0.000 0.015 0.000 0.000

0.015 0.000 0.000 0.000 0.000 0.000 0.008 0.000 0.060 0.067

0.062 0.052

>Non-Methylation code ID 21152

0.009 0.000 0.000 0.000 0.000 0.004 0.000 0.000 0.000 0.009

0.000 0.000 0.017 0.000 0.009 0.000 0.000 0.004 0.004 0.000

0.066 0.049 0.077 0.085 0.017 0.006 0.000 0.000 0.006 0.006

0.000 0.000 0.000 0.012 0.000 0.000 0.017 0.000 0.006 0.006

0.000 0.000 0.000 0.000 0.065 0.068 0.050 0.074 0.011 0.006

0.000 0.000 0.000 0.000 0.000 0.000 0.000 0.006 0.000 0.000

0.029 0.000 0.011 0.006 0.000 0.000 0.000 0.000 0.045 0.075

0.074 0.070

>Non-Methylation code ID 21152

0.000 0.000 0.000 0.000 0.000 0.000 0.000 0.000 0.000 0.011

0.000 0.000 0.042 0.000 0.011 0.005 0.000 0.000 0.000 0.000

0.071 0.053 0.069 0.071 0.009 0.004 0.000 0.000 0.000 0.000

0.000 0.000 0.000 0.000 0.000 0.000 0.027 0.000 0.013 0.004

0.000 0.000 0.000 0.000 0.074 0.056 0.071 0.075 0.051 0.000

0.000 0.000 0.000 0.000 0.000 0.000 0.000 0.013 0.000 0.000

0.090 0.000 0.000 0.000 0.000 0.000 0.000 0.000 0.061 0.019

0.065 0.034

>Non-Methylation code ID 21152

0.014 0.000 0.000 0.000 0.000 0.009 0.000 0.000 0.000 0.005

0.000 0.000 0.005 0.009 0.014 0.000 0.000 0.000 0.005 0.000

0.048 0.062 0.085 0.077 0.014 0.000 0.005 0.000 0.000 0.009

0.000 0.000 0.000 0.005 0.000 0.005 0.000 0.005 0.014 0.005

0.000 0.000 0.000 0.000 0.065 0.066 0.049 0.091 0.005 0.000

0.000 0.000 0.000 0.016 0.000 0.000 0.000 0.000 0.005 0.000

0.011 0.005 0.011 0.005 0.005 0.000 0.000 0.000 0.049 0.073

0.062 0.083

>Non-Methylation code ID 21152

0.028 0.000 0.000 0.000 0.000 0.000 0.000 0.000 0.000 0.007

0.000 0.000 0.043 0.000 0.014 0.000 0.000 0.000 0.000 0.000

0.043 0.055 0.065 0.077 0.000 0.000 0.000 0.000 0.000 0.004

0.000 0.000 0.000 0.008 0.000 0.000 0.019 0.000 0.012 0.000

0.000 0.004 0.004 0.000 0.074 0.063 0.075 0.072 0.021 0.005

0.000 0.000 0.005 0.005 0.000 0.000 0.000 0.000 0.000 0.000

0.010 0.000 0.010 0.005 0.000 0.000 0.000 0.000 0.072 0.069

0.055 0.076

>Non-Methylation code ID 21152

0.000 0.005 0.005 0.000 0.005 0.005 0.005 0.000 0.000 0.016

0.000 0.000 0.005 0.000 0.005 0.010 0.005 0.000 0.000 0.000

0.071 0.056 0.062 0.076 0.017 0.006 0.000 0.000 0.006 0.000

0.000 0.011 0.000 0.011 0.000 0.000 0.006 0.006 0.006 0.000

0.000 0.000 0.000 0.000 0.064 0.065 0.068 0.068 0.011 0.000

0.000 0.000 0.011 0.011 0.000 0.000 0.000 0.042 0.000 0.011

0.011 0.000 0.000 0.021 0.000 0.000 0.000 0.000 0.068 0.051

0.053 0.046

>Non-Methylation code ID 21152

0.047 0.000 0.000 0.000 0.000 0.000 0.000 0.000 0.000 0.012

0.000 0.000 0.095 0.000 0.000 0.000 0.000 0.000 0.000 0.000

0.065 0.018 0.067 0.030 0.000 0.000 0.000 0.000 0.000 0.000

0.000 0.000 0.000 0.009 0.000 0.000 0.033 0.000 0.014 0.005

0.000 0.000 0.000 0.000 0.060 0.061 0.072 0.080 0.010 0.005

0.000 0.000 0.000 0.000 0.000 0.000 0.000 0.000 0.000 0.000

0.031 0.000 0.010 0.005 0.000 0.000 0.000 0.000 0.073 0.052

0.073 0.073

>Non-Methylation code ID 21166

0.000 0.000 0.000 0.000 0.000 0.000 0.000 0.000 0.000 0.011

0.000 0.000 0.037 0.000 0.011 0.011 0.000 0.000 0.000 0.000

0.063 0.054 0.070 0.077 0.010 0.005 0.000 0.000 0.000 0.000

0.000 0.000 0.000 0.000 0.000 0.000 0.035 0.000 0.010 0.005

0.000 0.000 0.000 0.000 0.072 0.050 0.069 0.077 0.038 0.000

0.000 0.000 0.000 0.000 0.000 0.000 0.000 0.025 0.000 0.000

0.089 0.000 0.000 0.000 0.000 0.000 0.000 0.000 0.074 0.011

0.077 0.019

>Non-Methylation code ID 21166

0.036 0.000 0.000 0.000 0.000 0.000 0.000 0.000 0.000 0.024

0.000 0.000 0.096 0.000 0.000 0.000 0.000 0.000 0.000 0.000

0.069 0.011 0.071 0.026 0.000 0.000 0.000 0.000 0.000 0.000

0.000 0.000 0.000 0.011 0.000 0.000 0.037 0.000 0.011 0.011

0.000 0.000 0.000 0.000 0.063 0.054 0.070 0.077 0.010 0.005

0.000 0.000 0.000 0.000 0.000 0.000 0.000 0.000 0.000 0.000

0.030 0.000 0.010 0.005 0.000 0.000 0.000 0.000 0.070 0.052

0.071 0.080

>Non-Methylation code ID 21166

0.014 0.000 0.000 0.000 0.000 0.014 0.000 0.000 0.000 0.010

0.000 0.000 0.000 0.010 0.014 0.000 0.000 0.000 0.000 0.000

0.040 0.055 0.088 0.088 0.016 0.000 0.005 0.000 0.000 0.010

0.000 0.000 0.000 0.000 0.000 0.005 0.000 0.005 0.016 0.005

0.000 0.000 0.000 0.005 0.064 0.058 0.062 0.081 0.006 0.000

0.000 0.000 0.000 0.019 0.000 0.000 0.000 0.000 0.000 0.000

0.013 0.006 0.013 0.000 0.019 0.000 0.000 0.000 0.056 0.076

0.049 0.076

>Non-Methylation code ID 21166

0.008 0.004 0.000 0.000 0.000 0.004 0.000 0.000 0.000 0.008

0.000 0.000 0.008 0.000 0.013 0.008 0.000 0.000 0.000 0.000

0.060 0.079 0.071 0.068 0.019 0.000 0.000 0.000 0.000 0.006

0.000 0.000 0.000 0.012 0.000 0.000 0.019 0.000 0.006 0.000

0.000 0.006 0.012 0.000 0.070 0.065 0.067 0.051 0.017 0.008

0.000 0.000 0.008 0.017 0.000 0.000 0.000 0.025 0.000 0.000

0.017 0.000 0.000 0.008 0.000 0.000 0.000 0.000 0.057 0.083

0.057 0.037

>Non-Methylation code ID 21166

0.012 0.006 0.000 0.000 0.006 0.000 0.000 0.012 0.000 0.012

0.000 0.000 0.000 0.012 0.006 0.000 0.000 0.000 0.000 0.000

0.063 0.071 0.080 0.052 0.010 0.000 0.000 0.000 0.010 0.010

0.000 0.000 0.000 0.030 0.000 0.030 0.000 0.000 0.000 0.030

0.000 0.000 0.000 0.000 0.050 0.053 0.054 0.057 0.000 0.006

0.006 0.000 0.006 0.006 0.000 0.000 0.000 0.019 0.000 0.000

0.006 0.000 0.000 0.006 0.013 0.006 0.000 0.000 0.070 0.057

0.063 0.066

>Non-Methylation code ID 21166

0.010 0.000 0.000 0.000 0.000 0.015 0.000 0.000 0.000 0.010

0.000 0.000 0.000 0.010 0.015 0.000 0.005 0.000 0.000 0.000

0.044 0.058 0.092 0.075 0.016 0.000 0.005 0.000 0.000 0.011

0.000 0.000 0.000 0.000 0.000 0.005 0.005 0.005 0.011 0.005

0.000 0.000 0.000 0.005 0.070 0.052 0.072 0.070 0.006 0.000

0.000 0.000 0.000 0.012 0.000 0.000 0.000 0.000 0.000 0.000

0.012 0.006 0.018 0.000 0.018 0.000 0.000 0.000 0.047 0.064

0.065 0.086

>Non-Methylation code ID 21166

0.000 0.000 0.000 0.000 0.000 0.000 0.005 0.000 0.000 0.011

0.000 0.000 0.027 0.000 0.011 0.016 0.000 0.000 0.000 0.000

0.061 0.054 0.067 0.082 0.010 0.010 0.000 0.000 0.000 0.000

0.000 0.005 0.000 0.005 0.000 0.000 0.025 0.000 0.010 0.000

0.000 0.000 0.000 0.000 0.075 0.053 0.059 0.082 0.030 0.000

0.000 0.000 0.010 0.000 0.000 0.000 0.000 0.020 0.000 0.000

0.050 0.000 0.000 0.010 0.000 0.000 0.000 0.000 0.089 0.015

0.086 0.023

>Non-Methylation code ID 21166

0.000 0.000 0.006 0.000 0.000 0.006 0.006 0.000 0.000 0.011

0.000 0.000 0.000 0.006 0.011 0.000 0.011 0.006 0.000 0.006

0.059 0.066 0.069 0.071 0.000 0.006 0.000 0.006 0.006 0.006

0.000 0.000 0.000 0.000 0.000 0.006 0.006 0.000 0.006 0.006

0.018 0.006 0.000 0.000 0.069 0.072 0.056 0.065 0.000 0.000

0.000 0.000 0.009 0.017 0.009 0.000 0.000 0.009 0.000 0.009

0.000 0.009 0.000 0.017 0.009 0.017 0.000 0.000 0.062 0.064

0.050 0.055

>Non-Methylation code ID 21166

0.005 0.005 0.000 0.000 0.000 0.009 0.000 0.000 0.000 0.014

0.000 0.000 0.009 0.000 0.009 0.005 0.000 0.000 0.005 0.000

0.079 0.075 0.050 0.070 0.005 0.000 0.005 0.005 0.000 0.005

0.000 0.000 0.000 0.000 0.000 0.000 0.005 0.005 0.011 0.011

0.000 0.011 0.000 0.005 0.078 0.072 0.046 0.068 0.011 0.000

0.000 0.000 0.006 0.000 0.000 0.000 0.000 0.011 0.000 0.000

0.006 0.000 0.011 0.000 0.011 0.006 0.000 0.000 0.074 0.074

0.064 0.059

>Non-Methylation code ID 21166

0.000 0.000 0.000 0.005 0.010 0.000 0.000 0.000 0.010 0.005

0.000 0.000 0.005 0.005 0.010 0.005 0.010 0.000 0.000 0.000

0.061 0.069 0.057 0.081 0.006 0.000 0.000 0.006 0.000 0.006

0.000 0.000 0.012 0.012 0.000 0.000 0.000 0.006 0.012 0.018

0.000 0.000 0.000 0.000 0.065 0.069 0.068 0.052 0.000 0.000

0.000 0.012 0.000 0.006 0.000 0.000 0.012 0.006 0.000 0.006

0.012 0.000 0.000 0.006 0.000 0.006 0.000 0.006 0.062 0.061

0.071 0.068

>Non-Methylation code ID 21166

0.000 0.000 0.000 0.000 0.000 0.016 0.000 0.000 0.000 0.008

0.000 0.016 0.008 0.000 0.000 0.032 0.000 0.016 0.000 0.008

0.057 0.062 0.048 0.063 0.006 0.000 0.000 0.006 0.012 0.006

0.000 0.000 0.000 0.012 0.000 0.000 0.006 0.000 0.006 0.000

0.018 0.000 0.000 0.000 0.063 0.068 0.060 0.071 0.000 0.000

0.000 0.005 0.005 0.005 0.000 0.000 0.005 0.015 0.000 0.000

0.000 0.005 0.015 0.005 0.000 0.000 0.000 0.000 0.053 0.058

0.077 0.084

>Non-Methylation code ID 21166

0.000 0.000 0.000 0.011 0.006 0.000 0.000 0.000 0.006 0.006

0.000 0.000 0.006 0.000 0.017 0.022 0.000 0.000 0.000 0.000

0.053 0.060 0.070 0.078 0.006 0.000 0.000 0.000 0.000 0.006

0.000 0.000 0.006 0.006 0.000 0.006 0.000 0.000 0.017 0.017

0.000 0.012 0.000 0.000 0.056 0.093 0.076 0.033 0.015 0.000

0.000 0.000 0.015 0.015 0.000 0.000 0.007 0.015 0.000 0.000

0.007 0.000 0.000 0.000 0.007 0.000 0.000 0.000 0.065 0.065

0.073 0.048

>Non-Methylation code ID 21166

0.000 0.006 0.000 0.000 0.006 0.006 0.000 0.000 0.000 0.000

0.006 0.000 0.006 0.006 0.006 0.022 0.006 0.000 0.000 0.000

0.064 0.068 0.062 0.073 0.012 0.000 0.004 0.004 0.004 0.000

0.000 0.000 0.000 0.008 0.000 0.000 0.004 0.000 0.012 0.000

0.000 0.000 0.000 0.000 0.065 0.084 0.084 0.051 0.016 0.000

0.000 0.000 0.016 0.008 0.008 0.008 0.000 0.024 0.000 0.008

0.008 0.000 0.000 0.000 0.000 0.000 0.000 0.000 0.068 0.065

0.055 0.048

>Non-Methylation code ID 21166

0.012 0.000 0.004 0.000 0.004 0.000 0.000 0.000 0.004 0.004

0.000 0.000 0.004 0.000 0.016 0.004 0.000 0.000 0.000 0.000

0.071 0.096 0.077 0.038 0.014 0.000 0.000 0.000 0.014 0.014

0.007 0.007 0.007 0.021 0.000 0.000 0.007 0.000 0.000 0.000

0.000 0.000 0.000 0.000 0.067 0.080 0.057 0.040 0.000 0.006

0.000 0.006 0.006 0.006 0.000 0.000 0.000 0.000 0.000 0.000

0.006 0.006 0.006 0.029 0.000 0.000 0.000 0.000 0.063 0.060

0.062 0.079

>Non-Methylation code ID 21166

0.008 0.000 0.004 0.000 0.004 0.000 0.000 0.000 0.004 0.004

0.000 0.000 0.004 0.000 0.016 0.008 0.000 0.000 0.000 0.000

0.072 0.094 0.080 0.035 0.014 0.000 0.000 0.000 0.014 0.014

0.000 0.007 0.007 0.021 0.000 0.000 0.014 0.000 0.000 0.000

0.000 0.000 0.000 0.000 0.066 0.077 0.064 0.033 0.000 0.000

0.000 0.006 0.006 0.006 0.000 0.000 0.000 0.006 0.000 0.000

0.006 0.006 0.006 0.032 0.000 0.000 0.000 0.000 0.063 0.064

0.061 0.068

>Non-Methylation code ID 21166

0.004 0.000 0.000 0.000 0.004 0.000 0.000 0.000 0.000 0.004

0.000 0.000 0.018 0.000 0.013 0.004 0.004 0.004 0.000 0.000

0.075 0.076 0.045 0.080 0.011 0.006 0.000 0.000 0.000 0.006

0.006 0.000 0.000 0.017 0.000 0.000 0.017 0.000 0.006 0.006

0.000 0.000 0.000 0.000 0.051 0.065 0.073 0.073 0.012 0.000

0.000 0.000 0.000 0.000 0.000 0.000 0.000 0.012 0.000 0.000

0.012 0.000 0.012 0.006 0.006 0.006 0.000 0.006 0.076 0.067

0.039 0.077

>Non-Methylation code ID 21166

0.007 0.000 0.000 0.000 0.000 0.014 0.000 0.000 0.007 0.000

0.000 0.007 0.007 0.000 0.007 0.029 0.000 0.014 0.000 0.000

0.062 0.072 0.051 0.056 0.006 0.000 0.000 0.006 0.006 0.011

0.000 0.000 0.006 0.017 0.000 0.000 0.006 0.000 0.006 0.000

0.006 0.000 0.000 0.000 0.075 0.066 0.061 0.063 0.000 0.000

0.000 0.010 0.005 0.005 0.000 0.000 0.005 0.005 0.000 0.000

0.000 0.000 0.015 0.015 0.000 0.000 0.000 0.000 0.051 0.064

0.080 0.080

>Non-Methylation code ID 21166

0.011 0.000 0.000 0.000 0.000 0.000 0.000 0.000 0.000 0.006

0.000 0.000 0.011 0.000 0.011 0.011 0.006 0.011 0.000 0.006

0.073 0.073 0.049 0.065 0.005 0.000 0.000 0.000 0.005 0.000

0.000 0.000 0.000 0.005 0.000 0.000 0.020 0.000 0.010 0.005

0.005 0.005 0.000 0.000 0.071 0.073 0.039 0.090 0.006 0.006

0.000 0.000 0.000 0.006 0.006 0.000 0.000 0.018 0.000 0.000

0.018 0.000 0.006 0.006 0.000 0.000 0.000 0.000 0.057 0.077

0.063 0.062

>Non-Methylation code ID 21166

0.019 0.000 0.000 0.000 0.000 0.000 0.000 0.000 0.000 0.013

0.000 0.000 0.013 0.000 0.013 0.006 0.013 0.006 0.000 0.000

0.072 0.069 0.037 0.071 0.004 0.000 0.000 0.000 0.004 0.000

0.000 0.000 0.000 0.004 0.000 0.000 0.022 0.000 0.013 0.004

0.000 0.004 0.000 0.000 0.084 0.076 0.036 0.082 0.010 0.005

0.000 0.000 0.000 0.005 0.010 0.000 0.000 0.005 0.000 0.000

0.016 0.000 0.005 0.005 0.000 0.000 0.000 0.000 0.054 0.066

0.076 0.075

>Non-Methylation code ID 21166

0.012 0.000 0.000 0.000 0.000 0.000 0.000 0.000 0.000 0.012

0.000 0.000 0.012 0.000 0.012 0.012 0.006 0.006 0.000 0.006

0.076 0.073 0.039 0.070 0.005 0.000 0.000 0.000 0.005 0.000

0.000 0.000 0.000 0.010 0.000 0.000 0.019 0.000 0.010 0.005

0.005 0.005 0.000 0.000 0.071 0.071 0.041 0.088 0.006 0.006

0.000 0.000 0.000 0.006 0.006 0.000 0.000 0.018 0.000 0.000

0.018 0.000 0.006 0.006 0.000 0.000 0.000 0.000 0.050 0.069

0.079 0.062

>Non-Methylation code ID 21166

0.008 0.000 0.004 0.000 0.004 0.004 0.000 0.000 0.000 0.008

0.000 0.000 0.000 0.000 0.017 0.004 0.000 0.000 0.000 0.000

0.071 0.067 0.074 0.070 0.025 0.000 0.000 0.000 0.000 0.008

0.000 0.000 0.000 0.016 0.000 0.000 0.008 0.000 0.000 0.016

0.008 0.008 0.016 0.000 0.062 0.039 0.064 0.061 0.000 0.005

0.000 0.000 0.005 0.011 0.000 0.000 0.000 0.005 0.000 0.000

0.021 0.000 0.011 0.005 0.000 0.000 0.000 0.000 0.053 0.057

0.074 0.086

>Non-Methylation code ID 21166

0.006 0.000 0.012 0.000 0.012 0.000 0.000 0.006 0.006 0.006

0.000 0.006 0.012 0.000 0.000 0.006 0.000 0.000 0.000 0.000

0.053 0.070 0.063 0.076 0.000 0.006 0.000 0.006 0.006 0.000

0.000 0.006 0.000 0.006 0.000 0.000 0.011 0.006 0.006 0.011

0.011 0.000 0.000 0.000 0.066 0.074 0.060 0.062 0.005 0.005

0.000 0.005 0.000 0.000 0.005 0.000 0.005 0.014 0.000 0.000

0.005 0.000 0.009 0.005 0.000 0.000 0.000 0.000 0.075 0.060

0.058 0.085

>Non-Methylation code ID 21166

0.000 0.004 0.000 0.000 0.008 0.000 0.008 0.000 0.000 0.000

0.000 0.004 0.011 0.000 0.011 0.004 0.000 0.000 0.000 0.000

0.070 0.070 0.066 0.078 0.023 0.000 0.000 0.000 0.015 0.000

0.008 0.008 0.000 0.000 0.000 0.000 0.015 0.000 0.000 0.000

0.015 0.015 0.000 0.000 0.047 0.060 0.060 0.066 0.000 0.000

0.000 0.000 0.010 0.000 0.000 0.000 0.000 0.010 0.000 0.000

0.048 0.010 0.000 0.019 0.010 0.000 0.000 0.000 0.068 0.029

0.074 0.056

>Non-Methylation code ID 21166

0.015 0.000 0.000 0.000 0.015 0.000 0.007 0.007 0.007 0.000

0.000 0.000 0.007 0.000 0.000 0.000 0.015 0.015 0.000 0.000

0.051 0.067 0.062 0.066 0.000 0.000 0.000 0.000 0.013 0.000

0.000 0.000 0.007 0.007 0.000 0.000 0.020 0.007 0.007 0.013

0.007 0.000 0.000 0.000 0.072 0.039 0.078 0.065 0.000 0.000

0.000 0.004 0.009 0.000 0.009 0.000 0.000 0.004 0.000 0.004

0.009 0.000 0.009 0.004 0.000 0.000 0.000 0.000 0.074 0.063

0.060 0.084

>Non-Methylation code ID 21166

0.000 0.000 0.000 0.000 0.008 0.000 0.000 0.000 0.000 0.017

0.000 0.000 0.042 0.017 0.000 0.008 0.008 0.000 0.000 0.000

0.062 0.041 0.076 0.055 0.000 0.004 0.000 0.000 0.008 0.000

0.004 0.000 0.000 0.000 0.000 0.008 0.011 0.000 0.011 0.004

0.000 0.000 0.000 0.000 0.073 0.073 0.064 0.073 0.024 0.000

0.000 0.000 0.008 0.000 0.008 0.000 0.000 0.000 0.000 0.000

0.016 0.000 0.000 0.000 0.024 0.016 0.000 0.000 0.046 0.066

0.068 0.058

>Non-Methylation code ID 21166

0.005 0.005 0.000 0.005 0.000 0.010 0.000 0.000 0.016 0.010

0.005 0.000 0.000 0.000 0.000 0.005 0.005 0.000 0.000 0.000

0.074 0.054 0.070 0.067 0.004 0.004 0.000 0.004 0.004 0.004

0.004 0.000 0.004 0.000 0.000 0.000 0.008 0.000 0.008 0.004

0.000 0.000 0.000 0.000 0.069 0.061 0.083 0.071 0.005 0.000

0.000 0.005 0.005 0.010 0.005 0.000 0.010 0.005 0.000 0.005

0.000 0.000 0.005 0.000 0.000 0.005 0.000 0.000 0.076 0.083

0.053 0.059

>Non-Methylation code ID 21166

0.011 0.000 0.000 0.000 0.000 0.017 0.000 0.000 0.000 0.000

0.000 0.000 0.000 0.006 0.011 0.023 0.000 0.000 0.006 0.000

0.056 0.080 0.056 0.067 0.031 0.000 0.006 0.000 0.000 0.012

0.000 0.000 0.000 0.000 0.000 0.000 0.006 0.000 0.012 0.000

0.000 0.006 0.000 0.000 0.053 0.079 0.064 0.063 0.005 0.000

0.000 0.005 0.000 0.011 0.000 0.000 0.000 0.005 0.000 0.000

0.005 0.011 0.011 0.005 0.000 0.000 0.005 0.000 0.067 0.073

0.054 0.074

>Non-Methylation code ID 21166

0.000 0.004 0.000 0.000 0.004 0.000 0.008 0.000 0.000 0.000

0.000 0.008 0.012 0.000 0.012 0.004 0.000 0.000 0.000 0.000

0.074 0.070 0.071 0.067 0.023 0.000 0.000 0.000 0.008 0.000

0.008 0.000 0.000 0.000 0.000 0.000 0.015 0.000 0.000 0.000

0.030 0.015 0.000 0.000 0.043 0.065 0.070 0.057 0.000 0.000

0.000 0.000 0.000 0.000 0.000 0.000 0.000 0.019 0.000 0.000

0.048 0.019 0.000 0.010 0.010 0.000 0.000 0.000 0.062 0.045

0.067 0.054

>Non-Methylation code ID 21166

0.004 0.000 0.000 0.004 0.000 0.000 0.004 0.000 0.000 0.004

0.000 0.000 0.004 0.004 0.009 0.009 0.004 0.000 0.009 0.000

0.077 0.049 0.076 0.074 0.013 0.000 0.000 0.000 0.007 0.013

0.020 0.007 0.000 0.007 0.000 0.007 0.007 0.000 0.000 0.007

0.000 0.000 0.000 0.000 0.063 0.051 0.072 0.061 0.013 0.000

0.000 0.000 0.007 0.007 0.000 0.007 0.000 0.013 0.000 0.000

0.007 0.000 0.007 0.007 0.013 0.000 0.000 0.000 0.056 0.062

0.063 0.073

>Non-Methylation code ID 21166

0.000 0.000 0.000 0.007 0.000 0.029 0.007 0.000 0.007 0.000

0.007 0.000 0.000 0.000 0.000 0.007 0.000 0.000 0.007 0.007

0.042 0.053 0.074 0.086 0.020 0.014 0.000 0.000 0.000 0.014

0.000 0.000 0.000 0.000 0.000 0.000 0.000 0.000 0.007 0.007

0.014 0.000 0.000 0.000 0.069 0.075 0.053 0.062 0.004 0.000

0.000 0.009 0.000 0.004 0.004 0.000 0.000 0.000 0.000 0.000

0.000 0.000 0.017 0.000 0.000 0.004 0.004 0.000 0.078 0.065

0.067 0.076

>Non-Methylation code ID 21166

0.000 0.000 0.000 0.017 0.000 0.006 0.000 0.000 0.006 0.012

0.000 0.006 0.006 0.000 0.006 0.006 0.006 0.000 0.000 0.000

0.072 0.067 0.061 0.063 0.000 0.000 0.000 0.013 0.000 0.006

0.000 0.000 0.013 0.006 0.000 0.006 0.006 0.006 0.006 0.006

0.006 0.000 0.000 0.000 0.072 0.069 0.070 0.044 0.005 0.000

0.000 0.010 0.000 0.000 0.000 0.000 0.005 0.010 0.000 0.005

0.000 0.000 0.015 0.000 0.005 0.000 0.005 0.000 0.077 0.081

0.076 0.039

>Non-Methylation code ID 21166

0.059 0.000 0.000 0.000 0.000 0.023 0.000 0.000 0.000 0.000

0.000 0.000 0.012 0.000 0.000 0.012 0.012 0.012 0.012 0.000

0.055 0.047 0.051 0.039 0.010 0.000 0.000 0.010 0.000 0.005

0.000 0.000 0.000 0.010 0.000 0.000 0.010 0.005 0.010 0.005

0.000 0.000 0.000 0.000 0.067 0.069 0.055 0.080 0.004 0.000

0.000 0.004 0.000 0.004 0.000 0.000 0.000 0.009 0.000 0.000

0.000 0.000 0.013 0.013 0.000 0.000 0.004 0.000 0.065 0.086

0.068 0.060

>Non-Methylation code ID 21206

0.000 0.000 0.000 0.006 0.000 0.000 0.006 0.012 0.000 0.006

0.000 0.000 0.006 0.000 0.012 0.012 0.006 0.000 0.000 0.006

0.047 0.074 0.076 0.066 0.000 0.000 0.000 0.006 0.000 0.006

0.000 0.000 0.006 0.012 0.000 0.000 0.006 0.000 0.006 0.012

0.012 0.006 0.006 0.000 0.076 0.051 0.065 0.064 0.000 0.000

0.007 0.000 0.000 0.007 0.007 0.000 0.007 0.013 0.000 0.007

0.007 0.007 0.000 0.013 0.000 0.007 0.000 0.000 0.068 0.071

0.056 0.058

>Non-Methylation code ID 21206

0.015 0.000 0.000 0.000 0.007 0.015 0.015 0.007 0.000 0.007

0.000 0.007 0.007 0.000 0.000 0.007 0.000 0.000 0.000 0.000

0.063 0.058 0.069 0.054 0.012 0.000 0.000 0.006 0.006 0.006

0.000 0.000 0.000 0.012 0.000 0.000 0.006 0.000 0.006 0.006

0.018 0.000 0.000 0.000 0.055 0.062 0.064 0.073 0.005 0.000

0.000 0.005 0.000 0.000 0.005 0.000 0.000 0.009 0.000 0.000

0.005 0.005 0.009 0.005 0.000 0.000 0.009 0.000 0.085 0.046

0.076 0.071

>Non-Methylation code ID 21206

0.005 0.000 0.005 0.005 0.000 0.005 0.000 0.000 0.000 0.014

0.000 0.000 0.000 0.000 0.014 0.009 0.000 0.000 0.000 0.000

0.059 0.082 0.087 0.050 0.022 0.000 0.000 0.000 0.000 0.007

0.000 0.000 0.000 0.007 0.000 0.000 0.007 0.000 0.000 0.015

0.007 0.007 0.015 0.000 0.059 0.042 0.076 0.070 0.008 0.008

0.000 0.008 0.000 0.023 0.000 0.000 0.000 0.008 0.000 0.000

0.031 0.000 0.008 0.000 0.000 0.000 0.000 0.000 0.045 0.061

0.060 0.076

>Non-Methylation code ID 21206

0.010 0.005 0.000 0.005 0.000 0.005 0.005 0.000 0.000 0.005

0.000 0.000 0.005 0.005 0.010 0.000 0.005 0.000 0.000 0.000

0.056 0.074 0.065 0.078 0.000 0.000 0.008 0.000 0.004 0.008

0.004 0.004 0.000 0.004 0.000 0.000 0.004 0.000 0.012 0.000

0.000 0.000 0.000 0.000 0.073 0.075 0.054 0.082 0.000 0.007

0.000 0.014 0.000 0.014 0.007 0.000 0.000 0.007 0.007 0.000

0.000 0.000 0.000 0.014 0.014 0.000 0.000 0.000 0.045 0.064

0.071 0.067

>Non-Methylation code ID 21206

0.009 0.000 0.004 0.004 0.004 0.000 0.000 0.000 0.000 0.009

0.000 0.000 0.000 0.000 0.017 0.004 0.000 0.000 0.000 0.000

0.062 0.081 0.076 0.063 0.021 0.000 0.000 0.000 0.000 0.007

0.000 0.000 0.000 0.014 0.000 0.000 0.007 0.000 0.000 0.021

0.007 0.000 0.014 0.000 0.064 0.046 0.065 0.066 0.007 0.007

0.000 0.000 0.007 0.020 0.000 0.000 0.000 0.007 0.000 0.000

0.026 0.000 0.007 0.000 0.000 0.000 0.000 0.000 0.047 0.067

0.065 0.075

>Non-Methylation code ID 21206

0.000 0.007 0.000 0.000 0.007 0.007 0.007 0.000 0.000 0.014

0.007 0.000 0.000 0.000 0.007 0.014 0.014 0.000 0.000 0.000

0.081 0.063 0.055 0.049 0.013 0.013 0.000 0.000 0.000 0.000

0.007 0.000 0.000 0.007 0.000 0.007 0.007 0.007 0.000 0.007

0.007 0.007 0.000 0.000 0.063 0.072 0.051 0.066 0.000 0.000

0.005 0.000 0.010 0.000 0.005 0.005 0.000 0.010 0.000 0.000

0.010 0.000 0.005 0.000 0.005 0.000 0.000 0.000 0.071 0.062

0.076 0.069

>Non-Methylation code ID 21206

0.000 0.000 0.000 0.000 0.021 0.007 0.000 0.000 0.000 0.014

0.000 0.000 0.000 0.000 0.007 0.021 0.014 0.000 0.000 0.000

0.069 0.064 0.045 0.073 0.006 0.006 0.000 0.000 0.006 0.000

0.006 0.000 0.000 0.013 0.000 0.006 0.006 0.006 0.000 0.013

0.000 0.006 0.000 0.000 0.068 0.069 0.055 0.065 0.000 0.000

0.005 0.000 0.021 0.000 0.000 0.005 0.000 0.011 0.000 0.000

0.005 0.000 0.005 0.000 0.005 0.005 0.000 0.000 0.066 0.065

0.075 0.065

>Non-Methylation code ID 21206

0.000 0.009 0.000 0.005 0.005 0.005 0.000 0.000 0.000 0.000

0.000 0.000 0.005 0.009 0.009 0.000 0.000 0.005 0.000 0.000

0.067 0.061 0.085 0.069 0.005 0.000 0.005 0.000 0.005 0.009

0.005 0.000 0.009 0.000 0.000 0.005 0.000 0.000 0.009 0.000

0.000 0.005 0.000 0.005 0.066 0.091 0.051 0.064 0.008 0.000

0.000 0.000 0.000 0.008 0.000 0.017 0.000 0.000 0.008 0.000

0.008 0.000 0.008 0.025 0.008 0.008 0.000 0.000 0.067 0.039

0.070 0.056

>Non-Methylation code ID 21206

0.000 0.005 0.000 0.005 0.005 0.000 0.000 0.005 0.000 0.005

0.000 0.000 0.010 0.005 0.010 0.005 0.010 0.000 0.000 0.000

0.066 0.061 0.063 0.075 0.004 0.004 0.000 0.008 0.000 0.000

0.004 0.000 0.004 0.008 0.000 0.000 0.008 0.000 0.008 0.004

0.000 0.000 0.000 0.000 0.074 0.060 0.061 0.085 0.007 0.000

0.013 0.000 0.000 0.000 0.000 0.007 0.007 0.007 0.000 0.007

0.013 0.007 0.000 0.007 0.000 0.000 0.000 0.000 0.061 0.067

0.053 0.079

>Non-Methylation code ID 21206

0.000 0.005 0.000 0.005 0.000 0.010 0.000 0.000 0.000 0.000

0.000 0.000 0.005 0.005 0.005 0.005 0.010 0.000 0.010 0.000

0.065 0.073 0.080 0.053 0.023 0.008 0.000 0.000 0.000 0.030

0.000 0.000 0.000 0.008 0.000 0.000 0.008 0.008 0.008 0.008

0.000 0.000 0.000 0.000 0.073 0.053 0.044 0.065 0.006 0.000

0.013 0.000 0.000 0.013 0.000 0.000 0.000 0.013 0.000 0.000

0.000 0.006 0.013 0.000 0.000 0.000 0.000 0.000 0.055 0.079

0.060 0.077

>Non-Methylation code ID 21206

0.009 0.000 0.000 0.005 0.000 0.000 0.005 0.000 0.000 0.005

0.000 0.000 0.000 0.005 0.009 0.009 0.005 0.000 0.009 0.000

0.076 0.046 0.069 0.084 0.013 0.000 0.000 0.000 0.007 0.013

0.013 0.007 0.000 0.007 0.000 0.007 0.013 0.000 0.000 0.007

0.000 0.000 0.000 0.000 0.068 0.052 0.070 0.056 0.012 0.000

0.000 0.000 0.006 0.006 0.006 0.006 0.000 0.012 0.000 0.000

0.006 0.000 0.006 0.006 0.006 0.000 0.000 0.000 0.058 0.067

0.060 0.075

>Non-Methylation code ID 21206

0.000 0.004 0.000 0.000 0.004 0.004 0.008 0.000 0.000 0.000

0.000 0.008 0.013 0.004 0.008 0.000 0.000 0.000 0.000 0.000

0.070 0.077 0.066 0.066 0.026 0.000 0.000 0.000 0.006 0.000

0.006 0.000 0.000 0.000 0.000 0.000 0.013 0.000 0.006 0.000

0.026 0.000 0.000 0.000 0.053 0.066 0.068 0.061 0.000 0.000

0.000 0.000 0.000 0.009 0.000 0.000 0.000 0.018 0.000 0.000

0.053 0.018 0.000 0.000 0.009 0.000 0.000 0.000 0.069 0.048

0.064 0.047

>Non-Methylation code ID 21206

0.012 0.000 0.000 0.000 0.000 0.018 0.000 0.000 0.000 0.006

0.000 0.000 0.000 0.006 0.012 0.018 0.000 0.000 0.006 0.000

0.060 0.076 0.050 0.070 0.025 0.000 0.006 0.000 0.000 0.013

0.000 0.000 0.000 0.000 0.000 0.000 0.006 0.000 0.013 0.000

0.000 0.006 0.000 0.000 0.053 0.082 0.067 0.062 0.006 0.000

0.000 0.006 0.000 0.012 0.000 0.000 0.000 0.006 0.000 0.000

0.006 0.012 0.006 0.006 0.006 0.000 0.006 0.000 0.071 0.064

0.063 0.063

>Non-Methylation code ID 21206

0.000 0.004 0.000 0.000 0.000 0.004 0.009 0.000 0.000 0.004

0.000 0.009 0.013 0.004 0.009 0.000 0.000 0.000 0.000 0.000

0.065 0.075 0.067 0.068 0.027 0.000 0.000 0.000 0.000 0.000

0.007 0.000 0.000 0.000 0.000 0.000 0.013 0.000 0.007 0.000

0.027 0.000 0.000 0.007 0.062 0.064 0.060 0.060 0.000 0.000

0.000 0.000 0.000 0.009 0.000 0.000 0.000 0.009 0.000 0.000

0.054 0.018 0.000 0.000 0.018 0.000 0.000 0.000 0.067 0.054

0.058 0.045

>Non-Methylation code ID 21206

0.005 0.005 0.000 0.005 0.000 0.010 0.000 0.005 0.016 0.010

0.005 0.000 0.000 0.000 0.000 0.005 0.000 0.000 0.000 0.000

0.077 0.049 0.067 0.073 0.004 0.004 0.000 0.004 0.004 0.004

0.000 0.000 0.004 0.000 0.000 0.000 0.009 0.000 0.009 0.004

0.000 0.000 0.000 0.000 0.069 0.060 0.085 0.072 0.005 0.000

0.000 0.005 0.005 0.010 0.000 0.000 0.010 0.005 0.000 0.005

0.000 0.000 0.010 0.000 0.000 0.005 0.000 0.000 0.081 0.093

0.044 0.053

>Non-Methylation code ID 21206

0.000 0.007 0.000 0.000 0.014 0.007 0.007 0.007 0.000 0.014

0.000 0.000 0.007 0.007 0.000 0.000 0.007 0.007 0.000 0.007

0.044 0.056 0.061 0.081 0.006 0.006 0.000 0.000 0.006 0.006

0.006 0.013 0.000 0.000 0.000 0.006 0.006 0.000 0.000 0.019

0.000 0.000 0.000 0.000 0.063 0.063 0.065 0.066 0.006 0.000

0.000 0.000 0.006 0.000 0.000 0.000 0.000 0.022 0.000 0.006

0.000 0.000 0.011 0.000 0.011 0.000 0.000 0.006 0.060 0.052

0.073 0.081

>Non-Methylation code ID 21206

0.005 0.005 0.000 0.005 0.000 0.011 0.000 0.000 0.000 0.005

0.000 0.000 0.005 0.011 0.005 0.005 0.005 0.000 0.005 0.000

0.069 0.063 0.069 0.063 0.032 0.000 0.000 0.000 0.000 0.016

0.000 0.000 0.000 0.008 0.000 0.000 0.000 0.008 0.008 0.024

0.000 0.000 0.008 0.000 0.063 0.060 0.045 0.061 0.022 0.000

0.006 0.000 0.000 0.006 0.000 0.000 0.000 0.006 0.000 0.000

0.006 0.000 0.011 0.000 0.000 0.000 0.000 0.000 0.055 0.070

0.076 0.077

>Non-Methylation code ID 21206

0.016 0.000 0.011 0.000 0.000 0.005 0.000 0.000 0.000 0.011

0.000 0.000 0.000 0.005 0.011 0.000 0.000 0.000 0.000 0.000

0.060 0.091 0.057 0.068 0.000 0.005 0.000 0.005 0.000 0.010

0.000 0.000 0.000 0.000 0.000 0.000 0.005 0.010 0.005 0.005

0.010 0.000 0.010 0.000 0.067 0.073 0.079 0.049 0.040 0.000

0.000 0.000 0.000 0.054 0.000 0.000 0.000 0.013 0.000 0.000

0.013 0.013 0.000 0.027 0.000 0.000 0.000 0.000 0.051 0.041

0.038 0.042

>Non-Methylation code ID 21206

0.010 0.000 0.000 0.000 0.000 0.000 0.005 0.005 0.000 0.000

0.000 0.000 0.005 0.000 0.015 0.010 0.010 0.000 0.000 0.005

0.056 0.079 0.065 0.067 0.000 0.000 0.000 0.008 0.000 0.017

0.000 0.000 0.000 0.008 0.000 0.000 0.033 0.008 0.000 0.008

0.017 0.008 0.000 0.000 0.063 0.071 0.039 0.053 0.000 0.006

0.000 0.000 0.000 0.006 0.006 0.000 0.006 0.006 0.000 0.006

0.012 0.012 0.000 0.006 0.000 0.006 0.000 0.000 0.068 0.066

0.055 0.071

>Non-Methylation code ID 21206

0.000 0.000 0.000 0.007 0.000 0.027 0.007 0.000 0.007 0.007

0.007 0.000 0.007 0.000 0.000 0.007 0.000 0.000 0.000 0.007

0.045 0.061 0.071 0.074 0.022 0.014 0.000 0.000 0.000 0.007

0.007 0.000 0.000 0.000 0.000 0.000 0.000 0.000 0.007 0.007

0.014 0.000 0.000 0.000 0.059 0.067 0.070 0.057 0.008 0.000

0.000 0.008 0.000 0.000 0.004 0.004 0.000 0.000 0.000 0.000

0.000 0.000 0.017 0.000 0.000 0.004 0.004 0.000 0.078 0.069

0.069 0.066

>Non-Methylation code ID 21206

0.000 0.000 0.007 0.007 0.000 0.007 0.007 0.000 0.000 0.014

0.000 0.007 0.007 0.007 0.000 0.014 0.007 0.000 0.000 0.000

0.059 0.061 0.063 0.065 0.000 0.000 0.000 0.013 0.000 0.000

0.006 0.013 0.006 0.006 0.000 0.000 0.006 0.000 0.006 0.013

0.006 0.000 0.000 0.000 0.060 0.071 0.073 0.053 0.000 0.000

0.000 0.000 0.000 0.000 0.000 0.000 0.005 0.016 0.000 0.005

0.000 0.000 0.011 0.011 0.005 0.005 0.005 0.000 0.076 0.063

0.066 0.064

>Non-Methylation code ID 21206

0.005 0.000 0.000 0.005 0.005 0.010 0.005 0.005 0.005 0.005

0.000 0.000 0.005 0.000 0.005 0.005 0.005 0.000 0.000 0.000

0.072 0.066 0.065 0.065 0.010 0.000 0.000 0.000 0.000 0.005

0.005 0.000 0.010 0.005 0.000 0.000 0.000 0.000 0.010 0.010

0.005 0.000 0.005 0.000 0.087 0.043 0.068 0.072 0.011 0.000

0.000 0.006 0.006 0.006 0.011 0.000 0.000 0.006 0.000 0.000

0.011 0.000 0.011 0.000 0.000 0.000 0.000 0.000 0.080 0.043

0.086 0.058

>Non-Methylation code ID 21206

0.000 0.000 0.000 0.008 0.016 0.000 0.008 0.008 0.000 0.016

0.000 0.000 0.008 0.008 0.000 0.000 0.016 0.008 0.000 0.008

0.044 0.057 0.061 0.070 0.000 0.006 0.000 0.000 0.006 0.000

0.006 0.012 0.000 0.006 0.000 0.006 0.006 0.000 0.006 0.018

0.000 0.000 0.000 0.000 0.070 0.064 0.071 0.059 0.006 0.000

0.006 0.000 0.006 0.000 0.000 0.000 0.000 0.018 0.000 0.006

0.000 0.000 0.006 0.006 0.012 0.000 0.000 0.006 0.053 0.057

0.074 0.079

>Non-Methylation code ID 21210

0.005 0.005 0.000 0.000 0.005 0.010 0.000 0.000 0.000 0.005

0.000 0.000 0.019 0.000 0.010 0.005 0.000 0.000 0.000 0.000

0.059 0.052 0.080 0.080 0.008 0.000 0.004 0.000 0.004 0.004

0.000 0.000 0.000 0.008 0.000 0.000 0.000 0.000 0.017 0.004

0.000 0.000 0.000 0.000 0.071 0.067 0.074 0.070 0.029 0.000

0.000 0.000 0.000 0.010 0.000 0.000 0.000 0.019 0.000 0.000

0.010 0.000 0.000 0.019 0.010 0.010 0.010 0.000 0.063 0.047

0.054 0.054

>Non-Methylation code ID 21210

0.027 0.000 0.000 0.000 0.000 0.000 0.007 0.000 0.000 0.000

0.000 0.000 0.013 0.000 0.007 0.000 0.027 0.000 0.000 0.007

0.062 0.064 0.060 0.060 0.000 0.000 0.000 0.000 0.000 0.008

0.000 0.000 0.000 0.017 0.000 0.000 0.050 0.017 0.000 0.000

0.017 0.000 0.000 0.000 0.067 0.052 0.060 0.046 0.000 0.005

0.000 0.000 0.000 0.005 0.009 0.000 0.000 0.000 0.000 0.009

0.014 0.005 0.009 0.000 0.000 0.000 0.000 0.000 0.065 0.078

0.065 0.068

>Non-Methylation code ID 21226

0.004 0.004 0.000 0.004 0.004 0.000 0.009 0.000 0.000 0.009

0.000 0.000 0.004 0.004 0.009 0.004 0.000 0.000 0.000 0.000

0.076 0.060 0.065 0.077 0.005 0.000 0.005 0.000 0.005 0.000

0.005 0.000 0.000 0.000 0.000 0.000 0.000 0.000 0.016 0.010

0.010 0.000 0.000 0.005 0.065 0.086 0.060 0.061 0.020 0.000

0.000 0.000 0.000 0.007 0.000 0.007 0.000 0.007 0.007 0.000

0.014 0.000 0.007 0.007 0.007 0.000 0.000 0.000 0.055 0.064

0.056 0.077

>Non-Methylation code ID 21226

0.021 0.000 0.011 0.000 0.000 0.021 0.000 0.021 0.000 0.011

0.011 0.000 0.000 0.000 0.000 0.011 0.011 0.011 0.000 0.000

0.059 0.056 0.049 0.043 0.000 0.005 0.005 0.005 0.000 0.005

0.000 0.000 0.000 0.009 0.000 0.000 0.005 0.000 0.009 0.005

0.005 0.000 0.005 0.000 0.078 0.059 0.067 0.074 0.000 0.005

0.005 0.000 0.000 0.010 0.005 0.005 0.000 0.000 0.000 0.005

0.005 0.000 0.005 0.005 0.000 0.000 0.000 0.005 0.074 0.049

0.076 0.077

>Non-Methylation code ID 21226

0.007 0.000 0.007 0.000 0.007 0.007 0.000 0.007 0.007 0.014

0.000 0.000 0.014 0.000 0.000 0.014 0.007 0.000 0.000 0.000

0.071 0.064 0.052 0.058 0.000 0.000 0.000 0.006 0.006 0.000

0.000 0.000 0.000 0.006 0.006 0.000 0.012 0.012 0.006 0.018

0.000 0.000 0.000 0.000 0.067 0.072 0.059 0.063 0.005 0.000

0.005 0.000 0.011 0.000 0.005 0.000 0.000 0.005 0.000 0.000

0.005 0.005 0.005 0.005 0.000 0.005 0.005 0.000 0.080 0.062

0.068 0.060

>Non-Methylation code ID 21226

0.000 0.000 0.000 0.005 0.010 0.000 0.000 0.000 0.000 0.010

0.000 0.005 0.000 0.010 0.010 0.000 0.000 0.010 0.005 0.000

0.072 0.059 0.067 0.073 0.000 0.000 0.000 0.006 0.011 0.011

0.000 0.000 0.000 0.006 0.000 0.006 0.000 0.000 0.006 0.017

0.006 0.000 0.006 0.000 0.072 0.073 0.058 0.057 0.031 0.000

0.000 0.010 0.010 0.010 0.000 0.000 0.000 0.021 0.000 0.000

0.010 0.000 0.000 0.010 0.010 0.000 0.000 0.000 0.043 0.065

0.064 0.048

>Non-Methylation code ID 21226

0.000 0.000 0.007 0.000 0.007 0.022 0.000 0.007 0.000 0.000

0.000 0.000 0.007 0.007 0.000 0.022 0.000 0.000 0.000 0.007

0.052 0.071 0.064 0.058 0.006 0.000 0.000 0.000 0.006 0.006

0.000 0.006 0.000 0.000 0.006 0.000 0.006 0.000 0.011 0.006

0.000 0.006 0.000 0.000 0.056 0.070 0.074 0.077 0.000 0.000

0.000 0.005 0.010 0.005 0.000 0.000 0.000 0.000 0.000 0.000

0.005 0.005 0.005 0.000 0.000 0.010 0.010 0.005 0.060 0.070

0.077 0.064

>Non-Methylation code ID 21226

0.000 0.006 0.000 0.006 0.012 0.000 0.006 0.000 0.000 0.012

0.006 0.000 0.012 0.000 0.000 0.006 0.006 0.000 0.000 0.000

0.069 0.071 0.054 0.066 0.000 0.005 0.000 0.000 0.016 0.000

0.000 0.005 0.000 0.005 0.000 0.000 0.000 0.010 0.010 0.005

0.000 0.005 0.000 0.000 0.056 0.078 0.072 0.066 0.000 0.000

0.014 0.000 0.007 0.000 0.000 0.000 0.000 0.007 0.000 0.000

0.007 0.000 0.007 0.034 0.000 0.007 0.000 0.000 0.049 0.072

0.067 0.064

>Non-Methylation code ID 21226

0.017 0.000 0.009 0.000 0.000 0.009 0.000 0.000 0.009 0.017

0.000 0.017 0.000 0.000 0.000 0.026 0.009 0.000 0.000 0.000

0.040 0.056 0.062 0.065 0.000 0.000 0.000 0.005 0.000 0.000

0.000 0.005 0.000 0.005 0.000 0.000 0.010 0.005 0.010 0.000

0.010 0.005 0.005 0.000 0.067 0.072 0.060 0.075 0.000 0.000

0.000 0.012 0.012 0.006 0.000 0.000 0.000 0.006 0.000 0.006

0.000 0.012 0.006 0.006 0.000 0.000 0.006 0.000 0.074 0.077

0.055 0.055

>Non-Methylation code ID 21226

0.007 0.000 0.000 0.007 0.000 0.014 0.000 0.000 0.000 0.022

0.007 0.000 0.007 0.000 0.000 0.007 0.000 0.007 0.014 0.000

0.067 0.056 0.050 0.067 0.013 0.000 0.000 0.000 0.006 0.019

0.000 0.000 0.000 0.013 0.000 0.000 0.000 0.006 0.006 0.000

0.000 0.000 0.013 0.000 0.058 0.073 0.061 0.066 0.005 0.005

0.005 0.005 0.005 0.009 0.000 0.000 0.000 0.009 0.000 0.000

0.005 0.000 0.005 0.005 0.000 0.000 0.000 0.000 0.064 0.074

0.070 0.070

>Non-Methylation code ID 21226

0.007 0.000 0.000 0.000 0.000 0.007 0.000 0.014 0.000 0.014

0.000 0.000 0.021 0.000 0.007 0.000 0.014 0.000 0.000 0.000

0.062 0.077 0.057 0.054 0.005 0.000 0.000 0.000 0.005 0.000

0.010 0.000 0.000 0.010 0.000 0.000 0.000 0.005 0.015 0.005

0.000 0.005 0.000 0.000 0.068 0.063 0.067 0.075 0.012 0.000

0.006 0.000 0.000 0.000 0.006 0.000 0.000 0.000 0.000 0.006

0.000 0.000 0.012 0.012 0.012 0.000 0.000 0.006 0.082 0.053

0.066 0.061

>Non-Methylation code ID 21226

0.008 0.000 0.000 0.008 0.000 0.008 0.008 0.000 0.008 0.030

0.000 0.000 0.000 0.008 0.000 0.015 0.008 0.000 0.000 0.000

0.070 0.050 0.040 0.075 0.005 0.000 0.000 0.000 0.000 0.000

0.000 0.000 0.000 0.005 0.000 0.000 0.005 0.000 0.015 0.010

0.005 0.005 0.015 0.000 0.050 0.054 0.085 0.081 0.009 0.000

0.009 0.009 0.000 0.027 0.000 0.000 0.000 0.018 0.000 0.000

0.009 0.009 0.000 0.018 0.000 0.000 0.000 0.000 0.063 0.050

0.053 0.061

>Non-Methylation code ID 21226

0.005 0.000 0.005 0.000 0.005 0.000 0.000 0.005 0.000 0.000

0.000 0.000 0.000 0.000 0.018 0.009 0.009 0.000 0.000 0.000

0.072 0.083 0.067 0.056 0.024 0.000 0.000 0.000 0.000 0.008

0.000 0.000 0.000 0.008 0.008 0.000 0.016 0.000 0.008 0.016

0.000 0.008 0.000 0.000 0.061 0.068 0.047 0.062 0.005 0.005

0.005 0.005 0.005 0.000 0.009 0.000 0.000 0.000 0.000 0.000

0.009 0.005 0.009 0.000 0.000 0.000 0.000 0.000 0.082 0.059

0.069 0.069

>Non-Methylation code ID 21226

0.000 0.000 0.000 0.011 0.011 0.005 0.000 0.000 0.000 0.005

0.000 0.005 0.000 0.011 0.011 0.005 0.000 0.000 0.005 0.000

0.066 0.084 0.059 0.055 0.023 0.000 0.008 0.000 0.000 0.008

0.000 0.000 0.008 0.008 0.000 0.016 0.000 0.000 0.000 0.023

0.008 0.000 0.000 0.000 0.045 0.051 0.063 0.072 0.000 0.000

0.000 0.005 0.000 0.000 0.000 0.005 0.000 0.005 0.000 0.000

0.011 0.005 0.011 0.000 0.011 0.005 0.000 0.000 0.070 0.079

0.057 0.067

>Non-Methylation code ID 21226

0.000 0.000 0.000 0.007 0.000 0.022 0.000 0.000 0.007 0.015

0.000 0.000 0.000 0.000 0.007 0.015 0.015 0.007 0.000 0.000

0.078 0.043 0.057 0.059 0.005 0.000 0.005 0.000 0.000 0.011

0.005 0.000 0.005 0.005 0.000 0.000 0.000 0.000 0.011 0.005

0.000 0.000 0.000 0.000 0.043 0.069 0.082 0.087 0.000 0.000

0.010 0.010 0.000 0.005 0.000 0.000 0.000 0.000 0.000 0.000

0.000 0.005 0.010 0.000 0.005 0.000 0.010 0.000 0.075 0.066

0.063 0.071

>Non-Methylation code ID 21226

0.006 0.006 0.006 0.006 0.012 0.000 0.006 0.000 0.000 0.006

0.000 0.000 0.006 0.000 0.000 0.012 0.006 0.006 0.000 0.000

0.066 0.069 0.057 0.063 0.014 0.000 0.000 0.000 0.007 0.000

0.000 0.007 0.000 0.027 0.000 0.000 0.007 0.000 0.007 0.014

0.007 0.000 0.000 0.000 0.073 0.067 0.052 0.052 0.000 0.000

0.006 0.000 0.011 0.000 0.000 0.000 0.000 0.006 0.000 0.000

0.006 0.006 0.011 0.011 0.000 0.000 0.000 0.000 0.081 0.074

0.051 0.071

>Non-Methylation code ID 21226

0.007 0.007 0.000 0.015 0.000 0.000 0.000 0.000 0.007 0.007

0.007 0.000 0.007 0.015 0.000 0.007 0.007 0.000 0.000 0.000

0.077 0.062 0.052 0.054 0.004 0.000 0.000 0.004 0.004 0.000

0.000 0.000 0.004 0.000 0.000 0.000 0.008 0.004 0.013 0.004

0.000 0.004 0.004 0.000 0.088 0.058 0.086 0.047 0.000 0.000

0.011 0.000 0.000 0.011 0.000 0.000 0.011 0.006 0.000 0.000

0.011 0.000 0.006 0.006 0.000 0.000 0.000 0.000 0.087 0.045

0.090 0.049

>Non-Methylation code ID 21226

0.005 0.000 0.005 0.000 0.005 0.000 0.005 0.005 0.000 0.000

0.000 0.000 0.000 0.000 0.014 0.009 0.009 0.000 0.000 0.000

0.064 0.077 0.068 0.067 0.022 0.000 0.000 0.000 0.000 0.007

0.000 0.000 0.000 0.007 0.007 0.000 0.015 0.000 0.007 0.015

0.007 0.000 0.000 0.000 0.060 0.068 0.058 0.059 0.005 0.005

0.005 0.005 0.005 0.000 0.009 0.000 0.000 0.005 0.000 0.000

0.005 0.005 0.009 0.000 0.000 0.000 0.000 0.000 0.083 0.071

0.056 0.070

>Non-Methylation code ID 21226

0.004 0.000 0.004 0.000 0.000 0.004 0.004 0.000 0.000 0.004

0.000 0.000 0.004 0.000 0.013 0.004 0.004 0.004 0.004 0.000

0.045 0.060 0.086 0.087 0.000 0.000 0.015 0.008 0.000 0.015

0.000 0.000 0.000 0.015 0.008 0.000 0.008 0.008 0.000 0.015

0.008 0.000 0.000 0.000 0.067 0.051 0.049 0.067 0.009 0.000

0.000 0.000 0.000 0.009 0.009 0.000 0.009 0.018 0.000 0.000

0.000 0.009 0.000 0.018 0.018 0.000 0.000 0.000 0.063 0.062

0.044 0.063

>Non-Methylation code ID 21226

0.007 0.007 0.000 0.007 0.007 0.000 0.000 0.000 0.007 0.007

0.000 0.000 0.000 0.007 0.000 0.015 0.022 0.000 0.000 0.000

0.067 0.055 0.064 0.058 0.000 0.000 0.000 0.005 0.000 0.000

0.000 0.000 0.005 0.005 0.000 0.000 0.014 0.000 0.009 0.014

0.000 0.009 0.000 0.000 0.075 0.057 0.062 0.077 0.000 0.000

0.005 0.000 0.010 0.005 0.005 0.000 0.005 0.005 0.000 0.000

0.010 0.005 0.005 0.000 0.000 0.000 0.000 0.000 0.089 0.053

0.065 0.070

>Non-Methylation code ID 21226

0.007 0.000 0.000 0.007 0.000 0.007 0.014 0.000 0.000 0.007

0.000 0.000 0.007 0.021 0.007 0.000 0.000 0.007 0.000 0.000

0.061 0.061 0.052 0.077 0.006 0.006 0.000 0.000 0.000 0.000

0.000 0.000 0.006 0.000 0.000 0.000 0.006 0.000 0.013 0.025

0.013 0.000 0.000 0.000 0.068 0.072 0.058 0.059 0.030 0.010

0.000 0.000 0.000 0.020 0.000 0.000 0.000 0.000 0.000 0.000

0.050 0.000 0.000 0.010 0.000 0.000 0.000 0.000 0.053 0.081

0.042 0.036

>Non-Methylation code ID 21226

0.017 0.000 0.000 0.000 0.000 0.009 0.000 0.000 0.000 0.009

0.000 0.000 0.009 0.000 0.000 0.052 0.009 0.000 0.009 0.000

0.048 0.052 0.056 0.065 0.022 0.000 0.000 0.000 0.000 0.004

0.000 0.000 0.000 0.000 0.000 0.000 0.013 0.000 0.013 0.000

0.000 0.000 0.004 0.000 0.046 0.077 0.081 0.073 0.007 0.000

0.000 0.007 0.000 0.007 0.007 0.000 0.000 0.014 0.000 0.000

0.000 0.020 0.014 0.000 0.000 0.000 0.000 0.000 0.080 0.067

0.049 0.062

>Non-Methylation code ID 21226

0.011 0.005 0.000 0.000 0.000 0.000 0.000 0.000 0.000 0.000

0.000 0.000 0.005 0.000 0.011 0.021 0.016 0.000 0.000 0.000

0.070 0.066 0.075 0.052 0.027 0.000 0.000 0.000 0.000 0.005

0.000 0.000 0.000 0.000 0.000 0.000 0.027 0.000 0.011 0.000

0.000 0.000 0.000 0.000 0.057 0.066 0.072 0.069 0.000 0.000

0.000 0.000 0.000 0.006 0.013 0.000 0.000 0.000 0.000 0.000

0.019 0.019 0.006 0.000 0.000 0.006 0.000 0.000 0.066 0.052

0.087 0.058

>Non-Methylation code ID 21251

0.006 0.006 0.000 0.006 0.000 0.000 0.000 0.000 0.006 0.000

0.000 0.000 0.013 0.000 0.006 0.019 0.000 0.006 0.000 0.000

0.065 0.074 0.050 0.076 0.009 0.000 0.005 0.005 0.000 0.000

0.014 0.000 0.005 0.005 0.000 0.000 0.005 0.000 0.005 0.005

0.000 0.000 0.005 0.000 0.071 0.059 0.068 0.077 0.000 0.006

0.000 0.006 0.000 0.006 0.000 0.012 0.000 0.000 0.006 0.000

0.006 0.012 0.006 0.006 0.006 0.000 0.000 0.000 0.048 0.064

0.067 0.083

>Non-Methylation code ID 21251

0.020 0.014 0.000 0.000 0.000 0.007 0.000 0.000 0.000 0.000

0.000 0.007 0.014 0.007 0.000 0.000 0.000 0.014 0.000 0.000

0.063 0.063 0.073 0.053 0.008 0.000 0.000 0.000 0.000 0.000

0.000 0.000 0.000 0.000 0.000 0.000 0.008 0.000 0.017 0.004

0.004 0.000 0.004 0.004 0.067 0.082 0.063 0.071 0.005 0.005

0.000 0.000 0.000 0.015 0.000 0.000 0.000 0.010 0.000 0.000

0.005 0.005 0.010 0.000 0.005 0.000 0.000 0.000 0.044 0.070

0.082 0.075

>Non-Methylation code ID 21251

0.011 0.006 0.000 0.000 0.000 0.011 0.000 0.000 0.000 0.011

0.000 0.006 0.006 0.006 0.006 0.006 0.000 0.006 0.000 0.000

0.073 0.079 0.047 0.061 0.005 0.000 0.000 0.000 0.000 0.005

0.000 0.000 0.000 0.000 0.000 0.000 0.005 0.005 0.014 0.005

0.005 0.000 0.010 0.000 0.056 0.076 0.072 0.076 0.007 0.007

0.000 0.007 0.000 0.014 0.000 0.000 0.000 0.007 0.000 0.000

0.022 0.000 0.000 0.007 0.000 0.007 0.007 0.000 0.069 0.076

0.065 0.037

>Non-Methylation code ID 21251

0.017 0.008 0.000 0.000 0.000 0.008 0.000 0.000 0.000 0.017

0.000 0.008 0.017 0.008 0.000 0.000 0.000 0.017 0.000 0.000

0.059 0.063 0.057 0.054 0.010 0.000 0.000 0.000 0.000 0.000

0.000 0.000 0.005 0.000 0.000 0.000 0.010 0.000 0.010 0.019

0.005 0.000 0.005 0.000 0.069 0.071 0.065 0.067 0.005 0.000

0.000 0.000 0.000 0.010 0.000 0.000 0.000 0.005 0.000 0.000

0.014 0.005 0.014 0.005 0.000 0.000 0.000 0.000 0.058 0.074

0.077 0.066

>Non-Methylation code ID 21251

0.016 0.005 0.000 0.000 0.000 0.000 0.000 0.000 0.000 0.000

0.000 0.000 0.005 0.000 0.016 0.021 0.005 0.000 0.000 0.000

0.088 0.082 0.068 0.028 0.022 0.000 0.000 0.000 0.000 0.004

0.004 0.000 0.000 0.000 0.000 0.000 0.013 0.000 0.013 0.000

0.000 0.000 0.000 0.000 0.071 0.057 0.073 0.075 0.007 0.000

0.000 0.000 0.000 0.007 0.007 0.007 0.000 0.000 0.000 0.000

0.020 0.013 0.007 0.000 0.000 0.007 0.000 0.000 0.080 0.051

0.083 0.048

>Non-Methylation code ID 21251

0.012 0.000 0.000 0.000 0.000 0.024 0.000 0.006 0.006 0.006

0.000 0.000 0.000 0.000 0.012 0.000 0.006 0.000 0.000 0.000

0.068 0.055 0.077 0.062 0.010 0.005 0.000 0.005 0.005 0.010

0.000 0.000 0.005 0.000 0.000 0.000 0.005 0.010 0.005 0.000

0.000 0.000 0.000 0.000 0.065 0.070 0.067 0.073 0.000 0.000

0.005 0.000 0.000 0.009 0.005 0.000 0.005 0.009 0.000 0.000

0.000 0.005 0.009 0.005 0.000 0.000 0.005 0.000 0.085 0.056

0.068 0.068

>Non-Methylation code ID 21251

0.013 0.000 0.000 0.007 0.000 0.013 0.000 0.000 0.000 0.007

0.000 0.000 0.020 0.000 0.000 0.007 0.000 0.007 0.013 0.000

0.056 0.069 0.073 0.048 0.005 0.005 0.000 0.000 0.000 0.016

0.000 0.000 0.000 0.011 0.000 0.005 0.005 0.000 0.011 0.005

0.000 0.005 0.000 0.000 0.085 0.081 0.027 0.071 0.006 0.000

0.000 0.000 0.000 0.017 0.000 0.000 0.000 0.000 0.000 0.000

0.006 0.006 0.006 0.006 0.006 0.000 0.011 0.000 0.062 0.068

0.056 0.085

>Non-Methylation code ID 21251

0.012 0.000 0.000 0.000 0.000 0.012 0.000 0.006 0.000 0.012

0.000 0.000 0.000 0.006 0.012 0.000 0.006 0.000 0.000 0.006

0.076 0.073 0.059 0.053 0.004 0.004 0.000 0.004 0.009 0.009

0.000 0.000 0.004 0.004 0.000 0.000 0.004 0.004 0.004 0.000

0.004 0.000 0.000 0.000 0.073 0.065 0.060 0.079 0.011 0.000

0.006 0.000 0.000 0.006 0.006 0.000 0.006 0.017 0.000 0.000

0.000 0.006 0.006 0.006 0.000 0.000 0.000 0.000 0.067 0.057

0.077 0.065

>Non-Methylation code ID 21251

0.007 0.000 0.000 0.007 0.000 0.007 0.007 0.007 0.000 0.007

0.007 0.000 0.007 0.014 0.007 0.007 0.000 0.007 0.000 0.000

0.063 0.064 0.053 0.064 0.011 0.006 0.000 0.000 0.000 0.000

0.000 0.000 0.006 0.000 0.000 0.000 0.006 0.000 0.011 0.022

0.006 0.000 0.000 0.000 0.062 0.081 0.064 0.059 0.008 0.008

0.000 0.000 0.000 0.016 0.016 0.000 0.000 0.000 0.000 0.000

0.033 0.008 0.000 0.008 0.000 0.000 0.000 0.000 0.049 0.078

0.064 0.045

>Non-Methylation code ID 21251

0.025 0.000 0.000 0.000 0.000 0.016 0.000 0.000 0.000 0.008

0.000 0.000 0.008 0.008 0.000 0.033 0.000 0.000 0.008 0.000

0.056 0.061 0.050 0.060 0.012 0.000 0.000 0.000 0.000 0.008

0.000 0.000 0.000 0.004 0.000 0.000 0.004 0.004 0.012 0.004

0.000 0.000 0.004 0.000 0.056 0.082 0.080 0.066 0.011 0.006

0.000 0.006 0.000 0.006 0.000 0.000 0.000 0.006 0.000 0.000

0.000 0.006 0.011 0.000 0.000 0.011 0.000 0.000 0.071 0.065

0.066 0.071

>Non-Methylation code ID 21251

0.023 0.000 0.000 0.000 0.000 0.005 0.000 0.000 0.000 0.000

0.000 0.000 0.019 0.000 0.014 0.000 0.000 0.000 0.000 0.000

0.072 0.055 0.074 0.072 0.006 0.000 0.000 0.000 0.000 0.006

0.017 0.000 0.000 0.000 0.000 0.000 0.017 0.011 0.006 0.000

0.000 0.006 0.000 0.000 0.071 0.047 0.090 0.056 0.011 0.005

0.000 0.000 0.000 0.000 0.000 0.000 0.000 0.000 0.000 0.000

0.005 0.000 0.016 0.016 0.011 0.000 0.000 0.000 0.081 0.078

0.080 0.031

>Non-Methylation code ID 21259

0.012 0.006 0.000 0.000 0.000 0.000 0.000 0.000 0.000 0.000

0.000 0.000 0.006 0.000 0.012 0.023 0.012 0.000 0.000 0.000

0.078 0.064 0.071 0.050 0.033 0.007 0.000 0.000 0.000 0.007

0.000 0.000 0.000 0.000 0.000 0.000 0.033 0.000 0.007 0.000

0.000 0.000 0.000 0.000 0.060 0.065 0.070 0.052 0.000 0.000

0.000 0.000 0.000 0.006 0.012 0.000 0.000 0.000 0.000 0.000

0.018 0.012 0.012 0.000 0.000 0.006 0.000 0.000 0.076 0.052

0.074 0.064

>Non-Methylation code ID 21259

0.000 0.000 0.000 0.006 0.000 0.006 0.012 0.000 0.000 0.000

0.000 0.000 0.012 0.017 0.012 0.000 0.000 0.006 0.000 0.000

0.063 0.062 0.075 0.064 0.012 0.006 0.000 0.000 0.000 0.000

0.000 0.000 0.006 0.000 0.000 0.000 0.006 0.000 0.012 0.018

0.012 0.000 0.000 0.000 0.065 0.063 0.065 0.069 0.022 0.007

0.000 0.000 0.000 0.015 0.000 0.000 0.000 0.000 0.000 0.000

0.037 0.000 0.007 0.000 0.000 0.000 0.000 0.000 0.070 0.060

0.052 0.062

>Non-Methylation code ID 21259

0.012 0.006 0.000 0.000 0.000 0.000 0.000 0.000 0.006 0.000

0.000 0.000 0.006 0.000 0.012 0.018 0.012 0.000 0.000 0.000

0.065 0.063 0.065 0.069 0.021 0.007 0.000 0.000 0.000 0.014

0.000 0.000 0.000 0.000 0.000 0.000 0.034 0.000 0.007 0.007

0.000 0.000 0.000 0.000 0.065 0.061 0.059 0.058 0.000 0.000

0.000 0.007 0.000 0.007 0.014 0.000 0.000 0.000 0.000 0.000

0.014 0.021 0.007 0.000 0.000 0.007 0.000 0.000 0.059 0.056

0.071 0.070

>Non-Methylation code ID 21259

0.006 0.000 0.000 0.000 0.000 0.006 0.012 0.006 0.000 0.000

0.000 0.000 0.018 0.012 0.006 0.000 0.000 0.006 0.000 0.000

0.075 0.053 0.084 0.051 0.010 0.005 0.000 0.000 0.000 0.000

0.000 0.000 0.000 0.000 0.000 0.000 0.005 0.000 0.016 0.021

0.010 0.000 0.000 0.000 0.087 0.075 0.076 0.029 0.024 0.000

0.000 0.000 0.000 0.005 0.000 0.000 0.000 0.000 0.000 0.000

0.014 0.000 0.014 0.000 0.000 0.000 0.000 0.000 0.072 0.054

0.073 0.076

>Non-Methylation code ID 21259

0.013 0.000 0.000 0.007 0.000 0.013 0.000 0.000 0.000 0.020

0.000 0.000 0.007 0.000 0.013 0.007 0.000 0.007 0.000 0.000

0.044 0.062 0.068 0.074 0.011 0.000 0.000 0.000 0.000 0.005

0.000 0.000 0.000 0.000 0.000 0.005 0.011 0.005 0.005 0.005

0.000 0.000 0.011 0.000 0.069 0.071 0.075 0.060 0.000 0.000

0.000 0.000 0.000 0.012 0.000 0.006 0.000 0.012 0.000 0.000

0.012 0.000 0.012 0.006 0.000 0.006 0.006 0.000 0.085 0.035

0.064 0.079

>Non-Methylation code ID 21259

0.017 0.000 0.000 0.000 0.000 0.006 0.000 0.000 0.000 0.011

0.000 0.000 0.006 0.006 0.011 0.006 0.006 0.000 0.000 0.006

0.060 0.062 0.060 0.080 0.005 0.005 0.000 0.000 0.005 0.010

0.000 0.000 0.000 0.010 0.000 0.000 0.015 0.005 0.005 0.000

0.005 0.000 0.000 0.000 0.071 0.066 0.054 0.079 0.010 0.000

0.005 0.000 0.005 0.005 0.000 0.000 0.000 0.010 0.000 0.000

0.000 0.005 0.015 0.005 0.000 0.000 0.000 0.000 0.063 0.080

0.062 0.068

>Non-Methylation code ID 21259

0.017 0.000 0.006 0.000 0.000 0.028 0.000 0.006 0.006 0.000

0.006 0.000 0.000 0.000 0.006 0.000 0.000 0.000 0.000 0.000

0.070 0.048 0.060 0.082 0.010 0.000 0.000 0.010 0.005 0.005

0.000 0.000 0.000 0.000 0.005 0.000 0.010 0.005 0.005 0.000

0.000 0.000 0.005 0.000 0.059 0.060 0.076 0.077 0.000 0.004

0.000 0.000 0.000 0.012 0.004 0.000 0.000 0.000 0.000 0.000

0.004 0.004 0.012 0.004 0.000 0.000 0.004 0.000 0.089 0.051

0.068 0.076

>Non-Methylation code ID 21259

0.007 0.000 0.000 0.000 0.000 0.007 0.000 0.000 0.000 0.020

0.000 0.007 0.007 0.007 0.013 0.007 0.000 0.000 0.000 0.000

0.083 0.050 0.049 0.079 0.000 0.009 0.004 0.004 0.000 0.000

0.000 0.004 0.000 0.000 0.000 0.009 0.004 0.000 0.004 0.000

0.000 0.009 0.009 0.000 0.090 0.045 0.074 0.067 0.024 0.000

0.000 0.000 0.012 0.024 0.000 0.000 0.000 0.012 0.000 0.000

0.000 0.012 0.000 0.012 0.036 0.000 0.000 0.000 0.062 0.050

0.056 0.035

>Non-Methylation code ID 21259

0.012 0.006 0.000 0.000 0.000 0.018 0.006 0.000 0.000 0.000

0.000 0.000 0.006 0.012 0.006 0.006 0.006 0.000 0.000 0.000

0.064 0.059 0.073 0.060 0.018 0.000 0.006 0.006 0.000 0.000

0.000 0.000 0.000 0.012 0.000 0.000 0.006 0.000 0.012 0.012

0.006 0.000 0.000 0.000 0.072 0.062 0.059 0.062 0.015 0.000

0.000 0.000 0.000 0.005 0.000 0.000 0.000 0.010 0.000 0.000

0.000 0.000 0.020 0.005 0.000 0.000 0.000 0.005 0.051 0.075

0.082 0.066

>Non-Methylation code ID 21259

0.007 0.000 0.000 0.000 0.014 0.000 0.007 0.000 0.020 0.007

0.000 0.000 0.000 0.007 0.000 0.014 0.014 0.000 0.000 0.000

0.060 0.079 0.057 0.049 0.000 0.005 0.000 0.000 0.005 0.000

0.000 0.005 0.015 0.000 0.000 0.000 0.005 0.005 0.010 0.010

0.000 0.005 0.000 0.000 0.074 0.070 0.042 0.084 0.008 0.000

0.000 0.008 0.008 0.000 0.000 0.000 0.015 0.000 0.000 0.008

0.000 0.008 0.000 0.015 0.000 0.015 0.000 0.008 0.058 0.066

0.054 0.064

>Non-Methylation code ID 21259

0.009 0.000 0.000 0.000 0.000 0.014 0.005 0.000 0.000 0.005

0.000 0.000 0.005 0.000 0.014 0.005 0.000 0.000 0.000 0.000

0.058 0.072 0.079 0.067 0.039 0.000 0.008 0.008 0.000 0.000

0.000 0.000 0.000 0.016 0.000 0.000 0.000 0.000 0.008 0.008

0.008 0.000 0.000 0.000 0.064 0.080 0.048 0.047 0.000 0.000

0.000 0.005 0.000 0.005 0.000 0.000 0.000 0.000 0.000 0.000

0.019 0.000 0.019 0.005 0.005 0.000 0.000 0.000 0.063 0.076

0.057 0.082

>Non-Methylation code ID 21259

0.013 0.000 0.000 0.000 0.000 0.000 0.000 0.000 0.000 0.020

0.000 0.007 0.007 0.007 0.007 0.013 0.007 0.007 0.000 0.000

0.055 0.054 0.073 0.066 0.016 0.005 0.000 0.000 0.000 0.000

0.000 0.000 0.000 0.011 0.000 0.005 0.000 0.000 0.011 0.011

0.005 0.000 0.000 0.005 0.079 0.071 0.039 0.073 0.014 0.007

0.000 0.000 0.000 0.007 0.007 0.000 0.000 0.000 0.000 0.000

0.007 0.021 0.007 0.000 0.014 0.000 0.000 0.000 0.074 0.068

0.062 0.047

>Non-Methylation code ID 21259

0.000 0.007 0.000 0.000 0.007 0.000 0.028 0.000 0.000 0.000

0.000 0.000 0.014 0.000 0.000 0.007 0.028 0.000 0.000 0.000

0.068 0.049 0.075 0.049 0.008 0.000 0.000 0.000 0.008 0.000

0.033 0.000 0.000 0.017 0.000 0.000 0.008 0.000 0.000 0.000

0.033 0.000 0.000 0.000 0.066 0.040 0.073 0.046 0.000 0.000

0.000 0.000 0.000 0.000 0.049 0.000 0.000 0.025 0.000 0.000

0.000 0.000 0.000 0.025 0.049 0.000 0.000 0.000 0.060 0.028

0.057 0.039

>Non-Methylation code ID 21259

0.005 0.000 0.000 0.000 0.005 0.000 0.000 0.005 0.000 0.005

0.000 0.005 0.005 0.000 0.010 0.015 0.000 0.000 0.000 0.000

0.057 0.069 0.073 0.077 0.000 0.000 0.004 0.004 0.004 0.004

0.009 0.000 0.000 0.000 0.000 0.000 0.000 0.000 0.013 0.009

0.004 0.004 0.000 0.000 0.084 0.064 0.071 0.056 0.007 0.000

0.007 0.000 0.000 0.000 0.000 0.007 0.007 0.007 0.000 0.000

0.007 0.007 0.007 0.007 0.007 0.007 0.000 0.000 0.066 0.071

0.062 0.052

>Non-Methylation code ID 21259

0.009 0.000 0.000 0.000 0.000 0.009 0.005 0.000 0.000 0.005

0.000 0.000 0.009 0.000 0.014 0.005 0.000 0.000 0.000 0.000

0.057 0.073 0.092 0.055 0.041 0.000 0.008 0.000 0.000 0.000

0.000 0.000 0.000 0.016 0.000 0.000 0.008 0.000 0.008 0.008

0.008 0.000 0.000 0.000 0.065 0.074 0.053 0.043 0.000 0.000

0.000 0.005 0.000 0.005 0.000 0.000 0.000 0.000 0.000 0.000

0.020 0.000 0.015 0.010 0.005 0.000 0.000 0.000 0.067 0.076

0.056 0.075

>Non-Methylation code ID 21259

0.000 0.000 0.000 0.014 0.007 0.007 0.007 0.000 0.000 0.007

0.000 0.000 0.007 0.000 0.000 0.007 0.020 0.000 0.007 0.000

0.049 0.060 0.075 0.069 0.000 0.000 0.000 0.006 0.000 0.006

0.006 0.000 0.000 0.000 0.000 0.006 0.018 0.000 0.006 0.018

0.006 0.000 0.000 0.000 0.056 0.077 0.064 0.062 0.006 0.000

0.006 0.000 0.000 0.006 0.006 0.000 0.000 0.018 0.000 0.006

0.000 0.000 0.006 0.000 0.006 0.012 0.000 0.000 0.065 0.067

0.064 0.064

>Non-Methylation code ID 21259

0.000 0.000 0.000 0.005 0.000 0.000 0.005 0.005 0.016 0.016

0.000 0.005 0.000 0.000 0.005 0.011 0.000 0.000 0.000 0.000

0.069 0.079 0.061 0.055 0.006 0.000 0.000 0.000 0.000 0.006

0.000 0.006 0.000 0.000 0.000 0.012 0.006 0.000 0.012 0.006

0.006 0.000 0.000 0.006 0.066 0.093 0.054 0.054 0.000 0.000

0.007 0.007 0.007 0.007 0.000 0.007 0.014 0.007 0.000 0.000

0.007 0.014 0.000 0.000 0.007 0.000 0.000 0.000 0.061 0.070

0.048 0.072

>Non-Methylation code ID 21259

0.000 0.007 0.000 0.000 0.013 0.000 0.007 0.000 0.000 0.013

0.000 0.000 0.020 0.000 0.000 0.020 0.007 0.000 0.000 0.000

0.066 0.091 0.053 0.036 0.007 0.007 0.000 0.000 0.013 0.000

0.000 0.007 0.000 0.013 0.000 0.000 0.033 0.000 0.000 0.000

0.000 0.000 0.000 0.007 0.057 0.053 0.050 0.087 0.000 0.000

0.000 0.000 0.014 0.000 0.007 0.000 0.000 0.014 0.007 0.000

0.020 0.000 0.000 0.020 0.000 0.000 0.000 0.000 0.073 0.055

0.059 0.065

>Non-Methylation code ID 21259

0.006 0.000 0.006 0.000 0.000 0.006 0.000 0.000 0.000 0.012

0.000 0.000 0.006 0.000 0.006 0.006 0.019 0.000 0.006 0.006

0.083 0.048 0.065 0.057 0.006 0.000 0.000 0.006 0.000 0.006

0.006 0.006 0.000 0.011 0.000 0.000 0.000 0.000 0.011 0.011

0.006 0.000 0.000 0.000 0.082 0.057 0.088 0.040 0.010 0.000

0.010 0.000 0.000 0.010 0.010 0.000 0.000 0.010 0.010 0.000

0.000 0.010 0.000 0.019 0.000 0.000 0.000 0.010 0.074 0.059

0.059 0.046

>Non-Methylation code ID 21259

0.005 0.000 0.000 0.000 0.000 0.009 0.000 0.005 0.000 0.000

0.000 0.005 0.005 0.000 0.018 0.000 0.005 0.000 0.000 0.005

0.070 0.092 0.054 0.061 0.000 0.000 0.013 0.013 0.006 0.013

0.000 0.006 0.006 0.006 0.000 0.000 0.000 0.013 0.000 0.000

0.006 0.000 0.000 0.000 0.063 0.065 0.054 0.068 0.007 0.000

0.000 0.007 0.000 0.000 0.007 0.000 0.013 0.013 0.000 0.007

0.000 0.000 0.007 0.013 0.007 0.000 0.000 0.000 0.069 0.067

0.059 0.058

>Non-Methylation code ID 21259

0.000 0.000 0.000 0.016 0.000 0.022 0.000 0.000 0.011 0.000

0.000 0.011 0.000 0.000 0.005 0.000 0.000 0.000 0.000 0.000

0.072 0.085 0.061 0.049 0.009 0.000 0.000 0.004 0.000 0.009

0.000 0.000 0.009 0.000 0.000 0.000 0.000 0.000 0.009 0.004

0.009 0.000 0.004 0.000 0.061 0.066 0.075 0.074 0.000 0.000

0.000 0.011 0.000 0.016 0.000 0.000 0.011 0.000 0.000 0.000

0.000 0.000 0.027 0.000 0.000 0.000 0.000 0.000 0.059 0.070

0.050 0.089

>Non-Methylation code ID 21259

0.021 0.000 0.000 0.007 0.000 0.000 0.000 0.000 0.000 0.014

0.000 0.007 0.007 0.000 0.007 0.014 0.007 0.007 0.000 0.000

0.063 0.055 0.073 0.051 0.016 0.000 0.000 0.000 0.000 0.000

0.000 0.000 0.000 0.010 0.000 0.000 0.000 0.000 0.021 0.010

0.005 0.000 0.000 0.005 0.049 0.075 0.073 0.070 0.006 0.006

0.000 0.000 0.000 0.012 0.006 0.000 0.000 0.000 0.000 0.000

0.006 0.018 0.006 0.006 0.006 0.000 0.000 0.000 0.067 0.064

0.073 0.058

>Non-Methylation code ID 21259

0.010 0.000 0.005 0.000 0.000 0.005 0.005 0.000 0.000 0.000

0.000 0.000 0.010 0.000 0.010 0.010 0.000 0.005 0.000 0.000

0.068 0.075 0.074 0.054 0.024 0.000 0.000 0.000 0.000 0.012

0.000 0.000 0.000 0.024 0.000 0.000 0.024 0.000 0.000 0.012

0.024 0.000 0.000 0.000 0.033 0.061 0.064 0.057 0.000 0.010

0.000 0.005 0.000 0.005 0.000 0.000 0.000 0.000 0.000 0.000

0.010 0.005 0.014 0.010 0.000 0.000 0.000 0.000 0.077 0.081

0.057 0.061

>Non-Methylation code ID 21259

0.000 0.000 0.007 0.013 0.007 0.007 0.000 0.013 0.000 0.007

0.000 0.000 0.007 0.007 0.000 0.000 0.020 0.000 0.000 0.000

0.062 0.069 0.059 0.057 0.005 0.000 0.000 0.000 0.000 0.000

0.005 0.000 0.005 0.021 0.000 0.010 0.000 0.000 0.010 0.005

0.005 0.000 0.000 0.000 0.076 0.070 0.037 0.083 0.000 0.000

0.005 0.000 0.000 0.005 0.000 0.005 0.000 0.000 0.000 0.005

0.005 0.000 0.010 0.000 0.010 0.000 0.000 0.010 0.066 0.077

0.069 0.066

>Non-Methylation code ID 21259

0.015 0.000 0.000 0.000 0.000 0.000 0.000 0.000 0.000 0.010

0.000 0.000 0.000 0.000 0.021 0.010 0.005 0.000 0.000 0.005

0.057 0.084 0.056 0.070 0.006 0.006 0.000 0.000 0.000 0.017

0.006 0.000 0.000 0.000 0.000 0.000 0.006 0.017 0.006 0.006

0.006 0.000 0.000 0.000 0.067 0.069 0.067 0.058 0.015 0.000

0.000 0.007 0.000 0.000 0.000 0.000 0.000 0.015 0.000 0.007

0.007 0.000 0.007 0.015 0.007 0.007 0.000 0.000 0.058 0.054

0.078 0.056

>Non-Methylation code ID 21259

0.000 0.000 0.000 0.000 0.008 0.000 0.025 0.000 0.000 0.025

0.000 0.000 0.008 0.000 0.000 0.017 0.025 0.000 0.000 0.000

0.065 0.058 0.055 0.047 0.000 0.000 0.000 0.000 0.007 0.000

0.022 0.000 0.000 0.000 0.000 0.000 0.022 0.000 0.000 0.022

0.022 0.000 0.000 0.000 0.062 0.060 0.060 0.054 0.000 0.000

0.000 0.000 0.009 0.000 0.017 0.000 0.000 0.017 0.000 0.000

0.034 0.000 0.000 0.000 0.026 0.000 0.000 0.000 0.049 0.045

0.061 0.075

>Non-Methylation code ID 21259

0.000 0.009 0.000 0.000 0.000 0.009 0.028 0.000 0.000 0.009

0.000 0.000 0.000 0.000 0.000 0.019 0.028 0.009 0.000 0.000

0.062 0.049 0.067 0.043 0.008 0.000 0.000 0.000 0.000 0.008

0.024 0.000 0.000 0.000 0.000 0.000 0.000 0.000 0.008 0.024

0.024 0.000 0.000 0.000 0.066 0.051 0.073 0.047 0.009 0.009

0.000 0.000 0.000 0.000 0.028 0.000 0.000 0.009 0.000 0.000

0.000 0.009 0.000 0.000 0.028 0.009 0.000 0.000 0.058 0.041

0.077 0.053

>Non-Methylation code ID 21259

0.000 0.000 0.000 0.000 0.022 0.022 0.000 0.000 0.000 0.044

0.000 0.000 0.000 0.000 0.000 0.011 0.011 0.000 0.000 0.011

0.040 0.054 0.063 0.055 0.000 0.012 0.006 0.000 0.006 0.006

0.000 0.000 0.000 0.000 0.000 0.006 0.006 0.006 0.000 0.018

0.006 0.000 0.000 0.000 0.055 0.067 0.064 0.076 0.000 0.000

0.000 0.000 0.000 0.000 0.000 0.000 0.000 0.017 0.000 0.006

0.000 0.000 0.017 0.006 0.011 0.011 0.000 0.000 0.074 0.054

0.078 0.060

>Non-Methylation code ID 21259

0.005 0.005 0.000 0.000 0.000 0.015 0.005 0.000 0.000 0.005

0.000 0.000 0.000 0.000 0.015 0.010 0.000 0.000 0.000 0.000

0.053 0.070 0.080 0.070 0.039 0.000 0.007 0.007 0.000 0.000

0.000 0.000 0.000 0.000 0.000 0.000 0.007 0.000 0.007 0.007

0.007 0.000 0.000 0.000 0.062 0.074 0.055 0.064 0.000 0.000

0.000 0.005 0.000 0.005 0.000 0.000 0.000 0.005 0.000 0.000

0.018 0.000 0.018 0.000 0.005 0.000 0.000 0.000 0.054 0.074

0.082 0.068

>Non-Methylation code ID 21259

0.000 0.000 0.000 0.000 0.008 0.000 0.025 0.000 0.000 0.017

0.000 0.000 0.033 0.000 0.000 0.000 0.025 0.000 0.000 0.000

0.051 0.043 0.063 0.070 0.000 0.000 0.000 0.000 0.008 0.000

0.025 0.000 0.000 0.025 0.000 0.000 0.008 0.000 0.000 0.017

0.025 0.000 0.000 0.000 0.065 0.058 0.055 0.047 0.000 0.000

0.000 0.000 0.008 0.000 0.023 0.000 0.000 0.000 0.000 0.000

0.023 0.000 0.000 0.015 0.023 0.000 0.000 0.000 0.064 0.056

0.064 0.058

>Non-Methylation code ID 21259

0.000 0.000 0.005 0.005 0.000 0.005 0.000 0.005 0.000 0.000

0.000 0.005 0.005 0.000 0.005 0.005 0.000 0.009 0.014 0.000

0.084 0.070 0.075 0.045 0.014 0.000 0.000 0.000 0.014 0.068

0.000 0.000 0.000 0.014 0.000 0.000 0.000 0.014 0.000 0.014

0.027 0.000 0.000 0.000 0.052 0.037 0.056 0.025 0.012 0.000

0.000 0.006 0.000 0.006 0.000 0.000 0.000 0.006 0.000 0.006

0.000 0.000 0.012 0.006 0.000 0.000 0.000 0.000 0.080 0.059

0.047 0.093

>Non-Methylation code ID 21259

0.000 0.000 0.000 0.000 0.013 0.000 0.007 0.000 0.000 0.020

0.007 0.000 0.020 0.000 0.000 0.020 0.000 0.000 0.000 0.000

0.073 0.056 0.056 0.063 0.000 0.007 0.000 0.000 0.013 0.000

0.007 0.000 0.000 0.013 0.000 0.000 0.020 0.000 0.000 0.020

0.007 0.000 0.000 0.000 0.066 0.091 0.053 0.036 0.008 0.000

0.000 0.000 0.016 0.000 0.000 0.008 0.000 0.016 0.000 0.000

0.040 0.000 0.000 0.000 0.000 0.000 0.000 0.008 0.047 0.054

0.049 0.088

>Non-Methylation code ID 21259

0.008 0.008 0.000 0.000 0.000 0.000 0.000 0.004 0.000 0.000

0.000 0.004 0.004 0.000 0.008 0.004 0.004 0.004 0.004 0.000

0.077 0.057 0.050 0.094 0.025 0.000 0.000 0.000 0.008 0.016

0.008 0.000 0.000 0.000 0.000 0.000 0.008 0.025 0.000 0.000

0.008 0.000 0.000 0.000 0.067 0.066 0.042 0.061 0.007 0.000

0.000 0.000 0.000 0.000 0.000 0.000 0.000 0.014 0.000 0.014

0.007 0.007 0.007 0.014 0.007 0.007 0.000 0.000 0.072 0.040

0.070 0.065

>Non-Methylation code ID 21259

0.000 0.000 0.006 0.006 0.000 0.006 0.006 0.006 0.000 0.000

0.000 0.011 0.000 0.000 0.006 0.006 0.006 0.006 0.000 0.006

0.067 0.071 0.067 0.059 0.000 0.000 0.007 0.007 0.007 0.007

0.000 0.013 0.007 0.000 0.000 0.000 0.013 0.000 0.000 0.000

0.020 0.000 0.000 0.000 0.061 0.071 0.058 0.065 0.000 0.000

0.000 0.000 0.006 0.006 0.000 0.000 0.006 0.011 0.000 0.011

0.006 0.006 0.011 0.006 0.000 0.000 0.000 0.000 0.080 0.054

0.063 0.069

>Non-Methylation code ID 21259

0.008 0.008 0.000 0.008 0.000 0.000 0.032 0.000 0.000 0.000

0.000 0.000 0.000 0.000 0.000 0.008 0.024 0.008 0.000 0.000

0.067 0.045 0.065 0.060 0.000 0.000 0.000 0.000 0.000 0.000

0.026 0.000 0.009 0.009 0.000 0.000 0.000 0.000 0.009 0.009

0.035 0.000 0.000 0.009 0.074 0.058 0.044 0.052 0.006 0.006

0.000 0.000 0.000 0.006 0.018 0.006 0.000 0.006 0.000 0.000

0.000 0.000 0.006 0.000 0.018 0.000 0.000 0.000 0.079 0.048

0.078 0.055

>Non-Methylation code ID 21259

0.000 0.000 0.000 0.000 0.000 0.000 0.044 0.011 0.000 0.011

0.000 0.000 0.011 0.000 0.000 0.011 0.044 0.000 0.000 0.000

0.064 0.025 0.070 0.042 0.008 0.008 0.000 0.008 0.000 0.000

0.033 0.000 0.000 0.000 0.000 0.000 0.000 0.008 0.000 0.008

0.033 0.000 0.000 0.000 0.072 0.036 0.078 0.038 0.009 0.000

0.000 0.000 0.000 0.000 0.036 0.000 0.009 0.009 0.000 0.009

0.000 0.000 0.000 0.000 0.036 0.000 0.000 0.000 0.076 0.027

0.068 0.054

>Non-Methylation code ID 21259

0.014 0.000 0.000 0.000 0.014 0.007 0.000 0.000 0.014 0.007

0.000 0.000 0.000 0.000 0.000 0.014 0.007 0.014 0.000 0.000

0.075 0.061 0.055 0.054 0.005 0.005 0.000 0.000 0.000 0.000

0.000 0.000 0.005 0.009 0.000 0.000 0.005 0.000 0.014 0.014

0.000 0.000 0.000 0.000 0.061 0.061 0.077 0.079 0.000 0.000

0.000 0.000 0.000 0.011 0.000 0.000 0.006 0.011 0.000 0.000

0.000 0.006 0.011 0.011 0.000 0.006 0.000 0.006 0.082 0.054

0.075 0.054

>Non-Methylation code ID 21285

0.006 0.000 0.000 0.006 0.000 0.000 0.006 0.000 0.012 0.018

0.000 0.006 0.000 0.000 0.006 0.012 0.006 0.000 0.000 0.000

0.076 0.076 0.045 0.058 0.005 0.000 0.000 0.000 0.000 0.005

0.000 0.005 0.000 0.000 0.000 0.005 0.005 0.000 0.018 0.000

0.005 0.000 0.000 0.009 0.064 0.089 0.065 0.062 0.000 0.000

0.007 0.007 0.007 0.014 0.000 0.007 0.007 0.007 0.000 0.000

0.000 0.014 0.000 0.000 0.014 0.000 0.000 0.000 0.068 0.063

0.053 0.064

>Non-Methylation code ID 21285

0.000 0.000 0.000 0.005 0.000 0.016 0.000 0.000 0.005 0.000

0.000 0.005 0.011 0.005 0.011 0.005 0.005 0.000 0.000 0.000

0.071 0.072 0.076 0.044 0.014 0.000 0.000 0.009 0.000 0.005

0.000 0.005 0.014 0.005 0.000 0.000 0.005 0.000 0.005 0.000

0.000 0.000 0.000 0.000 0.078 0.058 0.066 0.070 0.006 0.000

0.000 0.006 0.000 0.000 0.000 0.000 0.006 0.000 0.000 0.006

0.011 0.006 0.017 0.000 0.000 0.000 0.006 0.000 0.076 0.080

0.058 0.057

>Non-Methylation code ID 21285

0.027 0.000 0.000 0.000 0.000 0.013 0.000 0.000 0.000 0.000

0.000 0.000 0.007 0.000 0.007 0.013 0.013 0.000 0.007 0.000

0.056 0.068 0.063 0.059 0.014 0.000 0.007 0.000 0.000 0.014

0.000 0.000 0.000 0.007 0.000 0.000 0.042 0.000 0.007 0.000

0.000 0.000 0.000 0.000 0.064 0.052 0.062 0.063 0.000 0.000

0.000 0.011 0.000 0.000 0.011 0.000 0.000 0.000 0.005 0.000

0.000 0.000 0.033 0.000 0.000 0.005 0.000 0.000 0.043 0.058

0.085 0.082

>Non-Methylation code ID 21285

0.005 0.000 0.000 0.005 0.000 0.005 0.000 0.000 0.000 0.005

0.000 0.000 0.010 0.000 0.015 0.015 0.000 0.000 0.000 0.000

0.083 0.058 0.064 0.069 0.011 0.006 0.000 0.011 0.000 0.011

0.006 0.000 0.000 0.000 0.000 0.000 0.017 0.000 0.006 0.000

0.000 0.006 0.000 0.000 0.071 0.061 0.066 0.063 0.013 0.000

0.000 0.007 0.000 0.000 0.000 0.000 0.000 0.007 0.000 0.000

0.007 0.007 0.013 0.013 0.007 0.007 0.000 0.000 0.074 0.050

0.061 0.068

>Non-Methylation code ID 21285

0.009 0.009 0.000 0.004 0.000 0.004 0.004 0.000 0.000 0.004

0.000 0.000 0.004 0.000 0.009 0.004 0.000 0.004 0.000 0.000

0.077 0.054 0.073 0.071 0.014 0.005 0.000 0.000 0.000 0.000

0.000 0.000 0.000 0.005 0.005 0.000 0.000 0.000 0.014 0.010

0.000 0.005 0.000 0.000 0.058 0.082 0.069 0.067 0.020 0.013

0.000 0.000 0.000 0.013 0.000 0.000 0.000 0.020 0.000 0.000

0.007 0.000 0.007 0.000 0.000 0.000 0.000 0.000 0.071 0.066

0.068 0.050

>Non-Methylation code ID 21285

0.005 0.005 0.005 0.000 0.000 0.000 0.000 0.005 0.000 0.000

0.000 0.000 0.009 0.000 0.014 0.005 0.005 0.000 0.000 0.000

0.089 0.053 0.070 0.072 0.004 0.004 0.000 0.000 0.000 0.009

0.004 0.000 0.000 0.000 0.000 0.000 0.004 0.000 0.018 0.004

0.004 0.000 0.000 0.000 0.070 0.087 0.087 0.037 0.000 0.008

0.015 0.000 0.000 0.015 0.000 0.008 0.000 0.000 0.000 0.000

0.023 0.008 0.000 0.000 0.000 0.008 0.000 0.000 0.060 0.048

0.078 0.064

>Non-Methylation code ID 21285

0.024 0.000 0.000 0.000 0.000 0.018 0.000 0.000 0.000 0.000

0.000 0.000 0.006 0.000 0.006 0.006 0.012 0.000 0.006 0.000

0.055 0.061 0.073 0.066 0.015 0.000 0.008 0.000 0.000 0.023

0.000 0.000 0.000 0.000 0.000 0.000 0.045 0.000 0.008 0.000

0.000 0.000 0.000 0.000 0.055 0.049 0.072 0.060 0.006 0.000

0.000 0.012 0.000 0.000 0.012 0.000 0.000 0.000 0.006 0.000

0.000 0.000 0.035 0.000 0.000 0.000 0.000 0.000 0.054 0.061

0.075 0.074

>Non-Methylation code ID 21285

0.020 0.000 0.000 0.010 0.000 0.010 0.000 0.000 0.000 0.010

0.000 0.000 0.000 0.005 0.010 0.000 0.000 0.000 0.000 0.000

0.047 0.065 0.084 0.073 0.010 0.000 0.000 0.000 0.000 0.010

0.000 0.000 0.010 0.010 0.000 0.000 0.005 0.000 0.010 0.010

0.000 0.000 0.000 0.000 0.087 0.071 0.025 0.087 0.017 0.000

0.000 0.000 0.000 0.006 0.000 0.000 0.000 0.000 0.000 0.000

0.011 0.000 0.011 0.011 0.000 0.000 0.006 0.000 0.063 0.068

0.078 0.064

>Non-Methylation code ID 21285

0.000 0.005 0.000 0.010 0.000 0.005 0.010 0.000 0.000 0.000

0.000 0.000 0.005 0.000 0.026 0.000 0.000 0.005 0.000 0.000

0.045 0.048 0.075 0.099 0.069 0.000 0.000 0.000 0.000 0.014

0.000 0.000 0.000 0.014 0.000 0.000 0.000 0.000 0.000 0.041

0.028 0.014 0.000 0.000 0.023 0.040 0.050 0.041 0.014 0.000

0.000 0.000 0.000 0.000 0.000 0.000 0.000 0.007 0.000 0.000

0.034 0.000 0.014 0.007 0.000 0.000 0.000 0.000 0.069 0.037

0.075 0.077

>Non-Methylation code ID 21285

0.019 0.005 0.005 0.005 0.000 0.000 0.000 0.000 0.005 0.000

0.000 0.000 0.000 0.000 0.005 0.014 0.000 0.000 0.005 0.000

0.069 0.064 0.066 0.071 0.009 0.000 0.000 0.005 0.000 0.005

0.000 0.000 0.000 0.005 0.000 0.000 0.009 0.000 0.019 0.000

0.005 0.000 0.000 0.000 0.086 0.093 0.038 0.060 0.000 0.011

0.000 0.006 0.000 0.011 0.000 0.000 0.000 0.011 0.000 0.000

0.000 0.011 0.006 0.006 0.000 0.006 0.000 0.000 0.059 0.058

0.070 0.079

>Non-Methylation code ID 21285

0.033 0.000 0.000 0.000 0.000 0.000 0.000 0.000 0.000 0.008

0.000 0.000 0.008 0.008 0.008 0.016 0.016 0.008 0.000 0.000

0.057 0.059 0.056 0.055 0.005 0.000 0.000 0.000 0.000 0.005

0.000 0.000 0.000 0.005 0.000 0.000 0.019 0.000 0.014 0.010

0.000 0.000 0.000 0.000 0.067 0.053 0.075 0.081 0.006 0.006

0.000 0.006 0.000 0.012 0.012 0.000 0.000 0.000 0.000 0.000

0.018 0.000 0.006 0.000 0.000 0.006 0.000 0.000 0.051 0.052

0.076 0.083

>Non-Methylation code ID 21285

0.055 0.000 0.000 0.000 0.000 0.011 0.000 0.000 0.000 0.011

0.000 0.000 0.000 0.000 0.000 0.022 0.022 0.011 0.011 0.000

0.029 0.050 0.056 0.055 0.013 0.000 0.000 0.000 0.000 0.007

0.000 0.000 0.000 0.007 0.000 0.000 0.033 0.000 0.013 0.007

0.000 0.000 0.000 0.000 0.066 0.036 0.076 0.078 0.000 0.006

0.000 0.006 0.000 0.006 0.011 0.000 0.000 0.000 0.000 0.000

0.006 0.000 0.028 0.000 0.000 0.006 0.000 0.000 0.043 0.047

0.073 0.103

>Non-Methylation code ID 21285

0.011 0.000 0.000 0.000 0.000 0.005 0.000 0.000 0.000 0.005

0.005 0.000 0.000 0.000 0.016 0.011 0.000 0.005 0.000 0.000

0.060 0.088 0.056 0.070 0.020 0.013 0.000 0.000 0.000 0.013

0.000 0.000 0.000 0.020 0.000 0.000 0.007 0.000 0.000 0.007

0.000 0.000 0.007 0.000 0.068 0.066 0.068 0.046 0.009 0.009

0.000 0.005 0.000 0.009 0.005 0.000 0.000 0.000 0.000 0.000

0.005 0.000 0.009 0.005 0.000 0.000 0.000 0.000 0.069 0.062

0.072 0.074

>Non-Methylation code ID 21285

0.005 0.000 0.000 0.000 0.000 0.015 0.000 0.000 0.000 0.000

0.000 0.000 0.010 0.005 0.010 0.015 0.000 0.000 0.005 0.000

0.080 0.074 0.056 0.059 0.010 0.000 0.005 0.000 0.000 0.015

0.000 0.000 0.000 0.000 0.000 0.000 0.019 0.000 0.010 0.000

0.000 0.005 0.000 0.000 0.072 0.046 0.077 0.074 0.009 0.000

0.000 0.009 0.000 0.005 0.000 0.000 0.000 0.005 0.005 0.000

0.005 0.000 0.014 0.005 0.000 0.000 0.000 0.000 0.084 0.047

0.071 0.074

>Non-Methylation code ID 21285

0.005 0.000 0.000 0.000 0.010 0.005 0.005 0.000 0.000 0.010

0.000 0.000 0.015 0.000 0.005 0.005 0.000 0.005 0.000 0.000

0.058 0.070 0.074 0.065 0.006 0.000 0.000 0.000 0.006 0.006

0.000 0.000 0.000 0.000 0.000 0.000 0.018 0.006 0.006 0.018

0.006 0.000 0.000 0.006 0.072 0.067 0.071 0.043 0.005 0.000

0.000 0.000 0.005 0.000 0.000 0.000 0.000 0.005 0.000 0.000

0.019 0.000 0.014 0.000 0.005 0.005 0.000 0.000 0.071 0.055

0.073 0.076

>Non-Methylation code ID 21285

0.000 0.004 0.000 0.000 0.000 0.013 0.000 0.000 0.000 0.009

0.004 0.000 0.000 0.000 0.017 0.004 0.000 0.000 0.000 0.000

0.068 0.071 0.058 0.084 0.027 0.007 0.007 0.000 0.000 0.020

0.000 0.000 0.000 0.000 0.000 0.000 0.000 0.000 0.000 0.013

0.000 0.000 0.007 0.000 0.063 0.062 0.066 0.062 0.005 0.000

0.000 0.005 0.000 0.005 0.005 0.000 0.000 0.000 0.000 0.000

0.014 0.000 0.009 0.000 0.005 0.000 0.005 0.000 0.069 0.065

0.072 0.075

>Non-Methylation code ID 21285

0.010 0.000 0.000 0.000 0.000 0.010 0.000 0.000 0.010 0.010

0.000 0.000 0.005 0.000 0.010 0.010 0.000 0.000 0.000 0.000

0.087 0.071 0.025 0.087 0.015 0.000 0.000 0.000 0.000 0.005

0.000 0.000 0.000 0.000 0.000 0.000 0.010 0.000 0.015 0.010

0.000 0.000 0.005 0.000 0.070 0.070 0.079 0.055 0.016 0.000

0.000 0.011 0.000 0.011 0.000 0.000 0.000 0.011 0.000 0.000

0.000 0.005 0.011 0.000 0.000 0.000 0.000 0.000 0.053 0.073

0.078 0.064

>Non-Methylation code ID 21285

0.020 0.007 0.007 0.000 0.000 0.020 0.000 0.000 0.000 0.000

0.000 0.000 0.000 0.000 0.000 0.013 0.000 0.007 0.007 0.000

0.064 0.062 0.066 0.061 0.004 0.000 0.000 0.004 0.004 0.009

0.004 0.000 0.000 0.000 0.000 0.000 0.009 0.000 0.009 0.000

0.004 0.000 0.004 0.000 0.076 0.070 0.077 0.058 0.000 0.005

0.000 0.000 0.000 0.021 0.000 0.000 0.000 0.010 0.005 0.000

0.000 0.000 0.010 0.005 0.000 0.000 0.000 0.000 0.074 0.081

0.042 0.079

>Non-Methylation code ID 21285

0.004 0.004 0.004 0.000 0.000 0.000 0.000 0.004 0.000 0.000

0.000 0.000 0.009 0.000 0.013 0.004 0.004 0.000 0.000 0.000

0.088 0.059 0.073 0.066 0.004 0.004 0.000 0.000 0.000 0.009

0.004 0.000 0.000 0.000 0.000 0.000 0.004 0.000 0.017 0.004

0.004 0.000 0.000 0.000 0.074 0.096 0.065 0.046 0.008 0.008

0.015 0.000 0.000 0.015 0.000 0.000 0.000 0.008 0.000 0.000

0.015 0.008 0.000 0.000 0.000 0.008 0.000 0.000 0.052 0.049

0.072 0.079

>Non-Methylation code ID 21285

0.005 0.000 0.000 0.000 0.000 0.005 0.000 0.000 0.000 0.010

0.005 0.000 0.000 0.000 0.020 0.005 0.000 0.005 0.000 0.000

0.057 0.089 0.068 0.063 0.020 0.013 0.000 0.000 0.000 0.020

0.000 0.000 0.000 0.013 0.000 0.000 0.000 0.000 0.000 0.013

0.000 0.000 0.007 0.000 0.062 0.070 0.076 0.041 0.011 0.011

0.000 0.005 0.000 0.011 0.005 0.000 0.000 0.000 0.000 0.000

0.016 0.000 0.005 0.000 0.000 0.000 0.000 0.000 0.071 0.065

0.067 0.067

>Non-Methylation code ID 21285

0.004 0.000 0.004 0.000 0.004 0.000 0.000 0.004 0.000 0.000

0.000 0.000 0.013 0.000 0.013 0.004 0.004 0.000 0.000 0.004

0.090 0.053 0.076 0.056 0.005 0.000 0.000 0.000 0.005 0.005

0.005 0.000 0.000 0.000 0.000 0.000 0.010 0.000 0.014 0.005

0.010 0.000 0.000 0.000 0.080 0.058 0.087 0.051 0.008 0.008

0.008 0.000 0.000 0.008 0.008 0.008 0.000 0.008 0.000 0.000

0.031 0.000 0.000 0.000 0.000 0.008 0.000 0.000 0.076 0.048

0.073 0.041

>Non-Methylation code ID 21285

0.012 0.000 0.000 0.000 0.004 0.008 0.000 0.000 0.000 0.000

0.000 0.000 0.000 0.000 0.012 0.004 0.004 0.000 0.008 0.000

0.074 0.082 0.038 0.085 0.014 0.000 0.000 0.000 0.005 0.024

0.000 0.000 0.000 0.000 0.000 0.000 0.000 0.005 0.014 0.000

0.000 0.000 0.000 0.000 0.057 0.072 0.073 0.069 0.013 0.000

0.006 0.000 0.000 0.019 0.000 0.000 0.000 0.006 0.000 0.000

0.000 0.006 0.013 0.006 0.000 0.006 0.000 0.000 0.071 0.081

0.035 0.068

>Non-Methylation code ID 21285

0.005 0.000 0.005 0.000 0.000 0.014 0.000 0.000 0.000 0.005

0.000 0.000 0.018 0.000 0.009 0.000 0.000 0.005 0.000 0.000

0.072 0.041 0.078 0.083 0.009 0.000 0.000 0.009 0.004 0.004

0.000 0.000 0.000 0.004 0.004 0.000 0.004 0.000 0.013 0.004

0.000 0.000 0.000 0.000 0.077 0.056 0.073 0.072 0.005 0.000

0.000 0.000 0.000 0.016 0.000 0.000 0.000 0.000 0.000 0.000

0.011 0.005 0.011 0.011 0.000 0.000 0.005 0.000 0.079 0.070

0.061 0.059

>Non-Methylation code ID 21285

0.000 0.005 0.000 0.010 0.000 0.005 0.010 0.000 0.000 0.000

0.005 0.000 0.000 0.000 0.024 0.000 0.000 0.005 0.000 0.000

0.042 0.048 0.079 0.102 0.041 0.000 0.000 0.000 0.000 0.008

0.000 0.000 0.000 0.000 0.000 0.000 0.000 0.000 0.008 0.017

0.017 0.008 0.008 0.000 0.044 0.051 0.064 0.067 0.013 0.000

0.006 0.000 0.000 0.006 0.000 0.000 0.000 0.006 0.000 0.000

0.032 0.000 0.013 0.000 0.000 0.000 0.000 0.000 0.063 0.042

0.073 0.078

>Non-Methylation code ID 21285

0.008 0.008 0.000 0.000 0.000 0.004 0.000 0.000 0.000 0.004

0.000 0.000 0.004 0.000 0.012 0.004 0.000 0.004 0.000 0.000

0.078 0.060 0.054 0.092 0.014 0.009 0.000 0.000 0.000 0.005

0.000 0.000 0.000 0.000 0.000 0.000 0.000 0.005 0.014 0.005

0.000 0.005 0.000 0.000 0.068 0.071 0.065 0.074 0.018 0.006

0.000 0.006 0.000 0.006 0.000 0.000 0.000 0.018 0.000 0.000

0.006 0.000 0.006 0.006 0.000 0.000 0.000 0.000 0.059 0.061

0.062 0.080

>Non-Methylation code ID 21285

0.012 0.000 0.000 0.006 0.000 0.000 0.000 0.000 0.000 0.006

0.000 0.000 0.006 0.006 0.018 0.012 0.006 0.006 0.000 0.000

0.078 0.045 0.073 0.059 0.005 0.000 0.000 0.005 0.000 0.011

0.000 0.000 0.000 0.005 0.000 0.000 0.005 0.000 0.016 0.016

0.000 0.000 0.000 0.000 0.075 0.062 0.068 0.066 0.012 0.006

0.000 0.012 0.000 0.012 0.000 0.000 0.000 0.000 0.000 0.000

0.019 0.000 0.006 0.000 0.000 0.006 0.000 0.000 0.073 0.059

0.064 0.062

>Non-Methylation code ID 21285

0.015 0.005 0.000 0.005 0.000 0.000 0.000 0.000 0.005 0.005

0.000 0.000 0.005 0.000 0.005 0.015 0.000 0.000 0.005 0.000

0.068 0.074 0.057 0.071 0.009 0.000 0.000 0.000 0.000 0.004

0.000 0.000 0.000 0.004 0.000 0.000 0.009 0.000 0.018 0.004

0.000 0.004 0.000 0.000 0.095 0.070 0.034 0.082 0.010 0.010

0.000 0.005 0.000 0.005 0.000 0.000 0.000 0.005 0.000 0.000

0.000 0.010 0.005 0.005 0.000 0.005 0.000 0.000 0.061 0.063

0.073 0.075

>Non-Methylation code ID 21352

0.021 0.000 0.000 0.011 0.000 0.011 0.000 0.000 0.000 0.011

0.000 0.000 0.000 0.005 0.011 0.000 0.000 0.000 0.000 0.000

0.052 0.070 0.081 0.060 0.009 0.000 0.000 0.000 0.000 0.009

0.000 0.000 0.009 0.005 0.000 0.000 0.005 0.000 0.014 0.009

0.000 0.000 0.000 0.000 0.089 0.066 0.023 0.095 0.017 0.000

0.000 0.000 0.000 0.006 0.000 0.000 0.000 0.000 0.000 0.000

0.011 0.000 0.017 0.011 0.000 0.000 0.000 0.000 0.064 0.071

0.082 0.053

>Non-Methylation code ID 21352

0.005 0.000 0.000 0.005 0.000 0.011 0.000 0.000 0.000 0.005

0.000 0.000 0.005 0.000 0.016 0.016 0.000 0.000 0.000 0.000

0.075 0.062 0.068 0.066 0.011 0.006 0.006 0.011 0.000 0.011

0.000 0.000 0.000 0.000 0.000 0.000 0.017 0.000 0.006 0.000

0.000 0.006 0.000 0.000 0.073 0.065 0.058 0.064 0.012 0.000

0.000 0.006 0.000 0.000 0.000 0.000 0.000 0.006 0.000 0.000

0.006 0.006 0.019 0.012 0.000 0.006 0.000 0.000 0.080 0.044

0.075 0.060

>Non-Methylation code ID 21352

0.011 0.000 0.000 0.017 0.000 0.017 0.000 0.000 0.000 0.000

0.000 0.000 0.000 0.006 0.017 0.006 0.000 0.000 0.000 0.000

0.075 0.055 0.084 0.048 0.010 0.000 0.000 0.005 0.000 0.015

0.000 0.000 0.005 0.000 0.000 0.000 0.010 0.000 0.010 0.010

0.000 0.000 0.000 0.000 0.073 0.078 0.053 0.065 0.013 0.000

0.000 0.000 0.000 0.025 0.000 0.000 0.000 0.006 0.000 0.000

0.006 0.000 0.025 0.000 0.000 0.000 0.000 0.000 0.062 0.050

0.074 0.071

>Non-Methylation code ID 21352

0.000 0.011 0.000 0.000 0.000 0.000 0.004 0.000 0.000 0.011

0.000 0.000 0.004 0.000 0.014 0.000 0.000 0.000 0.004 0.000

0.095 0.073 0.084 0.035 0.038 0.008 0.008 0.000 0.000 0.008

0.000 0.008 0.000 0.008 0.000 0.000 0.000 0.000 0.000 0.008

0.000 0.008 0.000 0.008 0.061 0.067 0.048 0.059 0.023 0.000

0.000 0.000 0.000 0.000 0.000 0.000 0.000 0.035 0.000 0.000

0.035 0.000 0.000 0.023 0.023 0.000 0.000 0.000 0.054 0.047

0.041 0.051

>Non-Methylation code ID 21352

0.005 0.000 0.000 0.000 0.000 0.014 0.000 0.000 0.000 0.009

0.000 0.000 0.014 0.000 0.014 0.005 0.000 0.000 0.000 0.000

0.062 0.088 0.083 0.039 0.009 0.009 0.005 0.005 0.000 0.009

0.005 0.000 0.000 0.009 0.000 0.000 0.005 0.000 0.005 0.000

0.000 0.000 0.000 0.000 0.073 0.054 0.083 0.064 0.017 0.000

0.000 0.000 0.006 0.006 0.000 0.000 0.000 0.000 0.000 0.000

0.017 0.000 0.006 0.000 0.006 0.006 0.006 0.000 0.075 0.071

0.066 0.052

>Non-Methylation code ID 21352

0.005 0.000 0.000 0.000 0.005 0.005 0.000 0.000 0.000 0.000

0.000 0.000 0.016 0.000 0.005 0.011 0.011 0.000 0.005 0.000

0.063 0.057 0.074 0.075 0.006 0.006 0.000 0.000 0.000 0.006

0.006 0.000 0.000 0.011 0.000 0.000 0.011 0.000 0.011 0.006

0.000 0.011 0.000 0.000 0.080 0.040 0.073 0.068 0.012 0.000

0.006 0.000 0.012 0.006 0.000 0.000 0.000 0.006 0.000 0.000

0.006 0.006 0.006 0.000 0.006 0.000 0.000 0.006 0.057 0.068

0.062 0.075

>Non-Methylation code ID 21352

0.020 0.000 0.007 0.000 0.007 0.014 0.000 0.000 0.000 0.014

0.000 0.000 0.007 0.000 0.000 0.000 0.007 0.007 0.000 0.007

0.057 0.066 0.054 0.067 0.007 0.000 0.000 0.000 0.000 0.000

0.000 0.000 0.000 0.015 0.000 0.000 0.022 0.000 0.007 0.015

0.015 0.000 0.007 0.000 0.064 0.059 0.054 0.069 0.000 0.009

0.000 0.000 0.000 0.004 0.004 0.000 0.000 0.009 0.000 0.000

0.013 0.000 0.009 0.000 0.000 0.000 0.004 0.000 0.077 0.049

0.087 0.068

>Non-Methylation code ID 21352

0.008 0.004 0.000 0.008 0.000 0.008 0.000 0.000 0.000 0.000

0.000 0.000 0.000 0.004 0.015 0.000 0.000 0.000 0.000 0.000

0.054 0.061 0.083 0.089 0.006 0.006 0.006 0.006 0.000 0.012

0.000 0.000 0.012 0.000 0.000 0.000 0.006 0.006 0.000 0.006

0.000 0.006 0.000 0.000 0.076 0.064 0.068 0.052 0.017 0.006

0.000 0.000 0.000 0.006 0.000 0.000 0.000 0.006 0.000 0.000

0.000 0.006 0.011 0.011 0.006 0.000 0.000 0.000 0.061 0.064

0.080 0.060

>Non-Methylation code ID 21352

0.000 0.011 0.000 0.000 0.004 0.000 0.004 0.000 0.000 0.011

0.000 0.000 0.004 0.000 0.014 0.000 0.000 0.000 0.000 0.000

0.090 0.060 0.081 0.056 0.034 0.009 0.000 0.000 0.000 0.017

0.009 0.009 0.000 0.000 0.000 0.000 0.000 0.000 0.000 0.026

0.000 0.009 0.000 0.000 0.056 0.055 0.045 0.067 0.020 0.000

0.000 0.000 0.000 0.000 0.000 0.000 0.000 0.020 0.010 0.000

0.049 0.000 0.000 0.010 0.000 0.010 0.000 0.000 0.067 0.042

0.051 0.055

>Non-Methylation code ID 21352

0.013 0.000 0.006 0.000 0.000 0.026 0.000 0.000 0.000 0.019

0.000 0.000 0.000 0.006 0.006 0.000 0.000 0.000 0.000 0.000

0.064 0.052 0.070 0.070 0.004 0.004 0.000 0.004 0.000 0.015

0.000 0.004 0.000 0.000 0.000 0.000 0.004 0.000 0.008 0.000

0.000 0.000 0.008 0.000 0.072 0.069 0.072 0.071 0.029 0.000

0.000 0.000 0.007 0.029 0.000 0.000 0.000 0.007 0.000 0.000

0.000 0.000 0.007 0.007 0.000 0.000 0.000 0.000 0.061 0.062

0.055 0.068

>Non-Methylation code ID 21352

0.011 0.000 0.000 0.016 0.000 0.016 0.000 0.000 0.000 0.000

0.000 0.000 0.000 0.011 0.011 0.005 0.000 0.000 0.000 0.000

0.060 0.062 0.076 0.064 0.010 0.000 0.000 0.005 0.000 0.010

0.000 0.000 0.010 0.000 0.000 0.000 0.010 0.000 0.010 0.010

0.000 0.000 0.000 0.000 0.079 0.070 0.062 0.059 0.013 0.000

0.000 0.000 0.000 0.013 0.000 0.000 0.000 0.004 0.000 0.000

0.004 0.000 0.018 0.000 0.000 0.000 0.000 0.000 0.052 0.059

0.090 0.080

>Non-Methylation code ID 21352

0.037 0.000 0.000 0.000 0.009 0.019 0.009 0.000 0.000 0.000

0.000 0.000 0.000 0.000 0.000 0.028 0.009 0.009 0.000 0.000

0.052 0.047 0.038 0.076 0.007 0.000 0.000 0.000 0.000 0.000

0.000 0.000 0.000 0.014 0.007 0.000 0.042 0.000 0.007 0.000

0.000 0.007 0.000 0.000 0.080 0.063 0.043 0.063 0.000 0.011

0.000 0.000 0.004 0.004 0.004 0.000 0.000 0.007 0.000 0.000

0.004 0.000 0.011 0.000 0.000 0.000 0.000 0.000 0.081 0.055

0.081 0.074

>Non-Methylation code ID 21352

0.003 0.007 0.000 0.000 0.003 0.003 0.003 0.000 0.000 0.010

0.000 0.000 0.003 0.000 0.010 0.000 0.000 0.000 0.000 0.000

0.071 0.056 0.083 0.079 0.026 0.000 0.000 0.000 0.009 0.018

0.009 0.000 0.000 0.000 0.000 0.000 0.009 0.000 0.000 0.026

0.009 0.009 0.000 0.000 0.065 0.047 0.045 0.061 0.007 0.000

0.000 0.000 0.000 0.000 0.000 0.000 0.000 0.007 0.007 0.000

0.043 0.000 0.007 0.000 0.000 0.007 0.000 0.000 0.076 0.059

0.066 0.054

>Non-Methylation code ID 21352

0.011 0.000 0.006 0.000 0.011 0.011 0.000 0.000 0.000 0.006

0.000 0.000 0.006 0.006 0.006 0.000 0.006 0.000 0.000 0.006

0.060 0.070 0.056 0.075 0.005 0.000 0.000 0.000 0.005 0.005

0.000 0.000 0.000 0.000 0.000 0.000 0.016 0.000 0.005 0.011

0.011 0.000 0.005 0.000 0.063 0.057 0.074 0.075 0.006 0.006

0.000 0.000 0.000 0.006 0.006 0.000 0.000 0.012 0.000 0.000

0.012 0.000 0.012 0.006 0.000 0.006 0.000 0.000 0.083 0.040

0.077 0.064

>Non-Methylation code ID 21352

0.004 0.004 0.004 0.000 0.004 0.004 0.004 0.000 0.000 0.012

0.000 0.000 0.008 0.000 0.008 0.000 0.000 0.000 0.000 0.000

0.077 0.050 0.086 0.068 0.007 0.000 0.000 0.000 0.007 0.015

0.007 0.000 0.000 0.000 0.000 0.000 0.015 0.000 0.000 0.015

0.015 0.007 0.007 0.000 0.066 0.054 0.044 0.075 0.006 0.000

0.000 0.000 0.000 0.006 0.000 0.000 0.000 0.006 0.000 0.000

0.028 0.000 0.011 0.000 0.000 0.006 0.000 0.000 0.071 0.055

0.069 0.077

>Non-Methylation code ID 21352

0.013 0.000 0.006 0.000 0.013 0.013 0.000 0.000 0.000 0.013

0.000 0.000 0.006 0.006 0.000 0.000 0.006 0.000 0.000 0.006

0.065 0.071 0.048 0.066 0.006 0.000 0.000 0.000 0.006 0.000

0.000 0.000 0.000 0.006 0.000 0.000 0.017 0.000 0.006 0.012

0.012 0.000 0.006 0.000 0.068 0.057 0.064 0.074 0.000 0.005

0.000 0.000 0.000 0.005 0.005 0.000 0.000 0.011 0.000 0.000

0.011 0.000 0.011 0.005 0.000 0.005 0.005 0.000 0.088 0.039

0.080 0.062

>Non-Methylation code ID 21352

0.006 0.006 0.000 0.000 0.000 0.006 0.000 0.000 0.000 0.006

0.000 0.000 0.006 0.012 0.006 0.019 0.006 0.000 0.000 0.000

0.057 0.057 0.071 0.074 0.008 0.008 0.000 0.004 0.000 0.004

0.000 0.000 0.000 0.004 0.000 0.000 0.004 0.000 0.011 0.004

0.000 0.000 0.000 0.000 0.051 0.074 0.075 0.088 0.012 0.006

0.006 0.006 0.000 0.000 0.000 0.000 0.006 0.000 0.000 0.000

0.006 0.006 0.006 0.006 0.000 0.006 0.000 0.000 0.075 0.060

0.072 0.059

>Non-Methylation code ID 21352

0.004 0.004 0.004 0.004 0.004 0.008 0.004 0.000 0.000 0.008

0.000 0.000 0.004 0.000 0.008 0.000 0.000 0.000 0.000 0.000

0.079 0.059 0.078 0.064 0.006 0.000 0.000 0.000 0.006 0.012

0.000 0.000 0.000 0.000 0.000 0.000 0.012 0.000 0.006 0.006

0.012 0.012 0.006 0.000 0.067 0.059 0.054 0.076 0.006 0.000

0.000 0.000 0.000 0.011 0.000 0.000 0.000 0.006 0.000 0.000

0.017 0.000 0.011 0.006 0.000 0.006 0.000 0.000 0.074 0.063

0.072 0.061

>Non-Methylation code ID 21352

0.009 0.009 0.000 0.005 0.005 0.000 0.005 0.005 0.000 0.000

0.000 0.000 0.000 0.000 0.005 0.005 0.005 0.005 0.005 0.000

0.056 0.077 0.070 0.072 0.006 0.000 0.000 0.000 0.006 0.006

0.013 0.000 0.000 0.000 0.000 0.000 0.006 0.006 0.006 0.006

0.006 0.013 0.000 0.000 0.066 0.072 0.071 0.048 0.000 0.000

0.000 0.000 0.006 0.006 0.006 0.000 0.000 0.011 0.006 0.006

0.000 0.000 0.017 0.006 0.006 0.000 0.000 0.000 0.051 0.066

0.067 0.082

>Non-Methylation code ID 21352

0.010 0.000 0.000 0.005 0.000 0.014 0.000 0.000 0.000 0.010

0.000 0.000 0.005 0.005 0.014 0.000 0.000 0.000 0.000 0.000

0.078 0.076 0.048 0.069 0.009 0.000 0.013 0.000 0.000 0.000

0.004 0.000 0.004 0.000 0.000 0.000 0.004 0.000 0.009 0.000

0.000 0.004 0.004 0.000 0.071 0.067 0.081 0.060 0.000 0.000

0.000 0.000 0.000 0.019 0.000 0.000 0.000 0.006 0.000 0.000

0.000 0.006 0.013 0.013 0.019 0.000 0.000 0.000 0.072 0.039

0.080 0.065

>Non-Methylation code ID 21352

0.006 0.000 0.006 0.006 0.000 0.017 0.006 0.000 0.000 0.000

0.000 0.006 0.006 0.006 0.006 0.011 0.000 0.000 0.000 0.000

0.067 0.063 0.062 0.068 0.006 0.000 0.000 0.011 0.000 0.006

0.000 0.000 0.006 0.006 0.000 0.000 0.006 0.000 0.011 0.000

0.017 0.000 0.000 0.000 0.087 0.052 0.046 0.083 0.000 0.000

0.000 0.005 0.000 0.009 0.000 0.000 0.005 0.005 0.000 0.000

0.014 0.000 0.009 0.005 0.000 0.000 0.005 0.000 0.071 0.061

0.058 0.086

>Non-Methylation code ID 21352

0.009 0.000 0.000 0.000 0.000 0.009 0.000 0.000 0.000 0.004

0.000 0.000 0.009 0.004 0.018 0.004 0.000 0.000 0.000 0.000

0.068 0.091 0.061 0.056 0.010 0.005 0.005 0.000 0.000 0.010

0.000 0.000 0.000 0.000 0.000 0.000 0.015 0.000 0.010 0.005

0.000 0.000 0.000 0.000 0.061 0.061 0.078 0.075 0.016 0.000

0.000 0.000 0.000 0.005 0.000 0.000 0.000 0.000 0.000 0.000

0.016 0.005 0.005 0.000 0.005 0.005 0.005 0.000 0.055 0.076

0.079 0.058

>Non-Methylation code ID 21352

0.015 0.000 0.005 0.005 0.005 0.000 0.000 0.000 0.005 0.000

0.000 0.005 0.010 0.000 0.005 0.005 0.000 0.005 0.000 0.000

0.080 0.063 0.069 0.059 0.000 0.000 0.000 0.000 0.005 0.005

0.000 0.005 0.000 0.005 0.000 0.000 0.014 0.005 0.009 0.009

0.005 0.000 0.000 0.000 0.059 0.066 0.067 0.081 0.004 0.000

0.000 0.004 0.009 0.009 0.000 0.000 0.000 0.000 0.000 0.000

0.009 0.000 0.013 0.000 0.000 0.000 0.000 0.000 0.062 0.097

0.067 0.058

>Non-Methylation code ID 21352

0.004 0.000 0.000 0.000 0.000 0.004 0.000 0.000 0.008 0.004

0.000 0.000 0.012 0.000 0.012 0.004 0.000 0.000 0.004 0.000

0.063 0.065 0.075 0.079 0.010 0.000 0.005 0.005 0.000 0.010

0.005 0.000 0.000 0.000 0.000 0.005 0.005 0.005 0.010 0.005

0.000 0.000 0.000 0.000 0.068 0.074 0.064 0.065 0.006 0.000

0.000 0.013 0.000 0.006 0.000 0.000 0.006 0.006 0.000 0.000

0.006 0.006 0.006 0.000 0.019 0.000 0.000 0.000 0.084 0.044

0.066 0.064

>Non-Methylation code ID 21352

0.000 0.000 0.006 0.006 0.000 0.018 0.006 0.000 0.000 0.000

0.000 0.006 0.006 0.006 0.006 0.012 0.000 0.006 0.000 0.000

0.071 0.052 0.068 0.066 0.006 0.000 0.000 0.006 0.000 0.011

0.000 0.000 0.006 0.000 0.000 0.000 0.006 0.000 0.011 0.006

0.017 0.000 0.000 0.000 0.079 0.062 0.056 0.069 0.000 0.005

0.000 0.005 0.000 0.011 0.000 0.000 0.005 0.005 0.000 0.000

0.016 0.005 0.011 0.000 0.000 0.000 0.000 0.000 0.076 0.061

0.058 0.075

>Non-Methylation code ID 21352

0.005 0.000 0.000 0.005 0.009 0.005 0.000 0.000 0.000 0.005

0.000 0.000 0.009 0.005 0.014 0.000 0.000 0.000 0.000 0.000

0.062 0.091 0.054 0.072 0.009 0.005 0.005 0.000 0.005 0.000

0.000 0.000 0.005 0.000 0.000 0.005 0.009 0.000 0.005 0.009

0.000 0.000 0.000 0.000 0.058 0.076 0.078 0.068 0.022 0.000

0.000 0.000 0.007 0.000 0.000 0.007 0.000 0.007 0.000 0.000

0.015 0.007 0.007 0.007 0.007 0.000 0.000 0.000 0.051 0.065

0.072 0.057

>Non-Methylation code ID 21352

0.000 0.000 0.000 0.004 0.008 0.012 0.000 0.000 0.000 0.004

0.000 0.000 0.008 0.000 0.016 0.000 0.000 0.000 0.000 0.000

0.072 0.105 0.055 0.049 0.016 0.000 0.011 0.005 0.005 0.000

0.000 0.000 0.000 0.000 0.000 0.005 0.005 0.000 0.005 0.005

0.000 0.005 0.000 0.000 0.082 0.065 0.073 0.048 0.000 0.000

0.000 0.000 0.005 0.005 0.000 0.005 0.000 0.000 0.000 0.000

0.010 0.005 0.015 0.005 0.010 0.000 0.000 0.000 0.064 0.073

0.060 0.076

>Non-Methylation code ID 21352

0.005 0.000 0.005 0.000 0.000 0.010 0.010 0.000 0.005 0.000

0.000 0.000 0.000 0.005 0.005 0.010 0.000 0.005 0.005 0.000

0.062 0.061 0.069 0.078 0.007 0.000 0.000 0.000 0.000 0.020

0.000 0.000 0.000 0.000 0.000 0.000 0.000 0.000 0.020 0.013

0.020 0.000 0.000 0.000 0.075 0.067 0.044 0.067 0.006 0.006

0.000 0.011 0.000 0.017 0.000 0.000 0.000 0.011 0.000 0.000

0.006 0.006 0.006 0.000 0.000 0.000 0.000 0.000 0.076 0.070

0.047 0.073

>Non-Methylation code ID 21352

0.000 0.000 0.000 0.004 0.008 0.013 0.000 0.000 0.000 0.000

0.000 0.000 0.008 0.000 0.017 0.000 0.000 0.000 0.000 0.000

0.075 0.101 0.056 0.052 0.015 0.000 0.010 0.005 0.005 0.000

0.000 0.000 0.005 0.000 0.000 0.005 0.005 0.000 0.005 0.005

0.000 0.005 0.000 0.000 0.080 0.070 0.068 0.051 0.000 0.000

0.000 0.000 0.005 0.005 0.000 0.005 0.000 0.005 0.000 0.000

0.011 0.005 0.011 0.005 0.011 0.000 0.000 0.000 0.061 0.067

0.064 0.078

>Non-Methylation code ID 21352

0.000 0.000 0.000 0.000 0.005 0.005 0.000 0.000 0.005 0.000

0.000 0.000 0.016 0.005 0.010 0.005 0.005 0.005 0.000 0.000

0.070 0.084 0.056 0.060 0.000 0.000 0.000 0.009 0.000 0.017

0.000 0.000 0.000 0.009 0.000 0.000 0.035 0.000 0.000 0.035

0.000 0.000 0.000 0.000 0.073 0.040 0.053 0.063 0.012 0.000

0.000 0.012 0.006 0.000 0.006 0.000 0.000 0.000 0.000 0.000

0.012 0.006 0.006 0.006 0.000 0.006 0.000 0.000 0.065 0.054

0.067 0.076

>Non-Methylation code ID 21352

0.010 0.000 0.000 0.005 0.005 0.000 0.005 0.000 0.000 0.005

0.000 0.000 0.020 0.005 0.005 0.000 0.000 0.005 0.000 0.000

0.069 0.058 0.074 0.066 0.000 0.000 0.000 0.000 0.011 0.000

0.000 0.000 0.005 0.005 0.000 0.000 0.021 0.005 0.005 0.005

0.005 0.005 0.000 0.000 0.071 0.065 0.057 0.071 0.000 0.000

0.000 0.000 0.000 0.009 0.000 0.000 0.000 0.009 0.000 0.000

0.045 0.000 0.000 0.045 0.000 0.000 0.000 0.000 0.074 0.041

0.042 0.069

>Non-Methylation code ID 21352

0.022 0.000 0.000 0.000 0.000 0.000 0.000 0.000 0.000 0.015

0.000 0.000 0.022 0.007 0.007 0.000 0.015 0.000 0.000 0.000

0.066 0.057 0.058 0.064 0.000 0.005 0.000 0.000 0.005 0.000

0.005 0.000 0.005 0.009 0.000 0.000 0.018 0.000 0.005 0.000

0.000 0.005 0.005 0.000 0.075 0.059 0.082 0.057 0.000 0.006

0.006 0.000 0.000 0.006 0.006 0.000 0.000 0.000 0.000 0.000

0.011 0.000 0.006 0.022 0.006 0.000 0.000 0.000 0.070 0.063

0.053 0.080

>Non-Methylation code ID 21352

0.011 0.000 0.000 0.011 0.005 0.000 0.005 0.000 0.000 0.000

0.000 0.000 0.016 0.005 0.005 0.005 0.000 0.005 0.000 0.000

0.072 0.051 0.066 0.075 0.000 0.000 0.000 0.000 0.006 0.000

0.000 0.000 0.006 0.006 0.000 0.000 0.017 0.006 0.011 0.006

0.006 0.006 0.000 0.000 0.060 0.079 0.066 0.061 0.000 0.000

0.000 0.000 0.000 0.020 0.000 0.000 0.000 0.010 0.000 0.000

0.040 0.000 0.000 0.040 0.000 0.000 0.000 0.000 0.073 0.035

0.050 0.066

>Non-Methylation code ID 21352

0.010 0.010 0.005 0.000 0.000 0.005 0.005 0.000 0.000 0.000

0.000 0.000 0.000 0.000 0.010 0.010 0.005 0.000 0.000 0.000

0.072 0.065 0.071 0.068 0.044 0.000 0.000 0.000 0.000 0.000

0.000 0.000 0.000 0.009 0.000 0.000 0.018 0.000 0.009 0.000

0.018 0.000 0.000 0.000 0.053 0.058 0.054 0.072 0.000 0.010

0.000 0.000 0.000 0.000 0.005 0.000 0.000 0.015 0.000 0.000

0.010 0.010 0.005 0.000 0.000 0.000 0.005 0.000 0.071 0.084

0.070 0.048

>Non-Methylation code ID 21352

0.022 0.000 0.000 0.007 0.000 0.000 0.000 0.000 0.015 0.007

0.000 0.000 0.007 0.000 0.000 0.015 0.007 0.000 0.007 0.000

0.063 0.045 0.066 0.072 0.000 0.000 0.000 0.000 0.000 0.006

0.000 0.000 0.000 0.012 0.000 0.000 0.031 0.000 0.006 0.012

0.000 0.000 0.006 0.000 0.068 0.060 0.082 0.051 0.000 0.006

0.000 0.012 0.000 0.012 0.000 0.000 0.000 0.018 0.000 0.000

0.006 0.000 0.006 0.000 0.000 0.000 0.000 0.000 0.076 0.060

0.069 0.068

>Non-Methylation code ID 21352

0.005 0.011 0.005 0.000 0.000 0.016 0.000 0.005 0.000 0.000

0.000 0.000 0.000 0.000 0.005 0.005 0.000 0.005 0.005 0.000

0.063 0.065 0.059 0.081 0.017 0.008 0.000 0.008 0.000 0.034

0.000 0.000 0.000 0.000 0.000 0.000 0.008 0.008 0.000 0.017

0.008 0.000 0.000 0.000 0.058 0.082 0.052 0.033 0.000 0.000

0.005 0.005 0.000 0.010 0.000 0.000 0.000 0.010 0.000 0.000

0.000 0.000 0.020 0.005 0.000 0.000 0.000 0.000 0.094 0.051

0.079 0.056

>Non-Methylation code ID 21352

0.000 0.006 0.000 0.006 0.000 0.000 0.000 0.006 0.011 0.000

0.000 0.000 0.017 0.000 0.006 0.011 0.000 0.000 0.006 0.000

0.046 0.057 0.079 0.083 0.013 0.000 0.000 0.000 0.000 0.013

0.000 0.000 0.013 0.000 0.000 0.000 0.019 0.000 0.006 0.006

0.000 0.006 0.006 0.000 0.069 0.047 0.063 0.073 0.000 0.000

0.000 0.007 0.000 0.013 0.007 0.000 0.013 0.013 0.000 0.000

0.007 0.013 0.000 0.007 0.000 0.000 0.000 0.000 0.066 0.061

0.068 0.058

>Non-Methylation code ID 21352

0.012 0.012 0.006 0.000 0.006 0.012 0.000 0.000 0.000 0.000

0.000 0.000 0.000 0.000 0.000 0.012 0.006 0.000 0.006 0.000

0.075 0.074 0.052 0.060 0.015 0.005 0.000 0.000 0.000 0.010

0.000 0.000 0.000 0.005 0.000 0.000 0.000 0.000 0.015 0.005

0.005 0.000 0.000 0.000 0.083 0.071 0.054 0.064 0.000 0.005

0.000 0.000 0.000 0.011 0.005 0.000 0.000 0.005 0.000 0.000

0.000 0.005 0.011 0.000 0.000 0.005 0.005 0.000 0.087 0.052

0.061 0.079

>Non-Methylation code ID 21352

0.000 0.000 0.000 0.000 0.009 0.017 0.000 0.000 0.000 0.017

0.000 0.000 0.026 0.009 0.000 0.034 0.000 0.000 0.000 0.000

0.062 0.059 0.054 0.047 0.011 0.006 0.000 0.000 0.000 0.011

0.000 0.000 0.000 0.000 0.000 0.000 0.023 0.000 0.006 0.011

0.000 0.000 0.006 0.000 0.063 0.062 0.066 0.068 0.009 0.000

0.000 0.009 0.000 0.009 0.000 0.000 0.000 0.009 0.000 0.000

0.044 0.009 0.000 0.000 0.000 0.018 0.000 0.000 0.075 0.045

0.050 0.058

>Non-Methylation code ID 21352

0.000 0.000 0.006 0.000 0.012 0.012 0.000 0.000 0.000 0.018

0.000 0.000 0.006 0.000 0.006 0.006 0.000 0.006 0.000 0.000

0.077 0.083 0.043 0.060 0.013 0.006 0.000 0.000 0.000 0.006

0.000 0.000 0.000 0.006 0.000 0.000 0.000 0.000 0.006 0.013

0.006 0.000 0.006 0.000 0.053 0.064 0.075 0.077 0.007 0.000

0.000 0.000 0.000 0.007 0.000 0.000 0.000 0.014 0.000 0.000

0.014 0.007 0.007 0.014 0.000 0.007 0.000 0.000 0.065 0.055

0.070 0.064

>Non-Methylation code ID 21352

0.004 0.008 0.000 0.004 0.008 0.000 0.000 0.000 0.000 0.004

0.004 0.000 0.004 0.000 0.004 0.004 0.004 0.000 0.004 0.000

0.068 0.067 0.069 0.079 0.011 0.006 0.000 0.000 0.017 0.011

0.000 0.000 0.006 0.011 0.000 0.000 0.011 0.000 0.000 0.000

0.000 0.000 0.000 0.000 0.060 0.069 0.057 0.073 0.000 0.000

0.000 0.000 0.009 0.000 0.018 0.000 0.000 0.018 0.000 0.000

0.018 0.000 0.000 0.009 0.000 0.018 0.000 0.000 0.057 0.061

0.070 0.058

>Non-Methylation code ID 21352

0.011 0.011 0.005 0.000 0.000 0.011 0.000 0.000 0.000 0.000

0.000 0.000 0.000 0.000 0.005 0.005 0.005 0.000 0.005 0.000

0.068 0.079 0.047 0.079 0.011 0.011 0.000 0.000 0.000 0.011

0.000 0.000 0.000 0.005 0.000 0.000 0.000 0.000 0.011 0.000

0.005 0.005 0.000 0.000 0.079 0.079 0.089 0.027 0.005 0.005

0.000 0.000 0.000 0.010 0.000 0.000 0.000 0.000 0.000 0.000

0.000 0.005 0.010 0.000 0.000 0.000 0.005 0.005 0.081 0.051

0.084 0.073

>Non-Methylation code ID 21352

0.000 0.000 0.000 0.000 0.008 0.008 0.008 0.000 0.000 0.017

0.000 0.000 0.017 0.017 0.000 0.033 0.000 0.000 0.000 0.000

0.057 0.051 0.047 0.071 0.014 0.007 0.000 0.000 0.000 0.007

0.000 0.007 0.000 0.000 0.000 0.000 0.028 0.000 0.000 0.021

0.000 0.000 0.007 0.000 0.056 0.053 0.057 0.075 0.018 0.000

0.000 0.009 0.000 0.009 0.000 0.000 0.000 0.009 0.000 0.000

0.046 0.009 0.000 0.000 0.000 0.009 0.000 0.000 0.077 0.043

0.056 0.047

>Non-Methylation code ID 21352

0.005 0.014 0.000 0.000 0.000 0.009 0.000 0.000 0.000 0.000

0.000 0.000 0.000 0.000 0.009 0.000 0.005 0.005 0.005 0.000

0.086 0.091 0.084 0.021 0.010 0.005 0.000 0.000 0.000 0.010

0.000 0.000 0.000 0.000 0.000 0.000 0.000 0.000 0.010 0.000

0.000 0.000 0.005 0.005 0.081 0.051 0.074 0.083 0.005 0.016

0.000 0.000 0.000 0.005 0.005 0.000 0.000 0.000 0.000 0.000

0.000 0.000 0.005 0.000 0.005 0.000 0.005 0.000 0.080 0.090

0.056 0.059

>Non-Methylation code ID 21352

0.000 0.000 0.005 0.000 0.005 0.010 0.000 0.000 0.005 0.005

0.000 0.000 0.005 0.000 0.010 0.010 0.000 0.005 0.000 0.000

0.066 0.083 0.069 0.052 0.012 0.000 0.000 0.006 0.000 0.006

0.000 0.000 0.000 0.006 0.000 0.000 0.000 0.000 0.018 0.012

0.006 0.000 0.000 0.000 0.075 0.085 0.064 0.042 0.007 0.000

0.000 0.007 0.000 0.007 0.000 0.000 0.007 0.022 0.000 0.000

0.007 0.000 0.007 0.015 0.000 0.000 0.000 0.000 0.058 0.066

0.059 0.067

>Non-Methylation code ID 21352

0.011 0.000 0.000 0.005 0.000 0.016 0.000 0.000 0.005 0.005

0.005 0.000 0.011 0.000 0.005 0.000 0.000 0.000 0.000 0.000

0.058 0.064 0.065 0.081 0.000 0.000 0.005 0.005 0.000 0.010

0.000 0.000 0.005 0.010 0.000 0.000 0.010 0.000 0.005 0.000

0.000 0.000 0.010 0.000 0.066 0.075 0.061 0.072 0.006 0.006

0.000 0.006 0.000 0.025 0.000 0.000 0.000 0.000 0.000 0.006

0.006 0.000 0.006 0.006 0.006 0.000 0.000 0.000 0.065 0.064

0.063 0.066

>Non-Methylation code ID 21352

0.005 0.011 0.000 0.000 0.000 0.005 0.005 0.000 0.000 0.000

0.005 0.000 0.000 0.000 0.011 0.000 0.005 0.005 0.000 0.000

0.089 0.079 0.060 0.052 0.008 0.023 0.000 0.000 0.000 0.000

0.000 0.000 0.000 0.008 0.000 0.000 0.000 0.008 0.000 0.000

0.008 0.000 0.008 0.008 0.084 0.044 0.086 0.049 0.013 0.027

0.000 0.000 0.000 0.007 0.013 0.000 0.000 0.000 0.000 0.000

0.000 0.000 0.000 0.007 0.007 0.000 0.000 0.000 0.086 0.047

0.068 0.059

>Non-Methylation code ID 21352

0.006 0.018 0.000 0.000 0.000 0.018 0.006 0.000 0.000 0.000

0.006 0.000 0.000 0.000 0.006 0.006 0.000 0.000 0.000 0.000

0.076 0.073 0.070 0.050 0.021 0.014 0.000 0.000 0.000 0.021

0.000 0.000 0.000 0.000 0.007 0.000 0.000 0.007 0.000 0.000

0.000 0.000 0.007 0.000 0.077 0.059 0.072 0.048 0.005 0.005

0.000 0.000 0.000 0.010 0.005 0.000 0.005 0.000 0.000 0.000

0.000 0.000 0.005 0.000 0.000 0.000 0.010 0.000 0.069 0.072

0.086 0.062

>Non-Methylation code ID 21352

0.000 0.006 0.006 0.000 0.000 0.006 0.000 0.000 0.000 0.006

0.000 0.006 0.000 0.006 0.011 0.006 0.000 0.006 0.000 0.000

0.077 0.051 0.076 0.074 0.011 0.011 0.000 0.011 0.006 0.000

0.000 0.000 0.006 0.000 0.000 0.000 0.000 0.000 0.000 0.006

0.011 0.000 0.006 0.000 0.055 0.072 0.066 0.074 0.016 0.000

0.000 0.000 0.000 0.005 0.000 0.000 0.011 0.011 0.000 0.000

0.000 0.000 0.016 0.005 0.000 0.000 0.000 0.000 0.072 0.060

0.057 0.080

>Non-Methylation code ID 21352

0.000 0.000 0.000 0.000 0.000 0.016 0.008 0.000 0.000 0.023

0.000 0.000 0.023 0.008 0.000 0.023 0.000 0.000 0.000 0.000

0.053 0.072 0.059 0.048 0.005 0.005 0.000 0.005 0.000 0.005

0.000 0.005 0.000 0.000 0.000 0.000 0.019 0.000 0.005 0.005

0.000 0.000 0.010 0.000 0.057 0.061 0.074 0.080 0.020 0.000

0.000 0.010 0.000 0.020 0.000 0.000 0.000 0.010 0.000 0.000

0.039 0.000 0.000 0.010 0.000 0.010 0.000 0.000 0.070 0.036

0.068 0.041

>Non-Methylation code ID 21352

0.007 0.007 0.007 0.000 0.000 0.007 0.000 0.000 0.000 0.000

0.000 0.007 0.000 0.015 0.000 0.015 0.007 0.007 0.000 0.000

0.050 0.065 0.065 0.071 0.019 0.000 0.000 0.006 0.006 0.000

0.000 0.000 0.006 0.000 0.000 0.000 0.006 0.006 0.000 0.012

0.012 0.000 0.006 0.000 0.069 0.066 0.045 0.072 0.005 0.000

0.000 0.000 0.000 0.005 0.000 0.000 0.009 0.009 0.000 0.005

0.005 0.000 0.018 0.000 0.000 0.000 0.000 0.000 0.075 0.074

0.059 0.070

>Non-Methylation code ID 21352

0.007 0.000 0.000 0.000 0.013 0.013 0.007 0.000 0.000 0.007

0.000 0.000 0.000 0.007 0.007 0.007 0.000 0.007 0.000 0.007

0.072 0.083 0.039 0.060 0.000 0.000 0.000 0.011 0.006 0.011

0.000 0.000 0.000 0.000 0.006 0.000 0.000 0.000 0.011 0.017

0.006 0.006 0.000 0.000 0.063 0.064 0.071 0.061 0.006 0.006

0.000 0.000 0.013 0.013 0.000 0.000 0.006 0.013 0.000 0.000

0.006 0.006 0.000 0.006 0.000 0.000 0.000 0.000 0.066 0.065

0.058 0.069

>Non-Methylation code ID 21352

0.000 0.004 0.004 0.004 0.000 0.004 0.004 0.000 0.000 0.013

0.000 0.000 0.009 0.000 0.009 0.000 0.000 0.000 0.000 0.000

0.072 0.076 0.073 0.059 0.023 0.000 0.008 0.008 0.000 0.000

0.000 0.008 0.008 0.000 0.000 0.000 0.015 0.000 0.000 0.023

0.008 0.000 0.000 0.000 0.051 0.056 0.066 0.060 0.000 0.000

0.000 0.000 0.000 0.000 0.000 0.000 0.000 0.011 0.000 0.000

0.027 0.005 0.011 0.005 0.005 0.000 0.000 0.000 0.062 0.059

0.077 0.069

>Non-Methylation code ID 21354

0.012 0.006 0.006 0.006 0.000 0.000 0.000 0.006 0.000 0.000

0.000 0.000 0.006 0.000 0.006 0.012 0.006 0.000 0.000 0.000

0.066 0.067 0.071 0.065 0.009 0.000 0.000 0.000 0.000 0.005

0.000 0.000 0.000 0.005 0.000 0.000 0.019 0.000 0.014 0.005

0.005 0.000 0.000 0.000 0.068 0.060 0.076 0.069 0.000 0.005

0.005 0.005 0.000 0.005 0.005 0.000 0.000 0.020 0.000 0.000

0.010 0.000 0.005 0.000 0.000 0.000 0.000 0.000 0.066 0.069

0.069 0.071

>Non-Methylation code ID 21354

0.006 0.006 0.000 0.006 0.000 0.012 0.000 0.006 0.000 0.000

0.000 0.000 0.025 0.000 0.000 0.006 0.000 0.000 0.012 0.000

0.052 0.056 0.065 0.081 0.016 0.000 0.000 0.008 0.000 0.016

0.000 0.000 0.008 0.008 0.000 0.000 0.032 0.000 0.000 0.008

0.000 0.008 0.000 0.000 0.069 0.037 0.070 0.052 0.000 0.000

0.000 0.000 0.000 0.013 0.006 0.000 0.000 0.013 0.000 0.000

0.013 0.006 0.006 0.019 0.000 0.000 0.000 0.000 0.062 0.070

0.050 0.075

>Non-Methylation code ID 21354

0.007 0.007 0.007 0.007 0.000 0.007 0.000 0.007 0.000 0.007

0.000 0.000 0.013 0.000 0.000 0.013 0.013 0.000 0.000 0.000

0.058 0.073 0.058 0.058 0.014 0.000 0.000 0.000 0.000 0.007

0.000 0.000 0.000 0.022 0.000 0.000 0.022 0.000 0.007 0.007

0.007 0.000 0.000 0.000 0.069 0.067 0.048 0.063 0.000 0.000

0.006 0.006 0.000 0.006 0.006 0.000 0.000 0.019 0.000 0.000

0.012 0.000 0.006 0.000 0.000 0.000 0.000 0.000 0.072 0.083

0.064 0.052

>Non-Methylation code ID 21354

0.007 0.007 0.007 0.000 0.007 0.028 0.000 0.000 0.000 0.000

0.000 0.000 0.000 0.007 0.000 0.014 0.014 0.000 0.000 0.000

0.063 0.072 0.057 0.052 0.010 0.000 0.005 0.005 0.000 0.005

0.000 0.000 0.000 0.010 0.000 0.000 0.000 0.000 0.015 0.005

0.005 0.000 0.000 0.000 0.075 0.073 0.037 0.090 0.000 0.000

0.000 0.000 0.000 0.016 0.005 0.005 0.000 0.005 0.000 0.000

0.000 0.005 0.011 0.005 0.000 0.005 0.005 0.000 0.077 0.069

0.070 0.052

>Non-Methylation code ID 21354

0.010 0.010 0.000 0.000 0.000 0.005 0.000 0.000 0.000 0.000

0.000 0.000 0.000 0.000 0.010 0.000 0.000 0.000 0.005 0.005

0.083 0.058 0.073 0.075 0.005 0.016 0.000 0.000 0.000 0.005

0.005 0.000 0.000 0.000 0.000 0.000 0.000 0.000 0.005 0.000

0.005 0.000 0.005 0.000 0.080 0.090 0.056 0.059 0.005 0.016

0.000 0.000 0.000 0.011 0.000 0.000 0.000 0.000 0.000 0.000

0.000 0.000 0.011 0.000 0.005 0.005 0.000 0.000 0.095 0.093

0.088 0.004

>Non-Methylation code ID 21354

0.006 0.017 0.000 0.000 0.000 0.011 0.000 0.000 0.000 0.000

0.006 0.000 0.000 0.000 0.006 0.006 0.006 0.000 0.006 0.000

0.064 0.080 0.070 0.057 0.016 0.011 0.000 0.000 0.000 0.016

0.000 0.000 0.000 0.000 0.000 0.000 0.000 0.005 0.005 0.000

0.000 0.000 0.005 0.000 0.082 0.067 0.081 0.043 0.000 0.006

0.000 0.000 0.000 0.012 0.012 0.000 0.006 0.000 0.000 0.000

0.000 0.000 0.006 0.000 0.000 0.000 0.006 0.000 0.075 0.070

0.076 0.066

>Non-Methylation code ID 21354

0.000 0.000 0.005 0.005 0.000 0.010 0.000 0.000 0.000 0.010

0.000 0.000 0.000 0.000 0.020 0.005 0.000 0.000 0.000 0.000

0.094 0.051 0.079 0.056 0.005 0.011 0.005 0.000 0.000 0.016

0.000 0.005 0.000 0.000 0.000 0.000 0.000 0.000 0.005 0.005

0.000 0.005 0.005 0.000 0.063 0.065 0.059 0.081 0.017 0.009

0.000 0.009 0.000 0.035 0.000 0.000 0.000 0.000 0.000 0.000

0.009 0.009 0.000 0.009 0.009 0.000 0.000 0.000 0.059 0.083

0.053 0.034

>Non-Methylation code ID 21354

0.006 0.032 0.000 0.000 0.000 0.006 0.000 0.000 0.000 0.000

0.006 0.000 0.000 0.000 0.000 0.000 0.006 0.000 0.006 0.000

0.075 0.079 0.055 0.060 0.014 0.014 0.000 0.000 0.000 0.010

0.000 0.000 0.000 0.000 0.000 0.000 0.000 0.000 0.010 0.000

0.000 0.000 0.005 0.000 0.094 0.080 0.069 0.038 0.000 0.008

0.000 0.000 0.000 0.025 0.016 0.000 0.000 0.000 0.000 0.000

0.000 0.000 0.008 0.000 0.000 0.000 0.000 0.000 0.066 0.076

0.056 0.078

>Non-Methylation code ID 21354

0.006 0.006 0.006 0.000 0.000 0.006 0.000 0.000 0.000 0.000

0.000 0.006 0.000 0.012 0.006 0.006 0.006 0.006 0.000 0.000

0.053 0.062 0.069 0.085 0.013 0.000 0.000 0.013 0.006 0.000

0.000 0.000 0.006 0.000 0.000 0.000 0.006 0.006 0.000 0.013

0.013 0.000 0.006 0.000 0.069 0.074 0.051 0.058 0.009 0.000

0.000 0.000 0.000 0.005 0.000 0.000 0.009 0.009 0.000 0.005

0.005 0.000 0.014 0.000 0.000 0.000 0.000 0.000 0.075 0.076

0.055 0.071

>Non-Methylation code ID 21354

0.016 0.011 0.000 0.000 0.000 0.000 0.000 0.000 0.000 0.000

0.000 0.000 0.005 0.000 0.011 0.016 0.011 0.000 0.000 0.000

0.056 0.085 0.081 0.042 0.011 0.000 0.000 0.000 0.000 0.011

0.000 0.000 0.000 0.011 0.000 0.000 0.006 0.017 0.011 0.000

0.000 0.000 0.000 0.000 0.072 0.076 0.043 0.075 0.000 0.011

0.005 0.011 0.000 0.005 0.000 0.000 0.005 0.016 0.000 0.005

0.000 0.000 0.000 0.005 0.000 0.000 0.000 0.000 0.070 0.062

0.066 0.072

>Non-Methylation code ID 21354

0.022 0.000 0.000 0.000 0.000 0.000 0.000 0.000 0.000 0.011

0.000 0.000 0.016 0.000 0.011 0.005 0.000 0.000 0.000 0.000

0.070 0.066 0.054 0.079 0.018 0.000 0.000 0.000 0.006 0.000

0.000 0.000 0.000 0.006 0.000 0.000 0.030 0.000 0.006 0.000

0.000 0.000 0.006 0.000 0.064 0.064 0.071 0.062 0.006 0.000

0.000 0.006 0.000 0.006 0.000 0.000 0.000 0.000 0.000 0.000

0.023 0.000 0.017 0.011 0.000 0.000 0.000 0.000 0.070 0.065

0.045 0.085

>Non-Methylation code ID 21354

0.016 0.000 0.000 0.000 0.000 0.000 0.005 0.000 0.000 0.011

0.000 0.005 0.011 0.000 0.011 0.011 0.000 0.000 0.000 0.000

0.065 0.054 0.064 0.080 0.018 0.009 0.000 0.000 0.000 0.000

0.000 0.000 0.000 0.000 0.000 0.000 0.014 0.000 0.009 0.000

0.009 0.000 0.000 0.000 0.076 0.051 0.063 0.085 0.018 0.000

0.000 0.000 0.000 0.006 0.000 0.000 0.000 0.000 0.000 0.000

0.018 0.018 0.012 0.000 0.000 0.000 0.000 0.000 0.063 0.085

0.062 0.051

>Non-Methylation code ID 21354

0.011 0.000 0.000 0.000 0.000 0.000 0.006 0.000 0.006 0.017

0.000 0.000 0.011 0.000 0.006 0.011 0.000 0.006 0.000 0.000

0.049 0.078 0.073 0.062 0.012 0.008 0.000 0.000 0.000 0.000

0.000 0.000 0.000 0.000 0.000 0.000 0.008 0.000 0.008 0.008

0.004 0.000 0.004 0.000 0.070 0.051 0.073 0.088 0.028 0.000

0.000 0.000 0.000 0.014 0.000 0.000 0.000 0.000 0.000 0.000

0.014 0.021 0.007 0.000 0.000 0.000 0.000 0.000 0.070 0.079

0.044 0.057

>Non-Methylation code ID 21354

0.012 0.004 0.000 0.000 0.000 0.000 0.000 0.000 0.000 0.000

0.000 0.000 0.000 0.000 0.016 0.012 0.008 0.000 0.000 0.000

0.062 0.058 0.082 0.079 0.012 0.000 0.000 0.000 0.000 0.004

0.000 0.000 0.000 0.000 0.000 0.000 0.008 0.004 0.016 0.000

0.000 0.008 0.000 0.000 0.070 0.067 0.061 0.083 0.010 0.000

0.005 0.005 0.010 0.000 0.005 0.000 0.000 0.000 0.000 0.000

0.005 0.000 0.010 0.005 0.000 0.000 0.000 0.000 0.075 0.058

0.072 0.072

>Non-Methylation code ID 21354

0.000 0.000 0.006 0.000 0.000 0.006 0.000 0.011 0.006 0.011

0.000 0.006 0.006 0.006 0.006 0.000 0.006 0.000 0.000 0.006

0.061 0.059 0.069 0.072 0.000 0.000 0.000 0.011 0.000 0.011

0.000 0.000 0.000 0.000 0.000 0.000 0.021 0.000 0.000 0.053

0.032 0.000 0.000 0.000 0.052 0.047 0.055 0.053 0.016 0.000

0.000 0.005 0.000 0.000 0.010 0.000 0.000 0.005 0.000 0.005

0.005 0.005 0.010 0.000 0.000 0.000 0.000 0.000 0.060 0.066

0.070 0.075

>Non-Methylation code ID 21354

0.000 0.012 0.006 0.006 0.000 0.006 0.000 0.000 0.000 0.035

0.000 0.006 0.000 0.000 0.000 0.006 0.000 0.000 0.000 0.000

0.067 0.054 0.062 0.074 0.015 0.020 0.000 0.000 0.000 0.000

0.000 0.000 0.000 0.000 0.000 0.000 0.000 0.000 0.005 0.015

0.010 0.000 0.000 0.000 0.053 0.077 0.086 0.052 0.034 0.000

0.000 0.000 0.000 0.000 0.000 0.000 0.000 0.014 0.000 0.000

0.007 0.007 0.014 0.000 0.000 0.000 0.000 0.000 0.070 0.061

0.050 0.078

>Non-Methylation code ID 21354

0.009 0.000 0.000 0.000 0.000 0.013 0.004 0.000 0.000 0.000

0.000 0.000 0.009 0.004 0.013 0.000 0.000 0.004 0.000 0.000

0.051 0.079 0.085 0.061 0.000 0.000 0.011 0.000 0.006 0.006

0.000 0.006 0.006 0.011 0.000 0.000 0.011 0.000 0.000 0.000

0.000 0.006 0.000 0.000 0.050 0.072 0.078 0.071 0.006 0.006

0.000 0.000 0.000 0.000 0.000 0.000 0.000 0.000 0.000 0.000

0.011 0.000 0.000 0.023 0.006 0.000 0.017 0.000 0.052 0.063

0.081 0.069

>Non-Methylation code ID 21354

0.014 0.018 0.000 0.000 0.000 0.000 0.000 0.000 0.000 0.000

0.000 0.000 0.000 0.000 0.005 0.009 0.009 0.000 0.005 0.000

0.055 0.074 0.085 0.060 0.032 0.000 0.000 0.000 0.000 0.006

0.000 0.000 0.000 0.013 0.000 0.000 0.006 0.006 0.013 0.000

0.000 0.000 0.000 0.000 0.082 0.059 0.046 0.070 0.000 0.013

0.006 0.006 0.000 0.006 0.000 0.000 0.000 0.032 0.000 0.006

0.000 0.000 0.000 0.006 0.000 0.000 0.000 0.000 0.067 0.049

0.069 0.071

>Non-Methylation code ID 21354

0.011 0.000 0.005 0.000 0.000 0.000 0.000 0.005 0.005 0.011

0.000 0.000 0.005 0.000 0.005 0.011 0.000 0.005 0.000 0.000

0.043 0.073 0.080 0.071 0.013 0.004 0.000 0.000 0.000 0.000

0.000 0.000 0.000 0.000 0.000 0.000 0.004 0.000 0.009 0.013

0.004 0.000 0.009 0.000 0.074 0.058 0.075 0.070 0.016 0.000

0.000 0.000 0.000 0.011 0.005 0.000 0.000 0.000 0.000 0.000

0.005 0.011 0.016 0.000 0.000 0.000 0.000 0.000 0.062 0.077

0.056 0.075

>Non-Methylation code ID 21354

0.019 0.013 0.000 0.000 0.000 0.006 0.000 0.000 0.000 0.000

0.000 0.000 0.019 0.000 0.006 0.000 0.019 0.000 0.000 0.000

0.071 0.057 0.066 0.058 0.016 0.000 0.000 0.000 0.000 0.005

0.000 0.000 0.000 0.000 0.000 0.000 0.021 0.016 0.011 0.000

0.000 0.000 0.000 0.000 0.072 0.082 0.052 0.057 0.005 0.000

0.005 0.000 0.000 0.000 0.005 0.000 0.000 0.011 0.000 0.005

0.016 0.000 0.005 0.011 0.000 0.000 0.000 0.000 0.075 0.051

0.072 0.071

>Non-Methylation code ID 21354

0.017 0.000 0.000 0.000 0.000 0.000 0.000 0.000 0.000 0.017

0.000 0.000 0.017 0.000 0.011 0.006 0.000 0.000 0.000 0.000

0.071 0.069 0.043 0.082 0.018 0.006 0.000 0.000 0.006 0.000

0.000 0.000 0.000 0.006 0.000 0.000 0.024 0.000 0.006 0.000

0.000 0.000 0.006 0.000 0.061 0.054 0.069 0.076 0.005 0.000

0.000 0.005 0.000 0.005 0.000 0.000 0.000 0.005 0.000 0.000

0.015 0.000 0.015 0.010 0.000 0.000 0.000 0.000 0.072 0.068

0.042 0.090

>Non-Methylation code ID 21354

0.006 0.006 0.000 0.000 0.000 0.000 0.000 0.000 0.000 0.000

0.000 0.000 0.011 0.000 0.000 0.022 0.011 0.000 0.017 0.000

0.053 0.067 0.077 0.065 0.005 0.000 0.000 0.000 0.000 0.014

0.005 0.000 0.000 0.005 0.000 0.000 0.009 0.005 0.014 0.000

0.000 0.005 0.000 0.000 0.066 0.078 0.080 0.049 0.000 0.000

0.012 0.000 0.006 0.006 0.000 0.006 0.006 0.006 0.000 0.000

0.012 0.000 0.000 0.000 0.000 0.006 0.000 0.000 0.052 0.077

0.073 0.072

>Non-Methylation code ID 21354

0.016 0.011 0.000 0.000 0.000 0.000 0.000 0.000 0.000 0.000

0.000 0.000 0.016 0.000 0.011 0.000 0.016 0.000 0.000 0.000

0.078 0.048 0.063 0.074 0.016 0.000 0.000 0.000 0.000 0.005

0.000 0.000 0.000 0.000 0.000 0.000 0.022 0.016 0.011 0.000

0.000 0.000 0.000 0.000 0.074 0.080 0.064 0.044 0.019 0.000

0.000 0.000 0.000 0.000 0.006 0.000 0.000 0.012 0.000 0.006

0.012 0.000 0.006 0.012 0.000 0.000 0.000 0.000 0.075 0.046

0.059 0.079

>Non-Methylation code ID 21354

0.000 0.000 0.000 0.015 0.000 0.005 0.000 0.000 0.000 0.005

0.000 0.005 0.000 0.000 0.010 0.005 0.005 0.000 0.005 0.000

0.066 0.067 0.078 0.069 0.005 0.000 0.005 0.005 0.000 0.005

0.000 0.000 0.005 0.005 0.000 0.000 0.000 0.000 0.011 0.011

0.005 0.000 0.005 0.000 0.061 0.070 0.053 0.084 0.008 0.008

0.000 0.000 0.000 0.031 0.008 0.000 0.008 0.016 0.000 0.000

0.000 0.000 0.000 0.008 0.008 0.000 0.000 0.000 0.068 0.068

0.059 0.043

>Non-Methylation code ID 21354

0.016 0.000 0.000 0.000 0.008 0.023 0.008 0.000 0.000 0.016

0.008 0.000 0.016 0.000 0.000 0.008 0.000 0.000 0.000 0.000

0.060 0.055 0.058 0.059 0.005 0.005 0.000 0.000 0.000 0.014

0.000 0.000 0.000 0.005 0.000 0.000 0.010 0.000 0.010 0.005

0.005 0.000 0.005 0.000 0.081 0.069 0.063 0.058 0.019 0.000

0.000 0.000 0.000 0.019 0.000 0.000 0.000 0.000 0.000 0.000

0.013 0.006 0.006 0.000 0.000 0.006 0.006 0.000 0.073 0.074

0.063 0.048

>Non-Methylation code ID 21354

0.006 0.000 0.000 0.000 0.000 0.006 0.000 0.000 0.000 0.012

0.000 0.000 0.019 0.012 0.006 0.006 0.006 0.000 0.000 0.000

0.065 0.069 0.050 0.076 0.004 0.004 0.000 0.000 0.000 0.000

0.000 0.000 0.004 0.008 0.000 0.000 0.008 0.000 0.015 0.004

0.000 0.004 0.000 0.000 0.065 0.062 0.066 0.090 0.018 0.000

0.009 0.000 0.000 0.009 0.000 0.000 0.000 0.009 0.000 0.000

0.037 0.000 0.000 0.018 0.000 0.009 0.000 0.000 0.059 0.059

0.052 0.051

>Non-Methylation code ID 21354

0.008 0.000 0.008 0.000 0.000 0.024 0.000 0.008 0.000 0.016

0.000 0.000 0.016 0.008 0.000 0.000 0.008 0.000 0.000 0.000

0.045 0.053 0.070 0.067 0.000 0.000 0.005 0.005 0.005 0.005

0.000 0.000 0.000 0.005 0.000 0.000 0.000 0.010 0.016 0.005

0.005 0.000 0.000 0.000 0.046 0.063 0.087 0.075 0.000 0.000

0.005 0.000 0.000 0.005 0.000 0.000 0.000 0.000 0.000 0.005

0.010 0.000 0.010 0.005 0.005 0.010 0.005 0.000 0.080 0.055

0.073 0.066

>Non-Methylation code ID 21354

0.000 0.000 0.000 0.014 0.000 0.014 0.000 0.000 0.000 0.010

0.000 0.005 0.000 0.000 0.010 0.000 0.000 0.000 0.005 0.000

0.060 0.072 0.074 0.070 0.005 0.000 0.005 0.011 0.000 0.011

0.000 0.000 0.005 0.000 0.000 0.000 0.000 0.000 0.011 0.011

0.005 0.000 0.000 0.000 0.055 0.066 0.060 0.087 0.006 0.000

0.000 0.006 0.000 0.018 0.000 0.000 0.006 0.012 0.000 0.000

0.006 0.000 0.012 0.000 0.006 0.000 0.000 0.000 0.065 0.092

0.057 0.047

>Non-Methylation code ID 21354

0.004 0.000 0.000 0.000 0.000 0.008 0.000 0.000 0.000 0.000

0.000 0.000 0.008 0.000 0.017 0.004 0.004 0.004 0.004 0.000

0.080 0.050 0.058 0.091 0.012 0.000 0.006 0.000 0.000 0.023

0.000 0.000 0.000 0.000 0.000 0.000 0.018 0.006 0.006 0.006

0.000 0.000 0.000 0.000 0.068 0.073 0.062 0.054 0.021 0.000

0.000 0.000 0.000 0.032 0.000 0.000 0.000 0.021 0.000 0.000

0.011 0.021 0.000 0.011 0.011 0.000 0.000 0.000 0.047 0.047

0.048 0.065

>Non-Methylation code ID 21354

0.011 0.000 0.000 0.000 0.005 0.000 0.005 0.000 0.000 0.005

0.000 0.000 0.016 0.000 0.005 0.000 0.016 0.000 0.005 0.000

0.070 0.085 0.054 0.055 0.006 0.000 0.000 0.000 0.006 0.006

0.012 0.000 0.000 0.006 0.000 0.000 0.025 0.006 0.000 0.006

0.006 0.000 0.000 0.000 0.050 0.073 0.067 0.062 0.000 0.000

0.000 0.000 0.000 0.007 0.007 0.000 0.000 0.014 0.000 0.007

0.014 0.000 0.007 0.014 0.014 0.000 0.000 0.000 0.065 0.060

0.050 0.074

>Non-Methylation code ID 21354

0.013 0.006 0.000 0.013 0.000 0.013 0.000 0.000 0.000 0.006

0.000 0.000 0.000 0.006 0.006 0.013 0.006 0.000 0.000 0.000

0.063 0.065 0.053 0.068 0.011 0.000 0.000 0.000 0.000 0.011

0.000 0.000 0.005 0.011 0.000 0.005 0.016 0.000 0.005 0.000

0.000 0.005 0.000 0.000 0.070 0.049 0.072 0.074 0.000 0.000

0.004 0.000 0.000 0.009 0.000 0.000 0.004 0.004 0.004 0.000

0.009 0.000 0.009 0.004 0.000 0.000 0.004 0.000 0.063 0.080

0.070 0.069

>Non-Methylation code ID 21354

0.011 0.000 0.000 0.000 0.000 0.011 0.000 0.000 0.005 0.011

0.000 0.005 0.016 0.000 0.005 0.000 0.000 0.005 0.000 0.000

0.070 0.049 0.072 0.074 0.000 0.000 0.004 0.000 0.004 0.008

0.000 0.000 0.004 0.004 0.004 0.000 0.008 0.000 0.008 0.004

0.000 0.000 0.004 0.000 0.066 0.075 0.064 0.077 0.013 0.007

0.000 0.013 0.000 0.007 0.000 0.000 0.000 0.007 0.000 0.000

0.000 0.007 0.007 0.013 0.007 0.000 0.000 0.000 0.059 0.067

0.056 0.070

>Non-Methylation code ID 21354

0.000 0.019 0.000 0.000 0.000 0.006 0.000 0.000 0.000 0.019

0.000 0.006 0.006 0.000 0.000 0.006 0.000 0.006 0.006 0.000

0.084 0.085 0.049 0.037 0.000 0.012 0.000 0.000 0.006 0.012

0.000 0.006 0.000 0.012 0.000 0.006 0.000 0.000 0.000 0.006

0.000 0.012 0.000 0.000 0.062 0.047 0.075 0.075 0.010 0.010

0.000 0.000 0.000 0.010 0.000 0.000 0.000 0.029 0.010 0.000

0.010 0.000 0.000 0.019 0.000 0.000 0.000 0.000 0.041 0.062

0.074 0.060

>Non-Methylation code ID 21354

0.000 0.000 0.000 0.000 0.007 0.007 0.007 0.000 0.000 0.013

0.000 0.000 0.026 0.007 0.000 0.013 0.007 0.000 0.000 0.000

0.060 0.064 0.048 0.075 0.006 0.000 0.000 0.000 0.012 0.000

0.006 0.000 0.000 0.012 0.000 0.006 0.023 0.000 0.000 0.000

0.006 0.000 0.000 0.006 0.064 0.067 0.063 0.064 0.000 0.000

0.000 0.000 0.007 0.000 0.007 0.013 0.000 0.000 0.000 0.000

0.034 0.000 0.000 0.013 0.007 0.000 0.000 0.000 0.046 0.061

0.074 0.072

>Non-Methylation code ID 21357

0.006 0.000 0.000 0.000 0.013 0.006 0.006 0.000 0.000 0.006

0.000 0.000 0.019 0.006 0.000 0.006 0.013 0.000 0.000 0.000

0.058 0.072 0.055 0.064 0.000 0.000 0.000 0.000 0.000 0.006

0.006 0.000 0.000 0.012 0.000 0.006 0.018 0.000 0.006 0.018

0.006 0.000 0.000 0.000 0.065 0.061 0.056 0.072 0.011 0.000

0.000 0.000 0.005 0.000 0.011 0.000 0.000 0.005 0.000 0.000

0.011 0.000 0.005 0.000 0.011 0.000 0.005 0.000 0.072 0.072

0.061 0.062

>Non-Methylation code ID 21357

0.000 0.000 0.000 0.017 0.009 0.026 0.000 0.000 0.000 0.017

0.000 0.000 0.000 0.017 0.000 0.000 0.017 0.000 0.000 0.000

0.048 0.046 0.070 0.065 0.000 0.000 0.000 0.000 0.000 0.010

0.000 0.000 0.005 0.000 0.000 0.005 0.010 0.000 0.010 0.015

0.000 0.005 0.005 0.000 0.084 0.046 0.085 0.055 0.007 0.000

0.007 0.000 0.000 0.027 0.000 0.007 0.000 0.007 0.000 0.000

0.020 0.000 0.007 0.000 0.000 0.000 0.000 0.000 0.059 0.063

0.057 0.073

>Non-Methylation code ID 21357

0.006 0.000 0.000 0.000 0.011 0.000 0.006 0.000 0.000 0.006

0.000 0.006 0.022 0.000 0.000 0.000 0.006 0.000 0.006 0.006

0.068 0.071 0.059 0.063 0.000 0.000 0.000 0.000 0.006 0.006

0.006 0.013 0.000 0.000 0.000 0.000 0.031 0.000 0.000 0.013

0.006 0.000 0.000 0.000 0.048 0.061 0.076 0.067 0.000 0.000

0.000 0.000 0.007 0.007 0.007 0.000 0.000 0.015 0.000 0.000

0.022 0.007 0.000 0.015 0.007 0.000 0.000 0.000 0.061 0.067

0.039 0.078

>Non-Methylation code ID 21357

0.000 0.000 0.000 0.000 0.007 0.000 0.007 0.013 0.000 0.007

0.000 0.000 0.033 0.000 0.000 0.013 0.007 0.000 0.000 0.000

0.048 0.059 0.074 0.068 0.000 0.006 0.000 0.000 0.006 0.000

0.006 0.000 0.000 0.012 0.000 0.000 0.024 0.006 0.000 0.012

0.006 0.000 0.000 0.000 0.051 0.066 0.053 0.085 0.000 0.000

0.000 0.000 0.013 0.000 0.006 0.000 0.000 0.013 0.000 0.006

0.025 0.000 0.000 0.000 0.006 0.000 0.000 0.006 0.066 0.067

0.057 0.068

>Non-Methylation code ID 21357

0.006 0.000 0.006 0.000 0.000 0.006 0.000 0.000 0.006 0.006

0.000 0.000 0.006 0.000 0.011 0.011 0.006 0.000 0.006 0.000

0.056 0.072 0.059 0.079 0.007 0.007 0.000 0.000 0.000 0.029

0.007 0.000 0.007 0.014 0.000 0.000 0.000 0.007 0.000 0.007

0.007 0.000 0.000 0.000 0.065 0.063 0.067 0.045 0.000 0.000

0.000 0.016 0.000 0.000 0.000 0.000 0.000 0.005 0.000 0.005

0.000 0.000 0.011 0.005 0.005 0.000 0.005 0.000 0.067 0.068

0.081 0.064

>Non-Methylation code ID 21357

0.000 0.013 0.000 0.000 0.013 0.000 0.000 0.006 0.000 0.013

0.000 0.013 0.000 0.000 0.000 0.006 0.000 0.006 0.000 0.006

0.071 0.049 0.067 0.070 0.009 0.009 0.000 0.000 0.000 0.000

0.000 0.018 0.000 0.018 0.009 0.000 0.009 0.000 0.000 0.027

0.000 0.000 0.000 0.000 0.048 0.051 0.077 0.059 0.000 0.014

0.000 0.000 0.007 0.000 0.000 0.000 0.000 0.027 0.000 0.007

0.007 0.000 0.000 0.007 0.000 0.007 0.000 0.000 0.054 0.077

0.063 0.065

>Non-Methylation code ID 21357

0.010 0.005 0.000 0.005 0.000 0.015 0.000 0.000 0.000 0.005

0.000 0.000 0.005 0.000 0.005 0.005 0.005 0.000 0.005 0.000

0.074 0.066 0.064 0.064 0.011 0.000 0.000 0.000 0.000 0.017

0.000 0.000 0.000 0.000 0.000 0.006 0.017 0.006 0.006 0.000

0.000 0.006 0.006 0.000 0.079 0.061 0.062 0.058 0.007 0.000

0.000 0.000 0.007 0.020 0.007 0.000 0.000 0.013 0.007 0.000

0.007 0.000 0.007 0.007 0.000 0.000 0.000 0.000 0.060 0.060

0.065 0.069

>Non-Methylation code ID 21357

0.021 0.000 0.000 0.000 0.000 0.042 0.000 0.000 0.000 0.021

0.010 0.010 0.010 0.000 0.000 0.010 0.010 0.000 0.000 0.000

0.045 0.064 0.046 0.042 0.009 0.005 0.000 0.000 0.000 0.009

0.000 0.000 0.000 0.014 0.000 0.000 0.000 0.000 0.009 0.000

0.005 0.000 0.005 0.000 0.076 0.057 0.070 0.074 0.000 0.005

0.000 0.005 0.005 0.014 0.005 0.000 0.000 0.005 0.000 0.005

0.000 0.000 0.005 0.000 0.000 0.005 0.005 0.000 0.071 0.066

0.069 0.069

>Non-Methylation code ID 21357

0.005 0.000 0.000 0.000 0.000 0.011 0.000 0.000 0.005 0.000

0.000 0.005 0.005 0.000 0.011 0.016 0.000 0.005 0.005 0.000

0.085 0.044 0.079 0.055 0.006 0.000 0.006 0.000 0.000 0.023

0.000 0.006 0.006 0.006 0.000 0.000 0.012 0.000 0.012 0.000

0.000 0.000 0.000 0.000 0.056 0.068 0.052 0.082 0.000 0.000

0.000 0.019 0.010 0.029 0.000 0.000 0.000 0.019 0.000 0.000

0.000 0.010 0.000 0.010 0.010 0.000 0.000 0.000 0.053 0.047

0.069 0.060

>Non-Methylation code ID 21357

0.018 0.000 0.000 0.000 0.000 0.028 0.000 0.000 0.000 0.018

0.000 0.000 0.018 0.018 0.000 0.009 0.009 0.000 0.000 0.000

0.048 0.051 0.050 0.065 0.004 0.000 0.000 0.000 0.000 0.008

0.000 0.000 0.000 0.000 0.000 0.000 0.012 0.000 0.016 0.004

0.000 0.004 0.004 0.000 0.076 0.053 0.057 0.095 0.013 0.000

0.006 0.000 0.000 0.025 0.000 0.000 0.000 0.000 0.000 0.000

0.019 0.000 0.006 0.006 0.000 0.000 0.000 0.000 0.068 0.075

0.063 0.051

>Non-Methylation code ID 21357

0.007 0.000 0.000 0.007 0.000 0.000 0.000 0.000 0.000 0.007

0.000 0.000 0.007 0.000 0.014 0.014 0.014 0.000 0.000 0.000

0.071 0.048 0.082 0.062 0.009 0.000 0.004 0.000 0.000 0.000

0.000 0.000 0.000 0.004 0.000 0.000 0.004 0.000 0.013 0.013

0.000 0.000 0.004 0.000 0.058 0.062 0.077 0.083 0.014 0.007

0.000 0.000 0.000 0.014 0.014 0.000 0.000 0.000 0.000 0.000

0.000 0.007 0.000 0.007 0.007 0.014 0.000 0.000 0.049 0.057

0.077 0.069

>Non-Methylation code ID 21357

0.005 0.000 0.000 0.010 0.000 0.010 0.000 0.000 0.000 0.005

0.000 0.000 0.015 0.005 0.015 0.000 0.000 0.000 0.000 0.000

0.068 0.085 0.071 0.045 0.006 0.000 0.006 0.000 0.000 0.018

0.000 0.000 0.000 0.018 0.000 0.000 0.006 0.000 0.006 0.018

0.000 0.000 0.000 0.000 0.054 0.065 0.062 0.073 0.018 0.000

0.000 0.012 0.000 0.006 0.000 0.000 0.000 0.000 0.000 0.000

0.012 0.000 0.006 0.006 0.000 0.000 0.006 0.000 0.052 0.076

0.075 0.065

>Non-Methylation code ID 21357

0.000 0.000 0.005 0.000 0.005 0.009 0.005 0.000 0.000 0.009

0.005 0.000 0.009 0.000 0.005 0.005 0.000 0.000 0.005 0.000

0.071 0.071 0.062 0.070 0.010 0.005 0.000 0.005 0.000 0.010

0.000 0.000 0.000 0.005 0.000 0.000 0.000 0.005 0.005 0.005

0.010 0.000 0.005 0.000 0.064 0.065 0.061 0.077 0.011 0.000

0.000 0.000 0.000 0.006 0.000 0.000 0.000 0.000 0.000 0.006

0.022 0.006 0.006 0.000 0.000 0.006 0.006 0.000 0.077 0.055

0.073 0.062

>Non-Methylation code ID 21357

0.025 0.000 0.008 0.000 0.000 0.008 0.000 0.000 0.000 0.008

0.000 0.000 0.034 0.000 0.000 0.017 0.000 0.008 0.000 0.000

0.059 0.066 0.047 0.052 0.006 0.000 0.000 0.000 0.000 0.006

0.000 0.000 0.000 0.012 0.000 0.000 0.019 0.012 0.006 0.006

0.006 0.000 0.000 0.000 0.065 0.069 0.050 0.076 0.004 0.004

0.000 0.000 0.000 0.000 0.000 0.000 0.004 0.004 0.000 0.000

0.008 0.000 0.016 0.004 0.000 0.004 0.000 0.000 0.059 0.060

0.072 0.092

>Non-Methylation code ID 21357

0.004 0.004 0.000 0.000 0.000 0.008 0.000 0.000 0.000 0.000

0.000 0.000 0.012 0.000 0.016 0.004 0.000 0.004 0.000 0.000

0.070 0.046 0.064 0.101 0.020 0.000 0.007 0.000 0.000 0.020

0.000 0.000 0.000 0.000 0.000 0.000 0.027 0.000 0.007 0.007

0.000 0.000 0.000 0.000 0.075 0.069 0.054 0.049 0.014 0.000

0.000 0.000 0.000 0.014 0.000 0.000 0.000 0.014 0.000 0.000

0.014 0.014 0.007 0.000 0.007 0.000 0.000 0.000 0.067 0.063

0.046 0.071

>Non-Methylation code ID 21357

0.000 0.012 0.000 0.000 0.006 0.012 0.006 0.006 0.000 0.006

0.000 0.006 0.000 0.000 0.000 0.006 0.000 0.012 0.000 0.000

0.067 0.054 0.079 0.064 0.010 0.010 0.000 0.000 0.000 0.019

0.000 0.000 0.000 0.029 0.010 0.000 0.010 0.000 0.000 0.010

0.010 0.000 0.000 0.000 0.040 0.069 0.065 0.053 0.007 0.021

0.000 0.000 0.000 0.007 0.000 0.000 0.000 0.021 0.000 0.007

0.000 0.000 0.000 0.000 0.000 0.007 0.007 0.000 0.084 0.082

0.053 0.038

>Non-Methylation code ID 21357

0.000 0.007 0.000 0.000 0.007 0.000 0.007 0.007 0.000 0.013

0.000 0.007 0.020 0.000 0.000 0.000 0.007 0.007 0.000 0.007

0.053 0.054 0.060 0.082 0.000 0.000 0.000 0.000 0.000 0.000

0.010 0.019 0.000 0.010 0.000 0.000 0.029 0.000 0.000 0.029

0.010 0.000 0.000 0.000 0.049 0.040 0.073 0.064 0.000 0.006

0.000 0.000 0.006 0.000 0.006 0.000 0.000 0.012 0.000 0.006

0.018 0.006 0.000 0.006 0.006 0.000 0.000 0.000 0.058 0.074

0.056 0.075

>Non-Methylation code ID 21357

0.006 0.000 0.000 0.000 0.012 0.000 0.006 0.000 0.000 0.012

0.000 0.006 0.023 0.000 0.000 0.000 0.006 0.000 0.000 0.006

0.064 0.067 0.063 0.064 0.000 0.000 0.000 0.000 0.007 0.000

0.007 0.013 0.000 0.007 0.000 0.000 0.033 0.000 0.000 0.013

0.007 0.000 0.000 0.000 0.048 0.059 0.074 0.068 0.000 0.000

0.000 0.000 0.007 0.000 0.007 0.000 0.000 0.014 0.000 0.000

0.028 0.007 0.000 0.014 0.007 0.000 0.000 0.000 0.058 0.066

0.050 0.075

>Non-Methylation code ID 21357

0.000 0.000 0.000 0.000 0.000 0.006 0.006 0.000 0.000 0.012

0.000 0.006 0.018 0.000 0.006 0.018 0.006 0.000 0.000 0.000

0.063 0.054 0.063 0.076 0.010 0.000 0.000 0.000 0.005 0.000

0.010 0.000 0.000 0.010 0.000 0.000 0.010 0.000 0.005 0.000

0.010 0.000 0.005 0.000 0.074 0.073 0.059 0.059 0.007 0.000

0.000 0.000 0.014 0.007 0.007 0.000 0.000 0.007 0.000 0.000

0.021 0.007 0.000 0.007 0.007 0.000 0.000 0.000 0.057 0.072

0.053 0.068

>Non-Methylation code ID 21357

0.000 0.000 0.000 0.000 0.006 0.006 0.006 0.012 0.000 0.000

0.000 0.000 0.031 0.000 0.000 0.012 0.006 0.000 0.000 0.000

0.045 0.065 0.076 0.067 0.000 0.000 0.000 0.000 0.007 0.007

0.007 0.000 0.000 0.021 0.000 0.000 0.021 0.007 0.000 0.014

0.007 0.000 0.000 0.000 0.061 0.069 0.036 0.076 0.006 0.000

0.000 0.000 0.012 0.000 0.006 0.000 0.000 0.000 0.000 0.006

0.023 0.000 0.000 0.000 0.006 0.000 0.006 0.006 0.070 0.075

0.056 0.063

>Non-Methylation code ID 21357

0.000 0.000 0.000 0.000 0.000 0.000 0.005 0.000 0.005 0.016

0.000 0.000 0.021 0.000 0.011 0.000 0.011 0.000 0.000 0.000

0.058 0.077 0.067 0.063 0.008 0.000 0.000 0.000 0.008 0.000

0.008 0.000 0.000 0.023 0.000 0.000 0.015 0.008 0.000 0.008

0.008 0.008 0.000 0.008 0.070 0.060 0.045 0.059 0.010 0.000

0.000 0.000 0.000 0.000 0.010 0.000 0.000 0.000 0.000 0.010

0.020 0.000 0.000 0.040 0.020 0.000 0.000 0.000 0.056 0.045

0.061 0.062

>Non-Methylation code ID 21357

0.000 0.009 0.000 0.000 0.000 0.000 0.009 0.018 0.000 0.009

0.000 0.000 0.027 0.000 0.000 0.027 0.000 0.000 0.000 0.000

0.054 0.043 0.074 0.064 0.000 0.011 0.000 0.000 0.005 0.000

0.005 0.000 0.000 0.011 0.000 0.005 0.011 0.005 0.000 0.005

0.005 0.005 0.000 0.000 0.063 0.073 0.057 0.069 0.000 0.008

0.000 0.000 0.008 0.000 0.000 0.008 0.000 0.016 0.000 0.008

0.016 0.000 0.000 0.000 0.008 0.008 0.000 0.008 0.054 0.048

0.070 0.076

>Non-Methylation code ID 21362

0.010 0.000 0.000 0.000 0.000 0.010 0.000 0.000 0.000 0.000

0.000 0.005 0.021 0.005 0.005 0.000 0.000 0.005 0.005 0.000

0.075 0.055 0.066 0.069 0.000 0.000 0.005 0.000 0.005 0.016

0.005 0.000 0.000 0.010 0.005 0.000 0.010 0.000 0.005 0.005

0.000 0.000 0.000 0.000 0.069 0.070 0.067 0.059 0.011 0.005

0.000 0.005 0.000 0.005 0.000 0.000 0.000 0.005 0.000 0.000

0.000 0.005 0.005 0.005 0.011 0.000 0.005 0.000 0.065 0.064

0.065 0.075

>Non-Methylation code ID 21362

0.000 0.000 0.006 0.000 0.006 0.006 0.006 0.000 0.000 0.006

0.000 0.000 0.012 0.000 0.006 0.006 0.018 0.000 0.000 0.000

0.069 0.063 0.062 0.068 0.005 0.000 0.000 0.005 0.000 0.000

0.000 0.000 0.000 0.011 0.000 0.000 0.011 0.000 0.011 0.005

0.011 0.005 0.005 0.000 0.078 0.065 0.052 0.069 0.008 0.000

0.000 0.000 0.000 0.008 0.016 0.000 0.000 0.008 0.000 0.008

0.008 0.008 0.000 0.016 0.000 0.008 0.000 0.000 0.062 0.061

0.041 0.080

>Non-Methylation code ID 21362

0.011 0.000 0.000 0.000 0.011 0.000 0.006 0.000 0.000 0.006

0.000 0.006 0.023 0.000 0.000 0.000 0.006 0.000 0.006 0.000

0.071 0.077 0.050 0.062 0.000 0.000 0.000 0.000 0.006 0.006

0.006 0.006 0.000 0.006 0.000 0.000 0.030 0.000 0.000 0.012

0.006 0.000 0.000 0.000 0.044 0.067 0.076 0.067 0.000 0.000

0.000 0.000 0.008 0.008 0.008 0.000 0.000 0.016 0.000 0.000

0.023 0.008 0.000 0.016 0.008 0.000 0.000 0.000 0.065 0.066

0.039 0.069

>Non-Methylation code ID 21362

0.011 0.000 0.000 0.000 0.000 0.042 0.000 0.000 0.000 0.021

0.000 0.011 0.011 0.000 0.000 0.021 0.011 0.000 0.000 0.000

0.050 0.058 0.042 0.056 0.009 0.000 0.004 0.000 0.004 0.009

0.004 0.000 0.000 0.004 0.000 0.000 0.004 0.000 0.009 0.000

0.004 0.000 0.004 0.000 0.082 0.059 0.069 0.067 0.000 0.000

0.000 0.005 0.000 0.016 0.005 0.000 0.000 0.011 0.000 0.000

0.000 0.000 0.005 0.000 0.011 0.005 0.005 0.000 0.061 0.058

0.064 0.085

>Non-Methylation code ID 21362

0.000 0.000 0.000 0.005 0.000 0.015 0.005 0.000 0.000 0.010

0.000 0.005 0.000 0.000 0.005 0.000 0.005 0.010 0.005 0.000

0.062 0.060 0.070 0.075 0.011 0.000 0.000 0.000 0.000 0.045

0.000 0.000 0.000 0.023 0.011 0.011 0.000 0.000 0.000 0.023

0.011 0.000 0.000 0.000 0.046 0.059 0.043 0.049 0.009 0.004

0.000 0.000 0.004 0.009 0.004 0.000 0.000 0.004 0.000 0.000

0.000 0.000 0.009 0.000 0.004 0.000 0.004 0.000 0.080 0.056

0.066 0.079

>Non-Methylation code ID 21362

0.008 0.000 0.000 0.000 0.008 0.000 0.008 0.000 0.000 0.023

0.000 0.000 0.015 0.008 0.000 0.008 0.008 0.008 0.000 0.008

0.070 0.060 0.045 0.059 0.008 0.000 0.000 0.000 0.000 0.000

0.008 0.000 0.000 0.000 0.000 0.008 0.016 0.000 0.008 0.032

0.016 0.000 0.000 0.000 0.059 0.041 0.060 0.076 0.000 0.000

0.000 0.000 0.000 0.000 0.006 0.000 0.006 0.012 0.000 0.000

0.023 0.000 0.012 0.000 0.012 0.000 0.000 0.000 0.055 0.070

0.075 0.065

>Non-Methylation code ID 21362

0.000 0.000 0.000 0.000 0.007 0.007 0.007 0.000 0.000 0.022

0.000 0.000 0.022 0.007 0.000 0.014 0.007 0.000 0.000 0.000

0.061 0.064 0.042 0.072 0.006 0.000 0.000 0.000 0.011 0.000

0.006 0.000 0.000 0.006 0.000 0.006 0.022 0.000 0.000 0.000

0.006 0.000 0.006 0.006 0.070 0.072 0.058 0.060 0.000 0.000

0.000 0.000 0.006 0.006 0.006 0.006 0.000 0.000 0.000 0.000

0.032 0.000 0.000 0.013 0.006 0.000 0.000 0.000 0.044 0.070

0.076 0.066

>Non-Methylation code ID 21362

0.008 0.000 0.000 0.000 0.008 0.000 0.000 0.000 0.000 0.024

0.000 0.000 0.024 0.008 0.000 0.008 0.008 0.008 0.000 0.008

0.065 0.056 0.048 0.058 0.009 0.000 0.000 0.000 0.000 0.000

0.009 0.000 0.000 0.009 0.000 0.009 0.027 0.000 0.000 0.036

0.009 0.000 0.000 0.000 0.056 0.045 0.057 0.068 0.000 0.005

0.000 0.000 0.000 0.000 0.005 0.000 0.005 0.016 0.000 0.000

0.021 0.000 0.005 0.000 0.005 0.000 0.000 0.000 0.057 0.080

0.072 0.063

>Non-Methylation code ID 21362

0.004 0.004 0.000 0.000 0.000 0.000 0.000 0.000 0.004 0.008

0.000 0.000 0.008 0.000 0.015 0.004 0.000 0.004 0.000 0.000

0.065 0.062 0.066 0.090 0.017 0.000 0.008 0.000 0.000 0.008

0.000 0.000 0.000 0.008 0.000 0.000 0.033 0.000 0.000 0.017

0.000 0.008 0.000 0.008 0.058 0.059 0.050 0.057 0.007 0.000

0.000 0.000 0.000 0.007 0.000 0.000 0.000 0.013 0.000 0.000

0.013 0.013 0.007 0.007 0.007 0.000 0.000 0.000 0.064 0.070

0.049 0.076

>Non-Methylation code ID 21362

0.012 0.000 0.000 0.000 0.000 0.012 0.000 0.000 0.012 0.000

0.000 0.012 0.000 0.006 0.006 0.012 0.000 0.000 0.006 0.000

0.077 0.083 0.041 0.053 0.012 0.000 0.000 0.000 0.000 0.012

0.000 0.000 0.006 0.006 0.006 0.000 0.012 0.006 0.006 0.006

0.006 0.000 0.000 0.000 0.057 0.057 0.065 0.080 0.004 0.004

0.000 0.008 0.004 0.000 0.000 0.000 0.004 0.008 0.000 0.000

0.004 0.000 0.012 0.000 0.000 0.000 0.000 0.000 0.088 0.048

0.092 0.057

>Non-Methylation code ID 21362

0.000 0.000 0.008 0.000 0.000 0.024 0.000 0.000 0.008 0.000

0.008 0.000 0.000 0.000 0.000 0.016 0.008 0.016 0.008 0.000

0.064 0.054 0.064 0.057 0.015 0.000 0.000 0.007 0.000 0.015

0.000 0.000 0.000 0.007 0.000 0.000 0.000 0.007 0.007 0.015

0.007 0.007 0.007 0.000 0.071 0.047 0.050 0.071 0.000 0.000

0.000 0.005 0.000 0.011 0.005 0.000 0.000 0.011 0.000 0.005

0.000 0.005 0.011 0.005 0.000 0.000 0.005 0.000 0.066 0.073

0.068 0.063

>Non-Methylation code ID 21362

0.005 0.005 0.005 0.000 0.005 0.009 0.005 0.000 0.000 0.009

0.000 0.000 0.005 0.000 0.005 0.009 0.000 0.000 0.000 0.000

0.071 0.068 0.063 0.071 0.014 0.000 0.007 0.007 0.000 0.007

0.000 0.014 0.000 0.000 0.000 0.000 0.007 0.014 0.000 0.014

0.000 0.000 0.000 0.007 0.058 0.067 0.049 0.064 0.000 0.000

0.000 0.000 0.005 0.000 0.000 0.000 0.005 0.005 0.000 0.000

0.010 0.005 0.014 0.005 0.010 0.000 0.000 0.000 0.060 0.071

0.064 0.081

>Non-Methylation code ID 21362

0.012 0.000 0.000 0.006 0.000 0.006 0.000 0.000 0.018 0.000

0.000 0.012 0.000 0.006 0.000 0.012 0.000 0.000 0.006 0.000

0.072 0.061 0.059 0.065 0.005 0.000 0.000 0.000 0.000 0.005

0.000 0.000 0.011 0.005 0.005 0.000 0.011 0.005 0.011 0.005

0.005 0.000 0.000 0.000 0.053 0.062 0.078 0.073 0.000 0.004

0.000 0.012 0.004 0.000 0.000 0.000 0.004 0.008 0.000 0.000

0.004 0.000 0.012 0.000 0.000 0.000 0.000 0.000 0.088 0.048

0.084 0.063

>Non-Methylation code ID 21362

0.005 0.005 0.000 0.000 0.005 0.027 0.005 0.000 0.000 0.005

0.000 0.000 0.005 0.000 0.005 0.000 0.005 0.000 0.000 0.000

0.068 0.065 0.053 0.078 0.018 0.000 0.009 0.000 0.000 0.037

0.000 0.009 0.000 0.009 0.000 0.000 0.009 0.009 0.000 0.009

0.000 0.000 0.000 0.009 0.052 0.065 0.043 0.053 0.000 0.004

0.004 0.004 0.004 0.004 0.000 0.000 0.000 0.004 0.000 0.000

0.004 0.000 0.013 0.000 0.008 0.000 0.000 0.000 0.081 0.089

0.039 0.074

>Non-Methylation code ID 21362

0.000 0.004 0.004 0.007 0.004 0.007 0.000 0.000 0.000 0.004

0.000 0.000 0.004 0.000 0.011 0.000 0.000 0.000 0.004 0.000

0.079 0.087 0.044 0.076 0.006 0.006 0.000 0.000 0.006 0.036

0.000 0.000 0.000 0.000 0.000 0.000 0.006 0.000 0.006 0.006

0.006 0.000 0.000 0.000 0.066 0.071 0.055 0.065 0.026 0.000

0.000 0.000 0.000 0.065 0.000 0.000 0.000 0.013 0.000 0.000

0.013 0.013 0.000 0.013 0.000 0.000 0.000 0.000 0.041 0.049

0.051 0.048

>Non-Methylation code ID 21362

0.004 0.000 0.000 0.000 0.004 0.004 0.004 0.000 0.000 0.004

0.000 0.000 0.009 0.000 0.013 0.004 0.000 0.000 0.004 0.000

0.082 0.056 0.075 0.069 0.006 0.000 0.006 0.006 0.000 0.011

0.000 0.000 0.000 0.011 0.000 0.000 0.006 0.000 0.006 0.006

0.006 0.011 0.000 0.000 0.059 0.068 0.055 0.077 0.013 0.006

0.000 0.006 0.000 0.000 0.000 0.000 0.006 0.006 0.006 0.000

0.013 0.000 0.006 0.006 0.000 0.000 0.000 0.000 0.065 0.057

0.065 0.077

>Non-Methylation code ID 21362

0.000 0.000 0.016 0.000 0.008 0.016 0.000 0.008 0.000 0.008

0.000 0.000 0.000 0.008 0.000 0.016 0.016 0.008 0.000 0.000

0.042 0.068 0.068 0.053 0.016 0.008 0.000 0.000 0.000 0.000

0.000 0.000 0.008 0.008 0.000 0.000 0.000 0.016 0.000 0.024

0.016 0.008 0.000 0.000 0.050 0.054 0.056 0.072 0.000 0.000

0.000 0.000 0.000 0.000 0.010 0.000 0.005 0.000 0.000 0.000

0.010 0.000 0.015 0.005 0.000 0.005 0.005 0.005 0.051 0.078

0.080 0.065

>Non-Methylation code ID 21362

0.007 0.000 0.000 0.000 0.000 0.014 0.021 0.000 0.000 0.000

0.000 0.000 0.007 0.000 0.000 0.007 0.000 0.007 0.007 0.007

0.040 0.063 0.067 0.084 0.000 0.006 0.000 0.006 0.000 0.011

0.000 0.006 0.000 0.006 0.000 0.000 0.000 0.000 0.006 0.006

0.017 0.006 0.006 0.000 0.074 0.069 0.062 0.055 0.008 0.000

0.000 0.000 0.000 0.024 0.000 0.000 0.000 0.008 0.000 0.000

0.000 0.008 0.008 0.032 0.000 0.008 0.000 0.000 0.047 0.066

0.056 0.068

>Non-Methylation code ID 21362

0.005 0.000 0.005 0.000 0.005 0.010 0.000 0.000 0.000 0.005

0.005 0.000 0.000 0.005 0.010 0.010 0.000 0.000 0.000 0.000

0.062 0.054 0.081 0.076 0.014 0.009 0.000 0.005 0.005 0.009

0.000 0.005 0.009 0.000 0.000 0.000 0.000 0.000 0.005 0.000

0.000 0.000 0.000 0.000 0.082 0.059 0.077 0.057 0.010 0.000

0.000 0.000 0.000 0.010 0.000 0.000 0.010 0.005 0.000 0.000

0.005 0.000 0.010 0.000 0.000 0.005 0.000 0.000 0.051 0.089

0.073 0.066

>Non-Methylation code ID 21362

0.006 0.000 0.006 0.006 0.000 0.006 0.000 0.000 0.000 0.017

0.000 0.000 0.006 0.000 0.006 0.006 0.006 0.011 0.000 0.000

0.058 0.072 0.062 0.069 0.012 0.006 0.000 0.000 0.000 0.000

0.000 0.000 0.006 0.012 0.006 0.000 0.012 0.000 0.006 0.012

0.000 0.000 0.000 0.000 0.061 0.058 0.061 0.082 0.005 0.000

0.000 0.000 0.005 0.005 0.005 0.000 0.000 0.005 0.000 0.000

0.015 0.000 0.010 0.005 0.000 0.000 0.000 0.000 0.075 0.061

0.077 0.064

>Non-Methylation code ID 21362

0.000 0.000 0.000 0.000 0.005 0.005 0.011 0.000 0.000 0.005

0.000 0.005 0.016 0.000 0.005 0.011 0.005 0.000 0.000 0.000

0.064 0.078 0.060 0.061 0.000 0.000 0.000 0.000 0.007 0.007

0.000 0.007 0.000 0.021 0.000 0.000 0.014 0.000 0.007 0.007

0.014 0.007 0.000 0.000 0.071 0.057 0.052 0.064 0.007 0.000

0.000 0.000 0.014 0.000 0.000 0.000 0.000 0.007 0.000 0.000

0.020 0.000 0.007 0.020 0.000 0.000 0.000 0.000 0.068 0.061

0.072 0.058

>Non-Methylation code ID 21362

0.022 0.000 0.000 0.000 0.000 0.000 0.000 0.000 0.000 0.055

0.000 0.000 0.011 0.011 0.000 0.022 0.022 0.000 0.000 0.000

0.053 0.043 0.050 0.045 0.000 0.000 0.000 0.000 0.000 0.000

0.000 0.000 0.000 0.011 0.000 0.000 0.011 0.000 0.011 0.033

0.000 0.005 0.000 0.000 0.048 0.059 0.078 0.077 0.006 0.000

0.000 0.000 0.000 0.006 0.012 0.000 0.000 0.000 0.000 0.000

0.018 0.006 0.006 0.012 0.000 0.006 0.000 0.000 0.046 0.075

0.073 0.067

>Non-Methylation code ID 21362

0.010 0.000 0.010 0.010 0.000 0.030 0.000 0.000 0.000 0.000

0.010 0.000 0.000 0.000 0.000 0.020 0.010 0.020 0.000 0.000

0.063 0.051 0.050 0.049 0.014 0.000 0.000 0.007 0.000 0.007

0.000 0.000 0.000 0.014 0.000 0.000 0.000 0.007 0.007 0.014

0.007 0.007 0.007 0.000 0.066 0.048 0.065 0.067 0.000 0.000

0.000 0.000 0.000 0.016 0.005 0.000 0.000 0.010 0.000 0.005

0.000 0.005 0.010 0.005 0.000 0.000 0.005 0.000 0.063 0.070

0.076 0.062

>Non-Methylation code ID 21362

0.000 0.000 0.000 0.000 0.012 0.000 0.000 0.000 0.000 0.006

0.000 0.012 0.018 0.000 0.006 0.018 0.000 0.000 0.000 0.000

0.061 0.064 0.072 0.064 0.000 0.000 0.000 0.000 0.006 0.006

0.012 0.006 0.000 0.006 0.000 0.006 0.018 0.000 0.000 0.012

0.006 0.000 0.000 0.000 0.059 0.078 0.058 0.061 0.000 0.000

0.000 0.000 0.011 0.000 0.000 0.011 0.000 0.032 0.000 0.000

0.021 0.000 0.000 0.011 0.021 0.011 0.000 0.000 0.065 0.059

0.036 0.057

>Non-Methylation code ID 21362

0.014 0.000 0.007 0.000 0.000 0.007 0.000 0.000 0.007 0.014

0.000 0.000 0.007 0.000 0.000 0.007 0.000 0.021 0.007 0.000

0.063 0.057 0.064 0.060 0.000 0.004 0.000 0.000 0.000 0.008

0.004 0.000 0.000 0.004 0.000 0.000 0.004 0.000 0.011 0.008

0.004 0.000 0.004 0.000 0.087 0.057 0.059 0.081 0.006 0.006

0.000 0.006 0.000 0.023 0.000 0.000 0.000 0.006 0.000 0.000

0.006 0.000 0.006 0.000 0.006 0.000 0.000 0.000 0.064 0.070

0.075 0.060

>Non-Methylation code ID 21362

0.005 0.005 0.009 0.000 0.005 0.005 0.005 0.000 0.000 0.009

0.000 0.000 0.000 0.000 0.005 0.014 0.000 0.000 0.000 0.000

0.062 0.078 0.066 0.067 0.023 0.000 0.008 0.008 0.000 0.000

0.000 0.023 0.000 0.000 0.000 0.000 0.008 0.008 0.000 0.016

0.000 0.000 0.000 0.008 0.063 0.072 0.050 0.046 0.000 0.000

0.000 0.000 0.000 0.000 0.000 0.000 0.006 0.019 0.000 0.000

0.006 0.006 0.013 0.006 0.013 0.000 0.000 0.000 0.064 0.082

0.050 0.068

>Non-Methylation code ID 21362

0.005 0.000 0.000 0.010 0.005 0.000 0.000 0.000 0.000 0.010

0.000 0.000 0.014 0.000 0.010 0.010 0.000 0.000 0.000 0.000

0.067 0.047 0.076 0.081 0.000 0.000 0.000 0.004 0.004 0.004

0.004 0.000 0.009 0.004 0.000 0.000 0.013 0.000 0.004 0.004

0.000 0.000 0.004 0.000 0.062 0.075 0.061 0.079 0.006 0.000

0.000 0.000 0.006 0.006 0.000 0.006 0.011 0.006 0.000 0.000

0.011 0.000 0.011 0.006 0.000 0.000 0.000 0.000 0.070 0.071

0.059 0.068

>Non-Methylation code ID 21362

0.000 0.000 0.000 0.000 0.000 0.000 0.000 0.000 0.000 0.011

0.000 0.000 0.011 0.000 0.011 0.029 0.000 0.011 0.000 0.000

0.048 0.064 0.076 0.070 0.006 0.000 0.000 0.000 0.000 0.006

0.011 0.000 0.000 0.000 0.000 0.000 0.017 0.006 0.006 0.017

0.000 0.000 0.006 0.000 0.056 0.066 0.065 0.074 0.024 0.000

0.000 0.000 0.000 0.012 0.000 0.000 0.000 0.059 0.000 0.000

0.012 0.000 0.000 0.012 0.024 0.000 0.000 0.000 0.054 0.040

0.047 0.050

>Non-Methylation code ID 21362

0.006 0.006 0.000 0.000 0.006 0.022 0.006 0.000 0.000 0.006

0.000 0.000 0.006 0.000 0.006 0.006 0.006 0.000 0.000 0.000

0.077 0.059 0.050 0.075 0.022 0.000 0.000 0.000 0.000 0.044

0.000 0.011 0.000 0.011 0.000 0.000 0.011 0.011 0.000 0.011

0.000 0.000 0.000 0.011 0.046 0.058 0.042 0.056 0.000 0.004

0.004 0.008 0.004 0.004 0.000 0.000 0.000 0.004 0.000 0.000

0.004 0.000 0.013 0.000 0.004 0.000 0.000 0.000 0.082 0.094

0.042 0.065

>Non-Methylation code ID 21362

0.000 0.000 0.000 0.005 0.000 0.000 0.000 0.000 0.005 0.011

0.005 0.000 0.005 0.000 0.011 0.016 0.000 0.000 0.005 0.000

0.062 0.059 0.072 0.077 0.000 0.005 0.005 0.000 0.005 0.009

0.005 0.000 0.000 0.000 0.000 0.000 0.009 0.005 0.005 0.009

0.000 0.000 0.000 0.000 0.065 0.060 0.076 0.076 0.014 0.000

0.007 0.007 0.000 0.000 0.000 0.000 0.000 0.021 0.000 0.000

0.000 0.000 0.007 0.000 0.007 0.021 0.000 0.000 0.065 0.056

0.067 0.063

>Non-Methylation code ID 21362

0.004 0.004 0.000 0.000 0.004 0.004 0.004 0.000 0.000 0.004

0.000 0.000 0.013 0.000 0.009 0.004 0.000 0.000 0.000 0.000

0.067 0.061 0.086 0.064 0.011 0.000 0.006 0.006 0.000 0.000

0.000 0.000 0.000 0.017 0.000 0.000 0.006 0.000 0.006 0.006

0.006 0.011 0.000 0.000 0.059 0.072 0.065 0.062 0.006 0.006

0.000 0.000 0.000 0.000 0.000 0.000 0.006 0.012 0.006 0.000

0.012 0.000 0.006 0.012 0.000 0.000 0.000 0.000 0.063 0.052

0.061 0.089

>Non-Methylation code ID 21362

0.020 0.000 0.000 0.000 0.000 0.010 0.000 0.000 0.000 0.051

0.000 0.000 0.020 0.000 0.000 0.010 0.020 0.000 0.000 0.000

0.054 0.044 0.050 0.053 0.000 0.000 0.000 0.000 0.000 0.000

0.000 0.000 0.000 0.013 0.000 0.000 0.013 0.000 0.013 0.033

0.000 0.013 0.000 0.000 0.053 0.068 0.070 0.058 0.006 0.000

0.000 0.000 0.000 0.006 0.012 0.000 0.000 0.000 0.000 0.000

0.018 0.006 0.006 0.012 0.000 0.000 0.006 0.000 0.049 0.066

0.068 0.078

>Non-Methylation code ID 21362

0.005 0.000 0.000 0.005 0.000 0.005 0.000 0.000 0.010 0.005

0.000 0.005 0.005 0.005 0.005 0.010 0.000 0.000 0.005 0.000

0.061 0.074 0.054 0.081 0.005 0.000 0.000 0.000 0.000 0.010

0.000 0.000 0.010 0.005 0.000 0.000 0.010 0.000 0.015 0.005

0.005 0.000 0.000 0.000 0.067 0.062 0.072 0.066 0.004 0.000

0.000 0.013 0.004 0.000 0.000 0.000 0.000 0.009 0.000 0.000

0.009 0.000 0.013 0.000 0.000 0.000 0.000 0.000 0.085 0.055

0.085 0.055

>Non-Methylation code ID 21362

0.005 0.005 0.010 0.000 0.005 0.005 0.000 0.000 0.000 0.005

0.000 0.000 0.000 0.005 0.005 0.020 0.000 0.000 0.000 0.000

0.068 0.080 0.067 0.052 0.029 0.000 0.000 0.007 0.000 0.000

0.000 0.014 0.000 0.000 0.000 0.000 0.007 0.007 0.007 0.014

0.000 0.007 0.000 0.000 0.073 0.063 0.065 0.040 0.000 0.000

0.000 0.006 0.000 0.000 0.000 0.000 0.006 0.017 0.000 0.000

0.006 0.006 0.011 0.011 0.000 0.000 0.000 0.000 0.074 0.079

0.041 0.078

>Non-Methylation code ID 21362

0.006 0.000 0.000 0.000 0.000 0.000 0.018 0.000 0.006 0.000

0.000 0.000 0.012 0.000 0.000 0.006 0.000 0.012 0.006 0.006

0.052 0.070 0.061 0.079 0.000 0.008 0.000 0.000 0.008 0.008

0.000 0.008 0.000 0.016 0.000 0.000 0.000 0.000 0.008 0.016

0.023 0.008 0.000 0.000 0.059 0.065 0.053 0.055 0.014 0.007

0.000 0.000 0.000 0.007 0.000 0.000 0.000 0.007 0.000 0.000

0.000 0.014 0.007 0.021 0.000 0.007 0.000 0.000 0.056 0.073

0.061 0.058

>Non-Methylation code ID 21362

0.006 0.000 0.000 0.000 0.000 0.013 0.006 0.013 0.000 0.006

0.000 0.000 0.000 0.000 0.013 0.019 0.000 0.000 0.000 0.000

0.069 0.079 0.060 0.050 0.012 0.000 0.000 0.000 0.000 0.018

0.000 0.000 0.006 0.000 0.000 0.000 0.006 0.000 0.006 0.006

0.006 0.006 0.000 0.006 0.069 0.073 0.060 0.060 0.006 0.000

0.006 0.000 0.000 0.006 0.000 0.000 0.006 0.006 0.000 0.000

0.000 0.011 0.011 0.000 0.006 0.011 0.000 0.000 0.081 0.045

0.077 0.063

>Non-Methylation code ID 21362

0.000 0.000 0.007 0.007 0.007 0.000 0.000 0.000 0.000 0.007

0.000 0.000 0.013 0.007 0.000 0.000 0.020 0.000 0.007 0.000

0.054 0.071 0.077 0.059 0.000 0.005 0.000 0.005 0.005 0.005

0.005 0.000 0.000 0.000 0.000 0.000 0.005 0.005 0.010 0.014

0.005 0.000 0.000 0.000 0.068 0.056 0.073 0.075 0.014 0.000

0.007 0.000 0.000 0.007 0.000 0.000 0.000 0.007 0.000 0.014

0.000 0.000 0.007 0.007 0.000 0.014 0.000 0.000 0.076 0.061

0.056 0.063

>Non-Methylation code ID 21368

0.014 0.000 0.007 0.000 0.000 0.007 0.007 0.000 0.000 0.007

0.000 0.007 0.000 0.000 0.007 0.007 0.014 0.014 0.000 0.000

0.062 0.060 0.066 0.055 0.000 0.000 0.000 0.008 0.008 0.008

0.008 0.000 0.000 0.008 0.000 0.000 0.016 0.016 0.000 0.000

0.023 0.000 0.000 0.000 0.057 0.077 0.062 0.044 0.000 0.006

0.006 0.000 0.006 0.000 0.006 0.000 0.000 0.000 0.000 0.000

0.006 0.006 0.012 0.018 0.006 0.000 0.000 0.000 0.071 0.060

0.066 0.065

>Non-Methylation code ID 21368

0.000 0.000 0.000 0.012 0.000 0.012 0.000 0.000 0.000 0.006

0.000 0.000 0.006 0.000 0.006 0.006 0.006 0.006 0.006 0.000

0.062 0.077 0.067 0.060 0.000 0.000 0.000 0.016 0.000 0.016

0.000 0.000 0.000 0.000 0.000 0.005 0.005 0.000 0.011 0.011

0.000 0.000 0.000 0.000 0.085 0.068 0.058 0.056 0.000 0.000

0.000 0.006 0.000 0.006 0.000 0.000 0.006 0.006 0.000 0.006

0.006 0.000 0.006 0.006 0.006 0.000 0.006 0.000 0.076 0.072

0.053 0.073

>Non-Methylation code ID 21368

0.000 0.004 0.000 0.000 0.000 0.009 0.000 0.000 0.000 0.009

0.000 0.000 0.009 0.000 0.013 0.000 0.000 0.004 0.004 0.000

0.073 0.075 0.054 0.079 0.017 0.006 0.000 0.006 0.000 0.011

0.006 0.000 0.000 0.000 0.000 0.000 0.000 0.000 0.006 0.017

0.000 0.000 0.000 0.000 0.052 0.071 0.074 0.070 0.013 0.006

0.000 0.000 0.000 0.006 0.000 0.000 0.000 0.006 0.000 0.000

0.019 0.000 0.006 0.006 0.006 0.000 0.000 0.000 0.053 0.069

0.069 0.072

>Non-Methylation code ID 21368

0.008 0.000 0.000 0.008 0.000 0.024 0.000 0.000 0.000 0.000

0.000 0.000 0.000 0.000 0.016 0.000 0.016 0.008 0.000 0.000

0.045 0.078 0.068 0.063 0.000 0.000 0.000 0.006 0.000 0.017

0.000 0.000 0.000 0.006 0.000 0.000 0.006 0.006 0.006 0.006

0.000 0.000 0.017 0.000 0.068 0.081 0.065 0.051 0.004 0.009

0.004 0.004 0.000 0.013 0.004 0.000 0.000 0.000 0.000 0.000

0.000 0.000 0.009 0.004 0.000 0.000 0.000 0.000 0.080 0.067

0.067 0.069

>Non-Methylation code ID 21368

0.004 0.008 0.004 0.004 0.000 0.016 0.004 0.000 0.000 0.000

0.000 0.000 0.000 0.000 0.008 0.004 0.000 0.000 0.000 0.000

0.076 0.074 0.069 0.062 0.008 0.000 0.000 0.008 0.000 0.025

0.000 0.000 0.000 0.000 0.000 0.000 0.000 0.000 0.017 0.000

0.017 0.008 0.000 0.000 0.042 0.075 0.072 0.061 0.000 0.000

0.000 0.007 0.000 0.022 0.000 0.000 0.000 0.007 0.000 0.000

0.007 0.007 0.000 0.007 0.000 0.000 0.022 0.000 0.066 0.082

0.068 0.038

>Non-Methylation code ID 21368

0.000 0.005 0.005 0.005 0.000 0.016 0.000 0.000 0.000 0.000

0.000 0.000 0.010 0.005 0.010 0.005 0.000 0.005 0.000 0.000

0.059 0.068 0.069 0.069 0.009 0.000 0.009 0.009 0.004 0.000

0.000 0.000 0.000 0.000 0.004 0.000 0.004 0.004 0.013 0.000

0.000 0.000 0.000 0.000 0.055 0.074 0.069 0.077 0.000 0.005

0.000 0.000 0.000 0.009 0.000 0.000 0.000 0.009 0.000 0.000

0.005 0.000 0.014 0.005 0.009 0.000 0.000 0.000 0.047 0.070

0.085 0.074

>Non-Methylation code ID 21368

0.004 0.000 0.000 0.000 0.009 0.004 0.000 0.004 0.000 0.004

0.000 0.004 0.013 0.000 0.009 0.004 0.000 0.000 0.000 0.000

0.072 0.078 0.069 0.057 0.009 0.000 0.000 0.000 0.000 0.009

0.000 0.009 0.000 0.009 0.000 0.000 0.026 0.000 0.009 0.018

0.000 0.018 0.000 0.000 0.052 0.064 0.046 0.066 0.000 0.000

0.000 0.007 0.007 0.014 0.007 0.000 0.000 0.007 0.000 0.000

0.022 0.007 0.000 0.007 0.000 0.000 0.000 0.000 0.063 0.060

0.058 0.072

>Non-Methylation code ID 21368

0.000 0.000 0.000 0.006 0.006 0.012 0.000 0.006 0.006 0.012

0.000 0.000 0.006 0.000 0.006 0.012 0.000 0.000 0.000 0.006

0.065 0.056 0.062 0.075 0.006 0.000 0.000 0.000 0.006 0.012

0.000 0.000 0.000 0.000 0.000 0.000 0.006 0.000 0.017 0.012

0.006 0.006 0.000 0.006 0.037 0.061 0.080 0.081 0.010 0.000

0.000 0.010 0.000 0.030 0.010 0.000 0.000 0.030 0.000 0.000

0.000 0.010 0.000 0.020 0.000 0.000 0.000 0.000 0.052 0.053

0.063 0.046

>Non-Methylation code ID 21368

0.005 0.000 0.000 0.015 0.005 0.005 0.000 0.005 0.000 0.005

0.000 0.005 0.000 0.000 0.010 0.000 0.000 0.010 0.000 0.000

0.078 0.056 0.092 0.044 0.000 0.000 0.000 0.004 0.009 0.009

0.000 0.004 0.004 0.009 0.000 0.000 0.004 0.000 0.009 0.000

0.000 0.000 0.000 0.000 0.065 0.074 0.073 0.068 0.000 0.004

0.004 0.004 0.004 0.009 0.000 0.000 0.000 0.000 0.000 0.000

0.004 0.000 0.009 0.009 0.000 0.000 0.004 0.000 0.078 0.056

0.073 0.075

>Non-Methylation code ID 21368

0.020 0.000 0.000 0.000 0.000 0.013 0.000 0.000 0.000 0.000

0.000 0.000 0.027 0.000 0.020 0.007 0.000 0.000 0.000 0.000

0.051 0.042 0.084 0.069 0.025 0.000 0.000 0.000 0.000 0.017

0.008 0.000 0.000 0.008 0.000 0.000 0.051 0.000 0.000 0.000

0.000 0.000 0.000 0.000 0.054 0.041 0.067 0.061 0.011 0.000

0.000 0.000 0.000 0.000 0.000 0.000 0.000 0.006 0.000 0.000

0.011 0.006 0.022 0.006 0.006 0.000 0.000 0.000 0.064 0.059

0.076 0.068

>Non-Methylation code ID 21368

0.004 0.004 0.000 0.004 0.004 0.004 0.000 0.000 0.000 0.004

0.000 0.000 0.000 0.000 0.008 0.008 0.004 0.000 0.004 0.004

0.071 0.061 0.074 0.073 0.007 0.000 0.000 0.000 0.000 0.020

0.000 0.000 0.007 0.000 0.000 0.000 0.007 0.000 0.013 0.013

0.007 0.013 0.000 0.000 0.077 0.044 0.076 0.050 0.000 0.000

0.000 0.007 0.007 0.014 0.000 0.000 0.000 0.021 0.000 0.000

0.007 0.007 0.007 0.007 0.000 0.007 0.000 0.000 0.074 0.059

0.054 0.062

>Non-Methylation code ID 21368

0.016 0.000 0.000 0.005 0.005 0.005 0.000 0.011 0.000 0.000

0.000 0.000 0.011 0.000 0.005 0.005 0.000 0.000 0.005 0.000

0.071 0.061 0.069 0.064 0.014 0.000 0.000 0.000 0.000 0.007

0.000 0.000 0.000 0.007 0.000 0.000 0.021 0.007 0.007 0.028

0.000 0.000 0.000 0.000 0.077 0.048 0.062 0.054 0.000 0.004

0.000 0.000 0.000 0.000 0.009 0.000 0.000 0.004 0.000 0.000

0.013 0.000 0.013 0.000 0.000 0.009 0.000 0.000 0.093 0.044

0.083 0.060

>Non-Methylation code ID 21368

0.000 0.004 0.004 0.000 0.000 0.008 0.004 0.000 0.000 0.008

0.000 0.000 0.008 0.000 0.012 0.000 0.000 0.004 0.000 0.000

0.083 0.041 0.086 0.070 0.016 0.000 0.000 0.005 0.000 0.010

0.000 0.005 0.000 0.000 0.000 0.000 0.005 0.000 0.005 0.005

0.005 0.000 0.010 0.000 0.078 0.063 0.060 0.065 0.010 0.000

0.000 0.000 0.000 0.029 0.000 0.000 0.000 0.010 0.000 0.000

0.019 0.019 0.000 0.019 0.000 0.010 0.000 0.000 0.058 0.049

0.060 0.051

>Non-Methylation code ID 21368

0.014 0.000 0.000 0.000 0.000 0.009 0.000 0.005 0.000 0.000

0.000 0.005 0.000 0.000 0.014 0.000 0.009 0.000 0.005 0.000

0.068 0.097 0.059 0.049 0.005 0.000 0.000 0.011 0.000 0.011

0.005 0.000 0.000 0.000 0.000 0.000 0.016 0.000 0.005 0.000

0.005 0.005 0.000 0.000 0.063 0.059 0.075 0.072 0.006 0.000

0.006 0.000 0.000 0.006 0.012 0.000 0.006 0.006 0.000 0.000

0.000 0.000 0.006 0.018 0.006 0.000 0.000 0.000 0.056 0.061

0.070 0.074

>Non-Methylation code ID 21368

0.017 0.000 0.000 0.000 0.006 0.006 0.000 0.011 0.006 0.000

0.000 0.006 0.011 0.000 0.006 0.000 0.000 0.000 0.000 0.000

0.069 0.063 0.072 0.062 0.011 0.000 0.000 0.005 0.000 0.000

0.000 0.005 0.000 0.005 0.000 0.000 0.011 0.005 0.011 0.016

0.000 0.000 0.000 0.000 0.085 0.039 0.071 0.069 0.000 0.005

0.000 0.000 0.000 0.005 0.011 0.000 0.005 0.000 0.000 0.000

0.016 0.000 0.011 0.000 0.000 0.005 0.000 0.000 0.100 0.034

0.074 0.067

>Non-Methylation code ID 21368

0.000 0.005 0.000 0.000 0.000 0.005 0.011 0.000 0.005 0.000

0.000 0.000 0.016 0.000 0.011 0.000 0.000 0.005 0.000 0.000

0.100 0.034 0.074 0.067 0.017 0.000 0.000 0.000 0.006 0.006

0.000 0.011 0.006 0.000 0.000 0.006 0.011 0.000 0.006 0.000

0.000 0.000 0.000 0.000 0.069 0.063 0.072 0.062 0.011 0.000

0.000 0.006 0.000 0.000 0.000 0.006 0.000 0.006 0.000 0.000

0.011 0.000 0.011 0.017 0.000 0.000 0.000 0.000 0.086 0.037

0.075 0.068

>Non-Methylation code ID 21368

0.000 0.005 0.000 0.000 0.000 0.009 0.009 0.000 0.000 0.009

0.000 0.000 0.014 0.000 0.009 0.000 0.000 0.005 0.000 0.000

0.087 0.028 0.091 0.065 0.015 0.000 0.000 0.005 0.000 0.005

0.000 0.010 0.000 0.000 0.000 0.000 0.010 0.000 0.005 0.005

0.000 0.000 0.010 0.000 0.080 0.049 0.073 0.067 0.010 0.000

0.000 0.000 0.000 0.019 0.000 0.000 0.000 0.010 0.000 0.000

0.029 0.010 0.000 0.029 0.000 0.010 0.000 0.000 0.060 0.043

0.063 0.051

>Non-Methylation code ID 21368

0.016 0.000 0.000 0.005 0.000 0.010 0.000 0.005 0.000 0.000

0.000 0.000 0.005 0.000 0.005 0.005 0.005 0.000 0.010 0.000

0.078 0.063 0.060 0.065 0.007 0.000 0.000 0.000 0.000 0.022

0.000 0.000 0.000 0.007 0.000 0.000 0.015 0.015 0.007 0.015

0.000 0.007 0.000 0.000 0.063 0.046 0.068 0.063 0.000 0.004

0.004 0.000 0.000 0.008 0.004 0.000 0.000 0.008 0.000 0.000

0.004 0.000 0.013 0.000 0.000 0.004 0.000 0.000 0.083 0.044

0.080 0.075

>Non-Methylation code ID 21368

0.000 0.005 0.000 0.000 0.000 0.005 0.009 0.000 0.000 0.009

0.000 0.000 0.014 0.000 0.009 0.000 0.000 0.009 0.000 0.000

0.085 0.033 0.089 0.068 0.010 0.000 0.000 0.005 0.005 0.005

0.000 0.010 0.000 0.000 0.000 0.000 0.010 0.000 0.005 0.005

0.000 0.000 0.010 0.000 0.081 0.049 0.073 0.066 0.010 0.000

0.000 0.000 0.000 0.010 0.000 0.000 0.000 0.010 0.000 0.000

0.029 0.010 0.000 0.038 0.000 0.010 0.000 0.000 0.065 0.043

0.063 0.048

>Non-Methylation code ID 21368

0.006 0.000 0.000 0.000 0.012 0.012 0.000 0.000 0.000 0.012

0.000 0.000 0.012 0.006 0.000 0.000 0.006 0.000 0.012 0.000

0.057 0.074 0.070 0.053 0.000 0.000 0.000 0.000 0.010 0.014

0.000 0.000 0.000 0.000 0.000 0.005 0.005 0.005 0.010 0.010

0.000 0.005 0.000 0.000 0.083 0.059 0.080 0.050 0.000 0.000

0.007 0.000 0.000 0.007 0.000 0.000 0.000 0.007 0.000 0.000

0.014 0.000 0.007 0.014 0.007 0.014 0.000 0.000 0.070 0.087

0.053 0.046

>Non-Methylation code ID 21368

0.004 0.000 0.000 0.000 0.004 0.000 0.000 0.004 0.000 0.004

0.000 0.000 0.013 0.000 0.009 0.004 0.009 0.000 0.004 0.000

0.065 0.071 0.078 0.061 0.006 0.000 0.000 0.000 0.000 0.013

0.000 0.000 0.000 0.000 0.000 0.000 0.032 0.000 0.006 0.013

0.000 0.006 0.000 0.000 0.062 0.075 0.053 0.065 0.000 0.000

0.007 0.007 0.000 0.007 0.014 0.000 0.000 0.007 0.000 0.000

0.036 0.000 0.000 0.007 0.000 0.000 0.000 0.000 0.075 0.051

0.061 0.059

>Non-Methylation code ID 21368

0.000 0.009 0.005 0.000 0.000 0.014 0.000 0.000 0.000 0.000

0.000 0.000 0.005 0.005 0.009 0.005 0.000 0.009 0.000 0.000

0.059 0.078 0.053 0.083 0.011 0.000 0.005 0.011 0.011 0.000

0.000 0.000 0.000 0.000 0.000 0.000 0.005 0.005 0.005 0.000

0.005 0.005 0.000 0.000 0.062 0.068 0.076 0.061 0.000 0.005

0.000 0.000 0.000 0.005 0.000 0.000 0.000 0.014 0.000 0.000

0.009 0.000 0.014 0.005 0.005 0.000 0.000 0.000 0.042 0.070

0.087 0.079

>Non-Methylation code ID 21368

0.000 0.000 0.006 0.000 0.000 0.012 0.000 0.000 0.000 0.012

0.000 0.000 0.012 0.000 0.006 0.006 0.006 0.012 0.000 0.000

0.060 0.067 0.064 0.068 0.006 0.000 0.000 0.000 0.006 0.012

0.000 0.000 0.000 0.006 0.000 0.000 0.012 0.006 0.000 0.006

0.006 0.000 0.018 0.000 0.062 0.056 0.077 0.062 0.000 0.000

0.000 0.000 0.006 0.025 0.000 0.000 0.000 0.000 0.000 0.006

0.012 0.006 0.006 0.006 0.000 0.006 0.000 0.000 0.075 0.052

0.077 0.054

>Non-Methylation code ID 21368

0.000 0.004 0.000 0.000 0.000 0.013 0.000 0.000 0.000 0.009

0.000 0.000 0.004 0.000 0.013 0.004 0.009 0.000 0.000 0.000

0.044 0.066 0.088 0.079 0.000 0.006 0.011 0.006 0.000 0.017

0.000 0.000 0.000 0.000 0.000 0.000 0.011 0.006 0.006 0.006

0.000 0.006 0.000 0.000 0.053 0.074 0.069 0.065 0.010 0.000

0.005 0.010 0.005 0.000 0.000 0.000 0.000 0.000 0.005 0.000

0.005 0.005 0.015 0.000 0.000 0.000 0.000 0.000 0.053 0.074

0.072 0.073

>Non-Methylation code ID 21368

0.013 0.000 0.000 0.000 0.000 0.013 0.000 0.000 0.000 0.000

0.000 0.000 0.038 0.000 0.006 0.006 0.000 0.006 0.000 0.000

0.063 0.070 0.055 0.064 0.000 0.000 0.006 0.006 0.000 0.006

0.019 0.000 0.000 0.013 0.000 0.000 0.025 0.000 0.000 0.006

0.000 0.000 0.000 0.000 0.075 0.053 0.059 0.064 0.005 0.000

0.000 0.000 0.005 0.000 0.000 0.000 0.000 0.000 0.000 0.000

0.015 0.000 0.010 0.005 0.015 0.000 0.005 0.000 0.073 0.059

0.070 0.074

>Non-Methylation code ID 21368

0.000 0.000 0.000 0.000 0.005 0.010 0.000 0.005 0.000 0.010

0.000 0.000 0.005 0.000 0.015 0.010 0.000 0.000 0.000 0.005

0.076 0.051 0.066 0.075 0.006 0.000 0.006 0.000 0.006 0.019

0.000 0.000 0.000 0.000 0.000 0.000 0.006 0.000 0.006 0.013

0.006 0.006 0.000 0.006 0.062 0.061 0.061 0.068 0.019 0.000

0.000 0.009 0.000 0.009 0.009 0.000 0.000 0.028 0.000 0.000

0.000 0.009 0.000 0.019 0.009 0.000 0.000 0.000 0.043 0.055

0.073 0.050

>Non-Methylation code ID 21368

0.009 0.000 0.000 0.005 0.000 0.009 0.000 0.000 0.005 0.000

0.000 0.000 0.009 0.000 0.018 0.005 0.000 0.000 0.000 0.000

0.040 0.049 0.076 0.109 0.006 0.000 0.000 0.006 0.000 0.018

0.006 0.000 0.012 0.000 0.000 0.000 0.023 0.000 0.000 0.000

0.000 0.006 0.000 0.000 0.063 0.065 0.050 0.080 0.013 0.000

0.000 0.000 0.000 0.000 0.000 0.000 0.000 0.006 0.000 0.000

0.013 0.006 0.019 0.006 0.006 0.000 0.000 0.000 0.034 0.056

0.081 0.090

>Non-Methylation code ID 21368

0.005 0.005 0.000 0.000 0.010 0.000 0.000 0.000 0.005 0.010

0.000 0.000 0.005 0.010 0.005 0.005 0.000 0.000 0.000 0.000

0.065 0.089 0.064 0.053 0.005 0.000 0.000 0.000 0.010 0.005

0.000 0.000 0.000 0.000 0.000 0.000 0.010 0.000 0.015 0.020

0.000 0.000 0.000 0.000 0.054 0.068 0.073 0.072 0.029 0.000

0.000 0.010 0.010 0.010 0.000 0.000 0.000 0.029 0.000 0.000

0.010 0.010 0.000 0.000 0.000 0.010 0.000 0.000 0.046 0.068

0.055 0.050

>Non-Methylation code ID 21368

0.005 0.000 0.000 0.000 0.005 0.005 0.011 0.000 0.005 0.016

0.000 0.000 0.000 0.005 0.005 0.011 0.000 0.000 0.000 0.000

0.051 0.065 0.070 0.076 0.006 0.000 0.000 0.000 0.013 0.006

0.000 0.000 0.006 0.006 0.000 0.000 0.006 0.000 0.006 0.013

0.013 0.000 0.000 0.000 0.065 0.074 0.051 0.065 0.007 0.000

0.000 0.007 0.007 0.000 0.000 0.000 0.000 0.000 0.000 0.000

0.027 0.013 0.000 0.013 0.000 0.000 0.007 0.000 0.069 0.073

0.061 0.050

>Non-Methylation code ID 21368

0.005 0.000 0.000 0.005 0.005 0.000 0.010 0.000 0.000 0.021

0.000 0.000 0.000 0.005 0.005 0.010 0.000 0.000 0.000 0.000

0.047 0.068 0.076 0.074 0.000 0.000 0.000 0.000 0.018 0.006

0.000 0.000 0.006 0.006 0.000 0.000 0.006 0.000 0.006 0.012

0.012 0.000 0.000 0.000 0.062 0.082 0.058 0.058 0.006 0.000

0.000 0.000 0.012 0.006 0.000 0.000 0.000 0.000 0.000 0.000

0.017 0.012 0.006 0.012 0.000 0.000 0.000 0.000 0.066 0.067

0.075 0.056

>Non-Methylation code ID 21368

0.010 0.005 0.005 0.005 0.000 0.000 0.005 0.005 0.005 0.005

0.000 0.000 0.000 0.005 0.005 0.000 0.005 0.000 0.000 0.000

0.055 0.076 0.080 0.063 0.011 0.000 0.000 0.000 0.011 0.000

0.000 0.000 0.000 0.011 0.006 0.011 0.006 0.000 0.006 0.006

0.006 0.000 0.000 0.000 0.076 0.051 0.073 0.060 0.000 0.009

0.000 0.000 0.000 0.009 0.018 0.000 0.000 0.009 0.000 0.000

0.009 0.009 0.000 0.000 0.018 0.009 0.000 0.000 0.056 0.076

0.063 0.048

>Non-Methylation code ID 21368

0.016 0.010 0.005 0.000 0.000 0.005 0.000 0.005 0.005 0.000

0.000 0.000 0.000 0.005 0.005 0.000 0.005 0.000 0.000 0.000

0.054 0.079 0.077 0.061 0.018 0.000 0.000 0.000 0.006 0.000

0.000 0.000 0.000 0.012 0.000 0.006 0.012 0.000 0.006 0.006

0.006 0.000 0.000 0.000 0.070 0.055 0.068 0.068 0.000 0.006

0.000 0.000 0.000 0.006 0.012 0.000 0.000 0.006 0.000 0.000

0.006 0.006 0.006 0.000 0.006 0.006 0.006 0.000 0.058 0.080

0.068 0.062

>Non-Methylation code ID 21368

0.006 0.000 0.000 0.000 0.000 0.012 0.012 0.000 0.006 0.018

0.000 0.000 0.000 0.006 0.006 0.012 0.000 0.000 0.000 0.000

0.051 0.071 0.071 0.061 0.006 0.000 0.000 0.000 0.006 0.013

0.000 0.000 0.006 0.006 0.000 0.000 0.006 0.000 0.006 0.013

0.013 0.000 0.000 0.000 0.056 0.079 0.065 0.055 0.007 0.000

0.000 0.007 0.000 0.000 0.000 0.000 0.000 0.000 0.000 0.000

0.027 0.013 0.000 0.013 0.000 0.000 0.013 0.000 0.067 0.074

0.070 0.042

>Non-Methylation code ID 21523

0.015 0.000 0.000 0.000 0.000 0.000 0.005 0.000 0.000 0.000

0.005 0.000 0.010 0.000 0.010 0.005 0.010 0.000 0.005 0.000

0.068 0.074 0.054 0.072 0.009 0.000 0.000 0.000 0.000 0.004

0.004 0.000 0.000 0.004 0.000 0.000 0.013 0.000 0.013 0.000

0.004 0.000 0.000 0.000 0.078 0.060 0.058 0.084 0.010 0.000

0.005 0.000 0.000 0.005 0.010 0.000 0.000 0.010 0.000 0.000

0.005 0.000 0.010 0.000 0.005 0.000 0.000 0.000 0.077 0.079

0.037 0.078

>Non-Methylation code ID 21523

0.005 0.005 0.000 0.000 0.010 0.000 0.000 0.000 0.005 0.010

0.000 0.000 0.010 0.005 0.005 0.005 0.000 0.000 0.000 0.000

0.065 0.083 0.068 0.061 0.005 0.000 0.000 0.000 0.010 0.005

0.000 0.000 0.000 0.000 0.000 0.000 0.015 0.000 0.010 0.020

0.000 0.000 0.000 0.000 0.063 0.068 0.067 0.071 0.028 0.000

0.000 0.009 0.009 0.000 0.000 0.000 0.000 0.028 0.000 0.000

0.009 0.009 0.000 0.009 0.000 0.009 0.000 0.000 0.054 0.065

0.060 0.042

>Non-Methylation code ID 21523

0.013 0.000 0.000 0.000 0.000 0.013 0.000 0.000 0.000 0.013

0.006 0.000 0.006 0.000 0.013 0.000 0.006 0.006 0.000 0.000

0.074 0.042 0.079 0.064 0.012 0.000 0.006 0.000 0.000 0.006

0.012 0.000 0.000 0.012 0.000 0.000 0.000 0.000 0.006 0.000

0.000 0.000 0.006 0.006 0.069 0.056 0.066 0.075 0.007 0.013

0.000 0.000 0.000 0.007 0.007 0.000 0.000 0.007 0.007 0.007

0.000 0.000 0.000 0.000 0.020 0.000 0.007 0.000 0.071 0.070

0.053 0.061

>Non-Methylation code ID 21523

0.011 0.011 0.000 0.000 0.000 0.006 0.006 0.000 0.000 0.006

0.000 0.000 0.000 0.000 0.006 0.011 0.017 0.000 0.000 0.000

0.071 0.071 0.058 0.059 0.014 0.005 0.005 0.000 0.000 0.000

0.009 0.000 0.000 0.009 0.000 0.000 0.009 0.000 0.005 0.000

0.005 0.000 0.000 0.000 0.065 0.059 0.075 0.072 0.005 0.000

0.000 0.000 0.005 0.005 0.011 0.011 0.000 0.011 0.000 0.000

0.000 0.000 0.011 0.000 0.005 0.000 0.000 0.000 0.085 0.060

0.052 0.072

>Non-Methylation code ID 21523

0.007 0.014 0.007 0.000 0.000 0.000 0.007 0.000 0.000 0.007

0.000 0.007 0.000 0.000 0.000 0.014 0.028 0.000 0.000 0.000

0.076 0.060 0.039 0.068 0.009 0.005 0.000 0.000 0.000 0.000

0.005 0.000 0.000 0.005 0.005 0.000 0.014 0.000 0.009 0.000

0.009 0.000 0.000 0.000 0.067 0.066 0.083 0.056 0.008 0.000

0.000 0.000 0.008 0.008 0.032 0.008 0.000 0.008 0.000 0.000

0.000 0.008 0.000 0.000 0.000 0.000 0.000 0.000 0.069 0.081

0.033 0.071

>Non-Methylation code ID 21523

0.000 0.000 0.006 0.006 0.000 0.006 0.013 0.000 0.000 0.013

0.000 0.000 0.019 0.000 0.000 0.019 0.000 0.000 0.000 0.000

0.052 0.061 0.072 0.064 0.017 0.000 0.000 0.000 0.000 0.000

0.000 0.000 0.008 0.008 0.000 0.000 0.033 0.000 0.000 0.008

0.025 0.000 0.000 0.000 0.057 0.058 0.061 0.058 0.000 0.000

0.000 0.000 0.000 0.000 0.000 0.000 0.006 0.018 0.000 0.000

0.029 0.000 0.012 0.006 0.000 0.000 0.000 0.000 0.079 0.045

0.072 0.067

>Non-Methylation code ID 21523

0.008 0.008 0.000 0.000 0.000 0.000 0.000 0.000 0.000 0.008

0.000 0.000 0.032 0.000 0.000 0.024 0.024 0.000 0.000 0.000

0.063 0.052 0.069 0.044 0.016 0.000 0.000 0.000 0.000 0.000

0.000 0.000 0.000 0.005 0.000 0.000 0.032 0.000 0.011 0.005

0.000 0.000 0.000 0.000 0.063 0.057 0.074 0.070 0.000 0.000

0.006 0.000 0.000 0.000 0.013 0.000 0.000 0.013 0.000 0.000

0.019 0.013 0.006 0.006 0.000 0.000 0.000 0.000 0.056 0.059

0.073 0.068

>Non-Methylation code ID 21523

0.000 0.008 0.000 0.004 0.004 0.004 0.004 0.000 0.000 0.004

0.000 0.000 0.004 0.004 0.008 0.004 0.000 0.000 0.004 0.000

0.067 0.076 0.070 0.067 0.023 0.000 0.000 0.006 0.000 0.012

0.000 0.000 0.006 0.000 0.000 0.000 0.000 0.000 0.012 0.012

0.006 0.000 0.000 0.000 0.069 0.064 0.061 0.064 0.013 0.000

0.000 0.007 0.000 0.000 0.000 0.000 0.007 0.020 0.000 0.000

0.020 0.000 0.007 0.000 0.000 0.000 0.000 0.000 0.054 0.086

0.066 0.055

>Non-Methylation code ID 21523

0.000 0.000 0.006 0.000 0.000 0.006 0.013 0.000 0.000 0.013

0.000 0.000 0.019 0.013 0.000 0.013 0.000 0.000 0.000 0.000

0.056 0.062 0.070 0.061 0.008 0.008 0.000 0.000 0.000 0.000

0.000 0.000 0.000 0.008 0.000 0.000 0.033 0.000 0.000 0.025

0.025 0.000 0.000 0.000 0.067 0.053 0.062 0.045 0.014 0.000

0.000 0.000 0.000 0.000 0.000 0.000 0.000 0.014 0.000 0.000

0.042 0.000 0.007 0.007 0.000 0.000 0.000 0.000 0.069 0.058

0.078 0.044

>Non-Methylation code ID 21523

0.020 0.007 0.000 0.007 0.000 0.000 0.000 0.000 0.000 0.020

0.000 0.000 0.007 0.000 0.007 0.013 0.007 0.000 0.000 0.000

0.065 0.058 0.059 0.066 0.007 0.007 0.000 0.000 0.000 0.000

0.000 0.000 0.000 0.021 0.000 0.000 0.028 0.000 0.007 0.014

0.000 0.000 0.000 0.000 0.054 0.066 0.057 0.072 0.011 0.011

0.000 0.000 0.005 0.000 0.000 0.000 0.000 0.011 0.000 0.000

0.016 0.000 0.005 0.000 0.000 0.000 0.000 0.000 0.071 0.071

0.060 0.074

>Non-Methylation code ID 21523

0.010 0.010 0.000 0.000 0.005 0.000 0.005 0.000 0.000 0.010

0.000 0.000 0.014 0.000 0.005 0.000 0.000 0.000 0.000 0.000

0.070 0.072 0.059 0.075 0.020 0.007 0.000 0.007 0.000 0.000

0.000 0.000 0.000 0.020 0.000 0.000 0.007 0.000 0.007 0.013

0.007 0.000 0.000 0.000 0.065 0.058 0.059 0.066 0.010 0.010

0.000 0.000 0.000 0.000 0.000 0.000 0.000 0.029 0.000 0.000

0.038 0.000 0.000 0.019 0.000 0.000 0.000 0.000 0.057 0.063

0.058 0.051

>Non-Methylation code ID 21523

0.006 0.000 0.006 0.000 0.000 0.023 0.000 0.000 0.006 0.012

0.000 0.000 0.000 0.000 0.006 0.006 0.006 0.000 0.006 0.000

0.074 0.066 0.054 0.066 0.011 0.000 0.005 0.005 0.000 0.011

0.000 0.000 0.000 0.000 0.000 0.000 0.005 0.000 0.005 0.005

0.005 0.000 0.011 0.000 0.070 0.062 0.069 0.068 0.000 0.000

0.000 0.012 0.000 0.012 0.000 0.000 0.000 0.006 0.000 0.006

0.000 0.006 0.012 0.000 0.006 0.006 0.000 0.000 0.072 0.048

0.073 0.076

>Non-Methylation code ID 21523

0.029 0.000 0.000 0.000 0.000 0.057 0.000 0.000 0.000 0.000

0.000 0.000 0.000 0.014 0.000 0.014 0.043 0.014 0.000 0.000

0.031 0.053 0.040 0.037 0.006 0.000 0.000 0.006 0.000 0.006

0.000 0.000 0.000 0.006 0.000 0.000 0.017 0.000 0.011 0.006

0.000 0.000 0.006 0.000 0.050 0.069 0.082 0.069 0.004 0.013

0.004 0.000 0.000 0.004 0.009 0.000 0.000 0.000 0.000 0.000

0.000 0.004 0.013 0.000 0.000 0.000 0.000 0.000 0.080 0.070

0.077 0.054

>Non-Methylation code ID 21523

0.000 0.000 0.006 0.000 0.000 0.006 0.012 0.000 0.000 0.012

0.000 0.000 0.017 0.012 0.006 0.006 0.000 0.000 0.000 0.000

0.066 0.058 0.071 0.062 0.008 0.008 0.000 0.000 0.000 0.008

0.000 0.000 0.000 0.008 0.000 0.000 0.024 0.000 0.000 0.024

0.024 0.000 0.000 0.000 0.057 0.047 0.063 0.061 0.017 0.000

0.000 0.000 0.000 0.000 0.000 0.000 0.000 0.006 0.000 0.000

0.028 0.000 0.011 0.006 0.000 0.000 0.000 0.000 0.060 0.059

0.073 0.075

>Non-Methylation code ID 21523

0.000 0.000 0.000 0.007 0.000 0.007 0.015 0.000 0.000 0.015

0.007 0.000 0.015 0.000 0.000 0.022 0.000 0.000 0.000 0.000

0.053 0.060 0.069 0.062 0.014 0.000 0.000 0.007 0.000 0.000

0.000 0.000 0.007 0.007 0.000 0.000 0.020 0.000 0.000 0.007

0.014 0.000 0.007 0.000 0.062 0.067 0.064 0.059 0.000 0.000

0.000 0.000 0.000 0.006 0.000 0.000 0.006 0.019 0.000 0.006

0.025 0.000 0.006 0.006 0.000 0.000 0.000 0.000 0.085 0.035

0.080 0.058

>Non-Methylation code ID 21523

0.008 0.008 0.000 0.000 0.000 0.000 0.000 0.000 0.000 0.008

0.000 0.000 0.033 0.000 0.000 0.025 0.025 0.000 0.000 0.000

0.067 0.053 0.062 0.045 0.019 0.000 0.000 0.000 0.000 0.000

0.000 0.000 0.000 0.013 0.000 0.000 0.039 0.000 0.006 0.006

0.000 0.000 0.000 0.000 0.063 0.052 0.074 0.062 0.000 0.000

0.007 0.000 0.000 0.000 0.014 0.000 0.000 0.014 0.000 0.000

0.022 0.014 0.000 0.014 0.000 0.000 0.000 0.000 0.062 0.064

0.066 0.056

>Non-Methylation code ID 21523

0.004 0.008 0.000 0.000 0.004 0.000 0.004 0.000 0.000 0.008

0.000 0.000 0.012 0.000 0.004 0.004 0.000 0.000 0.004 0.000

0.070 0.073 0.064 0.073 0.028 0.007 0.000 0.000 0.000 0.007

0.000 0.000 0.000 0.021 0.000 0.000 0.000 0.000 0.007 0.014

0.007 0.000 0.000 0.000 0.056 0.069 0.069 0.050 0.020 0.010

0.000 0.000 0.000 0.000 0.000 0.000 0.000 0.031 0.000 0.000

0.041 0.000 0.000 0.010 0.000 0.000 0.000 0.000 0.060 0.066

0.053 0.042

>Non-Methylation code ID 21523

0.005 0.000 0.005 0.000 0.000 0.015 0.000 0.000 0.000 0.005

0.000 0.000 0.000 0.000 0.015 0.005 0.005 0.005 0.005 0.000

0.069 0.075 0.053 0.073 0.012 0.000 0.006 0.006 0.000 0.018

0.000 0.000 0.000 0.000 0.000 0.000 0.006 0.000 0.006 0.000

0.006 0.000 0.006 0.000 0.072 0.080 0.057 0.056 0.006 0.000

0.000 0.006 0.000 0.012 0.000 0.000 0.000 0.000 0.000 0.006

0.006 0.006 0.012 0.000 0.006 0.000 0.000 0.000 0.071 0.062

0.063 0.077

>Non-Methylation code ID 21523

0.000 0.000 0.007 0.007 0.000 0.007 0.015 0.000 0.000 0.007

0.007 0.000 0.015 0.000 0.000 0.022 0.000 0.000 0.000 0.000

0.056 0.058 0.065 0.066 0.013 0.000 0.000 0.007 0.000 0.000

0.000 0.000 0.007 0.007 0.000 0.000 0.020 0.000 0.000 0.000

0.020 0.000 0.007 0.000 0.059 0.066 0.069 0.060 0.000 0.000

0.000 0.000 0.000 0.006 0.000 0.000 0.006 0.024 0.000 0.006

0.018 0.000 0.006 0.006 0.000 0.000 0.000 0.000 0.090 0.032

0.090 0.049

>Non-Methylation code ID 21523

0.010 0.000 0.005 0.000 0.000 0.005 0.000 0.000 0.000 0.005

0.000 0.000 0.005 0.000 0.005 0.010 0.005 0.000 0.010 0.000

0.072 0.062 0.066 0.072 0.007 0.000 0.000 0.007 0.000 0.021

0.000 0.000 0.000 0.000 0.000 0.000 0.000 0.014 0.014 0.000

0.007 0.007 0.000 0.000 0.067 0.042 0.068 0.077 0.008 0.000

0.008 0.008 0.000 0.030 0.000 0.000 0.008 0.015 0.000 0.000

0.008 0.000 0.000 0.008 0.000 0.000 0.000 0.000 0.065 0.060

0.054 0.064

>Non-Methylation code ID 21523

0.000 0.000 0.000 0.010 0.000 0.015 0.000 0.000 0.000 0.000

0.000 0.000 0.005 0.000 0.020 0.000 0.005 0.000 0.000 0.000

0.068 0.075 0.051 0.083 0.010 0.000 0.000 0.010 0.000 0.010

0.000 0.000 0.000 0.000 0.000 0.005 0.000 0.000 0.016 0.005

0.000 0.000 0.000 0.000 0.053 0.067 0.067 0.089 0.008 0.000

0.004 0.004 0.000 0.004 0.000 0.000 0.000 0.000 0.000 0.000

0.004 0.000 0.013 0.000 0.004 0.004 0.004 0.000 0.078 0.083

0.039 0.083

>Non-Methylation code ID 21523

0.000 0.000 0.000 0.000 0.000 0.006 0.000 0.000 0.006 0.018

0.000 0.006 0.031 0.000 0.006 0.006 0.000 0.000 0.000 0.000

0.083 0.034 0.082 0.055 0.000 0.000 0.000 0.007 0.000 0.007

0.014 0.000 0.000 0.014 0.007 0.000 0.021 0.000 0.000 0.021

0.000 0.000 0.000 0.000 0.054 0.055 0.070 0.066 0.015 0.000

0.000 0.000 0.000 0.000 0.000 0.000 0.008 0.008 0.000 0.000

0.023 0.000 0.000 0.008 0.015 0.000 0.008 0.000 0.057 0.068

0.065 0.059

>Non-Methylation code ID 21523

0.012 0.000 0.000 0.006 0.000 0.018 0.000 0.000 0.006 0.006

0.000 0.012 0.000 0.000 0.006 0.006 0.000 0.000 0.000 0.000

0.067 0.081 0.053 0.061 0.004 0.000 0.004 0.004 0.000 0.004

0.000 0.000 0.000 0.000 0.004 0.000 0.004 0.000 0.013 0.000

0.004 0.004 0.004 0.000 0.085 0.080 0.054 0.061 0.000 0.000

0.000 0.014 0.000 0.014 0.000 0.000 0.000 0.000 0.000 0.000

0.000 0.000 0.019 0.000 0.005 0.000 0.000 0.000 0.062 0.077

0.049 0.093

>Non-Methylation code ID 21523

0.006 0.000 0.006 0.000 0.000 0.032 0.000 0.000 0.006 0.013

0.000 0.000 0.006 0.000 0.006 0.006 0.000 0.000 0.000 0.000

0.066 0.071 0.054 0.058 0.011 0.000 0.005 0.005 0.000 0.011

0.000 0.000 0.000 0.000 0.000 0.000 0.005 0.000 0.005 0.005

0.005 0.000 0.011 0.000 0.074 0.056 0.070 0.070 0.000 0.000

0.000 0.012 0.000 0.019 0.000 0.000 0.000 0.000 0.000 0.000

0.000 0.006 0.019 0.000 0.006 0.006 0.000 0.000 0.062 0.050

0.053 0.100

>Non-Methylation code ID 21523

0.000 0.000 0.006 0.006 0.000 0.012 0.012 0.000 0.000 0.006

0.006 0.000 0.006 0.000 0.006 0.012 0.000 0.000 0.000 0.000

0.066 0.058 0.057 0.079 0.012 0.000 0.000 0.012 0.000 0.006

0.000 0.000 0.006 0.006 0.000 0.000 0.006 0.000 0.000 0.000

0.018 0.000 0.006 0.000 0.058 0.061 0.065 0.077 0.000 0.000

0.000 0.000 0.000 0.011 0.000 0.000 0.011 0.017 0.000 0.006

0.011 0.000 0.006 0.006 0.000 0.000 0.000 0.000 0.083 0.043

0.072 0.068

>Non-Methylation code ID 21523

0.000 0.000 0.006 0.000 0.000 0.000 0.012 0.000 0.000 0.012

0.000 0.000 0.018 0.012 0.006 0.012 0.000 0.000 0.000 0.000

0.058 0.061 0.073 0.062 0.008 0.008 0.000 0.000 0.000 0.000

0.000 0.000 0.000 0.008 0.000 0.000 0.032 0.000 0.000 0.024

0.024 0.000 0.000 0.000 0.063 0.052 0.069 0.044 0.020 0.000

0.000 0.000 0.000 0.000 0.000 0.000 0.000 0.007 0.000 0.000

0.040 0.000 0.007 0.007 0.000 0.000 0.000 0.000 0.061 0.052

0.073 0.067

>Non-Methylation code ID 21523

0.000 0.000 0.000 0.005 0.000 0.005 0.000 0.000 0.005 0.005

0.000 0.000 0.005 0.000 0.010 0.010 0.000 0.000 0.005 0.000

0.060 0.085 0.063 0.073 0.008 0.012 0.000 0.004 0.000 0.008

0.004 0.000 0.004 0.000 0.000 0.000 0.000 0.000 0.012 0.000

0.000 0.000 0.000 0.000 0.081 0.062 0.067 0.072 0.014 0.000

0.000 0.014 0.000 0.007 0.000 0.000 0.000 0.000 0.000 0.000

0.000 0.007 0.014 0.000 0.007 0.014 0.000 0.000 0.042 0.073

0.065 0.075

>Non-Methylation code ID 21523

0.023 0.000 0.000 0.006 0.000 0.012 0.000 0.000 0.006 0.000

0.000 0.000 0.000 0.000 0.012 0.012 0.006 0.000 0.000 0.000

0.069 0.064 0.061 0.064 0.013 0.000 0.000 0.006 0.000 0.000

0.000 0.000 0.006 0.019 0.000 0.000 0.025 0.000 0.006 0.000

0.000 0.000 0.000 0.000 0.051 0.087 0.068 0.051 0.000 0.009

0.000 0.000 0.004 0.004 0.004 0.000 0.000 0.004 0.000 0.000

0.004 0.004 0.009 0.004 0.000 0.000 0.004 0.000 0.063 0.080

0.070 0.069
